# Hidden modes of DNA binding by human nuclear receptors

**DOI:** 10.1038/s41467-023-39577-0

**Published:** 2023-07-13

**Authors:** Devesh Bhimsaria, José A. Rodríguez-Martínez, Jacqui L. Mendez-Johnson, Debostuti Ghoshdastidar, Ashwin Varadarajan, Manju Bansal, Danette L. Daniels, Parameswaran Ramanathan, Aseem Z. Ansari

**Affiliations:** 1 Department of Biosciences and Bioengineering, Indian Institute of Technology Roorkee, Roorkee, 247667 India; 2 Department of Biology, University of Puerto Rico Río Piedras, San Juan, PR 00925 USA; 3 Promega Corporation, Madison, WI 53711 USA; 4 Molecular Biophysics Unit, Indian Institute of Science, Bangalore, 560012 India; 5 Department of Electrical and Computer Engineering, University of Wisconsin–Madison, Madison, WI 53706 USA; 6 Department of Chemical Biology and Therapeutics, St. Jude Children’s Research Hospital, Memphis, TN 38105 USA; 7 Present Address: Foghorn Therapeutics, Cambridge, MA 02139 USA

**Keywords:** Nucleic acids, Target identification, Computational biology and bioinformatics

## Abstract

Human nuclear receptors (NRs) are a superfamily of ligand-responsive transcription factors that have central roles in cellular function. Their malfunction is linked to numerous diseases, and the ability to modulate their activity with synthetic ligands has yielded 16% of all FDA-approved drugs. NRs regulate distinct gene networks, however they often function from genomic sites that lack known binding motifs. Here, to annotate genomic binding sites of known and unexamined NRs more accurately, we use high-throughput SELEX to comprehensively map DNA binding site preferences of all full-length human NRs, in complex with their ligands. Furthermore, to identify non-obvious binding sites buried in DNA–protein interactomes, we develop *MinSeq Find*, a search algorithm based on the MinTerm concept from electrical engineering and digital systems design. The resulting MinTerm sequence set (MinSeqs) reveal a constellation of binding sites that more effectively annotate NR-binding profiles in cells. MinSeqs also unmask binding sites created or disrupted by 52,106 single-nucleotide polymorphisms associated with human diseases. By implicating druggable NRs as hidden drivers of multiple human diseases, our results not only reveal new biological roles of NRs, but they also provide a resource for drug-repurposing and precision medicine.

## Abstract (web summary)

Nuclear receptors (NR) are drug-responsive master regulators. Here, authors map DNA binding profiles of all human NRs. Their MinSeq Find algorithm identifies masked NR binding sites in genomes and maps ~10% of orphan SNPs linked to numerous diseases.

## Introduction

Nuclear receptors (NRs) are a unique superfamily of 48 transcription factors that bind cell-permeable small-molecule ligands and trigger distinct gene circuits in different cell types. In humans, members of this superfamily have been shown to regulate a wide range of processes, including inflammation, infection, development, behavior, circadian rhythms, hormonal and metabolic fluxes, and xenobiotic stress^1^. Malfunctioning NRs cause a wide array of diseases and inherited disorders^2^. Their ligand-responsive nature renders NRs susceptible to modulation by synthetic ligands, resulting in nearly 16% of all FDA-approved drugs^3^.

Natural or synthetic ligands of NRs trigger non-identical transcriptional programs in different cell types. Even within a specific cell, a ligand can instruct its target NR to stimulate the transcription of one set of genes while silencing another^1^. This complex and nuanced regulatory response integrates many signals, including the allosteric transmission of ligand-induced conformational changes in the ligand-binding domain (LBD) to the closely juxtaposed DNA-binding domain (DBD). Reciprocally, binding to different DNA sites can subtly alter the quaternary conformation of a given NR impacting its ligand affinity and co-factor engagement, thereby eliciting different regulatory outcomes at different genes within the same cell^4,5^. Although the importance of physical and functional communication between the LBD and DBD was recognized in the earliest studies of NRs, drug development, and DNA-binding studies have relied on isolated LBDs and DBDs. Recent high-resolution co-crystal structures of four different NRs comprising both the DBD and LBD are refocusing attention on the role of interdomain interfaces in integrating signals from DNA sequences and small-molecule ligands^4,6–12^. In each case, the DBD–LBD interface has emerged as a “convergence zone” through which allosteric signals between the domains are transmitted and distinct regulatory decisions defined.

**Fig. 1:**
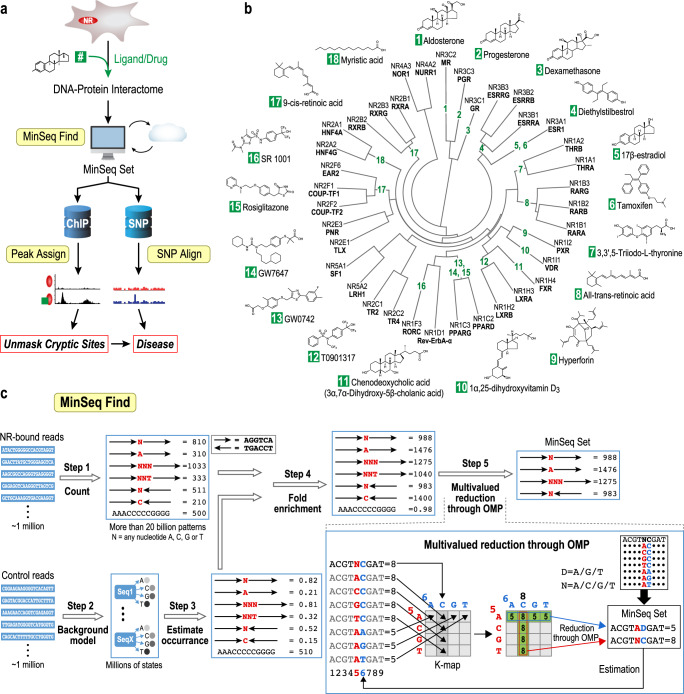
Strategy to map the full compendium of human nuclear receptor-binding sites. **a** A MinSeq set is extracted from the DNA–protein interactome (DPI) of full-length human nuclear receptors (NRs) using the *MinSeq Find* algorithm. The *Peak Assign* analysis uses the extracted MinSeq set to score and annotate chromatin immunoprecipitation (ChIP-seq)–derived genomic loci bound by NRs in vivo. In parallel, the *SNP Align* analysis evaluates the impact of single nucleotide polymorphisms (SNPs) on creating or disrupting NR-binding sites. **b** Phylogenetic tree (neighbor-end joining) of NRs and corresponding small-molecule ligands (numbered in green boxes) used in this study. (Source data are provided as a Source Data file). **c** A schema of the *MinSeq Find* algorithm. DNA-sequencing reads obtained via high throughput-SELEX are counted as patterns of nucleotide sequences of different lengths separated by linkers of varying sizes, referred to here as MinSeq (Online Methods). *MinSeq Find* can capture multiple patterns of binding, including a variable spacer sequence and length and NR-binding orientation. Fold enrichment for MinSeqs is calculated by normalization of the read count against a PAGLO library model. The iterative algorithm Orthogonal Matching Pursuit (OMP) then further minimizes and optimizes the MinSeq set. (Designed by Laura Vanderploeg).

Reaffirming the importance of ligand- and protein-interfacial interactions in influencing DNA binding site preferences, a recent study with a dozen full-length NRs and a well-crafted set of binding sites uncovered unexpected modes of DNA recognition^13^.The results of this focused study alluded to the existence of a wider range of binding modes beyond canonical motifs that were obtained with isolated DBD modules in a ligand- and partner-agnostic manner. Moreover, the limited ability of current motifs to annotate in vivo binding profiles of NRs, motivated us to investigate DNA-recognition properties of all 48 full-length human NRs along with their obligate partners and key small molecule ligands or drugs (Fig. 1a). Furthermore, to comprehensively capture the spectrum of binding sites embedded in the DNA–protein interactomes (DPI), we developed “*MinSeq Find*,” an algorithm based on Boolean algebra and principles of digital systems design optimization.

In this work, we present a compendium of MinSeqs containing previously unknown NR-binding sites. The biological relevance of herein identified sites is evident from their ability to annotate NR-bound genomic loci more effectively, especially at many sites where NRs were assumed to be indirectly tethered by other proteins due to the absence of known motifs^14^. Furthermore, MinSeqs not only capture known NR-linked SNPs (single nucleotide polymorphisms) in multiple large public databases but more importantly, identify 8–14% of the unassigned “orphan” SNPs as masked NR-binding sites. Identifying such disease-associated orphan SNPs as NR binding sites enables the repurposing of FDA-approved drugs for diseases not previously known to be regulated by this druggable class of transcription factors. In essence, our compendium of NR binding sites may serve as a resource for genome-guided precision medicine.

## Results

### DNA Interactome of human NRs

To investigate the full spectrum of sequences bound by full-length human NRs, we were successful in expressing 45 of the 48 members of this transcription factor family as Halo-Tag fusions in HEK293T cells (Fig. 1, Supplementary Table 1 and Supplementary Data 8). To determine the effects of ligand-binding on binding site preferences, 21 cognate ligands, including drugs such as dexamethasone (#3), and physiological ligands, such as β-estradiol (#5), were incubated with their cognate NRs (Fig. 1b—specific drugs/ligands with their corresponding numbers in green boxes). In parallel, 18 NRs known to dimerize with Retinoid X Receptors (RXRs) were incubated with RXRα to probe the impact of heterodimerization on DNA-sequence preferences. To obtain comprehensive DNA-recognition landscapes, we utilized a high throughput SELEX (HT-SELEX) approach and incubated cell lysates expressing each NR with a DNA library comprising every sequence permutation spanning a 20mer binding site (~10^12^ unique sequence permutations)^15,16^. The entire set of oligonucleotide sequences bound by each NR was captured using HaloTag beads; these sequences were amplified by PCR and subjected to two additional cycles of selection and enrichment (Supplementary Figs. 1 and 3 and Supplementary Table 2). Massively parallel sequencing of each round resulted in high-quality DPIs of 38 full-length NRs. DAX1, a receptor that lacks a DNA binding domain, failed to enrich DNA, thereby validating the fidelity of our approach. Importantly, 28 interactomes of ligand-bound NRs and 18 interactomes of RXRα–NR heterodimers yielded 83 high-quality DPI datasets from 214 individual HT-SELEX experiments. In addition, using our herein-described search algorithm, we reexamined and integrated all publicly available NR interactomes that were obtained through high throughput sequencing^16–19^. Thus, to our knowledge, this study provides the most comprehensive compendium of all human NR-binding sites, especially in complex with ligands and RXRα (Supplementary Data 2).

### *MinSeq Find* algorithm

**Fig. 2:**
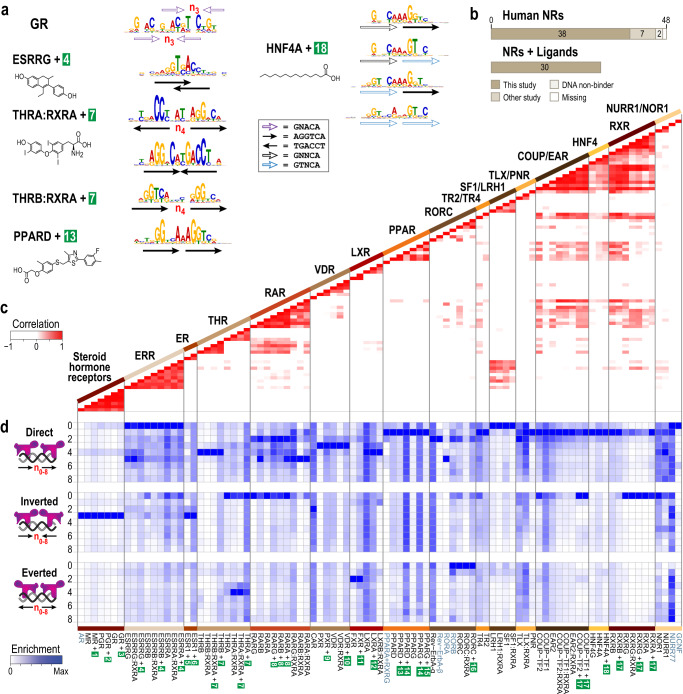
MinSeqs unmask distinct binding sites while capturing known motifs. **a** Representative examples of MinSeq-derived LOGOs of a subset of NRs. Different arrows represent distinct half-site sequences, and the spacing between half-sites is denoted by ***n*** (red). Small-molecule ligands (numbered in green boxes) are from Fig. 1b. **b** Interactome data for a total of 45 out of 46 DNA-binding human NRs is presented in this study, 7 of which were included from publicly available datasets. This includes a systematic study of DNA-interactomes of 30 ligand-bound NRs. **c** Clustergram of Pearson correlations of each NR–DNA interactome pair (row and column), with shades of white to red as 0 to 1 correlation and white 0 to −1. A total of 91 DPIs (7 publicly available NR interactomes labeled in blue letters) were calculated from the binding enrichment of the union of the top 100 MinSeqs from the Orthogonal Matching Pursuit. NRs are phylogenetically clustered as in Fig. 1b and the sub-families are further delineated with vertical black lines that extend to the diagonal color-coded bar. *MinSeq Find* analysis of all DNA-binding human NRs is included in Supplementary Data 2–4. **d** Heatmap summarizing binding preferences of NRs in the context of knowing half-site arrangements. Columns represent different NRs, and rows correspond to different monomer orientations (DR, direct repeat; IR, inverted repeat; ER, everted repeat), with spacer length (*n*) ranging from 0 to 8 nucleotides. Enrichment of different categories is colored from minima (white) to maxima (blue). Half-site sequences for different NRs are as follows: steroid hormone receptor—^5ʹ^GNACR^3ʹ^; TLX/PNR—^5ʹ^RRGTCR^3ʹ^; ERR/THR/RAR/PPAR and RXR—^5ʹ^RGGTCR^3ʹ^; and rest of the NR members—^5ʹ^RGKTCR^3ʹ^ (R = A/G, K = G/T, N = A/C/G/T). (Source data are provided as a Source Data file. Designed by Laura Vanderploeg).

To identify novel NR binding sites, we mined the DPIs of each NR with current motif-finding algorithms (Online Methods). The motifs that emerged displayed recognizable elements of classic NR-binding sites, including direct repeats (DR), inverted repeats (IR), everted repeats (ER), and monomeric “half” sites, but failed to identify previously reported non-canonical binding sites (schematic representation in Fig. 2d)^14,20,21^. To capture biologically relevant binding sites missed even by sophisticated deep learning-based motif finding algorithms, we adapted fundamental concepts from the field of a digital system design of electrical engineering to create *MinSeq Find*, an algorithm that identifies a subset of k-mers that effectively encapsulates DNA-binding preferences buried in a comprehensive DPI dataset (Fig. 1c, Supplementary Fig. 2 and Supplementary Data 1). Our approach is based on the concept of MinTerms in Boolean algebra, where any logic function can be expressed as a sum of MinTerms. MinTerms with Karnaugh map (K-map) reduction is used in digital circuit minimization to reduce the number of electronic gates needed to implement any given logic function^22^. Analogously, a defined set of sequences that comprehensively capture the complex spectrum of binding preferences embedded within a DPI can be expressed as a set of weighted “MinTerm Sequences” or MinSeqs. However, unlike MinTerms, MinSeqs are multivalued due to the different levels of enrichment of distinct DNA sequences. *MinSeq Find* algorithm starts by considering a set of all possible composite k-mer patterns in the given dataset. Because NR dimers are known to bind monomeric half-sites separated by varying number of “gap” nucleotides, *MinSeq Find* next calculates fold enrichment by normalizing reads in a NR–DNA interactome against PAGLO, a **P**osition-**A**ssociated **G**apped **Lo**cation–specific inhomogeneous Markov model of the DNA library (Supplementary Data 1). PAGLO is tailored to address sequences comprising nucleotide gaps in a 20mer-binding site. MinSeqs are ranked by their ability to capture different binding affinities (for algorithmic details see Supplementary Data 1). Similar to K-map reduction in digital systems design, further pruning and optimization of the initial set of MinSeqs is achieved by iterative multivalued reduction via the Orthogonal Matching Pursuit algorithm (OMP) used for sparse approximation and compressive sensing in the signal processing subfield of electrical engineering (Fig. 1c). This multi-tiered approach yields the final weighted MinSeq set from the comprehensive DPI dataset. It is important to note that adaptation of the OMP sparse approximation method permits *MinSeq Find* to identify a sparse, yet comprehensive, binding site profile of any given NR within the vast ~10^12^ sequence search space (Fig. 1c). These sparse k-mer sets (MinSeqs) facilitate more ready and robust comparisons of different DPI.

To identify converging patterns, sequence logos based on position weight matrices (PWM) are then constructed from the full MinSeq set iteratively. The first PWM is constructed from enrichment values calculated for sequences with zero or one mismatch to MinSeq with the highest weighted enrichment (Online Methods, Supplementary Fig. 2f). Next best PWM is chosen from the residual enriched MinSeqs retained after subtracting out MinSeqs that contribute to the earlier PWMs. This is repeated until a maximum number of iterations is reached, or minimum residual enrichment is achieved.

### *MinSeq Find* reveals distinct DNA binding modes

**Fig. 3:**
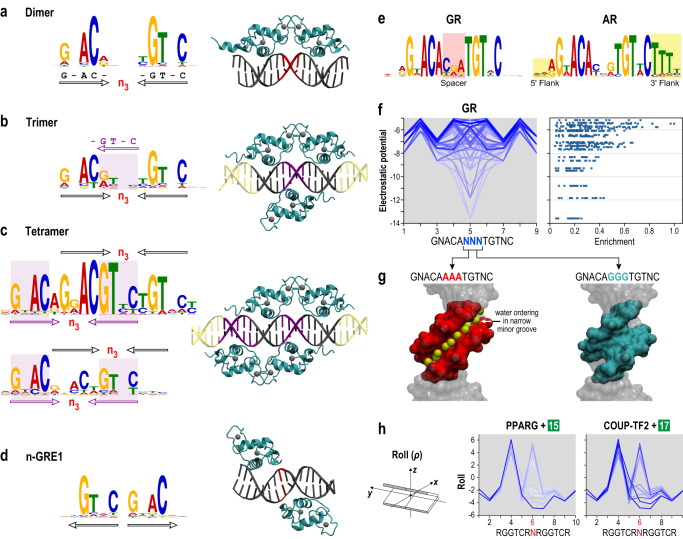
Unconventional binding modes and impact of the spacer and flanking sequences. **a**–**d** Multiple modes of DNA binding of Glucocorticoid receptor (GR)—**a** canonical homo-dimer bound to IR3 site (spacer in red), **b** trimer (overlaid half site in purple), **c** two tetramers (overlaid IR3 half sites in purple), and **d** monomers bound to non-canonical everted repeats of GR (n-GRE1 motif). The left panel presents motif representations of binding. Black and purple arrows represent GR binding to monomers G-AC- and -GT-C. The right panel presents corresponding structural representations—the crystal structure of the dimer (PDBid: 3g6q), and energy-minimized models of the trimer, tetramer-1, and n-GRE1 complexes. **e** Steroid hormone receptors GR and androgen receptor (AR) bind similar motifs but exhibit unique preferences for spacer (in red) and flanking DNA (in yellow) sequences. **f** GR binding affinity is correlated with the electrostatic potential (EP) of the spacer region in the canonical GR binding site. Left panel: binding affinity of GR to DNA sequences matching known motif ^5ʹ^GNACANNNTGTNC^3ʹ^ plotted as a function of EP reveals a strong correlation at the spacer region (center). The color of lines from minima (white) to maxima (blue) indicates enrichment. Right panel: scatter plot of enrichment at nucleotide position 5 (central N of the spacer) plotted as a function of EP. **g** DNA counterpart of two GR-DNA co-crystal structures demonstrates GR binding site with more negative EP (red, PDBid: 3g9i) harbors an ordered spine of hydration while one with a less negative EP (blue, PDBid: 3g6q) does not. The ordered hydration spine in the minor groove alters GR-DNA binding dynamics, thereby impacting binding affinity. **h** PPARG+ligand #15 and COUP-TF2+ligand #17 appear to display identical preferences for a direct repeat of ^5ʹ^RGGTCR^3ʹ^ half-sites separated by a 1-nucleotide spacer (DR1). However, each ligand-bound heterodimer displays different preferences for the DNA shape in the spacer and the second half site. Base step Roll values of different DNA sequences within DR1 are plotted and colored white (minima) to blue (maxima) corresponding to the enrichment values. Roll (ρ, inset) describes the rotational relationship between two stacked base pairs, with a positive role indicating that the base pairs are opened towards the minor groove. (Source data are provided as a Source Data file. Designed by Laura Vanderploeg).

In our 83 high-resolution DPI datasets, *MinSeq Find* unmasked a constellation of binding sites in addition to readily identifying known NR-binding modes (Fig. 2, tabulated in Supplementary Data 2–4). For example, in the case of the glucocorticoid receptor (GR), *MinSeq Find* identified the classic inverted repeats of GnACA half-sites separated by a 3-bp spacer (IR3), the exceedingly infrequent everted repeats (ER1), as well as the nonobvious tetramer sites that permit two GR dimers to simultaneously co-occupy overlapping IR3 sites on the opposite faces of the DNA helix (Figs. 2a and 3c)^5,23^. More unusual are the superimposed AGGTCA monomeric sites in the DNA interactome of the estrogen-related receptor (ESRRG) (Fig. 2a). In this arrangement, the steric clash would permit only one of the two overlapping sites to be occupied at any given time, raising the specter that overlapping sites may function as a kinetic trap^24^. In principle, such arrangements may increase the probability that upon dissociation from one monomeric site, the protein may more readily reassociate with an overlapping site and thus increase its cumulative dwell time at a given locus. Such increased engagement would be consistent with the intradomain association-dissociation feature of the classic “Facilitated Diffusion” model postulated by von Hippel and Berg^25^. However, further experiments are needed to determine the mechanistic basis for the enrichment of overlapping sites that sterically occlude co-occupancy by two ESRR monomers.

The enriched MinSeqs from our 83 DPI sets correlates exceedingly well within their phylogenetic subfamily clusters (Fig. 2c). Intriguingly, the cross-correlation of top 100 OMP enriched MinSeqs between sub-families suggest possible heterodimer formations or shared binding sites that might enable co-regulation of a common set of genes (Fig. 2c). The ability of the RXR subfamily to heterodimerize promiscuously with type II NRs is reflected in the number of binding sites shared by RXRs with multiple other NRs. However, the cross-correlation between MinSeqs of COUP-TFs or HNF4 with NRs other than RXR was surprising because these proteins are not known to share binding sites or heterodimerize with other NRs. Unexpected counterexamples of differences in binding site preferences between members of a sub-family are also evident. For example, two members of the THR sub-family prefer starkly different monomeric site arrangements: THRA prefers binding to Inverted and Everted Repeats (IR0 and ER4), whereas THRB prefers binding to DR (DR4). To our knowledge, these differences between THRA and THRB have not been described before. Another unexpected observation is that PPARD displays greater dependence on sequence fidelity of the downstream monomeric half-site whereas, in co-crystal structures of PPARD–RXRA, the 3′ half-site is bound by the promiscuous RXRΑ partner^6^. One explanation for such binding site dependence is that PPARD-DNA binding is enhanced by RXRA–DNA interaction. In the case of homodimeric HNF4A, the co-crystal structure shows the DNA binding domain of only one monomer to the central CAAAG sequence of a DR1 site^8^, whereas MinSeqs reveal distinct half-site sequences that are obscured within a consensus motif provided by traditional search algorithms^26^.

### A comprehensive compendium of all human NRs

Having demonstrated the power of *MinSeq Find* to identify known motifs and unmask additional modes of DNA binding in our 83 DPI datasets, we next mined publicly available NR–DNA interactomes that were obtained via high throughput sequencing^16–19^. Our approach yielded a comprehensive compendium of binding sites for all human NRs that bind DNA (Supplementary Fig. 4). This comprehensive NR MinSeq set contains, (i) binding sites identified herein, (ii) 28 previously known modes of interaction (monomeric “half-site” binding mode combined with dimeric binding to DR, IR, or ER half-sites with 0- to 8-bp spacers (*n* = 0–8) (Fig. 2d), (iii) specificity contributions of non-contacted internal spacer and external flanking sequences, (iv) contribution of DNA shape to selective binding by different NRs (Fig. 2d), and (v) shared binding modes between different sub-families of NRs (Supplementary Fig. 4 and Supplementary Data 2–5).

MinSeqs encapsulating the canonical half-site arrangements confirm expected adherence to the “3-4-5 rule” for VDR, THR, and RAR which bind DRs separated by 3, 4, or 5 base pairs, respectively^1^ (Fig. 2c). The data also suggest a “0-1-2 rule” for several NRs, for example, LRH/SF1 (DR0), COUP-TF/EAR (DR1), and Rev-ErbA (DR2) respectively. The latter observation is validated by a co-crystal structure of Rev-ErbA binding in DR2 fashion^27,28^. In agreement with a recent report of 12 NRs binding to a single monomeric half-site^13,28,29^, MinSeqs demonstrate that this recognition mode is utilized by most NRs (Supplementary Data 4). An unusual mode of monomeric binding to overlapping half-sites, as in the case of the ESRR subfamily is also identified by our approach.

### Structural evaluation of unconventional binding modes

To evaluate atypical binding modes we focused on GR, a highly scrutinized steroid hormone receptor that binds corticosteroids to upregulate anti-inflammatory genes and down-regulate inflammatory genes. The MinSeq compendium of GR included an unusual set of superimposed IR3 binding sites (Fig. 2a). Moreover, heat plots in Fig. 2d suggested binding to DRs DR0 and DR4 (Fig. 2d, rows 1 and 5, columns 6 and 7). To determine if GR could structurally occupy these unusual half-site arrangements, we used high-resolution GR-IR3 co-crystal structures (Fig. 3a) to generate energy-minimized models of DNA-bound GR trimers (Fig. 3b), tetramers (Fig. 3c), and monomers on everted half-sites (Fig. 3d and Supplementary Fig. 5) (Online Methods). All-atom molecular dynamics simulations were performed for the DNA-bound GR tetramer model for 500 ns in an explicit solvent under constant pressure and temperature conditions. The stability of the complex was tested by calculating root mean square deviation (RMSD) and the local stability using root mean square fluctuation (RMSF) of the backbone Cα atoms compared to the Protein Data Bank (PDB)-derived initial model (Supplementary Fig. 6). Despite overall high flexibility of the DBD, high stability was exhibited by conserved GR residues that make base-specific hydrogen-bonds with the canonical GnACA monomeric half-site (circled in Supplementary Fig. 6)^30^. On the other hand, binding in a DR0 orientation is disfavored. On closer examination of the DR0 and DR4 binding sites^31^, the misleading DR arrangement of monomeric sites appears to be a consequence of the degeneration of an external half-site of two superimposed IR3 motifs (PWM in Fig. 3b). While uncommon, such unconventional oligomeric arrangements are biologically functional^23^ and even the low affinity everted repeats reflect an arrangement observed in known negative-GRE or n-GRE sequence CTCC-n_0–2_-GGAGA^32^, however, binding to a diverse set of sequences in the everted arrangement was not previously reported.

### Shape selectivity conferred by the spacer and flanking sequences

Ensembles of related sequences bound by a family of NRs are often grouped into a single “shared motif.” For example, IR3 emerges as the common motif for all steroid hormone receptors (Fig. 2c, d). However, deconvoluting motifs into MinSeqs reveals that intervening spacers between half-sites and sequences flanking the core binding site diversify local microstructure and guide selective NR association. Among steroid hormone receptors, GR preferentially binds sites with a CGA spacer, whereas the androgen receptor (AR) favors T-stretches flanking the 3′ ends of the core IR3 motif (Fig. 3e). The preference for the CGA spacer correlates well with the electrostatic potential (EP) of the DNA minor groove (Fig. 3f). Inspection of GR-DNA co-crystal structures revealed that binding sites containing a highly negative EP in the spacer region (e.g., AAA) harbor an ordered spine of water within a narrow minor groove **(**Fig. 3g). The absence of similar hydration spine and decrease in EP in spacer sequences with a wider minor groove (e.g., GGG/CGA) enhances GR binding. These sequence preferences amongst members of a given family are also evident in ChIP-seq data^33^ in Supplementary Table 3.

The contribution of the seemingly non-descript spacer and flanking sequences on overall binding site conformation is broadly used to confer NR selectivity amongst “shared motifs.” In two contrasting examples, PPARG is exquisitely sensitive to DNA shape (roll) at the interface of a DR1-binding site, whereas COUP-TF2 tolerates a broad range of shape variations imparted by the spacer (Fig. 3h)^34^. Similarly, ligand-bound RARB and RORC prefer distinct DNA shapes in both the spacer and flanking sequence of otherwise identical DR2 motifs (Supplementary Fig. 7). Thus, the oft-neglected, non-conserved spacer and flanking sequences, in conjunction with nondescript variations in monomeric half-site composition, drive shape-selective NR binding amongst sequences that are represented as a single motif^35,36^. Because they are not compressed into consensus motifs, MinSeqs identify contributions of sequence context and underlying DNA microstructure to selective binding preferences of different NRs.

### Ligands and partners transform the sequence selectivity of certain NRs

Different small-molecule ligands and DNA-binding sites can alter the regulatory function of a given NR^31,32,37–42^. We, therefore, examined the impact of 21 natural or synthetic ligands on DNA binding sites preferred by their cognate NRs. Of these, 18 ligands targeting 25 cognate NRs yielded 30 DPI (Figs. 1b and 2d—individual ligands numbered in green boxes). Across NRs, ligand-binding subtly modulated site preferences of most NRs, counterintuitively over half of the interactomes displayed lower enrichment for sites bound by NRs in the unliganded state (Supplementary Data 3). Reassuringly, ligand-responsive reduction in affinity has been observed for the RXRA–RARB heterodimer^4^. To illustrate the contribution of ligand binding on DNA selectivity, we focused on RXRA because it displayed a marked change in its MinSeq set upon binding its ligand 9-cis-retinoic acid (#17). However, RXRA has multiple known partners along with co-crystal structures with three distinct partners, a wealth of genome-wide binding and gene regulation data, and diverse biological and pathological roles^1,4,6,7,20^.

**Fig. 4:**
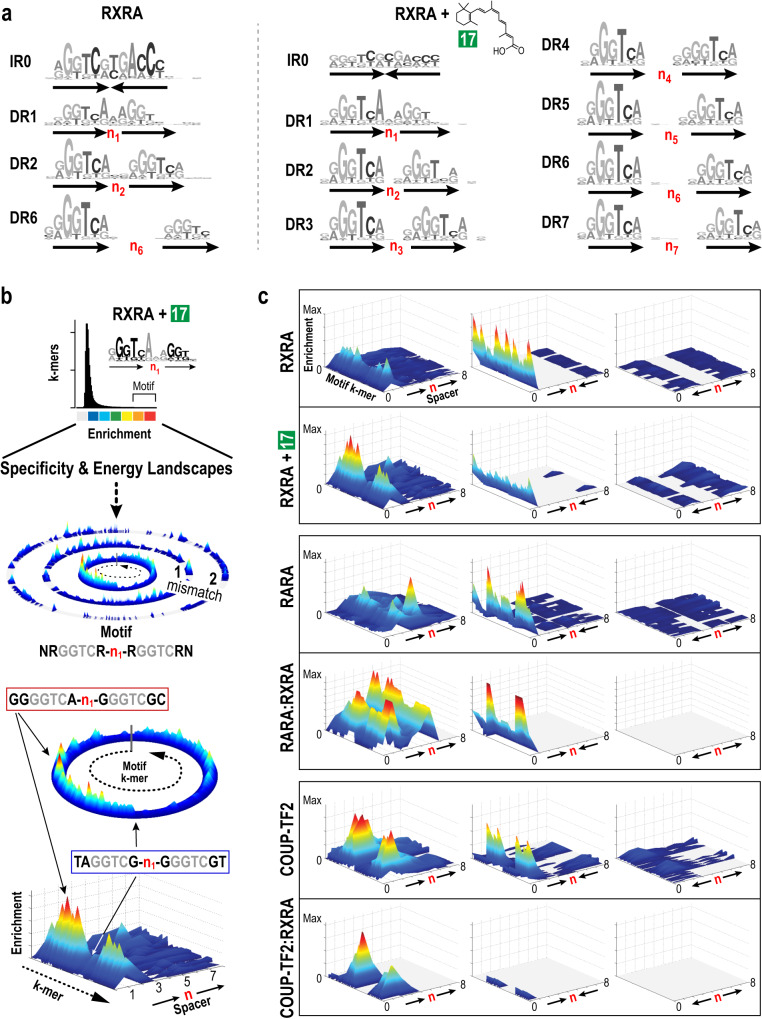
Contribution of ligand- and partner proteins on RXRA binding site preferences. **a** LOGO representation of DNA-binding preferences of RXRA with and without its ligand (#17) for different orientations of ^5ʹ^RGKTCR^3ʹ^ half site separated by different spacer lengths (n_1_–n_7_). **b** Specificity and binding-energy landscapes (SELs) of an example case with ^5ʹ^NRGGTCR-n_1_-RGGTCRN^3ʹ^ direct repeat as a seed to organize (where R = A/G, N = A/C/G/T, *n* = spacer). The top panel displays a histogram of enriched k-mers, with the highest affinity k-mers used to derive a position weight matrix-based motif (DR1). The color scale represents the extent of enrichment/affinity. In circular SELs, (second panel), the PWM motif is used as a seed to organize the rest of the k-mers in concentric circles. The central ring contains all k-mers that match the seed ^5’^NRGGTCR-n-RGGTCRN^3’^ motif but may differ in spacer and flanking sequences. Sequences with a hamming distance *m* from this motif are represented in corresponding *m*th (0,1, 2,…) mismatch rings. Color-coded enrichment values are proportional to the binding affinities of individual k-mers and variations in intensity reflect the contribution of the spacer and flanking sequences on binding to the core motif (third panel from the top). In the bottom panel, central rings (no mismatch to the motif) of SELs are linearized along the y-axis in the direction of the dotted arrow and arrayed along the x-axis by increasing increments in the intervening spacer length (*n*_0–8_). **c** Linear-SELs depict the effects of ligand and partnering RXRA on the binding preference of selected NRs. Along the *x*-axis in the left panel, the half-site is presented as a direct repeat (DR) with spacer *n* spanning 0–8 base pairs. The middle and right panels display the same half-site in an inverted (IR 0-8) or everted (ER 0-8) arrangement. Half-site is ^5ʹ^RGKTCR^3ʹ^ for COUP-TF2 and COUP-TF2:RXRA and ^5ʹ^RGGTCR^3ʹ^ for the rest. On the *y*-axis, k-mers belonging to specific groups (DR, IR, or ER) but bearing different spacer and flanking sequences are plotted in positional and alphabetical order. The *z*-axis displays the enrichment values of each k-mer as color-coded peaks. For additional details on SELs, see Supplementary Fig. 8. (Source data are provided as a Source Data file. Designed by Laura Vanderploeg).

RXRA in its unliganded state enriches IR0 followed by DR1 class of binding sites. The binding of 9-cis-retinoic acid (#17) flips this preference and greatly expands the range of RXRA-preferred binding-site arrangements (Fig. 4a). To display the multidimensional changes in sequence preferences of liganded-RXRA, we reconfigured our original concentric specificity and binding energy landscape (SEL) plots (Fig. 4b, Supplementary Fig. 8). In SEL plots, a binding motif is used to organize k-mers across all binding affinities or enrichment scores in concentric rings^15,35,43^. Sequences with the perfect match to the core motif are placed in the innermost ring, and those with increasing mismatches are placed in successive outer rings in an alpha-numeric order (Fig. 4b, second panel). Even in the innermost ring, with identical^5’^GGTC^3’^ monomeric half-sites, the spacer and flanking sequences can dramatically alter affinity for the core motif (Fig. 4b, sequences within the red and blue boxes). To compare the global shift in specificity we focused on the innermost ring and displayed it in a linear format along the *y*-axis (Fig. 4b, k-mer dotted arrow, bottom panel). Next, along the x-axis, we aligned SELs with increasing spacer lengths (n_0-8_) separating the DR ^5’^RGGTCR^3’^ half-sites (Fig. 4b, bottom panel). The enrichment score of each k-mer is displayed along the *z*-axis (Fig. 4b and Supplementary Data 6, for detailed description, see Supplementary Fig. 8). To compare between different half-site arrangements, in each panel of Fig. 4c, MinSeqs are organized in one of three canonical half-site arrangements (DR on the left, Inverted Repeats in the middle, and Everted Repeats on the right), with each unit “n” representing a spacer spanning 0–8 nucleotides. The composite landscapes unambiguously reveal that ligand-binding and heterodimerization with different partnering NRs, such as RARA or COUP-TF2, dramatically alter the specificity and affinity profile of RXRA (Fig. 4c and Supplementary Data 7).

### MinSeqs effectively annotate genome-wide binding sites

**Fig. 5:**
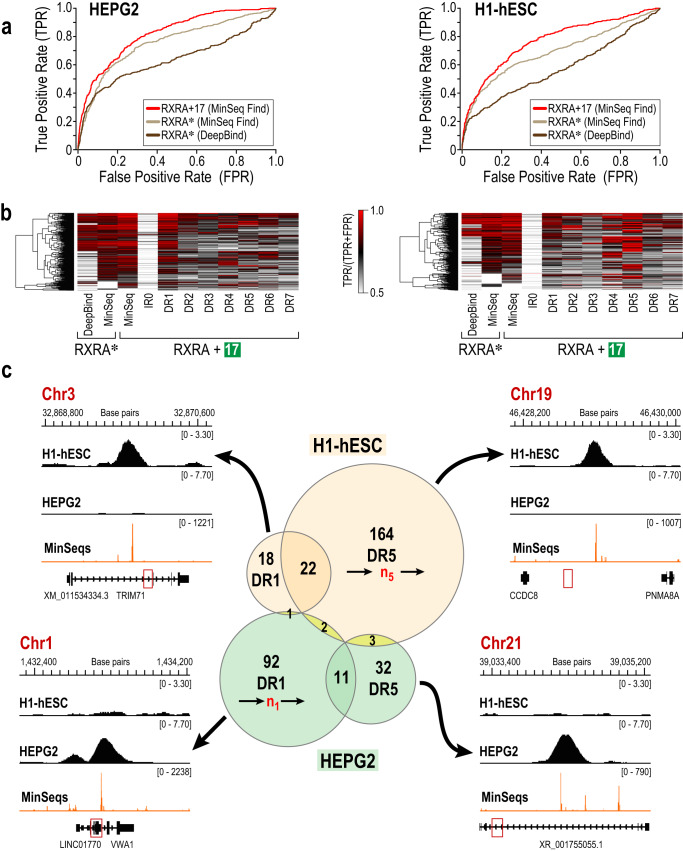
MinSeqs annotate genome-wide and cell-specific binding. **a** Receiver operating characteristic (ROC) curves obtained by scoring the top 500 RXRA ChIP-seq peaks in HEPG2 and H1-hESCs cells using DeepBind and *MinSeq Find* algorithms on published RXRA DNA–protein interactome data (RXRA*—dark brown and tan curves, respectively). The red curve displays ChIP-seq classification using MinSeqs from our new RXRA + 17 DNA–protein interactome data. **b** The heatmaps deconvolute the contribution of binding sites bearing different half-site arrangements for each of the 500 genomic loci identified by ChIP (scaled from high (red) to low (white) using normalized precision values TPR/(TPR + FPR)). **c** Venn diagram for ChIP peaks bearing direct repeat of ^5ʹ^RGKTCR^3ʹ^ half site with a 1- or 5-nucleotide spacer. These peaks (HG38) have normalized precision greater than 0.9 in the two cell lines. ChIP profiles aligned with the corresponding MinSeq scores of RXRA + 17 for representative ChIP peaks in the Venn Diagram. (Source data are provided as a Source Data file. Designed by Laura Vanderploeg).

To determine the biological relevance of MinSeq discovered binding modes, we examined genome-wide binding profiles of RXRA in the human hepatocyte cell line (HEPG2) and human embryonic stem cells (H1-hESCs)^44^ (Fig. 5a). To benchmark the ability of MinSeqs to annotate in vivo binding sites (identified by ChIP-seq methods), we compared our RXRA-binding MinSeq sites to those obtained by the automated deep-learning algorithm DeepBind^45^. DeepBind annotated fewer than half the RXRA ChIP peaks at the false-positive rate of 0.1 (Fig. 5a). In contrast, MinSeqs were far more effective in annotating RXRA-binding sites in two different cell lines. To ensure that the publicly available RXRA interactome used by DeepBind was not inherently limited, we performed *MinSeq Find* analysis of the published RXRA–DNA interactome used by DeepBind motif search algorithm. MinSeqs from that public DPI dataset also better predicted RXRA binding in the two cell lines than motifs obtained by DeepBind (Fig. 5a). Distributing MinSeqs into discrete canonical binding-site categories, such as DR1–7, clearly delineated distinct binding sites preferred by RXRA in different ChIP-seq peaks (Heat maps in Fig. 5b). These deconvoluted binding modes underscore the challenge of annotating varied genome-wide binding using consensus motifs provided by prevalent algorithms.

A comparison of binding arrangements reveals cell line-specific differences, for example, DR5-containing sites are preferred over the DR1-containing sites in H1-hESCs, whereas the converse is true in the HEPG2 cells. Interestingly, even where DR1 or DR5 binding sites are utilized, they occur in largely non-overlapping regions of the genome in the two cell lines (Fig. 5c). While multiple overlaying factors, including chromatin accessibility and cell-type specific complement of transcription factors and co-regulators, contribute to differential genomic access, yet MinSeqs identify conventionally as well as non-obvious NR binding sites within annotated ChIP peaks. Thus, improved annotation of genome-wide binding profiles by MinSeqs affords greater precision in ascribing a regulatory function to different binding modes at distinct genomic loci. As a resource for the community, we now provide the MinSeq analysis of all NR ChIP-seq data from ENCODE^46^ and the LoVo cell line^47^ (Supplementary Data 9). When applied to published NR-DNA interactome data, MinSeq analysis better predicted ChIP-seq peaks compared to automated algorithms DeepBind^45^ and gkmSVM^48^, and semi-automated Autoseed^17^ (Supplementary Data 10).

### Disease-associated SNPs create or disrupt masked NR sites

**Fig. 6:**
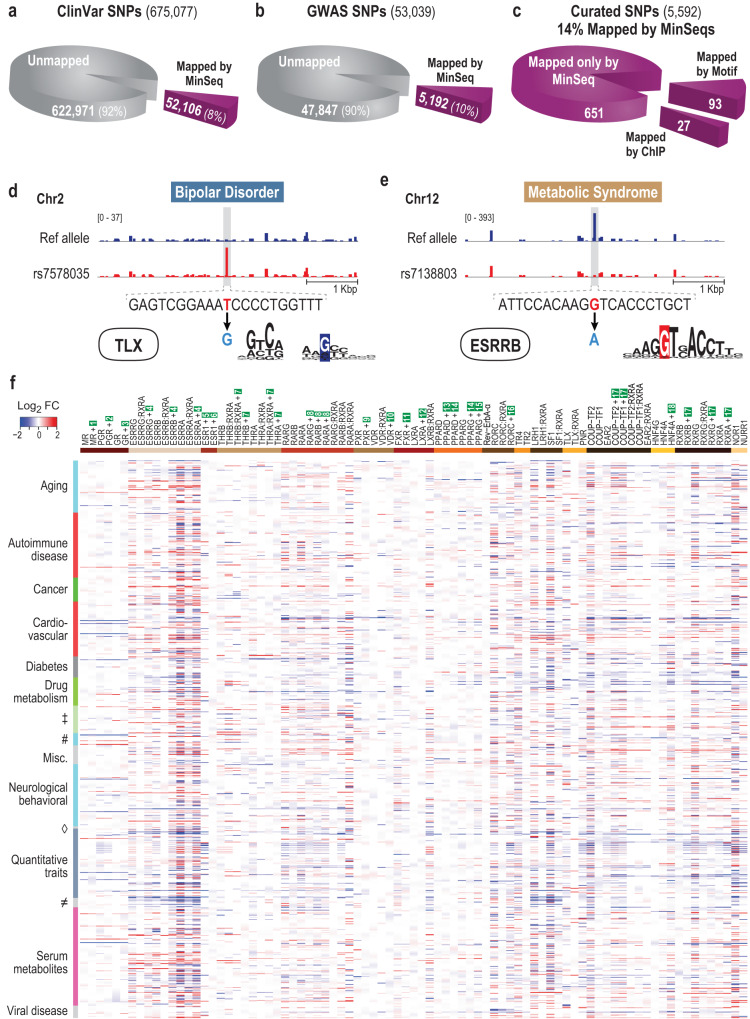
NR MinSeqs map to disease-associated single-nucleotide polymorphisms. **a** Mining the non-coding SNPs in ClinVar, the NCBI-run public archive with MinSeqs revealed ~8% of SNPs as creating or disrupting NR binding sites. **b** Mining the NHGRI-supported database of SNPs from genome-wide association studies annotated ~10% as potential NR-binding sites. **c** Mining a manually curated set of SNPs that are closely associated with diseases and other quantitative traits, identified ~14% as hidden NR sites, of these 771 SNPs, 120 were previously mapped as NR sites by known NR motifs (93) or empirically via ChIP studies (27). **d**, **e** MinSeqs enrichment scores across 5 kbp windows centered at the curated SNP (in red) or the reference allele (blue). **d** The SNP rs7578035 reveals a gain of function that creates a potential binding site for TLX. **e** The SNP rs7138803 disrupts the uncommon ESSRB-binding site. **f** Heat map of 771 highly curated SNPs predicted to create or disrupt NR-binding sites mapped onto associated diseases and quantitative traits. MinSeq scores derived from different NRs are presented in columns, and the rows display individual SNPs categorized by disease class (‡ Hematological parameters, # Kidney, lung and liver-related, ◊ Parasitic bacterial disease, and ≠ Radiographic parameters). The impact of SNPs on potential NR binding is plotted as the fold change (FC) on a log2 scale (Online Methods). In this plot, Red indicates gain of function—i.e., the creation of a potential binding site, whereas Blue indicates loss of function or disruption of the binding site that was present on the reference allele. (Source data are provided as a Source Data file. Designed by Laura Vanderploeg).

Armed with the MinSeq collection for all human NRs, we examined both the 675,077 clinically relevant SNPs from the NCBI-supported ClinVar database as well as the 53,039 disease-associated SNPs from NHGRI-supported GWAS Catalog^49^ (Fig. 6a, b). While disease-associated SNPs may not be causal, MinSeqs readily mapped 52,106 ClinVar SNPs (~8%) and 5192 GWAS SNPs ( ~ 10%) as potential NR-binding sites (Fig. 6c and Supplementary Fig. 9). To further probe the nature of NR-binding sites identified by MinSeqs, we examined a manually curated set of 5592 SNPs more stringently associated with specific diseases^50^. Of these, we identified 771 (~14%) that lead to the creation or disruption of an NR-binding site (Fig. 6c). Remarkably, among these 771 SNPs, we captured 93 that were previously mapped using known NR motifs and 28 that were identified by ChIP-seq studies. Although the common perception is that SNPs primarily disrupt binding sites, statistically a sequence variant can just as readily create a new binding site that may contribute to the diseased state^51^. Consistent with this expectation, we mapped a SNP (rs7578035) linked to Bipolar disorder that creates a de novo TLX-binding site (Fig. 6d). Our success in mapping known disease-causing SNPs that affect NR binding, lends support to the hypothesis that SNPs identified by MinSeqs, such as rs7138803 reveal masked NR sites that were missed by traditional motif mapping algorithms (Fig. 6e). Incidentally, rs7138803 is particularly interesting because it implicates the unusual ESRR binding site with overlapping monomeric sites (highlighted in Fig. 2a) as a functional site with a role in predisposition to the metabolic syndrome. Consistent with our analysis, a functional role for such overlapping ESSR sites is supported by allelic imbalance analysis^52^ that identifies an atypical ESSRA binding site created by a C to T conversion in rs521991 (Supplementary Fig. 10). Conservatively, the impact of the 771 manually curated SNPs on NR binding (gain or loss) is displayed as a heat map of differing enrichment and grouped by disease class or trait (Fig. 6f and Supplementary Data 11). When compared to the richly annotated RegulomeDB database^53^, where only 120 of the 771 SNPs were annotated, ^53^we now implicate SNP-induced alteration of masked NR binding sites in a far wider set of diseases. More broadly, a table encapsulating similar disease associations of the 5192 SNPs in the GWAS Catalog that create or disrupt potential NR sites is presented in Supplementary Data 12 and Supplementary Fig. 11.

## Discussion

NRs play vital roles in all aspects of human biology and are among the most successfully drugged class of transcription factors encoded by the human genome. Yet, DNA sequence preferences of a substantial fraction of the human NR superfamily have not been determined nor have the impacts of interfacial protein interactions or small molecule ligands explored in a systematic manner. Moreover, current algorithms overlook unconventional binding sites and therefore poorly annotate biologically functional binding sites in vivo. To address these limitations, we first obtained high-resolution DNA–NR interactomes, using full-length NRs, by themselves and in complex with their cognate ligands and obligate partner RXRA. Next, to identify hidden binding sites, we developed a new algorithm, *MinSeq Find*, that is based on the principles of digital systems design optimization.

The *MinSeq Find* algorithm, (i) unmasked a large constellation of distinct binding sites in our newly determined NR-DNA Interactomes, (ii) unmasked such sites in published NR–DNA Interactomes, (iii) revealed the contribution of small molecule ligands and an obligate dimer partner, (iv) defined the role of DNA shape in selective binding amongst different members of the NR subfamilies, (v) annotated substantive fraction of cell-type specific genomic-binding sites in vivo, and (vi) revealed druggable NRs as potential regulators of disease-causing SNPs whose mode of action had thus far remained hidden.

This study provides fundamental new insights into the non-canonical modes of DNA recognition by NRs and as such it serves as a valuable resource for accurately mapping cell type/tissue-specific genomic binding profiles of human NRs. Moreover, we assign NR binding properties to 8–14% disease-associated non-coding SNPs whose mode of action thus far was completely opaque. Our collection of MinSeqs of all human NRs is also an invaluable resource for the clinical community and sets the stage for hypothesis-driven repurposing of NR drugs for a plethora of diseases that are linked to over 52,106 non-coding SNPs in the ClinVar database.

In essence, MinSeqs provide means to capture the full spectrum of TF-DNA binding modes, bypassing the limitations of traditional compressed PWM-based motifs on one hand and the comprehensive collections of k-mers from an entire DPI on the other. MinSeqs readily distinguish between closely related motifs while accommodating binding sites of variable length and type. They also capture the contributions of non-contacted sequences, including spacer and flanking sequences, on shape and affinity for the core binding site (Figs. 2a, 3a–e and Fig. 4a). The current version of *MinSeq Find* algorithm was developed for high throughput DPI datasets. We are currently working on optimizing it to extract hidden binding modes from low- to medium-throughput DPI data. More globally, *MinSeq Find* algorithm provides a tool to comprehensively mine the publicly available high throughput DPIs of over 1000 transcription factors^54,55^. Just as we demonstrated here for the NR superfamily, MinSeqs extracted from the publicly available TF-DNA datasets will reveal new binding modes and help annotate a substantial fraction of the non-canonical binding sites across the genome. Moreover, MinSeqs will enable the annotation of currently unassigned orphan SNPs as potential TF binding sites, thereby identifying TFs that contribute to regulatory dysfunction and the onset of numerous human diseases. Thus, extending beyond the NR superfamily, we define a path to annotate hidden regulatory elements in genomes and elucidate the biological functions of transcription factors while simultaneously laying the foundations for the new era of precision medicine.

## Methods

### Cloning and expression

Plasmids containing N-terminus HaloTag fusions of human NRs were obtained from Promega, as part of their Kazusa collection. Plasmid details can be found in Supplementary Table 1. HEK293T cells were grown in DMEM media supplemented with 10% FBS at 37 °C in an atmosphere of 5% CO_2_. Cells were transiently transfected using FuGENE HD Transfection Reagent (Promega, Madison, WI, USA) following the manufacturer’s protocol. After 24–48 h at 37 °C and 5% CO_2_, cells were washed with ice-cold PBS, scraped, and collected in a conical centrifuge tube. Cells were lysed in Mammalian Lysis Buffer (50 mM Tris-HCl pH 7.5, 150 mM NaCl, 1% Triton X-100, 0.1% sodium deoxycholate) supplemented with protease inhibitors. Cell lysates were centrifuged, the clear supernatant was transferred to a clean microcentrifuge tube, flash frozen in N_2_(*l*), and stored at −80 °C. Expression of the HaloTag fusions was confirmed by SDS-PAGE.

### Cognate site identification (CSI) by high-throughput-SELEX (HT-SELEX)

Cognate binding sites for HaloTag-human NR transcription factors were determined by HT-SELEX. A DNA library with a 20 bp random region flanked by constant sequences to allow PCR amplification was used (Supplementary Fig. 1). In vitro selections were performed by incubating the DNA library (100 nM in 20 μL) with cell lysate overexpressing a HaloTag-NR in binding buffer (25 mM HEPES (pH 7.4), 80 mM KCl, 0.2 mM EDTA, 1 mM MgCl_2_, 0.1 mM ZnSO_4,_ 2.5 mM DTT, 50 ng/μl poly dI-dC, 0.1% BSA) for 1 h at room temperature. When included, NR ligands were added to a final 100 nM concentration. HaloTag-NR-bound DNA was enriched using Magne® HaloTag® beads (Promega) following the manufacturer’s specifications. After covalent immobilization on the magnetic beads, three quick washes with 100 µL of ice-cold binding buffer were performed to remove unbound DNA. The magnetic beads were resuspended in a PCR master mix (EconoTaq® PLUS 2X Master Mix, Lucigen) and the DNA was amplified for 18 cycles. Amplified DNA was purified with QIAquick PCR Purification Kit (QIAGEN), quantified by UV absorbance at 260 nm, and used for subsequent binding rounds. A total of 3 rounds of selection were performed. After selection, an additional PCR was done to incorporate a 6 bp ‘barcode’ and Illumina sequencing adapters. The starting library (Round 0) was also barcoded. Samples were combined and sequenced in an Illumina HiSeq 2000 instrument.

### Sequencing data

Reads obtained from Illumina sequencing were de-multiplexed by matching the corresponding 6-bp barcode and truncated to obtain 20 bp derived from the random region (Supplementary Fig. 1). On average, we obtained more than 800 K reads per barcode. We use the Illumina sequencing reads for (a) just the library, (b) the enriched library with pulldowns done just with Halo beads, and (c) the enriched library with pulldowns done with the TF with Halo bead with/without the ligand with/without partner protein (RXRA). Three rounds of enrichment were done for (b) and (c) and each round was followed by a PCR step for exponential enrichment.

### Defining MinSeqs

A $\left({{{{{\boldsymbol{k}}}}}}{{{{{\boldsymbol{,}}}}}}{{{{{\boldsymbol{g}}}}}}{{{{{\boldsymbol{,}}}}}}{{{{{\boldsymbol{l}}}}}}\right)$**-MinSeq** is a $k$-mer DNA sequence followed by a spacer of the length of $g\ge 0$, followed an $l$-mer sequence. For example, the sequence AACGNNNGCTTA is a $\left(4,\,3,\,5\right)$-MinSeq with a $4$-mer AACG is followed by spacer NNN (where N can take any value A, C, G, or T) which is in turn followed by a $5$-mer GCTTA.

Protein–DNA binding data can be captured by MinSeqs into a sequence enrichment or affinity format using *MinSeq Find* algorithm, where sequences are of different lengths and exhibit gaps as well. Check Supplementary Data 1 for sequencing data analysis using *MinSeq Find* algorithm.

### *MinSeq Find* algorithm outline

Given: Raw $N$-mer bound data and PAGLO model probabilities of library or Halo-bead, against which we normalize our bound data.

Find: MinSeqs ${{{{{\boldsymbol{(}}}}}}{{{{{\boldsymbol{k}}}}}}{{{{{\boldsymbol{,}}}}}}{{{{{\boldsymbol{g}}}}}}{{{{{\boldsymbol{,}}}}}}{{{{{\boldsymbol{l}}}}}}{{{{{\boldsymbol{)}}}}}}$ above threshold ${C}^{T}$ counts and corresponding enrichment.

1. Bin and count each sub-sequence $x$ of each raw sequence in MinSeq $\left(k,g,{l}\right)$ format as ${C}_{b}\left(x\right)$.
2. Discard MinSeqs with less than ${C}^{T}$ count threshold (refer to section “Poisson distributed reads and threshold cutoff for sequences” Supplementary Data 1).
3. Get the estimated counts of each MinSeq above ${C}^{T}$ threshold in the library using PAGLO model ${C}^{*}\left(x\right)$.
4. Then calculate estimated enrichment (refer to “PAGLO model for MinSeqs” Supplementary Data 1) from the following formula:

1 $${E}^{*}\left(x\right)=\frac{{C}_{b}\left(x\right)/{T}_{b}}{{C}^{*}\left(x\right)/T}$$

Where ${T}_{b}$ and $T$ are the total number of sequence reads in the bound samples and the library samples, respectively. ${E}^{*}\left(x\right)$ serves as a full or uncompressed set of MinSeqs.

5. These enrichment values are weighted based on their length (refer to “Weighted Enrichment of MinSeqs” Supplementary Data 1). They are then rank-ordered by their weighted enrichment.
6. Further to reduce redundancy the MinSeqs are compressed using modified OMP (Supplementary Data 1).
7. The MinSeq set (compressed or full) can be used to obtain PWM sequence logos as well as to display binding (Online methods).

### MinSeqs to score sequences

Consider a sequence $v$-mer of length $v$ nucleotides to be scored using a set of MinSeqs with given enrichments. MinSeqs are of type $\left(k,g,l\right)$ with a maximum length $n$= $k+g+l$. A moving window of length $n$ is used to score the $v$-mer sequence, resulting in $v-n+1$ sub-sequences of length $n$ ($v\ge n$). One of the methods is assign the score to the $n$-mer as **maximum** enrichment among all the MinSeqs contained in that $n$-mer. A maximum of scores for all those $\left(v-n+1\right)$ $n$-mers is used as the final MinSeq score. Different methods can then be used to score these $\left(v-n+1\right)$ $n$-mers from the $v$-mer sequence explained in detail in Supplementary Data 1.

### Molecular dynamics simulations

The starting structure for DNA-bound GR tetramer simulation was prepared from existing crystal data of GR dimer:DNA complex (PDBid:3g6q). All-atom MD simulations were performed for 500 ns in an explicit solvent under constant pressure and temperature conditions. The solvent box dimensions were chosen such that any DNA/protein atom was at least 15 Å away from the box surface, preventing unwanted interactions with its image in translated unit cells. A required number of Na^+^ and Cl^−^ counterions were added to first neutralize the systems and then attain a physiological salt concentration of 150 mM. The solvated DNA-bound GR tetramer complex was equilibrated using an alternating heating and cooling protocol to enable optimal intermixing of the solvent and ions around the biomolecular complex. Following equilibration, a production MD run was performed using the pmemd CUDA version of the Amber14 MD suite^56^. The Amber OL15 force field for DNA, which incorporates parmbsc0 along with dihedral (beta, epsilon, zeta, chi) corrections, and the ff14SB force field for protein were adopted. Simulations were performed using a 2 fs time step, and snapshots were saved from the simulation for analysis every 2 ps. To enable volume variation, simulations were performed in an NPT ensemble using the Berendsen thermostat and barostat. SHAKE was used to constrain bond lengths between heavy atoms and hydrogens. Analyses of MD trajectories were carried out using in-house codes, NUPARM software suite^57^, and the cpptraj module in Amber 18^56^. The stability of the DNA-bound GR tetramer complex was tested by calculating the RMSF of the backbone Cα atoms compared to the PDB-derived initial model.

### Sequence specificity landscape (SSL) or specificity and energy landscape (SELs)

Sequence specificity landscapes (SSLs) or specificity and energy landscapes (SELs) provide a three-dimensional display of high-throughput protein–DNA (or protein–RNA) binding data through a series of concentric rings^15,43,58^. The height of each color-coded peak corresponds to the binding intensity, which can be measured by different experimental platforms. SEL for binding of all k-mers is built around a seed sequence/motif as a reference, relative to which sequences are arranged on SEL. The seed sequence is derived from the top-scored MinSeq or PWM and whose length has to be smaller than k. In SEL, the innermost ring (0 mismatch or 0 hamming distance ring) contains sequences that contain a perfect match to the given seed sequence and the next ring out (1 mismatch or 1 hamming distance ring) contains sequences that differ from the seed sequence at a single position. The subsequent rings, going outward, represent increasing mismatches from the seed sequence. The sequences are arranged clockwise on each ring. The sequences in the center ring are sorted by the nucleotides flanking the seed, and then by the position of the seed in the original sequence. In mismatch rings sequences are arranged first by the positions of the mismatches, and then by the alphabetical order of substituted nucleotide (A, C, G, or T) at the mismatch and then by flanking bases and position of seed. Sequences are arranged in such a way that similar sequences appear together and no sequence is repeated. The binding intensity for all k-mer sequences for SEL is obtained using MinSeqs in this paper.

### Gapped sequence specificity and energy landscapes (gapped-SELs)

We developed gapped SELs to display binding enrichment/intensity for DNA binding proteins which prefer sequences with multiple gaps or spaces in the form of Ns (i.e., any nucleotide) like NRs in this study-

1. First, a seed sequence is chosen as a combination of two monomers. Monomers are chosen from top-ranked MinSeqs or PWMs. The monomers are combined with multiple orientations, and different gaps are placed between monomers to construct multiple seeds. For example, we take RGGTCR as our starting monomer for RXRA, since RXRA likes to bind RGGTCR with multiple gaps and orientation, by adding multiple gaps between two such monomers in an inverted repeat fashion (Supplementary Fig. 8) with Ns surrounding those we get - NRGGTCR-YGACCYN, NRGGTCR-n-YGACCYN, NRGGTCR-nn-YGACCYN, and so on as seed for inverted repeats of GGTCA with gap 0, 1, 2, and so on. Similarly, seeds for direct repeats and everted repeats are also obtained.
2. All the sequences matching the seeds are then obtained by replacing capital Ns (or other degenerate nucleotides like R = A/G, etc.) with A, C, G, and T. In the above example there are total 4^4^ = 256 different sequences matching each seed.
3. A 3D plot for sequences for an exact match or zero mismatch sequences is plotted here. Sequences matching the original seeds are plotted, and all the sequences corresponding to gap = g are arranged along the *x*-axis with *Y* coordinate = g. The sequences along the *x*-axis are plotted in order as in Supplementary Fig. 8. The same order of sequences is followed for all gaps along the *x*-axis, example AAGGTCA-CGACCCA and AAGGTCA-n-CGACCCA will have same X-coordinate, but have *y* = 0 and *y* = 1 *Y*-coordinate respectively. After deciding *X* and *Y* coordinates, the enrichment or binding intensity is plotted at that coordinate with height and color representative of it. The binding intensity of all the sequences for Gapped-SEL is obtained using MinSeqs for this paper.

### DiSEL and gapped-DiSEL

Differential sequence specificity and energy Landscapes or DiSEL are SEL landscapes that are plotted to compare the DNA binding of two different proteins or the same protein in two different conditions. First scales for the enrichment/binding data for two samples are normalized by setting maximum binding intensity equal to 1 for both and then subtracting one from the other to get the final difference in binding, which is then plotted as a gapped SEL or SEL called as gapped-DiSEL or DiSEL. DiSEL of A over B displays binding preferred by A in comparison to B.

### Receiver operating characteristic (ROC) analysis using peak assign

The genomic sequence underlying ChIP-Seq peaks were used to generate ROC curves. In this analysis, ChIP-Seq peaks were taken as positives, and two random permutations (moving positions of DNA bases) of each peak were used as known negatives. Each peak (all positives and negatives) was scored using MinSeqs (or PWMs). A ROC curve between the false-positive rate (FPR) and true-positive rate (TPR) was plotted by varying a moving threshold, positive peaks scored above that threshold (true positives) are used to get TPR (true positives over total positives) and negative peaks scored above threshold (false positives) were used to get FPR (false positives over total negatives). The area under ROC (AUROC) curve is used to analyze how well in-vitro data predict a set of ChIP-Seq peaks. Where AUROC = 1 means complete prediction and AUROC = 0.5 means random prediction. AUROC is used to do a first level of comparison between two different sets of data in predicting a set of ChIP-Seq peaks. For deeper peak-by-peak comparison (which peak can be predicted by whom), we assigned a score S to each peak. S for a peak is defined as the maximum value of TPR/ (TPR + FPR) at which a true positive peak was detected as a positive peak (similar to precision TP/(TP + FP)). Score S represents the predictability of each peak using a given DNA binding data as opposed to the randomized region when considering all the ChIP-Seq peaks. The scale varies from the 1 (highest predictability i.e., peaks detected as positive at FPR = 0) to 0.5 (lowest predictability, peaks detected as positive at FPR = TPR). The S score for a given set of ChIP-Seq peaks is represented as a heatmap. In the case of random prediction i.e., a diagonal ROC curve (AUC–ROC = 0.5), there will not be a single peak that will be assigned as positive detected even at 0.6 S score (marginally better than random), had we chosen FPR cutoff as our metric we would get 10% peaks detected positive at FPR cutoff 0.1 (which is considered as good prediction). Thus, we used the S score instead of FPR cutoff here (which is similar to positive predictive value or precision).

### Clustering analysis

A union of 100 top-ranked MinSeqs (chosen by OMP) for each pair of protein–DNA binding data was used to get Pearson’s correlation coefficient *r*, which is used as a measure of similarity between the two. Dendrograms and heatmap are then plotted by unsupervised hierarchical clustering of such pair-wise binding profile (correlation coefficient r) using function *heatmap.2* in the R-package gplots with Euclidean distance function. Note, the MinSeqs which existed in the first DNA–protein binding set but not in the second set to which it is compared, then their scores are calculated for the second protein from the rest of the MinSeq set.

### Sequence Logos

Sequence Logos are constructed from the MinSeq set using the following iterative steps:

Step 1: MinSeqs are first sorted by their weighted enrichment (refer to “Weighted Enrichment of MinSeqs” Supplementary Data 1).

Step 2: Rank 1 MinSeq from the table is used as a seed to derive the first PWM (Supplementary Fig. 2f).

Step 3: Different algorithms can be used to derive PWM from the seed MinSeq. We extended the seed by adding 6 bp of N’s on both ends (N corresponds to any nucleotide). One at a time, at each position, an existing nucleotide is swapped with A, C, G, and T nucleotides. These sequences were then counted in the raw data and normalized to the library model to obtain enrichment. These enrichment values corresponding to each A, C, G, and T nucleotide at each position are used to obtain the position frequency matrix, which is used here as a position weight matrix (PWM).

Step 4: Enrichment estimates were made for all the MinSeqs using all PWMs obtained till this iteration. Residual enrichment was obtained after subtracting out the maximum estimated enrichment from the score of each MinSeqs (refer to “MinSeqs to score sequences” Supplementary Data 1).

Step 5: Resort and go to step 2 until maximum iterations are reached or minimum residual enrichment is achieved.

All the PWM logos were built using ceqlogo command from MEME suite^59^. NR PWMs from other computational methods were also used to compare to those obtained by MinSeqs^45,60–62^.

### ChIP-seq data overlap

Overlapping genomic regions of ChIP-Seq peaks were determined using bedops tool^63^.

### Single nucleotide polymorphism (SNP) scoring using SNP Align

Curated 5592 human SNPs associated with a disease or quantitative traits by GWAS were obtained from Maurano et al.^50^. Effect of each SNP on DNA binding of NR is estimated as log fold change in the enrichment due to SNP. First, MinSeqs were utilized to get enrichment of the sequence flanking (±20 bp) the SNP—for both, the reference allele (hg19) as well as the alternate allele (SNP). Next, log fold change in enrichment is calculated using ${\log }_{2}\left(\frac{E\left({{{{\rm{alt}}}}}.{allele}\right)+\eta }{E\left({{{{\rm{Ref}}}}}.{allele}\right)+\eta }\right)$, where *E*(alt.allele) and *E*(Ref.allele) are enrichment values as estimated by MinSeqs for the reference allele and the alternate allele, respectively. $\eta$ is added to address the unintended issues that arise as a consequence of division by small numbers. (*η* = least of the two, absolute enrichment $10$ and $10\%$ of maximum enrichment). Overall, a total of 771 SNPs crossed the threshold of 2-fold change for at least one NR^53^. Similarly, in Supplementary Fig. 11, 5192 SNPs were predicted to be associated with DNA binding of NR data from a set of 53,039 non-coding GWAS SNPs and clustered using heatmap.2 in R and ordered SNPs on the basis of Euclidean distance function in R. ClinVar SNPs were downloaded on 2022-04-16 from ftp.ncbi.nlm.nih.gov/pub/clinvar/, we removed missense or nonsense mutations and used SNPs with 2 alleles only. GWAS SNPs were downloaded on 2018-10-29 from https://www.ebi.ac.uk/gwas/docs/file-downloads, we removed missense or nonsense mutations to get a set of 82,733 SNPs, and from that we used 53,039 SNPs with 2 alleles only.

### Reporting summary

Further information on research design is available in the Nature Portfolio Reporting Summary linked to this article.

## Supplementary information

Supplementary Information

Description of Additional Supplementary Files

Supplementary Data 1

Supplementary Data 2

Supplementary Data 3

Supplementary Data 4

Supplementary Data 5

Supplementary Data 6

Supplementary Data 7

Supplementary Data 8

Supplementary Data 9

Supplementary Data 10

Supplementary Data 11

Supplementary Data 12

Reporting Summary

## Data Availability

The Sequencing data generated in this study are available in the National Center for Biotechnology Information (NCBI) database under BioProject PRJNA729962. The HT-SELEX data used in this study from Jolma et al. 2013 and Yin et al. 2017 are available in the European Nucleotide Archive (ENA) under accession code ERP001824, ERP001826, and PRJEB9797^16,17^. The SelexGLM data used in this study are available in the Sequence Read Archive (SRA) under BioProject PRJNA379022^18^. The SMiLE-seq data used in this study are available in the Sequence Read Archive (SRA) under BioProject PRJNA318578^19^. The SNP data used in this study are from Maurano et al.^50^. Supplementary Table S2, the NCBI-supported ClinVar database and NHGRI-supported GWAS Catalog. LoVo cell line ChIP-seq peak data used in this study are available in the NCBI database under GEO accession code GSE49402^47^. ENCODE ChIP-seq peak data used in this study are available on the UCSC ftp server (ftp://hgdownload.cse.ucsc.edu/goldenPath/hg19/encodeDCC/wgEncodeAwgTfbsUniform)^46^. AR and GR ChIP Exo peak data from the U2OS cell line used in this study are available in the European Bioinformatics Institute (EBI) database under accession code E-MTAB-9616^33^. RXRA ChIP-seq peak data for the HEPG2 and H1-hESCs cell lines used in this study are available in the CistromeDB^64^ under id 46242 and 46192^44^. The crystal structure data used in this study are available in the Protein Data Bank (PDB) database under accession codes 3g6q and 3g9i^38^. Source data are provided in this paper.

## Source data

Source Data

## Code availability

Custom code with instructions to run *MinSeq Find* algorithm is available at GitHub: https://github.com/dev11ume/MinSeq-Find^65^.
