# Supplementary material for: Hidden modes of DNA binding by human nuclear receptors
Source: Nat Commun. 2023 Jul 13;14:4179. doi: 10.1038/s41467-023-39577-0 (PMC10345098; doi:10.1038/s41467-023-39577-0)
Supplement: Supplementary file 1 — Supplementary Information [file 41467_2023_39577_MOESM1_ESM.pdf]

# Supplementary Information for

# Hidden Modes of DNA Binding by Human Nuclear Receptors

**Table of contents**


- **Supplementary Figures 1-11**
- **Supplementary Table 1-3**
- **Supplementary References**

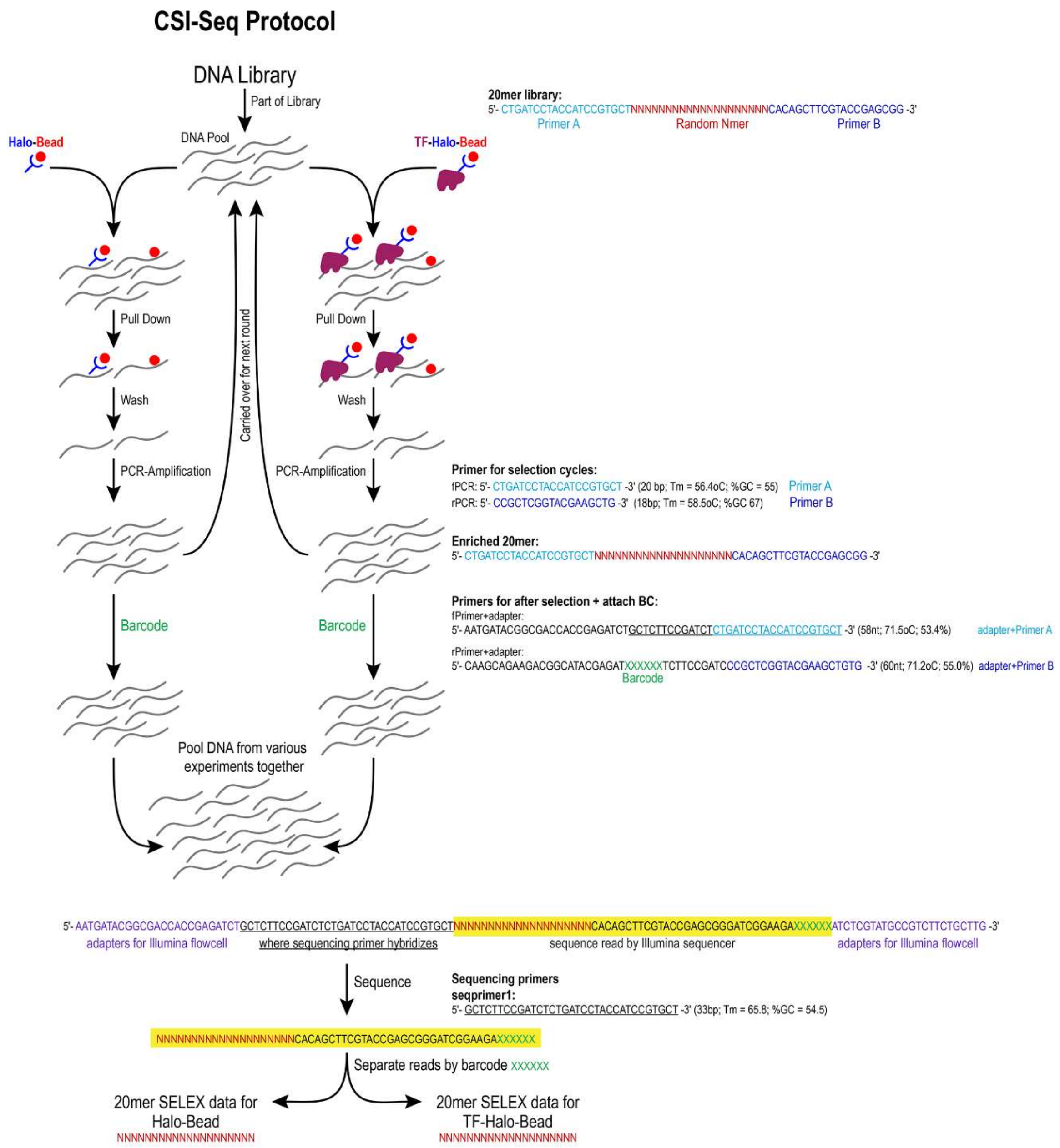


**Supplementary Figure 1 | Cognate site identification by HT-SELEX-seq** - A HEK293 cell lysate over expressing a HaloTag-hNR fusion was equilibrated with a 20bp randomized DNA library. The central 20bp randomized region (NNNNNNNNNNNNNNNNNNNN) is flanked by 20bp constant regions that enable PCR amplification (Primer A: CTGATCCTACCATCCGTGCT & Primer B: CACAGCTTCGTACCGAGCGG). HaloTag magnetic beads were added to isolate bound DNA, washed 3 times, and the DNA was amplified by PCR. Amplified DNA was column purified, quantified by UV/Vis, and used for two additional binding rounds for a total of 3 selection rounds. In parallel, HT-SELEX was

performed using HEK293 lysate overexpressing the HaloTag protein. A final PCR reaction was performed on all samples to incorporate Illumina sequencing adapters and a unique 6bp barcode. Samples were combined and sequenced using Illumina technology. After sequencing, reads were computationally de-multiplexed according to the corresponding barcodes and only the 20bp random region was retained for analysis.

# Computational pipeline for normalization against gapped Markov Model of library

**a**

20mer SELEX data for Halo Bead

5′-NNNNNNNNNNNNNNNNNNNN-3′

Create position specific probability matrix for 8th order Markov Model

Model creation. Example - for sequences starting at position 1

| | |
|---|---|
| example seq: | ACGTACGTACGTACGTACGT |
| Counts of 8mer (1-8) | ACGTACGT |
| MM Prob 9th given 1-8 | ACGTACGTA |
| MM Prob 10th given 2-9 | CGTACGTAC |
| MM Prob 11th given 3-10 | GTACGTACG |
| | ...... |
| MM Prob 20th given 12-19 | TACGTACGT |

**b**

20mer SELEX data for Halo Bead

5′-NNNNNNNNNNNNNNNNNNNN-3′

Create gapped position specific probability matrix for 8th order Markov Model

Model creation. Example - for sequences starting at position 1

| | |
|---|---|
| example seq: (gap at 6 & 7) | ACGTACGTACGTACGTACGT |
| Counts 8 mer (1-5 + 8-10) | ACGTA__TAC |
| MM Prob 11th given 1-5 + 8-10 | ACGTA__TACG |
| MM Prob 12th given 2-5 + 8-11 | CGTA__TACGT |
| MM Prob 13th given 3-5 + 8-12 | GTA__TACGTA |
| | ...... |
| MM Prob 20th given 12-19 | TACGTACGT |

**c**

20mer SELEX data for TF+Halo+Bead

NNNNNNNNNNNNNNNNNNNN

Counting all 10-16mers without gaps

Counting in TF sample. Example - 10mer **ACGTACGTAC**

| NNNNNNNNNNNNNNNNNNNN | Counts |
|---|---|
| ACGTACGTAC | 102 |
| ACGTACGTAC | 123 |
| ACGTACGTAC | 115 |
| ACGTACGTAC | 118 |
| ... | |
| ACGTACGTAC | 125 |

A= Total counts of **ACGTACGTAC** in TF data= 1277

Expected counts in Halo + Bead

| NNNNNNNNNNNNNNNNNNNN | Expected counts |
|---|---|
| starting at position 1 | |
| ACGTACGTAC | =70*0.22*0.27= 4.16 |
| ACGTACGT | 70 |
| ACGTACGTA | 0.22 |
| CGTACGTAC | 0.26 |
| starting at position 2 | |
| ACGTACGTAC | =86*0.28*0.23= 6.50 |
| ACGTACGT | 86 |
| ACGTACGTA | 0.28 |
| CGTACGTAC | 0.23 |
| ACGTACGTAC | |
| ACGTACGTAC | |
| ... | |
| ACGTACGTAC | |

B= Total Expected counts of **ACGTACGTAC** in Halo+Bead = 57.23

C= Total 20mer counts in TF data= 500,0000

D= Total 20mer counts in TF data in Halo+Bead = 2,000,000

Then,

Enrichment = (A/B)/(C/D)

= (1277/57.23)/(500,000/2,000,000)= 89.25

if A<20 assign NA (Not available)

**d**

20mer SELEX data for TF+Halo+Bead

NNNNNNNNNNNNNNNNNNNN

Counting all 10-16mers with gaps

Counting in TF sample. Example - 10mer **ACGTA__TACGT**

| NNNNNNNNNNNNNNNNNNNN | Counts |
|---|---|
| ACGTA__TACGT | 102 |
| ACGTA__TACGT | 123 |
| ACGTA__TACGT | 115 |
| ACGTA__TACGT | 118 |
| ... | |
| ACGTA__TACGT | 125 |

A= Total counts of **ACGTACGTAC** in TF data= 1012

Expected counts in Halo + Bead

| NNNNNNNNNNNNNNNNNNNN | Expected counts |
|---|---|
| starting at position 1 | |
| ACGTA__TACGT | =70*0.22*0.27= 4.16 |
| ACGTA__TAC | 70 |
| ACGTA__TACG | 0.22 |
| CGTA__TACGT | 0.26 |
| starting at position 2 | |
| ACGTA__TACGT | =86*0.28*0.23= 6.50 |
| ACGTA__TAC | 86 |
| ACGTA__TACG | 0.28 |
| CGTA__TACGT | 0.23 |
| ACGTACGTAC | |
| ACGTACGTAC | |
| ... | |
| ACGTACGTAC | |

B= Total Expected counts of **ACGTACGTAC** in Halo+Bead = 46.22

C= Total 20mer counts in TF data= 500,0000

D= Total 20mer counts in TF data in Halo+Bead = 2,000,000

Then,

Enrichment = (A/B)/(C/D)

= (1012/46.22)/(500,000/2,000,000)= 87.58

if A<20 assign NA (Not available)

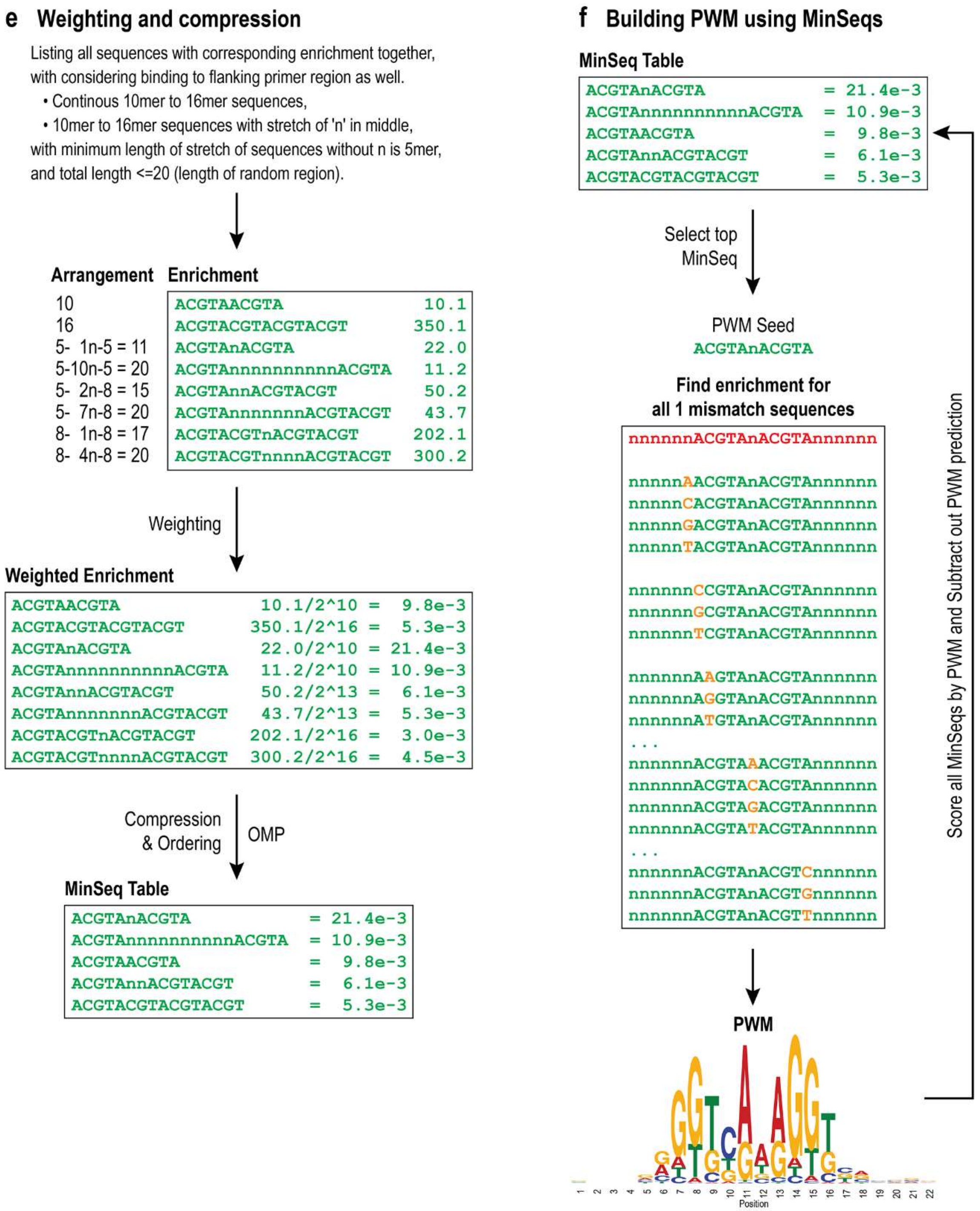


**Supplementary Figure 2 | *MinSeq* Find analysis pipeline with Position Associated Gapped LOcation-specific (PAGLO) model.** In the example here 8th order Markov Model (MM) is constructed to capture initial DNA bias in pseudo random library and any possible DNA binding for Halo+Bead combination. 8th order MM gives out probability of 9th nucleotide A, C, G or T given the previous 8 bases. MM constructed here has additional properties, it is - *i)* Position

Associated (Inhomogeneous MM) – in the random library of length 20, probability of nucleotide at position 9$^{th}$ given 1-8 position will be different than, probability of nucleotide at position 10$^{th}$ given 2-9 position and so on; *ii)* Gapped – since MinSeqs accommodate gaps or spaces in the form of Ns (N=A,C,G or T), model is constructed to tolerate gaps too, thus it also incorporates probability of nucleotide say at position 12$^{th}$ given nucleotide 1 to 11 with 3 gaps or spaces in the form of N; *iii)* Location specific – in the model location of gap is also incorporated eg. probability of nucleotide say at position 12$^{th}$ given nucleotide 1 to 11 with 3 gaps at position 7, 8 and 9. MM is then used to get expected counts for Halo+Bead to normalize for TF+Halo+Bead, where TF is transcription factor. **a,** Example of sequence without gaps – Markov Model starting from position 1 is shown. In purple is 8mer, for which count is used and further probabilities are used to get like A at position 9 given 1-8 and so on. **b,** Example of sequence with gaps - Example showing gap at position 6 & 7. **c**, Calculating enrichment for 10-16mer without gaps- First counting sub-sequences of length 10-16 with no gaps for TF+Halo+Bead and then calculating expected count for the same sequence for Halo+Bead using PAGLO model as shown in A. **d,** Enrichment for 10-16mer with gaps. **e,** Weighted enrichment of all sub-sequences, followed by MinSeq compression- First enrichment of all sub-sequences (10-16mer with or without gap) is combined together in a list. Then enrichment is weighted by multiplying by 2^(number of nucleotides excluding N or n). Orthogonal Matching Pursuit (OMP) is used for compression and select MinSeq ordered according to their weighted enrichment. **f,** Iterative process of PWM extraction from MinSeqs- Top MinSeq is selected as the seed to get PWM. Seed is then extended by 6bp on each side by adding ‘n’ to consider flanking binding. Enrichment corresponding to seed and sequences exhibiting 1 mismatch to the seed are used to construct PWM. Weight of a nucleotide at a position is calculated to be the enrichment of sequence with that nucleotide at that position. ‘n’ is substituted with A, C, G & T one at a time and enrichment is used as weights in PWM at those positions. Next, we subtract out the enrichment predicted by PWM from calculated enrichment (or residual enrichment) for all MinSeqs. Then choose the next top MinSeq from the weighted residual enrichment for next PWM.

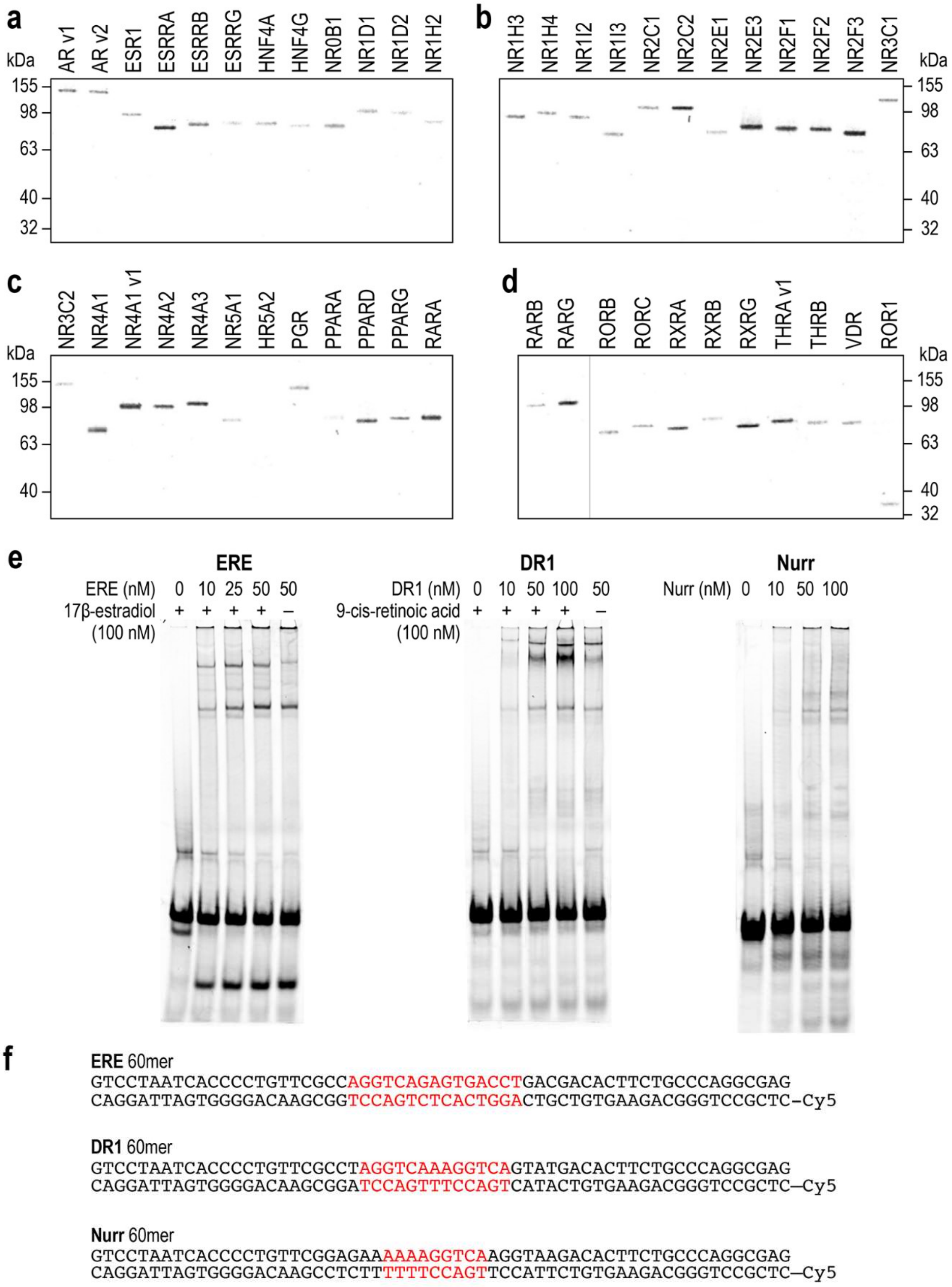


**Supplementary Figure 3 | Active HaloTag-hNRs were overexpressed in HEK293 cells. a-d,** Expression of HaloTag fusions of full-length human nuclear receptors. SDS-PAGE of HaloTag-

hNRs. 10 mL of cell lysate were reacted with HaloTag TMR (Catalog number G8252; Promega Corporation, USA). Products were resolved by denaturing SDS-PAGE and imaged in a fluorescence scanner. **e,** Representative electrophoretic mobility-shift assay (EMSA) used for testing binding activity of HaloTag human nuclear receptors ESR1 (left), RXRA (middle), and NR4A2 (right) to their respective known cognate sites. Cell lysate of HEK293 cells overexpressing a HaloTag-hNR was equilibrated with 150 nM of Cy5-labeled oligonucleotide in binding buffer (25 mM HEPES, 80 mM KCl, 0.2 mM EDTA, 1 mM MgCl2, 0.1 mM ZnSO4, 1 mM DTT, 0.05% NP-40, 200 ug of bovine serum albumin, 1 μg of poly(dI:dC). Samples were equilibrated for 1 hour at room temperature. Total volume of the binding reaction was 20 μL. Protein-DNA complexes were resolved in a 6% acrylamide non-denaturing gel ran at 130 V at 4 °C. Gels were imaged in a GE Typhoon FLA 9000 fluorescence scanner. For RXRA, this was one of two independent DNA-binding tests. **f,** Sequences of Cy5-labeled oligonucleotides used in EMSA experiment.

**Preferences for all NRs including those done by other labs and various rounds (Clustergram and spacing preferences)**

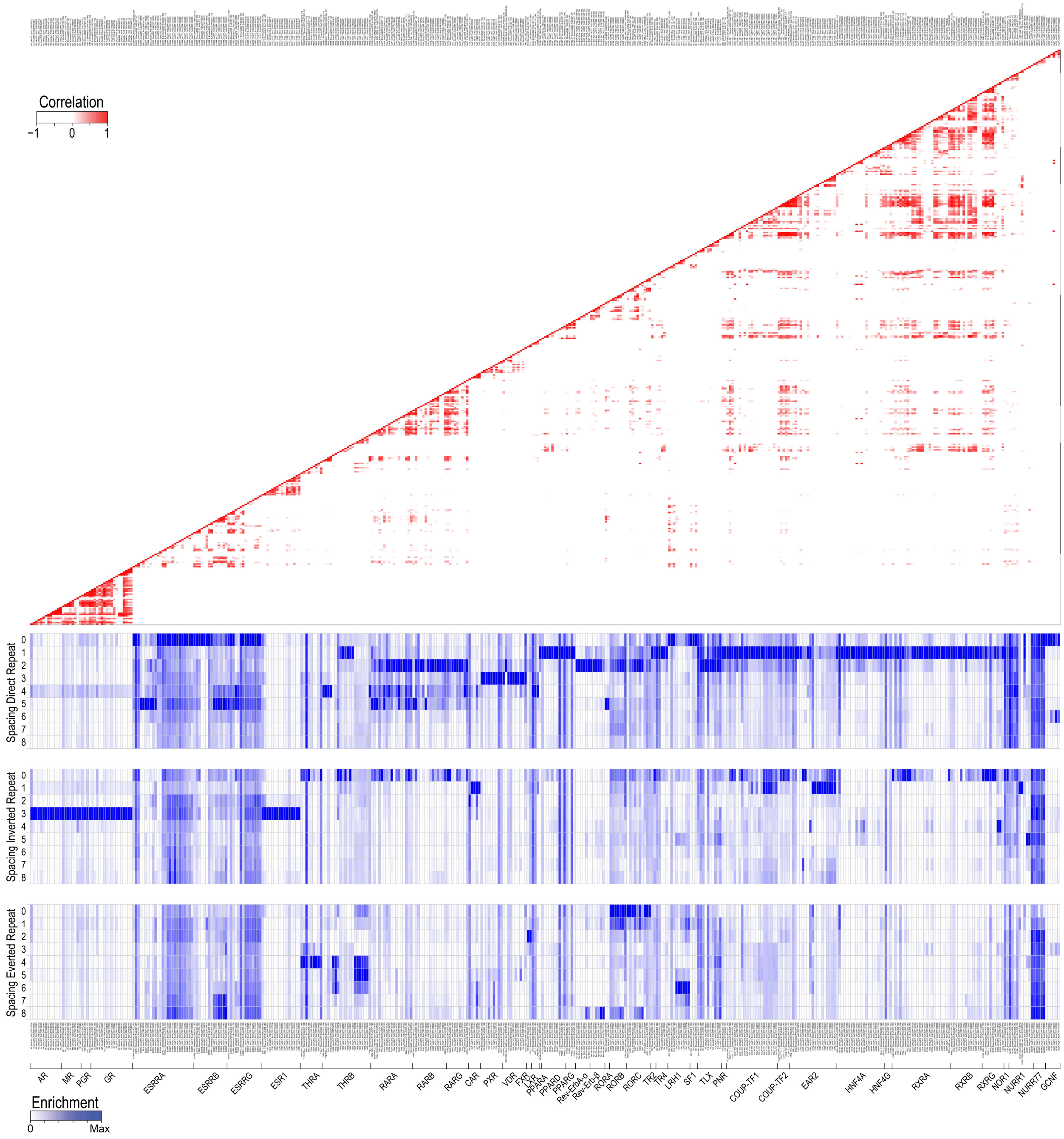


**Supplementary Figure 4 | Heatmap and Clustergram for all NRs** - similar to **Fig. 2c-d** for total 412 DPIs of NRs including different HT-SELEX rounds and replicates (127 from this study, 13 samples of SelexGLM[1], 112 samples from Jolma et al. 2013[2] and 160 from Yin et al. 2017[3] ). Correlations of binding enrichment of each pair (row and column) of DPI is plotted, with shades of white to red as 0 to 1 correlation and white for correlation 0 to -1. The proteins

are ordered on the basis of their phylogeny. (Source data are provided as a Source Data file. Designed by Laura Vanderploeg.)

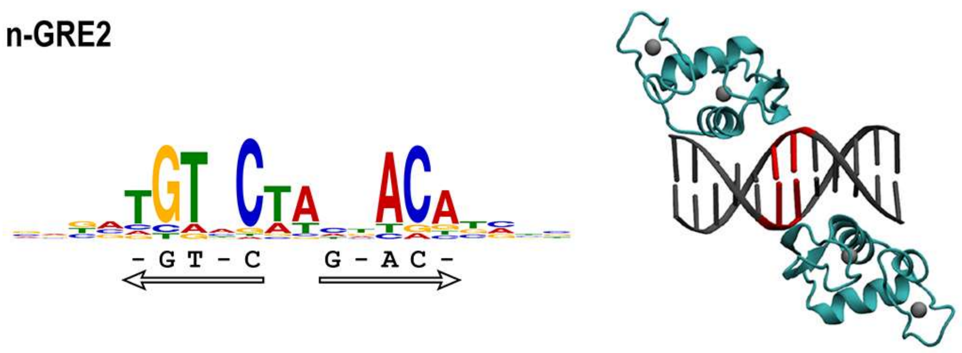


**Supplementary Figure 5 | n-GRE1 binding motif of GR with everted repeats.** The left panel presents PWM representations of binding. Black arrows represent GR binding to monomers G-AC- and -GT-C. The right panel presents the corresponding structural representation. (Designed by Laura Vanderploeg.)

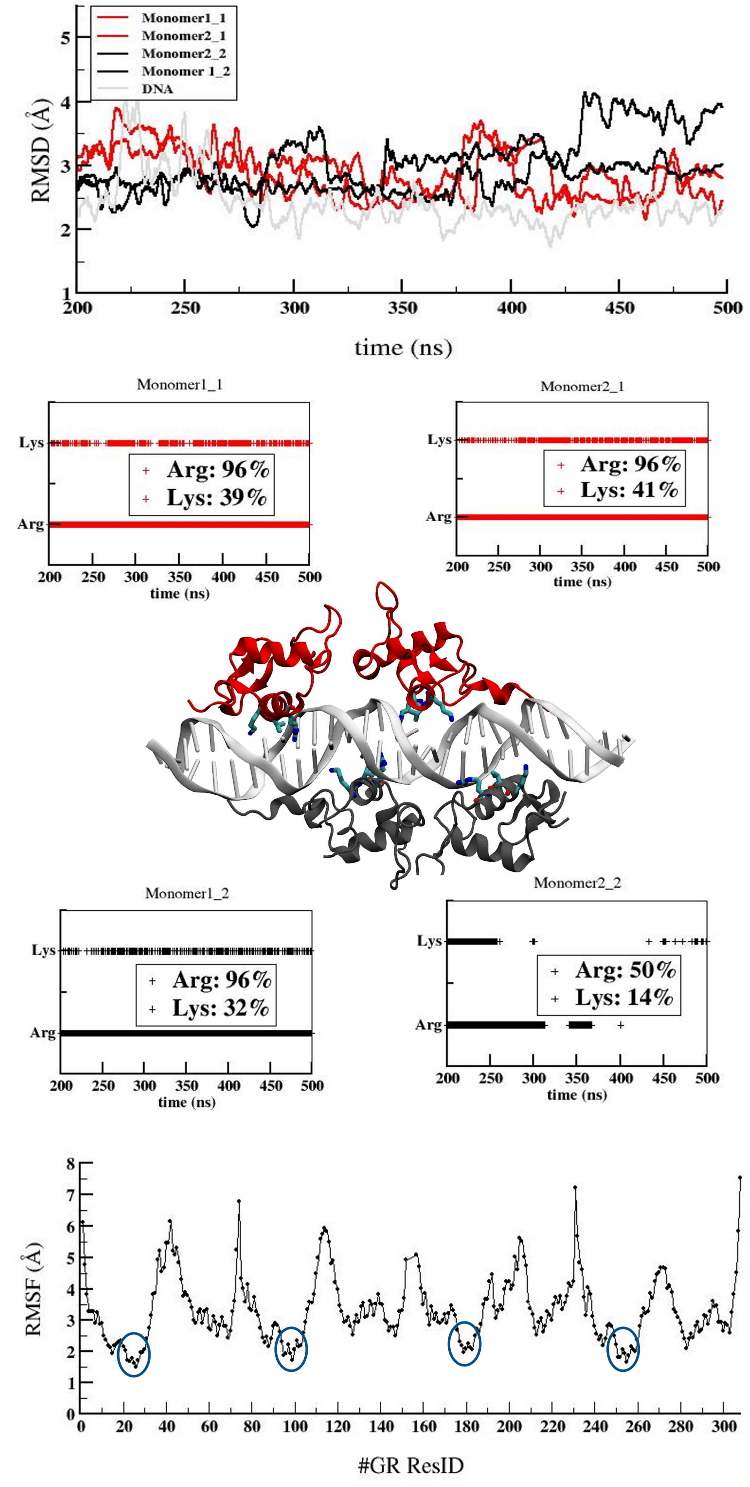
Monomer1_1
Monomer2_1
Monomer2_2
Monomer 1_2
DNA
RMSD (Å)
time (ns)
Monomer1_1
Arg: 96%
Lys: 39%
Monomer2_1
Arg: 96%
Lys: 41%
Monomer1_2
Arg: 96%
Lys: 32%
Monomer2_2
Arg: 50%
Lys: 14%
Lys
Arg
time (ns)
RMSF (Å)
#GR ResID

**Supplementary Figure 6 | Stability of DNA-bound GR tetramer model tested using all-atom molecular dynamics simulations.** The modelled DNA-bound GR tetramer complex (ribbon representation) is stable throughout the simulation period (RMSD plot). Persistence of H-bond interactions between DNA and the conserved Lys442 (ResID 25,98,181,254 in the model) and Arg447 (ResID 30,103,186,259 in the model) contribute to the stability of the complex (circled in RMSF plot). Slight perturbations are observed in one of the dimers (Dimer 1). This results in the slightly increased RMSD of monomer 1 of dimer 2 (black) and a partial loss of Arg-DNA interaction in monomer 2 of dimer 2 (black). Dimer 1 is completely stable throughout the simulation period. This indicates that the dimer bound state of GR is more stable than the tetramer bound state, in complete agreement with our MinSeq analyses. H-bonds are defined using a distance criteria of < 3.0Å between heavy atoms and an angle cut off of >135°. Atoms used for calculating GR:DNA H-bonds: Lys-NZ:DG-N7/O6 and Arg-NH1/NH2:DG-N7/O6. All data is shown for the last 300ns of the simulation.

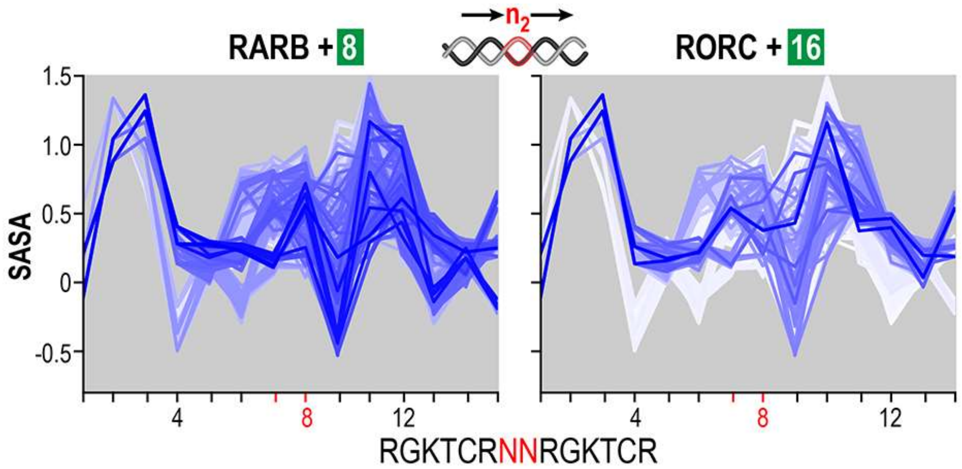


**Supplementary Figure 7 | Binding preferences of RARB+8 and RORC+16.** RARB+8 and RORC+16 exhibit distinct preferences for the DR2 site with different solvent-accessible minor groove surface areas (SASA), as determined by Orchid2 analysis[4]. Enrichment are colored from minima (white) to maxima (blue). (Source data are provided as a Source Data file. Designed by Laura Vanderploeg.)

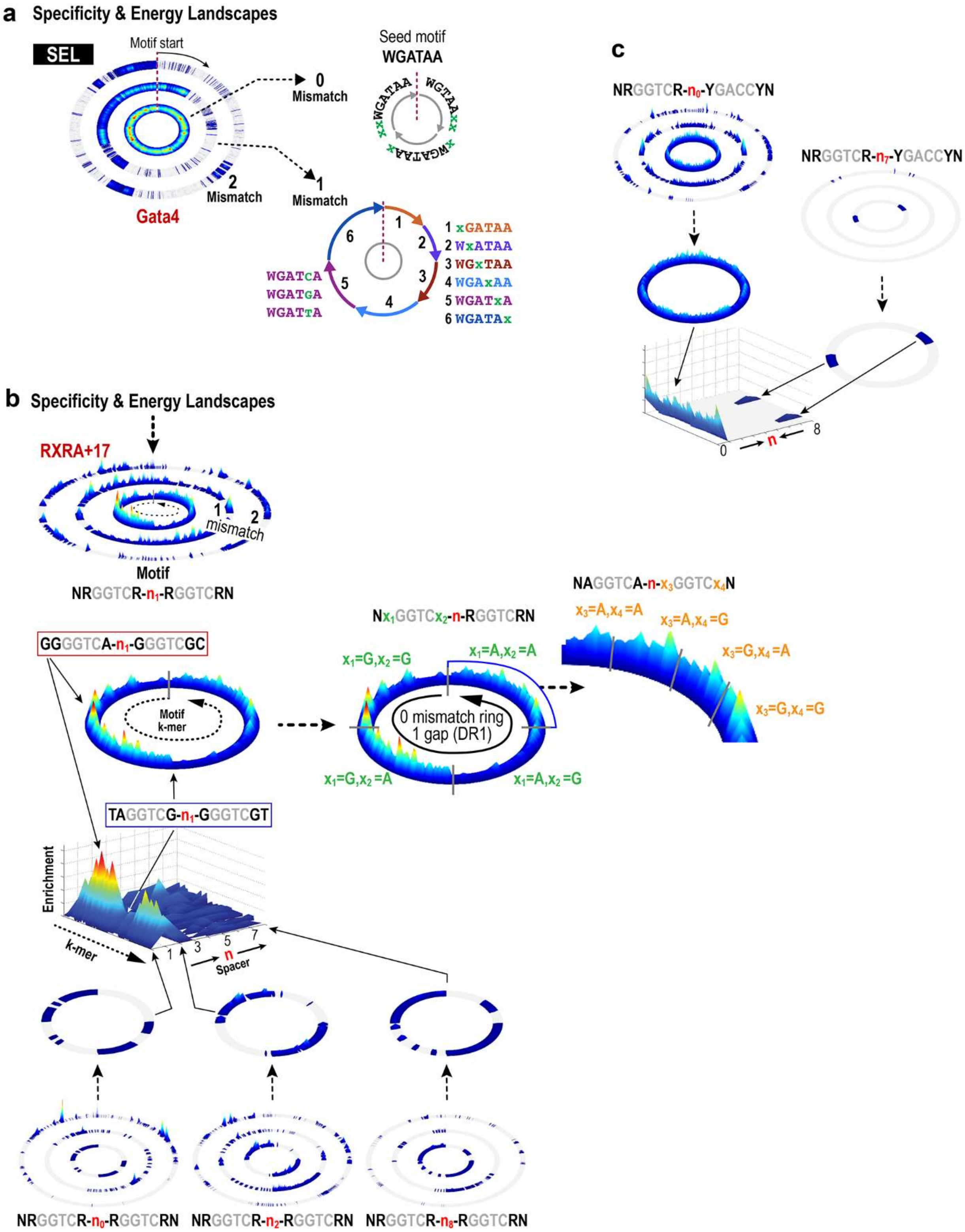

a Specificity & Energy Landscapes
SEL
Motif start
Seed motif
WGATAA
0 Mismatch
1 Mismatch
2 Mismatch
Gata4
1 xGATAA
2 WxATAA
3 WGxTAA
4 WGAxAA
5 WGATxA
6 WGATAx
WGATCA
WGATGA
WGATTA
b Specificity & Energy Landscapes
RXRA+17
1 2 mismatch
Motif
NRGGTCR-n1-RGGTCRN
GGGGTCA-n1-GGGTCGC
Motif k-mer
TAGGTCG-n1-GGGTCGT
Enrichment
k-mer
n Spacer
Nx1GGTCx2-n-RGGTCRN
x1=G,x2=G
x1=A,x2=A
0 mismatch ring 1 gap (DR1)
x1=G,x2=A
x1=A,x2=G
NAGGTCA-n-x3GGTCx4N
x3=A,x4=A
x3=A,x4=G
x3=G,x4=A
x3=G,x4=G
NRGGTCR-n0-RGGTCRN
NRGGTCR-n2-RGGTCRN
NRGGTCR-n8-RGGTCRN
c
NRGGTCR-n0-YGACCYN
NRGGTCR-n7-YGACCYN
n

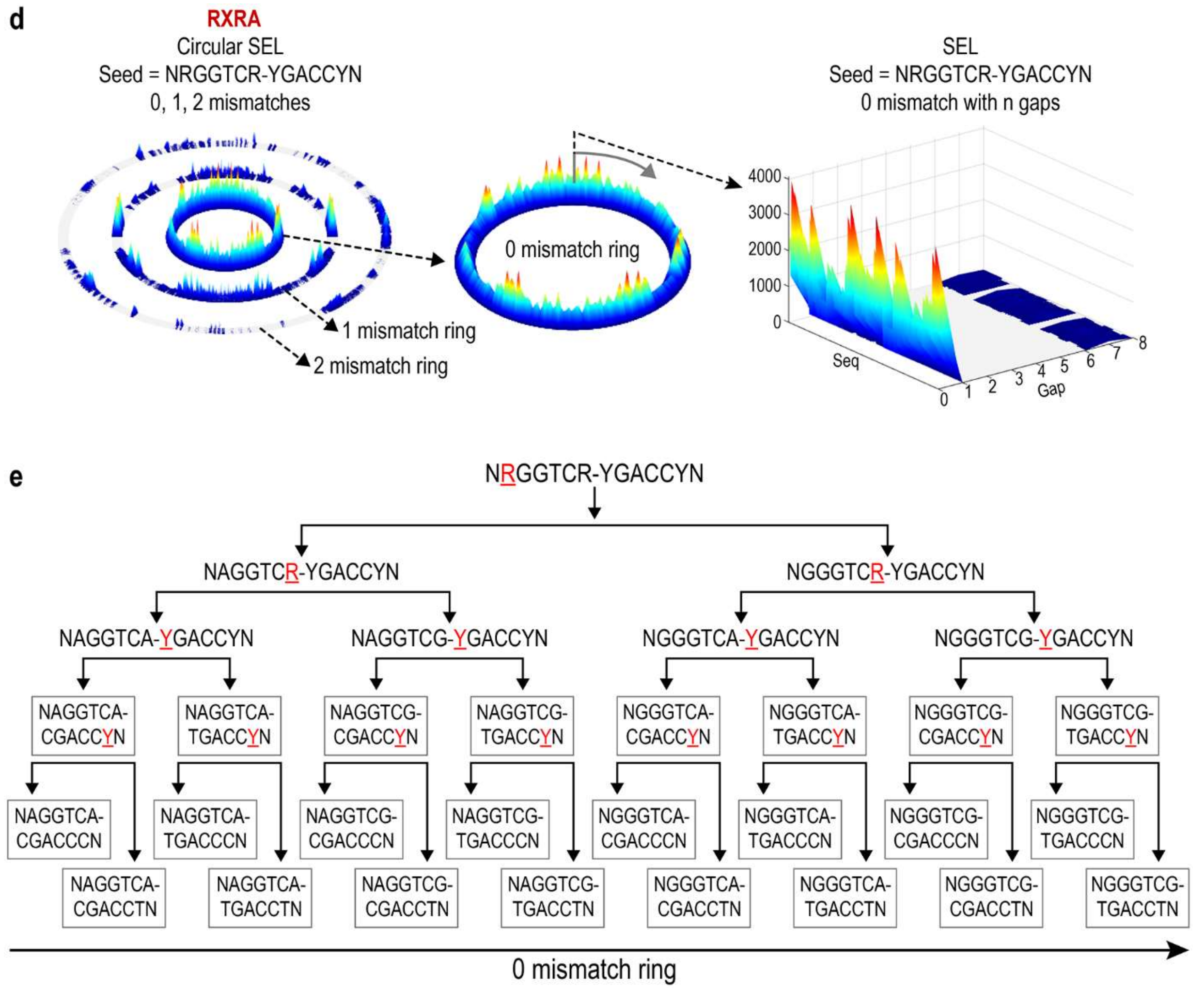


**Supplementary Figure 8 | Specificity and Energy Landscapes (SEL) and Gapped SELs. a,** Top view of SEL of DNA protein interactome for Gata4 using 5′WGATAA3′ as seed motif, where W = A or G[5]. The innermost zero-mismatch ring contains all sequences that have an exact match to the seed motif. The sequences within the ring are arranged by the nucleotides flanking the seed. One ring out the one-mismatch ring contains all sequences that differ from the seed motif at any one nucleotide position or a Hamming distance of one and so on. The height of each color-coded peak corresponds to the DNA binding intensity. **b,** SELs for RXRA+17 interactome with 5′NRGGTCR-$n_p$-RGGTCRN3′ as seed (where R= A or G, N = A, C, G or T, $n_p$ = p spaces, plotted for p=0, 1, 2 and 8). Central 0 mismatch ring contains all sequences matching seed NRGGTCR-n-RGGTCRN. Zero mismatch rings of SELs for various DRs of 5′RGKTCR3′ are opened up and merged together as Gapped-SEL, displaying change in binding preferences with spacing for RXRA+17. Different sections of SEL for p=0 are shown. **c,** Gapped SEL

corresponding to interactome data of RXRA+17 for IRs of 5′RGKTCR3′ **d,** Another example, Left - Circular SEL of RXRA in the form of concentric rings with seed NRGGTCR-YGACCYN (i.e., inverted repeat with gap or spacer 0 of monomer RGGTCR (IR0 of RGGTCR)) flanked by 1 N. Sequences matching the seed are plotted within the 0-mismatch ring. 1-mismatch sequences relative to seed are plotted one ring out and so on[5–7]. Right, Gapped SEL representing only the 0-mismatch ring and plotting the circular SEL peaks on the x-axis Gapped SEL with y = 0, then sequences corresponding to seed with 1bp gap or space in between the monomers are plotted on y = 1 (i.e. seed NRGGTCR-$n_1$-YGACCYN (IR1 of RGGTCR flanked with 1 N) and so on until y = 8 (or 8 gap). Z-axis corresponds to color coded peaks for enrichment values. The dash (-) between seed and sequence is used for convenience only to separate the monomers and does not correspond to any nucleotide. **e,** Arrangement of the sequences within 0-mismatch ring or x-axis with y = 0. Left to right is the order of the sequences on x-axis. Capital N is further permutated with A, C, G, and T, thus first sequence in y = 0 will be AAGGTCA-CGACCCA. Same ordering is followed for seeds with gaps. Small letter n within the seed is treated as gap and isn't replaced with A,C,G or T, thus the first sequence in y = 1 will be sequence AAGGTCA-n-CGACCCA and enrichment is obtained from MinSeq for this sequence, which is then plotted. (Source data are provided as a Source Data file. Designed by Laura Vanderploeg.)

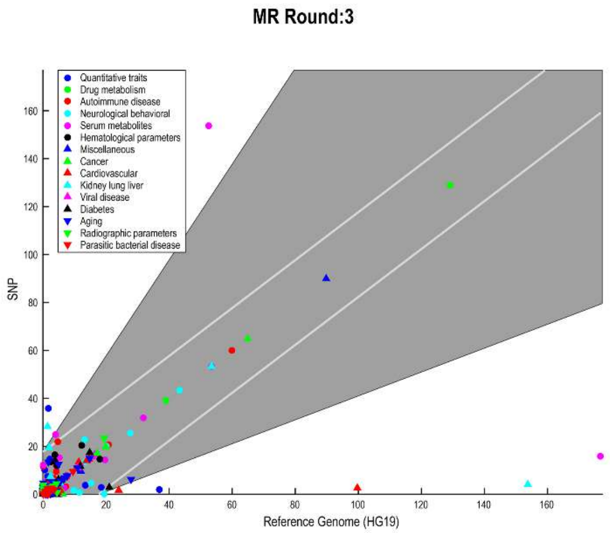


| S. No. | SNP | Chr. | Position | Diesease Class | Diesease Trait | HG19 | SNP | $log_2\left(\frac{SNP+\eta}{HG19+\eta}\right)$ |
|---|---|---|---|---|---|---|---|---|
| 1 | rs11858836 | chr15 | 78783277 | Kidney lung liver | COPD | 153.80 | 4.21 | -2.97 |
| 2 | rs4921914 | chr8 | 18272438 | Serum metabolites | Formate-succinate ratio | 176.85 | 16.00 | -2.53 |
| 3 | rs4977574 | chr9 | 22098574 | Cardiovascular | Coronary heart disease | 99.65 | 2.72 | -2.52 |
| 4 | rs4977574 | chr9 | 22098574 | Cardiovascular | Myocardial infarction | 99.65 | 2.72 | -2.52 |
| 5 | rs710841 | chr4 | 82149831 | Quantitative traits | Height | 36.89 | 1.88 | -1.48 |
| 6 | rs2073233 | chr20 | 12874585 | Quantitative traits | Brain structure | 1.83 | 35.91 | 1.46 |
| 7 | rs4363657 | chr12 | 21368722 | Serum metabolites | Bilirubin | 52.61 | 153.80 | 1.29 |
| 8 | rs2499604 | chr1 | 238103501 | Kidney lung liver | NAFLDH | 1.41 | 28.26 | 1.27 |
| 9 | rs4370013 | chr3 | 2654691 | Cardiovascular | Diastolic blood pressure | 24.07 | 1.69 | -1.11 |
| 10 | rs11880706 | chr19 | 6080482 | Neurological behavioral | Biplolar disorder & schizophrenia | 19.47 | 0.00 | -1.07 |

**Supplementary Figure 9 | Effect of disease associated SNPs on DNA binding on MR.** Top, scatter plot of predicted change in DNA binding enrichment of MR from reference (hg19) on x-axis to the SNP on y-axis from Maurano et al. study[8]. SNPs are categorized by its associated disease class and trait. SNPs falling in the region outside grey area represent those causing larger change in the binding and those are listed at the bottom by descending order of the absolute value of 2-fold change of ratio (enrichment of alternate allele +η) and (enrichment of reference allele + η). SNPs with a predicted 2-fold change or greater are listed. A factor of e is added to create an offset to exclude low enrichment changes, η = minimum of i) 10 enrichment and ii) 10% of maximum enrichment. (Source data are provided as a Source Data file.)

| Sequence | MinSeq Enrichment |
| --- | --- |
| CTGGTGACCT**C**CC | 70.84 Ref. allele |
| CTGGTGACCT**T**CC | 94.51 rs521991 SNP |

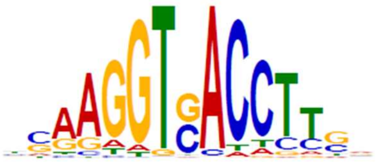

**Supplementary Figure 10 | Allele specific binding by ESRRA.** The SNP rs521991(C to T) creates an atypical ESSRA-binding site. ESRRA showed allele specific binding for rs521991 SNP (log10 fold change = 1.886 as reported by Abramov et al.[9]), which is concordant to what found by MinSeq analysis of HT-SELEX data of ESRRA from this study as increase in MinSeq based enrichment is from 70.84 to 94.51.

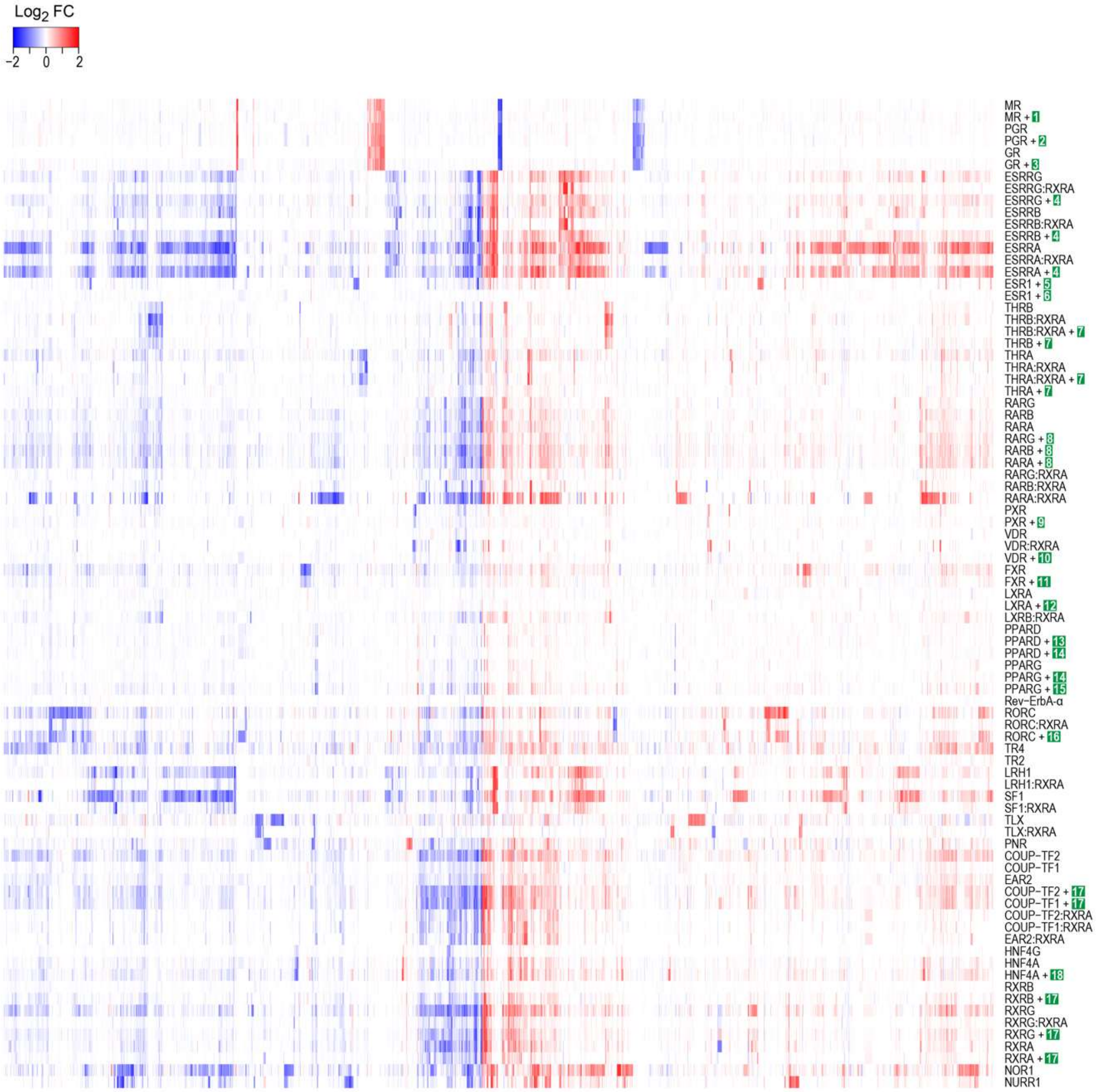


**Supplementary Figure 11 | Heatmap of 5192 non-coding SNPs predicted to alter DNA binding of hNRs.** 53,040 non-coding GWAS SNPs were evaluated (NHGRI-EBI GWAS Catalog v1.0.2-associations_e93_r2018-10-29_GRCh38.p12 https://www.ebi.ac.uk/gwas/[10]). NR samples are represented along the rows and SNPs along columns. Change in binding for SNPs from reference allele to alternate allele is plotted on a log2 scale (see methods). Red indicates gain of function, whereas blue indicates loss of function. (Source data are provided as a Source Data file.)

| **Nuclear Receptor** | **Gene and Kazusa Number** |
|---|---|
| AR v2 | androgen receptor pFN21AE1197 (v2) |
| AR v3 | androgen receptor pFN21AE1198 (v3) |
| ESR1 | estrogen receptor 1 pFN21AB7810 |
| ESRRA | estrogen-related receptor alpha pFN21AB7713 |
| ESRRB | estrogen-related receptor beta pFN21AB9737 |
| ESRRG | estrogen-related receptor gamma pFN21ASDA0832 |
| HNF4A | hepatocyte nuclear factor 4, alpha pFN21AB9731 |
| HNF4G | hepatocyte nuclear factor 4, gamma pFN21AB7751 |
| NR0B1 | nuclear receptor subfamily 0, group B, member 1 pFN21AB7043 |
| NR1D1 | nuclear receptor subfamily 1, group D, member 1 pFN21AB7838 |
| NR1D2 | nuclear receptor subfamily 1, group D, member 2 pFN21AB9131 |
| NR1H2 | nuclear receptor subfamily 1, group H, member 2 pFN21AB8155 |
| NR1H3 | nuclear receptor subfamily 1, group H, member 3 pFN21AB7759 |
| NR1H4 | nuclear receptor subfamily 1, group H, member 4 pFN21AB7740 |
| NR1I2 | nuclear receptor subfamily 1, group I, member 2 pFN21AB7714 |
| NR1I3 | nuclear receptor subfamily 1, group I, member 3 pFN21AB7608 |
| NR2C1 | nuclear receptor subfamily 2, group C, member 1 pFN21AB6166 |
| NR2C2 | nuclear receptor subfamily 2, group C, member 2 pFN21AB5580 |
| NR2E1 | nuclear receptor subfamily 2, group E, member 1 pFN21AB4185 |
| NR2E3 | nuclear receptor subfamily 2, group E, member 3 pFN21AB7686 |
| NR2F1 | nuclear receptor subfamily 2, group F, member 1 pFN21AB8385 |
| NR2F2 | nuclear receptor subfamily 2, group F, member 2 pFN21AB7696 |
| NR2F6 | nuclear receptor subfamily 2, group F, member 6 pFN21AB9720 |
| NR3C1 | nuclear receptor subfamily 3, group C, member 1 (glucocorticoid receptor) pFN21AB9466 |
| NR3C2 | nuclear receptor subfamily 3, group C, member 2 pFN21AB0468 |
| NR4A1 | nuclear receptor subfamily 4, group A, member 1 pFN21AB9634 |
| NR4A1 v1 | nuclear receptor subfamily 4, group A, member 1 pFN21AE1384 (v1) |
| NR4A2 | nuclear receptor subfamily 4, group A, member 2 pFN21AB7818 |
| NR4A3 | nuclear receptor subfamily 4, group A, member 3 pFN21AB3311 |
| NR5A1 | nuclear receptor subfamily 5, group A, member 1 pFN21AB7702 |
| NR5A2 | nuclear receptor subfamily 5, group A, member 2 pFN21AB5946 |
| PGR | progesterone receptor pFN21AB9766 |
| PPARA | peroxisome proliferator-activated receptor alpha pFN21AB9549 |
| PPARD | peroxisome proliferator-activated receptor delta pFN21AB4629 |
| PPARG | peroxisome proliferator-activated receptor gamma pFN21AB7770 |
| RARA | retinoic acid receptor, alpha pFN21AE1591 |
| RARB | retinoic acid receptor, beta pFN21AB5810 |
| RARG | retinoic acid receptor, gamma pFN21AB5808 |
| RARRES3 | retinoic acid receptor responder (tazarotene induced) 3 pFN21AE3658 |
| RORB | RAR-related orphan receptor B pFN21AE0940 |

| | |
|---|---|
| RORC | RAR-related orphan receptor C pFN21AE3637 |
| RXRA | retinoid X receptor, alpha pFN21AB9770 |
| RXRB | retinoid X receptor, beta pFN21AB4058 |
| RXRG | retinoid X receptor, gamma pFN21AB5869 |
| THRA v1 | thyroid hormone receptor, pFN21AB7752 (v1) |
| THRB | thyroid hormone receptor, beta pFN21AB3732 |
| VDR | vitamin D (1,25- dihydroxyvitamin D3) receptor pFN21AB7719 |
| ROR1 | Homo sapiens receptor tyrosine kinase-like orphan receptor 1 (ROR1), pFN21AE3329 |

**Supplementary Table 1 | Plasmids details**

| **Oligonucleotides for HT-SELEX** | |
|---|---|
| **Name** | **sequence (5' --> 3')** |
| 20mer DNA library | CTGATCCTACCATCCGTGCTNNNNNNNNNNNNNNNNNNNNCACAGCTTCGTACCGAGCGG |
| reversePrimer | CCGCTCGGTACGAAGCTG |
| forwardPrimer | CTGATCCTACCATCCGTGCT |
| forwardPrimer + Illumina adapter | AATGATACGGCGACCACCGAGATCTGCTCTTCCGATCTCTGATCCTACCATCCGTGCT |
| reversePrimer + Illumina adapter | CAAGCAGAAGACGGCATACGAGATXXXXXXTCTTCCGATCCCGCTCGGTACGAAGCTGTG |
| squencingPrimer | GCTCTTCCGATCTCTGATCCTACCATCCGTGCT |
| **Oligonucleotides for EMSA** | |
| ERE_60mer_v2 | GTCCTAATCACCCCTGTTCGcCAGGTCAGAGTGACCTGacGACACTTCTGCCCAGGCGAG |
| DR1_60mer_v2 | GTCCTAATCACCCCTGTTCGcctAGGTCAaAGGTCAgtatGACACTTCTGCCCAGGCGAG |
| Nurr_60mer_v2 | GTCCTAATCACCCCTGTTCGgagaaAAAAGGTCAaggtaaGACACTTCTGCCCAGGCGAG |
| Cy5_rPCR | /cy5/CTCGCCTGGGCAGAAGTGTCGTC |

**Supplementary Table 2 | Oligonucleotide sequences used in this study.** All oligonucleotides were purchased from IDT (Iowa, USA) as standard desalting purification, except for the Cy5_rPCR which was purchased as HPLC purified.

| | AR | GR | GR/AR Ratio |
|---|---|---|---|
| Dimer | 638 | 645 | 1.01 |
| Trimer | 45 | 101 | 2.24 |
| Tetramer | 39 | 103 | 2.64 |
| n-GRE1 | 117 | 168 | 1.44 |
| CGA intermediate | 6 | 9 | 1.5 |
| As/Ts stretch flanks | 22 | 13 | 0.59 |

**Supplementary Table 3 | Abundance and ratio of different sequences**- As identified by *MinSeq Find* algorithm in top 2000 AR and GR ChIP Exo peaks in U2OS cell line[11]. The length of the peaks are limited to 100 base pairs spanning the center of the peaks. Expected inverted dimer (IR3) motif is abundant in both AR and GR peaks (top row). The trimer, other multimers and n-GRE1 binding sequences are better represented in GR peaks in comparison to AR peaks (rows 2-4). While CGA in the intermediate region of IR3 is enriched 1.5-fold in GR than in AR ChIP peaks. By contrast, stretches of As/Ts on flanking were more prevalent in AR peaks (bottom row).

## Supplementary References


1. Zhang, L. *et al.* SelexGLM differentiates androgen and glucocorticoid receptor DNA-binding preference over an extended binding site. *Genome Res* **28**, 111–121 (2018).

2. Jolma, A. *et al.* DNA-binding specificities of human transcription factors. *Cell* **152**, 327–339 (2013).

3. Yin, Y. *et al.* Impact of cytosine methylation on DNA binding specificities of human transcription factors. *Science* **356**, eaaj2239 (2017).

4. Bishop, E. P. *et al.* A Map of Minor Groove Shape and Electrostatic Potential from Hydroxyl Radical Cleavage Patterns of DNA. *ACS Chem Biol* **6**, 1314–1320 (2011).

5. Bhimsaria, D. *et al.* Specificity landscapes unmask submaximal binding site preferences of transcription factors. *Proc Natl Acad Sci U S A* **115**, E10586–E10595 (2018).

6. Carlson, C. D. *et al.* Specificity landscapes of DNA binding molecules elucidate biological function. *Proc Natl Acad Sci U S A* **107**, 4544–4549 (2010).

7. Tietjen, J. R., Donato, L. J., Bhimsaria, D. & Ansari, A. Z. Sequence-specificity and energy landscapes of DNA-binding molecules. *Methods Enzymol* **497**, 3–30 (2011).

8. Maurano, M. T. *et al.* Systematic Localization of Common Disease-Associated Variation in Regulatory DNA. *Science* **337**, 1190–1195 (2012).

9. Abramov, S. *et al.* Landscape of allele-specific transcription factor binding in the human genome. *Nat Commun* **12**, 2751 (2021).

10. Buniello, A. *et al.* The NHGRI-EBI GWAS Catalog of published genome-wide association studies, targeted arrays and summary statistics 2019. *Nucleic Acids Res* **47**, D1005–D1012 (2019).

11. Kulik, M. *et al.* Androgen and glucocorticoid receptor direct distinct transcriptional programs by receptor-specific and shared DNA binding sites. *Nucleic Acids Res* **49**, 3856–3875 (2021).
