# Supplementary material for: Hidden modes of DNA binding by human nuclear receptors
Source: Nat Commun. 2023 Jul 13;14:4179. doi: 10.1038/s41467-023-39577-0 (PMC10345098; doi:10.1038/s41467-023-39577-0)
Supplement: Supplementary file 2 — Description of Additional Supplementary Files [file 41467_2023_39577_MOESM2_ESM.pdf]

Description of Additional Supplementary Files

File name: Supplementary Data 1

Description: Detailing MinSeq Find algorithm.

File name: Supplementary Data 2

Description: PWM logos for all the factors - MinSeq Find derived PWM logos for all publicly available Nuclear Receptors DPIs - PWM logos (as explained in methods) are displayed in the first column for all the NR samples. In subsequent columns, seed MinSeq sequence and seed intensity/enrichment are displayed. The last column represents if the seed sequence is a dimer with orientation- DR, IR, or ER, with gap/space between the monomers, is denoted after repeat orientation; if there is a monomer detected or no-repeat is detected by the program, it is represented by M. Minimum and maximum value of k and l (**Fig. 1c**) used are 4 and 8 respectively.

File name: Supplementary Data 3

Description: MinSeq Find derived PWM logos for Nuclear Receptors in this study. The minimum and maximum values of k and l (**Fig. 1c**) used are 5 and 8, respectively.

File name: Supplementary Data 4

Description: Monomer binding of Nuclear Receptors – PWM logos selected from Supplementary Data 3, which represent monomer binding for the NRs.

File name: Supplementary Data 5

Description: MinSeq Find derived PWMs in matrix form – PWMs from Supplementary Data 3 are represented here in matrix format.

File name: Supplementary Data 6

Description: Gapped-SELs for all Nuclear Receptors – Gapped-SELs are plotted for all the samples and shown separately for each sub-family for dimers corresponding to different seeds

(details in **Supplementary Fig. 8**). Similarities and differences between each binding profile can be viewed across each sub-family in such plots.

File name: Supplementary Data 7

Description: Comparing different Nuclear Receptors – Different NR samples are compared here and presented according to the sub-family. In each comparison at the top is the scatter plot comparison of all sequences on gapped-SELs. Further gapped-DiSEL (Differential SEL) are plotted by normalizing and subtracting the intensity from gapped SELs (X over Y is normalized X subtracted by normalized Y). Gapped-DiSEL gives a visual representation of the difference between the two NR samples binding as dimers.

File name: Supplementary Data 8

Description: Amino acid sequence of Full-length NRs used in this study.

File name: Supplementary Data 9

Description: Prediction of Nuclear Receptor ChIP-seq peaks – NRs DPIs from this study are used to evaluate and predict different in vivo bound peaks from LoVo Cell line (Yan, J. *et al.* Transcription factor binding in human cells occurs in dense clusters formed around cohesin anchor sites. *Cell* **154**, 801–813 (2013)) and the ENCODE project (Dunham, I. *et al.* An integrated encyclopedia of DNA elements in the human genome. *Nature* **489**, 57–74 (2012)). ROC curves are shown first (methods), and S scores were then used to show predictions colored red-to-green, red being 1 (highest confidence in detection) and green being 0.5 (least confidence or random). Prediction is also done for the intersection of RXRA-bound peaks by other NRs in the same cell line, and the ChIP intersection is shown by the ":" sign.

File name: Supplementary Data 10

Description: Prediction of Nuclear Receptor ChIP-seq peaks from ENCODE (Dunham, I. *et al.* An integrated encyclopedia of DNA elements in the human genome. *Nature* **489**, 57–74 (2012)) and the LoVo cell line (Yan, J. *et al.* Transcription factor binding in human cells occurs in dense clusters formed around cohesin anchor sites. *Cell* **154**, 801–813 (2013)) using MinSeqs Find

algorithm, DeepBind (Alipanahi, B., Delong, A., Weirauch, M. T. & Frey, B. J. Predicting the sequence specificities of DNA- and RNA-binding proteins by deep learning. *Nat Biotechnol* **33**, 831–838 (2015)), Autoseed PWM and gkm-SVM (Mohammad-Noori, M. *et al.* gkmSVM: an R package for gapped-kmer SVM. *Bioinformatics* **32**, 2205–2207 (2016)) (default parameters with 5000 sequences) for HT-SELEX data in the form of the area under ROC curve.

File name: Supplementary Data 11

Description: Impact of single nucleotide polymorphisms on the genomic binding of Nuclear Receptors – 5592 human SNPs curated in Maurano et al. (Maurano, M. T. *et al.* Systematic Localization of Common Disease-Associated Variation in Regulatory DNA. *Science (1979)* **337**, 1190–1195 (2012)) and associated to diseases and quantitative traits were scored using MinSeqs for all DPIs of NRs in this study. Scatter plots, with binding corresponding to reference alleles (HG19) are displayed on X-axis, and alternate alleles on Y-axis. Different SNPs are categorized according to their disease or trait class in the plot. The region of the plot is greyed to show reject SNPs which showed a difference lesser than 2-fold in binding and possible noise (see methods). SNPs that didn't fall into the greyed region are chosen to be possibly affected by corresponding NR and are then tabulated below the plot, with the SNP id, position in the genome (HG19), disease class, predicted binding intensity for reference allele (HG19) and alternate allele and finally log2 fold change (see methods). SNPs are ordered according to the absolute value of log2 fold change. A factor of η is added to create an offset to exclude low enrichment changes, η =minimum of i) 10 enrichment and ii) 10% of maximum enrichment. A positive value for log2 fold change represents increased binding because of the SNP, and a negative represents a decrease.

File name: Supplementary Data 12

Description: Selected 5192 SNPs from NHGRI-supported GWAS Catalog, which showed two-fold change for at least one Nuclear Receptor from this study.
