# Supplementary material for: Hidden modes of DNA binding by human nuclear receptors
Source: Nat Commun. 2023 Jul 13;14:4179. doi: 10.1038/s41467-023-39577-0 (PMC10345098; doi:10.1038/s41467-023-39577-0)
Supplement: Supplementary file 3 — Supplementary Data 1 [file 41467_2023_39577_MOESM3_ESM.docx]

**Supplementary Data 1**

**Detailing *MinSeq Find* algorithm**

**Table of contents**

[1. Position Weight Matrix (PWM) 2](#_Toc113365657)

[a. Scoring with PWMs 2](#_Toc113365658)

[2. Introducing MinSeqs 2](#_Toc113365659)

[a. Why introduce the concept of MinSeqs? 3](#_Toc113365660)

[b. MinSeq Find: Sequencing Data Analysis & MinSeq extraction 3](#_Toc113365661)

[c. Weighted Enrichment of MinSeqs 4](#_Toc113365663)

[3. Compressed sensing based analysis 6](#_Toc113365664)

[4. MinSeqs to score sequences 7](#_Toc113365665)

[5. Position Associated Gapped LOcation-specific (PAGLO) model for MinSeqs 8](#_Toc113365666)

[6. Poisson distributed reads and threshold cutoff for sequences 11](#_Toc113365667)

[7. MinSeqs and PWMs derived from MinSeq Find for HT-SELEX data from Jolma et al. 12](#_Toc113365668)

# Position Weight Matrix (PWM)

Position weight matrix^1–3^ captures protein-DNA binding by compressing the information obtained from DNA binding experiment into a $4\times p$ matrix, where $4$ values in each column corresponds to probability (or frequency) of binding of nucleotides A, C, G and T at that position, and number of columns $p$ is the length of the PWM or DNA binding region. Recently developed high-throughput methods^4–19^ to capture in vitro protein-DNA binding contains lot more information, which is compressed into $4\times p$ such motifs by existing computational analysis methods^20–27^. Thus, new method is developed here to capture all the information.

# Scoring with PWMs

PWM (length $p$) corresponding to DNA-protein binding can be used to score any DNA sequence (of length $m \geq$ p) for prediction of binding to the protein. A moving window of length $p$ is used over given DNA sequence to obtain $m-p+1$ sub-sequences of length $p$. PWM is then used to calculated binding intensity for each sub-sequence, by multiplying weights (or frequency in case of frequency matrix) corresponding to each nucleotide respective to position in PWM. After that maximum of all such binding intensity is used to get a binding score for given DNA sequence. The maximum possible binding score in such a case can be obtained by multiplication of maximum weight at each position of PWM. This can be used for a comparison, how well the sequence is expected to bound to a given protein in comparison to the best binding sequence. Such scoring was used for PWMs obtained from Jolma et al. and from this paper.

# Introducing MinSeqs

**Definition**: A $\left( \boldsymbol{k, g, l} \right)$***-MinSeq*** is a $k$-mer comprised of A, C, G, and T followed by a spacer (gap) sequence of $g \geq0$ Ns (N means any nucleotide), followed an $l$-mer comprised A, C, G, and T. For example, the sequence AACGNNNGCTTA is a $(4, 3, 5)$-MinSeq because a $4$-mer AACG is followed by NNN which is in turn followed by a $5$-mer GCTTA. Here we have used only A, C, G, T, and N nucleotides to build MinSeqs because of computational limitations, but one can use other degenerate nucleotides like W (A/T) and D (A/G/T) to build MinSeqs.

**Definition**: A $k_{1}$-mer is said to be ***left-contained*** in $k_{2}$-mer if $k_{1}\leq k_{2}$ and the sequence corresponding to $k_{1}$-mer is followed to the right by a sequence of A, C, G, and T to obtain the $k_{2}$-mer. Likewise, a $k_{1}$-mer is said to be ***right-contained*** in $k_{2}$-mer if $k_{1}\leq k_{2}$ and the sequence corresponding to $k_{1}$-mer is preceded to the left by a sequence of A, C, G, and T to obtain the $k_{2}$-mer. For example, ACG is said to be left-contained in ACGATT but right-contained in ATTACG.

**Definition**: A $\left( k_{1}, g_{1},l_{1} \right)$-MinSeq is said to be a **direct** **subsequence** of a $\left( k_{2}, g_{2},l_{2} \right)$-MinSeq if exactly one of the following five conditions hold.

1. The two MinSeqs are identical **OR**
2. $k_{1}+1=k_{2}$, $g_{1}=g_{2}$, $l_{1}=l_{2}$, $k_{1}$-mer is right-contained in $k_{2}$-mer, and $l_{1}$-mer is identical to $l_{2}$-mer **OR**
3. $k_{1}=k_{2}$, $g_{1}=g_{2}$, $l_{1}+1=l_{2}$, $l_{1}$-mer is left-contained in $l_{2}$-mer, and $k_{1}$-mer is identical to $k_{2}$-mer **OR**
4. $k_{1}+1=k_{2}$, $g_{1}-1=g_{2}$, $l_{1}=l_{2}$, $k_{1}$-mer is left-contained in $k_{2}$-mer, and $l_{1}$-mer is identical to $l_{2}$-mer **OR**
5. $k_{1}=k_{2}$, $g_{1}-1=g_{2}$, $l_{1}+1=l_{2}$,, $l_{1}$-mer is right-contained in $l_{2}$-mer and $k_{1}$-mer is identical to $k_{2}$-mer.

**Definition:** A $\left( k_{1}, g_{1},l_{1} \right)$-MinSeq is said to be a ***subsequence*** of $\left( k_{2}, g_{2},l_{2} \right)$-MinSeq if there is a sequence of MinSeqs $m_{1}$, $m_{2}$, …, $m_{v}$, such that $\left( k_{1}, g_{1},l_{1} \right)$-MinSeq is a direct subsequence of $m_{1}$, $m_{1}$ is a direct subsequence of $m_{2}$, …, $m_{v-1}$ is a direct subsequence of $m_{v}$, and $m_{v}$ is a direct subsequence of $\left( k_{2}, g_{2},l_{2} \right)$-MinSeq. We use the notation $\subseteq$ to denote this relationship. Example CGTNNA is a subsequence of sequence ACGTNNAAA, as CGTNNA is a direct subsequence of CGTNNAA, which is direct subsequence of CGTNNAAA, which is direct subsequence of ACGTNNAAA.

**Note:** MinSeqs $(k, g, l)$ with $g=0$ can fall into multiple category of MinSeqs. Like MinSeq $(k, 0, l)$ can also be written as MinSeq $(k+1, 0, l-1)$ example – MinSeq ACGTAAA can regarded as MinSeq $(4, 0, 3)$ – $4$-mer ACGT followed by no gap/space and a $3$-mer AAA, and can also be assigned as MinSeq $(3, 0, 4)$ – $3$-mer ACG followed by no gap and a $4$-mer TAAAA. Thus, MinSeq obtained in our analysis with no gap $\left( g=0 \right)$ are treated differently, if we get a MinSeq with $g=0$, we convert all MinSeqs into a format such that $l=0$, thus for the case ACGTAAA we use it as MinSeq $(7,0,0)$ with $7$-mer ACGTAAA followed by no gap and a $0$-length sequence.

Note: Further improvement in performance of MinSeqs can be obtained by use of not only A, C, G, T and N, but also K (G/T), M (A/C), R (A/G), Y (C/T), S (C/G), W (A/T), B (C/G/T), V (A/C/G), H (A/C/T), and D (A/G/T).

# Why introduce the concept of MinSeqs?

Unlike PWM, $k$-mer intensity data includes binding intensity (obtained from array, fluidics or sequencing) for all sequences of length $k$ and thus can capture different binding pattern. MinSeq is an alternate representation of the DNA-binding data instead of PWMs and k-mer sequence intensity. MinSeqs, on the other hand, can be of different lengths and with gaps.

If binding is with gaps or the binding motifs are longer than $10$ base pair, it is not feasible to get $k$-mer intensity data for such case, for $12$-mer around $8$ million separate probes are needed to measure binding for all $12$-mers. In case of sequencing all sequences of length $12$ will not have enough representation (occurrence less than $10$ in all the reads) in the output DNA reads. To counter this problem, we introduce the concept of MinSeqs.

# *MinSeq Find*: Sequencing Data Analysis & MinSeq extraction

Reads obtained from Illumina sequencing were de-multiplexed by matching corresponding $6$-bp barcode and truncated to obtain $20$ bp derived from the random region (**Supplementary Fig. 1**). On average, we obtained 900K reads per barcode. We use the Illumina sequencing reads for a) just the library, b) enriched library with pulldowns done just with Halo beads, and c) the enriched library with pulldowns done with the transcription factor (TF) with Halo bead, with/without the ligand, with/without partner protein (RXRA). Three rounds of enrichment were done for b) and c) and each round was followed by PCR step for exponential enrichment.

To get binding preferences of TF, there are few sources of sequence preference in TF with Halo-bead, which are not part of TF binding and needs to be normalized.

1. The $20$ bp random library is not completely random and thus it has to be modeled.
2. Ideally, the Halo-bead should have similar binding preferences to all sequences. However, we found that this is not the case. Thus, in characterizing the binding preferences of a TF, we should account for the bias introduced by the binding preferences of the Halo-bead.

First, we studied enrichment of Halo-bead by normalizing against starting random library, in following 2 steps-

- Model the relative abundance of all possible sequences in the library $\leq20$ of form MinSeq $\left( k, g, l \right)$.
- From the reads of Halo-bead, consider all possible MinSeqs of $\left( k, g, l \right)$ format $\geq$ count threshold $C^{T}$. Threshold $C^{T}$ is chosen according to maximum tolerable error threshold (explained in detail in section “Poisson distributed reads and threshold cutoff for sequences”). Next, normalize it against library to analyze the binding of Halo-bead.

Above normalization takes care of randomness in the starting library.

Second, we analyzed the binding preferences of the TF. This analysis must account for the biases introduced by the “not-perfectly-random” library, the binding preferences of the Halo bead and variability caused by PCR or other factors. Enrichment of TF was characterized and compared to Halo bead via following two step-

- Model the relative abundance of all possible sequences in the Halo-bead $\leq20$ of form MinSeq $\left( k, g, l \right)$. We combine data from multiple Halo-bead experiment to create model.
- From the reads of TF with Halo-bead, consider all possible MinSeqs of $\left( k, g, l \right)$ format $\geq$ count threshold $C^{T}$ and normalize against Halo-bead to analyze the binding of TF.

Halo-bead experiment was done using the same library as for TF with Halo-bead. Thus, above analysis normalizes randomness in the starting library as well as binding caused by Halo-bead.

Following explains how the modeling and normalization was done for TF-Halo-bead against Halo-bead, similar procedure was followed to normalize Halo-bead against library sample (not used in this paper) -

*Characterization of Halo-bead*. For a $20$ bp library/sample there can $4^{20}= 1$ trillion different sequences in different abundance, to get relative abundance of all $20$-mers we need to get $>100$ trillion reads, which is not feasible with current technology, and we get only a small portion or sample i.e. $1-2$ million $20$-mer reads when $20$ bp library/sample is barcoded and sequenced. This is random sampling and sequenced reads varies each time. We create a model for relative abundance of all sequences of length $\leq20$ from limited reads obtained. Even though a random $20$-mer library was used (ordered from IDT), but library isn’t perfectly random and the probability of any nucleotide at a particular position depends on the previous nucleotides. Thus, to capture imperfection in library and binding of DNA binding by Halo-bead we constructed a $5$^th^-order Markov Model. From the $20$ bp reads, we counted the occurrence of every MinSeq $\left( k_{x}, g_{x},l_{x} \right)$- with $1\leq k_{x}\leq8$, $0\leq l_{x}\leq8$ such that $k_{x},+g_{x}+l_{x}\leq20$. Counts for sequence and its reverse complement were merged. If the count for a particular MinSeq was below a minimum count threshold $C^{T}$, then that MinSeq was discarded. The counts for other MinSeqs were retained. In this study to characterize binding of a TF or Halo-bead, we used MinSeq $\left( k, g, l \right)$ with $5\leq k\leq8$, $5\leq l\leq8$, and $k+g+l\leq20$. Since all such MinSeqs does not have enough representation, we need an estimated abundance or counts for any MinSeq $\left( k, g, l \right)$. Such estimate is done using a $5$^th^ order Markov model (in **Supplementary Fig. 1B** for example an $8$^th^ order Markov model is shown). Model thus obtained contains gaps and is position specific, means there will be different model for sequence starting at position $1$ of $20$ mer and same sequence starting from position $2$ of $20$-mer. This novel model is named position-associated gapped location–specific library (PAGLO) model.

*Enrichment of TF-Halo-bead by normalizing against starting Halo-bead-* From the 20bp reads for the TF-Halo-bead sample, we counted the occurrence of every $\left( k, g, l \right)$ - MinSeq with $5\leq k\leq8$, $5\leq l\leq8$, and $k+g+l\leq20$ (**Supplementary Fig. 1B**). Counts for sequence and its reverse complement were merged (in case of protein-DNA interaction). If the count for a particular MinSeq was below a minimum count threshold $C^{T}$, then that MinSeq was discarded. The counts for other MinSeqs were retained and normalized against the Halo-bead model by dividing the counts of MinSeqs in TF-Halo-bead by expected read counts in Halo-bead using PAGLO model. To get enrichment values (how many times a sequence got enriched in comparison to Halo-bead), counts for TF-Halo-bead and Halo-bead were divided by total number of $20$-mer reads in TF-Halo-bead and Halo-bead respectively.

Note that, TF or Halo-bead can bind to region flanking the random $20$ bp region partly or even completely. Since we considered a position specific model, such binding is normalized and taken care by above analysis pipeline. This whole process of modelling and normalization is termed *MinSeq Find* algorithm.

# Weighted Enrichment of MinSeqs

MinSeq $\left( k, g, l \right)$ have $k$ and $l$ part of the sequence consisting of nucleotides A, C, G or T, whereas g (gap/space) part consists of Ns. Number of different MinSeqs that can exhibit same $\left( k, g, l \right)$ pattern, i.e. a $k$-length sequence followed by $g$-length stretch of Ns, followed by $l$-length sequence, thus is $4^{k+l}$, as there can be one out of $4$ nucleotides at each position on $k$ and $l$ part. In a random situation, the probability of occurrence of MinSeq $\left( k_{1}, g_{1},l_{1} \right)$ in a sequence of length $L\geq\left( k_{1}+g_{1}+l_{1} \right)$ is thus $4^{k_{1}+l_{1}}\times$ $\left( L-k_{1}-g_{1}-l_{1}+1 \right)$. Unlike $k$-mers, MinSeqs are thus not equally likely to present, probability ratio of MinSeq $\left( k_{1}, g_{1},l_{1} \right)$ and MinSeq $\left( k_{2}, g_{2},l_{2} \right)$ is thus, $4^{{{(k}_{2}+l_{2})-(k}_{1}+l_{1})}\times\frac{\left( L-k_{1}-g_{1}-l_{1}+1 \right)}{\left( L-k_{2}-g_{2}-l_{2}+1 \right)}$.

Ordering MinSeqs based on their descending value of enrichment points out the best enrichment values and best binding sequence/MinSeqs possible, but if the $k+l$ value (part of sequence containing A/C/G/T) for the best MinSeq is higher, then it is less likely to occur in a random case as well as in genome, thus carries lesser information. Thus, there must be a trade-off between desired higher-enrichment value and desired lower $k+l$ value for a MinSeq.

This trade-off is the most important part in defining the value of a particular MinSeq in capturing the binding. We used minimum mean square error (MMSE) as the criteria, i.e. prefer/rank MinSeq $\left( k_{1}, g_{1},l_{1} \right)$ over MinSeq $\left( k_{2}, g_{2},l_{2} \right)$ if former gives a lesser MSE in predicting back the data (from which MinSeqs are derived) in comparison to the later.

*Lemma 1:* In predicting back the data, if $k_{1}=k_{2}$, MinSeq1 $\left( k_{1}, 0, 0 \right)$ gives lower MSE when compared to MinSeq2 $\left( k_{2}, 0, 0 \right)$ if $E_{\left( k_{1}, 0, 0 \right)}>E_{\left( k_{2}, 0, 0 \right)}$, where $E_{\left( k, g, l \right)}$ is enrichment of MinSeq $\left( k, g, l \right)$.

Proof: Considering enrichment for sequences of length $k$. MinSeq1 with enrichment $a$ and MinSeq2 with $b$. Since there will not other $k$-mer sequence matching these two MinSeqs. MSE in estimation can be minimized by minimizing MSE just for the two sequences matching MinSeq, MSE $= \left( a-a` \right)^{2} + \left( b-b` \right)^{2}$, where $a`$ is estimated enrichment for $a$. Thus, choosing MinSeq1 will give MSE $b^{2}$ whereas choosing MinSeq2 will give error $a^{2}$. Choose MinSeq1 over MinSeq2, if MSE for MinSeq1 is less i.e. $b^{2}<a^{2}$, which implies $a>b$ (enrichment values are non-negative) or $E_{\left( k_{1}, 0, 0 \right)}>E_{\left( k_{2}, 0, 0 \right)}$, hence proved the *Lemma 1*.

The proof can be extended to case where $k_{1}+l_{1}=k_{2}+l_{2}$, MMSE is achieved by ranking according to the higher enrichment (like $k$-mer enrichment).

*Lemma 2:* In predicting back the data, MinSeq $\left( k_{1}, g_{1},l_{1} \right)$ gives lower MSE when compared to MinSeq $\left( k_{2}, g_{2},l_{2} \right)$ if $E_{\left( k_{1}, g_{1},l_{1} \right)}\times2^{-\left( k_{1}+l_{1} \right)}>E_{\left( k_{2}, g_{2},l_{2} \right)}\times2^{-\left( k_{2}+l_{2} \right)}$, $E_{\left( k, g, l \right)}$ is enrichment of MinSeq $\left( k, g, l \right)$.

*Proof:* Consider two MinSeqs $\left( k_{1}, g_{1},l_{1} \right)$ and $\left( k_{2}, g_{2},l_{2} \right)$, such that $\left( k_{1}, g_{1},l_{1} \right)$ is direct subsequence of $\left( k_{2}, g_{2},l_{2} \right)$, such that $k_{1}+1=k_{2}$, $g_{1}=g_{2}$, $l_{1}=l_{2}$.

Let us consider $g_{1}=g_{2}=l_{1}=l_{2}=0$, i.e. the two sequence doesn’t contain any gap and aim is to minimize MSE for a $k$-mer binding data $k=k_{2}$ in a context of a much longer genomic sequence of i.e. length $\gg k$. Example MinSeq1=ACTA and MinSeq2=ACTAT, thus $k_{1}=4$, $k_{2}=5$ and trying to minimize MSE for a $5$-mer binding data. MinSeq1 can be left contained sub-sequence of $4$ different $5$-mer sequences ACTAA, ACTAC, ACTAG and ACTAT, whereas MinSeq2 is a sub-sequence of only ACTAT. Note, although MinSeq1 is right contained sub-sequence of $4$ other $5$-mers- AACTA, CACTA, GACTA and TACTA, but we don’t use those for MSE estimation for longer sequences. Sequences ACTAA, ACTAC, ACTAG and ACTAT covers all possibilities in AACTA, CACTA, GACTA and TACTA, example in AAAACTAAAAA - underlined sequence ACTAA is a $5$-mer which has left contained sub-sequence ACTA, this also captures AACTA, which has ACTA as right contained sub-sequence.

Let us assume enrichment for ACTA, ACTAA, ACTAC, ACTAG and ACTAT is $m$, $a$, $c$, $g$ and $t$ respectively. Further assumption made for simplification, $a=c=g$ and $t>a$. Given these details, we need to choose 1 MinSeq ACTA ($m$) or ACTAT ($t$) which minimizes MSE in estimating $a$, $c$, $g$ and $t$. MSE in estimation $=\left( a-a` \right)^{2} + \left( c-c` \right)^{2}+ \left( g-g` \right)^{2}+ \left( t-t` \right)^{2}$, where ($a`$ is estimated enrichment for $a$). Since enrichment of ACTA is average (mean) of enrichment of ACTAA, ACTAC, ACTAG and ACTAT -$4m=a+c+g+t$, this can be written as $4m=3a+t$.

If $m$ was picked as preferred MinSeq – $a`=m$, $c`=m$, $g`=m$, $t`=m$. Thus, MSE in estimation $=\left( a-m \right)^{2} + \left( c-m \right)^{2}+ \left( g-m \right)^{2}+ \left( t-m \right)^{2}=3\left( a-m \right)^{2}+\left( t-m \right)^{2}$. If $t$ was picked– $a`=0$, $c`=0$, $g`=0$, $t`=t.$ Thus, MSE in estimation $=\left( a-0 \right)^{2} + \left( c-0 \right)^{2}+ \left( g-0 \right)^{2}+ \left( t-t \right)^{2}=3a^{2}$.

Choose $t$ over $m$ if-

$3a^{2}<3\left( a-m \right)^{2}+\left( t-m \right)^{2}$, substitute $t=4m-3a$

$$4m^{2}-8am+3a^{2}>0$$

( )

OR $2m > 3a$

On further substituting,

$2m+t<0$ (Not possible as enrichment has to be positive only)

Other solution $2m<t$ i.e. $t>2m$

Thus, choose ACTAT ($t$) over ACTA ($m$) if $t>2m$ to minimize MSE. Similarly, can prove if $t<2m$ choose $m$ over $t$ for MMSE.

A general case thus, select MinSeq $\left( k_{1}, g_{1},l_{1} \right)$ over MinSeq $\left( k_{2}, g_{2},l_{2} \right)$, where $\left( k_{1}, g_{1},l_{1} \right)$ is direct subsequence of $\left( k_{2}, g_{2},l_{2} \right)$, such that $k_{1}+1=k_{2}$, $g_{1}=g_{2}=l_{1}=l_{2}=0$, if $E_{\left( k_{1}, g_{1},l_{1} \right)}\times2^{-k_{1}}>E_{\left( k_{2}, g_{2},l_{2} \right)}\times2^{-\left( k_{1}+1 \right)}$. And select MinSeq $\left( k_{2}, g_{2},l_{2} \right)$ over MinSeq $\left( k_{3}, g_{3},l_{3} \right)$ such that ($k_{2}+1=k_{3}$) if $E_{\left( k_{2}, g_{2},l_{2} \right)}\times2^{-\left( k_{1}+1 \right)}>E_{\left( k_{3}, g_{3},l_{3} \right)}\times2^{-\left( k_{1}+2 \right)}$. This implies select MinSeq $\left( k_{1}, g_{1},l_{1} \right)$ over MinSeq $\left( k_{3}, g_{3},l_{3} \right)$ if $E_{\left( k_{1}, g_{1},l_{1} \right)}\times2^{-k_{1}}>E_{\left( k_{3}, g_{3},l_{3} \right)}\times2^{-\left( k_{1}+2 \right)}$. This can be extended thus select MinSeq $\left( k_{1}, g_{1},l_{1} \right)$ over MinSeq $\left( k_{2}, g_{2},l_{2} \right)$, where $\left( k_{1}, g_{1},l_{1} \right)$ is a subsequence of $\left( k_{2}, g_{2},l_{2} \right)$, such that $g_{1}=g_{2}=l_{1}=l_{2}=0$ if $E_{\left( k_{1}, g_{1},l_{1} \right)}\times2^{-k_{1}}>E_{\left( k_{2}, g_{2},l_{2} \right)}\times2^{-k_{2}}$. With similar analogy constraint $g_{2}=l_{1}=l_{2}=0$ can be relaxed.

We used $\left( k_{1}, g_{1},l_{1} \right)$ is a subsequence of $\left( k_{2}, g_{2},l_{2} \right)$, this constraint is removed given *Lemma 1*. Thus, MinSeq $\left( k_{1}, g_{1},l_{1} \right)$ gives lower MSE when compared to MinSeq $\left( k_{2}, g_{2},l_{2} \right)$ if $E_{\left( k_{1}, g_{1},l_{1} \right)}\times2^{-\left( k_{1}+l_{1} \right)}>E_{\left( k_{2}, g_{2},l_{2} \right)}\times2^{-\left( k_{2}+l_{2} \right)}$. Till now we considered case where $k_{1}\leq k_{2}$, but same equation holds for $k_{1}\geq k_{2}$ as well. Hence proved the *Lemma 2*.

Thus, weighted enrichment of MinSeq $\left( k, g, l \right)=E_{\left( k, g, l \right)}\times2^{-\left( k+l \right)}$ is used when MinSeqs of different lengths $(k+l)$ are compared. Weighted enrichment ranks MinSeqs on the basis of their importance/predictive-capability in minimizing MSE.

# Compressed sensing based analysis

MinSeqs include all $k$ mer – $g$ spacer – $l$ mer sequences with varying $k$, $g$ and $l$. The data has a lot of redundancy, for example, if a protein prefers to bind to only a $4$-mer sequence ACGA, then the top MinSeq will be ACGA according to the weighted enrichment, but the algorithm may also capture ACGAA, ACGAC, … In essence, this includes all MinSeqs that cross the threshold of the minimum number of sequences $C^{T}$.

To capture binding information in a compressed set of MinSeqs (MinSeq set) we prune out the redundancies. In the above example, we can remove all $5$-mer and longer sequences, as they provide no additional information beyond the $4$-mer MinSeq ACGA. To algorithmically identify these pruning possibilities, we use compressed sensing (CS) based methods from the field of signal processing. Specifically, we use a modified orthogonal matching pursuit (OMP)^28–30^ approach to retrieve K-best MinSeqs (**Supplementary Fig. 1B**). Other techniques from signal processing literature such as compressive sampling matching pursuit (CoSaMP) ^31^ may also be used instead of OMP.

**Orthogonal Matching Pursuit (OMP) for MinSeq extraction**

OMP approach strives to minimize the “**residual weighted enrichment**” (see the algorithm below) for a given number of desired MinSeqs, i.e., this is the function which is being optimized by OMP. However, OMP is a greedy algorithm, the theoretical guarantees of when OMP finds the optimal solution are well described by Tropp, J. A. et al.^32^.

From binding data, we obtain:

**MinSeqs** $(k, g, l)$ with their corresponding enrichment $E_{\left( k, g, l \right)}$ and weighted enrichment $F_{(k, g, l)}=E_{\left( k, g, l \right)}\times2^{-\left( k+l \right)}$ (refer to “Weighted Enrichment of MinSeqs” section of **Supplementary Data 1**).

$\boldsymbol{a} =$ desired maximum AUROC (area under the receiver operating characteristic) curve for predicting back the raw data (bound data as a positive set and library as a negative set) to terminate the algorithm as a stopping criterion, and

$\boldsymbol{p} =$ maximum number of final MinSeqs to be used.

1. Initialize $i=0$, residual weighted enrichment $R=F$, set $S=\{\}$.

$i=i+1$

2. Choose MinSeq $(k_{i},g_{i},l_{i})$ with maximum R.
3. Add it to set S i.e. $S=\{S, MinSeq (k_{i},g_{i},l_{i})\}$
4. if $i>=p$ go to step 11. Else continue.
5. Estimate AUROC $= b$ for prediction using current set $S$.
6. If $b>=a$ go to step 11. Else continue.
7. Use weighted enrichment for set S to score all the MinSeqs in $F$ as $N$
8. Subtract to get residual weighted enrichment $R = F-N$
9. Go to step 2.
10. Set $S$ defines the final MinSeqs.

Instead of OMP, we also tried compressive sampling matching pursuit (CoSaMP)^31^ Under the assumption of some sparsity constraints, CoSaMP guarantees a local minimum “residual weighted enrichment” for a given number of MinSeqs. However, because OMP has much less complexity, we chose to proceed with this approach.

# MinSeqs to score sequences

Consider a sequence $v$-mer of length $v$ nucleotides to be scored using a set of MinSeqs with given enrichments. MinSeqs are of type $\left( k,g,l \right)$ with a maximum length $n$, where $k+g+l=n$. A moving window of length $n$ is used to score the $v$-mer sequence, resulting in $v-n+1$ sub-sequences ($v\geq n$). The following methods can then be used to score these $\left( v-n+1 \right)$ $n$-mers from the $v$-mer sequence. A maximum of all those scores is used as the final MinSeq score.

1. **Max Scoring with MinSeqs**

In this case, the sequence is assigned a score equal to the maximum of all the matching MinSeqs. Below we describe a procedure for scoring the $v$-mer.

For $i = 1$ to $v-n+1$ do

For the $n$-mer starting at $i$-th position, assign the score as **maximum** enrichment among all the MinSeqs contained in the $n$-mer.

Endfor

A maximum of all the above scores is used as the final MinSeq score for the $v$-mer.

Max scoring method (**maximum** in bold) can lead to the wrong conclusion if a sequence $S$ contains two MinSeqs $X$ and $Y$; $X$ has a higher enrichment than $Y$; and $X$ is a subsequence of $Y$ (refer to “Introducing MinSeqs” of **Supplementary Data1**). In such cases, max scoring method will assign the enrichment value of $S$ to that of $X$ and not $Y$, although a longer match implies a better match. This issue can be rectified using the following method.

1. **Scoring with the longest matching MinSeq**

For $i = 1$ to $v-n+1$ do

For $j = 0$ to $n-1$ do

For $q = 1$ to $n-j$ do

Find all MinSeqs matching the subseq starting at $i+j$-th basepair and length $q$ basepairs.

Reject MinSeqs which are direct-subsequence of another MinSeq covering the same nucleotide space.

Endfor

Endfor

For the $n$-mer starting at $i$-th position, assign the score as **maximum** enrichment among the remaining MinSeq set.

Endfor

1. **Scoring with the longest matching or derived MinSeq:**

Consider a situation if a sequence $S$ contains MinSeqs $X$ and $Xa$ (Nucleotide A at the end of MinSeq $X$). But MinSeq $Xa$, doesn’t have enough counts and didn’t cross the threshold so rejected, whereas $Xc$ crossed the threshold, but isn’t a subsequence of $S$. As we know the dependence between $X, Xa, Xc, Xg, Xt$ i.e., $Xa+Xc+Xg+Xt=4\times X$. We derive $Xa$ as

- $Xa = (4\times X-Xc)/3$ if only $X$ and $Xc$ crossed the threshold,
- $Xa = (4\times X-Xc-Xg)/2$ if only $X$, $Xc$ and $Xg$ crossed the threshold,
- $Xa = (4\times X-Xc-Xg-Xt)$ if only $X$, $Xc$, $Xg$ and $Xt$ crossed the threshold,

Similarly, we can calculate for other cases as well. This is the scoring mechanism for derived MinSeq for the cases when there exists MinSeqs $Y$ longer than the longest MinSeq $X$ in sequence $S$, such that $X$ is a subsequence of $Y$.

After scoring via longest matching or derived MinSeq, the score of the full $v$-mer sequence is assigned as the **maximum score over all the $\left( \boldsymbol{v}\boldsymbol{-}\boldsymbol{n+1} \right)$ $\boldsymbol{n}$-mer subsequences**.

In this study scoring of genomic sequences is done using the “longest matching or derived MinSeq” method described above.

We visually display MinSeq scores for these $v-n+1$ sub-sequences of length $n$ as color-coded bar plots and use them to predict binding potential to each sub-sequence across the genome (termed Genomescapes). To assign the binding score to the full DNA sequence (length $v$) we use the maximum binding intensity of all sub-sequences of length $n$. While we use the full list of MinSeqs for scoring, MinSeq compression can be used when there is a need to reduce the number of variables (redundancy). Moreover, the compression step is especially useful when analyzing a limited number of binding sequences, such as a comparison of binding patterns of different samples in **Fig.** **2c**.

# Position Associated Gapped LOcation-specific (PAGLO) model for MinSeqs

The $20$-mer DNA library used for experiments is not entirely random due to chemical synthesis and PCR amplification biases. To achieve accurate enrichment values, the counts of sequences enriched in the raw data must be divided by the number of times the same sequence appears in the initial DNA library. However, due to current technological limitations, only a fraction of the members of the initial library can be sequenced (typically $<{10}^{7}$ reads of the $>{10}^{15}$ DNA molecules present in multiple copies of ${10}^{12}$ distinct sequences that comprise a $20$-mer library). Even exhaustive sequencing of the DNA library would not cover all the sequences and therefore it is not possible to obtain actual number of counts for each $20$-mer in the library. Thus, enrichment of a sequence is estimated by taking ratio of observed fraction of the sequence (or MinSeq) in the bound sample and estimated fraction of that sequence in the random library. Fraction of the sequence in the library is estimated by dividing estimated counts by the total number of the sequences in the library. Counts of sequences are usually estimated using inhomogeneous $m$-th order Markov model, if there is sufficient representation of $(m+1)$-mer sequences. For MinSeqs/sequences comprising nucleotide gaps we developed a model called **P**osition **A**ssociated **G**apped **LO**cation-specific (PAGLO) model to better estimate the counts of sequences in the library.

Consider the MinSeq ACGTANNCG. A standard 5^th^ order Markov model estimates the counts of the sequence in library by multiplying the following five probabilities: a) ACGTA, b) N given ACGTA, c) N given CGTAN, d) C given GTANN, e) G given TANNC, where N is any nucleotide. This standard model assumes that the likelihood of a nucleotide at a particular position depends on the previous five nucleotides, ignoring the way a dimer typically binds to the DNA. When a dimer binds to a DNA site, there is usually a “gap” between the binding sites of its two halves. The nucleotides in this gap often minimally impact binding. To capture this physical effect, we propose a gapped Markov model called PAGLO, which estimates the counts by multiplying the following three probabilities: a) ACGTA, b) C given ACGTANN, c) G given CGTANNC. Since N represents any nucleotide, adding N after $m$-nucleotides doesn’t decrease the number of represented sequences; starting from a given position (inhomogeneity) there will be same number of ACGTA and ACGTANN and thus treating the Ns in the gap differently as compared to the other nucleotides. This is the basis of PAGLO model, a mathematical explanation for which is given below.

For example, to estimate the enrichment of a DNA sequence or MinSeq of length $n$, $x=[x_{1}x_{2}\ldots. x_{n}]$, where $x_{i}\in\{A,C,G,T,N\}$ is the $i$-th nucleotide of sequence $x$, where N is any nucleotide. Enrichment of $x$ with respect to the library can be defined as-

$$E\left( x \right)=\frac{B(x)}{R(x)}$$

( )

where $B\left( x \right)$ and $R\left( x \right)$ are the fractions of $x$ in the bound sample and in the random library sample reads (treated with Halo-beads to eliminate any contribution of the beads towards enrichment), respectively. We are assuming these fractions are much smaller than 1. We can get these fractions by: $B\left( x \right)=\frac{C_{b}\left( x \right)}{T_{b}}$ and $R\left( x \right)=\frac{C\left( x \right)}{T}$, where $C_{b}\left( x \right)$ and $C\left( x \right)$ are counts (number of times) of a sequence $x$ appeared in the bound samples and the library samples, respectively; $T_{b}$ and $T$ are the total number of sequence-reads in the bound samples and the library samples, respectively. Thus, enrichment can be written as

$$E\left( x \right)=\frac{B(x)}{R(x)}=\frac{C_{b}\left( x \right)/T_{b}}{C\left( x \right)/T}$$

( )

is counted by considering different MinSeqs $x$ of type $(k,g,l)$ type. A $(k, g, l)$-MinSeq is a $\boldsymbol{k}$-mer comprised of A, C, G, and T followed by a spacer (gap $\boldsymbol{g}$) sequence of $g >= 0$ Ns, where N denotes any nucleotide, followed by an $\boldsymbol{l}$-mer comprised of A, C, G, and T. For example, ACTNNGGTC is a $(k3,g2, l4)$ with ($k$) $3$-mer ACT, followed by a ($g$) spacer of $2$ nucleotides NN, followed by ($l$) $4$-mer GGTC. Enrichment $E\left( x \right)$ is considered only for sequences such that count $C_{b}\left( x \right)\geq C^{T}$for a chosen count threshold $C^{T}$. Threshold $C^{T}$ is chosen according to maximum tolerable error threshold (explained in detail below in the section “Poisson distributed reads and threshold cutoff for sequences”). $C\left( x \right)$ is required only for $\forall x, C_{b}\left( x \right)\geq C^{T}$. $C\left( x \right)$ for $\forall x$ is not necessarily greater than $C^{T}$ and can even be zero. We thus use estimated counts of $x$ in the library sample as $C^{*}\left( x \right)$. Thus, enrichment from estimated counts $C^{*}\left( x \right)$ can be written as-

$$E\left( x \right)=\frac{B\left( x \right)/T_{b}}{C^{*}\left( x \right)/T}$$

( )

The sequence $x$ is considered such that the length $L$ of the random region in the library sequences is greater than the length of sequence $x$, which is $k+g+l=n$, i.e. $L\geq n$. $C^{*}\left( x \right)$ is thus estimated as the sum of the number of times $x$ appeared starting at different positions of the library sequence.

$$C^{*}\left( x \right)=C_{1}^{*}\left( x \right)+C_{2}^{*}\left( x \right)+\ldots+C_{L-n+1}^{*}\left( x \right)$$

( )

where $C_{t}^{*}\left( x \right)$ is the estimated counts of the sequence $x$ starting at position t.

A probabilistic model is created to obtain the estimated count $C_{t}^{*}\left( x \right)$.

$$C_{t}^{*}\left( x \right)=P_{t}\left( x \right)\times T$$

( )

where $P_{t}\left( x \right)$ is the probability of finding sequence $x$ in any library sequence starting from position $t$.

Now consider an $m$-th order Markov model, such that all sequences of length $m$ are sufficiently represented in the library. For $m$-th order Markov model $(m<n)$, using Markov chain rule-

$$P_{t}\left( x \right)=P_{t}\left( x_{1}x_{2}\ldots x_{m}x_{m+1}\ldots x_{n} \right)$$

$$=P_{t}\left( x_{1}x_{2}\ldots x_{m} \right)P_{t}\left( x_{m+1} | x_{1}x_{2}\ldots x_{m} \right)P_{t+1}\left( x_{m+2} | x_{2}x_{3}\ldots x_{m+1} \right) \ldots P_{t+n-m-1}\left( x_{n} | x_{n-m}x_{n-m+1}\ldots x_{n-1} \right)$$

( )

Where $P_{t}\left( x_{1}x_{2}\ldots x_{m} \right)$ is the probability of finding sequence $x_{1}x_{2}\ldots x_{m}$ in the library sequence starting from position $t$. $P_{t}\left( x_{m+1} | x_{1}\ldots x_{m} \right)$ is Markov probability of nucleotide $x_{m+1}$ at position $t+m$ given previous $m$ nucleotides as $x_{1}\ldots x_{m}$ from position $t$ to $t+m-1$. These probabilities are calculated from the library data via maximum likelihood estimate-

$$P_{t}\left( x_{1}x_{2}\ldots x_{m} \right)=\frac{n_{t}\left( x_{1}x_{2}\ldots x_{m} \right)}{T}$$

( )

( )

Where $n_{t}\left( x_{1}\ldots x_{m}x_{m+1} \right)$ is the number of counts of sequences $x_{1}\ldots x_{m}x_{m+1}$ starting from position $t$.

Such a model is position specific Markov model (or inhomogeneous Markov model) as the model is specific to the position $t$ from where the sequence starts. Thus $P_{t}\left( x \right)$ is obtained, which is then used to calculate $C_{t}^{*}\left( x \right)$, and from that enrichment of sequence $x$ is estimated as $E^{*}\left( x \right)$.

Now considering $m$-th order Markov model with gap or spacers (stretches of Ns). Note that, we choose the order $m$ on the basis that all sequences of length $m$ are sufficiently represented in the library. Adding gaps or Ns (any nucleotide) in between doesn’t drastically change the number of sequences found in the library, unlike adding A, C, G or T. Thus, we can consider a new type of an $m$-th order Markov model with a gap or spacers (stretches of Ns) such that all sequences of length $m$ (without counting Ns) are sufficiently represented in the library.

The newly defined model consists of the probability of $x_{g+m+1}$ given $x_{1}\ldots x_{g+m}$, with a gap or spacer region (a stretch of Ns) $x_{d+1}{\ldots x}_{d+g}$ i.e., $x_{d+i}=N \forall1\leq i\leq g$, where gap $g\geq0$ and gap starting from $d\leq m$. Where $g=0$ means there is no gap, and it is the same as the Markov model without gaps. Thus-

$$x_{h}=\left\{ \begin{aligned} N \forall d+1\leq h\leq d+g \\ \left\{ A,C,G,T \right\} else \end{aligned} \right.$$

( )

The probability of such a model is also calculated using maximum likelihood criteria as:

$$P_{t}\left( x_{g+m+1} | x_{1}\ldots x_{d},g,x_{d+g+1}\ldots x_{g+m} \right)=\frac{n_{t}\left( x_{1}\ldots x_{d},g,x_{d+g+1}\ldots x_{g+m}x_{g+m+1} \right)}{\sum_{x_{g+m+1}} n_{t}\left( x_{1}\ldots x_{d},g,x_{d+g+1}\ldots x_{g+m}x_{g+m+1} \right)}$$

( )

Where $n_{t}\left( x_{1}\ldots x_{d},g,x_{d+g+1}\ldots x_{g+m}x_{g+m+1} \right)$ is the number of counts of sequences $x_{1}\ldots x_{g+m+1}$ with a gap length $g$ starting after $d$-th nucleotide i.e. $x_{d+1}{\ldots x}_{d+g}$, with sequence starting from $t$.

The probability of the full sequence with gap thus can be defined as:

$$P_{t}\left( x \right)=P_{t}\left( x_{1}x_{2}\ldots x_{k}x_{k+1}\ldots x_{k+g}x_{k+g+1}\ldots x_{n} \right)$$

( )

Where $x_{k+1}\ldots x_{k+g}$ is the gap region i.e. $x_{k+i}=N \forall1\leq i\leq g$

Let’s consider a case where $k>m$

$P_{t}\left( x \right)=P_{t}\left( x_{1}x_{2}\ldots x_{k}x_{k+1}\ldots x_{k+g}x_{k+g+1}\ldots x_{n} \right)$

$=P_{t}\left( x_{1}x_{2}\ldots x_{k} \right) P_{t}\left( x_{k+1}\ldots x_{k+g}x_{k+g+1}\ldots x_{n}|x_{1}x_{2}\ldots x_{k} \right)$

$=P_{t}\left( x_{1}x_{2}\ldots x_{k} \right) P_{t}\left( x_{k+g+1}\ldots x_{n}|x_{1}x_{2}\ldots x_{k} \right)$, since $x_{k+1}\ldots x_{k+g}$ are all Ns

$=P_{t}\left( x_{1}x_{2}\ldots x_{k} \right) P_{t+k-m}\left( x_{k+g+1}\ldots x_{n}|x_{k-m+1}\ldots x_{k} \right)$,

( )

as we are considering $m$-th order Markov model, so no dependency on nucleotides before $x_{k-m+1}$.

Now,

$$P_{t}\left( x_{1}x_{2}\ldots x_{k} \right)=P_{t}\left( x_{1}x_{2}\ldots x_{m} \right)P_{t}\left( x_{m+1} | x_{1}x_{2}\ldots x_{m} \right)P_{t+1}\left( x_{m+2} | x_{2}x_{3}\ldots x_{m+1} \right) \ldots P_{t+k-m-1}\left( x_{k} | x_{k-m}x_{k-m+1}\ldots x_{k-1} \right)$$

( )

And

$P_{t+k-m}\left( x_{k+g+1}\ldots x_{n}|x_{k-m+1}\ldots x_{k} \right)=P_{t+k-m}\left( x_{k+g+1}|x_{k-m+1}\ldots x_{k} \right)$

$\times P_{t+k-m+1}\left( x_{k+g+2}|x_{k-m+2}\ldots x_{k}x_{k+g+1} \right)\ldots P_{t+k-1}\left( x_{k+g+m}|x_{k}x_{k+g+1}\ldots x_{k+g+m-1} \right)$

$\times P_{t+k+g}\left( x_{k+g+m+1}|x_{k+g+1}\ldots x_{k+g+m-1} \right)\ldots P_{t+n-m-1}\left( x_{n}|x_{n-m}x_{n-m+1}\ldots x_{n-1} \right)$

( )

Together

$$P_{t}\left( x \right)=P_{t}\left( x_{1}x_{2}\ldots x_{m} \right)P_{l,t}\left( x_{m+1} | x_{1}x_{2}\ldots x_{m} \right)P_{t+1}\left( x_{m+2} | x_{2}x_{3}\ldots x_{m+1} \right) \ldots P_{t+k-m-1}\left( x_{k} | x_{k-m}x_{k-m+1}\ldots x_{k-1} \right)$$

$\times P_{t+k-m}\left( x_{k+g+1}|x_{k-m+1}\ldots x_{k} \right)$

$\times P_{t+k-m+1}\left( x_{k+g+2}|x_{k-m+2}\ldots x_{k}x_{k+g+1} \right)\ldots P_{t+k-1}\left( x_{k+g+m}|x_{k}x_{k+g+1}\ldots x_{k+g+m-1} \right)$

$\times P_{t+k+g}\left( x_{k+g+m+1}|x_{k+g+1}\ldots x_{k+g+m-1} \right)\ldots P_{t+n-m-1}\left( x_{n}|x_{n-m}x_{n-m+1}\ldots x_{n-1} \right)$

( )

We call this model with gaps the Position Associated Gapped LOcation-specific (PAGLO) model. Position associated as $P_{t}\left( x \right)$ depends on $t$-th position. Gapped and location specific as gaps are incorporated which are also position or location specific.

Now enrichment $E\left( x \right)$ of MinSeqs of type $(k,g,l)$ $E\left( x \right)=\frac{B\left( x \right)/T_{b}}{C^{*}\left( x \right)/T}$ can be estimated using estimated counts $C^{*}\left( x \right)$ and PAGLO model probabilities $P_{t}\left( x \right)$ as defined above.

# Poisson distributed reads and threshold cutoff for sequences

To sequence, we amplify and barcode the DNA samples. As noted above, however, only a fraction of the sample is sequenced. Let’s say $C_{b}\left( x \right)$ is the count of occurrences of any sequence or MinSeqs $x$ in the sequenced data. Assuming that $C_{b}\left( x \right)$ is Poisson distributed with expected value $\lambda$. The standard deviation and standard error for such a Poisson distribution is $\sqrt{\lambda}$ and $\sqrt{\frac{\lambda}{T_{b}}}$ respectively, where $T_{b}$ is the total number of reads in the bound sample. Thus, the fraction of standard deviation and error to the expected value $\lambda$ is $\frac{1}{\sqrt{\lambda}}$ and $\sqrt{\frac{1}{{\lambda T}_{b}}}$ respectively. Thus, higher expected value $\lambda$ results in a lower fraction of standard deviation. Also, higher $\lambda$ and higher $T_{b}$ result in lower standard error. As $\lambda$ is not known we use $C_{b}\left( x \right)$ as a proxy to restrict the error in the observed fraction. We generally consider data only if $T_{b}\geq10,000$ and $C_{b}\left( x \right)\geq50$. $C_{b}\left( x \right)=50$ results in standard deviation fraction of $\frac{1}{\sqrt{\lambda}}\approx\frac{1}{\sqrt{C_{b}\left( x \right)}}=\frac{1}{\sqrt{50}}=0.14$. That is, considering sequences only above a threshold of $C^{T}=50$ counts (i.e., using cutoff for $C_{b}\left( x \right)\geq50$), results in less than or equal to 14% of standard deviation in the estimated results. Using a higher threshold $C^{T}$ will result in the loss of valuable information as we’ll miss out on data for many MinSeqs, while a lower threshold $C^{T}$ will result in higher standard error. $C^{T}=50$ provides a good balance in this tradeoff. We systematically examined different thresholds and settled on $C_{b}\left( x \right)\geq50$ as our cutoff for this study.

# MinSeqs and PWMs derived from *MinSeq Find* for HT-SELEX data from Jolma et al.

We also applied *MinSeq Find* algorithm to high-throughput SELEX data obtained from other study^5^. HT-SELEX data for multiple rounds of selection is available, we used results of the round used by the authors in the paper to compare. Previous round data was used to normalize against the provided round data (mostly round 3 data was used by Jolma et al. of the TF to get enriched MinSeqs and PWMs from those MinSeqs.

HT-SELEX raw data suffers from lot of experimental noise and variability. There are many N-mer sequences in TF-DNA binding data of N-mer size which appears multiple (>20) times and are not related to TF binding, but are artifacts of experimental methods. We got rid of all N-mer sequences which appeared at least 20 times more than its reverse complementary sequence in the raw data, we also removed 1 mismatch to those sequences since sequencer sometimes can give error in reads. Further there were few sequences and patterns (shorter than N-mer) appeared randomly in high numbers because those were carried over and exponentially enriched by PCR, we didn’t remove such sequences as one cannot distinguish if which are really binding to TF and which are not.

**References**

1. Schneider, T. D. & Stephens, R. M. Sequence logos: a new way to display consensus sequences. *Nucleic Acids Res* **18**, 6097–6100 (1990).

2. Stormo, G. D., Schneider, T. D. & Gold, L. Quantitative analysis of the relationship between nucleotide sequence and functional activity. *Nucleic Acids Res* **14**, 6661–79 (1986).

3. Schneider, T. D., Stormo, G. D., Gold, L. & Ehrenfeucht, a. Information content of binding sites on nucleotide sequences. *J Mol Biol* **188**, 415–31 (1986).

4. Jolma, A. *et al.* Multiplexed massively parallel SELEX for characterization of human transcription factor binding specificities. *Genome Res* **20**, 861–873 (2010).

5. Jolma, A. *et al.* DNA-binding specificities of human transcription factors. *Cell* **152**, 327–339 (2013).

6. Berger, M. F. *et al.* Compact, universal DNA microarrays to comprehensively determine transcription-factor binding site specificities. *Nat Biotechnol* **24**, 1429–1435 (2006).

7. Badis, G. *et al.* Diversity and complexity in DNA recognition by transcription factors. *Science* **324**, 1720–1723 (2009).

8. Warren, C. L. *et al.* Defining the sequence-recognition profile of DNA-binding molecules. *Proc Natl Acad Sci U S A* **103**, 867–872 (2006).

9. Slattery, M. *et al.* Cofactor binding evokes latent differences in DNA binding specificity between hox proteins. *Cell* **147**, 1270–1282 (2011).

10. Maerkl, S. J. & Quake, S. R. A systems approach to measuring the binding energy landscapes of transcription factors. *Science* **315**, 233–7 (2007).

11. Nutiu, R. *et al.* Direct measurement of DNA affinity landscapes on a high-throughput sequencing instrument. *Nat Biotechnol* **29**, 659–664 (2011).

12. Zykovich, A., Korf, I. & Segal, D. J. Bind-n-Seq: high-throughput analysis of in vitro protein–DNA interactions using massively parallel sequencing. *Nucleic Acids Res* **37**, e151–e151 (2009).

13. Carlson, C. D. *et al.* Specificity landscapes of DNA binding molecules elucidate biological function. *Proc Natl Acad Sci U S A* **107**, 4544–4549 (2010).

14. Tietjen, J. R., Donato, L. J., Bhimsaria, D. & Ansari, A. Z. Sequence-specificity and energy landscapes of DNA-binding molecules. *Methods Enzymol* **497**, 3–30 (2011).

15. Stormo, G. D. & Zhao, Y. Determining the specificity of protein-DNA interactions. *Nat Rev Genet* **11**, 751–760 (2010).

16. Fordyce, P. M. *et al.* De novo identification and biophysical characterization of transcription-factor binding sites with microfluidic affinity analysis. *Nat Biotechnol* **28**, 970–975 (2010).

17. Campbell, Z. T. *et al.* Cooperativity in RNA-Protein Interactions: Global Analysis of RNA Binding Specificity. *Cell Rep* **1**, 570–581 (2012).

18. Meng, X., Brodsky, M. H. & Wolfe, S. a. A bacterial one-hybrid system for determining the DNA-binding specificity of transcription factors. *Nat Biotechnol* **23**, 988–994 (2005).

19. Noyes, M. B. *et al.* Analysis of Homeodomain Specificities Allows the Family-wide Prediction of Preferred Recognition Sites. *Cell* **133**, 1277–1289 (2008).

20. Weirauch, M. T. *et al.* Evaluation of methods for modeling transcription factor sequence specificity. *Nat Biotechnol* **31**, 126–34 (2013).

21. Alipanahi, B., Delong, A., Weirauch, M. T. & Frey, B. J. Predicting the sequence specificities of DNA- and RNA-binding proteins by deep learning. *Nat Biotechnol* **33**, 831–838 (2015).

22. Nitta, K. R. *et al.* Conservation of transcription factor binding specificities across 600 million years of bilateria evolution. *Elife* **4**, e04837 (2015).

23. Zhao, Y. & Stormo, G. D. Quantitative analysis demonstrates most transcription factors require only simple models of specificity. *Nat Biotechnol* **29**, 480–483 (2011).

24. Chen, X., Hughes, T. R. & Morris, Q. RankMotif++: a motif-search algorithm that accounts for relative ranks of K-mers in binding transcription factors. *Bioinformatics* **23**, i72-9 (2007).

25. Foat, B. C., Morozov, A. V & Bussemaker, H. J. Statistical mechanical modeling of genome-wide transcription factor occupancy data by MatrixREDUCE. *Bioinformatics* **22**, e141-9 (2006).

26. Annala, M., Laurila, K., Lähdesmäki, H. & Nykter, M. A linear model for transcription factor binding affinity prediction in protein binding microarrays. *PLoS One* **6**, e20059 (2011).

27. Riley, T. R., Lazarovici, A., Mann, R. S. & Bussemaker, H. J. Building accurate sequence-to-affinity models from high-throughput in vitro protein-DNA binding data using featureREDUCE. *Elife* **4**, e06397 (2015).

28. Duarte, M. F. & Eldar, Y. C. Structured compressed sensing: From theory to applications. *IEEE Transactions on Signal Processing* **59**, 4053–4085 (2011).

29. Pati, Y. C. C., Rezaiifar, R. & Krishnaprasad, P. S. S. Orthogonal matching pursuit: recursive function approximation with applications to wavelet decomposition. in *Conference Record of the Asilomar Conference on Signals, Systems & Computers* vol. 1 40–44 (1993).

30. Mallat, S. G. & Zhang, Z. Matching Pursuits With Time-Frequency Dictionaries. *IEEE Transactions on Signal Processing* **41**, 3397–3415 (1993).

31. Needell, D. & Tropp, J. A. CoSaMP: Iterative signal recovery from incomplete and inaccurate samples. *Communications of the ACM* **53** (12), 93–100 (2010).

32. Tropp, J. A. & Gilbert, A. C. Signal recovery from random measurements via orthogonal matching pursuit. *IEEE Trans Inf Theory* **53**, 4655–4666 (2007).
