# Supplementary material for: Hidden modes of DNA binding by human nuclear receptors
Source: Nat Commun. 2023 Jul 13;14:4179. doi: 10.1038/s41467-023-39577-0 (PMC10345098; doi:10.1038/s41467-023-39577-0)
Supplement: Supplementary file 5 — Supplementary Data 3 [file 41467_2023_39577_MOESM5_ESM.pdf]

Supplementary Data 3: MinSeq Find derived PWM logos for Nuclear Receptors in this study

Contents

1 MR Round 3 5

2 MR+1 Round 3 8

3 PGR Round 3 11

4 PGR+2 Round 3 14

5 GR Round 3 17

6 GR+3 Round 3 20

7 ESRRG Round 3 23

8 ESRRG:RXRA Round 3 26

9 ESRRG+4 Round 3 29

10 ESRRB Round 3 32

11 ESRRB:RXRA Round 3 35

12 ESRRB+4 Round 3 38

13 ESRRA Round 3 41

14 ESRRA:RXRA Round 3 44

15 ESRRA+4 Round 3 47

16 ESR1+5 Round 3 50

17 ESR1+6 Round 3 53

18 THRB Round 3 56

19 THRB:RXRA Round 3 59

20 THRB:RXRA+7 Round 2 62

21 THRB+7 Round 3 65

22 THRA Round 3 68

23 THRA:RXRA Round 3 **71**

24 THRA:RXRA+7 Round 2 **74**

25 THRA+7 Round 3 **77**

26 RARG Round 3 **80**

27 RARB Round 3 **83**

28 RARA Round 3 **86**

29 RARG+8 Round 2 **89**

30 RARB+8 Round 3 **92**

31 RARA+8 Round 3 **95**

32 RARG:RXRA Round 3 **98**

33 RARB:RXRA Round 3 **101**

34 RARA:RXRA Round 3 **104**

35 PXR Round 3 **107**

36 PXR+9 Round 3 **110**

37 VDR Round 3 **113**

38 VDR:RXRA Round 3 **116**

39 VDR+10 Round 2 **119**

40 FXR Round 3 **122**

41 FXR+11 Round 3 **125**

42 LXRA Round 3 **128**

43 LXRA+12 Round 3 **131**

44 LXRB:RXRA Round 3 **134**

45 PPARD Round 3 **137**

46 PPARD+13 Round 3 **140**

47 PPARD+14 Round 3 **143**

48 PPARG Round 3 **146**

49 PPARG+14 Round 3 **149**

50 PPARG+15 Round 3 **152**

51 Rev-ErbA-Alpha Round 3 **155**

52 RORC Round 3 **158**

53 RORC:RXRA Round 3 **161**

54 RORC+16 Round 3 **164**

55 TR4 Round 3 **167**

56 TR2 Round 3 **170**

57 LRH1 Round 3 **173**

58 LRH1:RXRA Round 3 **176**

59 SF1 Round 3 **179**

60 SF1:RXRA Round 3 **182**

61 TLX Round 3 **185**

62 TLX:RXRA Round 3 **188**

63 PNR Round 3 **191**

64 COUP-TF2 Round 3 **194**

65 COUP-TF1 Round 3 **197**

66 EAR2 Round 3 **200**

67 COUP-TF2+17 Round 3 **203**

68 COUP-TF1+17 Round 3 **206**

69 COUP-TF2:RXRA Round 3 **209**

70 COUP-TF1:RXRA Round 3 **212**

71 EAR2:RXRA Round 3 **215**

72 HNF4G Round 3 **218**

73 HNF4A Round 3 **221**

74 HNF4A+18 Round 3 **224**

75 RXRB Round 3 **227**

76 RXRB+17 Round 3 **230**

77 RXRG Round 3 **233**

78 RXRG:RXRA Round 3 **236**

79 RXRG+17 Round 3 **239**

80 RXRA Round 3 **242**

81 RXRA+17 Round 3 **245**

82 NOR1 Round 3 **248**

83 NURR1 Round 3 **251**

1 MR Round 3

PWM	Seed Sequence	Seed Seq Enrichment	Repeat
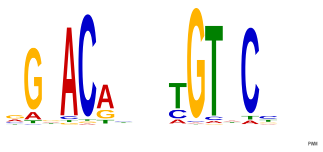	*NNNGGACANNNTGTACNNN*	105.26	IR:3
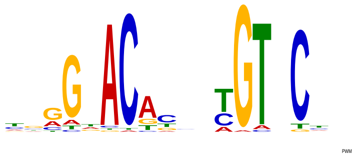	*NGGGTACANNNNGTCCTN*	163.09	IR:3
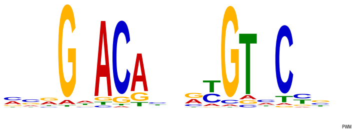	*NNAGAACANNNNGTCCTNN*	88.44	IR:3
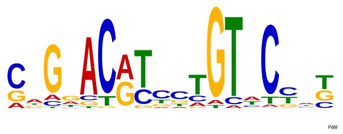	*NNNGGACGTNCTGTACNNN*	148.90	IR:3
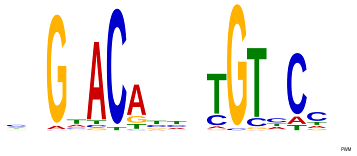	*NCGTACATNNTGTCCCN*	235.59	IR:3
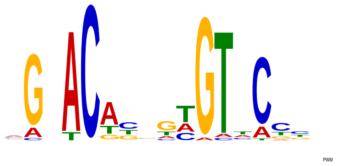	*NAGTACANNNNGTACCCN*	110.66	IR:3
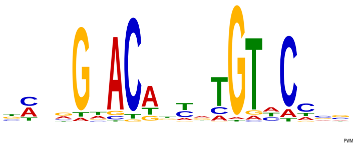	*NNNCGTACANNNTGTCCNNN*	66.01	IR:3

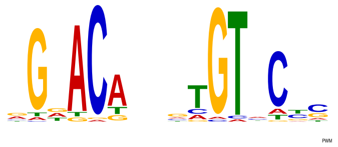	NAGAACANNNTGTGCCN	86.97	IR:3
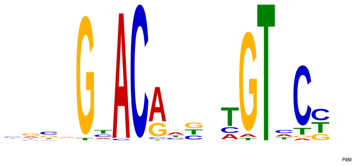	NNCTGTACANNNTGTCCNN	90.80	IR:3
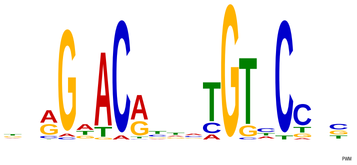	NNAGAACGNNNTGTCCCNN	116.01	IR:3
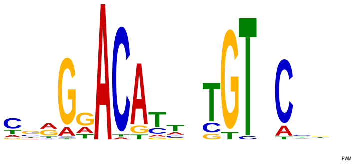	NNNGGACATTNTGTGCNNN	129.30	IR:3
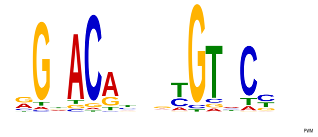	NNNAGAACNNNNTGTCCNNN	50.46	IR:3
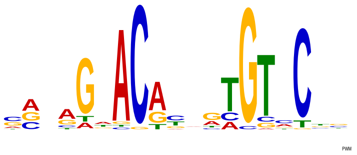	NNAAGAACANNNTGTCCNN	147.61	IR:3
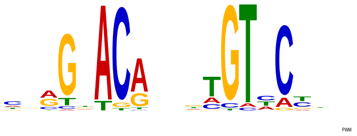	NNGGCACANNNNGTTCTNN	36.83	IR:3

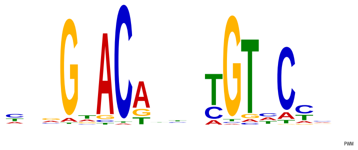	$NN\overrightarrow{CGTACA}NNN\overleftarrow{NGTCCC}NN$	63.22	IR:3
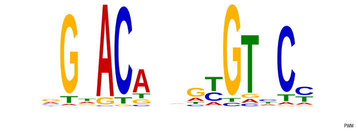	$NN\overrightarrow{CGTACA}NNN\overleftarrow{NGTCCT}NN$	41.18	IR:3
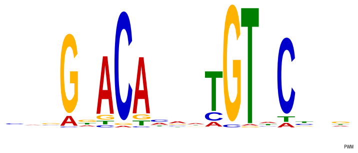	$NNN\overrightarrow{GGACAA}A\overleftarrow{NTGTTC}NNN$	134.55	IR:3
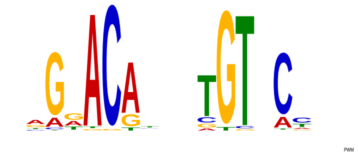	$NN\overrightarrow{GGACAT}NN\overleftarrow{TGTGCC}NN$	131.08	IR:3
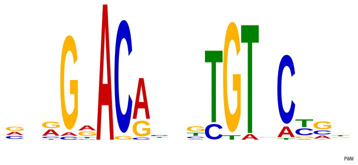	$NN\overrightarrow{AGAACA}NNN\overleftarrow{TGTGCT}NN$	90.55	IR:3
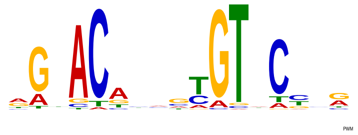	$N\overrightarrow{GGACAA}NNN\overleftarrow{GTTCCC}N$	74.93	IR:3

2 MR+1 Round 3

PWM	Seed Sequence	Seed Seq Enrichment	Repeat
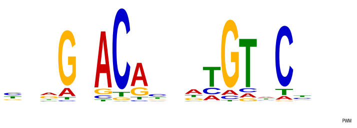	*NNNGGACANNNNTGTACNNN*	31.38	IR:3
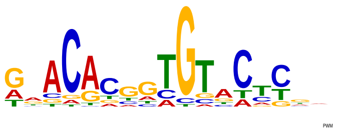	*NNNACACGGNGTACTNNN*	25.89	IR:3
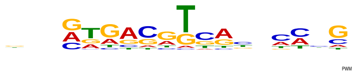	*NNNNTGACGTCACGNNNN*	3.70	M
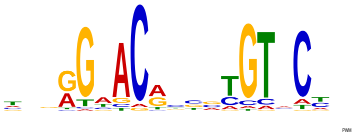	*NNGGGAACNNNNTGTACNN*	39.78	IR:3
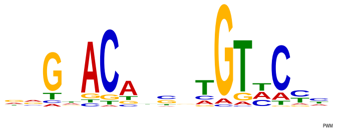	*NNAGCACANNNTGTCCNN*	20.15	IR:3
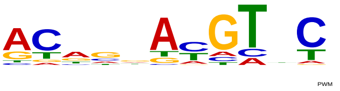	*ACAGGACGTCC*	10.04	M
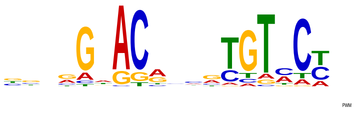	*NNCGGAACNNNNTGTCCNN*	27.03	IR:3

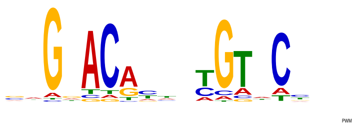	*NNGGACATNNNGTACTNN*	28.22	IR:3
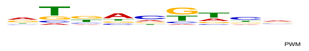	*AGTGACGTCA*	2.41	M
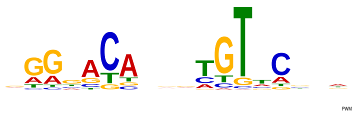	*NNNGACAAANTGTTCNNN*	12.05	IR:3
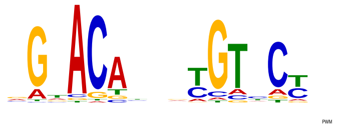	*NCGTACANNNNGTCCTN*	18.10	IR:3
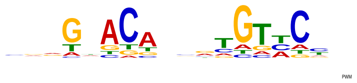	*NNAGCACNNNNTGTCCNN*	8.86	IR:3
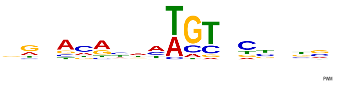	*NNGGACANNNNNNTACGGNN*	3.63	DR:3
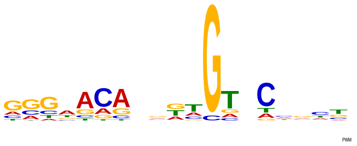	*NNGAACANNNNNNTACAGNN*	5.18	DR:3

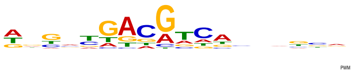	NNNNTGACGTCACANNNN	4.11	M
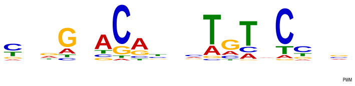	NNAGGACNNNNNGTACGNN	6.03	DR:3
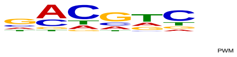	GGTGACGTCA	3.04	M
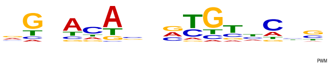	NAGCACNNNNNGTCCTN	5.44	IR:3
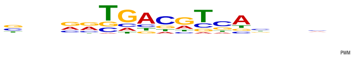	NNNNATGACGTCACNNNN	4.36	M
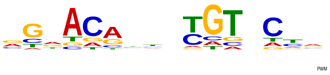	NAGAACNNNNNGTGCTN	6.93	IR:3

3 PGR Round 3

PWM	Seed Sequence	Seed Seq Enrichment	Repeat
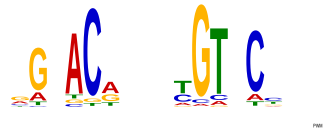	NNNGGACANNNTGTACNNN	63.02	IR:3
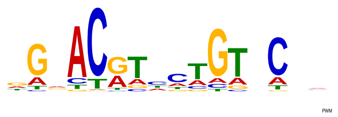	NNGGACGTNCTGTACNN	144.93	IR:3
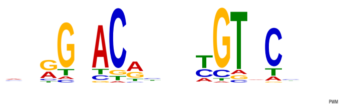	NNNAGAACNNNNTGTCCNNN	44.10	IR:3
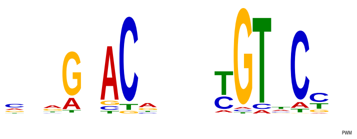	NTAGGACNNNNNGTTCCN	43.84	IR:3
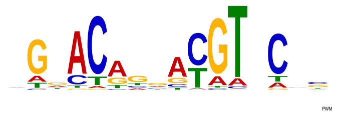	NNGGACAGNACGTACNN	123.08	IR:3
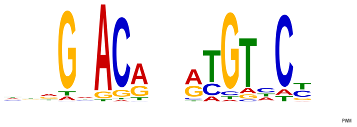	NNAGCACANNNNGTCCTNN	44.07	IR:3
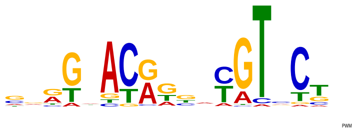	NNGGAACANNACGTCCNN	104.91	IR:3

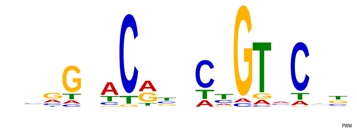	$NN\overrightarrow{AGAACG}TNN\overleftarrow{TGTCC}NN$	83.19	IR:3
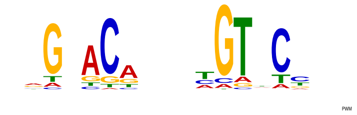	$NN\overrightarrow{GGACA}ANN\overleftarrow{NGTTCT}NN$	40.16	IR:3
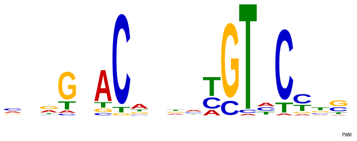	$NN\overrightarrow{GGCACA}NNN\overleftarrow{NGTTCT}NN$	37.07	IR:3
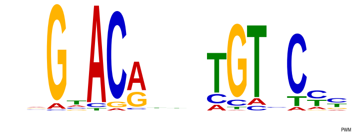	$N\overrightarrow{CGTACA}TNN\overleftarrow{NGTCCC}N$	67.28	IR:3
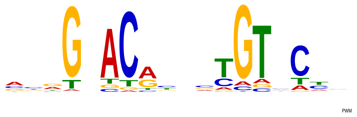	$NN\overrightarrow{AGTACG}NNN\overleftarrow{TGTCC}NN$	55.41	IR:3
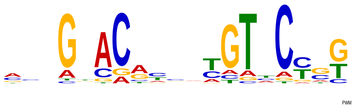	$NNT\overrightarrow{GGACA}NNN\overleftarrow{NGTTCT}NN$	29.04	IR:3
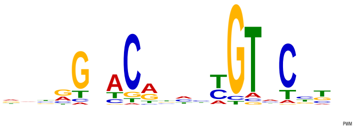	$NNN\overrightarrow{AGAACG}NNN\overleftarrow{TGTCC}NNN$	52.95	IR:3

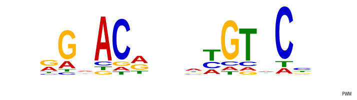	$NN\overrightarrow{AGGACN}NNNN\overrightarrow{GTACG}NN$	16.63	DR:3
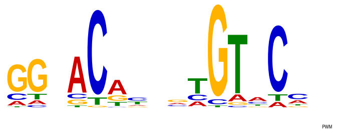	$N\overrightarrow{NGGACA}CGN\overleftarrow{TGTAC}NN$	101.02	IR:3
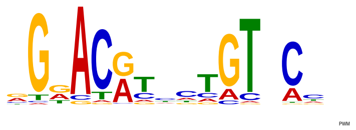	$\overrightarrow{NGGACG}TNN\overleftarrow{TGTGCC}N$	79.20	IR:3
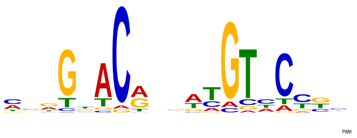	$NN\overrightarrow{AGCACA}NNN\overleftarrow{NGTTCT}NN$	31.69	IR:3
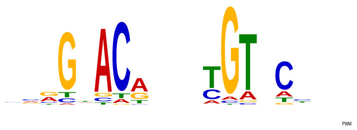	$NN\overrightarrow{AGAACA}NNN\overleftarrow{NGTCCC}NN$	59.99	IR:3
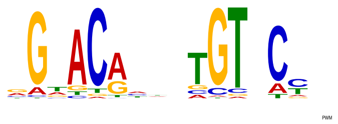	$N\overrightarrow{CGAACA}NNN\overleftarrow{TGTCCC}N$	62.32	IR:3

4 PGR+2 Round 3

PWM	Seed Sequence	Seed Seq Enrichment	Repeat
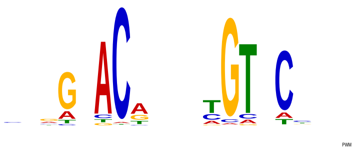	NNNGGACANNNTGTACNNN	113.79	IR:3
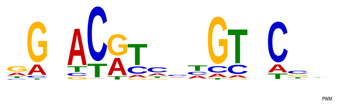	NNGGACGTNCTGTACNN	170.68	IR:3
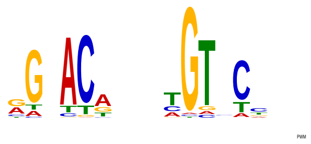	NNNAGAACNNNNTGTCCNNN	83.56	IR:3
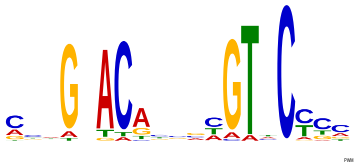	NNAGTACANNNCGTTCCNN	175.20	IR:3
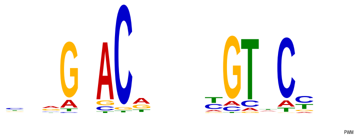	NNAGGACNNNNNGTTCCNN	69.30	IR:3
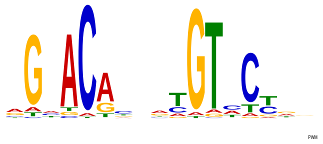	NNAGCACANNNNGTCCTNN	87.48	IR:3
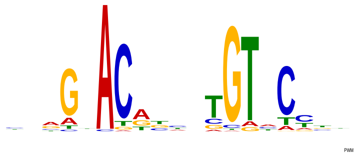	NNGGCACANNNNGTCCTNN	61.75	IR:3

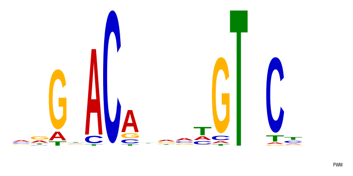	$NN\overrightarrow{AGGACA}NNN\overleftarrow{TGTGCC}NN$	135.67	IR:3
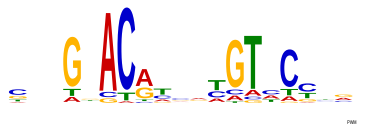	$NN\overrightarrow{AGCACA}NNN\overleftarrow{NGTTCT}NN$	54.51	IR:3
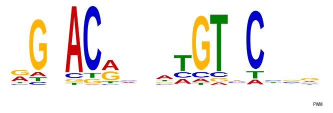	$NN\overrightarrow{AGAACN}NNN\overrightarrow{NGTACG}NN$	30.02	DR:3
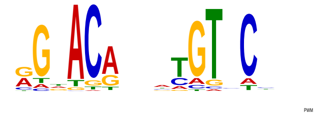	$NNN\overrightarrow{AGAACN}NNN\overleftarrow{TGTGCN}NN$	50.62	IR:3
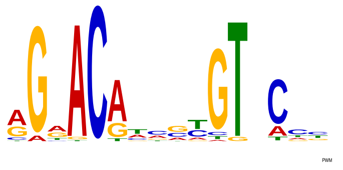	$N\overrightarrow{AGAACA}TNN\overleftarrow{NGTGCC}N$	134.42	IR:3
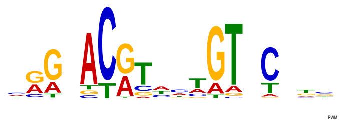	$NN\overrightarrow{NGAACG}TNC\overleftarrow{TGTCCN}NN$	149.41	IR:3
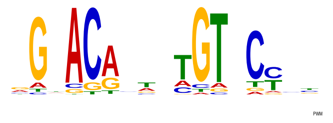	$NN\overrightarrow{CGAACA}NNN\overleftarrow{NGTCCT}NN$	50.56	IR:3

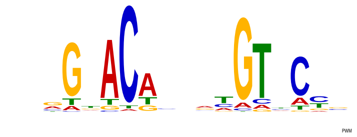	$N G\overrightarrow{GGTACN}NNN\overleftarrow{NGTTCC}N$	85.71	IR:3
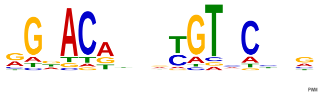	$NN\overrightarrow{AGAACN}NNN\overleftarrow{NGTGCT}NN$	25.34	IR:3
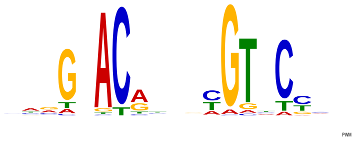	$NN\overrightarrow{AGCACA}NNN\overleftarrow{NGTTCC}NN$	82.15	IR:3
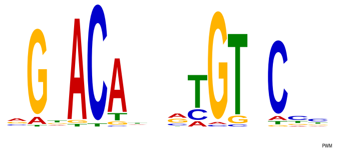	$N\overrightarrow{CGTACA}NNN\overleftarrow{TGTGCC}N$	96.29	IR:3
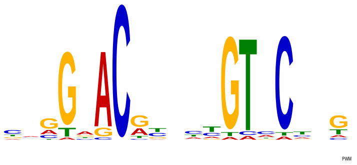	$NN\overrightarrow{GGAACG}TNN\overleftarrow{NGTCCT}NN$	150.89	IR:3
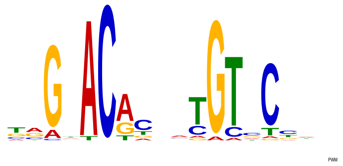	$NN\overrightarrow{AGTACA}NNN\overleftarrow{TGTGCC}NN$	174.98	IR:3

5 GR Round 3

PWM	Seed Sequence	Seed Seq Enrichment	Repeat
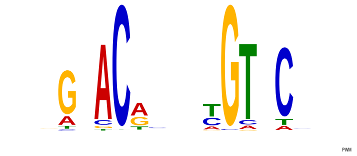	*NNNGGACANNNNTGTACNNN*	1082.88	IR:3
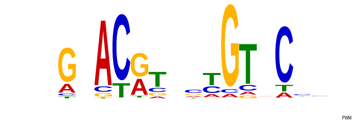	*NNNGGACGTNCTGTACNNN*	2135.34	IR:3
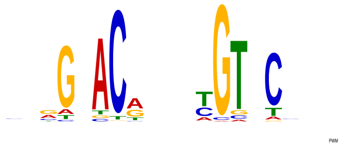	*NNNAGAACNNNNTGTCCNNN*	567.17	IR:3
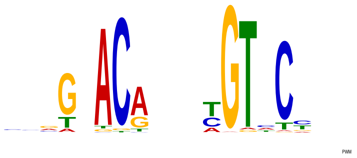	*NNGGCACANNNNNGTCCTNN*	612.27	IR:3
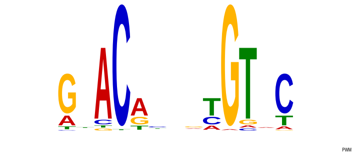	*NNNGGACACNGTGTCCNNN*	1579.49	IR:3
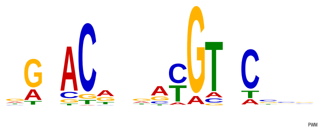	*NNNGGACAGNACGTACNNN*	1246.67	IR:3
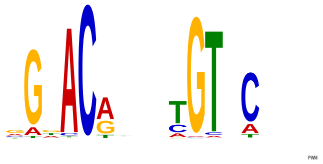	*NNNGGACATNNTGTGCNNN*	899.11	IR:3

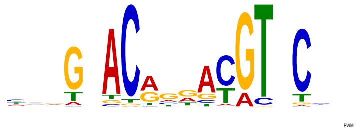	$NN\overrightarrow{AGCACA}GNA\overleftarrow{CGTCC}NN$	1828.62	IR:3
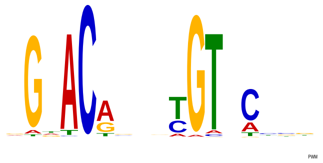	$NN\overrightarrow{AGTACA}NNN\overleftarrow{TGTGCC}NN$	1194.46	IR:3
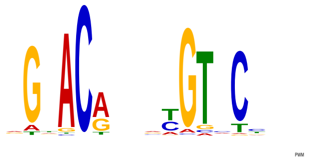	$NNN\overrightarrow{AGTACA}NNN\overleftarrow{CGTCC}NNN$	1035.53	IR:3
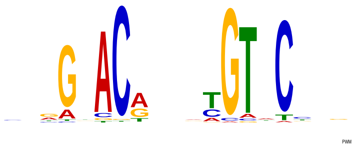	$NN\overrightarrow{AGGACN}NNN\overrightarrow{NGTACG}NN$	249.88	DR:3
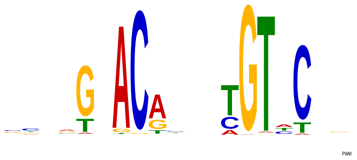	$NNT\overrightarrow{GGCACA}NNN\overleftarrow{TGTCC}NN$	797.13	IR:3
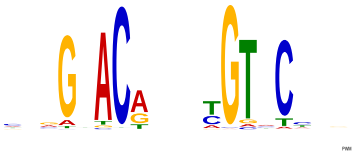	$NN\overrightarrow{CGAACA}NNN\overleftarrow{NGTCCT}NN$	420.42	IR:3
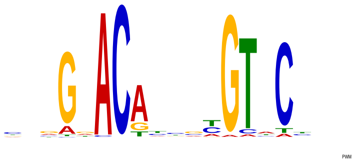	$NNN\overrightarrow{GGACAT}NG\overleftarrow{CGTAC}NNN$	1443.85	IR:3

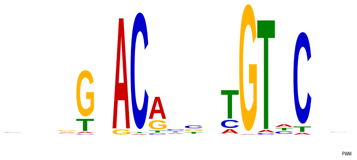	$NNT\overrightarrow{GGCACA}NNN\overleftarrow{TGTAC}NN$	870.21	IR:3
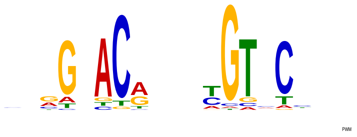	$NN\overrightarrow{AGAACN}NNN\overleftarrow{NGTCCT}NN$	314.19	IR:3
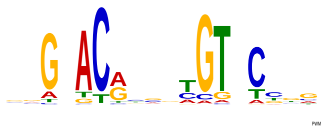	$NNN\overrightarrow{AGTACG}TNN\overleftarrow{TGTCC}NNN$	1159.29	IR:3
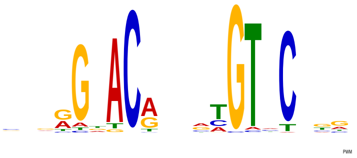	$NNG\overrightarrow{GGTACN}NNN\overrightarrow{NGTACG}NN$	477.96	DR:3
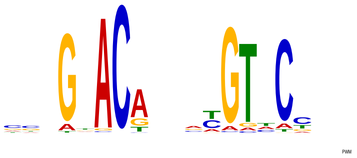	$NN\overrightarrow{CGTACA}NNN\overleftarrow{CGTTCC}NN$	1039.28	IR:3
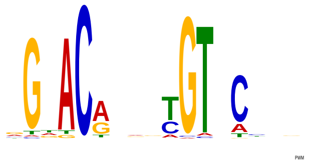	$NNN\overrightarrow{AGTACA}NNN\overleftarrow{TGTGC}NNN$	857.31	IR:3

6 GR+3 Round 3

PWM	Seed Sequence	Seed Seq Enrichment	Repeat
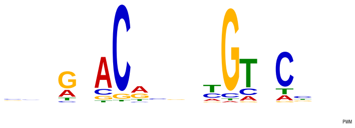	NNNGGACANNNTGTACNNN	48.95	IR:3
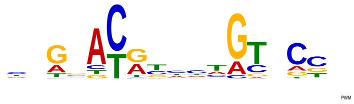	NNGGACGTNCTGTACNN	76.93	IR:3
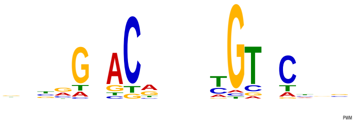	NNNAGAACNNNNTGTCCNNN	31.38	IR:3
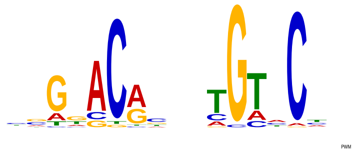	NNGGACACNTTGTACNN	77.76	IR:3
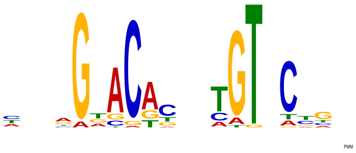	NNNAGTACANNNTGTGCNNN	68.23	IR:3
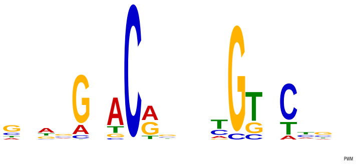	NNNCGTACANNNTGTCCNNN	46.76	IR:3
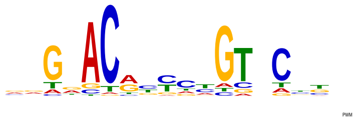	NNGGACGTNNNGTACTNN	28.82	IR:3

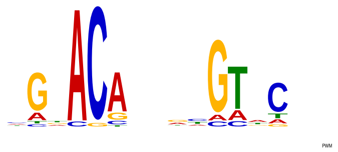	NNGGACATNGCGTACNN	62.52	IR:3
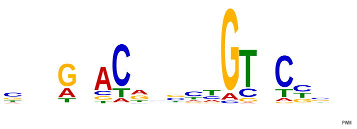	NNNGGACGTNNTGTACNNN	37.44	IR:3
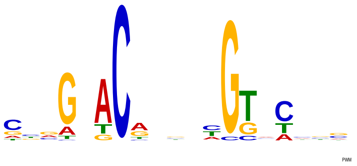	NNCGTACANNNTGTCCCNN	56.66	IR:3
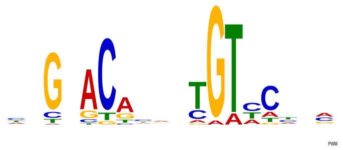	NNNGCACACNATGTCCNNN	67.23	IR:3
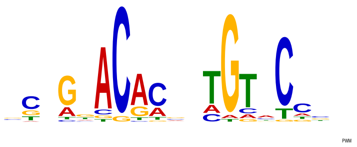	NNCGGACANNNTGTACCNN	91.00	IR:3
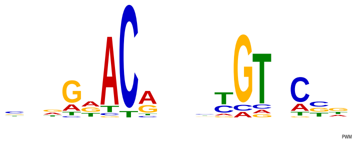	NNGGGACATNNTGTGCNN	66.35	IR:3
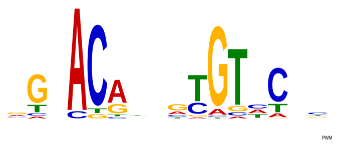	NAGCACANNNTGTCCCN	65.80	IR:3

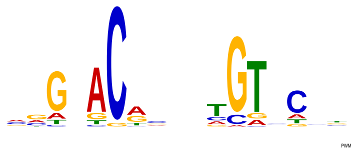	$NN\overrightarrow{GTACA}CTN\overleftarrow{TGTCC}NN$	64.32	IR:3
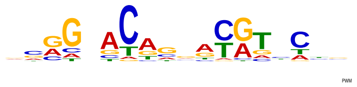	$NN\overrightarrow{NGTACA}GNA\overleftarrow{CGTCN}NN$	31.22	IR:3
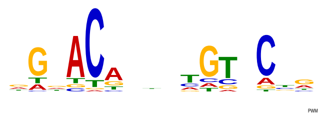	$NN\overrightarrow{NGGACA}T\overleftarrow{NGTGTG}NNN$	15.49	IR:1
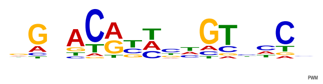	$NN\overrightarrow{NGAACG}TNC\overleftarrow{TGTCN}NN$	21.59	IR:3
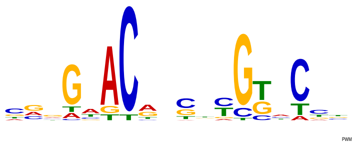	$NN\overrightarrow{CGTACA}TNN\overleftarrow{TGTCC}NN$	41.58	IR:3
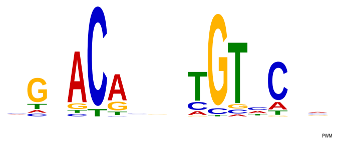	$NN\overrightarrow{GCACA}CTN\overleftarrow{TGTCC}NN$	51.48	IR:3

7 ESRRG Round 3

PWM	Seed Sequence	Seed Seq Enrichment	Repeat
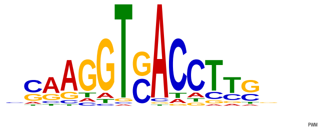	*NNNNAAGGTNACCTTNNNN*	41.63	M
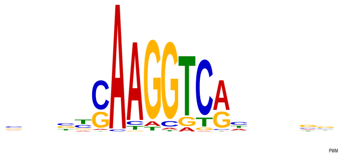	*NNNNNTCAAGGTCATNNNNN*	46.46	DR:0
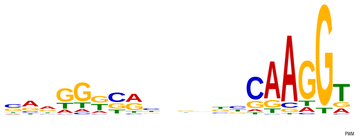	*CAAGGNNNNNNNNNCAAGGT*	101.86	DR:8
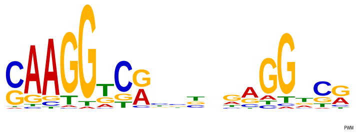	*CAAGGTCNNNNNNNGGTCG*	144.01	DR:5
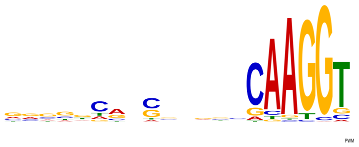	*GGGGTNNNNNNNNNCAAGGT*	68.41	DR:9
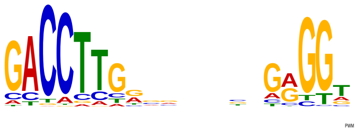	*GACCTTNNNNNNNNNGGGGT*	69.38	ER:11
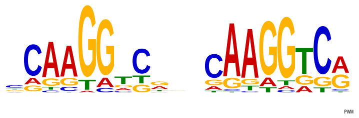	*NCAAGGNNNNNCAAGGTCN*	113.05	DR:4

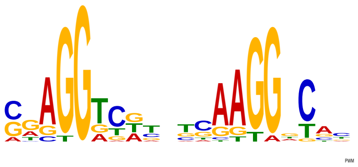	*NNAGGTCNNNNCAAGGGCNN*	150.40	DR:5
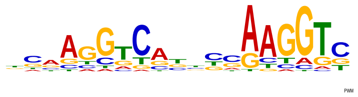	*NCAAGGNNNNNNNCAAGGTN*	31.42	DR:5
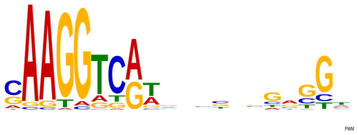	*CAAGGTCNNNNNNNNGGGGT*	99.08	DR:8
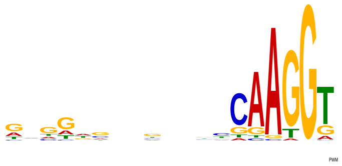	*AGAGGNNNNNNNNNCAAGGT*	67.30	DR:8
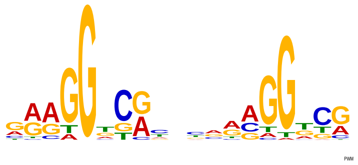	*NGAGGTCNNNNNGAGGTCN*	83.12	DR:5
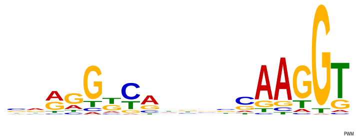	*CAAGGNNNNNNNCAAGGT*	44.51	DR:6
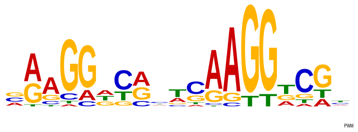	*NNAGGGCNNNCAAGGTCNN*	67.89	DR:4

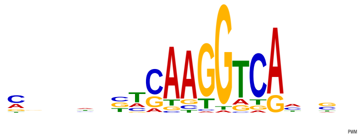	*NNNNNGTTCAAGGTCNNNNN*	15.94	DR:0
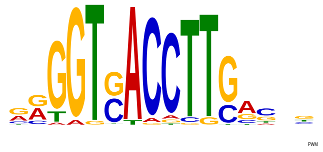	*NNNGGGTCACCTTGANNN*	98.73	IR:3
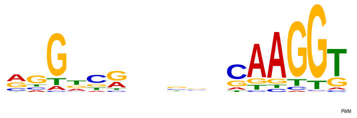	*AGGTCNNNNNNCAAGGT*	36.52	DR:7
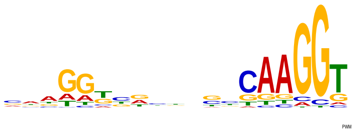	*CAAGGNNNNNNNNCAAGGT*	41.75	DR:7
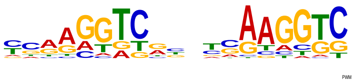	*NGAAGGNNNNNNCAAGGTN*	15.43	DR:5
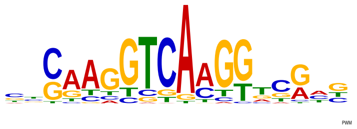	*NNNAAGGTCAAGGTCNN*	53.62	DR:0

8 ESRRG:RXRA Round 3

PWM	Seed Sequence	Seed Seq Enrichment	Repeat
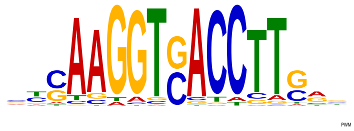	*NNCAAGGTNACCTTGNN*	365.41	M
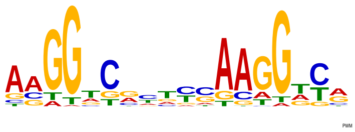	*NAGGTCNNNNCAAGGTCN*	277.83	DR:5
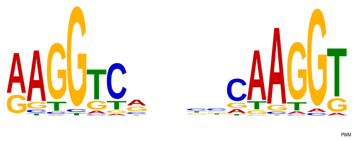	*AAGGTCNNNNNCAAGGT*	280.68	DR:6
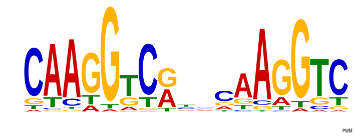	*NCAAGGTCNNNNAAGGTN*	192.73	DR:4
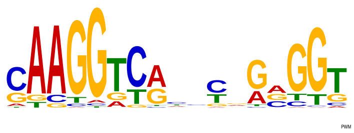	*CAAGGTCNNNNNGGGGT*	173.28	DR:5
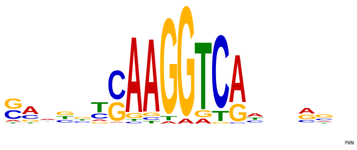	*NNNNNTCAAGGTCATNNNNN*	44.81	DR:0
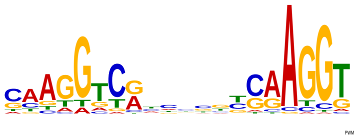	*CAAGGNNNNNNNNNNAAGGT*	43.48	DR:8

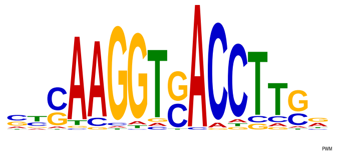	NNTCAAGGTNACCTTNN	315.61	DR:0
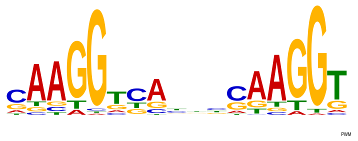	CAAGGTNNNNNCAAGGT	308.52	DR:5
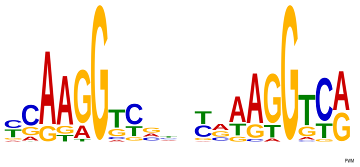	NCAAGGTCNNNNNNAGGTCN	372.57	DR:5
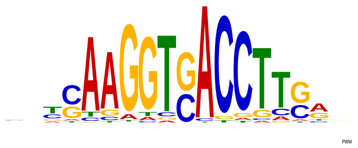	NNNCAAGGTNACCTTNNN	141.22	M
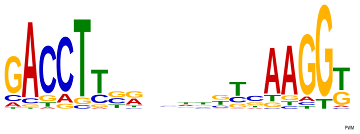	NACCTTNNNNNNNNCAAGGN	56.01	ER:11
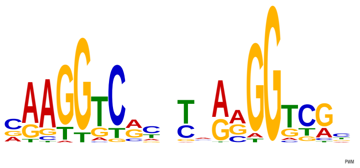	NNAGGTCNNNNNGAGGTCNN	103.61	DR:5
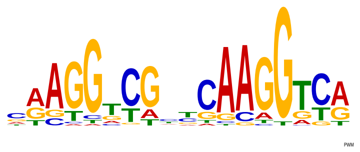	NNAGGTCNNTCAAGGTNN	212.79	DR:4

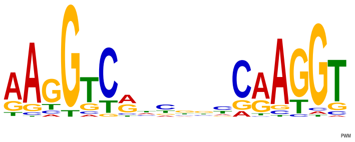	*AAGGTCNNNNNNNAAGGT*	68.53	DR:7
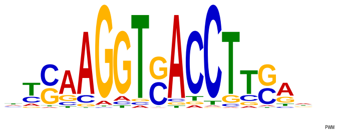	*NNNNAAGGTNACCTTNNNN*	50.64	M
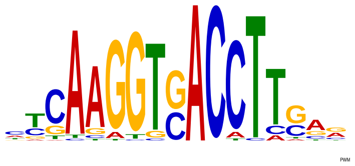	*NNCAAGGTNACCTTCNN*	222.07	M
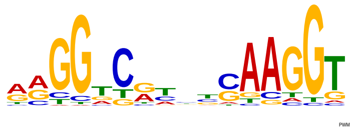	*GAGGTCNNNNCAAGGT*	193.09	DR:5
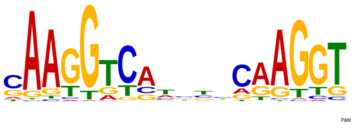	*CAAGGTNNNNNNNAAGGT*	83.18	DR:6
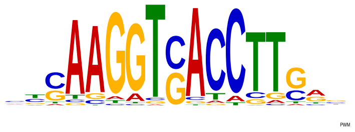	*NNCAAGGTNNCCTTGNN*	104.08	M

9 ESRRG+4 Round 3

PWM	Seed Sequence	Seed Seq Enrichment	Repeat
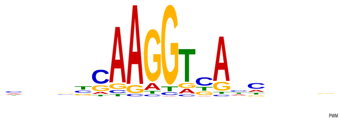	*NNNNNCCAAGGTCACNNNNN*	28.14	M
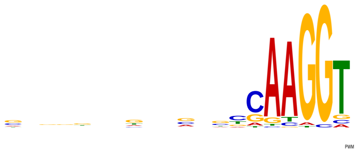	*GGGGGNNNNNNNNNCAAGGT*	63.21	M
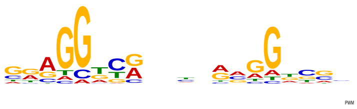	*NNAGGTCNNNNNNAGGTCNN*	18.60	DR:5
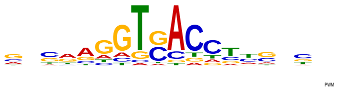	*NNNNAAGGTNACCTTNNNN*	21.20	M
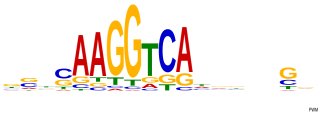	*NNNNNCAAGGTCAAGNNNNN*	21.94	M
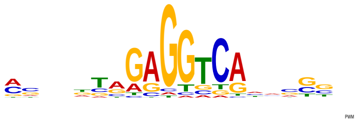	*NNNNNTAGAGGTCAANNNNN*	16.99	M
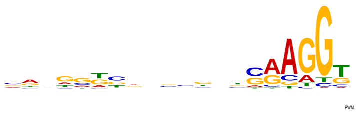	*CAAGGNNNNNNNNNNAAGGT*	21.33	DR:8

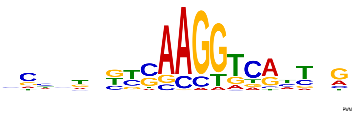	*NNNNNGTTCAAGGTCNNNNN*	11.87	DR:0
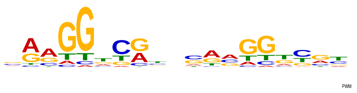	*NNAGGTCNNNNNAGGTCNN*	10.70	DR:4
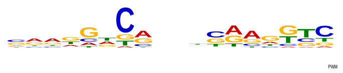	*NCAAGGNNNNNNNAAGGTN*	6.92	DR:5
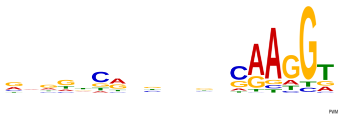	*AGAGGNNNNNNNNNNAAGGT*	18.73	DR:8
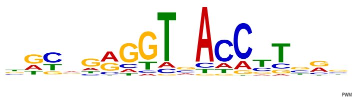	*NNNAGAGGTNACCTTNNN*	20.35	M
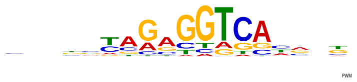	*NNNNCCAGAGGTCANNNN*	8.39	M
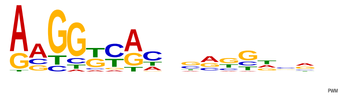	*NNAGGTCNNNGGGGTCNN*	8.00	DR:3

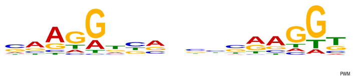	CAAGGTNNNNNNAAGGT	24.90	DR:5
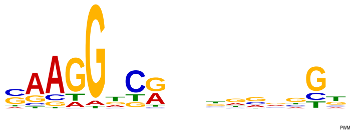	CAAGGTCNNNNNGGGGT	43.75	DR:5
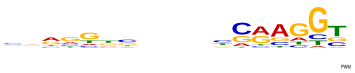	CAAGGNNNNNNNNAAGGT	10.18	DR:6
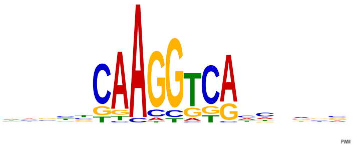	NNNNCCAAGGTCACANNNN	25.73	M
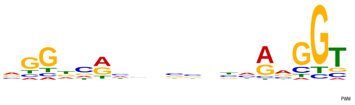	AGGTCNNNNNNNNNGAGGT	7.96	DR:9
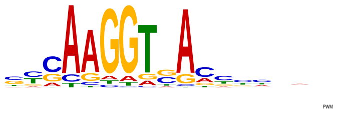	NNNCAAGGTNACCCCNNN	32.21	M

10 ESRRB Round 3

PWM	Seed Sequence	Seed Seq Enrichment	Repeat
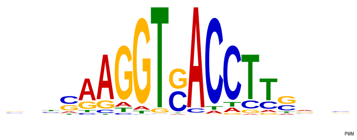	*NNNNAAGGTNACCTTNNNN*	416.57	M
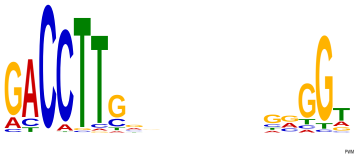	*GACCTTGNNNNNNNNGGGGT*	1527.29	ER:11
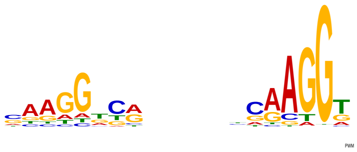	*CAAGGNNNNNNNNNNNAAGGT*	309.82	DR:8
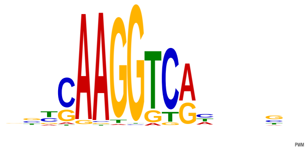	*NNNNNTCAAGGTCATNNNNN*	355.99	DR:0
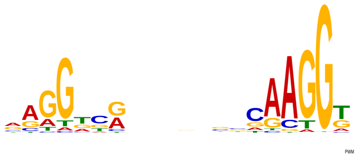	*AAGGTCNNNNNNNNNCAAGGT*	1012.71	DR:9
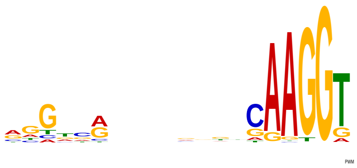	*AGGTCNNNNNNNNNNCAAGGT*	414.90	DR:10
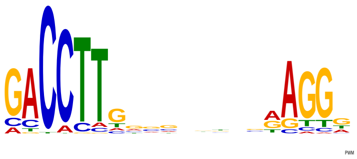	*GACCTTGGNNNNNNNNAAGGG*	2159.31	ER:11

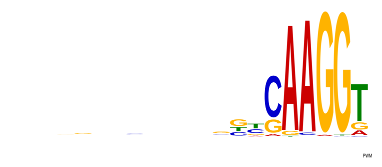	*GGGGGNNNNNNNNNTCAAGGT*	866.18	DR:0
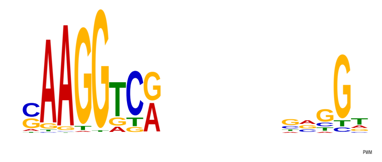	*CAAGGTCNNNNNNNNGGGGT*	847.10	DR:8
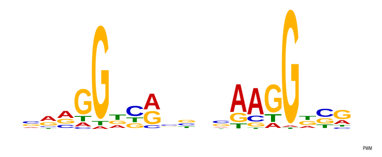	*NNAGGTCNNNNCAAGGTCNN*	628.39	DR:5
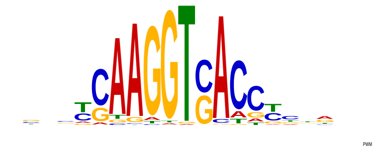	*NNNNCAAGGTNACCCCNNNN*	383.73	M
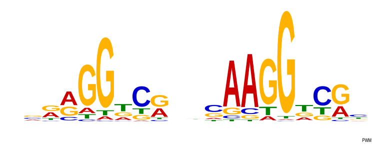	*NNAGGTCNNNCAAGGTCNN*	557.37	DR:4
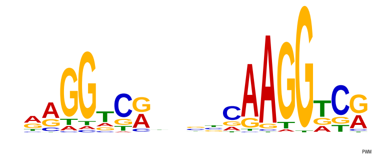	*NAGGTCNNNNNCAAGGTCN*	588.31	DR:6
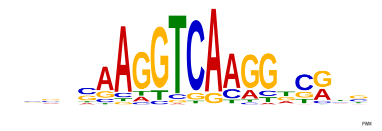	*NNNCAAGGTCAAGGTCNNN*	1330.29	DR:0

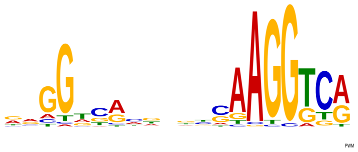	$N\overrightarrow{AGGTCN}NNNNNCA\overrightarrow{AGGTCN}$	383.37	DR:7
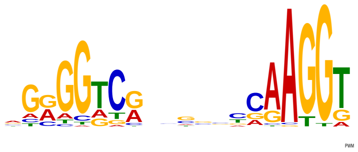	$AG\overrightarrow{GGGTCG}NNNNNNNA\overrightarrow{AGGT}$	1145.05	DR:8
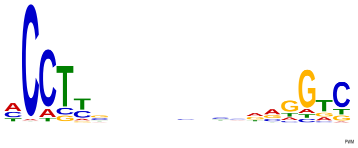	$\overleftarrow{ACCT}TNNNNNNNNNN\overrightarrow{AGGTC}$	272.57	ER:11
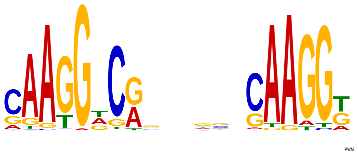	$CA\overrightarrow{AGGACN}NNNNNNCA\overrightarrow{AGGG}$	1502.97	DR:8
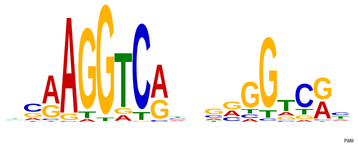	$NNA\overrightarrow{AGGTCN}NNG\overrightarrow{GGGTCN}N$	297.96	DR:3
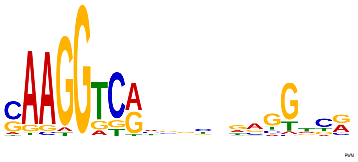	$CA\overrightarrow{AGGTCN}NNNNNN\overrightarrow{NGGTCG}$	526.65	DR:6

11 ESRRB:RXRA Round 3

PWM	Seed Sequence	Seed Seq Enrichment	Repeat
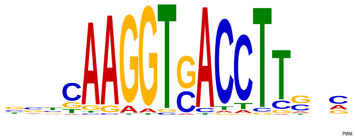	NNNCAAGGTNACCTTNNN	91.39	M
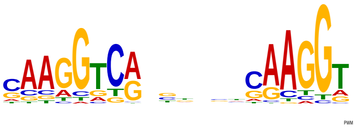	CAAGGNNNNNNNNNNAAGGT	48.34	DR:8
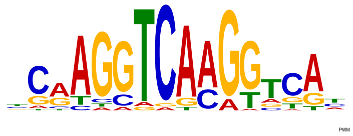	NNAAGGTCAAGGTCNN	60.76	DR:0
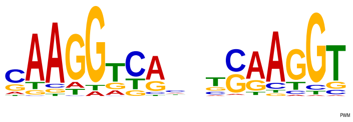	CAAGGTNNNNNNAAGGT	55.76	DR:5
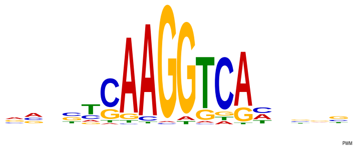	NNNNTCAAGGTCAANNNN	29.82	DR:0
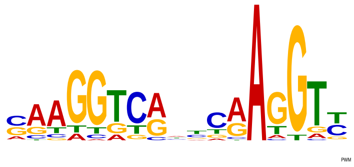	NAAGGTCNNNNAAGGTN	26.37	DR:4
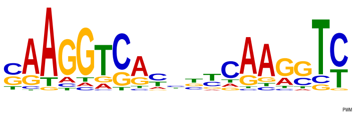	NAAGGTNNNNNNNAAGGTN	13.55	DR:6

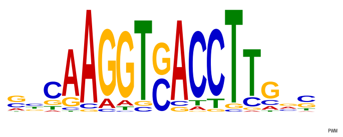	$NNNNA\overrightarrow{AGGTNA}CCTTNNNN$	30.18	M
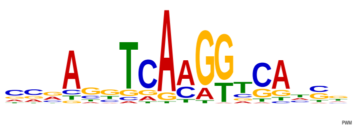	$NNN\overrightarrow{NGGTCA}\overrightarrow{AGGTCN}NNNN$	13.17	DR:0
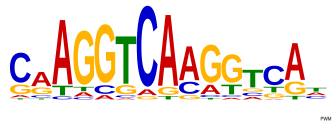	$NNA\overrightarrow{AGGTCN}\overrightarrow{AGGTCN}N$	22.17	DR:0
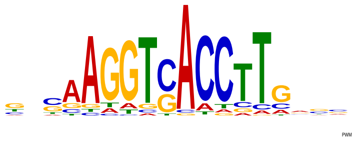	$NNNA\overrightarrow{AGGTNN}CCTTGNNN$	26.17	M
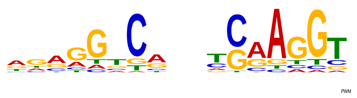	$AGA\overrightarrow{GGNNNN}NNNNA\overrightarrow{AGGT}$	10.28	DR:5
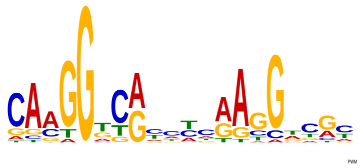	$NN\overrightarrow{AGGTCN}NNNNN\overrightarrow{AGGTCN}N$	17.65	DR:5
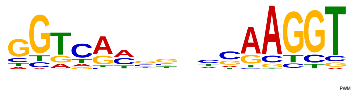	$\overrightarrow{GGTCA}NNNNNNNA\overrightarrow{AGGT}$	9.96	DR:7

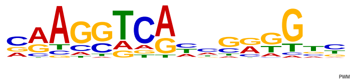	$NA\overrightarrow{AGGTCN}NNG\overrightarrow{GGGTN}$	11.56	DR:3
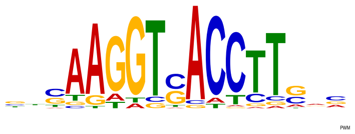	$NNCA\overrightarrow{AGGTNN}CCTTGNN$	49.21	M
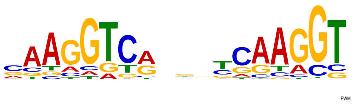	$CA\overrightarrow{AGGNNN}NNNNNA\overrightarrow{AGGT}$	17.42	DR:6
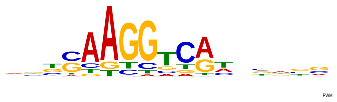	$NNNNCA\overrightarrow{AGGTCA}TTNNNN$	11.39	M
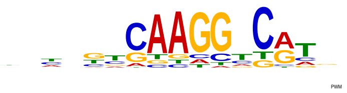	$NN\overrightarrow{NGTTCA}\overrightarrow{AGGTCN}NN$	10.17	DR:0
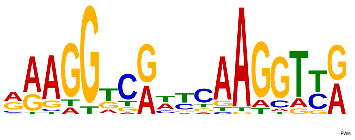	$NN\overrightarrow{AGGTCN}NNCA\overrightarrow{AGGTNN}$	35.08	DR:4

12 ESRRB+4 Round 3

PWM	Seed Sequence	Seed Seq Enrichment	Repeat
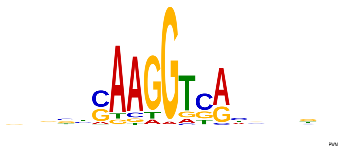	NNNNNTCAAGGTCACNNNNN	67.48	DR:0
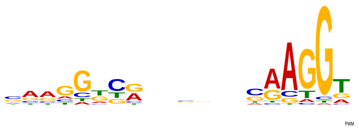	CAAGGNNNNNNNNNNAAGGT	52.51	DR:8
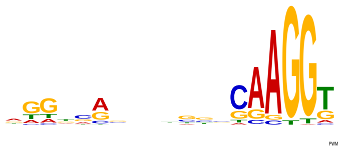	AAGGTNNNNNNNNNCAAGGT	93.33	DR:9
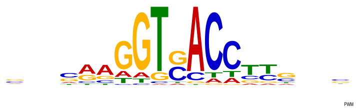	NNNNAAGGTNACCTTNNNN	44.65	M
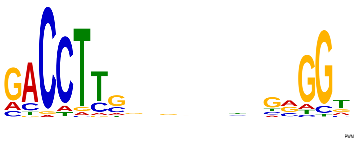	GACCTTNNNNNNNNNGGGGT	99.20	ER:11
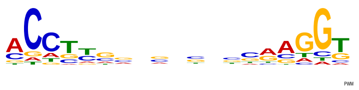	ACCTTNNNNNNNNNNAAGGT	37.57	ER:11
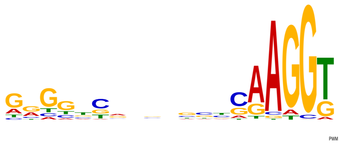	AGGGGTNNNNNNNNNAAGGT	60.96	DR:10

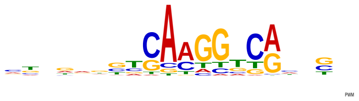	$NNNN\overrightarrow{NGGTCA}\overrightarrow{AGGTCN}NNNN$	31.71	DR:0
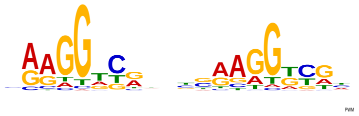	$NN\overrightarrow{AGGTCN}NNNNA\overrightarrow{AGGTCN}N$	60.84	DR:5
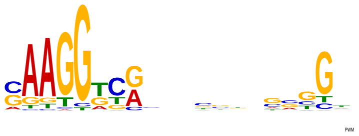	$CA\overrightarrow{AGGTCN}NNNNNNNNG\overrightarrow{GGGT}$	112.07	DR:8
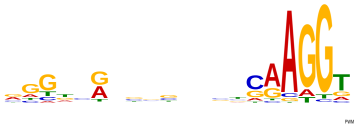	$\overrightarrow{AGGTCN}NNNNNNNNNA\overrightarrow{AGGT}$	28.26	DR:10
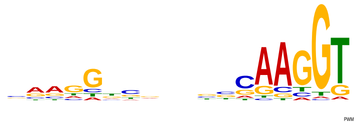	$CA\overrightarrow{AGGNNN}NNNNCA\overrightarrow{AGGT}$	51.03	DR:6
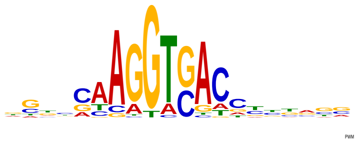	$NNNNCA\overrightarrow{AGGTNA}CCCCNNNN$	51.84	M
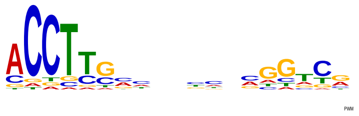	$\overleftarrow{ACCTT}NNNNNNNNN\overrightarrow{NGGTCG}$	20.44	ER:9

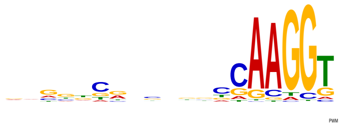	*GGAGGNNNNNNNNNCAAGGT*	102.40	DR:8
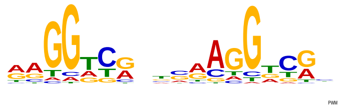	*NNAGGTCNNNNAAGGTCNN*	36.57	DR:4
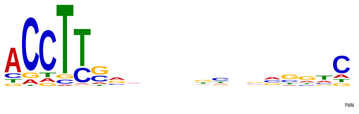	*ACCTTNNNNNNNNNNCGGTC*	34.77	ER:11
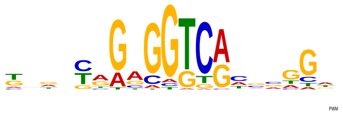	*NNNNNTAGAGGTCATNNNNN*	19.34	M
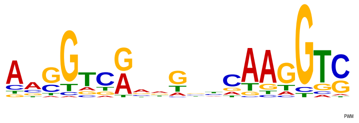	*NAGGTCNNNNNNCAAGGTN*	25.69	DR:7
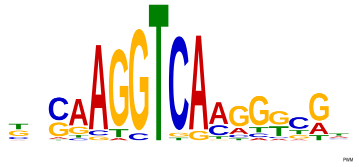	*NNCAAGGTCAAGGGCNN*	207.57	DR:0

13 ESRRA Round 3

PWM	Seed Sequence	Seed Seq Enrichment	Repeat
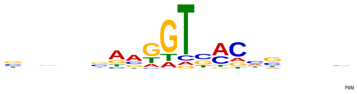	*NNNNNCAAGGTGACCNNNNN*	74.40	M
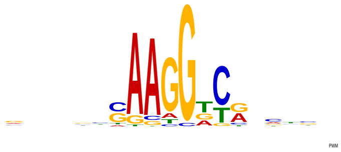	*NNNNNCTCAAGGTCGNNNNN*	49.49	DR:0
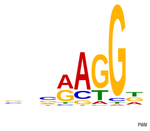	*GGGGGNNNNNNNNNNAAGGT*	56.29	M
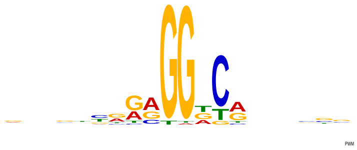	*NNNNNTAGAGGTCGTNNNNN*	35.75	M
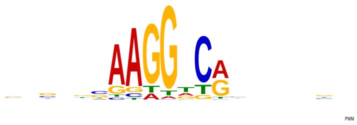	*NNNNNCAAGGTCGTGNNNNN*	55.67	M
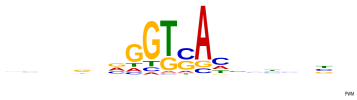	*NNNNNGAGGTCACCTNNNNN*	52.07	M
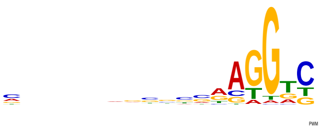	*CCCCCNNNNNNNNNNAAGGTC*	71.78	M

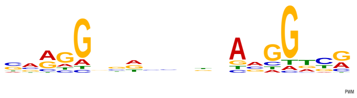	CAAGGNNNNNNNNNNAGGTCG	70.57	DR:6
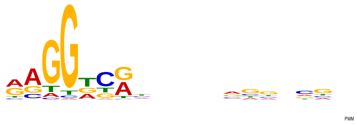	NAGGTCNNNNNNNGGACGN	37.81	DR:5
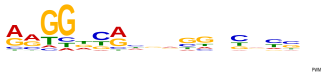	NNNAGGTCNNNNGGTCGNNN	19.97	DR:2
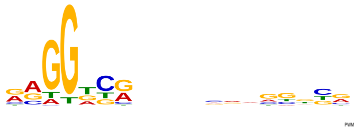	NAGGTCNNNNNNNAGGGCN	30.47	DR:6
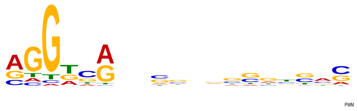	AGGTCNNNNNNNNNGTGAC	16.94	IR:9
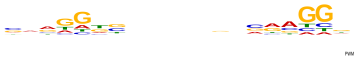	CAAGGNNNNNNNNNNNAAGGG	50.95	DR:8
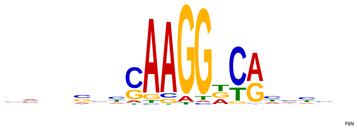	NNNNNCTCAAGGGCANNNNN	41.78	DR:0

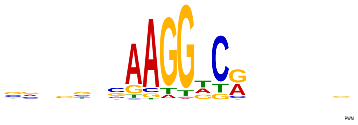	NNNNNCCAAGGTCGTNNNNN	66.20	M
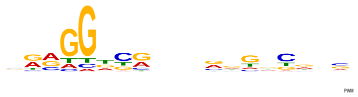	NNAGGTCNNNNNGGTCGNN	31.57	DR:3
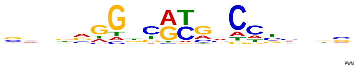	NNNNNGGTCATGACCNNNNN	40.86	IR:0
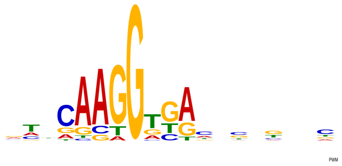	NNNNCAAGGTNACACGNNNN	64.04	M
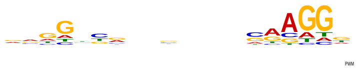	AAGGTNNNNNNNNNNAAGGG	36.90	DR:9
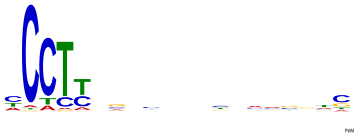	CCCTTNNNNNNNNNNCGGTC	41.40	ER:11

14 ESRRA:RXRA Round 3

PWM	Seed Sequence	Seed Seq Enrichment	Repeat
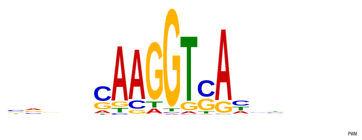	*NNNNNCAAGGTCACCNNNNNN*	487.45	M
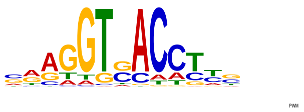	*NNNNAAGGTNACCTTNNNN*	396.87	M
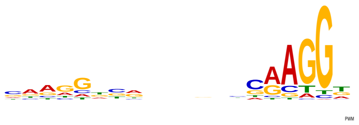	*CAAGGNNNNNNNNNNAAGGT*	314.39	DR:8
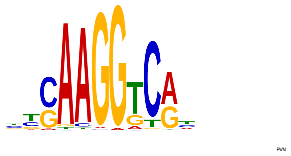	*NNNNNTCAAGGTCGTNNNNN*	299.07	DR:0
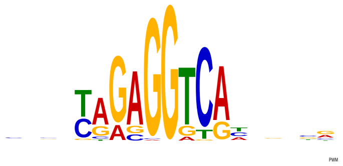	*NNNNNTAGAGGTCATNNNNN*	268.60	M
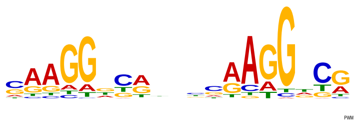	*CAAGGNNNNNNNAAGGTCG*	819.28	DR:5
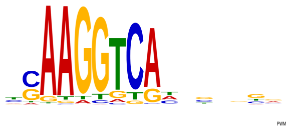	*NNNNNCAAGGTCAAGNNNNNN*	305.52	M

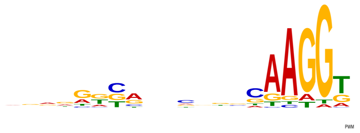	AGAGGNNNNNNNNNCAAGGT	500.89	DR:8
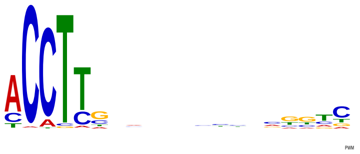	ACCTTNNNNNNNNNNCGGTC	213.37	ER:11
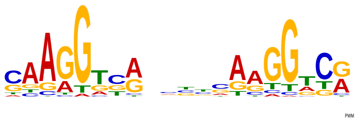	CAAGGNNNNNNNNAGGTCG	331.67	DR:6
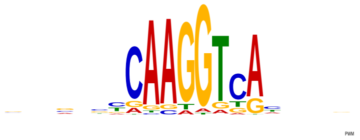	NNNNNCCCAAGGTCANNNNN	391.92	M
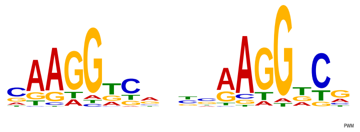	CAAGGNNNNNNAAGGTCG	595.38	DR:4
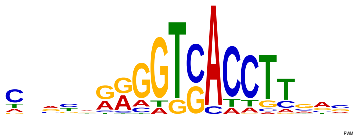	NNNCAGAGGTNACCTTNNN	674.49	M
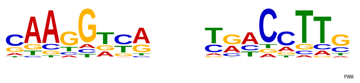	CAAGGNNNNNNNNNCCTTG	160.71	IR:3

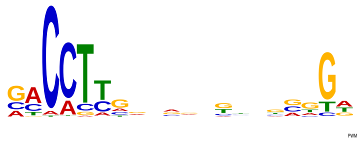	$\overleftarrow{GACCT}TNNNNNNNNNG\overrightarrow{GGGT}$	358.76	ER:11
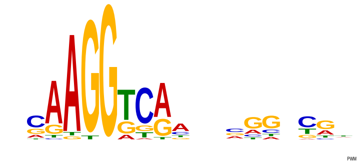	$NCA\overrightarrow{AGGTCN}NNNN\overrightarrow{NGGTCG}N$	403.25	DR:3
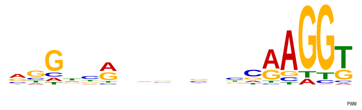	$\overrightarrow{AGGTCN}NNNNNNNNNA\overrightarrow{AGGT}$	117.43	DR:9
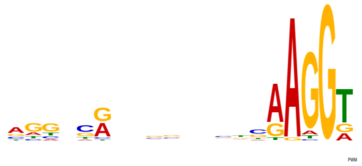	$\overrightarrow{AGGTCN}NNNNNNNNNNA\overrightarrow{AGGT}$	144.18	DR:10
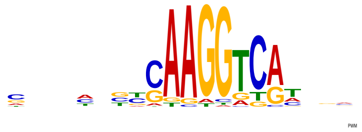	$NNNN\overrightarrow{NGCTCA}\overrightarrow{AGGTCN}NNNN$	160.37	DR:0
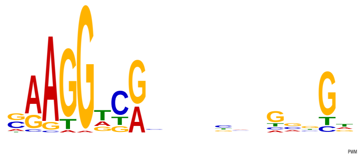	$GA\overrightarrow{AGGTCN}NNNNNNNNG\overrightarrow{GGGT}$	476.66	DR:8

15 ESRRA+4 Round 3

PWM	Seed Sequence	Seed Seq Enrichment	Repeat
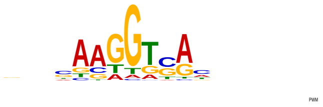	*NNNNNCAAGGTCACCNNNNN*	92.24	M
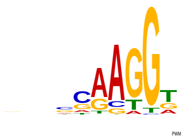	*GGGGGNNNNNNNNNCAAGGT*	119.46	M
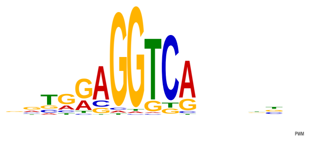	*NNNNNTGGAGGTCAANNNNN*	68.10	M
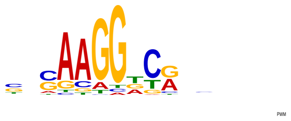	*NNNNNTCAAGGTCGTNNNNN*	52.17	DR:0
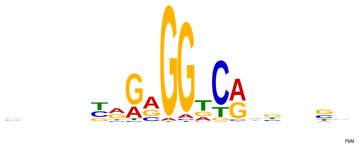	*NNNNNTAGAGGTCGTNNNNN*	34.10	M
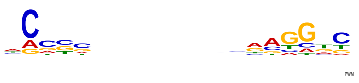	*ACCCCNNNNNNNNNNAGGTC*	33.28	ER:11
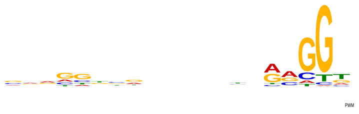	*GAAGGNNNNNNNNNNGAGGT*	36.02	DR:8

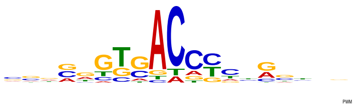	*NNNNGGTCACCTCTANNNN*	43.86	M
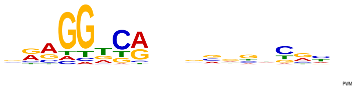	*NNAGGTCNNNNNGGTCGNN*	26.07	DR:3
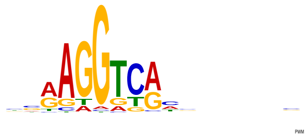	*NNNNNAAGGTCACGGNNNNN*	48.69	M
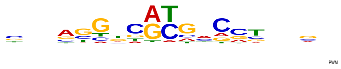	*NNNNNGGTCATGACCNNNNN*	33.06	IR:0
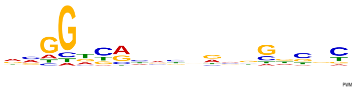	*NNGGTCACNNNNAGGTCNN*	39.34	DR:5
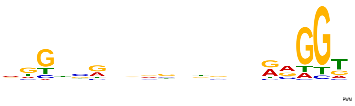	*AGGTCNNNNNNNNNNGAGGT*	28.90	DR:10
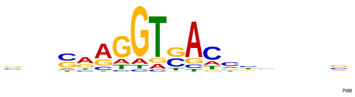	*NNNNAAGGTNACCCCNNNN*	48.28	M

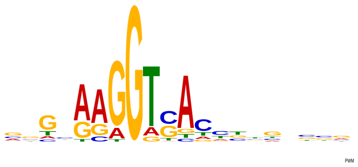	*NNNNAAGGTNACCGTGNNNN*	52.24	M
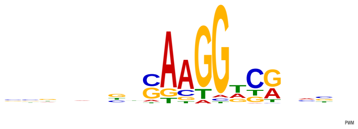	*NNNNNGTTCAAGGTCNNNNN*	27.08	DR:0
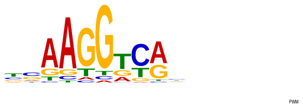	*NNNNCAAGGNCACGGNNNN*	30.85	M
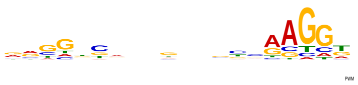	*GAGGTNNNNNNNNNNAAGGG*	23.59	DR:9
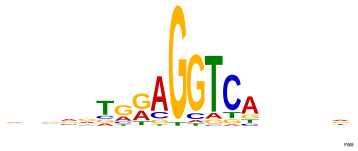	*NNNNCTGGANGTCAGNNNN*	12.87	M
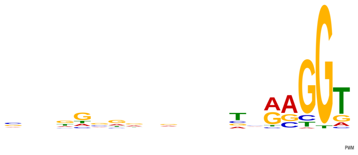	*CCTGGNNNNNNNNNNGAGGT*	24.54	M

16 ESR1+5 Round 3

PWM	Seed Sequence	Seed Seq Enrichment	Repeat
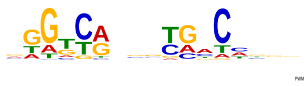	NNNNGGTCANNGTGACNNNN	604.83	IR:3
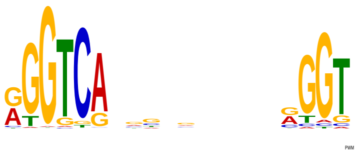	AGGTCANNNNNNNNNGAGGT	540.86	DR:10
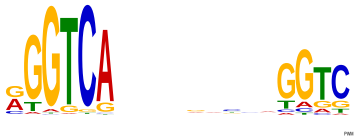	AGGTCANNNNNNNNAGGTC	123.74	DR:8
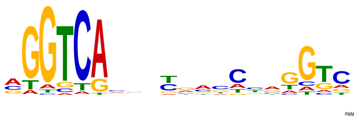	AGGTCANNNNNNNNNAGGTC	99.12	DR:9
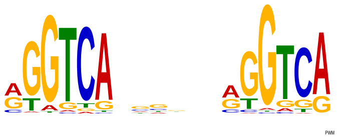	NAGGTCANNNNNNAGGTCN	164.11	DR:6
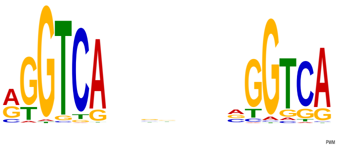	NAGGTCANNNNNNNAGGTCN	152.93	DR:7
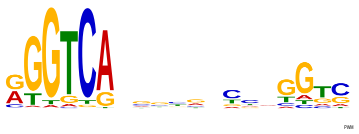	AGGTCANNNNNNNNGGGTC	84.70	DR:8

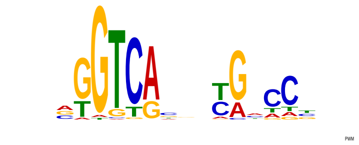	$NNN\overrightarrow{AGGTCA}NNN\overleftarrow{CGCCC}NNN$	626.74	IR:3
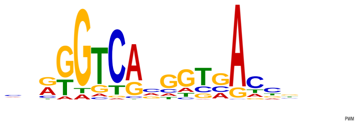	$NNN\overrightarrow{NGTCA}\overrightarrow{CGGTCA}CCNNNN$	537.82	DR:0
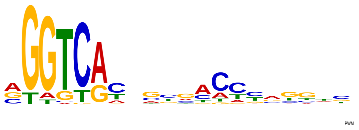	$\overrightarrow{AGGTCA}NNNNNNNNN\overrightarrow{GGGTC}$	64.20	DR:9
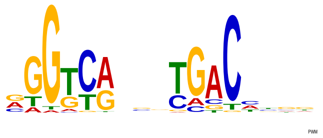	$NN\overrightarrow{NGGTCA}GGN\overleftarrow{TGACA}NNN$	755.36	IR:3
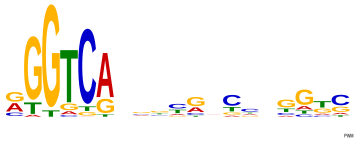	$\overrightarrow{AGGTCA}NNNNNNNC\overrightarrow{GGGTC}$	153.24	DR:8
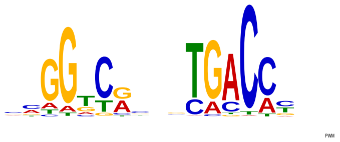	$NN\overrightarrow{CGGTCG}NNN\overleftarrow{TGACC}CNN$	1161.44	IR:3
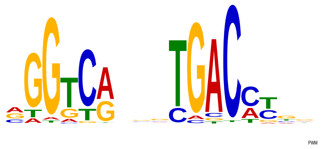	$NN\overrightarrow{NGGTCG}GNN\overleftarrow{TGACA}NNN$	354.21	IR:3

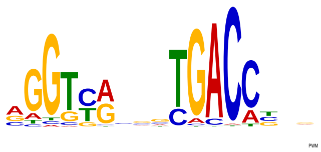	NNAGGTTGNNNTGACCCNN	693.50	IR:3
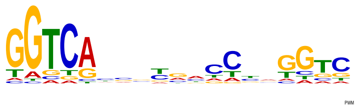	GGTCANNNNNNNNNAGGTC	46.82	DR:9
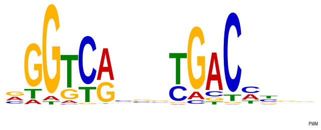	NNNGGTCAGNNTGACANNN	539.98	IR:3
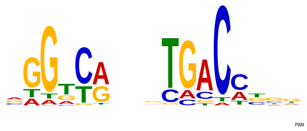	NNNNGGTCGGNGTGACNNNN	467.27	IR:3
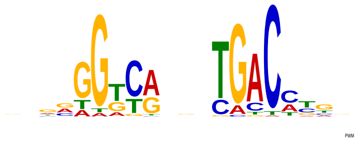	NNNAGGTCGGNNTGACANNN	448.36	IR:3
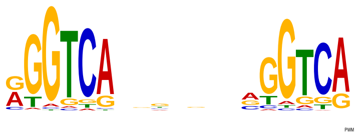	NGGTCANNNNNNNAGGTCN	123.38	DR:7

17 ESR1+6 Round 3

PWM	Seed Sequence	Seed Seq Enrichment	Repeat
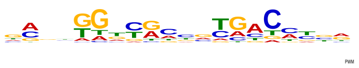	NNNNGGTCANNGTGACNNNN	6.34	IR:3
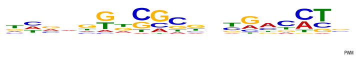	NNNGGGTCNNNGTGACNNN	4.54	IR:3
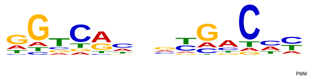	NNGGTCACNGTGACNN	13.11	IR:3
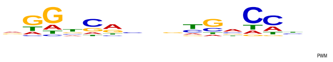	NNNGTCACNGTGACNNN	5.53	IR:3
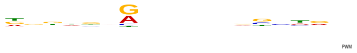	NGGTGANNNNNNGGCTGN	2.29	M
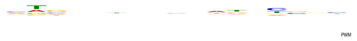	NNGAGTGNNNATGCGNN	2.11	M
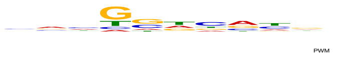	CACGGTCATG	2.57	M

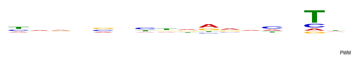	NNAAGACNNAAACGNN	2.08	M
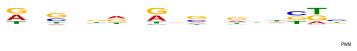	NNNCGAGGNGTGTTNNN	1.93	M
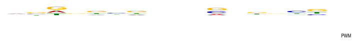	NGGGGCNNNNNAGGCGN	2.28	DR:4
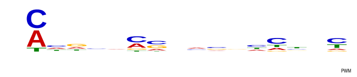	NNNGCACCNACGCCNNN	2.47	ER:0
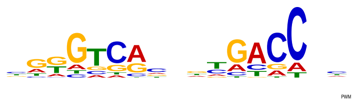	NNTGTCACNNTGACCNN	9.47	IR:3
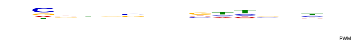	NNATGCANGTTGANN	2.04	M
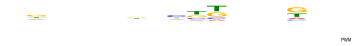	NGCGGANNNNNGGTGTN	1.68	M

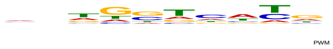	*ATTGGTCATG*	2.45	M
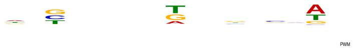	*NNNGGGAANATGACNNN*	1.51	IR:2
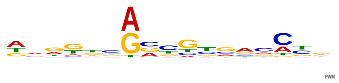	*NNGGGTCNNNNTGACANN*	3.48	IR:3
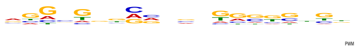	*NGGCGGNNNNNNNGGGTGN*	3.56	M
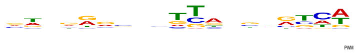	*NNGGGGCNNNNNTGTGTNN*	2.42	M
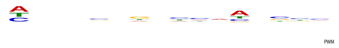	*NNACACANNNCAATCNN*	1.96	M

18 THRB Round 3

PWM	Seed Sequence	Seed Seq Enrichment	Repeat
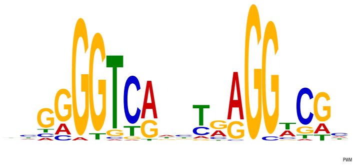	NNGGGGTCNNNTGAGGTCNN	846.65	DR:4
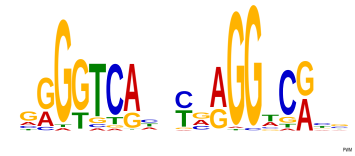	NNNGGTCACNNNAGGTCNNN	166.87	DR:4
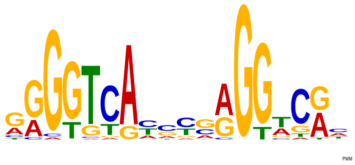	NNGGTCATNCGGGGTCNN	380.62	DR:4
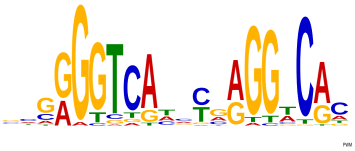	NNGGGGTCNNNNNGGGACNN	58.43	DR:4
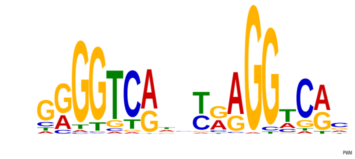	NNNGGGTCNNNTGAGGTNNN	126.24	DR:4
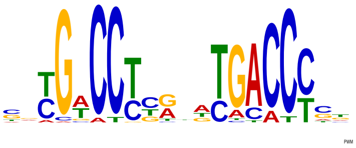	NNNGTCCCCANNTGACCNNN	136.09	IR:5
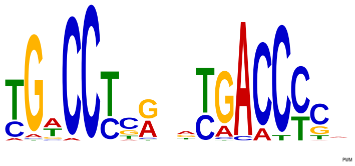	NGTCCCCANNNGACCCCN	353.31	IR:5

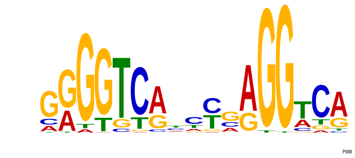	$NNG\overrightarrow{GGGTCN}NNT\overrightarrow{GGGGTN}N$	140.87	DR:4
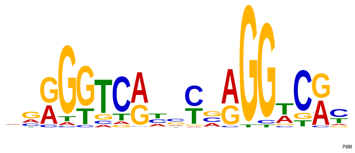	$NN\overrightarrow{AGGTCA}CNNN\overrightarrow{AGGTCN}N$	176.24	DR:4
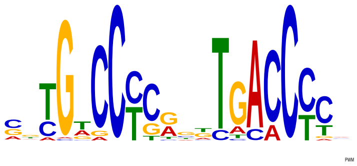	$N\overrightarrow{NNGTCC}CCCGNN\overleftarrow{TGACCN}NN$	173.03	IR:5
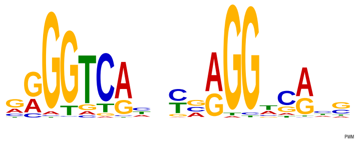	$N\overrightarrow{NGGTCA}CNNN\overrightarrow{NGGTCA}NN$	140.35	DR:4
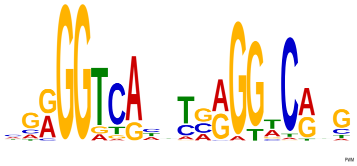	$NN\overrightarrow{NGGTCA}TNNN\overrightarrow{GGGAC}NNN$	67.01	DR:4
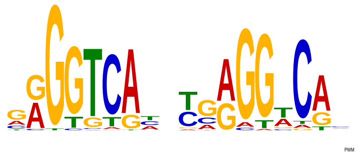	$N\overrightarrow{NGGTCA}TN\overrightarrow{NGGGGA}CNN$	131.50	DR:2
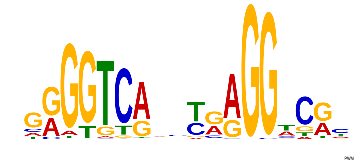	$NN\overrightarrow{GGGTCN}NNTG\overrightarrow{AGGTCN}N$	361.03	DR:4

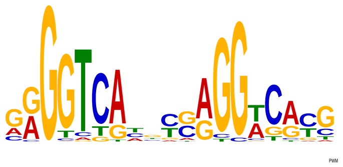	$NN\overrightarrow{NGGTCA}CNNN\overrightarrow{GGGTCN}NN$	83.03	DR:4
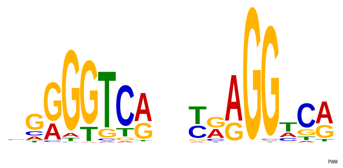	$NNG\overrightarrow{GGGTCN}NNTG\overrightarrow{AGGTNN}$	340.12	DR:4
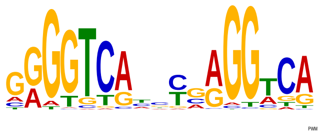	$NNG\overrightarrow{GGGTCN}NNNG\overrightarrow{GGGTNN}$	76.27	DR:4
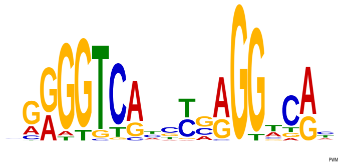	$NNG\overrightarrow{GGGTCN}NNNG\overrightarrow{GGGTCN}N$	151.25	DR:4
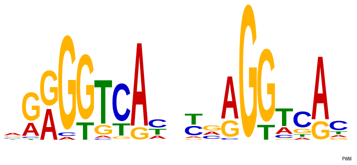	$NN\overrightarrow{NGGTCA}TNCG\overrightarrow{GGGTNN}N$	103.75	DR:4
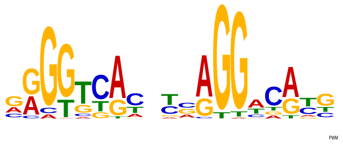	$NN\overrightarrow{NGGTCA}NNCG\overrightarrow{GGGTCN}NN$	113.56	DR:4

19 THRB:RXRA Round 3

PWM	Seed Sequence	Seed Seq Enrichment	Repeat
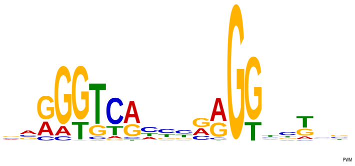	NNNGGTCACNNNAGGTCNNN	420.50	DR:4
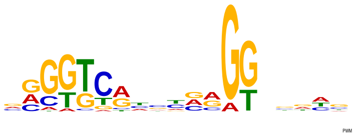	NNGGTCACNNNNGGTCANN	213.32	DR:4
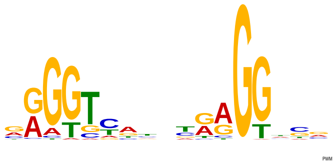	NGGGGTNNNNTGAGGTCN	474.38	DR:4
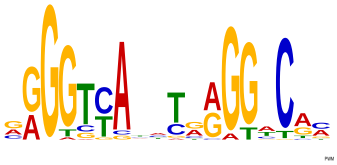	NNGGGTTANNNNGGGACNN	149.90	DR:4
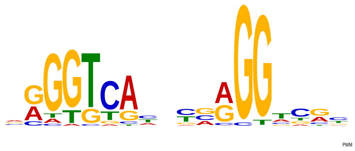	NNGGTCACNNNGGGTCNN	266.28	DR:4
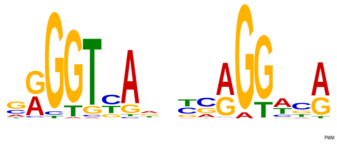	NGGGGTTANNNNGGGTCN	296.52	DR:4
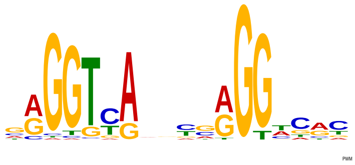	NNGGTTANNNGGGGTCNN	136.33	DR:4

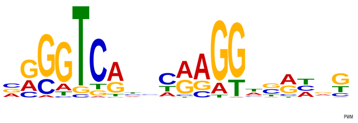	$NN\overrightarrow{NGTCA}CNNNN\overrightarrow{NGGTCA}NNN$	80.78	DR:4
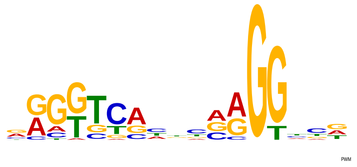	$N\overrightarrow{AGGTCA}CNNN\overrightarrow{AGGTCN}$	526.56	DR:4
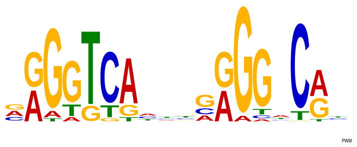	$NN\overrightarrow{GGTCA}AN\overrightarrow{NGGGGA}CNN$	374.35	DR:2
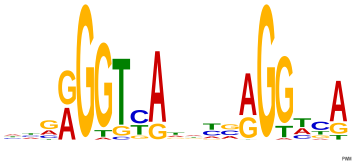	$NNG\overrightarrow{GGGTTA}NNNG\overrightarrow{GGGTN}N$	258.62	DR:4
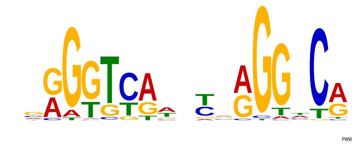	$NG\overrightarrow{GGGTCN}NNNN\overrightarrow{GGGACN}$	239.45	DR:4
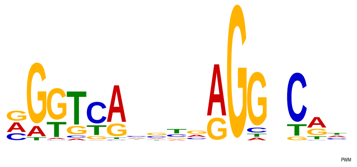	$\overrightarrow{NGGTCA}CNNN\overrightarrow{NGGACA}N$	217.82	DR:4
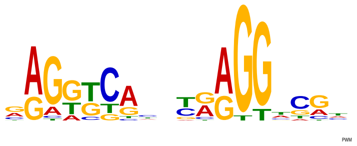	$NN\overrightarrow{GGGCA}NNT\overrightarrow{GAGGTC}NN$	351.03	DR:4

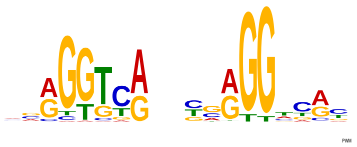	*NNAGGTCNNNNGGGGTCNN*	214.87	DR:4
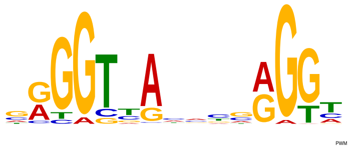	*GGGGTTANNCGGGGT*	920.33	DR:4
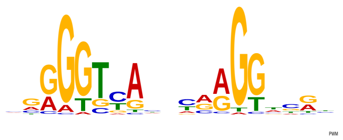	*NNGGGGTNNNNNNAGGTCNN*	118.52	DR:4
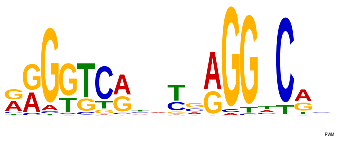	*NNAGGTCNNNNNGGGACNN*	83.11	DR:4
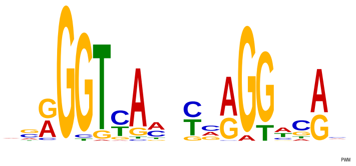	*NNGGGTTANNNNGGGTCNN*	133.46	DR:4
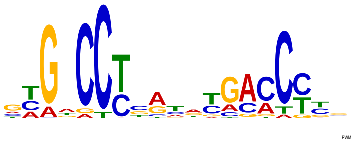	*NNGTCCCCNNNNGACCTNN*	133.24	IR:5

20 THRB:RXRA+7 Round 2

PWM	Seed Sequence	Seed Seq Enrichment	Repeat
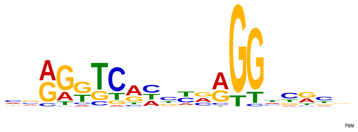	NNNGGTCANNNNAGGTCNNN	43.52	DR:4
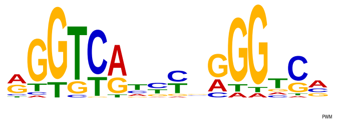	NNGGTCATNCGGGGTNN	111.68	DR:4
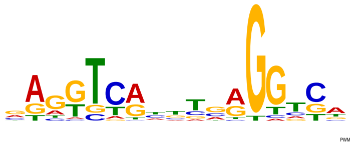	NNGGTCATNTGAGGTNN	148.61	DR:4
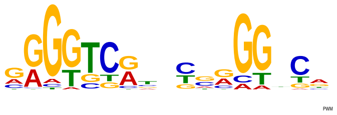	NGGGGTCNNNNNGGGGCN	31.46	DR:4
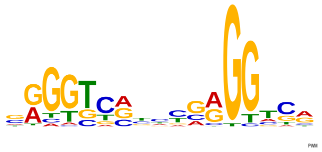	NNGGGGTCNNNNGAGGTNN	63.89	DR:4
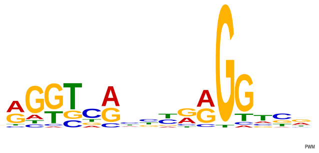	NNGGGTCNNNTGAGGTNN	66.34	DR:4
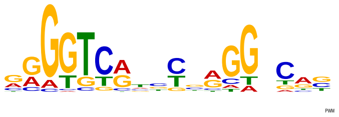	NNGGGTCNNNNNGGGGCNN	14.77	DR:4

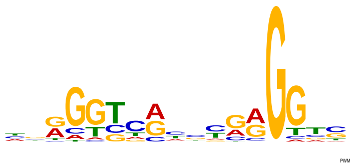	$NG\overrightarrow{GGGTCN}NNT\overrightarrow{GAGGTN}$	88.69	DR:4
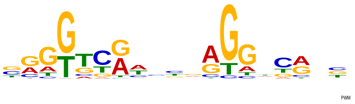	$N\overrightarrow{NGGTCA}NNNN\overrightarrow{NGGGCA}NN$	16.22	DR:4
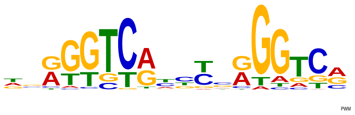	$NN\overrightarrow{NGGTCA}TNCGGGGNNN$	46.23	M
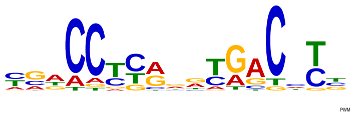	$\overleftarrow{NNNCCT}C\overrightarrow{AGATGA}CNNN$	46.70	ER:1
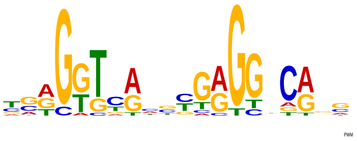	$NN\overrightarrow{NGGTCA}NNNN\overrightarrow{GGGACN}NN$	24.46	DR:4
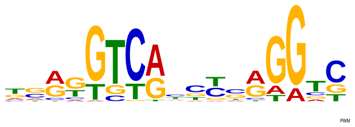	$NN\overrightarrow{GGGTCA}TNCGGGGNN$	54.56	M
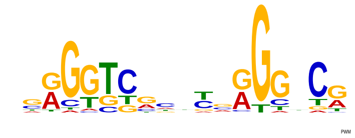	$NG\overrightarrow{GGGTCN}NNNN\overrightarrow{GGGACN}$	41.38	DR:4

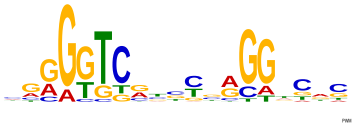	$NN\overrightarrow{GGGTCN}NNNN\overrightarrow{GGGGGCN}N$	21.26	DR:4
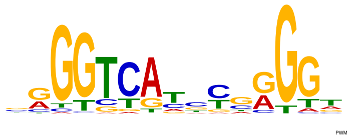	$N\overrightarrow{NGGTCA}TCCGGGNN$	83.26	M
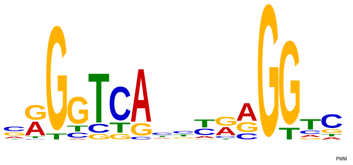	$N\overrightarrow{GGGTCA}CNNG\overrightarrow{AGGTN}$	102.52	DR:4
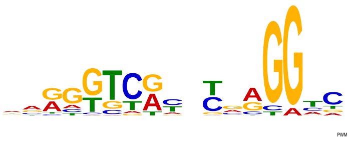	$NN\overrightarrow{AGGTCN}NCTG\overrightarrow{AGGN}N$	37.10	DR:4
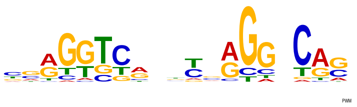	$NN\overrightarrow{GGGTCN}NNNNN\overrightarrow{GGGACN}N$	17.37	DR:4
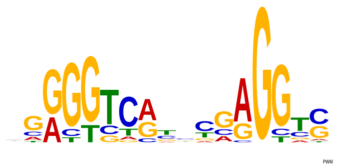	$NAG\overrightarrow{GGGTCN}NNNG\overrightarrow{AGGTN}$	70.43	DR:4

21 THRB+7 Round 3

PWM	Seed Sequence	Seed Seq Enrichment	Repeat
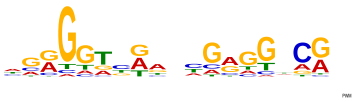	NNGGGTCNNNNNGGGACNN	6.51	DR:4
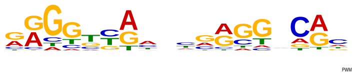	NNGGTCANNNNGGGACNN	6.65	DR:4
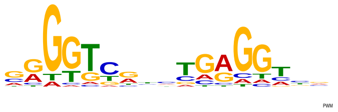	NNGGGTCNNNTGAGGTNN	25.33	DR:4
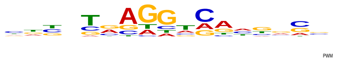	NNNNTTAAGGTCACNNNN	6.06	M
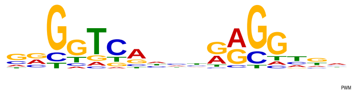	NNGGTCACNNGGGGTNN	12.01	DR:4
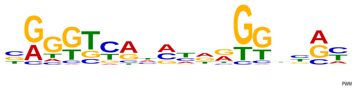	NNGGTCATNNNGGGTCNN	10.41	DR:4
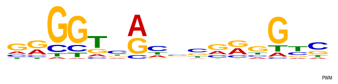	NGGGGTCNNNNCAGGTN	7.97	DR:4

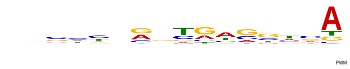	*NACCCCNNNNAGGTCN*	2.68	ER:5
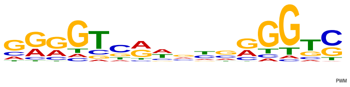	*NGGGTCNNNTCGGGTN*	7.25	DR:4
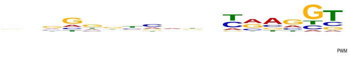	*GAGGGNNNNNNNAAGGT*	4.10	DR:6
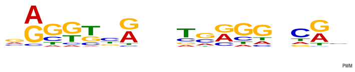	*NGGGTCNNNNNNGGACAN*	6.11	DR:4
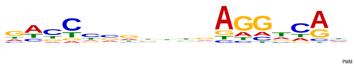	*NNCCCCGNNNNAGGTCNN*	3.35	M
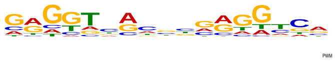	*NNGGGTCNNNNCAGGTNN*	5.04	DR:4
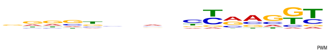	*AGGGGNNNNNNNAAGGT*	3.92	DR:7

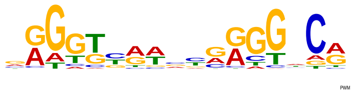	$N\overrightarrow{GGGTCA}NNNN\overrightarrow{GGGACN}$	11.90	DR:4
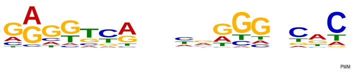	$N\overrightarrow{AGGTCN}NNNN\overrightarrow{NGGACA}N$	6.32	DR:4
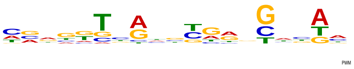	$NN\overrightarrow{GGGGCN}NNNN\overrightarrow{AGGTCN}N$	2.93	DR:4
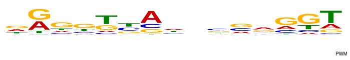	$N\overrightarrow{GGGGCN}NNCC\overrightarrow{AGGN}$	3.57	DR:4
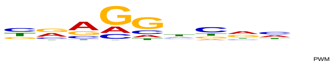	$TA\overrightarrow{AGGTCA}CA$	4.33	M
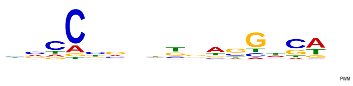	$NNCCCGGNNN\overrightarrow{AGGTCN}N$	3.29	M

22 THRA Round 3

PWM	Seed Sequence	Seed Seq Enrichment	Repeat
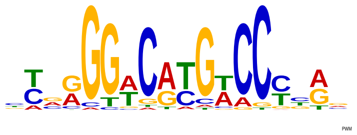	NNNAGGTCATGACCTNNN	85.50	IR:0
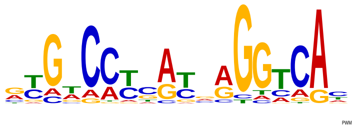	NNGTCCCCNNNAGGTCNN	23.40	DR:5
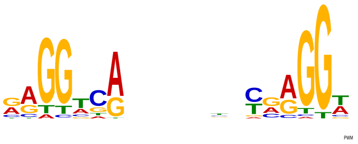	GAGGTCNNNNNNNNNGAGGT	68.59	DR:9
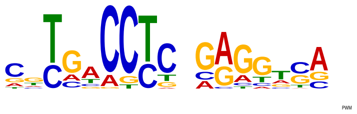	NNNGACCTCNGAGGTNNN	26.05	ER:3
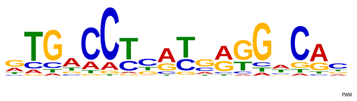	NNGACCTNNNNGGGACNN	11.93	ER:4
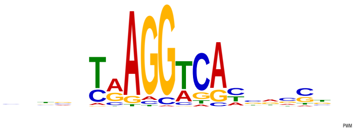	NNNNNTAAGGTCACGNNNNN	26.67	M
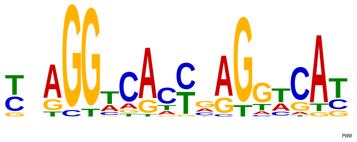	NNAGGTCACNNAGGTCNN	49.92	DR:3

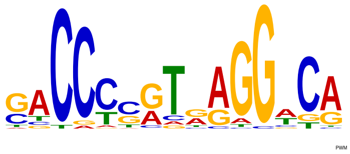	$\overleftarrow{NACCCC}GT\overrightarrow{NAGGACN}$	49.02	ER:4
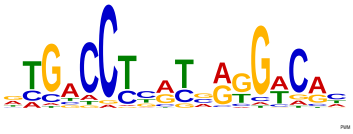	$N\overleftarrow{NGACCT}CNNN\overrightarrow{GGGACN}N$	23.87	ER:4
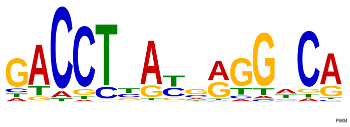	$\overleftarrow{NACCT}TANN\overrightarrow{GGGACN}$	24.83	ER:4
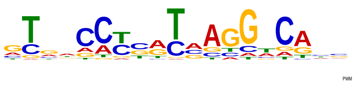	$N\overleftarrow{NNACCCC}NNN\overrightarrow{AGGTCN}NN$	8.55	ER:4
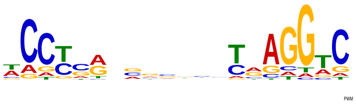	$\overleftarrow{ACCCC}NNNNNNNNNNN\overrightarrow{AGGTC}$	22.07	ER:11
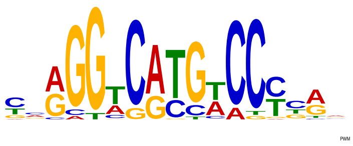	$NN\overrightarrow{AGGTCA}\overleftarrow{TGACCT}CNN$	175.56	IR:0
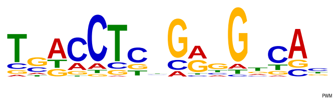	$N\overleftarrow{NGACCT}CNN\overrightarrow{GGGTCN}N$	14.72	ER:3

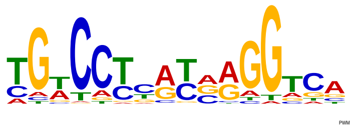	NGTCCTCNNNGGGTCN	28.64	ER:4
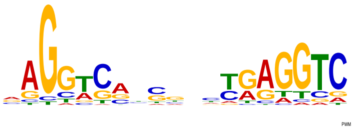	NAGGTCNNNNNNTGAGGTN	23.25	DR:7
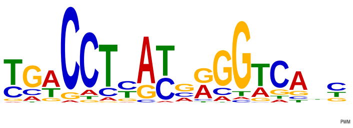	NNNCCTCANGGGGTCNNN	24.79	DR:2
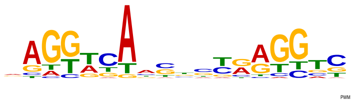	NAGGTCNNNNNTGAGGTN	11.79	DR:6
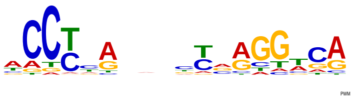	NCCTCANNNNNNAGGTCN	15.76	ER:8
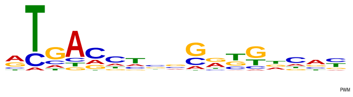	NNGACCTNNNGGGACNN	4.43	ER:3

23 THRA:RXRA Round 3

PWM	Seed Sequence	Seed Seq Enrichment	Repeat
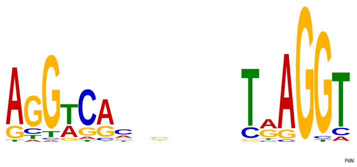	AGGTCNNNNNNNNNTAAGGT	122.24	DR:9
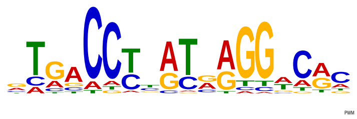	NNGACCTNNNNAGGACNN	13.92	ER:4
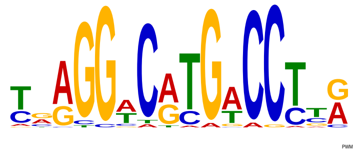	NNAGGACATGACCTNN	112.17	IR:0
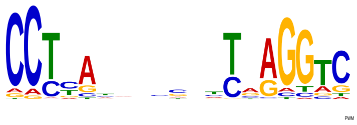	CCTCANNNNNNNNNNAGGTC	40.87	DR:9
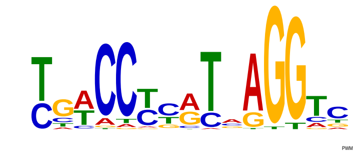	NNGACCTCNTAAGGNN	33.04	ER:4
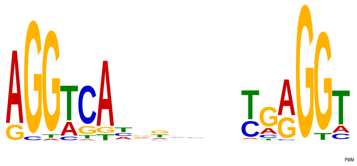	AGGTCANNNNNNNTGGGGT	114.65	DR:9
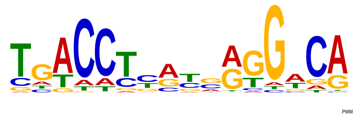	NGACCTNNNNGGGACN	13.77	ER:4

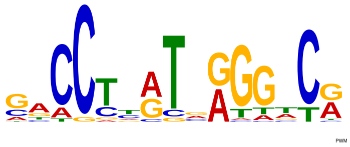	NACCTCANNAGGACN	22.74	ER:4
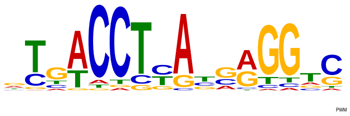	NTGACCTNNNGGGGTN	25.85	ER:4
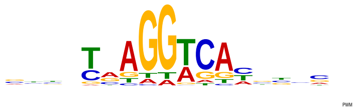	NNNNTAAGGTCACGNNNN	16.62	M
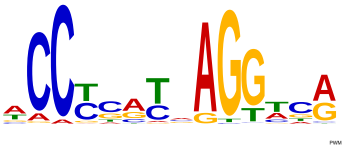	ACCCCANNAGGTCA	102.32	ER:4
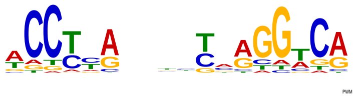	NCCTCANNNNNNAGGTCN	17.67	ER:8
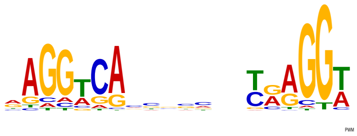	AAGGTNNNNNNNNNGAGGT	28.31	DR:9
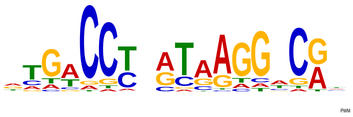	NNGACCCNNNNAGGACNN	9.08	ER:4

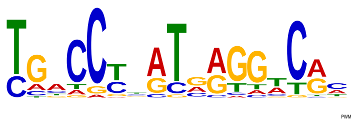	NNACCTCNNNAGGACNN	8.45	ER:4
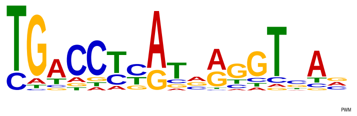	NGACCTNNNNNGGTGAN	10.44	ER:4
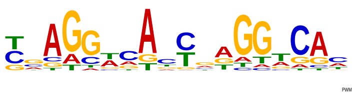	NNAGGTCNNNNAGGTCNN	6.59	DR:3
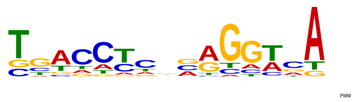	NNACCTCNNAGGTCNN	7.25	ER:3
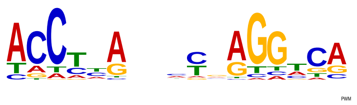	NCCTTANNNNNNAGGTCN	11.97	ER:7
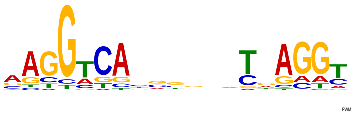	NAGGTCNNNNNNTGAGGN	15.82	IR:6

24 THRA:RXRA+7 Round 2

PWM	Seed Sequence	Seed Seq Enrichment	Repeat
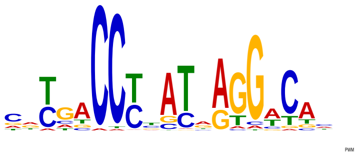	NNNGACCTNNNNAGGACNNN	95.83	ER:4
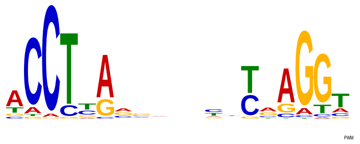	ACCTTANNNNNNNNNAAGGT	226.95	ER:11
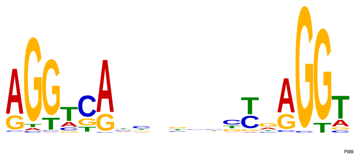	AGGTCANNNNNNNTGAGGT	526.18	DR:9
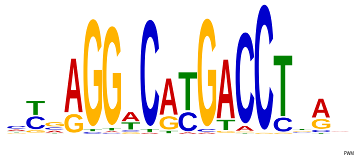	NNNAGGACATGACCTNNN	687.28	IR:0
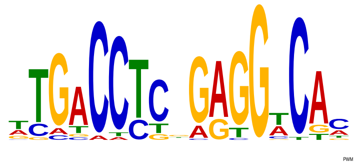	NNGACCTCNGAGGACNN	248.34	ER:3
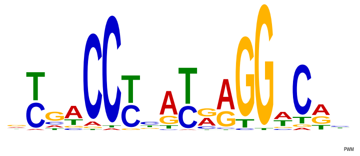	NNGACCTCNNNAGGACNN	128.41	ER:4
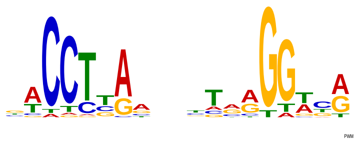	NACCTTANNNNNNAGGTCN	102.71	ER:8

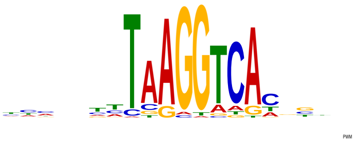	*NNNNNTTTAAGGTCANNNNN*	72.07	M
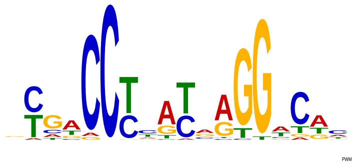	*NNGACCTCANNAGGACNN*	336.13	ER:4
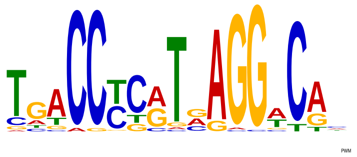	*NNACCCCGTNAGGACNN*	271.25	ER:4
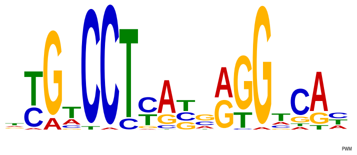	*NNGTCCTCANNGGGTCNN*	339.61	ER:4
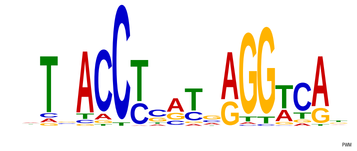	*NNTCACCTNNNNAGGTCNN*	126.05	ER:4
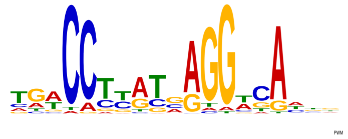	*NNNNCCTCANNAGGTCNNN*	62.94	ER:4
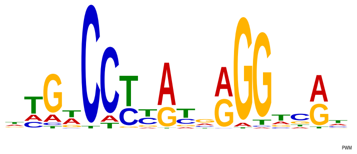	*NNGTCCTCNNNAGGTCNN*	126.12	ER:4

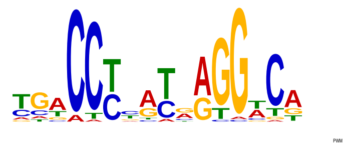	$N\overleftarrow{NNACCT}CANN\overrightarrow{AGGACN}NN$	142.43	ER:4
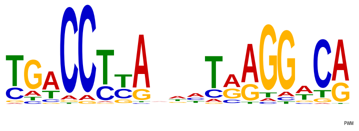	$\overleftarrow{NGACCT}NNNNNNN\overrightarrow{AGGACN}$	44.21	ER:7
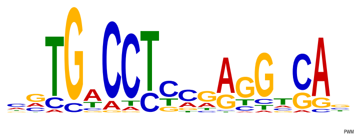	$NN\overleftarrow{NGACCT}CNG\overrightarrow{GGGTN}NN$	50.30	ER:3
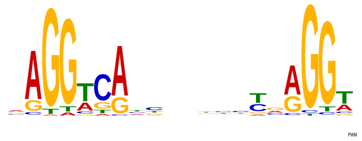	$A\overrightarrow{AGGTCN}NNNNNNNNG\overrightarrow{AGGT}$	209.48	DR:9
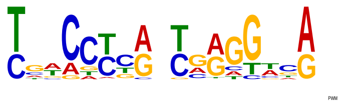	$N\overleftarrow{NGACCT}NNNNN\overrightarrow{AGGTCN}N$	16.75	ER:5
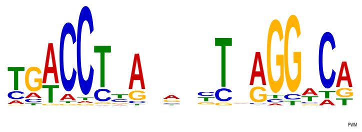	$\overleftarrow{NGACCT}NNNNNNNN\overrightarrow{AGGACN}$	73.45	ER:8

25 THRA+7 Round 3

PWM	Seed Sequence	Seed Seq Enrichment	Repeat
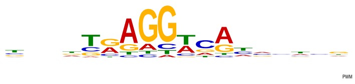	NNNNTAAGGTCACGNNNN	6.71	M
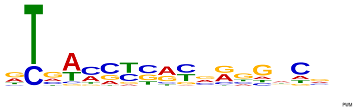	NNGACCTNNNNAGGACNN	4.19	ER:4
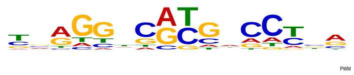	NNAGGTCNNGACCTNN	6.61	IR:0
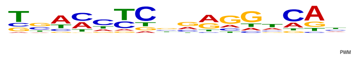	NNACCTCNNAGGTCNN	4.01	ER:3
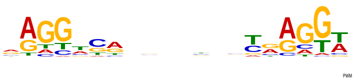	NAGGTCNNNNNNNTGAGGN	16.91	IR:7
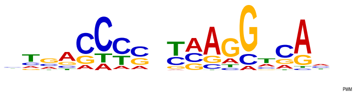	NNNACCCCNNNAGGTCNNN	5.44	ER:4
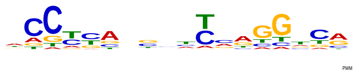	NCCTCANNNNNNNAGGTCN	6.23	ER:8

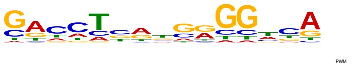	NNCCTCANNGGGTCNN	4.03	ER:4
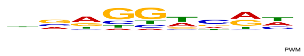	CTAAGGTCAC	4.67	M
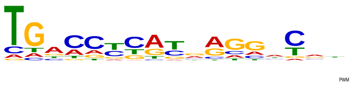	NNACCTCNNNAGGACNN	5.44	ER:4
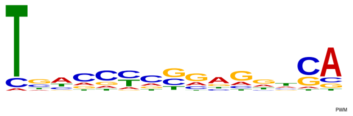	NNACCCCNGAGGTNN	3.96	ER:3
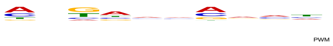	ATGAAAAAAT	2.49	M
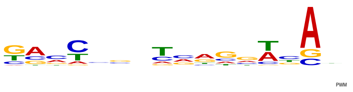	NNCCCCGNNAGGTCNN	2.52	M
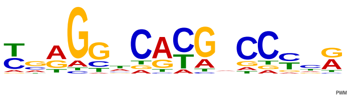	NNAGGTCANGTCCTNN	8.44	IR:0

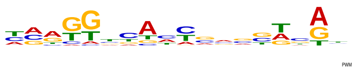	*NNAGGTCNNNNAGGTCNN*	2.76	DR:3
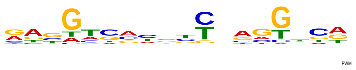	*NAGGTCNNNNNNAGGTCN*	5.14	DR:5
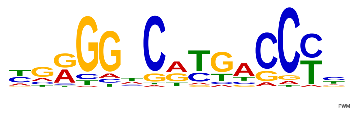	*NNAGGACATGACCNN*	11.93	IR:0
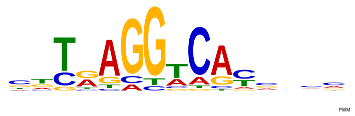	*NNTAAGGTCACGGNN*	10.22	M
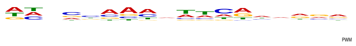	*NNNNCAAAATTCAANNNN*	3.59	M
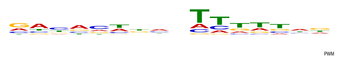	*NNACACTNNNTTTTTNN*	2.68	M

26 RARG Round 3

PWM	Seed Sequence	Seed Seq Enrichment	Repeat
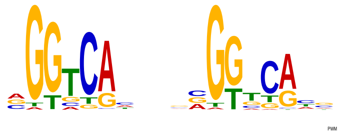	NNGGTCACNNNNGGTCANN	794.62	DR:4
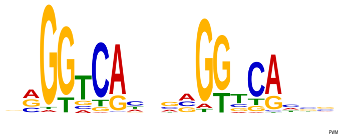	NNNGGTCACNNNGGTCANNN	907.04	DR:3
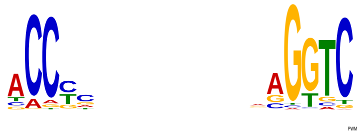	ACCCCNNNNNNNNNNAGGTC	459.37	ER:11
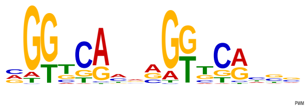	NNNNGGTCANNAGGTCNNNN	266.53	DR:2
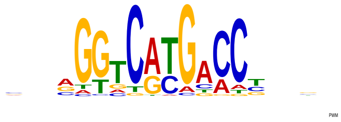	NNNNNGGTCATGACCNNNNN	507.96	IR:0
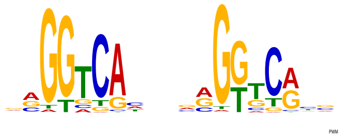	NNNGGTCACNNNAGGTCNNN	660.01	DR:4
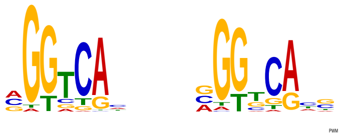	NNGGTCACNNNNNGGTCANN	390.51	DR:5

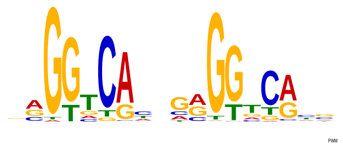	$NN\overrightarrow{NGGTCA}TNG\overrightarrow{GGGTCN}NN$	728.30	DR:3
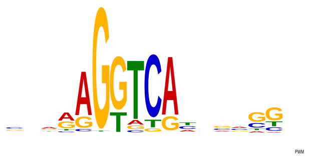	$NNNNNAA\overrightarrow{AGGTCA}CGNNNNN$	171.95	M
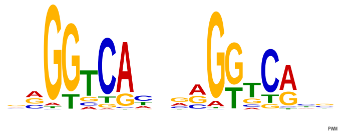	$NN\overrightarrow{NGGTCA}CNN\overrightarrow{AGGTCN}NN$	643.49	DR:3
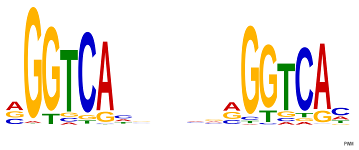	$\overrightarrow{NGGTCA}CNNNNN\overrightarrow{NGGTCA}N$	207.35	DR:6
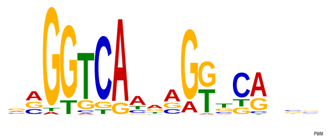	$NNN\overrightarrow{NGGTCA}AN\overrightarrow{AGGGCN}NNN$	358.11	DR:2
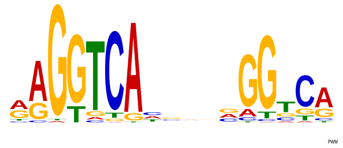	$NA\overrightarrow{AGGTCA}CNNN\overrightarrow{CGGTCN}$	909.37	DR:4
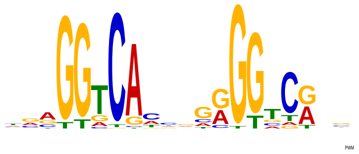	$NN\overrightarrow{NGGTCA}CCNG\overrightarrow{GGGTCN}NN$	2018.96	DR:4

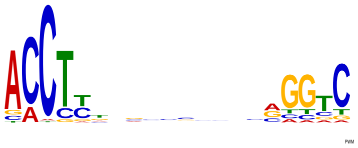	$\overleftarrow{ACCTT}NNNNNNNNNN\overrightarrow{CGGTC}$	162.58	ER:11
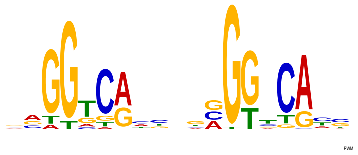	$N\overrightarrow{GGGTCA}CNNN\overrightarrow{NGGTCA}AN$	1070.56	DR:4
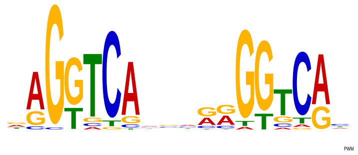	$N\overrightarrow{AGTTCA}CCNN\overrightarrow{NGGTCA}N$	596.77	DR:4
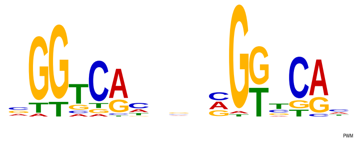	$\overrightarrow{GGGTCA}CNNN\overrightarrow{AGGTCA}A$	1712.20	DR:4
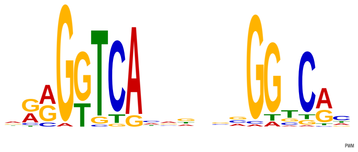	$NN\overrightarrow{AGGTCA}CNNNN\overrightarrow{CGGTCN}N$	386.79	DR:5
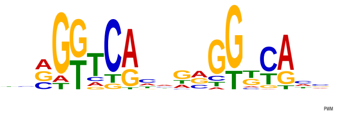	$NNN\overrightarrow{AGGTCA}CCN\overrightarrow{GGGTCN}NN$	689.89	DR:3

27 RARB Round 3

PWM	Seed Sequence	Seed Seq Enrichment	Repeat
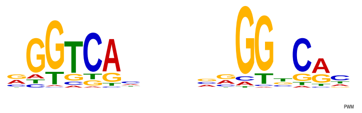	NGGTCACNNNNNGGTCAN	30.21	DR:5
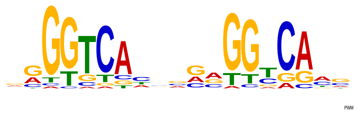	NNGGTCACNNNNGGTCANN	25.00	DR:4
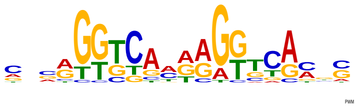	NNNNGGTCAANAGGTCNNNN	29.44	DR:2
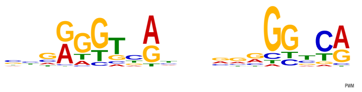	NNGGGGTCNNNNNGGGGTNN	18.28	DR:5
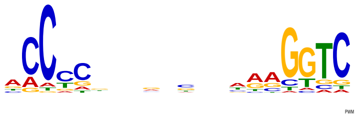	ACCCCNNNNNNNNNNNAGGTC	22.64	ER:11
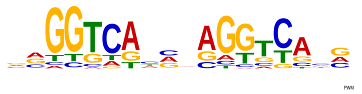	NNGGTCACNNNGGTCANN	19.67	DR:3
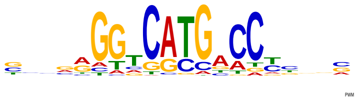	NNNNNGGTCATGACCNNNNN	29.15	IR:0

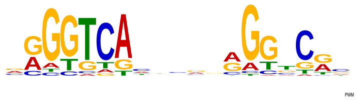	*NNGGTCACNNNNAGGTCNN*	31.99	DR:5
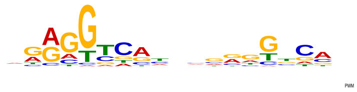	*NNNGGTCANNNGGGGTNNN*	8.00	DR:4
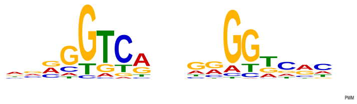	*NNNGGTCANNGGGGTCNNN*	14.57	DR:3
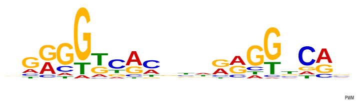	*NNNGGTCANNNNGGGGTNNN*	9.63	DR:5
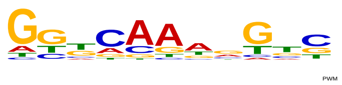	*GGTCAAAGGTC*	12.37	DR:1
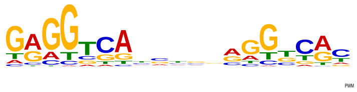	*NNGGTCANNNNNCGGTCNN*	7.64	DR:5
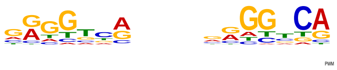	*NNGGGTCNNNNNGGGGTNN*	9.44	DR:5

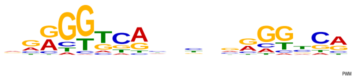	NNAGGTCNNNNNGGGGTNN	8.55	DR:5
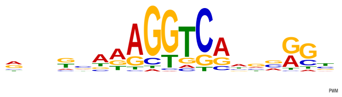	NNNNNCAAAGGTCAANNNNN	14.74	M
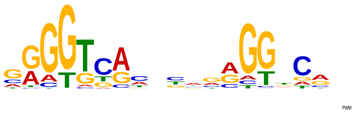	NNGGTCANNNNNGGGGTCNN	22.13	DR:5
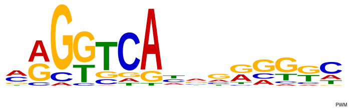	NAGGTCACNGGGGTN	19.48	DR:3
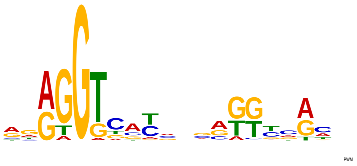	NNNGGTCANNGGGGTCNNN	13.35	DR:4
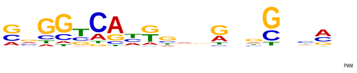	NNGGTCANNNNNNGGGTCNN	5.35	DR:6

28 RARA Round 3

PWM	Seed Sequence	Seed Seq Enrichment	Repeat
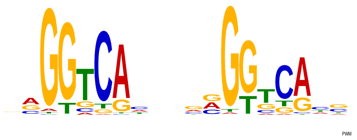	NNGGTCACNNNNGGTCANN	393.20	DR:4
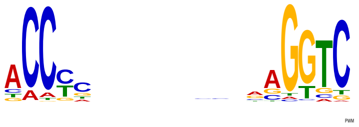	ACCCCNNNNNNNNNNAGGTC	273.20	ER:11
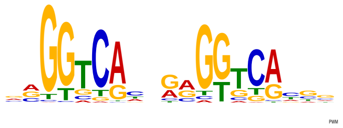	NNNGGTCACNNNGGTCANNN	345.19	DR:3
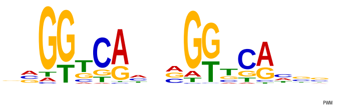	NNNGGTCANNNGGTCANNN	164.46	DR:2
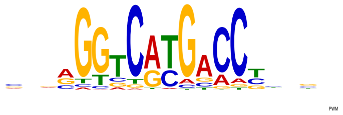	NNNNNGGTCATGACCNNNNN	314.14	IR:0
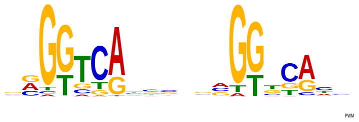	NNGGTCACNNNNNGGTCANN	200.63	DR:5
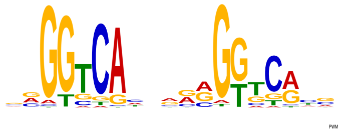	NNNGGTCACNNNAGGTCNNN	289.84	DR:4

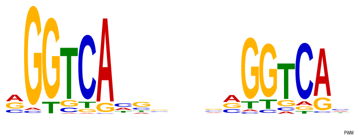	$N\overrightarrow{GGTCA}CNNNNNN\overrightarrow{GGTCA}N$	136.87	DR:6
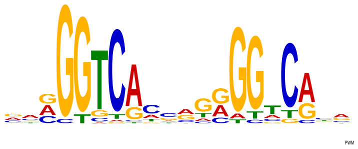	$NN\overrightarrow{NGGTCA}CCNG\overrightarrow{GGGTCN}NN$	962.66	DR:4
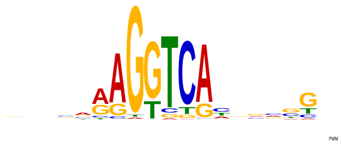	$NNNNNAA\overrightarrow{AGGTCA}CGNNNNN$	126.65	M
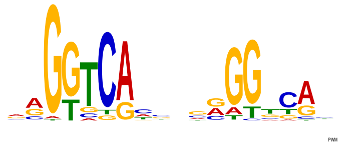	$NN\overrightarrow{AGGTCA}CNNN\overrightarrow{GGGTCN}N$	419.79	DR:4
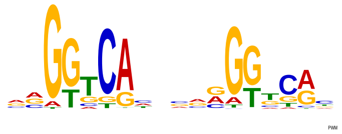	$NN\overrightarrow{AGGTCA}CNNN\overrightarrow{AGGTCN}N$	348.05	DR:4
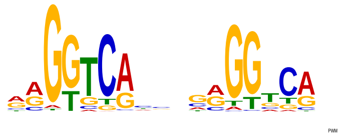	$NN\overrightarrow{AGGTCN}NNNG\overrightarrow{GGGTCN}N$	178.92	DR:4
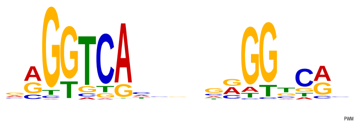	$N\overrightarrow{NGGTCA}CNNNN\overrightarrow{GGGTCN}N$	179.51	DR:5

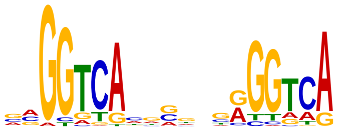	$NN\overrightarrow{GGTCA}CNNNNG\overrightarrow{GGGTCN}N$	181.14	DR:6
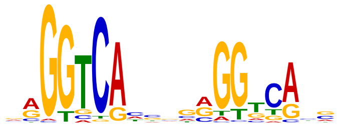	$NN\overrightarrow{NGGTCA}CNNN\overrightarrow{GGGTCN}NN$	280.22	DR:4
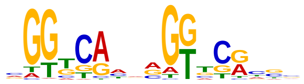	$NNN\overrightarrow{NGGTCA}AN\overrightarrow{AGGTCN}NNN$	272.66	DR:2
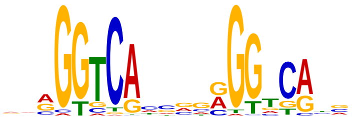	$NN\overrightarrow{NGGTCA}CNNG\overrightarrow{GGGTCN}NN$	527.66	DR:4
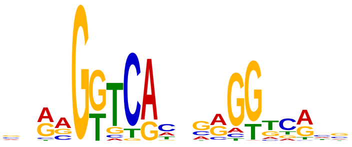	$NNN\overrightarrow{AGGTCA}CNN\overrightarrow{GGGTCN}NN$	324.05	DR:3
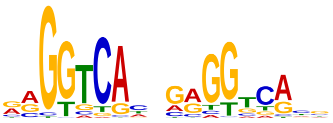	$NN\overrightarrow{NGGTCA}CNN\overrightarrow{GGGTCN}NN$	204.07	DR:3

29 RARG+8 Round 2

PWM	Seed Sequence	Seed Seq Enrichment	Repeat
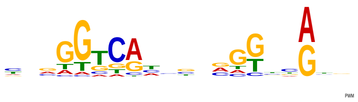	*NNNGGTCANNNNGGGTCNNN*	10.85	DR:4
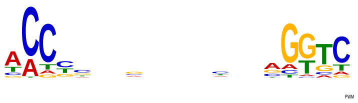	*ACCCCNNNNNNNNNNAGGTC*	20.61	ER:11
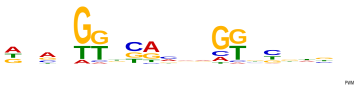	*NNNNGGTCANNAGGTCNNNN*	9.85	DR:2
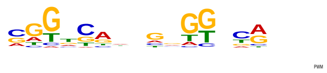	*NNNGGTCANNNNGGTCANNN*	13.48	DR:3
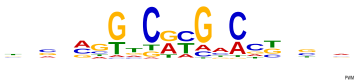	*NNNNNGGTCATGACCNNNNN*	15.05	IR:0
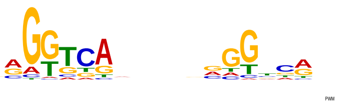	*NNGGTCANNNNNGGGTCNN*	11.82	DR:5
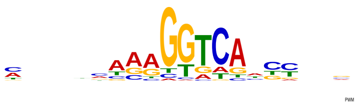	*NNNNNCAAAGGTCATNNNNN*	13.97	M

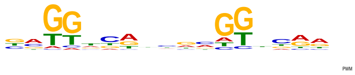	NNGGTCACNNNGGTCANN	16.91	DR:3
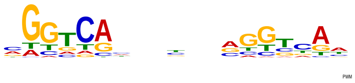	NGGTCANNNNNNNGGTCAN	7.86	DR:6
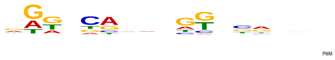	NNNGTCAANNGGTCANNN	8.52	DR:2
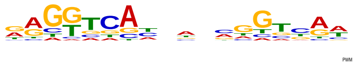	NAGTTCNNNNNNGGTCAN	7.54	DR:4
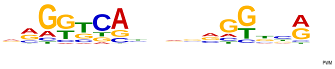	NNAGGTCNNNNNGGGTCNN	7.68	DR:4
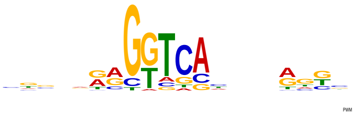	NNNNNGAGGTCACGGNNNNN	12.32	M
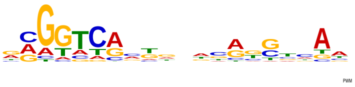	NNGGTCANNNNNNGGGTCNN	5.99	DR:6

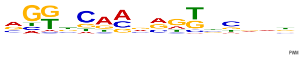	$NNN\overrightarrow{AGGTC}NNN\overrightarrow{AGGTC}NNN$	7.66	DR:2
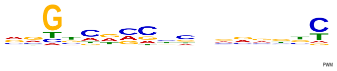	$N\overrightarrow{AGGTC}NNNNNNA\overrightarrow{AGGTN}$	3.61	DR:6
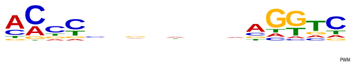	$\overleftarrow{ACCC}CNNNNNNNN\overrightarrow{AGGTC}$	5.46	ER:8
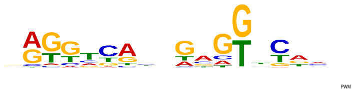	$N\overrightarrow{NNGTCA}CNN\overrightarrow{AGGTC}NNN$	8.13	DR:3
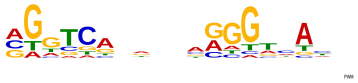	$\overrightarrow{NNGTCA}CNNNN\overrightarrow{NGGTCA}NN$	8.46	DR:5
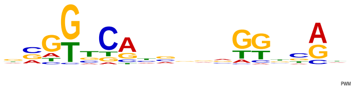	$NN\overrightarrow{GGTCA}ANNN\overrightarrow{GGGTCN}N$	12.22	DR:4

30 RARB+8 Round 3

PWM	Seed Sequence	Seed Seq Enrichment	Repeat
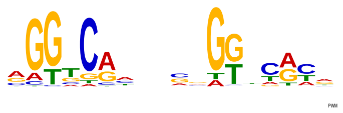	NNGGTCACNNNNGGTCANN	40.50	DR:4
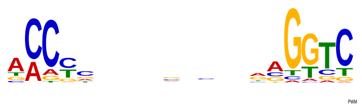	ACCCCNNNNNNNNNNNAGGTC	38.67	ER:11
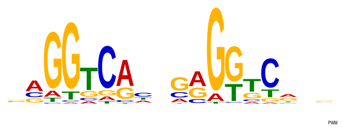	NNNGGTCACNNAGGTCNNN	47.45	DR:3
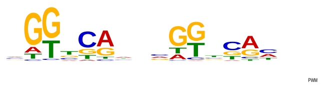	NNNGGTCANNNGGTCANNN	25.86	DR:2
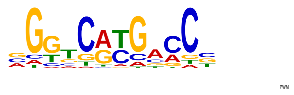	NNNNNGGTCATGACCNNNNN	36.72	IR:0
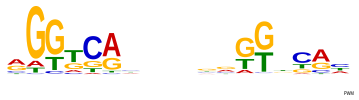	NGGTCACNNNNNGGTCAN	26.69	DR:5
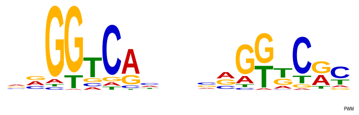	NNGGTCACNNNAGGTCNN	39.34	DR:4

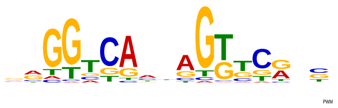	NNNGGTCAANAGTTCNNN	51.57	DR:2
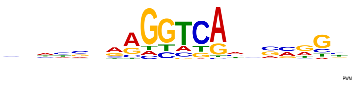	NNNNNAAAGGTCACGNNNNN	14.12	M
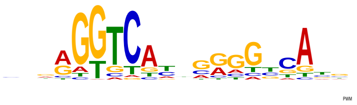	NNNNGGTCATNGGGGTNNNN	31.89	DR:3
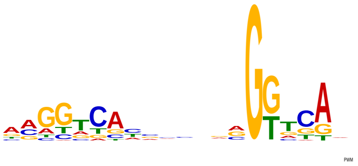	NNGGTCANNNNNNAGGTCNN	10.02	DR:6
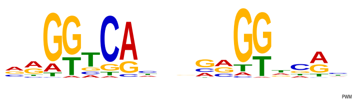	NNGGTCACNNNGGGTCNN	34.52	DR:4
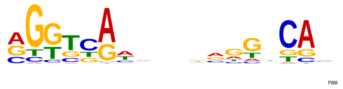	NNGGTCANNNNNCGGTCNN	10.31	DR:5
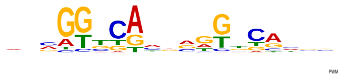	NNNNGGTCANNGGGTCNNNN	17.73	DR:2

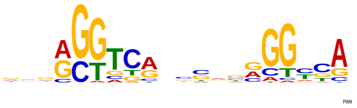	$NNG\overrightarrow{GGGTCN}NNNNG\overrightarrow{GGGTNN}$	20.42	DR:5
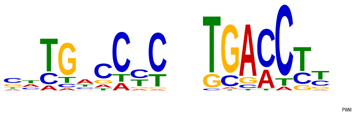	$N\overrightarrow{NNGTCC}CCNN\overleftarrow{TGACCN}NN$	18.43	IR:4
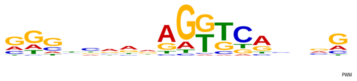	$NNNNCAAG\overrightarrow{AGTTCA}NNNN$	11.42	M
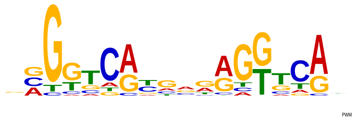	$N\overrightarrow{NGGTCA}NNNG\overrightarrow{AGTTCN}N$	24.00	DR:4
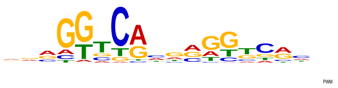	$NNN\overrightarrow{AGGTCN}NN\overrightarrow{GGGTCN}NN$	13.71	DR:2
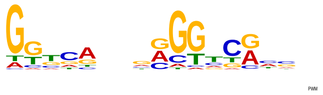	$N\overrightarrow{NNGTCA}CNN\overrightarrow{NGGTCA}NNN$	13.39	DR:3

31 RARA+8 Round 3

PWM	Seed Sequence	Seed Seq Enrichment	Repeat
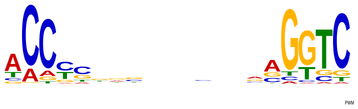	ACCCCNNNNNNNNNNAGGTC	34.38	ER:11
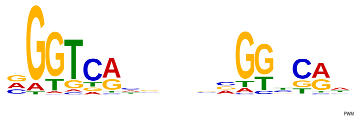	NGGTCACNNNNNGGTCAN	31.94	DR:5
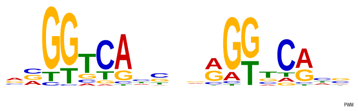	NNGGTCACNNNGGTCANN	37.59	DR:4
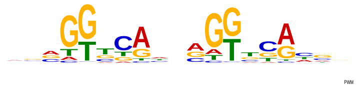	NNNGGTCANNNGGTCANNN	20.23	DR:2
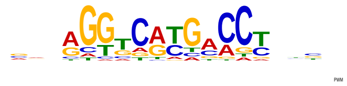	NNNNNGGTCATGACCNNNNN	28.83	IR:0
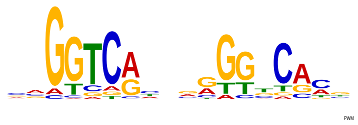	NNGGTCACNNNGGTCANN	31.12	DR:3
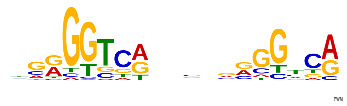	NNGGGGTCNNNNNGGGGTNN	25.44	DR:5

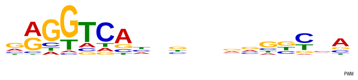	$NN\overrightarrow{GGTCA}NNNNNNG\overrightarrow{GGGTN}N$	8.13	DR:6
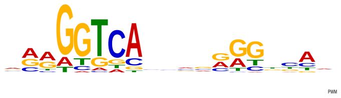	$NNN\overrightarrow{AGGTCN}NNNG\overrightarrow{GGGTN}NN$	9.12	DR:4
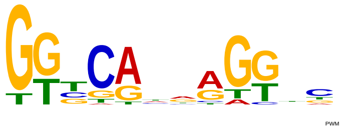	$\overrightarrow{GGTCA}AG\overrightarrow{GGGTC}$	29.99	DR:2
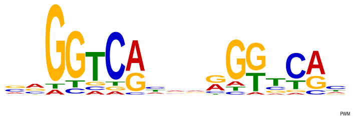	$NN\overrightarrow{GGTCA}CNN\overrightarrow{AGGTCN}N$	21.20	DR:3
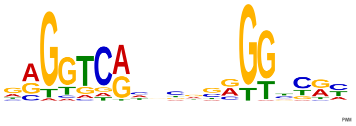	$N\overrightarrow{NGGTCA}CNNNN\overrightarrow{GGGTCN}N$	30.26	DR:5
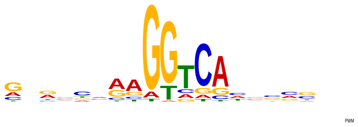	$NNNNNAA\overrightarrow{AGGTCA}CGNNNNN$	16.52	M
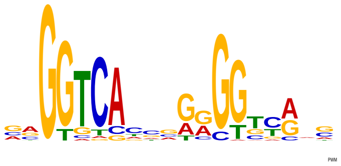	$NN\overrightarrow{NGGTCA}CNNN\overrightarrow{GGGTCN}NN$	42.91	DR:4

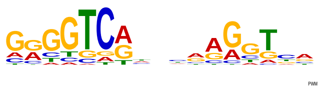	$NNG\overrightarrow{GGGTCN}NNNA\overrightarrow{AGGTNN}$	19.78	DR:4
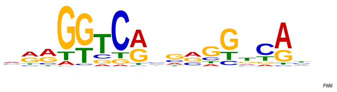	$NNN\overrightarrow{AGGTCN}NN\overrightarrow{GGGTCN}NN$	10.93	DR:2
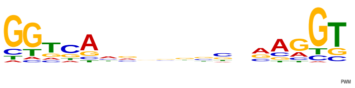	$\overrightarrow{GGTCA}NNNNNNNNA\overrightarrow{AGGT}$	12.72	DR:9
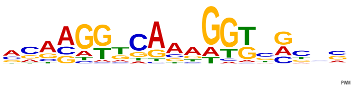	$NNN\overrightarrow{NGGTCA}A\overrightarrow{AGGTCN}NNN$	18.55	DR:1
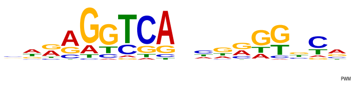	$NNA\overrightarrow{AGGTCN}NNG\overrightarrow{GGGTNN}$	12.09	DR:3
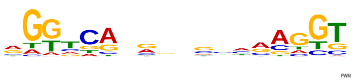	$\overrightarrow{AGGTCN}NNNNNNNA\overrightarrow{AGGT}$	10.69	DR:8

32 RARG:RXRA Round 3

PWM	Seed Sequence	Seed Seq Enrichment	Repeat
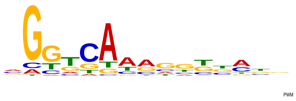	*NNNNGGTCAAAGGTNNNN*	10.43	DR:1
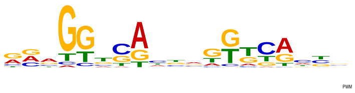	*NNNGGTCANNNGGTCANNN*	9.54	DR:2
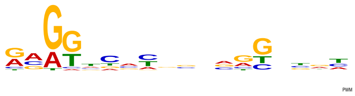	*NNGGTCANNNNGGGTCNN*	5.25	DR:4
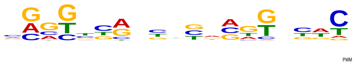	*NNGGTCANNNNNAGGTCNN*	8.24	DR:5
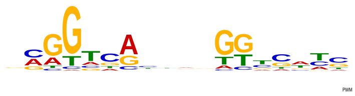	*NNGGTCANNNNGGTCANN*	7.34	DR:3
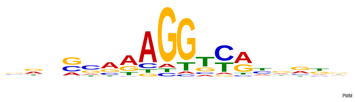	*NNNNCAAAGGTCATNNNN*	10.55	M
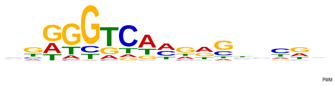	*NNNNGGTCAAGAGTNNNN*	8.37	M

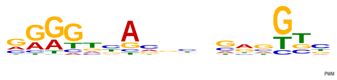	NGGGGTNNNNNNGAGGTN	7.68	DR:5
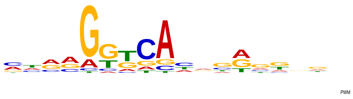	NNNNGGTCACGGGGNNNN	11.38	M
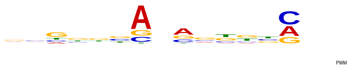	NNNGTCAAAGGTCNNN	5.00	DR:1
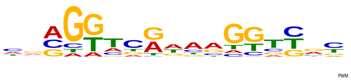	NNNGGTCGNAAGGTNNN	5.28	DR:2
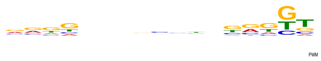	AAGGGNNNNNNNNNGGGGT	6.65	DR:8
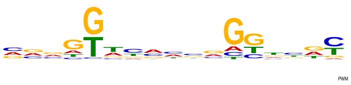	NNGGGTCNNNGGGTCNN	5.38	DR:2
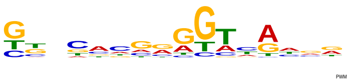	NNNCACGGGGTCANNN	7.07	M

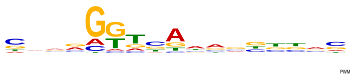	*NNNGGGTCNAAGGTNNN*	6.29	DR:1
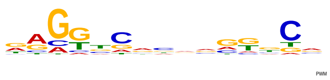	*NNAGGTCNNGAGGTNN*	4.89	DR:2
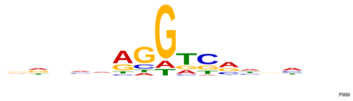	*NNNAAAGGTCAATNNN*	5.71	M
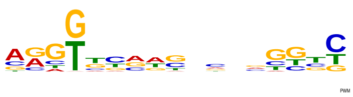	*NAGGTCNNNNNGAGGTN*	5.78	DR:5
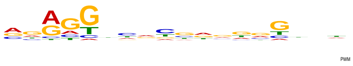	*NNGGGTCNNNNGGGGTNN*	5.13	DR:4
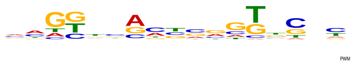	*NNGGTCANNNAGGTCNN*	4.46	DR:3

33 RARB:RXRA Round 3

PWM	Seed Sequence	Seed Seq Enrichment	Repeat
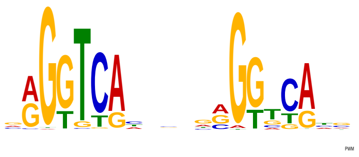	NNGGTCACNNNNNGGTCANN	1914.70	DR:5
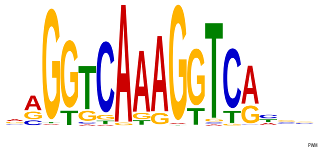	NNNNGGTCAAAGGTCNNNN	1042.60	DR:1
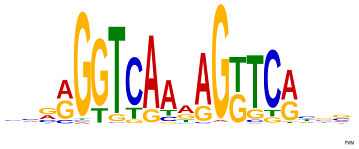	NNNNGGTCAANAGGTCNNNN	1217.15	DR:2
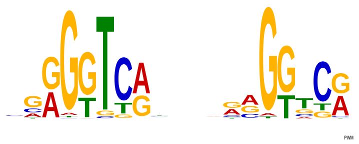	NGGGGTCNNNNNNAGGTCN	1839.66	DR:5
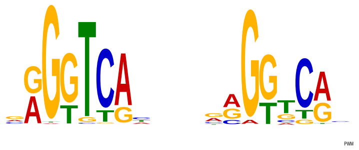	NNGGTCACNNNNAGGTCNN	1644.95	DR:5
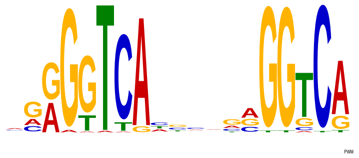	NGGGGTCACNNNNCGGTCN	3309.21	DR:5
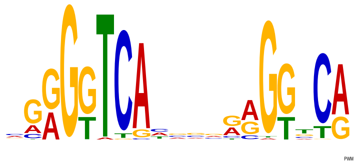	NGGGGTCACNNNNAGGGCN	2958.56	DR:5

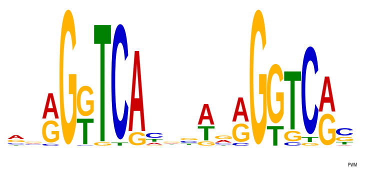	NNAGTTCACNNNGAGGTCNN	3010.66	DR:5
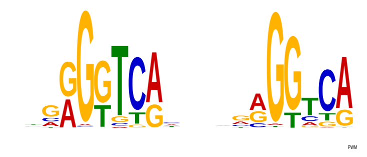	NNGGGGTCNNNNNGGGGTNN	723.10	DR:5
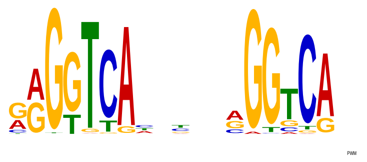	NNGGTCAANNNNCGGTCNN	585.24	DR:5
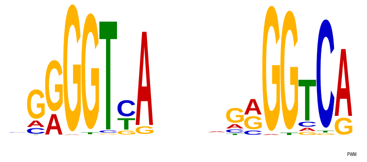	NGGGGTTANNNNGGGGTCN	2594.08	DR:5
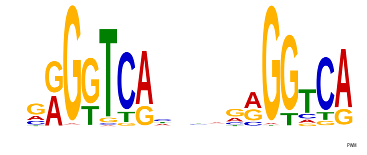	NNGGGTCNNNNNGGGGTNN	365.71	DR:5
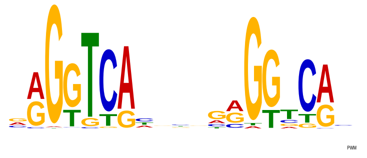	NNGGTCACNNNGGGGTCNN	2620.62	DR:5
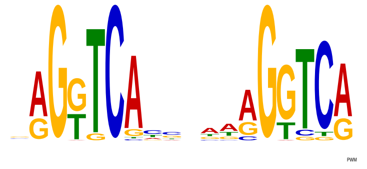	NAGTTCACCNNNAGGTCN	2489.40	DR:5

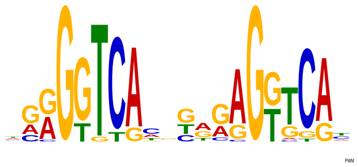	$NN\overrightarrow{AGGTCA}CNNNG\overrightarrow{AGTTCN}N$	3449.46	DR:5
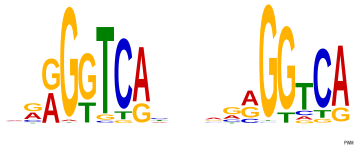	$NN\overrightarrow{AGGTCN}NNNNG\overrightarrow{GGGTNN}$	340.74	DR:5
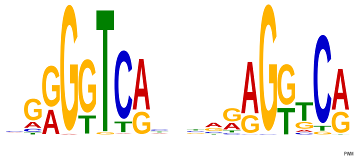	$NG\overrightarrow{GGGTCN}NNNNG\overrightarrow{AGTTCN}$	2031.49	DR:5
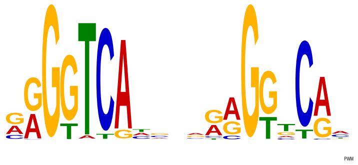	$N\overrightarrow{NGGTCA}CNNNG\overrightarrow{AGGGCN}N$	1301.92	DR:5
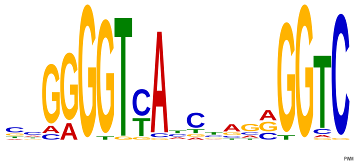	$NCG\overrightarrow{GGGTTA}NNNNG\overrightarrow{GGGTN}$	604.98	DR:5
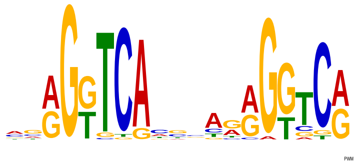	$NG\overrightarrow{AGTTCA}CNNNN\overrightarrow{AGGTCN}$	3404.94	DR:5

34 RARA:RXRA Round 3

PWM	Seed Sequence	Seed Seq Enrichment	Repeat
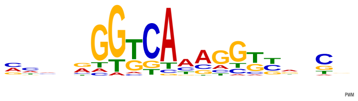	NNNNNGGTCAAAGGTNNNNN	43.94	DR:1
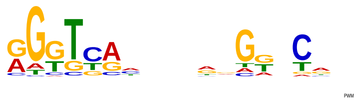	NGGTCANNNNNNGGGCAN	35.06	DR:5
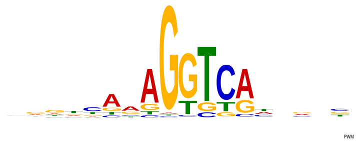	NNNNCAAAGGTCATNNNN	44.72	M
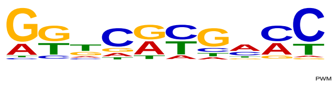	GGTCATGACC	46.82	IR:0
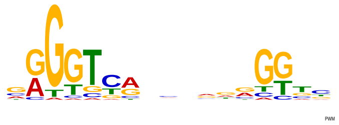	NGGGGTCNNNNNGGGGTN	56.26	DR:5
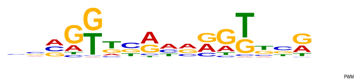	NNNGGTCANNAGGTCNNN	20.78	DR:2
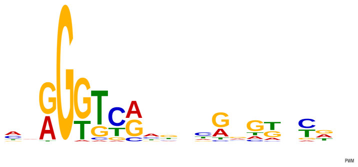	NNAGGTCNNNNNNAGGGCNN	35.01	DR:5

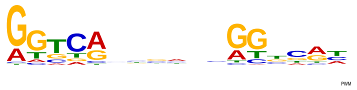	$\overrightarrow{NGTCA}CNNNN\overrightarrow{NGGTCA}N$	24.17	DR:5
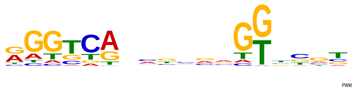	$\overrightarrow{NNGTCA}CNNNN\overrightarrow{AGGTCN}N$	23.26	DR:5
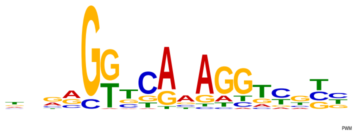	$NNN\overrightarrow{AGGTCN}A\overrightarrow{AGGTCN}NN$	68.75	DR:1
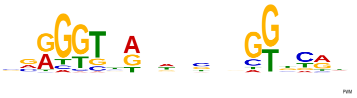	$NG\overrightarrow{GGGTNN}NNNNN\overrightarrow{NGGTCA}N$	37.44	DR:5
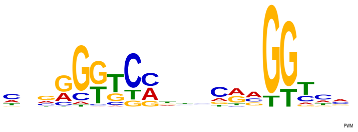	$NNG\overrightarrow{GGGTNN}NNNNA\overrightarrow{AGGTNN}$	34.17	DR:5
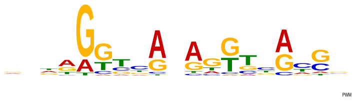	$NNN\overrightarrow{NGGTCA}N\overrightarrow{GGGTCN}NNN$	17.89	DR:1
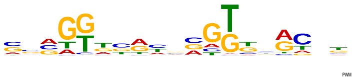	$NN\overrightarrow{NGGTCA}NN\overrightarrow{NGGTCA}NNN$	22.91	DR:2

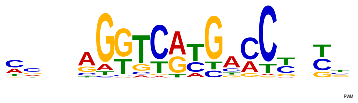	NNNNAGGTCATGACCNNNN	125.11	IR:0
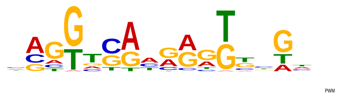	NNNGGTCAANAGGTCNNN	45.15	DR:2
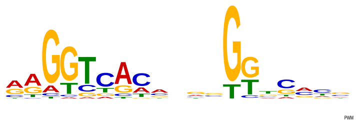	NNGGTCANNNNNGGTCANN	18.29	DR:4
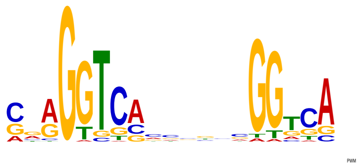	NNAGGTCNNNNNNCGGTCNN	69.15	DR:5
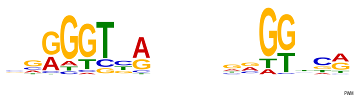	NGGGGTNNNNNNNGGGTCN	36.34	DR:5
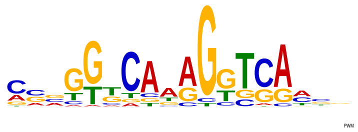	NNNNGTCAAAGTTCNNNN	22.00	DR:1

35 PXR Round 3

PWM	Seed Sequence	Seed Seq Enrichment	Repeat
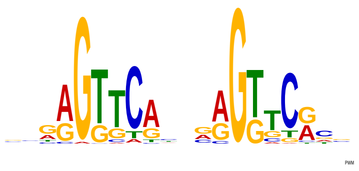	NNNAGTTCANNNAGTTCNNN	256.43	DR:3
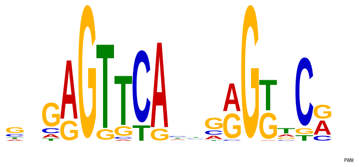	NNGAGTTCANNGGGGTCNN	510.61	DR:3
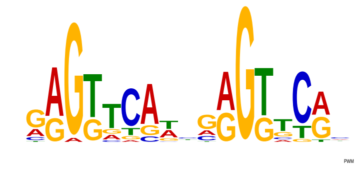	NNAGTTCNNCGGGTTCNN	198.71	DR:3
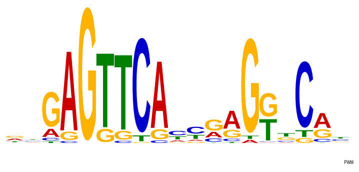	NNGAGTTCANNNAGGGCNN	215.71	DR:3
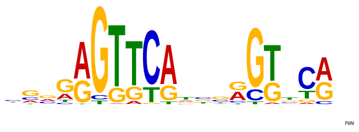	NNNGAGTTCNNNGGGGTNNN	53.86	DR:3
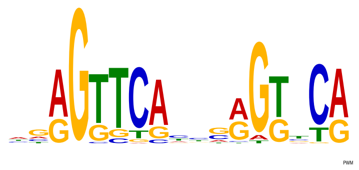	NNAGTTCANCGGGGTNN	106.88	DR:3
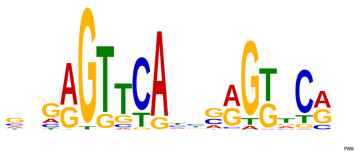	NNGAGTTCNNNGGGGTCNN	171.41	DR:3

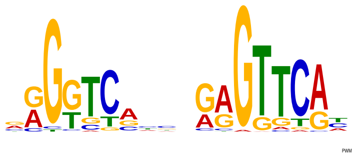	NNGGTCANNNGAGTTCNN	64.16	DR:4
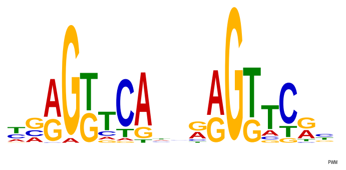	NNGGGTTCNNNGAGTTCNN	246.50	DR:3
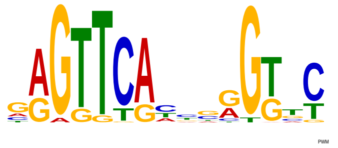	NGAGTTCANCGGGGTN	245.36	DR:3
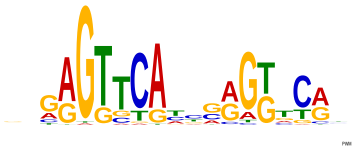	NNGAGTTCNNNNGGGTCNN	88.46	DR:3
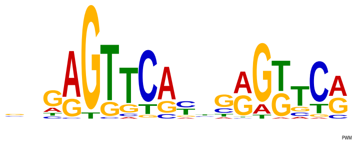	NNGAGTTCNNTGGGGTNN	101.47	DR:3
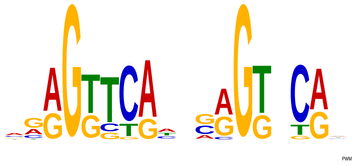	NNAGTTCNNCGGGGTCNN	103.90	DR:3
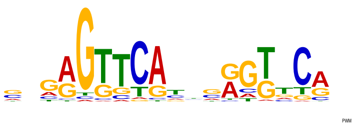	NNNAGTTCNNNGGGGTNNN	25.34	DR:3

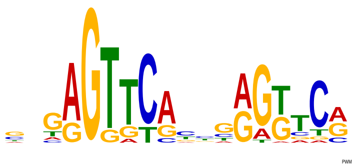	$NNG\overrightarrow{AGTTCA}N\overleftarrow{TGGGGT}NN$	234.81	IR:1
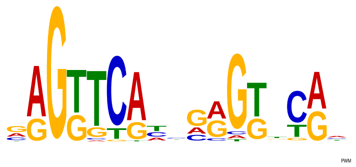	$N\overrightarrow{NGTTCA}NCG\overrightarrow{GGGTCN}N$	131.90	DR:3
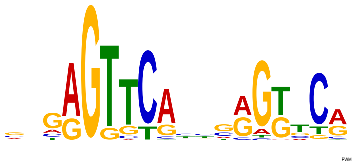	$NNG\overrightarrow{AGTTCA}NNG\overrightarrow{GGGTNN}$	172.61	DR:3
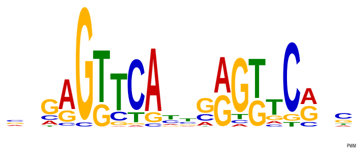	$NNN\overrightarrow{AGTTCN}NNG\overrightarrow{GGGTCN}NN$	91.83	DR:3
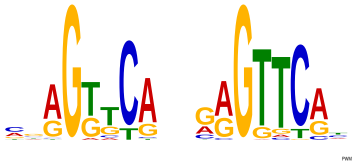	$NN\overrightarrow{AGTGCA}NNG\overrightarrow{AGTTCN}N$	219.02	DR:3
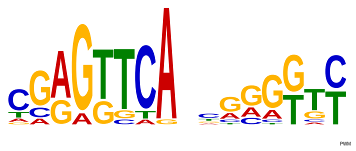	$NG\overrightarrow{GGTTCA}NCG\overrightarrow{GGGTN}$	130.13	DR:3

36 PXR+9 Round 3

PWM	Seed Sequence	Seed Seq Enrichment	Repeat
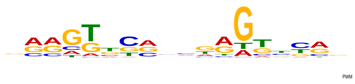	NNAGTTCNNNNAGTTCNN	8.90	DR:3
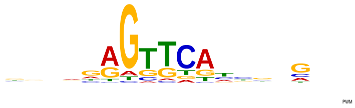	NNNNGAGTTCATCGNNNN	12.29	M
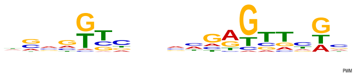	NNNGGTCANNNGAGTTNNN	5.00	DR:4
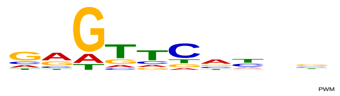	GAGTTCACAT	5.29	M
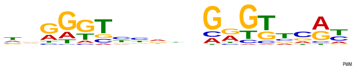	NNGGGTCNNNNNGGTTCNN	5.05	DR:4
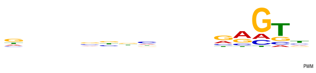	GGGGGNNNNNNNNGAGTT	5.26	M
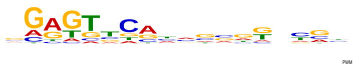	NNAGTTCNNNNGGGGCNN	5.46	DR:3

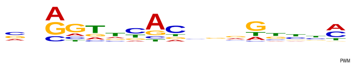	*NNAGTTCNNNCAGTTNN*	4.02	DR:3
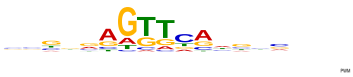	*NNNNGAGTTCAAGTNNNN*	6.46	M
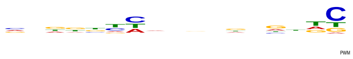	*NGGGTCNNNNNGAGTTN*	2.76	DR:5
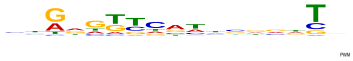	*NNNGGTTCNTTCGGNNN*	3.45	M
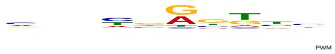	*CGTCGGGTTC*	3.26	DR:0
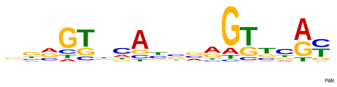	*NNGGGTTNNNNNAGTTCNN*	6.94	DR:4
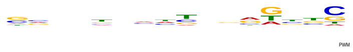	*NGGGGCNNNNGAGTTN*	2.71	DR:4

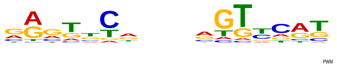	$NN\overrightarrow{AGTGCN}NNN\overrightarrow{AGTTCN}N$	6.17	DR:3
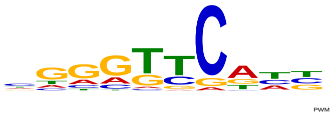	$CG\overrightarrow{GGTTCA}TG$	9.80	M
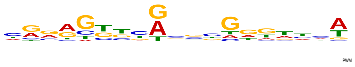	$NNN\overrightarrow{AGGTCN}NNC\overrightarrow{GGGTNN}N$	3.90	DR:3
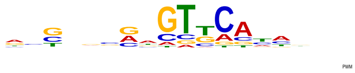	$NNNNGCA\overrightarrow{AGTTCA}TNNNN$	6.54	M
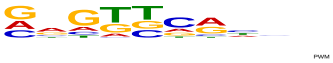	$G\overrightarrow{AGTTCA}CTA$	4.74	M
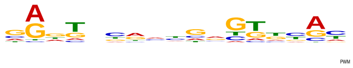	$N\overrightarrow{NGGGCA}NNN\overrightarrow{AGTTCN}N$	4.17	DR:3

37 VDR Round 3

PWM	Seed Sequence	Seed Seq Enrichment	Repeat
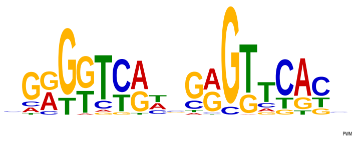	NNNGGTCANNGGGTTCNNN	26.21	DR:3
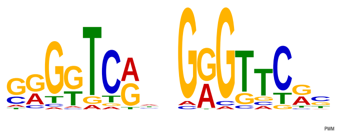	NNGGGTCNNNGGGTTCNN	32.33	DR:3
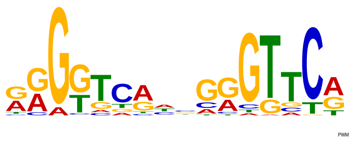	NNGGTCATNGGGTTNN	15.47	DR:2
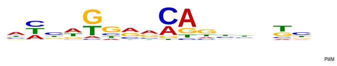	NNNNAGGAACAGTTNNNN	3.33	DR:0
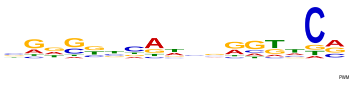	NNNGGTCANCGGGGNNN	2.73	M
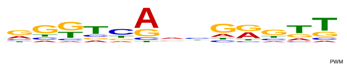	GGGTCNNNGAGTG	4.03	M
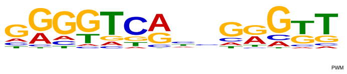	GGGGTCNNNGGGTG	10.00	DR:2

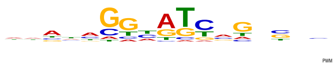	*NNNNTAGGTATCAGNNNN*	3.91	M
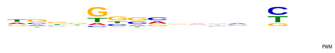	*NNNGGGGGNGAGAANNN*	2.41	M
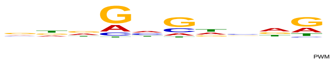	*GTAGAGTCAG*	2.96	M
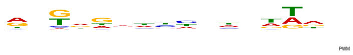	*NNNAGATCCTTATNNN*	2.06	M
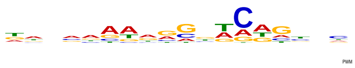	*NNNAAAAANNGTCAGNNN*	3.12	M
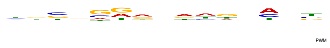	*NNNGGGGATAAGANNN*	2.20	M
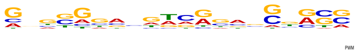	*NNGGGGANNNNNNAGGGGNN*	2.24	DR:6

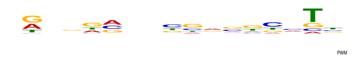	*NNNGGAGGNGAGGCNNN*	2.72	M
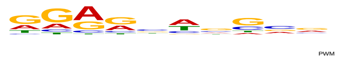	*GGAGCAGGCG*	2.81	M
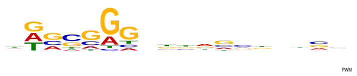	*NNNNGGGGGTAGCGNNNN*	3.39	M
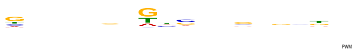	*NNGGGGCNNNNAGGGGNN*	1.79	DR:3
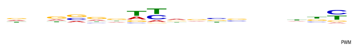	*NAGGGGNNNNNGAGTGN*	2.48	M
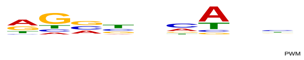	*GAGGTTCAGT*	2.43	M

38 VDR:RXRA Round 3

PWM	Seed Sequence	Seed Seq Enrichment	Repeat
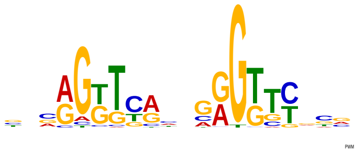	*NNNNGGTCANNGGGTTNNNN*	45.62	DR:2
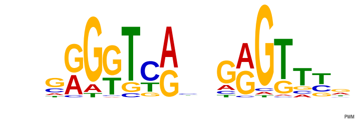	*NNGGGGTCNNNGAGTTNN*	90.82	IR:1
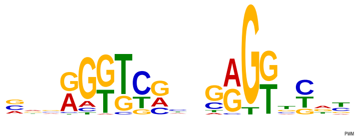	*NNGGGGTCNNNNGGTTCNN*	58.72	DR:2
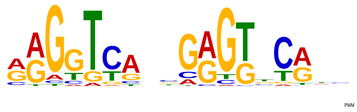	*NNGGTCANNNNGTGCANN*	25.96	DR:3
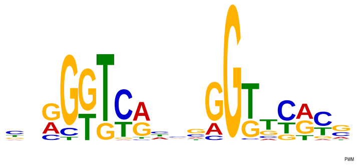	*NNNGGTCATNNGGTTCNNN*	89.20	DR:2
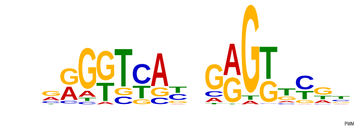	*NNGGGGTNNNNGGGTTCNN*	57.20	DR:3
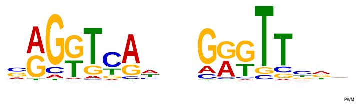	*NNGGTCANNNNGGTTCNN*	19.67	DR:4

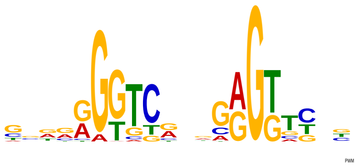	$NNNG\overrightarrow{GGGTCN}NN\overrightarrow{GGGTTN}NN$	90.02	DR:2
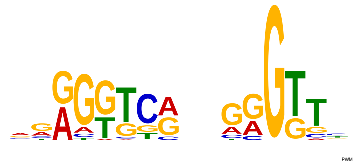	$NG\overrightarrow{GGGTCN}NT\overrightarrow{GGGTTN}$	99.68	DR:2
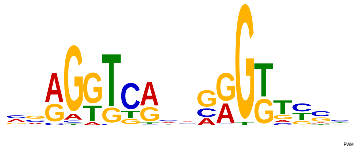	$NN\overrightarrow{NGGTCA}AN\overrightarrow{GGGTTN}NN$	66.21	DR:2
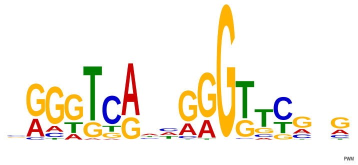	$N\overrightarrow{NNGTCA}ANG\overrightarrow{GGTTCN}NN$	80.72	DR:3
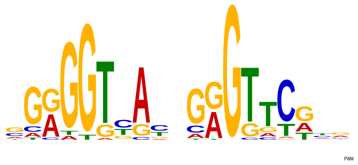	$NN\overrightarrow{NGGTTA}NNG\overrightarrow{GGTTCN}NN$	82.81	DR:3
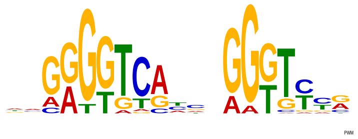	$NNG\overrightarrow{GGGTCA}NNN\overrightarrow{GGGTTN}N$	102.68	DR:3
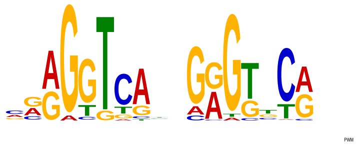	$NN\overrightarrow{NGGTCA}NN\overrightarrow{GGGTGC}NNN$	77.47	DR:2

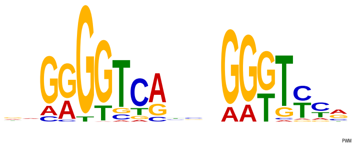	$NNG\overrightarrow{GGGTCN}NNN\overrightarrow{GGGTTN}N$	60.96	DR:3
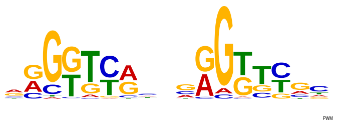	$NN\overrightarrow{NGGTCA}TN\overrightarrow{GGGTTN}NN$	64.98	DR:2
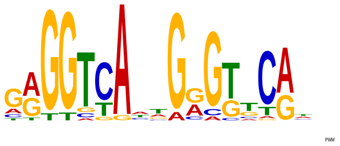	$NN\overrightarrow{NGGTCA}NNG\overrightarrow{GGGGCN}NN$	37.19	DR:3
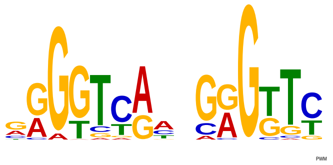	$NG\overrightarrow{GGGTCN}NA\overrightarrow{GGGTTN}$	90.07	DR:2
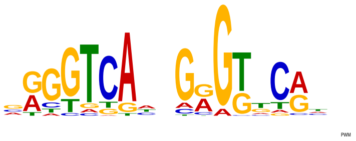	$N\overrightarrow{NGGTCA}NNN\overrightarrow{NGGGCA}NN$	14.59	DR:3
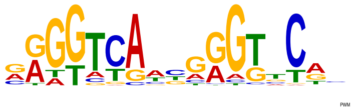	$N\overrightarrow{NGGTCA}NN\overrightarrow{NGGTAC}NN$	15.08	DR:2

39 VDR+10 Round 2

PWM	Seed Sequence	Seed Seq Enrichment	Repeat
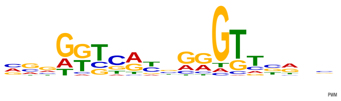	*NNNNGGTCANNGGGTTNNNN*	8.70	DR:2
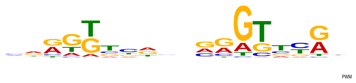	*NNAGGTCNNNNGGTTCNN*	6.48	DR:2
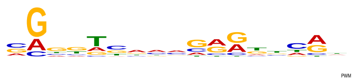	*NNGGTCANNNAGTGCNN*	3.50	DR:3
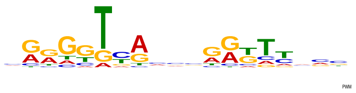	*NNNGGTCANNNGGGTTNN*	5.65	DR:3
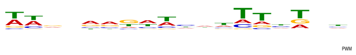	*NNNNAAGATCATTTNNNN*	3.69	M
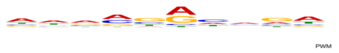	*AAAAGACAGA*	3.44	M
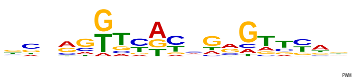	*NNNAGGTCNNNGAGTTNNN*	5.63	DR:3

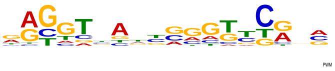	*NNNGGTTANNNGGTTCNNN*	5.75	DR:3
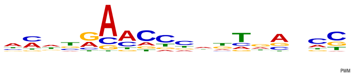	*NNNNGAACCCATTGNNNN*	5.24	M
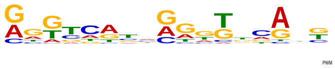	*NNNGTCATNNGGGGCNNN*	2.88	DR:3
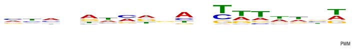	*NNNNATCAGAGTTTNNNN*	3.10	DR:1
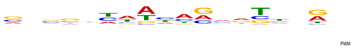	*NNGGTCANNNNGGGTCNN*	1.63	DR:4
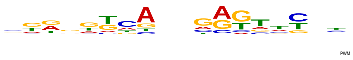	*NNNGGGCANNGAGTTNNN*	3.74	DR:3
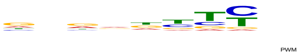	*AGGGAGTTCA*	2.25	M

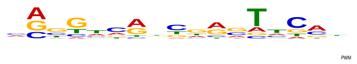	NNNGTCGGNGAGTTNNN	2.85	DR:3
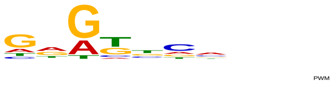	GAGTTCATTG	3.77	M
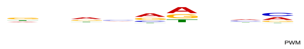	TGGAGAAGAA	2.01	M
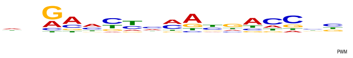	NNNACCTCNACGACNNN	2.54	ER:2
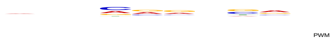	AAAAGGTCAA	2.04	M
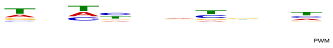	TATTTGTGGT	2.20	M

40 FXR Round 3

PWM	Seed Sequence	Seed Seq Enrichment	Repeat
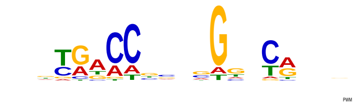	NNNTGACCNNNNGGGCANNN	46.92	ER:2
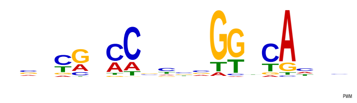	NNNCGACCNNNNGGTCANNN	41.10	ER:2
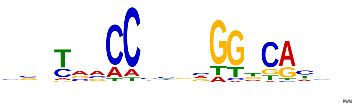	NNNTAACCNNNNGGTCANNN	40.88	ER:2
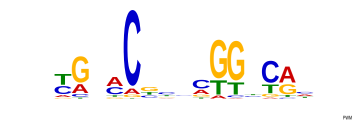	NNNTGAACNNNNGGTCANNN	30.04	ER:2
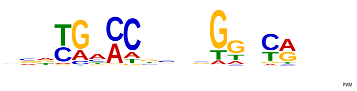	NNNTGACCNNNNGGCCANNN	35.42	ER:2
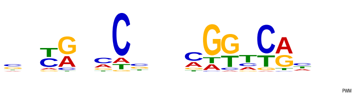	NNNGCCCTNNNGGTCANNN	23.54	ER:2
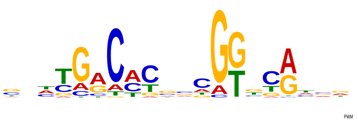	NNNTGACACNNNGGTCANNN	41.74	ER:2

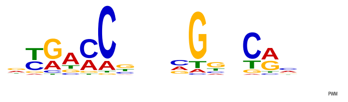	*NNNGACCTNNNGGGCANNN*	23.74	ER:2
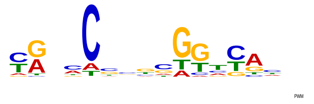	*NNATGCCCNNNNGGTCANN*	47.95	ER:2
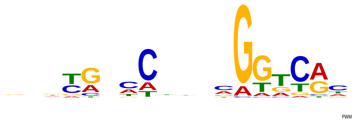	*NNATGGCCNNNCGGTCNN*	36.73	ER:2
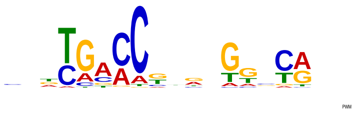	*NNTTGACCNNNNGGCCANN*	70.64	ER:2
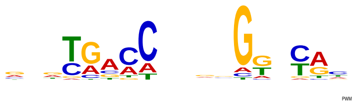	*NNTTGACCNNNCGGGCNN*	34.23	ER:2
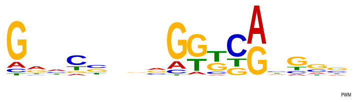	*NNNCTAGCGGTCAANNN*	13.59	M
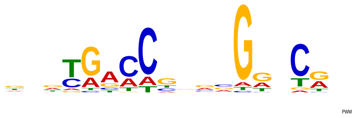	*NNATGACCNNNCGGGCNN*	45.38	ER:2

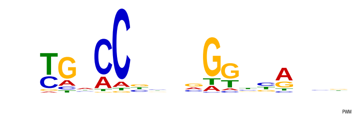	*NNNGACCGNNNGGTTANNN*	22.93	ER:2
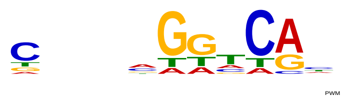	*CTAGCGGTCAT*	17.91	M
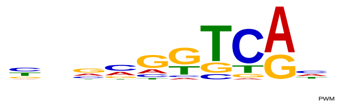	*TAGCGGTCAA*	9.49	M
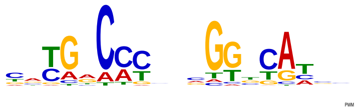	*NNNGACACCNNGGTCANNN*	26.08	DR:3
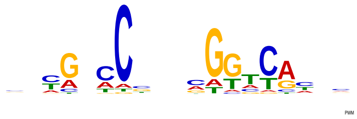	*NNNGCCCGNNNGGTCANNN*	35.13	ER:2
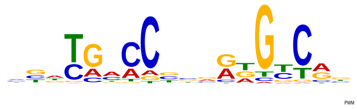	*NNTTGACCNNNGTGTCNN*	24.24	ER:2

41 FXR+11 Round 3

PWM	Seed Sequence	Seed Seq Enrichment	Repeat
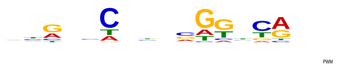	*NNCGCCCNNNNGGTCANN*	10.00	ER:2
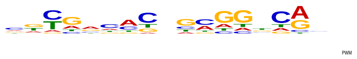	*NNNGACACNNCGGTCNNN*	7.44	DR:3
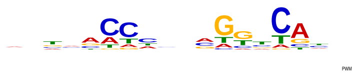	*NNTCACCNNNNGGTCANN*	7.98	ER:2
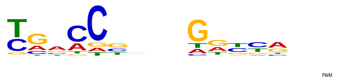	*NNGACCGNNNGGCTANN*	8.07	ER:2
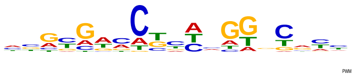	*NNNCGACCNNNCGGGCNNN*	7.46	ER:2
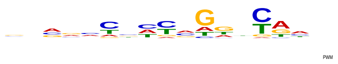	*NNNCACCTNNCGGTCNNN*	3.11	ER:2
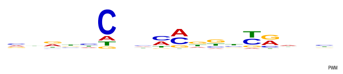	*NNNGCCCGNNNGGTCANNN*	6.89	ER:2

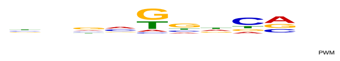	TAGCGGTCAT	3.58	M
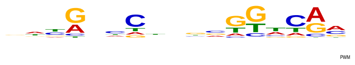	NNCGCCCNNNGGGTCNN	5.70	ER:2
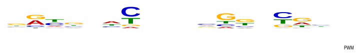	NNNGAACGNNCGGTCNNN	5.75	ER:2
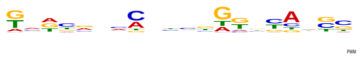	NNNCGACCNNNNGGTCANNN	8.30	ER:2
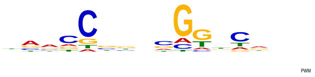	NNNAACCGNNCGGTCNNN	7.76	ER:2
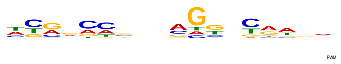	NNNGACCCNNNGTTCANNN	6.36	ER:2
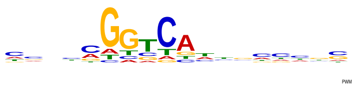	NNNNCGGTCATTGCNNNN	9.36	M

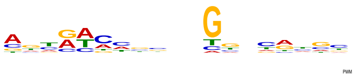	$NN\overleftarrow{NGACCC}NN\overrightarrow{NGGGCA}NNN$	7.28	ER:2
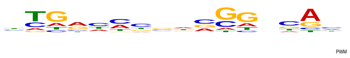	$\overrightarrow{NNGGCA}CNN\overrightarrow{CGGTCN}N$	4.94	DR:3
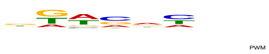	$GC\overrightarrow{GGTCA}TCG$	3.82	M
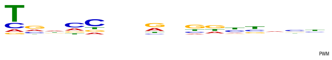	$N\overleftarrow{NGACCG}NN\overrightarrow{NGGTGA}NN$	4.50	ER:2
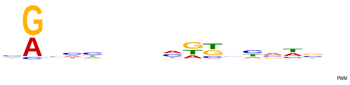	$NNNTCGCG\overrightarrow{NGGTCA}NNN$	3.58	M
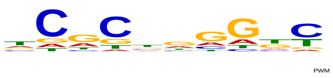	$TCGCT\overrightarrow{GGGTC}$	5.08	M

42 LXRA Round 3

PWM	Seed Sequence	Seed Seq Enrichment	Repeat
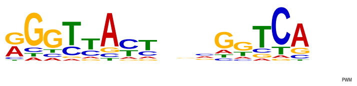	*NNGTTACNNNNGGTCANN*	7.07	DR:4
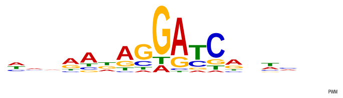	*NNNNAAGAGNATCAANNNN*	5.92	M
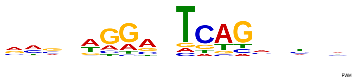	*NNNNAGGACTCAGANNNN*	5.10	M
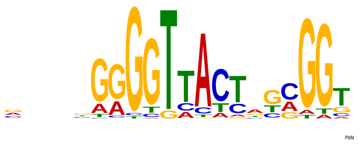	*NNNNNGGGGTTACTANNNNN*	34.49	M
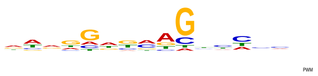	*NNNNAGGAGAAGTTNNNN*	3.46	DR:0
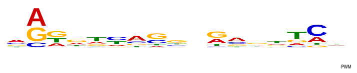	*NNGGTCANNNGAGTTNN*	3.00	DR:4
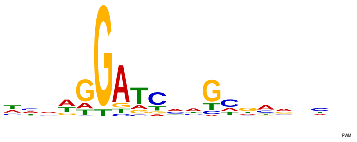	*NNNNGGATCNAGCGANNNN*	7.44	M

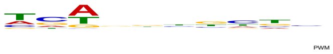	*TCAGTCGCTC*	2.70	M
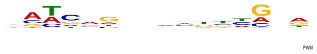	*NNNATCAGNNAATTCNNN*	3.03	DR:3
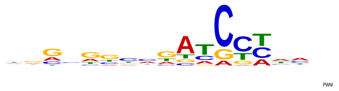	*NNNNCGGCCGATCCNNNN*	4.14	M
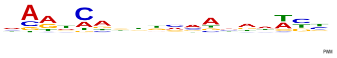	*NNNATCAGNNNAAAAANNN*	4.28	M
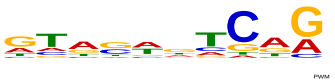	*GTAGAGTCAG*	4.42	M
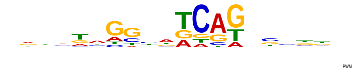	*NNNNNAGGTATCAGGNNNNN*	4.24	DR:1
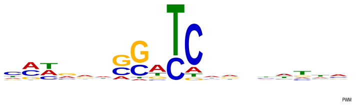	*NNNNAAGGANCAACTNNNN*	4.05	M

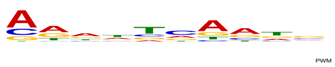	*AAATTCAATG*	3.03	M
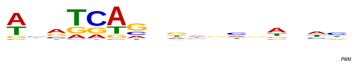	*NNATCAGNNNGCGACNN*	3.15	IR:5
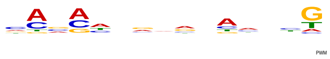	*NNNGAATGAAAAANNN*	2.28	M
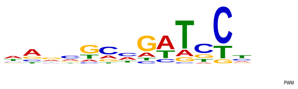	*NNNNAGCGCCGATCNNNN*	3.73	M
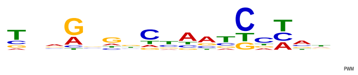	*NNNNCGTCTAATCCNNNN*	3.58	M
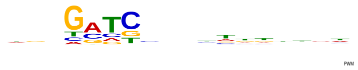	*NNAGATCNNNNTTTTTNN*	4.73	M

43 LXRA+12 Round 3

PWM	Seed Sequence	Seed Seq Enrichment	Repeat
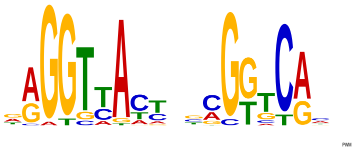	NNGGTTACTNNNGGTCANN	85.68	DR:4
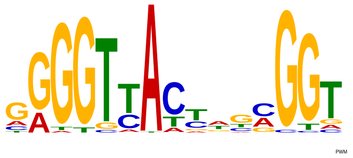	GGGGTTACNNGCGGT	244.70	DR:4
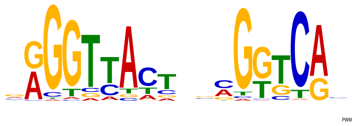	NNGGTTACNNNAGGTCNN	28.64	DR:4
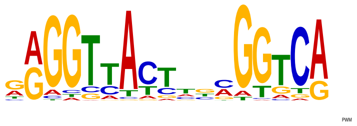	NNGGTTACNNNCGGTCNN	34.70	DR:4
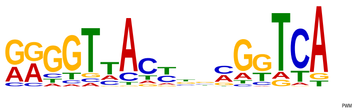	NNGGTTACNNNGGGTCNN	12.69	DR:4
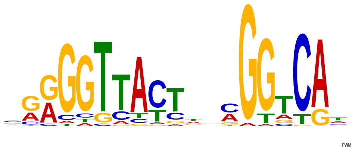	NNGGGTTANNNNCGGTCNN	23.92	DR:4
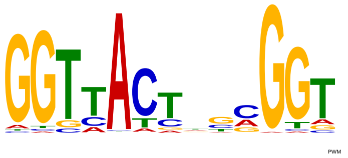	GGTTACTNGCGGT	106.17	DR:4

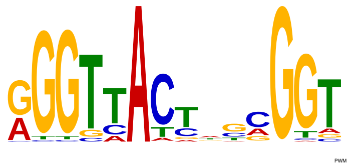	GGGTTACTNGCGGT	261.76	DR:4
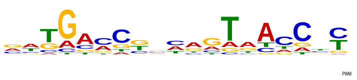	NNNGACCCNNNGTAACNNN	4.86	ER:1
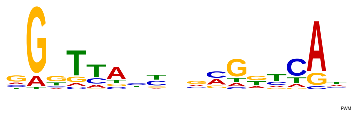	NNGTCACNNNAGGTCNN	3.77	DR:4
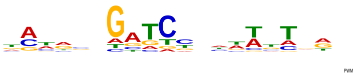	NNNNNAGATCCTTTTNNNNN	3.39	M
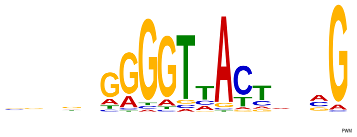	NNNNCGGGGTTACTNNNN	16.54	M
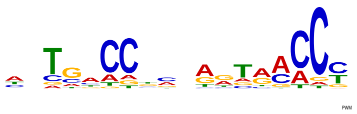	NNNGACCGNNAGTAANNN	5.49	ER:1
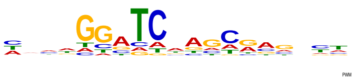	NNNNGGATCNAGCGANNNN	5.48	M

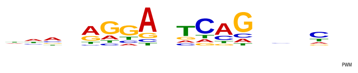	NNNNAGGACTCAGANNNN	4.15	M
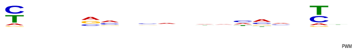	NNAGGATNNNNAAAAANN	2.49	M
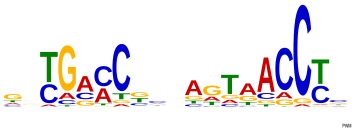	NNNGACCTNNNGTAACNNN	8.46	ER:1
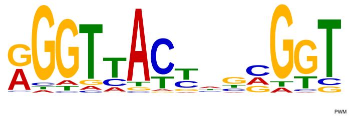	AGGTTACNNGGGGT	30.24	DR:4
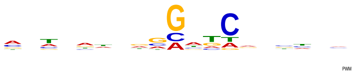	NNNNAAAAGNATCAANNNN	3.73	M
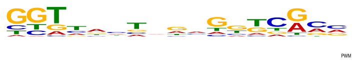	NNNTACTANAGGTCNNN	3.07	M

44 LXRB:RXRA Round 3

PWM	Seed Sequence	Seed Seq Enrichment	Repeat
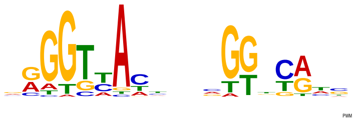	*NNGGTTACNNNNGGTCANN*	446.58	DR:4
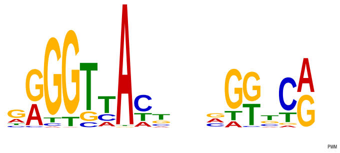	*NNGGGTTACNNNGGGTCNN*	733.76	DR:4
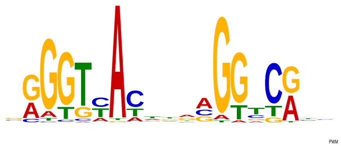	*NNNGGTCACNNNAGGTCNNN*	385.91	DR:4
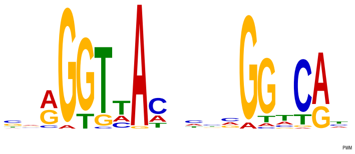	*NNAGGTTACNNNCGGTCNN*	696.27	DR:4
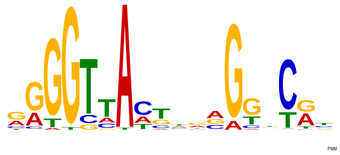	*NNGGGTTACNNNAGGGCNN*	585.53	DR:4
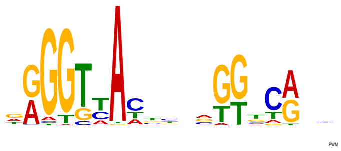	*NNNGGTTACNNNGGGTCNNN*	317.59	DR:4
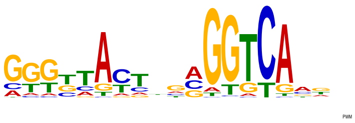	*NNNCTACTNNNGGTCANNN*	31.77	M

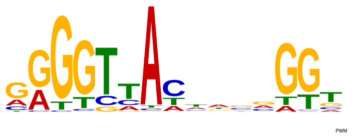	*GGGGTTACNNCGGGT*	1037.35	DR:4
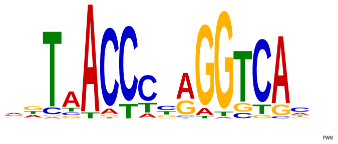	*NNNTAACCCNAGGTCNNN*	126.60	ER:1
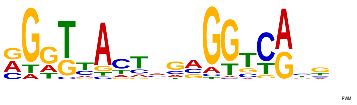	*NNNNTACTANAGGTCNNNN*	40.10	M
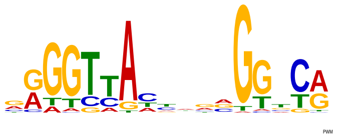	*NGGGGTTANNNNCGGTCN*	491.60	DR:4
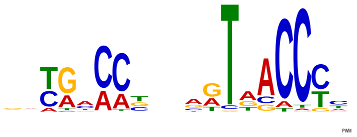	*NNNGACCCNNNGTAACNNN*	92.87	ER:1
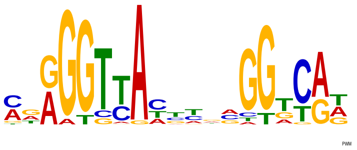	*NNGGGTTAANNNCGGTCNN*	290.49	DR:4
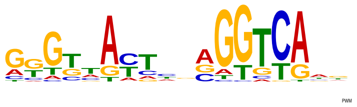	*NNNCTACTNNAGGTCNNN*	20.45	M

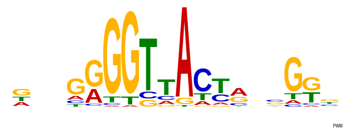	*NNNNGGGGTTACTATNNNN*	392.00	M
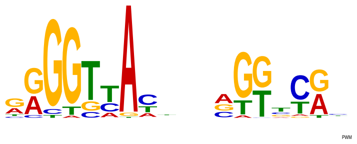	*NNGGTTACNNGGGGTCNN*	442.79	DR:4
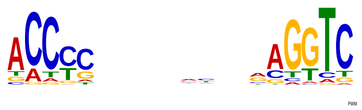	*ACCCCNNNNNNNNNNAGGTC*	91.09	ER:11
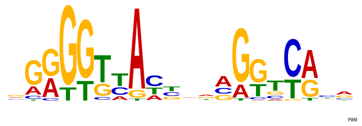	*NNNGGTCACNNNGGGTCNNN*	201.52	DR:4
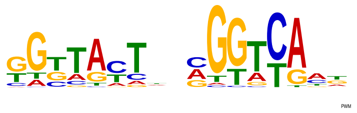	*NNCTACTANNGGTCANN*	39.41	M
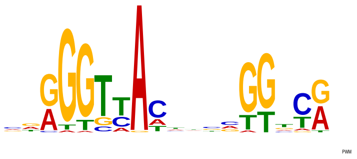	*NNAGGTTACNNNGGGTCNN*	478.08	DR:4

45 PPARD Round 3

PWM	Seed Sequence	Seed Seq Enrichment	Repeat
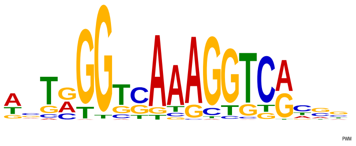	NNNNGGTCAAAGGTCNNNN	46.04	DR:1
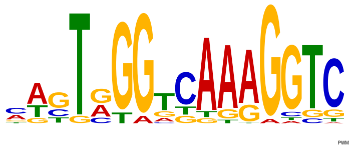	NAGTGGGNCAAAGGTN	96.27	DR:1
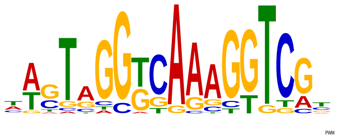	NNAGTAGNNNAAAGGTCNN	44.39	DR:1
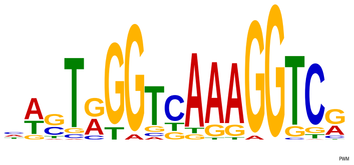	NAGTGGGNNAAAGGTCN	121.80	DR:1
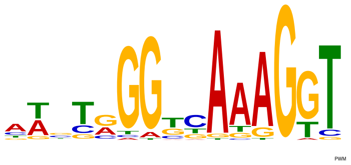	ATGTGGGNNAAAGGT	97.20	DR:5
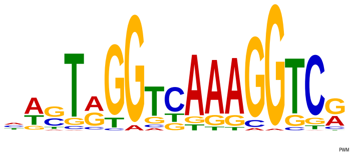	NAGTGGNNNAAAGGTCN	40.69	M
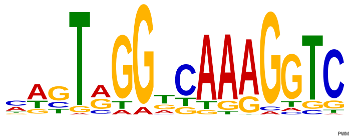	NAGTGGNNCAAAGGTN	30.64	DR:1

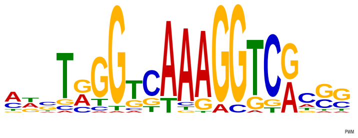	*NNNNGGGTCNAAGGTCNNNN*	38.38	DR:1
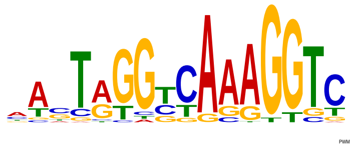	*NACTAGGNNAAAGGTN*	34.19	DR:1
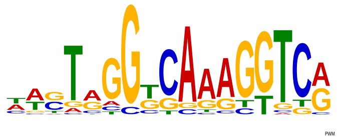	*NNAGTAGNNNAAAGGTNN*	11.74	DR:1
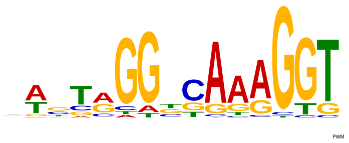	*AAGTAGNNNAAAGGT*	35.26	DR:5
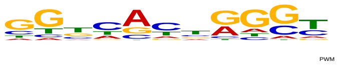	*GGTCACTGGGT*	5.60	DR:2
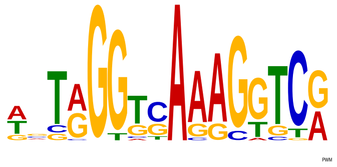	*NNGTAGGTGAAAGGTNN*	50.50	DR:1
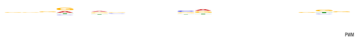	*GGGGTNNNNNNNNNNGGGGG*	4.07	M

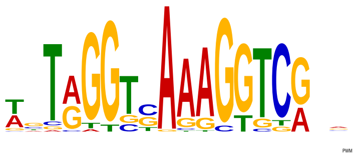	*NNNAGGTGAAAGGTCNNN*	41.06	DR:1
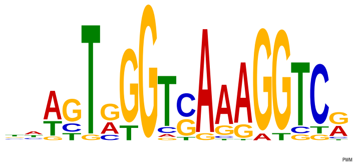	*NNAGTGGGTNAAAGGTNN*	86.65	DR:1
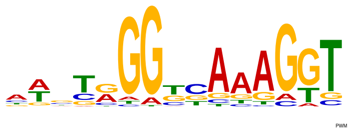	*ATGTGNNNNAAAGGT*	11.59	DR:5
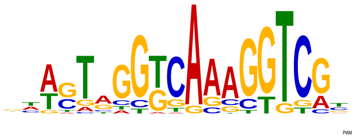	*NNAGTAGNNNNAAGGTCNN*	13.43	M
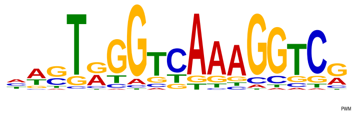	*NAGTGGNNNNAAGGTCN*	13.93	M
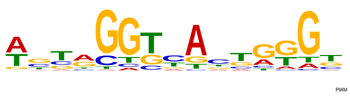	*NGTAGGNNACTGGGN*	6.52	M

46 PPARD+13 Round 3

PWM	Seed Sequence	Seed Seq Enrichment	Repeat
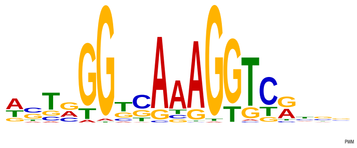	NNNNGGTCAAAGGTCNNNN	68.97	DR:1
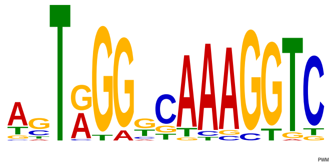	NAGTGGNGCAAAGGTN	77.12	DR:1
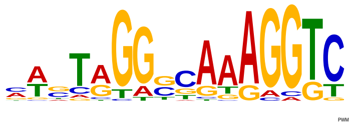	NACTAGNNCAAAGGTN	53.03	DR:1
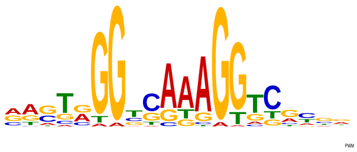	NNNNAGGTCNAAGGTCNNNN	43.93	DR:1
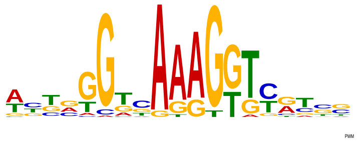	NNNGGGTCNAAGGTCANNN	50.50	DR:1
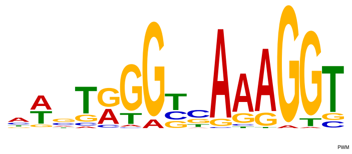	ATGTGGGNNAAAGGT	108.65	DR:5
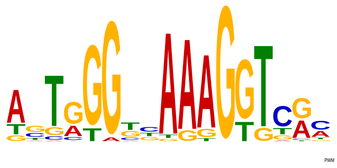	NAGTGGNNNAAAGGTCAN	85.15	M

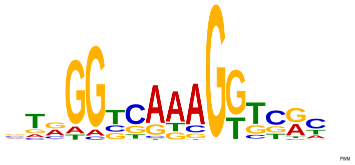	NNAGGTCNAAGGTCANN	43.06	DR:1
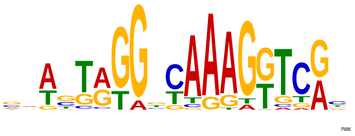	NNAGTGGNNNAAAGGTCNN	40.33	M
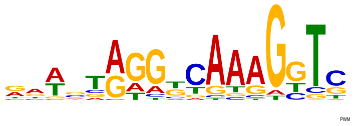	NATGTANNNNAAAGGTN	8.96	DR:5
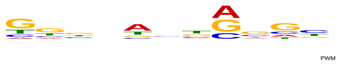	GGTCACTGGGT	3.34	DR:2
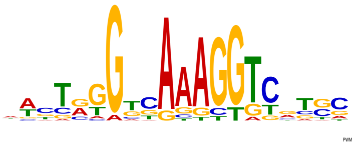	NNNNGGGTCNAAGGTCNNNN	44.60	DR:1
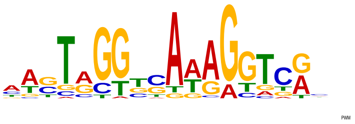	NNNTAGGTCNNGGGTCNNN	10.34	DR:1
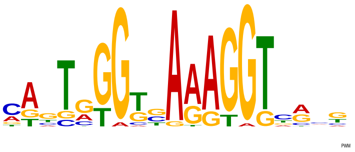	NNGTGGGTNAAAGGTCANN	262.53	DR:1

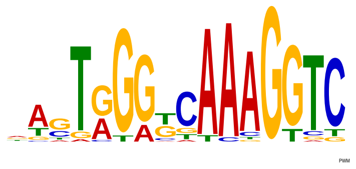	$NAGT\overrightarrow{GGGGCN}A\overrightarrow{AGGTN}$	73.37	DR:1
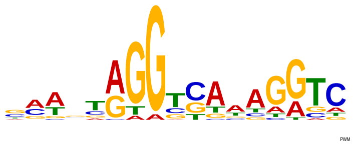	$N\overrightarrow{ATGTGN}NNNNAA\overrightarrow{AGGTN}$	6.11	DR:5
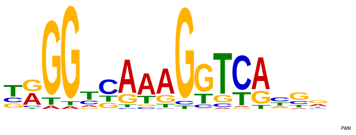	$NN\overrightarrow{NGTCA}A\overrightarrow{ANGTCA}CNNN$	15.90	DR:1
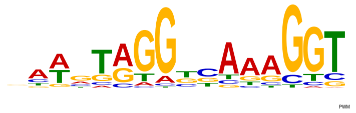	$G\overrightarrow{ATGTNN}NNNNAA\overrightarrow{AGGT}$	13.72	DR:5
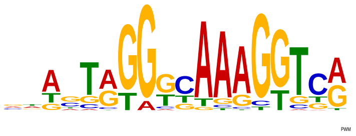	$NNAGT\overrightarrow{GGNNCA}A\overrightarrow{AGGTNN}$	42.48	DR:1
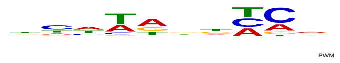	$CTAA\overrightarrow{AGGTCA}$	1.54	M

47 PPARD+14 Round 3

PWM	Seed Sequence	Seed Seq Enrichment	Repeat
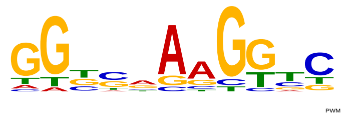	*GGTCAAAGGTC*	12.82	DR:1
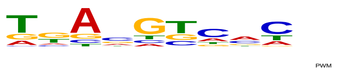	*TGACGTCACG*	3.58	M
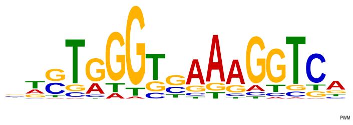	*NNGTGGGTNAAAGGTNN*	19.40	DR:1
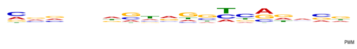	*NNNNAACTAGGTCANNNN*	2.86	ER:0
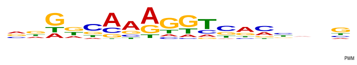	*NNNNCAAAGGTCACNNNN*	4.81	M
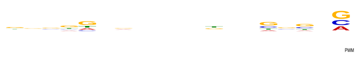	*GGGGGNNNNNNNNNGGGAG*	3.03	M
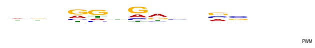	*NNNGGGGTNACTGGNNN*	2.34	M

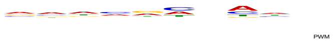	AAACACAAAC	2.22	M
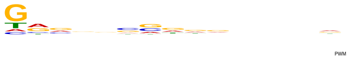	NNGGGCGNGGAAANN	2.64	DR:0
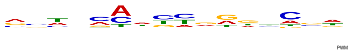	NNTACAANNGGGTCNN	2.19	M
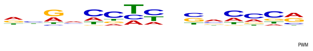	NNACGACNNNTCACCNN	2.73	IR:2
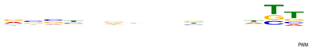	NNGGGTCNNNTGTGTNN	1.96	M
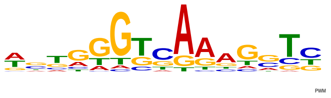	NAGTGGGNNNAAGGTN	5.28	M
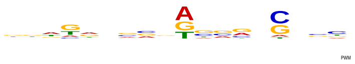	NNNGGGCANNGCGGGNNN	2.07	M

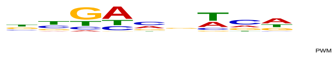	GTGACGTCAT	2.70	M
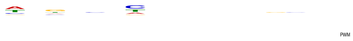	NCGTCANNNNNCCCCCN	2.61	M
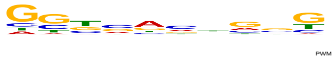	GGTCACTGGG	3.12	M
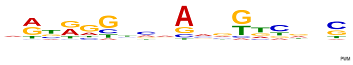	NNNAGGTCNNAGGTCNNN	3.76	DR:1
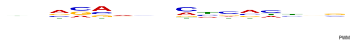	NNACAACNNTCACTNN	2.28	M
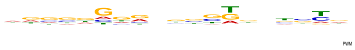	NGGGGGNNNNNNNGTTGTN	3.00	M

48 PPARG Round 3

PWM	Seed Sequence	Seed Seq Enrichment	Repeat
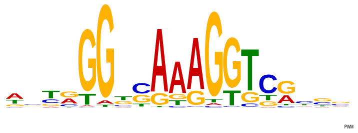	NNNNGGTCAAAGGTCNNNN	156.40	DR:1
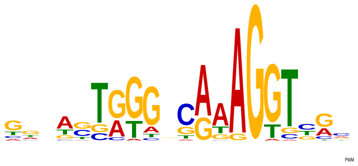	NNNAGTGGGNCAAAGGTNNN	518.37	DR:1
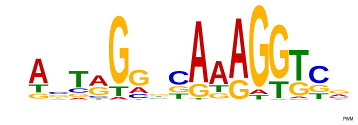	NACTAGNNCAAAGGTCN	372.93	DR:1
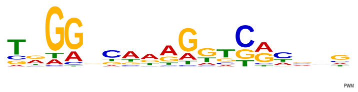	NNNNCTAAAGGTCANNNN	4.06	M
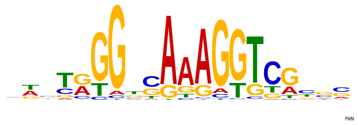	NNNNGGGTCNAAGGTCNNNN	115.55	DR:1
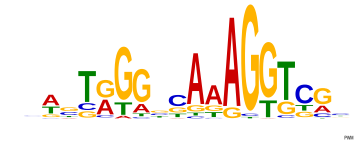	NNAGTGGNNNAAAGGTCNN	146.03	M
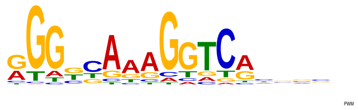	NNNNTAAAGGTCATNNNN	9.82	M

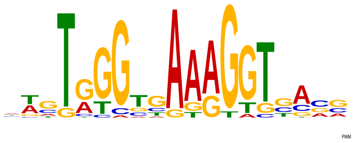	*NNGTGGGTNAAAGGTGANN*	534.59	DR:1
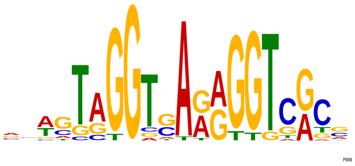	*NNNGTAGGTGANGGGTCNNN*	148.48	DR:1
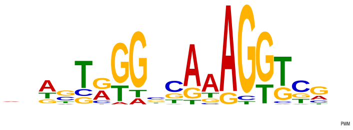	*NNAGTGGNNCAAAGGTCNN*	328.80	DR:1
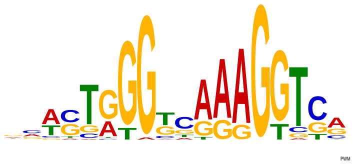	*NNACTGGGTCNAAGGTNN*	294.72	DR:1
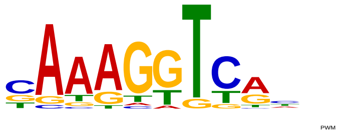	*TAAAGGTCACG*	17.17	M
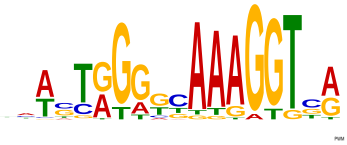	*NNACTGGNGCAAAGGTNN*	252.77	DR:1
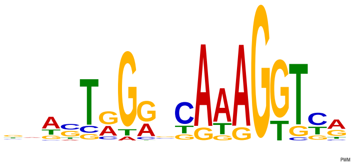	*NNAGTGGNGCAAAGGTNN*	251.11	DR:1

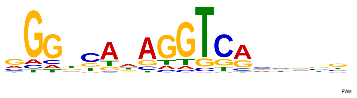	*NNNNTAAAGGTCAANNNN*	8.52	M
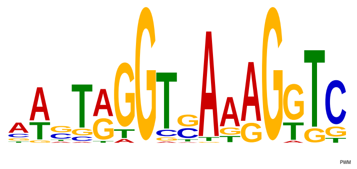	*NAGTGGGTGAGAGGTN*	154.42	DR:1
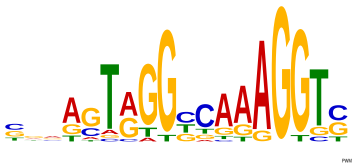	*NGGAGTNNNNCAAAGGTN*	61.04	M
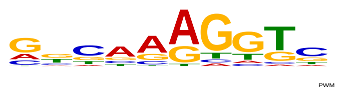	*GCTAAAGGTC*	6.78	DR:0
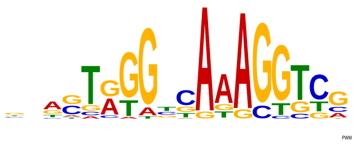	*NGAGTGNNNNAAAGGTCN*	54.53	M
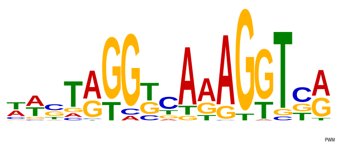	*NNAGTGGGNCAAGGGTNN*	87.52	DR:1

49 PPARG+14 Round 3

PWM	Seed Sequence	Seed Seq Enrichment	Repeat
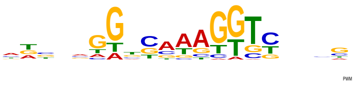	NNNNNGGTCAAAGGTNNNNN	7.66	DR:1
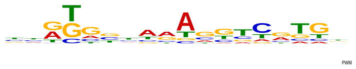	NNNNGCTAAAGGTCNNNN	3.71	DR:0
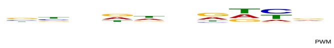	CTAAAGGTCA	1.77	M
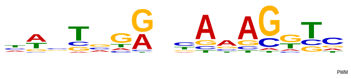	NAGTGGNNNAAAGGTN	7.37	M
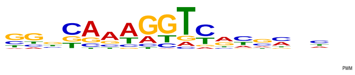	NNNNAAAGGTCACGNNNN	5.16	M
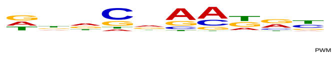	GTACAAAGGT	2.57	DR:1
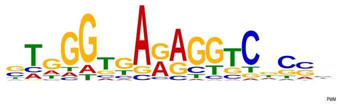	NNNNGGTGAGAGGTCNNNN	10.28	DR:1

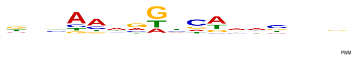	*NNNAAAGGNCAAACNNN*	3.63	M
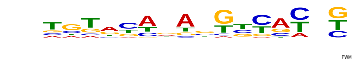	*NNNGTACANNGGTCANNN*	3.29	DR:1
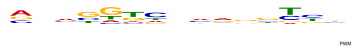	*NNAGGTCNCAGGTNN*	3.09	DR:1
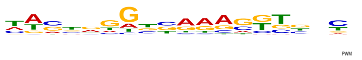	*NNNTGGGTNNAAGGTNNN*	4.24	DR:1
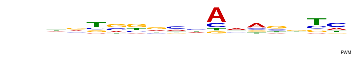	*NATGTGNNNNNAAGGTN*	2.59	DR:5
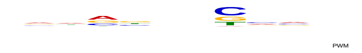	*AAAGGTGAAC*	1.79	M
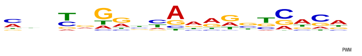	*NNNNGGGGCNAAGGTNNNN*	3.35	DR:0

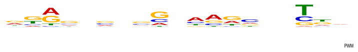	NNNNGG$\overrightarrow{GGGNAA}$GCGNNNN	2.57	M
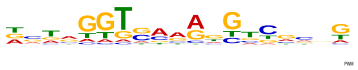	NNN$\overrightarrow{NGGTGA}$N$\overrightarrow{GGGTCN}$NNN	4.21	DR:1
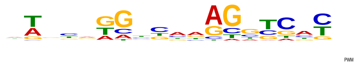	NNNN$\overrightarrow{GGGTCN}$A$\overrightarrow{AGGTNN}$NN	4.25	DR:1
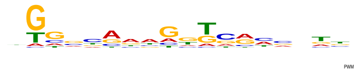	NNNNCAA$\overrightarrow{AGGTCA}$ANNNNN	4.70	M
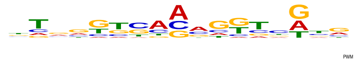	NN$\overrightarrow{NGGTGA}$N$\overrightarrow{AGTTCN}$NN	3.73	DR:1
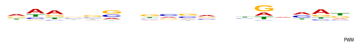	NNNGG$\overrightarrow{GGGNNA}$A$\overleftarrow{TGANN}$N	2.02	IR:1

50 PPARG+15 Round 3

PWM	Seed Sequence	Seed Seq Enrichment	Repeat
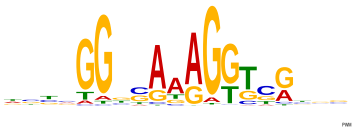	*NNNNGGTCAAAGGTCNNNN*	140.80	DR:1
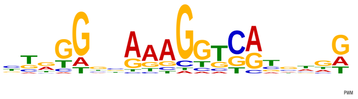	*NNNNNCTAAAGGTCANNNNN*	9.47	M
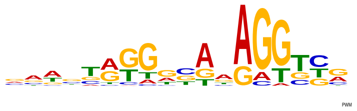	*NNACTGGGNCAAAGGTNN*	259.71	DR:1
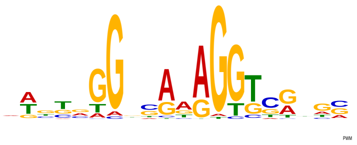	*NNNNGGGTCNAAGGTCNNNN*	88.75	DR:1
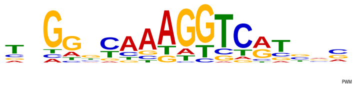	*NNNNCTAAAGGTCGNNNN*	5.50	M
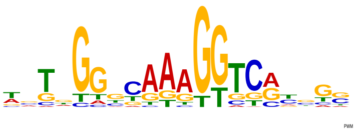	*NNNNNGCTAAAGGTCNNNNN*	11.07	DR:0
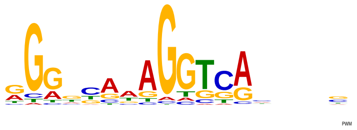	*NNNNTAAAGGTCAANNNN*	12.99	M

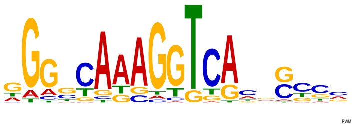	*NNNNTAAAGGTCACGNNNN*	24.77	M
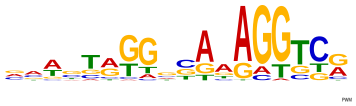	*NNACTGGNNCAAAGGTNN*	98.37	DR:1
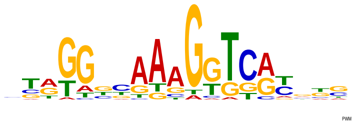	*NNNNGCTAAAGGTCANNNN*	20.83	DR:0
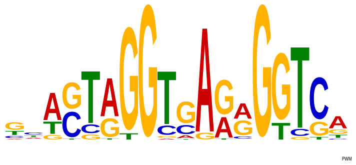	*NNAGTAGGTGAGGGGTNN*	249.56	DR:1
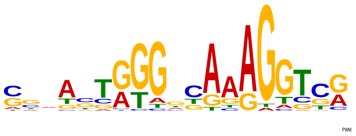	*NNNACTGGGNCAAAGGNNN*	133.06	DR:1
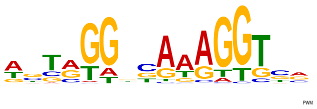	*NNAGTGGNNCAAAGGTNN*	128.89	DR:1
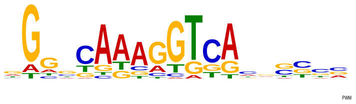	*NNNNTAAAGNTCACGNNNN*	9.15	M

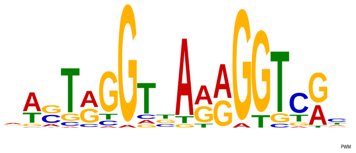	$NNGT\overrightarrow{GGGTNA}G\overrightarrow{GGGTCN}N$	116.30	DR:1
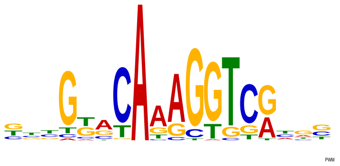	$NNN\overrightarrow{NGTACA}A\overrightarrow{AGGTCN}NNN$	46.95	DR:1
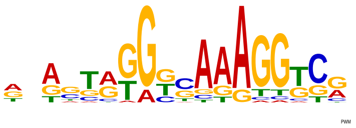	$NGAGTGNNNNAA\overrightarrow{AGGTCN}$	65.27	M
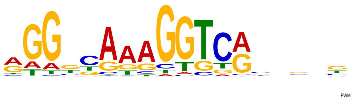	$NNNNTAA\overrightarrow{AGGTCA}TNNNN$	12.46	M
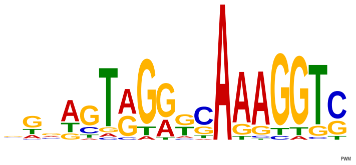	$NGGAGTNNNNCAA\overrightarrow{AGGTN}$	87.30	M
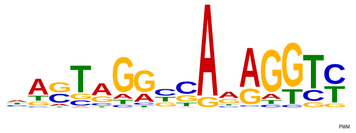	$NACT\overrightarrow{AGNNCA}A\overrightarrow{GGGTN}$	55.56	DR:1

51 Rev-ErbA-Alpha Round 3

PWM	Seed Sequence	Seed Seq Enrichment	Repeat
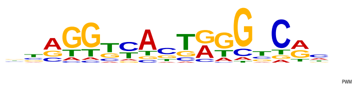	NNNGGTCANTGGGTCNNN	9.67	DR:2
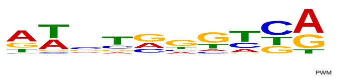	ATCTGGGTCA	4.18	ER:0
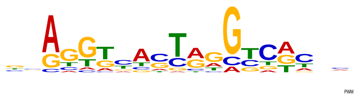	NNNNGTCAGTGGGTCNNN	6.78	DR:2
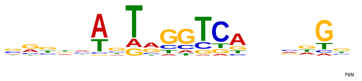	NNNNNAGTAGGTCAANNNNN	7.11	M
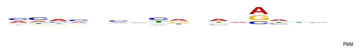	NNACTCANNNAAAATNN	2.42	M
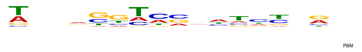	NNNAGGTCNAATCTNNN	2.84	M
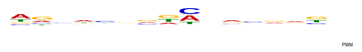	NNCACGGNNGACAANN	2.05	M

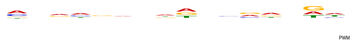	NNAAGAANNCCAACNN	2.07	M
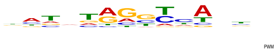	NNNNTATATAGGTCNNNN	3.37	M
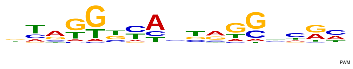	NNGGGTCNNTGGGTCNN	4.31	DR:2
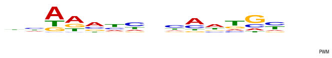	NNNAAATCNCAATGNNN	2.97	M
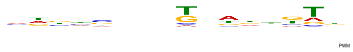	NNGTCTANNGATTGNN	1.78	M
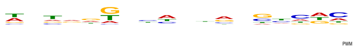	NNNGGGTCNNTGGGTNNN	2.27	DR:2
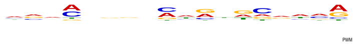	NNAAGGGNNNNTGCAANN	2.29	M

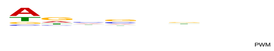	$\overleftarrow{AATCT}\overrightarrow{AGGTC}$	1.22	ER:0
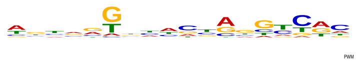	$NNN\overrightarrow{GGGTCA}NT\overrightarrow{GGGTNN}N$	3.27	DR:2
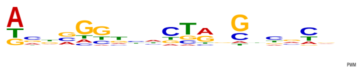	$NNN\overrightarrow{NGGTCA}NN\overrightarrow{GGGTCN}NNN$	3.38	DR:2
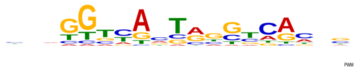	$NN\overrightarrow{NGGTCA}NN\overrightarrow{NGGTCA}NNN$	4.77	DR:2
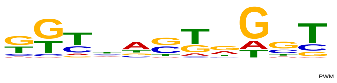	$\overrightarrow{GGTTA}GT\overrightarrow{GGGT}$	3.51	DR:2
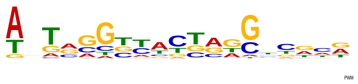	$NNN\overrightarrow{GGGTCA}NN\overrightarrow{GGGTCN}NN$	4.14	DR:2

52 RORC Round 3

PWM	Seed Sequence	Seed Seq Enrichment	Repeat
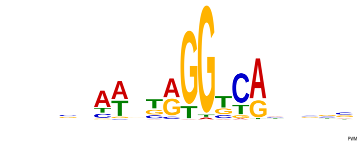	*NNNNNAACTGGGTCANNNNN*	62.64	ER:0
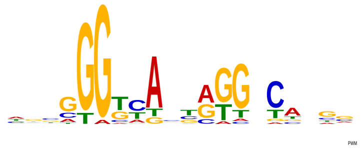	*NNNGGTCACTGGGTCNNN*	214.15	DR:2
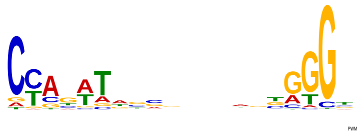	*CCAGATNNNNNNNNNTGGGT*	132.77	DR:8
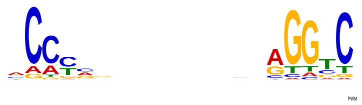	*ACCCCNNNNNNNNNNAGGTC*	77.03	ER:11
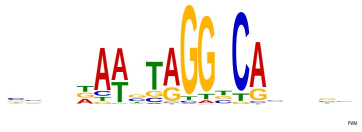	*NNNNNAACTAGGCCANNNNN*	50.50	ER:0
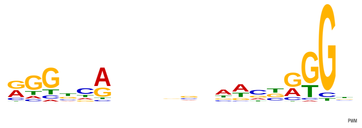	*GGGTCNNNNNNNNNNTGGGT*	50.41	DR:10
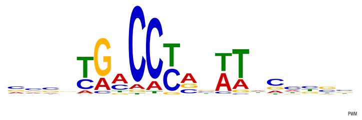	*NNNNNGACCCAGATANNNNN*	53.11	ER:0

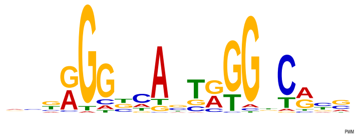	NNNAGGGCANTGGGTCNNN	135.86	DR:2
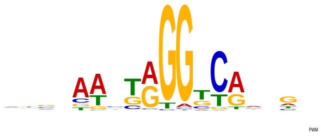	NNNNNGAACTGGGTCNNNNN	54.25	ER:0
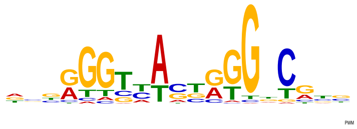	NNNAGGTTANTGGGTCNNN	154.24	DR:2
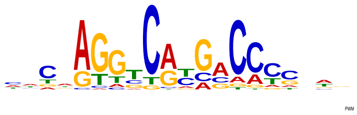	NNNNAGGTCATGACCCNNNN	176.03	IR:0
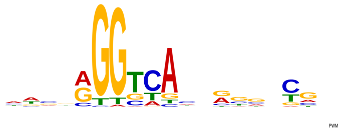	NNNNNAGGTCACTGGNNNNN	36.49	M
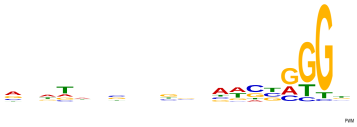	AGATANNNNNNNNNNTGGGT	29.63	DR:10
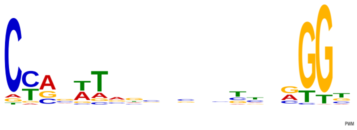	CCAGTTNNNNNNNNNGAGGT	92.53	DR:8

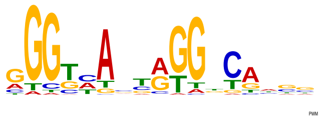	*NNNNGTCACTGGGTCNNNN*	74.70	DR:2
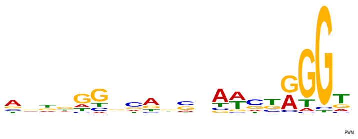	*AGTGGNNNNNNNNNCTGGGT*	62.30	M
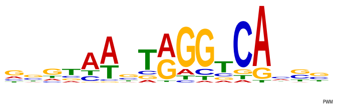	*NNNNCTAGTGGGTCNNNN*	9.62	M
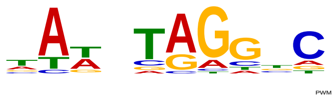	*TATATAGGCC*	19.88	M
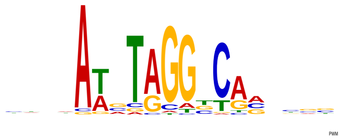	*NNNNNATATAGGCCANNNNN*	27.64	M
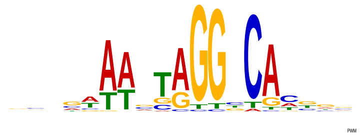	*NNNNTATCTAGGCCANNNN*	63.11	ER:0

53 RORC:RXRA Round 3

PWM	Seed Sequence	Seed Seq Enrichment	Repeat
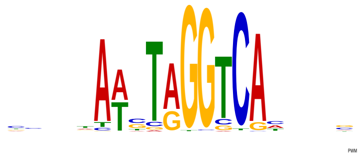	NNNNNAACTAGGTCANNNNN	420.22	ER:0
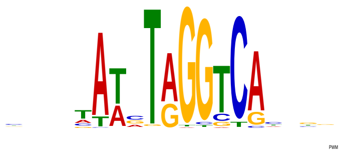	NNNNNTATATAGGTCNNNNN	220.95	M
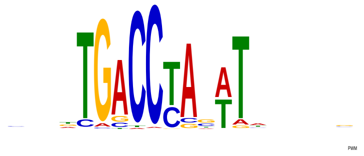	NNNNNGACCTAGTTANNNNN	350.56	ER:0
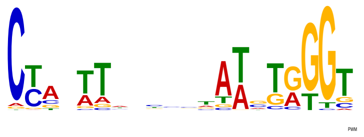	CCAGTTNNNNNNNNNTGGGT	253.73	DR:8
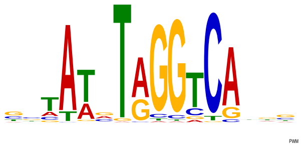	NNNNCTATATAGGTCNNNN	246.02	M
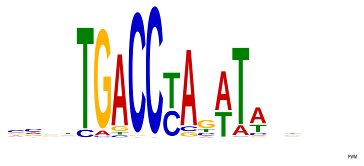	NNNNNGACCCAGATANNNNN	264.94	ER:0
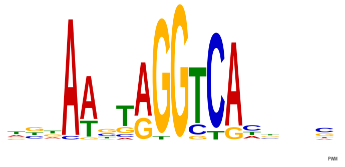	NNNTAATTNGGTCACGNNN	432.81	M

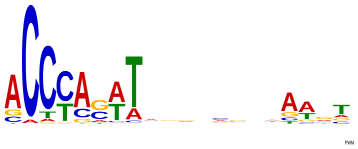	*ACCCANNNNNNNNNNAAAGT*	92.09	ER:12
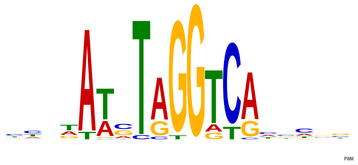	*NNNTATATNGGTCACGNNN*	399.31	M
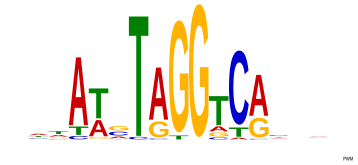	*NNTATATAGNTCACGNN*	293.60	M
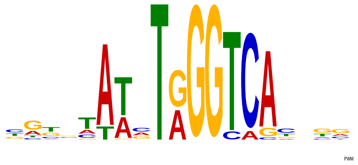	*NNNCTATATNGGTCATNNN*	380.01	M
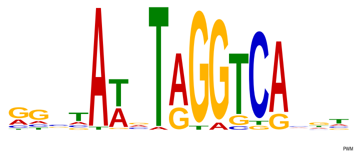	*NNCTATATAGGTCAANN*	817.59	M
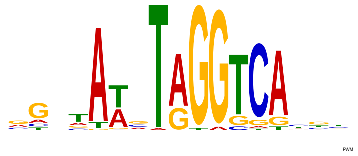	*NNCTATATNGGTCAANN*	312.89	M
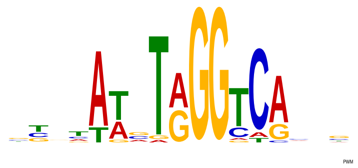	*NNCTATATAGGTCATNN*	660.16	M

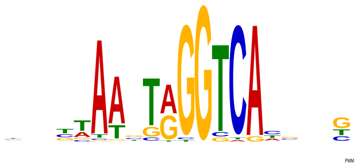	NNNNTAATT$\overrightarrow{NGGTCA}$TNNNN	307.56	M
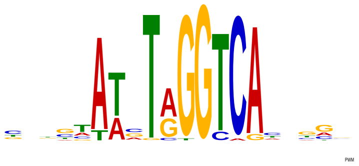	NNNNTATAT$\overrightarrow{NGGTCA}$TNNNN	292.20	M
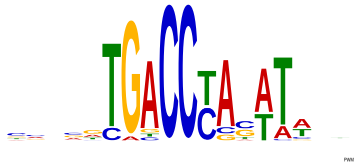	NNCCG$\overleftarrow{TGACC}$N$\overrightarrow{AGATAN}$N	1076.61	ER:0
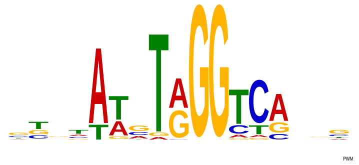	NNCTATAT$\overrightarrow{ANGTCA}$TNN	161.78	M
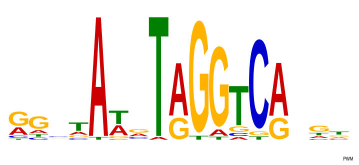	NNCTATAT$\overrightarrow{ANGTCA}$ANN	205.92	M
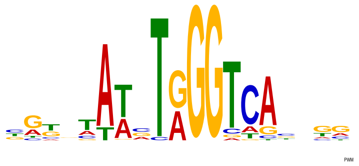	NNNCTATAT$\overrightarrow{NNGTCA}$TNNN	78.11	M

54 RORC+16 Round 3

PWM	Seed Sequence	Seed Seq Enrichment	Repeat
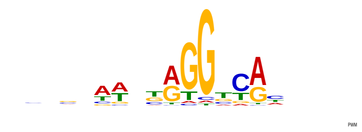	*NNNNNAACTGGGTCANNNNN*	22.80	ER:0
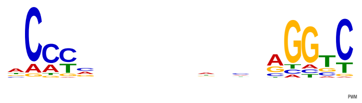	*ACCCCNNNNNNNNNNAGGTC*	37.21	ER:11
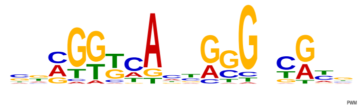	*NNNGGTCACTGGGTCNNN*	74.89	DR:2
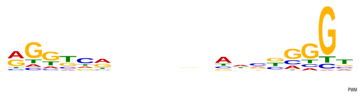	*AGGTCNNNNNNNNNNTGGGT*	22.14	DR:10
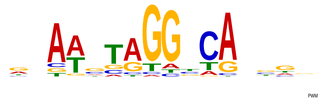	*NNNNAACTAGGCCANNNN*	23.66	ER:0
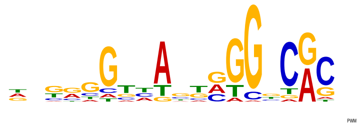	*NNNGGGTTANTGGGTCNNN*	55.94	DR:2
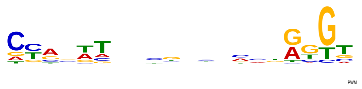	*CCAGTNNNNNNNNNNGGGGT*	16.73	M

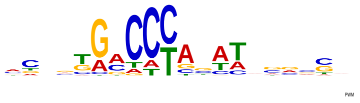	NNNNNGACCCAGATANNNNN	21.94	ER:0
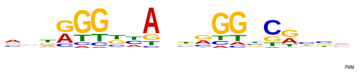	NNNNGGTCANNAGGTCNNNN	18.73	DR:2
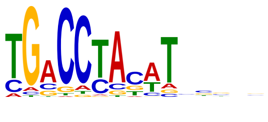	NNNNNGACCTACATCNNNNN	40.76	ER:0
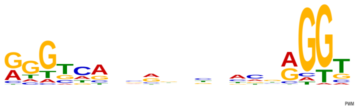	GGGTCNNNNNNNNNNGAGGT	22.92	DR:10
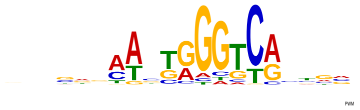	NNNNNGAACTGGGTCNNNNN	22.89	ER:0
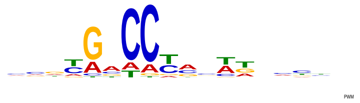	NNNNGACCCAGTTANNNN	13.71	ER:0
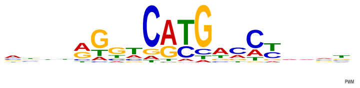	NNNNNGGTCATGACCNNNNN	22.99	IR:0

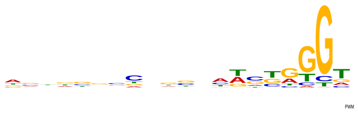	*AGTGGNNNNNNNNNNTGGGT*	14.83	M
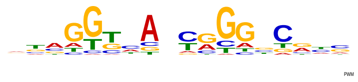	*NNNGGTCACNAGGTCNNN*	24.11	DR:2
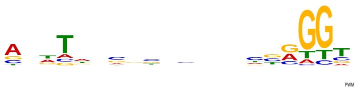	*AGATANNNNNNNNNNGGGGT*	13.27	DR:10
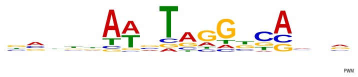	*NNNNTATATAGGCCNNNN*	9.12	M
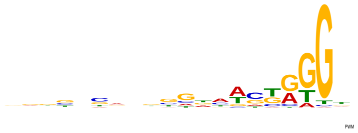	*GGGGTNNNNNNNNNNTGGGT*	13.03	DR:9
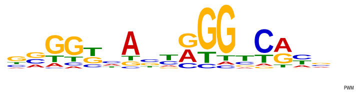	*NNNGTCACTNGGTCANNN*	23.75	DR:2

55 TR4 Round 3

PWM	Seed Sequence	Seed Seq Enrichment	Repeat
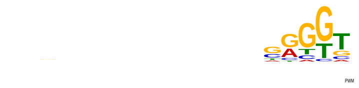	*GGGGGNNNNNNNNNNGGGGT*	34.95	M
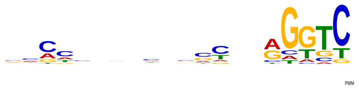	*CCCCCNNNNNNNNNNAGGTC*	22.87	M
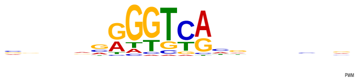	*NNNNNGGGGTCACGGNNNNN*	29.23	M
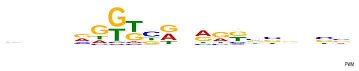	*NNNNGGGTCNNAGGGCNNNN*	20.25	DR:1
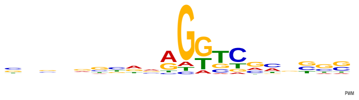	*NNNNNGCAAAGGTCGNNNNN*	20.34	M
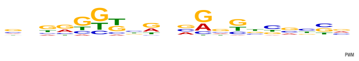	*NNNGGGTCNNNNGGTCGNNN*	14.67	DR:2
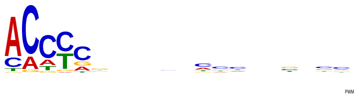	*ACCCCNNNNNNNNNNGGGCC*	16.50	ER:10

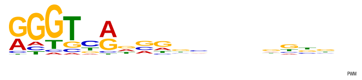	*GGGTCNNNNNNNNNGGTGC*	12.52	DR:7
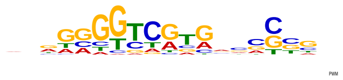	*NNNNNGGGTCGTGACNNNNN*	21.71	IR:0
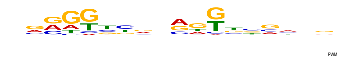	*NNNNGGTCGNGGGTCNNNN*	15.59	DR:1
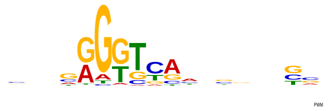	*NNNNNGGGGTCAACGNNNNN*	20.70	M
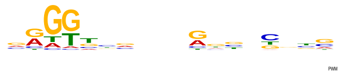	*NNGGGTCNNNNNGGTCGNN*	11.13	DR:3
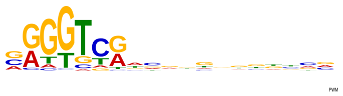	*NNGGGTCNNNNNNGGGGCNN*	16.47	DR:5
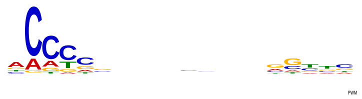	*ACCCCNNNNNNNNNNGGTTC*	23.43	ER:10

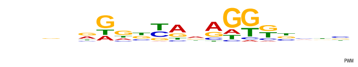	*NNNNGGGGCNAAGGTNNNN*	12.60	DR:0
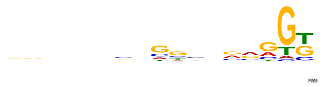	*GGGGGNNNNNNNNNAAGGT*	11.90	M
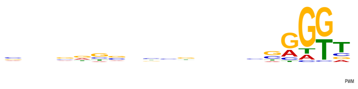	*CCCGGNNNNNNNNNNGGGGT*	21.03	M
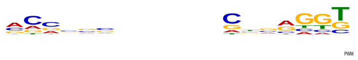	*ACCCGNNNNNNNNNGAGGT*	12.98	ER:11
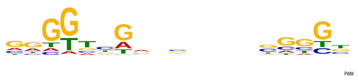	*GGGGTCNNNNNNNNCGGGT*	34.39	DR:8
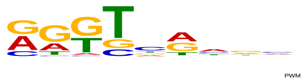	*GGGGTCACCG*	11.72	M

56 TR2 Round 3

PWM	Seed Sequence	Seed Seq Enrichment	Repeat
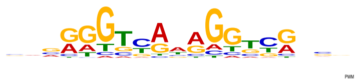	*NNNNGGTCANAGGTCNNNN*	10.97	DR:1
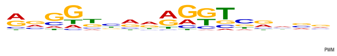	*NNNNGTCAGGGGTCNNNN*	4.96	DR:1
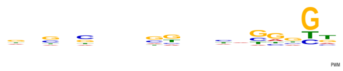	*GGGGTNNNNNNNNNNGGGGT*	5.24	DR:9
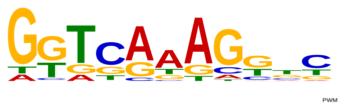	*GGTCAAAGGGC*	14.36	DR:1
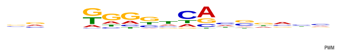	*NNNNCGGGGTCACGNNNN*	4.01	M
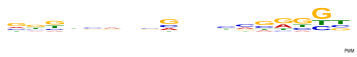	*GGGTCNNNNNNNNGGGGT*	4.38	DR:8
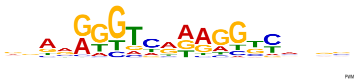	*NNNNAGGTCNNAGGTCNNNN*	8.05	DR:1

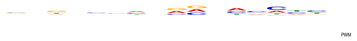	*NCGGCCNNNNNACCTCN*	3.29	IR:2
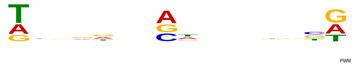	*NNGGGCGNNAGCGGNN*	2.17	M
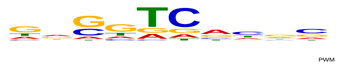	*GAGGTCACGC*	4.56	M
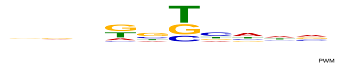	*CGAGGTCAAG*	2.72	M
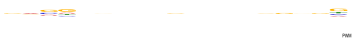	*GAGGTNNNNNNNNNGGGGG*	3.36	M
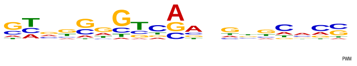	*NNNNGGGTCNAAGTGNNNN*	4.13	DR:0
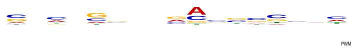	*NNCAGGTNNNNCCCCCNN*	2.89	M

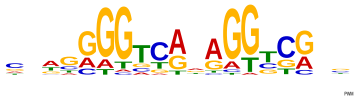	*NNNGGGGTCNNAGGTCNNN*	19.39	DR:1
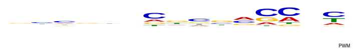	*NNCGTCCNCGACCNN*	3.02	IR:1
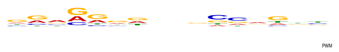	*NNGAGGGNNNGCCAGNN*	3.45	M
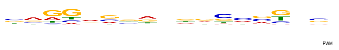	*NNAGGAGNNNNGCCGGNN*	3.31	M
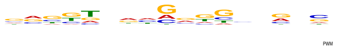	*NNNGGTCANNGGGTCNNN*	2.71	DR:2
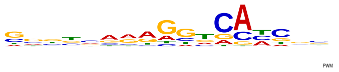	*NNNNCCAAAGGTCANNNN*	3.90	M

57 LRH1 Round 3

PWM	Seed Sequence	Seed Seq Enrichment	Repeat
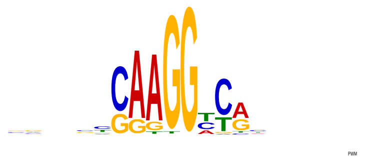	NNNNNCCAAGGTCACNNNNN	151.05	M
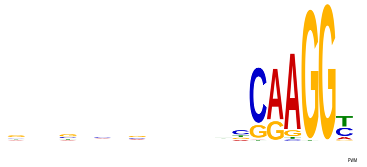	GGGGTNNNNNNNNNCAAGGT	188.04	DR:9
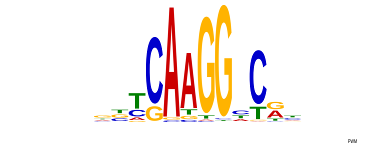	NNNNNGTTCAAGGCCNNNNN	103.46	DR:0
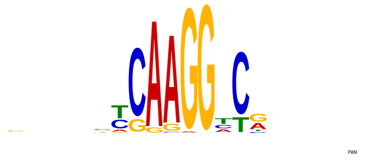	NNNNNCTCAAGGTCGNNNNN	131.40	DR:0
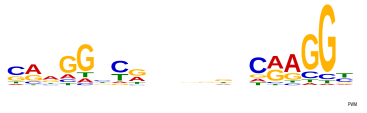	CAAGGNNNNNNNNNCGAGGT	143.62	DR:8
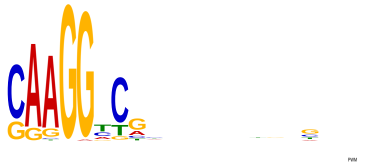	CAAGGTCNNNNNNNNGTGGG	220.69	M
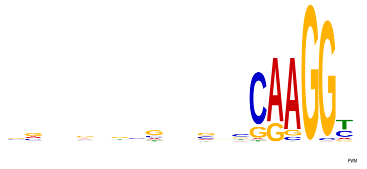	CGGAGNNNNNNNNNCAAGGT	133.30	M

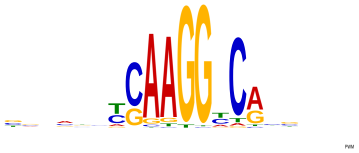	*NNNNNTCCAAGGCCANNNNN*	84.40	M
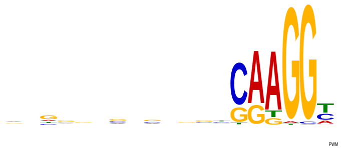	*CATGGNNNNNNNNNCAAGGT*	144.74	M
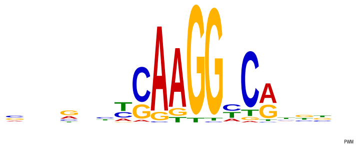	*NNNNGTCCAAGGCCANNNN*	103.59	DR:1
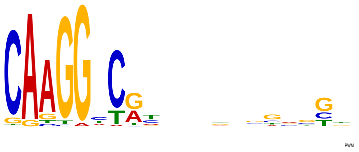	*CAAGGCCNNNNNNNNGGGGT*	207.34	DR:8
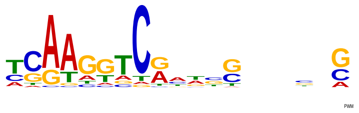	*NCAAGGNNNNNNNTTCGTN*	6.79	M
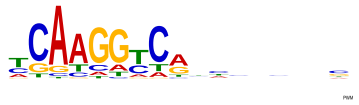	*NCAAGGNNNNNGCTTCN*	6.98	M
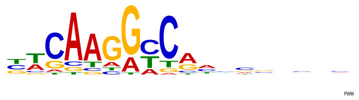	*NNCAAGGNNNNAGCTTNN*	5.06	DR:1

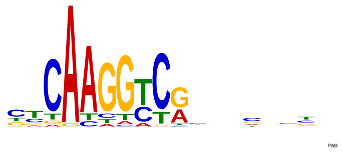	*NNNCAAGGNNNCAGCTNNN*	9.83	DR:1
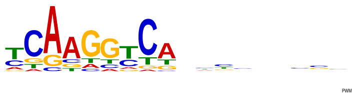	*NCAAGGNNNNNNNCTTCGN*	9.10	M
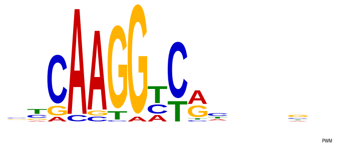	*NNCCAAGGNNACAGCNN*	24.94	M
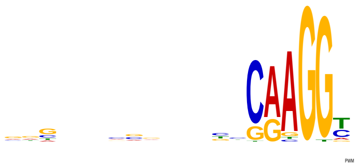	*GGGTCNNNNNNNNNCAAGGT*	147.95	DR:10
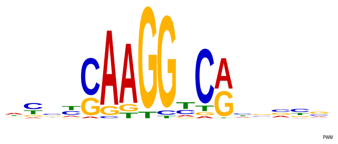	*NNNTCCAAGGCCACANNN*	77.66	M
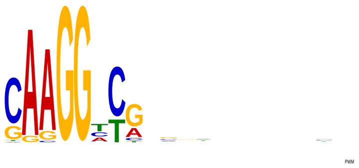	*CAAGGTCNNNNNNNNGGTGG*	182.59	DR:6

58 LRH1:RXRA Round 3

PWM	Seed Sequence	Seed Seq Enrichment	Repeat
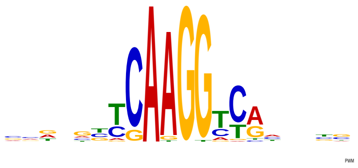	NNNNNTTCAAGGTCANNNNN	161.43	DR:0
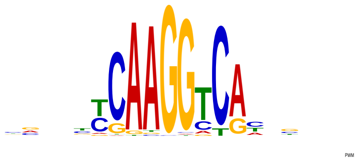	NNNNNCCAAGGTCACNNNNN	155.71	M
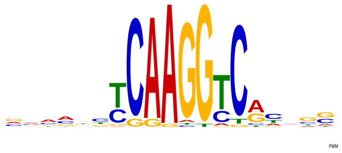	NNNNNTCTCAAGGTCNNNNN	108.74	DR:0
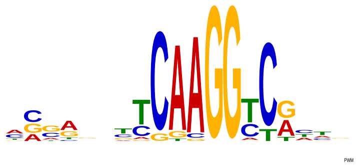	NNNNGTTTCAAGGTCNNNN	136.62	DR:0
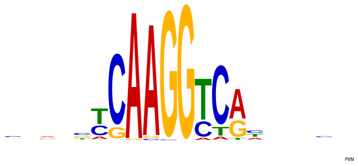	NNNNNTCAAGGTCATNNNNN	181.90	DR:0
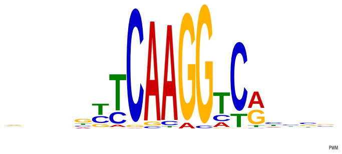	NNNNNGTTTCAAGGTCNNNNN	146.92	DR:0
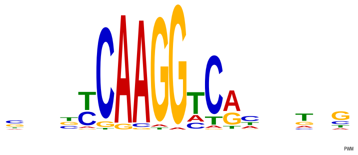	NNNNTCAAGGTCACGNNNN	218.18	DR:0

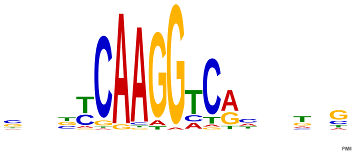	NNNNTCAAGNTCACGNNNN	62.48	DR:0
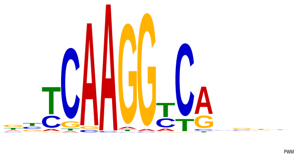	NNNNTTCAAGGCCANNNN	93.04	DR:0
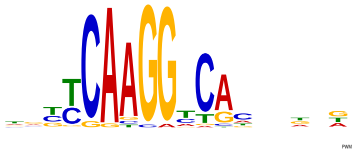	NNNTCAAGGNCACGCNNN	138.48	DR:0
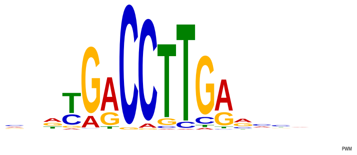	NNNNGACCTTGGACNNNN	64.54	ER:0
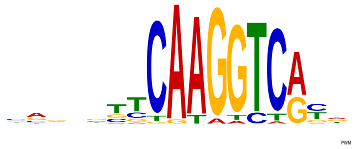	NNNCGGTCAAGGTCNNN	63.72	DR:0
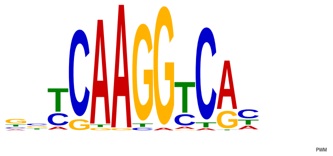	NNCTCAAGNTCACGNN	96.22	DR:0
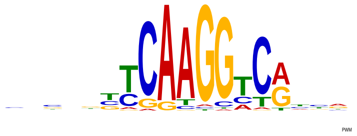	NNNNGTTCAAGGCCNNNN	55.53	DR:0

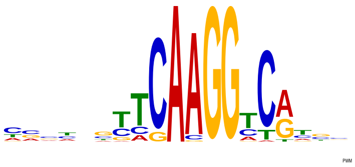	NNNN$\overrightarrow{AGTTCA}\overrightarrow{AGGTCN}$NNNN	161.17	DR:0
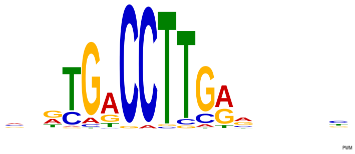	NNN$\overleftarrow{NGACCT}\ \overrightarrow{TGGGCN}$NNN	54.51	ER:0
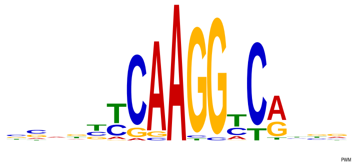	NN$\overrightarrow{NGTTCA}\overrightarrow{AGGCCA}$NNN	140.72	DR:0
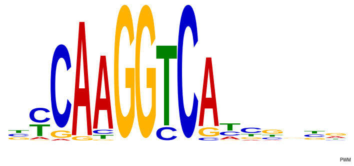	NNCA$\overrightarrow{AGGTCA}$TCGGNN	189.40	M
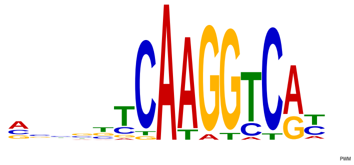	NN$\overrightarrow{CGGTCA}\overrightarrow{AGGTCA}$NN	158.96	DR:0
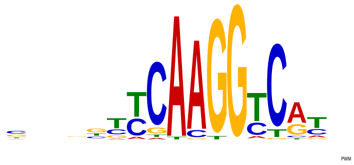	NN$\overrightarrow{NGGTCC}$A$\overrightarrow{AGGTCN}$NN	91.50	DR:1

59 SF1 Round 3

PWM	Seed Sequence	Seed Seq Enrichment	Repeat
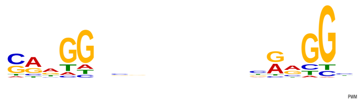	CAAGGNNNNNNNNNNGAGGT	95.55	DR:8
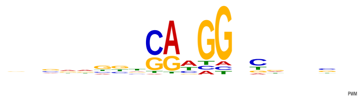	NNNNNGGTCAAGGCCNNNNN	81.83	DR:0
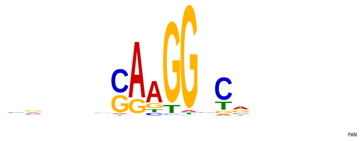	NNNNNCCAAGGCCACNNNNN	68.97	M
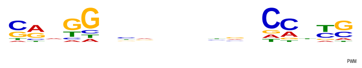	CAAGGNNNNNNNNNNCCTTG	60.48	IR:3
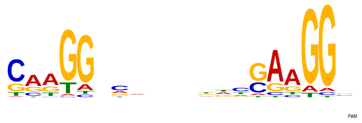	CAAGGNNNNNNNNNCGAAGGG	246.87	DR:8
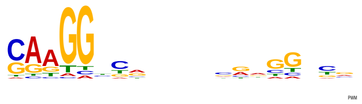	CAAGGNNNNNNNNNNNGGTCG	60.61	DR:6
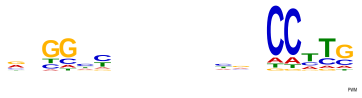	AAGGCNNNNNNNNNNNCCTTG	52.05	IR:6

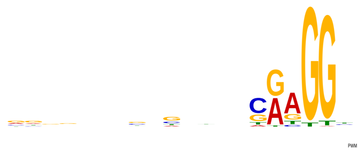	*GGGGCNNNNNNNNNCAAGGT*	91.27	DR:10
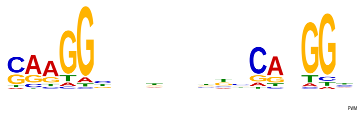	*CAAGGNNNNNNNNNCATGGT*	135.09	DR:8
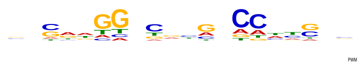	*NNCAAGGNNNNNNCCTTGNN*	43.88	IR:0
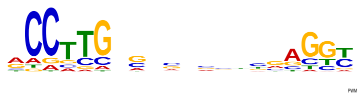	*ACCTTGNNNNNNNNGAGGT*	80.41	ER:12
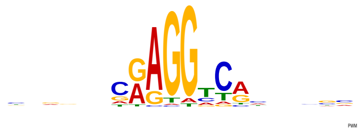	*NNNNNTCGAGGTCACNNNNN*	44.80	M
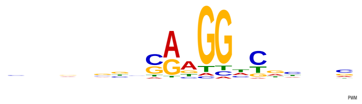	*NNNNNGGCCAAGGTCNNNNN*	64.27	DR:0
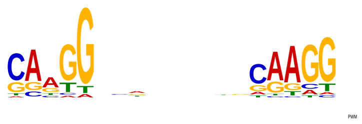	*CAAGGNNNNNNNNGCAAGGG*	251.93	DR:8

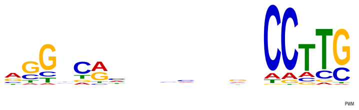	*AGGTCNNNNNNNNNNNCCTTG*	45.27	IR:6
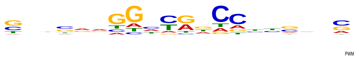	*NNNCAAGGNNNNNCCTTGNNN*	24.40	M
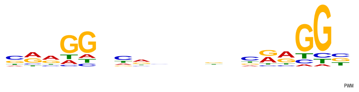	*CAAGGNNNNNNNNNCGAGGT*	81.45	DR:7
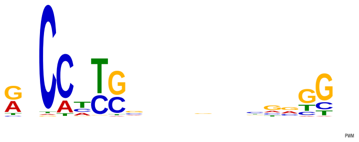	*GGCCTTGNNNNNNNNGGGGT*	132.09	ER:11
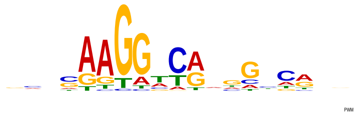	*NNNCAAGGNCGAGGCCNNN*	129.13	DR:0
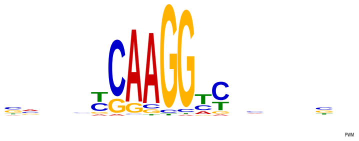	*NNNNNTCAAGGTCCCNNNNN*	50.99	DR:0

60 SF1:RXRA Round 3

PWM	Seed Sequence	Seed Seq Enrichment	Repeat
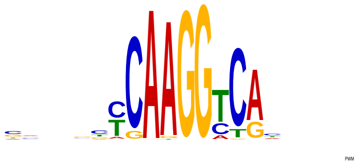	NNNNNCCCAAGGTCANNNNN	1811.77	M
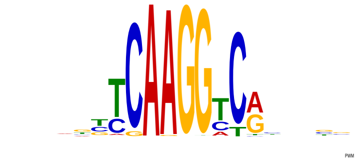	NNNNNTTCAAGGTCGNNNNN	1348.43	DR:0
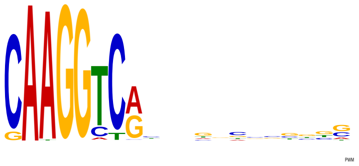	CAAGGTCNNNNNNNNCGGGG	2635.47	M
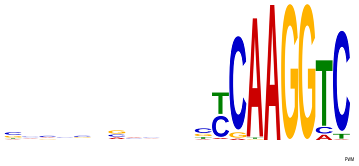	CCCCCNNNNNNNNCAAGGTC	2228.43	M
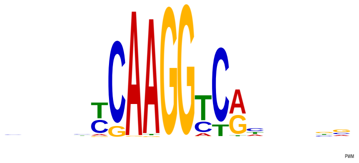	NNNNNTCAAGGTCATNNNNN	1124.00	DR:0
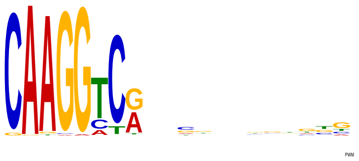	CAAGGTCNNNNNNNNGTGTG	2379.06	M
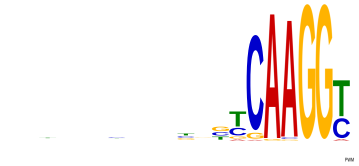	CATGTNNNNNNNNNCAAGGT	673.08	DR:9

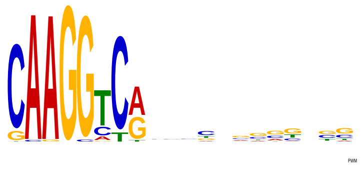	CAAGGTCNNNNNNNNGGTCG	2148.89	DR:6
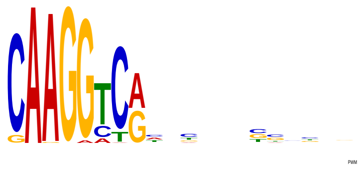	CAAGGTCNNNNNNNNGCCGG	1735.94	M
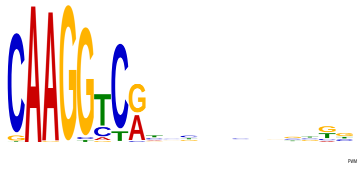	CAAGGTCNNNNNNNNTGTGG	1624.41	M
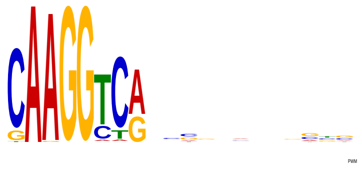	CAAGGTCNNNNNNNNTGGCG	1641.15	IR:7
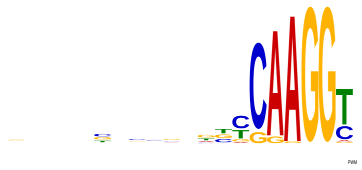	GGGCGNNNNNNNNNNCAAGGT	617.44	DR:11
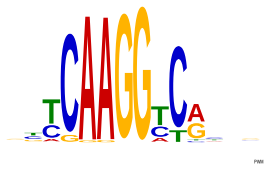	NNNNCGTTCAAGGTCNNNN	1036.79	DR:0
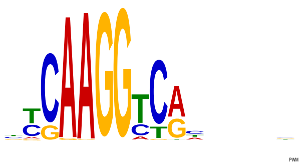	NNNNTCAAGNTCACGNNNN	511.73	DR:0

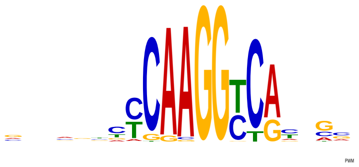	*NNNNGTCCCAAGGTCANNNNN*	2441.21	DR:2
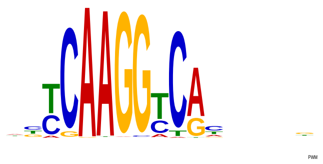	*NNNNTCAAGGCCACGNNNN*	921.21	DR:0
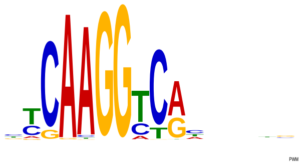	*NNNNTCAAGNTCATGNNNN*	367.61	DR:0
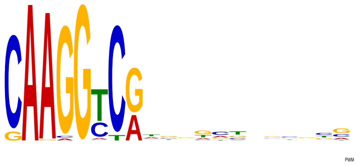	*CAAGGTCNNNNNNNNGCGCG*	2090.65	M
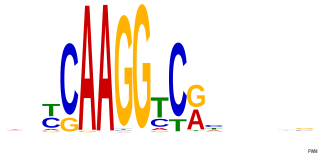	*NNNNTCAAGNTCATTNNNN*	259.18	DR:0
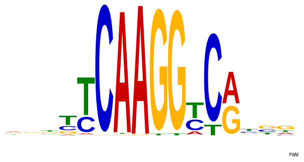	*NNNNGTTTCAAGGCCNNNN*	764.73	DR:0

61 TLX Round 3

PWM	Seed Sequence	Seed Seq Enrichment	Repeat
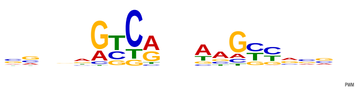	*NNNNAGTCANNAAGCCNNNN*	53.97	DR:2
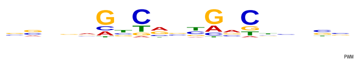	*NNNNAGTCANTGACTNNNN*	29.94	IR:1
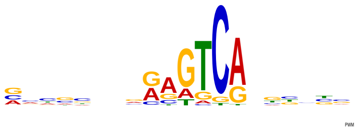	*NNNNNCAGGAGTCAANNNNN*	24.71	M
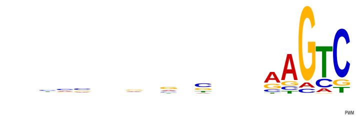	*CCCCCNNNNNNNNNNAAGTC*	22.43	M
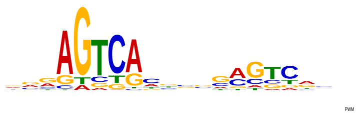	*NNNAGTCANNNNGAGTCNNN*	29.38	DR:4
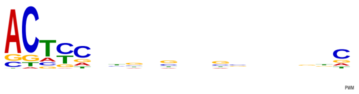	*ACTCCNNNNNNNNNNCCGTC*	19.15	M
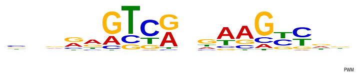	*NNGGAGTCNNNAAGCCNN*	57.63	DR:2

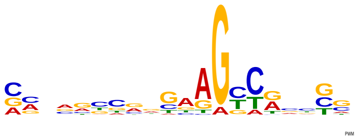	$N\overrightarrow{NNNGCCA}AN\overrightarrow{AAGTCA}NNNN$	55.03	DR:2
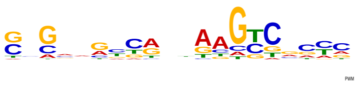	$NNN\overrightarrow{NCGTCA}NN\overrightarrow{AAGTCN}NNNN$	26.04	DR:2
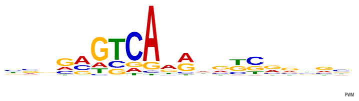	$NNN\overrightarrow{NAGTCA}N\overrightarrow{NAGTCG}NNNN$	23.90	DR:1
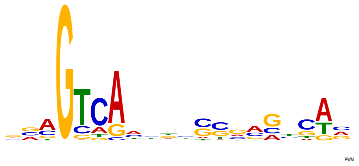	$N\overrightarrow{NAGTCA}NNNNNN\overrightarrow{GAGTCN}N$	18.32	DR:6
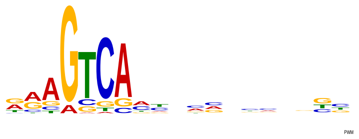	$N\overrightarrow{AAGTCA}NNNNNNCCGGGN$	32.01	M
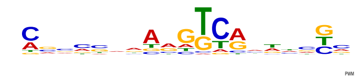	$NNNNNCAA\overrightarrow{AGGTCA}ANNNNN$	14.61	M
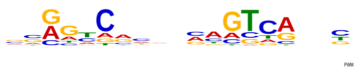	$N\overrightarrow{NNGTCA}CNN\overrightarrow{NAGTCA}NNN$	16.42	DR:3

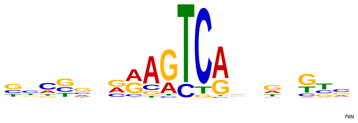	*NNNNNCGGAGTCAACNNNNN*	23.81	M
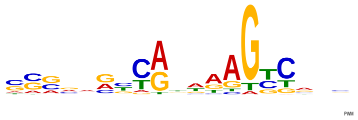	*NNNGCGTCANNAAGTCNNN*	37.52	DR:2
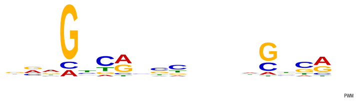	*NNAGTCANNNNNAAGTCNN*	13.13	DR:5
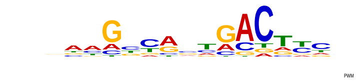	*NNNAAGCCANTGACTNNN*	41.55	IR:1
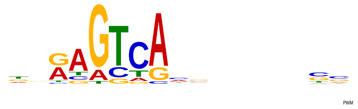	*NNAAGTCANNCGGCCNN*	21.63	DR:2
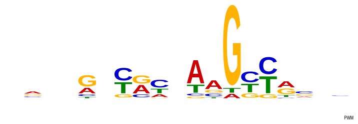	*NNNCGTCANNAAGCCANNN*	41.73	DR:2

62 TLX:RXRA Round 3

PWM	Seed Sequence	Seed Seq Enrichment	Repeat
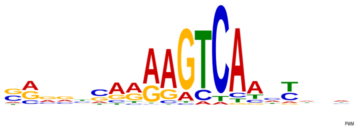	*NNNNNCAAAAGTCAANNNNNN*	48.86	M
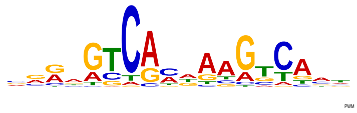	*NNNAGTCANNAAGTCNNN*	25.45	DR:2
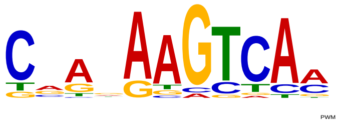	*CAAGAAGTCAA*	68.51	M
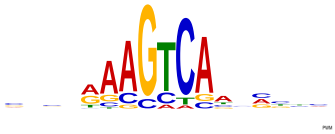	*NNNNTAAAGTCAATNNNN*	12.41	M
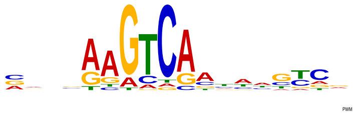	*NNNNAAGTCAATAANNNN*	14.92	M
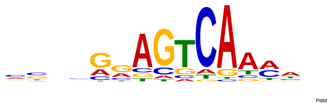	*NNNCCGGAGTCAANNN*	16.19	M
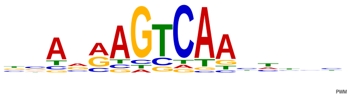	*NNNGAAGTCAAGTNNN*	11.23	M

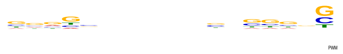	GGGGGNNNNNNNNNGGGGG	9.43	M
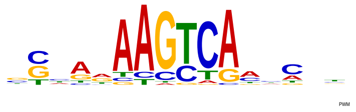	NNNATAAGTCAATNNN	11.81	M
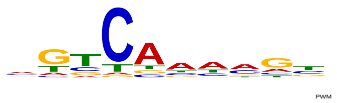	AGTCAAAAGT	9.67	DR:1
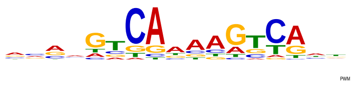	NNNAGTCANAAGTCNNN	16.16	DR:1
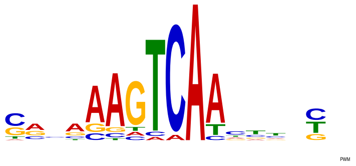	NNNAAAGTCAACTTNNN	38.62	M
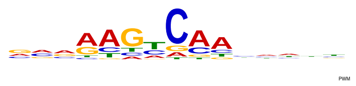	NNAAAGTNAACAANN	7.66	M
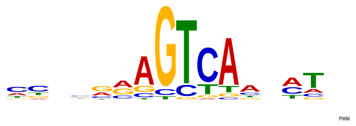	NNNCGGAGTCAATNNN	11.21	M

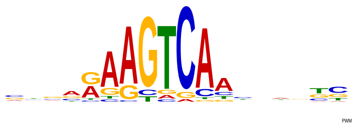	NNNNAAAGTCAATTNNNN	15.29	M
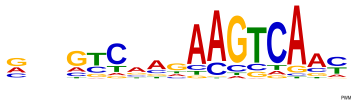	NNNGCCAANAAGTCNNN	14.83	DR:2
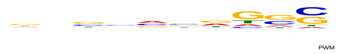	GGGCGCGGGG	4.82	M
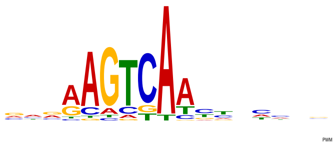	NNNNAAGTCAACTGNNNN	12.45	M
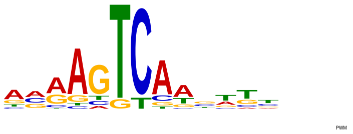	NNNAGTCAAGTTTNNN	10.44	DR:0
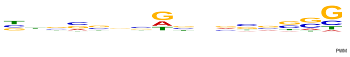	NNGGGGGNNCGCGGNN	4.83	M

63 PNR Round 3

PWM	Seed Sequence	Seed Seq Enrichment	Repeat
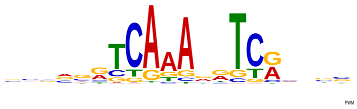	*NNNNNGTCAAAGGTCNNNNN*	138.45	DR:1
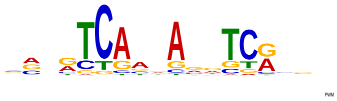	*NNNNGTCAANAGGTCNNNN*	74.49	DR:2
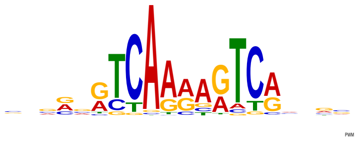	*NNNNNGTCAAGAGTCNNNNN*	98.71	DR:1
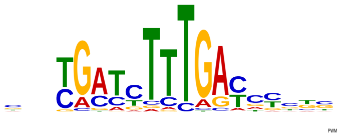	*NNNNNGCTCTTTGACNNNNN*	52.09	IR:1
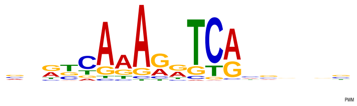	*NNNNCAAAGNTCACGNNNN*	107.43	M
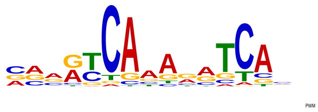	*NNNNCATCAAAGAGNNNN*	8.41	M
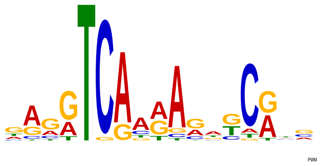	*NNNAGGTCAANAGAGCNNN*	91.76	DR:2

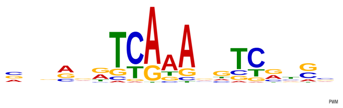	$NNNN\overrightarrow{AGGTCN}A\overrightarrow{AGGTCN}NNN$	115.43	DR:1
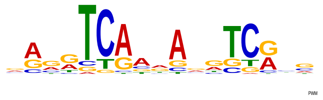	$NNN\overrightarrow{AGGTCN}NN\overrightarrow{AGGTCN}NN$	36.55	DR:2
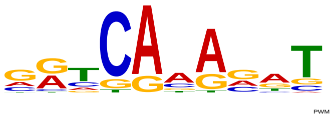	$\overrightarrow{CGTCA}AAGAG$	9.53	M
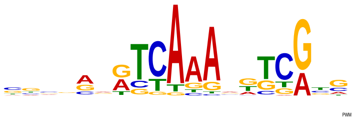	$NNNG\overrightarrow{GGGTCN}A\overrightarrow{AGGTCN}NN$	108.20	DR:1
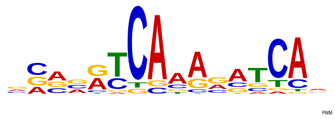	$NNN\overrightarrow{ACATCA}AAGAGNNN$	12.38	M
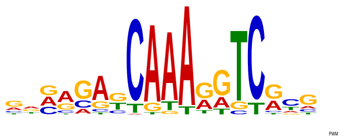	$NNNN\overrightarrow{AGAGCA}A\overrightarrow{AGGTCN}NNN$	251.49	DR:1
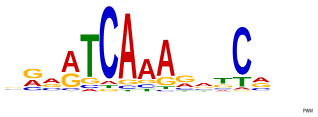	$NNNN\overrightarrow{AATCA}AAGAGNNNN$	14.47	M

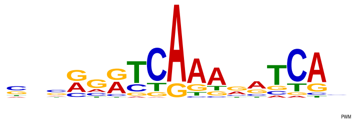	$NNN\overrightarrow{ACGTCA}AAGAGNNN$	12.48	M
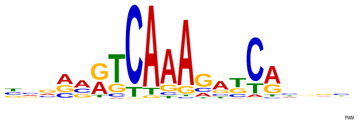	$NNNN\overrightarrow{NATCA}A\overrightarrow{AGAGCN}NNNN$	38.82	DR:1
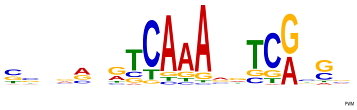	$NNNN\overrightarrow{GGGTCN}A\overrightarrow{AGGTCN}NNN$	80.10	DR:1
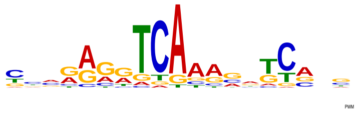	$NNNN\overrightarrow{AGGTCN}AAGAGNNNN$	14.79	M
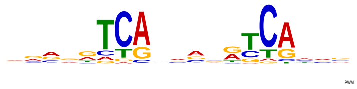	$NN\overrightarrow{AGGTCN}NN\overrightarrow{NNGTCA}ANN$	42.87	DR:2
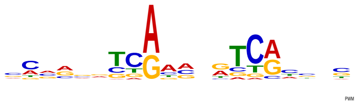	$NNN\overrightarrow{AGGTCN}N\overrightarrow{NNGTCA}ANNN$	33.40	DR:1

64 COUP-TF2 Round 3

PWM	Seed Sequence	Seed Seq Enrichment	Repeat
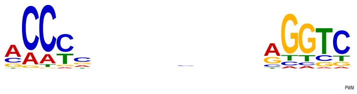	ACCCCNNNNNNNNNNNAGGTC	83.91	ER:11
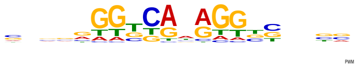	NNNNNGGTCAAAGGTNNNNN	60.55	DR:1
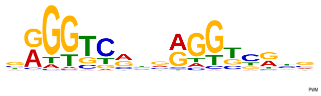	NNNGGGTCNNNGGGTCNNN	39.15	DR:2
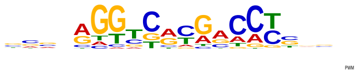	NNNNAGGTCNNGACCCNNNN	43.43	IR:0
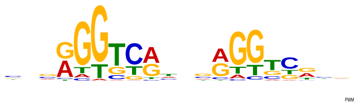	NNNGGGTCNNNNGGGTCNNN	29.92	DR:3
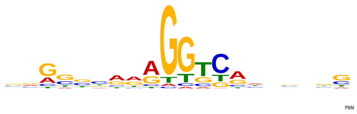	NNNNNCAAAGGTCAANNNNN	43.23	M
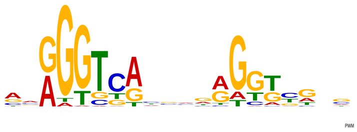	NNNGGTCACNNNGGGTCNNN	48.33	DR:4

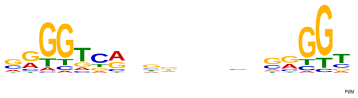	*GGGGTCNNNNNNNNNGGGGT*	64.79	DR:9
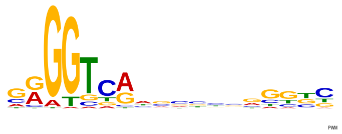	*NGGGGTCNNNNNNGGGGTN*	29.00	DR:6
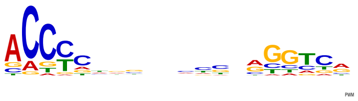	*ACCCCNNNNNNNNNNGGTCG*	28.32	ER:10
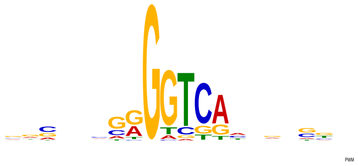	*NNNNNCGGGGTCACGNNNNN*	34.20	M
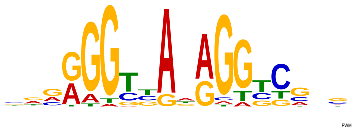	*NNGGGGTTANAGGTCGNN*	211.24	DR:1
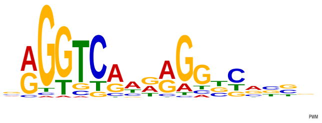	*NNNNGGTCAANAGGGCNNNN*	58.37	DR:2
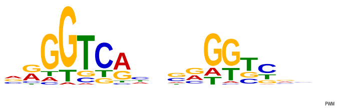	*NNNGGTCACNNGGGTCNNN*	63.51	DR:3

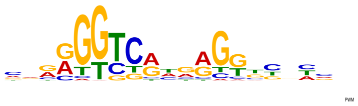	NNNNGGTCACNGGGTCNNNN	50.52	DR:2
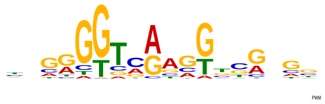	NNNNGGGGTCNAGGTCNNNN	46.84	DR:0
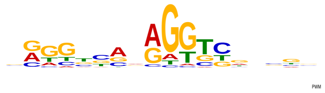	NNNNNGTCAGGGGTCNNNNN	26.68	DR:1
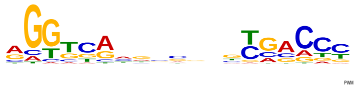	AGGTCNNNNNNNNNGACCC	20.24	IR:7
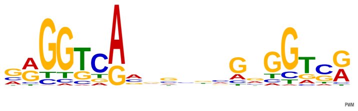	NGGGTCNNNNNNNNGGGTCN	22.68	DR:7
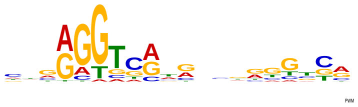	NNGGGGTCNNNNNGGGGTNN	28.99	DR:5

65 COUP-TF1 Round 3

PWM	Seed Sequence	Seed Seq Enrichment	Repeat
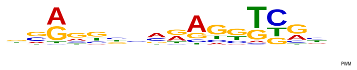	*NNNGGCCANAGGTCNNN*	6.41	DR:1
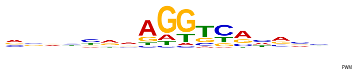	*NNNNCAAAGGTCAANNNN*	9.96	M
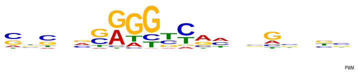	*NNNNNGGGGTCAAGGNNNNN*	6.10	DR:0
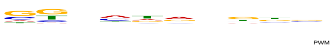	*GTCAAAGGCC*	3.43	DR:1
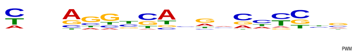	*NNNAGGTCNNGACCCNNN*	6.45	IR:0
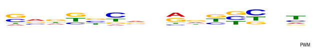	*NNNGGGTCNAAGGTNNN*	4.35	DR:1
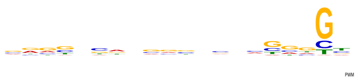	*GGGGTNNNNNNNNNNGGGGT*	5.43	DR:9

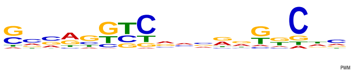	*NNNAGGTCNNCAGGTNNN*	3.57	DR:2
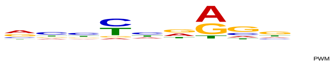	*ACCCCGAGGT*	3.12	ER:2
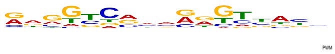	*NNAGGTCNNNNGGTTANN*	4.92	DR:2
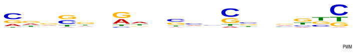	*NGGGTCNNNNNNNGGGGTN*	3.00	DR:7
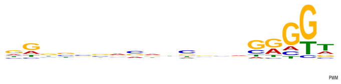	*AGGGGNNNNNNNNNNGGGGT*	5.75	DR:10
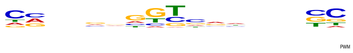	*NNNAGGGGNCAAAGNNN*	3.82	M
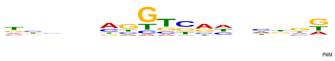	*NNNNAAAGGTCACTNNNN*	4.66	M

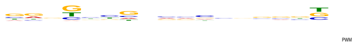	NGGGTCNNNNNNGGGGTN	3.27	DR:6
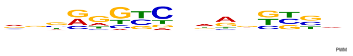	NNGGGGTCNAAGGTNN	9.21	DR:1
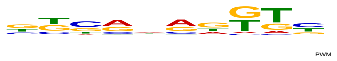	GTCAAAGGTC	7.00	DR:1
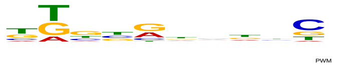	GGTCAAAGGT	3.40	DR:1
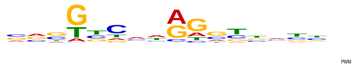	NNNGTCAANGGTTANNN	4.75	DR:1
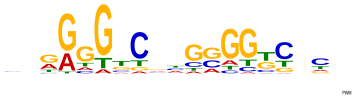	NNNGGGTCNNGGGGTCNNN	12.22	DR:2

66 EAR2 Round 3

PWM	Seed Sequence	Seed Seq Enrichment	Repeat
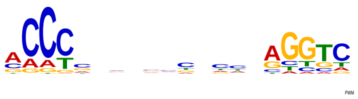	ACCCCNNNNNNNNNNNAGGTC	21.00	ER:11
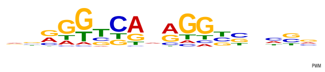	NNNNNGGTCAAAGGTNNNNN	14.73	DR:1
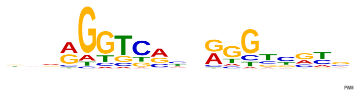	NNNAGGTCNNNGGGTCNNN	9.67	DR:2
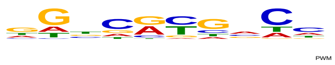	GGTCATGACC	8.69	IR:0
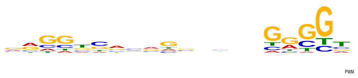	GAGGTNNNNNNNNNNGGGGT	9.85	DR:9
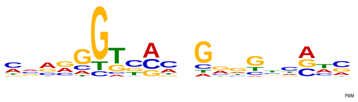	NNNAGGTCNNNNGGGTCNNN	5.51	DR:3
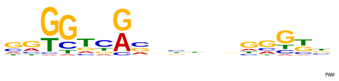	NGGGGTCNNNNNNGGGGTN	10.76	DR:6

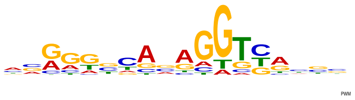	NNNGGCCANAGGTCANNN	17.34	DR:1
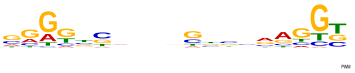	GGGGTNNNNNNNNNAAGGT	7.07	DR:8
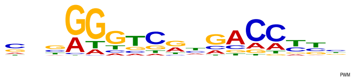	NNGGGGTCNNGACCTNN	19.09	IR:0
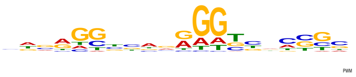	NNNNNGTCAGGGGTCNNNNN	8.50	DR:1
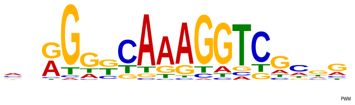	NNNGGGCAAAGGTCGNNN	50.71	DR:1
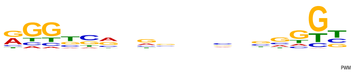	AGGTCNNNNNNNNGGGGT	8.12	DR:8
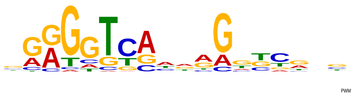	NNNGGTCAANAGGGCNNN	12.44	DR:2

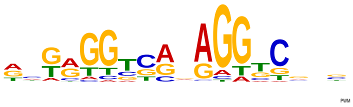	*NNGGGGTCNNAGGTCGNN*	41.07	DR:1
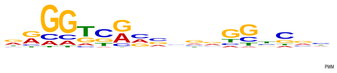	*NNAGGTCNNNNNGGGTCNN*	4.72	DR:4
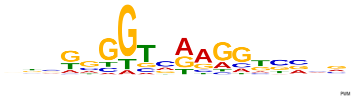	*NNNGGGGTCNAGGTCNNN*	12.30	DR:0
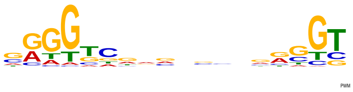	*GGGGTCNNNNNNNGAGGT*	16.22	DR:7
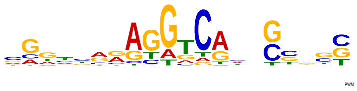	*NNNNNAAAGGTCACGNNNNN*	8.62	M
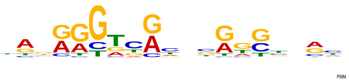	*NNGGGGTCNNNNGGGTCNN*	9.61	DR:3

67 COUP-TF2+17 Round 3

PWM	Seed Sequence	Seed Seq Enrichment	Repeat
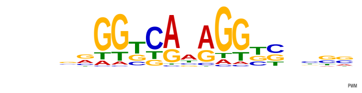	NNNNNGGTCAAAGGTNNNNN	254.53	DR:1
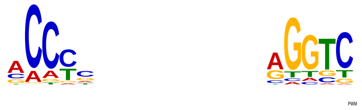	ACCCCNNNNNNNNNNNAGGTC	257.18	ER:11
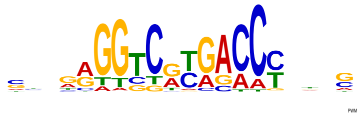	NNNNAGGTCGTGACCCNNNN	763.99	IR:0
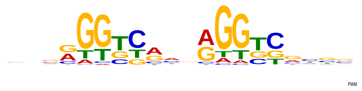	NNNNGGTCANNAGGTCNNNN	153.38	DR:2
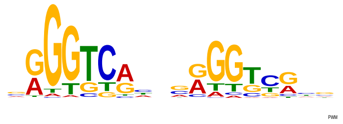	NNNGGTCACNNGGGTCNN	206.51	DR:3
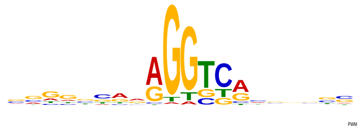	NNNNNCAAAGGTCAANNNNN	132.79	M
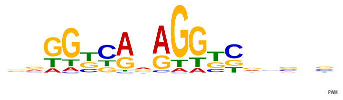	NNNNGTCAAAGGTCGNNNN	313.65	DR:1

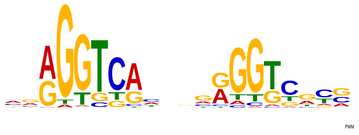	*NNNGGTCACNNNGGGTCNNN*	175.09	DR:4
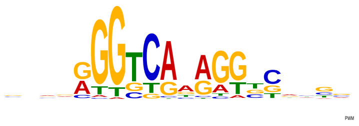	*NNNNAGGTCNAAGGGCNNNN*	212.94	DR:1
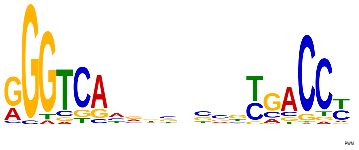	*GGGTCANNNNNNNNNGACCT*	150.42	IR:8
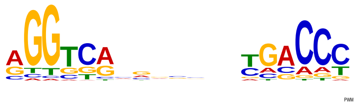	*AGGTCNNNNNNNNNGACCC*	77.26	IR:7
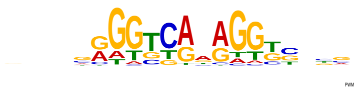	*NNNNGGGGTCNAAGGTNNNN*	273.14	DR:1
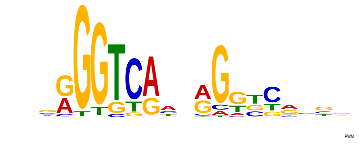	*NNNNGGTCAANAGGGCNNNN*	176.39	DR:2
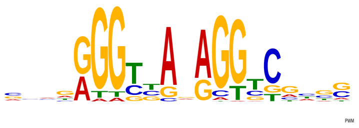	*NNNGGGGTTANAGGTCGNNN*	840.41	DR:1

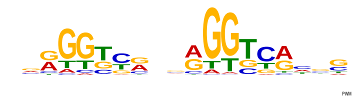	*NNGGGTCNNNNGGTCAANN*	202.19	DR:2
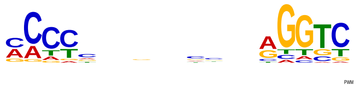	*ACCCCNNNNNNNNNNAGGTC*	81.78	ER:10
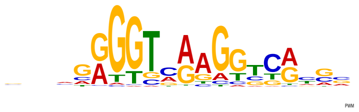	*NNNNGGGGTCNAGGTCNNNN*	142.33	DR:0
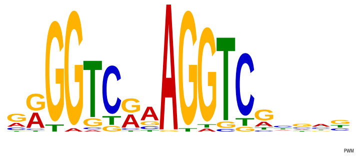	*NNGGTCGAAGGTCGCGNN*	1508.05	DR:1
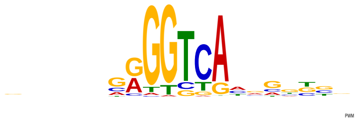	*NNNNNCGGGGTCAAGNNNNN*	102.71	M
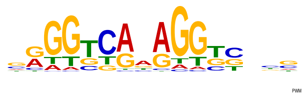	*NNNNGGGTCNAAGGTNNNN*	179.05	DR:1

68 COUP-TF1+17 Round 3

PWM	Seed Sequence	Seed Seq Enrichment	Repeat
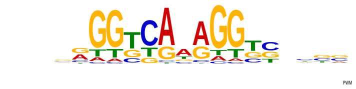	*NNNNNGGTCAAAGGTNNNNN*	235.63	DR:1
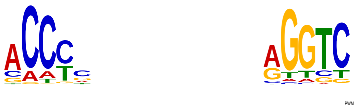	*ACCCCNNNNNNNNNNNAGGTC*	268.37	ER:11
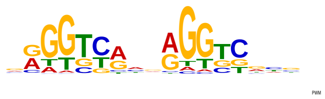	*NNNNGGTCANNAGGTCNNNN*	134.27	DR:2
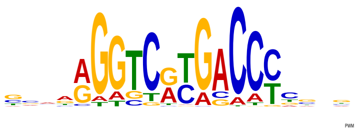	*NNNNAGGTCGTGACCCNNNN*	718.58	IR:0
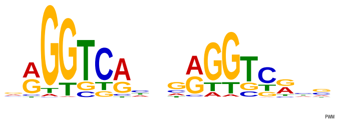	*NNNGGTCACNNGGGTCNNN*	175.79	DR:3
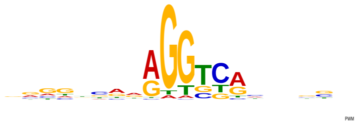	*NNNNNCAAAGGTCAANNNNN*	121.07	M
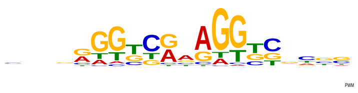	*NNNNGGGTCNAAGGTCNNNN*	376.74	DR:1

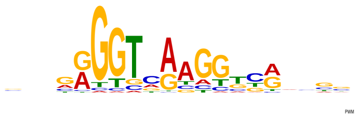	*NNNNNGGTCAAGGTCNNNNN*	81.54	DR:0
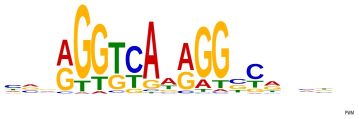	*NNNNGGTCAAAGGCCANNNN*	423.86	DR:1
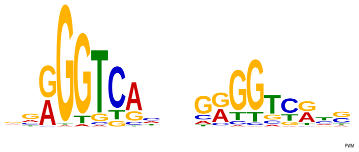	*NNNGGTCACNNNGGGTCNNN*	146.27	DR:4
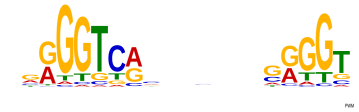	*AGGGGTCNNNNNNNNGGGGT*	313.69	DR:8
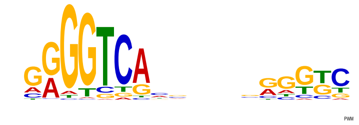	*NGGGGTCNNNNNNGGGGTN*	71.13	DR:6
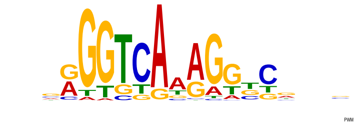	*NNNNGGTCAAAGGGCNNNN*	351.84	DR:1
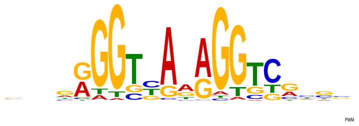	*NNNNGGGTTANAGGTCNNNN*	245.61	DR:1

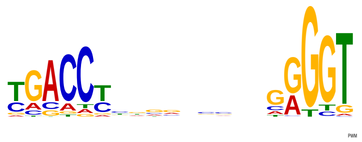	$\overleftarrow{CGACCT}NNNNNNNNNG\overrightarrow{GGGT}$	134.56	ER:10
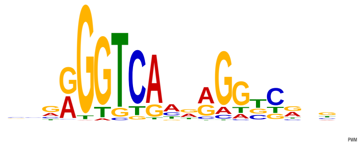	$NNN\overrightarrow{NGGTCA}AN\overrightarrow{AGGGCN}NNN$	149.33	DR:2
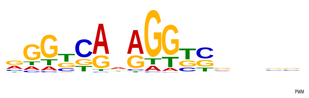	$NNN\overrightarrow{NNGTCA}A\overrightarrow{AGGTCN}NNNN$	184.94	DR:1
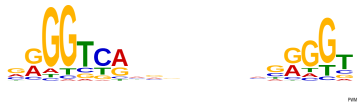	$G\overrightarrow{GGGTCA}NNNNNNNNNG\overrightarrow{GGGT}$	274.17	DR:9
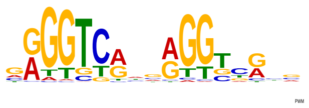	$NNNG\overrightarrow{GGGTCN}NN\overrightarrow{GGGTCN}NN$	173.30	DR:2
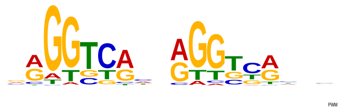	$NN\overrightarrow{NGGTCA}CN\overrightarrow{NGGTCA}NNN$	188.24	DR:2

69 COUP-TF2:RXRA Round 3

PWM	Seed Sequence	Seed Seq Enrichment	Repeat
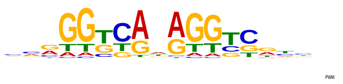	*NNNNGGTCANAGGTCNNNN*	31.73	DR:1
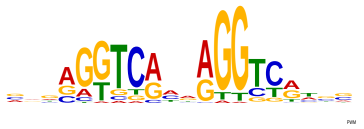	*NNNNGGTCANNAGGTCNNNN*	29.79	DR:2
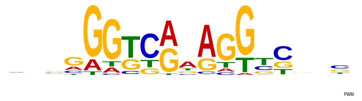	*NNNGGGTCNAAGGTCNN*	46.42	DR:1
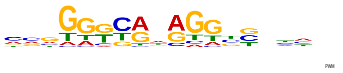	*NNNNGGTCAAAGGTCNNNN*	44.50	DR:1
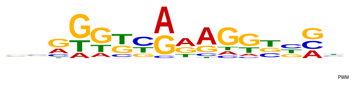	*NNAGGTCNAAGGTCNN*	31.84	DR:1
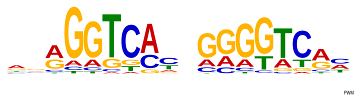	*NNAGGTCNNNGGGGTCNN*	23.20	DR:3
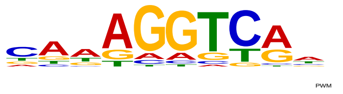	*CAAAGGTCAA*	28.07	M

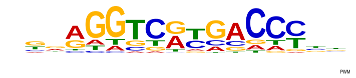	$NNN\overrightarrow{AGGTCN}\overleftarrow{TGACCN}NN$	12.89	IR:0
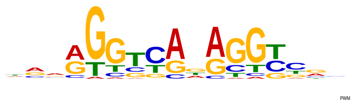	$NNA\overrightarrow{AGGTCN}N\overrightarrow{AGGTCN}N$	26.62	DR:1
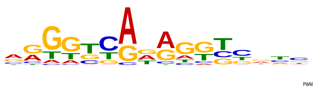	$NNN\overrightarrow{AGGTCN}N\overrightarrow{AGGTCN}NN$	16.03	DR:1
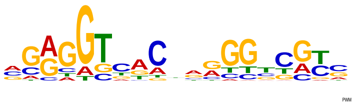	$NNN\overrightarrow{GGTCAN}NN\overrightarrow{AGGTCN}NN$	7.90	DR:3
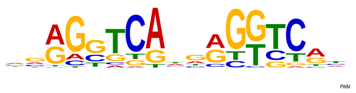	$NN\overrightarrow{GGGTCN}NG\overrightarrow{GGGTCN}N$	18.93	DR:2
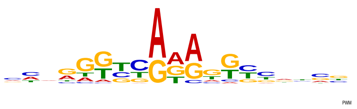	$NNN\overrightarrow{AGGTCN}N\overrightarrow{NGGTCA}NNN$	11.68	DR:1
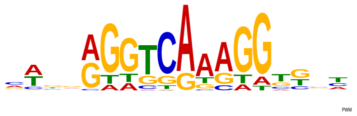	$NNNG\overrightarrow{AGGTCN}A\overrightarrow{AGGTNN}N$	27.81	DR:1

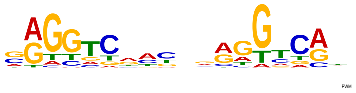	*NNGGTCANNNNAGGTCNN*	7.04	DR:4
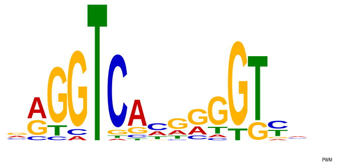	*NNNGGTCACGGGGTNNN*	26.68	DR:2
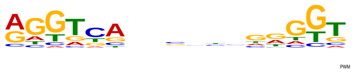	*AGGTCNNNNNNNGGGGT*	8.15	DR:7
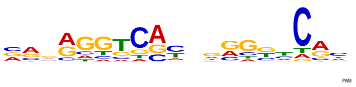	*NNNAGGTCNNNGGGGTNNN*	7.32	DR:3
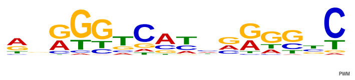	*NGAGGTCNNNGGGGTN*	10.07	DR:3
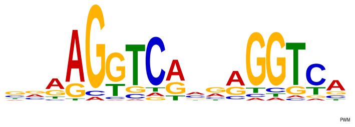	*NNAAGGTCNNGAGGTNN*	21.94	DR:2

70 COUP-TF1:RXRA Round 3

PWM	Seed Sequence	Seed Seq Enrichment	Repeat
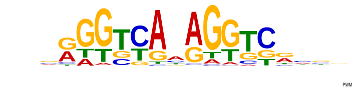	*NNNNGGTCANAGGTCNNNN*	36.46	DR:1
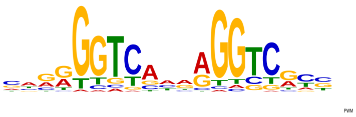	*NNNNGGTCANNAGGTCNNNN*	25.11	DR:2
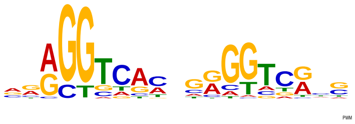	*NNNGGTCANNNGGGTCNNN*	11.63	DR:3
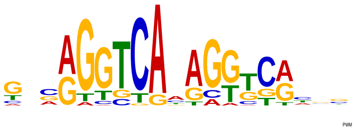	*NNNNGGTCATAGGTCNNNN*	34.45	DR:1
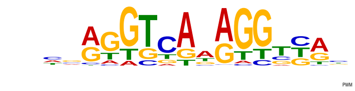	*NNNGGGGTCNAAGGTNNN*	31.62	DR:1
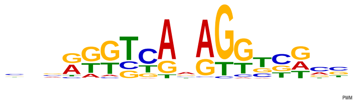	*NNNGGGTCNAAGGTCNNN*	43.44	DR:1
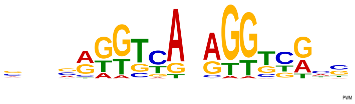	*NNNGGGGTCNNAGGTCNNN*	34.97	DR:1

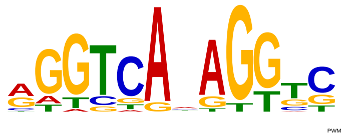	GGGTCATAGGTC	52.28	DR:1
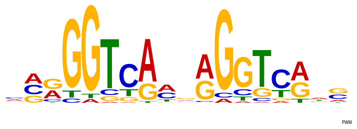	NNNGGTCANGGGGTCNNN	20.59	DR:2
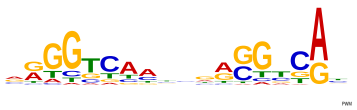	NNGGTCANNNNGGGTCNN	4.61	DR:4
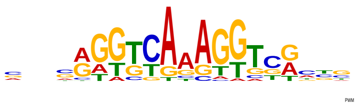	NNNNGGGTCNNAGGTCNNNN	25.14	DR:1
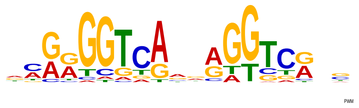	NNNAGGTCNNNGGGTCNNN	9.90	DR:2
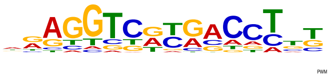	NNNAGGTCNTGACCNNN	10.02	IR:0
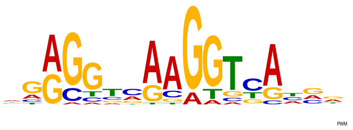	NNNGGTCAAGGTCANNN	13.14	DR:0

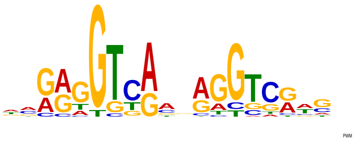	*NNNNGGTCANNGGGTCNNNN*	14.63	DR:2
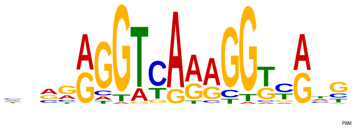	*NNNGGGGTCNNGGGTCNN*	17.10	DR:1
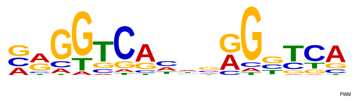	*NNGGTCATNGGGGTNN*	10.71	DR:3
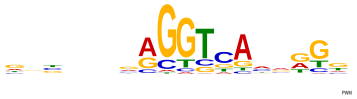	*NNNNCAGGGGTCAANNNN*	10.35	M
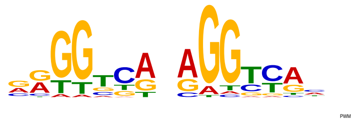	*NAGGTCNNNGGTCAAN*	31.14	DR:1
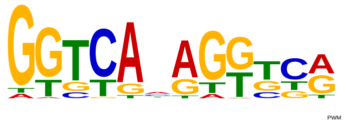	*GGTCATAGGTCA*	57.98	DR:1

71 EAR2:RXRA Round 3

PWM	Seed Sequence	Seed Seq Enrichment	Repeat
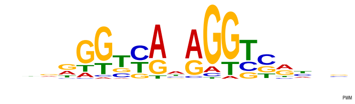	NNNNGGTCANAGGTCNNNN	41.02	DR:1
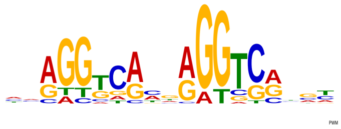	NNNNGGTCANNAGGTCNNN	19.67	DR:2
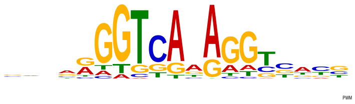	NNNNAGGTCNAAGGTNNNN	24.93	DR:1
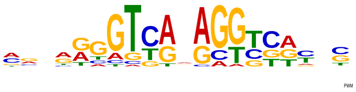	NNNNAGGTCNNAGGTCNNN	28.20	DR:1
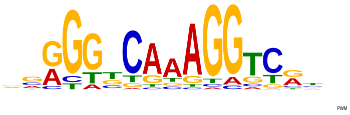	NNNGGGCAAAGGTCNN	49.30	DR:1
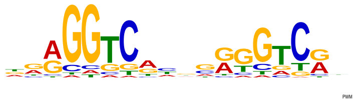	NNAGGTCNNNNGGGTCNN	9.82	DR:3
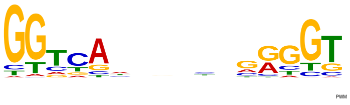	GGTCANNNNNNGGGGT	11.30	DR:7

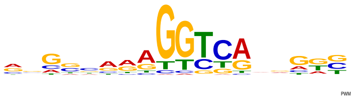	$NNNNCAA\overrightarrow{AGGTCA}ANNNN$	23.75	M
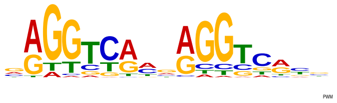	$NNN\overrightarrow{GGTCA}NN\overrightarrow{GGGTC}NNN$	13.56	DR:2
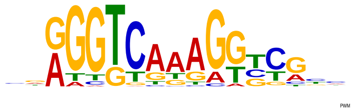	$NNN\overrightarrow{GGTCA}A\overrightarrow{AGGGC}NNN$	43.37	DR:1
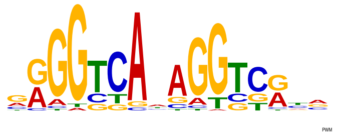	$NNN\overrightarrow{GGTCA}T\overrightarrow{AGGTC}NNN$	51.23	DR:1
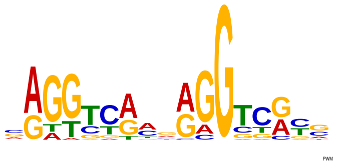	$NNN\overrightarrow{GGTCA}NG\overrightarrow{GGGTC}NNN$	22.01	DR:2
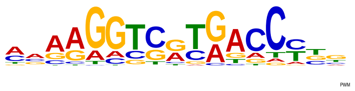	$NNN\overrightarrow{AGGTCN}\overleftarrow{TGACC}NNN$	13.71	IR:0
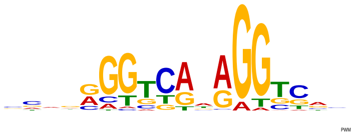	$NNNG\overrightarrow{GGGTCN}A\overrightarrow{AGGTN}NN$	41.78	DR:1

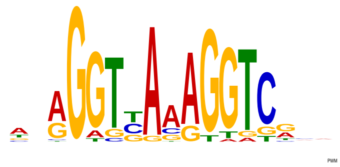	$NNN\overrightarrow{AGGTGA}N\overrightarrow{AGGTCN}NN$	31.95	DR:1
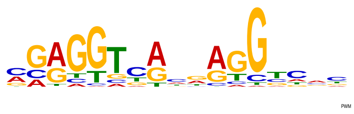	$NN\overrightarrow{GGGTCN}NN\overrightarrow{GGGTCN}N$	8.29	DR:2
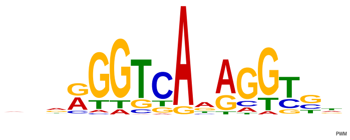	$NNG\overrightarrow{AGGTCN}A\overrightarrow{AGGTNN}$	40.10	DR:1
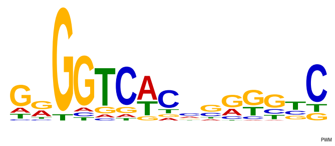	$NG\overrightarrow{GGGTCN}NNG\overrightarrow{GGGTN}$	10.98	DR:3
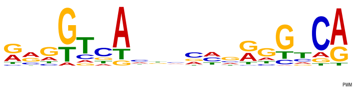	$N\overrightarrow{NGGTCA}NNNNNG\overrightarrow{GGGTNN}$	6.62	DR:6
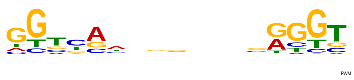	$\overrightarrow{GGTCA}NNNNNNNG\overrightarrow{GGGT}$	8.14	DR:8

72 HNF4G Round 3

PWM	Seed Sequence	Seed Seq Enrichment	Repeat
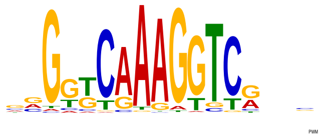	NNNNGGTCAAAGGTCNNNN	156.62	DR:1
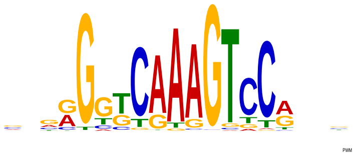	NNNNGGTCAAAGTCCNNNN	184.06	DR:1
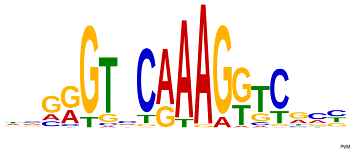	NNGGGTCCAAAGGTCGNN	860.90	DR:1
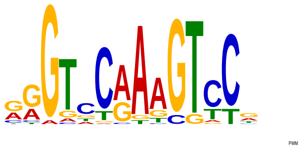	NNNGGGTCCAAAGTCCNNN	666.93	DR:1
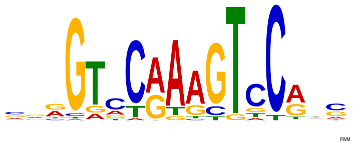	NNAGTCCAAAGTCCANN	524.58	DR:1
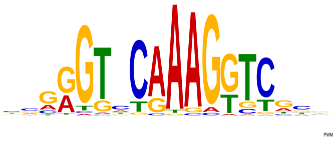	NNNGGGTCCNAAGGTCNNN	203.46	DR:1
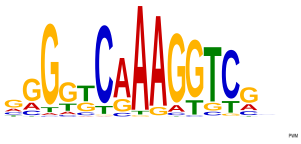	NNNGGGGTCNAAGGTCNNN	212.71	DR:1

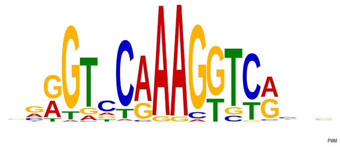	*NNNGGTCCNAAGGTCANNN*	148.10	DR:1
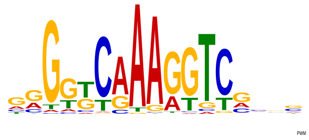	*NNNNGGGTCNAAGGTCNNNN*	118.66	DR:1
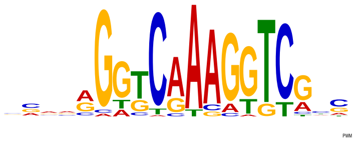	*NNNAAGGTCNAAGGTCNNN*	97.83	DR:1
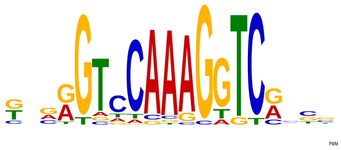	*NNNNAGTCCNAAGGTCNNNN*	80.31	DR:1
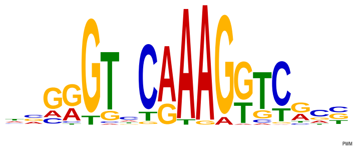	*NNGGGTCCNAAGGTCGNN*	352.35	DR:1
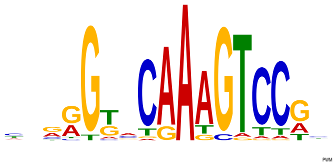	*NNAGGGTNCAAAGTCCNN*	312.60	DR:1
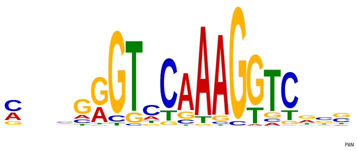	*NNNNGGGTCCNAAGGTNNNN*	67.21	DR:1

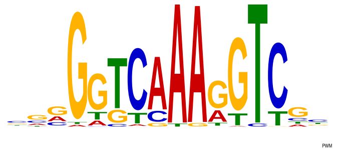	$NNC\overrightarrow{GGGTCN}A\overrightarrow{AGGTCN}N$	105.89	DR:1
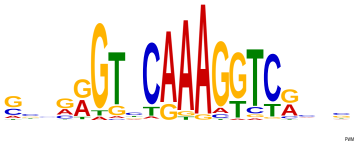	$NNNN\overrightarrow{GGTCCN}A\overrightarrow{AGGTCN}NNN$	94.10	DR:1
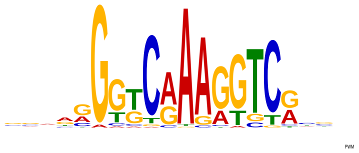	$NNNN\overrightarrow{AGGTCN}A\overrightarrow{AGGTCN}NNN$	63.33	DR:1
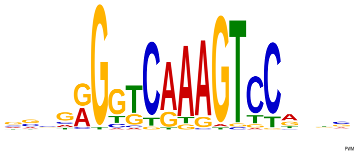	$NNNN\overrightarrow{GGGTCN}A\overrightarrow{AGTCCN}NNN$	141.55	DR:1
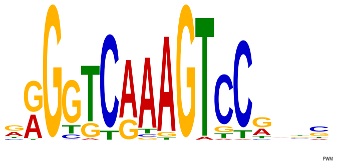	$NN\overrightarrow{GGGTCN}A\overrightarrow{AGTCCG}GNN$	264.95	DR:1
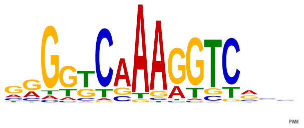	$NNNN\overrightarrow{GGGTCN}A\overrightarrow{AGGTNN}NN$	30.35	DR:1

73 HNF4A Round 3

PWM	Seed Sequence	Seed Seq Enrichment	Repeat
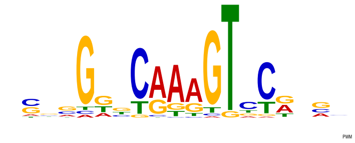	NNNNGGGCAAAGTCCNNNN	66.38	DR:1
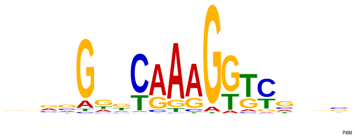	NNNNGGGCAAAGGTCNNNN	60.39	DR:1
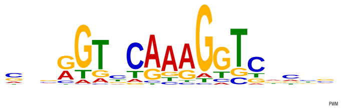	NNNNNGTCCAAAGGTNNNNN	25.35	DR:1
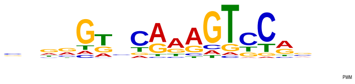	NNNNGTCCANAGTCCNNNN	24.54	DR:1
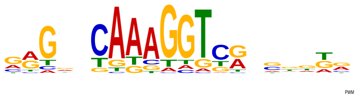	NNNNNCAAAGGTCGCNNNNNN	22.26	M
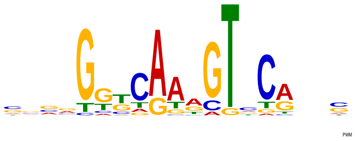	NNNNGGTCAAGGTCCNNNN	35.42	DR:1
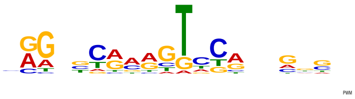	NNNNNCAAAGTCCAANNNNN	18.59	M

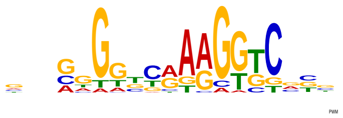	*NNNNGGGGTCNAAGGTNNNN*	24.41	DR:1
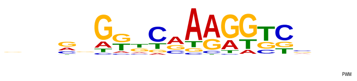	*NNNNGGGTCNAAGGTNNNN*	15.11	DR:1
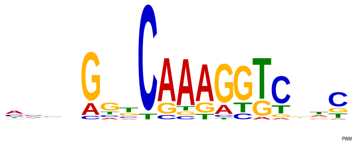	*NNNCGGTCAAAGGTCNNN*	68.99	DR:1
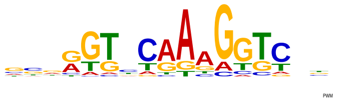	*NNNGGGTCCNAAGGTNNN*	26.30	DR:1
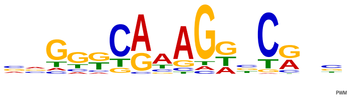	*NNGGTCAAAGGGCANN*	37.74	DR:1
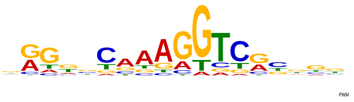	*NNNNCTAAAGGTCGNNNN*	6.54	M
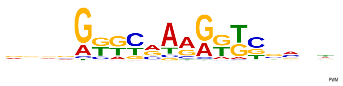	*NNNNGGGTCNAAGGTCNNNN*	38.26	DR:1

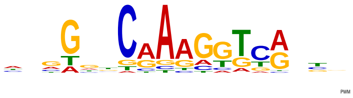	*NNNNCTCAAAGGTCNNNN*	7.98	DR:1
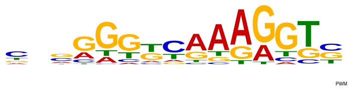	*NCGGGGTCNAAGGTN*	38.06	DR:1
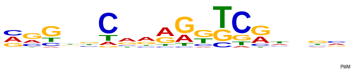	*NNNNCTAAAGGTCANNNN*	4.38	M
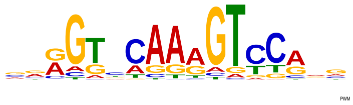	*NNNGTCCAAGGTCCNNN*	33.14	DR:1
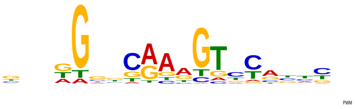	*NNNNNGTCAAAGTCCNNNNN*	20.13	DR:1
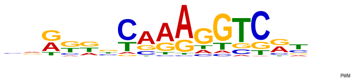	*NNNNGCTAAAGGTCNNNN*	6.51	DR:0

74 HNF4A+18 Round 3

PWM	Seed Sequence	Seed Seq Enrichment	Repeat
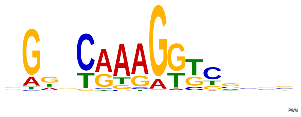	*NNNNGGGCAAAGGTCNNNN*	262.31	DR:1
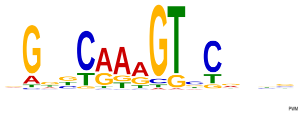	*NNNNGGGCAAAGTCCNNNN*	323.22	DR:1
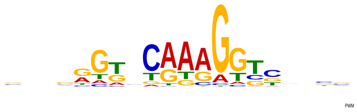	*NNNNNGTCCAAAGGTNNNNN*	84.36	DR:1
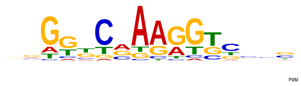	*NNNNGGGTCNAAGGTNNNN*	73.71	DR:1
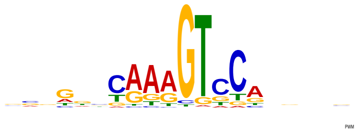	*NNNNNCCAAAGTCCANNNNN*	115.73	M
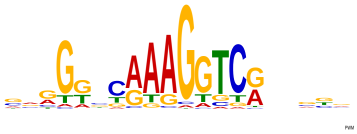	*NNNNNCTAAAGGTCGNNNNN*	39.33	M
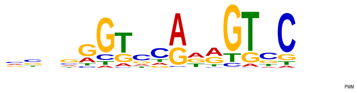	*NNNGGGTCCANAGTCCNNN*	410.09	DR:1

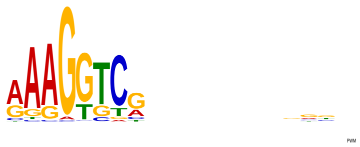	AAAGGTCNNNNNNNNGGGGG	215.19	M
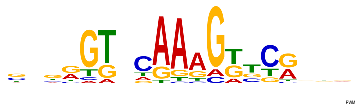	NNNNGTCCAAAGGGCNNNN	96.63	DR:1
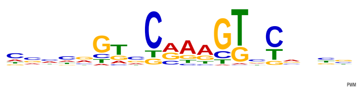	NNNNAGTCCNAAGTCCNNNN	151.96	DR:1
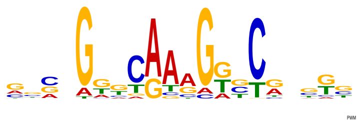	NNNNGGTCAAAGGGCANNNN	170.77	DR:1
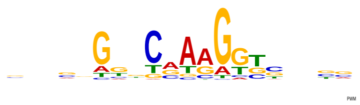	NNNNNGGTCAAAGGTNNNNN	83.09	DR:1
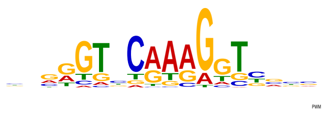	NNNNGGGTCCNAAGGTNNNN	114.61	DR:1
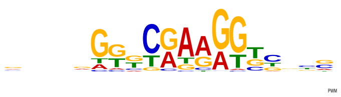	NNNNGGGGTCNAAGGTNNNN	91.86	DR:1

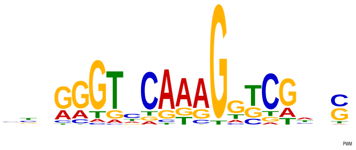	*NNNGGGTCCNNAGGTCGNNN*	269.98	DR:1
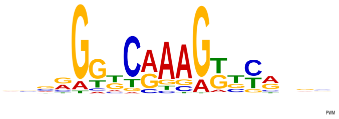	*NNNNGGGTCNAAGGGCNNNN*	101.16	DR:1
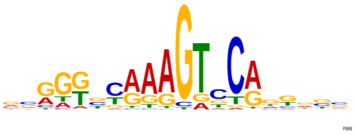	*NNNNNCTAAAGTCCGNNNNN*	25.20	M
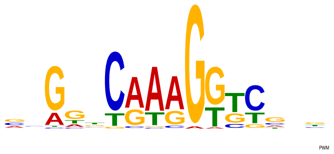	*NNCGGTCAAAGGTCGNN*	456.90	DR:1
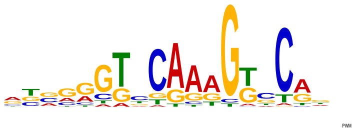	*NNNGGGTCCNAAGTGCNNN*	181.94	DR:1
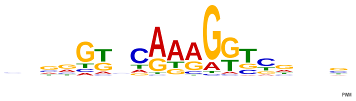	*NNNNGTCCANAGGTCNNNN*	86.82	DR:1

75 RXRB Round 3

PWM	Seed Sequence	Seed Seq Enrichment	Repeat
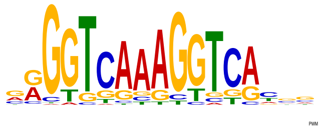	NNNNGGTCAAAGGTCNNNN	54.30	DR:1
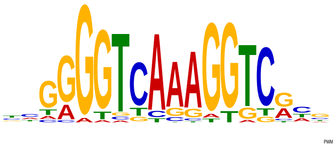	NNNGGGGTCNAAGGTCNNN	83.39	DR:1
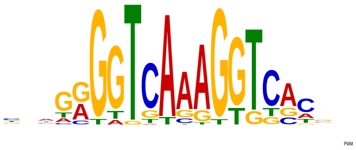	NNNNGGGTCNAAGGTCNNNN	37.39	DR:1
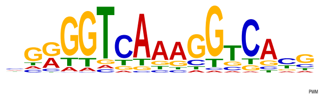	NNNNGGGTCNAAGGTNNNN	9.69	DR:1
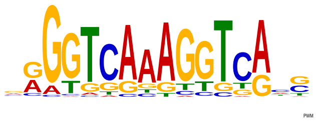	NNNAGGTCNAAGGTCNNN	25.30	DR:1
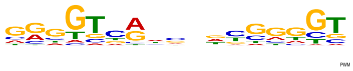	GGGGTCNNNNNNGGGGT	9.57	DR:6
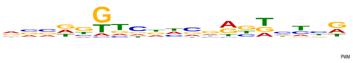	NNNGGGTCNNNGGGTCNNN	3.00	DR:2

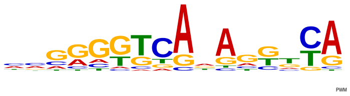	*NNGGGGTCNAGGGTNN*	6.16	DR:0
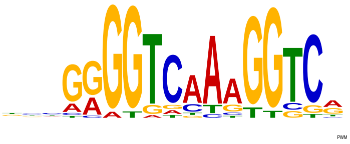	*NNCGGGGTCNAAGGTNN*	38.54	DR:1
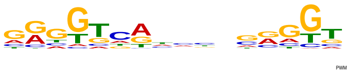	*GGGGTCNNNNNGGGGT*	8.49	DR:5
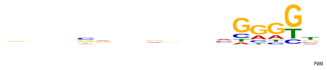	*GGGGGNNNNNNNNNNGGGGT*	5.54	M
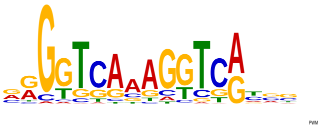	*NNNNNGTCAAAGGTCNNNNN*	17.10	DR:1
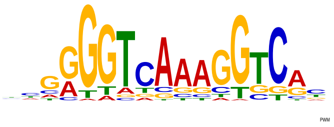	*NNNGGGGTCNAAGGTNNN*	21.42	DR:1
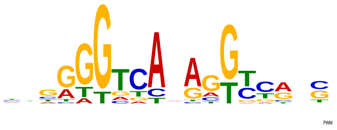	*NNNGGGGTCNNGGGTCNNN*	10.16	DR:1

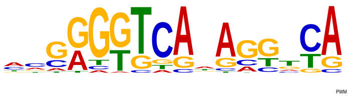	*NNGGGGTCAAGGGTNN*	13.91	DR:1
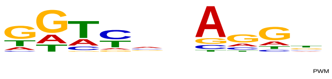	*GGTCAAGGGT*	3.26	DR:1
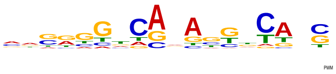	*NNNNGGGTCNAGGGTNNNN*	3.18	DR:1
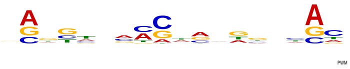	*NNGGTCANNNNAGGTCNN*	2.19	DR:4
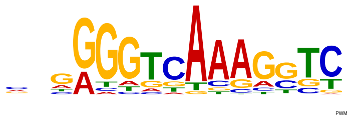	*NGGGGGNCAAAGGTN*	14.83	DR:1
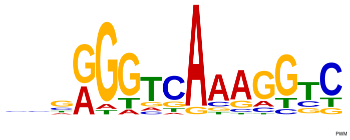	*NGGGGGTCNAAGGTN*	14.16	DR:1

76 RXRB+17 Round 3

PWM	Seed Sequence	Seed Seq Enrichment	Repeat
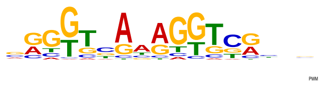	NNNNGGTCANAGGTCNNNN	21.52	DR:1
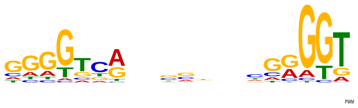	GGGGTCNNNNNNNNNGGGGT	41.21	DR:9
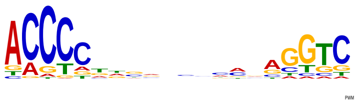	ACCCCNNNNNNNNNNNAGGTC	23.26	ER:11
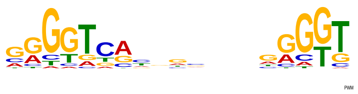	GGGGTCNNNNNNNNGGGGT	33.61	DR:8
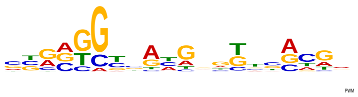	NNNNGGTCANNGGGTCNNNN	6.06	DR:2
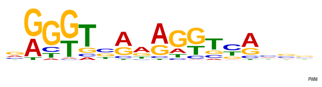	NNNNGGTCANGGGTCNNNN	11.11	DR:1
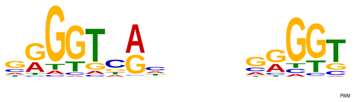	GGGGTCNNNNNNGGGGT	23.09	DR:6

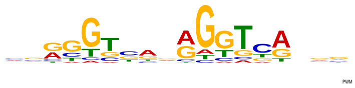	NNNNGTCAGGGGTCNNNN	9.78	DR:1
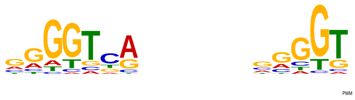	GGGGTCNNNNNNNGGGGT	28.75	DR:7
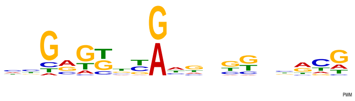	NNNGGGTCNNGGGTCNN	4.02	DR:2
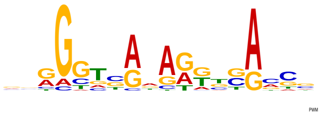	NNNNNGGTCAAGGGTNNNNN	7.17	DR:1
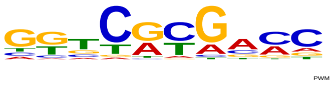	GGTCATGACC	15.06	IR:0
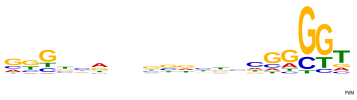	GGGTCNNNNNNNNNNGGGGT	11.77	DR:10
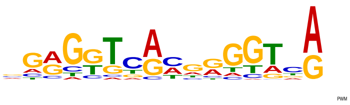	NNGGGTCNNGGGGTCNN	11.58	DR:2

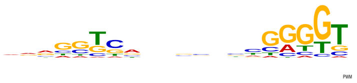	*AAAGGNNNNNNNNNNGGGGT*	9.81	DR:8
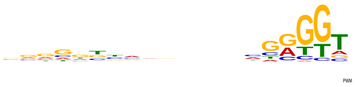	*CGGGGNNNNNNNNNNGGGGT*	14.83	M
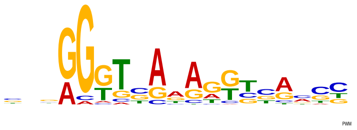	*NNNNGGTCAAGGGTCNNNN*	18.69	DR:1
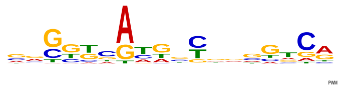	*NNGGGTCNNNNNGGGGTNN*	4.64	DR:5
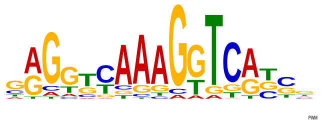	*NNNNGGGCAAAGGTCNNNN*	25.65	DR:1
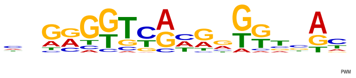	*NNGGGGTCNNNGGGTCNN*	10.10	DR:2

77 RXRG Round 3

PWM	Seed Sequence	Seed Seq Enrichment	Repeat
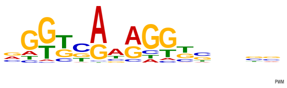	*NNNNNGGTCAAAGGTNNNNN*	154.50	DR:1
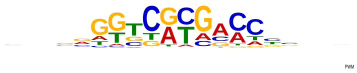	*NNNNNGGTCATGACCNNNNN*	176.74	IR:0
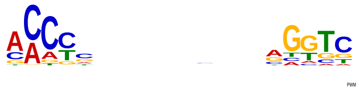	*ACCCCNNNNNNNNNNAGGTC*	133.05	ER:11
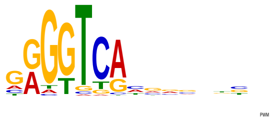	*NNNNNGGGGTCACGGNNNNN*	91.92	M
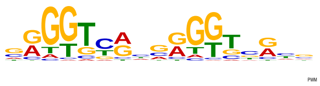	*NNNGGGTCNNGGGTCNN*	75.58	DR:2
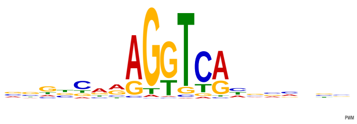	*NNNNNAAAGGTCACGNNNNN*	73.71	M
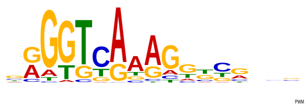	*NNNNGGGTCNAAGGGCNNNN*	177.10	DR:1

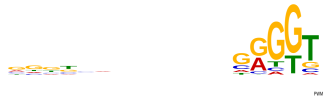	*CGGGGNNNNNNNNNNGGGGT*	80.70	M
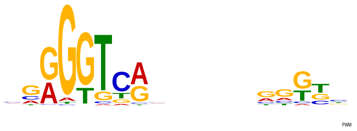	*NGGGGTCNNNNNNGGGGTN*	100.77	DR:6
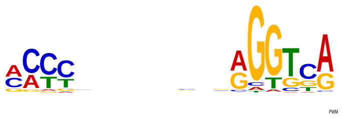	*NACCCCCNNNNNNNNAGGTCN*	43.14	ER:9
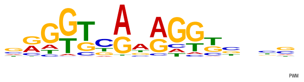	*NNNNGGGTCNAAGGTNNNN*	105.52	DR:1
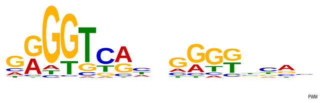	*NNNGGGTCNNNGGGGTNNN*	46.15	DR:3
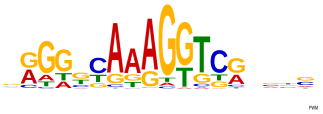	*NNNNGGGCAAAGGTCGNNNN*	320.44	DR:1
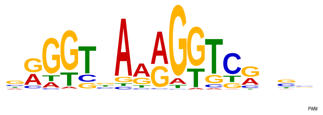	*NNNGGGTGANAGGTCGNNN*	270.07	DR:1

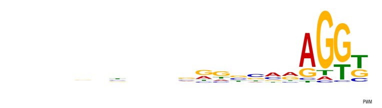	GGGGGNNNNNNNNNNAAAGGT	114.55	M
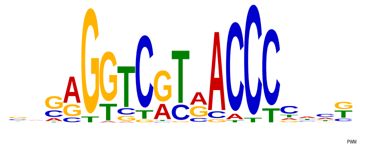	NNNAGGTCGTAACCCCNNN	591.81	IR:0
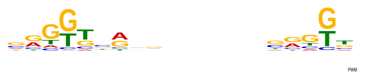	GGGGTCANNNNNNNNGGGGT	201.66	DR:9
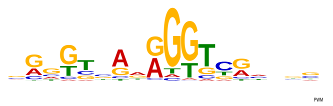	NNNNNGTCAGGGGTCNNNNN	60.19	DR:1
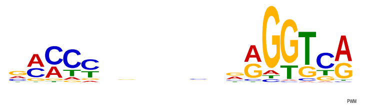	NACCCCNNNNNNNAGGTCN	34.15	ER:8
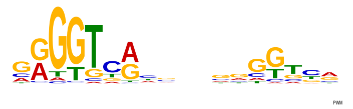	NNGGGTCNNNNNGGGGTNN	47.02	DR:5

78 RXRG:RXRA Round 3

PWM	Seed Sequence	Seed Seq Enrichment	Repeat
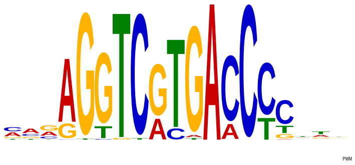	NNNAGGTCGTGACCCCNNN	4196.36	IR:0
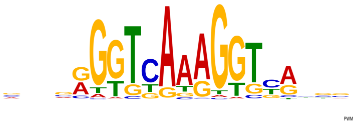	NNNNNGGTCAAAGGTNNNNN	328.88	DR:1
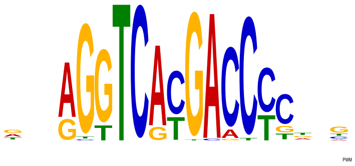	NNNAGGTCACGACCCCNNN	3245.46	IR:0
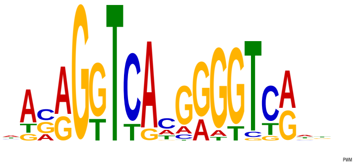	NNNNGGTCACGGGGTCNNNN	669.11	DR:2
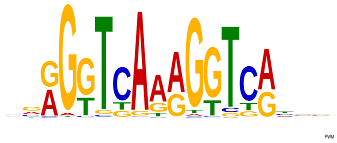	NNNNGGTCANGGGTCNNNN	156.08	DR:1
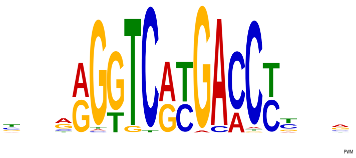	NNNNAGGTCATGACCTNNNN	1394.93	IR:0
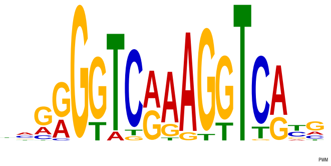	NNNGGGGTCGAAGGTCNNN	789.49	DR:1

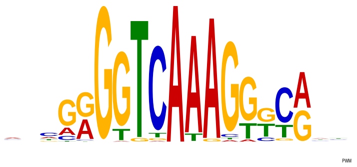	NNNGGGGTCAAAGGGCNNN	1056.56	DR:1
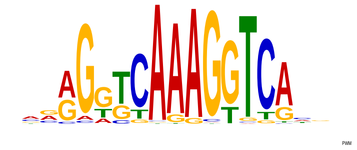	NNNNGGGCAAAGGTCNNN	285.60	DR:1
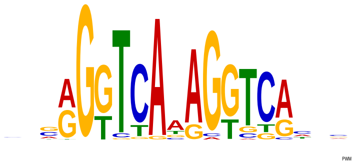	NNNNGGTCATAGGTCNNN	235.56	DR:1
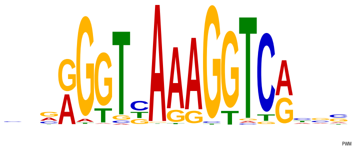	NNNNGGTGAAAGGTCNNN	346.23	DR:1
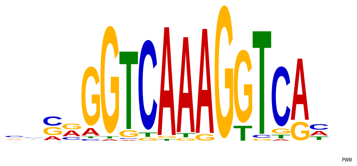	NNNCGGTCAAAGGTCNN	402.71	DR:1
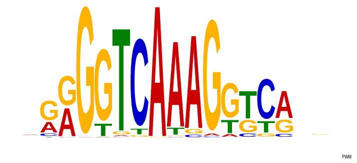	NNNNGGTCAAAGGGCNNN	303.10	DR:1
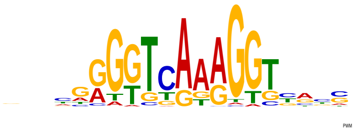	NNNNGGGGTCNAAGGTNNNN	365.80	DR:1

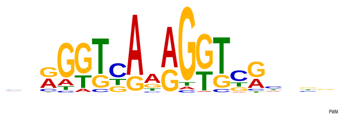	NNNNNGTCAAAGGTCNNNNN	235.72	DR:1
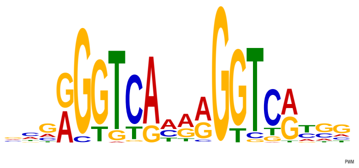	NNNNGGTCAANAGGTCNNNN	182.30	DR:2
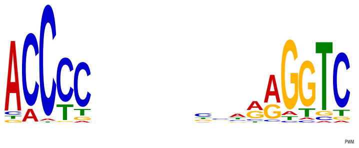	ACCCCNNNNNNNNNNAGGTC	124.02	ER:11
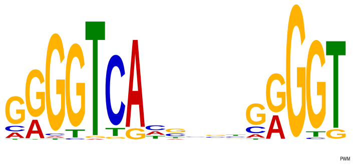	GGGGTCACNNNNGGGT	799.38	DR:6
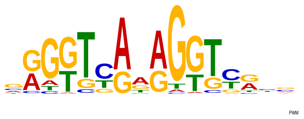	NNNGGGGTCNNAGGTCNNN	303.30	DR:1
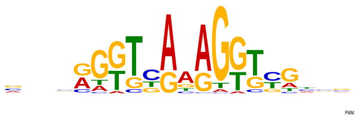	NNNNGGGTCNAAGGTCNNNN	430.00	DR:1

79 RXRG+17 Round 3

PWM	Seed Sequence	Seed Seq Enrichment	Repeat
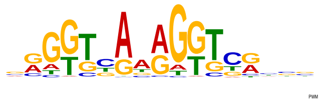	NNNNGGTCANAGGTCNNNN	185.75	DR:1
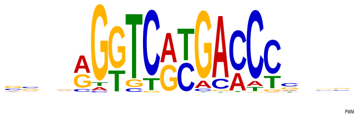	NNNNAGGTCATGACCCNNNN	1030.13	IR:0
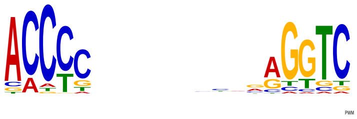	ACCCCNNNNNNNNNNAGGTC	181.20	ER:11
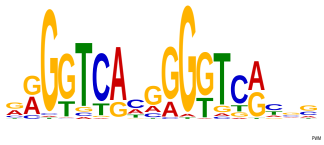	NNNNGGTCACGGGGTCNNNN	515.82	DR:2
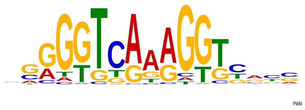	NNNNGGGGTCNAAGGTNNNN	269.66	DR:1
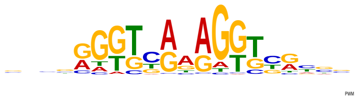	NNNNGGGTCNAAGGTCNNNN	335.60	DR:1
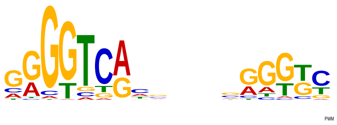	NGGGGTCNNNNNNGGGGTN	60.62	DR:6

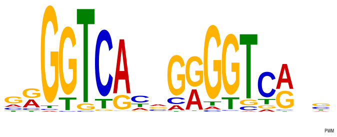	NNNGGTCACNGGGGTCNNN	260.14	DR:3
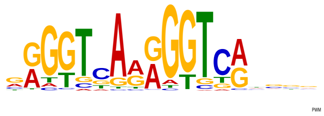	NNNNNGTCAGGGGTCNNNNN	70.28	DR:1
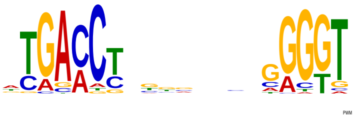	ATGACCTNNNNNNNNGGGGT	322.77	ER:9
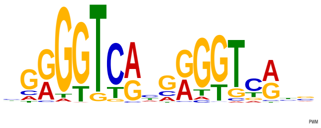	NNNGGGGTCNNNGGGTCNNN	138.25	DR:2
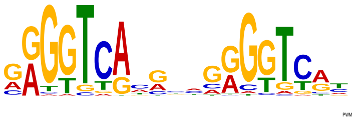	NNGGTCACNNNGGGGTCNN	154.84	DR:5
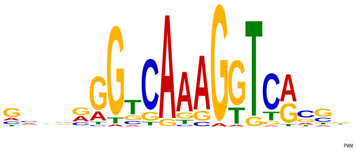	NNNNCGGTCAAAGGTCNNNN	256.61	DR:1
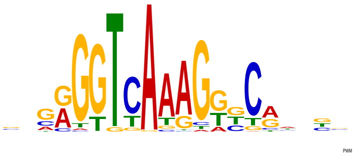	NNNNGGTCAAAGGGCANNNN	399.70	DR:1

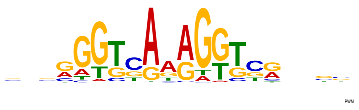	*NNNNNGTCAAAGGTCNNNNN*	178.77	DR:1
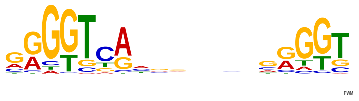	*GGGGTCANNNNNNNGGGGT*	268.88	DR:8
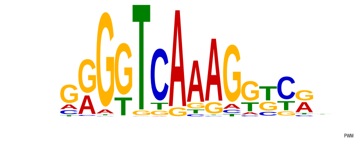	*NNNGGGGTCNAAGGGCNNN*	350.35	DR:1
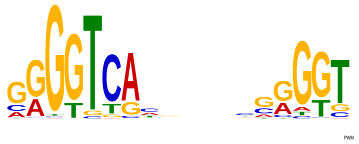	*GGGGTCACNNNNNGGGGT*	594.20	DR:7
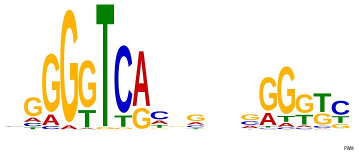	*NGGGGTCACNNNNGGGGTN*	304.02	DR:6
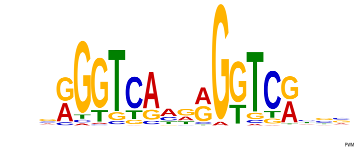	*NNNNGGTCAANAGGTCNNNN*	146.06	DR:2

80 RXRA Round 3

PWM	Seed Sequence	Seed Seq Enrichment	Repeat
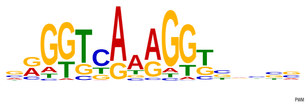	NNNNNGGTCAAAGGTNNNNN	527.24	DR:1
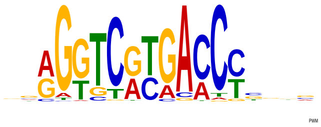	NNNNAGGTCGTGACCCNNNN	2248.08	IR:0
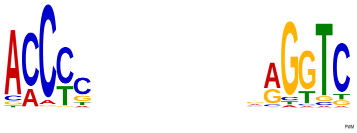	ACCCCNNNNNNNNNNNAGGTC	362.16	ER:11
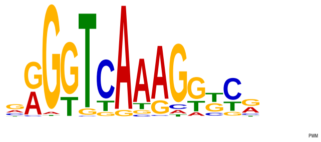	NNNNGGTCAAAGGGCNNNN	773.09	DR:1
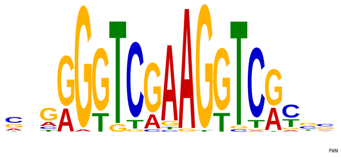	NNNGGGGTCGAAGGTCGNNN	4536.87	DR:1
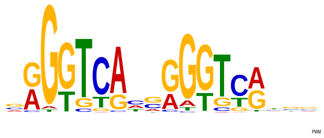	NNNNGGTCACNGGGTCNNNN	493.93	DR:2
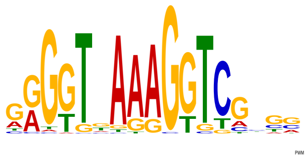	NNNGGGGTGAAAGGTCGNNN	3952.42	DR:1

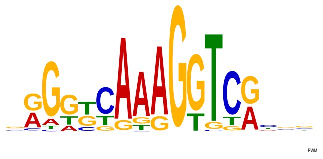	NNNNGGGCAAAGGTCNNNN	654.52	DR:1
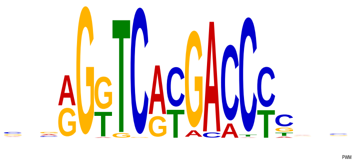	NNNAGTTCACGACCCCNNN	2824.24	IR:0
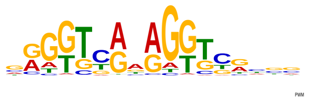	NNNNGGGTCNNAGGTCNNNN	357.77	DR:1
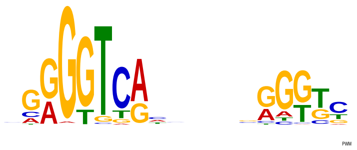	NGGGGTCNNNNNNGGGGTN	197.23	DR:6
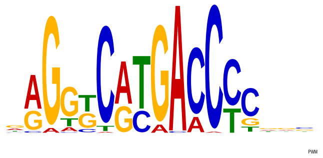	NNNAGGGCATGACCCCNNN	1637.84	IR:0
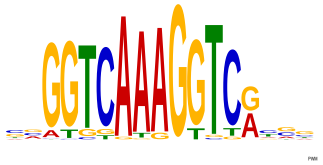	NNNCGGTCAAAGGTCGNNN	1418.65	DR:1
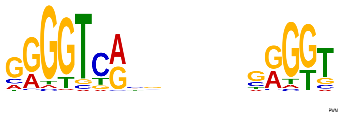	CGGGGTCNNNNNNNNGGGGT	654.28	DR:8

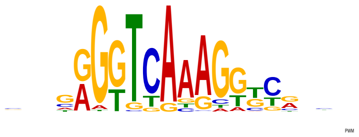	*NNNNGGGTCNAAGGGCNNNN*	520.29	DR:1
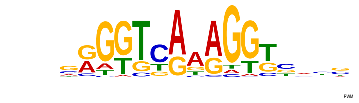	*NNNNGGGTCNAAGGTNNNN*	335.76	DR:1
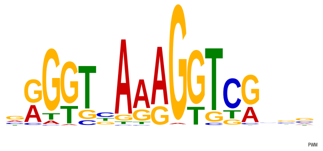	*NNNNGGTGAAAGGTCNNNN*	525.88	DR:1
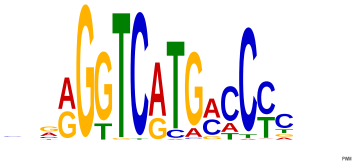	*NNNAGGTCATGCCCCCNNN*	1506.10	IR:0
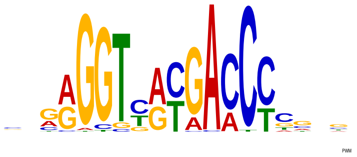	*NNNAGGTTATGACCCCNNN*	1349.85	IR:0
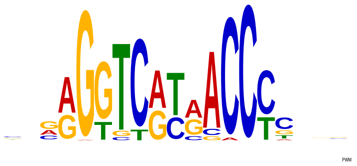	*NNNAGGTCATAACCCCNNN*	2026.13	IR:0

81 RXRA+17 Round 3

PWM	Seed Sequence	Seed Seq Enrichment	Repeat
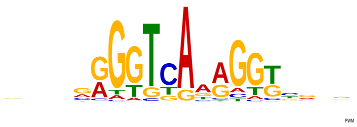	NNNNNGGGTCAAAGGNNNNNN	459.76	DR:1
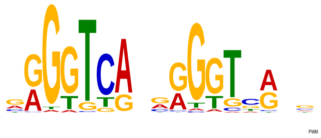	NNNGGGTCANNGGGTCNNN	874.54	DR:2
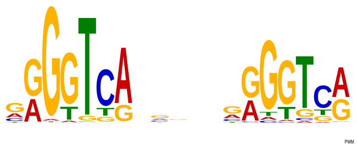	NGGGTCANNNNNNGGGTCN	627.59	DR:6
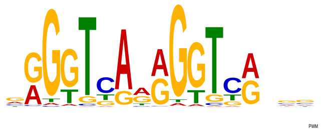	NNNNGGTCAGGGGTCNNNN	614.13	DR:1
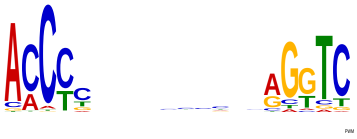	ACCCCNNNNNNNNNNNAGGTC	402.52	ER:11
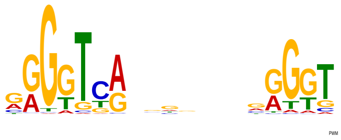	CGGGGTCANNNNNNNGGGGT	2399.34	DR:8
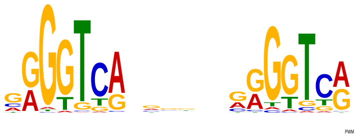	NGGGTCANNNNNNGGGGTCN	887.21	DR:7

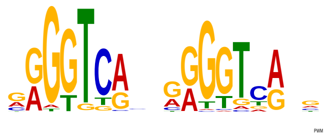	*NNNGGGTCANNNGGGTCNNN*	385.74	DR:3
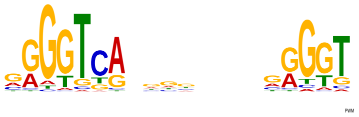	*GGGGTCANNNNNNNNNGGGGT*	1918.11	DR:9
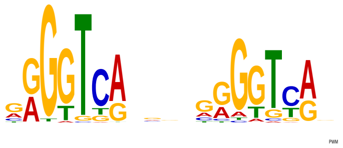	*NNGGGTCANNNNNGGGTCNN*	425.91	DR:5
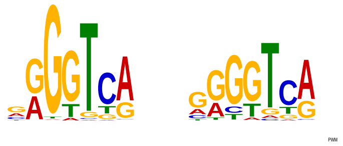	*NNGGGTCANNNGGGGTCNN*	715.35	DR:4
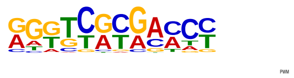	*NNNNNGGTCATGACCNNNNN*	180.43	IR:0
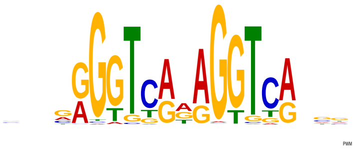	*NNNNGGGTCGNAGGTCNNNN*	531.25	DR:1
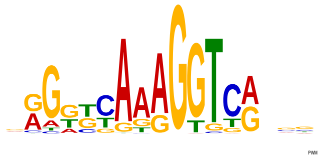	*NNNNGGGCAAAGGTCNNNN*	551.76	DR:1

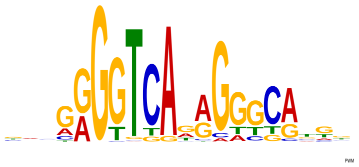	$NNNG\overrightarrow{GGGTCA}G\overrightarrow{AGGGCA}NNN$	2637.36	DR:1
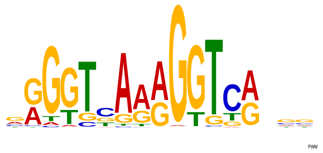	$NNN\overrightarrow{NGGTGA}N\overrightarrow{AGGTCN}NNN$	242.84	DR:1
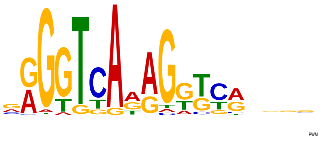	$NNN\overrightarrow{NGGTCA}N\overrightarrow{AGGGCA}NNNN$	442.05	DR:1
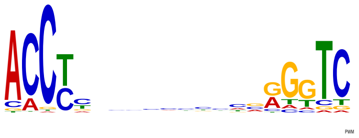	$\overleftarrow{ACCTT}NNNNNNNNNN\overrightarrow{GGGTC}$	152.64	ER:11
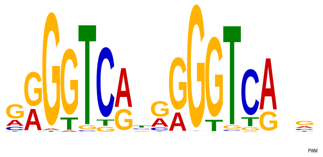	$NNN\overrightarrow{GGGTCG}TN\overrightarrow{GGGTCN}NN$	472.42	DR:2
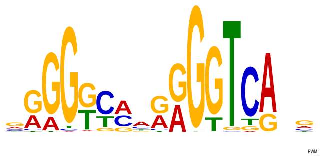	$NNN\overrightarrow{GGGTTN}NG\overrightarrow{GGGTCN}NN$	266.93	DR:2

82 NOR1 Round 3

PWM	Seed Sequence	Seed Seq Enrichment	Repeat
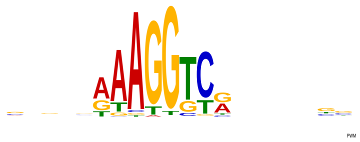	*NNNNNAAAGGTCGCGNNNNN*	80.10	M
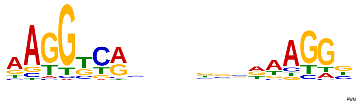	*AAGGTCNNNNNNNNNAAGGG*	165.31	DR:9
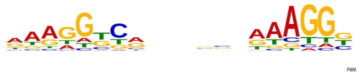	*AAAGGNNNNNNNNNNAAGGG*	76.64	DR:8
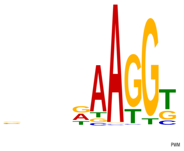	*GGGGGNNNNNNNNNNAAGGT*	67.99	M
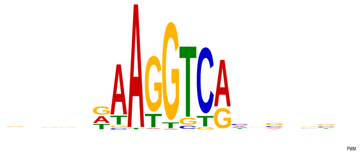	*NNNNNGAAGGTCACGNNNNN*	87.26	M
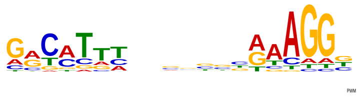	*GGCATTNNNNNNNNNAAGGG*	87.12	DR:12
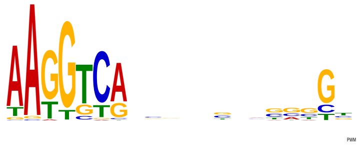	*AAGGTCANNNNNNNNGGGGT*	167.41	DR:9

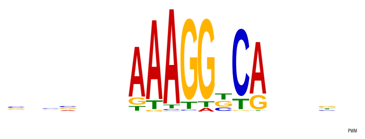	$NNNNNCTAA\overrightarrow{AGGGCA}NNNNN$	40.68	M
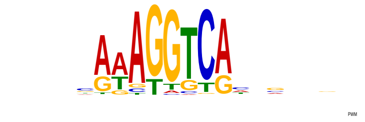	$NNNNNAT\overrightarrow{AGGTCA}CGNNNNN$	43.18	M
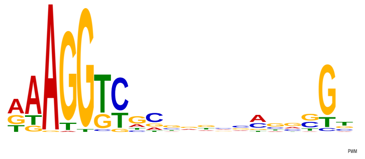	$AA\overrightarrow{AGGTCG}NNNNNNNG\overrightarrow{GGGT}$	311.85	DR:8
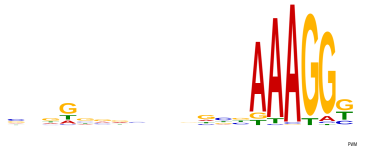	$CCGGGNNNNNNNNNAA\overrightarrow{AGGG}$	97.49	M
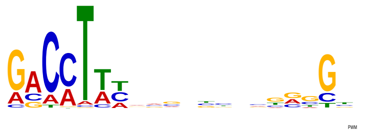	$\overleftarrow{GACCT}TNNNNNNNNNG\overrightarrow{GGGT}$	105.49	ER:11
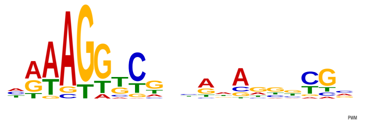	$NNA\overrightarrow{AGGTCN}NNNA\overrightarrow{AGGGCN}N$	101.74	DR:4
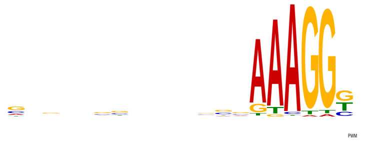	$\overrightarrow{GGGGCN}NNNNNNNNAA\overrightarrow{AGGG}$	108.23	DR:10

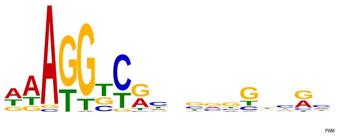	*NNAAGGTCNNNGGGGTCNN*	71.88	DR:3
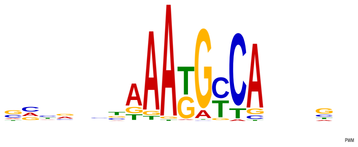	*NNNNNCTAAATGCCANNNNN*	32.19	M
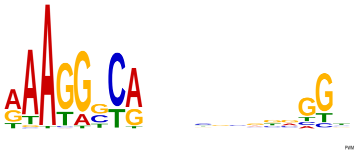	*AAAGGGCANNNNNNNGGGGT*	277.10	DR:8
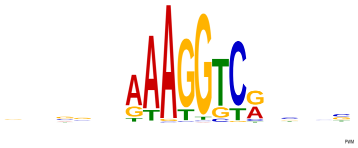	*NNNNNCTAAAGGTCGNNNNN*	50.05	M
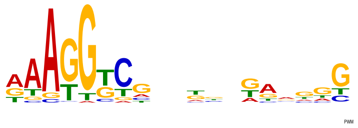	*AAAGGTCGNNNNNNAAGGG*	330.83	DR:7
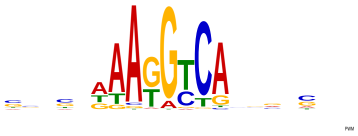	*NNNNNAAATGTCACGNNNNN*	43.08	M

83 NURR1 Round 3

PWM	Seed Sequence	Seed Seq Enrichment	Repeat
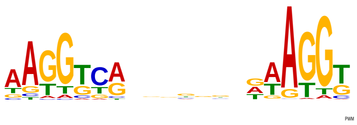	AAGGTCNNNNNNNNNAAGGT	1232.42	DR:9
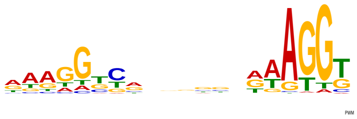	AAAGGNNNNNNNNNNAAGGT	644.46	DR:8
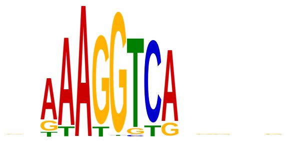	NNNNNAAAGGTCACGNNNNN	480.38	M
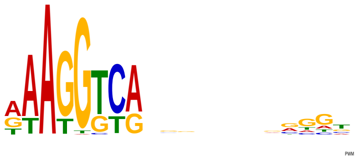	AAAGGTCANNNNNNNGGGGT	2976.80	DR:8
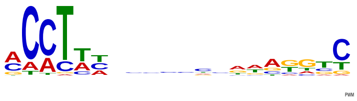	ACCTTNNNNNNNNNNAGGTC	355.28	ER:11
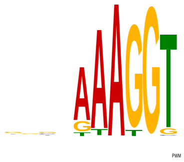	CGGGGNNNNNNNNAAAGGT	676.92	M
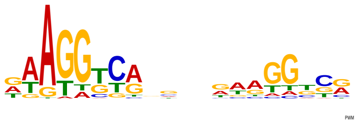	NAAGGTCANNNNNNAGGTCN	908.14	DR:6

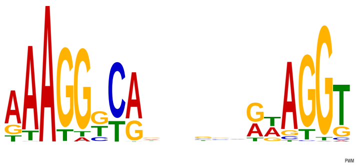	*AAAGGGCANNNNNNNGTAGGT*	5366.36	DR:8
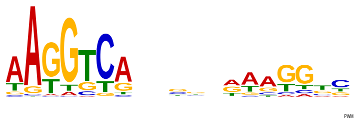	*AAGGTCANNNNNNNAGGTC*	533.12	DR:7
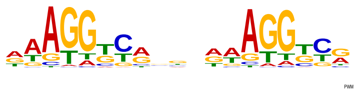	*NAAGGTCNNNNNAAGGTCN*	742.57	DR:5
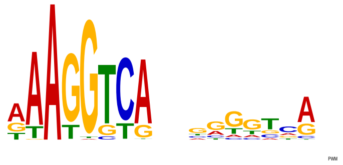	*NNAAGGTCANNGGGGTCNN*	1135.26	DR:3
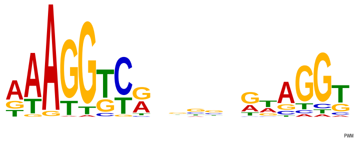	*AAAGGTCNNNNNNGTAGGT*	1858.72	DR:7
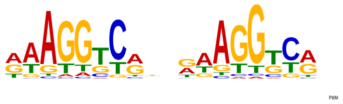	*NNAAGGTCNNNNAAGGTCNN*	734.07	DR:4
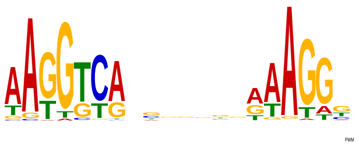	*AAGGTCANNNNNNNNAAAGGG*	3422.16	DR:9

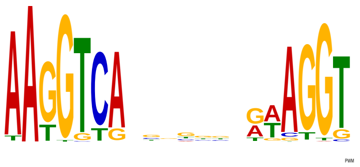	$A\overrightarrow{AGGTCA}NNNNNNNGT\overrightarrow{AGGT}$	3762.74	DR:9
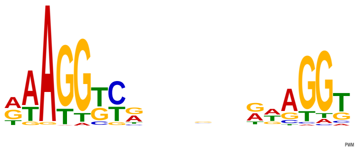	$AA\overrightarrow{AGGTCN}NNNNNNGT\overrightarrow{AGGT}$	3150.63	DR:8
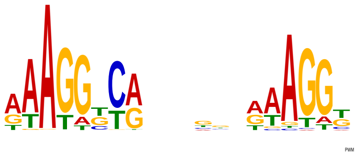	$AA\overrightarrow{AGGGCA}NNNNNNNA\overrightarrow{AGGG}$	2671.38	DR:8
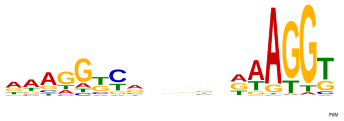	$CAA\overrightarrow{AGGNNN}NNNNNNA\overrightarrow{AGGT}$	531.12	DR:7
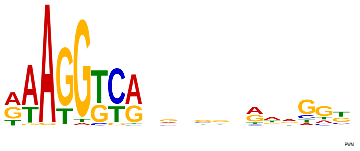	$AA\overrightarrow{AGGTCA}NNNNNNNT\overrightarrow{AGGG}$	2076.70	DR:8
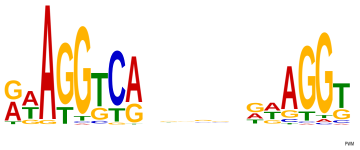	$AT\overrightarrow{AGGTCA}NNNNNNNT\overrightarrow{AGGT}$	1987.57	DR:8
