# Supplementary material for: Hidden modes of DNA binding by human nuclear receptors
Source: Nat Commun. 2023 Jul 13;14:4179. doi: 10.1038/s41467-023-39577-0 (PMC10345098; doi:10.1038/s41467-023-39577-0)
Supplement: Supplementary file 6 — Supplementary Data 4 [file 41467_2023_39577_MOESM6_ESM.pdf]

Supplementary Data 4: Monomer binding of Nuclear Receptors

NR	PWM	Seed Sequence	Seed Seq Enrichment
MR Round 3	No Monomer motif	0	0
MR+1 Round 3	No Monomer motif	0	0
PGR Round 3	No Monomer motif	0	0
PGR+2 Round 3	No Monomer motif	0	0
GR Round 3	No Monomer motif	0	0
GR+3 Round 3	No Monomer motif	0	0
ESRRG Round 3	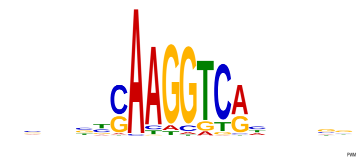	NNNNNTCAAGGTCATNNNNN	46.46
ESRRG:RXRA Round 3	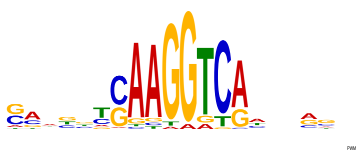	NNNNNTCAAGGTCATNNNNN	44.81
ESRRG+4 Round 3	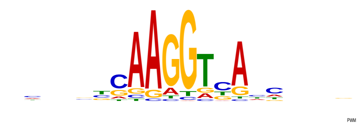	NNNNNCCAAGGTCACNNNNN	28.14
ESRRB Round 3	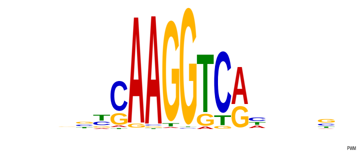	NNNNNTCAAGGTCATNNNNN	355.99
ESRRB:RXRA Round 3	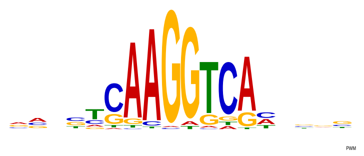	NNNNTCAAGGTCAANNNN	29.82
ESRRB+4 Round 3	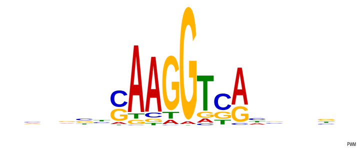	NNNNNTCAAGGTCACNNNNN	67.48
ESRRA Round 3	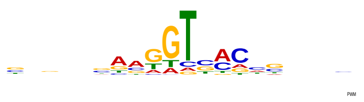	NNNNNCAAGGTGACCNNNNN	74.40
ESRRA:RXRA Round 3	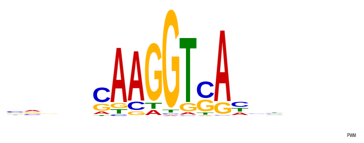	NNNNNCAAGGTCACCNNNNN	487.45
ESRRA+4 Round 3	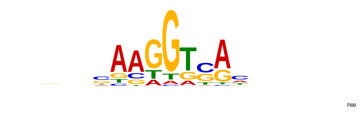	NNNNNCAAGGTCACCNNNNN	92.24

ESR1+5 Round 3	No Monomer motif	0	0
ESR1+6 Round 3	No Monomer motif	0	0
THRB Round 3	No Monomer motif	0	0
THRB:RXRA Round 3	No Monomer motif	0	0
THRB:RXRA+7 Round 2	No Monomer motif	0	0
THRB+7 Round 3	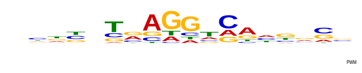	$NNNNTTA\overrightarrow{AGGTCA}CNNNN$	6.06
THRA Round 3	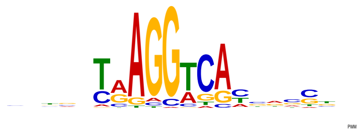	$NNNNNTA\overrightarrow{AGGTCA}CGNNNNN$	26.67
THRA:RXRA Round 3	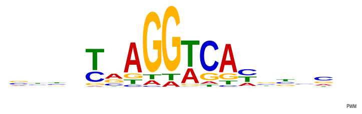	$NNNNTA\overrightarrow{AGGTCA}CGNNNN$	16.62
THRA:RXRA+7 Round 2	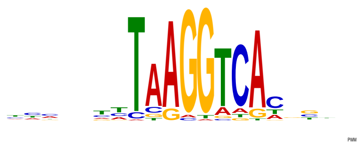	$NNNNNTTTA\overrightarrow{AGGTCA}NNNNN$	72.07
THRA+7 Round 3	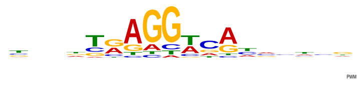	$NNNNTA\overrightarrow{AGGTCA}CGNNNN$	6.71
RARG Round 3	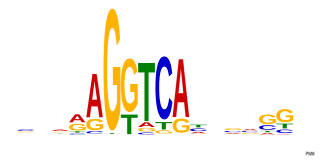	$NNNNNAA\overrightarrow{AGGTCA}CGNNNNN$	171.95
RARB Round 3	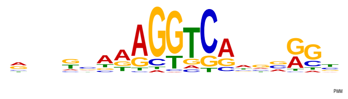	$NNNNNCAA\overrightarrow{AGGTCA}ANNNNN$	14.74
RARA Round 3	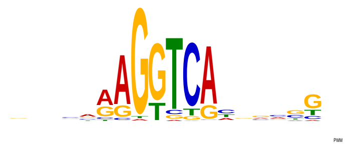	$NNNNNAA\overrightarrow{AGGTCA}CGNNNNN$	126.65
RARG+8 Round 2	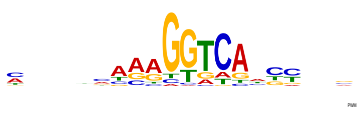	$NNNNNCAA\overrightarrow{AGGTCA}TNNNNN$	13.97

RARB+8 Round 3	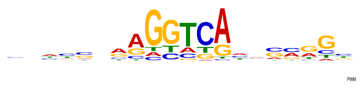	NNNNNAAAGGTCACGNNNNN	14.12
RARA+8 Round 3	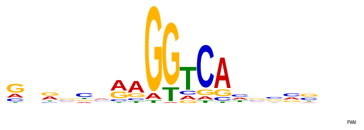	NNNNNAAAGGTCACGNNNNN	16.52
RARG:RXRA Round 3	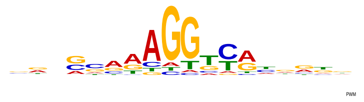	NNNNCAAAGGTCATNNNN	10.55
RARB:RXRA Round 3	No Monomer motif	0	0
RARA:RXRA Round 3	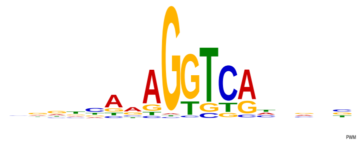	NNNNCAAAGGTCATNNNN	44.72
PXR Round 3	No Monomer motif	0	0
PXR+9 Round 3	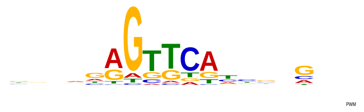	NNNNGAGTTCATCGNNNN	12.29
VDR Round 3	No Monomer motif	0	0
VDR:RXRA Round 3	No Monomer motif	0	0
VDR+10 Round 2	No Monomer motif	0	0
FXR Round 3	No Monomer motif	0	0
FXR+11 Round 3	No Monomer motif	0	0
LXRA Round 3	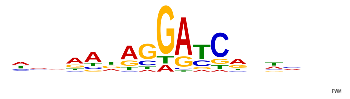	NNNNAAGAGNATCAANNNN	5.92
LXRA+12 Round 3	No Monomer motif	0	0
LXRB:RXRA Round 3	No Monomer motif	0	0
PPARD Round 3	No Monomer motif	0	0
PPARD+13 Round 3	No Monomer motif	0	0
PPARD+14 Round 3	No Monomer motif	0	0
PPARG Round 3	No Monomer motif	0	0
PPARG+14 Round 3	No Monomer motif	0	0
PPARG+15 Round 3	No Monomer motif	0	0
Rev-ErbA-Alpha Round 3	No Monomer motif	0	0
RORC Round 3	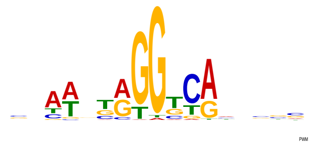	NNNNNNAACTGGGTCANNNNN	62.64

RORC:RXRA Round 3	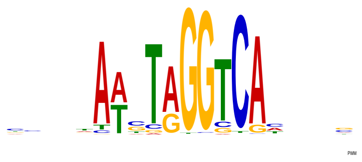	$NNN\overleftarrow{NNAACT}\,\overrightarrow{AGGTCA}NNNNN$	420.22
RORC+16 Round 3	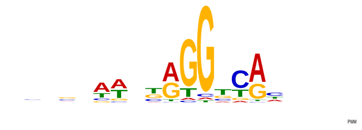	$NNN\overleftarrow{NNAACT}\,\overrightarrow{GGGTCA}NNNNN$	22.80
TR4 Round 3	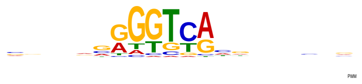	$NNNNNG\overrightarrow{GGGTCA}CGGNNNNN$	29.23
TR2 Round 3	No Monomer motif	0	0
LRH1 Round 3	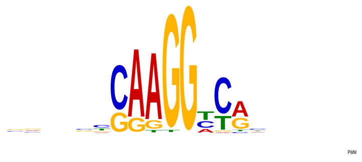	$NNNNNCCA\overrightarrow{AGGTCA}CNNNNN$	151.05
LRH1:RXRA Round 3	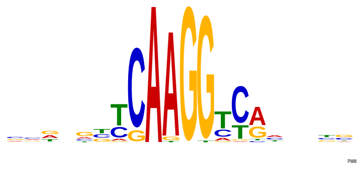	$NNNN\overrightarrow{NTTCA}\,\overrightarrow{AGGTCA}NNNNN$	161.43
SF1 Round 3	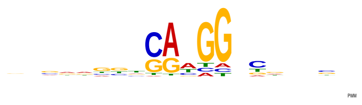	$NNNN\overrightarrow{NGGTCA}\,\overrightarrow{AGGCCN}NNNN$	81.83
SF1:RXRA Round 3	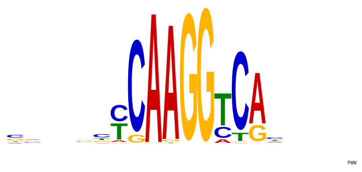	$NNNNNCCCA\overrightarrow{AGGTCA}NNNNN$	1811.77
TLX Round 3	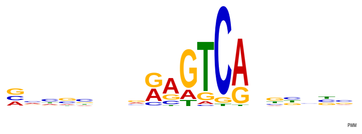	$NNNNNC\overrightarrow{AGGAGT}CAANNNNN$	24.71
TLX:RXRA Round 3	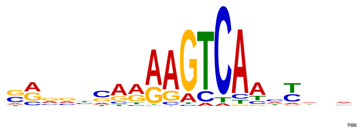	$NNNNNCAA\overrightarrow{AAGTCA}ANNNNN$	48.86
PNR Round 3	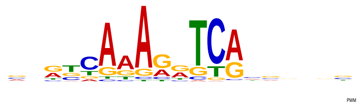	$NNNNCAA\overrightarrow{AGNTCA}CGNNNN$	107.43

COUP-TF2 Round 3	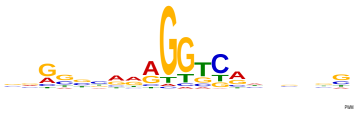	$NNNNNCAA\overrightarrow{AGGTC}AANNNNN$	43.23
COUP-TF1 Round 3	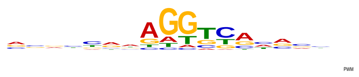	$NNNNCAA\overrightarrow{AGGTC}AANNNN$	9.96
EAR2 Round 3	No Monomer motif	0	0
COUP-TF2+17 Round 3	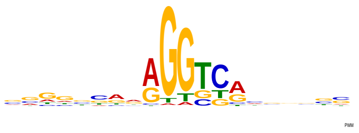	$NNNNNCAA\overrightarrow{AGGTC}AANNNNN$	132.79
COUP-TF1+17 Round 3	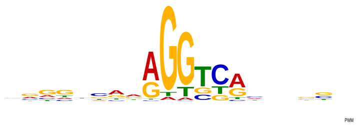	$NNNNNCAA\overrightarrow{AGGTC}AANNNNN$	121.07
COUP-TF2:RXRA Round 3	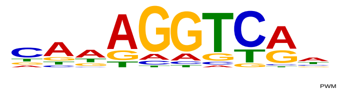	$CAA\overrightarrow{AGGTC}AA$	28.07
COUP-TF1:RXRA Round 3	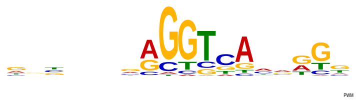	$NNNNC\overrightarrow{AGGGG}TCAANNNN$	10.35
EAR2:RXRA Round 3	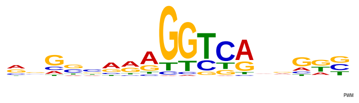	$NNNNCAA\overrightarrow{AGGTC}AANNNN$	23.75
HNF4G Round 3	No Monomer motif	0	0
HNF4A Round 3	No Monomer motif	0	0
HNF4A+18 Round 3	No Monomer motif	0	0
RXRB Round 3	No Monomer motif	0	0
RXRB+17 Round 3	No Monomer motif	0	0
RXRG Round 3	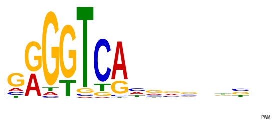	$NNNNNG\overrightarrow{GGGTC}ACGGNNNNN$	91.92
RXRG:RXRA Round 3	No Monomer motif	0	0
RXRG+17 Round 3	No Monomer motif	0	0
RXRA Round 3	No Monomer motif	0	0
RXRA+17 Round 3	No Monomer motif	0	0

NOR1 Round 3	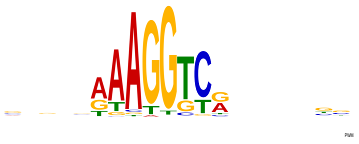	$NNNNNAA\overrightarrow{AGGTCG}CGNNNNN$	80.10
NURR1 Round 3	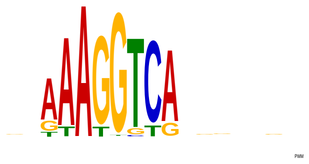	$NNNNNAA\overrightarrow{AGGTCA}CGNNNNN$	480.38
