# Supplementary material for: Hidden modes of DNA binding by human nuclear receptors
Source: Nat Commun. 2023 Jul 13;14:4179. doi: 10.1038/s41467-023-39577-0 (PMC10345098; doi:10.1038/s41467-023-39577-0)
Supplement: Supplementary file 8 — Supplementary Data 6 [file 41467_2023_39577_MOESM8_ESM.pdf]

Supplementary Data 6: Gapped-SELs for all Nuclear Receptors

Contents

1 Steroid-Hormone-Receptor FAMILY 2

2 ERR FAMILY 3

3 ER FAMILY 5

4 THR FAMILY 6

5 RAR FAMILY 7

6 VDR FAMILY 9

7 LXR FAMILY 10

8 PPAR FAMILY 11

9 RORC FAMILY 12

10 TR2/TR4 FAMILY 13

11 SF1/LRH1 FAMILY 14

12 TLX/PNR FAMILY 15

13 COUP/EAR FAMILY 16

14 HNF4 FAMILY 17

15 RXR FAMILY 18

16 NURR1/NOR1 FAMILY 19

1 Steroid-Hormone-Receptor FAMILY

GNACR

NR	DR	IR	ER
MR	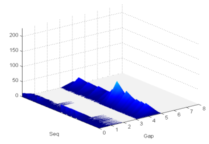	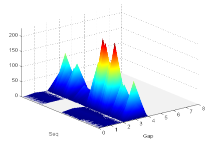	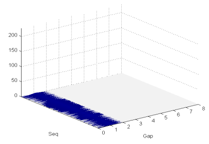
MR+1	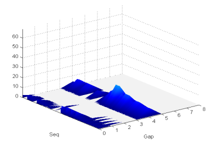	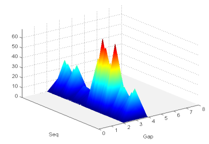	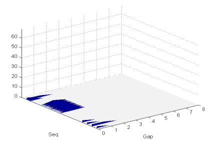
PGR	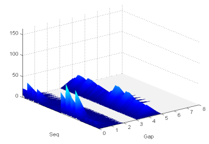	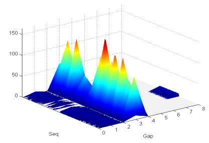	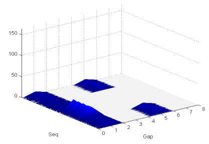
PGR+2	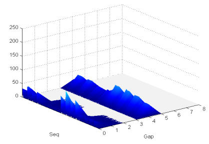	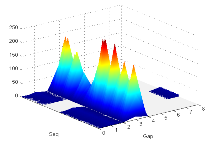	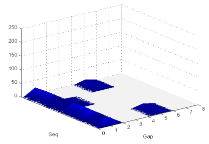
GR	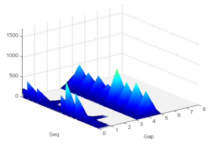	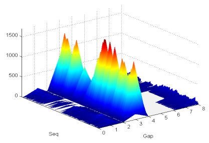	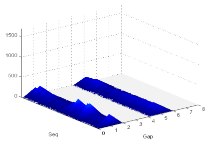
GR+3	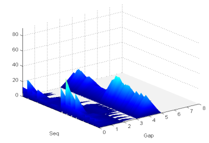	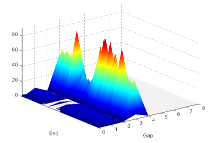	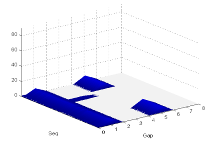

2 ERR FAMILY

RGGTCR

NR	DR	IR	ER
ESRRG	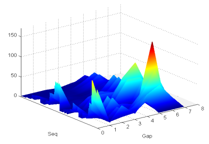	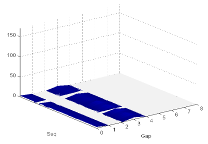	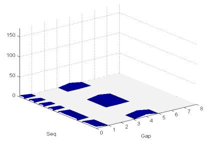
ESRRG:RXRA	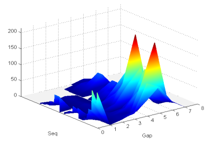	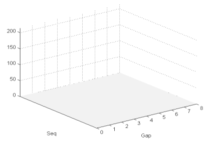	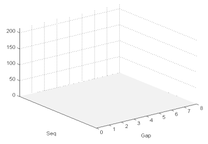
ESRRG+4	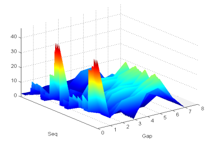	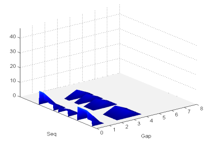	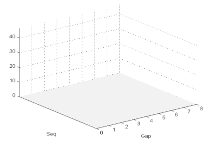
ESRRB	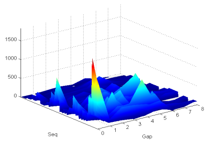	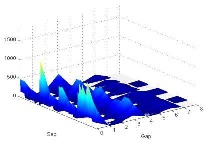	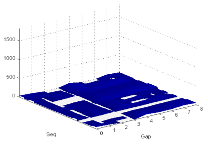
ESRRB:RXRA	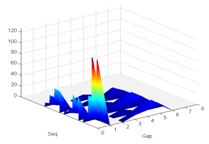	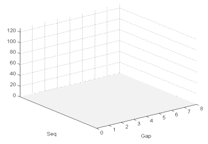	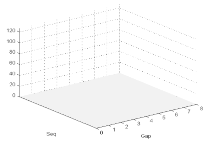
ESRRB+4	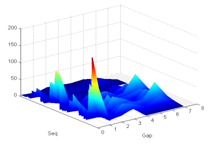	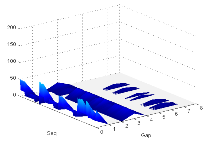	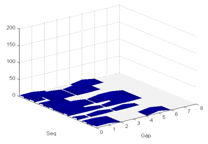
ESRRA	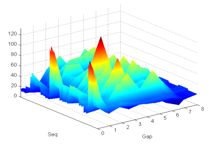	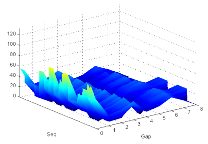	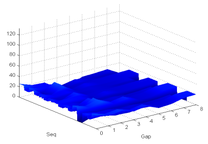
ESRRA:RXRA	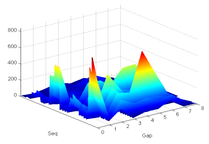	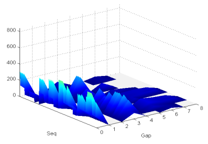	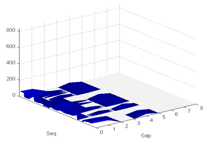

ESRRA+4	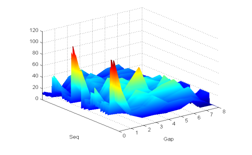	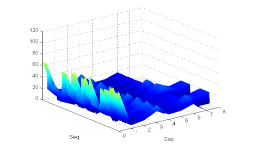	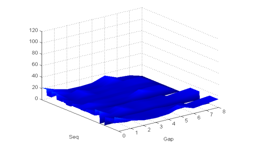

3 ER FAMILY

RGKTCR

NR	DR	IR	ER
ESR1+5	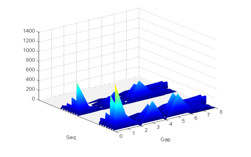	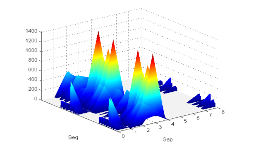	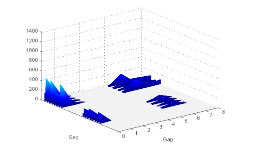
ESR1+6	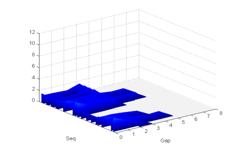	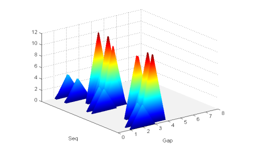	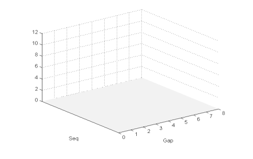

4 THR FAMILY

RGGTCR

NR	DR	IR	ER
THRB	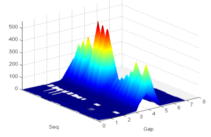	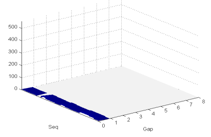	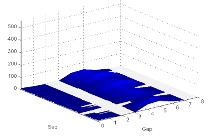
THRB:RXRA	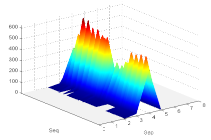	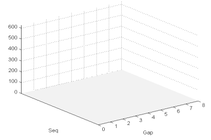	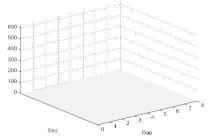
THRB:RXRA+7	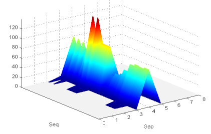	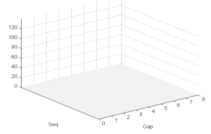	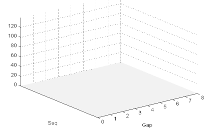
THRB+7	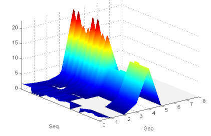	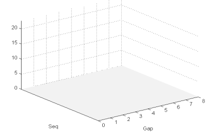	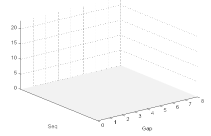
THRA	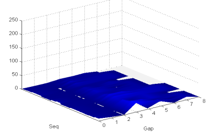	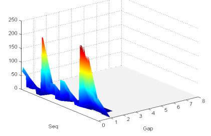	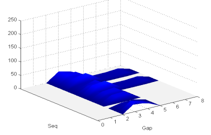
THRA:RXRA	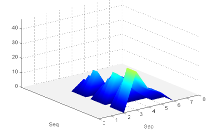	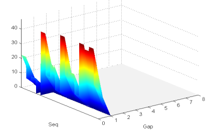	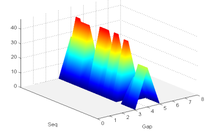
THRA:RXRA+7	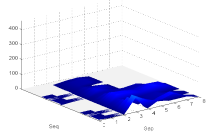	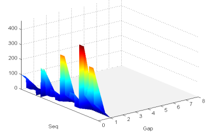	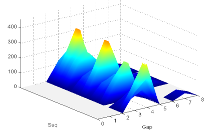
THRA+7	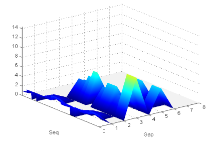	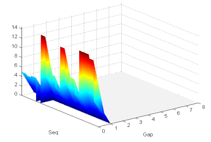	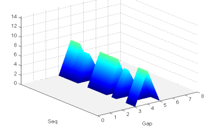

5 RAR FAMILY

RGGTCR

NR	DR	IR	ER
RARG	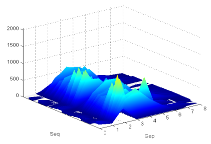	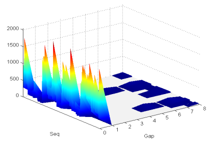	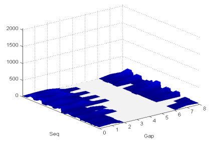
RARB	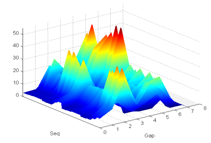	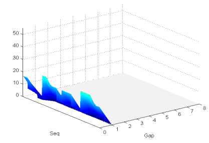	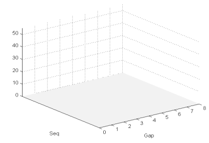
RARA	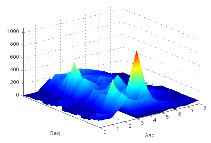	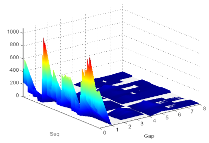	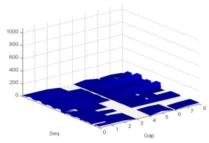
RARG+8	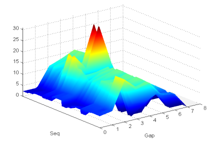	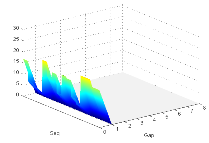	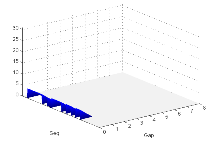
RARB+8	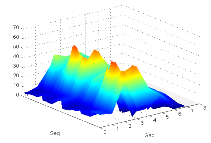	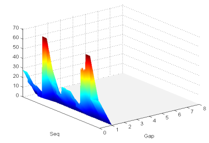	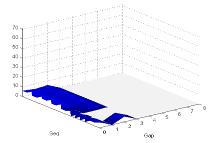
RARA+8	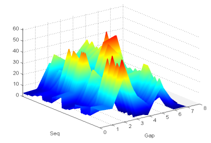	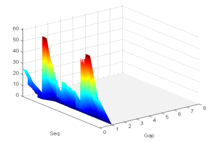	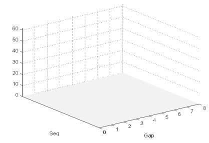
RARG:RXRA	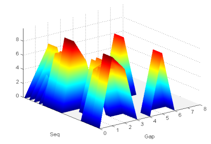	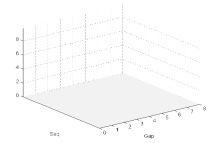	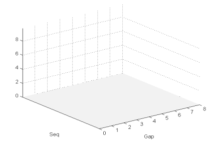
RARB:RXRA	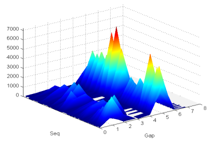	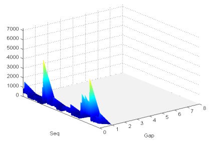	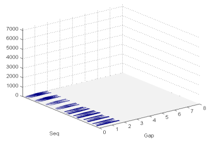

RARA:RXRA	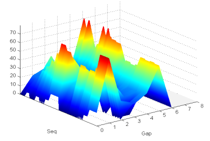	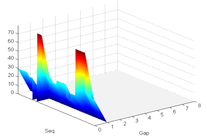	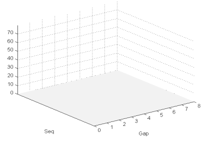

6 VDR FAMILY

RGKTCR

NR	DR	IR	ER
PXR	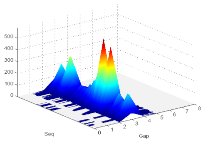	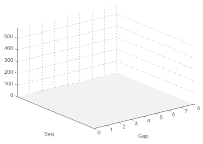	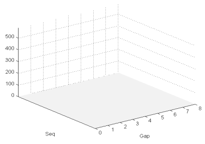
PXR+9	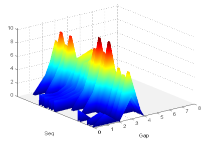	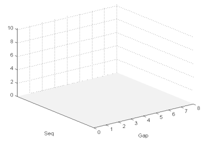	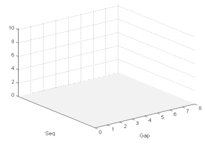
VDR	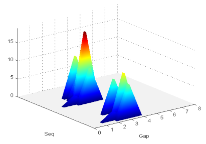	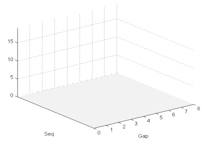	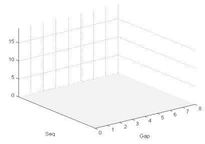
VDR:RXRA	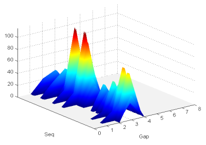	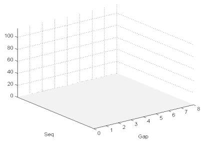	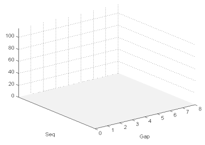
VDR+10	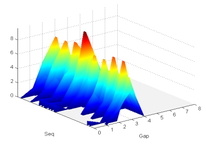	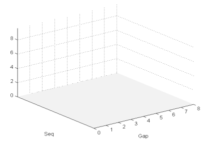	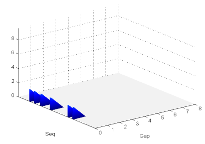

7 LXR FAMILY

RGKTCR

NR	DR	IR	ER
FXR	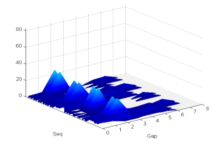	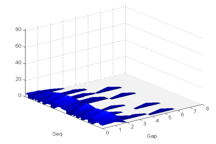	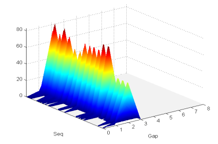
FXR+11	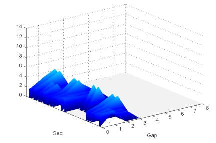	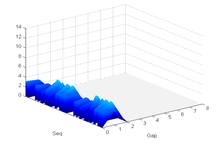	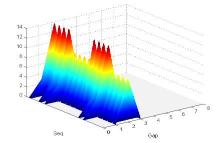
LXRA	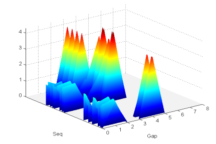	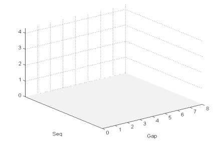	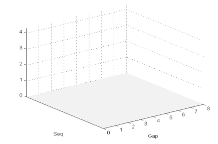
LXRA+12	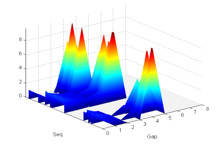	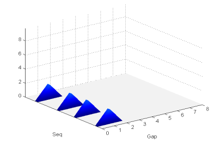	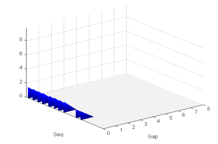
LXRB:RXRA	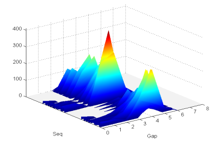	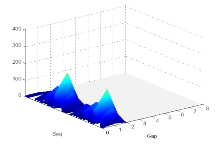	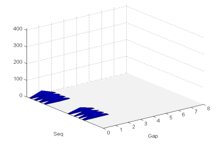

8 PPAR FAMILY

RGGTCR

NR	DR	IR	ER
PPARD	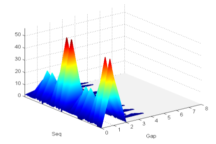	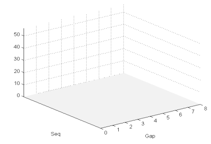	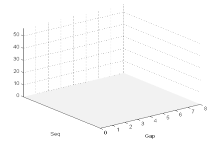
PPARD+13	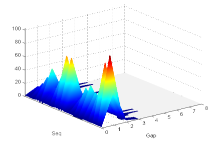	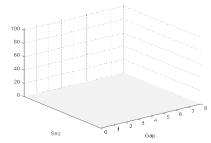	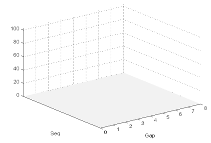
PPARD+14	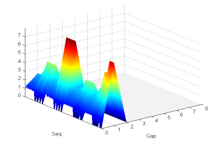	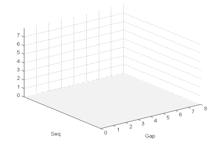	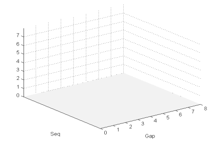
PPARG	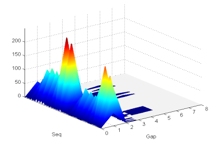	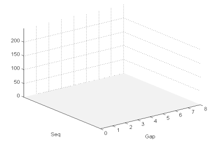	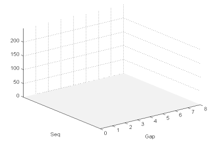
PPARG+14	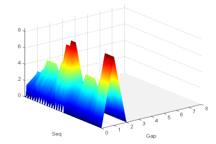	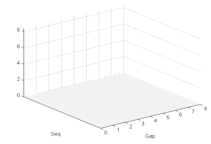	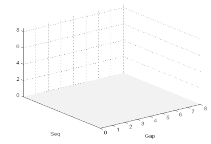
PPARG+15	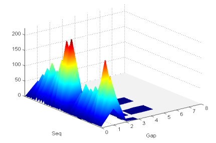	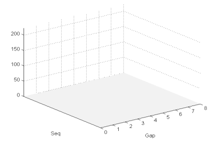	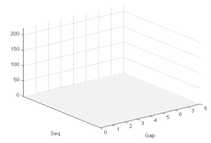

9 RORC FAMILY

RGKTCR

NR	DR	IR	ER
Rev-ErbA-Alpha	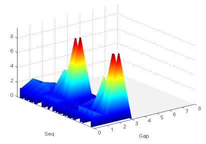	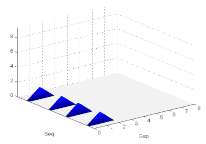	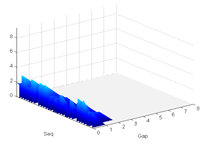
RORC	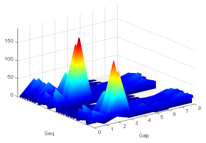	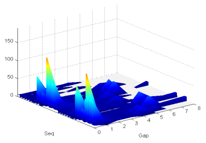	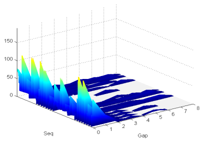
RORC:RXRA	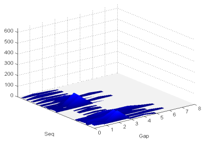	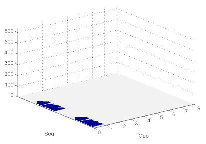	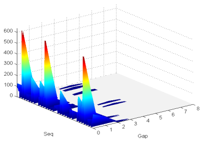
RORC+16	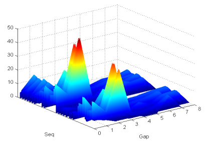	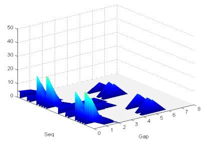	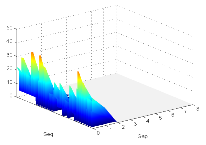

10 TR2/TR4 FAMILY

RGKTCR

NR	DR	IR	ER
TR4	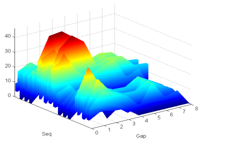	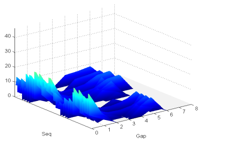	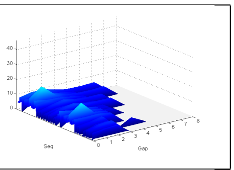
TR2	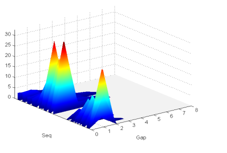	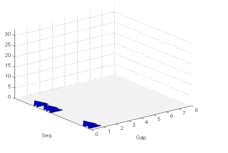	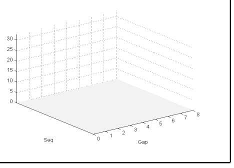

11 SF1/LRH1 FAMILY

RGKTCR

NR	DR	IR	ER
LRH1	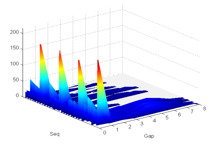	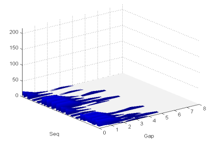	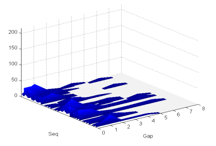
LRH1:RXRA	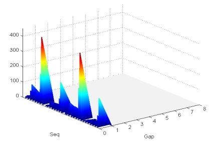	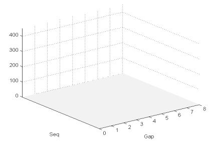	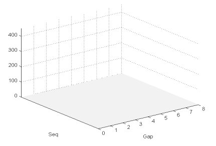
SF1	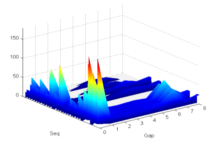	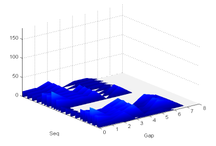	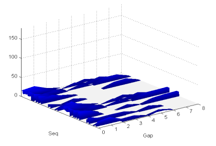
SF1:RXRA	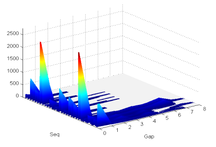	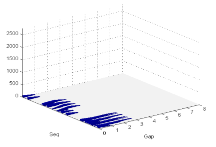	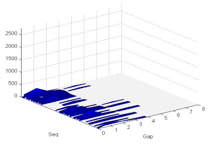

12 TLX/PNR FAMILY

RRGTCR

NR	DR	IR	ER
TLX	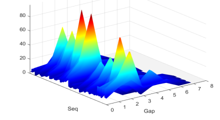	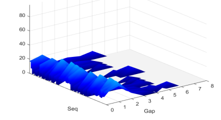	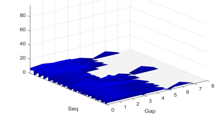
TLX:RXRA	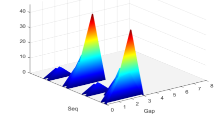	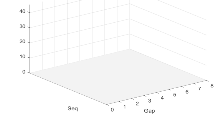	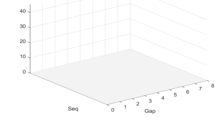
PNR	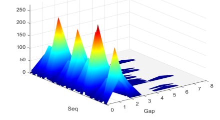	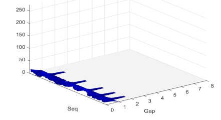	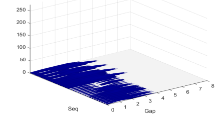

13 COUP/EAR FAMILY

RGKTCR

NR	DR	IR	ER
COUP-TF2	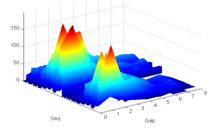	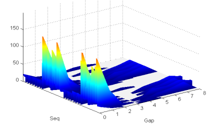	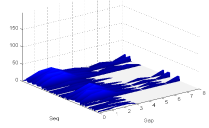
COUP-TF1	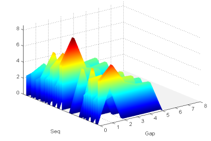	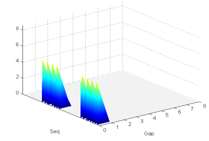	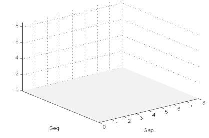
EAR2	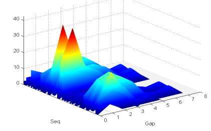	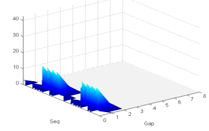	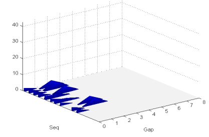
COUP-TF2+17	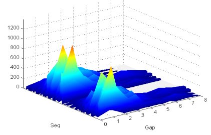	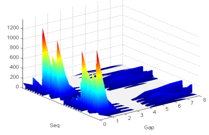	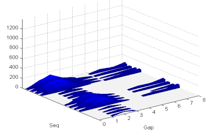
COUP-TF1+17	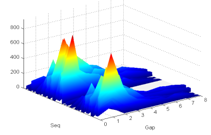	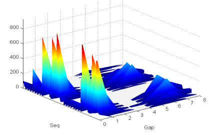	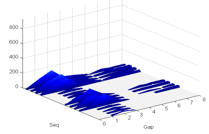
COUP-TF2:RXRA	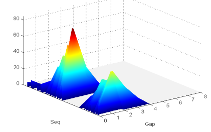	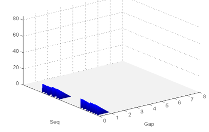	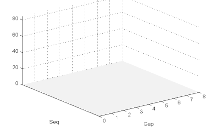
COUP-TF1:RXRA	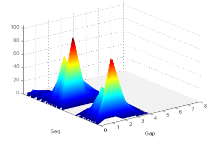	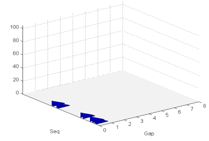	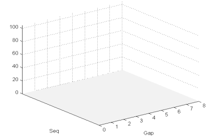
EAR2:RXRA	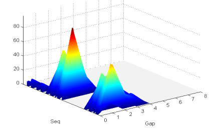	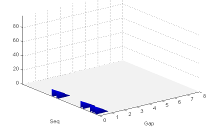	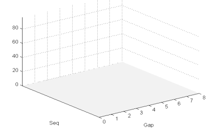

14 HNF4 FAMILY

RGKTCR

NR	DR	IR	ER
HNF4G	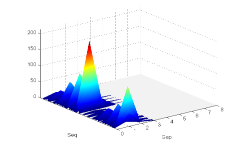	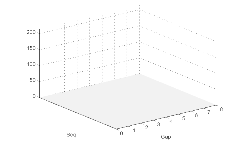	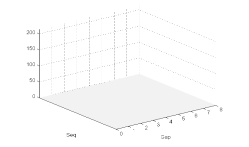
HNF4A	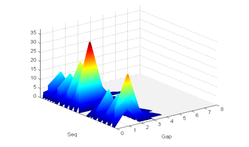	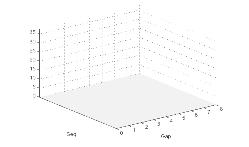	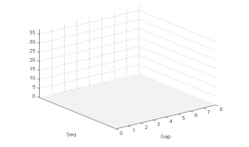
HNF4A+18	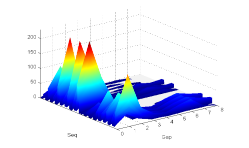	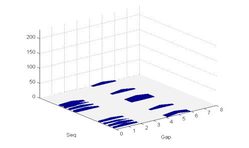	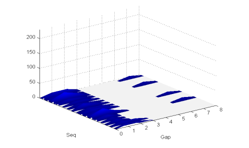

15 RXR FAMILY

RGGTCR

NR	DR	IR	ER
RXRB	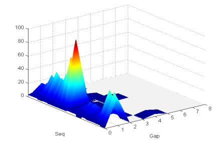	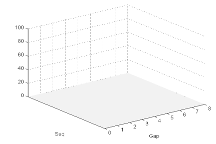	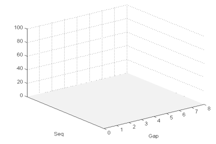
RXRB+17	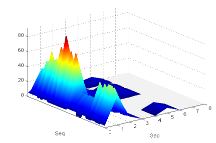	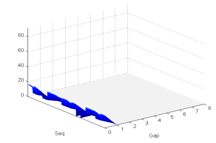	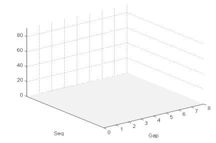
RXRG	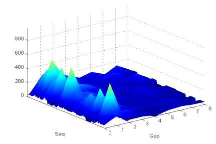	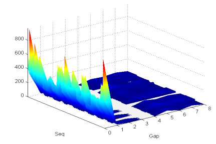	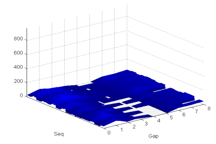
RXRG:RXRA	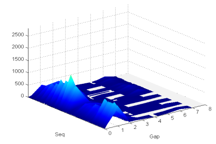	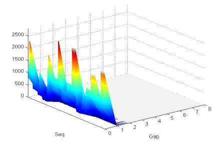	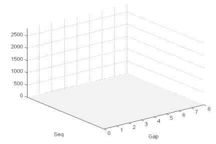
RXRG+17	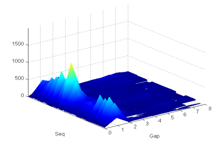	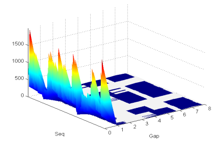	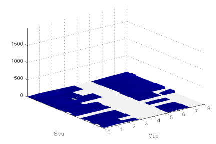
RXRA	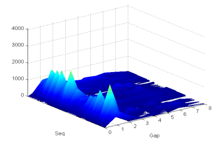	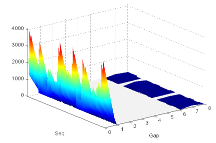	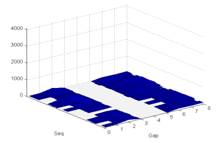
RXRA+17	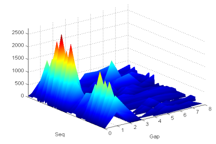	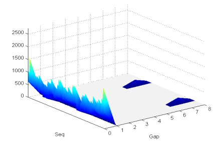	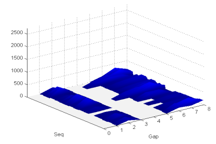

16 NURR1/NOR1 FAMILY

RGKTCR

NR	DR	IR	ER
NOR1	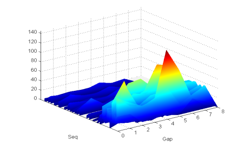	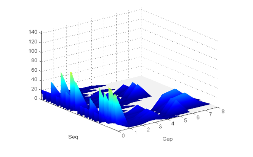	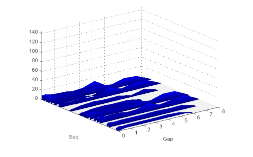
NURR1	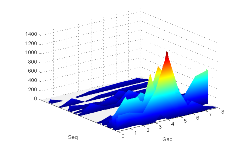	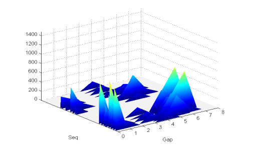	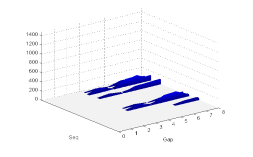
