# Supplementary material for: Hidden modes of DNA binding by human nuclear receptors
Source: Nat Commun. 2023 Jul 13;14:4179. doi: 10.1038/s41467-023-39577-0 (PMC10345098; doi:10.1038/s41467-023-39577-0)
Supplement: Supplementary file 9 — Supplementary Data 7 [file 41467_2023_39577_MOESM9_ESM.pdf]

Supplementary Data 7: Comparing different Nuclear receptors

Contents

1 Steroid-Hormone-Receptor FAMILY **5**
1.1 MR Vs MR+1 5
1.2 PGR Vs PGR+2 6
1.3 GR Vs GR+3 7
1.4 MR Vs PGR 8
1.5 GR Vs MR 9
1.6 GR Vs PGR 10
1.7 MR+1 Vs PGR+2 11
1.8 GR+3 Vs MR+1 12
1.9 GR+3 Vs PGR+2 13

2 ERR FAMILY **14**
2.1 ESRRG Vs ESRRG:RXRA 14
2.2 ESRRG:RXRA Vs ESRRG+4 15
2.3 ESRRG Vs ESRRG+4 16
2.4 ESRRB Vs ESRRB:RXRA 17
2.5 ESRRB:RXRA Vs ESRRB+4 18
2.6 ESRRB Vs ESRRB+4 19
2.7 ESRRA Vs ESRRA:RXRA 20
2.8 ESRRA:RXRA Vs ESRRA+4 21
2.9 ESRRA Vs ESRRA+4 22
2.10 ESRRB Vs ESRRG 23
2.11 ESRRA Vs ESRRG 24
2.12 ESRRA Vs ESRRB 25
2.13 ESRRB:RXRA Vs ESRRG:RXRA 26
2.14 ESRRA:RXRA Vs ESRRG:RXRA 27
2.15 ESRRA:RXRA Vs ESRRB:RXRA 28
2.16 ESRRB+4 Vs ESRRG+4 29
2.17 ESRRA+4 Vs ESRRG+4 30
2.18 ESRRA+4 Vs ESRRB+4 31

3 ER FAMILY **32**
3.1 ESR1+5 Vs ESR1+6 32

4 THR FAMILY **33**
4.1 THRB Vs THRB:RXRA 33
4.2 THRB Vs THRB:RXRA+7 34
4.3 THRB Vs THRB+7 35
4.4 THRB:RXRA Vs THRB:RXRA+7 36

4.5 THRA Vs THRA:RXRA 37
4.6 THRA Vs THRA:RXRA+7 38
4.7 THRA Vs THRA+7 39
4.8 THRA:RXRA Vs THRA:RXRA+7 40

5 RAR FAMILY 41
5.1 RARB Vs RARG 41
5.2 RARA Vs RARG 42
5.3 RARA Vs RARB 43
5.4 RARB+8 Vs RARG+8 44
5.5 RARA+8 Vs RARG+8 45
5.6 RARA+8 Vs RARB+8 46
5.7 RARB:RXRA Vs RARG:RXRA 47
5.8 RARA:RXRA Vs RARG:RXRA 48
5.9 RARA:RXRA Vs RARB:RXRA 49
5.10 RARG Vs RARG+8 50
5.11 RARG Vs RARG:RXRA 51
5.12 RARB Vs RARB+8 52
5.13 RARB Vs RARB:RXRA 53
5.14 RARA Vs RARA+8 54
5.15 RARA Vs RARA:RXRA 55

6 VDR FAMILY 56
6.1 PXR Vs PXR+9 56
6.2 VDR Vs VDR:RXRA 57
6.3 VDR Vs VDR+10 58
6.4 PXR Vs VDR 59
6.5 PXR+9 Vs VDR+10 60

7 LXR FAMILY 61
7.1 FXR Vs FXR+11 61
7.2 LXRA Vs LXRA+12 62
7.3 LXRA Vs LXRB:RXRA 63

8 PPAR FAMILY 64
8.1 PPARD Vs PPARD+13 64
8.2 PPARD Vs PPARD+14 65
8.3 PPARD+13 Vs PPARD+14 66
8.4 PPARG Vs PPARG+14 67
8.5 PPARG Vs PPARG+15 68
8.6 PPARG+14 Vs PPARG+15 69

8.7 PPARD Vs PPARG 70
8.8 PPARD+14 Vs PPARG+14 71

9 RORC FAMILY 72
9.1 Rev-ErbA-Alpha Vs RORC 72
9.2 RORC Vs RORC:RXRA 73
9.3 RORC Vs RORC+16 74

10 TR2/TR4 FAMILY 75
10.1 TR2 Vs TR4 75

11 SF1/LRH1 FAMILY 76
11.1 LRH1 Vs LRH1:RXRA 76
11.2 SF1 Vs SF1:RXRA 77
11.3 LRH1 Vs SF1 78
11.4 LRH1:RXRA Vs SF1:RXRA 79

12 TLX/PNR FAMILY 80
12.1 TLX Vs TLX:RXRA 80
12.2 TLX Vs PNR 81

13 COUP/EAR FAMILY 82
13.1 COUP-TF1 Vs COUP-TF2 82
13.2 COUP-TF2 Vs EAR2 83
13.3 COUP-TF1 Vs EAR2 84
13.4 COUP-TF2 Vs COUP-TF2+17 85
13.5 COUP-TF2 Vs COUP-TF2:RXRA 86
13.6 COUP-TF1 Vs COUP-TF1+17 87
13.7 COUP-TF1 Vs COUP-TF1:RXRA 88
13.8 EAR2 Vs EAR2:RXRA 89
13.9 COUP-TF1+17 Vs COUP-TF2+17 90
13.10 COUP-TF1:RXRA Vs COUP-TF2:RXRA 91
13.11 COUP-TF1:RXRA Vs EAR2:RXRA 92
13.12 COUP-TF2:RXRA Vs EAR2:RXRA 93

14 HNF4 FAMILY 94
14.1 HNF4A Vs HNF4G 94
14.2 HNF4A Vs HNF4A+18 95

15 RXR FAMILY **96**
15.1 RXRB Vs RXRB+17 96
15.2 RXRG Vs RXRG:RXRA 97
15.3 RXRG Vs RXRG+17 98
15.4 RXRG:RXRA Vs RXRG+17 99
15.5 RXRA Vs RXRA+17 100
15.6 RXRB Vs RXRG 101
15.7 RXRA Vs RXRB 102
15.8 RXRA Vs RXRG 103
15.9 RXRB+17 Vs RXRG+17 104
15.10RXRA+17 Vs RXRG+17 105
15.11RXRA+17 Vs RXRB+17 106

16 NURR1/NOR1 FAMILY **107**
16.1 NOR1 Vs NURR1 107

1 Steroid-Hormone-Receptor FAMILY

1.1 MR Vs MR+1

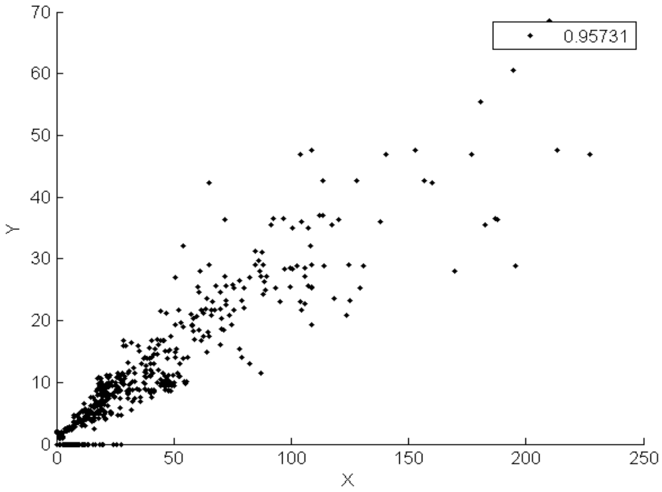

DiSEL	DR	IR	ER
MR (SSL: GNACR = X)	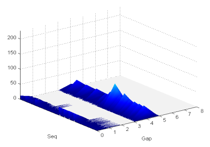	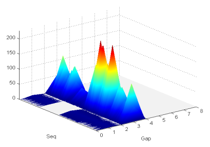	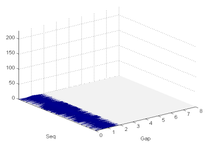
MR+1 (SSL: GNACR = Y)	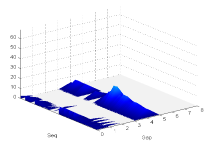	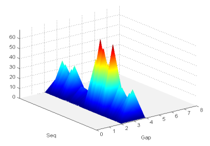	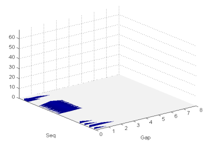
DiSEL: GNACR = X over Y	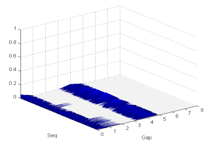	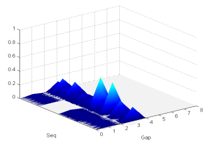	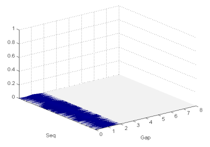
DiSEL: GNACR = Y over X	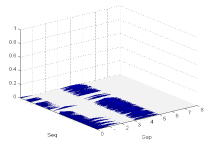	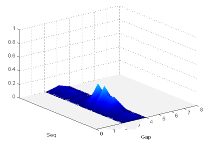	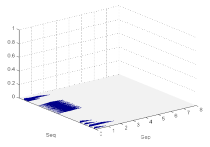

1.2 PGR Vs PGR+2

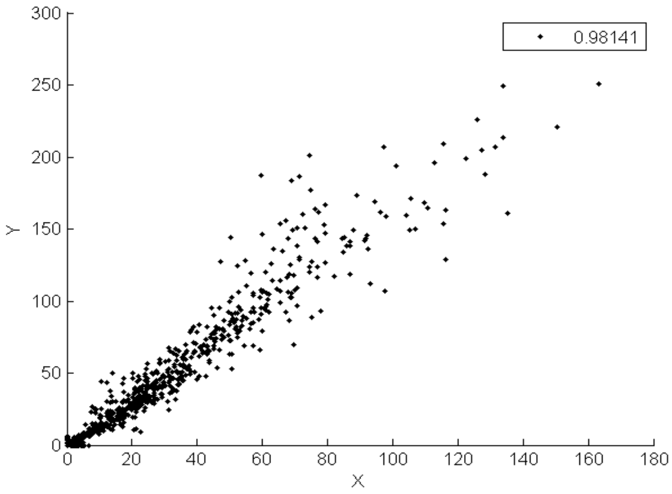

DiSEL	DR	IR	ER
PGR (SSL: GNACR = X)	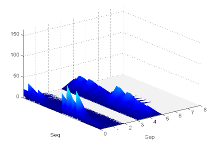	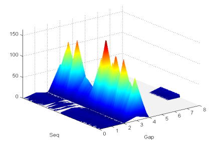	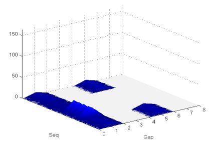
PGR+2 (SSL: GNACR = Y)	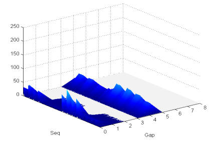	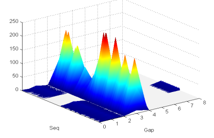	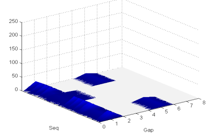
DiSEL: GNACR = X over Y	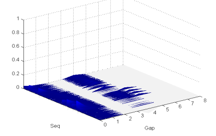	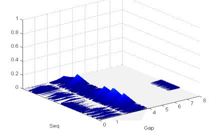	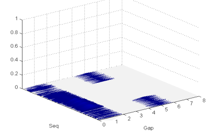
DiSEL: GNACR = Y over X	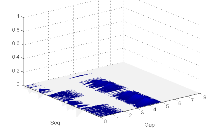	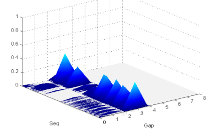	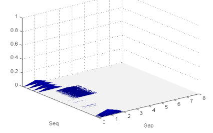

1.3 GR Vs GR+3

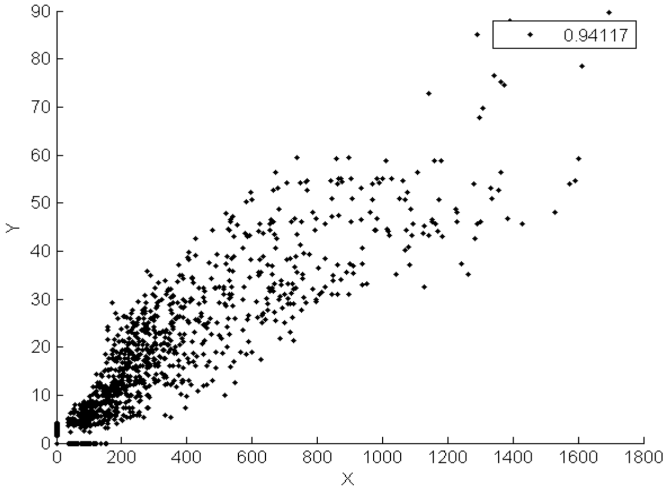

DiSEL	DR	IR	ER
GR (SSL: GNACR = X)	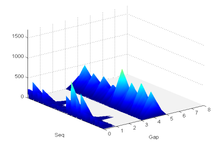	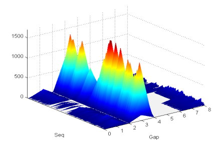	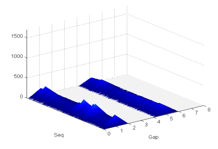
GR+3 (SSL: GNACR = Y)	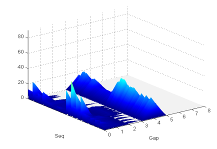	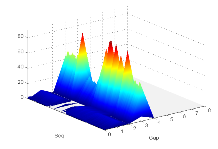	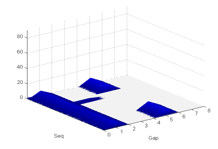
DiSEL: GNACR = X over Y	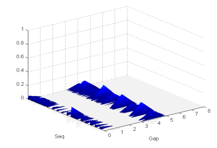	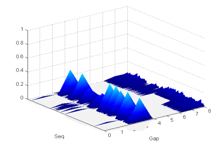	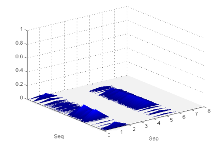
DiSEL: GNACR = Y over X	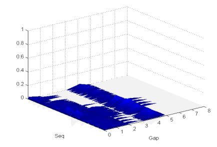	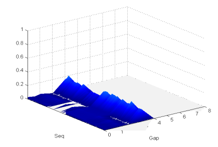	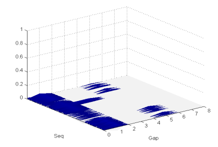

1.4 MR Vs PGR

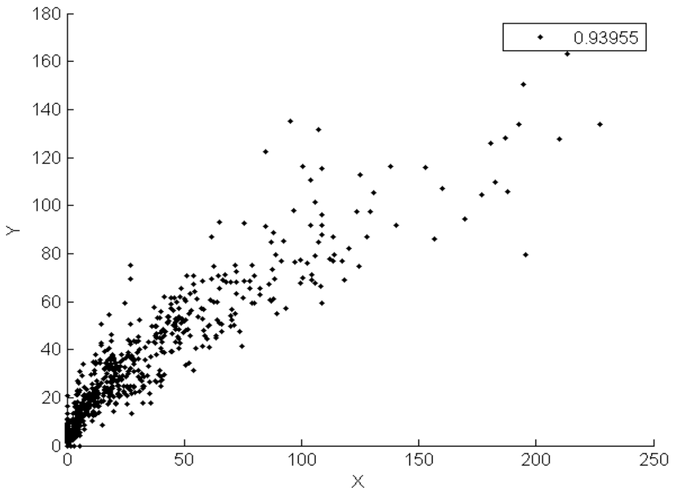

DiSEL	DR	IR	ER
MR (SSL: GNACR = X)	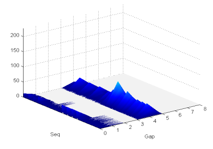	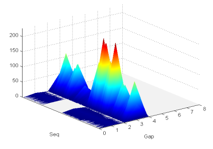	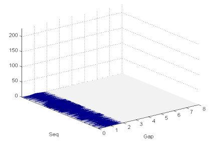
PGR (SSL: GNACR = Y)	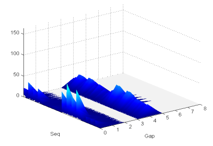	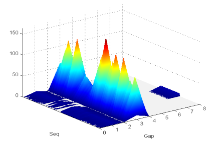	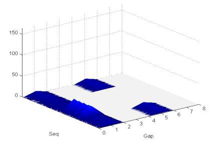
DiSEL: GNACR = X over Y	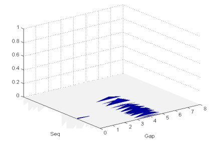	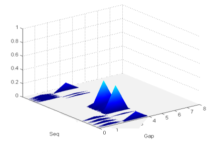	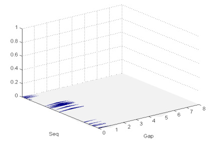
DiSEL: GNACR = Y over X	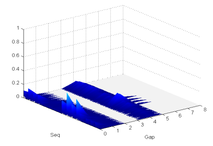	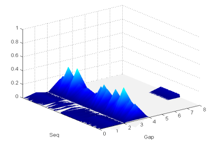	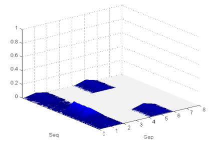

1.5 GR Vs MR

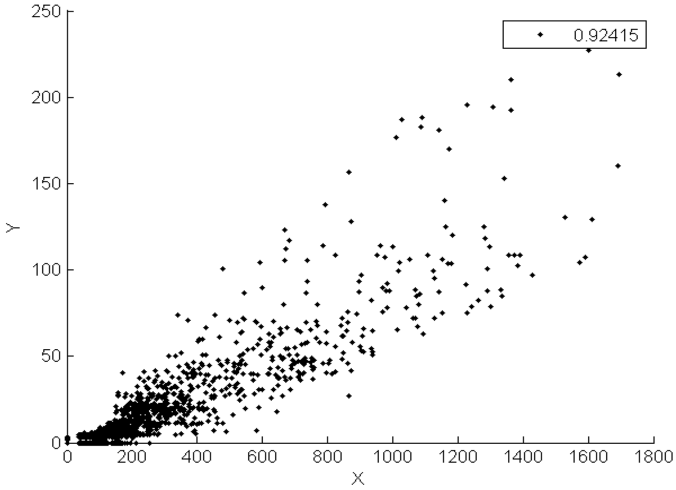

DiSEL	DR	IR	ER
GR (SSL: GNACR = X)	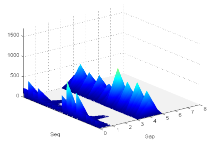	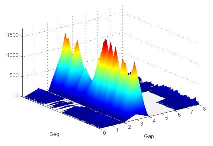	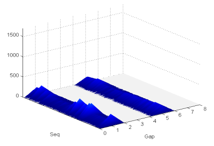
MR (SSL: GNACR = Y)	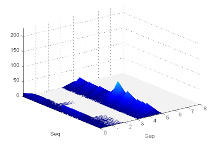	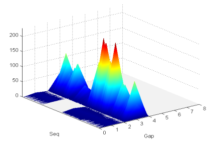	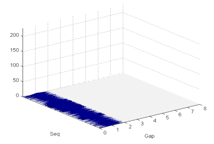
DiSEL: GNACR = X over Y	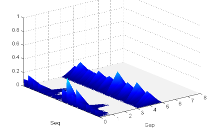	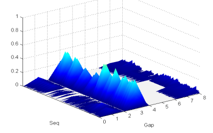	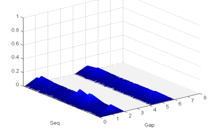
DiSEL: GNACR = Y over X	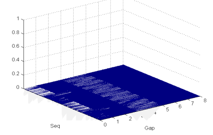	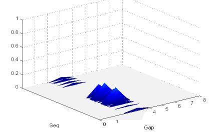	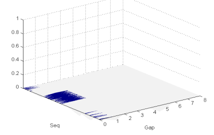

1.6 GR Vs PGR

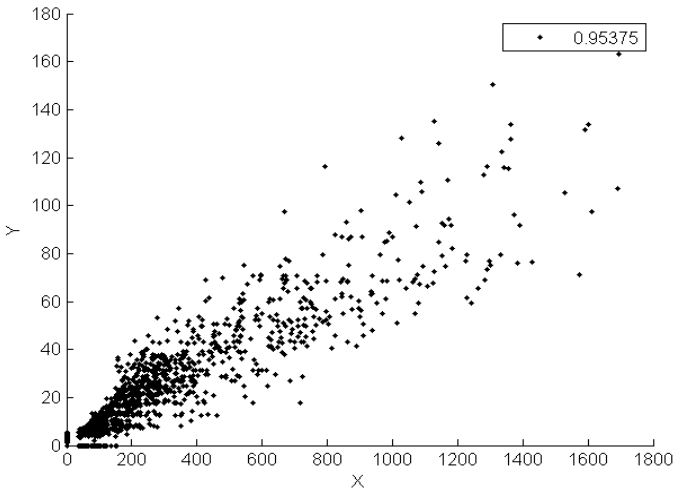

DiSEL	DR	IR	ER
GR (SSL: GNACR = X)	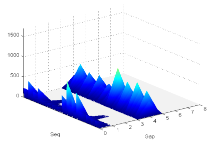	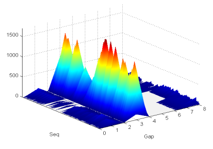	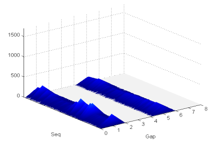
PGR (SSL: GNACR = Y)	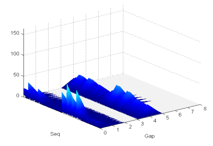	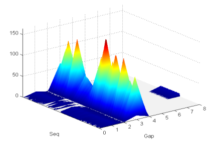	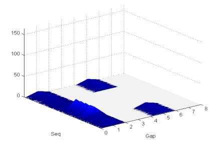
DiSEL: GNACR = X over Y	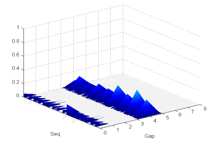	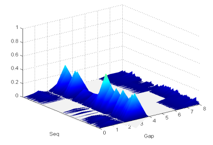	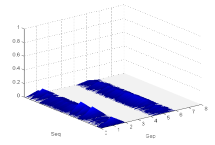
DiSEL: GNACR = Y over X	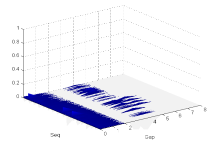	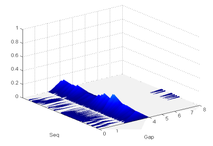	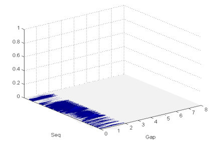

1.7 MR+1 Vs PGR+2

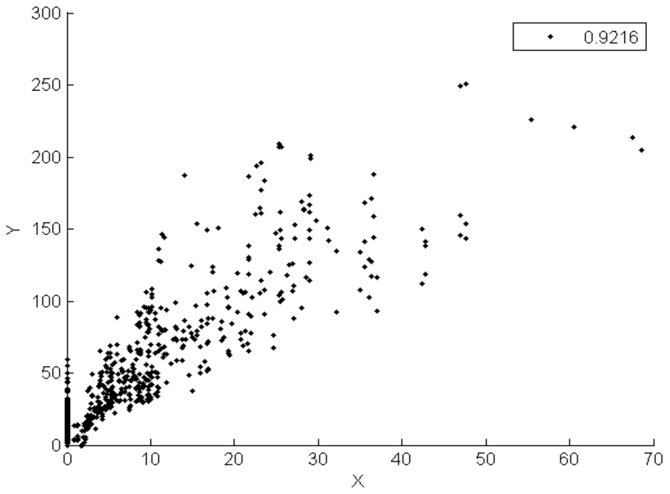

DiSEL	DR	IR	ER
MR+1 (SSL: GNACR = X)	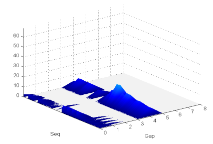	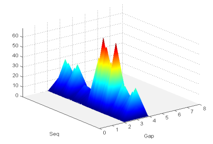	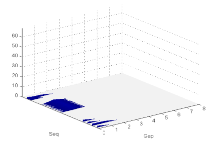
PGR+2 (SSL: GNACR = Y)	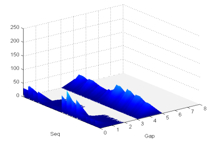	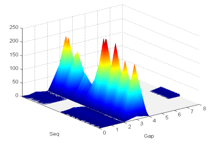	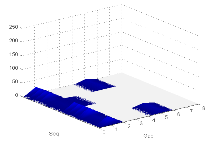
DiSEL: GNACR = X over Y	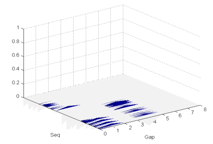	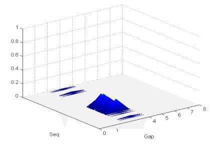	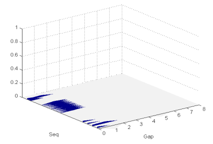
DiSEL: GNACR = Y over X	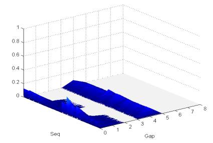	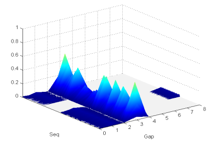	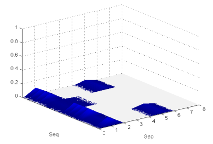

1.8 GR+3 Vs MR+1

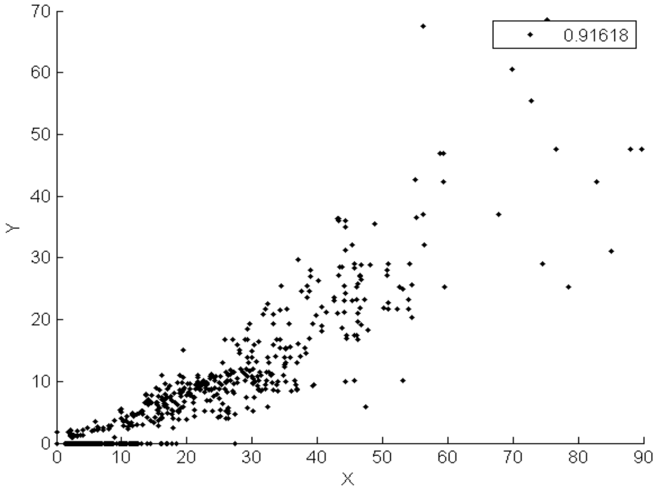

DiSEL	DR	IR	ER
GR+3 (SSL: GNACR = X)	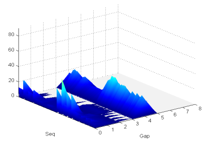	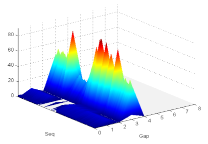	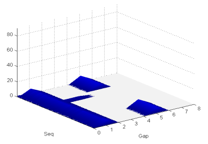
MR+1 (SSL: GNACR = Y)	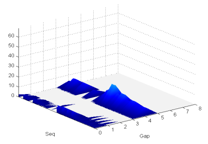	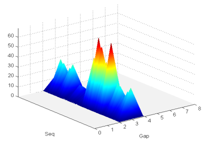	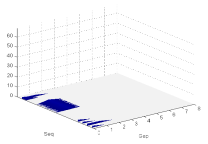
DiSEL: GNACR = X over Y	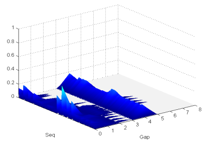	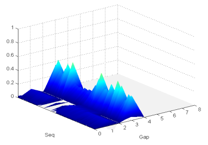	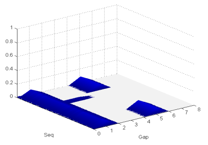
DiSEL: GNACR = Y over X	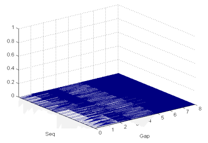	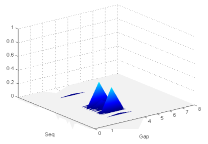	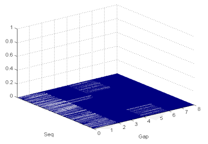

1.9 GR+3 Vs PGR+2

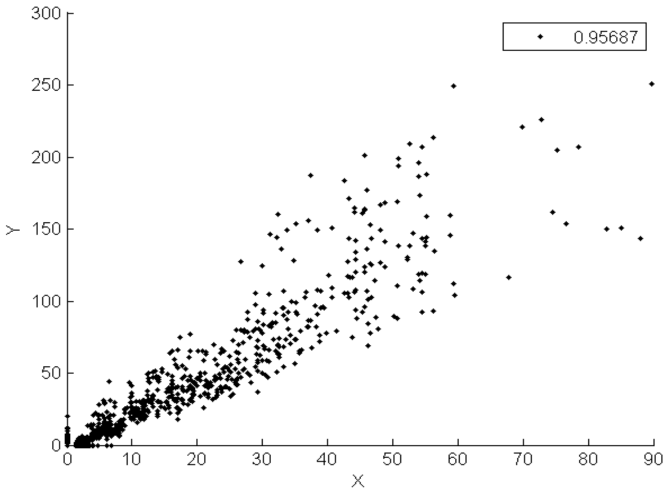

DiSEL	DR	IR	ER
GR+3 (SSL: GNACR = X)	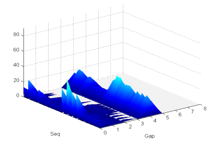	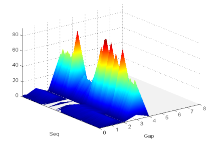	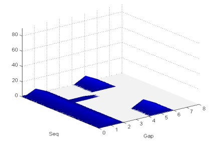
PGR+2 (SSL: GNACR = Y)	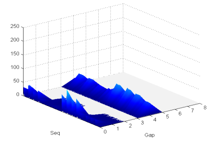	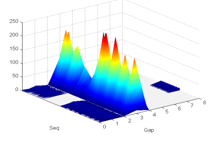	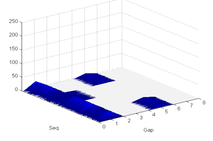
DiSEL: GNACR = X over Y	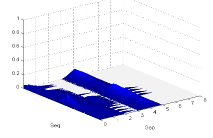	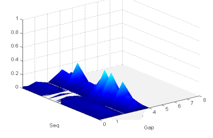	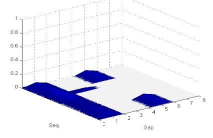
DiSEL: GNACR = Y over X	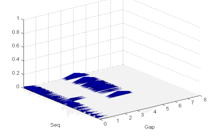	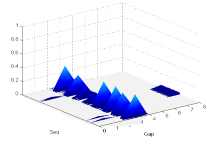	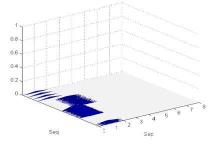

2 ERR FAMILY

2.1 ESRRG Vs ESRRG:RXRA

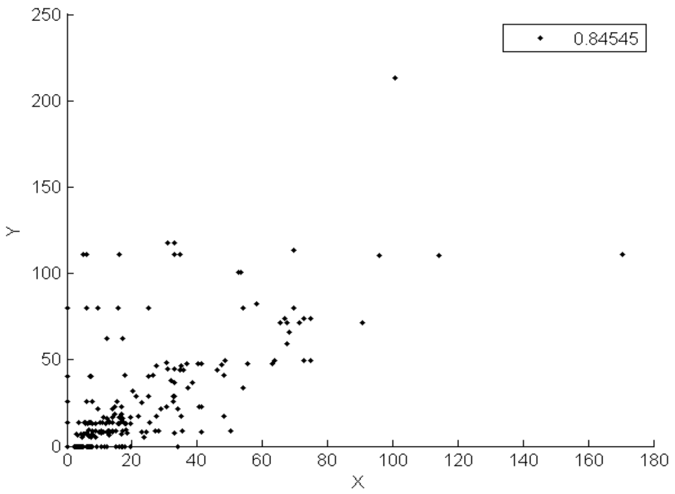

DiSEL	DR	IR	ER
ESRRG (SSL: RGGTCR = X)	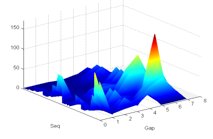	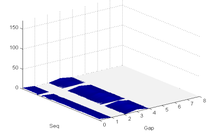	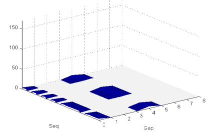
ESRRG:RXRA (SSL: RGGTCR = Y)	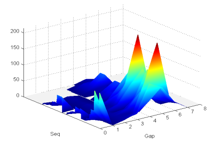	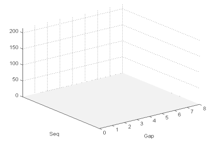	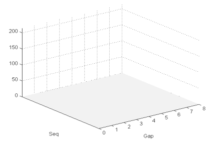
DiSEL: RGGTCR = X over Y	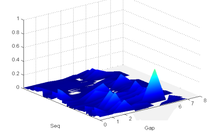	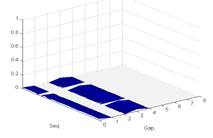	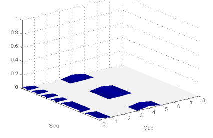
DiSEL: RGGTCR = Y over X	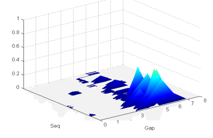	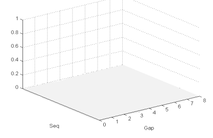	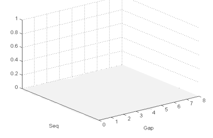

2.2 ESRRG:RXRA Vs ESRRG+4

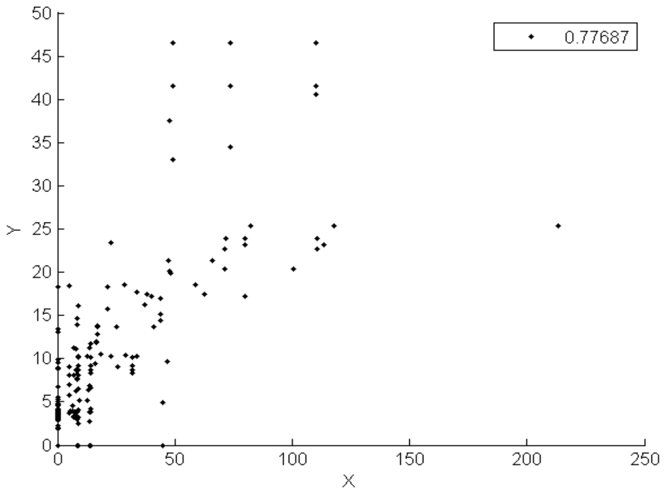

DiSEL	DR	IR	ER
ESRRG:RXRA (SSL: RGGTCR = X)	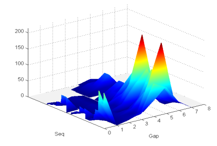	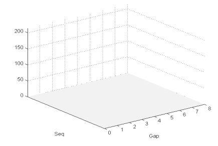	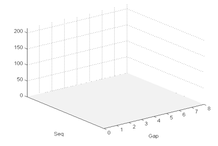
ESRRG+4 (SSL: RGGTCR = Y)	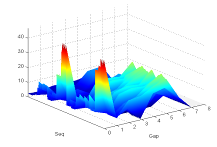	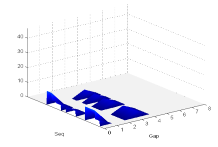	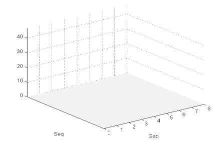
DiSEL: RGGTCR = X over Y	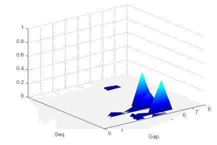	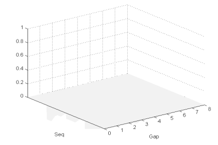	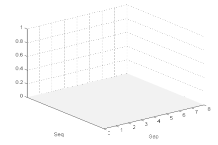
DiSEL: RGGTCR = Y over X	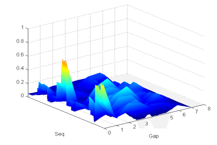	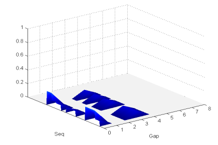	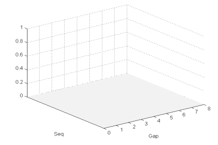

2.3 ESRRG Vs ESRRG+4

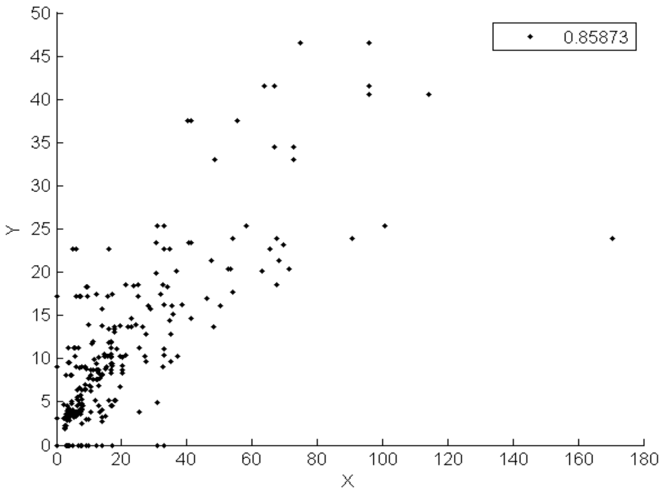

DiSEL	DR	IR	ER
ESRRG (SSL: RGGTCR = X)	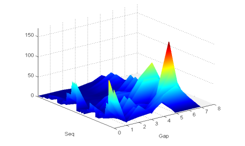	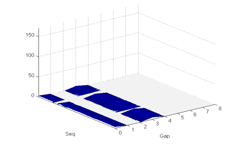	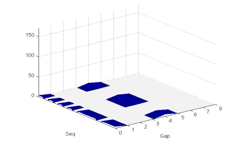
ESRRG+4 (SSL: RGGTCR = Y)	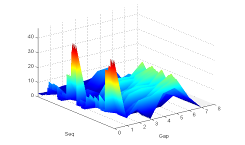	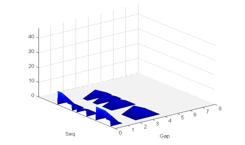	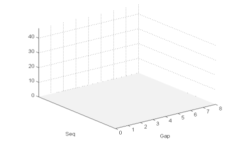
DiSEL: RGGTCR = X over Y	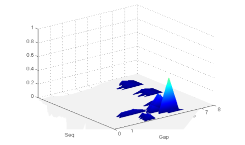	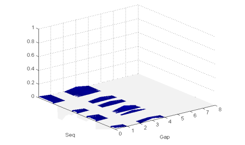	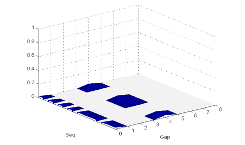
DiSEL: RGGTCR = Y over X	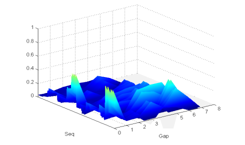	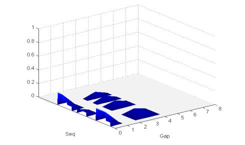	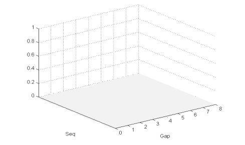

2.4 ESRRB Vs ESRRB:RXRA

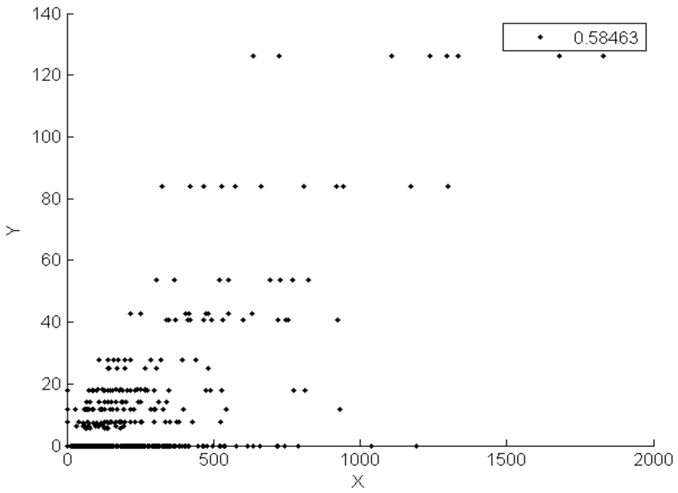

DiSEL	DR	IR	ER
ESRRB (SSL: RGGTCR = X)	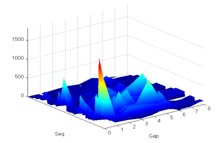	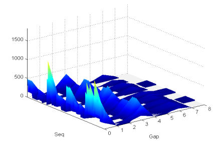	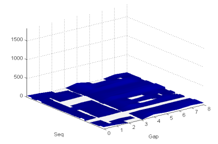
ESRRB:RXRA (SSL: RGGTCR = Y)	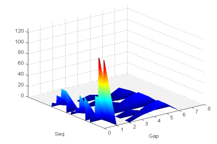	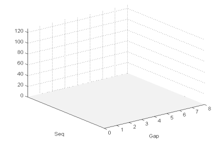	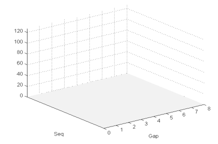
DiSEL: RGGTCR = X over Y	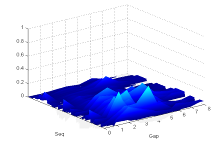	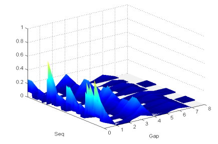	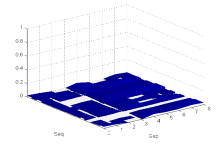
DiSEL: RGGTCR = Y over X	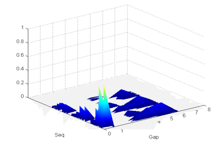	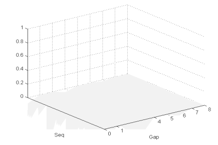	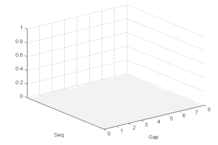

2.5 ESRRB:RXRA Vs ESRRB+4

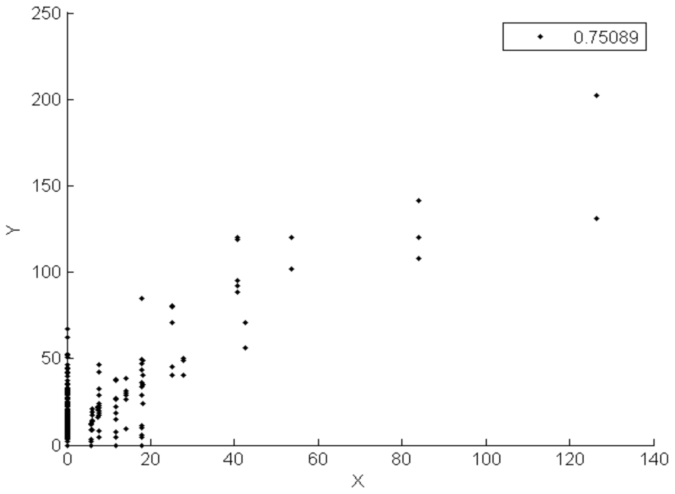

DiSEL	DR	IR	ER
ESRRB:RXRA (SSL: RGGTCR = X)	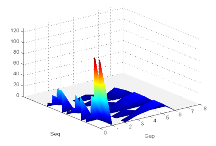	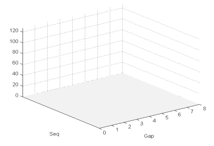	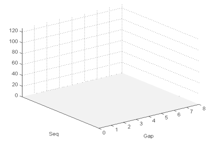
ESRRB+4 (SSL: RGGTCR = Y)	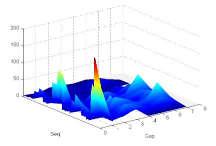	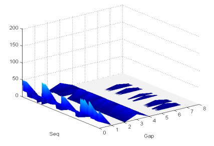	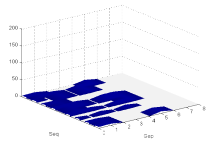
DiSEL: RGGTCR = X over Y	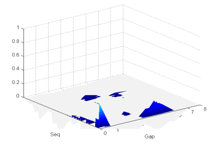	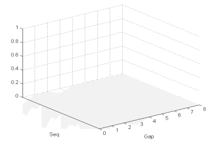	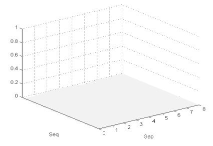
DiSEL: RGGTCR = Y over X	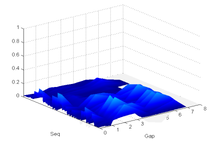	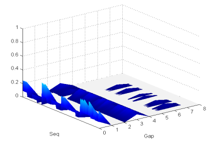	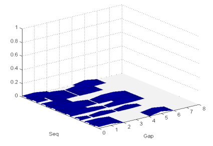

2.6 ESRRB Vs ESRRB+4

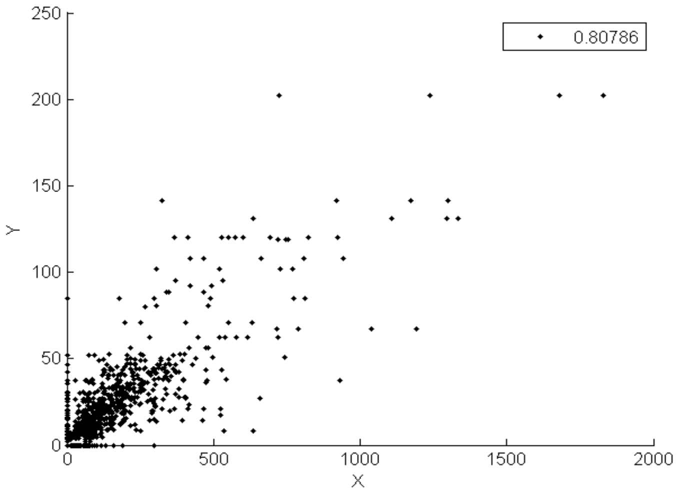

DiSEL	DR	IR	ER
ESRRB (SSL: RGGTCR = X)	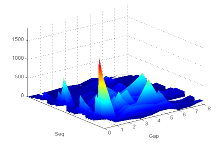	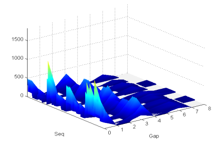	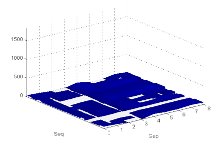
ESRRB+4 (SSL: RGGTCR = Y)	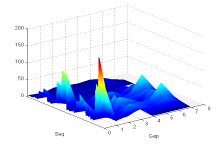	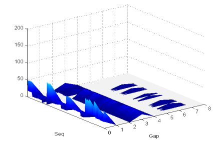	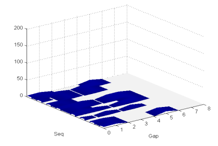
DiSEL: RGGTCR = X over Y	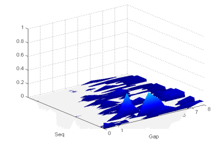	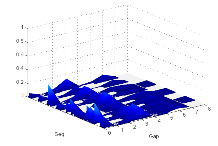	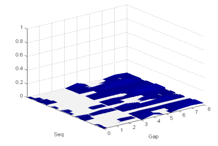
DiSEL: RGGTCR = Y over X	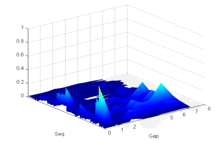	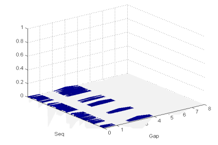	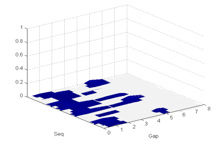

2.7 ESRRA Vs ESRRA:RXRA

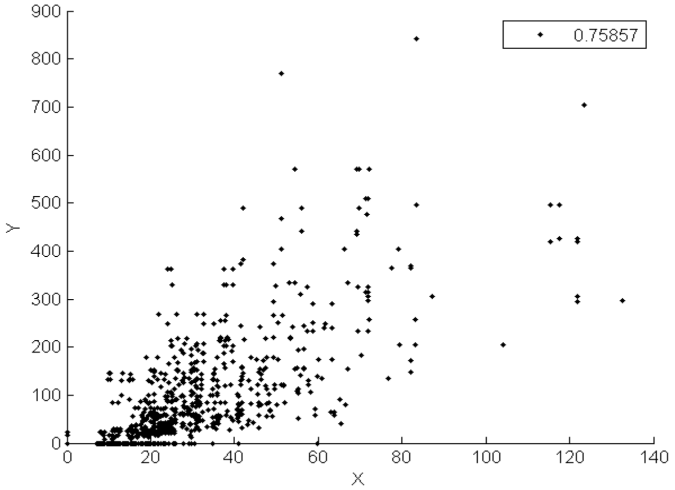

DiSEL	DR	IR	ER
ESRRA (SSL: RGGTCR = X)	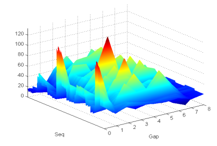	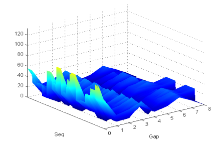	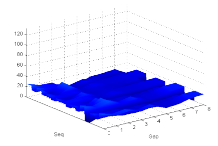
ESRRA:RXRA (SSL: RGGTCR = Y)	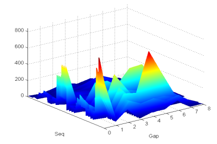	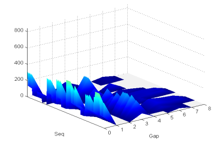	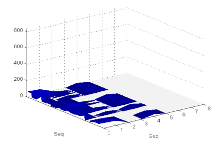
DiSEL: RGGTCR = X over Y	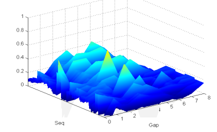	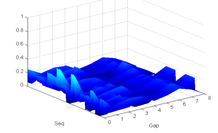	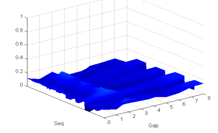
DiSEL: RGGTCR = Y over X	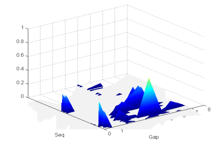	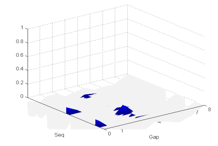	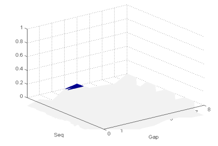

2.8 ESRRA:RXRA Vs ESRRA+4

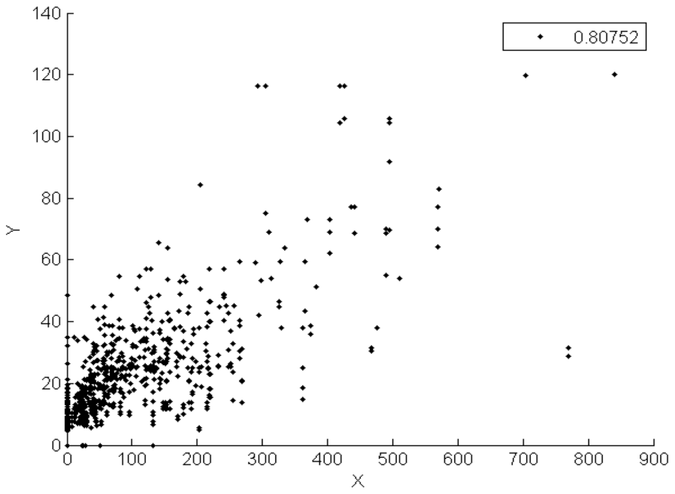

DiSEL	DR	IR	ER
ESRRA:RXRA (SSL: RGGTCR = X)	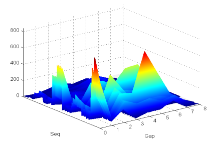	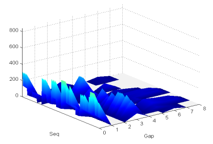	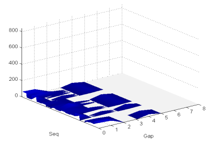
ESRRA+4 (SSL: RGGTCR = Y)	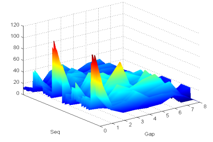	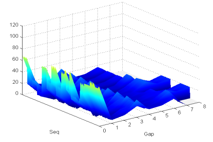	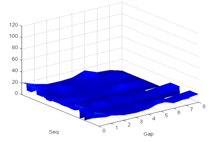
DiSEL: RGGTCR = X over Y	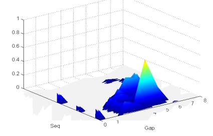	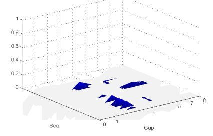	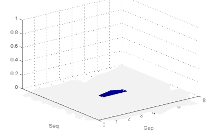
DiSEL: RGGTCR = Y over X	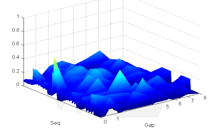	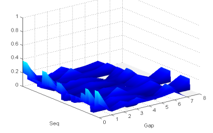	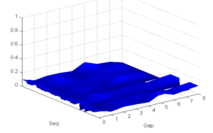

2.9 ESRRA Vs ESRRA+4

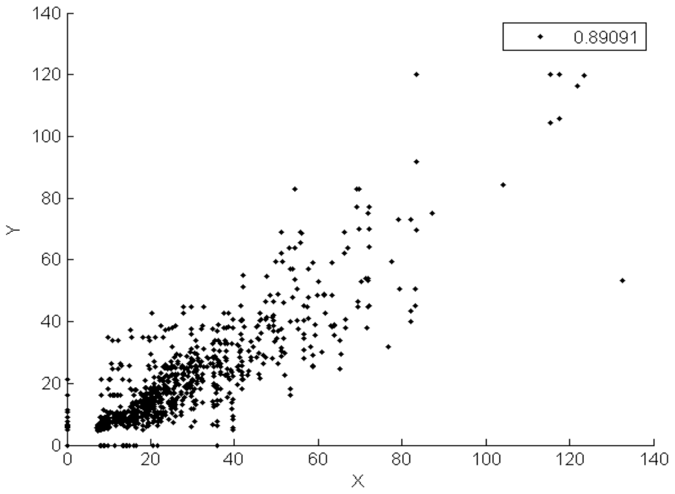

DiSEL	DR	IR	ER
ESRRA (SSL: RGGTCR = X)	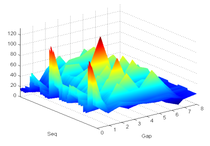	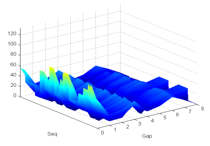	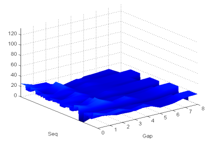
ESRRA+4 (SSL: RGGTCR = Y)	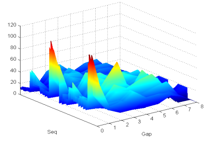	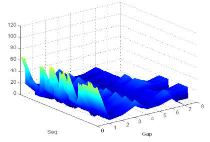	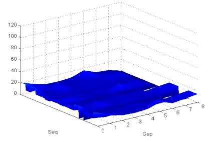
DiSEL: RGGTCR = X over Y	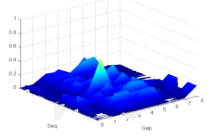	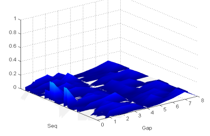	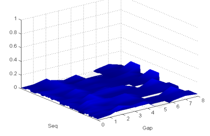
DiSEL: RGGTCR = Y over X	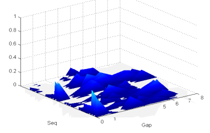	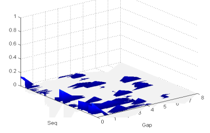	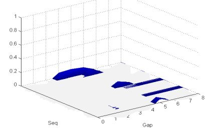

2.10 ESRRB Vs ESRRG

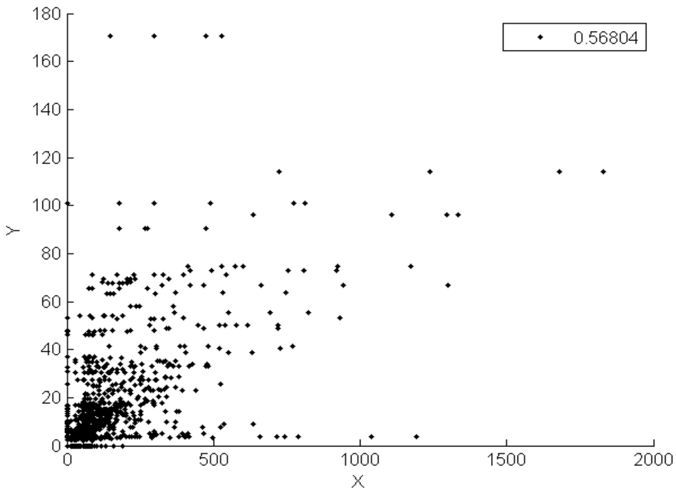

DiSEL	DR	IR	ER
ESRRB (SSL: RGGTCR = X)	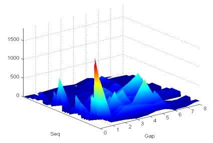	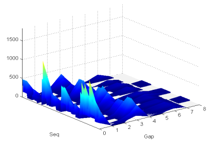	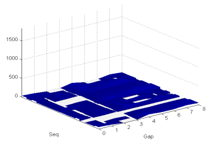
ESRRG (SSL: RGGTCR = Y)	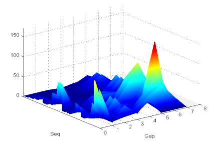	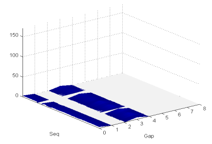	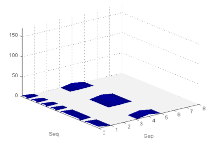
DiSEL: RGGTCR = X over Y	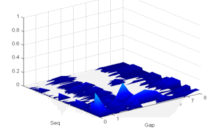	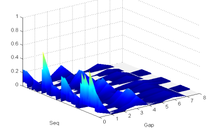	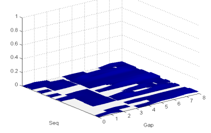
DiSEL: RGGTCR = Y over X	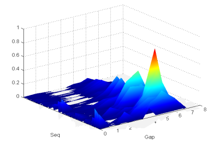	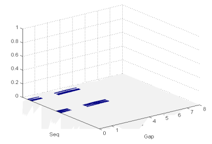	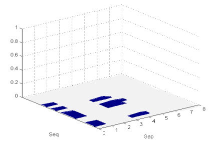

2.11 ESRRA Vs ESRRG

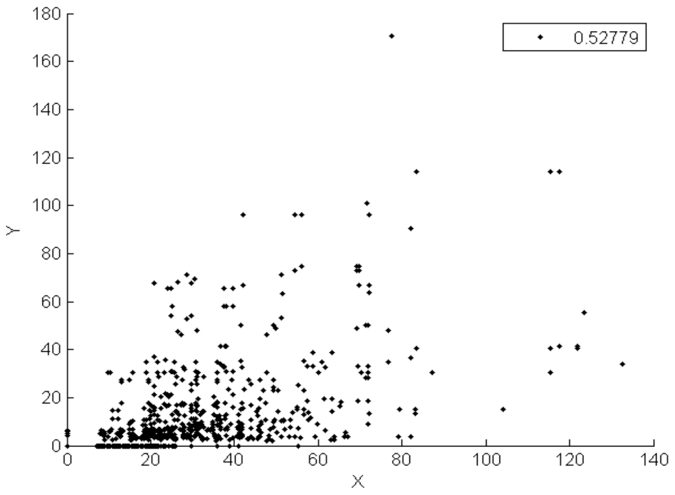

DiSEL	DR	IR	ER
ESRRA (SSL: RGGTCR = X)	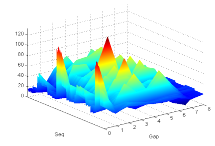	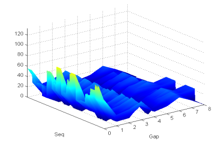	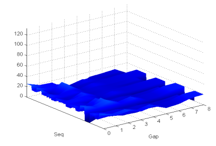
ESRRG (SSL: RGGTCR = Y)	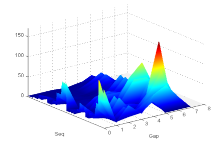	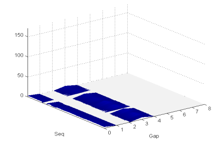	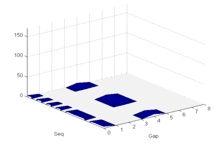
DiSEL: RGGTCR = X over Y	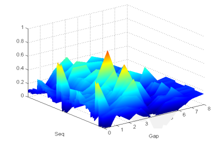	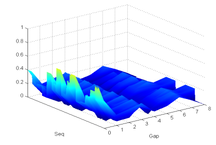	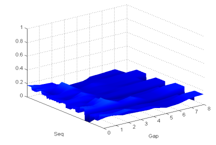
DiSEL: RGGTCR = Y over X	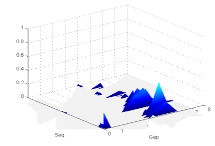	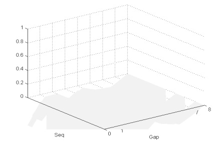	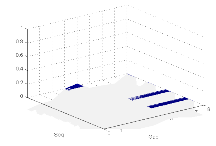

2.12 ESRRA Vs ESRRB

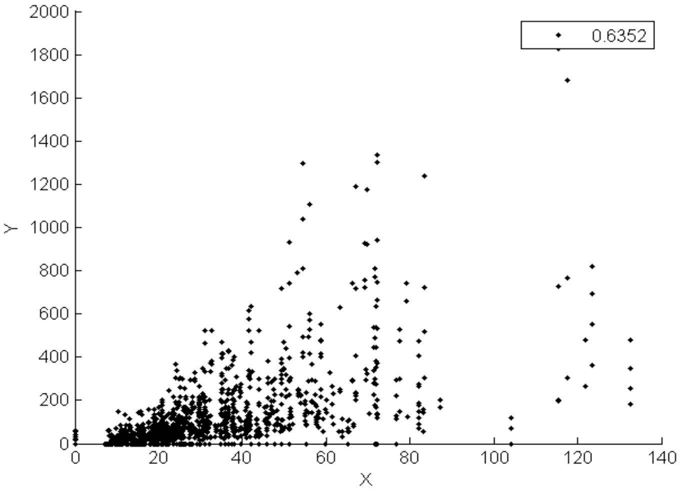

DiSEL	DR	IR	ER
ESRRA (SSL: RGGTCR = X)	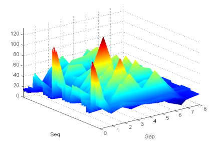	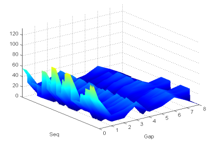	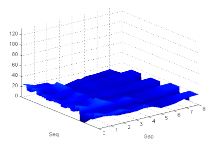
ESRRB (SSL: RGGTCR = Y)	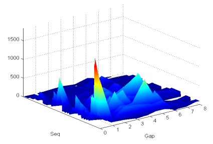	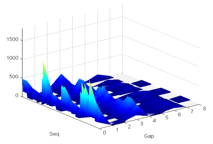	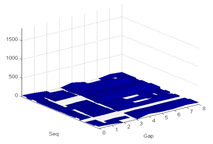
DiSEL: RGGTCR = X over Y	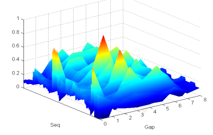	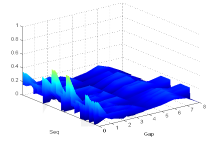	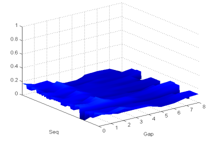
DiSEL: RGGTCR = Y over X	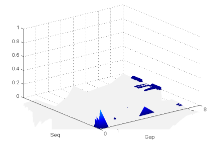	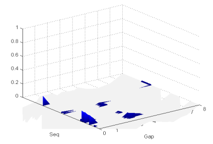	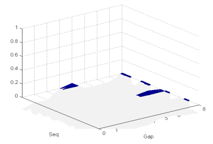

2.13 ESRRB:RXRA Vs ESRRG:RXRA

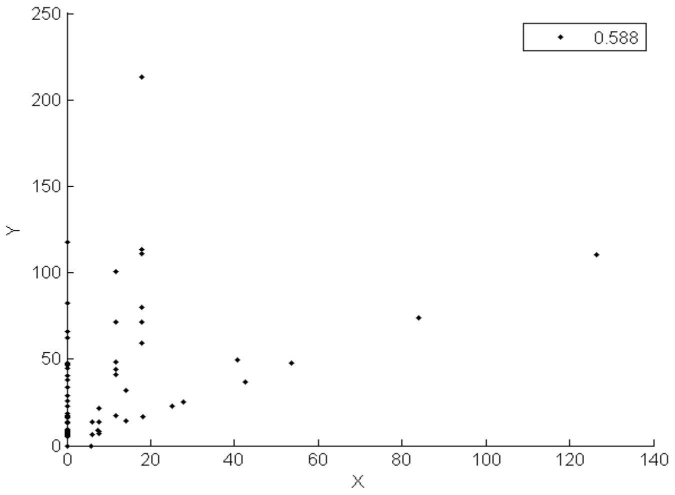

DiSEL	DR	IR	ER
ESRRB:RXRA (SSL: RGGTCR = X)	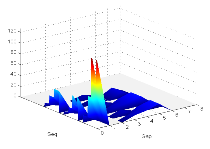	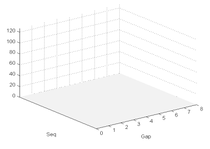	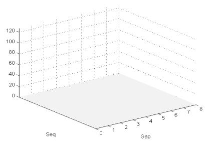
ESRRG:RXRA (SSL: RGGTCR = Y)	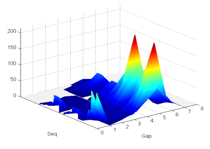	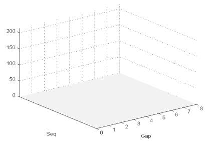	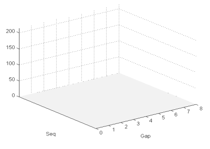
DiSEL: RGGTCR = X over Y	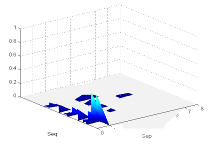	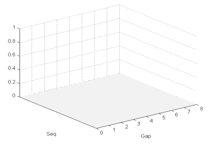	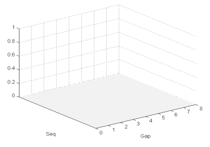
DiSEL: RGGTCR = Y over X	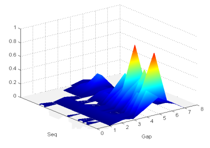	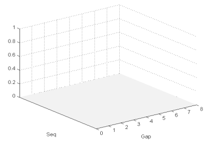	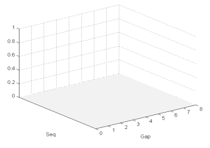

2.14 ESRRA:RXRA Vs ESRRG:RXRA

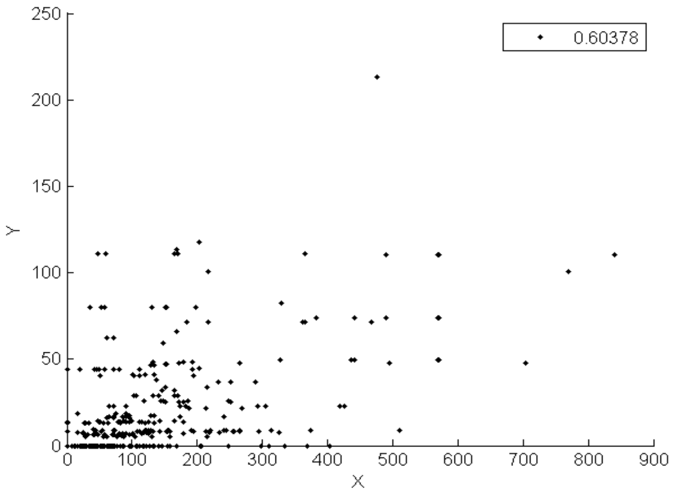

DiSEL	DR	IR	ER
ESRRA:RXRA (SSL: RGGTCR = X)	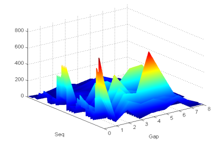	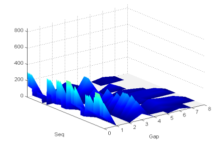	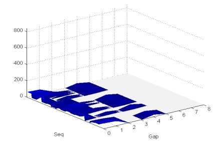
ESRRG:RXRA (SSL: RGGTCR = Y)	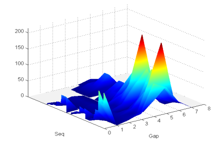	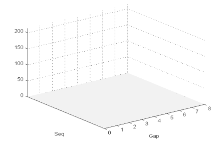	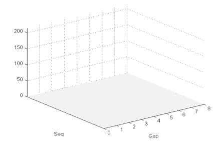
DiSEL: RGGTCR = X over Y	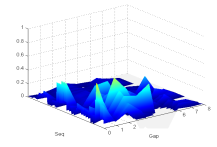	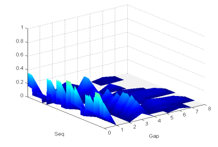	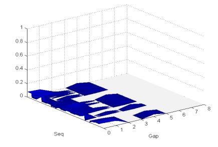
DiSEL: RGGTCR = Y over X	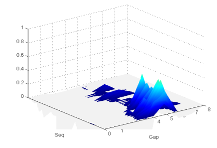	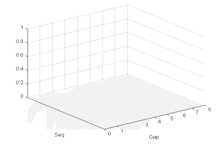	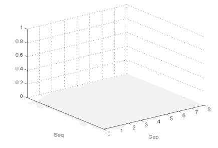

2.15 ESRRA:RXRA Vs ESRRB:RXRA

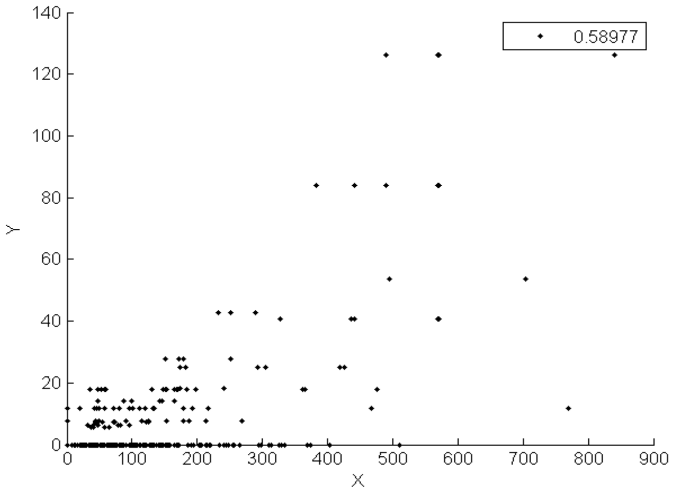

DiSEL	DR	IR	ER
ESRRA:RXRA (SSL: RGGTCR = X)	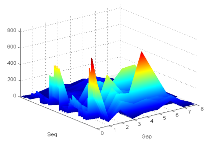	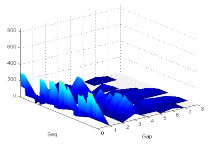	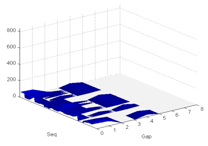
ESRRB:RXRA (SSL: RGGTCR = Y)	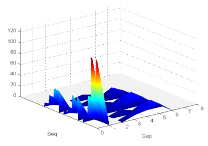	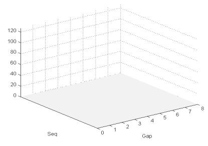	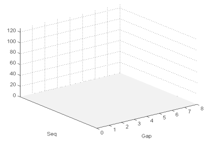
DiSEL: RGGTCR = X over Y	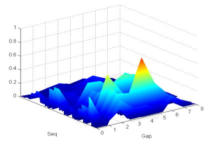	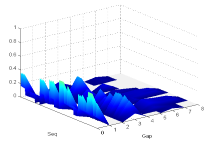	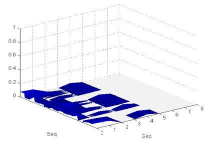
DiSEL: RGGTCR = Y over X	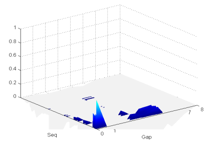	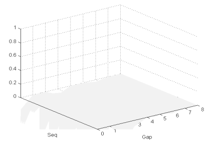	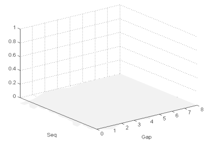

2.16 ESRRB+4 Vs ESRRG+4

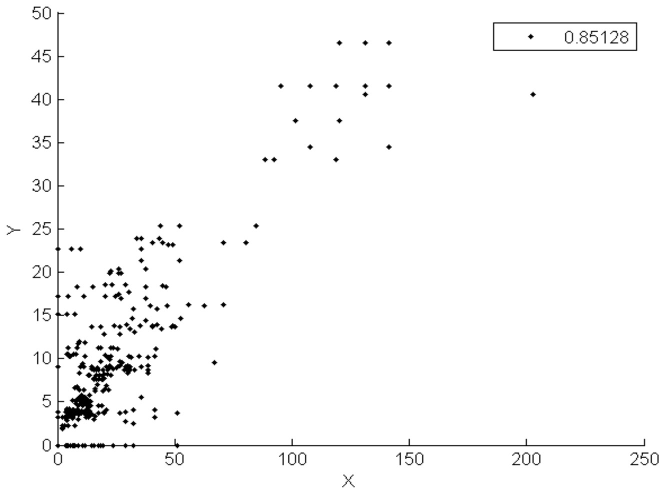

DiSEL	DR	IR	ER
ESRRB+4 (SSL: RGGTCR = X)	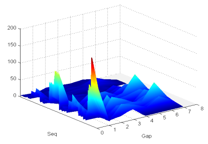	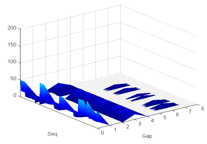	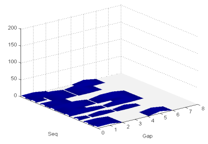
ESRRG+4 (SSL: RGGTCR = Y)	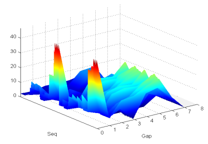	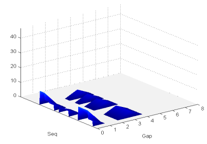	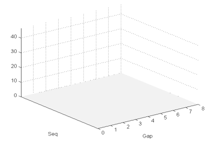
DiSEL: RGGTCR = X over Y	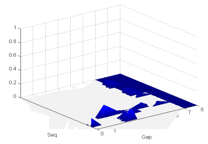	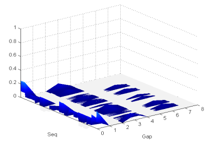	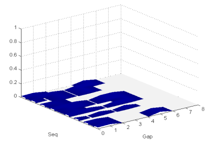
DiSEL: RGGTCR = Y over X	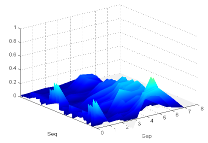	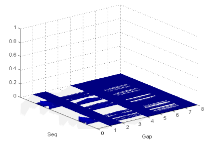	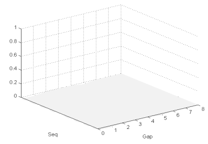

2.17 ESRRA+4 Vs ESRRG+4

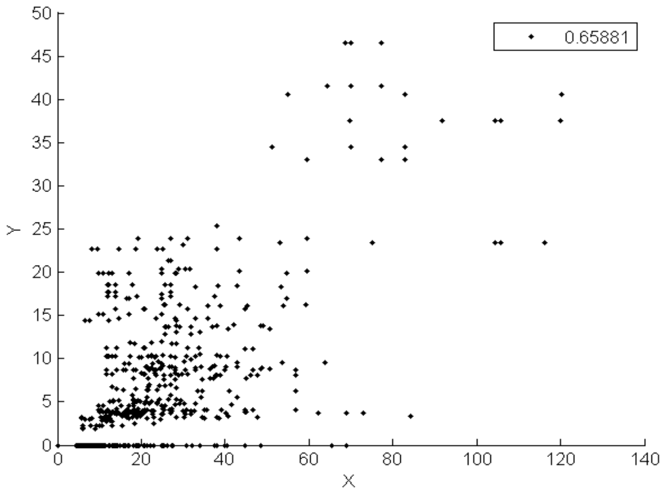

DiSEL	DR	IR	ER
ESRRA+4 (SSL: RGGTCR = X)	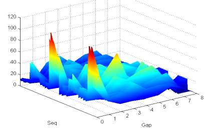	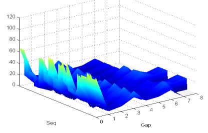	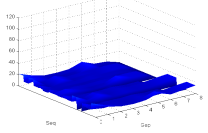
ESRRG+4 (SSL: RGGTCR = Y)	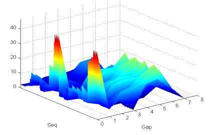	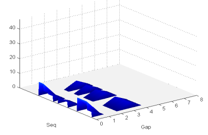	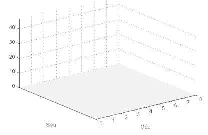
DiSEL: RGGTCR = X over Y	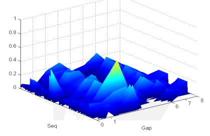	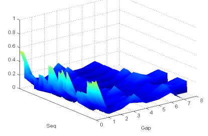	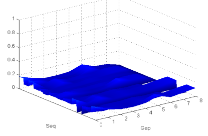
DiSEL: RGGTCR = Y over X	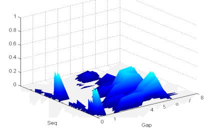	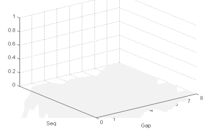	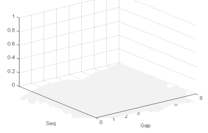

2.18 ESRRA+4 Vs ESRRB+4

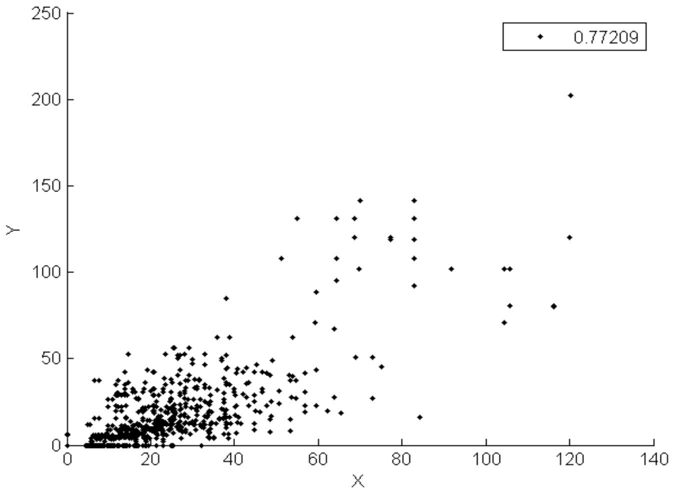

DiSEL	DR	IR	ER
ESRRA+4 (SSL: RGGTCR = X)	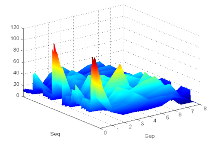	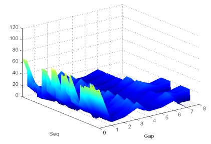	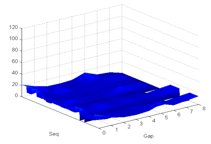
ESRRB+4 (SSL: RGGTCR = Y)	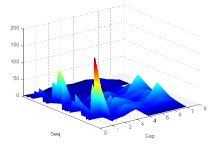	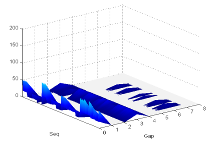	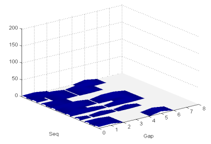
DiSEL: RGGTCR = X over Y	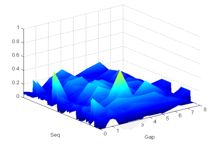	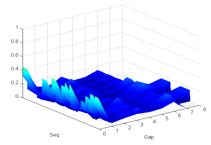	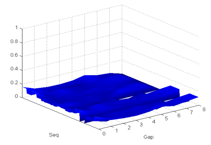
DiSEL: RGGTCR = Y over X	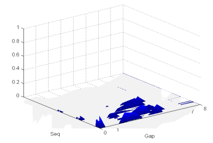	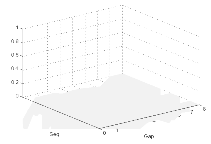	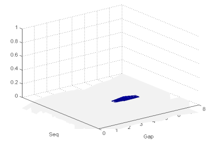

3 ER FAMILY

3.1 ESR1+5 Vs ESR1+6

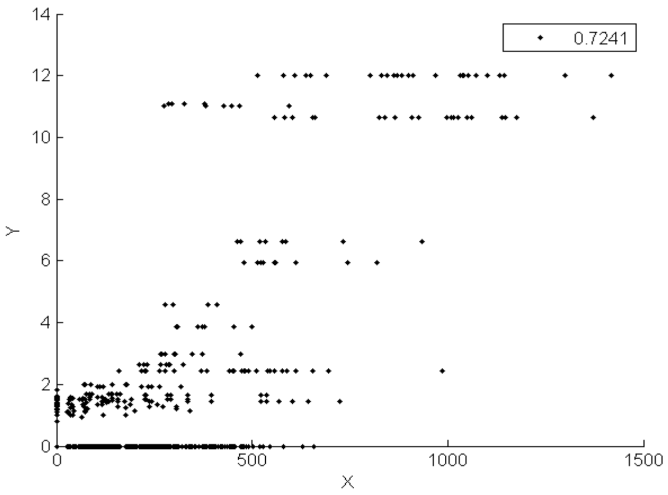

DiSEL	DR	IR	ER
ESR1+5 (SSL: RGK-TCR = X)	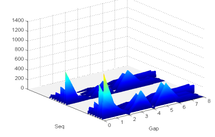	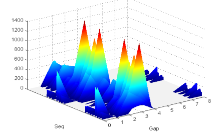	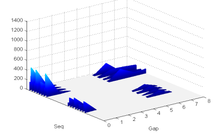
ESR1+6 (SSL: RGK-TCR = Y)	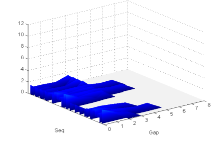	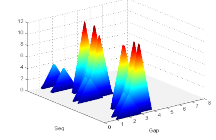	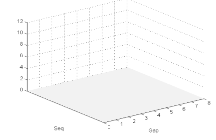
DiSEL: RGKTCR = X over Y	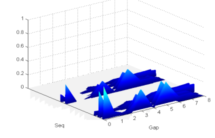	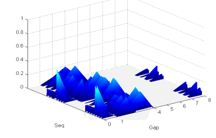	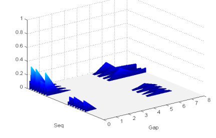
DiSEL: RGKTCR = Y over X	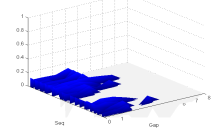	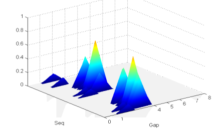	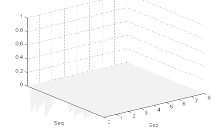

4 THR FAMILY

4.1 THRB Vs THRB:RXRA

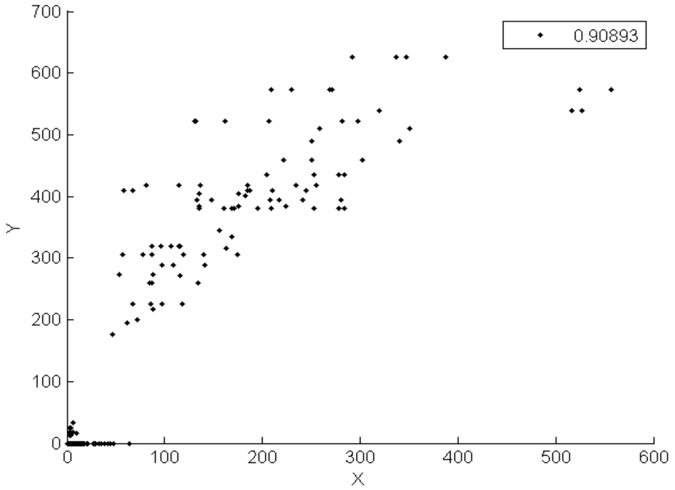

DiSEL	DR	IR	ER
THRB (SSL: RGGTCR = X)	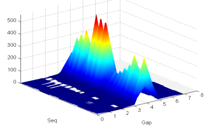	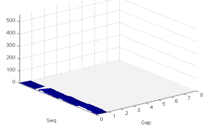	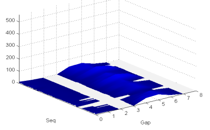
THRB:RXRA (SSL: RGGTCR = Y)	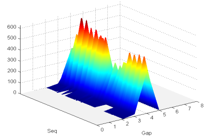	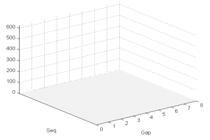	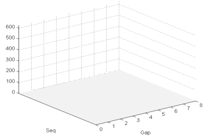
DiSEL: RGGTCR = X over Y	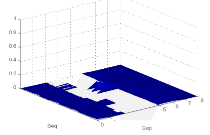	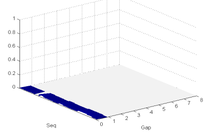	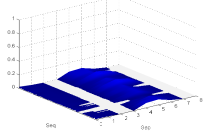
DiSEL: RGGTCR = Y over X	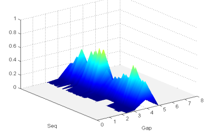	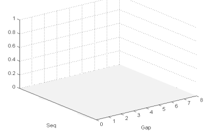	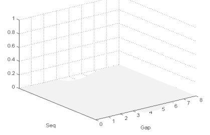

4.2 THRB Vs THRB:RXRA+7

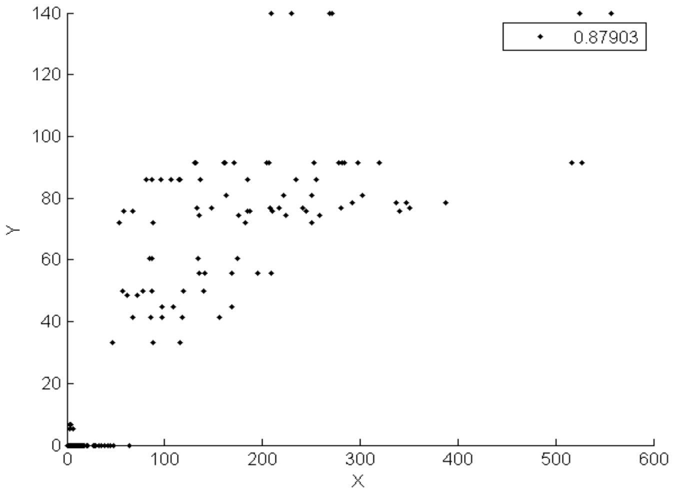

DiSEL	DR	IR	ER
THRB (SSL: RGGTCR = X)	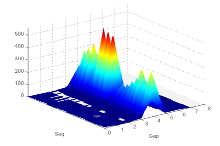	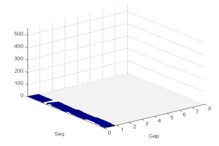	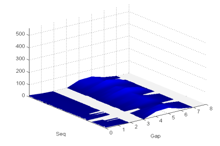
THRB:RXRA+7 (SSL: RGGTCR = Y)	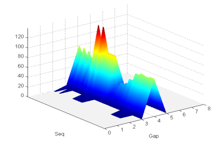	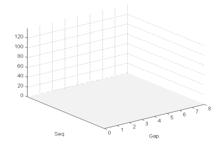	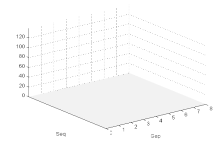
DiSEL: RGGTCR = X over Y	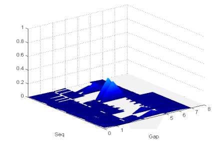	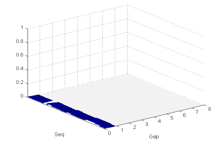	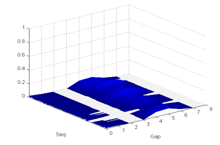
DiSEL: RGGTCR = Y over X	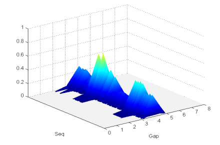	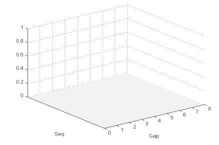	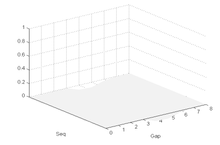

4.3 THRB Vs THRB+7

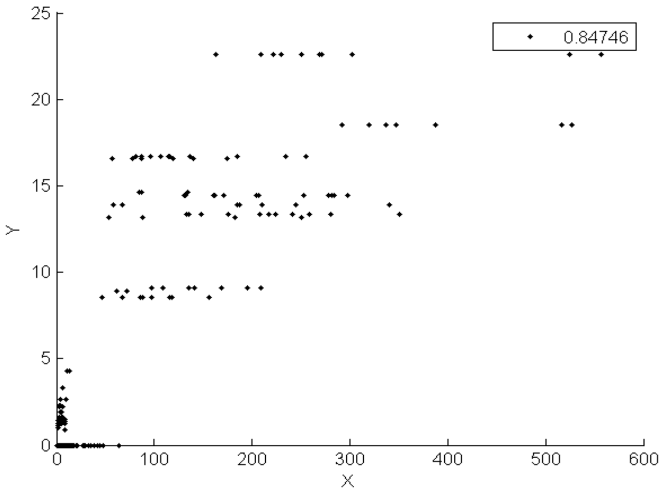

DiSEL	DR	IR	ER
THRB (SSL: RGGTCR = X)	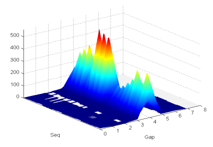	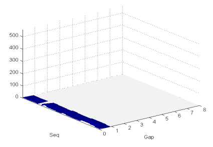	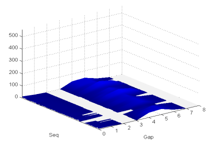
THRB+7 (SSL: RGGTCR = Y)	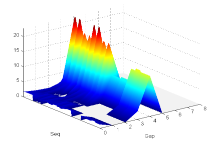	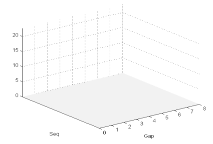	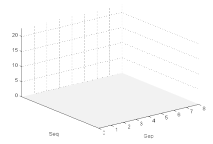
DiSEL: RGGTCR = X over Y	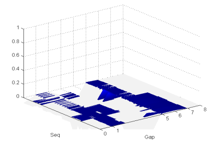	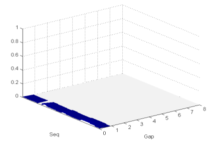	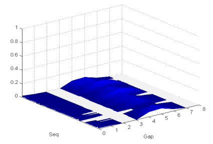
DiSEL: RGGTCR = Y over X	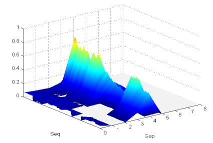	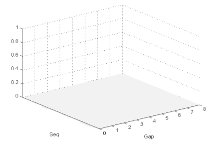	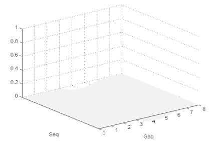

4.4 THRB:RXRA Vs THRB:RXRA+7

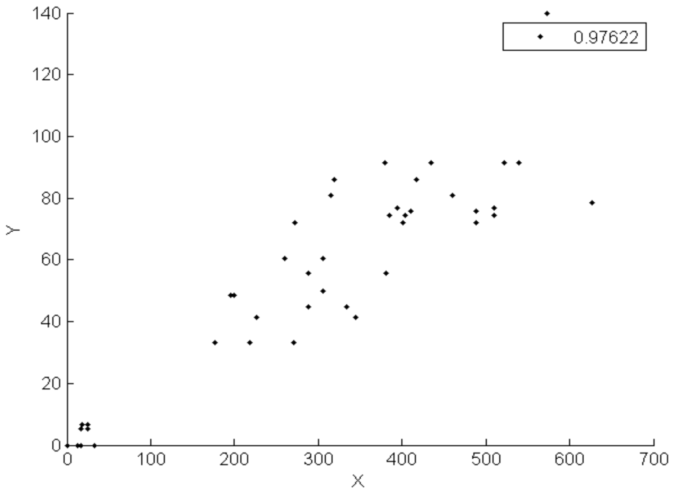

DiSEL	DR	IR	ER
THRB:RXRA (SSL: RGGTCR = X)	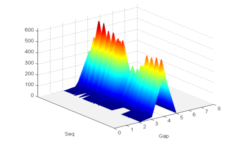	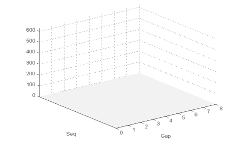	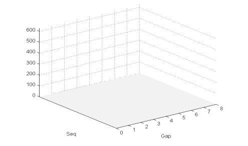
THRB:RXRA+7 (SSL: RGGTCR = Y)	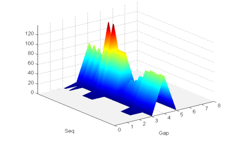	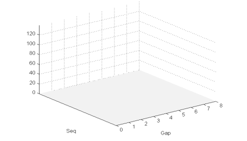	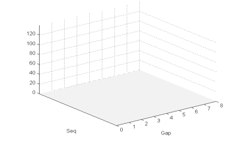
DiSEL: RGGTCR = X over Y	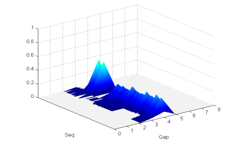	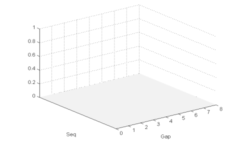	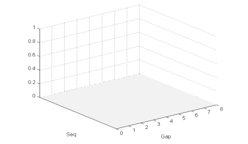
DiSEL: RGGTCR = Y over X	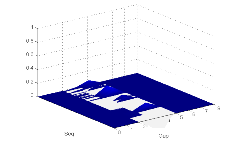	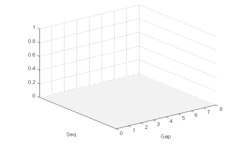	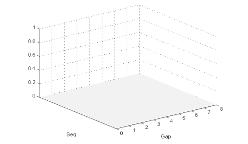

4.5 THRA Vs THRA:RXRA

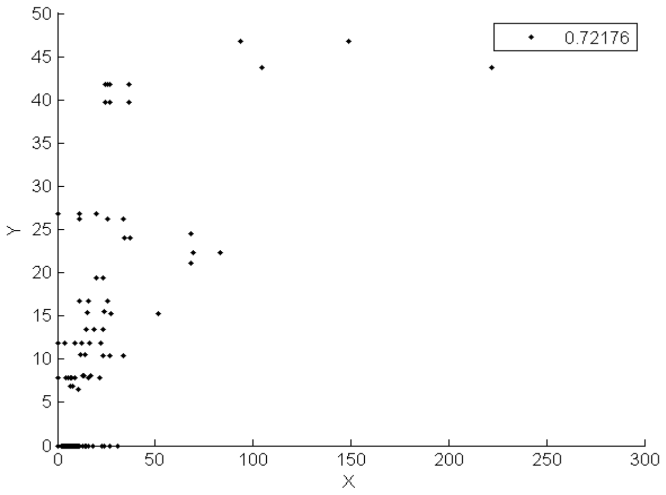

DiSEL	DR	IR	ER
THRA (SSL: RGGTCR = X)	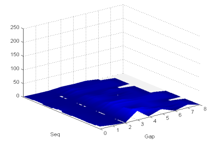	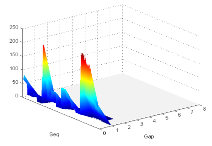	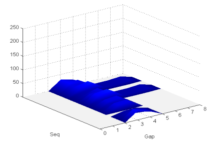
THRA:RXRA (SSL: RGGTCR = Y)	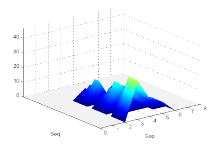	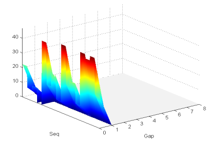	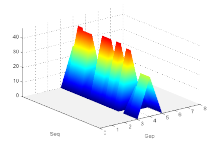
DiSEL: RGGTCR = X over Y	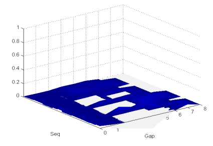	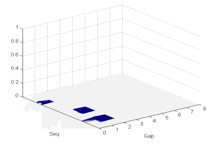	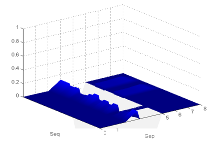
DiSEL: RGGTCR = Y over X	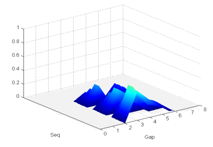	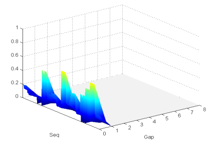	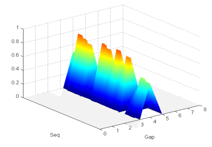

4.6 THRA Vs THRA:RXRA+7

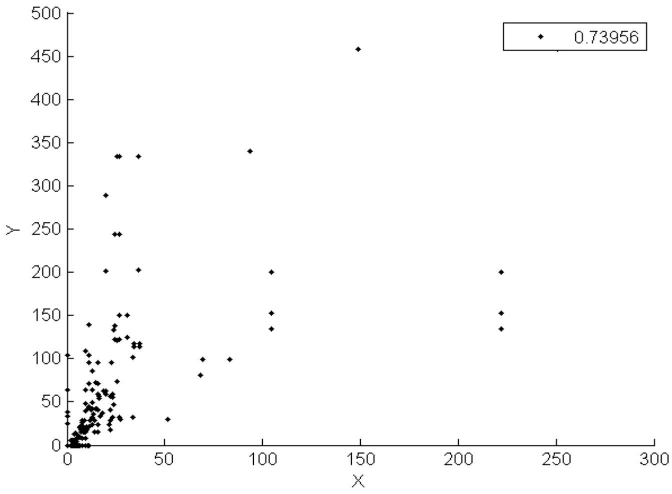

DiSEL	DR	IR	ER
THRA (SSL: RGGTCR = X)	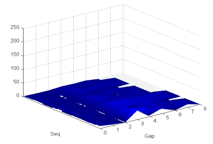	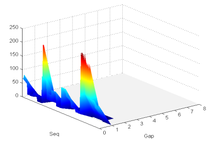	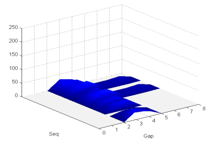
THRA:RXRA+7 (SSL: RGGTCR = Y)	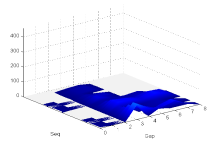	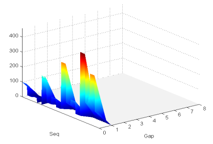	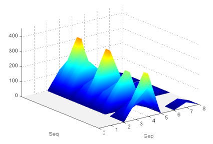
DiSEL: RGGTCR = X over Y	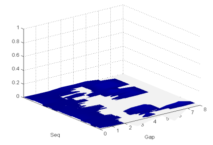	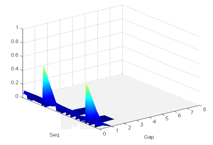	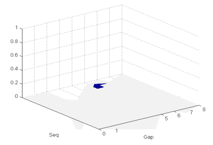
DiSEL: RGGTCR = Y over X	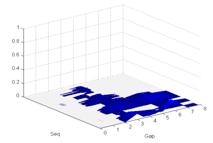	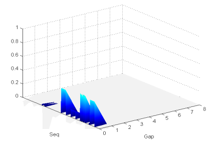	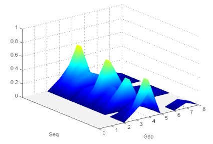

4.7 THRA Vs THRA+7

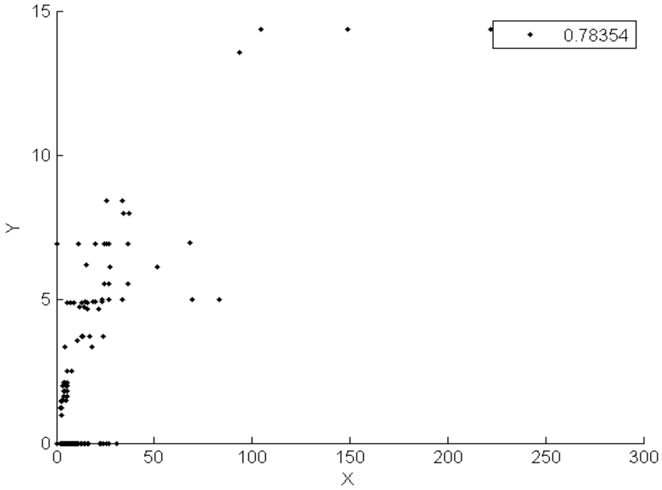

DiSEL	DR	IR	ER
THRA (SSL: RGGTCR = X)	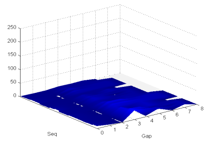	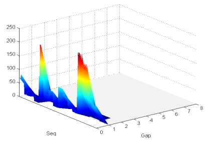	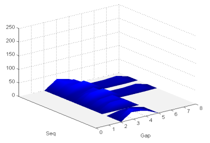
THRA+7 (SSL: RGGTCR = Y)	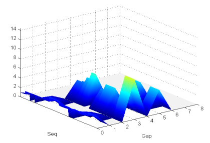	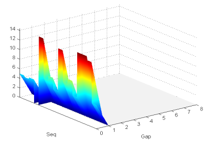	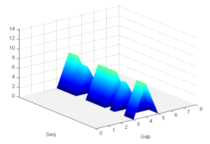
DiSEL: RGGTCR = X over Y	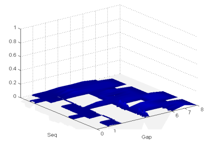	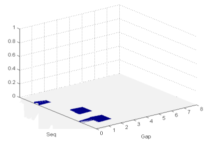	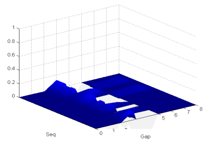
DiSEL: RGGTCR = Y over X	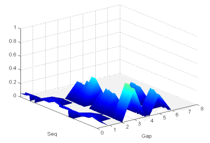	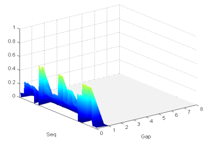	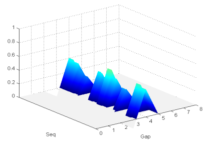

4.8 THRA:RXRA Vs THRA:RXRA+7

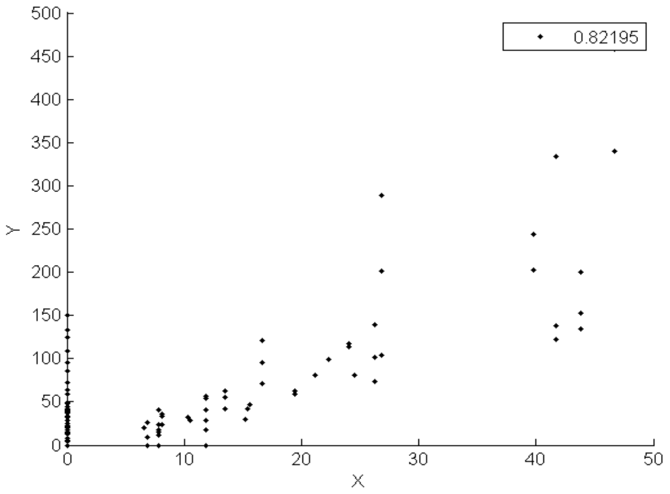

DiSEL	DR	IR	ER
THRA:RXRA (SSL: RGGTCR = X)	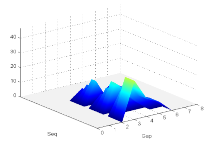	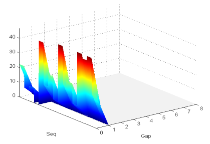	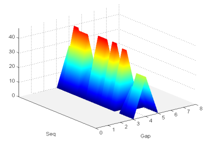
THRA:RXRA+7 (SSL: RGGTCR = Y)	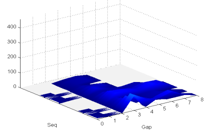	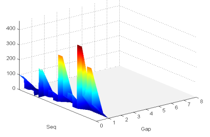	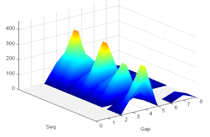
DiSEL: RGGTCR = X over Y	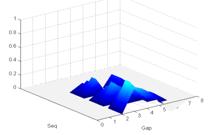	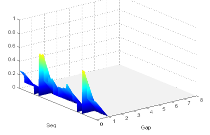	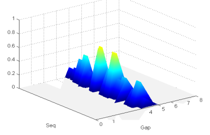
DiSEL: RGGTCR = Y over X	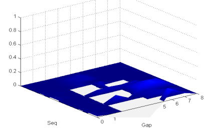	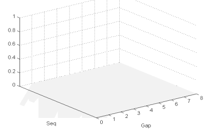	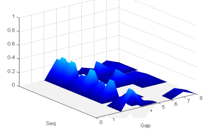

5 RAR FAMILY

5.1 RARB Vs RARG

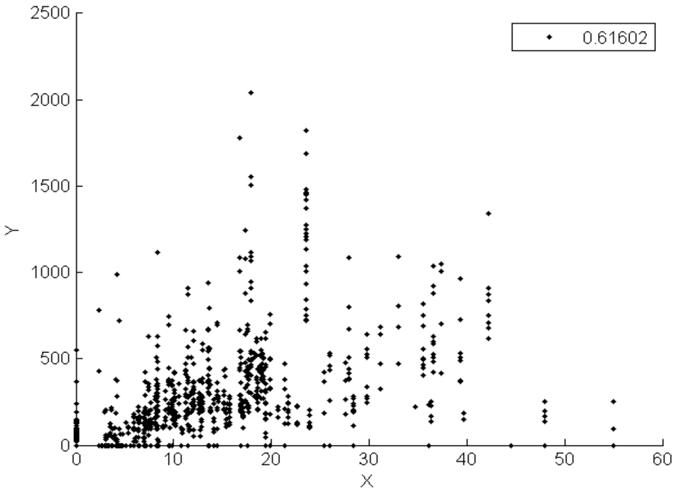

DiSEL	DR	IR	ER
RARB (SSL: RGGTCR = X)	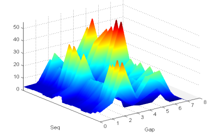	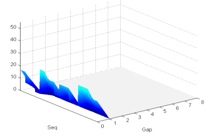	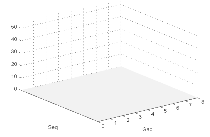
RARG (SSL: RGGTCR = Y)	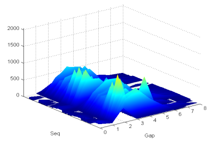	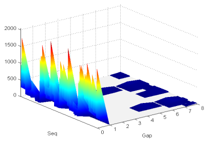	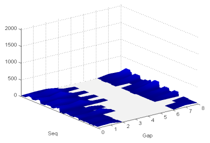
DiSEL: RGGTCR = X over Y	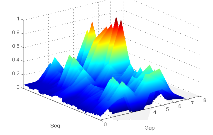	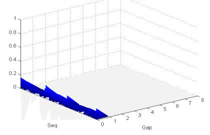	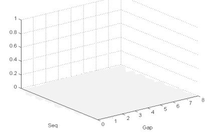
DiSEL: RGGTCR = Y over X	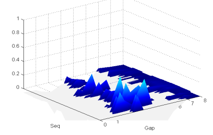	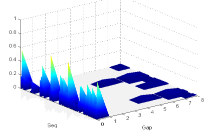	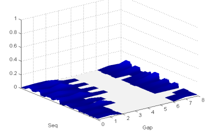

5.2 RARA Vs RARG

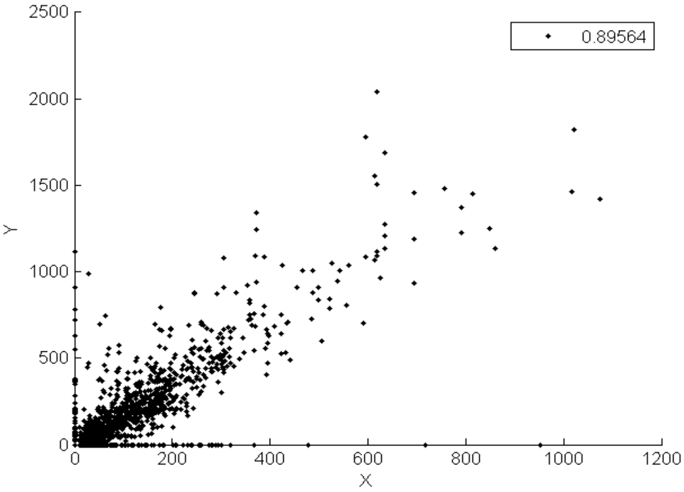

DiSEL	DR	IR	ER
RARA (SSL: RGGTCR = X)	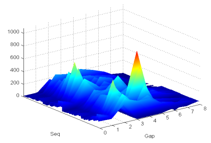	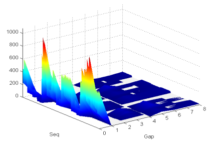	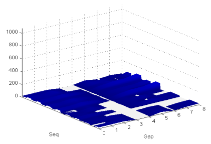
RARG (SSL: RGGTCR = Y)	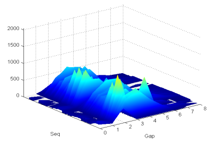	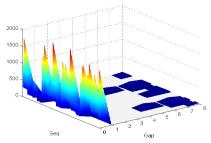	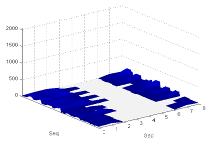
DiSEL: RGGTCR = X over Y	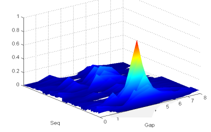	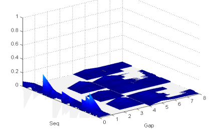	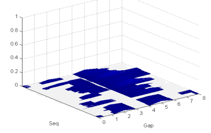
DiSEL: RGGTCR = Y over X	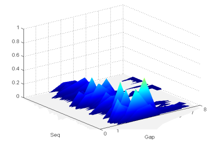	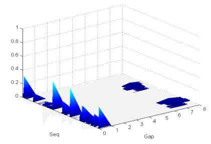	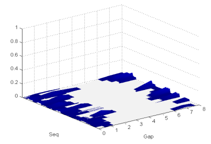

5.3 RARA Vs RARB

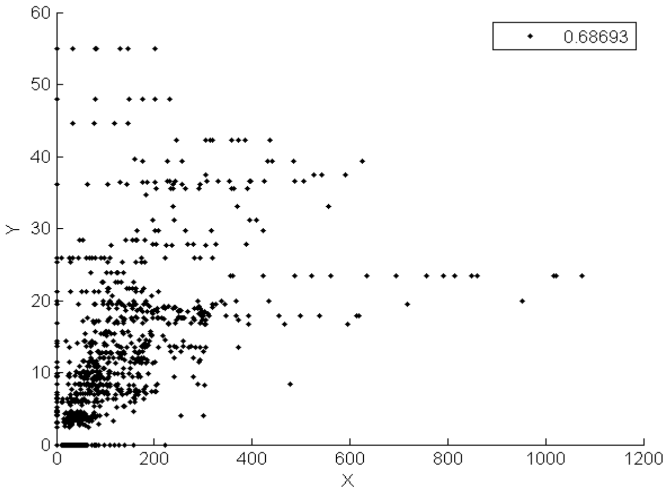

DiSEL	DR	IR	ER
RARA (SSL: RGGTCR = X)	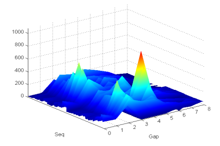	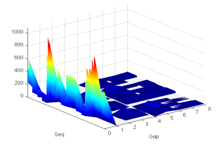	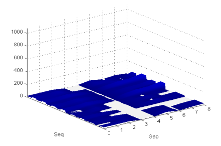
RARB (SSL: RGGTCR = Y)	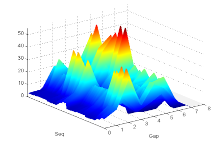	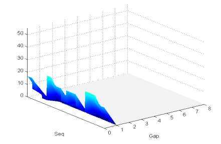	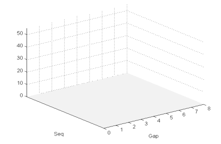
DiSEL: RGGTCR = X over Y	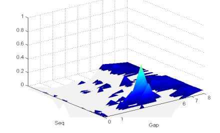	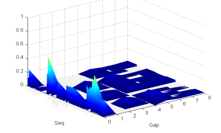	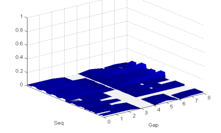
DiSEL: RGGTCR = Y over X	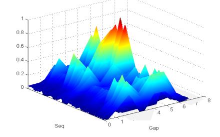	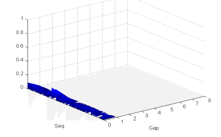	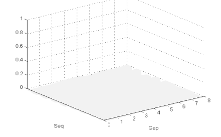

5.4 RARB+8 Vs RARG+8

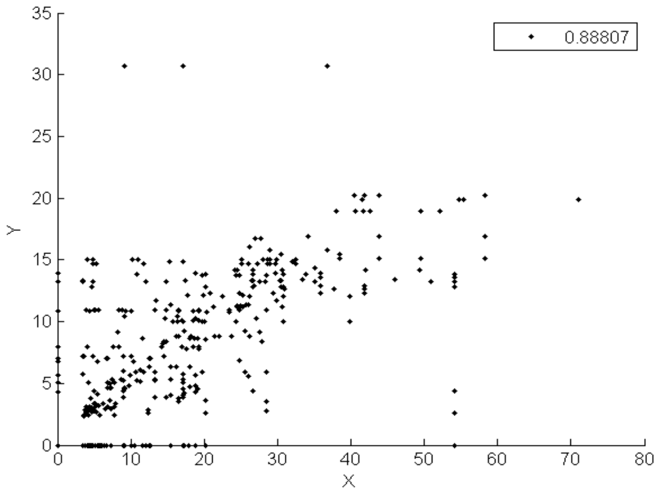

DiSEL	DR	IR	ER
RARB+8 (SSL: RGGTCR = X)	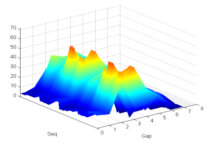	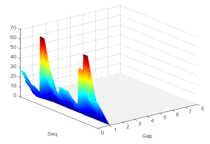	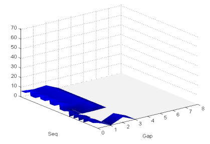
RARG+8 (SSL: RGGTCR = Y)	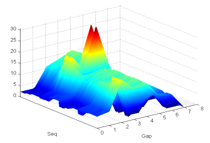	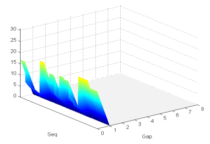	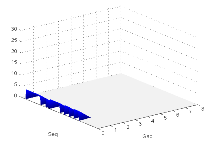
DiSEL: RGGTCR = X over Y	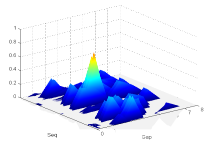	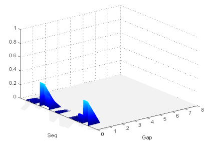	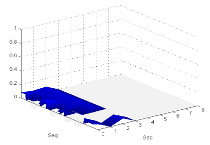
DiSEL: RGGTCR = Y over X	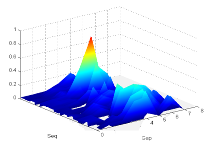	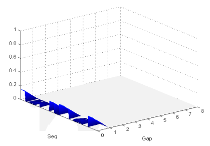	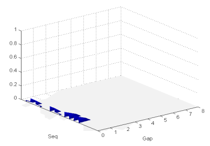

5.5 RARA+8 Vs RARG+8

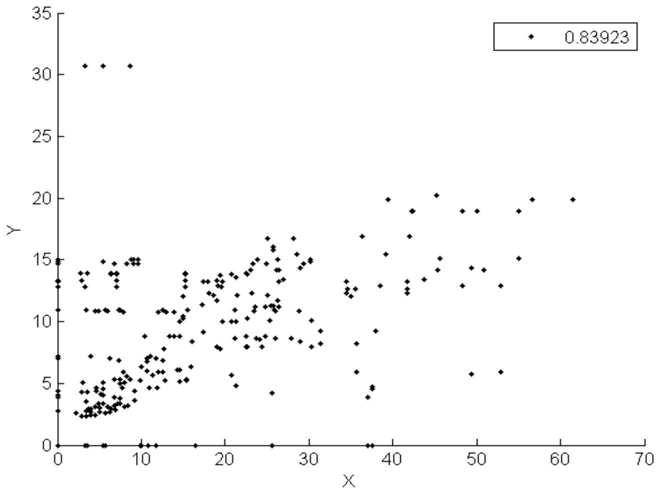

DiSEL	DR	IR	ER
RARA+8 (SSL: RGGTCR = X)	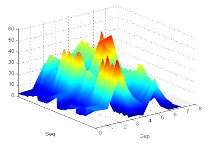	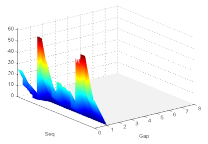	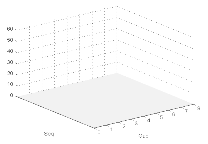
RARG+8 (SSL: RGGTCR = Y)	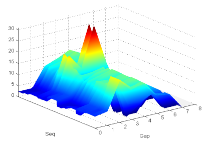	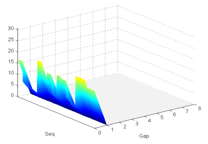	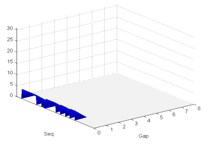
DiSEL: RGGTCR = X over Y	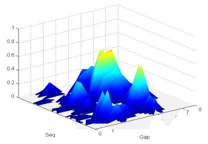	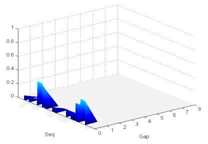	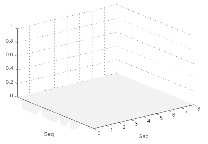
DiSEL: RGGTCR = Y over X	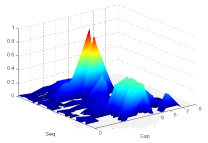	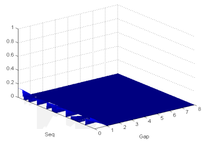	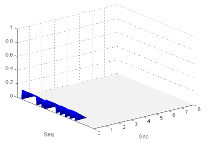

5.6 RARA+8 Vs RARB+8

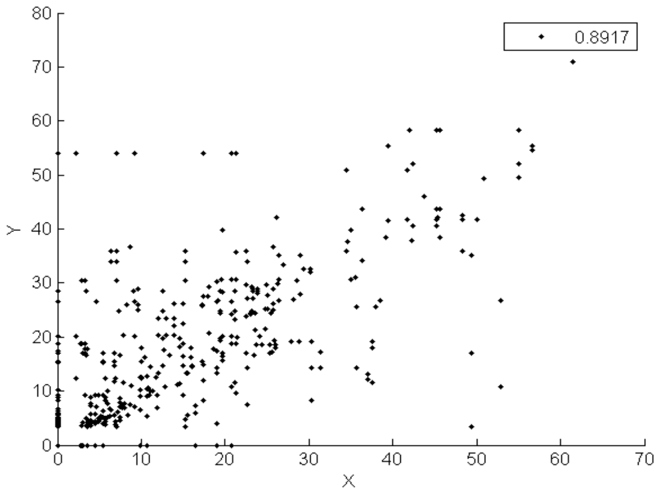

DiSEL	DR	IR	ER
RARA+8 (SSL: RGGTCR = X)	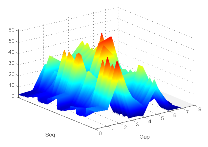	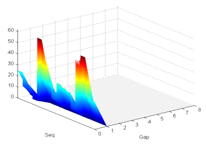	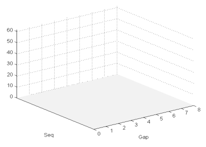
RARB+8 (SSL: RGGTCR = Y)	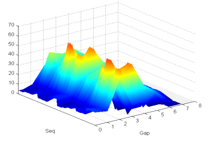	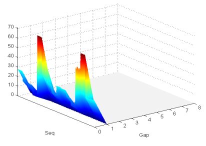	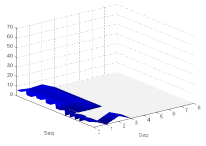
DiSEL: RGGTCR = X over Y	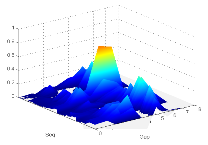	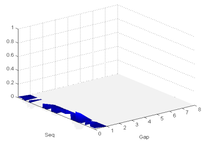	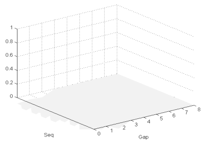
DiSEL: RGGTCR = Y over X	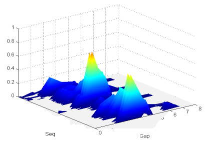	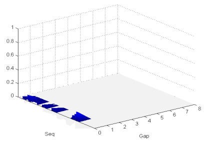	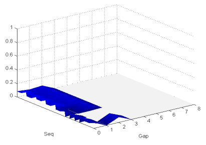

5.7 RARB:RXRA Vs RARG:RXRA

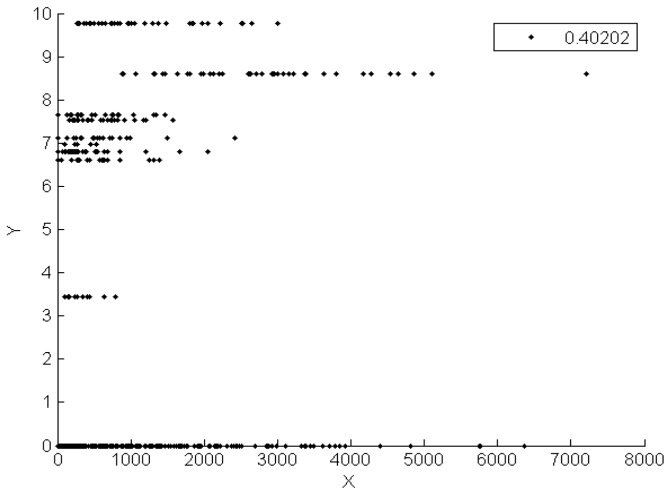

DiSEL	DR	IR	ER
RARB:RXRA (SSL: RGGTCR = X)	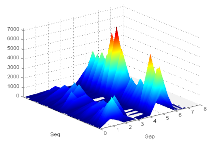	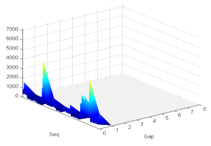	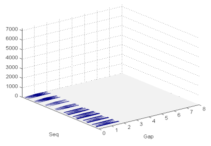
RARG:RXRA (SSL: RGGTCR = Y)	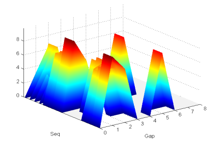	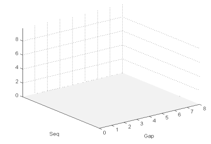	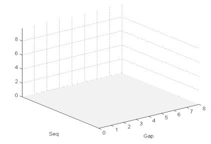
DiSEL: RGGTCR = X over Y	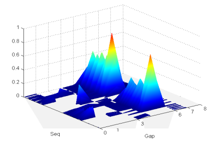	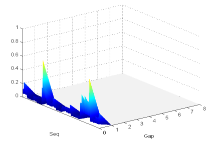	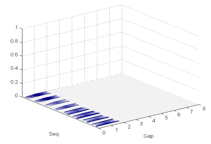
DiSEL: RGGTCR = Y over X	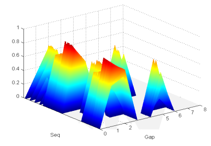	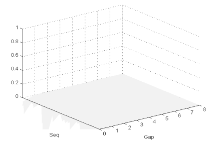	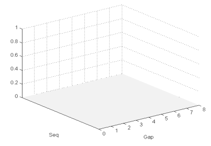

5.8 RARA:RXRA Vs RARG:RXRA

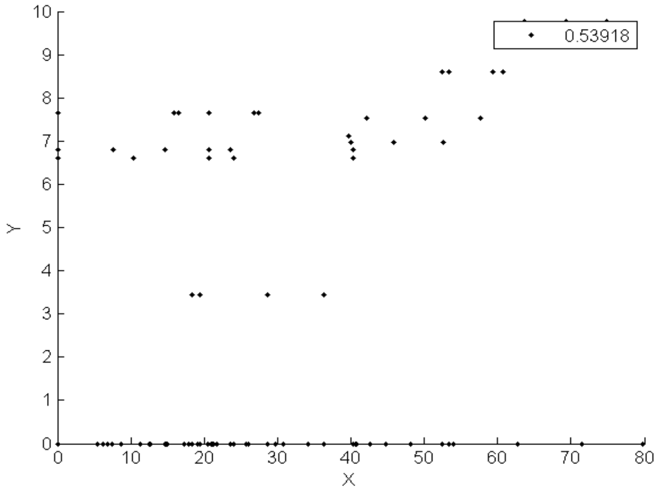

DiSEL	DR	IR	ER
RARA:RXRA (SSL: RGGTCR = X)	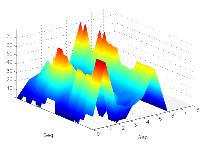	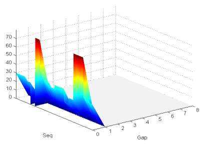	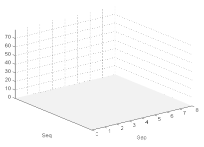
RARG:RXRA (SSL: RGGTCR = Y)	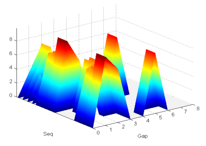	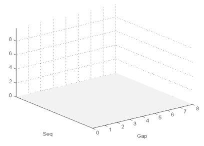	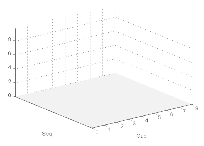
DiSEL: RGGTCR = X over Y	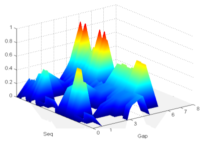	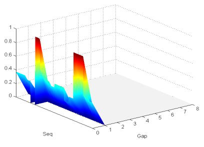	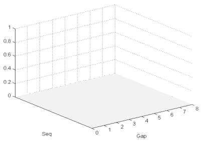
DiSEL: RGGTCR = Y over X	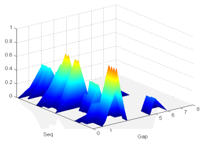	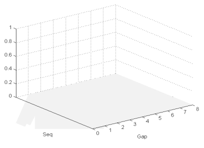	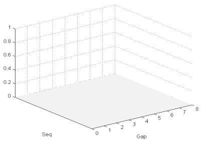

5.9 RARA:RXRA Vs RARB:RXRA

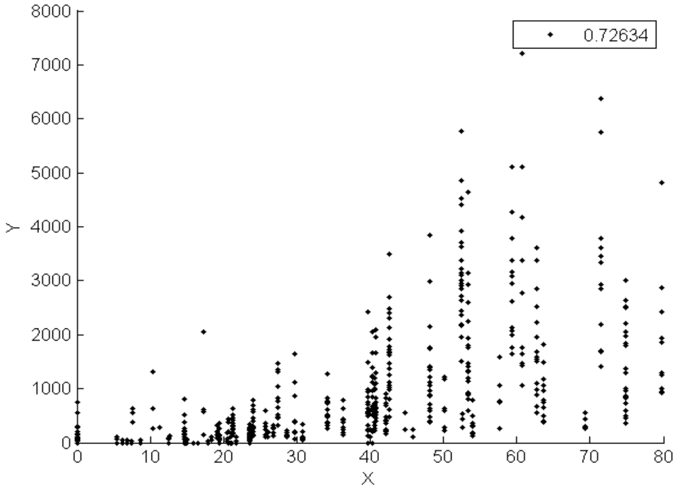

DiSEL	DR	IR	ER
RARA:RXRA (SSL: RGGTCR = X)	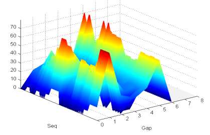	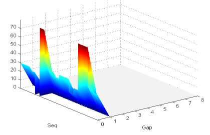	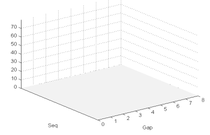
RARB:RXRA (SSL: RGGTCR = Y)	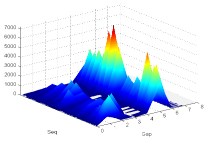	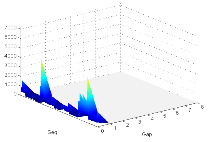	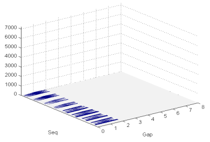
DiSEL: RGGTCR = X over Y	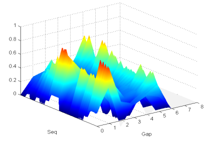	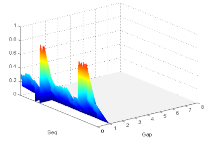	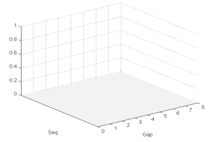
DiSEL: RGGTCR = Y over X	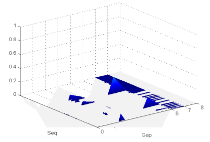	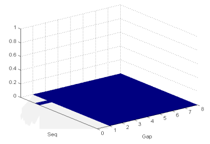	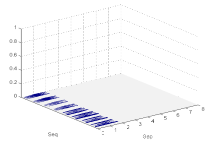

5.10 RARG Vs RARG+8

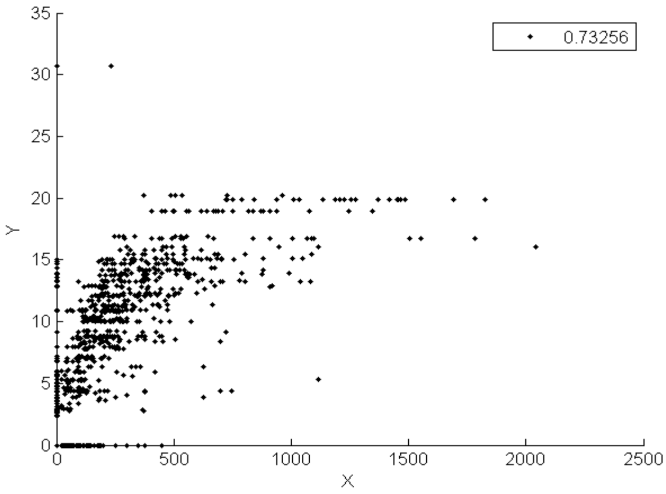

DiSEL	DR	IR	ER
RARG (SSL: RGGTCR = X)	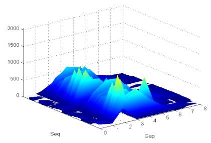	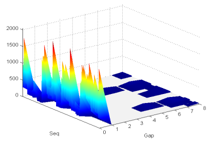	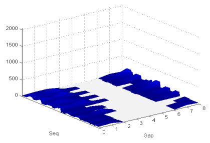
RARG+8 (SSL: RGGTCR = Y)	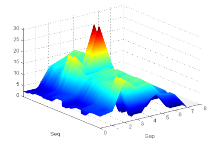	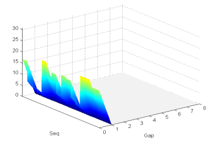	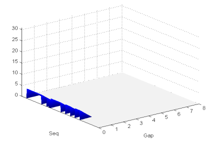
DiSEL: RGGTCR = X over Y	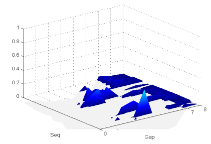	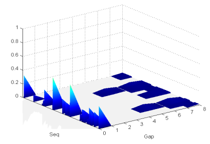	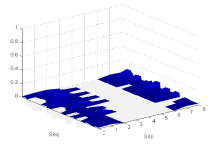
DiSEL: RGGTCR = Y over X	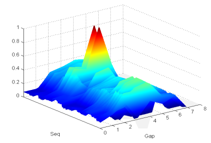	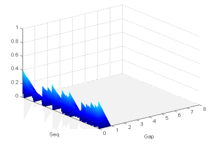	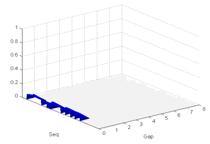

5.11 RARG Vs RARG:RXRA

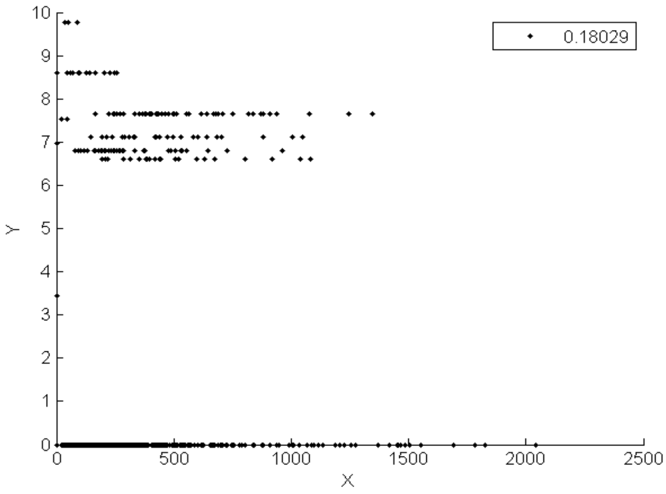

DiSEL	DR	IR	ER
RARG (SSL: RGGTCR = X)	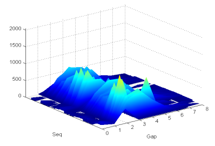	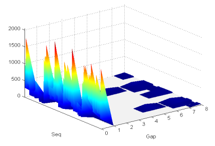	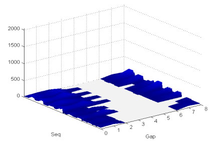
RARG:RXRA (SSL: RGGTCR = Y)	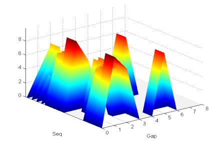	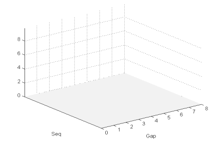	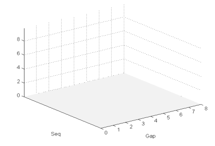
DiSEL: RGGTCR = X over Y	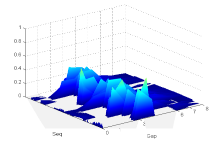	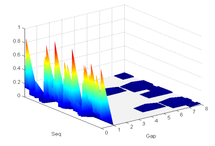	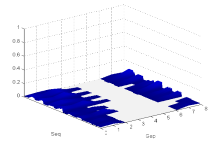
DiSEL: RGGTCR = Y over X	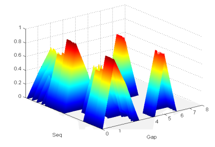	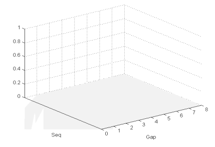	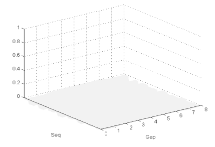

5.12 RARB Vs RARB+8

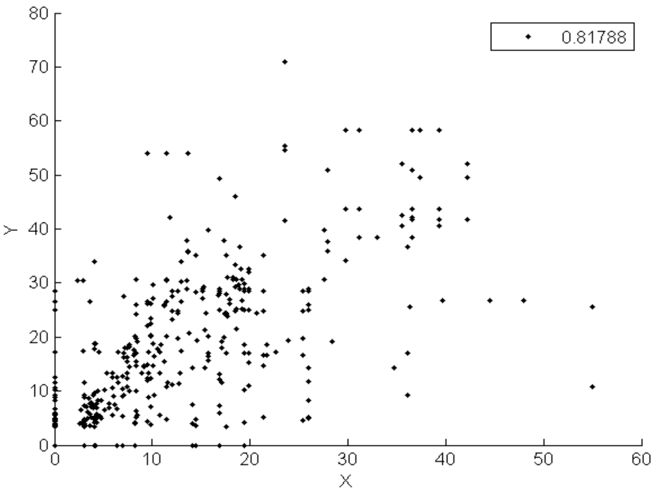

DiSEL	DR	IR	ER
RARB (SSL: RGGTCR = X)	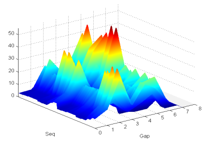	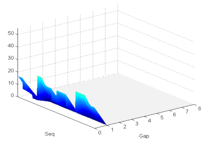	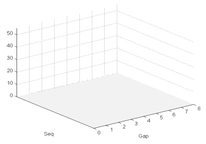
RARB+8 (SSL: RGGTCR = Y)	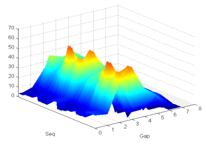	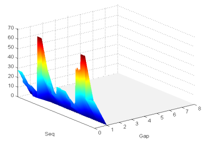	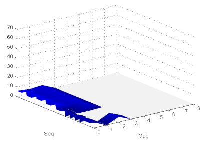
DiSEL: RGGTCR = X over Y	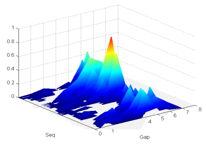	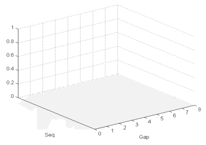	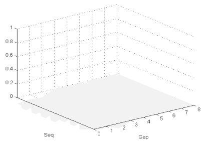
DiSEL: RGGTCR = Y over X	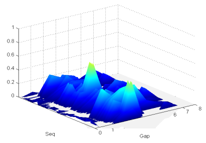	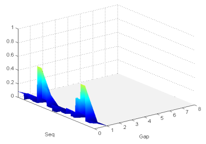	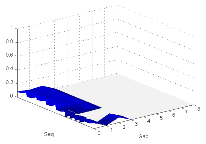

5.13 RARB Vs RARB:RXRA

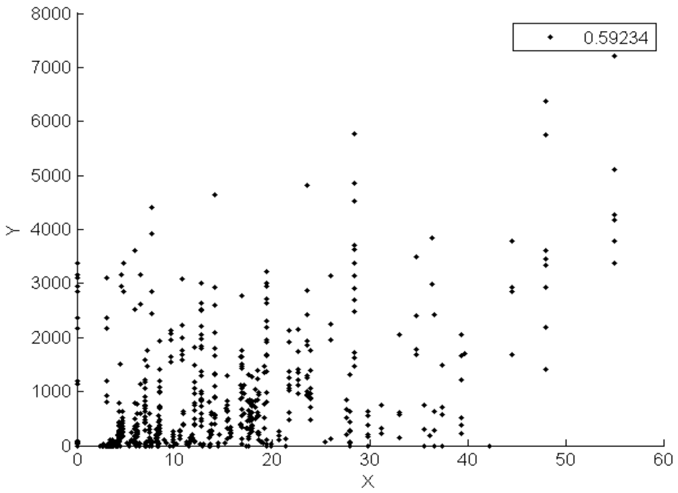

DiSEL	DR	IR	ER
RARB (SSL: RGGTCR = X)	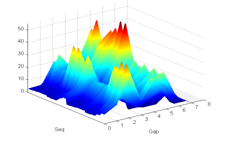	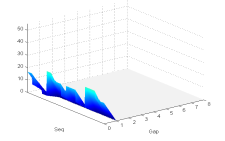	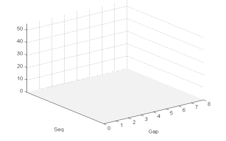
RARB:RXRA (SSL: RGGTCR = Y)	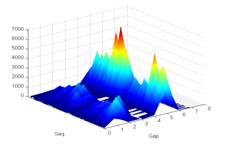	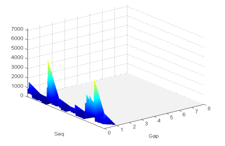	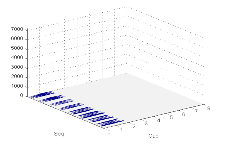
DiSEL: RGGTCR = X over Y	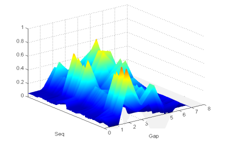	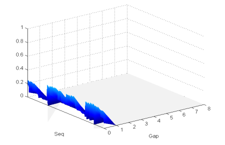	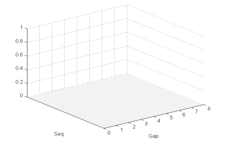
DiSEL: RGGTCR = Y over X	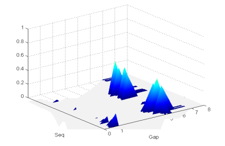	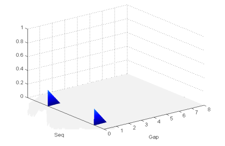	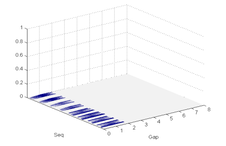

5.14 RARA Vs RARA+8

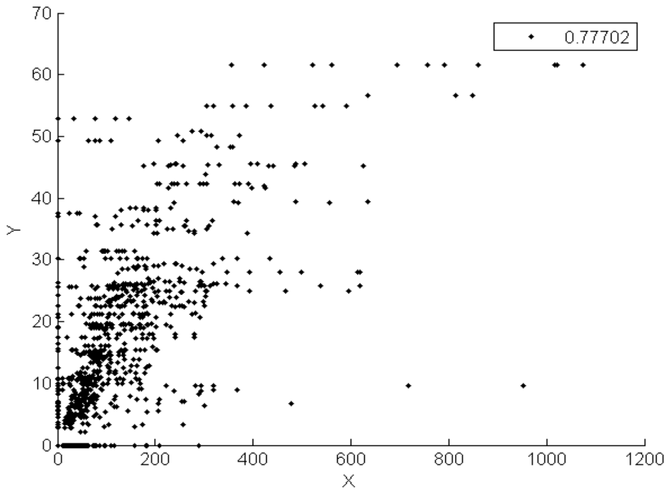

DiSEL	DR	IR	ER
RARA (SSL: RGGTCR = X)	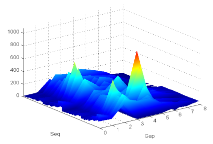	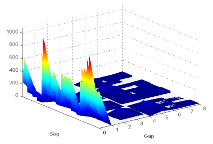	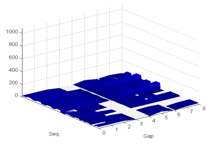
RARA+8 (SSL: RGGTCR = Y)	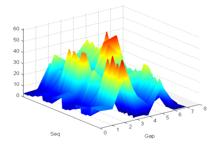	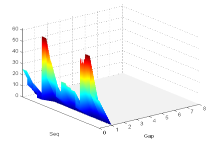	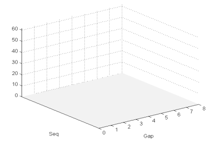
DiSEL: RGGTCR = X over Y	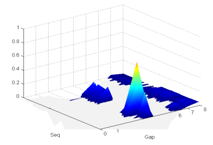	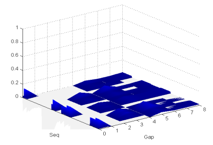	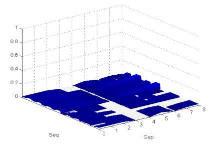
DiSEL: RGGTCR = Y over X	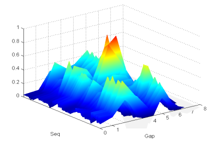	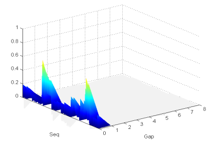	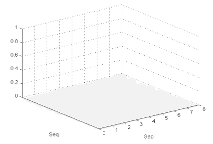

5.15 RARA Vs RARA:RXRA

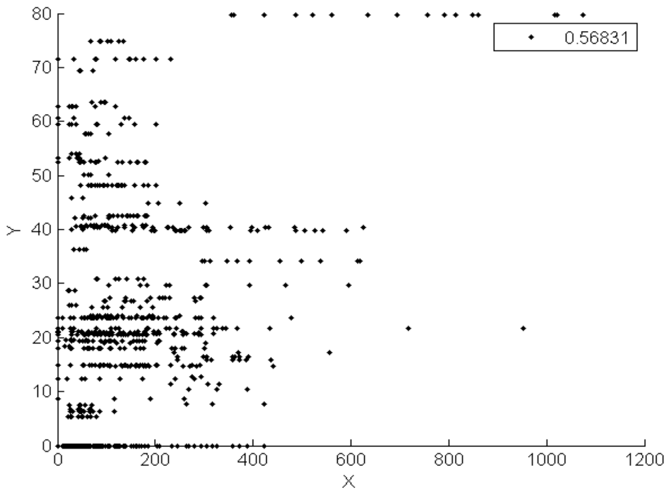

DiSEL	DR	IR	ER
RARA (SSL: RGGTCR = X)	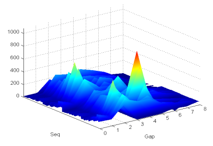	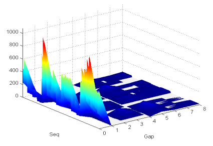	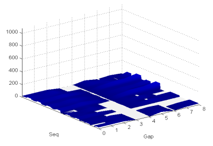
RARA:RXRA (SSL: RGGTCR = Y)	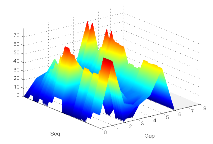	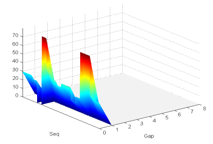	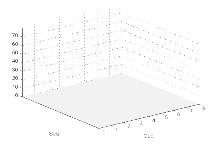
DiSEL: RGGTCR = X over Y	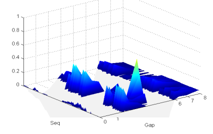	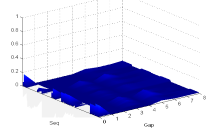	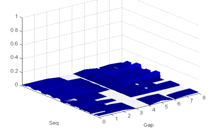
DiSEL: RGGTCR = Y over X	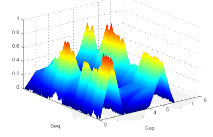	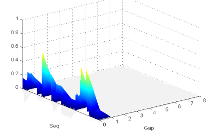	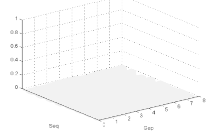

6 VDR FAMILY

6.1 PXR Vs PXR+9

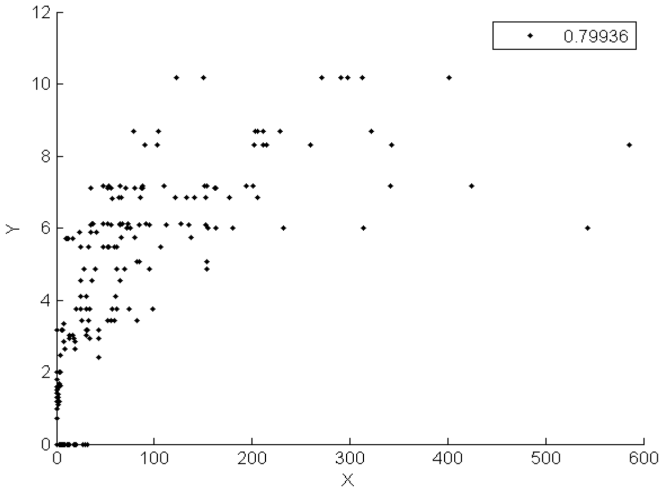

DiSEL	DR	IR	ER
PXR (SSL: RGKTCR = X)	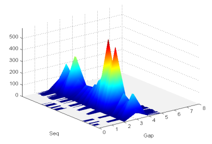	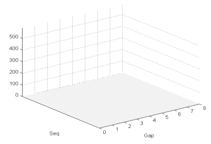	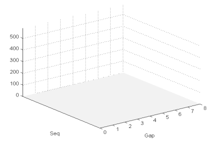
PXR+9 (SSL: RGKTCR = Y)	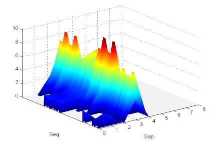	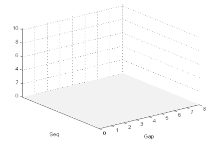	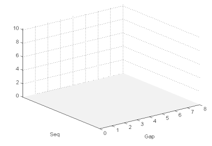
DiSEL: RGKTCR = X over Y	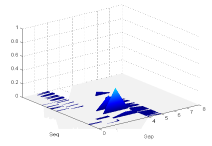	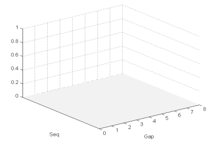	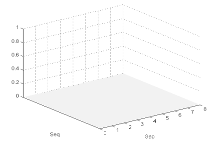
DiSEL: RGKTCR = Y over X	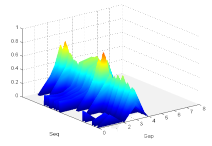	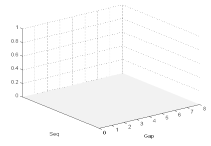	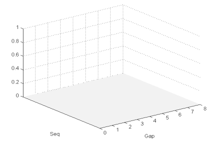

6.2 VDR Vs VDR:RXRA

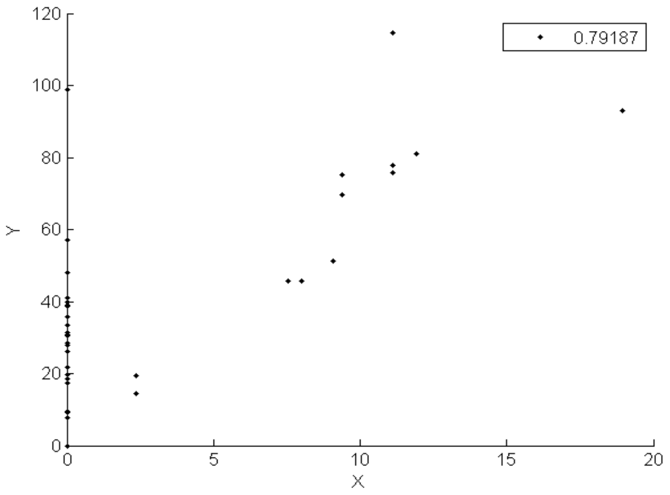

DiSEL	DR	IR	ER
VDR (SSL: RGKTCR = X)	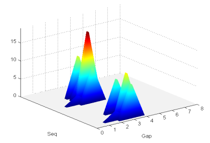	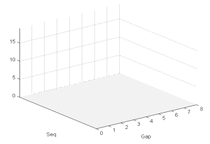	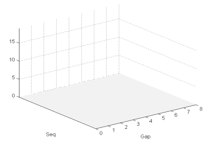
VDR:RXRA (SSL: RGKTCR = Y)	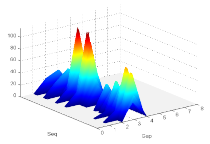	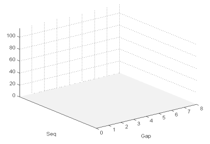	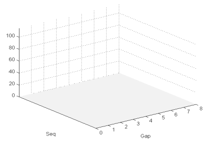
DiSEL: RGKTCR = X over Y	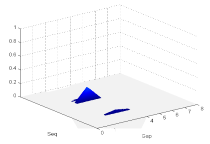	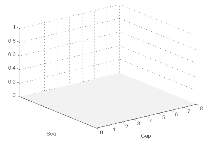	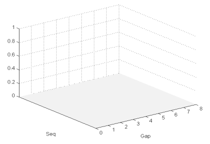
DiSEL: RGKTCR = Y over X	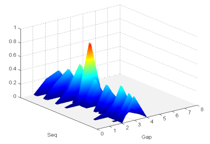	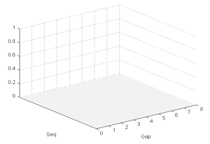	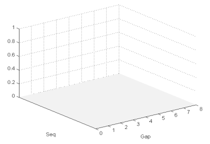

6.3 VDR Vs VDR+10

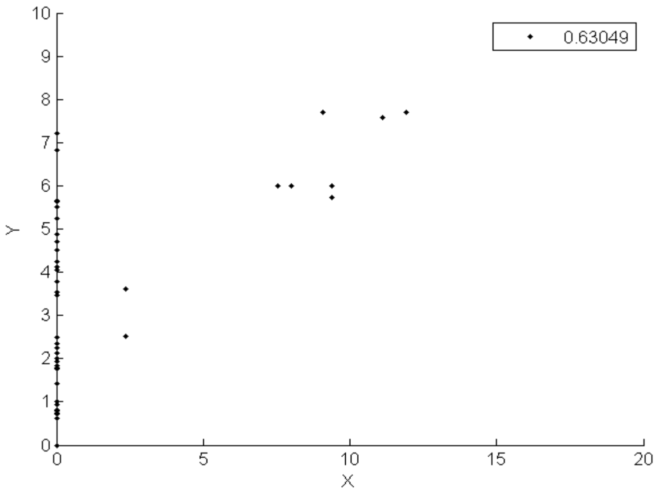

DiSEL	DR	IR	ER
VDR (SSL: RGKTCR = X)	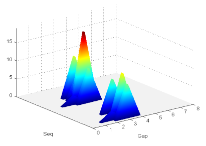	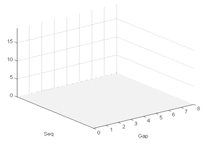	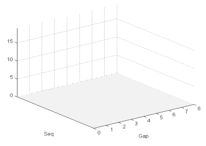
VDR+10 (SSL: RGK-TCR = Y)	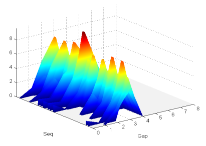	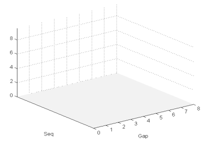	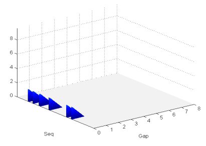
DiSEL: RGKTCR = X over Y	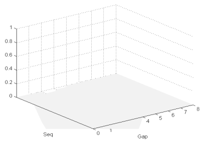	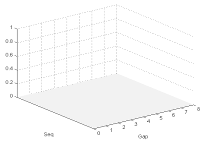	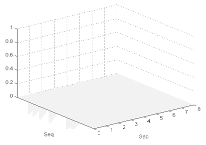
DiSEL: RGKTCR = Y over X	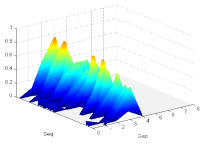	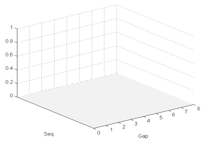	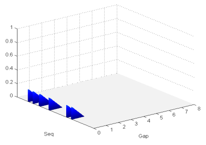

6.4 PXR Vs VDR

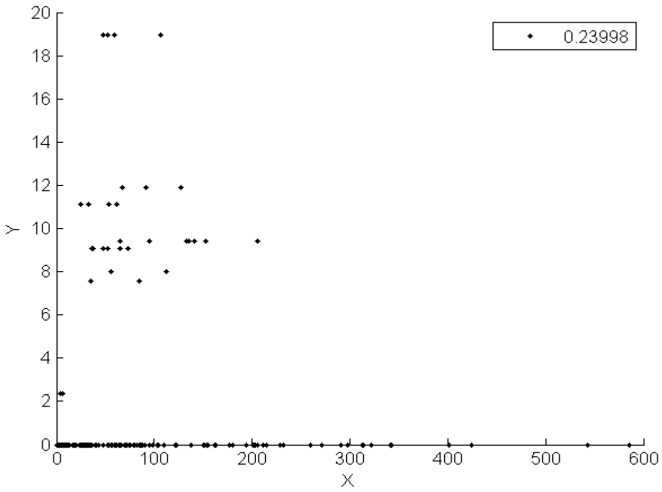

DiSEL	DR	IR	ER
PXR (SSL: RGKTCR = X)	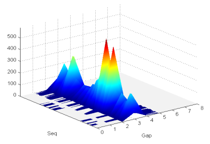	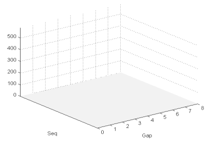	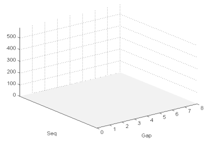
VDR (SSL: RGKTCR = Y)	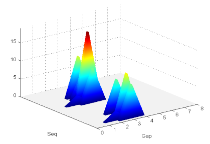	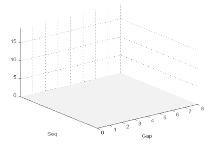	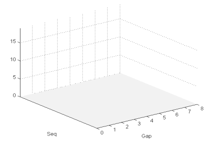
DiSEL: RGKTCR = X over Y	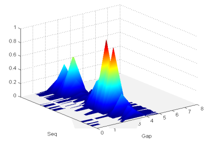	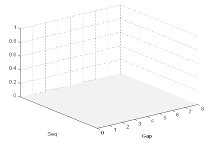	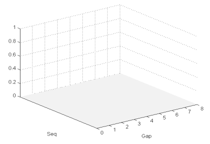
DiSEL: RGKTCR = Y over X	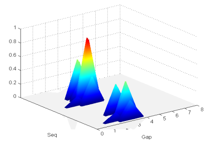	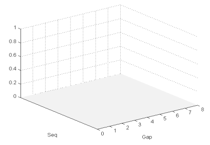	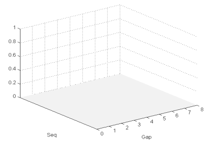

6.5 PXR+9 Vs VDR+10

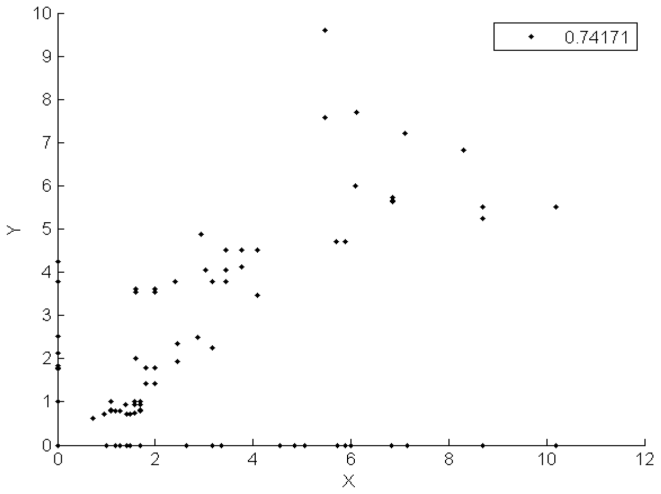

DiSEL	DR	IR	ER
PXR+9 (SSL: RGK-TCR = X)	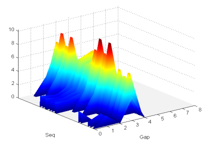	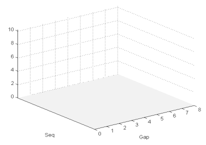	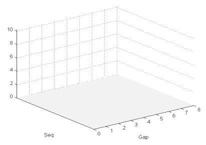
VDR+10 (SSL: RGK-TCR = Y)	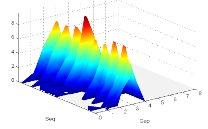	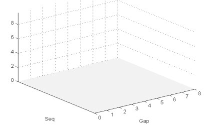	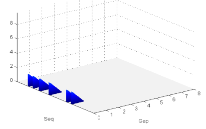
DiSEL: RGKTCR = X over Y	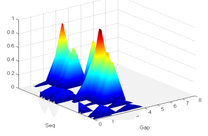	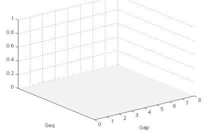	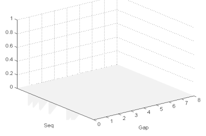
DiSEL: RGKTCR = Y over X	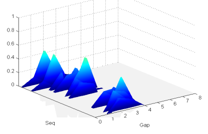	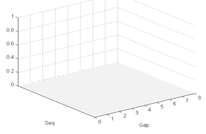	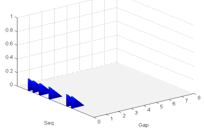

7 LXR FAMILY

7.1 FXR Vs FXR+11

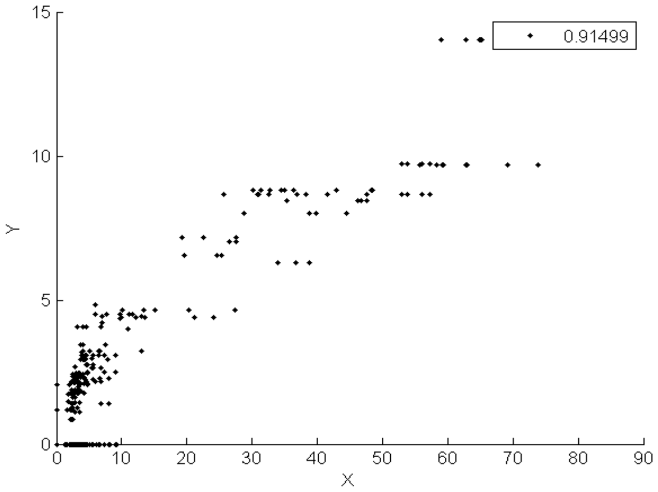

DiSEL	DR	IR	ER
FXR (SSL: RGKTCR = X)	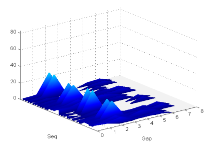	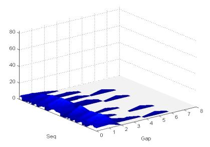	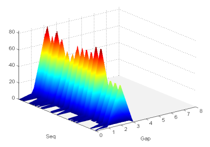
FXR+11 (SSL: RGK-TCR = Y)	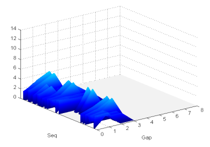	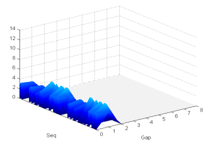	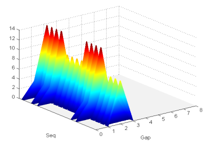
DiSEL: RGKTCR = X over Y	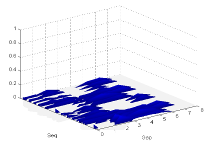	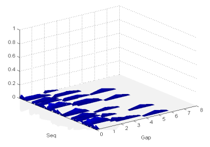	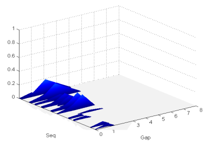
DiSEL: RGKTCR = Y over X	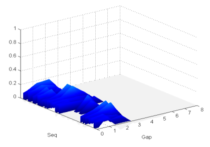	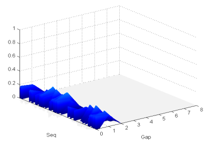	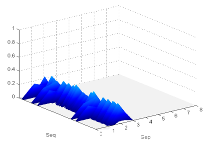

7.2 LXRA Vs LXRA+12

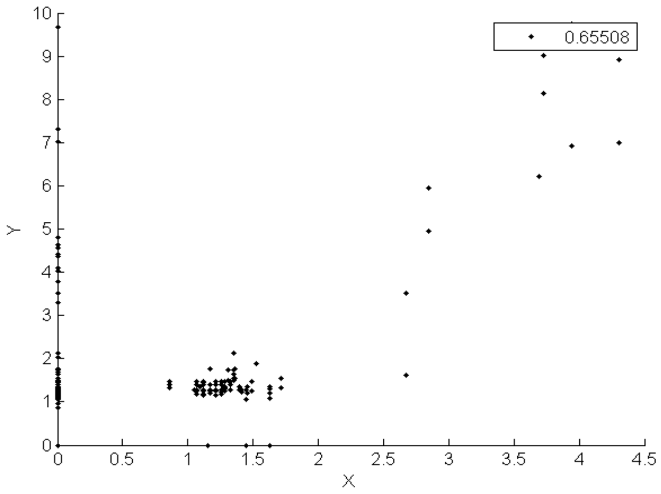

DiSEL	DR	IR	ER
LXRA (SSL: RGK-TCR = X)	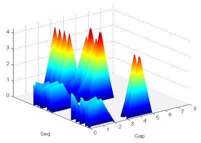	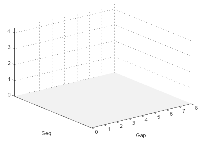	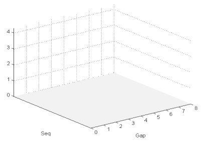
LXRA+12 (SSL: RGKTCR = Y)	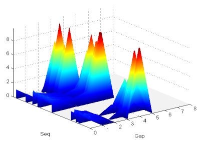	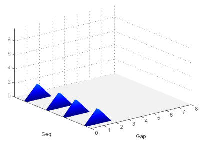	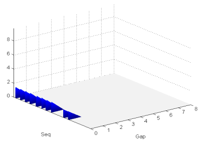
DiSEL: RGKTCR = X over Y	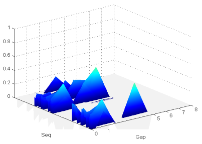	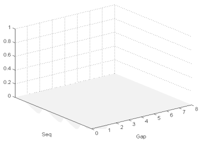	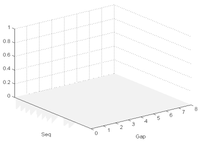
DiSEL: RGKTCR = Y over X	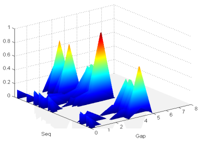	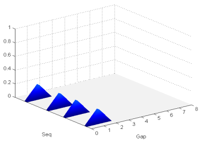	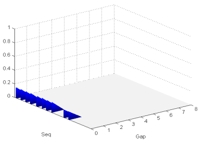

7.3 LXRA Vs LXRB:RXRA

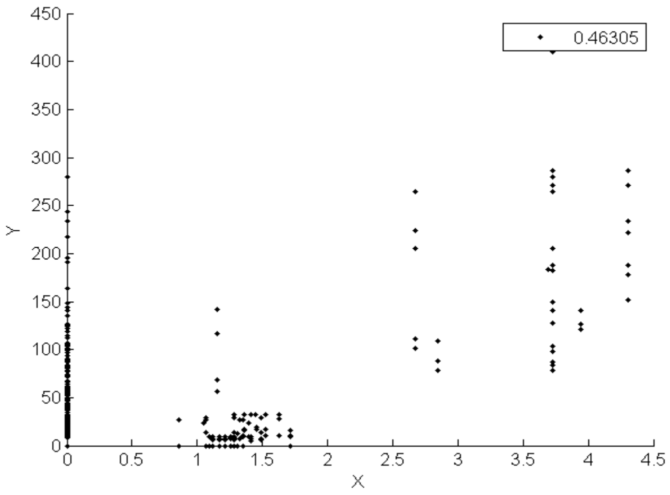

DiSEL	DR	IR	ER
LXRA (SSL: RGK-TCR = X)	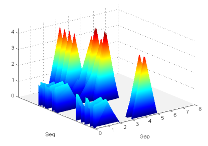	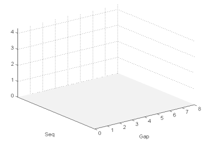	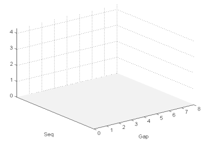
LXRB:RXRA (SSL: RGKTCR = Y)	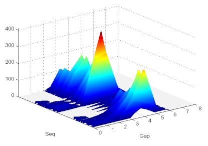	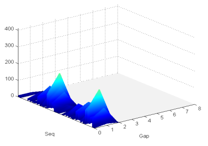	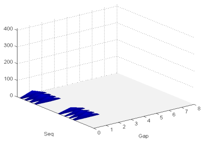
DiSEL: RGKTCR = X over Y	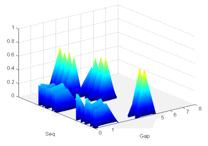	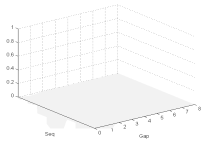	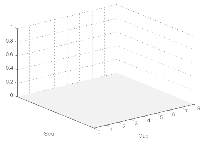
DiSEL: RGKTCR = Y over X	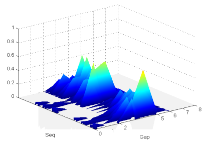	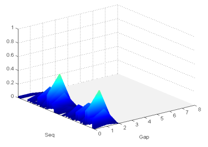	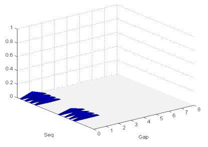

8 PPAR FAMILY

8.1 PPARD Vs PPARD+13

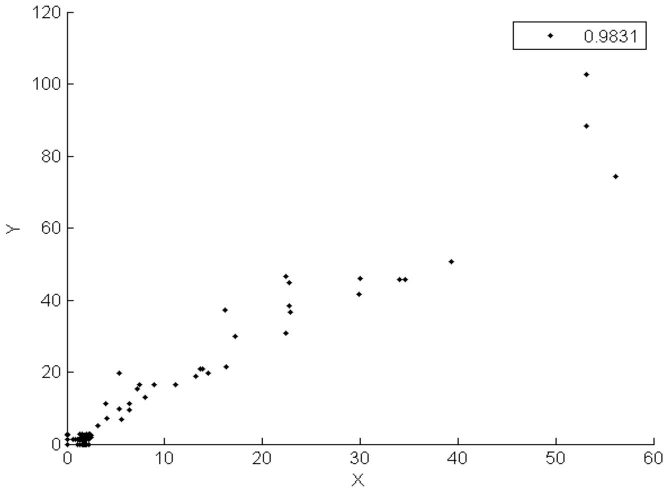

DiSEL	DR	IR	ER
PPARD (SSL: RGGTCR = X)	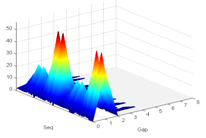	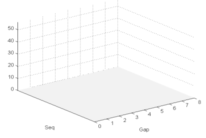	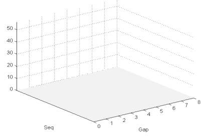
PPARD+13 (SSL: RGGTCR = Y)	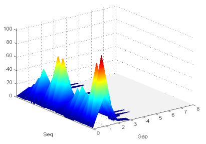	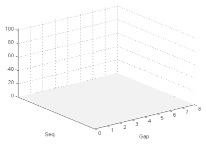	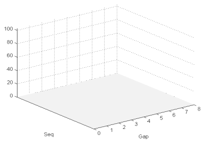
DiSEL: RGGTCR = X over Y	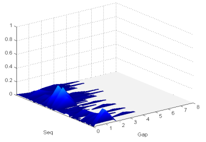	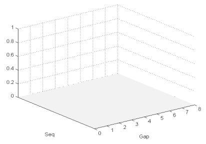	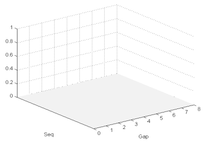
DiSEL: RGGTCR = Y over X	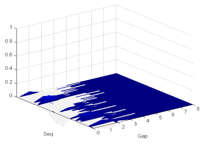	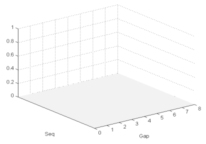	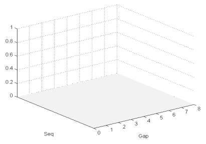

8.2 PPARD Vs PPARD+14

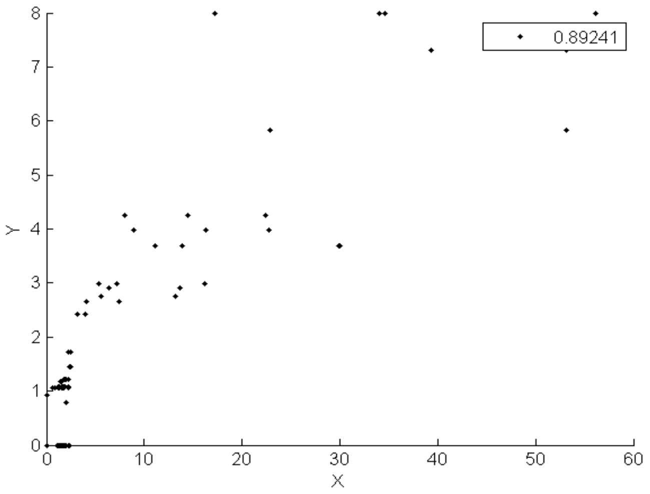

DiSEL	DR	IR	ER
PPARD (SSL: RGGTCR = X)	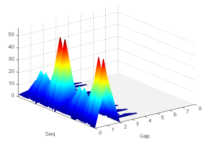	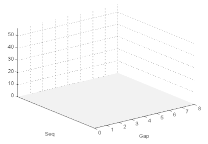	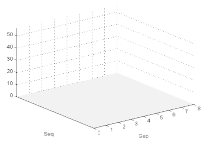
PPARD+14 (SSL: RGGTCR = Y)	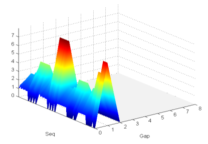	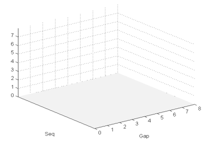	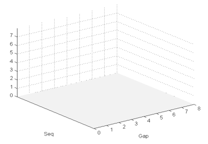
DiSEL: RGGTCR = X over Y	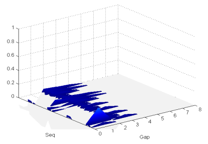	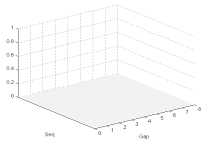	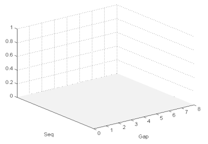
DiSEL: RGGTCR = Y over X	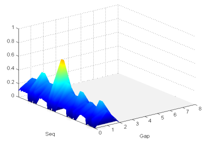	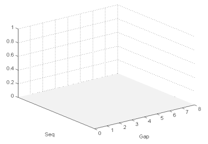	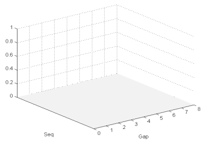

8.3 PPARD+13 Vs PPARD+14

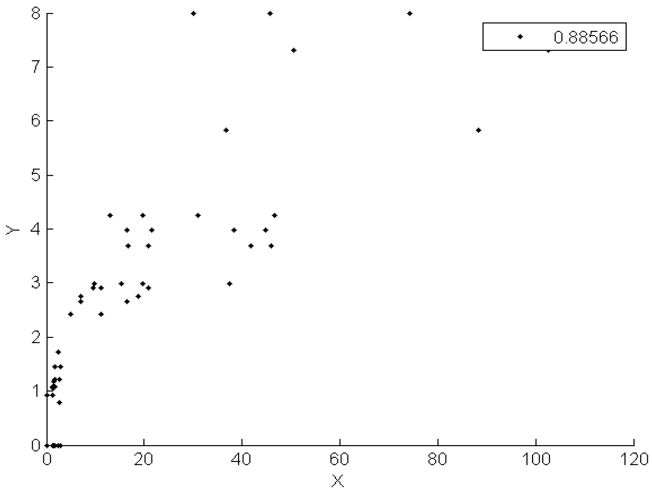

DiSEL	DR	IR	ER
PPARD+13 (SSL: RGGTCR = X)	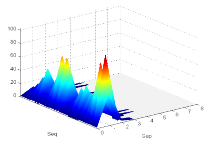	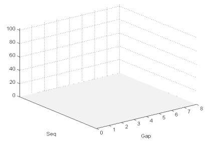	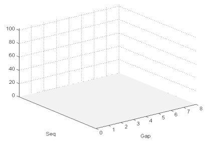
PPARD+14 (SSL: RGGTCR = Y)	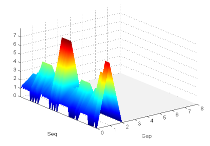	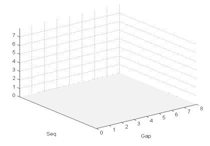	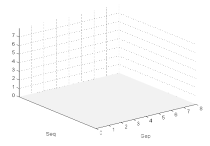
DiSEL: RGGTCR = X over Y	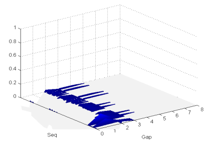	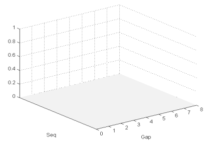	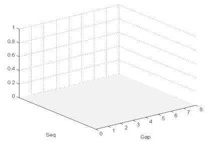
DiSEL: RGGTCR = Y over X	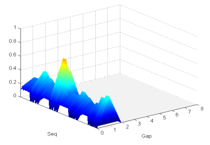	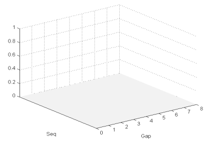	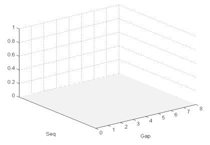

8.4 PPARG Vs PPARG+14

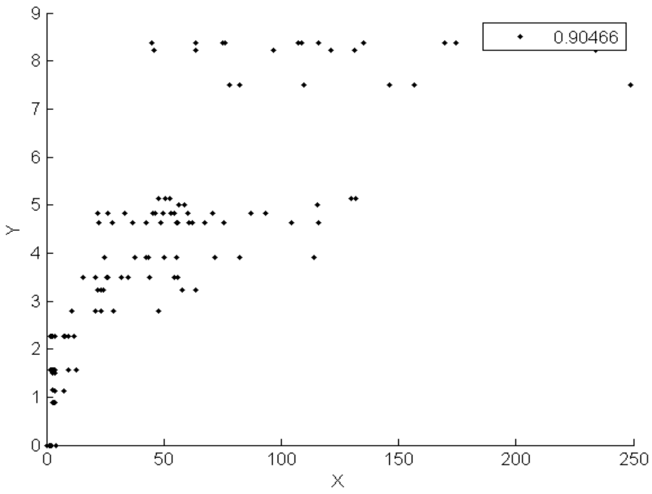

DiSEL	DR	IR	ER
PPARG (SSL: RGGTCR = X)	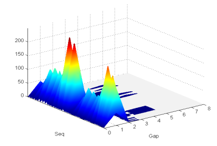	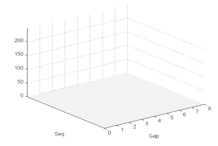	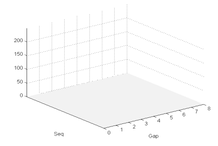
PPARG+14 (SSL: RGGTCR = Y)	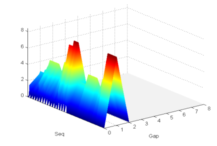	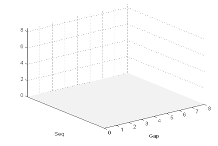	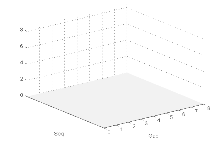
DiSEL: RGGTCR = X over Y	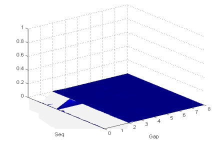	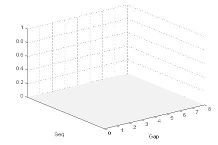	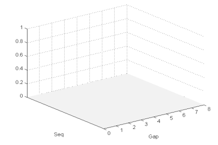
DiSEL: RGGTCR = Y over X	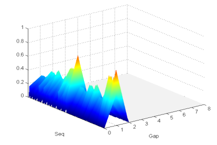	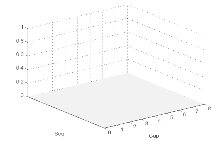	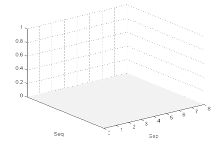

8.5 PPARG Vs PPARG+15

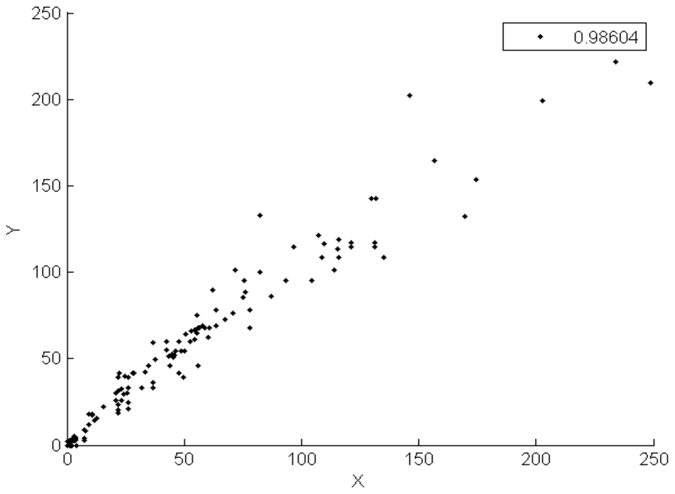

DiSEL	DR	IR	ER
PPARG (SSL: RGGTCR = X)	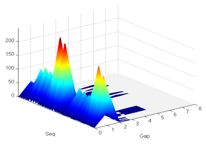	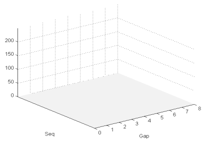	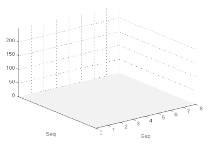
PPARG+15 (SSL: RGGTCR = Y)	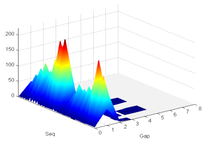	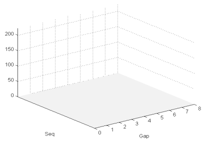	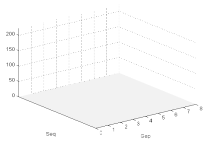
DiSEL: RGGTCR = X over Y	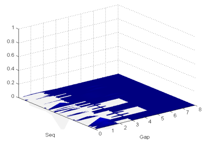	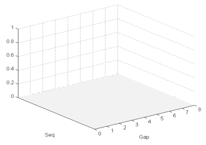	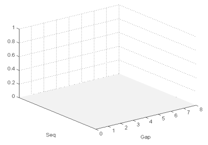
DiSEL: RGGTCR = Y over X	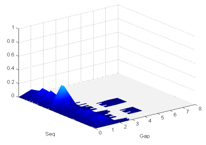	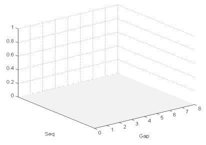	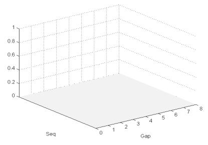

8.6 PPARG+14 Vs PPARG+15

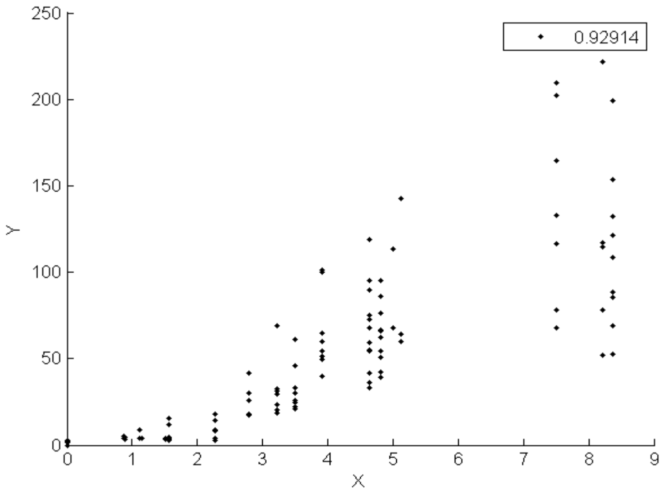

DiSEL	DR	IR	ER
PPARG+14 (SSL: RGGTCR = X)	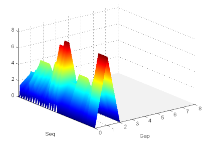	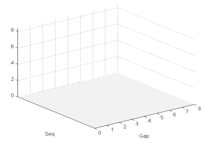	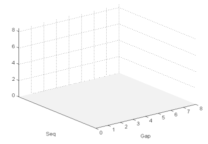
PPARG+15 (SSL: RGGTCR = Y)	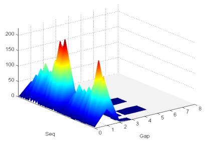	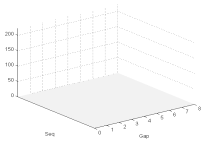	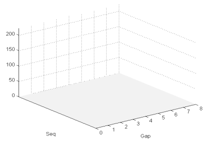
DiSEL: RGGTCR = X over Y	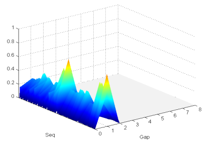	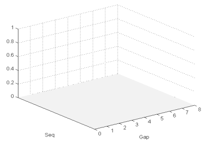	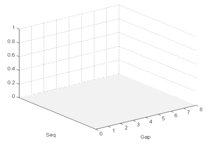
DiSEL: RGGTCR = Y over X	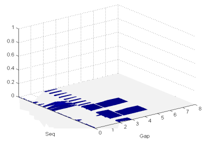	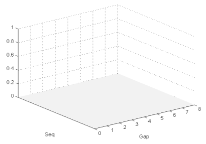	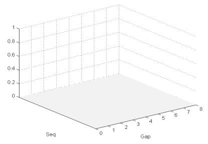

8.7 PPARD Vs PPARG

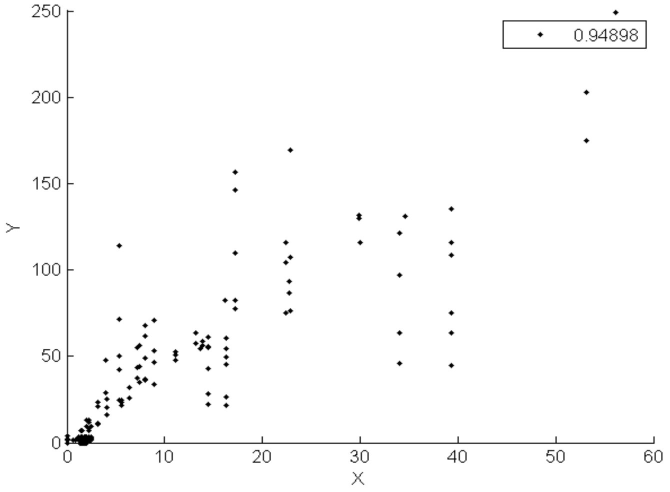

DiSEL	DR	IR	ER
PPARD (SSL: RGGTCR = X)	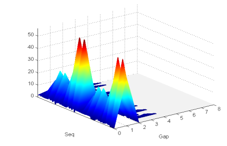	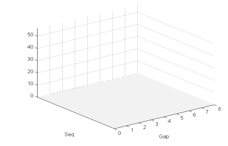	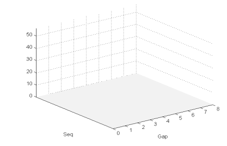
PPARG (SSL: RGGTCR = Y)	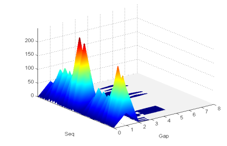	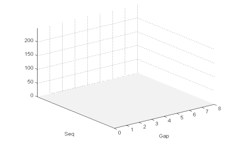	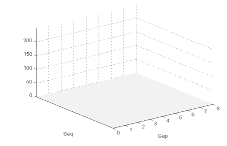
DiSEL: RGGTCR = X over Y	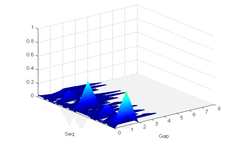	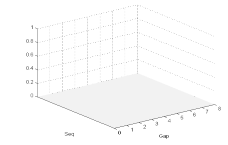	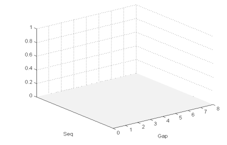
DiSEL: RGGTCR = Y over X	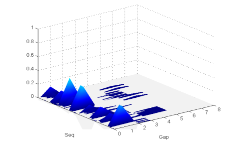	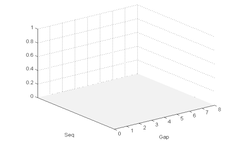	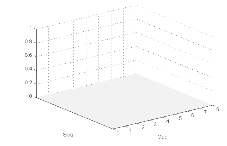

8.8 PPARD+14 Vs PPARG+14

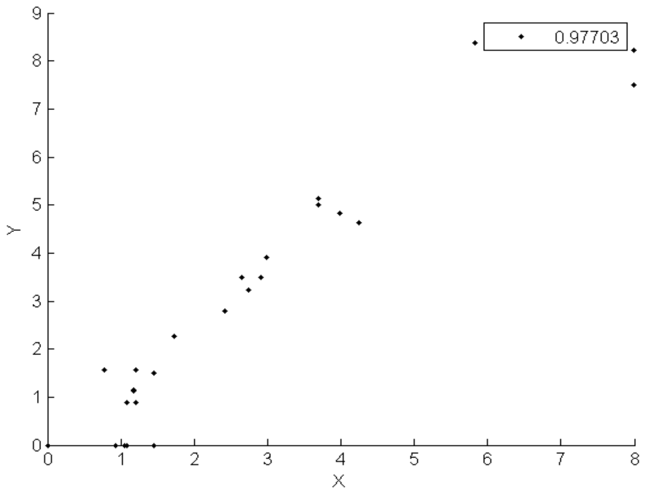

DiSEL	DR	IR	ER
PPARD+14 (SSL: RGGTCR = X)	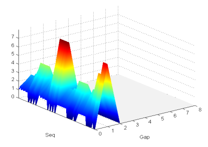	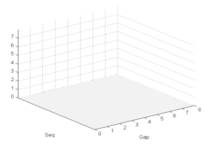	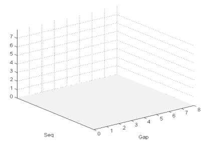
PPARG+14 (SSL: RGGTCR = Y)	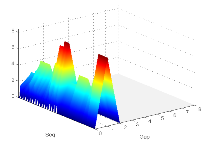	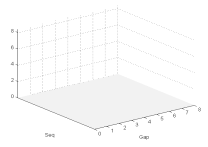	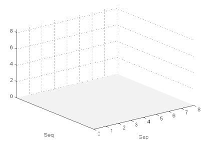
DiSEL: RGGTCR = X over Y	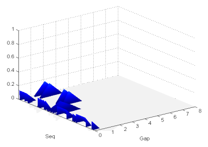	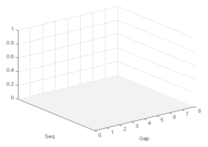	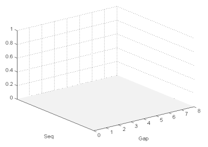
DiSEL: RGGTCR = Y over X	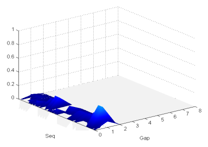	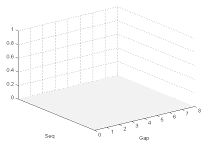	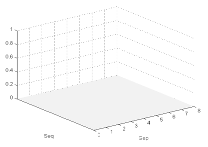

9 RORC FAMILY

9.1 Rev-ErbA-Alpha Vs RORC

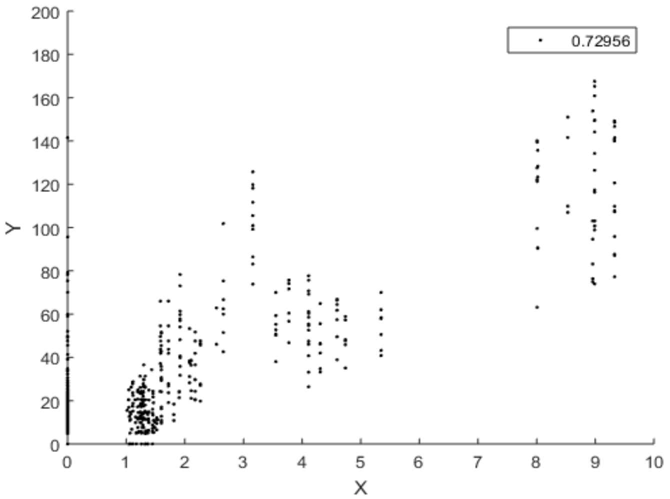

DiSEL	DR	IR	ER
Rev-ErbA-Alpha (SSL: RGKTCR = X)	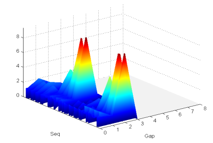	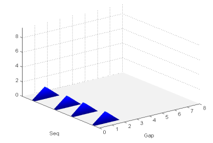	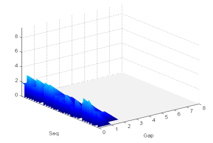
RORC (SSL: RGKTCR = Y)	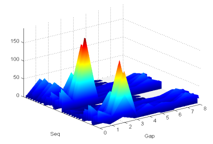	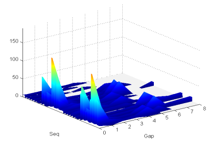	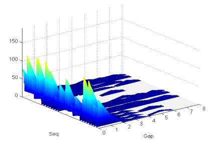
DiSEL: RGKTCR = X over Y	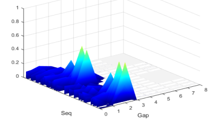	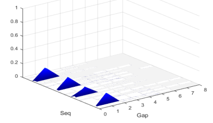	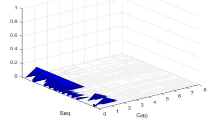
DiSEL: RGKTCR = Y over X	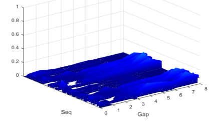	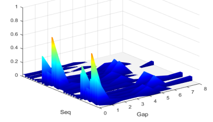	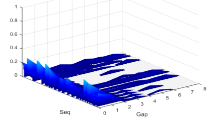

9.2 RORC Vs RORC:RXRA

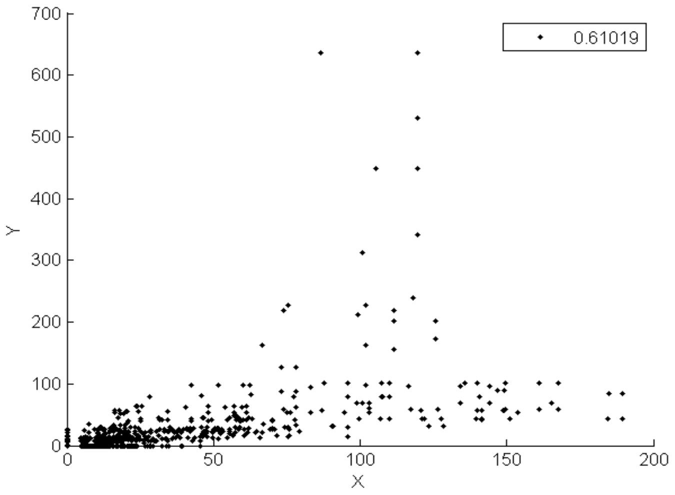

DiSEL	DR	IR	ER
RORC (SSL: RGKTCR = X)	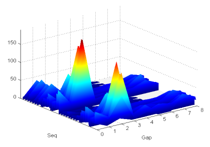	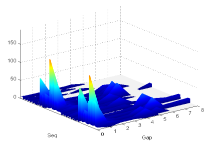	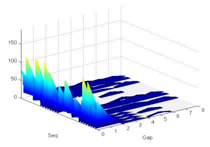
RORC:RXRA (SSL: RGKTCR = Y)	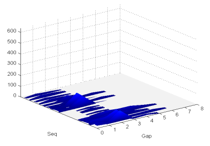	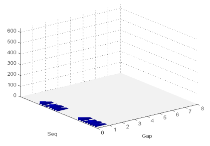	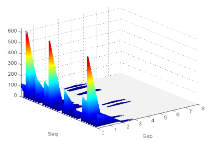
DiSEL: RGKTCR = X over Y	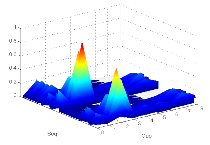	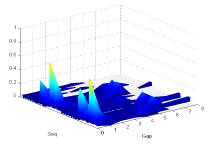	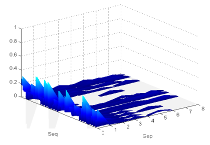
DiSEL: RGKTCR = Y over X	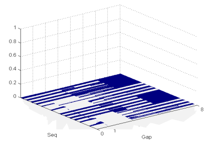	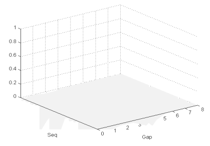	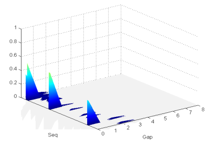

9.3 RORC Vs RORC+16

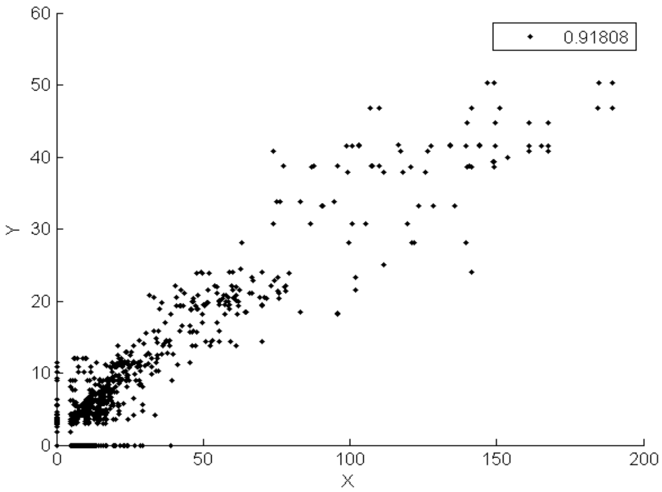

DiSEL	DR	IR	ER
RORC (SSL: RGKTCR = X)	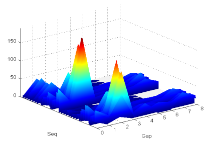	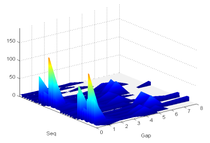	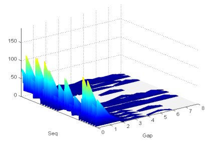
RORC+16 (SSL: RGKTCR = Y)	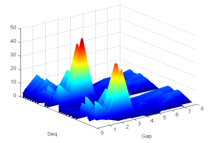	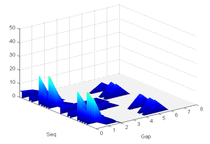	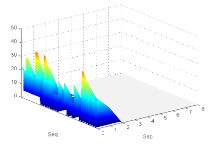
DiSEL: RGKTCR = X over Y	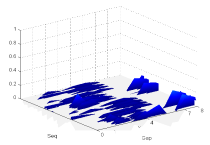	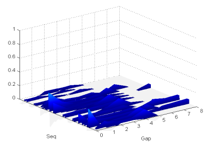	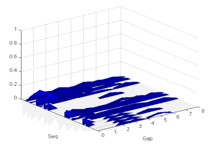
DiSEL: RGKTCR = Y over X	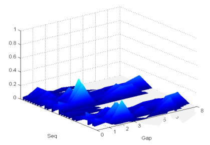	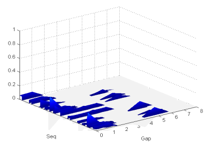	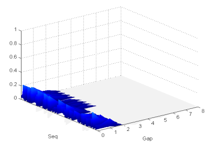

10 TR2/TR4 FAMILY

10.1 TR2 Vs TR4

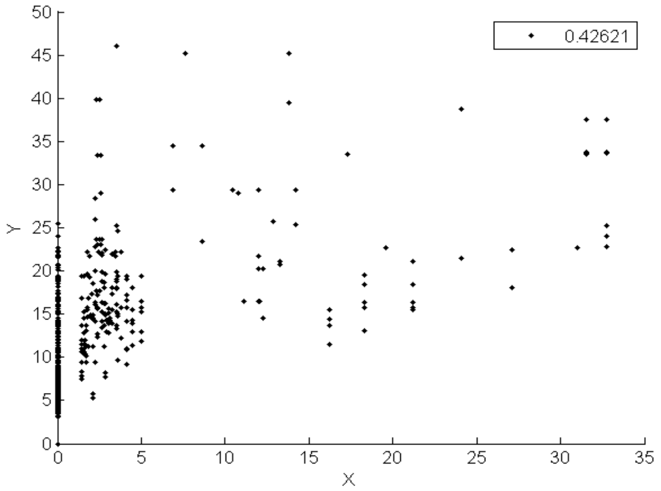

DiSEL	DR	IR	ER
TR2 (SSL: RGKTCR = X)	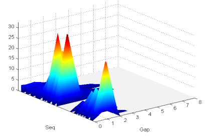	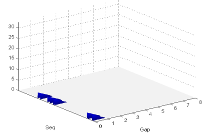	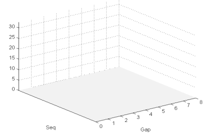
TR4 (SSL: RGKTCR = Y)	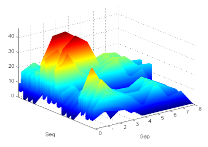	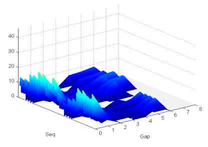	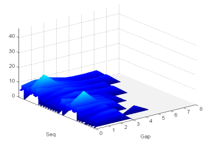
DiSEL: RGKTCR = X over Y	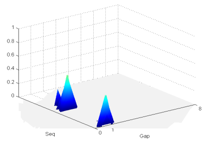	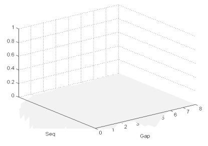	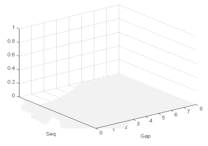
DiSEL: RGKTCR = Y over X	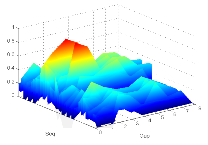	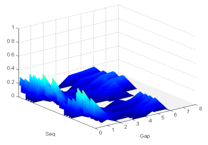	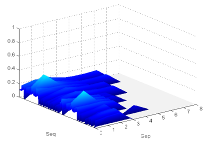

11 SF1/LRH1 FAMILY

11.1 LRH1 Vs LRH1:RXRA

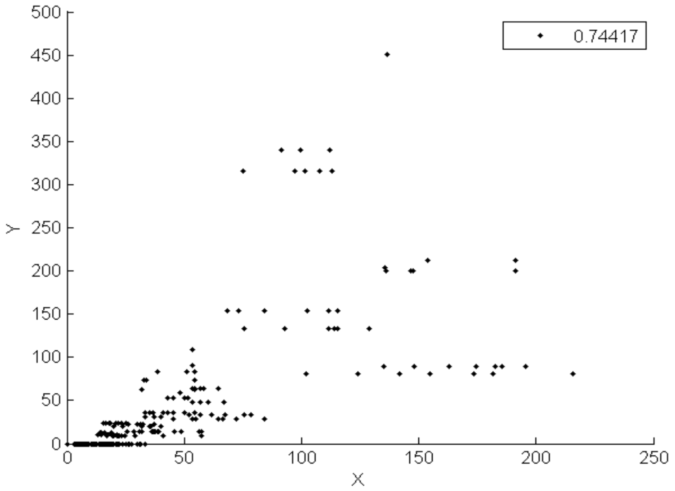

DiSEL	DR	IR	ER
LRH1 (SSL: RGKTCR = X)	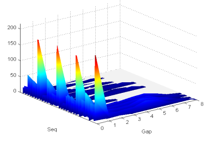	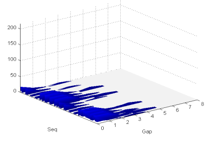	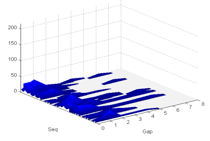
LRH1:RXRA (SSL: RGKTCR = Y)	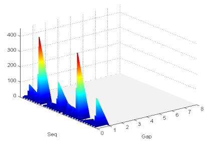	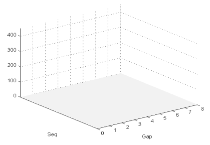	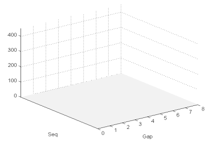
DiSEL: RGKTCR = X over Y	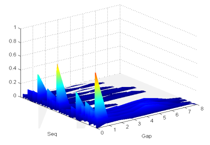	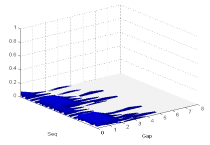	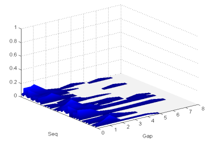
DiSEL: RGKTCR = Y over X	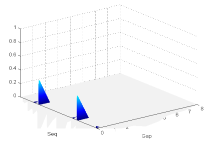	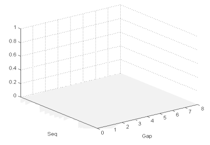	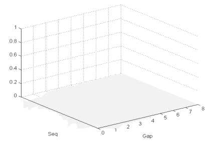

11.2 SF1 Vs SF1:RXRA

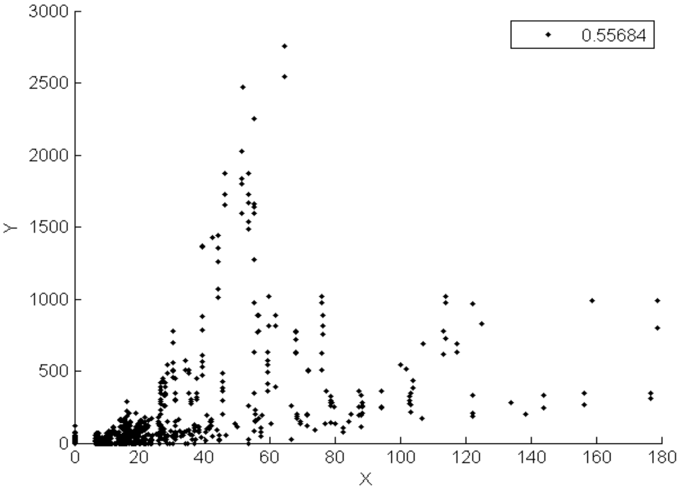

DiSEL	DR	IR	ER
SF1 (SSL: RGKTCR = X)	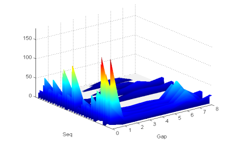	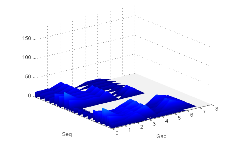	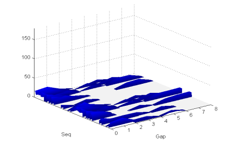
SF1:RXRA (SSL: RGKTCR = Y)	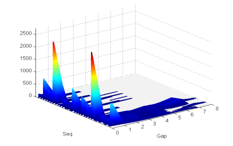	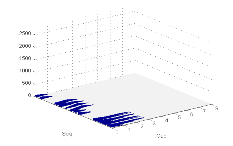	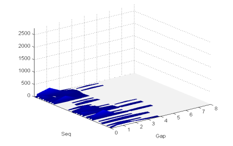
DiSEL: RGKTCR = X over Y	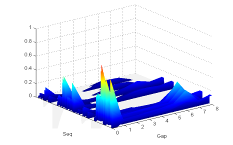	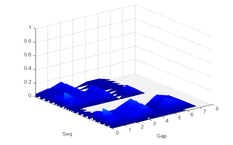	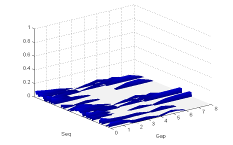
DiSEL: RGKTCR = Y over X	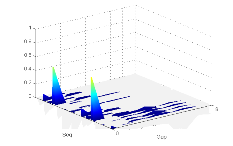	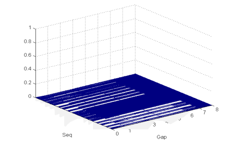	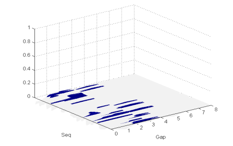

11.3 LRH1 Vs SF1

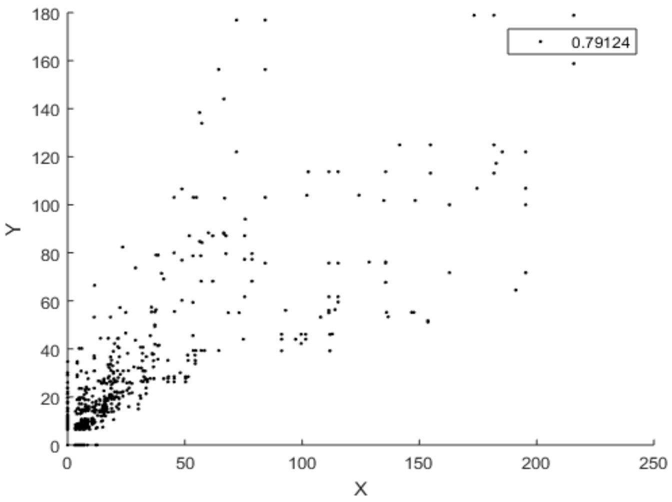

DiSEL	DR	IR	ER
LRH1 (SSL: RGK-TCR = X)	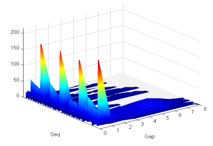	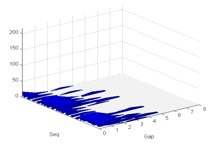	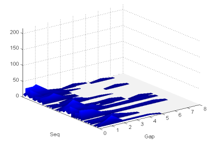
SF1 (SSL: RGKTCR = Y)	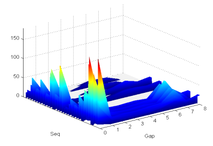	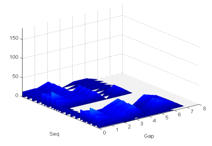	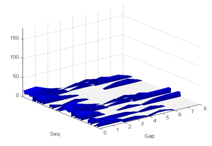
DiSEL: RGKTCR = X over Y	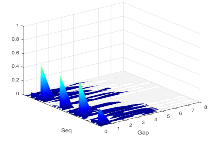	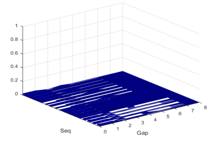	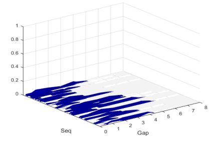
DiSEL: RGKTCR = Y over X	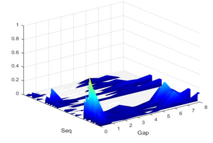	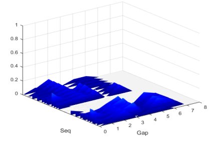	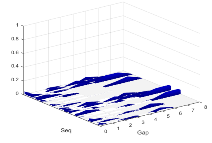

11.4 LRH1:RXRA Vs SF1:RXRA

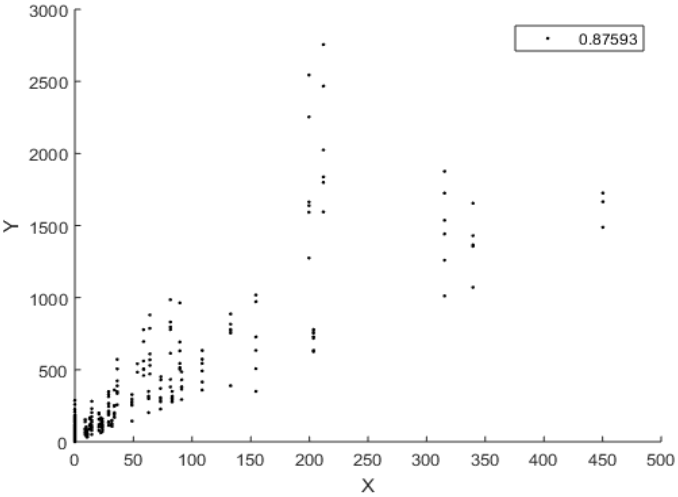

DiSEL	DR	IR	ER
LRH1:RXRA (SSL: RGKTCR = X)	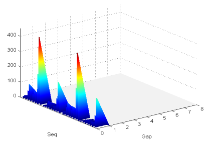	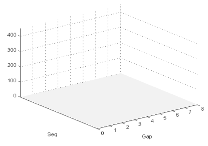	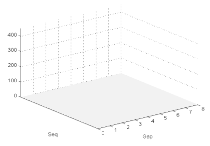
SF1:RXRA (SSL: RGKTCR = Y)	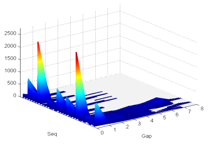	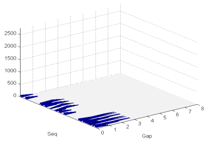	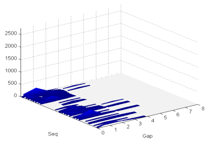
DiSEL: RGKTCR = X over Y	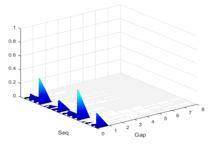	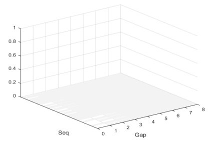	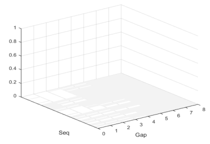
DiSEL: RGKTCR = Y over X	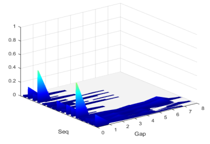	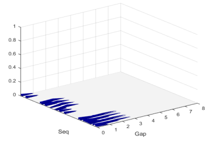	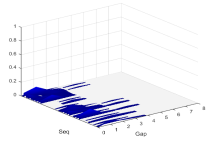

12 TLX/PNR FAMILY

12.1 TLX Vs TLX:RXRA

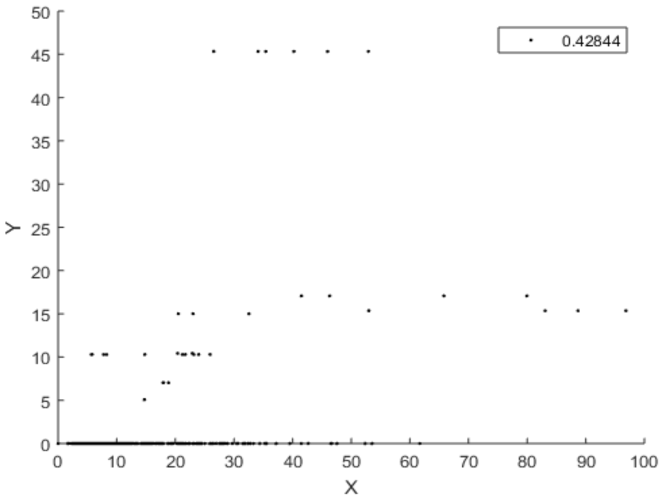

DiSEL	DR	IR	ER
TLX (SSL: RRGTCR = X)	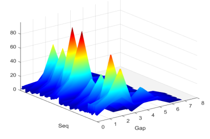	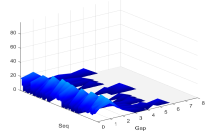	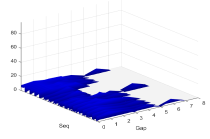
TLX:RXRA (SSL: RRGTCR = Y)	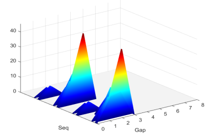	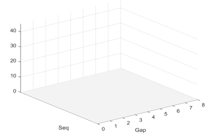	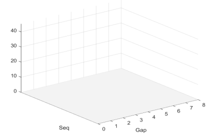
DiSEL: RRGTCR = X over Y	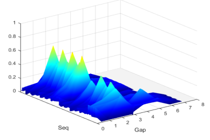	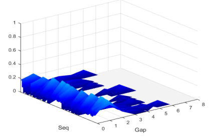	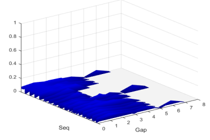
DiSEL: RRGTCR = Y over X	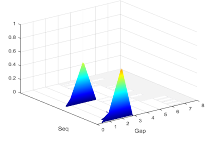	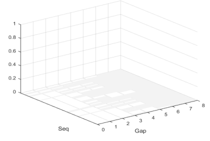	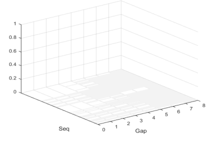

12.2 TLX Vs PNR

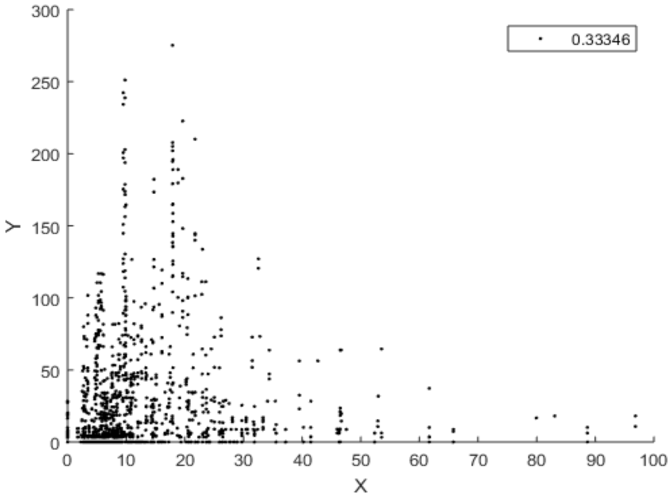

DiSEL	DR	IR	ER
TLX (SSL: RRGTCR = X)	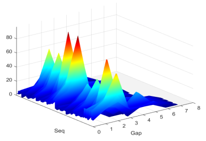	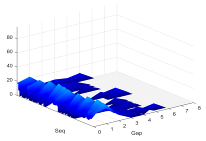	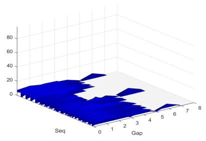
PNR (SSL: RRGTCR = Y)	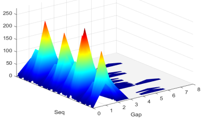	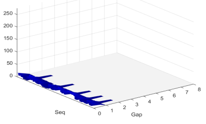	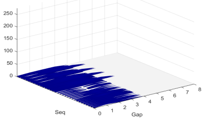
DiSEL: RRGTCR = X over Y	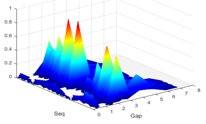	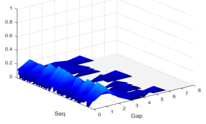	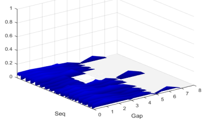
DiSEL: RRGTCR = Y over X	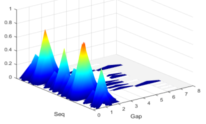	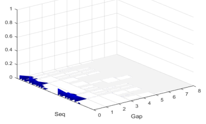	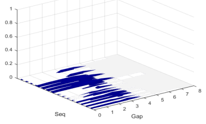

13 COUP/EAR FAMILY

13.1 COUP-TF1 Vs COUP-TF2

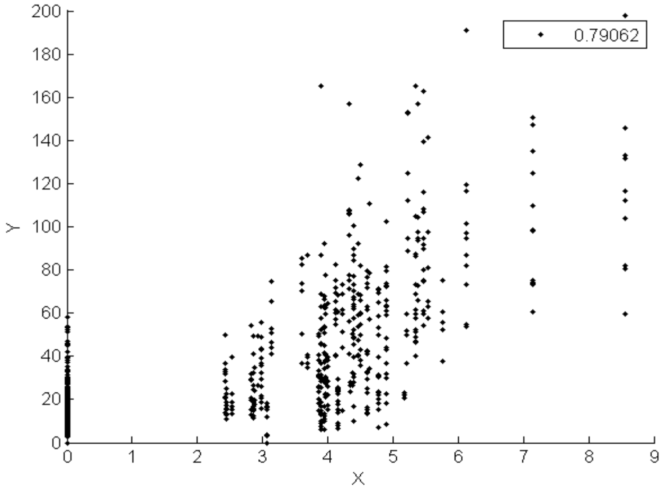

DiSEL	DR	IR	ER
COUP-TF1 (SSL: RGKTCR = X)	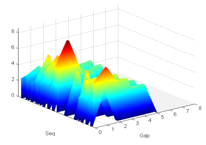	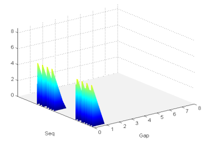	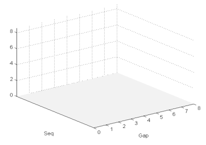
COUP-TF2 (SSL: RGKTCR = Y)	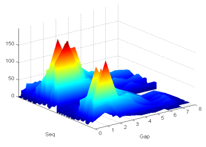	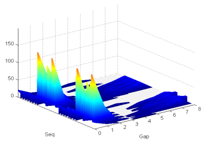	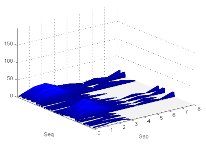
DiSEL: RGKTCR = X over Y	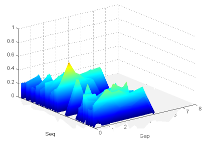	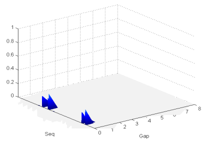	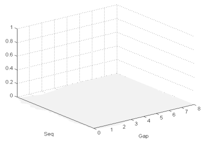
DiSEL: RGKTCR = Y over X	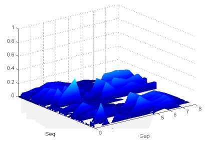	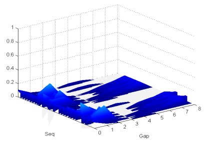	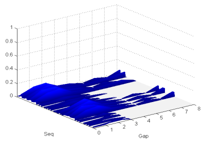

13.2 COUP-TF2 Vs EAR2

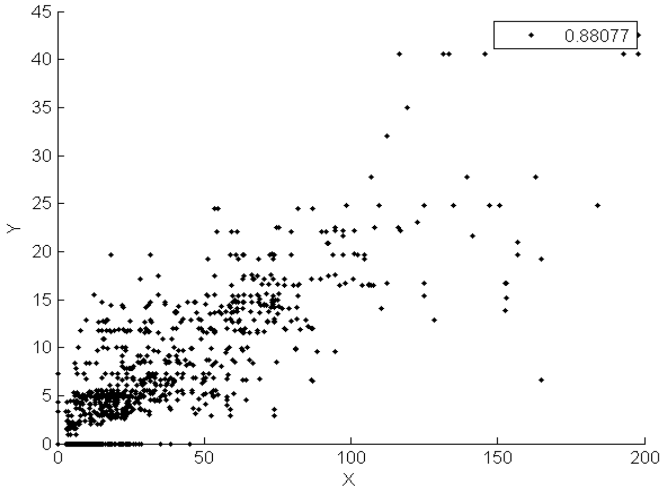

DiSEL	DR	IR	ER
COUP-TF2 (SSL: RGKTCR = X)	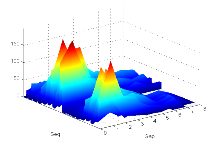	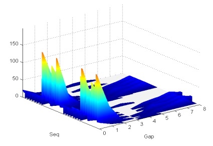	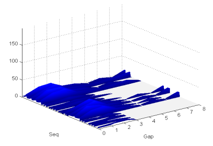
EAR2 (SSL: RGK-TCR = Y)	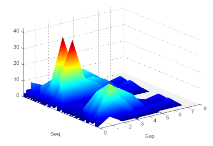	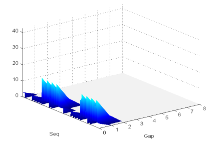	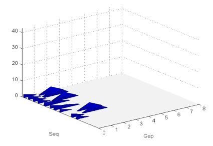
DiSEL: RGKTCR = X over Y	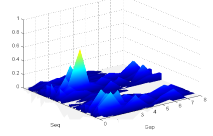	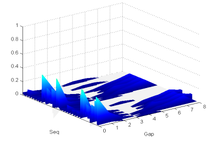	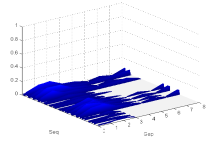
DiSEL: RGKTCR = Y over X	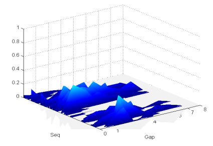	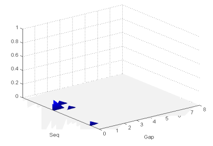	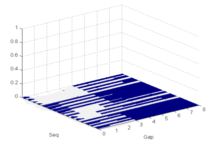

13.3 COUP-TF1 Vs EAR2

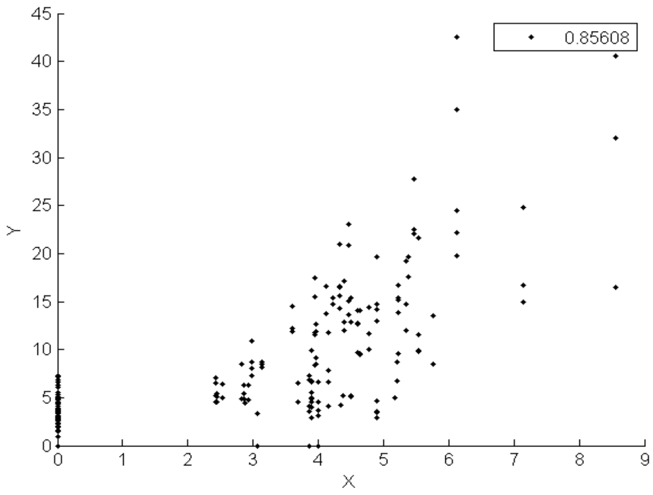

DiSEL	DR	IR	ER
COUP-TF1 (SSL: RGKTCR = X)	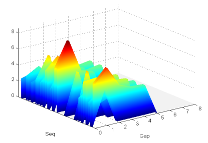	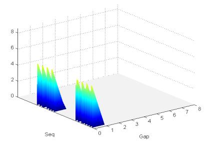	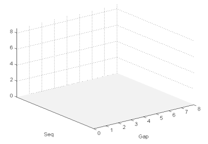
EAR2 (SSL: RGK-TCR = Y)	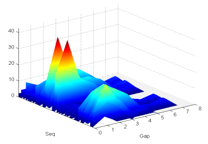	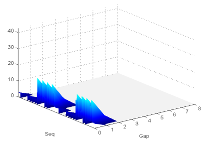	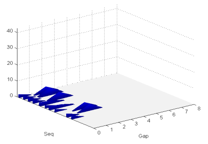
DiSEL: RGKTCR = X over Y	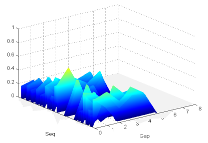	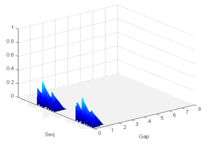	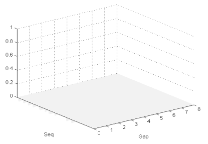
DiSEL: RGKTCR = Y over X	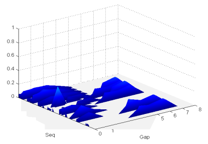	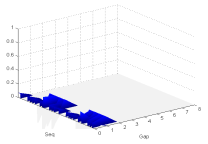	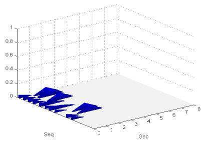

13.4 COUP-TF2 Vs COUP-TF2+17

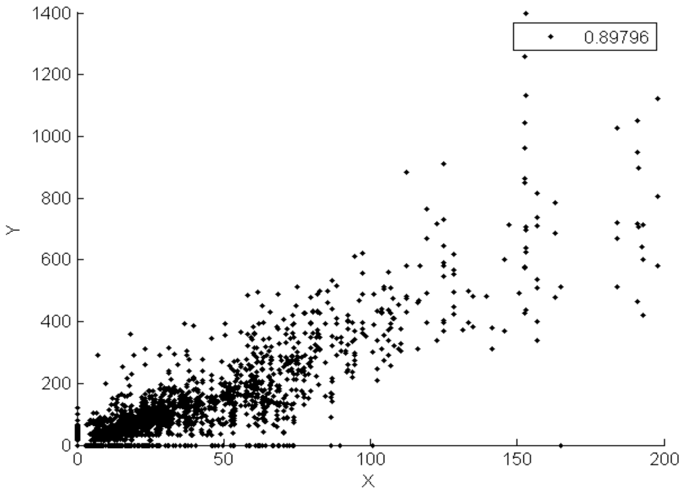

DiSEL	DR	IR	ER
COUP-TF2 (SSL: RGKTCR = X)	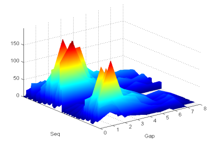	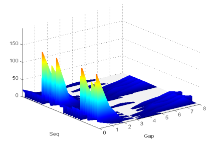	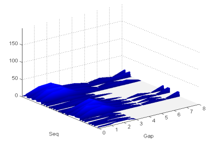
COUP-TF2+17 (SSL: RGKTCR = Y)	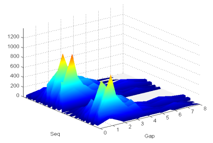	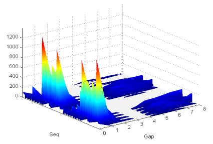	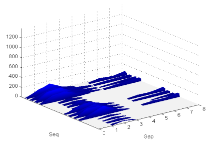
DiSEL: RGKTCR = X over Y	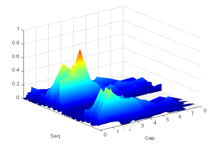	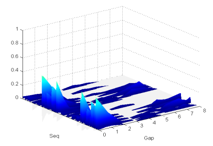	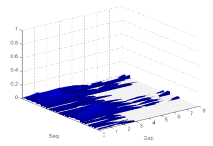
DiSEL: RGKTCR = Y over X	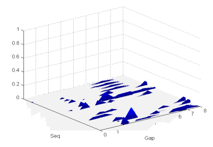	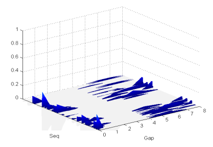	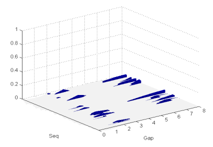

13.5 COUP-TF2 Vs COUP-TF2:RXRA

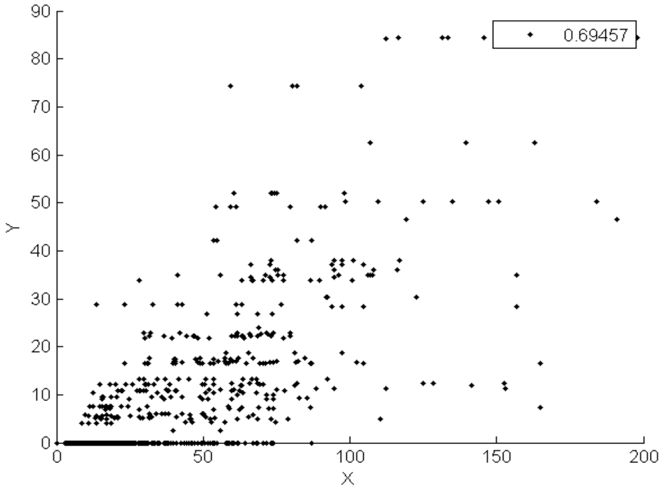

DiSEL	DR	IR	ER
COUP-TF2 (SSL: RGKTCR = X)	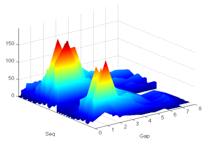	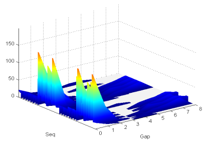	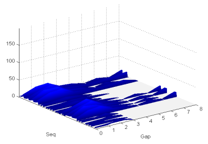
COUP-TF2:RXRA (SSL: RGKTCR = Y)	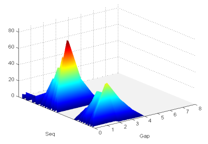	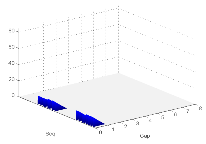	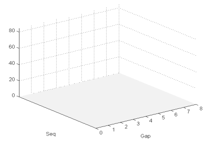
DiSEL: RGKTCR = X over Y	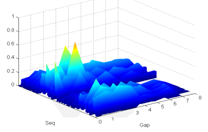	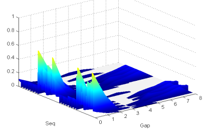	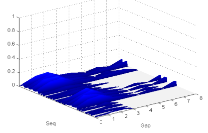
DiSEL: RGKTCR = Y over X	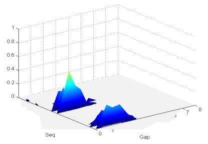	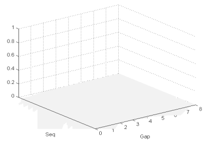	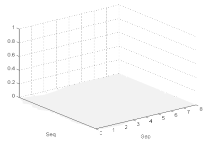

13.6 COUP-TF1 Vs COUP-TF1+17

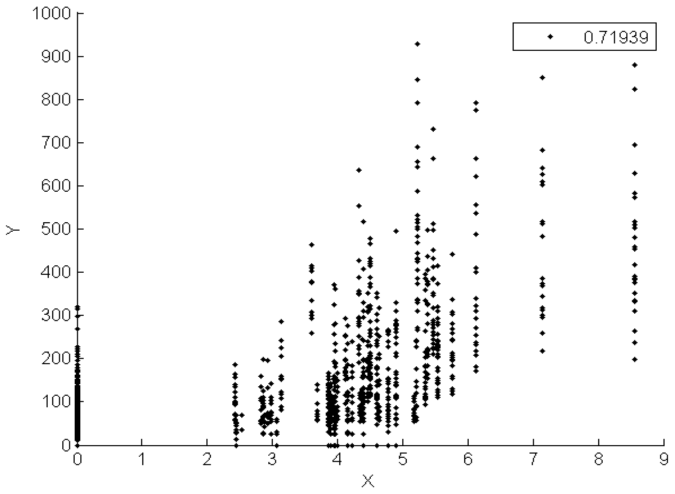

DiSEL	DR	IR	ER
COUP-TF1 (SSL: RGKTCR = X)	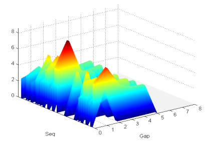	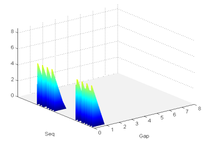	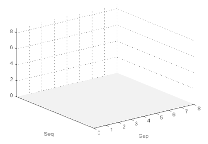
COUP-TF1+17 (SSL: RGKTCR = Y)	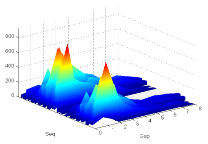	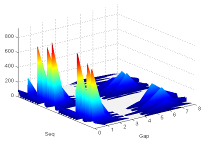	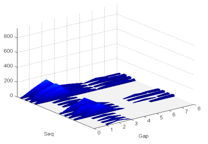
DiSEL: RGKTCR = X over Y	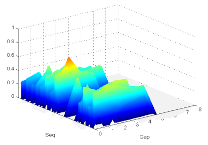	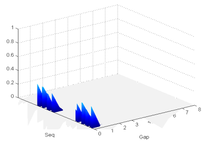	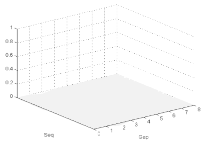
DiSEL: RGKTCR = Y over X	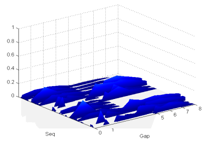	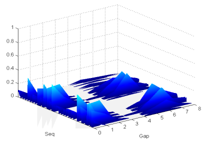	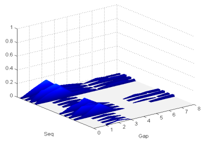

13.7 COUP-TF1 Vs COUP-TF1:RXRA

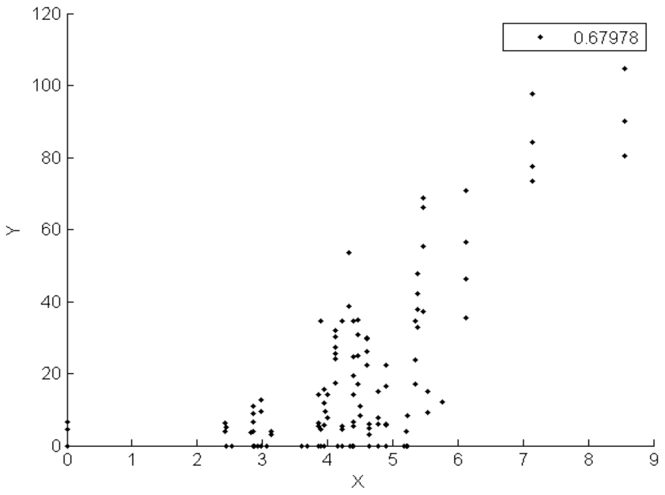

DiSEL	DR	IR	ER
COUP-TF1 (SSL: RGKTCR = X)	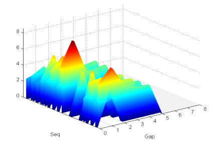	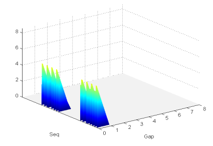	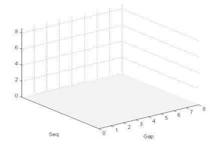
COUP-TF1:RXRA (SSL: RGKTCR = Y)	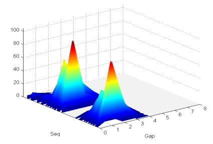	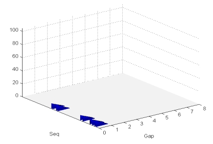	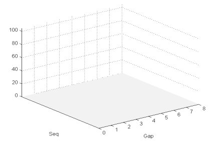
DiSEL: RGKTCR = X over Y	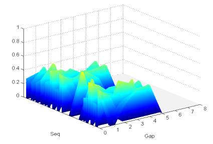	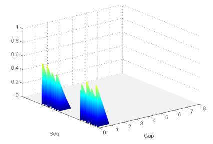	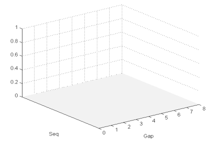
DiSEL: RGKTCR = Y over X	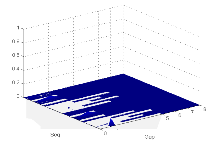	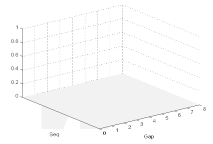	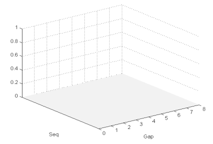

13.8 EAR2 Vs EAR2:RXRA

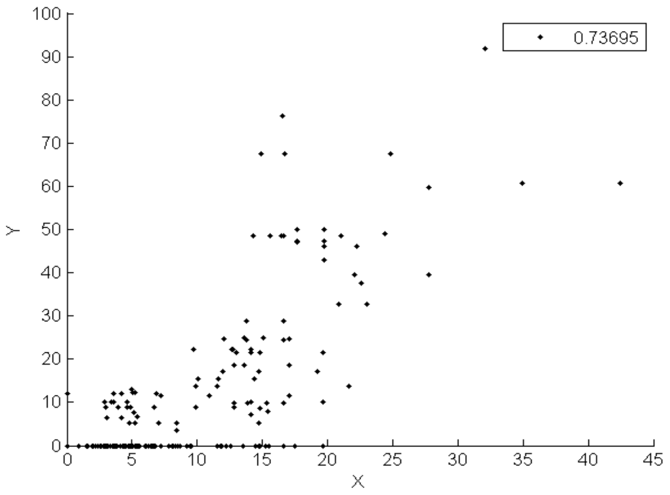

DiSEL	DR	IR	ER
EAR2 (SSL: RGKTCR = X)	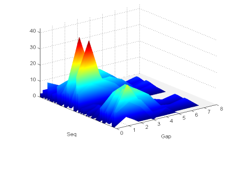	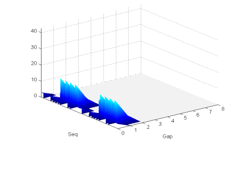	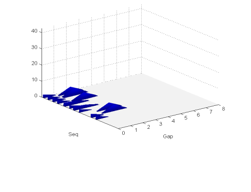
EAR2:RXRA (SSL: RGKTCR = Y)	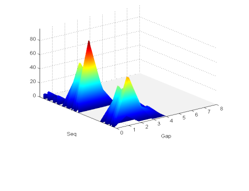	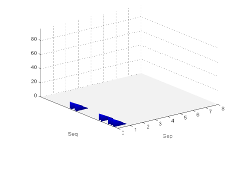	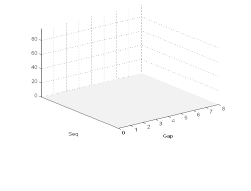
DiSEL: RGKTCR = X over Y	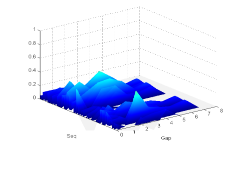	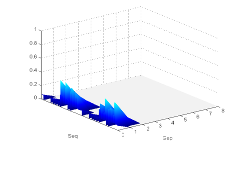	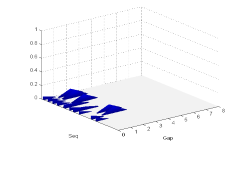
DiSEL: RGKTCR = Y over X	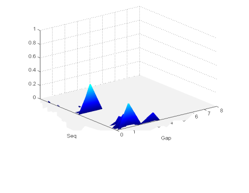	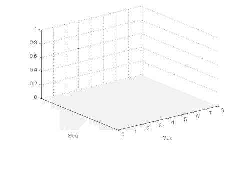	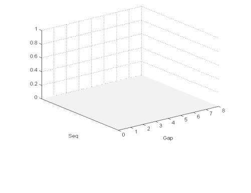

13.9 COUP-TF1+17 Vs COUP-TF2+17

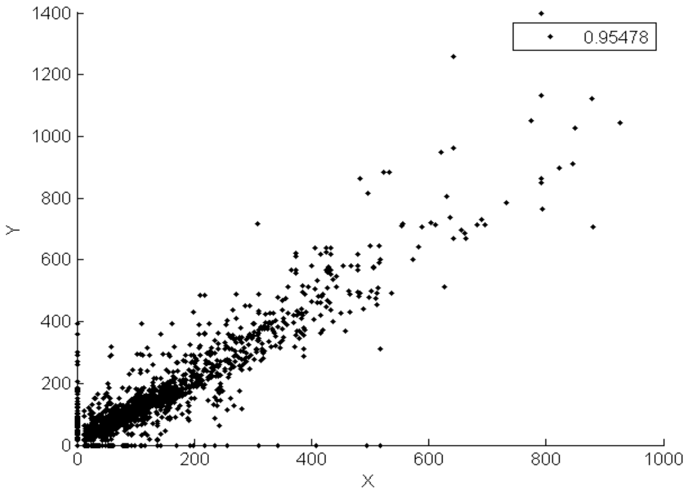

DiSEL	DR	IR	ER
COUP-TF1+17 (SSL: RGKTCR = X)	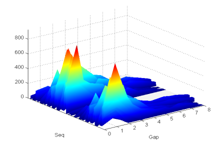	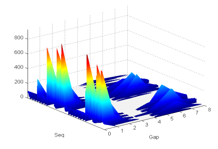	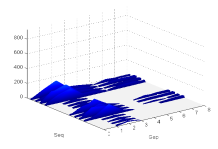
COUP-TF2+17 (SSL: RGKTCR = Y)	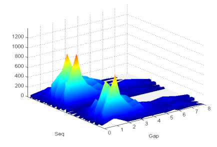	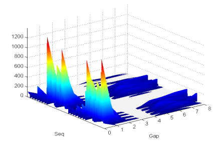	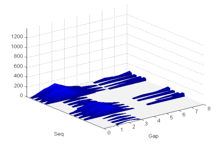
DiSEL: RGKTCR = X over Y	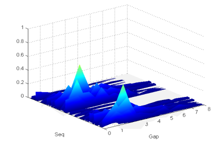	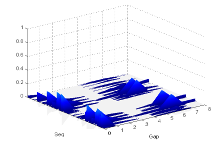	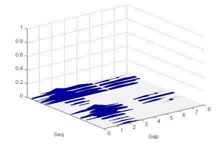
DiSEL: RGKTCR = Y over X	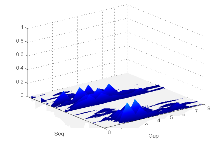	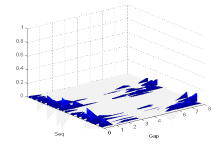	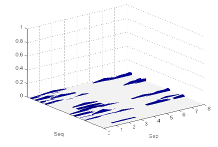

13.10 COUP-TF1:RXRA Vs COUP-TF2:RXRA

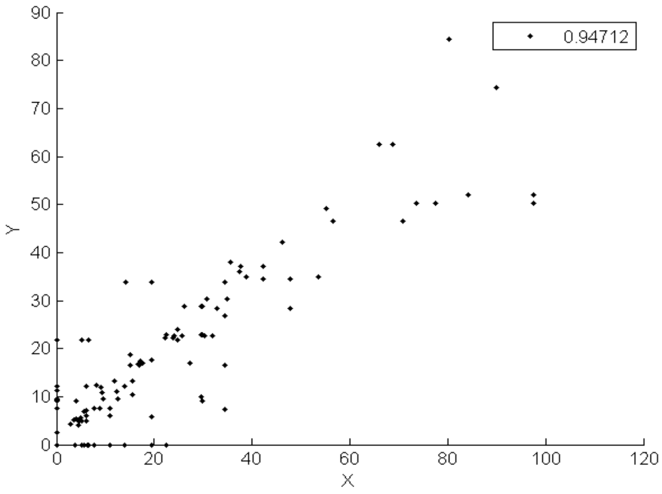

DiSEL	DR	IR	ER
COUP-TF1:RXRA (SSL: RGKTCR = X)	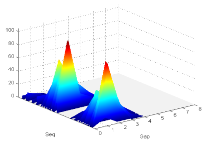	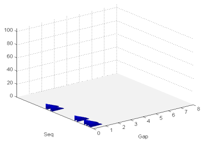	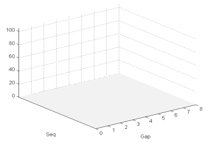
COUP-TF2:RXRA (SSL: RGKTCR = Y)	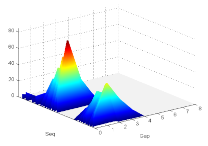	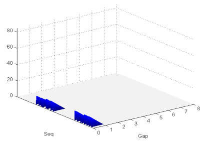	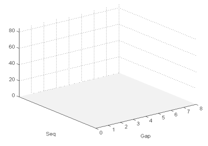
DiSEL: RGKTCR = X over Y	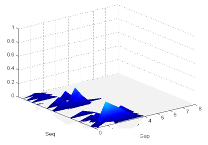	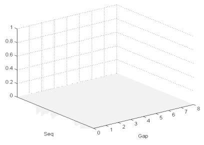	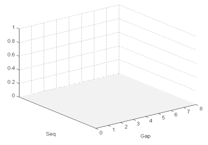
DiSEL: RGKTCR = Y over X	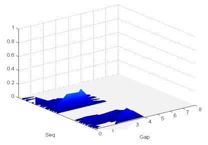	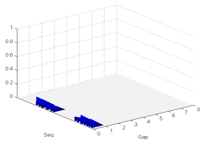	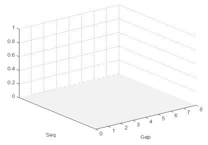

13.11 COUP-TF1:RXRA Vs EAR2:RXRA

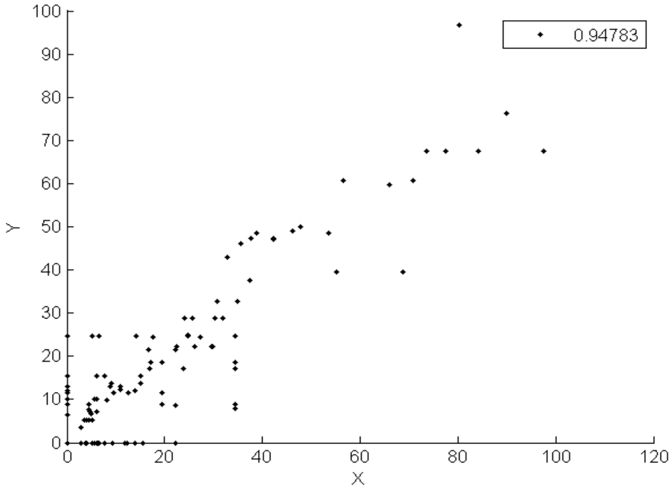

DiSEL	DR	IR	ER
COUP-TF1:RXRA (SSL: RGKTCR = X)	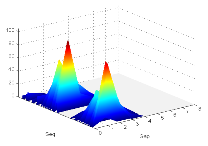	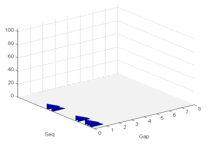	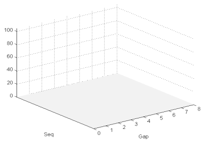
EAR2:RXRA (SSL: RGKTCR = Y)	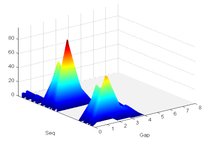	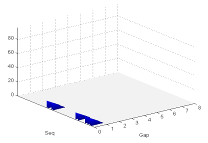	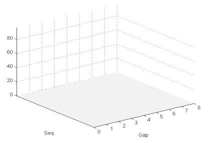
DiSEL: RGKTCR = X over Y	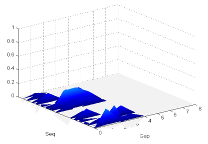	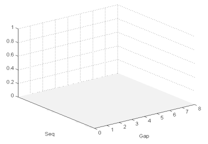	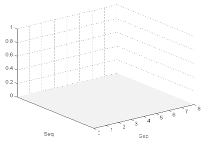
DiSEL: RGKTCR = Y over X	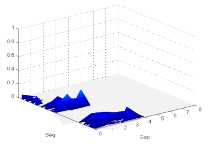	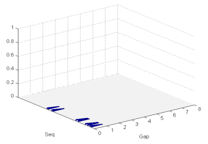	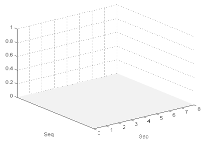

13.12 COUP-TF2:RXRA Vs EAR2:RXRA

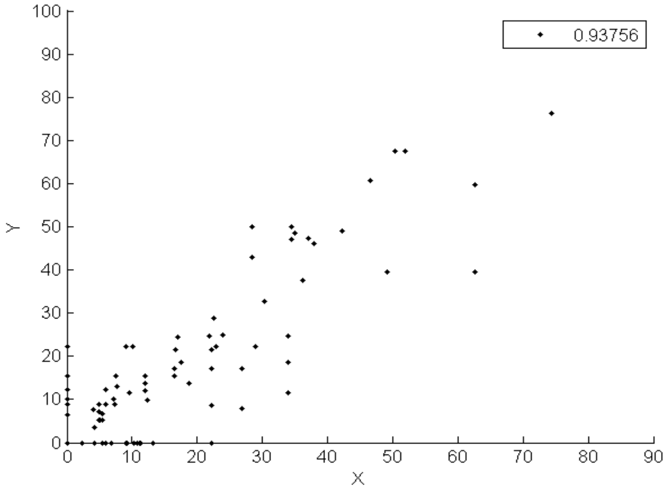

DiSEL	DR	IR	ER
COUP-TF2:RXRA (SSL: RGKTCR = X)	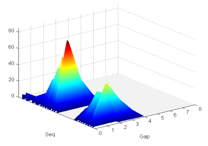	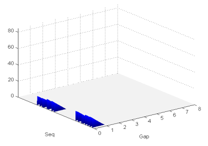	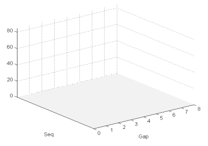
EAR2:RXRA (SSL: RGKTCR = Y)	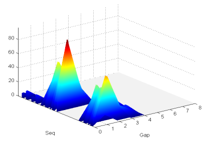	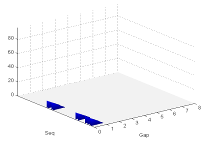	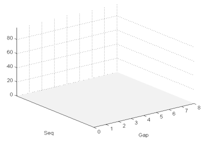
DiSEL: RGKTCR = X over Y	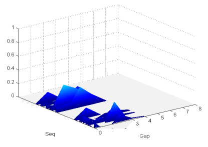	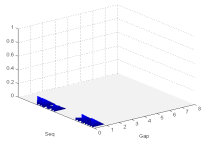	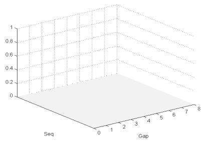
DiSEL: RGKTCR = Y over X	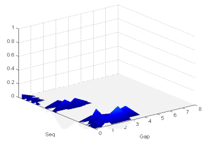	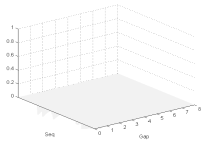	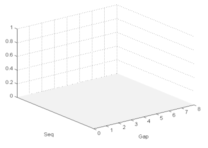

14 HNF4 FAMILY

14.1 HNF4A Vs HNF4G

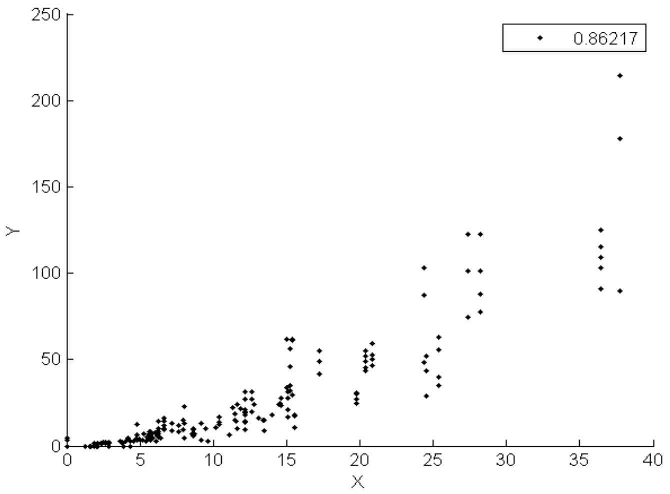

DiSEL	DR	IR	ER
HNF4A (SSL: RGKTCR = X)	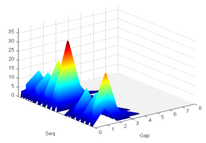	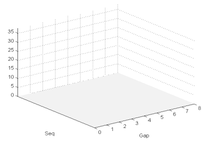	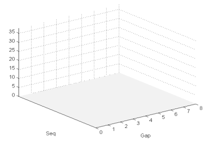
HNF4G (SSL: RGKTCR = Y)	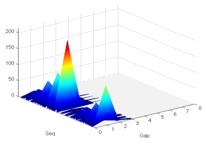	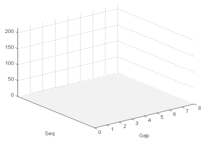	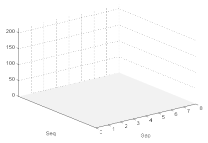
DiSEL: RGKTCR = X over Y	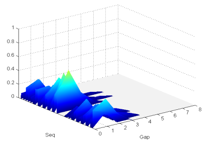	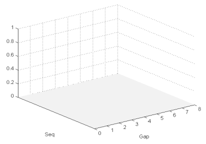	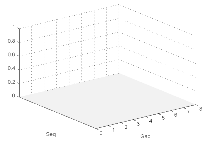
DiSEL: RGKTCR = Y over X	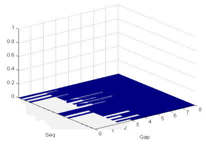	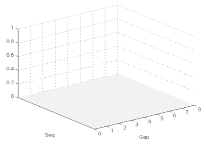	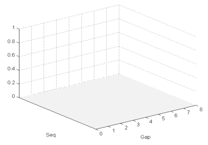

14.2 HNF4A Vs HNF4A+18

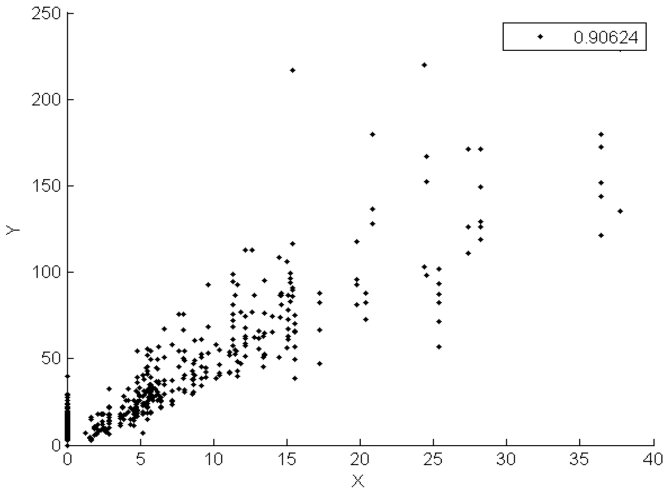

DiSEL	DR	IR	ER
HNF4A (SSL: RGK-TCR = X)	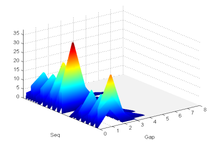	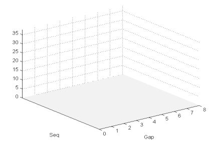	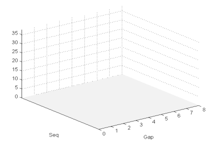
HNF4A+18 (SSL: RGKTCR = Y)	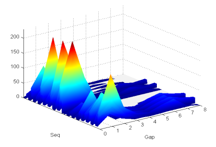	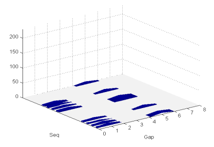	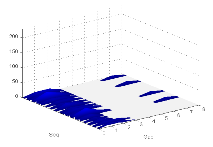
DiSEL: RGKTCR = X over Y	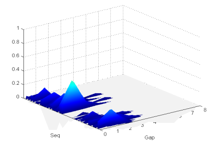	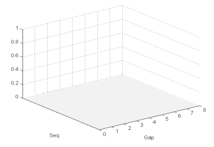	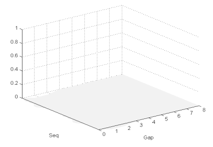
DiSEL: RGKTCR = Y over X	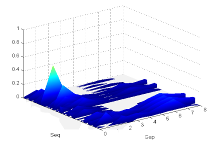	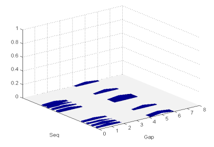	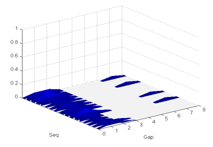

15 RXR FAMILY

15.1 RXRB Vs RXRB+17

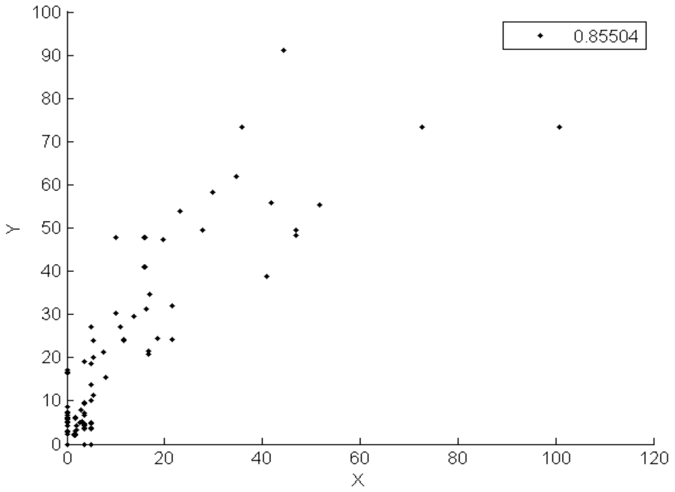

DiSEL	DR	IR	ER
RXRB (SSL: RGGTCR = X)	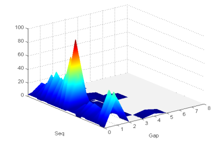	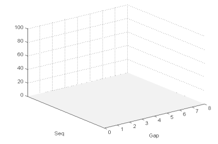	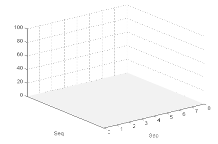
RXRB+17 (SSL: RGGTCR = Y)	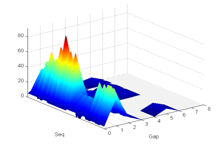	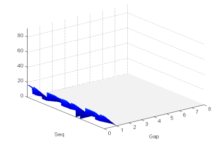	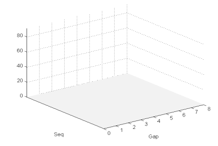
DiSEL: RGGTCR = X over Y	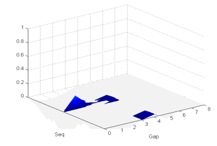	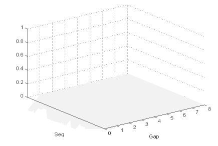	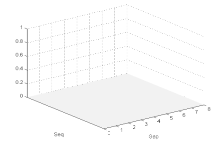
DiSEL: RGGTCR = Y over X	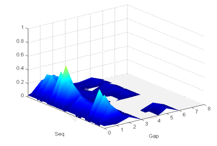	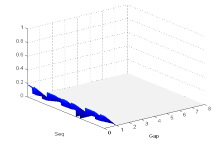	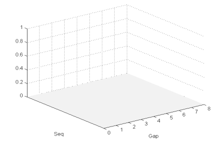

15.2 RXRG Vs RXRG:RXRA

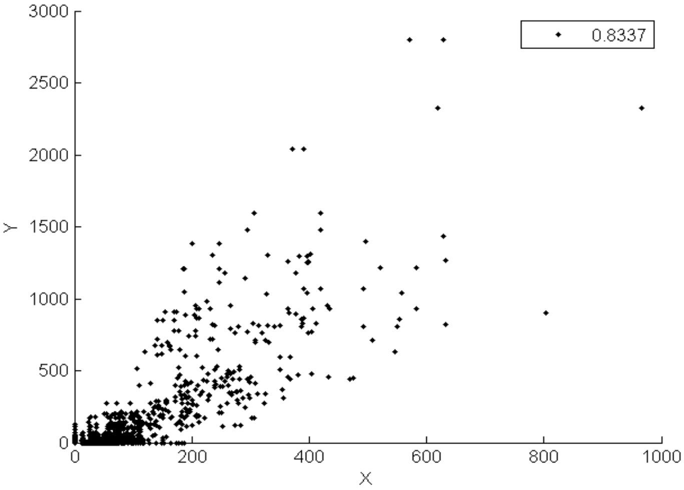

DiSEL	DR	IR	ER
RXRG (SSL: RGGTCR = X)	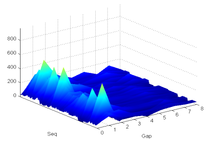	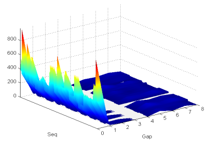	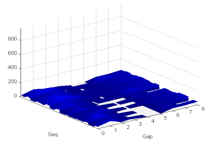
RXRG:RXRA (SSL: RGGTCR = Y)	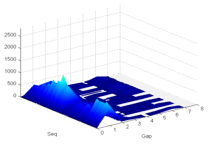	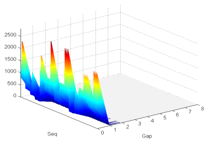	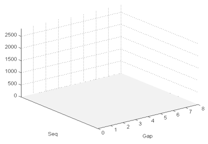
DiSEL: RGGTCR = X over Y	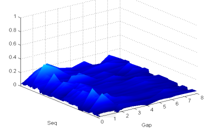	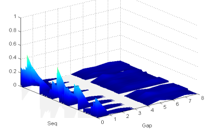	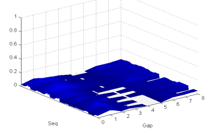
DiSEL: RGGTCR = Y over X	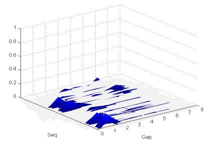	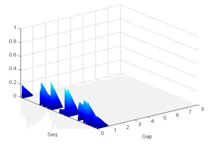	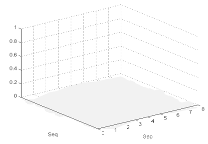

15.3 RXRG Vs RXRG+17

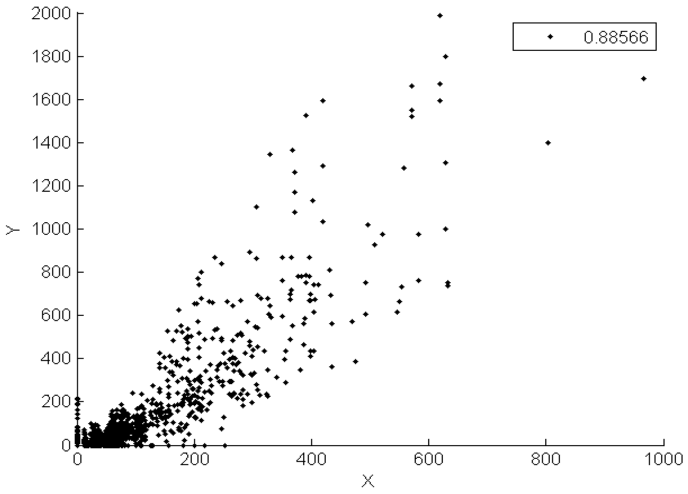

DiSEL	DR	IR	ER
RXRG (SSL: RGGTCR = X)	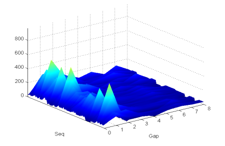	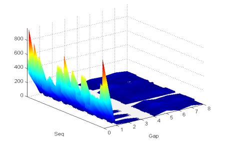	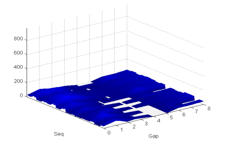
RXRG+17 (SSL: RGGTCR = Y)	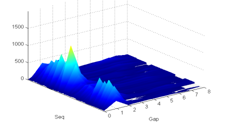	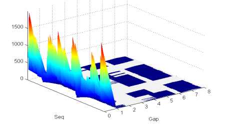	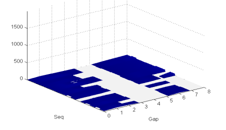
DiSEL: RGGTCR = X over Y	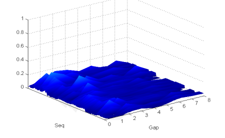	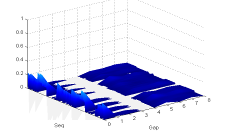	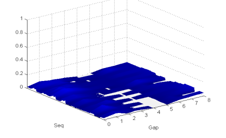
DiSEL: RGGTCR = Y over X	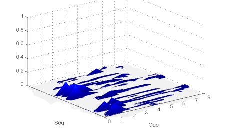	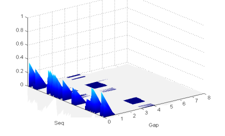	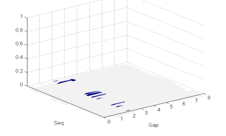

15.4 RXRG:RXRA Vs RXRG+17

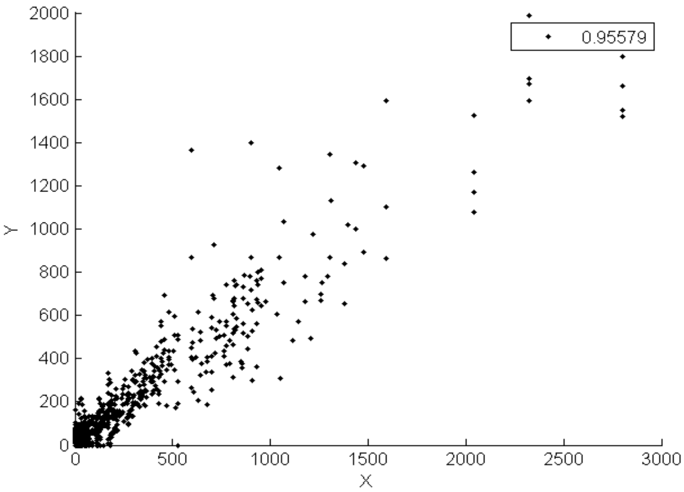

DiSEL	DR	IR	ER
RXRG:RXRA (SSL: RGGTCR = X)	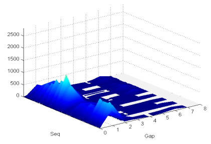	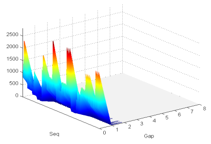	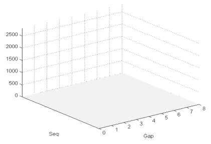
RXRG+17 (SSL: RGGTCR = Y)	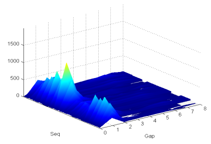	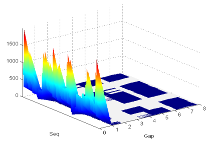	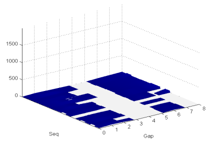
DiSEL: RGGTCR = X over Y	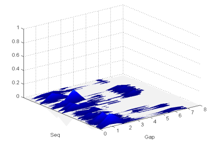	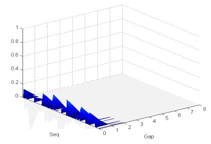	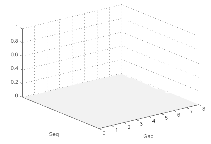
DiSEL: RGGTCR = Y over X	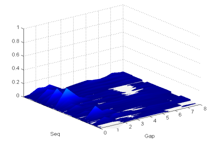	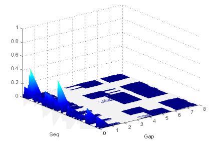	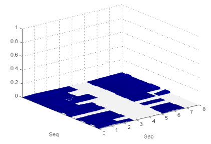

15.5 RXRA Vs RXRA+17

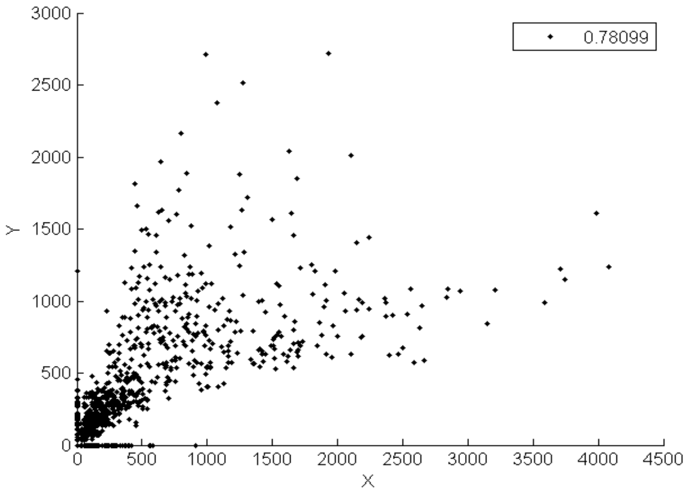

DiSEL	DR	IR	ER
RXRA (SSL: RGGTCR = X)	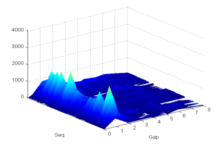	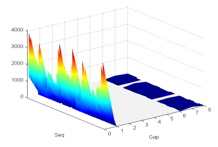	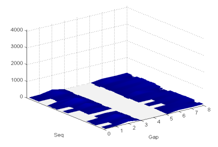
RXRA+17 (SSL: RGGTCR = Y)	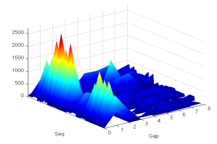	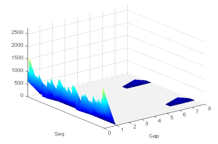	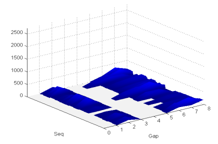
DiSEL: RGGTCR = X over Y	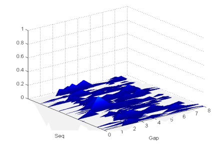	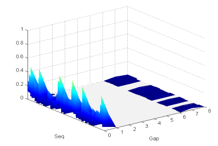	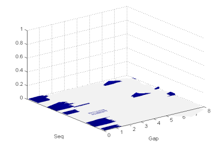
DiSEL: RGGTCR = Y over X	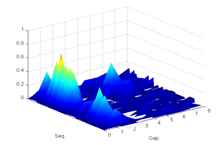	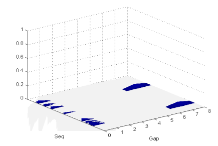	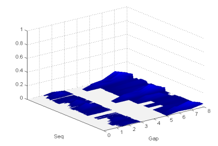

15.6 RXRB Vs RXRG

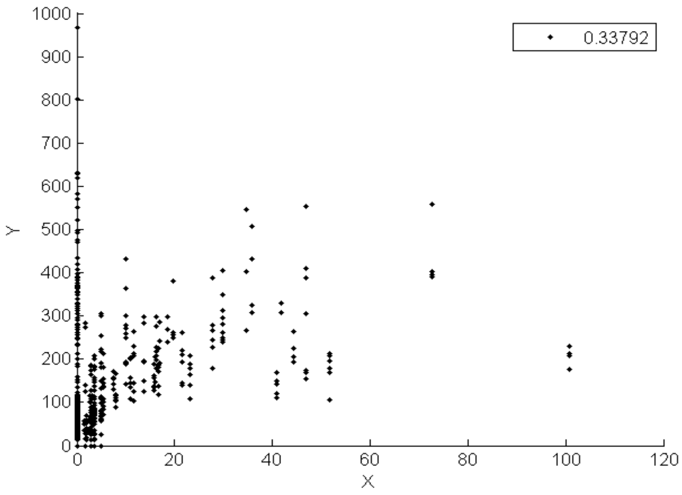

DiSEL	DR	IR	ER
RXRB (SSL: RGGTCR = X)	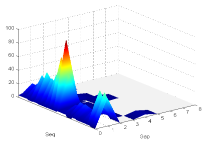	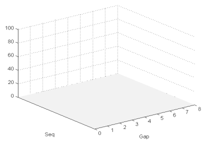	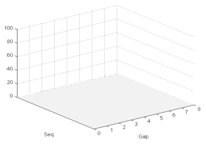
RXRG (SSL: RGGTCR = Y)	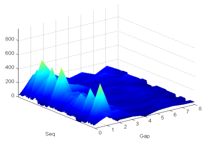	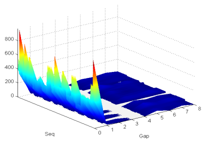	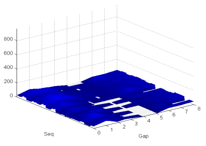
DiSEL: RGGTCR = X over Y	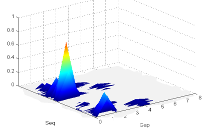	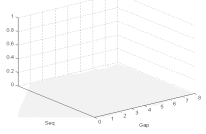	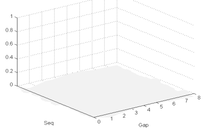
DiSEL: RGGTCR = Y over X	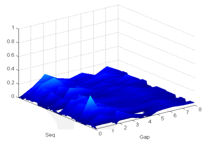	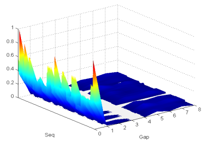	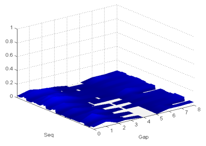

15.7 RXRA Vs RXRB

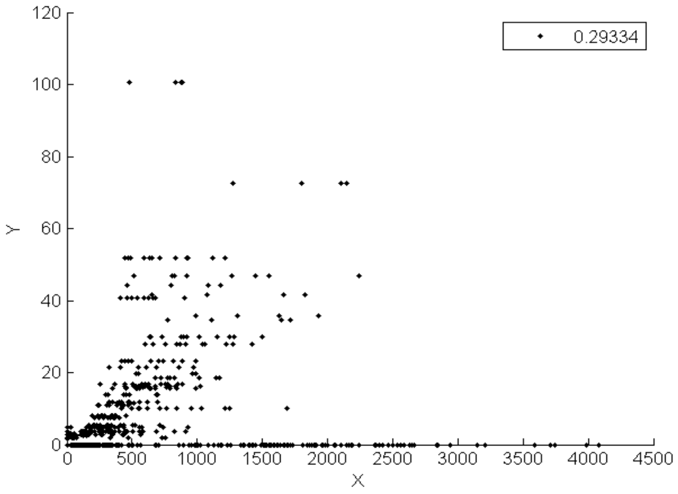

DiSEL	DR	IR	ER
RXRA (SSL: RGGTCR = X)	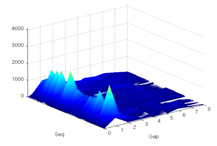	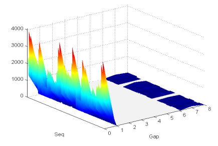	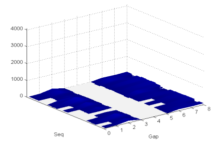
RXRB (SSL: RGGTCR = Y)	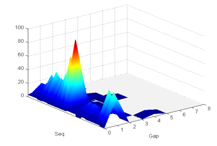	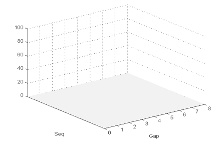	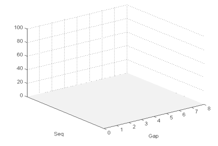
DiSEL: RGGTCR = X over Y	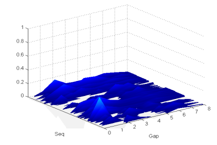	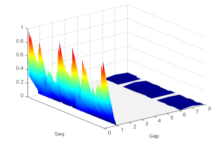	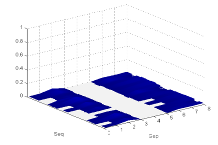
DiSEL: RGGTCR = Y over X	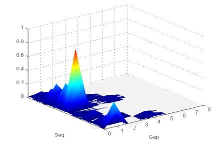	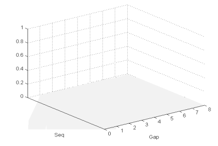	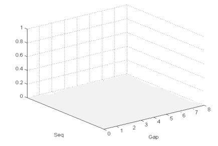

15.8 RXRA Vs RXRG

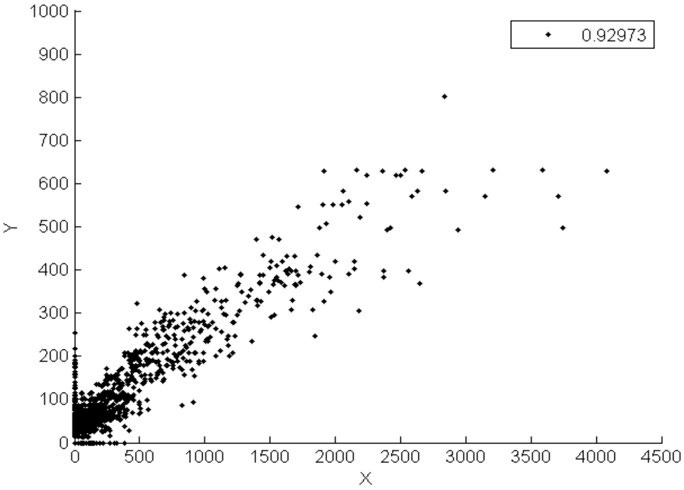

DiSEL	DR	IR	ER
RXRA (SSL: RGGTCR = X)	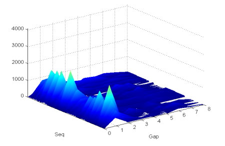	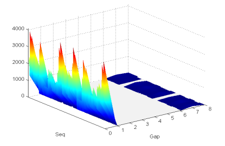	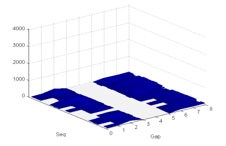
RXRG (SSL: RGGTCR = Y)	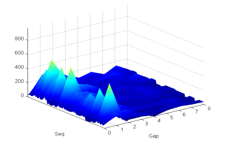	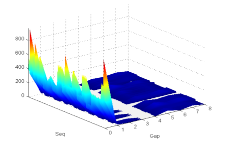	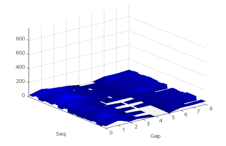
DiSEL: RGGTCR = X over Y	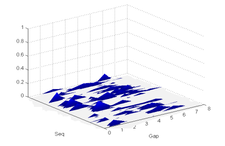	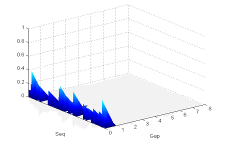	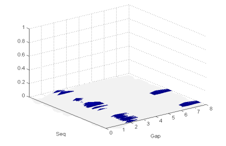
DiSEL: RGGTCR = Y over X	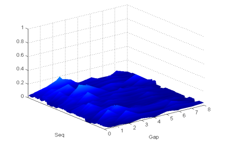	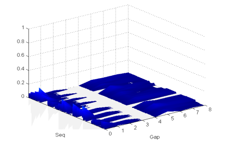	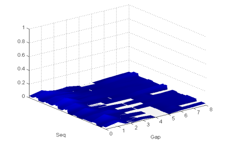

15.9 RXRB+17 Vs RXRG+17

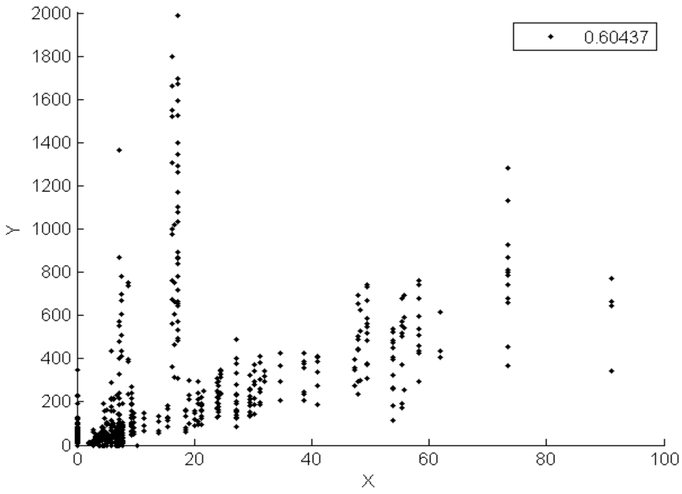

DiSEL	DR	IR	ER
RXRB+17 (SSL: RGGTCR = X)	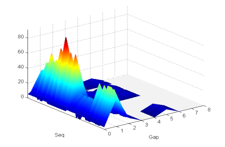	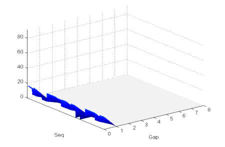	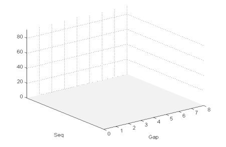
RXRG+17 (SSL: RGGTCR = Y)	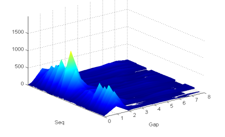	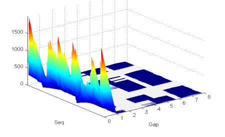	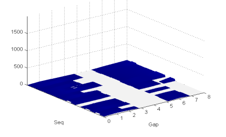
DiSEL: RGGTCR = X over Y	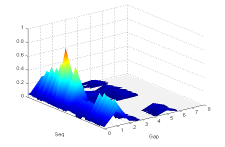	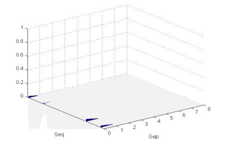	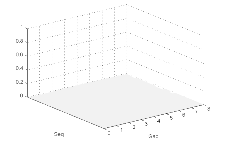
DiSEL: RGGTCR = Y over X	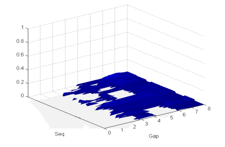	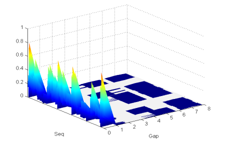	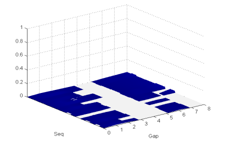

15.10 RXRA+17 Vs RXRG+17

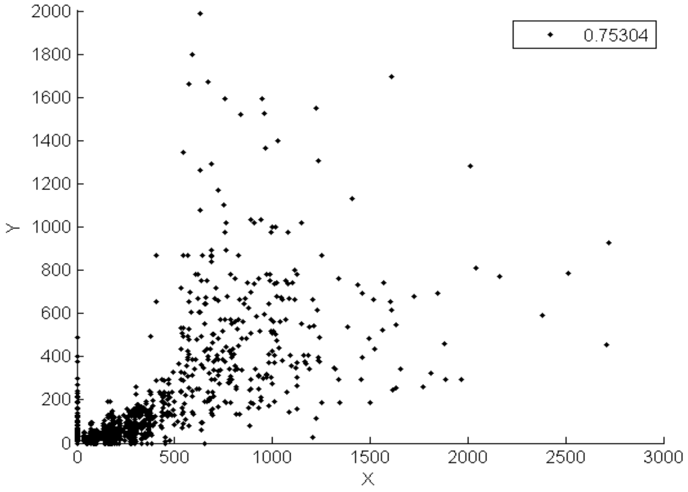

DiSEL	DR	IR	ER
RXRA+17 (SSL: RGGTCR = X)	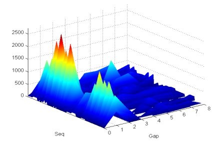	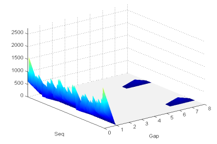	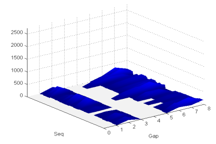
RXRG+17 (SSL: RGGTCR = Y)	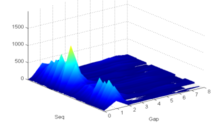	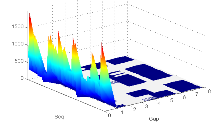	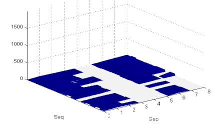
DiSEL: RGGTCR = X over Y	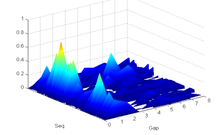	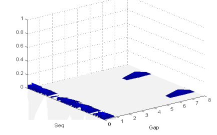	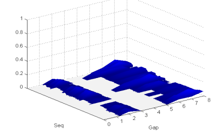
DiSEL: RGGTCR = Y over X	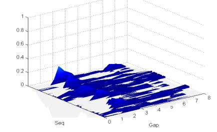	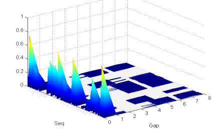	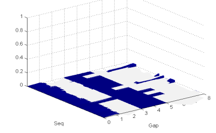

15.11 RXRA+17 Vs RXRB+17

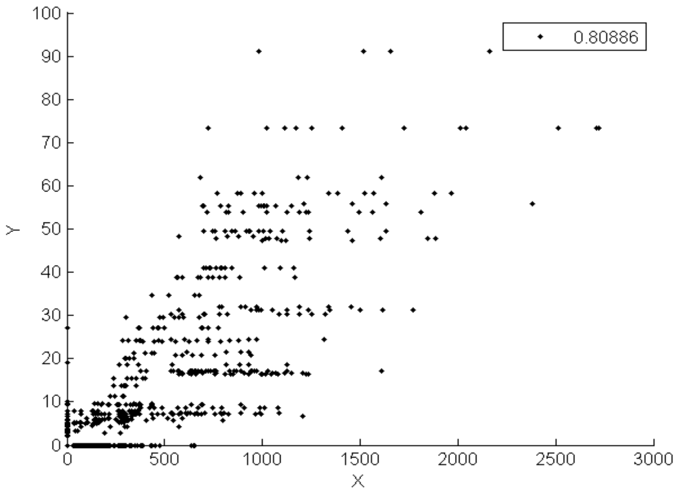

DiSEL	DR	IR	ER
RXRA+17 (SSL: RGGTCR = X)	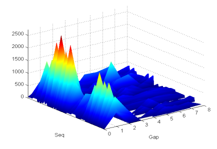	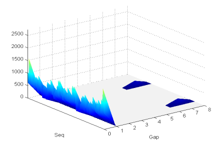	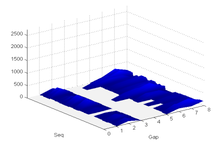
RXRB+17 (SSL: RGGTCR = Y)	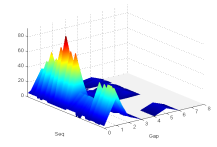	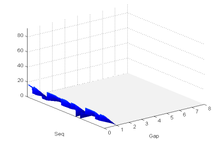	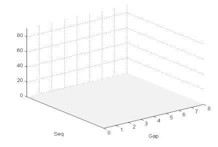
DiSEL: RGGTCR = X over Y	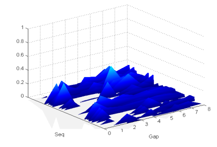	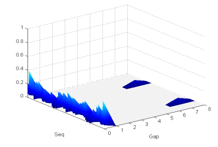	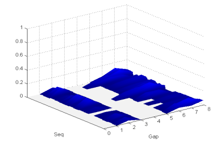
DiSEL: RGGTCR = Y over X	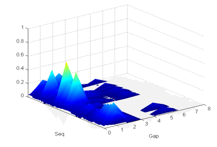	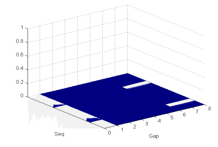	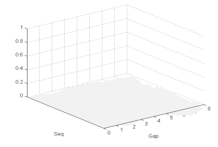

16 NURR1/NOR1 FAMILY

16.1 NOR1 Vs NURR1

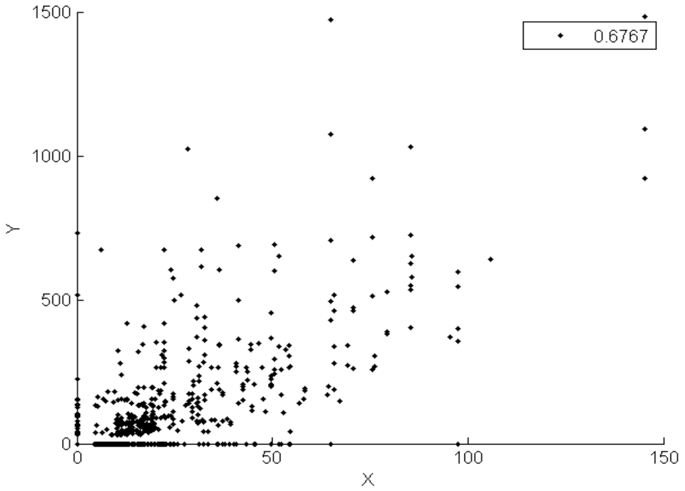

DiSEL	DR	IR	ER
NOR1 (SSL: RGK-TCR = X)	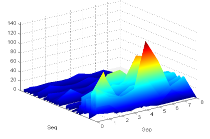	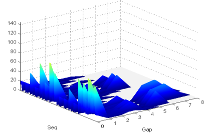	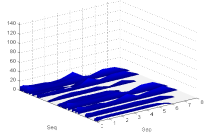
NURR1 (SSL: RGK-TCR = Y)	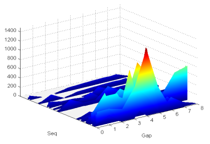	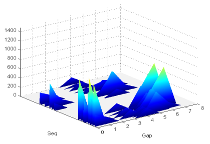	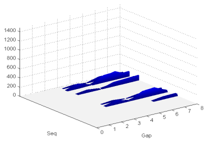
DiSEL: RGKTCR = X over Y	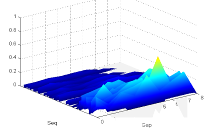	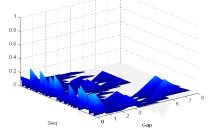	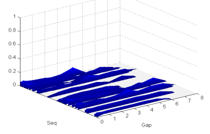
DiSEL: RGKTCR = Y over X	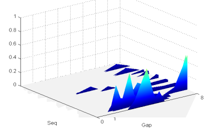	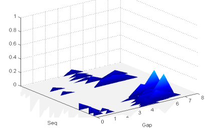	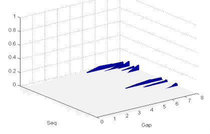
