# Supplementary material for: Hidden modes of DNA binding by human nuclear receptors
Source: Nat Commun. 2023 Jul 13;14:4179. doi: 10.1038/s41467-023-39577-0 (PMC10345098; doi:10.1038/s41467-023-39577-0)

Supplementary Data 9: Prediction of Nuclear receptor ChIP-seq peaks

Contents

1 LoVo ESRRA **3**

2 LoVo HNF4A **7**

3 LoVo NR2F1 **10**

4 LoVo NR2F2 **14**

5 LoVo NR3C1 **18**

6 LoVo RXRA **21**

7 LoVo ESR1 **24**

8 LoVo RARG **27**

9 LoVo ESR1:RXRA **31**

10 LoVo ESRRA:RXRA **34**

11 LoVo HNF4A:RXRA **38**

12 LoVo NR2F1:RXRA **41**

13 LoVo NR2F2:RXRA **45**

14 LoVo NR3C1:RXRA **49**

15 LoVo RARG:RXRA **52**

16 A549 GR treatment:Dex 500pm **56**

17 A549 GR treatment:Dex 50nm **59**

18 A549 GR treatment:Dex 5nm **62**

19 A549 GR treatment:Dex 100nm **65**

20 ECC-1 ERAA treatment=BPA 100nM **68**

21 ECC-1 ERAA treatment=Estradiol 10nM **71**

22 ECC-1 ERAA treatment=Genistein 100nM **74**

23 ECC-1 GR treatment=DEX 100nM **77**

24 GM12878 RXRA **80**

25 H1-hESC RXRA **83**

26 HepG2 HNF4A (SC-8987) **86**

27 HepG2 HNF4G (SC-6558) **89**

28 HepG2 RXRA **90**

29 K562 NR2F2 (SC-271940) **93**

30 T-47D ERAA treatment=BPA 100nM **97**

31 T-47D ERAA treatment=Genistein 100nM **100**

32 T-47D ERAA treatment=Estradiol 10nM **103**

33 GM12878 TR4 **106**

34 HeLa-S3 TR4 **107**

35 HepG2 ERRA treatment=forskolin **108**

36 HepG2 HNF4A treatment=forskolin **112**

37 HepG2 TR4 **115**

38 K562 TR4 **116**

1 LoVo ESRRA

ESRRA All peaks

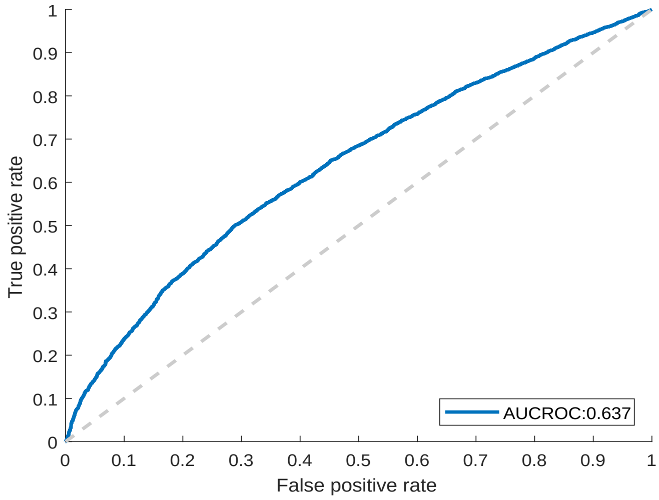

ESRRA Top 500 peaks

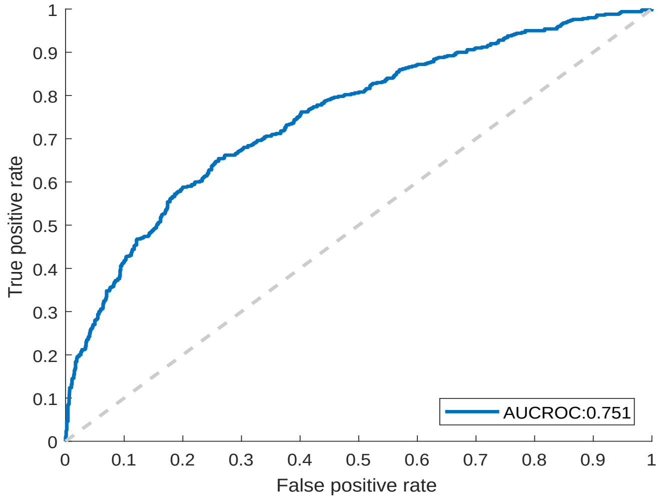

ESRRA:RXRA All peaks

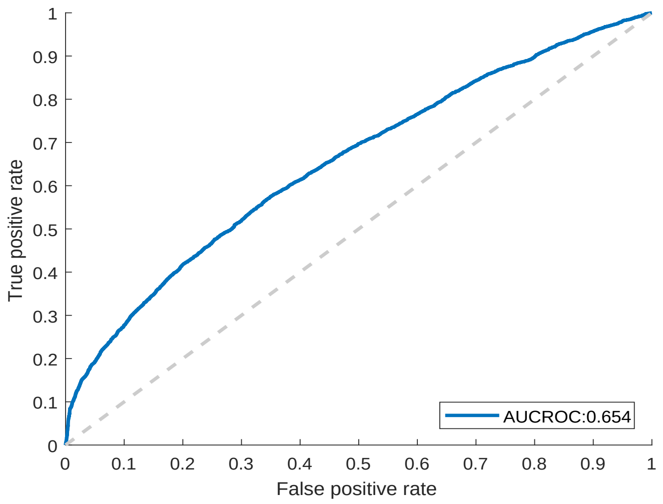

ESRRA:RXRA Top 500 peaks

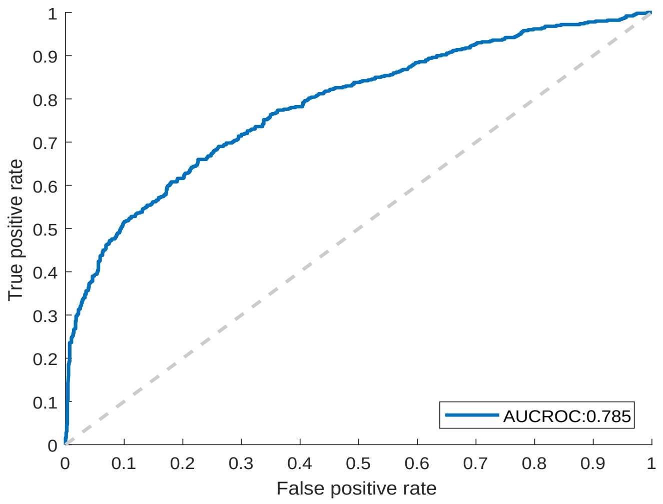

ESRRA+4 All peaks

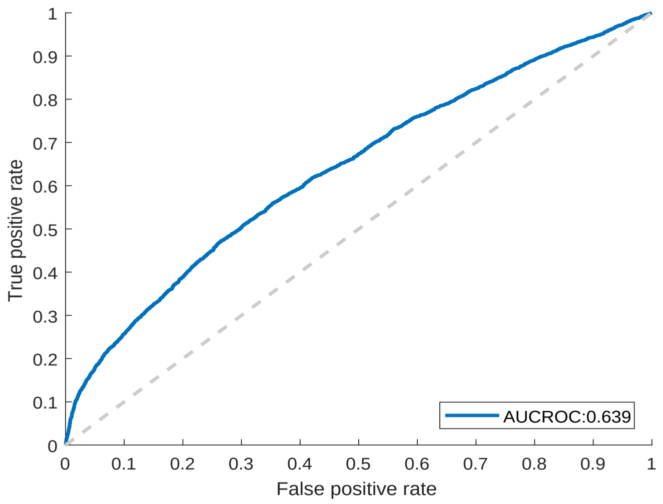

ESRRA+4 Top 500 peaks

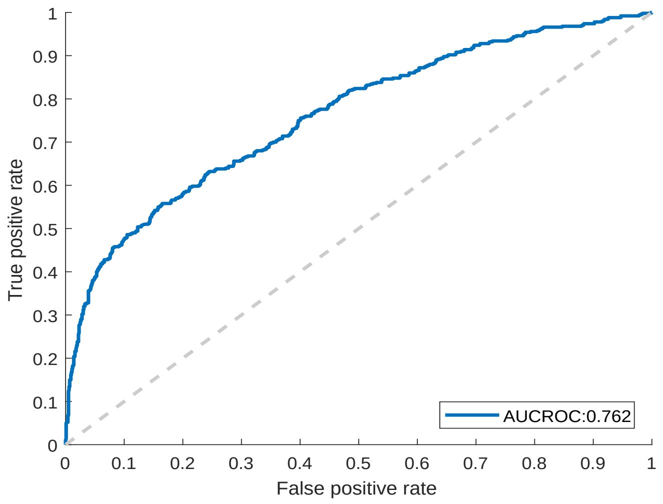

All peaks

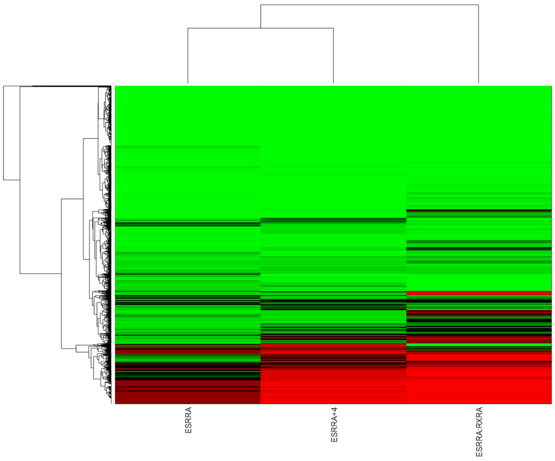

Top 500 peaks

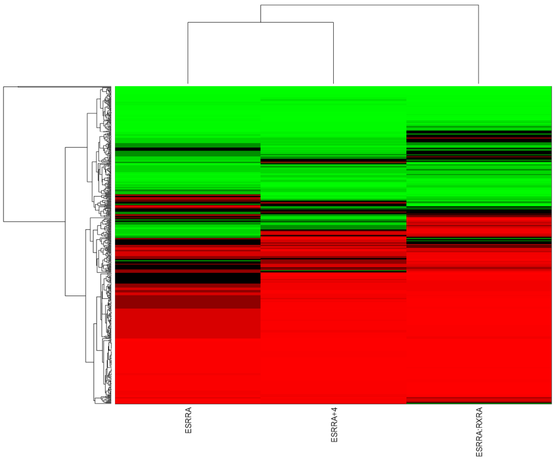

2 LoVo HNF4A

HNF4A All peaks

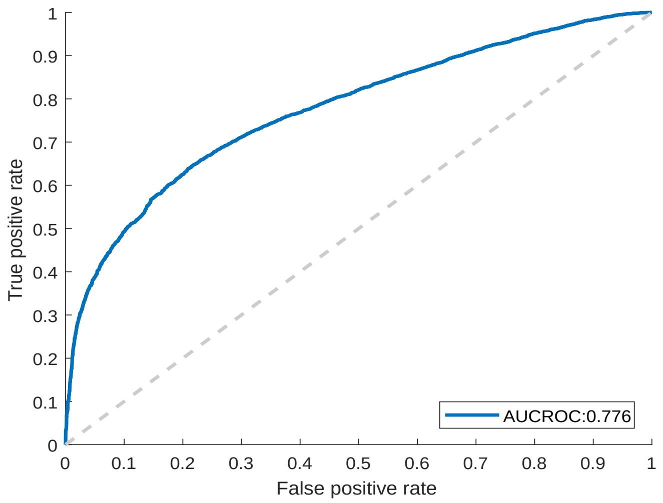

HNF4A Top 500 peaks

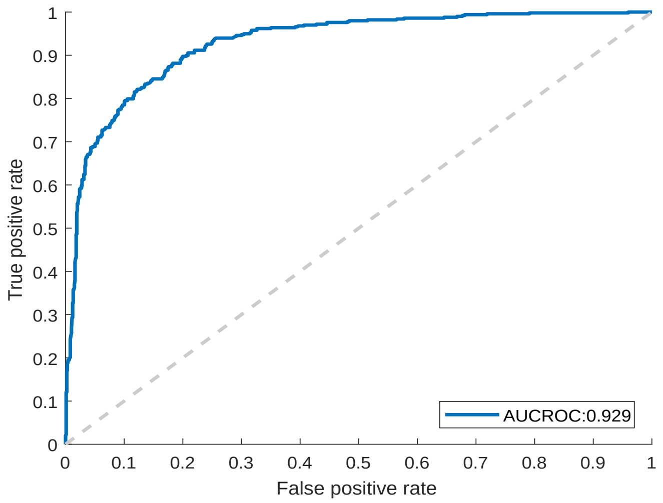

HNF4A+18 All peaks

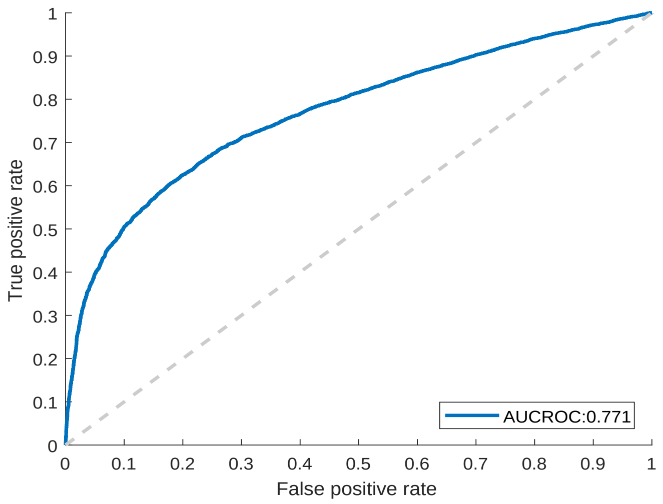

HNF4A+18 Top 500 peaks

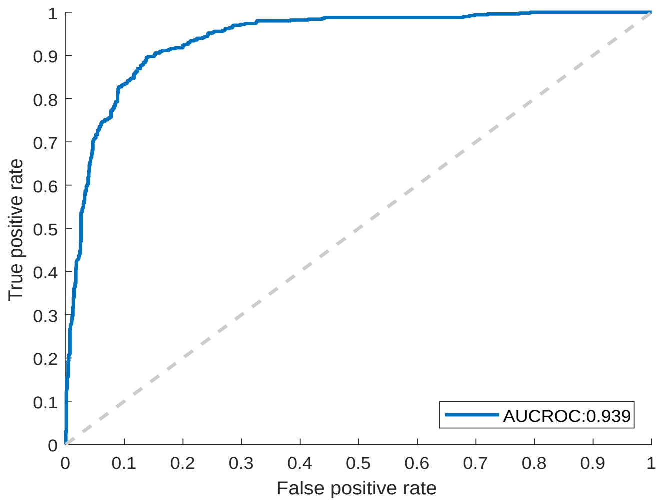

All peaks

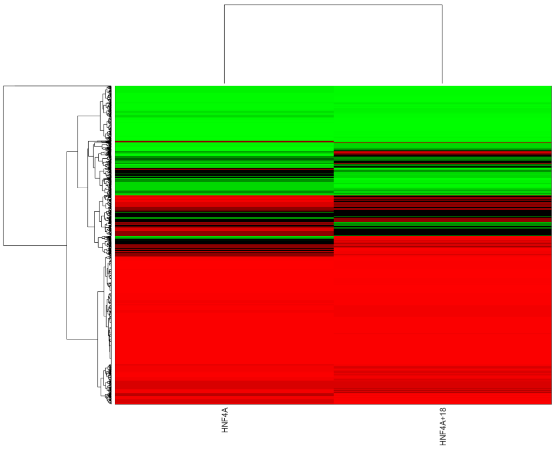

Top 500 peaks

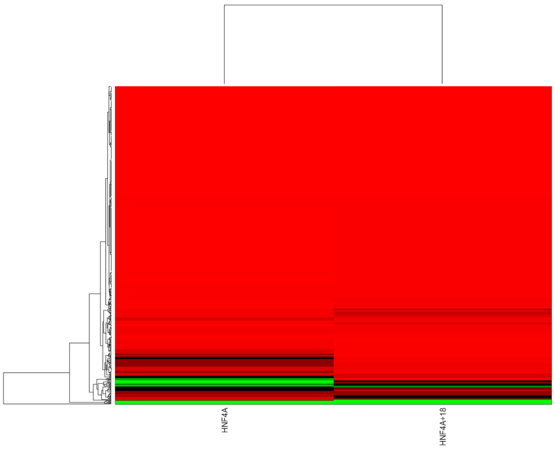

3 LoVo NR2F1

COUP-TF1 All peaks

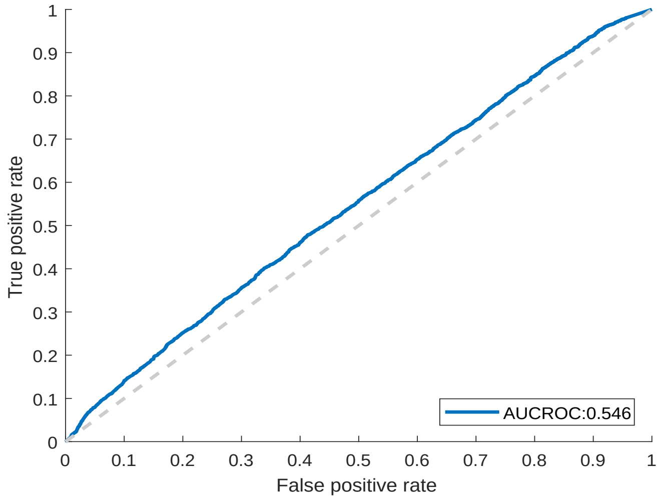

COUP-TF1 Top 500 peaks

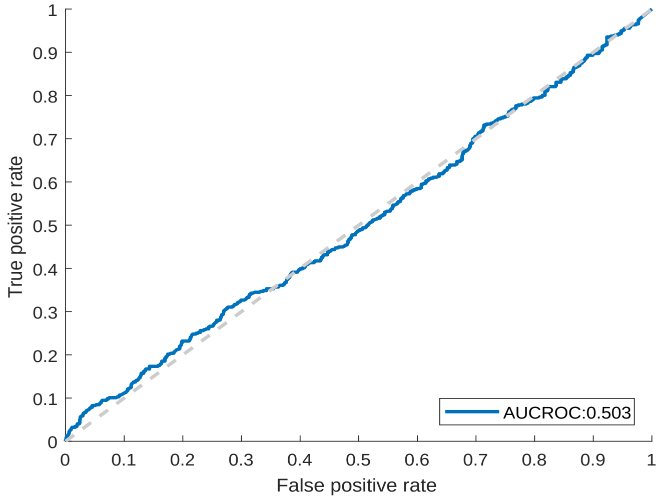

COUP-TF1+17 All peaks

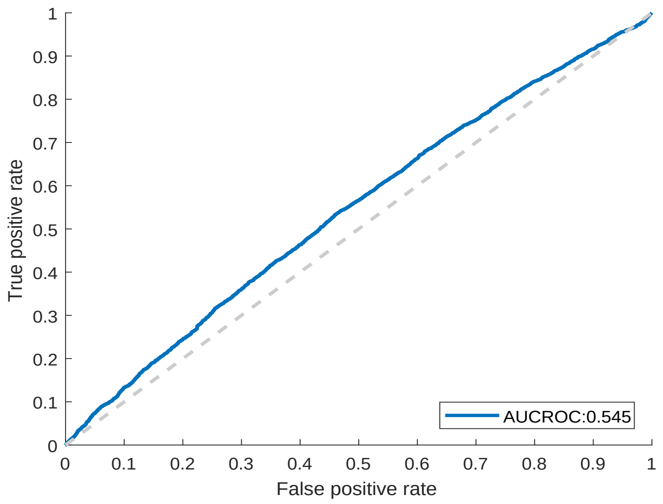

COUP-TF1+17 Top 500 peaks

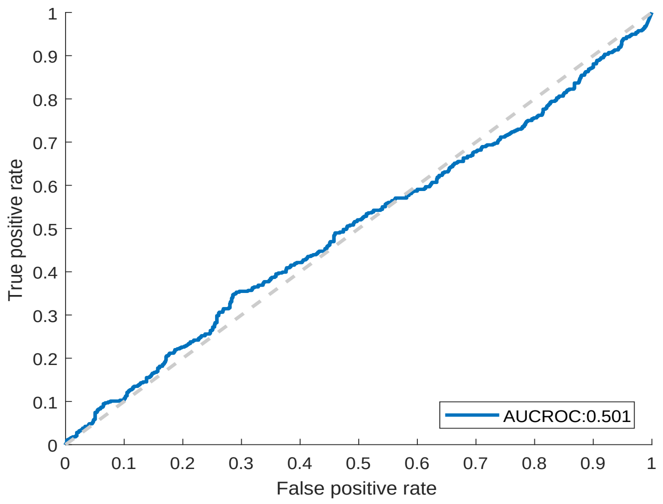

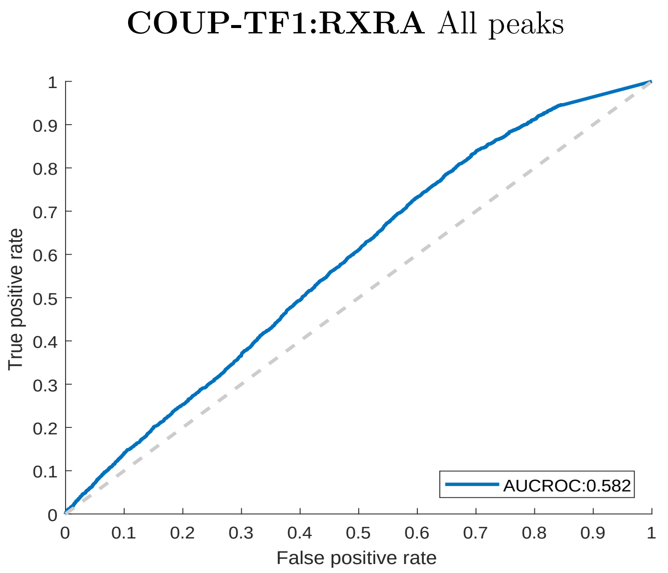
COUP-TF1:RXRA All peaks
True positive rate
False positive rate
AUCROC:0.582
0
0.1
0.2
0.3
0.4
0.5
0.6
0.7
0.8
0.9
1

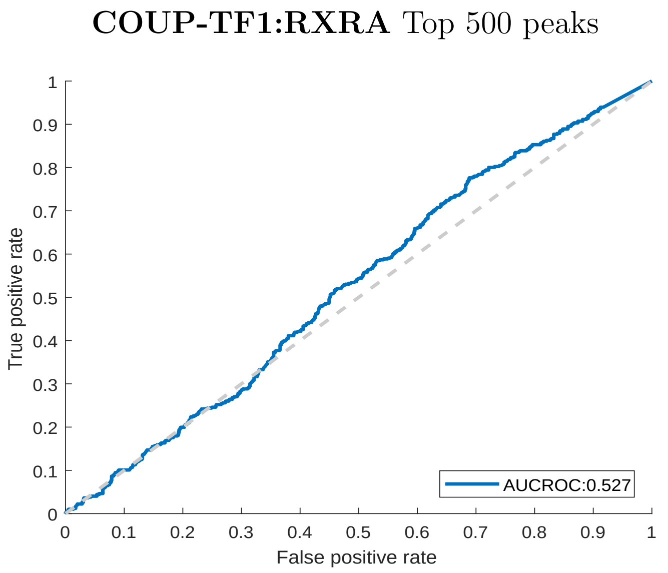
COUP-TF1:RXRA Top 500 peaks
True positive rate
False positive rate
AUCROC:0.527
0
0.1
0.2
0.3
0.4
0.5
0.6
0.7
0.8
0.9
1

All peaks

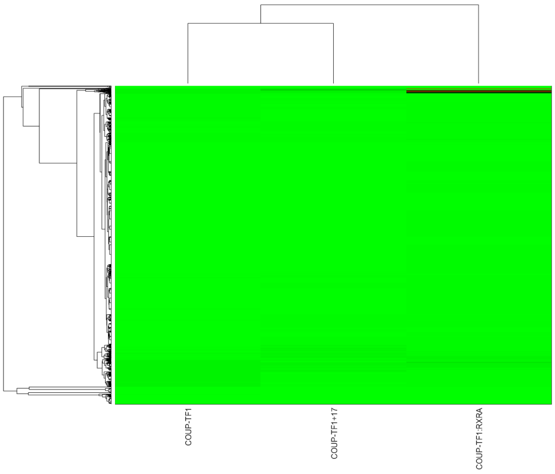

Top 500 peaks

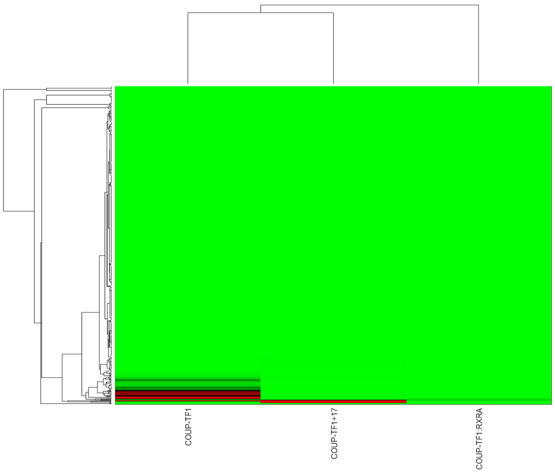

4 LoVo NR2F2

COUP-TF2 All peaks

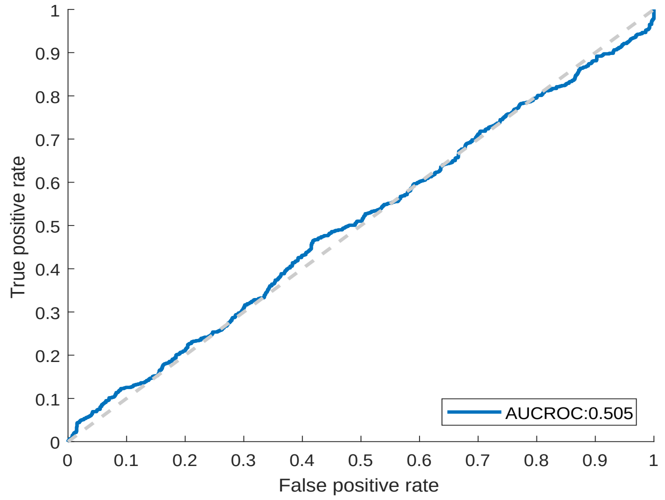

COUP-TF2 Top 500 peaks

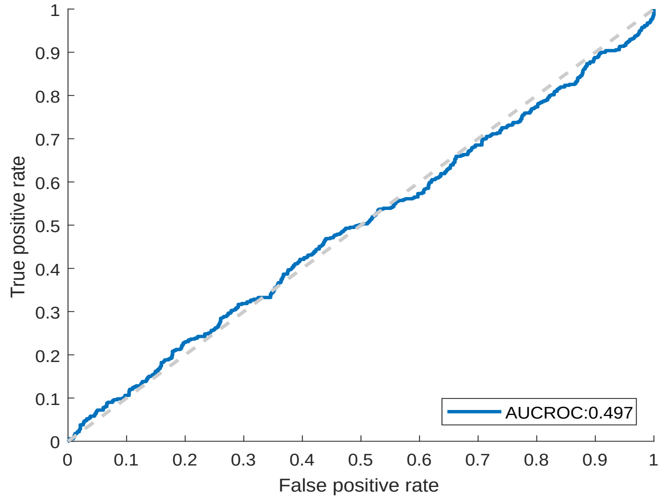

COUP-TF2+17 All peaks

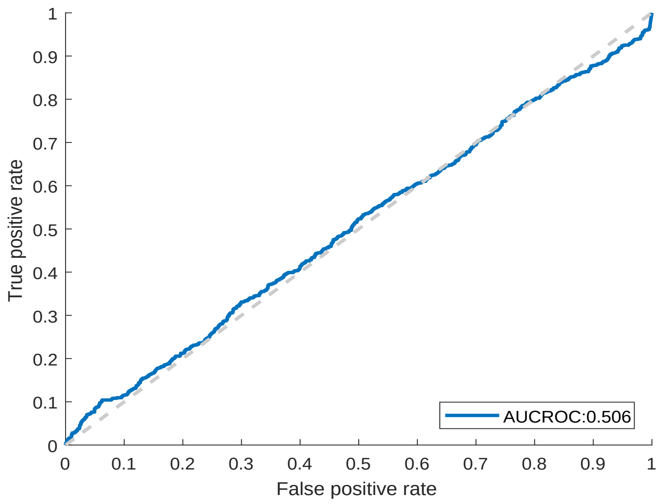

COUP-TF2+17 Top 500 peaks

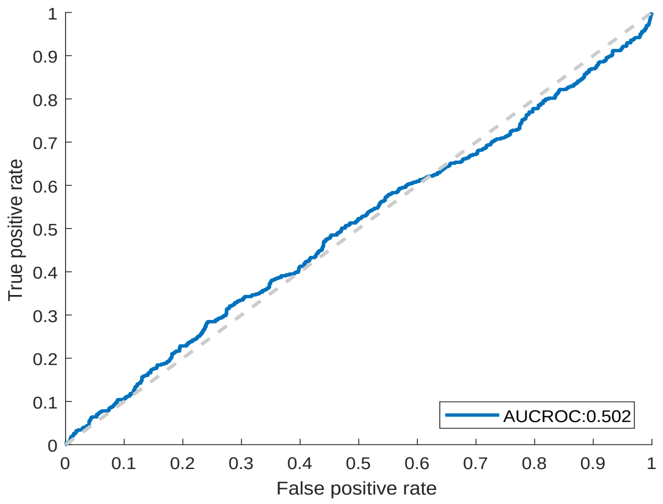

COUP-TF2:RXRA All peaks

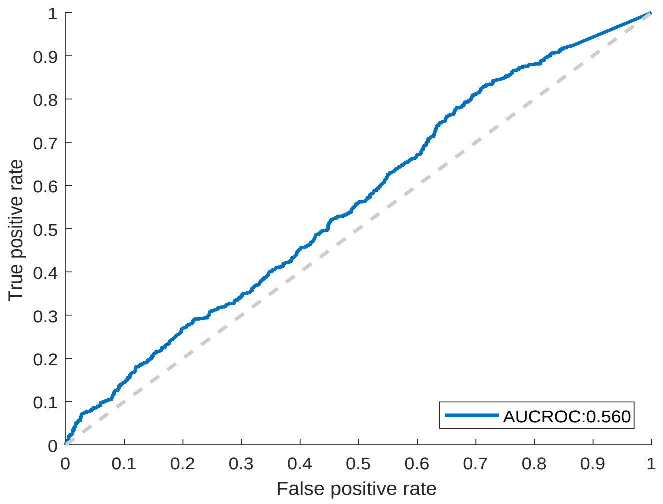

COUP-TF2:RXRA Top 500 peaks

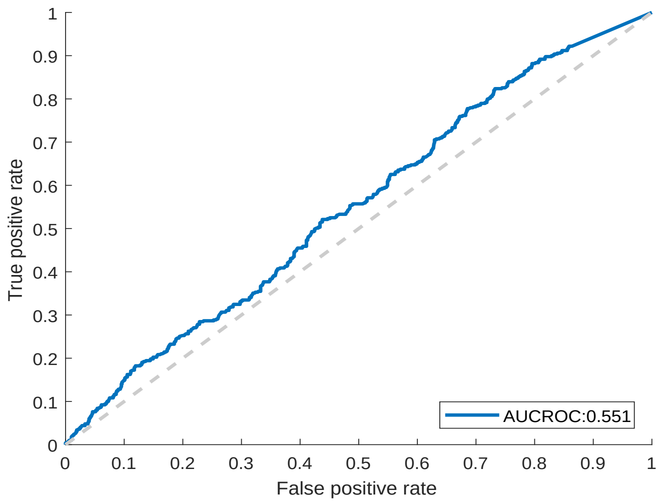

All peaks

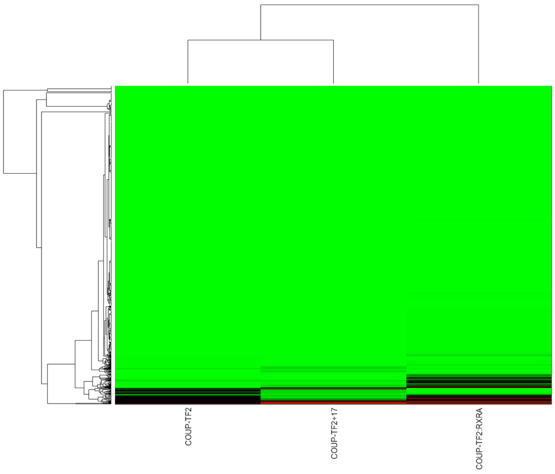

Top 500 peaks

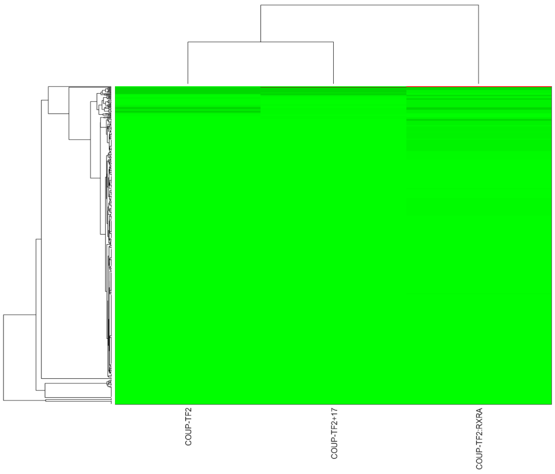

5 LoVo NR3C1

GR All peaks

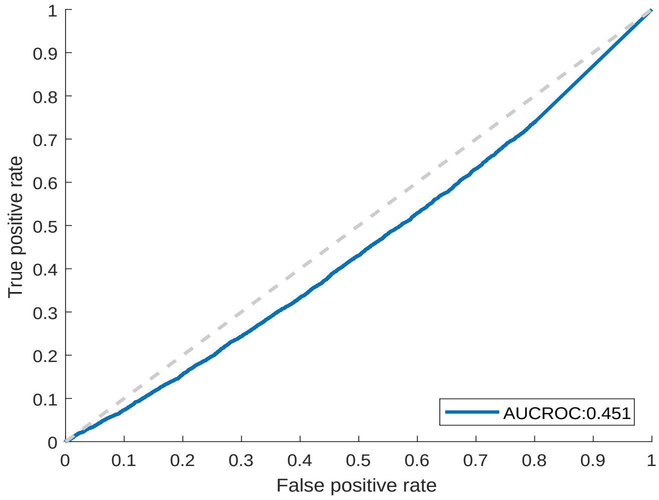

GR Top 500 peaks

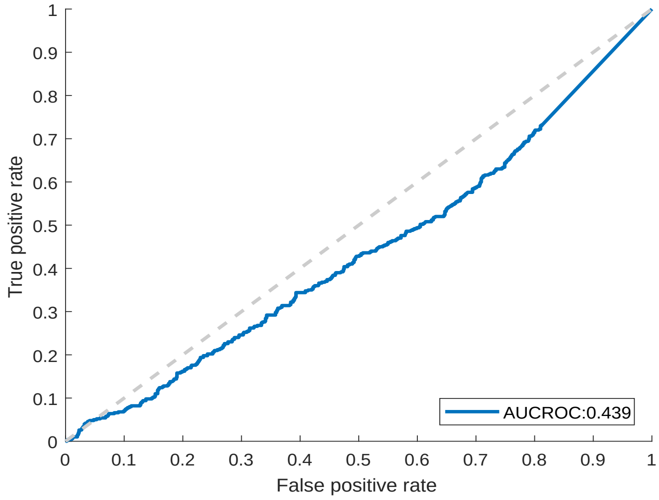

GR+3 All peaks

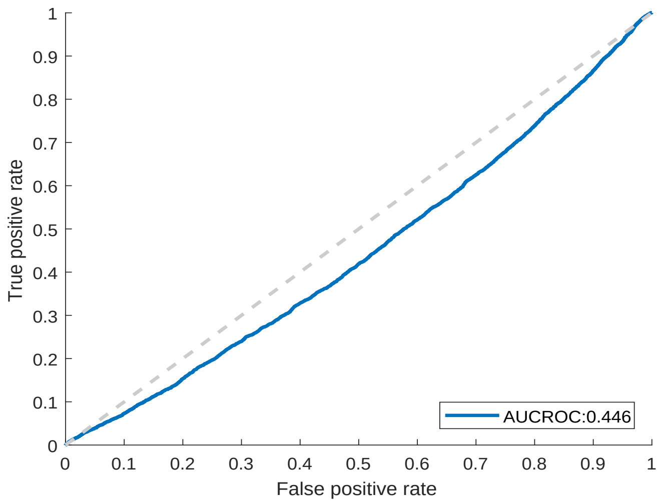

GR+3 Top 500 peaks

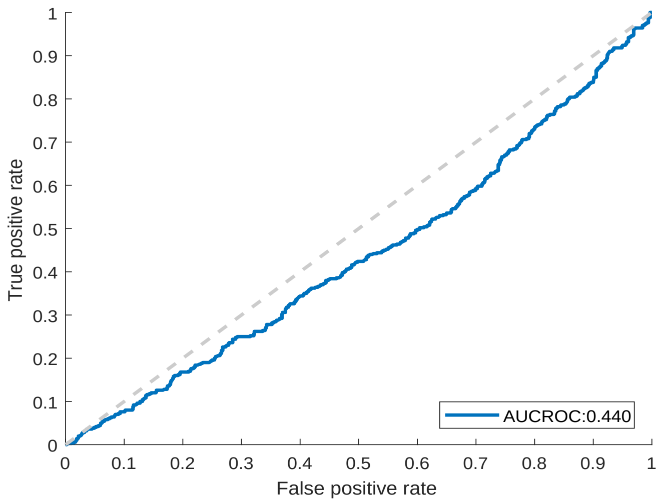

All peaks

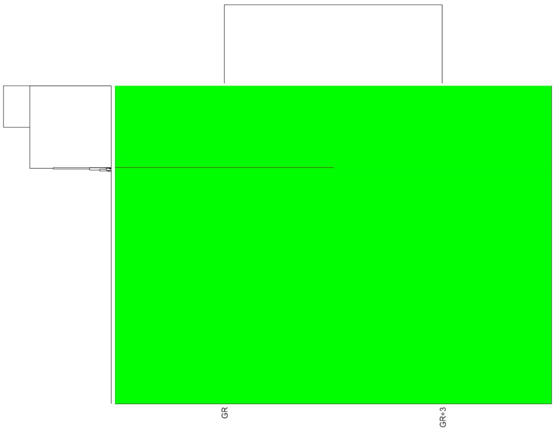

Top 500 peaks

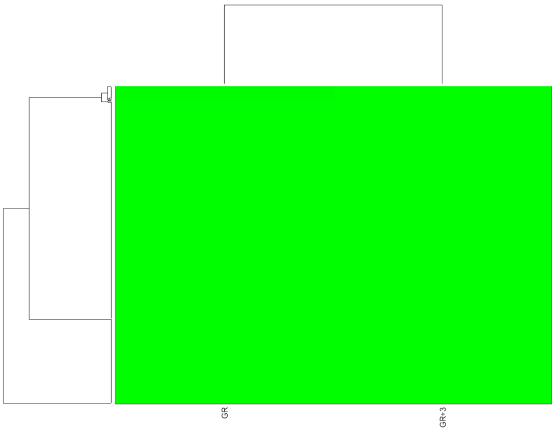

6 LoVo RXRA

RXRA All peaks

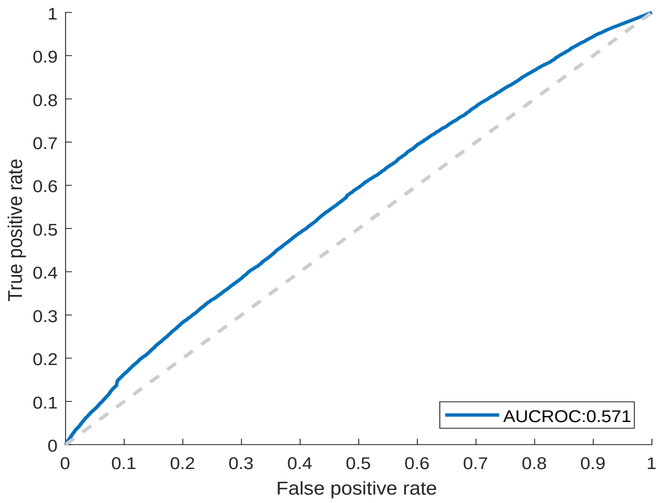

RXRA Top 500 peaks

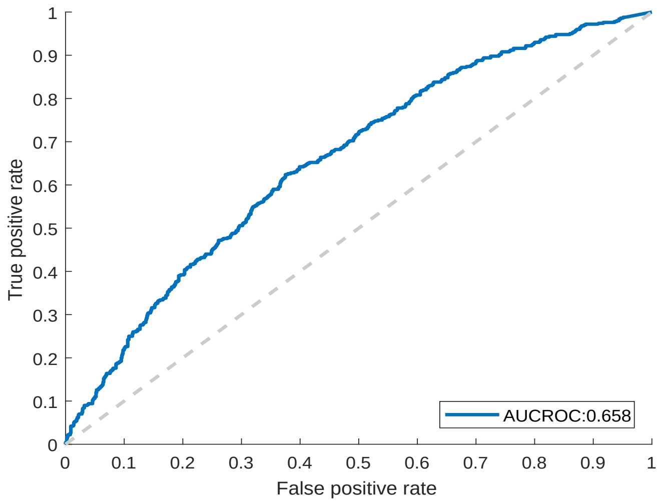

RXRA+17 All peaks

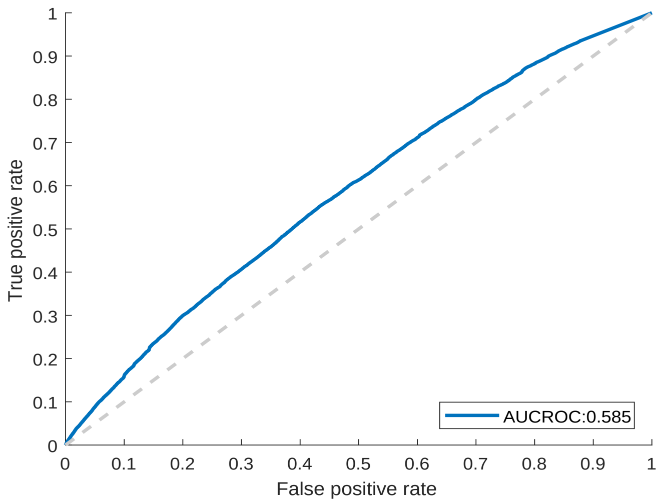

RXRA+17 Top 500 peaks

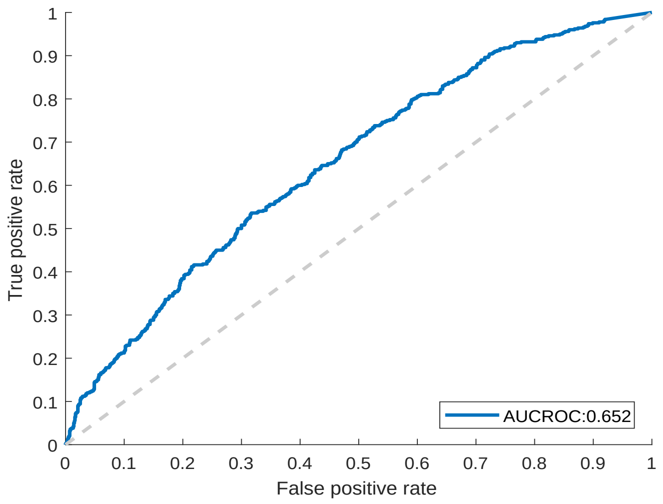

All peaks

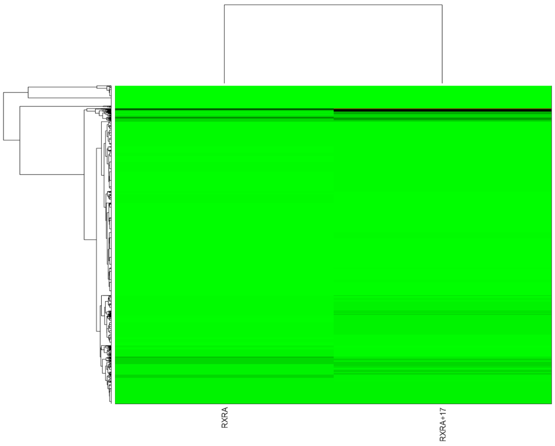

Top 500 peaks

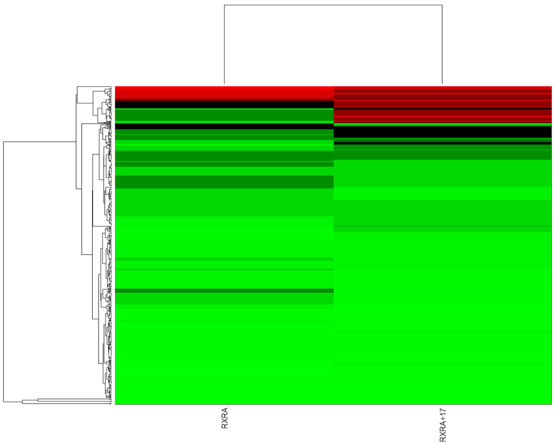

7 LoVo ESR1

ESR1+5 All peaks

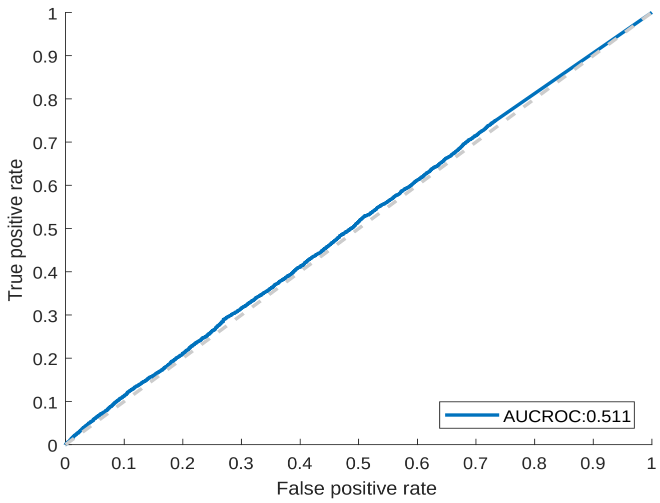

ESR1+5 Top 500 peaks

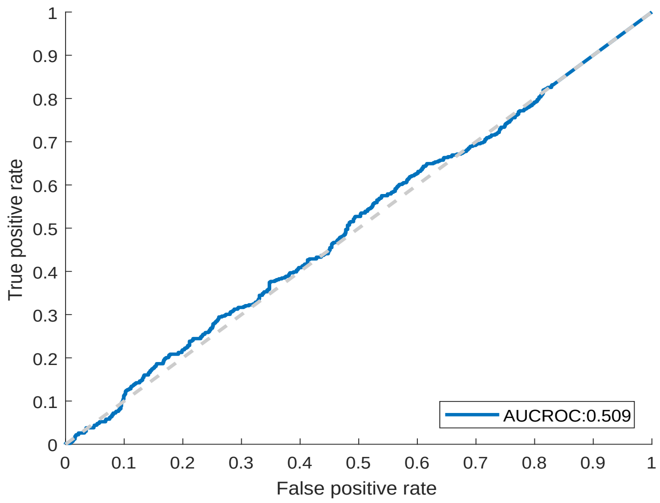

ESR1+6 All peaks

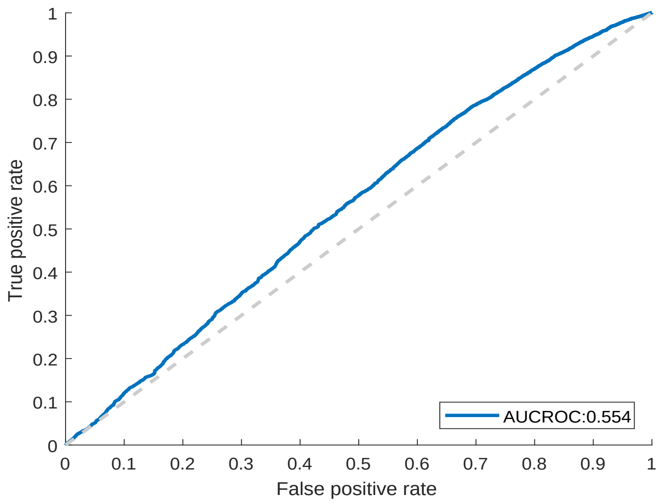

ESR1+6 Top 500 peaks

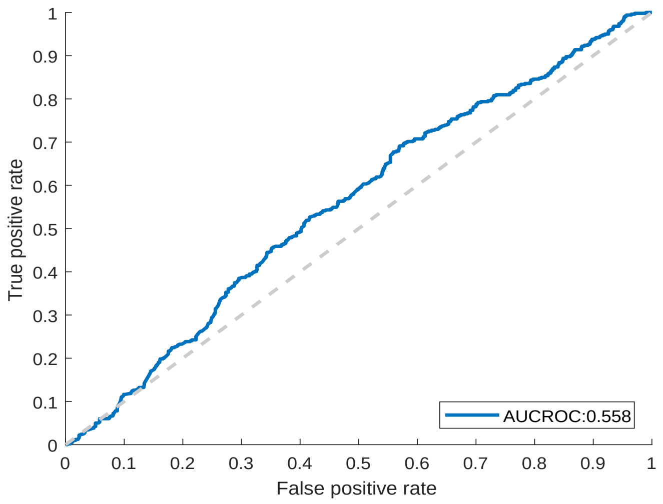

All peaks

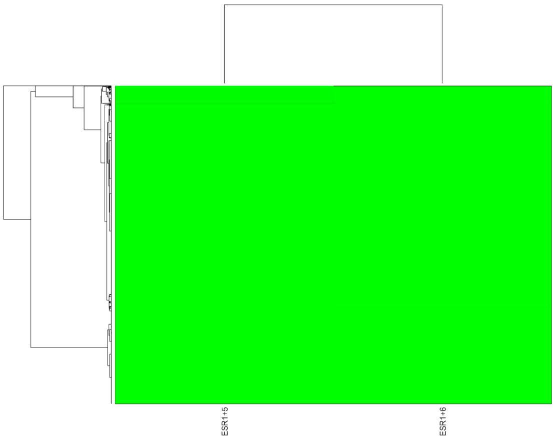

Top 500 peaks

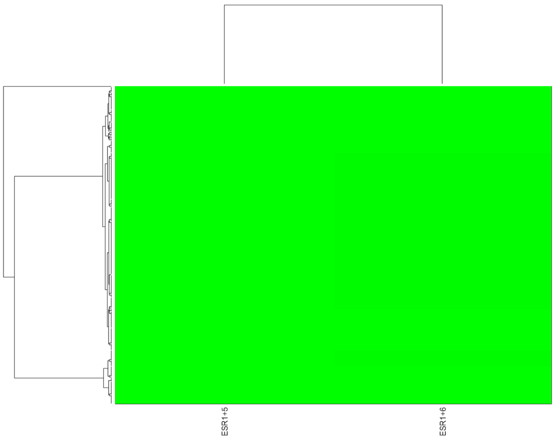

8 LoVo RARG

RARG All peaks

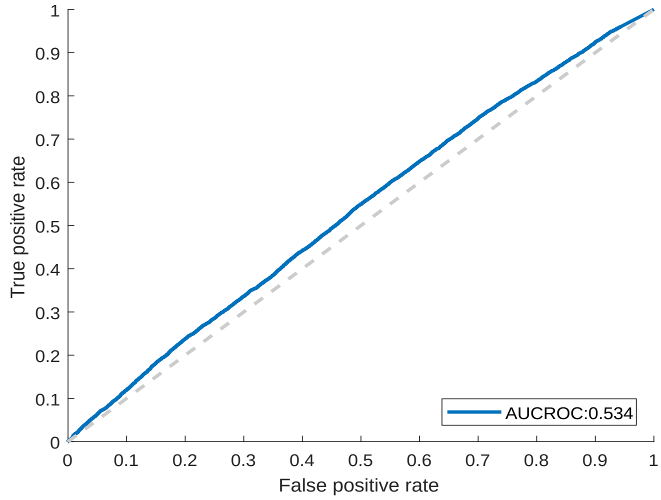

RARG Top 500 peaks

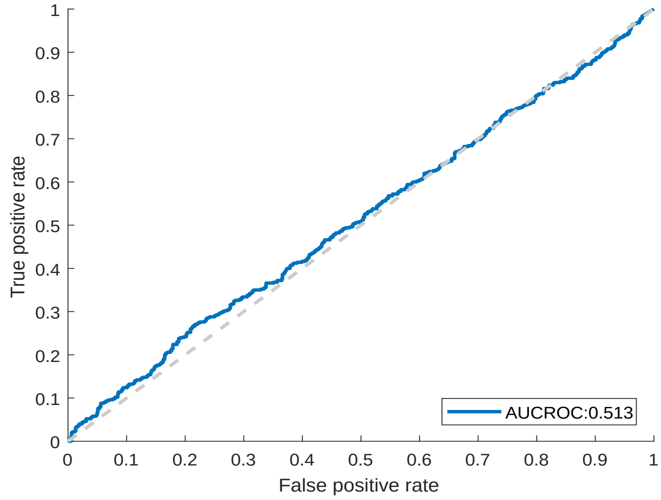

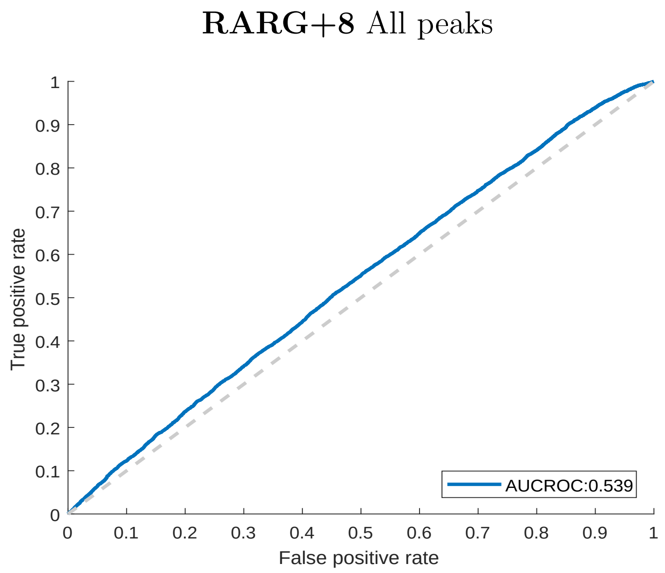
RARG+8 All peaks
True positive rate
False positive rate
AUCROC:0.539
0
0.1
0.2
0.3
0.4
0.5
0.6
0.7
0.8
0.9
1

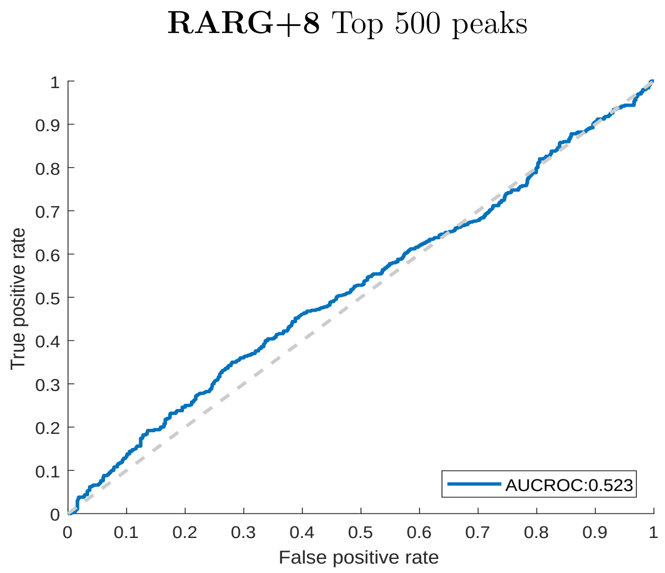
RARG+8 Top 500 peaks
True positive rate
False positive rate
AUCROC:0.523
0
0.1
0.2
0.3
0.4
0.5
0.6
0.7
0.8
0.9
1

RARG:RXRA All peaks

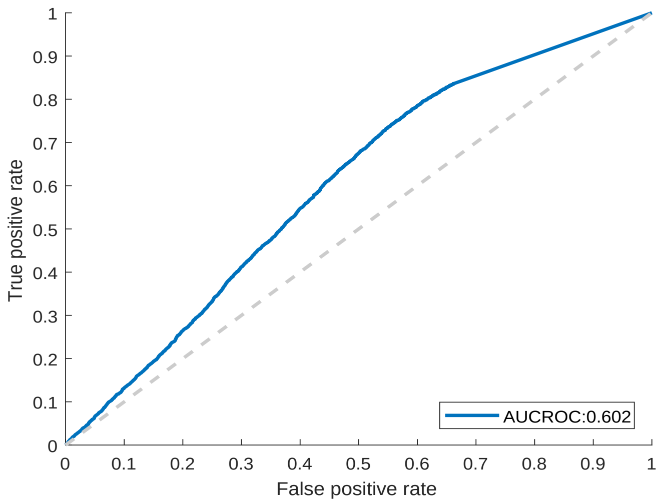

RARG:RXRA Top 500 peaks

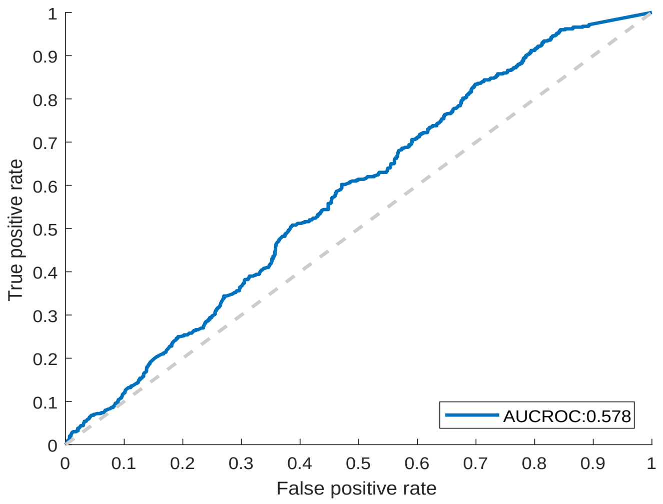

All peaks

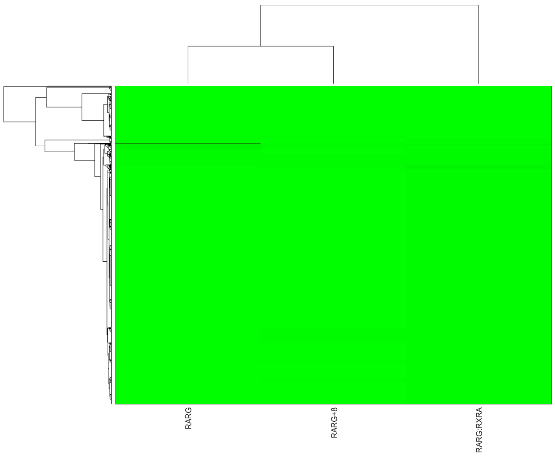

Top 500 peaks

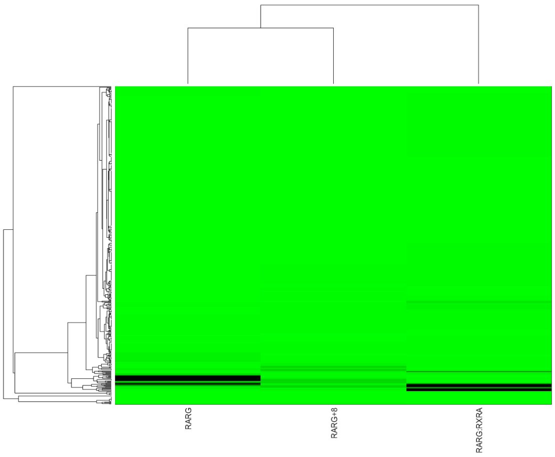

9 LoVo ESR1:RXRA

ESR1+5 All peaks

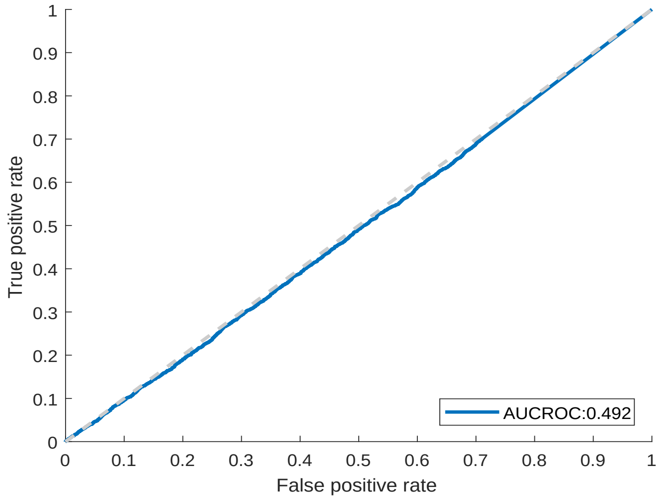

ESR1+6 All peaks

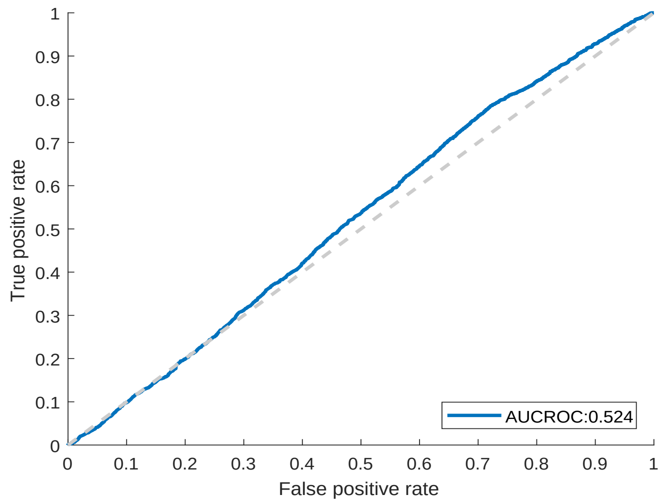

All peaks

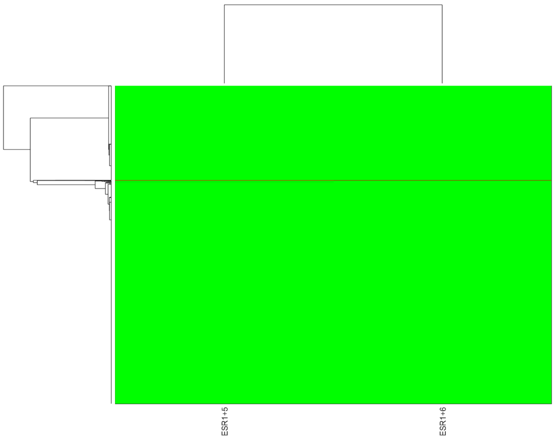

10 LoVo ESRRA:RXRA

ESRRA All peaks

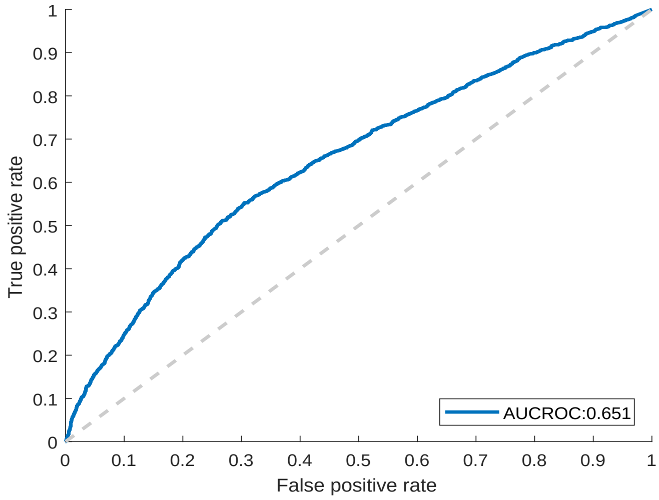

ESRRA:RXRA All peaks

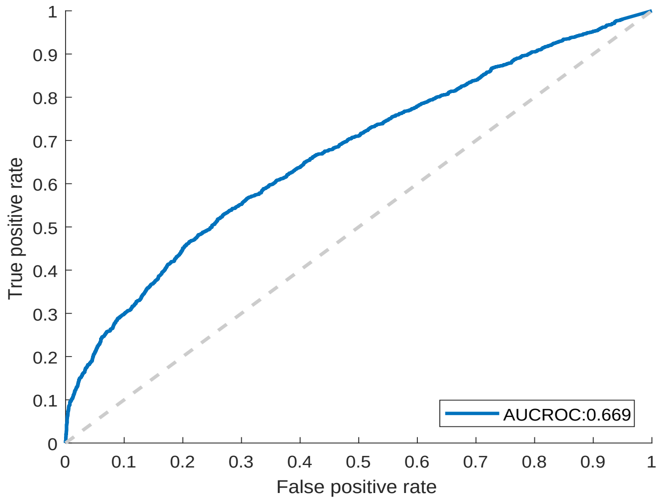

ESRRA+4 All peaks

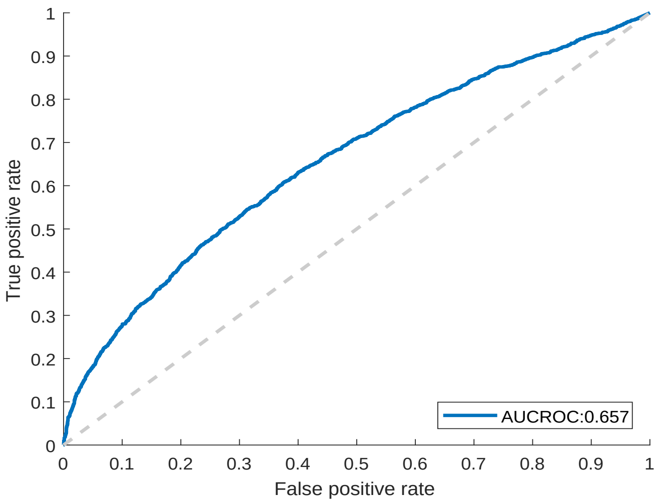

All peaks

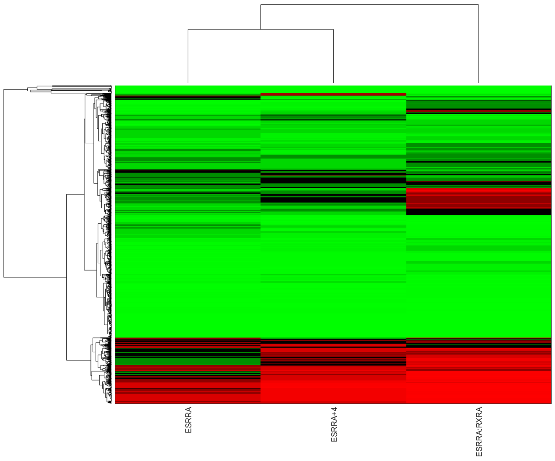

11 LoVo HNF4A:RXRA

HNF4A All peaks

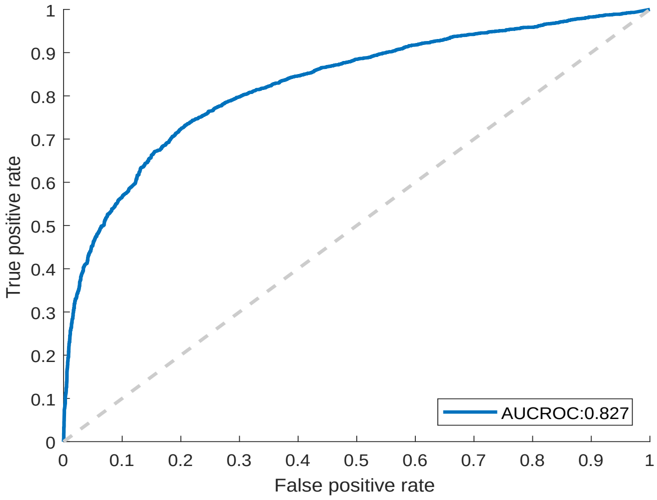

HNF4A+18 All peaks

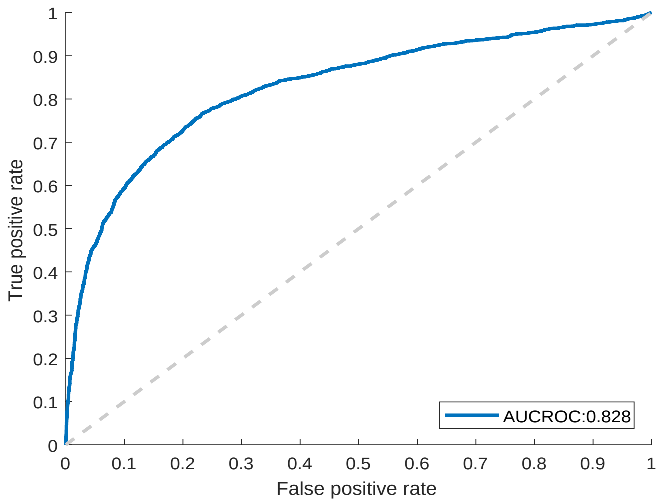

All peaks

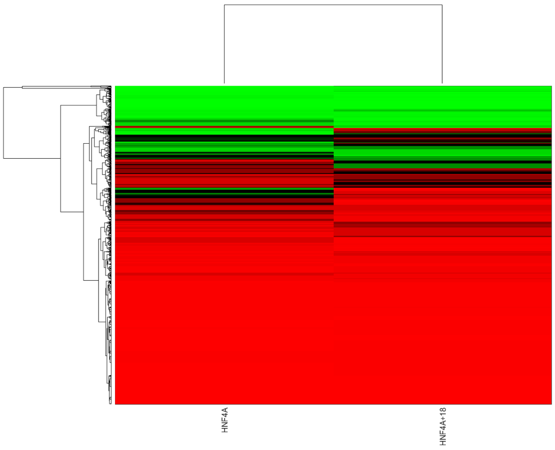

12 LoVo NR2F1:RXRA

COUP-TF1 All peaks

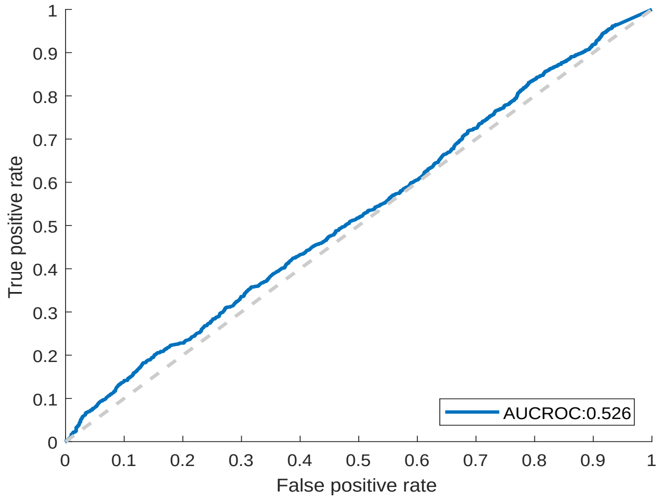

COUP-TF1+17 All peaks

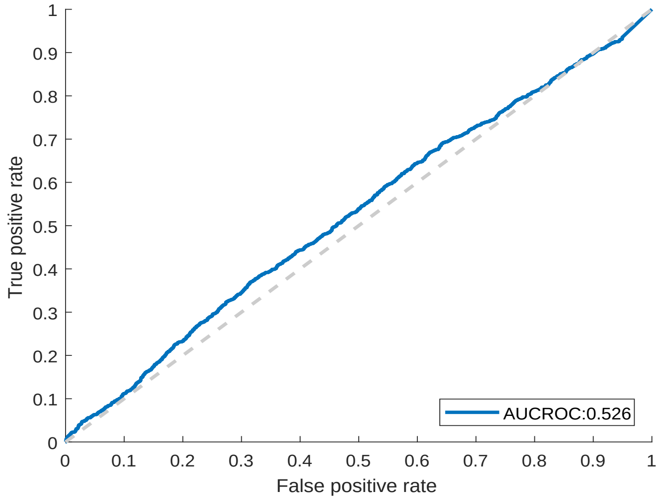

COUP-TF1:RXRA All peaks

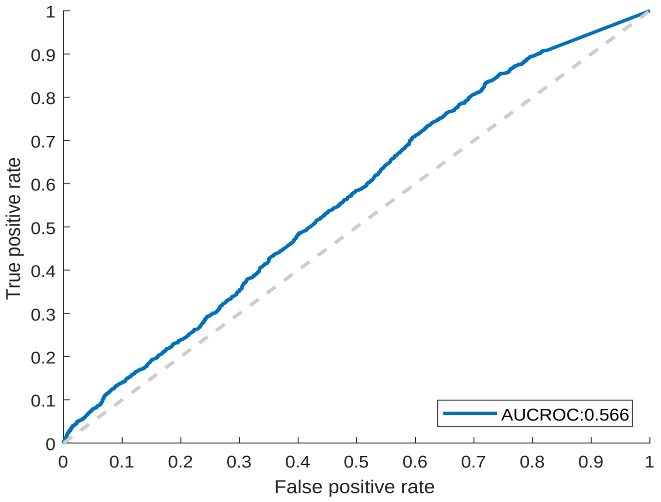

All peaks

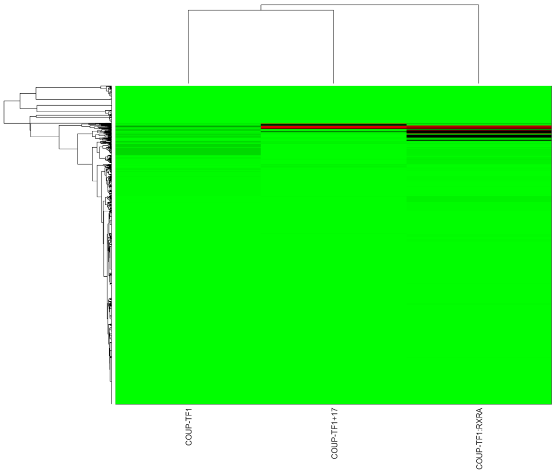

13 LoVo NR2F2:RXRA

COUP-TF2 All peaks

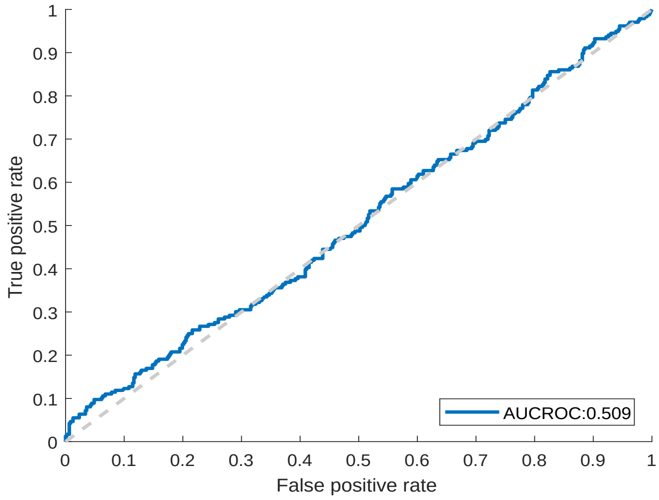

COUP-TF2+17 All peaks

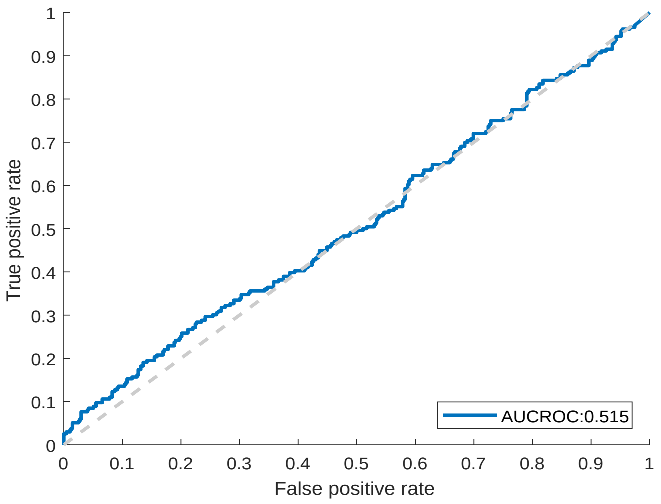

COUP-TF2:RXRA All peaks

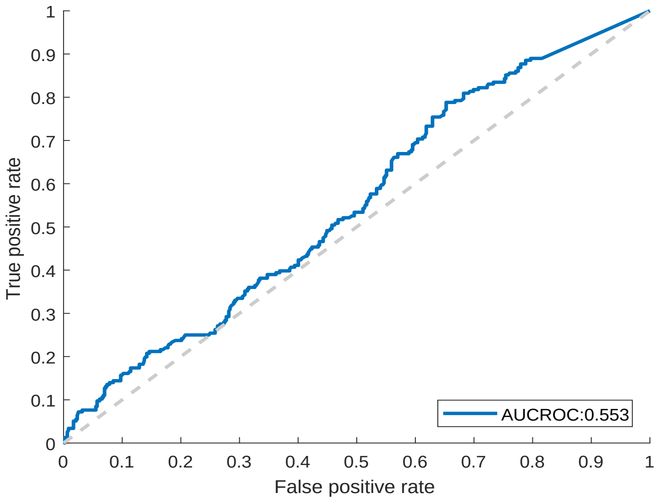

All peaks

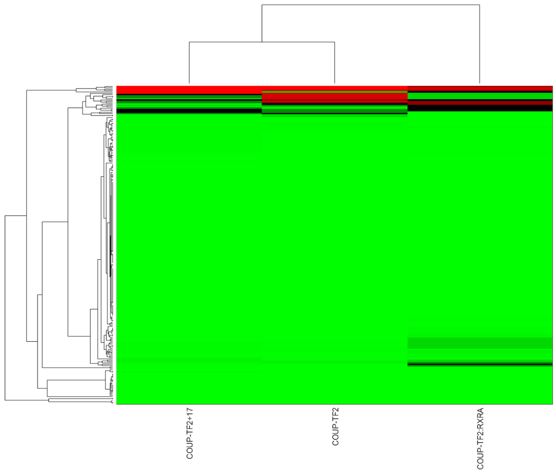

14 LoVo NR3C1:RXRA

GR All peaks

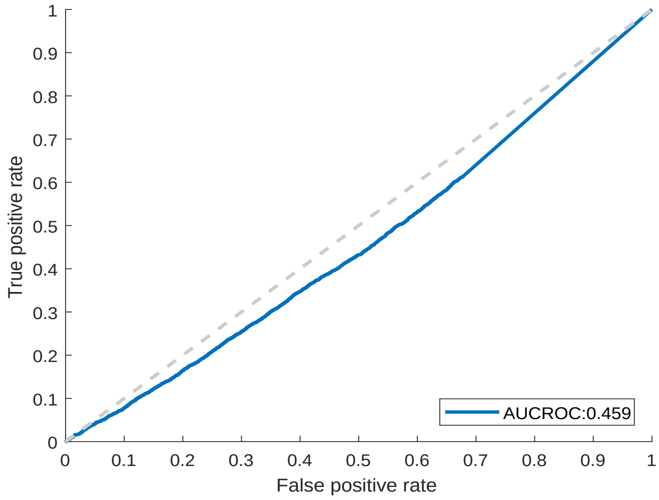

GR+3 All peaks

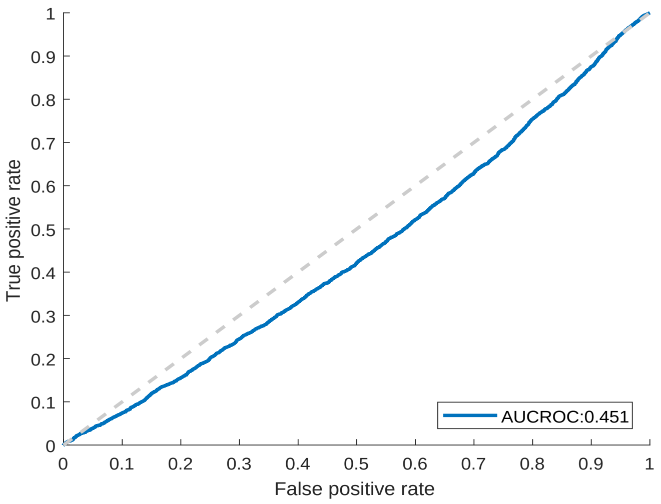

All peaks

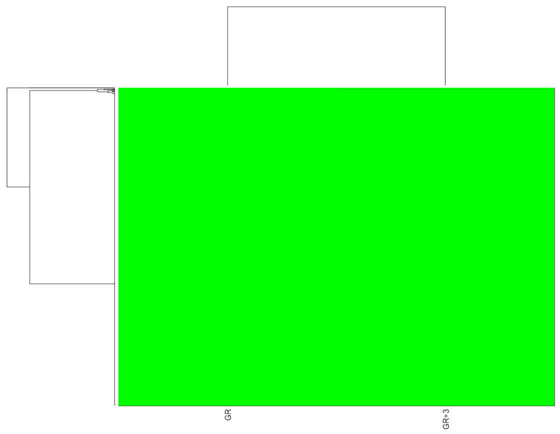

15 LoVo RARG:RXRA

RARG All peaks

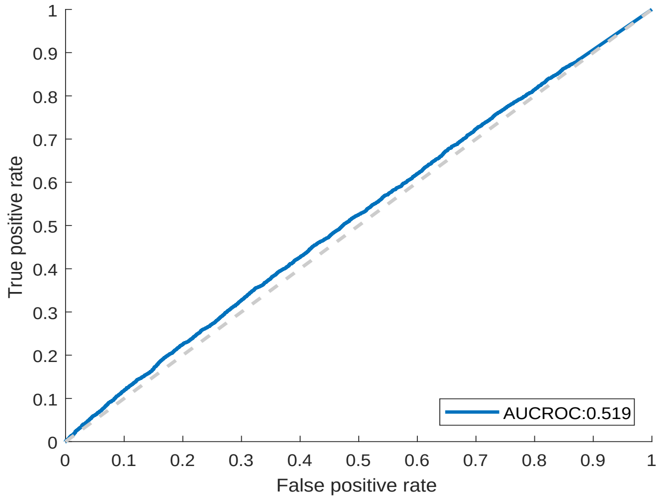

RARG+8 All peaks

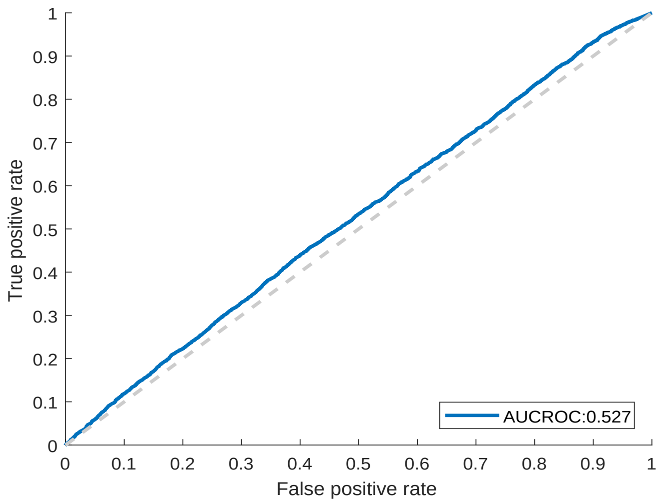

RARG:RXRA All peaks

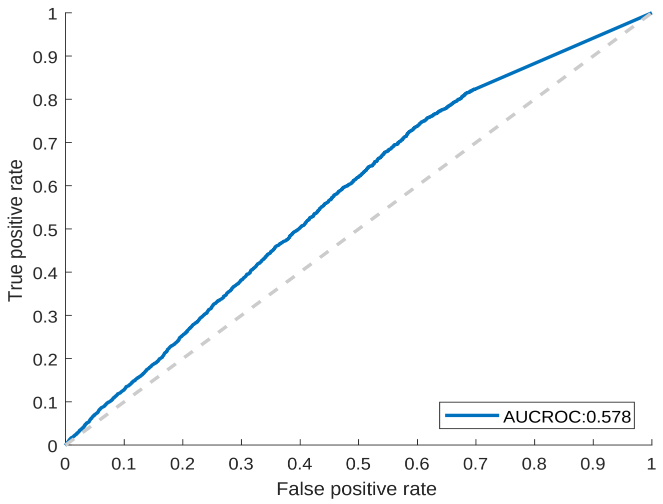

All peaks

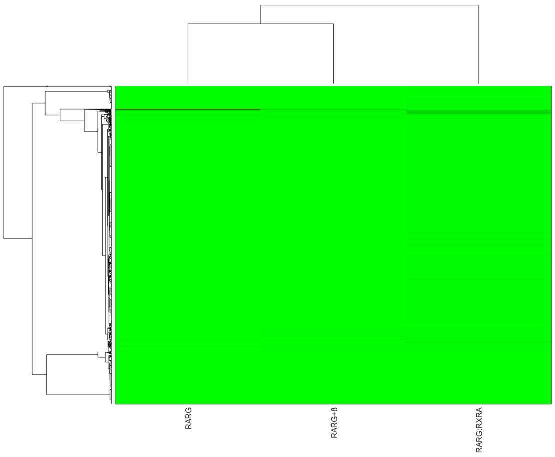

16 A549 GR treatment:Dex 500pm

GR All peaks

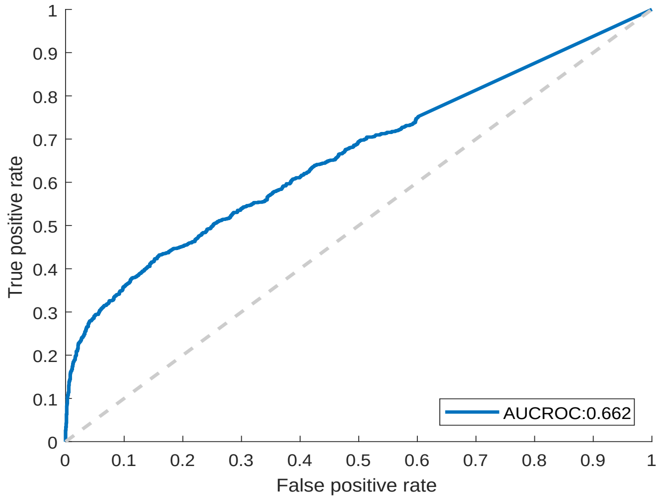

GR Top 500 peaks

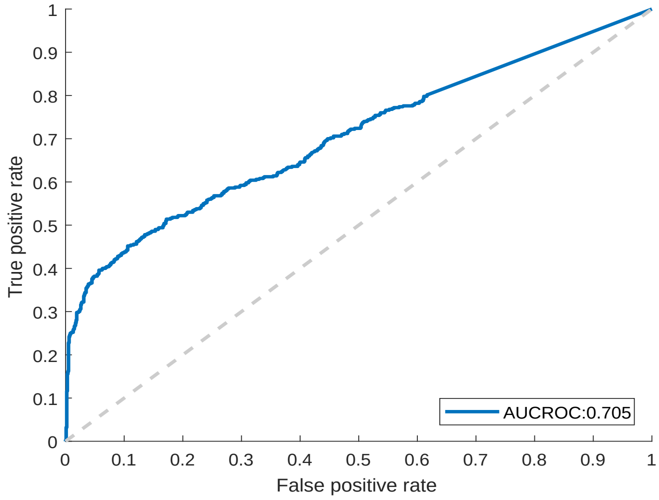

GR+3 All peaks

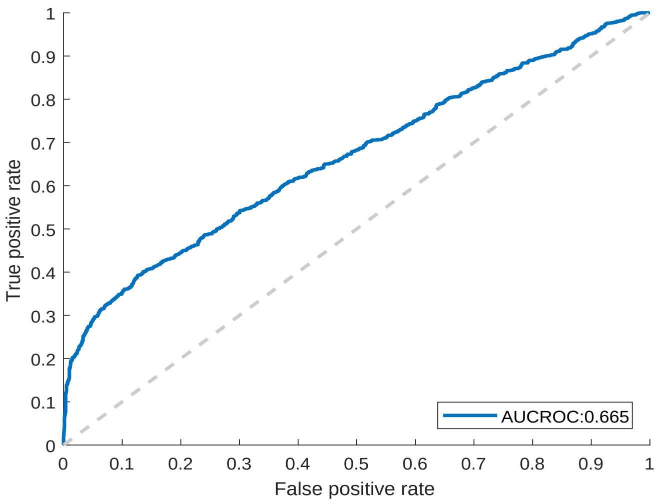

GR+3 Top 500 peaks

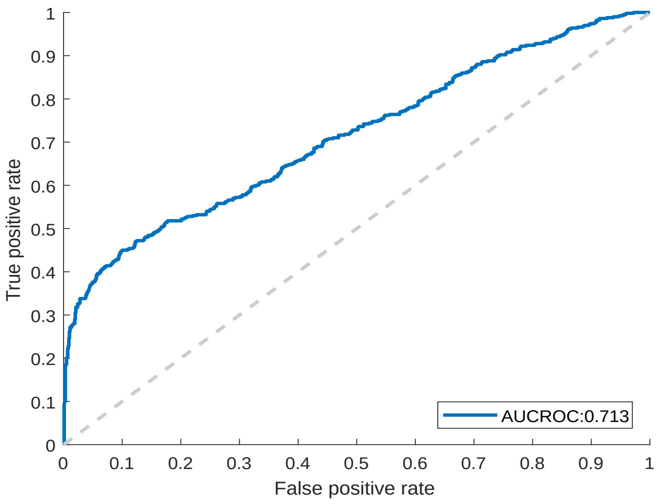

All peaks

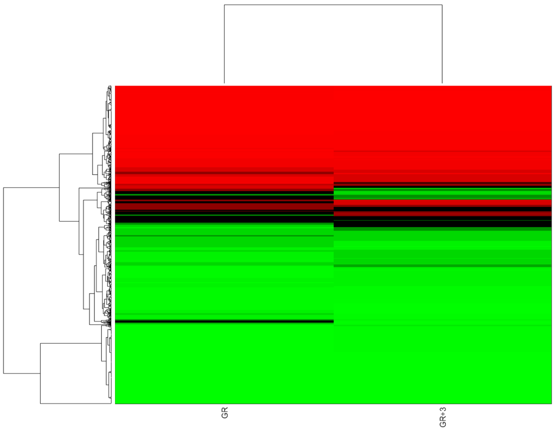

Top 500 peaks

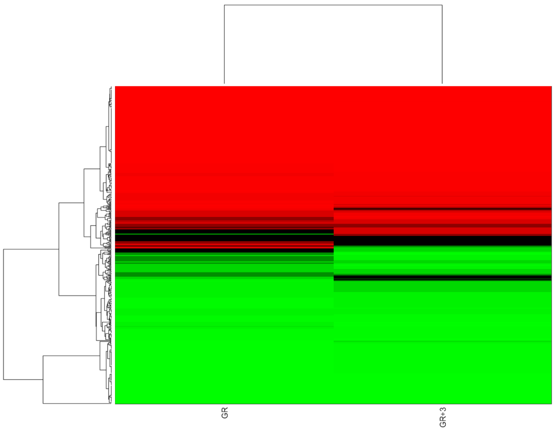

17 A549 GR treatment:Dex 50nm

GR All peaks

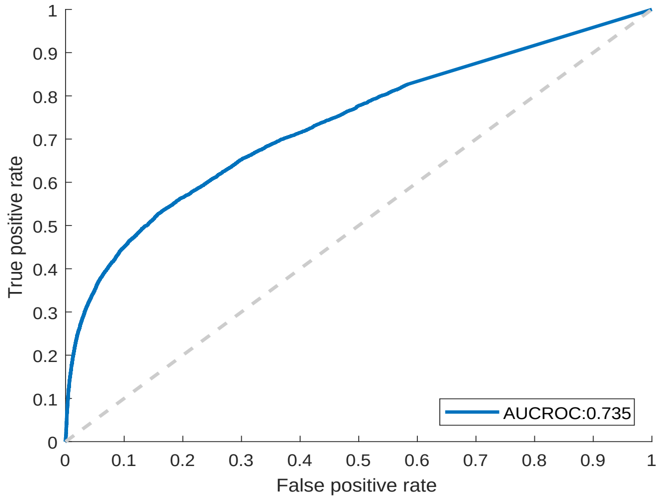

GR Top 500 peaks

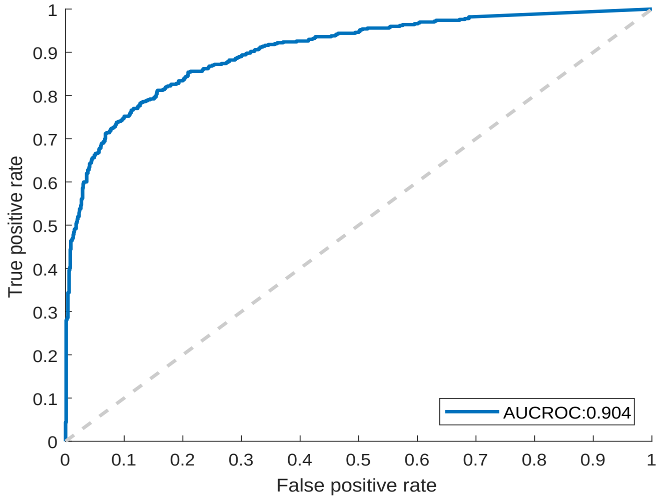

GR+3 All peaks

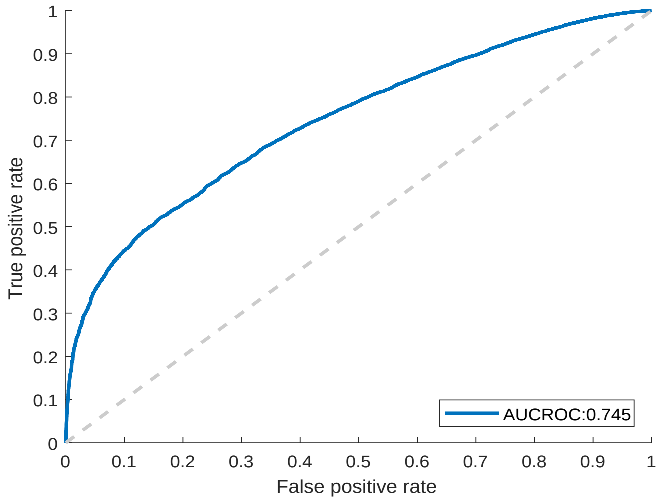

GR+3 Top 500 peaks

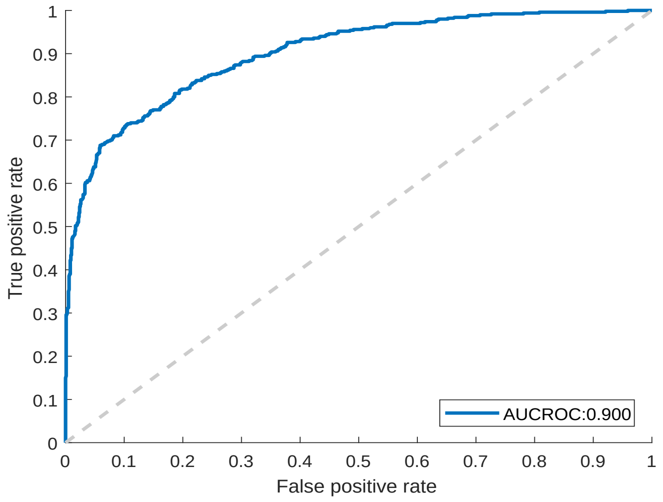

All peaks

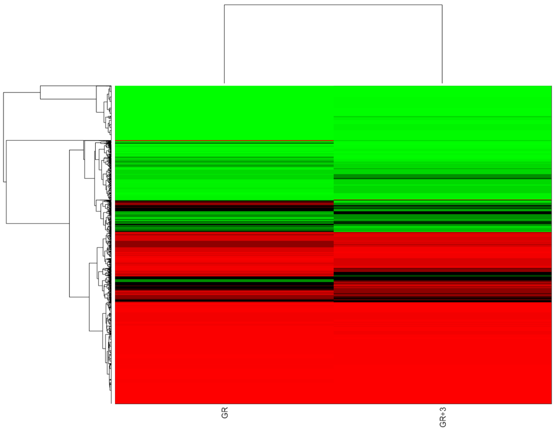

Top 500 peaks

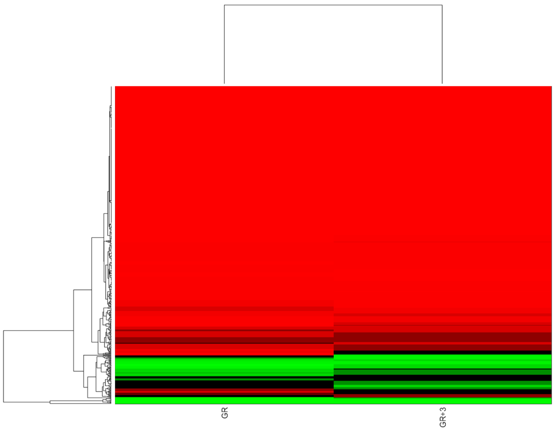

18 A549 GR treatment:Dex 5nm

GR All peaks

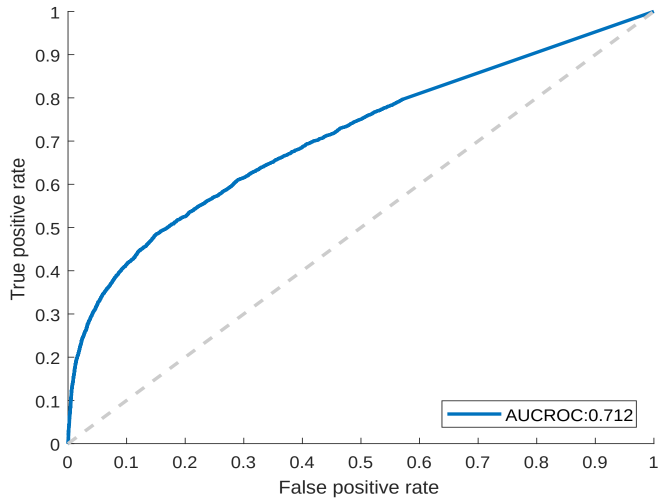

GR Top 500 peaks

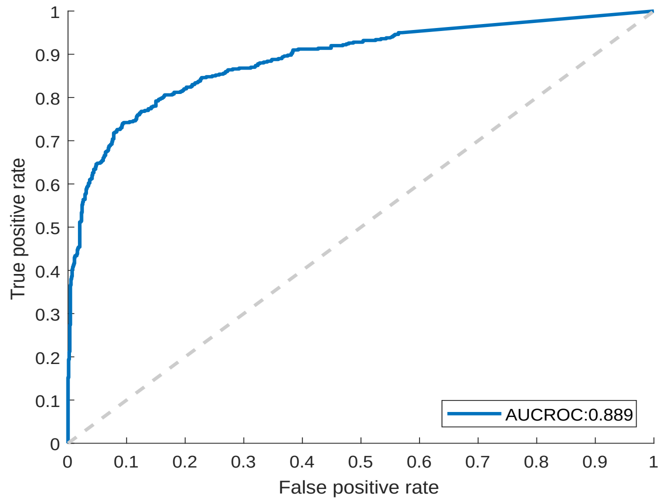

GR+3 All peaks

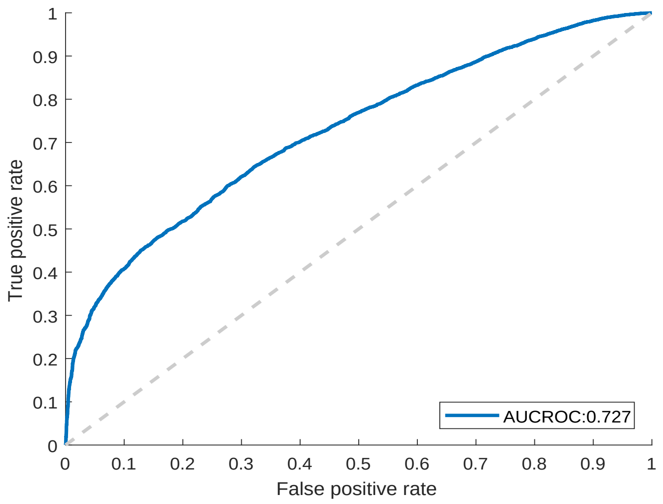

GR+3 Top 500 peaks

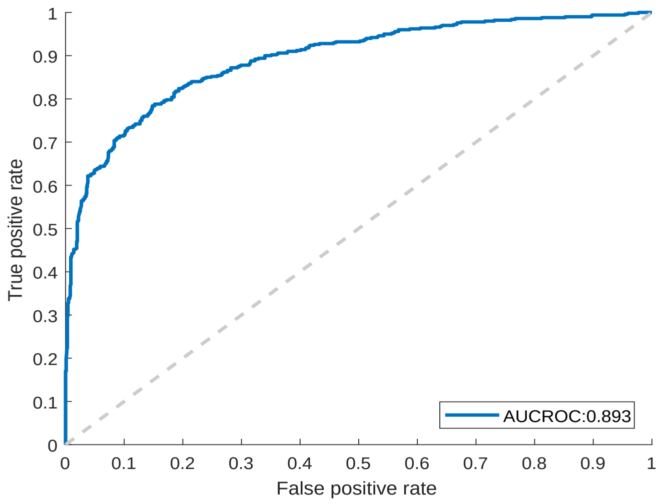

All peaks

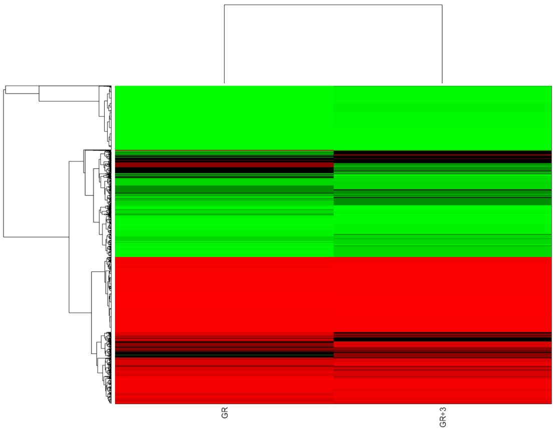

Top 500 peaks

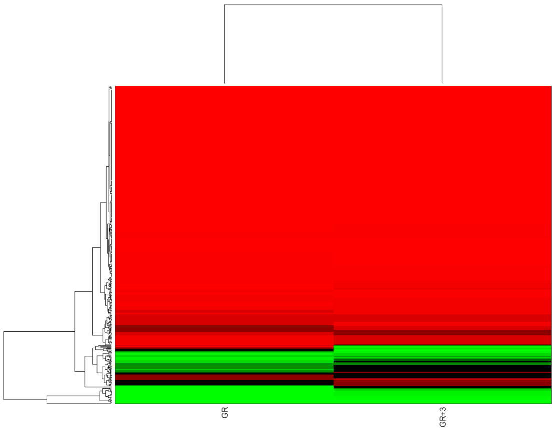

19 A549 GR treatment:Dex 100nm

GR All peaks

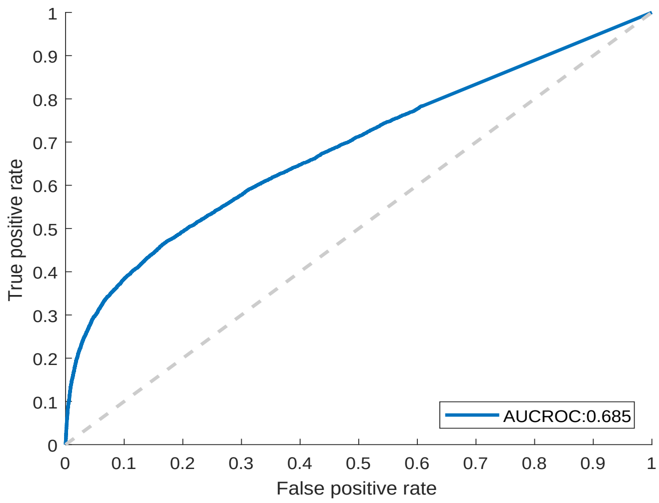

GR Top 500 peaks

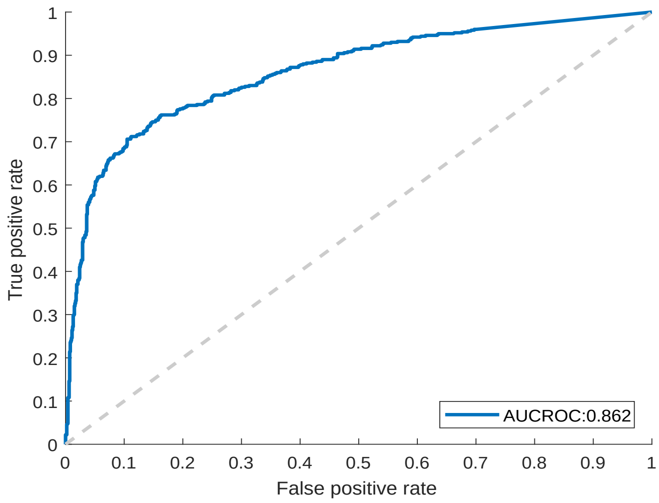

GR+3 All peaks

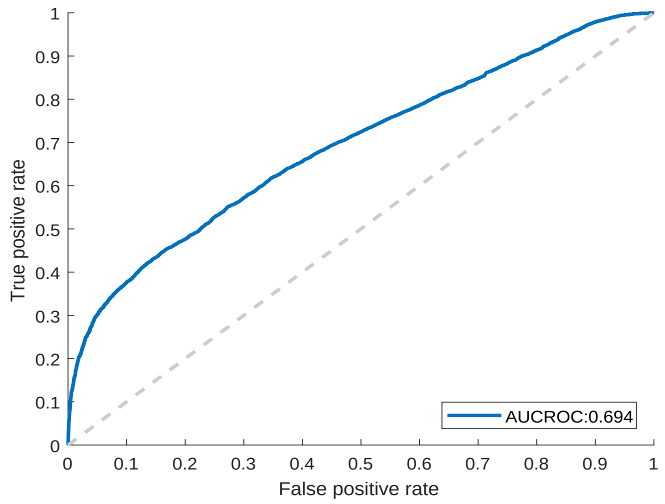

GR+3 Top 500 peaks

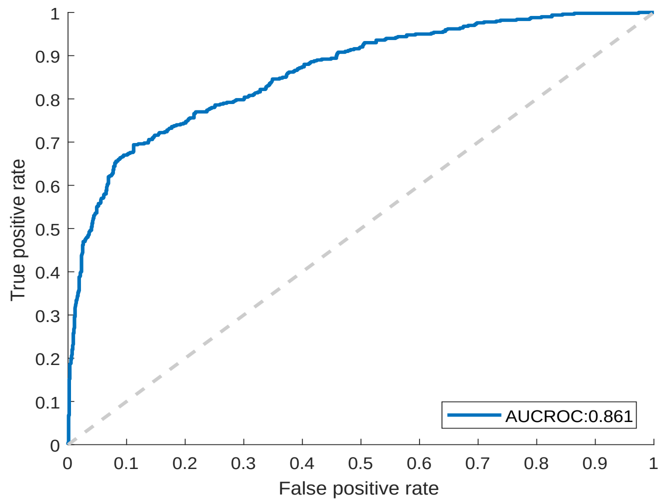

All peaks

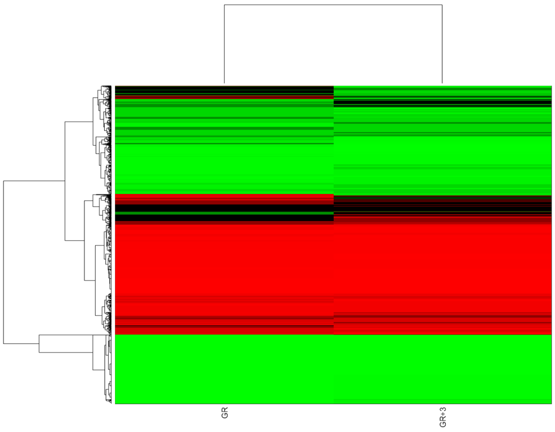

Top 500 peaks

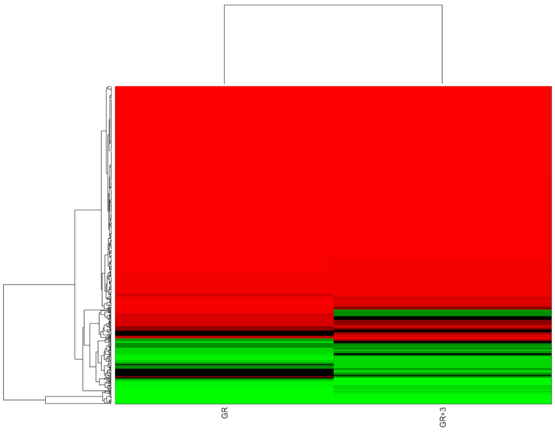

20 ECC-1 ERAA treatment=BPA 100nM

ESR1+5 All peaks

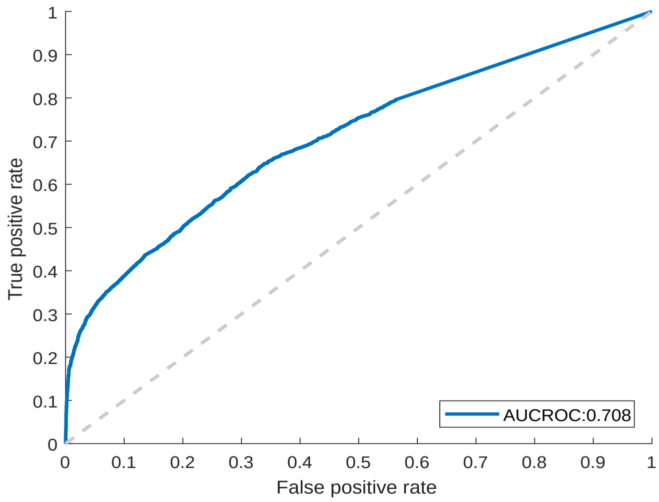

ESR1+5 Top 500 peaks

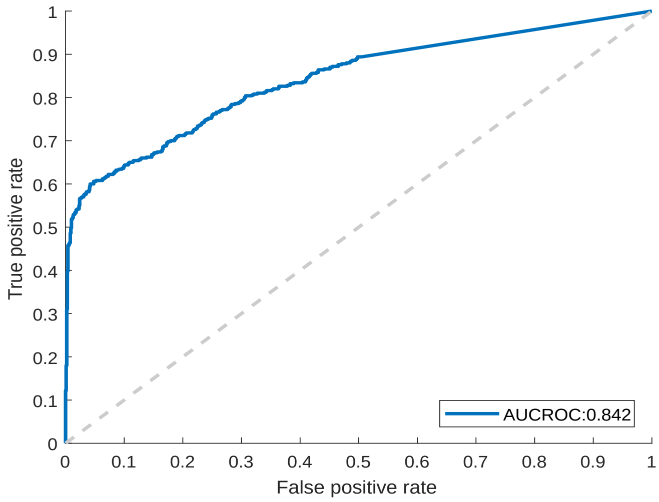

ESR1+6 All peaks

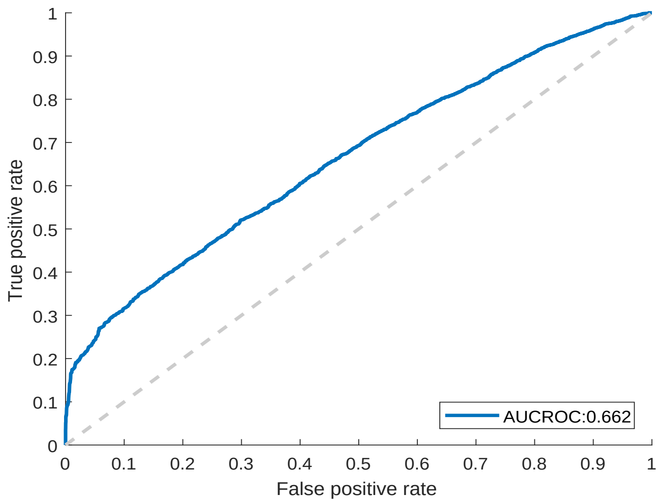

ESR1+6 Top 500 peaks

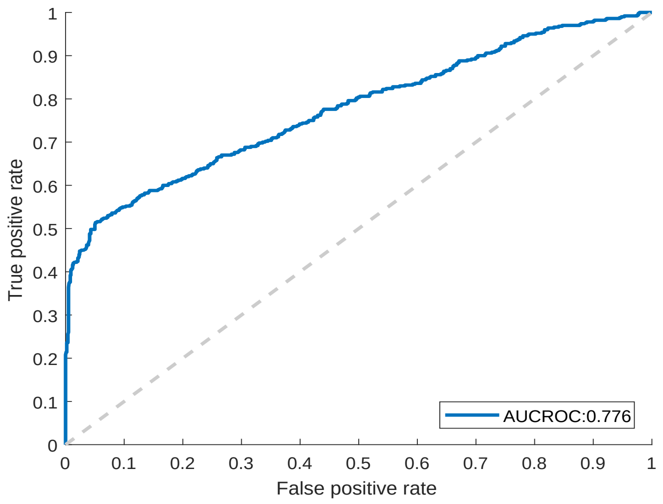

All peaks

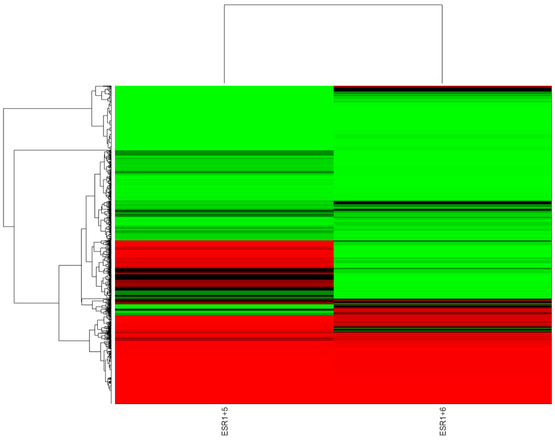

Top 500 peaks

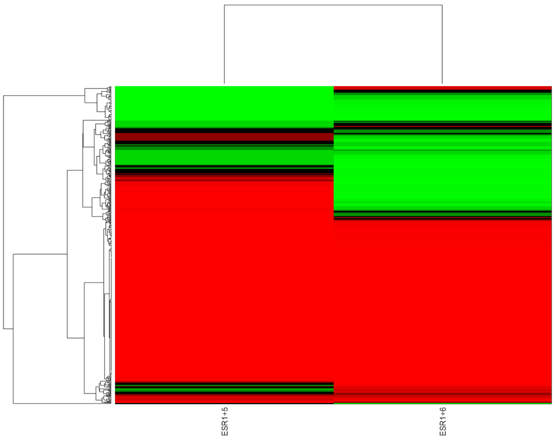

21 ECC-1 ERAA treatment=Estradiol 10nM

ESR1+5 All peaks

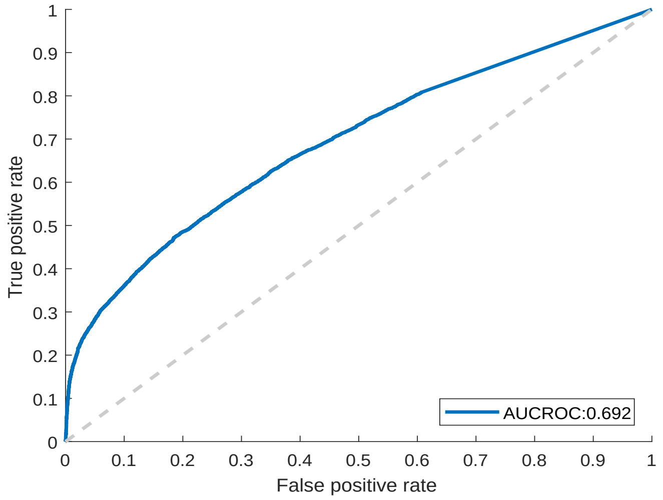

ESR1+5 Top 500 peaks

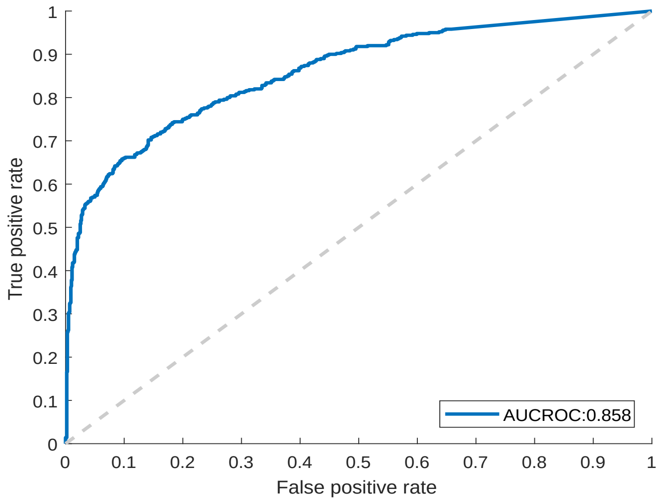

ESR1+6 All peaks

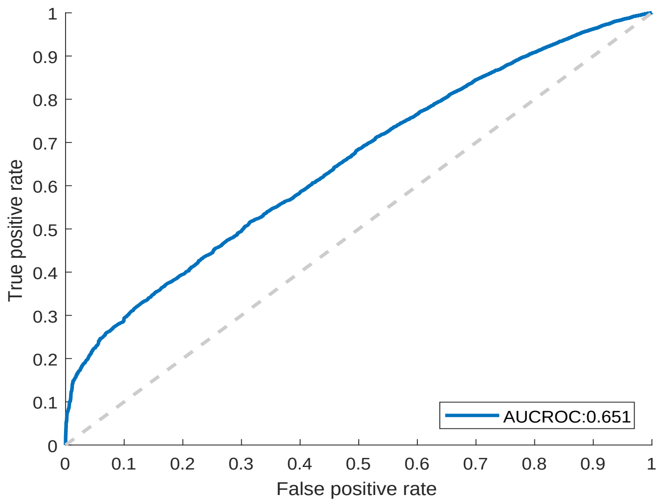

ESR1+6 Top 500 peaks

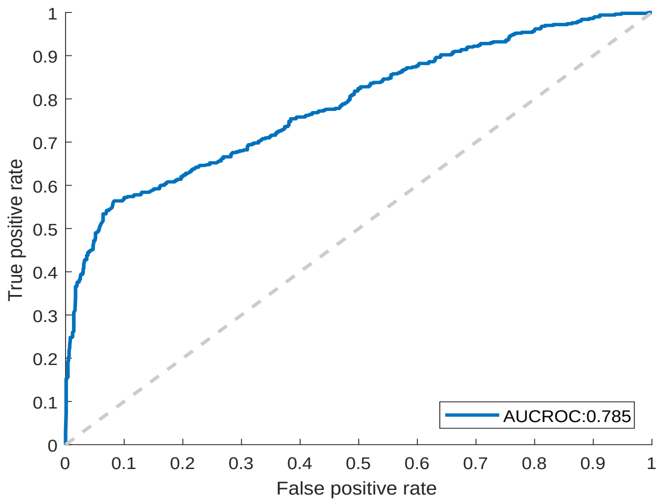

All peaks

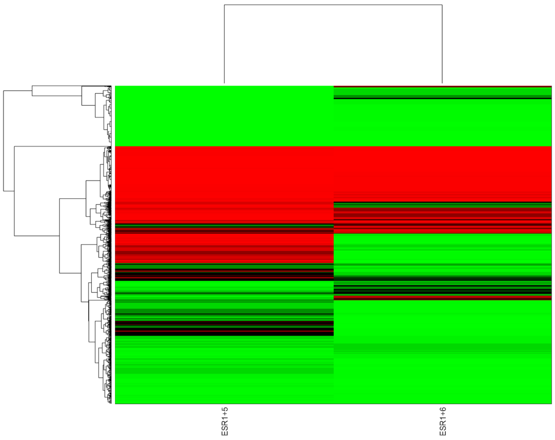

Top 500 peaks

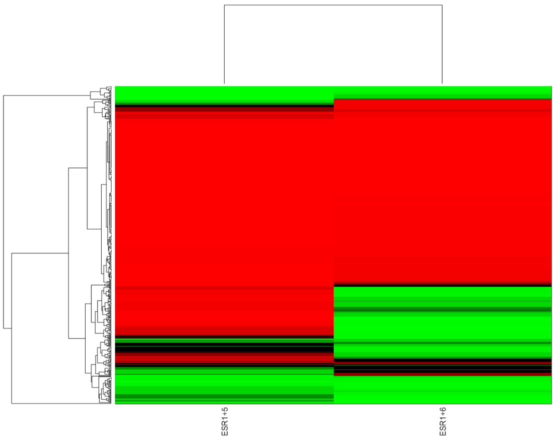

22 ECC-1 ERAA treatment=Genistein 100nM

ESR1+5 All peaks

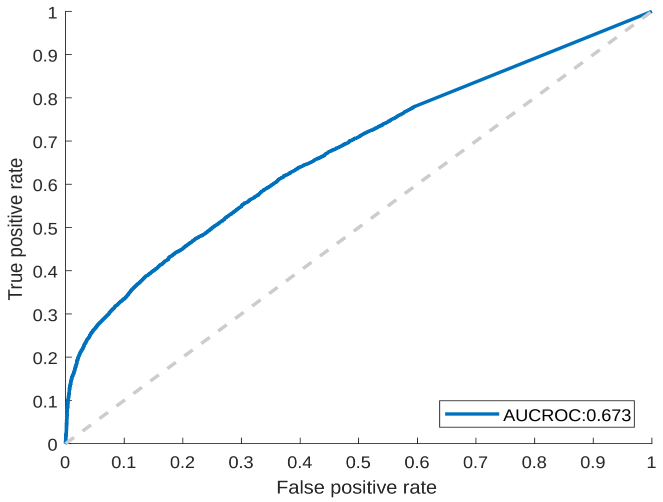

ESR1+5 Top 500 peaks

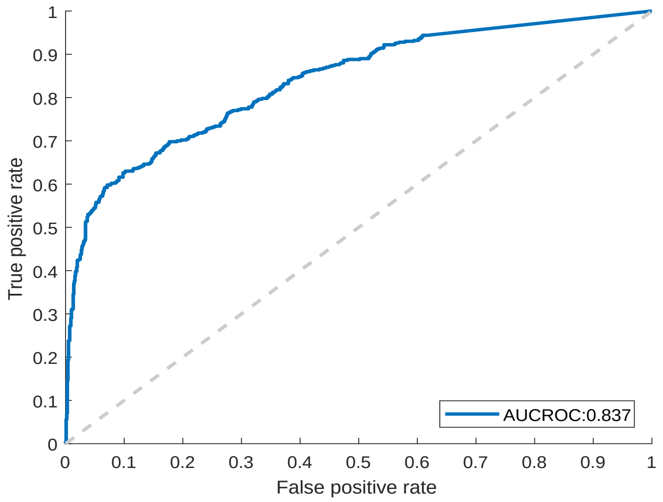

ESR1+6 All peaks

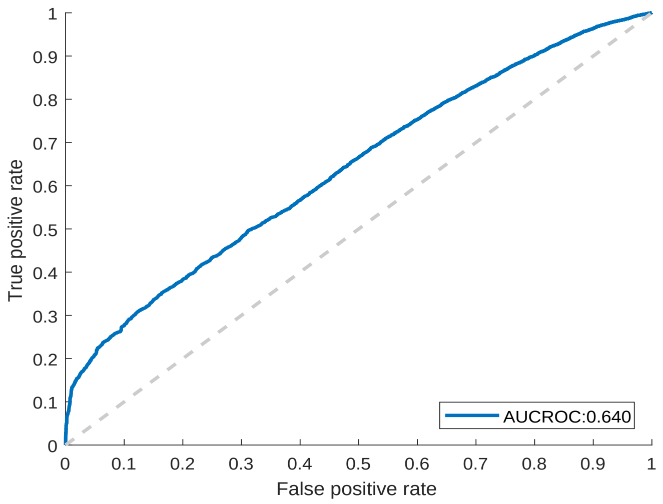

ESR1+6 Top 500 peaks

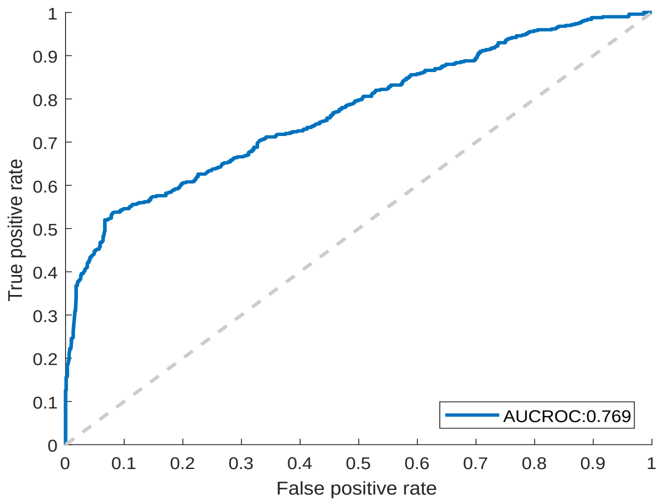

All peaks

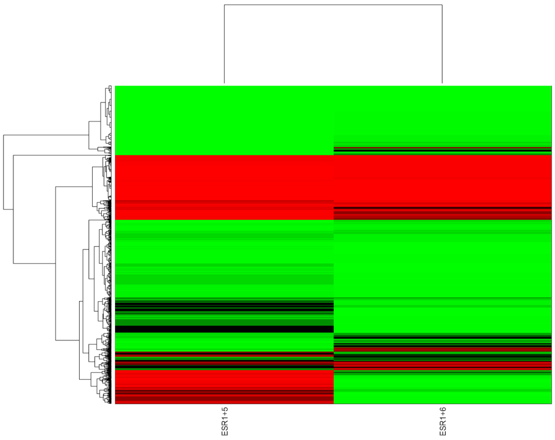

Top 500 peaks

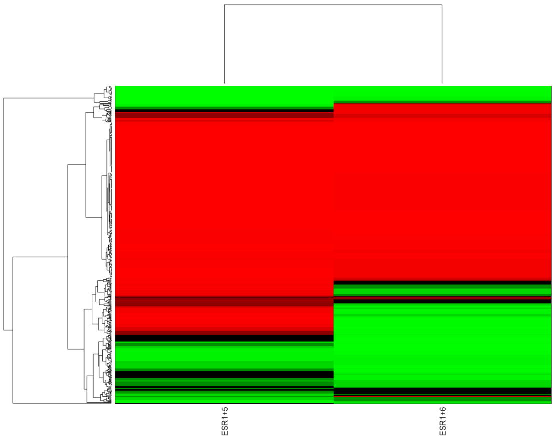

23 ECC-1 GR treatment=DEX 100nM

GR All peaks

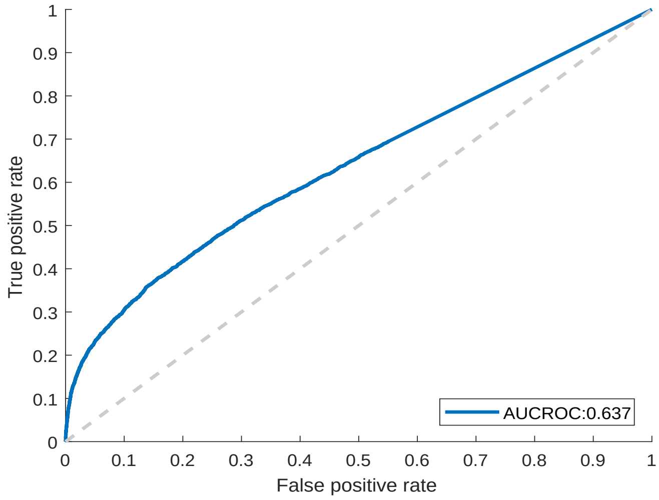

GR Top 500 peaks

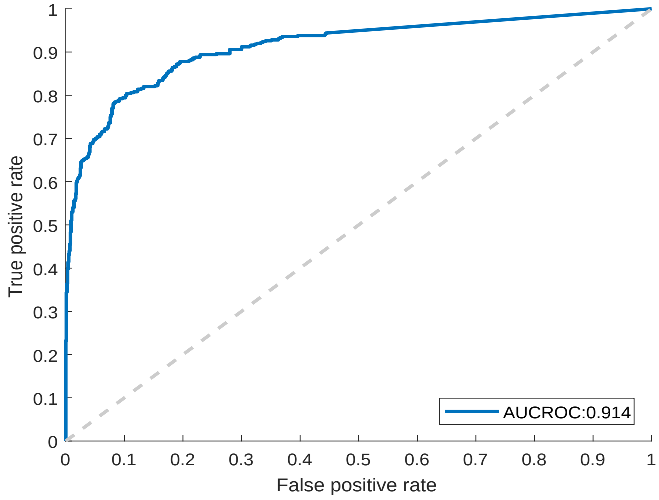

GR+3 All peaks

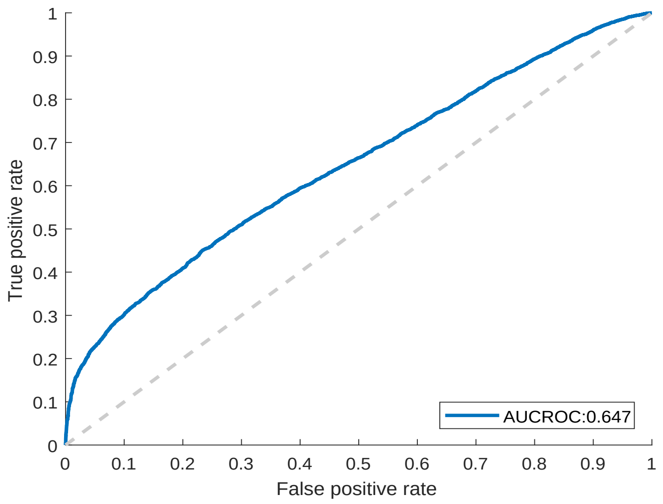

GR+3 Top 500 peaks

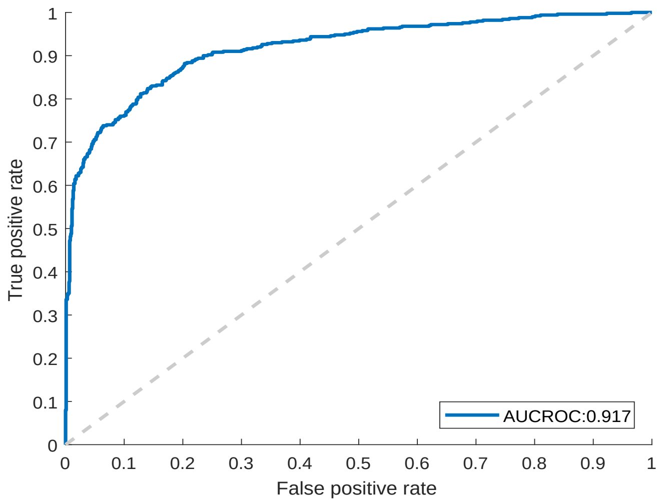

All peaks

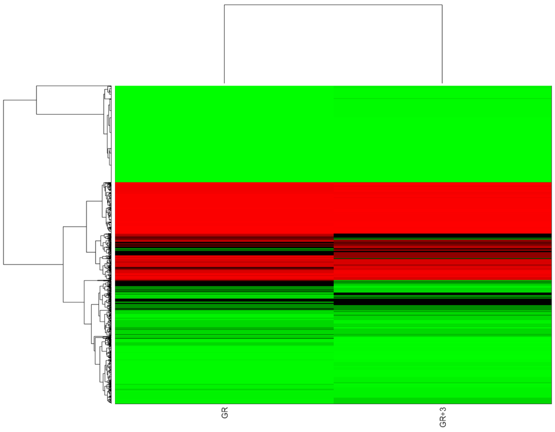

Top 500 peaks

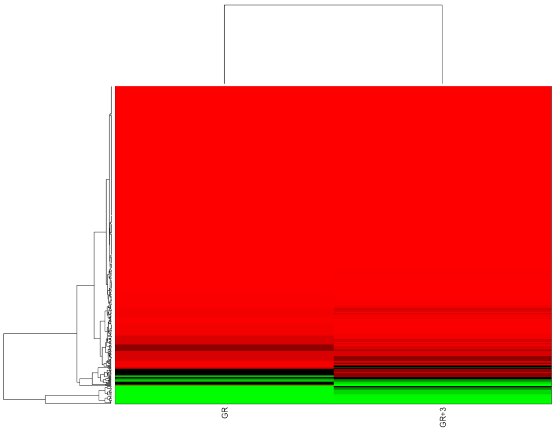

24 GM12878 RXRA

RXRA All peaks

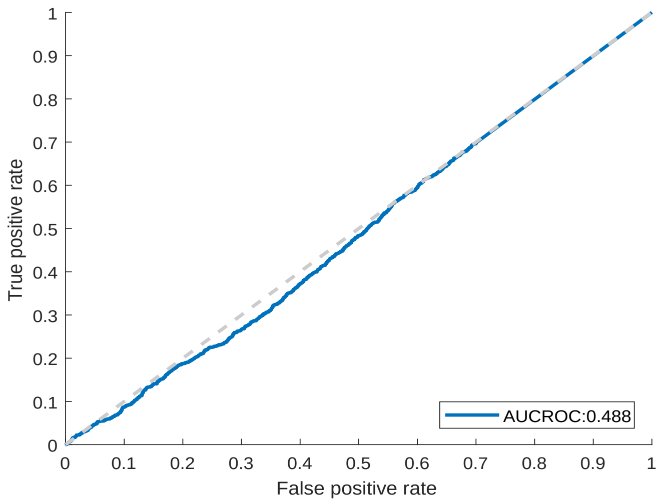

RXRA Top 500 peaks

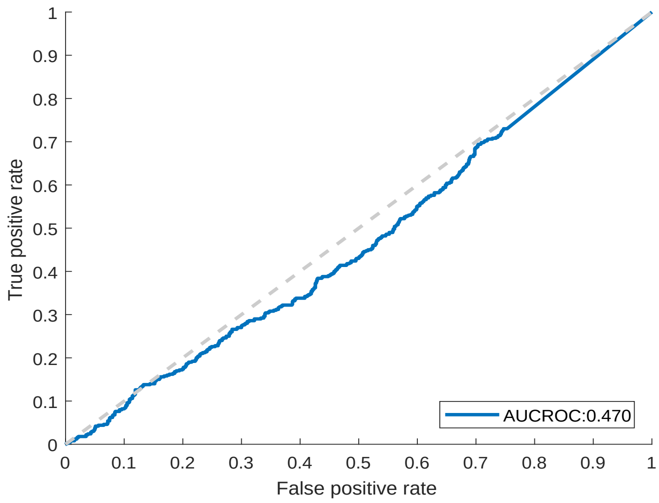

RXRA+17 All peaks

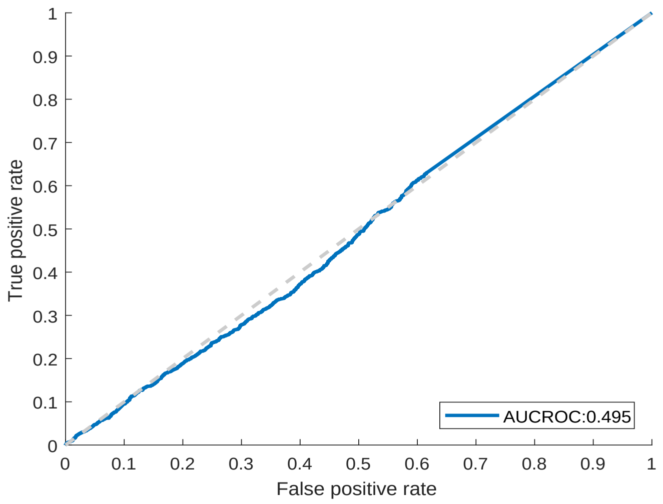

RXRA+17 Top 500 peaks

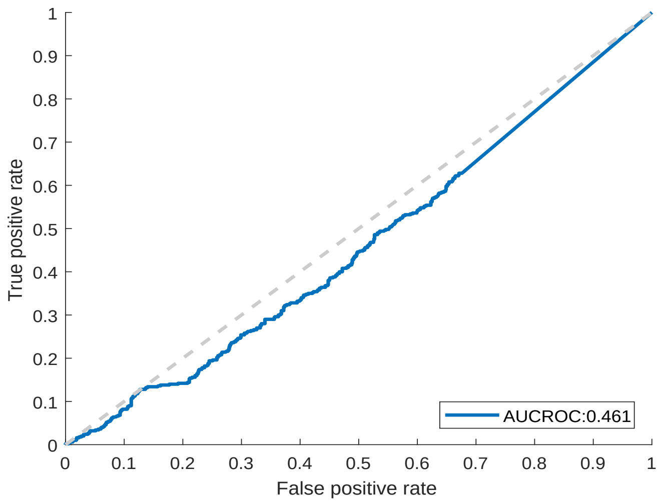

All peaks

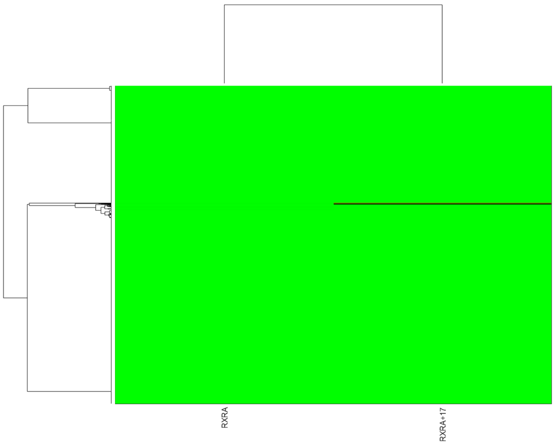

Top 500 peaks

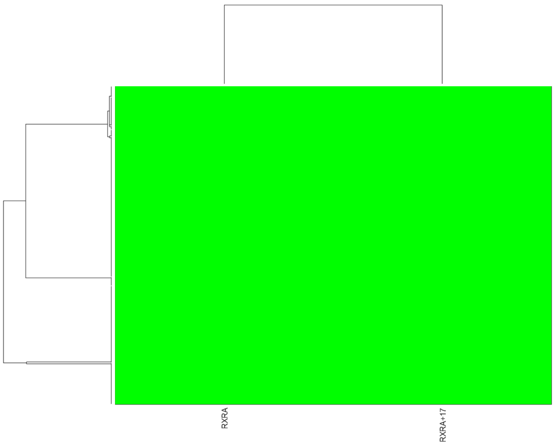

25 H1-hESC RXRA

RXRA All peaks

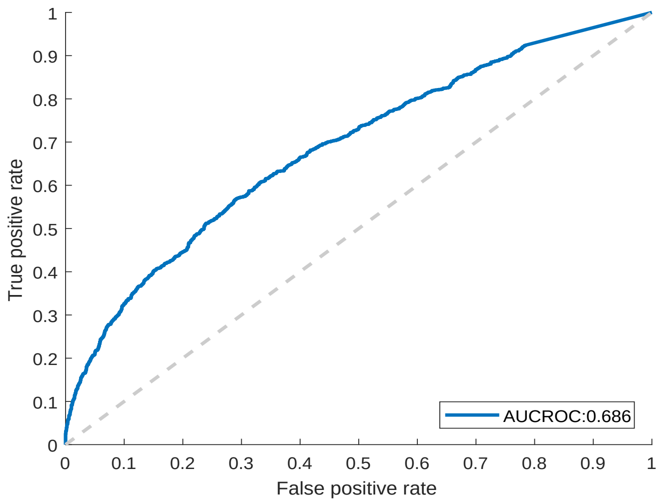

RXRA Top 500 peaks

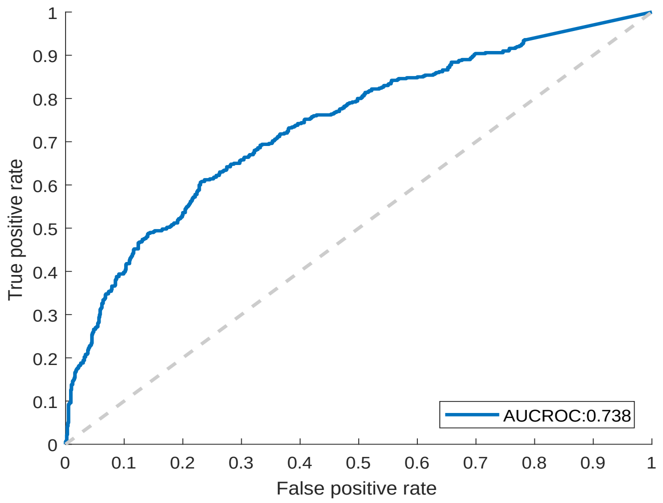

RXRA+17 All peaks

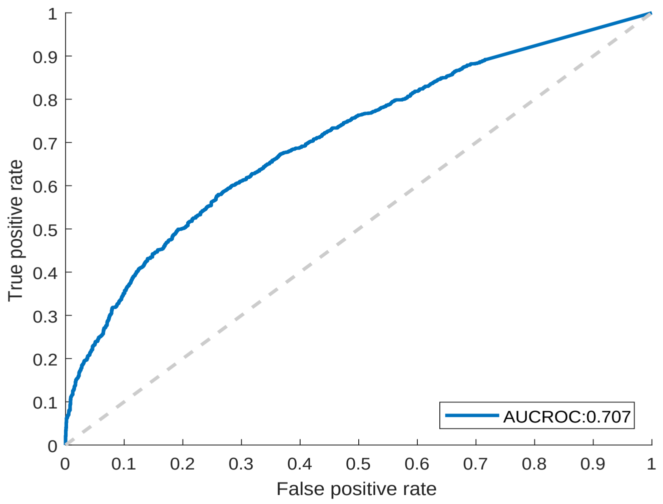

RXRA+17 Top 500 peaks

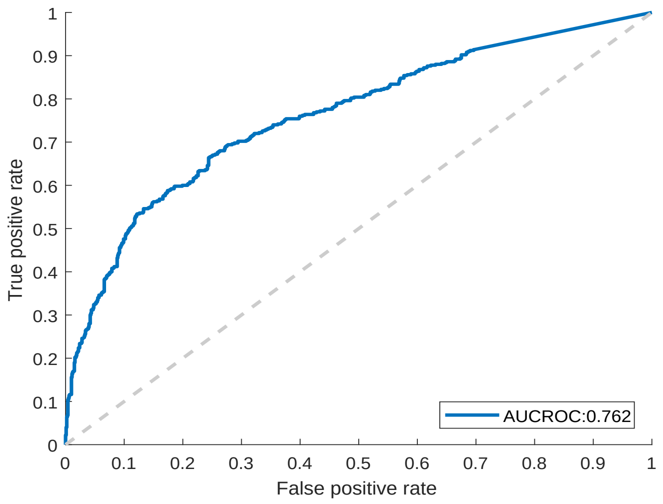

All peaks

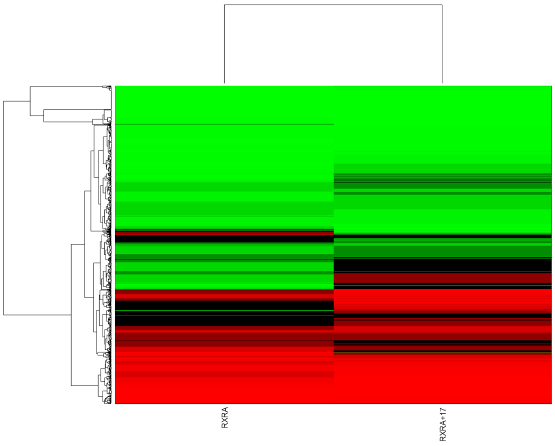

Top 500 peaks

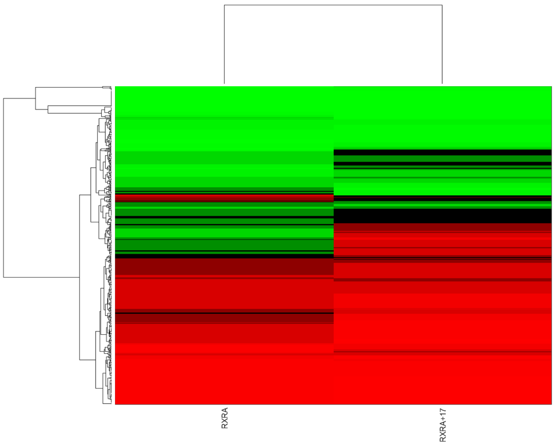

26 HepG2 HNF4A (SC-8987)

HNF4A All peaks

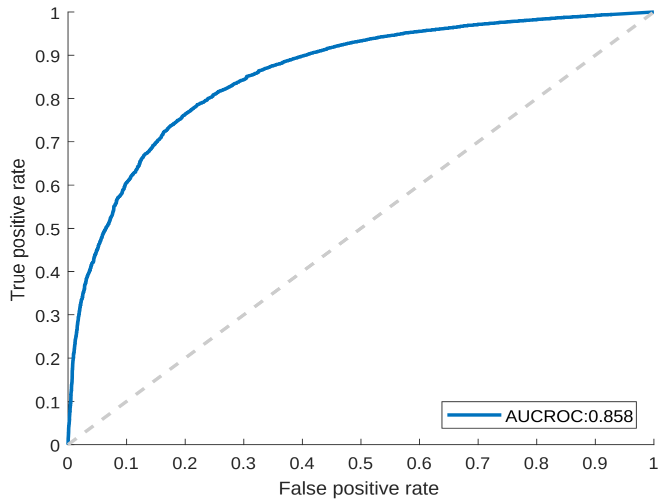

HNF4A Top 500 peaks

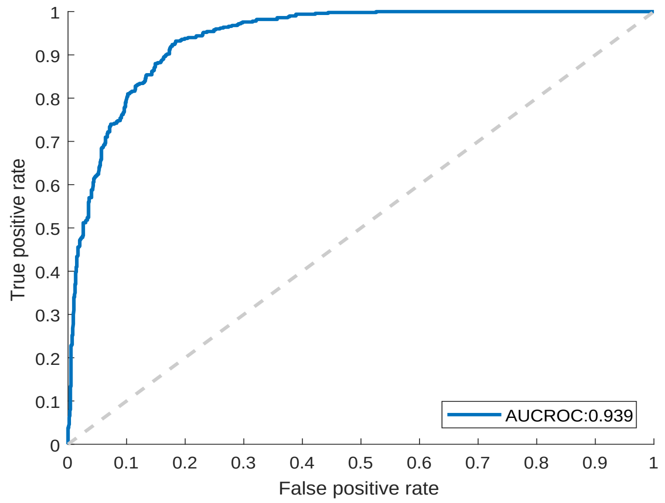

HNF4A+18 All peaks

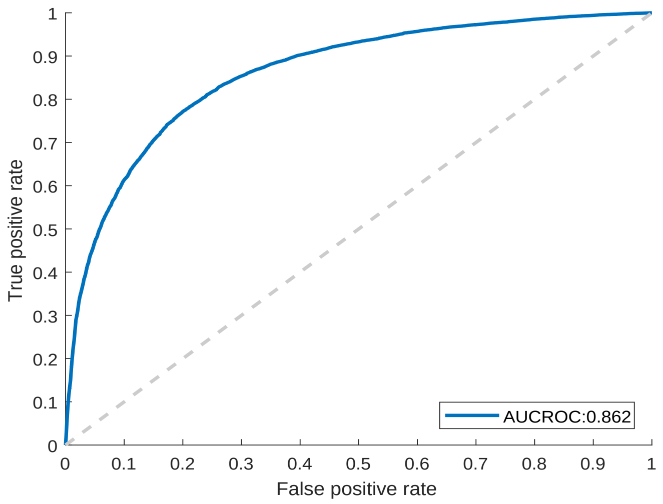

HNF4A+18 Top 500 peaks

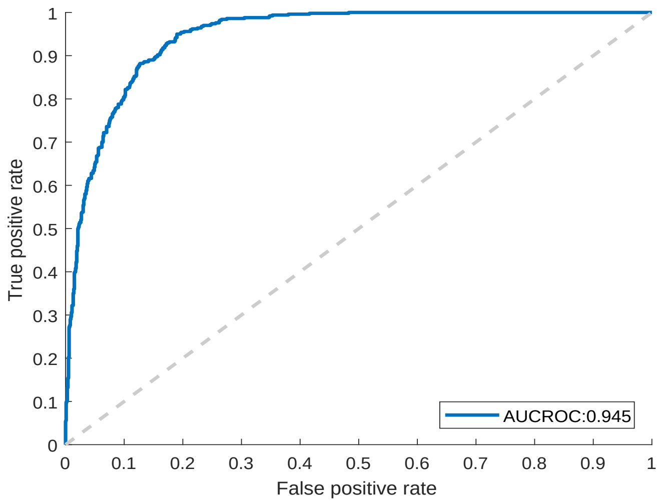

All peaks

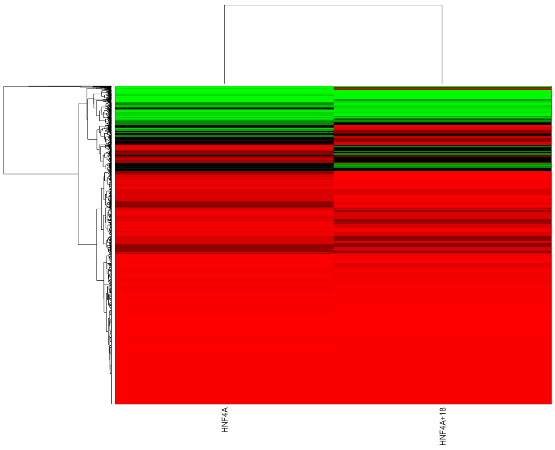

Top 500 peaks

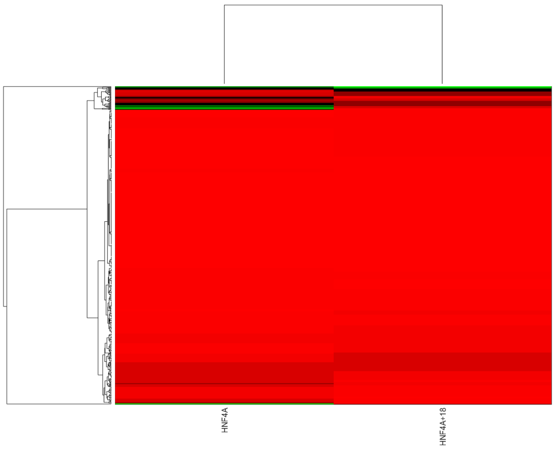

27 HepG2 HNF4G (SC-6558)

HNF4G All peaks

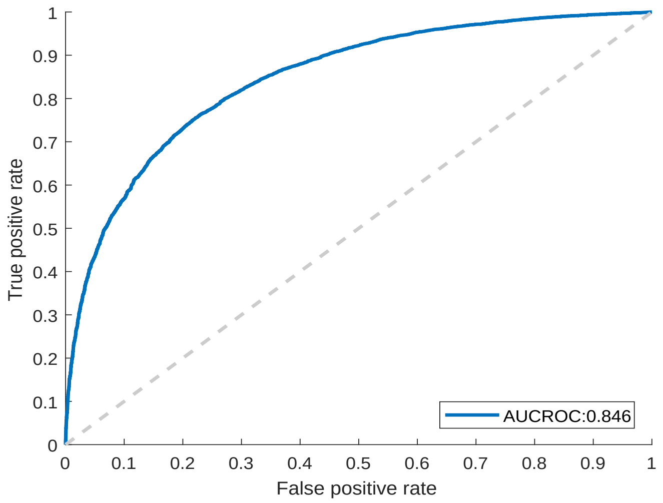

HNF4G Top 500 peaks

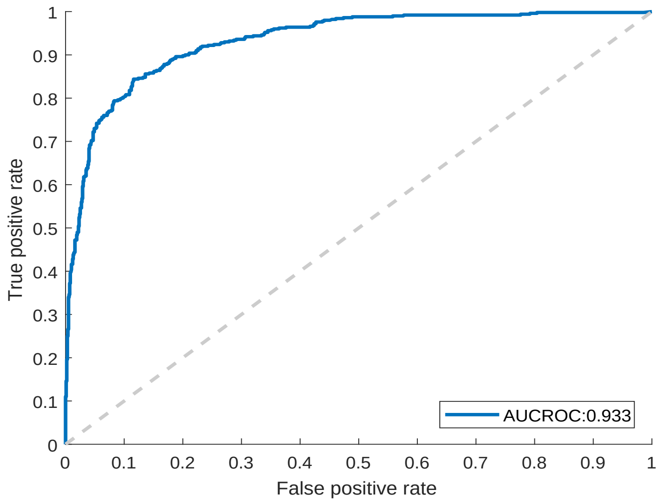

28 HepG2 RXRA

RXRA All peaks

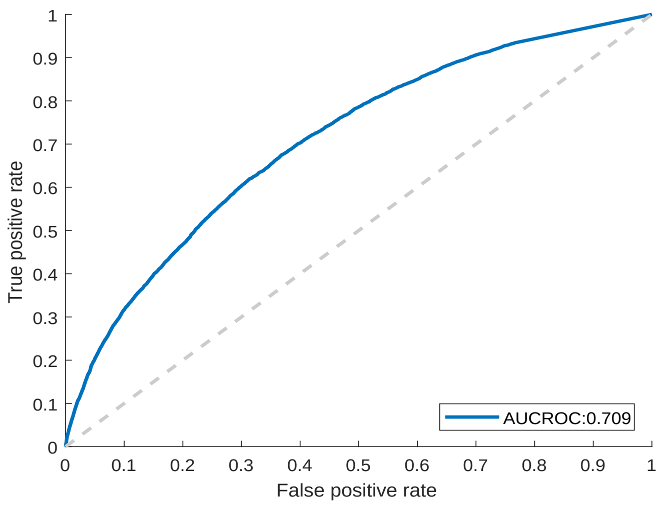

RXRA Top 500 peaks

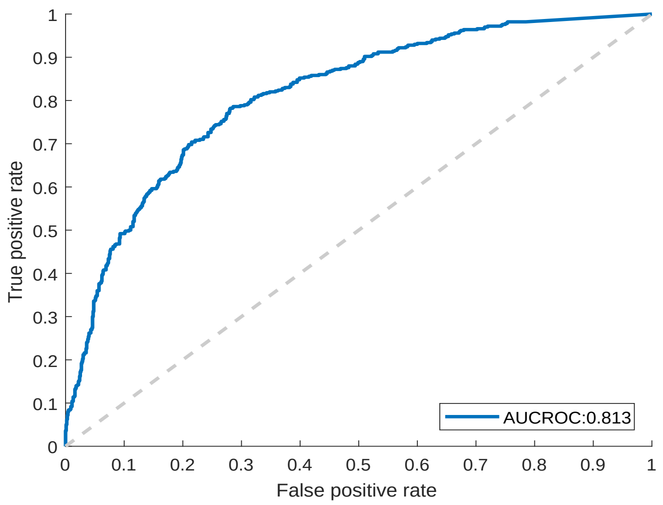

RXRA+17 All peaks

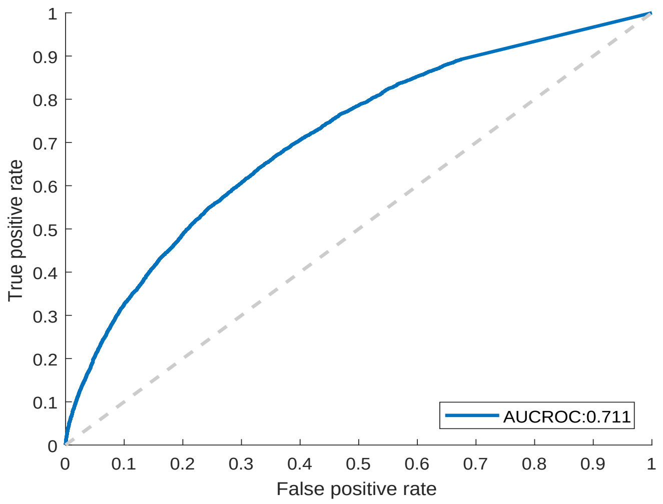

RXRA+17 Top 500 peaks

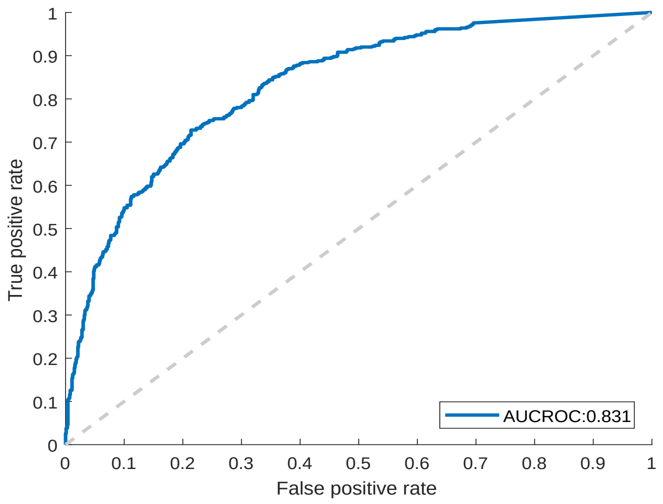

All peaks

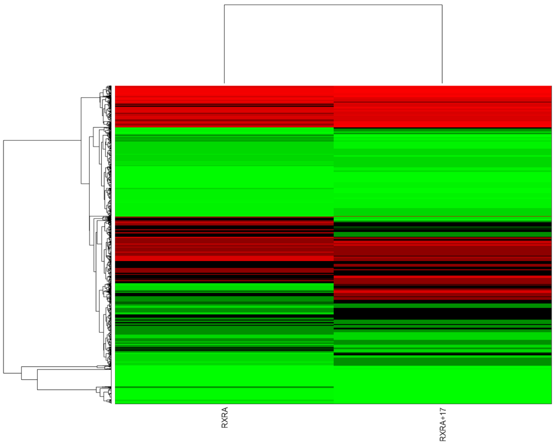

Top 500 peaks

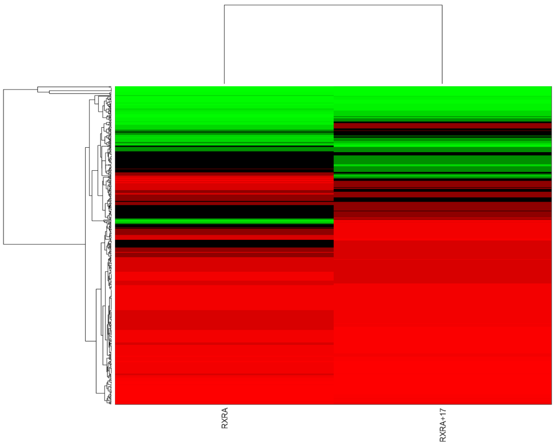

29 K562 NR2F2 (SC-271940)

COUP-TF2 All peaks

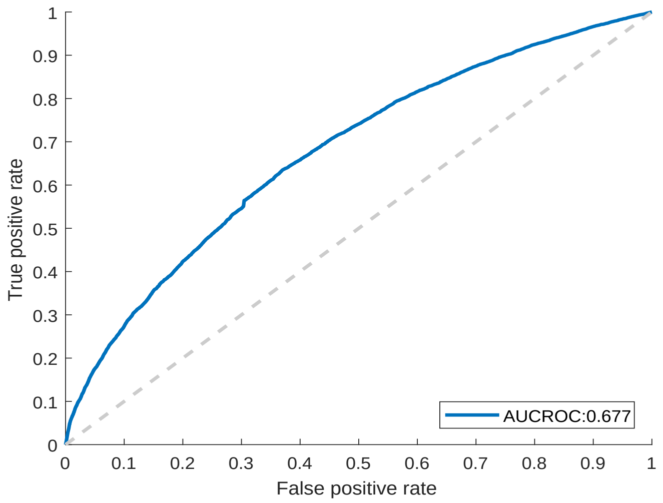

COUP-TF2 Top 500 peaks

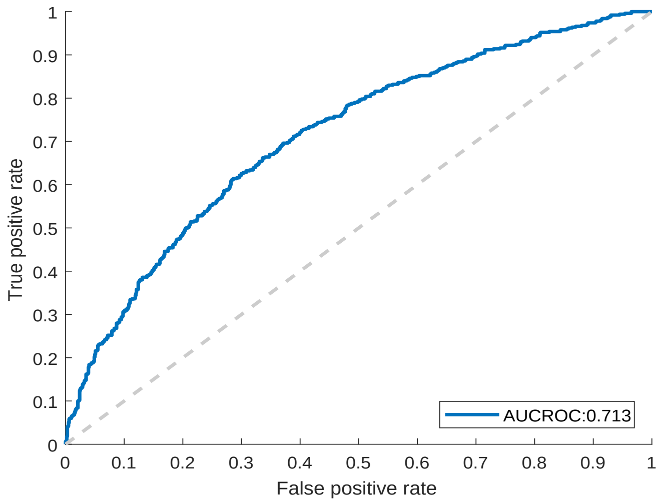

COUP-TF2+17 All peaks

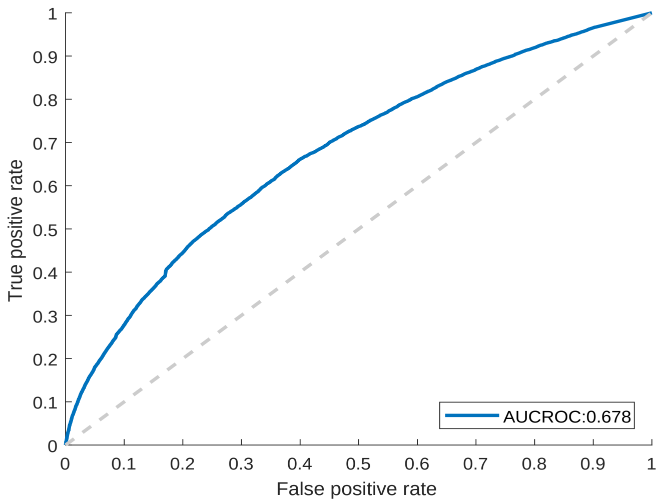

COUP-TF2+17 Top 500 peaks

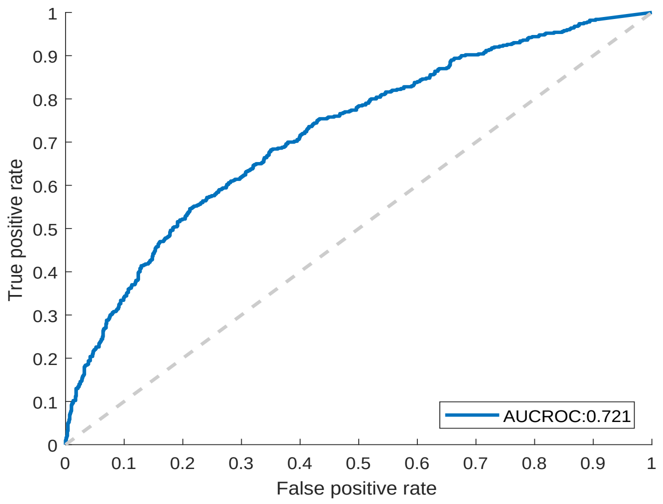

COUP-TF2:RXRA All peaks

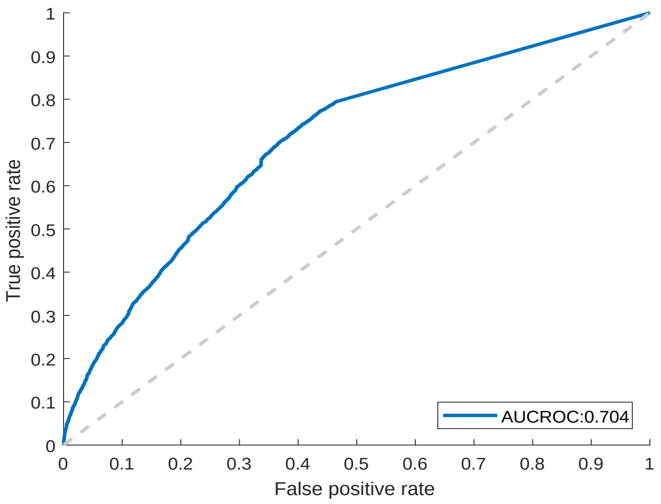

COUP-TF2:RXRA Top 500 peaks

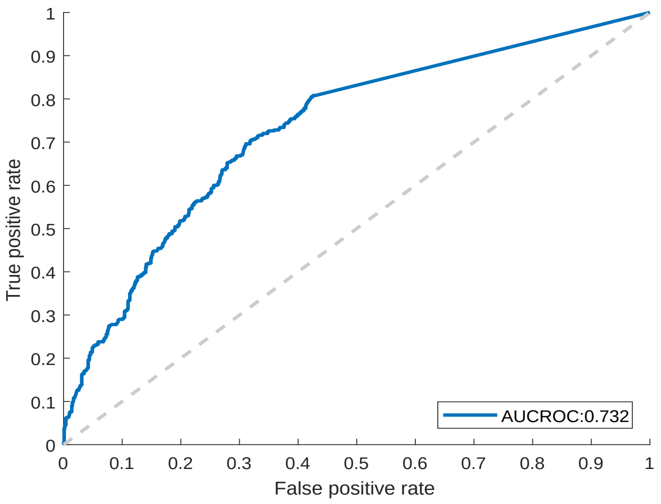

All peaks

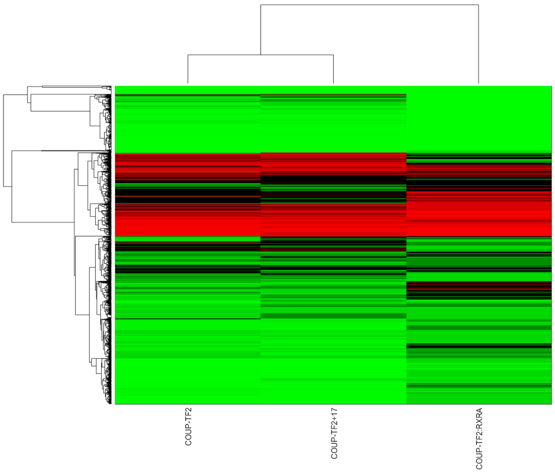

Top 500 peaks

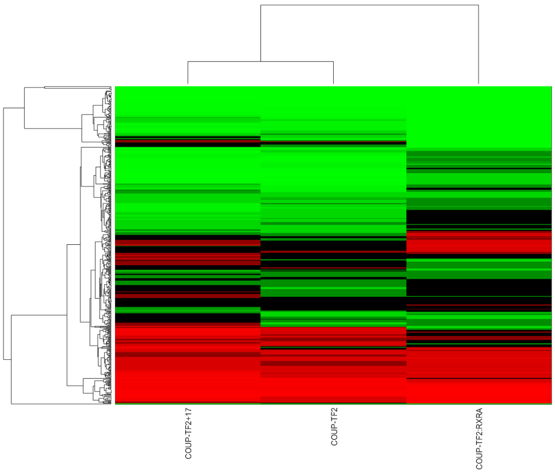

30 T-47D ERAA treatment=BPA 100nM

ESR1+5 All peaks

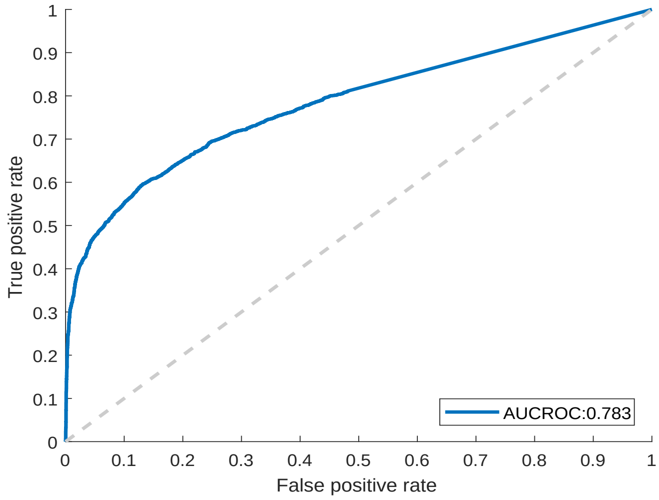

ESR1+5 Top 500 peaks

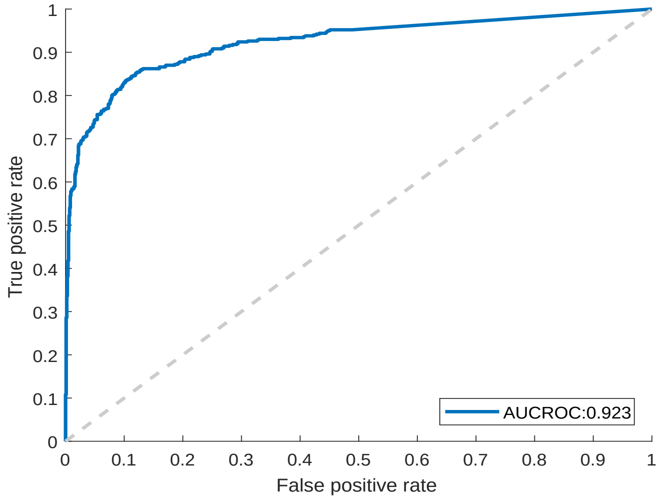

ESR1+6 All peaks

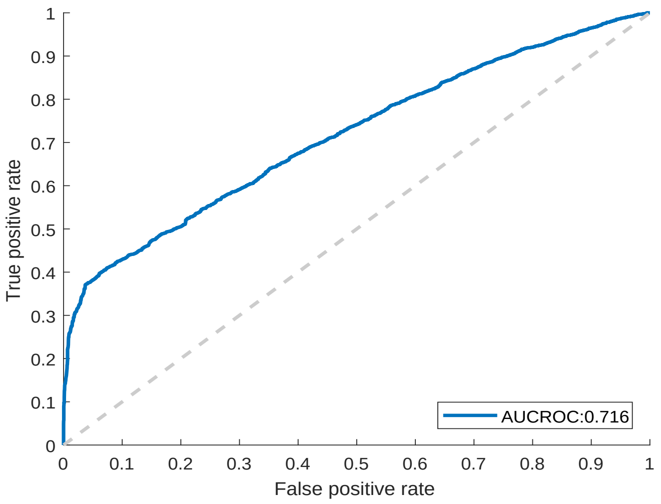

ESR1+6 Top 500 peaks

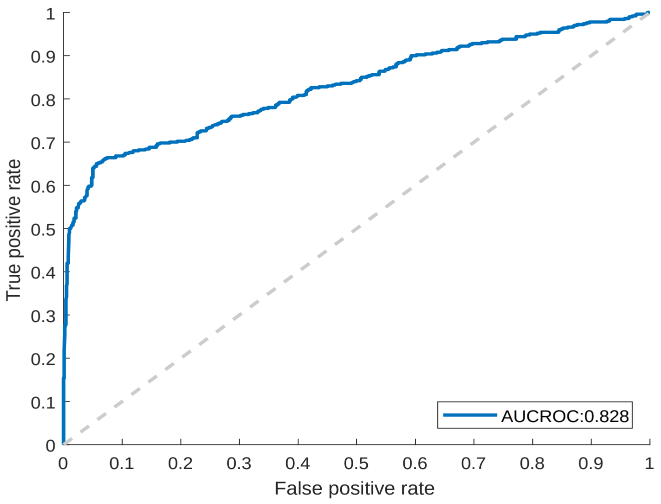

All peaks

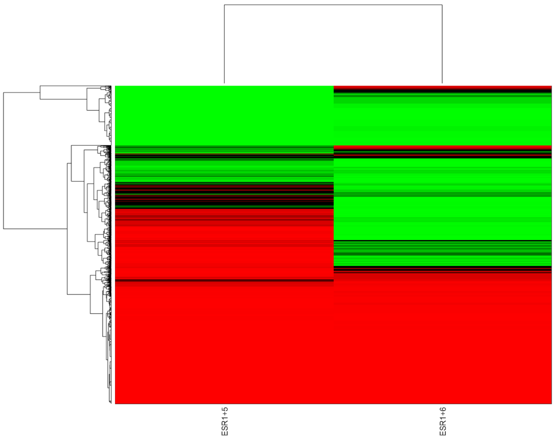

Top 500 peaks

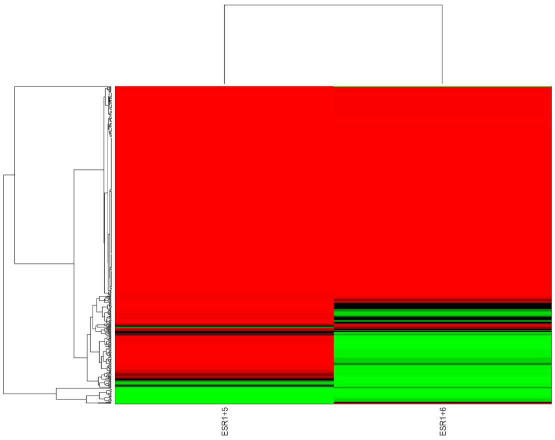

31 T-47D ERAA treatment=Genistein 100nM

ESR1+5 All peaks

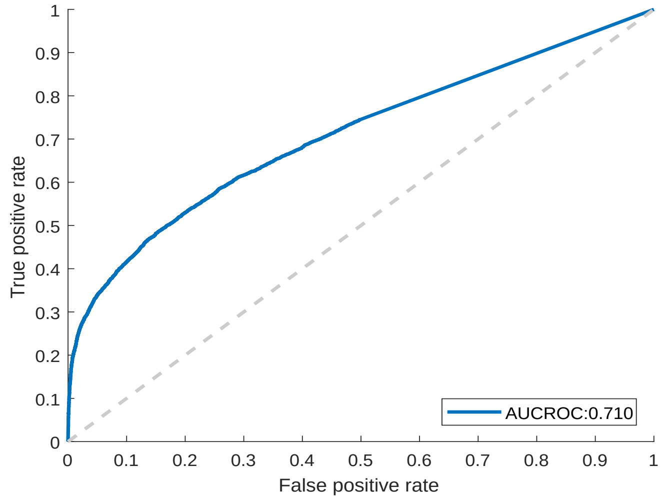

ESR1+5 Top 500 peaks

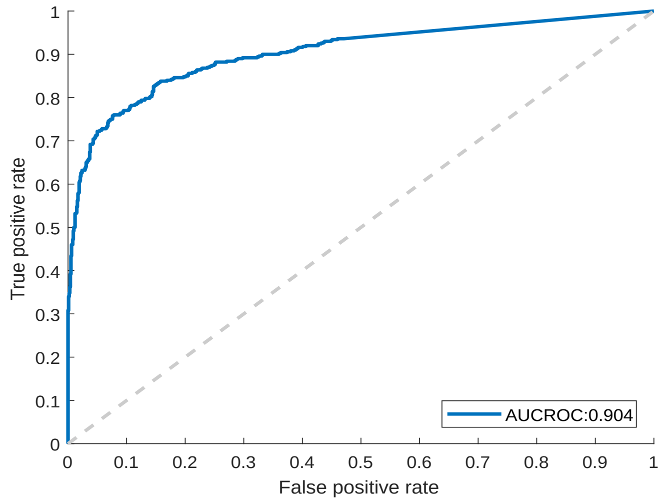

ESR1+6 All peaks

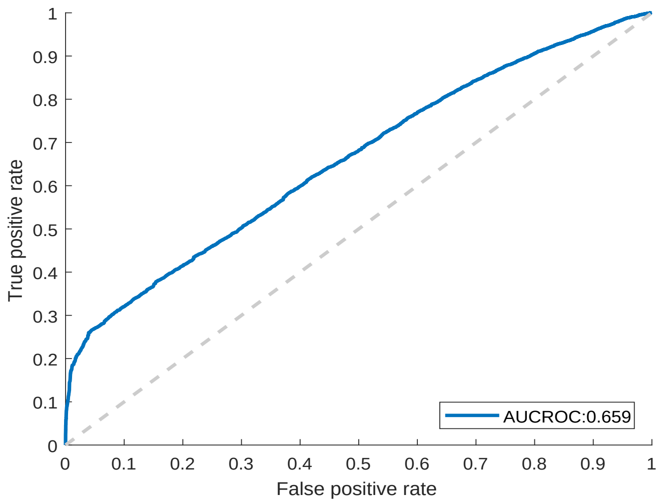

ESR1+6 Top 500 peaks

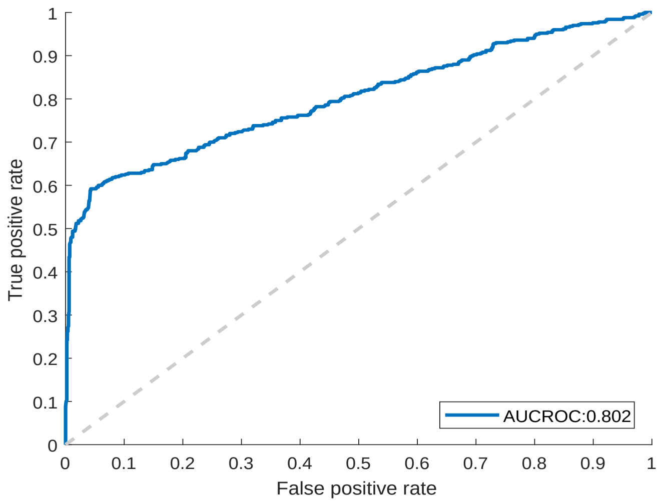

All peaks

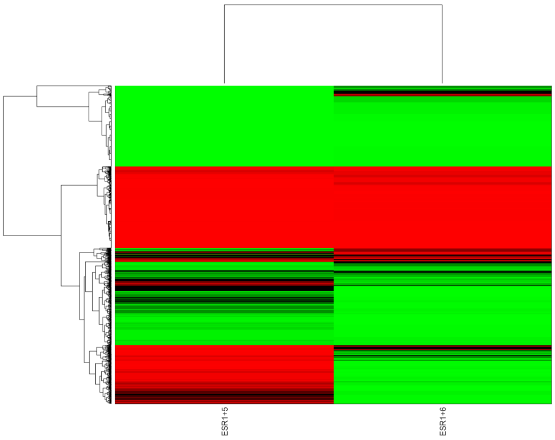

Top 500 peaks

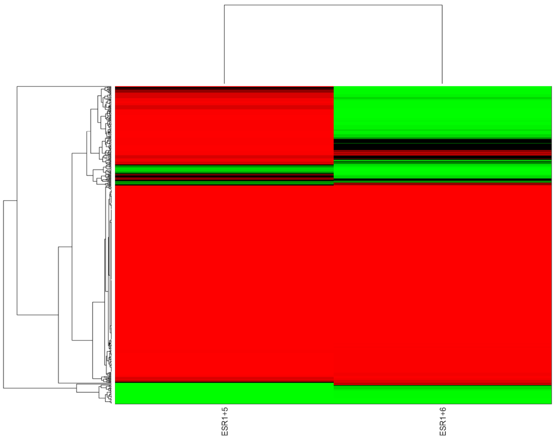

32 T-47D ERAA treatment=Estradiol 10nM

ESR1+5 All peaks

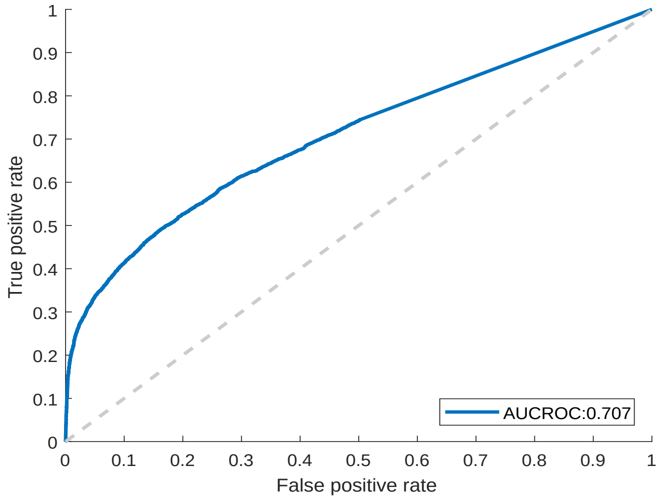

ESR1+5 Top 500 peaks

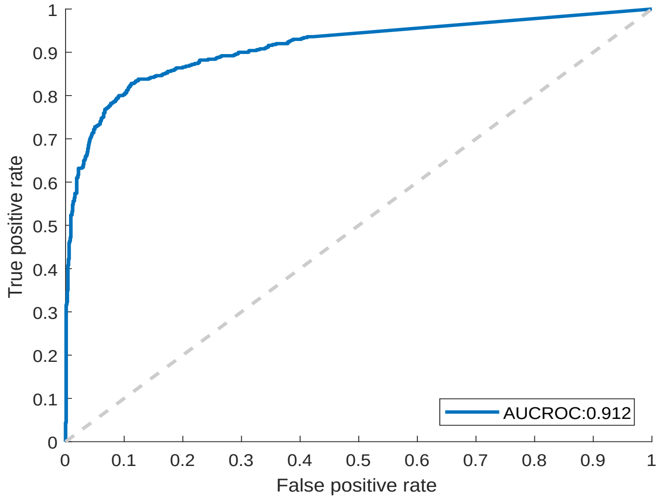

ESR1+6 All peaks

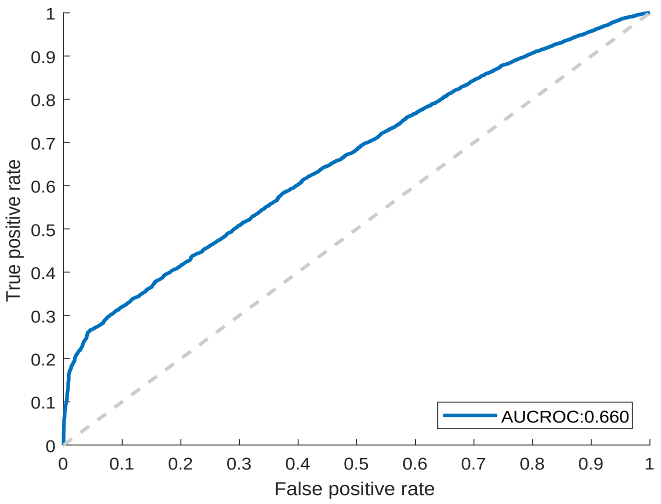

ESR1+6 Top 500 peaks

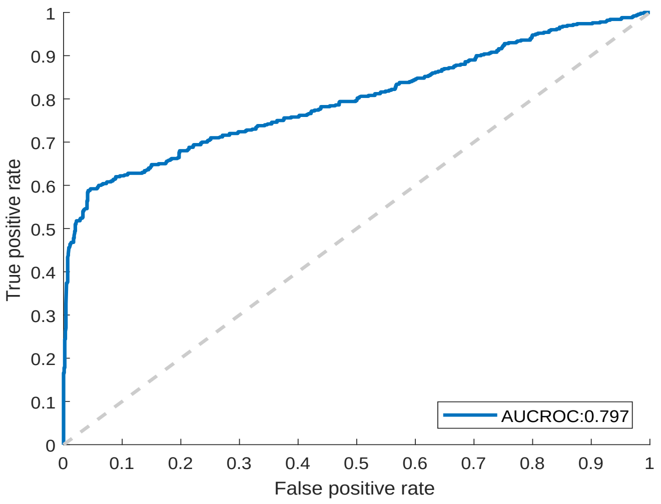

All peaks

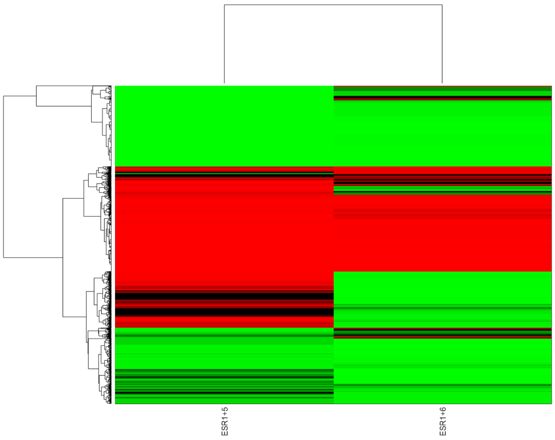

Top 500 peaks

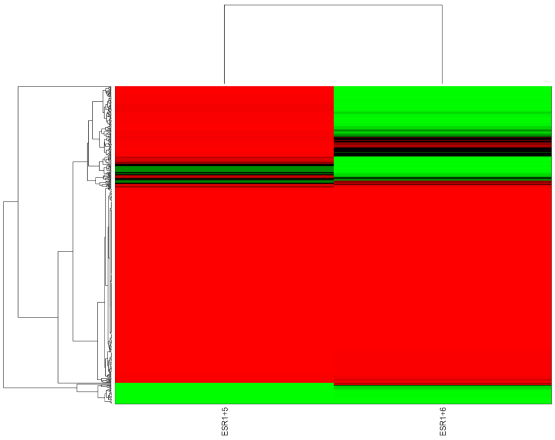

33 GM12878 TR4

TR4 All peaks

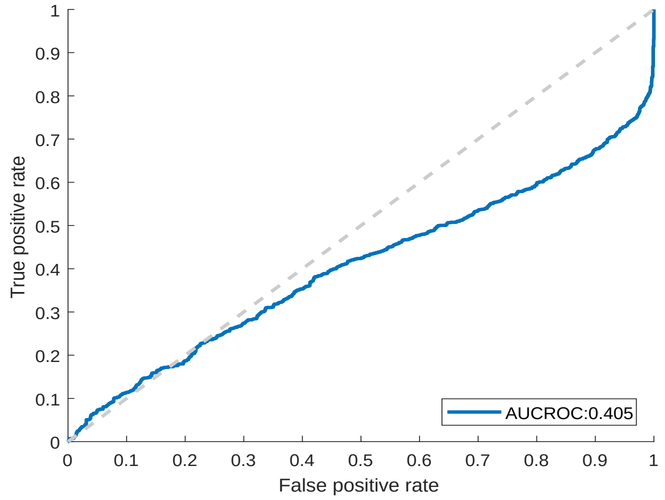

TR4 Top 500 peaks

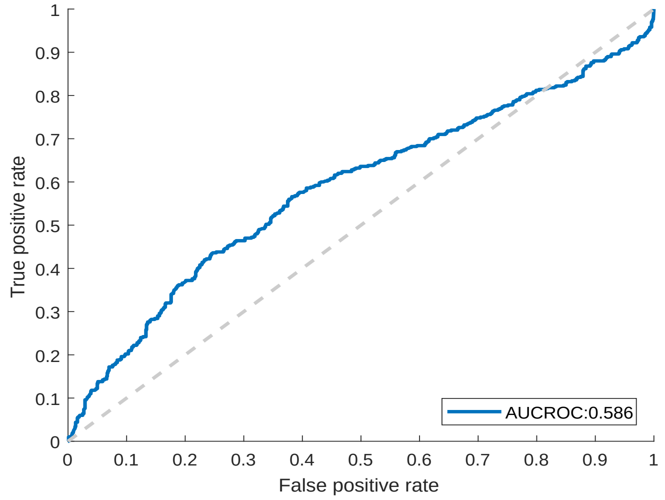

34 HeLa-S3 TR4

TR4 All peaks

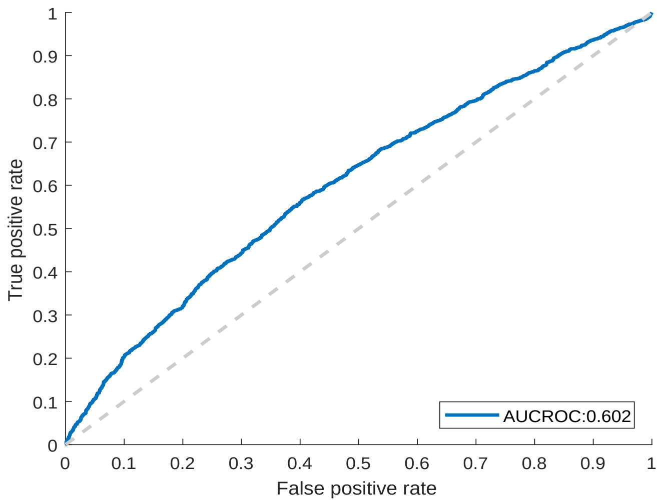

TR4 Top 500 peaks

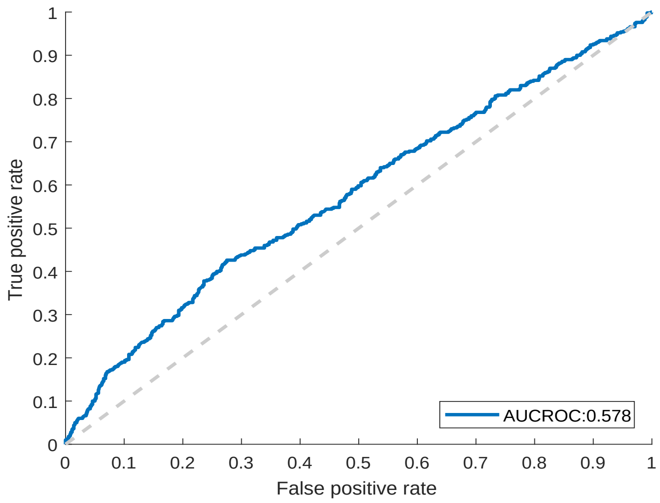

35 HepG2 ERRA treatment=forskolin

ESRRA All peaks

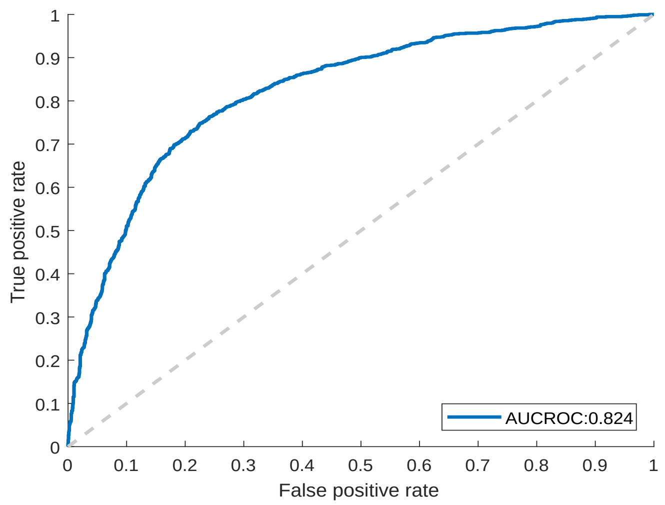

ESRRA Top 500 peaks

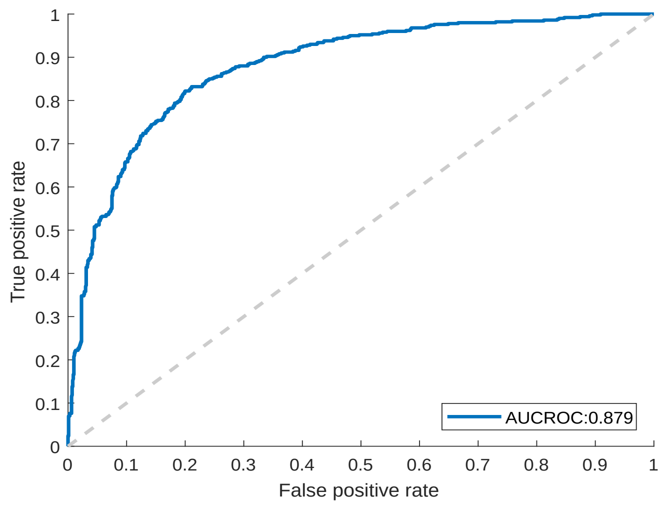

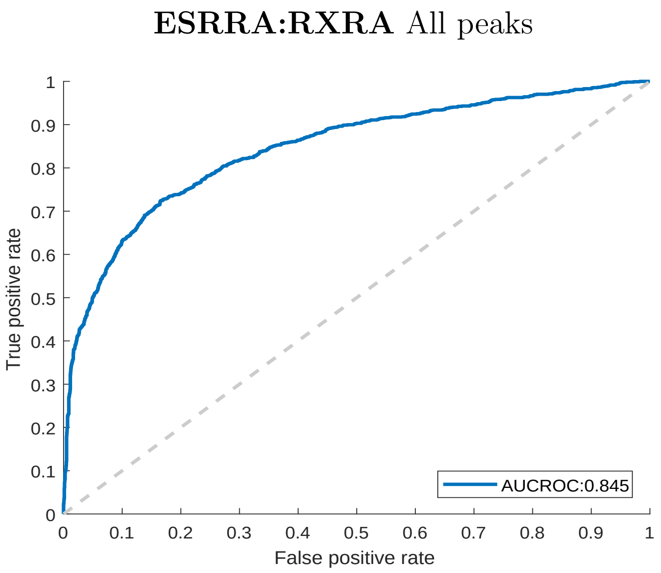
ESRRA:RXRA All peaks
True positive rate
False positive rate
AUCROC:0.845
0
0.1
0.2
0.3
0.4
0.5
0.6
0.7
0.8
0.9
1

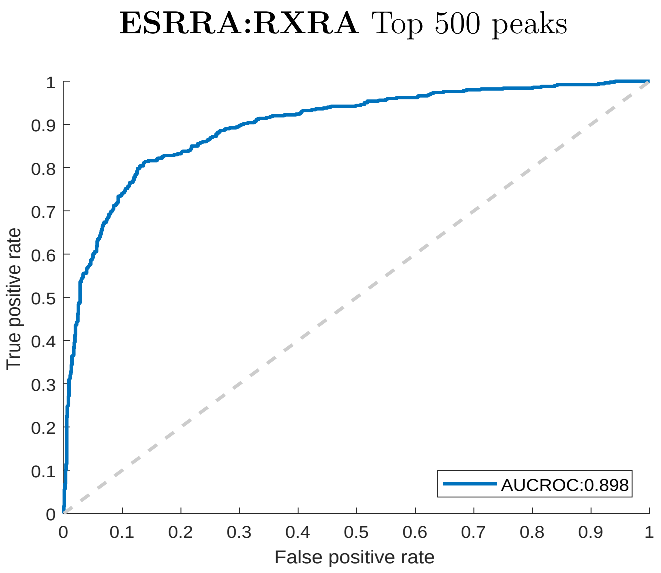
ESRRA:RXRA Top 500 peaks
True positive rate
False positive rate
AUCROC:0.898
0
0.1
0.2
0.3
0.4
0.5
0.6
0.7
0.8
0.9
1

ESRRA+4 All peaks

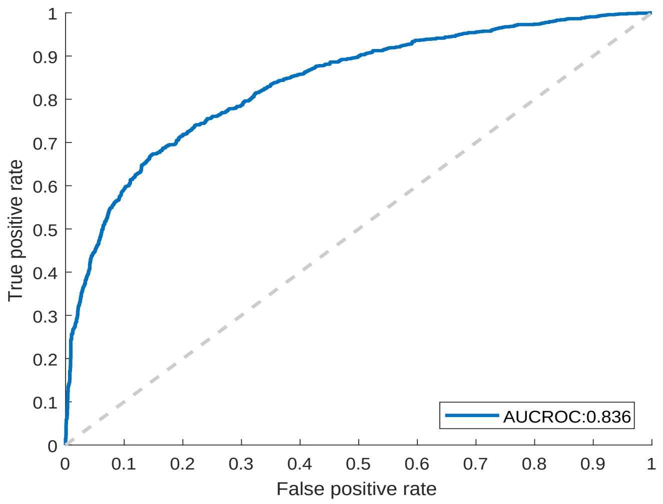

ESRRA+4 Top 500 peaks

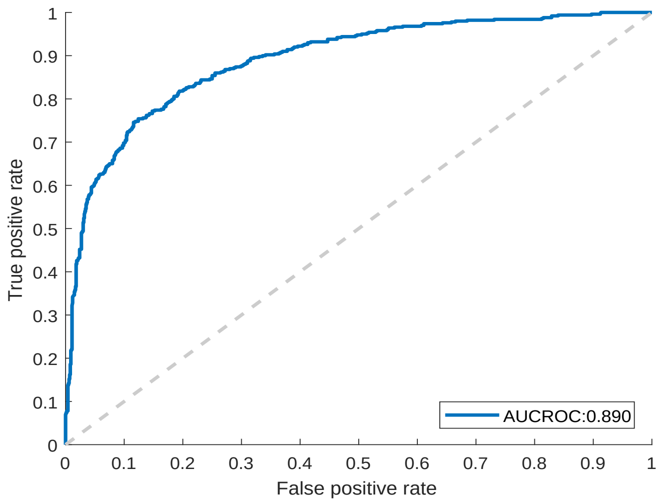

All peaks

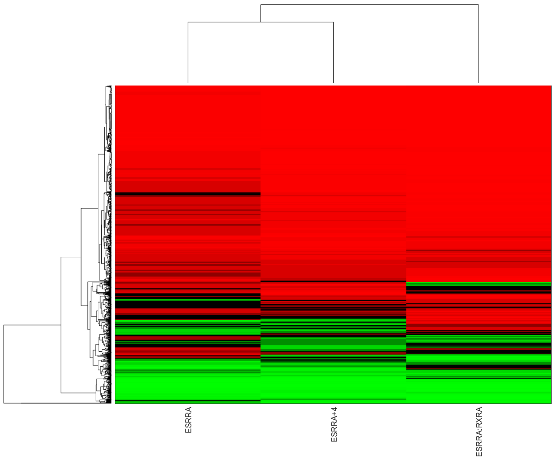

Top 500 peaks

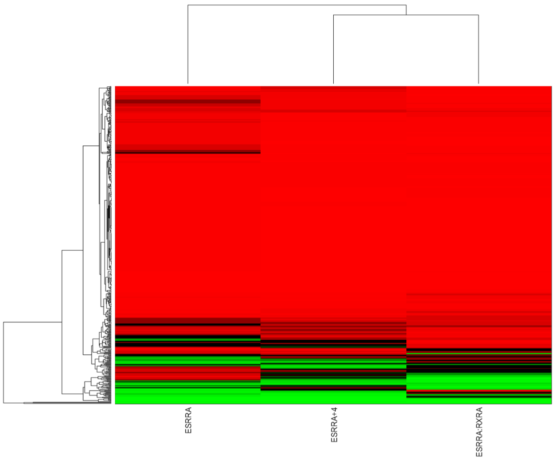

36 HepG2 HNF4A treatment=forskolin

HNF4A All peaks

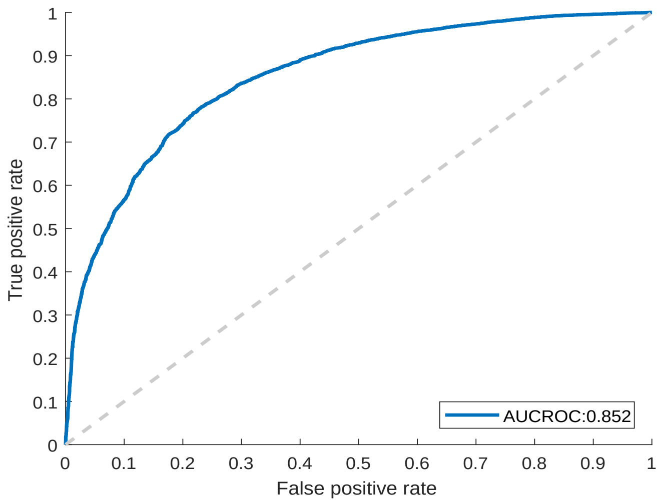

HNF4A Top 500 peaks

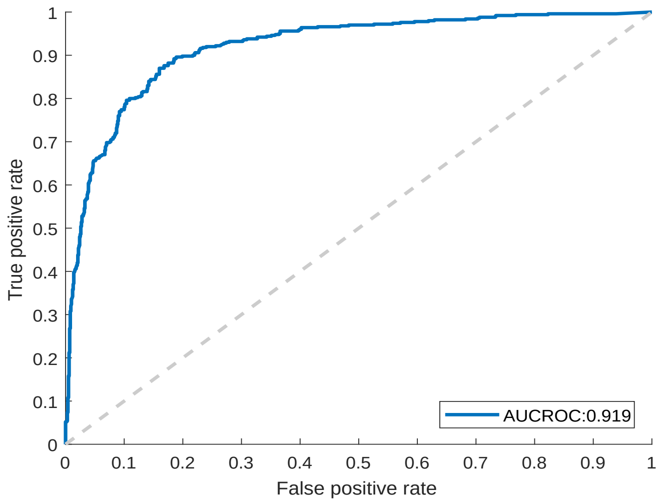

HNF4A+18 All peaks

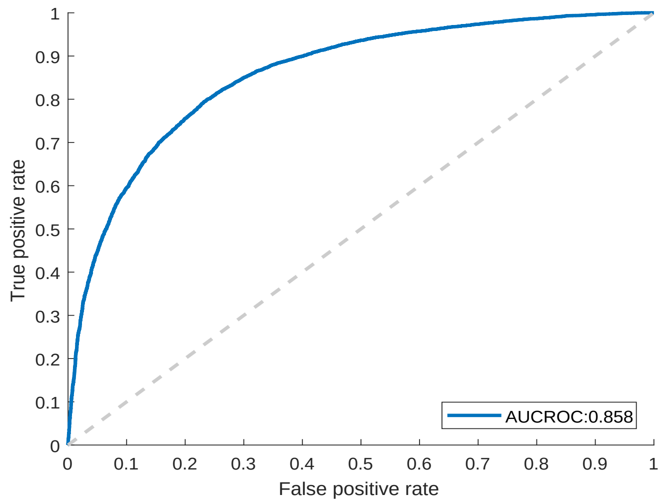

HNF4A+18 Top 500 peaks

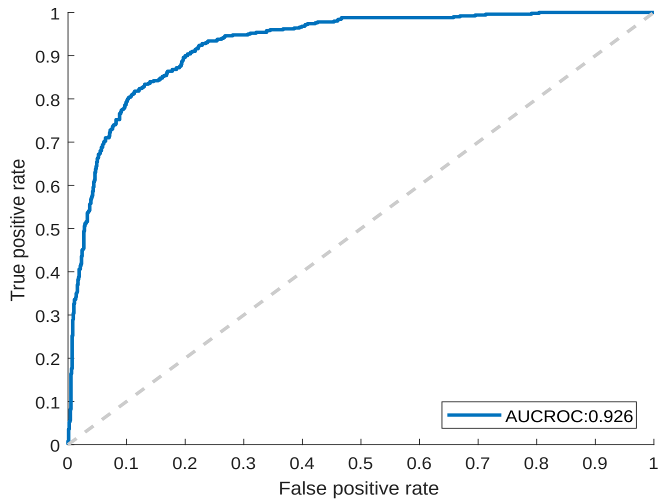

All peaks

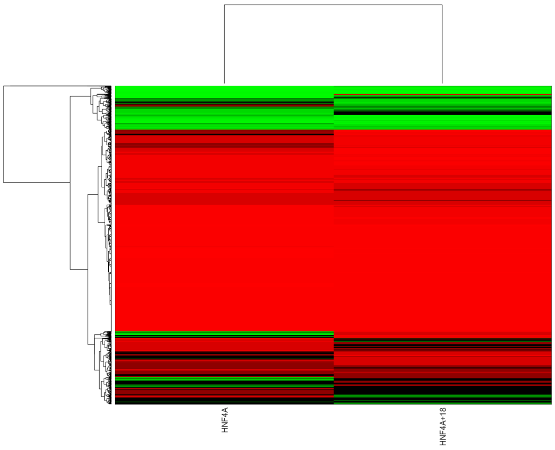

Top 500 peaks

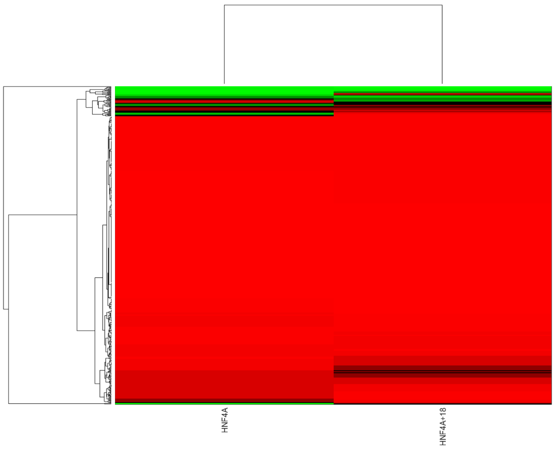

37 HepG2 TR4

TR4 All peaks

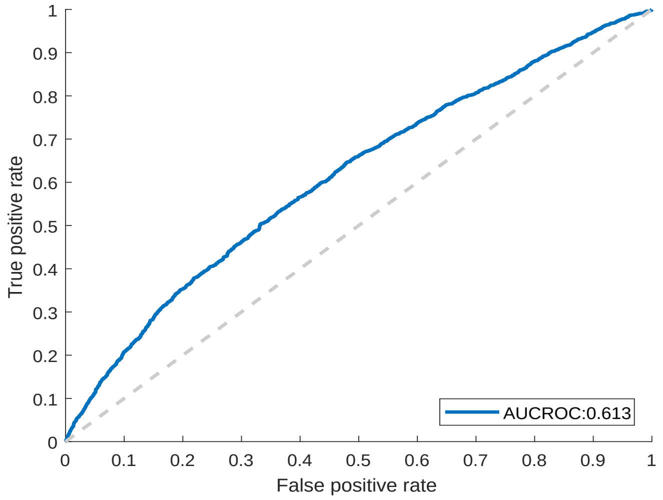

TR4 Top 500 peaks

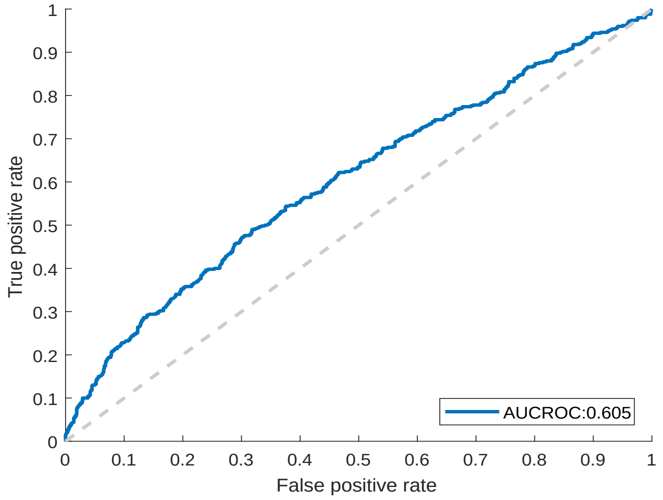

38 K562 TR4

TR4 All peaks

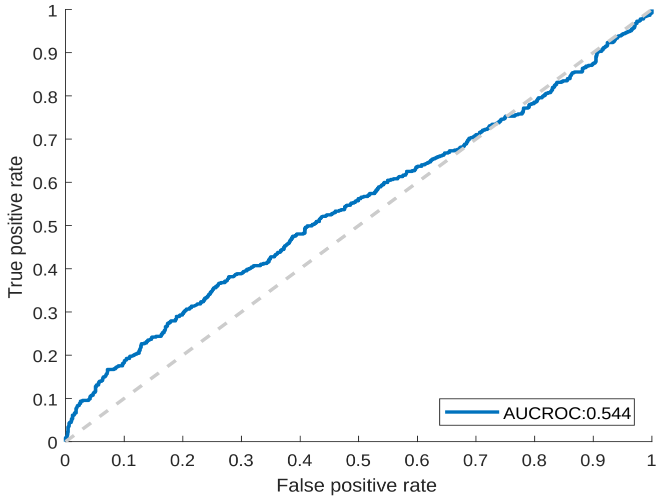

TR4 Top 500 peaks

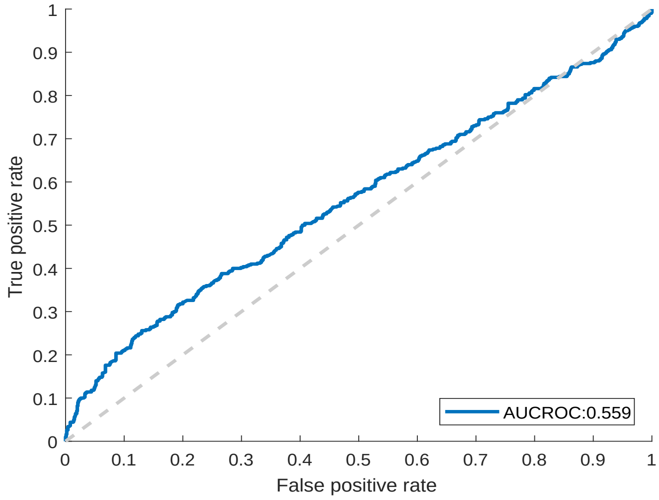

Supplement: Supplementary file 11 — Supplementary Data 9 [file 41467_2023_39577_MOESM11_ESM.pdf]
