# Supplementary material for: Hidden modes of DNA binding by human nuclear receptors
Source: Nat Commun. 2023 Jul 13;14:4179. doi: 10.1038/s41467-023-39577-0 (PMC10345098; doi:10.1038/s41467-023-39577-0)
Supplement: Supplementary file 13 — Supplementary Data 11 [file 41467_2023_39577_MOESM13_ESM.pdf]

Supplementary Data 11: Impact of single nucleotide polymorphisms on genomic binding of Nuclear Receptors

Contents

1 MR Round:3 4

2 MR+1 Round:3 5

3 PGR Round:3 6

4 PGR+2 Round:3 7

5 GR Round:3 8

6 GR+3 Round:3 9

7 ESRRG Round:3 10

8 ESRRG:RXRA Round:3 12

9 ESRRG+4 Round:3 13

10 ESRRB Round:3 14

11 ESRRB:RXRA Round:3 16

12 ESRRB+4 Round:3 18

13 ESRRA Round:3 20

14 ESRRA:RXRA Round:3 28

15 ESRRA+4 Round:3 31

16 ESR1+5 Round:3 36

17 ESR1+6 Round:3 38

18 THRB Round:3 39

19 THRB:RXRA Round:3 40

20 THRB:RXRA+7 Round:2 42

21 THRB+7 Round:3 44

22 THRA Round:3 45

23 THRA:RXRA Round:3 46

24 THRA:RXRA+7 Round:2 47

25 THRA+7 Round:3 48

26 RARG Round:3 49

27 RARB Round:3 51

28 RARA Round:3 52

29 RARG+8 Round:2 54

30 RARB+8 Round:3 55

31 RARA+8 Round:3 56

32 RARG:RXRA Round:3 57

33 RARB:RXRA Round:3 58

34 RARA:RXRA Round:3 **60**
35 PXR Round:3 **65**
36 PXR+9 Round:3 **66**
37 VDR Round:3 **67**
38 VDR:RXRA Round:3 **68**
39 VDR+10 Round:2 **69**
40 FXR Round:3 **70**
41 FXR+11 Round:3 **71**
42 LXRA Round:3 **72**
43 LXRA+12 Round:3 **73**
44 LXRB:RXRA Round:3 **74**
45 PPARD Round:3 **76**
46 PPARD+13 Round:3 **77**
47 PPARD+14 Round:3 **78**
48 PPARG Round:3 **79**
49 PPARG+14 Round:3 **80**
50 PPARG+15 Round:3 **81**
51 Rev-ErbA-Alpha Round:3 **82**
52 RORC Round:3 **83**
53 RORC:RXRA Round:3 **86**
54 RORC+16 Round:3 **87**
55 TR4 Round:3 **89**
56 TR2 Round:3 **90**
57 LRH1 Round:3 **91**
58 LRH1:RXRA Round:3 **94**
59 SF1 Round:3 **95**
60 SF1:RXRA Round:3 **100**
61 TLX Round:3 **101**
62 TLX:RXRA Round:3 **103**
63 PNR Round:3 **105**
64 COUP-TF2 Round:3 **106**
65 COUP-TF1 Round:3 **109**
66 EAR2 Round:3 **110**
67 COUP-TF2+17 Round:3 **111**

68 COUP-TF1+17 Round:3 **113**

69 COUP-TF2:RXRA Round:3 **115**

70 COUP-TF1:RXRA Round:3 **116**

71 EAR2:RXRA Round:3 **117**

72 HNF4G Round:3 **118**

73 HNF4A Round:3 **119**

74 HNF4A+18 Round:3 **120**

75 RXRB Round:3 **122**

76 RXRB+17 Round:3 **123**

77 RXRG Round:3 **124**

78 RXRG:RXRA Round:3 **127**

79 RXRG+17 Round:3 **128**

80 RXRA Round:3 **130**

81 RXRA+17 Round:3 **132**

82 NOR1 Round:3 **134**

83 NURR1 Round:3 **139**

1 MR Round:3

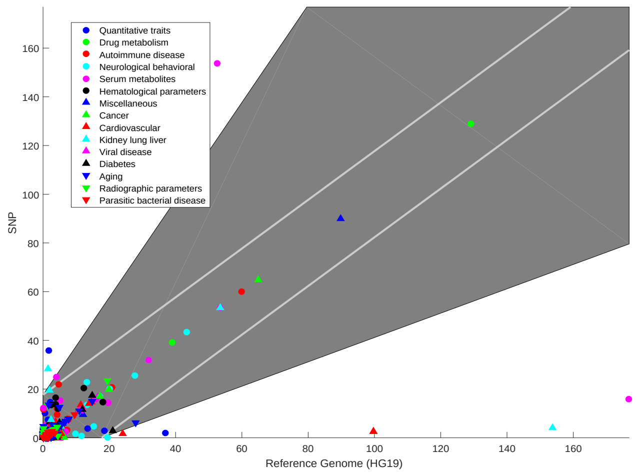

S.No.	SNP	Chr.	Position	Disease Class	Disease Trait	HG19	SNP	$log_2(\frac{SNP+\eta}{HG19+\eta})$
1	rs11858836	chr15	78783277	Kidney lung liver	COPD	153.80	4.21	-2.97
2	rs4921914	chr8	18272438	Serum metabolites	Formate-succinate ratio	176.85	16.00	-2.53
3	rs4977574	chr9	22098574	Cardiovascular	Coronary heart disease	99.65	2.72	-2.52
4	rs4977574	chr9	22098574	Cardiovascular	Myocardial infarction	99.65	2.72	-2.52
5	rs710841	chr4	82149831	Quantitative traits	Height	36.89	1.88	-1.48
6	rs2073233	chr20	12874585	Quantitative traits	Brain structure	1.83	35.91	1.46
7	rs4363657	chr12	21368722	Serum metabolites	Bilirubin	52.61	153.80	1.29
8	rs2499604	chr1	238103501	Kidney lung liver	NAFLDH	1.41	28.26	1.27
9	rs4370013	chr3	2654691	Cardiovascular	Diastolic blood pressure	24.07	1.69	-1.11
10	rs11880706	chr19	6080482	Neurological behavioral	Biplolar disorder and schizophrenia	19.47	0.00	-1.07

2 MR+1 Round:3

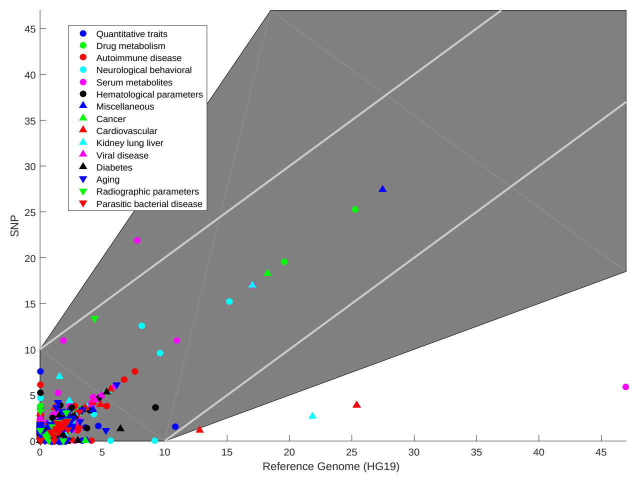

S.No.	SNP	Chr.	Position	Disease Class	Disease Trait	HG19	SNP	$log_2(\frac{SNP+\eta}{HG19+\eta})$
1	rs4921914	chr8	18272438	Serum metabolites	Formate-succinate ratio	46.99	5.87	-1.84
2	rs4977574	chr9	22098574	Cardiovascular	Coronary heart disease	25.41	3.92	-1.35
3	rs4977574	chr9	22098574	Cardiovascular	Myocardial infarction	25.41	3.92	-1.35
4	rs11858836	chr15	78783277	Kidney lung liver	COPD	21.87	2.67	-1.33
5	rs4370013	chr3	2654691	Cardiovascular	Diastolic blood pressure	12.78	1.14	-1.03

3 PGR Round:3

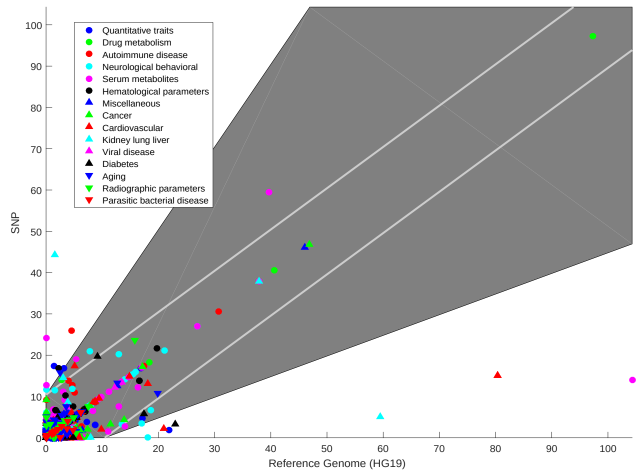

S.No.	SNP	Chr.	Position	Disease Class	Disease Trait	HG19	SNP	$log_2(\frac{SNP+\eta}{HG19+\eta})$
1	rs4921914	chr8	18272438	Serum metabolites	Formate-succinate ratio	104.30	14.06	-2.23
2	rs2499604	chr1	238103501	Kidney lung liver	NAFLDH	1.54	44.32	2.19
3	rs11858836	chr15	78783277	Kidney lung liver	COPD	59.46	5.07	-2.17
4	rs4977574	chr9	22098574	Cardiovascular	Coronary heart disease	80.34	15.01	-1.84
5	rs4977574	chr9	22098574	Cardiovascular	Myocardial infarction	80.34	15.01	-1.84
6	rs12029080	chr1	95053353	Serum metabolites	Fibrin-D-dimer levels	0.00	24.08	1.73
7	rs11880706	chr19	6080482	Neurological behavioral	Biplolar disorder and schizophrenia	18.15	0.00	-1.45
8	rs710841	chr4	82149831	Quantitative traits	Height	21.87	1.80	-1.40
9	rs4370013	chr3	2654691	Cardiovascular	Diastolic blood pressure	21.00	2.24	-1.31
10	rs7111341	chr11	2213166	Diabetes	Type 1 diabetes	23.02	3.25	-1.29
11	rs9469003	chr6	31407828	Autoimmune disease	Stevens-Johnson syndrome and necrolysis	4.55	25.93	1.28
12	rs2073233	chr20	12874585	Quantitative traits	Brain structure	1.40	17.47	1.24
13	rs4014195	chr11	65506822	Serum metabolites	Creatinine	0.00	12.72	1.15
14	rs1427407	chr2	60718043	Hematological parameters	F-cell distribution	2.25	16.92	1.11
15	rs2160519	chr12	13787846	Neurological behavioral	Cognitive performance-IED	0.00	11.66	1.08
16	rs903027	chr8	62409428	Aging	Alzheimers-Whole-brain volume	2.55	15.80	1.02

4 PGR+2 Round:3

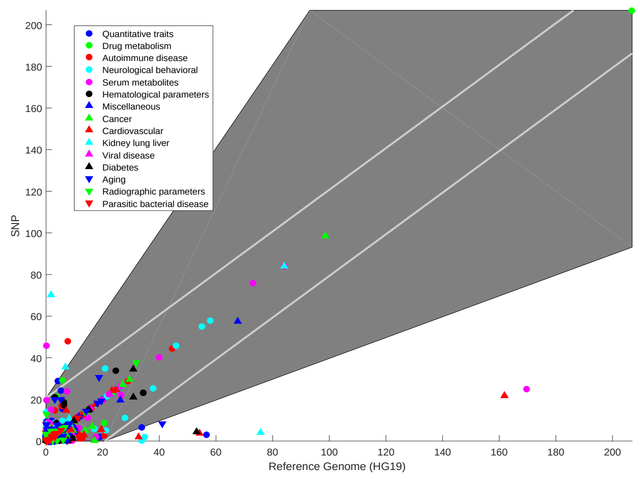

S.No.	SNP	Chr.	Position	Disease Class	Disease Trait	HG19	SNP	$log_2(\frac{SNP+\eta}{HG19+\eta})$
1	rs4977574	chr9	22098574	Cardiovascular	Coronary heart disease	161.74	21.69	-2.11
2	rs4977574	chr9	22098574	Cardiovascular	Myocardial infarction	161.74	21.69	-2.11
3	rs4921914	chr8	18272438	Serum metabolites	Formate-succinate ratio	169.56	24.81	-2.06
4	rs2499604	chr1	238103501	Kidney lung liver	NAFLDH	1.72	70.15	2.02
5	rs11858836	chr15	78783277	Kidney lung liver	COPD	75.83	4.03	-1.96
6	rs710841	chr4	82149831	Quantitative traits	Height	56.75	2.88	-1.72
7	rs12029080	chr1	95053353	Serum metabolites	Fibrin-D-dimer levels	0.00	45.81	1.68
8	rs4370013	chr3	2654691	Cardiovascular	Diastolic blood pressure	54.20	3.56	-1.63
9	rs7111341	chr11	2213166	Diabetes	Type 1 diabetes	53.10	4.39	-1.56
10	rs11880706	chr19	6080482	Neurological behavioral	Biplolar disorder and schizophrenia	33.87	0.00	-1.40
11	rs6832769	chr4	56298194	Neurological behavioral	Personality dimensions-agreeableness	34.90	1.81	-1.31
12	rs9469003	chr6	31407828	Autoimmune disease	Stevens-Johnson syndrome and necrolysis	7.63	47.92	1.28
13	rs1371867	chr8	101330209	Cardiovascular	PR interval	32.79	1.92	-1.24
14	rs17153527	chr7	106495809	Aging	Menopause	41.05	8.44	-1.08
15	rs1928168	chr6	22017738	Kidney lung liver	FEV1/FVC	6.99	35.49	1.02
16	rs6470764	chr8	130725665	Quantitative traits	Height	33.82	6.42	-1.01
17	rs2073233	chr20	12874585	Quantitative traits	Brain structure	4.10	28.99	1.00

5 GR Round:3

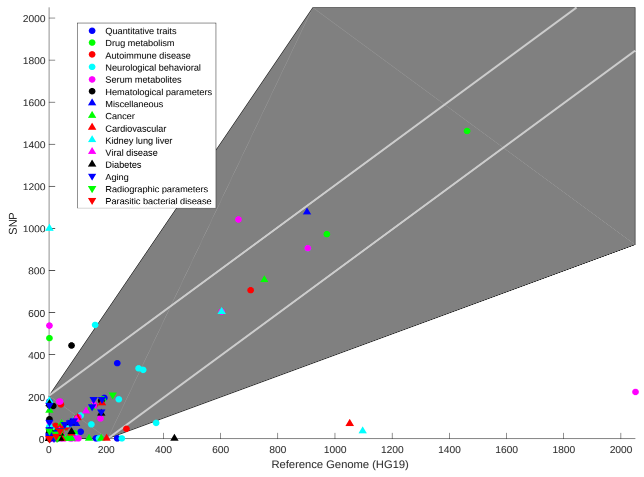

S.No.	SNP	Chr.	Position	Disease Class	Disease Trait	HG19	SNP	$log_2(\frac{SNP+\eta}{HG19+\eta})$
1	rs2499604	chr1	238103501	Kidney lung liver	NAFLDH	0.00	1000.76	2.56
2	rs11858836	chr15	78783277	Kidney lung liver	COPD	1097.61	37.01	-2.43
3	rs4921914	chr8	18272438	Serum metabolites	Formate-succinate ratio	2049.80	222.72	-2.40
4	rs4977574	chr9	22098574	Cardiovascular	Coronary heart disease	1051.38	72.64	-2.18
5	rs4977574	chr9	22098574	Cardiovascular	Myocardial infarction	1051.38	72.64	-2.18
6	rs12029080	chr1	95053353	Serum metabolites	Fibrin-D-dimer levels	0.00	536.77	1.86
7	rs1949733	chr4	8503359	Drug metabolism	Response to CLL treatment	0.00	478.99	1.74
8	rs7111341	chr11	2213166	Diabetes	Type 1 diabetes	438.72	0.00	-1.65
9	rs649729	chr2	31464385	Hematological parameters	Mean platelet volume	77.31	442.81	1.20
10	rs6832769	chr4	56298194	Neurological behavioral	Personality dimensions-agreeableness	253.58	0.00	-1.16
11	rs1440072	chr2	223936738	Quantitative traits	BMI	239.56	0.00	-1.12
12	rs1440072	chr2	223936738	Quantitative traits	Waist circumference	239.56	0.00	-1.12
13	rs9442235	chr1	16393357	Neurological behavioral	Cognitive performance-PC1	373.85	74.36	-1.05
14	rs7442317	chr4	29903052	Neurological behavioral	Attention deficit hyperactivity disorder motor coordination	160.83	541.87	1.03

6 GR+3 Round:3

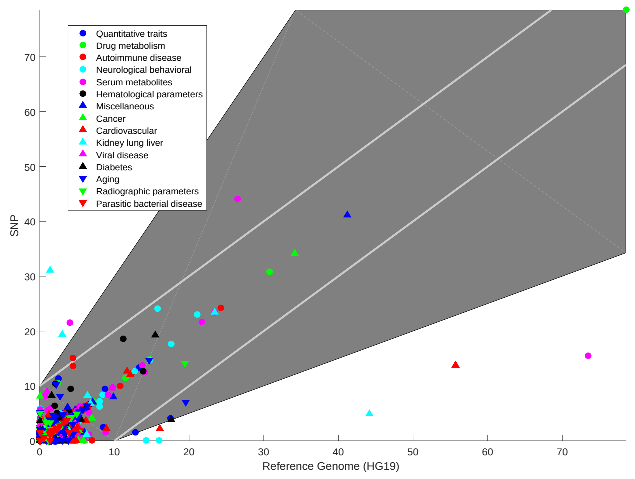

S.No.	SNP	Chr.	Position	Disease Class	Disease Trait	HG19	SNP	$log_2(\frac{SNP+\eta}{HG19+\eta})$
1	rs11858836	chr15	78783277	Kidney lung liver	COPD	44.11	4.91	-1.86
2	rs2499604	chr1	238103501	Kidney lung liver	NAFLDH	1.41	31.03	1.85
3	rs4921914	chr8	18272438	Serum metabolites	Formate-succinate ratio	73.45	15.55	-1.71
4	rs4977574	chr9	22098574	Cardiovascular	Coronary heart disease	55.68	13.72	-1.47
5	rs4977574	chr9	22098574	Cardiovascular	Myocardial infarction	55.68	13.72	-1.47
6	rs11880706	chr19	6080482	Neurological behavioral	Biplolar disorder and schizophrenia	16.02	0.00	-1.38
7	rs6832769	chr4	56298194	Neurological behavioral	Personality dimensions-agreeableness	14.27	0.00	-1.28
8	rs1928168	chr6	22017738	Kidney lung liver	FEV1/FVC	2.98	19.44	1.18
9	rs12029080	chr1	95053353	Serum metabolites	Fibrin-D-dimer levels	4.07	21.53	1.16
10	rs4370013	chr3	2654691	Cardiovascular	Diastolic blood pressure	16.11	2.24	-1.09

7 ESRRG Round:3

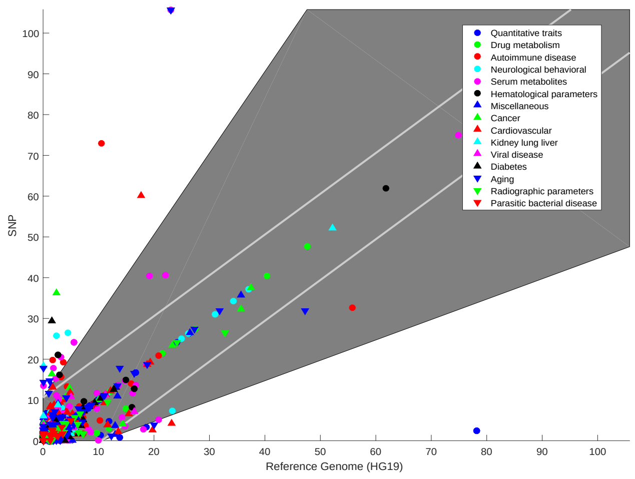

S.No.	SNP	Chr.	Position	Disease Class	Disease Trait	HG19	SNP	$log_2(\frac{SNP+\eta}{HG19+\eta})$
1	rs7138803	chr12	50247468	Quantitative traits	BMI	78.17	2.42	-2.77
2	rs7138803	chr12	50247468	Quantitative traits	Waist circumference	78.17	2.42	-2.77
3	rs7138803	chr12	50247468	Quantitative traits	Weight	78.17	2.42	-2.77
4	rs11761231	chr7	131370039	Autoimmune disease	Rheumatoid arthritis	10.58	72.93	1.98
5	rs7758229	chr6	160840252	Cancer	Colorectal cancer	2.44	36.18	1.84
6	rs157580	chr19	45395266	Aging	Alzheimers disease	23.00	105.77	1.79
7	rs157580	chr19	45395266	Aging	Alzheimers-AB1-42	23.00	105.77	1.79
8	rs157580	chr19	45395266	Serum metabolites	HDL cholesterol	23.00	105.77	1.79
9	rs157580	chr19	45395266	Serum metabolites	LDL cholesterol	23.00	105.77	1.79
10	rs4462262	chr10	59189178	Diabetes	Diabetic retinopathy	1.57	29.30	1.72
11	rs1329650	chr10	93348120	Neurological behavioral	Smoking behavior	2.36	25.71	1.49
12	rs56238310	chr3	111233239	Kidney lung liver	COPD	0.00	18.36	1.45
13	rs17188434	chr2	157096776	Aging	Age at menarche	0.00	17.73	1.42
14	rs4528684	chr19	14351574	Cardiovascular	Heart failure motality-EA	17.74	60.05	1.32
15	rs8007846	chr14	66262963	Autoimmune disease	Multiple sclerosis-Brain Glutamate Concentrations	1.70	19.73	1.30
16	rs1831521	chr9	93409357	Neurological behavioral	Cognitive performance-Nam	4.50	26.55	1.30
17	rs2814778	chr1	159174683	Hematological parameters	Neutrophil count	2.73	21.07	1.25
18	rs2814778	chr1	159174683	Hematological parameters	WBC count	2.73	21.07	1.25
19	rs2034764	chr9	2742771	Aging	Amyotrophic lateral sclerosis-age of onset	0.00	14.35	1.24

20	rs54211	chr22	39687484	Cardiovascular	Sudden cardiac arrest	19.73	2.59	-1.20
21	rs2194980	chr12	115502718	Serum metabolites	Tyrosine	1.85	17.82	1.19
22	rs2126259	chr8	9185146	Serum metabolites	LDL cholesterol	0.00	13.52	1.19
23	rs13038095	chr20	46425576	Cardiovascular	Atrial fibrillation	23.20	4.27	-1.19
24	rs1000778	chr11	61655305	Serum metabolites	Sphingolipid concentrations	3.25	20.54	1.17
25	rs7931342	chr11	68994497	Cancer	Prostate cancer	1.54	16.30	1.15
26	rs1925690	chr6	87867063	Aging	Alzheimers Entorhinal cortical thickness	1.17	14.78	1.11
27	rs174550	chr11	61571478	Serum metabolites	Fasting plasma glucose	5.57	24.22	1.11
28	rs174550	chr11	61571478	Serum metabolites	Insulin resistance	5.57	24.22	1.11
29	rs174550	chr11	61571478	Serum metabolites	Serum polyunsaturated fatty acids	5.57	24.22	1.11
30	rs2185570	chr10	96751270	Serum metabolites	Serum dehydroepiandrosterone	18.13	2.79	-1.10
31	rs6499640	chr16	53769677	Quantitative traits	BMI	13.88	0.88	-1.09
32	rs6499640	chr16	53769677	Quantitative traits	Weight	13.88	0.88	-1.09
33	rs1713985	chr4	57786450	Aging	Age-related macular degeneration	20.02	3.84	-1.09
34	rs668853	chr9	85311147	Autoimmune disease	Ulcerative colitis	3.60	19.33	1.08
35	rs9470004	chr6	35341850	Quantitative traits	Height	18.60	3.33	-1.07
36	rs10885122	chr10	113042093	Serum metabolites	Fasting plasma glucose	2.22	15.23	1.01
37	rs10885122	chr10	113042093	Serum metabolites	Insulin resistance	2.22	15.23	1.01
38	rs11959928	chr5	39397132	Serum metabolites	Creatinine	20.86	5.07	-1.01

8 ESRRG:RXRA Round:3

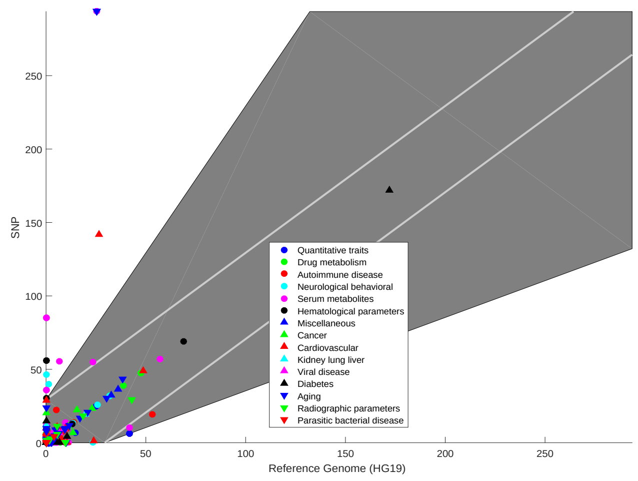

S.No.	SNP	Chr.	Position	Disease Class	Disease Trait	HG19	SNP	$log_2(\frac{SNP+\eta}{HG19+\eta})$
1	rs157580	chr19	45395266	Aging	Alzheimers disease	25.46	293.63	2.56
2	rs157580	chr19	45395266	Aging	Alzheimers-AB1-42	25.46	293.63	2.56
3	rs157580	chr19	45395266	Serum metabolites	HDL cholesterol	25.46	293.63	2.56
4	rs157580	chr19	45395266	Serum metabolites	LDL cholesterol	25.46	293.63	2.56
5	rs174550	chr11	61571478	Serum metabolites	Fasting plasma glucose	0.00	85.30	1.97
6	rs174550	chr11	61571478	Serum metabolites	Insulin resistance	0.00	85.30	1.97
7	rs174550	chr11	61571478	Serum metabolites	Serum polyunsaturated fatty acids	0.00	85.30	1.97
8	rs4528684	chr19	14351574	Cardiovascular	Heart failure motality-EA	26.42	141.70	1.62
9	rs2814778	chr1	159174683	Hematological parameters	Neutrophil count	0.00	55.99	1.54
10	rs2814778	chr1	159174683	Hematological parameters	WBC count	0.00	55.99	1.54
11	rs1831521	chr9	93409357	Neurological behavioral	Cognitive performance-Nam	0.00	46.28	1.37
12	rs2153960	chr6	108988184	Serum metabolites	IGF-1	6.80	55.59	1.23
13	rs1329650	chr10	93348120	Neurological behavioral	Smoking behavior	1.19	39.70	1.18
14	rs10885122	chr10	113042093	Serum metabolites	Fasting plasma glucose	0.00	36.05	1.16
15	rs10885122	chr10	113042093	Serum metabolites	Insulin resistance	0.00	36.05	1.16
16	rs11085824	chr19	13001547	Hematological parameters	Mean corpuscular hemoglobin	0.00	30.20	1.02

9 ESRRG+4 Round:3

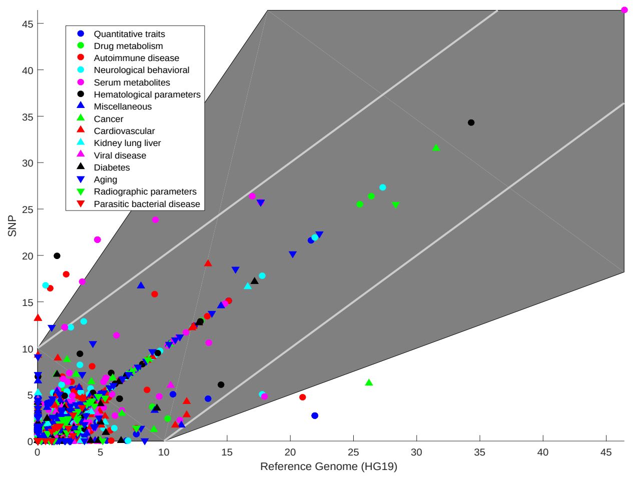

S.No.	SNP	Chr.	Position	Disease Class	Disease Trait	HG19	SNP	$log_2(\frac{SNP+\eta}{HG19+\eta})$
1	rs11085824	chr19	13001547	Hematological parameters	Mean corpuscular hemoglobin	1.52	19.99	1.38
2	rs1329650	chr10	93348120	Neurological behavioral	Smoking behavior	0.66	16.82	1.33
3	rs7138803	chr12	50247468	Quantitative traits	BMI	21.97	2.77	-1.32
4	rs7138803	chr12	50247468	Quantitative traits	Waist circumference	21.97	2.77	-1.32
5	rs7138803	chr12	50247468	Quantitative traits	Weight	21.97	2.77	-1.32
6	rs8007846	chr14	66262963	Autoimmune disease	Multiple sclerosis-Brain Glutamate Concentrations	0.97	16.49	1.27
7	rs3825214	chr12	114795443	Cardiovascular	PR interval	0.00	13.20	1.21
8	rs3825214	chr12	114795443	Cardiovascular	QRS duration	0.00	13.20	1.21
9	rs3825214	chr12	114795443	Cardiovascular	QT interval	0.00	13.20	1.21
10	rs12644284	chr4	154154000	Autoimmune disease	Multiple sclerosis	2.25	17.96	1.19
11	rs721048	chr2	63131731	Cancer	Prostate cancer	26.23	6.22	-1.16
12	rs174550	chr11	61571478	Serum metabolites	Fasting plasma glucose	4.74	21.70	1.10
13	rs174550	chr11	61571478	Serum metabolites	Insulin resistance	4.74	21.70	1.10
14	rs174550	chr11	61571478	Serum metabolites	Serum polyunsaturated fatty acids	4.74	21.70	1.10
15	rs8070463	chr17	45768836	Autoimmune disease	Ankylosing spondylitis	21.00	4.74	-1.07
16	rs1000778	chr11	61655305	Serum metabolites	Sphingolipid concentrations	3.53	17.16	1.01
17	rs7153703	chr14	51919822	Aging	Alzheimers Total ventricular volume	1.09	12.26	1.00

10 ESRRB Round:3

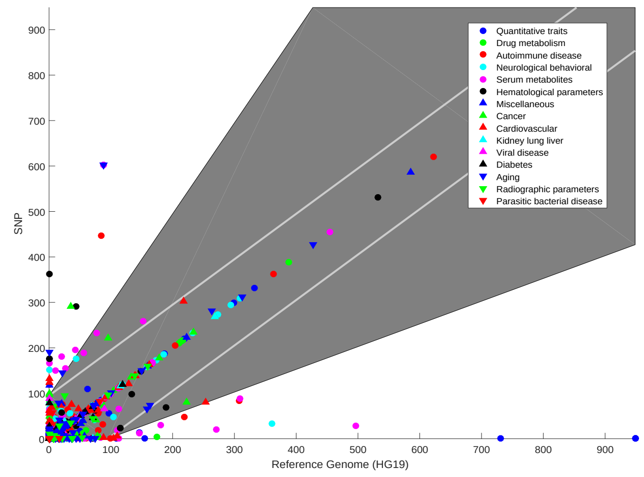

S.No.	SNP	Chr.	Position	Disease Class	Disease Trait	HG19	SNP	$log_2(\frac{SNP+\eta}{HG19+\eta})$
1	rs7138803	chr12	50247468	Quantitative traits	BMI	948.61	0.00	-3.46
2	rs7138803	chr12	50247468	Quantitative traits	Waist circumference	948.61	0.00	-3.46
3	rs7138803	chr12	50247468	Quantitative traits	Weight	948.61	0.00	-3.46
4	rs9470004	chr6	35341850	Quantitative traits	Height	730.44	1.11	-3.10
5	rs11085824	chr19	13001547	Hematological parameters	Mean corpuscular hemoglobin	0.00	362.34	2.27
6	rs4744712	chr9	71434707	Serum metabolites	Creatinine	497.10	28.21	-2.27
7	rs157580	chr19	45395266	Aging	Alzheimers disease	88.31	601.97	1.93
8	rs157580	chr19	45395266	Aging	Alzheimers-AB1-42	88.31	601.97	1.93
9	rs157580	chr19	45395266	Serum metabolites	HDL cholesterol	88.31	601.97	1.93
10	rs157580	chr19	45395266	Serum metabolites	LDL cholesterol	88.31	601.97	1.93
11	rs12579350	chr12	5797101	Neurological behavioral	Panic disorder	360.81	33.40	-1.83
12	rs10741657	chr11	14914878	Serum metabolites	Vitamin D insufficiency	271.19	19.59	-1.68
13	rs11761231	chr7	131370039	Autoimmune disease	Rheumatoid arthritis	84.42	447.45	1.60
14	rs1925690	chr6	87867063	Aging	Alzheimers Entorhinal cortical thickness	0.00	190.53	1.59
15	rs7758229	chr6	160840252	Cancer	Colorectal cancer	35.11	290.37	1.57
16	rs2814778	chr1	159174683	Hematological parameters	Neutrophil count	0.00	175.82	1.51
17	rs2814778	chr1	159174683	Hematological parameters	WBC count	0.00	175.82	1.51
18	rs10017284	chr4	121513215	Hematological parameters	F-cell distribution	43.55	290.47	1.48
19	rs1880887	chr12	41721430	Serum metabolites	Alkaline phosphatase	0.00	166.25	1.46

20	rs153091	chr16	13253956	Drug metabolism	Response to antipsychotic therapy perphenazine-triglycerides	174.95	3.93	-1.45
21	rs10506821	chr12	80496923	Quantitative traits	Hip geometry	154.65	0.00	-1.40
22	rs1329650	chr10	93348120	Neurological behavioral	Smoking behavior	0.00	151.41	1.38
23	rs10777317	chr12	91980374	Cardiovascular	Sudden cardiac arrest	0.00	131.54	1.26
24	rs11823543	chr11	116649135	Serum metabolites	Triglycerides blood pressure	20.72	180.47	1.25
25	rs12459897	chr19	31596778	Serum metabolites	Serum polyunsaturated fatty acids	10.63	149.66	1.21
26	rs2251393	chr17	60778932	Cardiovascular	Sudden cardiac arrest	0.00	124.61	1.21
27	rs8070463	chr17	45768836	Autoimmune disease	Ankylosing spondylitis	307.81	82.77	-1.18
28	rs1063635	chr6	31379931	Autoimmune disease	Rheumatoid arthritis	0.00	118.96	1.17
29	rs4963452	chr11	61815803	Serum metabolites	Serum polyunsaturated fatty acids	145.93	12.07	-1.17
30	rs681900	chr2	75074967	Miscellaneous	Femoral neck bone geometry	0.00	118.12	1.17
31	rs2815752	chr1	72812440	Quantitative traits	BMI	145.86	13.18	-1.16
32	rs10499559	chr7	22109459	Serum metabolites	Thyroid stimulating hormone	309.13	87.95	-1.14
33	rs1158167	chr20	23578189	Serum metabolites	Cystatin C	180.92	30.20	-1.14
34	rs6441286	chr3	159728878	Autoimmune disease	Primary biliary cirrhosis	219.24	48.01	-1.14
35	rs11959928	chr5	39397132	Serum metabolites	Creatinine	113.33	0.00	-1.13
36	rs6499640	chr16	53769677	Quantitative traits	BMI	109.19	0.00	-1.11
37	rs6499640	chr16	53769677	Quantitative traits	Weight	109.19	0.00	-1.11
38	rs9992101	chr4	77360431	Serum metabolites	Creatinine	43.06	195.49	1.07
39	rs12046278	chr1	10799577	Cardiovascular	Systolic blood pressure	104.78	0.00	-1.07
40	rs1539019	chr1	247600301	Serum metabolites	Fibrinogen	25.91	155.35	1.05
41	rs801114	chr1	228997835	Cancer	Basal cell carcinoma	0.00	100.49	1.04
42	rs2034764	chr9	2742771	Aging	Amyotrophic lateral sclerosis-age of onset	21.95	144.85	1.04
43	rs3745672	chr19	12146370	Autoimmune disease	Multiple sclerosis	98.90	0.00	-1.03
44	rs1550576	chr15	58213414	Cardiovascular	Hypertension	110.50	6.23	-1.02
45	rs471364	chr9	15289578	Serum metabolites	HDL cholesterol	0.00	96.07	1.01
46	rs7931342	chr11	68994497	Cancer	Prostate cancer	0.00	96.02	1.01

11 ESRRB:RXRA Round:3

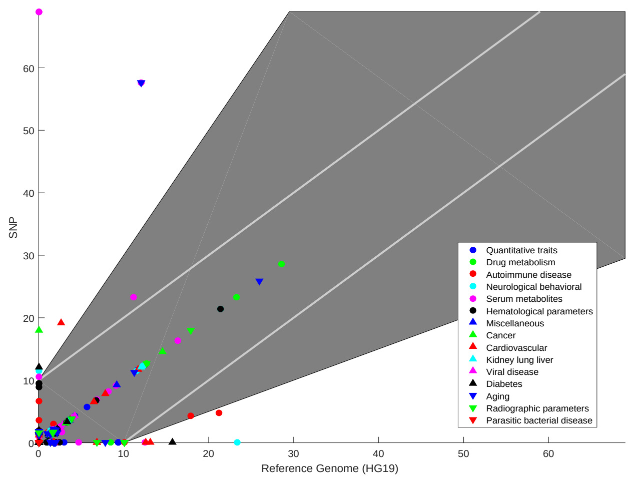

S.No.	SNP	Chr.	Position	Disease Class	Disease Trait	HG19	SNP	$log_2(\frac{SNP+\eta}{HG19+\eta})$
1	rs174550	chr11	61571478	Serum metabolites	Fasting plasma glucose	0.00	68.99	2.98
2	rs174550	chr11	61571478	Serum metabolites	Insulin resistance	0.00	68.99	2.98
3	rs174550	chr11	61571478	Serum metabolites	Serum polyunsaturated fatty acids	0.00	68.99	2.98
4	rs12579350	chr12	5797101	Neurological behavioral	Panic disorder	23.38	0.00	-1.74
5	rs157580	chr19	45395266	Aging	Alzheimers disease	12.04	57.64	1.62
6	rs157580	chr19	45395266	Aging	Alzheimers-AB1-42	12.04	57.64	1.62
7	rs157580	chr19	45395266	Serum metabolites	HDL cholesterol	12.04	57.64	1.62
8	rs157580	chr19	45395266	Serum metabolites	LDL cholesterol	12.04	57.64	1.62
9	rs7758229	chr6	160840252	Cancer	Colorectal cancer	0.00	17.93	1.48
10	rs7754840	chr6	20661250	Diabetes	Type 2 diabetes	15.74	0.00	-1.36
11	rs54211	chr22	39687484	Cardiovascular	Sudden cardiac arrest	13.14	0.00	-1.21
12	rs4528684	chr19	14351574	Cardiovascular	Heart failure motality-EA	2.66	19.20	1.21
13	rs13038095	chr20	46425576	Cardiovascular	Atrial fibrillation	12.57	0.00	-1.17
14	rs10741657	chr11	14914878	Serum metabolites	Vitamin D insufficiency	12.49	0.00	-1.17
15	rs4462262	chr10	59189178	Diabetes	Diabetic retinopathy	0.00	12.11	1.14
16	rs1831521	chr9	93409357	Neurological behavioral	Cognitive performance-Nam	0.00	11.51	1.11
17	rs8070463	chr17	45768836	Autoimmune disease	Ankylosing spondylitis	21.22	4.82	-1.07
18	rs2153960	chr6	108988184	Serum metabolites	IGF-1	0.00	10.53	1.04
19	rs7524102	chr1	22698447	Radiographic parameters	Bone mineral density-femoral neck	10.09	0.00	-1.01
20	rs7524102	chr1	22698447	Radiographic parameters	Bone mineral density-hip	10.09	0.00	-1.01

21	rs7524102	chr1	22698447	Radiographic parameters	Bone mineral density-spine	10.09	0.00	-1.01
22	rs7524102	chr1	22698447	Autoimmune disease	Ulcerative colitis	10.09	0.00	-1.01

12 ESRRB+4 Round:3

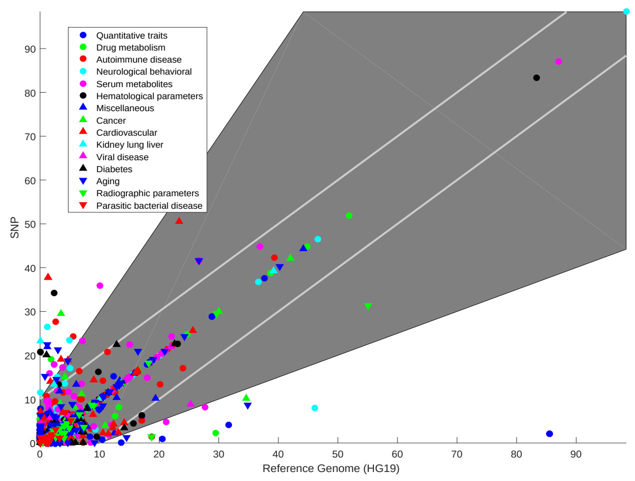

S.No.	SNP	Chr.	Position	Disease Class	Disease Trait	HG19	SNP	$log_2(\frac{SNP+\eta}{HG19+\eta})$
1	rs7138803	chr12	50247468	Quantitative traits	BMI	85.58	2.13	-2.98
2	rs7138803	chr12	50247468	Quantitative traits	Waist circumference	85.58	2.13	-2.98
3	rs7138803	chr12	50247468	Quantitative traits	Weight	85.58	2.13	-2.98
4	rs3825214	chr12	114795443	Cardiovascular	PR interval	1.39	37.72	2.07
5	rs3825214	chr12	114795443	Cardiovascular	QRS duration	1.39	37.72	2.07
6	rs3825214	chr12	114795443	Cardiovascular	QT interval	1.39	37.72	2.07
7	rs11085824	chr19	13001547	Hematological parameters	Mean corpuscular hemoglobin	2.42	34.15	1.83
8	rs56238310	chr3	111233239	Kidney lung liver	COPD	0.00	23.38	1.74
9	rs1329650	chr10	93348120	Neurological behavioral	Smoking behavior	1.16	26.56	1.71
10	rs153091	chr16	13253956	Drug metabolism	Response to antipsychotic therapy perphenazine-triglycerides	29.51	2.26	-1.69
11	rs12579350	chr12	5797101	Neurological behavioral	Panic disorder	46.08	8.02	-1.64
12	rs2814778	chr1	159174683	Hematological parameters	Neutrophil count	0.00	20.77	1.62
13	rs2814778	chr1	159174683	Hematological parameters	WBC count	0.00	20.77	1.62
14	rs12644284	chr4	154154000	Autoimmune disease	Multiple sclerosis	2.62	27.74	1.58
15	rs9470004	chr6	35341850	Quantitative traits	Height	31.60	4.12	-1.56
16	rs7758229	chr6	160840252	Cancer	Colorectal cancer	3.49	29.60	1.55
17	rs7153703	chr14	51919822	Aging	Alzheimers Total ventricular volume	1.25	22.49	1.53
18	rs681900	chr2	75074967	Miscellaneous	Femoral neck bone geometry	1.26	21.99	1.51
19	rs5757949	chr22	40820151	Quantitative traits	Height	20.50	0.89	-1.49
20	rs4462262	chr10	59189178	Diabetes	Diabetic retinopathy	1.09	20.06	1.44

21	rs7524102	chr1	22698447	Radiographic parameters	Bone mineral density-femoral neck	18.73	1.43	-1.33
22	rs7524102	chr1	22698447	Radiographic parameters	Bone mineral density-hip	18.73	1.43	-1.33
23	rs7524102	chr1	22698447	Radiographic parameters	Bone mineral density-spine	18.73	1.43	-1.33
24	rs7524102	chr1	22698447	Autoimmune disease	Ulcerative colitis	18.73	1.43	-1.33
25	rs1431005	chr4	188345368	Drug metabolism	Response to statin therapy-LDL diff	1.79	19.10	1.30
26	rs1713985	chr4	57786450	Aging	Age-related macular degeneration	34.85	8.61	-1.27
27	rs2034764	chr9	2742771	Aging	Amyotrophic lateral sclerosis-age of onset	3.03	21.31	1.27
28	rs10506821	chr12	80496923	Quantitative traits	Hip geometry	13.63	0.00	-1.24
29	rs1925690	chr6	87867063	Aging	Alzheimers Entorhinal cortical thickness	0.79	15.26	1.23
30	rs2153960	chr6	108988184	Serum metabolites	IGF-1	9.98	35.95	1.20
31	rs10885122	chr10	113042093	Serum metabolites	Fasting plasma glucose	2.38	17.91	1.17
32	rs10885122	chr10	113042093	Serum metabolites	Insulin resistance	2.38	17.91	1.17
33	rs4964805	chr12	104192824	Neurological behavioral	ADHD	4.88	23.38	1.17
34	rs2126259	chr8	9185146	Serum metabolites	LDL cholesterol	1.64	15.90	1.15
35	rs721048	chr2	63131731	Cancer	Prostate cancer	34.66	10.21	-1.14
36	rs8049439	chr16	28837515	Autoimmune disease	Inflammatory bowel disease-early onset	5.53	24.28	1.14
37	rs466639	chr1	165394882	Aging	Age at menarche	14.55	1.24	-1.13
38	rs9512637	chr13	27920611	Neurological behavioral	Alcohol consumption	0.00	11.56	1.11
39	rs10777317	chr12	91980374	Cardiovascular	Sudden cardiac arrest	3.54	19.09	1.10
40	rs675209	chr6	7102084	Serum metabolites	Serum urate	21.11	4.83	-1.07
41	rs4963452	chr11	61815803	Serum metabolites	Serum polyunsaturated fatty acids	27.74	8.10	-1.06
42	rs2782980	chr10	115781527	Cardiovascular	Blood pressure	1.67	14.06	1.04

13 ESRRA Round:3

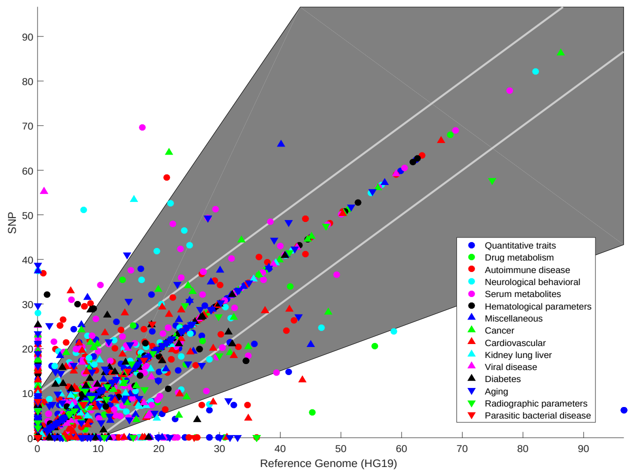

S.No.	SNP	Chr.	Position	Disease Class	Disease Trait	HG19	SNP	$log_2(\frac{SNP+\eta}{HG19+\eta})$
1	rs7138803	chr12	50247468	Quantitative traits	BMI	96.59	6.15	-2.72
2	rs7138803	chr12	50247468	Quantitative traits	Waist circumference	96.59	6.15	-2.72
3	rs7138803	chr12	50247468	Quantitative traits	Weight	96.59	6.15	-2.72
4	rs8099917	chr19	39743165	Viral disease	Chronic Hepatitis C infection	1.12	55.23	2.55
5	rs8099917	chr19	39743165	Viral disease	Response to hepatitis C treatment	1.12	55.23	2.55
6	rs7153703	chr14	51919822	Aging	Alzheimers Total ventricular volume	0.00	38.80	2.29
7	rs681900	chr2	75074967	Miscellaneous	Femoral neck bone geometry	0.00	37.45	2.25
8	rs1329650	chr10	93348120	Neurological behavioral	Smoking behavior	0.00	37.44	2.25
9	rs7524102	chr1	22698447	Radiographic parameters	Bone mineral density-femoral neck	36.15	0.00	-2.21
10	rs7524102	chr1	22698447	Radiographic parameters	Bone mineral density-hip	36.15	0.00	-2.21
11	rs7524102	chr1	22698447	Radiographic parameters	Bone mineral density-spine	36.15	0.00	-2.21
12	rs7524102	chr1	22698447	Autoimmune disease	Ulcerative colitis	36.15	0.00	-2.21
13	rs3772255	chr3	156102734	Aging	Aging traits-biologic age	33.01	0.00	-2.10
14	rs8007846	chr14	66262963	Autoimmune disease	Multiple sclerosis-Brain Glutamate Concentrations	1.00	36.94	2.09
15	rs466639	chr1	165394882	Aging	Age at menarche	31.68	0.00	-2.06
16	rs688034	chr22	26689635	Cardiovascular	Coronary heart disease	31.25	0.00	-2.04
17	rs2830840	chr21	28665347	Drug metabolism	Response to citalopram treatment	30.85	0.00	-2.03
18	rs13095226	chr3	99396272	Aging	Age-related macular degeneration	30.11	0.00	-2.00

19	rs10506821	chr12	80496923	Quantitative traits	Hip geometry	29.86	0.00	-1.99
20	rs1925690	chr6	87867063	Aging	Alzheimers Entorhinal cortical thickness	0.00	29.64	1.99
21	rs6499640	chr16	53769677	Quantitative traits	BMI	28.35	0.00	-1.94
22	rs6499640	chr16	53769677	Quantitative traits	Weight	28.35	0.00	-1.94
23	rs9804317	chr10	130248926	Neurological behavioral	Animals	0.00	27.97	1.92
24	rs7786877	chr7	100214015	Hematological parameters	Mean corpuscular volume	1.52	32.13	1.87
25	rs13314993	chr3	33015469	Autoimmune disease	Celiac disease	26.34	0.00	-1.86
26	rs16966142	chr16	89851033	Drug metabolism	Caffeine intake	26.04	0.00	-1.85
27	rs1451375	chr7	50622712	Parasitic bacterial disease	Malaria	25.86	0.00	-1.84
28	rs2943641	chr2	227093745	Diabetes	Type 2 diabetes	0.00	25.43	1.82
29	rs2500535	chr6	149329267	Drug metabolism	Response to antidepressants-nortryptyline	45.27	5.73	-1.81
30	rs6139030	chr20	3187733	Viral disease	Response to hepatitis C treatment	0.00	25.11	1.81
31	rs10181042	chr2	61224259	Autoimmune disease	Crohns disease	24.90	0.00	-1.80
32	rs1831521	chr9	93409357	Neurological behavioral	Cognitive performance-Nam	7.65	51.07	1.79
33	rs157350	chr5	156139569	Quantitative traits	Brachial circumference	24.32	0.00	-1.78
34	rs157350	chr5	156139569	Quantitative traits	Hip circumference	24.32	0.00	-1.78
35	rs10521157	chr17	9336370	Aging	Longevity	0.00	23.34	1.74
36	rs4254535	chr2	69198388	Cancer	Lung cancer	0.00	23.07	1.73
37	rs3120139	chr6	160741622	Serum metabolites	Lipoprotein A	23.01	0.00	-1.72
38	rs4547811	chr4	146794621	Serum metabolites	Gamma glutamyl transferase	0.00	22.96	1.72
39	rs907611	chr11	1874072	Autoimmune disease	Ulcerative colitis	0.00	22.81	1.71
40	rs7765379	chr6	32680928	Autoimmune disease	Rheumatoid arthritis	22.58	0.00	-1.70
41	rs10466868	chr12	131939920	Serum metabolites	Erythropoeitin	22.45	0.00	-1.70
42	rs1648707	chr3	186551711	Serum metabolites	Adiponectin	0.00	22.19	1.69
43	rs10260404	chr7	154210798	Aging	Amyotrophic lateral sclerosis	21.79	0.00	-1.67
44	rs6717918	chr2	233155110	Quantitative traits	Height	21.42	0.00	-1.65
45	rs12203592	chr6	396321	Quantitative traits	Freckling	0.00	21.27	1.64
46	rs12203592	chr6	396321	Quantitative traits	Hair color	0.00	21.27	1.64

47	rs12203592	chr6	396321	Quantitative traits	Hair color-Black vs. blond hair color	0.00	21.27	1.64
48	rs12203592	chr6	396321	Quantitative traits	Hair color-Black vs. red hair color	0.00	21.27	1.64
49	rs12203592	chr6	396321	Aging	Progressive supranuclear palsy	0.00	21.27	1.64
50	rs10850409	chr12	115381740	Cardiovascular	QRS duration	34.77	4.40	-1.64
51	rs2159324	chr19	45695738	Serum metabolites	C-reactive protein	20.88	0.00	-1.63
52	rs2159324	chr19	45695738	Serum metabolites	LDL cholesterol	20.88	0.00	-1.63
53	rs2008242	chr4	5221538	Cardiovascular	PR segment	0.00	20.81	1.62
54	rs10447248	chr5	107915736	Serum metabolites	Adiponectin	20.58	0.00	-1.61
55	rs4392868	chr8	96120072	Miscellaneous	Radiation response	20.57	0.00	-1.61
56	rs571312	chr18	57839769	Quantitative traits	BMI	19.90	0.00	-1.58
57	rs10412199	chr19	3927771	Aging	Aging traits-age free from disease	19.90	0.00	-1.58
58	rs2967605	chr19	8469738	Serum metabolites	HDL cholesterol	0.00	19.67	1.57
59	rs10411161	chr19	52372976	Cancer	Breast cancer	0.00	19.61	1.57
60	rs922948	chr3	69442637	Quantitative traits	Hip geometry	0.00	19.52	1.56
61	rs2042831	chr2	235857114	Aging	Longevity	1.90	25.11	1.56
62	rs771767	chr3	101748638	Autoimmune disease	Multiple sclerosis	0.00	19.48	1.56
63	rs1000778	chr11	61655305	Serum metabolites	Sphingolipid concentrations	17.26	69.66	1.55
64	rs515135	chr2	21286057	Serum metabolites	LDL cholesterol	0.00	19.17	1.54
65	rs3793917	chr10	124219275	Aging	Age-related macular degeneration	0.00	18.93	1.53
66	rs204993	chr6	32155581	Autoimmune disease	Asthma	0.00	18.79	1.53
67	rs17114036	chr1	56962821	Cardiovascular	Coronary heart disease	0.00	18.74	1.52
68	rs9395066	chr6	45095163	Quantitative traits	Height	0.00	18.62	1.52
69	rs17319721	chr4	77368847	Serum metabolites	Creatinine	18.54	0.00	-1.51
70	rs12625057	chr20	57790436	Drug metabolism	Response to antipsychotic therapy extrapyramidal side effects	0.00	18.49	1.51
71	rs2738113	chr8	6829085	Miscellaneous	Endometriosis	18.49	0.00	-1.51
72	rs6120849	chr20	33730387	Serum metabolites	Protein C	18.41	0.00	-1.51
73	rs7754840	chr6	20661250	Diabetes	Type 2 diabetes	18.29	0.00	-1.50
74	rs6738028	chr2	111949327	Serum metabolites	Serum dehydroepiandrosterone	0.00	18.09	1.49

75	rs7112513	chr11	117037361	Serum metabolites	Transferrin receptor	0.00	17.93	1.48
76	rs9512637	chr13	27920611	Neurological behavioral	Alcohol consumption	0.00	17.87	1.48
77	rs10777317	chr12	91980374	Cardiovascular	Sudden cardiac arrest	5.49	32.90	1.47
78	rs6964415	chr7	46242583	Cardiovascular	Sudden cardiac arrest	17.69	0.00	-1.47
79	rs4765623	chr12	125320850	Cancer	Renal cell carcinoma	0.00	17.63	1.47
80	rs11786458	chr8	40252701	Neurological behavioral	ADHD-Inattentive symptoms	17.59	0.00	-1.46
81	rs3745516	chr19	50926742	Autoimmune disease	Primary biliary cirrhosis	0.00	17.45	1.46
82	rs110419	chr11	8252853	Cancer	Neuroblastoma	0.00	17.37	1.45
83	rs16832015	chr1	160421916	Neurological behavioral	Cognitive performance-IED	0.00	17.33	1.45
84	rs9272105	chr6	32599999	Drug metabolism	Response to interferon beta therapy	17.28	0.00	-1.45
85	rs152528	chr5	142017860	Cardiovascular	Cardiac hypertrophy	17.25	0.00	-1.45
86	rs13106227	chr4	77418681	Autoimmune disease	Eosinophilic esophagitis pediatric	17.01	0.00	-1.43
87	rs2470893	chr15	75019449	Drug metabolism	Caffeine intake	0.00	16.98	1.43
88	rs2470893	chr15	75019449	Drug metabolism	coffee consumption	0.00	16.98	1.43
89	rs1939992	chr11	126809705	Serum metabolites	IGF-1	0.00	16.98	1.43
90	rs12576775	chr11	79077193	Neurological behavioral	Bipolar disorder	16.96	0.00	-1.43
91	rs2444217	chr16	4038387	Quantitative traits	BMI	16.83	0.00	-1.42
92	rs10914144	chr1	171949750	Hematological parameters	Mean platelet volume	0.00	16.78	1.42
93	rs10914144	chr1	171949750	Hematological parameters	Platelet count	0.00	16.78	1.42
94	rs12449157	chr16	67708897	Serum metabolites	HDL cholesterol	0.00	16.67	1.42
95	rs6503525	chr17	38095174	Neurological behavioral	Schizophrenia	16.60	0.00	-1.41
96	rs2126259	chr8	9185146	Serum metabolites	LDL cholesterol	0.00	16.54	1.41
97	rs2194980	chr12	115502718	Serum metabolites	Tyrosine	5.55	30.90	1.40
98	rs536841	chr11	85787824	Aging	Alzheimers disease	0.00	16.11	1.38
99	rs7703051	chr5	74625487	Serum metabolites	LDL cholesterol	16.07	0.00	-1.38
100	rs988712	chr11	27563382	Quantitative traits	BMI	0.00	15.99	1.38
101	rs6474359	chr8	41549194	Serum metabolites	HbA1C	0.00	15.86	1.37
102	rs4729260	chr7	96117918	Radiographic parameters	Bone mineral density-spine	15.79	0.00	-1.37

103	rs2201841	chr1	67694202	Autoimmune disease	Psoriasis	3.68	25.20	1.36
104	rs2201841	chr1	67694202	Autoimmune disease	Ulcerative colitis	3.68	25.20	1.36
105	rs9866141	chr3	156950579	Diabetes	Diabetic retinopathy	26.30	4.11	-1.36
106	rs6420094	chr5	176817636	Serum metabolites	Creatinine	0.00	15.68	1.36
107	rs1976403	chr1	21766453	Serum metabolites	Alkaline phosphatase	15.62	0.00	-1.36
108	rs6887695	chr5	158822645	Autoimmune disease	Crohns disease	4.27	26.56	1.36
109	rs10975003	chr9	5213687	Autoimmune disease	Ulcerative colitis	34.58	7.40	-1.36
110	rs10927875	chr1	16299312	Cardiovascular	Dilated cardiomyopathy	0.00	15.58	1.35
111	rs3803662	chr16	52586341	Cancer	Breast cancer	0.00	15.44	1.35
112	rs4823006	chr22	29451671	Quantitative traits	Waist-hip ratio	0.00	15.40	1.34
113	rs12127588	chr1	198595506	Hematological parameters	Mean corpuscular hemoglobin	15.40	0.00	-1.34
114	rs7914558	chr10	104775908	Neurological behavioral	Schizophrenia	15.39	0.00	-1.34
115	rs2681019	chr2	23187504	Miscellaneous	Dialysis-related mortality	15.08	0.00	-1.33
116	rs11013962	chr10	24495586	Quantitative traits	Earlobes	0.00	15.07	1.33
117	rs6015450	chr20	57751117	Cardiovascular	Blood pressure	0.00	15.02	1.32
118	rs6015450	chr20	57751117	Cardiovascular	Diastolic blood pressure	0.00	15.02	1.32
119	rs6015450	chr20	57751117	Cardiovascular	Systolic blood pressure	0.00	15.02	1.32
120	rs2236653	chr11	126283785	Serum metabolites	Alkaline phosphatase	2.64	21.61	1.32
121	rs17234657	chr5	40401509	Autoimmune disease	Crohns disease	0.00	14.97	1.32
122	rs10508343	chr10	8150713	Drug metabolism	Response to treatment for acute lymphoblastic leukemia	0.00	14.95	1.32
123	rs2853676	chr5	1288547	Cancer	Glioma	0.00	14.86	1.31
124	rs950063	chr4	126431919	Neurological behavioral	Smoking behavior	0.00	14.56	1.30
125	rs5757949	chr22	40820151	Quantitative traits	Height	32.37	7.30	-1.29
126	rs56238310	chr3	111233239	Kidney lung liver	COPD	15.86	53.35	1.29
127	rs7071247	chr10	105257786	Hematological parameters	Platelet aggregation-epinephrine	0.00	14.36	1.28
128	rs2282978	chr7	92264410	Quantitative traits	Height	0.00	14.35	1.28
129	rs883079	chr12	114793240	Cardiovascular	QRS duration	14.31	0.00	-1.28
130	rs11085824	chr19	13001547	Hematological parameters	Mean corpuscular hemoglobin	6.59	30.04	1.27
131	rs1474747	chr1	159148513	Serum metabolites	MCP1	14.10	0.00	-1.27

132	rs2797685	chr1	7879063	Autoimmune disease	Crohns disease	13.94	0.00	-1.26
133	rs4900109	chr14	92763391	Quantitative traits	Iris characteristics	28.31	6.11	-1.25
134	rs6554809	chr5	13740976	Autoimmune disease	IgE grass sensitization	13.41	0.00	-1.23
135	rs470490	chr18	63031236	Cardiovascular	PR interval	43.61	12.95	-1.22
136	rs7315438	chr12	115891403	Cancer	Colorectal cancer	21.72	64.03	1.22
137	rs10748128	chr12	69827658	Quantitative traits	Height	22.70	4.16	-1.21
138	rs724743	chr14	76129591	Quantitative traits	Height	0.00	13.07	1.21
139	rs3091315	chr17	32593665	Autoimmune disease	Crohns disease	3.00	19.90	1.20
140	rs1052483	chr2	219934348	Quantitative traits	Height	12.98	0.00	-1.20
141	rs12946454	chr17	43208121	Cardiovascular	Systolic blood pressure	0.00	12.92	1.20
142	rs2854160	chr17	61977248	Quantitative traits	Height	0.00	12.90	1.20
143	rs2901964	chr1	15792426	Miscellaneous	Erectile dysfunction and prostate cancer treatment	8.17	31.42	1.19
144	rs2061333	chr19	44614208	Aging	Alzheimers disease	31.49	8.36	-1.18
145	rs12447690	chr16	88298124	Quantitative traits	Central corneal thickness	12.59	0.00	-1.18
146	rs1884537	chr14	101251989	Quantitative traits	Optic disc size disc	0.00	12.50	1.17
147	rs1168013	chr1	62996838	Serum metabolites	Triglycerides	12.35	0.00	-1.16
148	rs7517847	chr1	67681669	Autoimmune disease	Crohns disease	7.74	29.46	1.15
149	rs7517847	chr1	67681669	Autoimmune disease	Inflammatory bowel disease	7.74	29.46	1.15
150	rs11156606	chrX	153006495	Neurological behavioral	Biplolar disorder and schizophrenia	12.24	0.00	-1.15
151	rs4869742	chr6	151907748	Cancer	Chronic myeloid leukemia	23.31	4.99	-1.15
152	rs5742692	chr12	102799598	Quantitative traits	Height	0.00	12.15	1.15
153	rs2383378	chr14	33282470	Neurological behavioral	Anorexia nervosa	3.40	19.40	1.13
154	rs6971925	chr7	14445917	Drug metabolism	Response to treatment for acute lymphoblastic leukemia	0.00	11.92	1.13
155	rs10774610	chr12	111340243	Neurological behavioral	Drinking behavior	28.33	7.49	-1.13
156	rs11761231	chr7	131370039	Autoimmune disease	Rheumatoid arthritis	21.26	58.42	1.13
157	rs11171739	chr12	56470625	Diabetes	Type 1 diabetes	11.73	0.00	-1.12
158	rs1834640	chr15	48392165	Quantitative traits	Skin pigmentation	0.00	11.70	1.12

159	rs10501293	chr11	43086094	Neurological behavioral	Cognitive performance-SWM Strategy	0.00	11.69	1.12
160	rs9992101	chr4	77360431	Serum metabolites	Creatinine	10.41	34.22	1.12
161	rs16861329	chr3	186666461	Diabetes	Type 2 diabetes	11.65	0.00	-1.11
162	rs7528684	chr1	157670816	Diabetes	Type 1 diabetes autoantibodies	11.62	0.00	-1.11
163	rs1656402	chr2	233426526	Cancer	Lung cancer-non-small cell	0.00	11.61	1.11
164	rs54211	chr22	39687484	Cardiovascular	Sudden cardiac arrest	28.94	8.08	-1.11
165	rs7274811	chr20	32333181	Quantitative traits	Height	5.95	24.28	1.10
166	rs153091	chr16	13253956	Drug metabolism	Response to antipsychotic therapy perphenazine-triglycerides	55.63	20.58	-1.10
167	rs631208	chr16	9399724	Autoimmune disease	IgE grass sensitization	4.68	21.44	1.10
168	rs8049439	chr16	28837515	Autoimmune disease	Inflammatory bowel disease-early onset	8.70	30.06	1.10
169	rs12700667	chr7	25901639	Miscellaneous	Endometriosis	0.00	11.39	1.10
170	rs1436900	chr1	217058479	Quantitative traits	Optic disc size cup	0.00	11.38	1.10
171	rs3790268	chr20	19571581	Serum metabolites	Serum matrix metalloproteinase	11.32	0.00	-1.09
172	rs5998432	chr22	32745916	Aging	Alzheimers T-tau	11.32	0.00	-1.09
173	rs7819412	chr8	11045161	Serum metabolites	Triglycerides	11.30	0.00	-1.09
174	rs6441961	chr3	46352384	Autoimmune disease	Celiac disease	27.04	7.42	-1.09
175	rs224136	chr10	64470675	Autoimmune disease	Crohns disease	11.23	0.00	-1.09
176	rs7686660	chr4	144003159	Autoimmune disease	Asthma	0.00	11.20	1.08
177	rs17124318	chr1	63480730	Quantitative traits	BMI	0.00	11.12	1.08
178	rs17124318	chr1	63480730	Quantitative traits	Weight	0.00	11.12	1.08
179	rs12042938	chr1	231816842	Neurological behavioral	DISC1	11.12	0.00	-1.08
180	rs7025486	chr9	124422403	Cardiovascular	Abdominal aortic aneurysm	11.11	0.00	-1.08
181	rs10958476	chr8	57095808	Quantitative traits	Height	2.38	16.12	1.08
182	rs7910620	chr10	87846959	Cardiovascular	Cardiac structure-LV wall thickness	11.09	0.00	-1.08
183	rs2841498	chr9	87930045	Neurological behavioral	Partial epilepsies	3.79	19.06	1.08
184	rs604381	chr2	37977017	Neurological behavioral	Conduct disorder interaction	11.06	0.00	-1.07
185	rs6426749	chr1	22711473	Radiographic parameters	Bone mineral density-hip	10.98	0.00	-1.07

186	rs16933812	chr9	36969205	Cardiovascular	Blood pressure	0.00	10.81	1.06
187	rs1527243	chr2	123291022	Miscellaneous	Erectile dysfunction and prostate cancer treatment	3.69	18.42	1.05
188	rs2815752	chr1	72812440	Quantitative traits	BMI	41.41	14.79	-1.05
189	rs689	chr11	2182224	Diabetes	Type 1 diabetes autoantibodies	8.72	28.63	1.05
190	rs2072590	chr2	177042633	Cancer	Ovarian cancer	0.00	10.63	1.04
191	rs12518099	chr5	89546109	Diabetes	Type 2 diabetes	10.62	0.00	-1.04
192	rs11167764	chr5	141479065	Autoimmune disease	Crohns disease	0.00	10.60	1.04
193	rs1329424	chr1	196646176	Aging	Age-related macular degeneration	14.74	40.95	1.04
194	rs2855812	chr6	31472720	Kidney lung liver	FEV1/FVC	19.63	4.40	-1.04
195	rs11989122	chr8	118827839	Quantitative traits	Height	10.54	0.00	-1.04
196	rs9977499	chr21	28734997	Miscellaneous	Dialysis-related mortality	7.22	25.34	1.04
197	rs3825214	chr12	114795443	Cardiovascular	PR interval	0.00	10.42	1.03
198	rs3825214	chr12	114795443	Cardiovascular	QRS duration	0.00	10.42	1.03
199	rs3825214	chr12	114795443	Cardiovascular	QT interval	0.00	10.42	1.03
200	rs11977526	chr7	46008110	Serum metabolites	IGFBP3	4.34	19.27	1.03
201	rs2871865	chr15	99194896	Quantitative traits	Height	2.64	15.78	1.03
202	rs1574192	chr2	241308505	Neurological behavioral	Brain imaging in schizophrenia interaction	2.77	16.03	1.03
203	rs1223271	chr20	13296912	Aging	Parkinsons disease	10.34	0.00	-1.02
204	rs12579350	chr12	5797101	Neurological behavioral	Panic disorder	58.70	23.81	-1.02
205	rs1532624	chr16	57005479	Serum metabolites	Cholesterol	10.31	0.00	-1.02
206	rs1532624	chr16	57005479	Serum metabolites	HDL cholesterol	10.31	0.00	-1.02
207	rs2814778	chr1	159174683	Hematological parameters	Neutrophil count	9.19	28.95	1.02
208	rs2814778	chr1	159174683	Hematological parameters	WBC count	9.19	28.95	1.02
209	rs3791675	chr2	56111309	Quantitative traits	Height	0.00	10.28	1.02
210	rs1986655	chr4	125993502	Serum metabolites	Bilirubin	10.18	0.00	-1.01
211	rs9822268	chr3	49719729	Autoimmune disease	Ulcerative colitis	0.00	10.14	1.01
212	rs10514585	chr16	83284338	Neurological behavioral	Major depressive disorder	10.12	0.00	-1.01
213	rs12730292	chr1	79254762	Neurological behavioral	Bipolar disorder	10.10	0.00	-1.01
214	rs1497406	chr1	16505320	Serum metabolites	Gamma glutamyl transferase	39.38	14.62	-1.00

14 ESRRA:RXRA Round:3

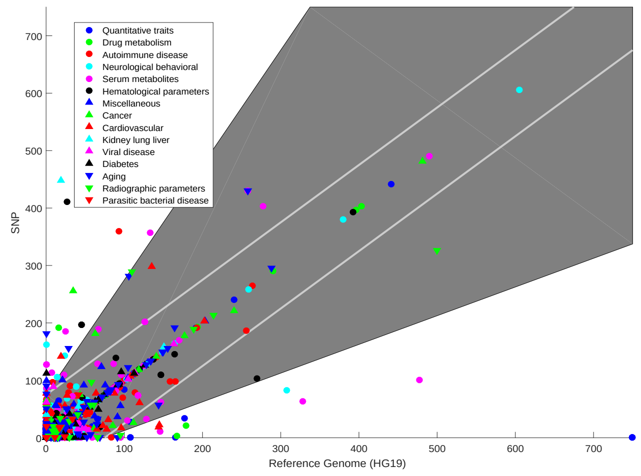

S.No.	SNP	Chr.	Position	Disease Class	Disease Trait	HG19	SNP	$log_2(\frac{SNP+\eta}{HG19+\eta})$
1	rs7138803	chr12	50247468	Quantitative traits	BMI	749.94	0.00	-3.46
2	rs7138803	chr12	50247468	Quantitative traits	Waist circumference	749.94	0.00	-3.46
3	rs7138803	chr12	50247468	Quantitative traits	Weight	749.94	0.00	-3.46
4	rs56238310	chr3	111233239	Kidney lung liver	COPD	18.75	447.45	2.48
5	rs11085824	chr19	13001547	Hematological parameters	Mean corpuscular hemoglobin	27.29	410.30	2.25
6	rs7153703	chr14	51919822	Aging	Alzheimers Total ventricular volume	0.00	181.59	1.77
7	rs10506821	chr12	80496923	Quantitative traits	Hip geometry	165.15	0.00	-1.68
8	rs1329650	chr10	93348120	Neurological behavioral	Smoking behavior	0.00	161.80	1.66
9	rs10741657	chr11	14914878	Serum metabolites	Vitamin D insufficiency	477.75	101.23	-1.65
10	rs153091	chr16	13253956	Drug metabolism	Response to antipsychotic therapy perphenazine-triglycerides	166.92	3.74	-1.62
11	rs7758229	chr6	160840252	Cancer	Colorectal cancer	34.26	255.90	1.60
12	rs16883019	chr6	19969638	Drug metabolism	Response to statin therapy-Triglyceride sum	15.98	192.22	1.55
13	rs4963452	chr11	61815803	Serum metabolites	Serum polyunsaturated fatty acids	328.58	62.97	-1.55
14	rs10885122	chr10	113042093	Serum metabolites	Fasting plasma glucose	0.00	127.40	1.43
15	rs10885122	chr10	113042093	Serum metabolites	Insulin resistance	0.00	127.40	1.43
16	rs2500535	chr6	149329267	Drug metabolism	Response to antidepressants-nortryptyline	178.85	21.01	-1.40
17	rs1000778	chr11	61655305	Serum metabolites	Sphingolipid concentrations	24.95	185.54	1.38

18	rs11761231	chr7	131370039	Autoimmune disease	Rheumatoid arthritis	92.92	359.57	1.37
19	rs17065323	chr13	44627788	Serum metabolites	Serum urate	145.32	11.17	-1.35
20	rs4462262	chr10	59189178	Diabetes	Diabetic retinopathy	0.00	111.93	1.32
21	rs6499640	chr16	53769677	Quantitative traits	BMI	107.79	0.00	-1.29
22	rs6499640	chr16	53769677	Quantitative traits	Weight	107.79	0.00	-1.29
23	rs12579350	chr12	5797101	Neurological behavioral	Panic disorder	307.71	82.87	-1.28
24	rs54211	chr22	39687484	Cardiovascular	Sudden cardiac arrest	143.74	18.49	-1.23
25	rs681900	chr2	75074967	Miscellaneous	Femoral neck bone geometry	0.00	99.72	1.22
26	rs9470004	chr6	35341850	Quantitative traits	Height	177.36	33.90	-1.21
27	rs10777317	chr12	91980374	Cardiovascular	Sudden cardiac arrest	18.79	141.30	1.21
28	rs2194980	chr12	115502718	Serum metabolites	Tyrosine	7.60	113.09	1.19
29	rs7034200	chr9	4289050	Serum metabolites	Fasting plasma glucose	94.88	0.00	-1.18
30	rs7034200	chr9	4289050	Serum metabolites	Insulin resistance	94.88	0.00	-1.18
31	rs2814778	chr1	159174683	Hematological parameters	Neutrophil count	45.80	197.09	1.17
32	rs2814778	chr1	159174683	Hematological parameters	WBC count	45.80	197.09	1.17
33	rs13038095	chr20	46425576	Cardiovascular	Atrial fibrillation	144.61	22.59	-1.17
34	rs10937470	chr3	191000808	Aging	Alzheimers Total ventricular volume	29.10	156.00	1.15
35	rs1223271	chr20	13296912	Aging	Parkinsons disease	91.36	0.00	-1.15
36	rs2034764	chr9	2742771	Aging	Amyotrophic lateral sclerosis-age of onset	0.00	90.61	1.14
37	rs1831521	chr9	93409357	Neurological behavioral	Cognitive performance-Nam	24.11	143.41	1.14
38	rs2126259	chr8	9185146	Serum metabolites	LDL cholesterol	0.00	86.69	1.11
39	rs4729260	chr7	96117918	Radiographic parameters	Bone mineral density-spine	95.01	3.94	-1.11
40	rs9512637	chr13	27920611	Neurological behavioral	Alcohol consumption	0.00	83.87	1.08
41	rs13106227	chr4	77418681	Autoimmune disease	Eosinophilic esophagitis pediatric	83.70	0.00	-1.08
42	rs2153960	chr6	108988184	Serum metabolites	IGF-1	133.44	356.61	1.05
43	rs668853	chr9	85311147	Autoimmune disease	Ulcerative colitis	8.72	97.02	1.04
44	rs3825214	chr12	114795443	Cardiovascular	PR interval	0.00	76.95	1.02
45	rs3825214	chr12	114795443	Cardiovascular	QRS duration	0.00	76.95	1.02
46	rs3825214	chr12	114795443	Cardiovascular	QT interval	0.00	76.95	1.02

47	rs10227331	chr7	157294938	Neurological behavioral	ADHD-Inattentive symptoms	14.60	105.36	1.01
48	rs1925690	chr6	87867063	Aging	Alzheimers Entorhinal cortical thickness	0.00	75.90	1.01
49	rs471364	chr9	15289578	Serum metabolites	HDL cholesterol	0.00	75.81	1.01

15 ESRRA+4 Round:3

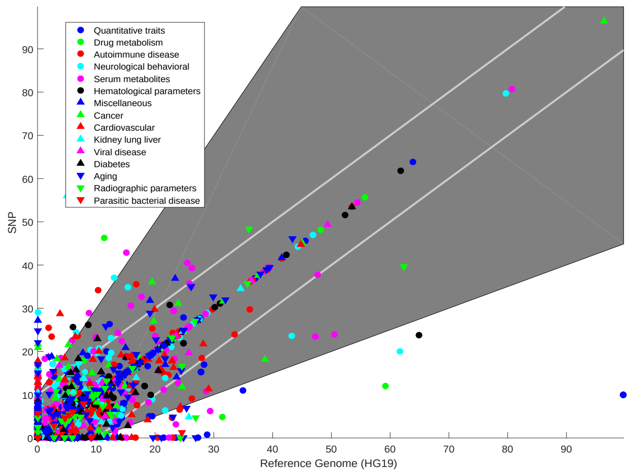

S.No.	SNP	Chr.	Position	Disease Class	Disease Trait	HG19	SNP	$log_2(\frac{SNP+\eta}{HG19+\eta})$
1	rs7138803	chr12	50247468	Quantitative traits	BMI	99.73	9.93	-2.46
2	rs7138803	chr12	50247468	Quantitative traits	Waist circumference	99.73	9.93	-2.46
3	rs7138803	chr12	50247468	Quantitative traits	Weight	99.73	9.93	-2.46
4	rs56238310	chr3	111233239	Kidney lung liver	COPD	4.98	55.97	2.14
5	rs1329650	chr10	93348120	Neurological behavioral	Smoking behavior	0.00	28.99	1.96
6	rs5757949	chr22	40820151	Quantitative traits	Height	27.28	0.00	-1.90
7	rs681900	chr2	75074967	Miscellaneous	Femoral neck bone geometry	0.00	27.22	1.90
8	rs3772255	chr3	156102734	Aging	Aging traits-biologic age	26.90	0.00	-1.88
9	rs10506821	chr12	80496923	Quantitative traits	Hip geometry	28.87	0.75	-1.85
10	rs10412199	chr19	3927771	Aging	Aging traits-age free from disease	24.78	0.00	-1.80
11	rs1925690	chr6	87867063	Aging	Alzheimers Entorhinal cortical thickness	0.00	24.75	1.80
12	rs7524102	chr1	22698447	Radiographic parameters	Bone mineral density-femoral neck	24.35	0.00	-1.78
13	rs7524102	chr1	22698447	Radiographic parameters	Bone mineral density-hip	24.35	0.00	-1.78
14	rs7524102	chr1	22698447	Radiographic parameters	Bone mineral density-spine	24.35	0.00	-1.78
15	rs7524102	chr1	22698447	Autoimmune disease	Ulcerative colitis	24.35	0.00	-1.78
16	rs6499640	chr16	53769677	Quantitative traits	BMI	23.48	0.00	-1.74
17	rs6499640	chr16	53769677	Quantitative traits	Weight	23.48	0.00	-1.74
18	rs10181042	chr2	61224259	Autoimmune disease	Crohns disease	22.98	0.00	-1.72
19	rs1474747	chr1	159148513	Serum metabolites	MCP1	22.42	0.00	-1.70

20	rs7153703	chr14	51919822	Aging	Alzheimers Total ventricular volume	0.00	22.12	1.68
21	rs7931342	chr11	68994497	Cancer	Prostate cancer	0.00	21.76	1.67
22	rs1424233	chr16	79682751	Quantitative traits	Obesity	21.48	0.00	-1.65
23	rs153091	chr16	13253956	Drug metabolism	Response to antipsychotic therapy perphenazine-triglycerides	59.15	12.01	-1.65
24	rs13095226	chr3	99396272	Aging	Age-related macular degeneration	21.13	0.00	-1.64
25	rs10411161	chr19	52372976	Cancer	Breast cancer	0.00	20.88	1.63
26	rs1451375	chr7	50622712	Parasitic bacterial disease	Malaria	24.55	1.24	-1.62
27	rs13314993	chr3	33015469	Autoimmune disease	Celiac disease	20.45	0.00	-1.61
28	rs8007846	chr14	66262963	Autoimmune disease	Multiple sclerosis-Brain Glutamate Concentrations	1.89	25.47	1.58
29	rs10260404	chr7	154210798	Aging	Amyotrophic lateral sclerosis	19.59	0.00	-1.57
30	rs10777317	chr12	91980374	Cardiovascular	Sudden cardiac arrest	3.79	28.71	1.49
31	rs2008242	chr4	5221538	Cardiovascular	PR segment	0.00	17.85	1.48
32	rs2500535	chr6	149329267	Drug metabolism	Response to antidepressants-nortryptyline	31.48	4.93	-1.47
33	rs2967605	chr19	8469738	Serum metabolites	HDL cholesterol	0.00	17.20	1.44
34	rs6887695	chr5	158822645	Autoimmune disease	Crohns disease	2.39	23.49	1.43
35	rs12203592	chr6	396321	Quantitative traits	Freckling	0.00	16.98	1.43
36	rs12203592	chr6	396321	Quantitative traits	Hair color	0.00	16.98	1.43
37	rs12203592	chr6	396321	Quantitative traits	Hair color-Black vs. blond hair color	0.00	16.98	1.43
38	rs12203592	chr6	396321	Quantitative traits	Hair color-Black vs. red hair color	0.00	16.98	1.43
39	rs12203592	chr6	396321	Aging	Progressive supranuclear palsy	0.00	16.98	1.43
40	rs13106227	chr4	77418681	Autoimmune disease	Eosinophilic esophagitis pediatric	16.73	0.00	-1.42
41	rs16883019	chr6	19969638	Drug metabolism	Response to statin therapy-Triglyceride sum	11.32	46.24	1.40
42	rs12625057	chr20	57790436	Drug metabolism	Response to antipsychotic therapy extrapyramidal side effects	0.00	16.01	1.38

43	rs688034	chr22	26689635	Cardiovascular	Coronary heart disease	15.97	0.00	-1.38
44	rs2185570	chr10	96751270	Serum metabolites	Serum dehydroepiandrosterone	23.55	3.07	-1.36
45	rs9512637	chr13	27920611	Neurological behavioral	Alcohol consumption	0.00	15.50	1.35
46	rs2194980	chr12	115502718	Serum metabolites	Tyrosine	1.17	18.35	1.34
47	rs3793917	chr10	124219275	Aging	Age-related macular degeneration	0.00	15.12	1.33
48	rs17319721	chr4	77368847	Serum metabolites	Creatinine	15.08	0.00	-1.33
49	rs724743	chr14	76129591	Quantitative traits	Height	0.00	15.06	1.33
50	rs7932354	chr11	46722221	Radiographic parameters	Bone mineral density-hip	26.87	4.74	-1.32
51	rs771767	chr3	101748638	Autoimmune disease	Multiple sclerosis	0.00	14.49	1.29
52	rs9303029	chr17	80408815	Serum metabolites	IGF-1	0.00	14.46	1.29
53	rs157350	chr5	156139569	Quantitative traits	Brachial circumference	14.41	0.00	-1.29
54	rs157350	chr5	156139569	Quantitative traits	Hip circumference	14.41	0.00	-1.29
55	rs9804317	chr10	130248926	Neurological behavioral	Animals	0.00	14.34	1.28
56	rs7765379	chr6	32680928	Autoimmune disease	Rheumatoid arthritis	14.28	0.00	-1.28
57	rs152528	chr5	142017860	Cardiovascular	Cardiac hypertrophy	14.25	0.00	-1.28
58	rs204993	chr6	32155581	Autoimmune disease	Asthma	0.00	14.19	1.27
59	rs6015450	chr20	57751117	Cardiovascular	Blood pressure	0.00	14.17	1.27
60	rs6015450	chr20	57751117	Cardiovascular	Diastolic blood pressure	0.00	14.17	1.27
61	rs6015450	chr20	57751117	Cardiovascular	Systolic blood pressure	0.00	14.17	1.27
62	rs1497406	chr1	16505320	Serum metabolites	Gamma glutamyl transferase	29.33	6.28	-1.27
63	rs7754840	chr6	20661250	Diabetes	Type 2 diabetes	14.12	0.00	-1.27
64	rs2042831	chr2	235857114	Aging	Longevity	1.96	18.84	1.27
65	rs7937	chr19	41302706	Kidney lung liver	COPD	25.74	4.85	-1.27
66	rs8099917	chr19	39743165	Viral disease	Chronic Hepatitis C infection	1.89	18.60	1.27
67	rs8099917	chr19	39743165	Viral disease	Response to hepatitis C treatment	1.89	18.60	1.27
68	rs2126259	chr8	9185146	Serum metabolites	LDL cholesterol	0.00	14.01	1.26
69	rs515135	chr2	21286057	Serum metabolites	LDL cholesterol	0.00	13.81	1.25
70	rs12579350	chr12	5797101	Neurological behavioral	Panic disorder	61.65	20.12	-1.25
71	rs2470893	chr15	75019449	Drug metabolism	Caffeine intake	0.00	13.66	1.24

72	rs2470893	chr15	75019449	Drug metabolism	coffee consumption	0.00	13.66	1.24
73	rs2823819	chr21	17828291	Neurological behavioral	ADHD	13.65	0.00	-1.24
74	rs4392868	chr8	96120072	Miscellaneous	Radiation response	13.60	0.00	-1.24
75	rs3120139	chr6	160741622	Serum metabolites	Lipoprotein A	13.27	0.00	-1.22
76	rs2901964	chr1	15792426	Miscellaneous	Erectile dysfunction and prostate cancer treatment	2.41	18.80	1.21
77	rs10227331	chr7	157294938	Neurological behavioral	ADHD-Inattentive symptoms	20.85	61.27	1.21
78	rs6120849	chr20	33730387	Serum metabolites	Protein C	12.99	0.00	-1.20
79	rs3825214	chr12	114795443	Cardiovascular	PR interval	0.00	12.95	1.20
80	rs3825214	chr12	114795443	Cardiovascular	QRS duration	0.00	12.95	1.20
81	rs3825214	chr12	114795443	Cardiovascular	QT interval	0.00	12.95	1.20
82	rs10508517	chr10	16997891	Cardiovascular	Diastolic blood pressure	12.75	0.00	-1.19
83	rs950063	chr4	126431919	Neurological behavioral	Smoking behavior	0.00	12.65	1.18
84	rs2797685	chr1	7879063	Autoimmune disease	Crohns disease	12.50	0.00	-1.17
85	rs12576775	chr11	79077193	Neurological behavioral	Bipolar disorder	12.47	0.00	-1.17
86	rs10975003	chr9	5213687	Autoimmune disease	Ulcerative colitis	22.84	4.75	-1.15
87	rs466639	chr1	165394882	Aging	Age at menarche	23.00	4.86	-1.15
88	rs11085824	chr19	13001547	Hematological parameters	Mean corpuscular hemoglobin	6.09	25.69	1.15
89	rs6092477	chr20	55991695	Hematological parameters	Mean corpuscular volume	64.91	23.81	-1.15
90	rs1052483	chr2	219934348	Quantitative traits	Height	12.06	0.00	-1.14
91	rs2738113	chr8	6829085	Miscellaneous	Endometriosis	11.90	0.00	-1.13
92	rs2722425	chr8	40484239	Serum metabolites	Fasting plasma glucose	0.00	11.81	1.12
93	rs8049439	chr16	28837515	Autoimmune disease	Inflammatory bowel disease-early onset	10.36	34.23	1.12
94	rs10927875	chr1	16299312	Cardiovascular	Dilated cardiomyopathy	0.00	11.61	1.11
95	rs2841498	chr9	87930045	Neurological behavioral	Partial epilepsies	2.57	17.07	1.11
96	rs7112513	chr11	117037361	Serum metabolites	Transferrin receptor	0.00	11.46	1.10
97	rs17086609	chr13	28929711	Neurological behavioral	Cognitive performance-IED	2.72	17.19	1.10
98	rs2815752	chr1	72812440	Quantitative traits	BMI	34.96	11.05	-1.10
99	rs2282978	chr7	92264410	Quantitative traits	Height	0.00	11.29	1.09
100	rs11013962	chr10	24495586	Quantitative traits	Earlobes	0.00	11.21	1.08
101	rs13387042	chr2	217905832	Cancer	Breast cancer	3.35	18.26	1.08

102	rs1000778	chr11	61655305	Serum metabolites	Sphingolipid concentrations	15.07	42.85	1.08
103	rs1831521	chr9	93409357	Neurological behavioral	Cognitive performance-Nam	6.53	24.53	1.06
104	rs2061333	chr19	44614208	Aging	Alzheimers disease	25.39	6.95	-1.06
105	rs10514585	chr16	83284338	Neurological behavioral	Major depressive disorder	10.76	0.00	-1.05
106	rs1880887	chr12	41721430	Serum metabolites	Alkaline phosphatase	8.74	28.90	1.05
107	rs16861329	chr3	186666461	Diabetes	Type 2 diabetes	10.75	0.00	-1.05
108	rs4765623	chr12	125320850	Cancer	Renal cell carcinoma	5.24	21.62	1.05
109	rs6441961	chr3	46352384	Autoimmune disease	Celiac disease	24.16	6.50	-1.05
110	rs12447690	chr16	88298124	Quantitative traits	Central corneal thickness	10.66	0.00	-1.05
111	rs1064395	chr19	19361735	Neurological behavioral	Bipolar disorder	10.64	0.00	-1.05
112	rs9992101	chr4	77360431	Serum metabolites	Creatinine	5.86	22.60	1.04
113	rs2718812	chr3	133399702	Serum metabolites	Transferrin	2.05	14.76	1.04
114	rs3734729	chr6	150570867	Kidney lung liver	FEV1/FVC	0.00	10.45	1.03
115	rs4964805	chr12	104192824	Neurological behavioral	ADHD	12.99	37.01	1.03
116	rs8109578	chr19	10213154	Serum metabolites	Thyroid stimulating hormone	3.94	18.44	1.03
117	rs4638289	chr11	18285774	Cardiovascular	Atherosclerosis	6.41	23.40	1.03
118	rs1884537	chr14	101251989	Quantitative traits	Optic disc size disc	0.00	10.32	1.02
119	rs11624704	chr14	78786077	Quantitative traits	Waist-hip ratio	0.00	10.28	1.02
120	rs11156606	chrX	153006495	Neurological behavioral	Biplolar disorder and schizophrenia	10.28	0.00	-1.02
121	rs4665630	chr2	23898317	Cardiovascular	Hypertension	18.71	4.19	-1.02
122	rs11099864	chr4	153893354	Aging	Amyotrophic lateral sclerosis	2.60	15.41	1.01
123	rs7578326	chr2	227020653	Diabetes	Type 2 diabetes	10.15	0.00	-1.01
124	rs2710833	chr4	169409958	Kidney lung liver	NAFLDH	0.00	10.15	1.01
125	rs1975174	chr19	22515251	Miscellaneous	Telomere length	10.13	0.00	-1.01
126	rs6503525	chr17	38095174	Neurological behavioral	Schizophrenia	10.09	0.00	-1.01
127	rs7517847	chr1	67681669	Autoimmune disease	Crohns disease	6.75	23.60	1.00
128	rs7517847	chr1	67681669	Autoimmune disease	Inflammatory bowel disease	6.75	23.60	1.00
129	rs7703051	chr5	74625487	Serum metabolites	LDL cholesterol	10.04	0.00	-1.00
130	rs2326810	chr6	6612467	Neurological behavioral	Major depressive disorder broad	10.00	0.00	-1.00

16 ESR1+5 Round:3

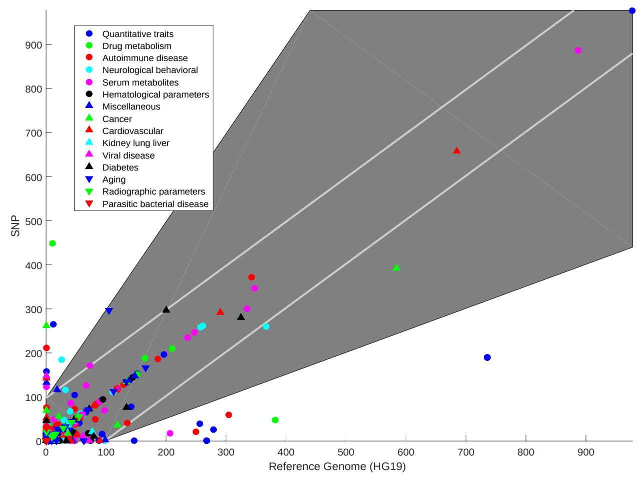

S.No.	SNP	Chr.	Position	Disease Class	Disease Trait	HG19	SNP	$log_2(\frac{SNP+\eta}{HG19+\eta})$
1	rs16909449	chr9	122861297	Drug metabolism	Response to statin therapy HDL sum	10.39	448.09	2.34
2	rs7138803	chr12	50247468	Quantitative traits	BMI	267.10	0.00	-1.90
3	rs7138803	chr12	50247468	Quantitative traits	Waist circumference	267.10	0.00	-1.90
4	rs7138803	chr12	50247468	Quantitative traits	Weight	267.10	0.00	-1.90
5	rs9868873	chr3	122730910	Cancer	Esophageal cancer	0.00	261.97	1.88
6	rs3825199	chr12	93976954	Quantitative traits	Height	11.72	264.32	1.73
7	rs2954038	chr8	126507389	Drug metabolism	Response to statin therapy-Triglyceride sum	382.30	47.95	-1.72
8	rs8049439	chr16	28837515	Autoimmune disease	Inflammatory bowel disease-early onset	0.00	211.12	1.66
9	rs9470004	chr6	35341850	Quantitative traits	Height	279.19	26.37	-1.60
10	rs12134279	chr1	197781198	Autoimmune disease	Primary biliary cirrhosis	250.39	20.37	-1.56
11	rs12896399	chr14	92773663	Quantitative traits	Eye color	735.51	189.97	-1.53
12	rs12896399	chr14	92773663	Quantitative traits	Eye color-blue vs. green eyes	735.51	189.97	-1.53
13	rs12896399	chr14	92773663	Quantitative traits	Eye color-green eyes	735.51	189.97	-1.53
14	rs12896399	chr14	92773663	Quantitative traits	Hair color	735.51	189.97	-1.53
15	rs12896399	chr14	92773663	Quantitative traits	Hair color-Black vs. blond hair color	735.51	189.97	-1.53
16	rs12896399	chr14	92773663	Quantitative traits	Hair color-blond vs. brown	735.51	189.97	-1.53
17	rs11013962	chr10	24495586	Quantitative traits	Earlobes	0.00	158.56	1.39
18	rs3120139	chr6	160741622	Serum metabolites	Lipoprotein A	206.19	18.18	-1.39
19	rs5751614	chr22	23593051	Quantitative traits	Height	256.23	39.53	-1.37

20	rs6441286	chr3	159728878	Autoimmune disease	Primary biliary cirrhosis	303.99	60.06	-1.35
21	rs17291045	chr4	161506897	Viral disease	HIV progression	0.00	148.05	1.33
22	rs26868	chr16	2249376	Quantitative traits	Height	147.14	0.00	-1.32
23	rs17114036	chr1	56962821	Cardiovascular	Coronary heart disease	0.00	143.61	1.30
24	rs681900	chr2	75074967	Miscellaneous	Femoral neck bone geometry	0.00	133.22	1.24
25	rs11856323	chr15	68892989	Neurological behavioral	Cognitive performance-PAL8	25.63	185.10	1.20
26	rs2194980	chr12	115502718	Serum metabolites	Tyrosine	0.00	122.17	1.17

17 ESR1+6 Round:3

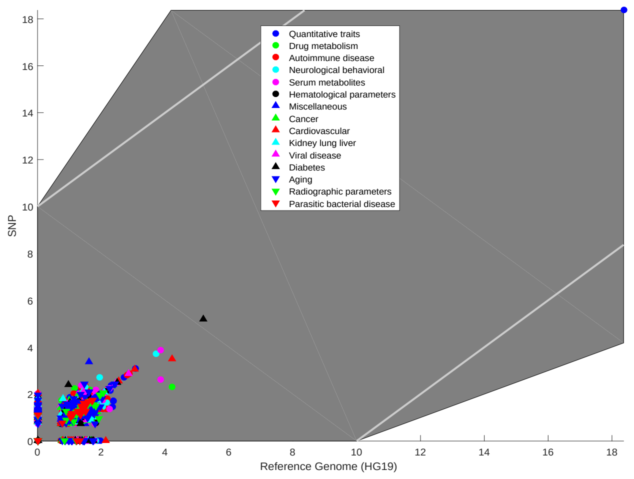

18 THRB Round:3

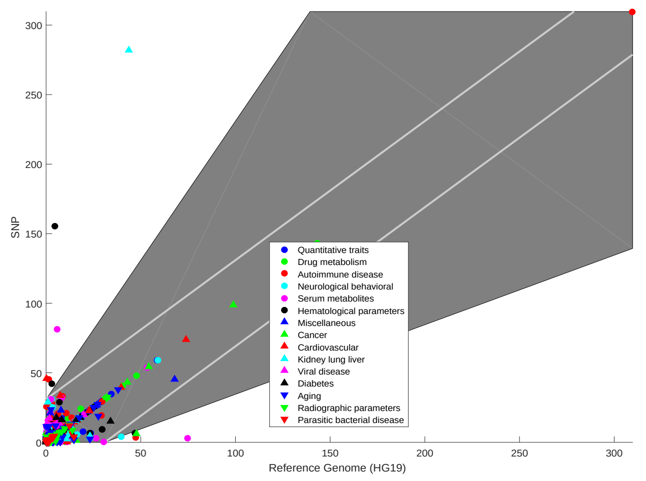

S.No.	SNP	Chr.	Position	Disease Class	Disease Trait	HG19	SNP	$log_2(\frac{SNP+\eta}{HG19+\eta})$
1	rs6995402	chr8	145005561	Hematological parameters	Platelet count	4.67	155.50	2.39
2	rs117607728	chr10	96058636	Kidney lung liver	COPD	43.87	281.93	2.06
3	rs2135319	chr3	72023700	Serum metabolites	Bilirubin	74.64	2.75	-1.65
4	rs2153960	chr6	108988184	Serum metabolites	IGF-1	5.73	81.24	1.61
5	rs8109578	chr19	10213154	Serum metabolites	Thyroid stimulating hormone	124.39	20.40	-1.60
6	rs1036476	chr15	48914775	Cardiovascular	Thoracic aortic aneurysm	0.00	45.86	1.31
7	rs11167764	chr5	141479065	Autoimmune disease	Crohns disease	1.38	45.07	1.23
8	rs231735	chr2	204693876	Autoimmune disease	Rheumatoid arthritis	47.44	3.25	-1.20
9	rs11085824	chr19	13001547	Hematological parameters	Mean corpuscular hemoglobin	3.14	42.11	1.10
10	rs721048	chr2	63131731	Cancer	Prostate cancer	47.84	6.32	-1.08
11	rs643381	chr6	139839423	Hematological parameters	Mean corpuscular volume	46.79	6.73	-1.04
12	rs3744064	chr17	75211208	Neurological behavioral	Cognitive performance-PAL6	39.51	3.96	-1.01

19 THRB:RXRA Round:3

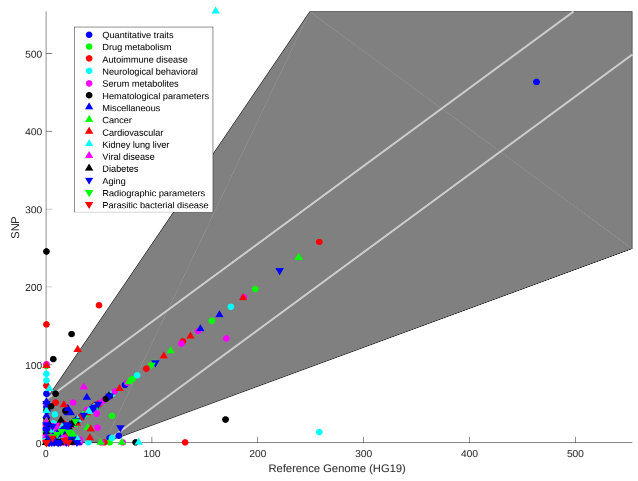

S.No.	SNP	Chr.	Position	Disease Class	Disease Trait	HG19	SNP	$log_2(\frac{SNP+\eta}{HG19+\eta})$
1	rs6995402	chr8	145005561	Hematological parameters	Platelet count	0.00	245.30	2.44
2	rs3744064	chr17	75211208	Neurological behavioral	Cognitive performance-PAL6	257.80	14.16	-2.17
3	rs11761231	chr7	131370039	Autoimmune disease	Rheumatoid arthritis	0.00	151.93	1.90
4	rs2153960	chr6	108988184	Serum metabolites	IGF-1	72.70	398.38	1.83
5	rs28493229	chr19	41224204	Autoimmune disease	Kawasaki disease	131.48	0.00	-1.75
6	rs117607728	chr10	96058636	Kidney lung liver	COPD	159.95	553.60	1.50
7	rs10885122	chr10	113042093	Serum metabolites	Fasting plasma glucose	0.00	100.49	1.49
8	rs10885122	chr10	113042093	Serum metabolites	Insulin resistance	0.00	100.49	1.49
9	rs6495122	chr15	75125645	Drug metabolism	Caffeine intake	0.00	99.23	1.48
10	rs6495122	chr15	75125645	Drug metabolism	coffee consumption	0.00	99.23	1.48
11	rs6495122	chr15	75125645	Cardiovascular	Diastolic blood pressure	0.00	99.23	1.48
12	rs7961894	chr12	122365583	Hematological parameters	Mean platelet volume	169.65	29.39	-1.41
13	rs7961894	chr12	122365583	Hematological parameters	Platelet count	169.65	29.39	-1.41
14	rs4794822	chr17	38156712	Hematological parameters	Neutrophil count	6.63	107.71	1.40
15	rs1329650	chr10	93348120	Neurological behavioral	Smoking behavior	0.00	88.59	1.38
16	rs2857595	chr6	31568469	Kidney lung liver	FEV1/FVC	87.19	0.00	-1.36
17	rs643381	chr6	139839423	Hematological parameters	Mean corpuscular volume	84.61	0.00	-1.34
18	rs11085824	chr19	13001547	Hematological parameters	Mean corpuscular hemoglobin	23.85	139.51	1.30
19	rs806276	chr6	91207351	Neurological behavioral	ADHD	0.00	80.23	1.29
20	rs11739663	chr5	594083	Autoimmune disease	Ulcerative colitis	0.00	73.18	1.22

21	rs10466868	chr12	131939920	Serum metabolites	Erythropoeitin	72.12	0.00	-1.20
22	rs721048	chr2	63131731	Cancer	Prostate cancer	71.60	0.00	-1.20
23	rs11676348	chr2	219010146	Autoimmune disease	Ulcerative colitis	50.36	176.92	1.14
24	rs1036429	chr12	96271428	Kidney lung liver	FEV1/FVC	3.03	69.85	1.10
25	rs17690232	chr4	55234825	Quantitative traits	Height	0.00	62.50	1.09
26	rs6724422	chr2	67976788	Drug metabolism	Response to antidepressants-bupropion	60.40	0.00	-1.06
27	rs1231206	chr17	2125605	Cardiovascular	Coronary heart disease	29.53	119.33	1.04

20 THRB:RXRA+7 Round:2

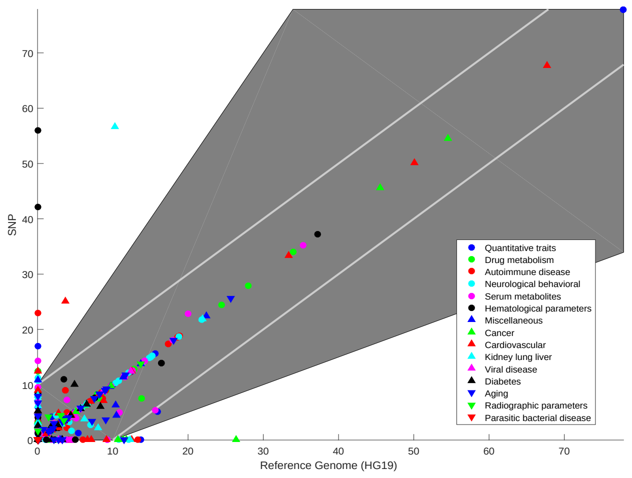

S.No.	SNP	Chr.	Position	Disease Class	Disease Trait	HG19	SNP	$log_2(\frac{SNP+\eta}{HG19+\eta})$
1	rs6995402	chr8	145005561	Hematological parameters	Platelet count	0.00	55.92	2.72
2	rs11085824	chr19	13001547	Hematological parameters	Mean corpuscular hemoglobin	0.00	42.19	2.38
3	rs721048	chr2	63131731	Cancer	Prostate cancer	26.41	0.00	-1.86
4	rs11761231	chr7	131370039	Autoimmune disease	Rheumatoid arthritis	0.00	23.01	1.72
5	rs117607728	chr10	96058636	Kidney lung liver	COPD	10.26	56.67	1.72
6	rs17690232	chr4	55234825	Quantitative traits	Height	0.00	16.96	1.43
7	rs1231206	chr17	2125605	Cardiovascular	Coronary heart disease	3.73	25.15	1.36
8	rs2153960	chr6	108988184	Serum metabolites	IGF-1	0.00	14.38	1.29
9	rs6794092	chr3	171558344	Quantitative traits	BMI	13.77	0.00	-1.25
10	rs28493229	chr19	41224204	Autoimmune disease	Kawasaki disease	13.36	0.00	-1.22
11	rs4725982	chr7	150637863	Cardiovascular	QT interval	12.55	0.00	-1.17
12	rs6495122	chr15	75125645	Drug metabolism	Caffeine intake	0.00	12.48	1.17
13	rs6495122	chr15	75125645	Drug metabolism	coffee consumption	0.00	12.48	1.17
14	rs6495122	chr15	75125645	Cardiovascular	Diastolic blood pressure	0.00	12.48	1.17
15	rs2857595	chr6	31568469	Kidney lung liver	FEV1/FVC	12.26	0.00	-1.15
16	rs2548145	chr5	40134777	Neurological behavioral	Alcohol use disorder	12.12	0.00	-1.15
17	rs9543325	chr13	73916628	Cancer	Pancreatic cancer	11.82	0.00	-1.13
18	rs11073328	chr15	38764843	Aging	Longevity	11.50	0.00	-1.10
19	rs2084881	chr17	46357120	Cancer	Ovarian cancer	11.39	0.00	-1.10
20	rs1329650	chr10	93348120	Neurological behavioral	Smoking behavior	0.00	11.24	1.09
21	rs9977499	chr21	28734997	Miscellaneous	Dialysis-related mortality	0.00	10.84	1.06
22	rs12575642	chr11	63979643	Neurological behavioral	ADHD	10.78	0.00	-1.06
23	rs716274	chr11	103418158	Cancer	Lung cancer-small cell	10.68	0.00	-1.05

24	rs6808138	chr3	161392576	Neurological behavioral	ADHD-Hyperactive-impulsive symptoms	0.00	10.68	1.05

21 THRB+7 Round:3

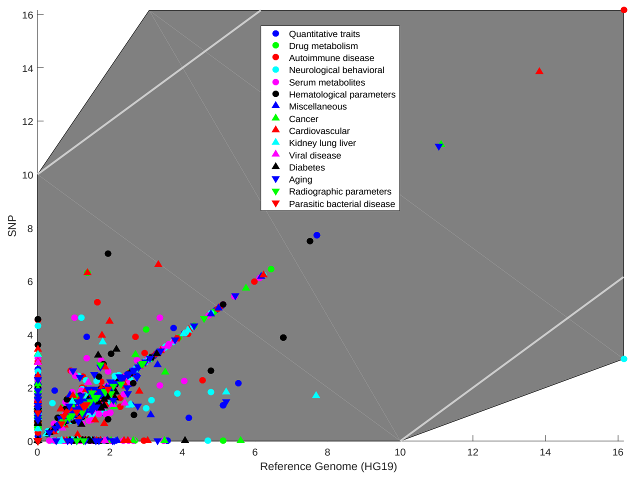

22 THRA Round:3

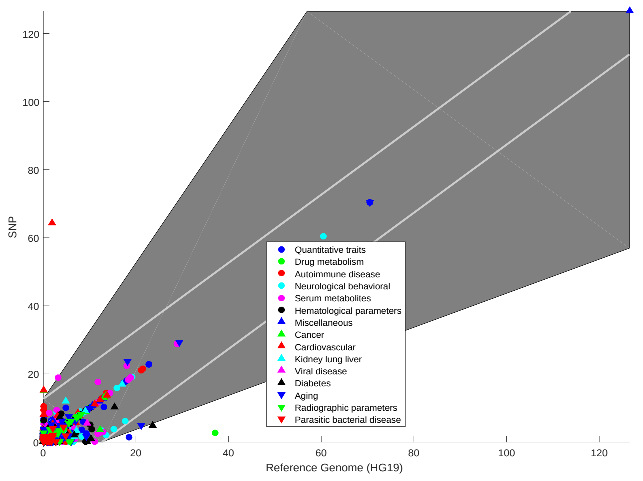

S.No.	SNP	Chr.	Position	Disease Class	Disease Trait	HG19	SNP	$log_2(\frac{SNP+\eta}{HG19+\eta})$
1	rs1036476	chr15	48914775	Cardiovascular	Thoracic aortic aneurysm	1.85	64.30	2.41
2	rs153091	chr16	13253956	Drug metabolism	Response to antipsychotic therapy perphenazine-triglycerides	37.02	2.63	-1.70
3	rs9470004	chr6	35341850	Quantitative traits	Height	18.56	1.45	-1.15
4	rs11710077	chr3	38657899	Cardiovascular	QRS duration	0.00	15.35	1.15
5	rs7315438	chr12	115891403	Cancer	Colorectal cancer	0.00	14.99	1.13
6	rs10786436	chr10	100300182	Diabetes	Type 1 diabetes	23.57	4.89	-1.05

23 THRA:RXRA Round:3

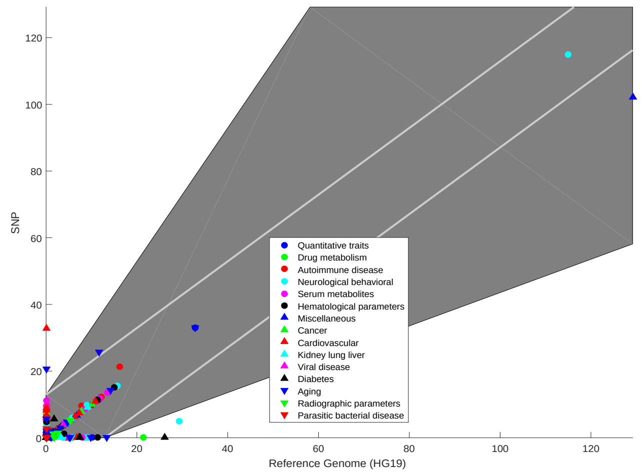

S.No.	SNP	Chr.	Position	Disease Class	Disease Trait	HG19	SNP	$log_2(\frac{SNP+\eta}{HG19+\eta})$
1	rs1036476	chr15	48914775	Cardiovascular	Thoracic aortic aneurysm	0.00	32.88	1.83
2	rs10786436	chr10	100300182	Diabetes	Type 1 diabetes	26.15	0.00	-1.60
3	rs153091	chr16	13253956	Drug metabolism	Response to antipsychotic therapy perphenazine-triglycerides	21.47	0.00	-1.41
4	rs7759938	chr6	105378954	Aging	Age at menarche	0.00	20.64	1.38
5	rs12575642	chr11	63979643	Neurological behavioral	ADHD	29.25	4.93	-1.24
6	rs7861820	chr9	108936674	Aging	Age at menarche	13.32	0.00	-1.02

24 THRA:RXRA+7 Round:2

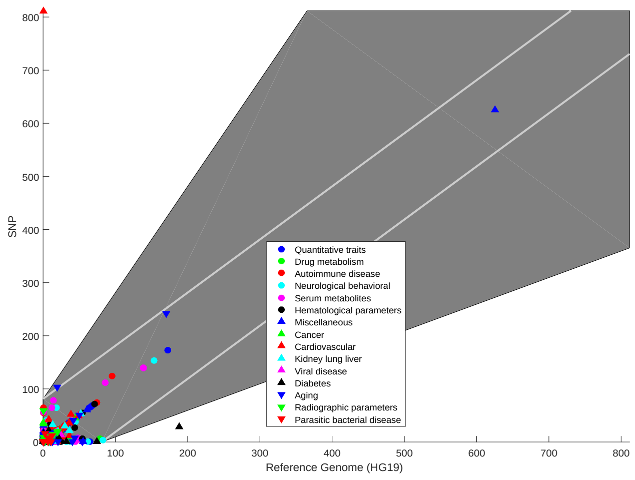

S.No.	SNP	Chr.	Position	Disease Class	Disease Trait	HG19	SNP	$log_2(\frac{SNP+\eta}{HG19+\eta})$
1	rs1036476	chr15	48914775	Cardiovascular	Thoracic aortic aneurysm	0.00	811.73	3.46
2	rs10786436	chr10	100300182	Diabetes	Type 1 diabetes	188.62	27.94	-1.31

25 THRA+7 Round:3

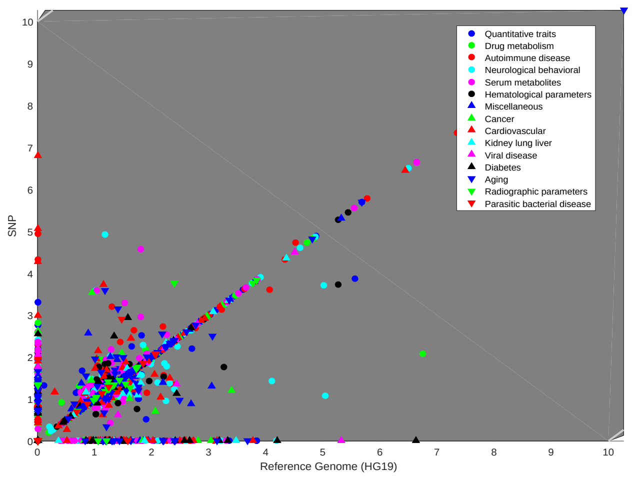

26 RARG Round:3

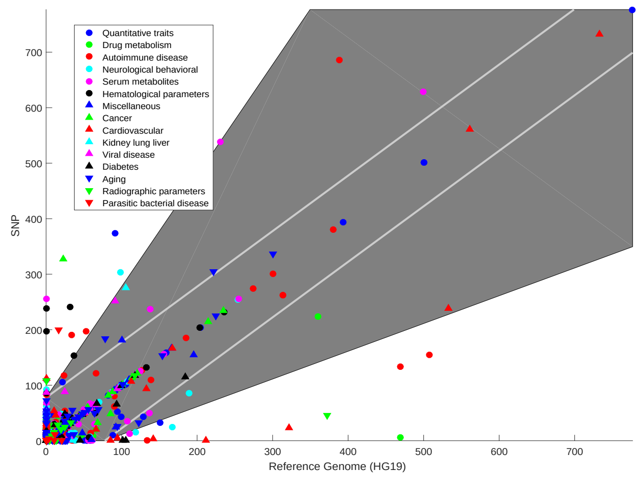

S.No.	SNP	Chr.	Position	Disease Class	Disease Trait	HG19	SNP	$log_2(\frac{SNP+\eta}{HG19+\eta})$
1	rs153091	chr16	13253956	Drug metabolism	Response to antipsychotic therapy perphenazine-triglycerides	468.99	6.18	-2.70
2	rs6720394	chr2	111989372	Autoimmune disease	Primary sclerosing cholangitis	46.57	570.26	2.38
3	rs1880887	chr12	41721430	Serum metabolites	Alkaline phosphatase	0.00	256.02	2.10
4	rs11085824	chr19	13001547	Hematological parameters	Mean corpuscular hemoglobin	0.00	238.86	2.03
5	rs7315438	chr12	115891403	Cancer	Colorectal cancer	23.11	327.32	2.01
6	rs1395821	chr4	148047550	Cardiovascular	Coronary heart disease	322.04	23.13	-1.99
7	rs688034	chr22	26689635	Cardiovascular	Coronary heart disease	211.79	0.00	-1.90
8	rs4811196	chr20	36469694	Radiographic parameters	Bone mineral density-trochanter	371.94	45.87	-1.86
9	rs10017284	chr4	121513215	Hematological parameters	F-cell distribution	0.00	197.34	1.82
10	rs8005962	chr14	96027153	Parasitic bacterial disease	Tuberculosis	16.22	199.19	1.56
11	rs6995402	chr8	145005561	Hematological parameters	Platelet count	31.97	240.45	1.54
12	rs28493229	chr19	41224204	Autoimmune disease	Kawasaki disease	134.04	0.00	-1.45
13	rs4937126	chr11	126281897	Cardiovascular	Coronary heart disease	142.00	3.45	-1.44
14	rs2429582	chr7	122202593	Quantitative traits	Brain structure	91.05	373.67	1.42
15	rs11243676	chr9	135096767	Autoimmune disease	Systemic lupus erythematosus	469.26	133.52	-1.37
16	rs6441286	chr3	159728878	Autoimmune disease	Primary biliary cirrhosis	507.05	154.57	-1.33
17	rs17609940	chr6	35034800	Cardiovascular	Coronary heart disease	0.00	111.77	1.29
18	rs2194980	chr12	115502718	Serum metabolites	Tyrosine	0.00	109.48	1.27
19	rs17309827	chr6	3433318	Autoimmune disease	Crohns disease	33.56	190.01	1.27

20	rs8049439	chr16	28837515	Autoimmune disease	Inflammatory bowel disease-early onset	0.00	108.96	1.26
21	rs12061304	chr1	111237685	Neurological behavioral	Panic disorder	166.75	24.41	-1.26
22	rs1038304	chr6	151933175	Radiographic parameters	Bone mineral density-hip	0.00	108.02	1.26
23	rs1038304	chr6	151933175	Radiographic parameters	Bone mineral density-spine	0.00	108.02	1.26
24	rs9866141	chr3	156950579	Diabetes	Diabetic retinopathy	105.25	0.48	-1.23
25	rs2903692	chr16	11238783	Diabetes	Type 1 diabetes	101.95	0.00	-1.21
26	rs17086609	chr13	28929711	Neurological behavioral	Cognitive performance-IED	0.00	92.68	1.13
27	rs7017212	chr8	10022938	Neurological behavioral	Schizophrenia	98.76	303.84	1.11
28	rs6139030	chr20	3187733	Viral disease	Response to hepatitis C treatment	0.00	87.62	1.09
29	rs4704970	chr5	155500992	Autoimmune disease	Multiple sclerosis	52.93	197.08	1.07
30	rs12579350	chr12	5797101	Neurological behavioral	Panic disorder	118.19	15.44	-1.07
31	rs12143842	chr1	162033890	Cardiovascular	QT interval	85.37	0.00	-1.07
32	rs9374080	chr6	109616420	Hematological parameters	Mean corpuscular volume	0.00	85.37	1.07
33	rs10447248	chr5	107915736	Serum metabolites	Adiponectin	110.67	12.75	-1.06
34	rs2856321	chr12	11855773	Quantitative traits	Height	150.69	32.19	-1.06
35	rs12190287	chr6	134214525	Cardiovascular	Coronary heart disease	94.30	5.09	-1.05
36	rs1975974	chr17	21707060	Autoimmune disease	Psoriasis	0.00	83.06	1.05
37	rs7527798	chr1	207872290	Hematological parameters	Erythrocyte sedimentation rate	36.59	152.87	1.01
38	rs12459897	chr19	31596778	Serum metabolites	Serum polyunsaturated fatty acids	230.40	538.84	1.00

27 RARB Round:3

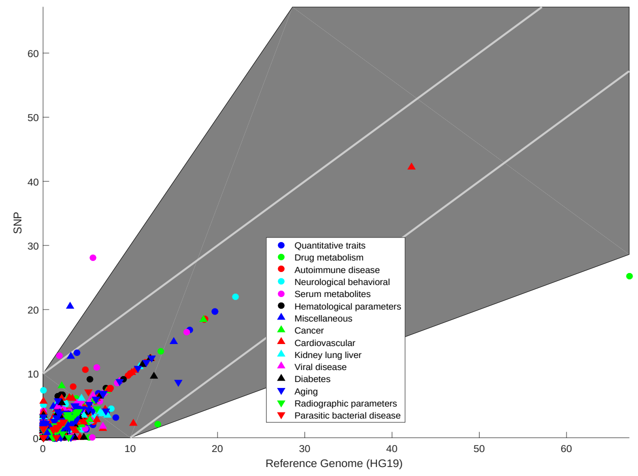

S.No.	SNP	Chr.	Position	Disease Class	Disease Trait	HG19	SNP	$log_2(\frac{SNP+\eta}{HG19+\eta})$
1	rs511154	chr3	135950921	Serum metabolites	Fibrinogen	5.75	28.10	1.27
2	rs1527243	chr2	123291022	Miscellaneous	Erectile dysfunction and prostate cancer treatment	3.11	20.51	1.22
3	rs10221833	chr2	165577164	Drug metabolism	Response to statin therapy-Triglyceride sum	67.18	25.23	-1.13

28 RARA Round:3

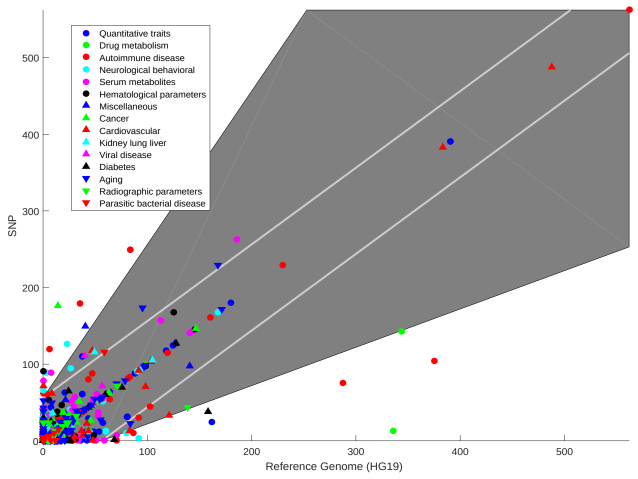

S.No.	SNP	Chr.	Position	Disease Class	Disease Trait	HG19	SNP	$log_2(\frac{SNP+\eta}{HG19+\eta})$
1	rs153091	chr16	13253956	Drug metabolism	Response to antipsychotic therapy perphenazine-triglycerides	336.17	12.65	-2.51
2	rs7315438	chr12	115891403	Cancer	Colorectal cancer	14.37	176.52	1.72
3	rs8049439	chr16	28837515	Autoimmune disease	Inflammatory bowel disease-early onset	6.11	119.40	1.49
4	rs9470004	chr6	35341850	Quantitative traits	Height	162.14	24.06	-1.44
5	rs6441286	chr3	159728878	Autoimmune disease	Primary biliary cirrhosis	375.40	104.64	-1.42
6	rs11243676	chr9	135096767	Autoimmune disease	Systemic lupus erythematosus	287.93	74.95	-1.39
7	rs11085824	chr19	13001547	Hematological parameters	Mean corpuscular hemoglobin	0.00	90.82	1.39
8	rs6720394	chr2	111989372	Autoimmune disease	Primary sclerosing cholangitis	35.54	179.17	1.36
9	rs4356203	chr11	17160148	Neurological behavioral	Schizophrenia	91.85	3.26	-1.32
10	rs16977195	chr15	86984240	Neurological behavioral	Schizophrenia	3.29	89.21	1.29
11	rs1880887	chr12	41721430	Serum metabolites	Alkaline phosphatase	0.00	78.40	1.26
12	rs7017212	chr8	10022938	Neurological behavioral	Schizophrenia	23.24	125.89	1.20
13	rs17609940	chr6	35034800	Cardiovascular	Coronary heart disease	0.00	71.93	1.19
14	rs10786436	chr10	100300182	Diabetes	Type 1 diabetes	158.41	38.06	-1.19
15	rs2194980	chr12	115502718	Serum metabolites	Tyrosine	7.98	88.99	1.18
16	rs1557351	chr18	54752314	Autoimmune disease	Multiple sclerosis	70.22	0.00	-1.17
17	rs1800693	chr12	6440009	Autoimmune disease	Multiple sclerosis	83.58	249.23	1.13
18	rs1800693	chr12	6440009	Autoimmune disease	Primary biliary cirrhosis	83.58	249.23	1.13
19	rs17086609	chr13	28929711	Neurological behavioral	Cognitive performance-IED	0.00	66.02	1.12

20	rs28493229	chr19	41224204	Autoimmune disease	Kawasaki disease	86.61	10.15	-1.11
21	rs9866141	chr3	156950579	Diabetes	Diabetic retinopathy	68.68	1.88	-1.10
22	rs2439312	chr8	32412359	Miscellaneous	Dialysis-related mortality	40.75	149.69	1.09
23	rs8007846	chr14	66262963	Autoimmune disease	Multiple sclerosis-Brain Glutamate Concentrations	0.00	62.10	1.07
24	rs2185570	chr10	96751270	Serum metabolites	Serum dehydroepiandrosterone	59.17	0.00	-1.04
25	rs12579350	chr12	5797101	Neurological behavioral	Panic disorder	80.11	10.26	-1.04
26	rs1395821	chr4	148047550	Cardiovascular	Coronary heart disease	83.01	12.78	-1.01
27	rs10221833	chr2	165577164	Drug metabolism	Response to statin therapy-Triglyceride sum	343.59	142.71	-1.01

29 RARG+8 Round:2

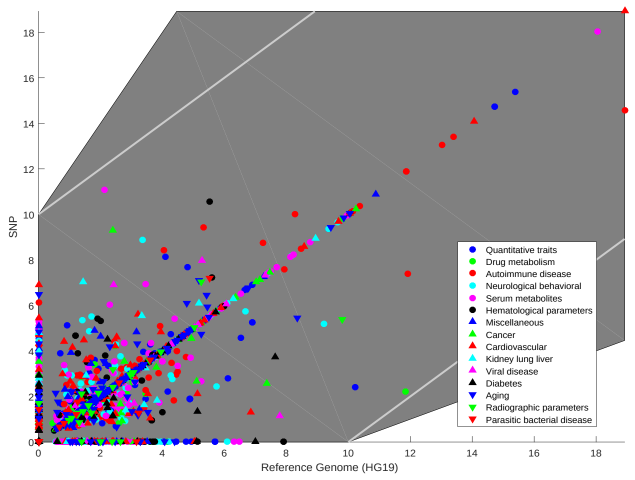

30 RARB+8 Round:3

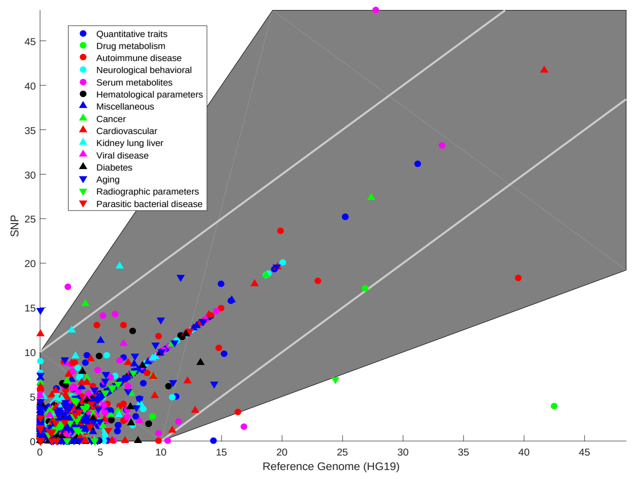

S.No.	SNP	Chr.	Position	Disease Class	Disease Trait	HG19	SNP	$log_2(\frac{SNP+\eta}{HG19+\eta})$
1	rs153091	chr16	13253956	Drug metabolism	Response to antipsychotic therapy perphenazine-triglycerides	42.51	3.91	-1.92
2	rs2042831	chr2	235857114	Aging	Longevity	0.00	14.70	1.30
3	rs2580816	chr2	232797966	Quantitative traits	Height	14.30	0.00	-1.28
4	rs8038465	chr15	73978337	Serum metabolites	Gamma glutamyl transferase	16.87	1.59	-1.21
5	rs2194980	chr12	115502718	Serum metabolites	Tyrosine	2.30	17.38	1.15
6	rs17609940	chr6	35034800	Cardiovascular	Coronary heart disease	0.00	12.06	1.14
7	rs6720394	chr2	111989372	Autoimmune disease	Primary sclerosing cholangitis	9.18	30.22	1.07
8	rs1834481	chr11	112023827	Serum metabolites	Interleukin-18 levels	10.58	0.00	-1.04
9	rs4811196	chr20	36469694	Radiographic parameters	Bone mineral density-trochanter	24.40	6.90	-1.03

31 RARA+8 Round:3

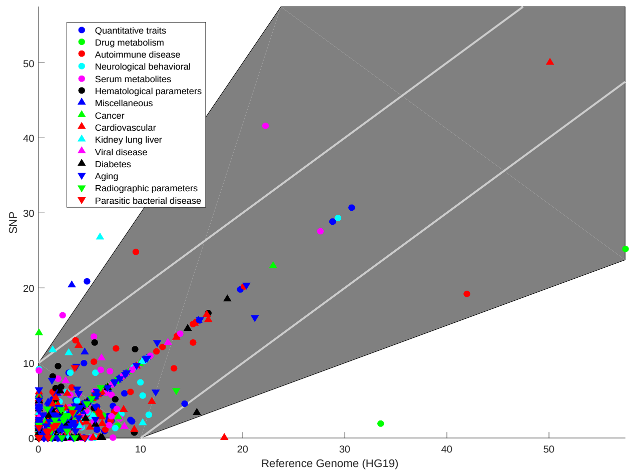

S.No.	SNP	Chr.	Position	Disease Class	Disease Trait	HG19	SNP	$log_2(\frac{SNP+\eta}{HG19+\eta})$
1	rs153091	chr16	13253956	Drug metabolism	Response to antipsychotic therapy perphenazine-triglycerides	33.52	1.91	-1.87
2	rs1550576	chr15	58213414	Cardiovascular	Hypertension	18.22	0.00	-1.50
3	rs7315438	chr12	115891403	Cancer	Colorectal cancer	0.00	14.00	1.26
4	rs117607728	chr10	96058636	Kidney lung liver	COPD	6.01	26.76	1.20
5	rs1527243	chr2	123291022	Miscellaneous	Erectile dysfunction and prostate cancer treatment	3.28	20.38	1.19
6	rs2194980	chr12	115502718	Serum metabolites	Tyrosine	2.32	16.32	1.09
7	rs2429582	chr7	122202593	Quantitative traits	Brain structure	4.74	20.87	1.07

32 RARG:RXRA Round:3

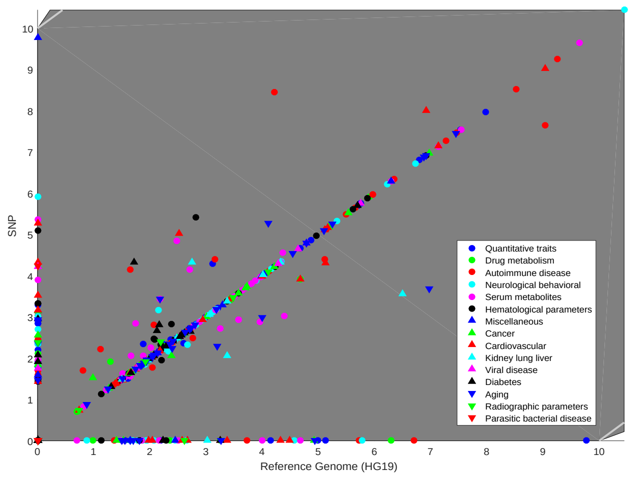

33 RARB:RXRA Round:3

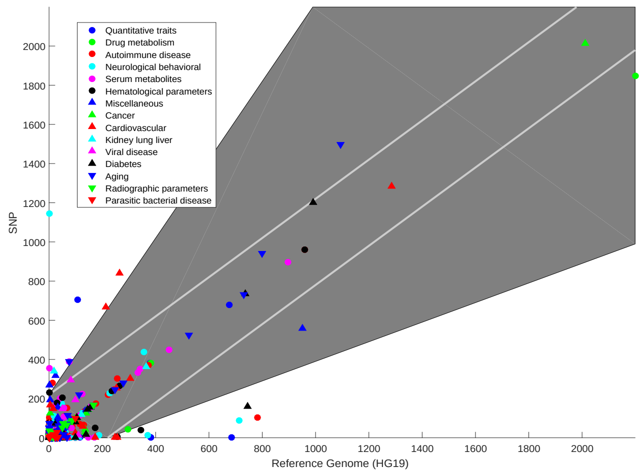

S.No.	SNP	Chr.	Position	Disease Class	Disease Trait	HG19	SNP	$log_2(\frac{SNP+\eta}{HG19+\eta})$
1	rs17086609	chr13	28929711	Neurological behavioral	Cognitive performance-IED	0.00	1144.06	2.63
2	rs571312	chr18	57839769	Quantitative traits	BMI	686.08	0.00	-2.04
3	rs12459897	chr19	31596778	Serum metabolites	Serum polyunsaturated fatty acids	232.46	1299.33	1.75
4	rs6441286	chr3	159728878	Autoimmune disease	Primary biliary cirrhosis	783.43	102.64	-1.64
5	rs12579350	chr12	5797101	Neurological behavioral	Panic disorder	713.20	89.05	-1.59
6	rs2429582	chr7	122202593	Quantitative traits	Brain structure	106.54	705.93	1.50
7	rs6499640	chr16	53769677	Quantitative traits	BMI	382.88	0.00	-1.45
8	rs6499640	chr16	53769677	Quantitative traits	Weight	382.88	0.00	-1.45
9	rs2194980	chr12	115502718	Serum metabolites	Tyrosine	0.00	355.75	1.39
10	rs10786436	chr10	100300182	Diabetes	Type 1 diabetes	744.97	161.11	-1.34
11	rs12061304	chr1	111237685	Neurological behavioral	Panic disorder	370.32	14.05	-1.34
12	rs1036429	chr12	96271428	Kidney lung liver	FEV1/FVC	18.87	341.71	1.23
13	rs681900	chr2	75074967	Miscellaneous	Femoral neck bone geometry	0.00	267.75	1.15
14	rs16826658	chr1	22485871	Miscellaneous	Endometriosis	24.71	318.29	1.14
15	rs643381	chr6	139839423	Hematological parameters	Mean corpuscular volume	344.68	37.68	-1.13
16	rs11781551	chr8	123408091	Cardiovascular	Internal carotid intimal medial thickness	265.76	838.84	1.12
17	rs12210810	chr6	118653204	Cardiovascular	QT interval	254.78	0.00	-1.11
18	rs6720394	chr2	111989372	Autoimmune disease	Primary sclerosing cholangitis	12.18	279.61	1.11
19	rs10508517	chr10	16997891	Cardiovascular	Diastolic blood pressure	246.52	0.00	-1.08
20	rs10758658	chr9	4856877	Hematological parameters	Mean corpuscular hemoglobin	256.80	6.37	-1.08
21	rs10758658	chr9	4856877	Hematological parameters	Mean corpuscular volume	256.80	6.37	-1.08

22	rs9494145	chr6	135432552	Hematological parameters	Mean corpuscular volume	0.00	232.46	1.04
23	rs4528684	chr19	14351574	Cardiovascular	Heart failure motality-EA	212.27	668.41	1.04
24	rs157580	chr19	45395266	Aging	Alzheimers disease	76.27	387.72	1.04
25	rs157580	chr19	45395266	Aging	Alzheimers-AB1-42	76.27	387.72	1.04
26	rs157580	chr19	45395266	Serum metabolites	HDL cholesterol	76.27	387.72	1.04
27	rs157580	chr19	45395266	Serum metabolites	LDL cholesterol	76.27	387.72	1.04

34 RARA:RXRA Round:3

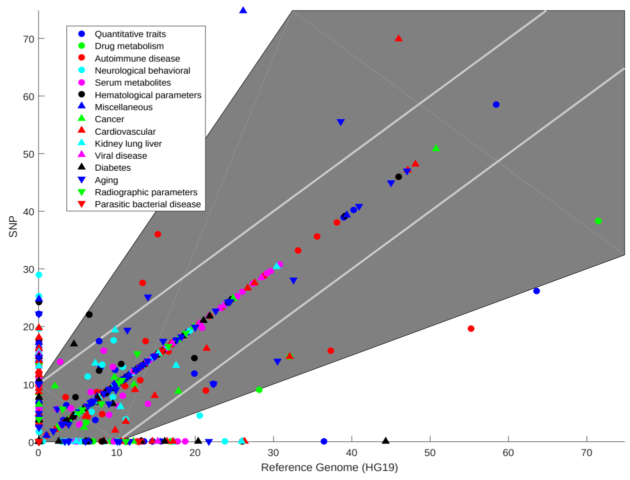

S.No.	SNP	Chr.	Position	Disease Class	Disease Trait	HG19	SNP	$log_2(\frac{SNP+\eta}{HG19+\eta})$
1	rs10786436	chr10	100300182	Diabetes	Type 1 diabetes	44.36	0.00	-2.44
2	rs2281845	chr1	201081943	Quantitative traits	Primary tooth development time to first tooth eruption	36.43	0.00	-2.21
3	rs2194980	chr12	115502718	Serum metabolites	Tyrosine	5.49	51.11	1.98
4	rs6017291	chr20	42854134	Neurological behavioral	Delayed Story Recall	0.00	29.03	1.96
5	rs688034	chr22	26689635	Cardiovascular	Coronary heart disease	26.36	0.00	-1.86
6	rs10758658	chr9	4856877	Hematological parameters	Mean corpuscular hemoglobin	26.07	0.00	-1.85
7	rs10758658	chr9	4856877	Hematological parameters	Mean corpuscular volume	26.07	0.00	-1.85
8	rs2855812	chr6	31472720	Kidney lung liver	FEV1/FVC	25.94	0.00	-1.85
9	rs17086609	chr13	28929711	Neurological behavioral	Cognitive performance-IED	0.00	25.32	1.82
10	rs1527243	chr2	123291022	Miscellaneous	Erectile dysfunction and prostate cancer treatment	0.00	24.72	1.80
11	rs9494145	chr6	135432552	Hematological parameters	Mean corpuscular volume	0.00	24.20	1.77
12	rs2905072	chr9	135845035	Neurological behavioral	Bipolar disorder	23.77	0.00	-1.76
13	rs7137869	chr12	119989646	Aging	Aging traits-walking speed	0.00	22.17	1.69
14	rs2429582	chr7	122202593	Quantitative traits	Brain structure	0.00	22.17	1.69
15	rs294636	chr3	14899778	Aging	Longevity	21.74	0.00	-1.67
16	rs358806	chr3	55313400	Diabetes	Type 2 diabetes	20.38	0.00	-1.60
17	rs7527798	chr1	207872290	Hematological parameters	Erythrocyte sedimentation rate	11.95	55.51	1.58
18	rs17609940	chr6	35034800	Cardiovascular	Coronary heart disease	0.00	19.72	1.57
19	rs1329650	chr10	93348120	Neurological behavioral	Smoking behavior	0.00	19.60	1.57
20	rs3120139	chr6	160741622	Serum metabolites	Lipoprotein A	18.72	0.00	-1.52

21	rs1395479	chr4	178318191	Cardiovascular	Heart rate variability LF HF	0.00	18.14	1.49
22	rs2185570	chr10	96751270	Serum metabolites	Serum dehydroepiandrosterone	17.75	0.00	-1.47
23	rs17655730	chr11	270715	Hematological parameters	Mean platelet volume	0.00	17.70	1.47
24	rs4547811	chr4	146794621	Serum metabolites	Gamma glutamyl transferase	0.00	17.45	1.46
25	rs6459804	chr7	157510195	Neurological behavioral	Schizophrenia	17.38	0.00	-1.45
26	rs4996815	chr13	106651661	Neurological behavioral	Biplolar disorder and schizophrenia	17.29	0.00	-1.45
27	rs681900	chr2	75074967	Miscellaneous	Femoral neck bone geometry	0.00	17.26	1.45
28	rs3118914	chr13	51116901	Quantitative traits	Height	17.21	0.00	-1.44
29	rs724743	chr14	76129591	Quantitative traits	Height	0.00	17.15	1.44
30	rs6660565	chr1	171261193	Cardiovascular	Sudden cardiac arrest	17.13	0.00	-1.44
31	rs153091	chr16	13253956	Drug metabolism	Response to antipsychotic therapy perphenazine-triglycerides	16.84	0.00	-1.42
32	rs2527866	chr7	157090296	Cardiovascular	Systolic blood pressure	16.41	0.00	-1.40
33	rs10037512	chr5	88354675	Quantitative traits	Height	0.00	16.36	1.40
34	rs10411161	chr19	52372976	Cancer	Breast cancer	0.00	16.22	1.39
35	rs11047543	chr12	24788339	Cardiovascular	PR interval	0.00	16.15	1.39
36	rs9866141	chr3	156950579	Diabetes	Diabetic retinopathy	16.15	0.00	-1.39
37	rs28927680	chr11	116619073	Serum metabolites	Triglycerides	0.00	16.03	1.38
38	rs1016988	chr5	131744574	Serum metabolites	Fibrinogen	0.00	15.68	1.36
39	rs3791950	chr2	218729865	Quantitative traits	Height	15.60	0.00	-1.36
40	rs1036429	chr12	96271428	Kidney lung liver	FEV1/FVC	0.00	15.54	1.35
41	rs41360247	chr2	44073656	Serum metabolites	Campesterol	0.00	15.32	1.34
42	rs641525	chr8	2740502	Kidney lung liver	COPD	15.22	0.00	-1.33
43	rs6139030	chr20	3187733	Viral disease	Response to hepatitis C treatment	0.00	15.18	1.33
44	rs558718	chr19	7909883	Viral disease	HIV progression	15.18	0.00	-1.33
45	rs6887695	chr5	158822645	Autoimmune disease	Crohns disease	0.00	14.98	1.32
46	rs7202877	chr16	75247245	Diabetes	Type 1 diabetes	0.00	14.76	1.31
47	rs536841	chr11	85787824	Aging	Alzheimers disease	0.00	14.68	1.30
48	rs515135	chr2	21286057	Serum metabolites	LDL cholesterol	0.00	14.59	1.30

49	rs12210810	chr6	118653204	Cardiovascular	QT interval	14.58	0.00	-1.30
50	rs7138803	chr12	50247468	Quantitative traits	BMI	14.53	0.00	-1.29
51	rs7138803	chr12	50247468	Quantitative traits	Waist circumference	14.53	0.00	-1.29
52	rs7138803	chr12	50247468	Quantitative traits	Weight	14.53	0.00	-1.29
53	rs9977499	chr21	28734997	Miscellaneous	Dialysis-related mortality	0.00	14.25	1.28
54	rs10777317	chr12	91980374	Cardiovascular	Sudden cardiac arrest	0.00	14.06	1.27
55	rs7315438	chr12	115891403	Cancer	Colorectal cancer	0.00	14.06	1.27
56	rs458685	chr21	31177511	Cancer	Breast cancer	0.00	13.78	1.25
57	rs10227331	chr7	157294938	Neurological behavioral	ADHD-Inattentive symptoms	0.00	13.76	1.25
58	rs16966460	chr15	38511983	Neurological behavioral	Bipolar disorder	0.00	13.72	1.25
59	rs11849538	chr14	96175978	Drug metabolism	Adverse response to aromatase inhibitors	0.00	13.66	1.24
60	rs11823543	chr11	116649135	Serum metabolites	Triglycerides blood pressure	0.00	13.57	1.24
61	rs2815752	chr1	72812440	Quantitative traits	BMI	13.48	0.00	-1.23
62	rs16826658	chr1	22485871	Miscellaneous	Endometriosis	26.15	74.83	1.23
63	rs4937126	chr11	126281897	Cardiovascular	Coronary heart disease	13.17	0.00	-1.21
64	rs3734729	chr6	150570867	Kidney lung liver	FEV1/FVC	0.00	13.10	1.21
65	rs7274811	chr20	32333181	Quantitative traits	Height	0.00	13.09	1.21
66	rs1539019	chr1	247600301	Serum metabolites	Fibrinogen	0.00	13.04	1.20
67	rs1893217	chr18	12809340	Autoimmune disease	Celiac disease	13.04	0.00	-1.20
68	rs1893217	chr18	12809340	Autoimmune disease	Rheumatoid arthritis celiac disease	13.04	0.00	-1.20
69	rs1893217	chr18	12809340	Diabetes	Type 1 diabetes	13.04	0.00	-1.20
70	rs2580816	chr2	232797966	Quantitative traits	Height	13.01	0.00	-1.20
71	rs8038465	chr15	73978337	Serum metabolites	Gamma glutamyl transferase	12.86	0.00	-1.19
72	rs10935268	chr3	137447485	Drug metabolism	Acenocoumarol maintenance dosage	12.77	0.00	-1.19
73	rs9373523	chr6	147701133	Parasitic bacterial disease	Tuberculosis	12.76	0.00	-1.19
74	rs10892279	chr11	118611781	Autoimmune disease	Rheumatoid arthritis celiac disease	12.57	0.00	-1.17
75	rs17114046	chr1	56966350	Cardiovascular	Coronary heart disease	0.00	12.38	1.16
76	rs9543325	chr13	73916628	Cancer	Pancreatic cancer	12.16	0.00	-1.15

77	rs6441286	chr3	159728878	Autoimmune disease	Primary biliary cirrhosis	55.26	19.66	-1.14
78	rs2074404	chr17	44865439	Autoimmune disease	Celiac disease	0.00	11.82	1.13
79	rs7078160	chr10	118827560	Miscellaneous	Nonsyndromic cleft lip with or without cleft palate	0.00	11.64	1.11
80	rs1480597	chr10	45161109	Aging	Parkinsons disease	11.58	0.00	-1.11
81	rs11154022	chr6	121748542	Cardiovascular	RR interval	0.00	11.51	1.11
82	rs1061235	chr6	29913298	Drug metabolism	Response to carbamapezine	11.42	0.00	-1.10
83	rs7774434	chr6	32657578	Autoimmune disease	Primary biliary cirrhosis	0.00	11.39	1.10
84	rs1514178	chr1	61205469	Serum metabolites	Serum polyunsaturated fatty acids	11.29	0.00	-1.09
85	rs7342306	chr12	6291093	Hematological parameters	Platelet count	11.24	0.00	-1.09
86	rs7971536	chr12	102373788	Quantitative traits	Height	0.00	11.16	1.08
87	rs599083	chr11	68192346	Radiographic parameters	Bone mineral density-spine	11.12	0.00	-1.08
88	rs4409785	chr11	95311422	Autoimmune disease	Multiple sclerosis	0.00	11.11	1.08
89	rs8023445	chr15	49192791	Neurological behavioral	Major depressive disorder	0.00	11.06	1.07
90	rs445925	chr19	45415640	Cardiovascular	Internal carotid intimal medial thickness	11.03	0.00	-1.07
91	rs445925	chr19	45415640	Serum metabolites	LDL cholesterol	11.03	0.00	-1.07
92	rs4332037	chr7	1950809	Neurological behavioral	Bipolar disorder	0.00	11.03	1.07
93	rs2841498	chr9	87930045	Neurological behavioral	Partial epilepsies	0.00	11.03	1.07
94	rs12579350	chr12	5797101	Neurological behavioral	Panic disorder	20.54	4.52	-1.07
95	rs2153960	chr6	108988184	Serum metabolites	IGF-1	0.00	10.84	1.06
96	rs301	chr8	19816934	Serum metabolites	HDL cholesterol waist circumference	0.00	10.84	1.06
97	rs860554	chr1	201262432	Neurological behavioral	Panic disorder	0.00	10.62	1.04
98	rs1395821	chr4	148047550	Cardiovascular	Coronary heart disease	10.57	0.00	-1.04
99	rs3788013	chr21	43841328	Diabetes	Type 1 diabetes autoantibodies	0.00	10.52	1.04
100	rs806276	chr6	91207351	Neurological behavioral	ADHD	0.00	10.47	1.03
101	rs8039808	chr15	49939846	Drug metabolism	Response to antidepressants-bupropion	10.40	0.00	-1.03
102	rs8001976	chr13	48387722	Aging	Aging traits-age free from disease	10.39	0.00	-1.03
103	rs4794822	chr17	38156712	Hematological parameters	Neutrophil count	0.00	10.39	1.03

104	rs571312	chr18	57839769	Quantitative traits	BMI	63.58	26.15	-1.03
105	rs1555543	chr1	96944797	Quantitative traits	BMI	0.00	10.24	1.02
106	rs3745516	chr19	50926742	Autoimmune disease	Primary biliary cirrhosis	0.00	10.18	1.01
107	rs10805321	chr4	13914373	Drug metabolism	Response to antipsychotic therapy extrapyramidal side effects	28.16	8.97	-1.01
108	rs2075650	chr19	45395619	Aging	Alzheimers disease	0.00	10.03	1.00
109	rs2075650	chr19	45395619	Aging	Alzheimers-AB1-42	0.00	10.03	1.00
110	rs2075650	chr19	45395619	Aging	Alzheimers-p-tau181p AB1-42	0.00	10.03	1.00
111	rs2075650	chr19	45395619	Neurological behavioral	Brain imaging	0.00	10.03	1.00
112	rs2075650	chr19	45395619	Cardiovascular	Cardiovascular disease risk factors	0.00	10.03	1.00
113	rs2075650	chr19	45395619	Serum metabolites	Cholesterol	0.00	10.03	1.00
114	rs2075650	chr19	45395619	Serum metabolites	C-reactive protein	0.00	10.03	1.00
115	rs2075650	chr19	45395619	Aging	Longevity	0.00	10.03	1.00
116	rs11099864	chr4	153893354	Aging	Amyotrophic lateral sclerosis	10.02	0.00	-1.00
117	rs1574192	chr2	241308505	Neurological behavioral	Brain imaging in schizophrenia interaction	10.02	0.00	-1.00

35 PXR Round:3

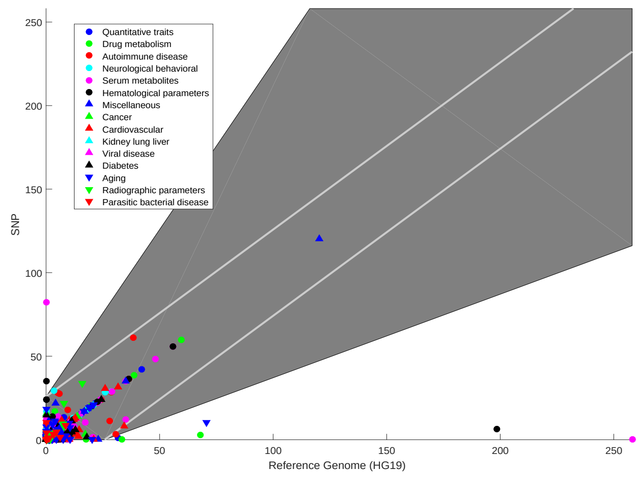

S.No.	SNP	Chr.	Position	Disease Class	Disease Trait	HG19	SNP	$log_2(\frac{SNP+\eta}{HG19+\eta})$
1	rs6009824	chr22	50086373	Serum metabolites	Natriuretic peptide	258.08	0.00	-3.46
2	rs8076739	chr17	27714587	Hematological parameters	Mean platelet volume	198.48	6.20	-2.81
3	rs9989419	chr16	56985139	Serum metabolites	HDL cholesterol	0.00	82.35	2.07
4	rs153091	chr16	13253956	Drug metabolism	Response to antipsychotic therapy perphenazine-triglycerides	67.97	2.75	-1.72
5	rs536841	chr11	85787824	Aging	Alzheimers disease	70.66	10.56	-1.41
6	rs559972	chr17	27814496	Hematological parameters	Platelet count	0.00	35.11	1.24
7	rs6125048	chr20	46208605	Drug metabolism	Response to treatment for acute lymphoblastic leukemia	33.41	0.00	-1.20
8	rs6905288	chr6	43758873	Quantitative traits	Waist-hip ratio	31.61	1.11	-1.09

36 PXR+9 Round:3

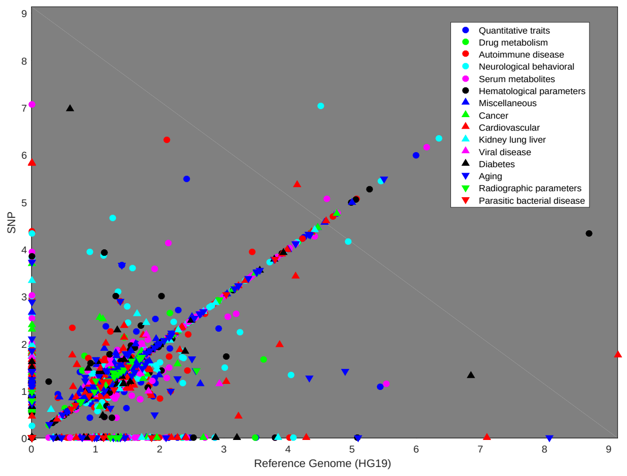

37 VDR Round:3

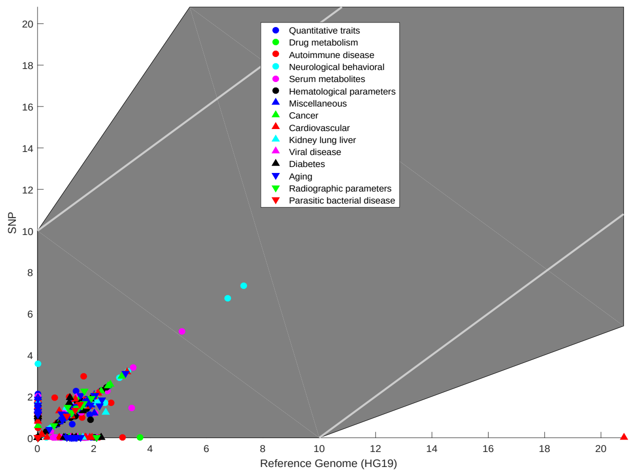

S.No.	SNP	Chr.	Position	Disease Class	Disease Trait	HG19	SNP	$log_2(\frac{SNP+\eta}{HG19+\eta})$
1	rs688034	chr22	26689635	Cardiovascular	Coronary heart disease	20.80	0.00	-1.62

38 VDR:RXRA Round:3

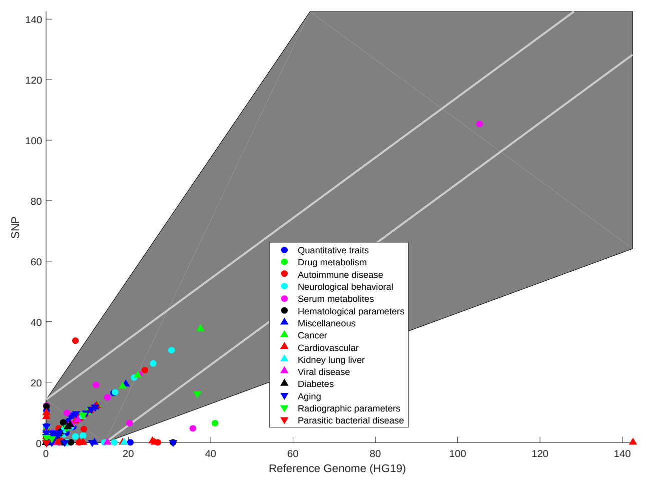

S.No.	SNP	Chr.	Position	Disease Class	Disease Trait	HG19	SNP	$log_2(\frac{SNP+\eta}{HG19+\eta})$
1	rs688034	chr22	26689635	Cardiovascular	Coronary heart disease	142.50	0.00	-3.46
2	rs900145	chr11	13293905	Aging	Age at menarche	30.84	0.00	-1.66
3	rs8076739	chr17	27714587	Hematological parameters	Mean platelet volume	30.84	0.00	-1.66
4	rs6441286	chr3	159728878	Autoimmune disease	Primary biliary cirrhosis	27.16	0.00	-1.54
5	rs11650066	chr17	32287449	Cardiovascular	Coronary heart disease	26.48	0.00	-1.51
6	rs10947055	chr6	30093364	Cardiovascular	Cardiac hypertrophy	25.86	0.60	-1.43
7	rs153091	chr16	13253956	Drug metabolism	Response to antipsychotic therapy perphenazine-triglycerides	40.99	6.38	-1.42
8	rs13146355	chr4	77412140	Serum metabolites	Serum magnesium levels	35.75	4.84	-1.39
9	rs2580816	chr2	232797966	Quantitative traits	Height	20.49	0.00	-1.29
10	rs9314986	chr13	30458737	Kidney lung liver	Biliary atresia	19.02	0.00	-1.22
11	rs17589290	chr4	112923237	Cardiovascular	Hypertension	18.60	0.00	-1.20
12	rs6556416	chr5	158818745	Autoimmune disease	Ankylosing spondylitis	7.05	33.72	1.17
13	rs10455248	chr6	72482458	Neurological behavioral	Trails A	16.50	0.00	-1.11
14	rs558718	chr19	7909883	Viral disease	HIV progression	14.98	0.00	-1.04

39 VDR+10 Round:2

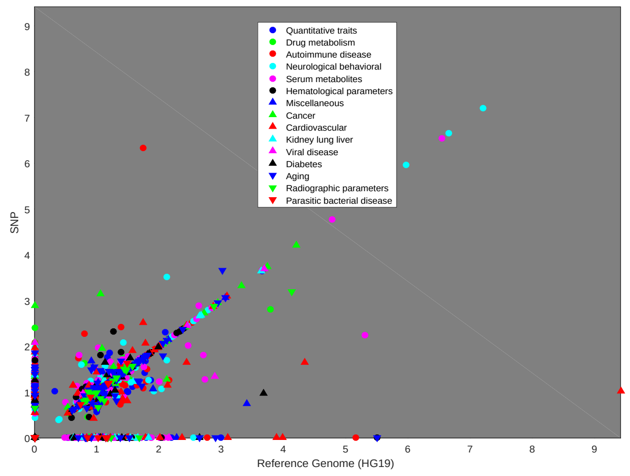

40 FXR Round:3

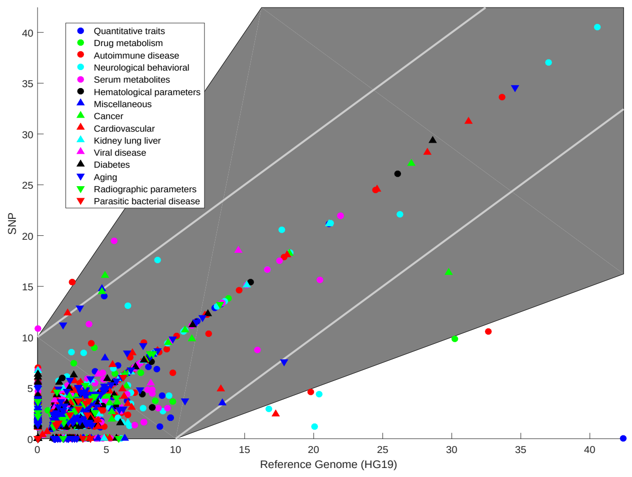

S.No.	SNP	Chr.	Position	Disease Class	Disease Trait	HG19	SNP	$log_2(\frac{SNP+\eta}{HG19+\eta})$
1	rs12735613	chr1	118883973	Quantitative traits	Height	42.45	0.00	-2.39
2	rs2002030	chr8	11276542	Neurological behavioral	Immediate Story Recall	2.46	30.47	1.70
3	rs2475335	chr9	10260263	Neurological behavioral	Partial epilepsies	20.06	1.20	-1.42
4	rs883079	chr12	114793240	Cardiovascular	QRS duration	17.27	2.45	-1.13
5	rs3892715	chr3	194758010	Neurological behavioral	ADHD	20.41	4.40	-1.08
6	rs515135	chr2	21286057	Serum metabolites	LDL cholesterol	0.00	10.82	1.06
7	rs11243676	chr9	135096767	Autoimmune disease	Systemic lupus erythematosus	32.65	10.58	-1.05
8	rs1064395	chr19	19361735	Neurological behavioral	Bipolar disorder	16.77	2.93	-1.05
9	rs12928822	chr16	11403893	Autoimmune disease	Celiac disease	19.76	4.61	-1.03
10	rs2201841	chr1	67694202	Autoimmune disease	Psoriasis	2.49	15.39	1.02
11	rs2201841	chr1	67694202	Autoimmune disease	Ulcerative colitis	2.49	15.39	1.02
12	rs11949289	chr5	28340173	Drug metabolism	Response to antidepressants-bupropion	30.25	9.82	-1.02

41 FXR+11 Round:3

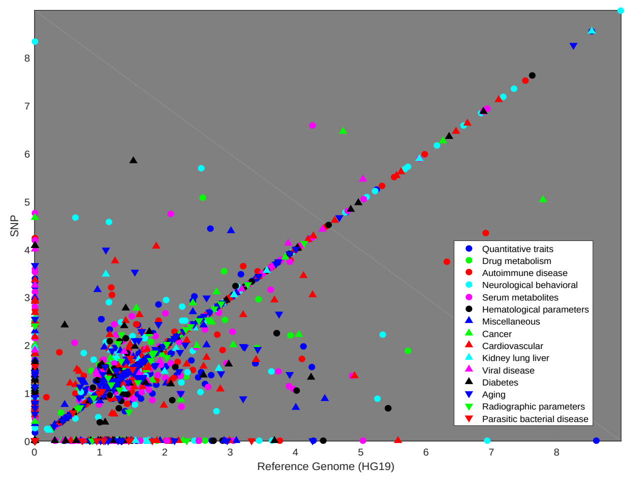

42 LXRA Round:3

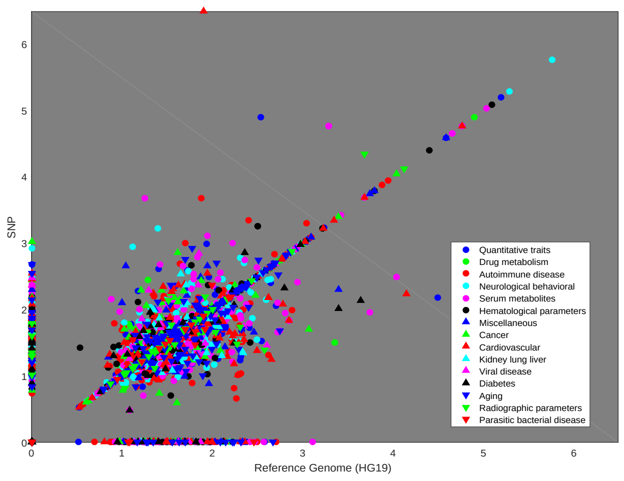

43 LXRA+12 Round:3

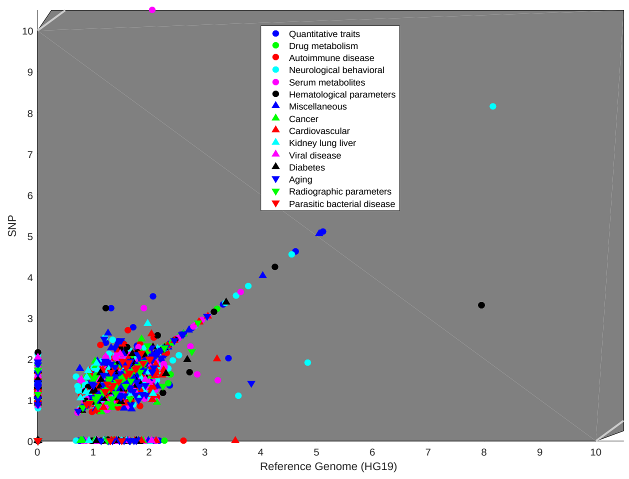

44 LXRB:RXRA Round:3

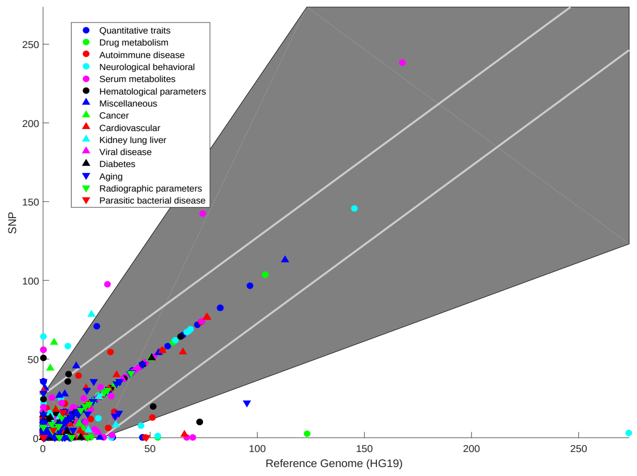

S.No.	SNP	Chr.	Position	Disease Class	Disease Trait	HG19	SNP	$log_2(\frac{SNP+\eta}{HG19+\eta})$
1	rs6747972	chr2	68070225	Neurological behavioral	Restless legs syndrome	273.58	2.97	-3.31
2	rs153091	chr16	13253956	Drug metabolism	Response to antipsychotic therapy perphenazine-triglycerides	123.35	2.72	-2.32
3	rs10892151	chr11	117531731	Serum metabolites	Triglycerides	69.99	0.00	-1.83
4	rs10447248	chr5	107915736	Serum metabolites	Adiponectin	66.90	0.00	-1.78
5	rs17007017	chr4	142142729	Neurological behavioral	Conduct disorder case status	0.00	64.25	1.74
6	rs10947055	chr6	30093364	Cardiovascular	Cardiac hypertrophy	65.91	2.30	-1.65
7	rs10885122	chr10	113042093	Serum metabolites	Fasting plasma glucose	0.00	55.78	1.60
8	rs10885122	chr10	113042093	Serum metabolites	Insulin resistance	0.00	55.78	1.60
9	rs7669317	chr4	106457330	Drug metabolism	Response to antipsychotic therapy extrapyramidal side effects	53.55	0.00	-1.56
10	rs11085824	chr19	13001547	Hematological parameters	Mean corpuscular hemoglobin	0.00	50.94	1.52
11	rs1064395	chr19	19361735	Neurological behavioral	Bipolar disorder	53.51	0.95	-1.51
12	rs1451375	chr7	50622712	Parasitic bacterial disease	Malaria	48.25	0.00	-1.47
13	rs1557351	chr18	54752314	Autoimmune disease	Multiple sclerosis	47.83	0.00	-1.46
14	rs9378805	chr6	417727	Cancer	Chronic lymphocytic leukemia	5.01	60.48	1.44
15	rs7138803	chr12	50247468	Quantitative traits	BMI	46.30	0.00	-1.43
16	rs7138803	chr12	50247468	Quantitative traits	Waist circumference	46.30	0.00	-1.43
17	rs7138803	chr12	50247468	Quantitative traits	Weight	46.30	0.00	-1.43
18	rs7961894	chr12	122365583	Hematological parameters	Mean platelet volume	72.95	10.15	-1.42

19	rs7961894	chr12	122365583	Hematological parameters	Platelet count	72.95	10.15	-1.42
20	rs1464108	chr12	131022010	Aging	Alzheimers disease	95.23	22.03	-1.31
21	rs12203592	chr6	396321	Quantitative traits	Freckling	0.00	35.84	1.21
22	rs12203592	chr6	396321	Quantitative traits	Hair color	0.00	35.84	1.21
23	rs12203592	chr6	396321	Quantitative traits	Hair color-Black vs. blond hair color	0.00	35.84	1.21
24	rs12203592	chr6	396321	Quantitative traits	Hair color-Black vs. red hair color	0.00	35.84	1.21
25	rs12203592	chr6	396321	Aging	Progressive supranuclear palsy	0.00	35.84	1.21
26	rs17483466	chr2	111797458	Cancer	Chronic lymphocytic leukemia	3.54	44.00	1.21
27	rs1329650	chr10	93348120	Neurological behavioral	Smoking behavior	11.39	58.09	1.14
28	rs571312	chr18	57839769	Quantitative traits	BMI	32.67	0.00	-1.13
29	rs2153960	chr6	108988184	Serum metabolites	IGF-1	29.97	97.40	1.12
30	rs2349775	chr7	8718080	Neurological behavioral	Neuroticism	31.76	0.00	-1.11
31	rs117607728	chr10	96058636	Kidney lung liver	COPD	22.64	78.16	1.08
32	rs8111998	chr19	22741675	Miscellaneous	Keratoconus	0.73	31.59	1.07
33	rs758642	chr17	3786907	Neurological behavioral	Smoking behavior	45.57	7.65	-1.06
34	rs1231206	chr17	2125605	Cardiovascular	Coronary heart disease	0.00	29.51	1.06
35	rs7315438	chr12	115891403	Cancer	Colorectal cancer	0.00	28.89	1.04
36	rs2292354	chr12	110368201	Serum metabolites	HDL cholesterol	32.03	1.77	-1.03
37	rs6120849	chr20	33730387	Serum metabolites	Protein C	28.30	0.00	-1.02
38	rs4746003	chr10	71538292	Aging	Alzheimers disease	0.00	28.12	1.02

45 PPARD Round:3

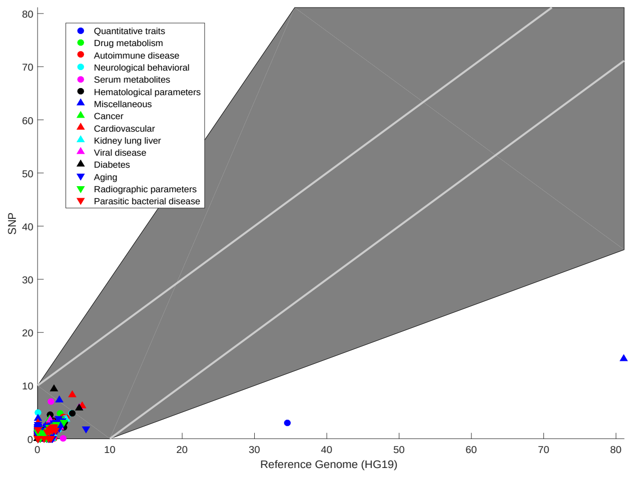

S.No.	SNP	Chr.	Position	Disease Class	Disease Trait	HG19	SNP	$log_2(\frac{SNP+\eta}{HG19+\eta})$
1	rs1585471	chr4	112702635	Miscellaneous	Myopia pathological	81.12	15.11	-1.86
2	rs571312	chr18	57839769	Quantitative traits	BMI	34.58	3.03	-1.77

46 PPARD+13 Round:3

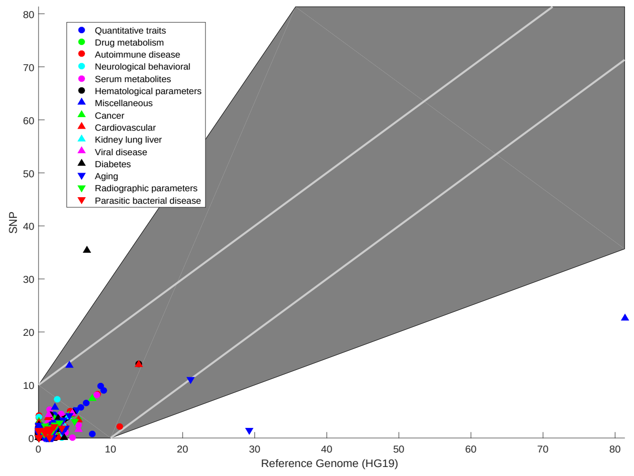

S.No.	SNP	Chr.	Position	Disease Class	Disease Trait	HG19	SNP	$log_2(\frac{SNP+\eta}{HG19+\eta})$
1	rs3764650	chr19	1046520	Aging	Alzheimers disease	29.24	1.51	-1.77
2	rs1585471	chr4	112702635	Miscellaneous	Myopia pathological	81.32	22.58	-1.49
3	rs11642841	chr16	53845487	Diabetes	Type 2 diabetes	6.73	35.41	1.44

47 PPARD+14 Round:3

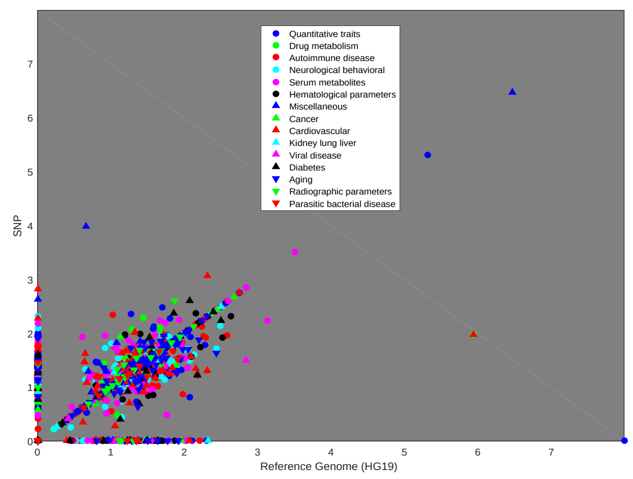

48 PPARG Round:3

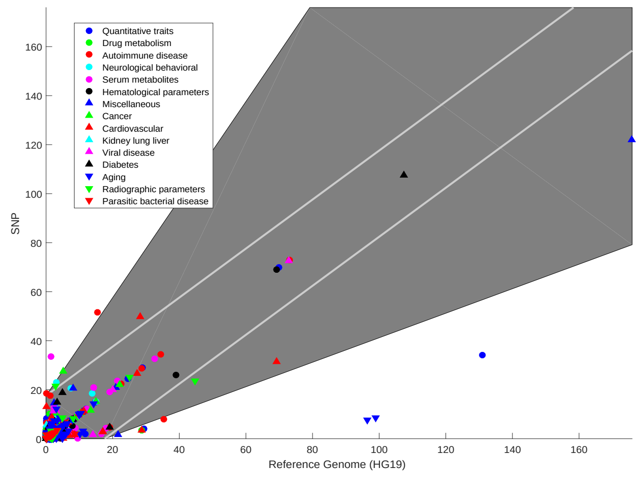

S.No.	SNP	Chr.	Position	Disease Class	Disease Trait	HG19	SNP	$log_2(\frac{SNP+\eta}{HG19+\eta})$
1	rs3764650	chr19	1046520	Aging	Alzheimers disease	96.35	7.72	-2.17
2	rs17565841	chr15	27997247	Aging	Parkinsons disease age of onset	98.86	8.72	-2.15
3	rs11642841	chr16	53845487	Diabetes	Type 2 diabetes	19.95	122.89	1.90
4	rs571312	chr18	57839769	Quantitative traits	BMI	131.06	34.20	-1.52
5	rs3093030	chr19	10397403	Serum metabolites	ICAM1	1.45	33.51	1.42
6	rs6495122	chr15	75125645	Drug metabolism	Caffeine intake	28.78	3.57	-1.13
7	rs6495122	chr15	75125645	Drug metabolism	coffee consumption	28.78	3.57	-1.13
8	rs6495122	chr15	75125645	Cardiovascular	Diastolic blood pressure	28.78	3.57	-1.13
9	rs2778031	chr9	90835726	Quantitative traits	Height	29.45	4.03	-1.12
10	rs11214966	chr11	114231255	Autoimmune disease	Asthma	15.49	51.45	1.06
11	rs3091315	chr17	32593665	Autoimmune disease	Crohns disease	35.32	8.08	-1.04
12	rs9657904	chr3	105586714	Autoimmune disease	Multiple sclerosis	0.00	18.42	1.03
13	rs7567389	chr2	127982645	Miscellaneous	Self-rated health	21.53	1.70	-1.02

49 PPARG+14 Round:3

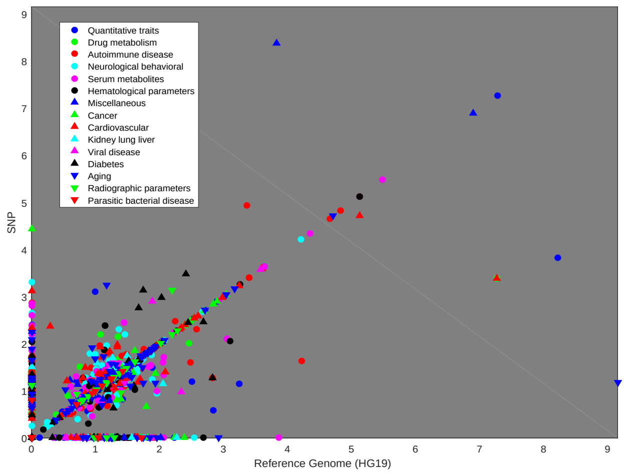

50 PPARG+15 Round:3

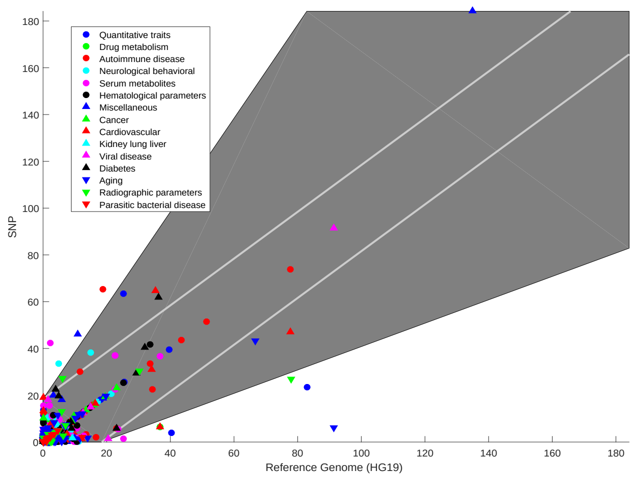

S.No.	SNP	Chr.	Position	Disease Class	Disease Trait	HG19	SNP	$log_2(\frac{SNP+\eta}{HG19+\eta})$
1	rs3764650	chr19	1046520	Aging	Alzheimers disease	91.33	6.21	-2.16
2	rs3093030	chr19	10397403	Serum metabolites	ICAM1	2.16	42.44	1.56
3	rs2778031	chr9	90835726	Quantitative traits	Height	40.33	3.79	-1.40
4	rs571312	chr18	57839769	Quantitative traits	BMI	82.99	23.37	-1.28
5	rs11214966	chr11	114231255	Autoimmune disease	Asthma	18.91	65.16	1.16
6	rs6495122	chr15	75125645	Drug metabolism	Caffeine intake	36.78	6.37	-1.16
7	rs6495122	chr15	75125645	Drug metabolism	coffee consumption	36.78	6.37	-1.16
8	rs6495122	chr15	75125645	Cardiovascular	Diastolic blood pressure	36.78	6.37	-1.16
9	rs6017291	chr20	42854134	Neurological behavioral	Delayed Story Recall	5.00	33.66	1.15
10	rs1076540	chr22	18439958	Serum metabolites	Gamma glutamyl transferase	25.26	1.30	-1.15
11	rs16826658	chr1	22485871	Miscellaneous	Endometriosis	10.98	46.13	1.14
12	rs7932354	chr11	46722221	Radiographic parameters	Bone mineral density-hip	78.05	26.82	-1.09
13	rs17609940	chr6	35034800	Cardiovascular	Coronary heart disease	0.00	18.96	1.02

51 Rev-ErbA-Alpha Round:3

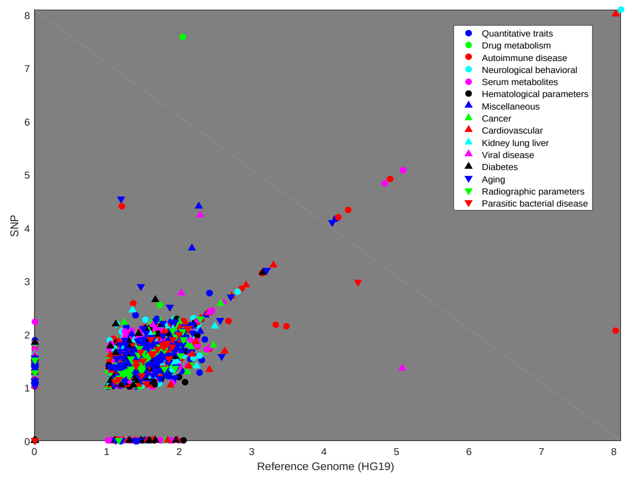

52 RORC Round:3

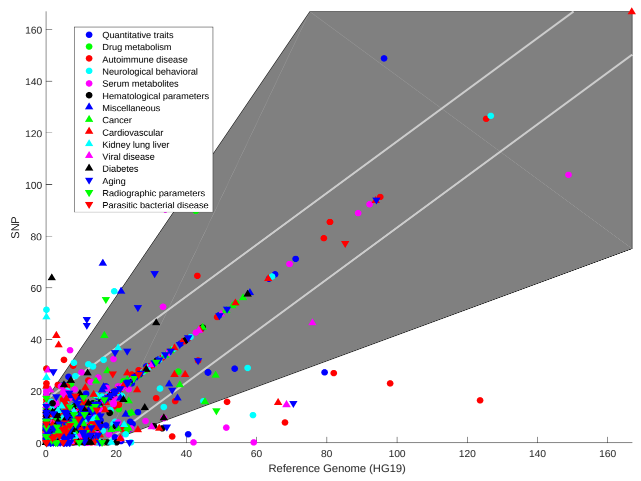

S.No.	SNP	Chr.	Position	Disease Class	Disease Trait	HG19	SNP	$log_2(\frac{SNP+\eta}{HG19+\eta})$
1	rs3120139	chr6	160741622	Serum metabolites	Lipoprotein A	59.26	0.00	-2.19
2	rs11642841	chr16	53845487	Diabetes	Type 2 diabetes	1.57	63.83	2.14
3	rs28493229	chr19	41224204	Autoimmune disease	Kawasaki disease	123.65	16.45	-2.08
4	rs4129148	chrY	940180	Neurological behavioral	Schizophrenia	0.00	51.41	2.03
5	rs3734729	chr6	150570867	Kidney lung liver	FEV1/FVC	0.00	48.75	1.97
6	rs1800788	chr4	155483914	Serum metabolites	Fibrinogen	42.01	0.00	-1.81
7	rs11243676	chr9	135096767	Autoimmune disease	Systemic lupus erythematosus	68.13	7.95	-1.78
8	rs12052359	chr2	81872922	Serum metabolites	Bilirubin	51.39	5.71	-1.60
9	rs11118620	chr1	221028508	Cardiovascular	Heart failure	2.85	41.52	1.57
10	rs16910061	chr9	97314741	Quantitative traits	Height	40.60	3.15	-1.53
11	rs485499	chr3	159745863	Autoimmune disease	Primary biliary cirrhosis	97.94	23.06	-1.53
12	rs3744028	chr17	73888672	Neurological behavioral	White matter hyperintensity	59.01	10.59	-1.47
13	rs231735	chr2	204693876	Autoimmune disease	Rheumatoid arthritis	35.88	2.29	-1.47
14	rs7359257	chr15	67702907	Aging	Age at menarche	70.50	15.14	-1.45
15	rs7635839	chr3	191667777	Drug metabolism	Response to antipsychotic therapy-PR interval	0.00	28.65	1.44
16	rs1790100	chr12	123656725	Autoimmune disease	Multiple sclerosis	0.00	28.62	1.44
17	rs9277535	chr6	33054861	Viral disease	Hepatitis B	68.45	14.74	-1.44
18	rs9277535	chr6	33054861	Viral disease	Hepatitis B vaccine response	68.45	14.74	-1.44
19	rs10777317	chr12	91980374	Cardiovascular	Sudden cardiac arrest	3.52	37.86	1.43
20	rs634990	chr15	35006073	Miscellaneous	Refractive error	16.13	69.39	1.39
21	rs17291045	chr4	161506897	Viral disease	HIV progression	0.53	28.08	1.38
22	rs1550576	chr15	58213414	Cardiovascular	Hypertension	66.09	15.67	-1.35
23	rs9292394	chr5	30661573	Kidney lung liver	COPD	0.00	25.25	1.33

24	rs1847461	chr12	91077508	Neurological behavioral	Smoking behavior	24.24	0.00	-1.29
25	rs13187289	chr5	133849177	Aging	Age at menarche	23.78	0.00	-1.28
26	rs6798928	chr3	151579873	Autoimmune disease	Immunoglobulin A	0.00	23.03	1.25
27	rs368331	chr7	21742974	Aging	Alzheimers Total ventricular volume	2.09	27.46	1.23
28	rs368331	chr7	21742974	Aging	Alzheimers-Whole-brain volume	2.09	27.46	1.23
29	rs319690	chr3	47927484	Cardiovascular	Blood pressure	0.00	21.72	1.20
30	rs11167764	chr5	141479065	Autoimmune disease	Crohns disease	0.00	21.45	1.19
31	rs10202497	chr2	238270894	Aging	Aging traits-age free from disease	11.59	47.74	1.19
32	rs6441286	chr3	159728878	Autoimmune disease	Primary biliary cirrhosis	81.91	26.97	-1.17
33	rs12047808	chr1	179469314	Autoimmune disease	Multiple sclerosis	5.02	32.14	1.17
34	rs4811196	chr20	36469694	Radiographic parameters	Bone mineral density-trochanter	48.52	12.40	-1.16
35	rs8109578	chr19	10213154	Serum metabolites	Thyroid stimulating hormone	6.78	35.92	1.16
36	rs12969657	chr18	67536496	Hematological parameters	Mean platelet volume	33.09	5.58	-1.16
37	rs10498514	chr14	64899055	Neurological behavioral	Cognitive performance-SWM Strategy	0.00	20.60	1.16
38	rs13182402	chr5	125918148	Aging	Osteoporosis	34.50	6.27	-1.16
39	rs6601606	chr8	11638244	Hematological parameters	Neutrophil count	20.01	0.00	-1.14
40	rs10947055	chr6	30093364	Cardiovascular	Cardiac hypertrophy	32.23	5.62	-1.13
41	rs8115854	chr20	35766337	Aging	Hippocampal atrophy	11.69	45.52	1.13
42	rs102275	chr11	61557803	Autoimmune disease	Crohns disease	0.00	19.88	1.13
43	rs102275	chr11	61557803	Serum metabolites	HDL cholesterol	0.00	19.88	1.13
44	rs102275	chr11	61557803	Serum metabolites	Serum polyunsaturated fatty acids	0.00	19.88	1.13
45	rs7466269	chr9	133464084	Quantitative traits	Height	79.25	27.28	-1.13
46	rs7138803	chr12	50247468	Quantitative traits	BMI	21.28	0.85	-1.11
47	rs7138803	chr12	50247468	Quantitative traits	Waist circumference	21.28	0.85	-1.11
48	rs7138803	chr12	50247468	Quantitative traits	Weight	21.28	0.85	-1.11
49	rs4355801	chr8	119923873	Radiographic parameters	Bone mineral density	17.03	55.41	1.10
50	rs757608	chr17	59497277	Quantitative traits	Height	0.00	18.72	1.08
51	rs9357155	chr6	32809848	Kidney lung liver	IgA nepropathy	18.61	0.00	-1.08

52	rs511154	chr3	135950921	Serum metabolites	Fibrinogen	33.97	90.37	1.08
53	rs2300747	chr1	117104215	Autoimmune disease	Multiple sclerosis	51.56	15.72	-1.07
54	rs713875	chr22	30592487	Autoimmune disease	Crohns disease	0.00	18.07	1.06
55	rs12915189	chr15	91114376	Neurological behavioral	Information processing speed inspection time	19.49	58.53	1.06
56	rs1408282	chr6	93852252	Serum metabolites	C-reactive protein	0.00	18.01	1.06
57	rs854555	chr7	94930391	Drug metabolism	Response to TNF antagonist treatment	0.00	17.96	1.05
58	rs11884476	chr2	206318593	Viral disease	HIV progression	1.28	20.51	1.05
59	rs7192086	chr16	13061611	Neurological behavioral	Schizophrenia	17.81	0.00	-1.05
60	rs646776	chr1	109818530	Serum metabolites	Cholesterol	21.18	1.64	-1.05
61	rs646776	chr1	109818530	Cardiovascular	Coronary heart disease	21.18	1.64	-1.05
62	rs646776	chr1	109818530	Serum metabolites	LDL cholesterol	21.18	1.64	-1.05
63	rs646776	chr1	109818530	Cardiovascular	Myocardial infarction	21.18	1.64	-1.05
64	rs646776	chr1	109818530	Serum metabolites	Progranulin	21.18	1.64	-1.05
65	rs646776	chr1	109818530	Drug metabolism	Response to statin therapy-chol sum	21.18	1.64	-1.05
66	rs2357013	chr2	53266129	Hematological parameters	Hemoglobin	17.58	0.00	-1.04
67	rs11239550	chr10	46024729	Hematological parameters	Mean corpuscular volume	30.76	6.53	-1.03
68	rs16914280	chr11	88321724	Viral disease	HIV progression	30.13	6.25	-1.03
69	rs1366594	chr5	88376061	Radiographic parameters	Bone mineral density-femoral neck	0.00	17.35	1.03
70	rs1366594	chr5	88376061	Radiographic parameters	Bone mineral density-hip	0.00	17.35	1.03
71	rs7665090	chr4	103551603	Autoimmune disease	Primary biliary cirrhosis	0.00	16.78	1.00
72	rs7142881	chr14	32093548	Drug metabolism	Response to iloperidone treatment QT prolongation	16.73	0.00	-1.00

53 RORC:RXRA Round:3

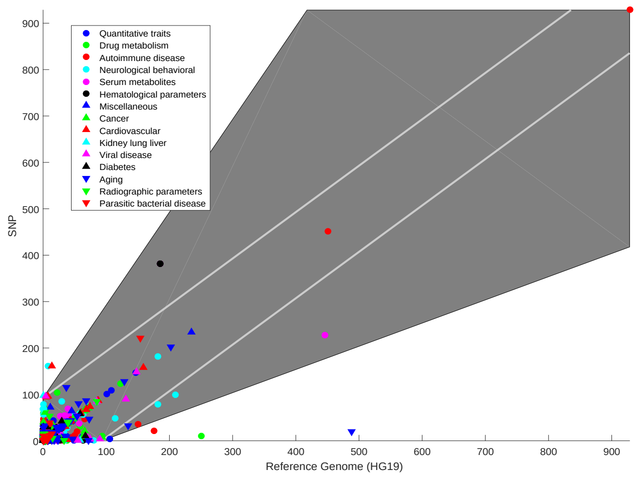

S.No.	SNP	Chr.	Position	Disease Class	Disease Trait	HG19	SNP	$log_2(\frac{SNP+\eta}{HG19+\eta})$
1	rs7359257	chr15	67702907	Aging	Age at menarche	488.00	19.43	-2.37
2	rs10935268	chr3	137447485	Drug metabolism	Acenocoumarol maintenance dosage	250.86	9.76	-1.74
3	rs9512637	chr13	27920611	Neurological behavioral	Alcohol consumption	8.06	160.65	1.33
4	rs13300284	chr9	25452812	Cardiovascular	RR interval	14.37	161.70	1.25
5	rs2300747	chr1	117104215	Autoimmune disease	Multiple sclerosis	175.17	20.90	-1.24
6	rs16910061	chr9	97314741	Quantitative traits	Height	105.44	3.34	-1.04
7	rs9292394	chr5	30661573	Kidney lung liver	COPD	0.00	97.62	1.04

54 RORC+16 Round:3

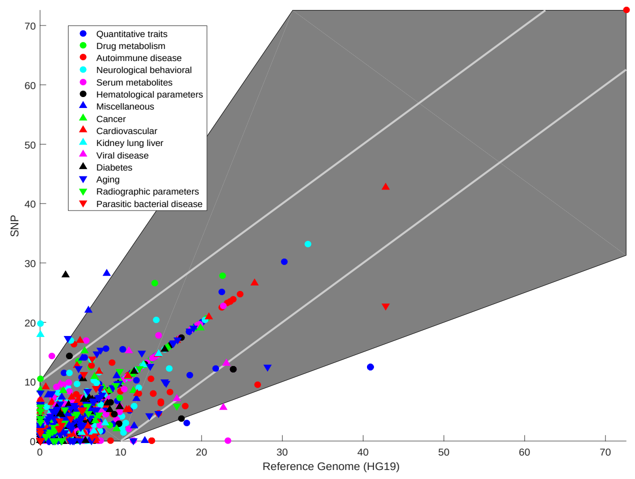

S.No.	SNP	Chr.	Position	Disease Class	Disease Trait	HG19	SNP	$log_2(\frac{SNP+\eta}{HG19+\eta})$
1	rs12052359	chr2	81872922	Serum metabolites	Bilirubin	23.22	0.00	-1.73
2	rs4129148	chrY	940180	Neurological behavioral	Schizophrenia	0.00	19.80	1.58
3	rs11642841	chr16	53845487	Diabetes	Type 2 diabetes	3.17	27.97	1.53
4	rs3734729	chr6	150570867	Kidney lung liver	FEV1/FVC	0.00	17.88	1.48
5	rs2155219	chr11	76299194	Autoimmune disease	Allergic rhinitis	13.82	0.00	-1.25
6	rs2155219	chr11	76299194	Autoimmune disease	IgE grass sensitization	13.82	0.00	-1.25
7	rs2155219	chr11	76299194	Autoimmune disease	Ulcerative colitis	13.82	0.00	-1.25
8	rs3213787	chr2	45646824	Miscellaneous	Glaucoma	12.95	0.00	-1.20
9	rs12896399	chr14	92773663	Quantitative traits	Eye color	40.87	12.50	-1.18
10	rs12896399	chr14	92773663	Quantitative traits	Eye color-blue vs. green eyes	40.87	12.50	-1.18
11	rs12896399	chr14	92773663	Quantitative traits	Eye color-green eyes	40.87	12.50	-1.18
12	rs12896399	chr14	92773663	Quantitative traits	Hair color	40.87	12.50	-1.18
13	rs12896399	chr14	92773663	Quantitative traits	Hair color-Black vs. blond hair color	40.87	12.50	-1.18
14	rs12896399	chr14	92773663	Quantitative traits	Hair color-blond vs. brown	40.87	12.50	-1.18
15	rs16910061	chr9	97314741	Quantitative traits	Height	18.17	3.04	-1.11
16	rs3764261	chr16	56993324	Aging	Age-related macular degeneration	11.53	0.00	-1.11
17	rs3764261	chr16	56993324	Serum metabolites	Blood pressure HDL cholesterol	11.53	0.00	-1.11
18	rs3764261	chr16	56993324	Serum metabolites	HDL cholesterol	11.53	0.00	-1.11
19	rs3764261	chr16	56993324	Serum metabolites	LDL cholesterol	11.53	0.00	-1.11
20	rs10518733	chr15	53940307	Serum metabolites	Creatinine	1.47	14.30	1.08
21	rs4452212	chr2	137015991	Miscellaneous	Telomere length	8.22	28.25	1.07
22	rs558718	chr19	7909883	Viral disease	HIV progression	22.74	5.63	-1.07

23	rs7635839	chr3	191667777	Drug metabolism	Response to antipsychotic therapy-PR interval	0.00	10.46	1.03
24	rs8115854	chr20	35766337	Aging	Hippocampal atrophy	3.49	17.25	1.01
25	rs634990	chr15	35006073	Miscellaneous	Refractive error	5.96	22.02	1.00

55 TR4 Round:3

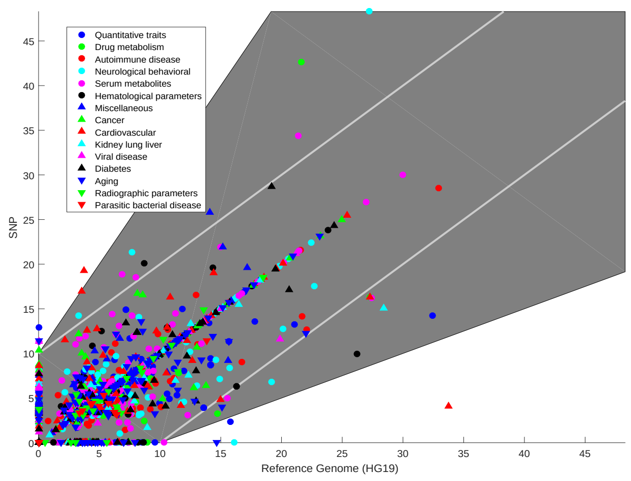

S.No.	SNP	Chr.	Position	Disease Class	Disease Trait	HG19	SNP	$log_2(\frac{SNP+\eta}{HG19+\eta})$
1	rs688034	chr22	26689635	Cardiovascular	Coronary heart disease	33.76	4.07	-1.64
2	rs9491640	chr6	98919264	Neurological behavioral	Brain imaging in schizophrenia interaction	16.12	0.00	-1.39
3	rs10412199	chr19	3927771	Aging	Aging traits-age free from disease	14.64	0.00	-1.30
4	rs4823006	chr22	29451671	Quantitative traits	Waist-hip ratio	0.00	12.90	1.20
5	rs10914144	chr1	171949750	Hematological parameters	Mean platelet volume	7.23	28.73	1.17
6	rs10914144	chr1	171949750	Hematological parameters	Platelet count	7.23	28.73	1.17
7	rs7153703	chr14	51919822	Aging	Alzheimers Total ventricular volume	0.00	11.46	1.10
8	rs10889353	chr1	63118196	Serum metabolites	Cholesterol	0.00	11.38	1.10
9	rs10889353	chr1	63118196	Serum metabolites	LDL cholesterol	0.00	11.38	1.10
10	rs10889353	chr1	63118196	Serum metabolites	Triglycerides	0.00	11.38	1.10
11	rs6751715	chr2	56363377	Viral disease	HIV progression	0.00	11.38	1.10
12	rs2782980	chr10	115781527	Cardiovascular	Blood pressure	3.75	19.26	1.09
13	rs2691543	chr7	77964788	Quantitative traits	Height	15.80	2.37	-1.06
14	rs7716600	chr5	44875005	Cancer	Breast cancer	0.00	10.39	1.03
15	rs4783244	chr16	82662268	Serum metabolites	Adiponectin	10.35	0.00	-1.03

56 TR2 Round:3

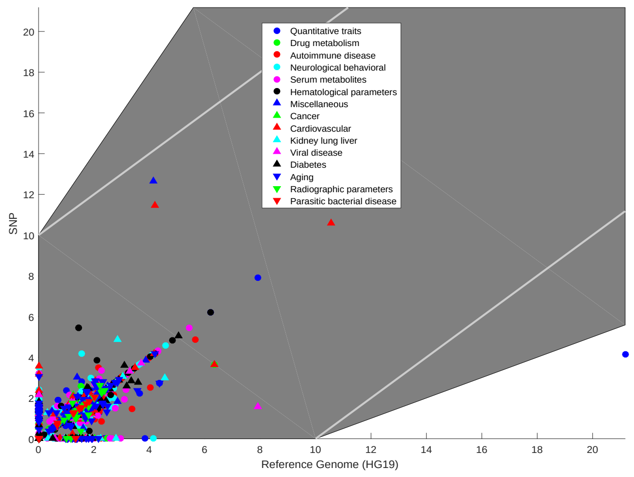

S.No.	SNP	Chr.	Position	Disease Class	Disease Trait	HG19	SNP	$log_2(\frac{SNP+\eta}{HG19+\eta})$
1	rs571312	chr18	57839769	Quantitative traits	BMI	21.17	4.13	-1.14

57 LRH1 Round:3

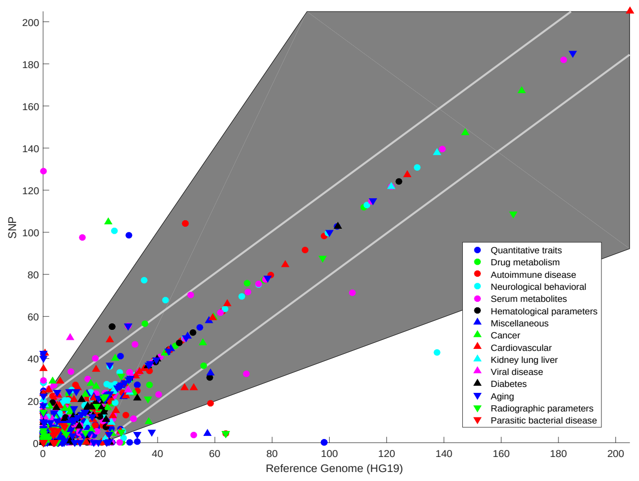

S.No.	SNP	Chr.	Position	Disease Class	Disease Trait	HG19	SNP	$log_2(\frac{SNP+\eta}{HG19+\eta})$
1	rs1000778	chr11	61655305	Serum metabolites	Sphingolipid concentrations	0.08	128.92	2.86
2	rs7138803	chr12	50247468	Quantitative traits	BMI	98.17	0.00	-2.53
3	rs7138803	chr12	50247468	Quantitative traits	Waist circumference	98.17	0.00	-2.53
4	rs7138803	chr12	50247468	Quantitative traits	Weight	98.17	0.00	-2.53
5	rs2153960	chr6	108988184	Serum metabolites	IGF-1	13.77	97.50	1.78
6	rs7524102	chr1	22698447	Radiographic parameters	Bone mineral density-femoral neck	63.70	4.29	-1.76
7	rs7524102	chr1	22698447	Radiographic parameters	Bone mineral density-hip	63.70	4.29	-1.76
8	rs7524102	chr1	22698447	Radiographic parameters	Bone mineral density-spine	63.70	4.29	-1.76
9	rs7524102	chr1	22698447	Autoimmune disease	Ulcerative colitis	63.70	4.29	-1.76
10	rs9921518	chr16	54494424	Miscellaneous	Dialysis-related mortality	57.42	4.33	-1.65
11	rs1925690	chr6	87867063	Aging	Alzheimers Entorhinal cortical thickness	0.00	42.53	1.62
12	rs1152846	chr3	188420897	Quantitative traits	BMI	0.00	42.22	1.61
13	rs1408282	chr6	93852252	Serum metabolites	C-reactive protein	52.64	3.72	-1.60
14	rs2008242	chr4	5221538	Cardiovascular	PR segment	0.61	42.62	1.58
15	rs7153703	chr14	51919822	Aging	Alzheimers Total ventricular volume	0.00	39.96	1.56
16	rs7176508	chr15	70018990	Cancer	Chronic lymphocytic leukemia	22.83	104.79	1.53
17	rs2251393	chr17	60778932	Cardiovascular	Sudden cardiac arrest	0.00	35.19	1.44
18	rs4964805	chr12	104192824	Neurological behavioral	ADHD	25.01	100.66	1.41
19	rs5757949	chr22	40820151	Quantitative traits	Height	32.78	0.43	-1.35
20	rs12579350	chr12	5797101	Neurological behavioral	Panic disorder	137.59	42.94	-1.32

21	rs1424233	chr16	79682751	Quantitative traits	Obesity	30.14	0.00	-1.31
22	rs471364	chr9	15289578	Serum metabolites	HDL cholesterol	0.00	29.67	1.29
23	rs1329650	chr10	93348120	Neurological behavioral	Smoking behavior	0.00	28.60	1.26
24	rs922948	chr3	69442637	Quantitative traits	Hip geometry	0.00	28.55	1.26
25	rs1927702	chr9	15986716	Quantitative traits	BMI	29.92	98.52	1.24
26	rs16966142	chr16	89851033	Drug metabolism	Caffeine intake	27.68	0.00	-1.23
27	rs7453920	chr6	32730012	Viral disease	Hepatitis B	9.54	49.81	1.23
28	rs8038465	chr15	73978337	Serum metabolites	Gamma glutamyl transferase	26.71	0.00	-1.20
29	rs466639	chr1	165394882	Aging	Age at menarche	37.94	5.06	-1.19
30	rs2282978	chr7	92264410	Quantitative traits	Height	0.00	24.59	1.14
31	rs2061333	chr19	44614208	Aging	Alzheimers disease	32.88	3.95	-1.13
32	rs157350	chr5	156139569	Quantitative traits	Brachial circumference	24.04	0.00	-1.12
33	rs157350	chr5	156139569	Quantitative traits	Hip circumference	24.04	0.00	-1.12
34	rs7427021	chr3	163761964	Neurological behavioral	Bipolar disorder	0.00	23.76	1.11
35	rs2159324	chr19	45695738	Serum metabolites	C-reactive protein	23.72	0.00	-1.11
36	rs2159324	chr19	45695738	Serum metabolites	LDL cholesterol	23.72	0.00	-1.11
37	rs4547811	chr4	146794621	Serum metabolites	Gamma glutamyl transferase	0.00	23.51	1.10
38	rs2896103	chr5	13764419	Cardiovascular	Ankle brachial index	0.00	23.43	1.10
39	rs12042938	chr1	231816842	Neurological behavioral	DISC1	28.05	2.16	-1.10
40	rs4305276	chr2	241495013	Hematological parameters	Mean platelet volume	0.00	23.28	1.10
41	rs7528684	chr1	157670816	Diabetes	Type 1 diabetes autoantibodies	23.19	0.00	-1.09
42	rs7914558	chr10	104775908	Neurological behavioral	Schizophrenia	22.87	0.00	-1.08
43	rs4665630	chr2	23898317	Cardiovascular	Hypertension	22.82	0.00	-1.08
44	rs4765623	chr12	125320850	Cancer	Renal cell carcinoma	3.21	29.37	1.07
45	rs7034200	chr9	4289050	Serum metabolites	Fasting plasma glucose	22.47	0.00	-1.07
46	rs7034200	chr9	4289050	Serum metabolites	Insulin resistance	22.47	0.00	-1.07
47	rs4968031	chr16	23765774	Hematological parameters	Platelet count	22.22	0.00	-1.06
48	rs3772255	chr3	156102734	Aging	Aging traits-biologic age	22.19	0.00	-1.06
49	rs10521157	chr17	9336370	Aging	Longevity	0.00	22.09	1.06
50	rs13095226	chr3	99396272	Aging	Age-related macular degeneration	23.14	0.61	-1.05

51	rs10885122	chr10	113042093	Serum metabolites	Fasting plasma glucose	2.37	25.86	1.02
52	rs10885122	chr10	113042093	Serum metabolites	Insulin resistance	2.37	25.86	1.02
53	rs3825214	chr12	114795443	Cardiovascular	PR interval	2.03	25.17	1.02
54	rs3825214	chr12	114795443	Cardiovascular	QRS duration	2.03	25.17	1.02
55	rs3825214	chr12	114795443	Cardiovascular	QT interval	2.03	25.17	1.02
56	rs370409	chr6	90921740	Autoimmune disease	Graves disease	58.58	18.79	-1.01
57	rs56238310	chr3	111233239	Kidney lung liver	COPD	0.00	20.58	1.00
58	rs651477	chr2	119395691	Autoimmune disease	Multiple sclerosis	20.52	0.00	-1.00

58 LRH1:RXRA Round:3

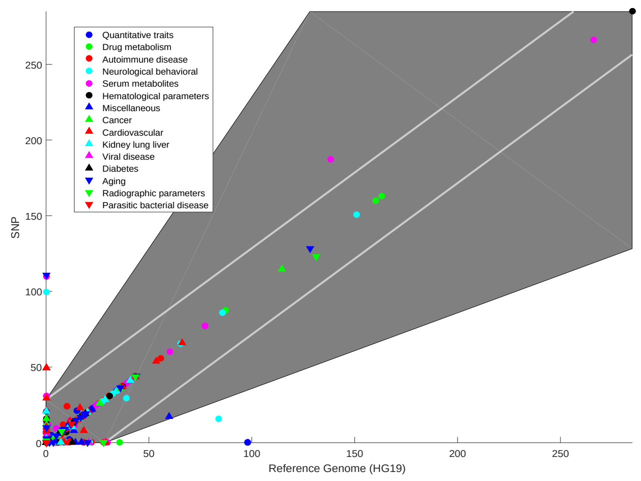

S.No.	SNP	Chr.	Position	Disease Class	Disease Trait	HG19	SNP	$log_2(\frac{SNP+\eta}{HG19+\eta})$
1	rs2034764	chr9	2742771	Aging	Amyotrophic lateral sclerosis-age of onset	0.00	110.57	2.29
2	rs1000778	chr11	61655305	Serum metabolites	Sphingolipid concentrations	0.00	109.94	2.28
3	rs4964805	chr12	104192824	Neurological behavioral	ADHD	0.00	99.81	2.17
4	rs7138803	chr12	50247468	Quantitative traits	BMI	98.03	0.00	-2.15
5	rs7138803	chr12	50247468	Quantitative traits	Waist circumference	98.03	0.00	-2.15
6	rs7138803	chr12	50247468	Quantitative traits	Weight	98.03	0.00	-2.15
7	rs3825214	chr12	114795443	Cardiovascular	PR interval	0.00	49.45	1.45
8	rs3825214	chr12	114795443	Cardiovascular	QRS duration	0.00	49.45	1.45
9	rs3825214	chr12	114795443	Cardiovascular	QT interval	0.00	49.45	1.45
10	rs12579350	chr12	5797101	Neurological behavioral	Panic disorder	83.80	15.75	-1.34
11	rs2500535	chr6	149329267	Drug metabolism	Response to antidepressants-nortryptyline	35.64	0.00	-1.17
12	rs651007	chr9	136153875	Serum metabolites	E-selectin	0.00	30.93	1.06
13	rs651007	chr9	136153875	Serum metabolites	LDL cholesterol	0.00	30.93	1.06
14	rs1408282	chr6	93852252	Serum metabolites	C-reactive protein	29.69	0.00	-1.03
15	rs9315204	chr13	33693837	Cardiovascular	Intracranial aneurysm	29.01	0.00	-1.01
16	rs1158167	chr20	23578189	Serum metabolites	Cystatin C	29.01	0.00	-1.01
17	rs17609940	chr6	35034800	Cardiovascular	Coronary heart disease	0.29	29.47	1.01

59 SF1 Round:3

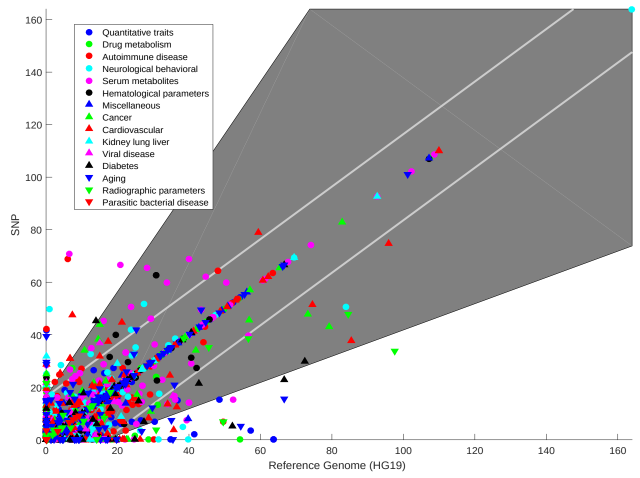

S.No.	SNP	Chr.	Position	Disease Class	Disease Trait	HG19	SNP	$log_2(\frac{SNP+\eta}{HG19+\eta})$
1	rs7138803	chr12	50247468	Quantitative traits	BMI	63.62	0.12	-2.28
2	rs7138803	chr12	50247468	Quantitative traits	Waist circumference	63.62	0.12	-2.28
3	rs7138803	chr12	50247468	Quantitative traits	Weight	63.62	0.12	-2.28
4	rs1459148	chr14	98840443	Drug metabolism	Response to antipsychotic therapy extrapyramidal side effects	54.27	0.00	-2.11
5	rs4964805	chr12	104192824	Neurological behavioral	ADHD	0.89	49.69	1.93
6	rs1000778	chr11	61655305	Serum metabolites	Sphingolipid concentrations	6.52	70.81	1.93
7	rs1063635	chr6	31379931	Autoimmune disease	Rheumatoid arthritis	6.18	68.70	1.91
8	rs9470004	chr6	35341850	Quantitative traits	Height	57.31	3.63	-1.88
9	rs8007846	chr14	66262963	Autoimmune disease	Multiple sclerosis-Brain Glutamate Concentrations	0.00	42.10	1.83
10	rs1152846	chr3	188420897	Quantitative traits	BMI	0.00	41.77	1.83
11	rs7914558	chr10	104775908	Neurological behavioral	Schizophrenia	39.73	0.00	-1.77
12	rs7153703	chr14	51919822	Aging	Alzheimers Total ventricular volume	0.00	39.40	1.77
13	rs466639	chr1	165394882	Aging	Age at menarche	54.56	5.12	-1.72
14	rs2237897	chr11	2858546	Diabetes	Type 2 diabetes	52.24	5.20	-1.67
15	rs3772255	chr3	156102734	Aging	Aging traits-biologic age	35.45	0.00	-1.66
16	rs6499640	chr16	53769677	Quantitative traits	BMI	34.80	0.00	-1.64
17	rs6499640	chr16	53769677	Quantitative traits	Weight	34.80	0.00	-1.64
18	rs5757949	chr22	40820151	Quantitative traits	Height	41.42	2.19	-1.64
19	rs2710833	chr4	169409958	Kidney lung liver	NAFLDH	0.00	31.84	1.56

20	rs36563	chr14	71352648	Neurological behavioral	Alcohol dependence	31.37	0.00	-1.54
21	rs7524102	chr1	22698447	Radiographic parameters	Bone mineral density-femoral neck	49.56	6.80	-1.51
22	rs7524102	chr1	22698447	Radiographic parameters	Bone mineral density-hip	49.56	6.80	-1.51
23	rs7524102	chr1	22698447	Radiographic parameters	Bone mineral density-spine	49.56	6.80	-1.51
24	rs7524102	chr1	22698447	Autoimmune disease	Ulcerative colitis	49.56	6.80	-1.51
25	rs651477	chr2	119395691	Autoimmune disease	Multiple sclerosis	30.09	0.00	-1.50
26	rs1925690	chr6	87867063	Aging	Alzheimers Entorhinal cortical thickness	0.00	29.45	1.48
27	rs2896103	chr5	13764419	Cardiovascular	Ankle brachial index	0.00	28.81	1.46
28	rs16966142	chr16	89851033	Drug metabolism	Caffeine intake	28.69	0.00	-1.46
29	rs10521157	chr17	9336370	Aging	Longevity	0.00	28.61	1.46
30	rs2126259	chr8	9185146	Serum metabolites	LDL cholesterol	0.00	28.51	1.45
31	rs2008242	chr4	5221538	Cardiovascular	PR segment	7.34	47.65	1.43
32	rs4665630	chr2	23898317	Cardiovascular	Hypertension	26.39	0.00	-1.38
33	rs10850409	chr12	115381740	Cardiovascular	QRS duration	35.83	3.72	-1.38
34	rs10260404	chr7	154210798	Aging	Amyotrophic lateral sclerosis	66.73	15.69	-1.37
35	rs11786458	chr8	40252701	Neurological behavioral	ADHD-Inattentive symptoms	38.26	4.83	-1.36
36	rs1329650	chr10	93348120	Neurological behavioral	Smoking behavior	0.00	25.22	1.34
37	rs4765623	chr12	125320850	Cancer	Renal cell carcinoma	0.00	25.12	1.34
38	rs7528684	chr1	157670816	Diabetes	Type 1 diabetes autoantibodies	24.75	0.00	-1.33
39	rs519113	chr19	45376284	Serum metabolites	HDL cholesterol	24.66	0.00	-1.32
40	rs11085824	chr19	13001547	Hematological parameters	Mean corpuscular hemoglobin	0.00	23.74	1.29
41	rs35390	chr5	33955326	Cancer	Melanoma	22.98	0.00	-1.26
42	rs2352028	chr13	92445229	Cancer	Lung cancer	22.43	0.00	-1.24
43	rs17007695	chr4	142709723	Drug metabolism	Response to treatment for acute lymphoblastic leukemia	0.00	22.30	1.24
44	rs2159324	chr19	45695738	Serum metabolites	C-reactive protein	22.15	0.00	-1.23
45	rs2159324	chr19	45695738	Serum metabolites	LDL cholesterol	22.15	0.00	-1.23
46	rs12907914	chr15	39315358	Cardiovascular	Cardiac hypertrophy	21.92	0.00	-1.22
47	rs4729260	chr7	96117918	Radiographic parameters	Bone mineral density-spine	30.75	3.80	-1.22
48	rs1408282	chr6	93852252	Serum metabolites	C-reactive protein	39.27	7.47	-1.22
49	rs2280543	chr11	203788	Miscellaneous	Uterine fibroids	21.83	0.00	-1.22

50	rs9921518	chr16	54494424	Miscellaneous	Dialysis-related mortality	39.86	7.87	-1.21
51	rs13394720	chr2	234502121	Viral disease	HIV progression	0.00	21.60	1.21
52	rs548097	chr13	75776268	Cardiovascular	Heart failure	21.49	0.00	-1.21
53	rs11949289	chr5	28340173	Drug metabolism	Response to antidepressants-bupropion	24.94	1.56	-1.20
54	rs1366594	chr5	88376061	Radiographic parameters	Bone mineral density-femoral neck	0.00	21.31	1.20
55	rs1366594	chr5	88376061	Radiographic parameters	Bone mineral density-hip	0.00	21.31	1.20
56	rs732505	chr19	5582535	Serum metabolites	Van Wildebrand factor antibodies	0.00	21.09	1.19
57	rs56238310	chr3	111233239	Kidney lung liver	COPD	0.00	21.00	1.19
58	rs6426749	chr1	22711473	Radiographic parameters	Bone mineral density-hip	97.55	33.92	-1.18
59	rs10411161	chr19	52372976	Cancer	Breast cancer	0.00	20.66	1.18
60	rs152528	chr5	142017860	Cardiovascular	Cardiac hypertrophy	20.53	0.00	-1.17
61	rs681900	chr2	75074967	Miscellaneous	Femoral neck bone geometry	0.00	20.25	1.16
62	rs17319721	chr4	77368847	Serum metabolites	Creatinine	20.25	0.00	-1.16
63	rs12896399	chr14	92773663	Quantitative traits	Eye color	20.20	0.00	-1.16
64	rs12896399	chr14	92773663	Quantitative traits	Eye color-blue vs. green eyes	20.20	0.00	-1.16
65	rs12896399	chr14	92773663	Quantitative traits	Eye color-green eyes	20.20	0.00	-1.16
66	rs12896399	chr14	92773663	Quantitative traits	Hair color	20.20	0.00	-1.16
67	rs12896399	chr14	92773663	Quantitative traits	Hair color-Black vs. blond hair color	20.20	0.00	-1.16
68	rs12896399	chr14	92773663	Quantitative traits	Hair color-blond vs. brown	20.20	0.00	-1.16
69	rs471364	chr9	15289578	Serum metabolites	HDL cholesterol	20.85	66.67	1.16
70	rs7931342	chr11	68994497	Cancer	Prostate cancer	0.00	20.14	1.16
71	rs2282978	chr7	92264410	Quantitative traits	Height	0.00	20.13	1.16
72	rs895636	chr2	45188353	Serum metabolites	Fasting plasma glucose	0.00	19.86	1.14
73	rs2499604	chr1	238103501	Kidney lung liver	NAFLDH	19.74	0.00	-1.14
74	rs515135	chr2	21286057	Serum metabolites	LDL cholesterol	0.00	19.72	1.14
75	rs8001976	chr13	48387722	Aging	Aging traits-age free from disease	19.71	0.00	-1.14
76	rs4547811	chr4	146794621	Serum metabolites	Gamma glutamyl transferase	0.00	19.70	1.14
77	rs4968031	chr16	23765774	Hematological parameters	Platelet count	19.65	0.00	-1.14
78	rs8038465	chr15	73978337	Serum metabolites	Gamma glutamyl transferase	19.64	0.00	-1.14

79	rs16861329	chr3	186666461	Diabetes	Type 2 diabetes	19.59	0.00	-1.13
80	rs10508517	chr10	16997891	Cardiovascular	Diastolic blood pressure	19.54	0.00	-1.13
81	rs10227331	chr7	157294938	Neurological behavioral	ADHD-Inattentive symptoms	4.08	28.45	1.13
82	rs2061333	chr19	44614208	Aging	Alzheimers disease	26.80	3.32	-1.13
83	rs2782980	chr10	115781527	Cardiovascular	Blood pressure	0.00	19.48	1.13
84	rs13021401	chr2	20688519	Autoimmune disease	Systemic sclerosis	19.32	0.00	-1.12
85	rs589691	chr11	64525216	Serum metabolites	Serum urate	52.35	15.20	-1.12
86	rs13095226	chr3	99396272	Aging	Age-related macular degeneration	35.10	7.34	-1.12
87	rs12459897	chr19	31596778	Serum metabolites	Serum polyunsaturated fatty acids	0.00	18.92	1.11
88	rs4846033	chr1	11788564	Neurological behavioral	Schizophrenia	0.41	19.72	1.10
89	rs1250552	chr10	81058027	Autoimmune disease	Celiac disease	0.00	18.82	1.10
90	rs17119280	chr1	59799161	Drug metabolism	Response to antipsychotic therapy extrapyramidal side effects	18.72	0.00	-1.10
91	rs12042938	chr1	231816842	Neurological behavioral	DISC1	26.26	3.64	-1.09
92	rs6534347	chr4	123198435	Diabetes	Type 1 diabetes	66.67	22.80	-1.08
93	rs1424233	chr16	79682751	Quantitative traits	Obesity	18.26	0.00	-1.08
94	rs12625057	chr20	57790436	Drug metabolism	Response to antipsychotic therapy extrapyramidal side effects	0.00	18.11	1.07
95	rs6495122	chr15	75125645	Drug metabolism	Caffeine intake	17.84	0.00	-1.06
96	rs6495122	chr15	75125645	Drug metabolism	coffee consumption	17.84	0.00	-1.06
97	rs6495122	chr15	75125645	Cardiovascular	Diastolic blood pressure	17.84	0.00	-1.06
98	rs7812879	chr8	11340181	Autoimmune disease	Systemic lupus erythematosus	17.68	0.00	-1.05
99	rs3825214	chr12	114795443	Cardiovascular	PR interval	6.62	31.09	1.04
100	rs3825214	chr12	114795443	Cardiovascular	QRS duration	6.62	31.09	1.04
101	rs3825214	chr12	114795443	Cardiovascular	QT interval	6.62	31.09	1.04
102	rs4725982	chr7	150637863	Cardiovascular	QT interval	3.92	25.50	1.04
103	rs1387153	chr11	92673828	Serum metabolites	Blood pressure fasting glucose	17.39	0.00	-1.04
104	rs1387153	chr11	92673828	Serum metabolites	Fasting plasma glucose	17.39	0.00	-1.04
105	rs1387153	chr11	92673828	Serum metabolites	HbA1C	17.39	0.00	-1.04
106	rs1387153	chr11	92673828	Serum metabolites	HDL cholesterol fasting glucose	17.39	0.00	-1.04
107	rs1387153	chr11	92673828	Diabetes	Type 2 diabetes	17.39	0.00	-1.04

108	rs10412199	chr19	3927771	Aging	Aging traits-age free from disease	17.26	0.00	-1.04
109	rs8049439	chr16	28837515	Autoimmune disease	Inflammatory bowel disease-early onset	3.69	24.79	1.04
110	rs7686660	chr4	144003159	Autoimmune disease	Asthma	2.36	22.04	1.03
111	rs10506821	chr12	80496923	Quantitative traits	Hip geometry	48.45	15.32	-1.03
112	rs2807278	chr6	131809920	Quantitative traits	Waist-hip ratio	17.00	0.00	-1.03
113	rs174546	chr11	61569830	Serum metabolites	HDL cholesterol	16.93	0.00	-1.02
114	rs174546	chr11	61569830	Serum metabolites	LDL cholesterol	16.93	0.00	-1.02
115	rs2815752	chr1	72812440	Quantitative traits	BMI	30.47	6.74	-1.02
116	rs2696835	chr16	86365571	Diabetes	Diabetic retinopathy	14.05	45.18	1.02
117	rs13187289	chr5	133849177	Aging	Age at menarche	16.70	0.00	-1.01
118	rs1059513	chr12	57489709	Serum metabolites	Serum IgE levels	16.61	0.00	-1.01
119	rs6120849	chr20	33730387	Serum metabolites	Protein C	16.47	0.00	-1.00

60 SF1:RXRA Round:3

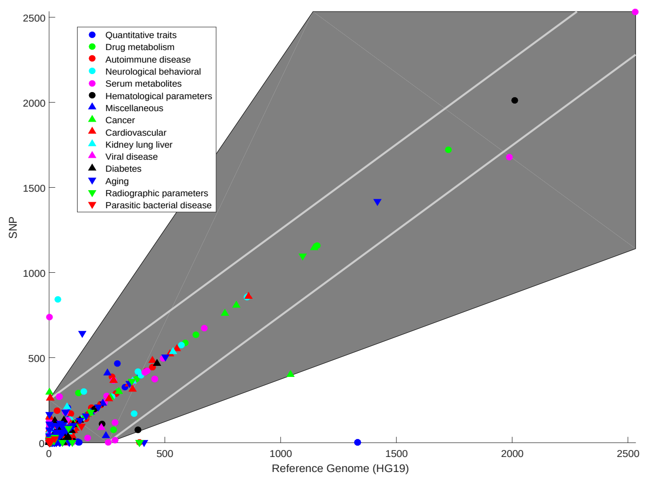

S.No.	SNP	Chr.	Position	Disease Class	Disease Trait	HG19	SNP	$log_2(\frac{SNP+\eta}{HG19+\eta})$
1	rs7138803	chr12	50247468	Quantitative traits	BMI	1332.45	0.00	-2.65
2	rs7138803	chr12	50247468	Quantitative traits	Waist circumference	1332.45	0.00	-2.65
3	rs7138803	chr12	50247468	Quantitative traits	Weight	1332.45	0.00	-2.65
4	rs1000778	chr11	61655305	Serum metabolites	Sphingolipid concentrations	0.00	739.21	1.97
5	rs4964805	chr12	104192824	Neurological behavioral	ADHD	37.14	842.87	1.92
6	rs466639	chr1	165394882	Aging	Age at menarche	408.86	0.00	-1.39
7	rs7524102	chr1	22698447	Radiographic parameters	Bone mineral density-femoral neck	389.68	0.00	-1.34
8	rs7524102	chr1	22698447	Radiographic parameters	Bone mineral density-hip	389.68	0.00	-1.34
9	rs7524102	chr1	22698447	Radiographic parameters	Bone mineral density-spine	389.68	0.00	-1.34
10	rs7524102	chr1	22698447	Autoimmune disease	Ulcerative colitis	389.68	0.00	-1.34
11	rs2034764	chr9	2742771	Aging	Amyotrophic lateral sclerosis-age of onset	143.78	643.33	1.18
12	rs4765623	chr12	125320850	Cancer	Renal cell carcinoma	0.00	297.27	1.12
13	rs7703051	chr5	74625487	Serum metabolites	LDL cholesterol	254.46	0.00	-1.00

61 TLX Round:3

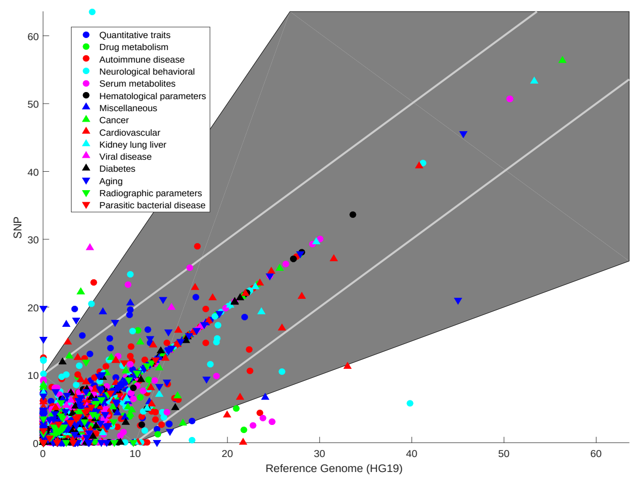

S.No.	SNP	Chr.	Position	Disease Class	Disease Trait	HG19	SNP	$log_2(\frac{SNP+\eta}{HG19+\eta})$
1	rs7578035	chr2	99382892	Neurological behavioral	Bipolar disorder	5.32	63.57	2.26
2	rs12190287	chr6	134214525	Cardiovascular	Coronary heart disease	21.72	0.00	-1.67
3	rs151222	chr16	20674492	Neurological behavioral	Schizophrenia	39.83	5.84	-1.65
4	rs10767971	chr11	32895664	Aging	Parkinsons disease age of onset	0.00	19.83	1.58
5	rs8093763	chr18	57503378	Drug metabolism	Bleomycin sensitivity	21.75	1.88	-1.42
6	rs2282679	chr4	72608383	Serum metabolites	Vitamin D insufficiency	24.80	3.06	-1.41
7	rs2282679	chr4	72608383	Serum metabolites	VitaminD-25OH	24.80	3.06	-1.41
8	rs174448	chr11	61639573	Serum metabolites	Serum polyunsaturated fatty acids	22.74	2.56	-1.38
9	rs2086512	chr6	99011501	Viral disease	Cytomegalovirus antibody response	5.12	28.77	1.36
10	rs766903	chr12	51703834	Aging	Aging traits-age at death	0.00	15.31	1.34
11	rs11789399	chr9	121359286	Neurological behavioral	Biplolar disorder and schizophrenia	16.16	0.38	-1.33
12	rs12051272	chr16	82663288	Serum metabolites	Adiponectin	23.84	3.64	-1.31
13	rs6601764	chr10	3862542	Autoimmune disease	Crohns disease	23.52	4.39	-1.22
14	rs6983267	chr8	128413305	Cancer	Colorectal cancer	4.09	22.19	1.19
15	rs6983267	chr8	128413305	Cancer	Prostate cancer	4.09	22.19	1.19
16	rs12188300	chr5	158829527	Autoimmune disease	Psoriasis	0.00	12.57	1.17
17	rs11755724	chr6	7118990	Aging	Age-related macular degeneration	12.33	0.00	-1.16
18	rs6437740	chr3	107465817	Neurological behavioral	Smoking behavior	0.00	12.21	1.15
19	rs4397449	chr8	90342186	Neurological behavioral	Animals	0.00	12.13	1.15

20	rs1884537	chr14	101251989	Quantitative traits	Optic disc size disc	3.43	19.70	1.14
21	rs10760706	chr9	102723692	Miscellaneous	Alopecia areata	2.52	17.48	1.13
22	rs17234657	chr5	40401509	Autoimmune disease	Crohns disease	5.49	23.68	1.12
23	rs281868	chr6	118574061	Cardiovascular	RR interval	19.99	4.03	-1.10
24	rs11739663	chr5	594083	Autoimmune disease	Ulcerative colitis	11.30	0.00	-1.09
25	rs11611208	chr12	20758613	Quantitative traits	Height	10.98	0.00	-1.07
26	rs2201841	chr1	67694202	Autoimmune disease	Psoriasis	10.87	0.00	-1.06
27	rs2201841	chr1	67694202	Autoimmune disease	Ulcerative colitis	10.87	0.00	-1.06
28	rs16984239	chr2	18234446	Aging	Amyotrophic lateral sclerosis	3.60	18.14	1.05
29	rs9491640	chr6	98919264	Neurological behavioral	Brain imaging in schizophrenia interaction	10.62	0.00	-1.04
30	rs8014204	chr14	75322794	Drug metabolism	Caffeine intake	20.96	5.05	-1.04
31	rs12188164	chr5	428236	Miscellaneous	Cystic fibrosis severity	24.08	6.67	-1.03
32	rs2326679	chr20	6035228	Aging	Menopause	13.77	1.70	-1.02
33	rs1916521	chr10	57416961	Cardiovascular	Cardiac hypertrophy	10.32	0.00	-1.02
34	rs12046278	chr1	10799577	Cardiovascular	Systolic blood pressure	33.05	11.25	-1.02
35	rs1202199	chr6	20156174	Neurological behavioral	ADHD-Hyperactive-impulsive symptoms	0.00	10.13	1.01

62 TLX:RXRA Round:3

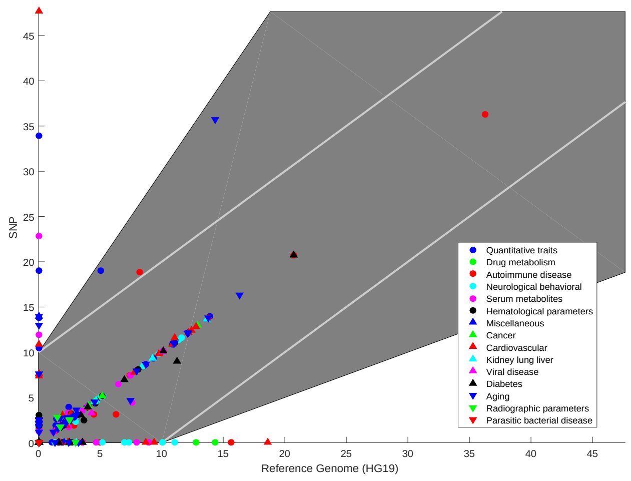

S.No.	SNP	Chr.	Position	Disease Class	Disease Trait	HG19	SNP	$log_2(\frac{SNP+\eta}{HG19+\eta})$
1	rs13038095	chr20	46425576	Cardiovascular	Atrial fibrillation	0.00	47.65	2.53
2	rs1956529	chr14	68788924	Quantitative traits	Primary tooth development number of teeth	0.00	33.94	2.14
3	rs3120139	chr6	160741622	Serum metabolites	Lipoprotein A	0.00	22.86	1.72
4	rs1875517	chr3	117307567	Quantitative traits	Waist circumference	0.00	19.01	1.54
5	rs10777317	chr12	91980374	Cardiovascular	Sudden cardiac arrest	18.62	0.00	-1.52
6	rs6601764	chr10	3862542	Autoimmune disease	Crohns disease	15.65	0.00	-1.36
7	rs6971925	chr7	14445917	Drug metabolism	Response to treatment for acute lymphoblastic leukemia	14.31	0.00	-1.28
8	rs6590322	chr11	128206410	Aging	Hippocampal atrophy	0.00	13.95	1.26
9	rs1527243	chr2	123291022	Miscellaneous	Erectile dysfunction and prostate cancer treatment	0.00	13.94	1.26
10	rs2429582	chr7	122202593	Quantitative traits	Brain structure	0.00	13.78	1.25
11	rs16984239	chr2	18234446	Aging	Amyotrophic lateral sclerosis	0.00	13.00	1.20
12	rs8014204	chr14	75322794	Drug metabolism	Caffeine intake	12.79	0.00	-1.19
13	rs12753193	chr1	66169679	Serum metabolites	C-reactive protein	0.00	11.96	1.13
14	rs151222	chr16	20674492	Neurological behavioral	Schizophrenia	11.04	0.00	-1.07
15	rs11624056	chr14	87506248	Cardiovascular	Sudden cardiac arrest	0.00	10.86	1.06
16	rs925098	chr4	17919811	Quantitative traits	Height	0.00	10.50	1.04
17	rs11789399	chr9	121359286	Neurological behavioral	Biplolar disorder and schizophrenia	10.07	0.00	-1.01

18	rs7584099	chr2	148478336	Drug metabolism	Response to statin therapy-CPD	10.04	0.00	-1.00

63 PNR Round:3

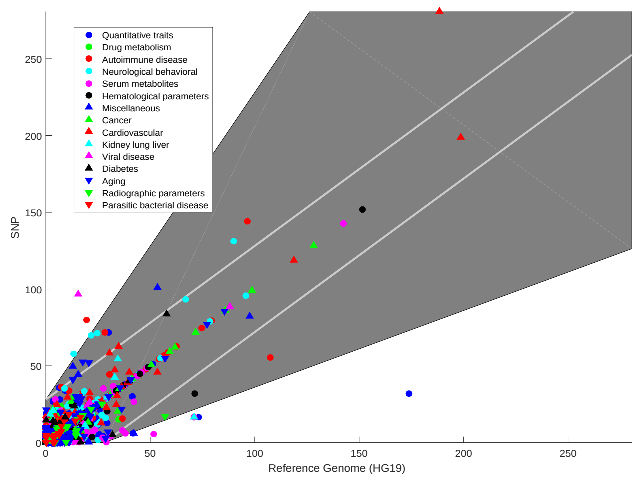

S.No.	SNP	Chr.	Position	Disease Class	Disease Trait	HG19	SNP	$log_2(\frac{SNP+\eta}{HG19+\eta})$
1	rs41360247	chr2	44073656	Serum metabolites	Campesterol	15.01	215.08	2.50
2	rs571312	chr18	57839769	Quantitative traits	BMI	173.83	32.09	-1.75
3	rs2086512	chr6	99011501	Viral disease	Cytomegalovirus antibody response	15.39	96.79	1.52
4	rs1591830	chr6	150651900	Serum metabolites	Phosphorus-serum	51.67	5.72	-1.24
5	rs1160312	chr20	22050503	Quantitative traits	Male-pattern baldness	73.24	16.68	-1.18
6	rs1975974	chr17	21707060	Autoimmune disease	Psoriasis	19.60	79.74	1.18
7	rs4129267	chr1	154426264	Autoimmune disease	Asthma	70.92	16.78	-1.14
8	rs4129267	chr1	154426264	Serum metabolites	C-reactive protein	70.92	16.78	-1.14
9	rs4129267	chr1	154426264	Kidney lung liver	FEF	70.92	16.78	-1.14
10	rs4129267	chr1	154426264	Serum metabolites	IL6R	70.92	16.78	-1.14
11	rs1574192	chr2	241308505	Neurological behavioral	Brain imaging in schizophrenia interaction	13.19	57.86	1.06
12	rs11130248	chr3	50352200	Miscellaneous	Keloid	42.04	5.95	-1.04
13	rs3791950	chr2	218729865	Quantitative traits	Height	41.87	6.05	-1.04
14	rs211718	chr1	76106675	Serum metabolites	C12 C10	28.87	0.00	-1.02

64 COUP-TF2 Round:3

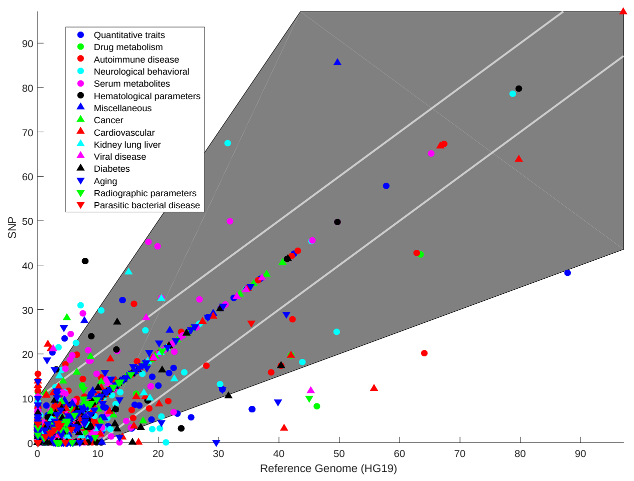

S.No.	SNP	Chr.	Position	Disease Class	Disease Trait	HG19	SNP	$log_2(\frac{SNP+\eta}{HG19+\eta})$
1	rs10412199	chr19	3927771	Aging	Aging traits-age free from disease	29.62	0.00	-1.99
2	rs688034	chr22	26689635	Cardiovascular	Coronary heart disease	40.89	3.27	-1.94
3	rs2548145	chr5	40134777	Neurological behavioral	Alcohol use disorder	21.29	0.00	-1.65
4	rs153091	chr16	13253956	Drug metabolism	Response to antipsychotic therapy perphenazine-triglycerides	46.28	8.14	-1.63
5	rs1395821	chr4	148047550	Cardiovascular	Coronary heart disease	55.76	12.15	-1.57
6	rs10914144	chr1	171949750	Hematological parameters	Mean platelet volume	7.88	40.96	1.51
7	rs10914144	chr1	171949750	Hematological parameters	Platelet count	7.88	40.96	1.51
8	rs4811196	chr20	36469694	Radiographic parameters	Bone mineral density-trochanter	44.97	9.99	-1.46
9	rs12946454	chr17	43208121	Cardiovascular	Systolic blood pressure	1.75	22.24	1.46
10	rs10927875	chr1	16299312	Cardiovascular	Dilated cardiomyopathy	16.73	0.00	-1.42
11	rs4764039	chr12	14064461	Aging	Alzheimers Total ventricular volume	39.85	9.13	-1.38
12	rs12896399	chr14	92773663	Quantitative traits	Eye color	35.55	7.60	-1.37
13	rs12896399	chr14	92773663	Quantitative traits	Eye color-blue vs. green eyes	35.55	7.60	-1.37
14	rs12896399	chr14	92773663	Quantitative traits	Eye color-green eyes	35.55	7.60	-1.37
15	rs12896399	chr14	92773663	Quantitative traits	Hair color	35.55	7.60	-1.37
16	rs12896399	chr14	92773663	Quantitative traits	Hair color-Black vs. blond hair color	35.55	7.60	-1.37
17	rs12896399	chr14	92773663	Quantitative traits	Hair color-blond vs. brown	35.55	7.60	-1.37
18	rs7315438	chr12	115891403	Cancer	Colorectal cancer	4.84	28.14	1.36

19	rs16861329	chr3	186666461	Diabetes	Type 2 diabetes	15.70	0.00	-1.36
20	rs7961894	chr12	122365583	Hematological parameters	Mean platelet volume	23.85	3.20	-1.36
21	rs7961894	chr12	122365583	Hematological parameters	Platelet count	23.85	3.20	-1.36
22	rs2008242	chr4	5221538	Cardiovascular	PR segment	2.19	21.18	1.36
23	rs8007846	chr14	66262963	Autoimmune disease	Multiple sclerosis-Brain Glutamate Concentrations	0.00	15.46	1.35
24	rs558718	chr19	7909883	Viral disease	HIV progression	45.26	11.71	-1.35
25	rs744373	chr2	127894615	Aging	Alzheimers disease	4.36	26.04	1.33
26	rs744373	chr2	127894615	Aging	Alzheimers disease late onset	4.36	26.04	1.33
27	rs6139030	chr20	3187733	Viral disease	Response to hepatitis C treatment	2.57	21.22	1.31
28	rs3793917	chr10	124219275	Aging	Age-related macular degeneration	1.55	18.70	1.31
29	rs11243676	chr9	135096767	Autoimmune disease	Systemic lupus erythematosus	64.09	20.15	-1.30
30	rs4823006	chr22	29451671	Quantitative traits	Waist-hip ratio	2.46	20.30	1.28
31	rs6887695	chr5	158822645	Autoimmune disease	Crohns disease	0.00	14.02	1.26
32	rs7153703	chr14	51919822	Aging	Alzheimers Total ventricular volume	0.00	13.91	1.26
33	rs16977195	chr15	86984240	Neurological behavioral	Schizophrenia	7.14	30.93	1.26
34	rs17609940	chr6	35034800	Cardiovascular	Coronary heart disease	0.00	13.81	1.25
35	rs211718	chr1	76106675	Serum metabolites	C12 C10	13.63	0.00	-1.24
36	rs806276	chr6	91207351	Neurological behavioral	ADHD	3.49	21.48	1.22
37	rs7274811	chr20	32333181	Quantitative traits	Height	4.48	23.43	1.21
38	rs17001239	chr21	27002377	Neurological behavioral	Cognitive performance-PRM	20.31	3.22	-1.20
39	rs3825214	chr12	114795443	Cardiovascular	PR interval	0.00	12.91	1.20
40	rs3825214	chr12	114795443	Cardiovascular	QRS duration	0.00	12.91	1.20
41	rs3825214	chr12	114795443	Cardiovascular	QT interval	0.00	12.91	1.20
42	rs2580816	chr2	232797966	Quantitative traits	Height	25.44	5.79	-1.17
43	rs1880887	chr12	41721430	Serum metabolites	Alkaline phosphatase	7.46	29.08	1.16
44	rs28927680	chr11	116619073	Serum metabolites	Triglycerides	5.55	24.55	1.15
45	rs3744028	chr17	73888672	Neurological behavioral	White matter hyperintensity	18.95	3.05	-1.15
46	rs713875	chr22	30592487	Autoimmune disease	Crohns disease	0.00	11.75	1.12

47	rs7635839	chr3	191667777	Drug metabolism	Response to antipsychotic therapy-PR interval	0.00	11.32	1.09
48	rs2527866	chr7	157090296	Cardiovascular	Systolic blood pressure	14.09	1.31	-1.09
49	rs2901964	chr1	15792426	Miscellaneous	Erectile dysfunction and prostate cancer treatment	7.77	27.46	1.08
50	rs6812193	chr4	77198986	Aging	Parkinsons disease	13.58	1.23	-1.07
51	rs2061333	chr19	44614208	Aging	Alzheimers disease	20.53	4.64	-1.06
52	rs2738113	chr8	6829085	Miscellaneous	Endometriosis	10.69	0.00	-1.05
53	rs12127588	chr1	198595506	Hematological parameters	Mean corpuscular hemoglobin	10.58	0.00	-1.04
54	rs2042831	chr2	235857114	Aging	Longevity	2.90	16.53	1.04
55	rs6499640	chr16	53769677	Quantitative traits	BMI	10.49	0.00	-1.04
56	rs6499640	chr16	53769677	Quantitative traits	Weight	10.49	0.00	-1.04
57	rs6474359	chr8	41549194	Serum metabolites	HbA1C	0.00	10.40	1.03
58	rs571312	chr18	57839769	Quantitative traits	BMI	87.80	38.18	-1.02
59	rs11085824	chr19	13001547	Hematological parameters	Mean corpuscular hemoglobin	3.08	16.53	1.02
60	rs9866141	chr3	156950579	Diabetes	Diabetic retinopathy	17.15	3.42	-1.02
61	rs10786436	chr10	100300182	Diabetes	Type 1 diabetes	31.57	10.55	-1.02
62	rs3791950	chr2	218729865	Quantitative traits	Height	23.21	6.52	-1.01

65 COUP-TF1 Round:3

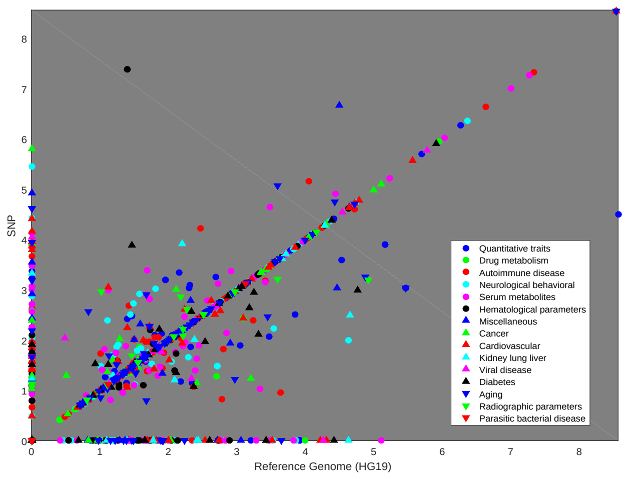

66 EAR2 Round:3

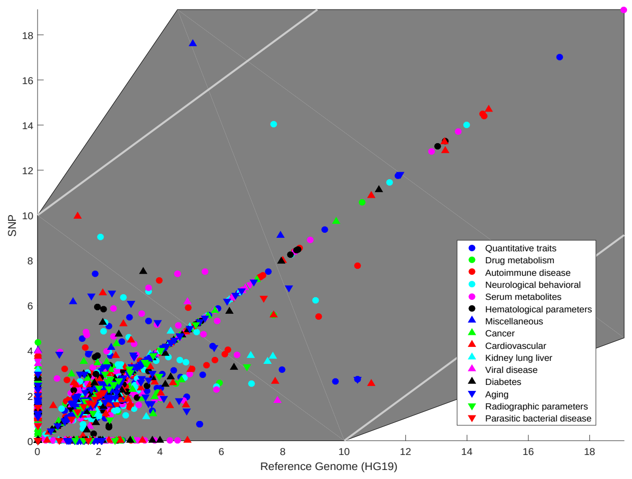

67 COUP-TF2+17 Round:3

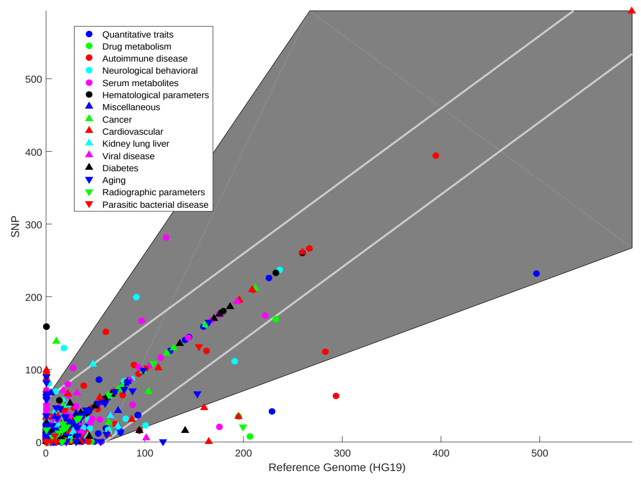

S.No.	SNP	Chr.	Position	Disease Class	Disease Trait	HG19	SNP	$log_2(\frac{SNP+\eta}{HG19+\eta})$
1	rs153091	chr16	13253956	Drug metabolism	Response to antipsychotic therapy perphenazine-triglycerides	206.52	7.30	-2.00
2	rs688034	chr22	26689635	Cardiovascular	Coronary heart disease	165.03	0.00	-1.92
3	rs10914144	chr1	171949750	Hematological parameters	Mean platelet volume	0.00	158.58	1.88
4	rs10914144	chr1	171949750	Hematological parameters	Platelet count	0.00	158.58	1.88
5	rs16826658	chr1	22485871	Miscellaneous	Endometriosis	91.14	448.43	1.75
6	rs4811196	chr20	36469694	Radiographic parameters	Bone mineral density-trochanter	199.76	21.07	-1.69
7	rs10412199	chr19	3927771	Aging	Aging traits-age free from disease	118.54	0.00	-1.58
8	rs1497406	chr1	16505320	Serum metabolites	Gamma glutamyl transferase	175.89	20.48	-1.56
9	rs11243676	chr9	135096767	Autoimmune disease	Systemic lupus erythematosus	293.39	63.14	-1.53
10	rs7315438	chr12	115891403	Cancer	Colorectal cancer	10.17	138.85	1.51
11	rs9470004	chr6	35341850	Quantitative traits	Height	228.65	42.36	-1.50
12	rs6495122	chr15	75125645	Drug metabolism	Caffeine intake	195.24	35.04	-1.43
13	rs6495122	chr15	75125645	Drug metabolism	coffee consumption	195.24	35.04	-1.43
14	rs6495122	chr15	75125645	Cardiovascular	Diastolic blood pressure	195.24	35.04	-1.43
15	rs10786436	chr10	100300182	Diabetes	Type 1 diabetes	141.13	15.44	-1.42
16	rs17609940	chr6	35034800	Cardiovascular	Coronary heart disease	0.00	99.21	1.42
17	rs8007846	chr14	66262963	Autoimmune disease	Multiple sclerosis-Brain Glutamate Concentrations	0.00	93.56	1.37
18	rs3793917	chr10	124219275	Aging	Age-related macular degeneration	0.00	89.53	1.33

19	rs558718	chr19	7909883	Viral disease	HIV progression	101.73	5.94	-1.30
20	rs8049439	chr16	28837515	Autoimmune disease	Inflammatory bowel disease-early onset	0.00	85.25	1.28
21	rs16977195	chr15	86984240	Neurological behavioral	Schizophrenia	18.15	128.93	1.28
22	rs536841	chr11	85787824	Aging	Alzheimers disease	0.00	81.29	1.24
23	rs16966460	chr15	38511983	Neurological behavioral	Bipolar disorder	2.46	80.52	1.18
24	rs2236653	chr11	126283785	Serum metabolites	Alkaline phosphatase	0.00	71.77	1.14
25	rs2008242	chr4	5221538	Cardiovascular	PR segment	0.00	67.82	1.10
26	rs2306677	chr12	26636386	Aging	Amyotrophic lateral sclerosis	0.00	66.70	1.09
27	rs11085824	chr19	13001547	Hematological parameters	Mean corpuscular hemoglobin	0.00	63.32	1.05
28	rs1395821	chr4	148047550	Cardiovascular	Coronary heart disease	160.52	47.60	-1.04
29	rs7153703	chr14	51919822	Aging	Alzheimers Total ventricular volume	0.00	62.32	1.04
30	rs1893217	chr18	12809340	Autoimmune disease	Celiac disease	94.26	16.05	-1.03
31	rs1893217	chr18	12809340	Autoimmune disease	Rheumatoid arthritis celiac disease	94.26	16.05	-1.03
32	rs1893217	chr18	12809340	Diabetes	Type 1 diabetes	94.26	16.05	-1.03

68 COUP-TF1+17 Round:3

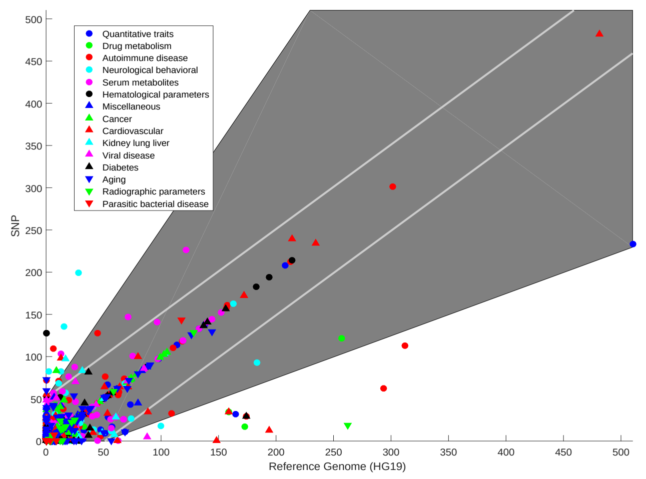

S.No.	SNP	Chr.	Position	Disease Class	Disease Trait	HG19	SNP	$log_2(\frac{SNP+\eta}{HG19+\eta})$
1	rs4811196	chr20	36469694	Radiographic parameters	Bone mineral density-trochanter	262.69	18.64	-2.17
2	rs688034	chr22	26689635	Cardiovascular	Coronary heart disease	148.04	0.00	-1.96
3	rs1395821	chr4	148047550	Cardiovascular	Coronary heart disease	194.18	12.30	-1.95
4	rs16826658	chr1	22485871	Miscellaneous	Endometriosis	87.66	474.65	1.92
5	rs10914144	chr1	171949750	Hematological parameters	Mean platelet volume	0.38	127.89	1.80
6	rs10914144	chr1	171949750	Hematological parameters	Platelet count	0.38	127.89	1.80
7	rs153091	chr16	13253956	Drug metabolism	Response to antipsychotic therapy perphenazine-triglycerides	172.56	17.10	-1.71
8	rs7178909	chr15	90447946	Neurological behavioral	Common traits optimism	28.48	199.65	1.66
9	rs11243676	chr9	135096767	Autoimmune disease	Systemic lupus erythematosus	293.25	62.38	-1.60
10	rs8049439	chr16	28837515	Autoimmune disease	Inflammatory bowel disease-early onset	6.18	109.51	1.49
11	rs1893217	chr18	12809340	Autoimmune disease	Celiac disease	174.09	29.21	-1.49
12	rs1893217	chr18	12809340	Autoimmune disease	Rheumatoid arthritis celiac disease	174.09	29.21	-1.49
13	rs1893217	chr18	12809340	Diabetes	Type 1 diabetes	174.09	29.21	-1.49
14	rs16977195	chr15	86984240	Neurological behavioral	Schizophrenia	15.62	135.47	1.48
15	rs9470004	chr6	35341850	Quantitative traits	Height	165.02	31.99	-1.38
16	rs558718	chr19	7909883	Viral disease	HIV progression	88.00	4.61	-1.32
17	rs16966460	chr15	38511983	Neurological behavioral	Bipolar disorder	2.60	82.59	1.32
18	rs6495122	chr15	75125645	Drug metabolism	Caffeine intake	158.79	34.36	-1.30
19	rs6495122	chr15	75125645	Drug metabolism	coffee consumption	158.79	34.36	-1.30

20	rs6495122	chr15	75125645	Cardiovascular	Diastolic blood pressure	158.79	34.36	-1.30
21	rs3793917	chr10	124219275	Aging	Age-related macular degeneration	0.00	72.88	1.28
22	rs8109578	chr19	10213154	Serum metabolites	Thyroid stimulating hormone	12.83	103.02	1.27
23	rs8007846	chr14	66262963	Autoimmune disease	Multiple sclerosis-Brain Glutamate Concentrations	0.00	72.03	1.27
24	rs17609940	chr6	35034800	Cardiovascular	Coronary heart disease	13.12	98.49	1.22
25	rs7315438	chr12	115891403	Cancer	Colorectal cancer	8.64	83.47	1.17
26	rs211718	chr1	76106675	Serum metabolites	C12 C10	63.01	0.00	-1.16
27	rs10947055	chr6	30093364	Cardiovascular	Cardiac hypertrophy	61.93	0.00	-1.15
28	rs6441286	chr3	159728878	Autoimmune disease	Primary biliary cirrhosis	312.05	113.08	-1.15
29	rs4356203	chr11	17160148	Neurological behavioral	Schizophrenia	100.00	18.00	-1.13
30	rs2869967	chr4	89869332	Kidney lung liver	FEV1/FVC	16.75	97.25	1.13
31	rs7153703	chr14	51919822	Aging	Alzheimers Total ventricular volume	0.00	59.68	1.12
32	rs2061333	chr19	44614208	Aging	Alzheimers disease	57.04	0.00	-1.08
33	rs10227331	chr7	157294938	Neurological behavioral	ADHD-Inattentive symptoms	12.93	82.43	1.06
34	rs2008242	chr4	5221538	Cardiovascular	PR segment	0.00	55.44	1.06
35	rs11085824	chr19	13001547	Hematological parameters	Mean corpuscular hemoglobin	0.00	53.61	1.04
36	rs806276	chr6	91207351	Neurological behavioral	ADHD	0.00	53.26	1.03
37	rs7669317	chr4	106457330	Drug metabolism	Response to antipsychotic therapy extrapyramidal side effects	0.00	53.12	1.03

69 COUP-TF2:RXRA Round:3

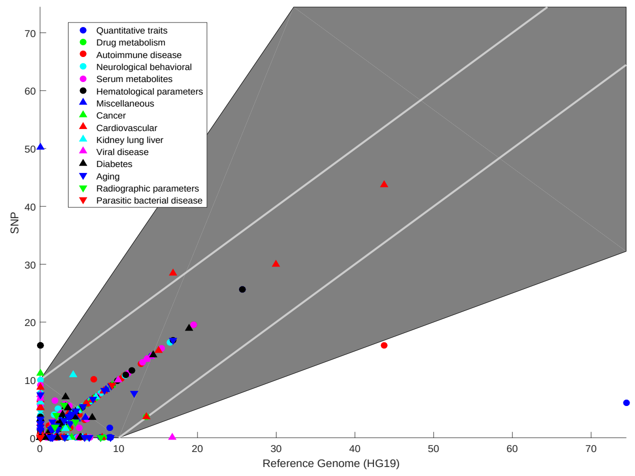

S.No.	SNP	Chr.	Position	Disease Class	Disease Trait	HG19	SNP	$log_2(\frac{SNP+\eta}{HG19+\eta})$
1	rs16826658	chr1	22485871	Miscellaneous	Endometriosis	0.00	50.14	2.59
2	rs571312	chr18	57839769	Quantitative traits	BMI	74.45	6.04	-2.40
3	rs558718	chr19	7909883	Viral disease	HIV progression	16.81	0.00	-1.42
4	rs10914144	chr1	171949750	Hematological parameters	Mean platelet volume	0.00	16.02	1.38
5	rs10914144	chr1	171949750	Hematological parameters	Platelet count	0.00	16.02	1.38
6	rs10411161	chr19	52372976	Cancer	Breast cancer	0.00	11.08	1.08
7	rs6441286	chr3	159728878	Autoimmune disease	Primary biliary cirrhosis	43.67	16.00	-1.05

70 COUP-TF1:RXRA Round:3

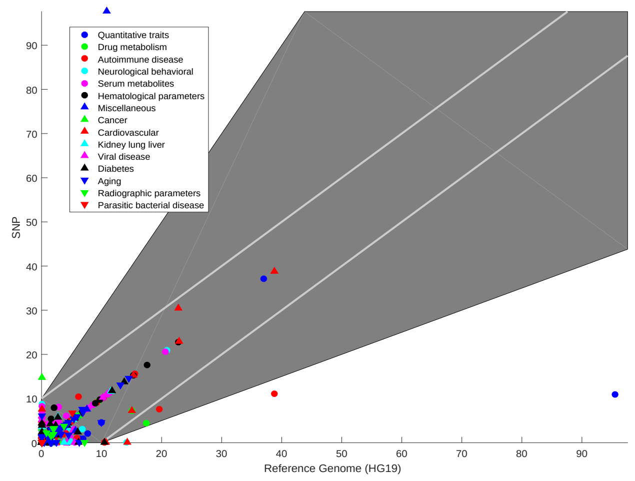

S.No.	SNP	Chr.	Position	Disease Class	Disease Trait	HG19	SNP	$log_2(\frac{SNP+\eta}{HG19+\eta})$
1	rs16826658	chr1	22485871	Miscellaneous	Endometriosis	10.88	97.61	2.37
2	rs571312	chr18	57839769	Quantitative traits	BMI	95.50	10.88	-2.34
3	rs7315438	chr12	115891403	Cancer	Colorectal cancer	0.00	14.72	1.31
4	rs1395821	chr4	148047550	Cardiovascular	Coronary heart disease	14.23	0.00	-1.28
5	rs4996815	chr13	106651661	Neurological behavioral	Biplolar disorder and schizophrenia	14.21	0.00	-1.28
6	rs6441286	chr3	159728878	Autoimmune disease	Primary biliary cirrhosis	38.73	11.17	-1.20
7	rs688034	chr22	26689635	Cardiovascular	Coronary heart disease	10.76	0.00	-1.05
8	rs1893217	chr18	12809340	Autoimmune disease	Celiac disease	10.47	0.00	-1.03
9	rs1893217	chr18	12809340	Autoimmune disease	Rheumatoid arthritis celiac disease	10.47	0.00	-1.03
10	rs1893217	chr18	12809340	Diabetes	Type 1 diabetes	10.47	0.00	-1.03

71 EAR2:RXRA Round:3

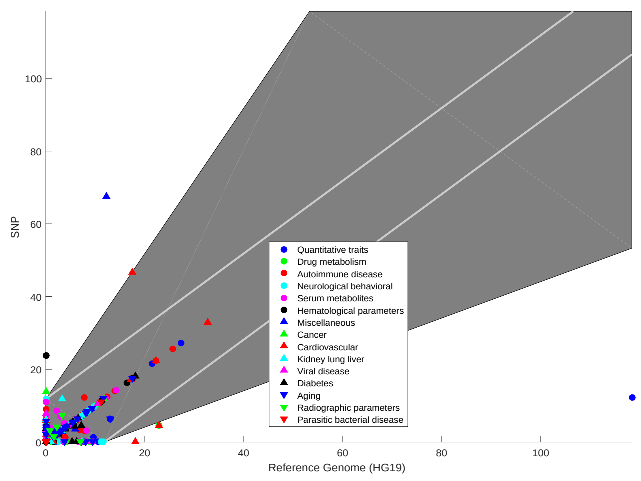

S.No.	SNP	Chr.	Position	Disease Class	Disease Trait	HG19	SNP	$log_2(\frac{SNP+\eta}{HG19+\eta})$
1	rs571312	chr18	57839769	Quantitative traits	BMI	118.42	12.31	-2.43
2	rs16826658	chr1	22485871	Miscellaneous	Endometriosis	12.31	67.58	1.72
3	rs10914144	chr1	171949750	Hematological parameters	Mean platelet volume	0.00	23.78	1.59
4	rs10914144	chr1	171949750	Hematological parameters	Platelet count	0.00	23.78	1.59
5	rs688034	chr22	26689635	Cardiovascular	Coronary heart disease	18.07	0.00	-1.34
6	rs10411161	chr19	52372976	Cancer	Breast cancer	0.00	13.81	1.11
7	rs6495122	chr15	75125645	Drug metabolism	Caffeine intake	22.92	4.62	-1.08
8	rs6495122	chr15	75125645	Drug metabolism	coffee consumption	22.92	4.62	-1.08
9	rs6495122	chr15	75125645	Cardiovascular	Diastolic blood pressure	22.92	4.62	-1.08
10	rs6017291	chr20	42854134	Neurological behavioral	Delayed Story Recall	0.00	11.90	1.00

72 HNF4G Round:3

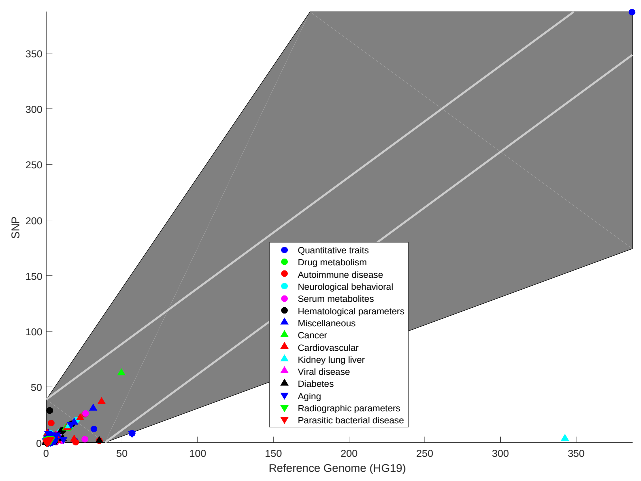

S.No.	SNP	Chr.	Position	Disease Class	Disease Trait	HG19	SNP	$log_2(\frac{SNP+\eta}{HG19+\eta})$
1	rs641525	chr8	2740502	Kidney lung liver	COPD	342.77	3.45	-3.18
2	rs314277	chr6	105407662	Quantitative traits	Digit length ratio	56.58	8.17	-1.02
3	rs314277	chr6	105407662	Quantitative traits	Height	56.58	8.17	-1.02
4	rs314277	chr6	105407662	Aging	Age at menarche	56.58	8.17	-1.02

73 HNF4A Round:3

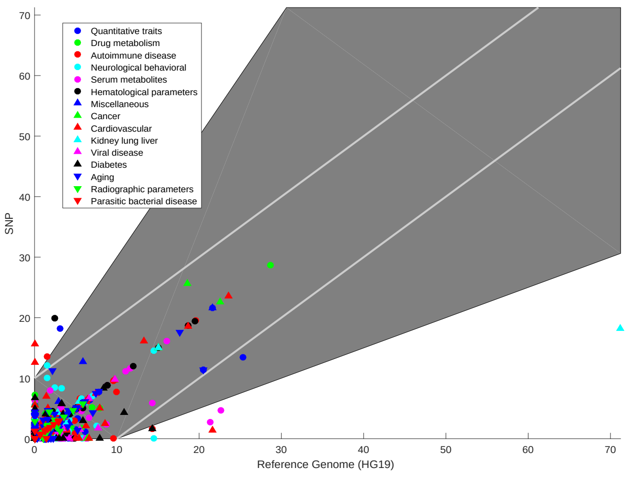

S.No.	SNP	Chr.	Position	Disease Class	Disease Trait	HG19	SNP	$log_2(\frac{SNP+\eta}{HG19+\eta})$
1	rs641525	chr8	2740502	Kidney lung liver	COPD	71.24	18.22	-1.53
2	rs17706439	chr1	213855588	Cardiovascular	RR interval	21.60	1.37	-1.47
3	rs17609940	chr6	35034800	Cardiovascular	Coronary heart disease	0.00	15.67	1.36
4	rs1967017	chr1	145723645	Serum metabolites	Serum urate	21.39	2.68	-1.31
5	rs4996815	chr13	106651661	Neurological behavioral	Biplolar disorder and schizophrenia	14.49	0.00	-1.29
6	rs10914144	chr1	171949750	Hematological parameters	Mean platelet volume	2.50	19.89	1.26
7	rs10914144	chr1	171949750	Hematological parameters	Platelet count	2.50	19.89	1.26
8	rs2008242	chr4	5221538	Cardiovascular	PR segment	0.00	12.61	1.18
9	rs12355784	chr10	65121565	Serum metabolites	Alkaline phosphatase	22.67	4.65	-1.16
10	rs757608	chr17	59497277	Quantitative traits	Height	3.06	18.22	1.11
11	rs1893217	chr18	12809340	Autoimmune disease	Celiac disease	14.35	1.64	-1.06
12	rs1893217	chr18	12809340	Autoimmune disease	Rheumatoid arthritis celiac disease	14.35	1.64	-1.06
13	rs1893217	chr18	12809340	Diabetes	Type 1 diabetes	14.35	1.64	-1.06
14	rs3745516	chr19	50926742	Autoimmune disease	Primary biliary cirrhosis	1.53	13.59	1.03

74 HNF4A+18 Round:3

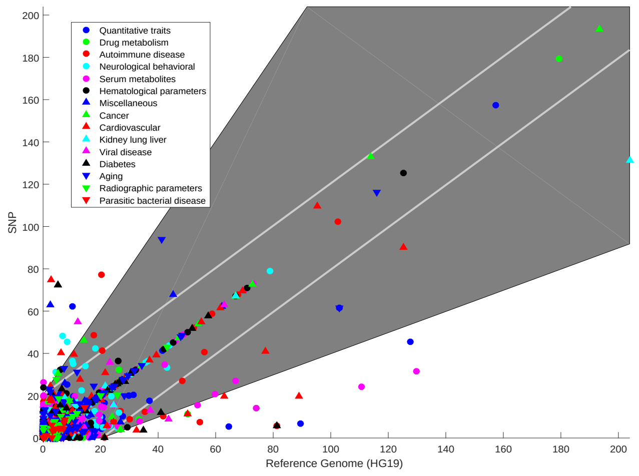

S.No.	SNP	Chr.	Position	Disease Class	Disease Trait	HG19	SNP	$log_2(\frac{SNP+\eta}{HG19+\eta})$
1	rs10914144	chr1	171949750	Hematological parameters	Mean platelet volume	17.53	146.21	2.14
2	rs10914144	chr1	171949750	Hematological parameters	Platelet count	17.53	146.21	2.14
3	rs2008242	chr4	5221538	Cardiovascular	PR segment	2.67	74.73	2.04
4	rs2778031	chr9	90835726	Quantitative traits	Height	89.37	6.94	-2.01
5	rs1893217	chr18	12809340	Autoimmune disease	Celiac disease	81.39	5.90	-1.95
6	rs1893217	chr18	12809340	Autoimmune disease	Rheumatoid arthritis celiac disease	81.39	5.90	-1.95
7	rs1893217	chr18	12809340	Diabetes	Type 1 diabetes	81.39	5.90	-1.95
8	rs10034228	chr4	112611750	Miscellaneous	Myopia pathological	2.46	62.83	1.86
9	rs4788084	chr16	28539848	Diabetes	Type 1 diabetes	5.04	72.17	1.86
10	rs4788084	chr16	28539848	Diabetes	Type 1 diabetes autoantibodies	5.04	72.17	1.86
11	rs6496932	chr15	85825567	Quantitative traits	Central corneal thickness	64.56	5.50	-1.71
12	rs12355784	chr10	65121565	Serum metabolites	Alkaline phosphatase	110.62	24.32	-1.55
13	rs1967017	chr1	145723645	Serum metabolites	Serum urate	129.83	31.61	-1.53
14	rs17706439	chr1	213855588	Cardiovascular	RR interval	88.89	19.52	-1.45
15	rs2240466	chr7	72856269	Drug metabolism	Caffeine intake	74.14	14.27	-1.45
16	rs2240466	chr7	72856269	Serum metabolites	Triglycerides	74.14	14.27	-1.45
17	rs805297	chr6	31622606	Autoimmune disease	Rheumatoid arthritis	54.52	7.34	-1.43
18	rs757608	chr17	59497277	Quantitative traits	Height	10.23	62.31	1.43
19	rs860554	chr1	201262432	Neurological behavioral	Panic disorder	6.85	48.41	1.34
20	rs3745516	chr19	50926742	Autoimmune disease	Primary biliary cirrhosis	20.23	77.27	1.27
21	rs2593321	chr3	22122396	Viral disease	HIV progression	12.11	55.04	1.21
22	rs17609940	chr6	35034800	Cardiovascular	Coronary heart disease	6.15	40.35	1.19
23	rs2212361	chr11	94312323	Neurological behavioral	ADHD	8.44	45.60	1.19

24	rs3775948	chr4	9995182	Serum metabolites	Serum urate	0.00	26.26	1.19
25	rs358806	chr3	55313400	Diabetes	Type 2 diabetes	34.74	3.79	-1.19
26	rs571312	chr18	57839769	Quantitative traits	BMI	127.67	45.46	-1.17
27	rs6495122	chr15	75125645	Drug metabolism	Caffeine intake	50.15	11.37	-1.15
28	rs6495122	chr15	75125645	Drug metabolism	coffee consumption	50.15	11.37	-1.15
29	rs6495122	chr15	75125645	Cardiovascular	Diastolic blood pressure	50.15	11.37	-1.15
30	rs2527866	chr7	157090296	Cardiovascular	Systolic blood pressure	32.35	3.59	-1.14
31	rs16948255	chr16	74881820	Viral disease	HIV progression	43.58	8.74	-1.13
32	rs1719271	chr15	65183801	Hematological parameters	Platelet count	0.00	23.98	1.12
33	rs9302001	chr13	95463392	Neurological behavioral	Panic disorder	4.36	31.20	1.06
34	rs1395821	chr4	148047550	Cardiovascular	Coronary heart disease	63.00	19.71	-1.06
35	rs675209	chr6	7102084	Serum metabolites	Serum urate	53.67	15.58	-1.04
36	rs4085613	chr1	152550018	Autoimmune disease	Psoriasis	21.41	0.00	-1.04
37	rs10460009	chr18	2948029	Diabetes	Type 2 diabetes	21.37	0.00	-1.03
38	rs2058660	chr2	103054449	Autoimmune disease	Crohns disease	41.88	10.12	-1.03
39	rs2893923	chr10	65261184	Hematological parameters	Platelet aggregation-epinephrine	5.96	32.34	1.00

75 RXRB Round:3

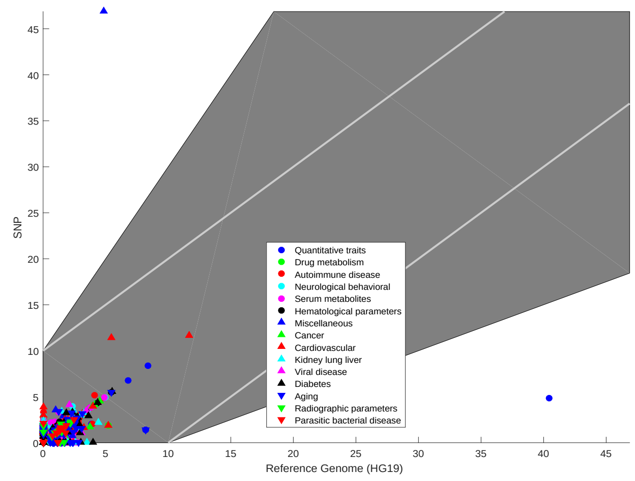

S.No.	SNP	Chr.	Position	Disease Class	Disease Trait	HG19	SNP	$log_2(\frac{SNP+\eta}{HG19+\eta})$
1	rs16826658	chr1	22485871	Miscellaneous	Endometriosis	4.82	46.87	1.94
2	rs571312	chr18	57839769	Quantitative traits	BMI	40.45	4.82	-1.77

76 RXRB+17 Round:3

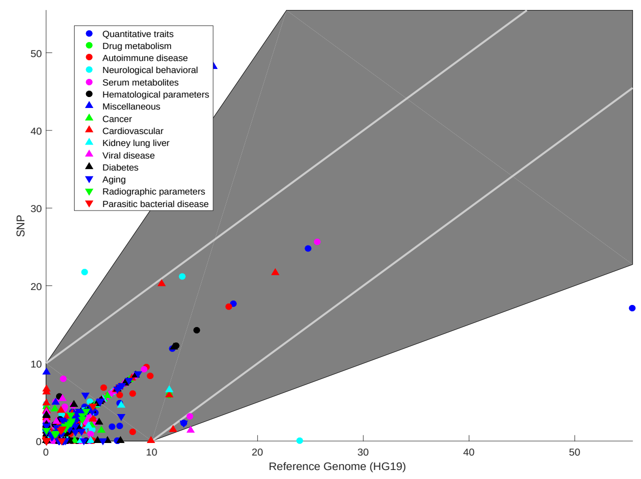

S.No.	SNP	Chr.	Position	Disease Class	Disease Trait	HG19	SNP	$log_2(\frac{SNP+\eta}{HG19+\eta})$
1	rs4996815	chr13	106651661	Neurological behavioral	Biplolar disorder and schizophrenia	23.99	0.00	-1.76
2	rs8109578	chr19	10213154	Serum metabolites	Thyroid stimulating hormone	7.30	32.72	1.30
3	rs571312	chr18	57839769	Quantitative traits	BMI	55.47	17.07	-1.27
4	rs16977195	chr15	86984240	Neurological behavioral	Schizophrenia	3.66	21.76	1.22
5	rs16826658	chr1	22485871	Miscellaneous	Endometriosis	15.81	48.26	1.17
6	rs558718	chr19	7909883	Viral disease	HIV progression	13.70	1.40	-1.06

77 RXRG Round:3

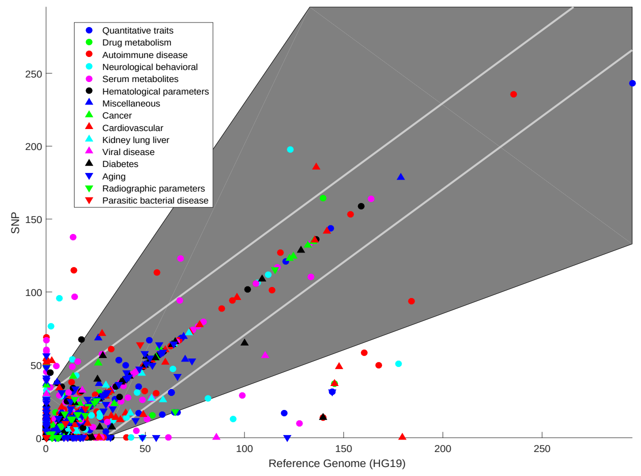

S.No.	SNP	Chr.	Position	Disease Class	Disease Trait	HG19	SNP	$log_2(\frac{SNP+\eta}{HG19+\eta})$
1	rs688034	chr22	26689635	Cardiovascular	Coronary heart disease	179.68	0.00	-2.82
2	rs2061333	chr19	44614208	Aging	Alzheimers disease	121.44	0.00	-2.35
3	rs1076540	chr22	18439958	Serum metabolites	Gamma glutamyl transferase	127.76	10.02	-1.99
4	rs558718	chr19	7909883	Viral disease	HIV progression	85.85	0.00	-1.97
5	rs1893217	chr18	12809340	Autoimmune disease	Celiac disease	139.50	14.05	-1.96
6	rs1893217	chr18	12809340	Autoimmune disease	Rheumatoid arthritis celiac disease	139.50	14.05	-1.96
7	rs1893217	chr18	12809340	Diabetes	Type 1 diabetes	139.50	14.05	-1.96
8	rs12459897	chr19	31596778	Serum metabolites	Serum polyunsaturated fatty acids	13.80	137.70	1.95
9	rs16977195	chr15	86984240	Neurological behavioral	Schizophrenia	6.64	95.53	1.79
10	rs7774434	chr6	32657578	Autoimmune disease	Primary biliary cirrhosis	13.89	115.03	1.74
11	rs8007846	chr14	66262963	Autoimmune disease	Multiple sclerosis-Brain Glutamate Concentrations	0.00	68.71	1.73
12	rs16966460	chr15	38511983	Neurological behavioral	Bipolar disorder	2.50	76.45	1.73
13	rs8109578	chr19	10213154	Serum metabolites	Thyroid stimulating hormone	39.29	195.58	1.71
14	rs2236653	chr11	126283785	Serum metabolites	Alkaline phosphatase	0.00	66.93	1.71
15	rs9470004	chr6	35341850	Quantitative traits	Height	119.95	16.70	-1.69
16	rs211718	chr1	76106675	Serum metabolites	C12 C10	61.67	0.00	-1.63
17	rs4875320	chr8	4164013	Serum metabolites	HDL cholesterol	0.00	60.37	1.61
18	rs3093030	chr19	10397403	Serum metabolites	ICAM1	0.00	59.10	1.59
19	rs4356203	chr11	17160148	Neurological behavioral	Schizophrenia	94.44	12.64	-1.56

20	rs3793917	chr10	124219275	Aging	Age-related macular degeneration	0.00	56.40	1.54
21	rs10412199	chr19	3927771	Aging	Aging traits-age free from disease	55.40	0.00	-1.52
22	rs1016988	chr5	131744574	Serum metabolites	Fibrinogen	14.43	96.48	1.52
23	rs2008242	chr4	5221538	Cardiovascular	PR segment	0.00	54.26	1.50
24	rs314277	chr6	105407662	Quantitative traits	Digit length ratio	144.28	31.75	-1.50
25	rs314277	chr6	105407662	Quantitative traits	Height	144.28	31.75	-1.50
26	rs314277	chr6	105407662	Aging	Age at menarche	144.28	31.75	-1.50
27	rs3745516	chr19	50926742	Autoimmune disease	Primary biliary cirrhosis	0.00	53.27	1.49
28	rs17672135	chr1	240445596	Cardiovascular	Coronary heart disease	0.00	51.79	1.46
29	rs848353	chr7	108548660	Cancer	Renal cell carcinoma	0.00	50.69	1.44
30	rs806276	chr6	91207351	Neurological behavioral	ADHD	0.00	50.11	1.43
31	rs8049439	chr16	28837515	Autoimmune disease	Inflammatory bowel disease-early onset	0.00	49.16	1.41
32	rs7669317	chr4	106457330	Drug metabolism	Response to antipsychotic therapy extrapyramidal side effects	0.00	48.96	1.41
33	rs2823819	chr21	17828291	Neurological behavioral	ADHD	48.56	0.00	-1.40
34	rs2782931	chr9	114850190	Aging	Amyotrophic lateral sclerosis	48.51	0.00	-1.40
35	rs7153703	chr14	51919822	Aging	Alzheimers Total ventricular volume	0.00	48.21	1.40
36	rs6495122	chr15	75125645	Drug metabolism	Caffeine intake	145.40	37.17	-1.39
37	rs6495122	chr15	75125645	Drug metabolism	coffee consumption	145.40	37.17	-1.39
38	rs6495122	chr15	75125645	Cardiovascular	Diastolic blood pressure	145.40	37.17	-1.39
39	rs4996815	chr13	106651661	Neurological behavioral	Biplolar disorder and schizophrenia	177.69	50.91	-1.37
40	rs2212361	chr11	94312323	Neurological behavioral	ADHD	0.00	45.17	1.34
41	rs254893	chr5	171076227	Cardiovascular	Diastolic blood pressure	3.06	52.79	1.34
42	rs2194980	chr12	115502718	Serum metabolites	Tyrosine	0.00	44.98	1.33
43	rs2188962	chr5	131770805	Autoimmune disease	Crohns disease	167.80	49.75	-1.32
44	rs904251	chr6	37451696	Neurological behavioral	Cognitive performance-SWM Strategy	42.79	0.00	-1.29
45	rs16826658	chr1	22485871	Miscellaneous	Endometriosis	78.21	232.85	1.28
46	rs1562990	chr11	60023087	Aging	Alzheimers disease	0.00	41.02	1.26

47	rs6554809	chr5	13740976	Autoimmune disease	IgE grass sensitization	40.37	0.00	-1.24
48	rs1539019	chr1	247600301	Serum metabolites	Fibrinogen	0.00	40.29	1.24
49	rs10914144	chr1	171949750	Hematological parameters	Mean platelet volume	2.18	44.88	1.23
50	rs10914144	chr1	171949750	Hematological parameters	Platelet count	2.18	44.88	1.23
51	rs3112530	chr5	152639677	Aging	Aging traits-age free from disease	0.00	39.61	1.23
52	rs41360247	chr2	44073656	Serum metabolites	Campesterol	5.03	49.24	1.19
53	rs1395821	chr4	148047550	Cardiovascular	Coronary heart disease	147.51	48.85	-1.18
54	rs10760706	chr9	102723692	Miscellaneous	Alopecia areata	0.00	36.92	1.17
55	rs4332037	chr7	1950809	Neurological behavioral	Bipolar disorder	0.00	36.07	1.15
56	rs7315438	chr12	115891403	Cancer	Colorectal cancer	0.00	35.99	1.15
57	rs1497406	chr1	16505320	Serum metabolites	Gamma glutamyl transferase	98.96	28.85	-1.14
58	rs1591830	chr6	150651900	Serum metabolites	Phosphorus-serum	45.58	4.64	-1.14
59	rs1458175	chr12	41965861	Autoimmune disease	Multiple sclerosis	160.52	58.34	-1.11
60	rs2369304	chr14	96206070	Hematological parameters	Neutrophil count	32.69	0.00	-1.08
61	rs3865444	chr19	51727962	Aging	Alzheimers disease late onset	32.32	0.00	-1.07
62	rs1202199	chr6	20156174	Neurological behavioral	ADHD-Hyperactive-impulsive symptoms	0.00	32.32	1.07
63	rs941207	chr12	57023284	Hematological parameters	Platelet count	17.76	67.28	1.03
64	rs2691543	chr7	77964788	Quantitative traits	Height	30.85	0.00	-1.03
65	rs2292303	chr12	102513531	Quantitative traits	Height	66.25	17.59	-1.02
66	rs3102947	chr2	8434185	Serum metabolites	Serum IgE levels	2.22	34.75	1.02
67	rs3791675	chr2	56111309	Quantitative traits	Height	0.00	30.03	1.01
68	rs6139030	chr20	3187733	Viral disease	Response to hepatitis C treatment	0.00	29.82	1.01
69	rs12946454	chr17	43208121	Cardiovascular	Systolic blood pressure	0.00	29.68	1.00
70	rs6971925	chr7	14445917	Drug metabolism	Response to treatment for acute lymphoblastic leukemia	0.00	29.66	1.00

78 RXRG:RXRA Round:3

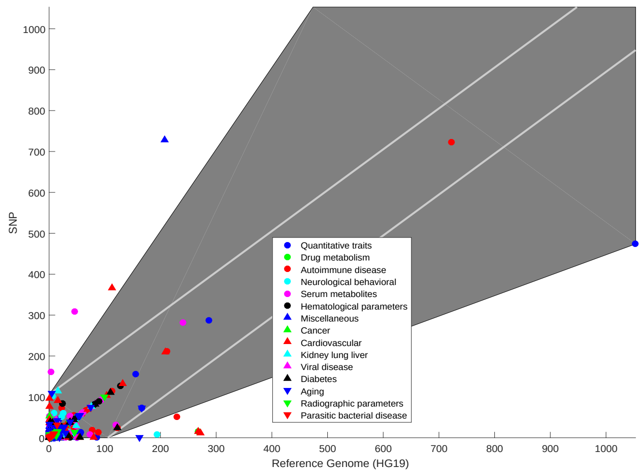

S.No.	SNP	Chr.	Position	Disease Class	Disease Trait	HG19	SNP	$log_2(\frac{SNP+\eta}{HG19+\eta})$
1	rs1395821	chr4	148047550	Cardiovascular	Coronary heart disease	272.40	11.74	-1.69
2	rs6495122	chr15	75125645	Drug metabolism	Caffeine intake	267.73	15.83	-1.62
3	rs6495122	chr15	75125645	Drug metabolism	coffee consumption	267.73	15.83	-1.62
4	rs6495122	chr15	75125645	Cardiovascular	Diastolic blood pressure	267.73	15.83	-1.62
5	rs8109578	chr19	10213154	Serum metabolites	Thyroid stimulating hormone	45.71	308.69	1.45
6	rs16826658	chr1	22485871	Miscellaneous	Endometriosis	208.08	728.94	1.41
7	rs4996815	chr13	106651661	Neurological behavioral	Biplolar disorder and schizophrenia	194.21	8.47	-1.40
8	rs2061333	chr19	44614208	Aging	Alzheimers disease	162.53	0.00	-1.35
9	rs12459897	chr19	31596778	Serum metabolites	Serum polyunsaturated fatty acids	2.77	160.64	1.30
10	rs11781551	chr8	123408091	Cardiovascular	Internal carotid intimal medial thickness	113.60	367.14	1.11
11	rs6441286	chr3	159728878	Autoimmune disease	Primary biliary cirrhosis	229.09	50.98	-1.10

79 RXRG+17 Round:3

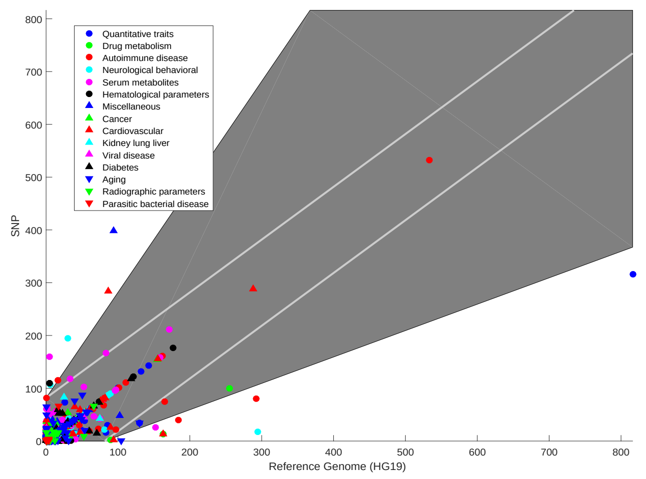

S.No.	SNP	Chr.	Position	Disease Class	Disease Trait	HG19	SNP	$log_2(\frac{SNP+\eta}{HG19+\eta})$
1	rs4996815	chr13	106651661	Neurological behavioral	Biplolar disorder and schizophrenia	294.67	17.72	-1.92
2	rs12459897	chr19	31596778	Serum metabolites	Serum polyunsaturated fatty acids	5.00	159.09	1.47
3	rs16826658	chr1	22485871	Miscellaneous	Endometriosis	93.41	398.70	1.46
4	rs6495122	chr15	75125645	Drug metabolism	Caffeine intake	162.59	13.28	-1.36
5	rs6495122	chr15	75125645	Drug metabolism	coffee consumption	162.59	13.28	-1.36
6	rs6495122	chr15	75125645	Cardiovascular	Diastolic blood pressure	162.59	13.28	-1.36
7	rs7178909	chr15	90447946	Neurological behavioral	Common traits optimism	29.92	194.11	1.31
8	rs6441286	chr3	159728878	Autoimmune disease	Primary biliary cirrhosis	292.01	79.85	-1.21
9	rs2061333	chr19	44614208	Aging	Alzheimers disease	104.48	0.00	-1.19
10	rs571312	chr18	57839769	Quantitative traits	BMI	816.13	316.33	-1.17
11	rs10914144	chr1	171949750	Hematological parameters	Mean platelet volume	4.35	108.90	1.15
12	rs10914144	chr1	171949750	Hematological parameters	Platelet count	4.35	108.90	1.15
13	rs1458175	chr12	41965861	Autoimmune disease	Multiple sclerosis	183.71	40.19	-1.12
14	rs11781551	chr8	123408091	Cardiovascular	Internal carotid intimal medial thickness	86.27	283.42	1.12
15	rs1497406	chr1	16505320	Serum metabolites	Gamma glutamyl transferase	152.79	26.38	-1.12
16	rs16977195	chr15	86984240	Neurological behavioral	Schizophrenia	6.33	106.51	1.10
17	rs688034	chr22	26689635	Cardiovascular	Coronary heart disease	93.52	1.64	-1.07
18	rs153091	chr16	13253956	Drug metabolism	Response to antipsychotic therapy perphenazine-triglycerides	89.28	2.09	-1.03

19	rs6720394	chr2	111989372	Autoimmune disease	Primary sclerosing cholangitis	16.28	114.88	1.01
20	rs8007846	chr14	66262963	Autoimmune disease	Multiple sclerosis-Brain Glutamate Concentrations	0.00	81.92	1.00

80 RXRA Round:3

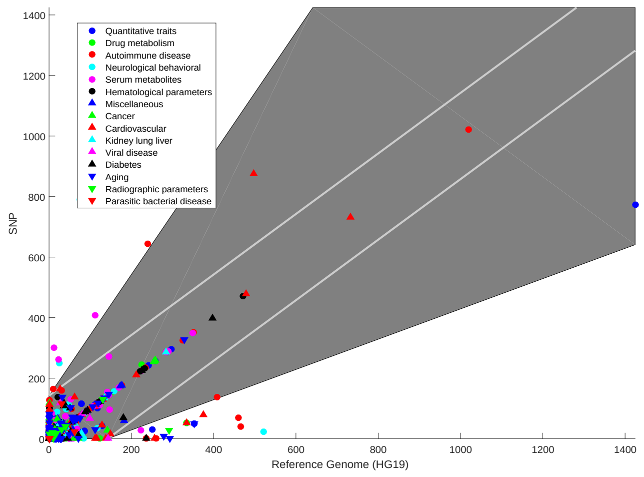

S.No.	SNP	Chr.	Position	Disease Class	Disease Trait	HG19	SNP	$log_2(\frac{SNP+\eta}{HG19+\eta})$
1	rs7178909	chr15	90447946	Neurological behavioral	Common traits optimism	75.41	789.21	2.10
2	rs4996815	chr13	106651661	Neurological behavioral	Biplolar disorder and schizophrenia	522.15	24.12	-2.00
3	rs2188962	chr5	131770805	Autoimmune disease	Crohns disease	465.70	40.22	-1.74
4	rs2061333	chr19	44614208	Aging	Alzheimers disease	293.08	0.00	-1.61
5	rs12459897	chr19	31596778	Serum metabolites	Serum polyunsaturated fatty acids	11.98	300.30	1.52
6	rs1458175	chr12	41965861	Autoimmune disease	Multiple sclerosis	460.75	70.31	-1.50
7	rs13314993	chr3	33015469	Autoimmune disease	Celiac disease	260.94	0.00	-1.50
8	rs536841	chr11	85787824	Aging	Alzheimers disease	278.81	7.95	-1.49
9	rs688034	chr22	26689635	Cardiovascular	Coronary heart disease	256.33	0.00	-1.49
10	rs1893217	chr18	12809340	Autoimmune disease	Celiac disease	236.26	0.00	-1.41
11	rs1893217	chr18	12809340	Autoimmune disease	Rheumatoid arthritis celiac disease	236.26	0.00	-1.41
12	rs1893217	chr18	12809340	Diabetes	Type 1 diabetes	236.26	0.00	-1.41
13	rs314277	chr6	105407662	Quantitative traits	Digit length ratio	352.80	49.05	-1.37
14	rs314277	chr6	105407662	Quantitative traits	Height	352.80	49.05	-1.37
15	rs314277	chr6	105407662	Aging	Age at menarche	352.80	49.05	-1.37
16	rs4811196	chr20	36469694	Radiographic parameters	Bone mineral density-trochanter	290.81	28.06	-1.35
17	rs6495122	chr15	75125645	Drug metabolism	Caffeine intake	334.99	51.32	-1.30
18	rs6495122	chr15	75125645	Drug metabolism	coffee consumption	334.99	51.32	-1.30
19	rs6495122	chr15	75125645	Cardiovascular	Diastolic blood pressure	334.99	51.32	-1.30
20	rs8109578	chr19	10213154	Serum metabolites	Thyroid stimulating hormone	22.93	262.76	1.29

21	rs1395821	chr4	148047550	Cardiovascular	Coronary heart disease	374.27	78.42	-1.23
22	rs16977195	chr15	86984240	Neurological behavioral	Schizophrenia	25.83	250.25	1.22
23	rs9470004	chr6	35341850	Quantitative traits	Height	250.89	29.88	-1.19
24	rs1016988	chr5	131744574	Serum metabolites	Fibrinogen	111.79	407.82	1.11
25	rs1497406	chr1	16505320	Serum metabolites	Gamma glutamyl transferase	223.63	26.97	-1.11
26	rs16826658	chr1	22485871	Miscellaneous	Endometriosis	387.59	969.65	1.07
27	rs11574637	chr16	31368874	Autoimmune disease	Systemic lupus erythematosus	240.36	643.68	1.04
28	rs7774434	chr6	32657578	Autoimmune disease	Primary biliary cirrhosis	10.19	164.71	1.01
29	rs558718	chr19	7909883	Viral disease	HIV progression	143.92	0.00	-1.01

81 RXRA+17 Round:3

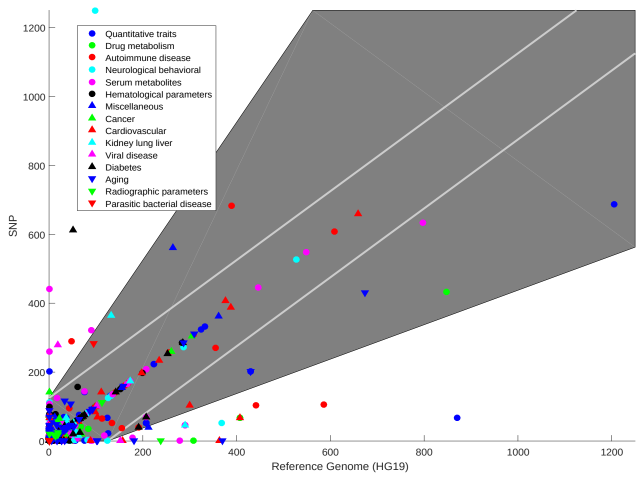

S.No.	SNP	Chr.	Position	Disease Class	Disease Trait	HG19	SNP	$log_2(\frac{SNP+\eta}{HG19+\eta})$
1	rs16977195	chr15	86984240	Neurological behavioral	Schizophrenia	97.93	1249.93	2.62
2	rs9470004	chr6	35341850	Quantitative traits	Height	869.78	66.84	-2.37
3	rs6474359	chr8	41549194	Serum metabolites	HbA1C	0.00	440.84	2.18
4	rs2281808	chr20	1610551	Diabetes	Type 1 diabetes	50.67	612.16	2.07
5	rs8109578	chr19	10213154	Serum metabolites	Thyroid stimulating hormone	78.57	725.60	2.06
6	rs7178909	chr15	90447946	Neurological behavioral	Common traits optimism	149.15	1012.61	2.05
7	rs2061333	chr19	44614208	Aging	Alzheimers disease	370.10	0.00	-1.99
8	rs688034	chr22	26689635	Cardiovascular	Coronary heart disease	363.38	0.00	-1.97
9	rs153091	chr16	13253956	Drug metabolism	Response to antipsychotic therapy perphenazine-triglycerides	307.78	0.00	-1.79
10	rs1076540	chr22	18439958	Serum metabolites	Gamma glutamyl transferase	277.95	0.00	-1.69
11	rs6441286	chr3	159728878	Autoimmune disease	Primary biliary cirrhosis	586.54	105.96	-1.62
12	rs12459897	chr19	31596778	Serum metabolites	Serum polyunsaturated fatty acids	0.00	259.65	1.62
13	rs4811196	chr20	36469694	Radiographic parameters	Bone mineral density-trochanter	237.62	0.00	-1.54
14	rs4996815	chr13	106651661	Neurological behavioral	Biplolar disorder and schizophrenia	368.38	51.52	-1.48
15	rs2086512	chr6	99011501	Viral disease	Cytomegalovirus antibody response	19.44	278.69	1.48
16	rs6495122	chr15	75125645	Drug metabolism	Caffeine intake	406.50	67.01	-1.47
17	rs6495122	chr15	75125645	Drug metabolism	coffee consumption	406.50	67.01	-1.47

18	rs6495122	chr15	75125645	Cardiovascular	Diastolic blood pressure	406.50	67.01	-1.47
19	rs11013962	chr10	24495586	Quantitative traits	Earlobes	0.00	202.33	1.39
20	rs1458175	chr12	41965861	Autoimmune disease	Multiple sclerosis	441.33	104.68	-1.30
21	rs536841	chr11	85787824	Aging	Alzheimers disease	181.16	0.00	-1.29
22	rs4129267	chr1	154426264	Autoimmune disease	Asthma	290.64	46.67	-1.28
23	rs4129267	chr1	154426264	Serum metabolites	C-reactive protein	290.64	46.67	-1.28
24	rs4129267	chr1	154426264	Kidney lung liver	FEF	290.64	46.67	-1.28
25	rs4129267	chr1	154426264	Serum metabolites	IL6R	290.64	46.67	-1.28
26	rs6720394	chr2	111989372	Autoimmune disease	Primary sclerosing cholangitis	47.50	290.34	1.27
27	rs12143842	chr1	162033890	Cardiovascular	QT interval	157.39	0.00	-1.18
28	rs1497406	chr1	16505320	Serum metabolites	Gamma glutamyl transferase	177.29	8.92	-1.17
29	rs558718	chr19	7909883	Viral disease	HIV progression	152.38	0.00	-1.15
30	rs7315438	chr12	115891403	Cancer	Colorectal cancer	0.00	142.54	1.10
31	rs12916	chr5	74656539	Serum metabolites	LDL cholesterol	91.00	321.58	1.05
32	rs7567389	chr2	127982645	Miscellaneous	Self-rated health	212.04	40.23	-1.03

82 NOR1 Round:3

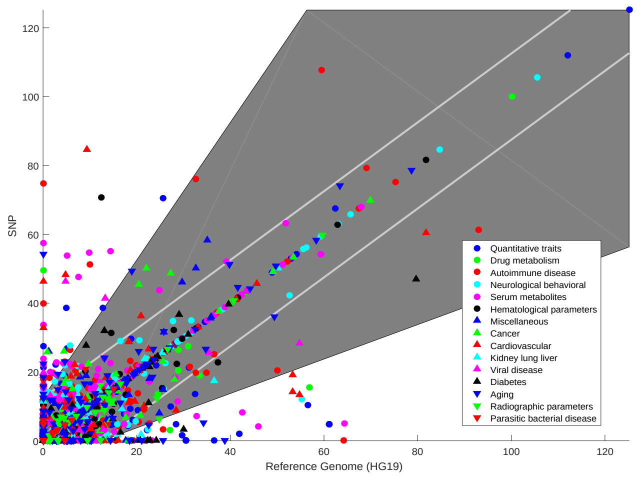

S.No.	SNP	Chr.	Position	Disease Class	Disease Trait	HG19	SNP	$log_2(\frac{SNP+\eta}{HG19+\eta})$
1	rs8007846	chr14	66262963	Autoimmune disease	Multiple sclerosis-Brain Glutamate Concentrations	0.05	74.82	2.80
2	rs7765379	chr6	32680928	Autoimmune disease	Rheumatoid arthritis	64.19	0.00	-2.62
3	rs1539019	chr1	247600301	Serum metabolites	Fibrinogen	0.00	57.50	2.48
4	rs7153703	chr14	51919822	Aging	Alzheimers Total ventricular volume	0.00	54.14	2.41
5	rs10458561	chr1	70921173	Drug metabolism	Risperidone influence on QT interval	0.00	49.49	2.31
6	rs2782980	chr10	115781527	Cardiovascular	Blood pressure	0.00	46.29	2.23
7	rs12946454	chr17	43208121	Cardiovascular	Systolic blood pressure	9.42	84.62	2.15
8	rs675209	chr6	7102084	Serum metabolites	Serum urate	64.36	5.04	-2.13
9	rs7138803	chr12	50247468	Quantitative traits	BMI	61.10	4.82	-2.09
10	rs7138803	chr12	50247468	Quantitative traits	Waist circumference	61.10	4.82	-2.09
11	rs7138803	chr12	50247468	Quantitative traits	Weight	61.10	4.82	-2.09
12	rs8049439	chr16	28837515	Autoimmune disease	Inflammatory bowel disease-early onset	0.00	39.88	2.07
13	rs3772255	chr3	156102734	Aging	Aging traits-biologic age	38.85	0.00	-2.04
14	rs5757949	chr22	40820151	Quantitative traits	Height	36.51	0.00	-1.97
15	rs589691	chr11	64525216	Serum metabolites	Serum urate	5.12	53.73	1.91
16	rs571312	chr18	57839769	Quantitative traits	BMI	41.99	2.07	-1.90
17	rs2153960	chr6	108988184	Serum metabolites	IGF-1	0.00	33.66	1.88
18	rs4528684	chr19	14351574	Cardiovascular	Heart failure motality-EA	0.00	32.92	1.86

19	rs1497406	chr1	16505320	Serum metabolites	Gamma glutamyl transferase	46.04	4.14	-1.81
20	rs10777317	chr12	91980374	Cardiovascular	Sudden cardiac arrest	4.85	48.24	1.81
21	rs12913832	chr15	28365618	Quantitative traits	Eye color	30.46	0.00	-1.78
22	rs12913832	chr15	28365618	Quantitative traits	Eye color-green eyes	30.46	0.00	-1.78
23	rs12913832	chr15	28365618	Quantitative traits	Hair color	30.46	0.00	-1.78
24	rs12913832	chr15	28365618	Quantitative traits	Hair color-Black vs. blond hair color	30.46	0.00	-1.78
25	rs12913832	chr15	28365618	Quantitative traits	Hair color-Black vs. red hair color	30.46	0.00	-1.78
26	rs6139030	chr20	3187733	Viral disease	Response to hepatitis C treatment	4.87	46.34	1.76
27	rs11085824	chr19	13001547	Hematological parameters	Mean corpuscular hemoglobin	12.50	70.71	1.73
28	rs1436900	chr1	217058479	Quantitative traits	Optic disc size cup	0.00	27.54	1.68
29	rs1223271	chr20	13296912	Aging	Parkinsons disease	25.57	0.00	-1.61
30	rs2815752	chr1	72812440	Quantitative traits	BMI	56.62	10.43	-1.59
31	rs11823543	chr11	116649135	Serum metabolites	Triglycerides blood pressure	9.85	54.65	1.59
32	rs10506821	chr12	80496923	Quantitative traits	Hip geometry	29.66	1.54	-1.58
33	rs12459897	chr19	31596778	Serum metabolites	Serum polyunsaturated fatty acids	7.57	47.69	1.58
34	rs1531343	chr12	66174894	Diabetes	Type 2 diabetes	24.15	0.00	-1.55
35	rs3791675	chr2	56111309	Quantitative traits	Height	5.04	38.69	1.54
36	rs515135	chr2	21286057	Serum metabolites	LDL cholesterol	0.00	23.82	1.54
37	rs3790268	chr20	19571581	Serum metabolites	Serum matrix metalloproteinase	23.19	0.00	-1.51
38	rs7931342	chr11	68994497	Cancer	Prostate cancer	0.94	25.74	1.51
39	rs4704970	chr5	155500992	Autoimmune disease	Multiple sclerosis	9.99	51.31	1.50
40	rs10460009	chr18	2948029	Diabetes	Type 2 diabetes	22.82	0.00	-1.50
41	rs681900	chr2	75074967	Miscellaneous	Femoral neck bone geometry	1.19	25.95	1.49
42	rs8066857	chr17	70696103	Aging	Amyotrophic lateral sclerosis-age of onset	0.00	22.46	1.48
43	rs16861329	chr3	186666461	Diabetes	Type 2 diabetes	22.31	0.00	-1.48
44	rs1354492	chr17	31737421	Serum metabolites	Van Wildebrand factor antibodies	0.00	22.18	1.47
45	rs157350	chr5	156139569	Quantitative traits	Brachial circumference	22.02	0.00	-1.46
46	rs157350	chr5	156139569	Quantitative traits	Hip circumference	22.02	0.00	-1.46

47	rs12579350	chr12	5797101	Neurological behavioral	Panic disorder	55.18	12.13	-1.46
48	rs10181042	chr2	61224259	Autoimmune disease	Crohns disease	21.69	0.00	-1.45
49	rs988712	chr11	27563382	Quantitative traits	BMI	0.00	21.30	1.43
50	rs5753037	chr22	30581722	Diabetes	Type 1 diabetes	29.97	3.23	-1.43
51	rs4140564	chr1	186725003	Aging	Knee osteoarthritis	21.14	0.00	-1.43
52	rs7754840	chr6	20661250	Diabetes	Type 2 diabetes	20.83	0.00	-1.41
53	rs4963452	chr11	61815803	Serum metabolites	Serum polyunsaturated fatty acids	42.63	8.30	-1.41
54	rs2061333	chr19	44614208	Aging	Alzheimers disease	34.32	5.16	-1.41
55	rs10917468	chr1	19843354	Miscellaneous	Goiter	20.23	0.00	-1.39
56	rs2527866	chr7	157090296	Cardiovascular	Systolic blood pressure	54.85	13.31	-1.38
57	rs17793829	chr14	91022185	Viral disease	Cytomegalovirus antibody response	0.00	19.82	1.37
58	rs1459148	chr14	98840443	Drug metabolism	Response to antipsychotic therapy extrapyramidal side effects	27.07	3.03	-1.35
59	rs3130573	chr6	31106268	Autoimmune disease	Systemic sclerosis	19.33	0.00	-1.35
60	rs1880887	chr12	41721430	Serum metabolites	Alkaline phosphatase	14.43	55.13	1.33
61	rs2251393	chr17	60778932	Cardiovascular	Sudden cardiac arrest	0.00	18.88	1.33
62	rs17396340	chr1	10286176	Hematological parameters	Mean platelet volume	18.77	0.00	-1.32
63	rs12203592	chr6	396321	Quantitative traits	Freckling	0.00	18.57	1.31
64	rs12203592	chr6	396321	Quantitative traits	Hair color	0.00	18.57	1.31
65	rs12203592	chr6	396321	Quantitative traits	Hair color-Black vs. blond hair color	0.00	18.57	1.31
66	rs12203592	chr6	396321	Quantitative traits	Hair color-Black vs. red hair color	0.00	18.57	1.31
67	rs12203592	chr6	396321	Aging	Progressive supranuclear palsy	0.00	18.57	1.31
68	rs5998432	chr22	32745916	Aging	Alzheimers T-tau	18.52	0.00	-1.31
69	rs2954038	chr8	126507389	Drug metabolism	Response to statin therapy-Triglyceride sum	56.95	15.50	-1.31
70	rs12046278	chr1	10799577	Cardiovascular	Systolic blood pressure	53.23	14.24	-1.30
71	rs6736997	chr2	235615197	Cancer	Pancreatic cancer	18.16	0.00	-1.29
72	rs11989122	chr8	118827839	Quantitative traits	Height	18.03	0.00	-1.29

73	rs1837253	chr5	110401872	Autoimmune disease	Asthma	0.00	17.79	1.28
74	rs7064929	chrX	64367019	Miscellaneous	Erectile dysfunction and prostate cancer treatment	17.70	0.00	-1.27
75	rs2300747	chr1	117104215	Autoimmune disease	Multiple sclerosis	25.60	3.35	-1.26
76	rs1424233	chr16	79682751	Quantitative traits	Obesity	28.34	4.79	-1.24
77	rs9314986	chr13	30458737	Kidney lung liver	Biliary atresia	20.98	1.79	-1.23
78	rs626277	chr13	72347696	Serum metabolites	Creatinine	1.47	19.95	1.22
79	rs2008242	chr4	5221538	Cardiovascular	PR segment	0.00	16.47	1.21
80	rs6474359	chr8	41549194	Serum metabolites	HbA1C	0.00	16.43	1.21
81	rs1514178	chr1	61205469	Serum metabolites	Serum polyunsaturated fatty acids	32.75	7.16	-1.20
82	rs17267292	chr13	93323146	Serum metabolites	Docosahexaonic acid	2.81	22.72	1.20
83	rs11624704	chr14	78786077	Quantitative traits	Waist-hip ratio	2.66	22.18	1.19
84	rs2207418	chr20	11174903	Cardiovascular	Cardiac hypertrophy	16.00	0.00	-1.19
85	rs6556756	chr5	163889280	Cancer	Breast cancer	4.51	26.03	1.18
86	rs757647	chr5	137707315	Aging	Age at menarche	0.00	15.77	1.18
87	rs10956483	chr8	130572110	Hematological parameters	Monocyte count	15.56	0.00	-1.17
88	rs17145713	chr7	72904810	Serum metabolites	Protein C	0.00	15.47	1.16
89	rs3791950	chr2	218729865	Quantitative traits	Height	5.13	26.88	1.16
90	rs2381416	chr9	6193455	Autoimmune disease	Asthma	2.00	19.77	1.15
91	rs12654264	chr5	74648603	Serum metabolites	LDL cholesterol	0.00	15.22	1.15
92	rs6651252	chr8	129567181	Autoimmune disease	Crohns disease	1.43	18.33	1.15
93	rs1816002	chr10	72483010	Quantitative traits	Weight	22.17	3.18	-1.14
94	rs2088885	chr3	170971291	Neurological behavioral	Brain imaging in schizophrenia interaction	22.35	3.26	-1.14
95	rs1329650	chr10	93348120	Neurological behavioral	Smoking behavior	5.73	27.63	1.14
96	rs2114039	chr4	55092626	Quantitative traits	Corneal curvature	25.69	70.43	1.12
97	rs7601713	chr2	157552860	Cardiovascular	QT interval	14.63	0.00	-1.12
98	rs448720	chr15	68198911	Neurological behavioral	Trails B	0.00	14.46	1.11
99	rs13095226	chr3	99396272	Aging	Age-related macular degeneration	14.46	0.00	-1.11
100	rs9271366	chr6	32586854	Autoimmune disease	Inflammatory bowel disease	5.77	26.44	1.09

101	rs9325032	chr5	146415216	Neurological behavioral	Cognitive performance-F2	13.84	0.00	-1.07
102	rs6719977	chr2	42985803	Neurological behavioral	ADHD-Hyperactive-impulsive symptoms	13.84	0.00	-1.07
103	rs2738113	chr8	6829085	Miscellaneous	Endometriosis	13.83	0.00	-1.07
104	rs994014	chr4	82165790	Quantitative traits	Height	13.79	0.00	-1.07
105	rs2032794	chr5	86432617	Neurological behavioral	Personality dimensions-openness	0.00	13.72	1.07
106	rs8099917	chr19	39743165	Viral disease	Chronic Hepatitis C infection	13.28	41.50	1.07
107	rs8099917	chr19	39743165	Viral disease	Response to hepatitis C treatment	13.28	41.50	1.07
108	rs1925690	chr6	87867063	Aging	Alzheimers Entorhinal cortical thickness	0.00	13.58	1.06
109	rs17021918	chr4	95562877	Cancer	Prostate cancer	13.54	0.00	-1.06
110	rs11709625	chr3	66823157	Serum metabolites	Urea nitrogen-blood	4.36	22.54	1.05
111	rs1395821	chr4	148047550	Cardiovascular	Coronary heart disease	53.33	19.21	-1.05
112	rs4815617	chr20	3827309	Autoimmune disease	Asthma	4.68	23.07	1.05
113	rs4815617	chr20	3827309	Aging	Longevity	4.68	23.07	1.05
114	rs2967605	chr19	8469738	Serum metabolites	HDL cholesterol	2.78	19.08	1.05
115	rs10983238	chr9	119333683	Neurological behavioral	ADHD	15.81	1.31	-1.03
116	rs3764650	chr19	1046520	Aging	Alzheimers disease	22.53	4.63	-1.03
117	rs7784776	chr7	46620145	Cardiovascular	QRS duration	12.94	0.00	-1.02
118	rs6834555	chr4	10062326	Aging	Alzheimers disease	3.18	19.33	1.02
119	rs9319321	chr13	27415673	Autoimmune disease	Asthma	12.81	0.00	-1.02
120	rs634552	chr11	75282052	Quantitative traits	Height	12.75	38.60	1.02
121	rs10488172	chr7	133335176	Cardiovascular	Tonometry	12.79	0.00	-1.02
122	rs17184557	chr18	67142857	Aging	Osteoporosis	0.00	12.57	1.00
123	rs4332037	chr7	1950809	Neurological behavioral	Bipolar disorder	4.62	21.83	1.00
124	rs10975003	chr9	5213687	Autoimmune disease	Ulcerative colitis	22.66	5.07	-1.00

83 NURR1 Round:3

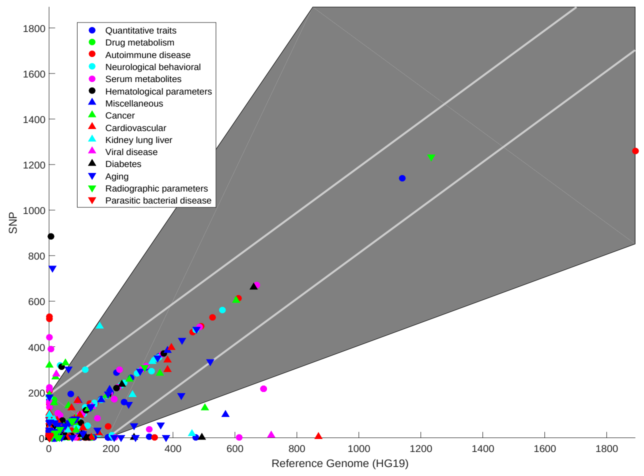

S.No.	SNP	Chr.	Position	Disease Class	Disease Trait	HG19	SNP	$log_2(\frac{SNP+\eta}{HG19+\eta})$
1	rs11085824	chr19	13001547	Hematological parameters	Mean corpuscular hemoglobin	6.73	884.49	2.45
2	rs12046278	chr1	10799577	Cardiovascular	Systolic blood pressure	869.59	5.59	-2.44
3	rs11761231	chr7	131370039	Autoimmune disease	Rheumatoid arthritis	146.72	1446.55	2.28
4	rs823156	chr1	205764640	Aging	Parkinsons disease	11.67	746.76	2.22
5	rs10789491	chr1	47179310	Viral disease	Response to hepatitis C treatment	717.20	11.83	-2.17
6	rs1497406	chr1	16505320	Serum metabolites	Gamma glutamyl transferase	614.56	0.00	-2.09
7	rs8007846	chr14	66262963	Autoimmune disease	Multiple sclerosis-Brain Glutamate Concentrations	0.00	531.85	1.93
8	rs8049439	chr16	28837515	Autoimmune disease	Inflammatory bowel disease-early onset	0.00	522.55	1.91
9	rs7754840	chr6	20661250	Diabetes	Type 2 diabetes	494.15	0.00	-1.85
10	rs1424233	chr16	79682751	Quantitative traits	Obesity	472.84	0.00	-1.81
11	rs11823543	chr11	116649135	Serum metabolites	Triglycerides blood pressure	0.00	441.81	1.74
12	rs9292394	chr5	30661573	Kidney lung liver	COPD	460.74	16.57	-1.66
13	rs3764650	chr19	1046520	Aging	Alzheimers disease	377.52	0.00	-1.58
14	rs1880887	chr12	41721430	Serum metabolites	Alkaline phosphatase	5.44	390.33	1.57
15	rs6554809	chr5	13740976	Autoimmune disease	IgE grass sensitization	341.50	0.00	-1.49
16	rs10411161	chr19	52372976	Cancer	Breast cancer	1.03	318.06	1.42
17	rs9470004	chr6	35341850	Quantitative traits	Height	324.55	5.06	-1.40
18	rs7567389	chr2	127982645	Miscellaneous	Self-rated health	569.98	100.76	-1.39
19	rs10517086	chr4	26085511	Diabetes	Type 1 diabetes	311.10	1066.95	1.33
20	rs3772255	chr3	156102734	Aging	Aging traits-biologic age	279.68	0.00	-1.31
21	rs5753037	chr22	30581722	Diabetes	Type 1 diabetes	275.26	0.00	-1.30

22	rs12686004	chr9	107653426	Serum metabolites	HDL cholesterol	322.83	36.28	-1.18
23	rs9804317	chr10	130248926	Neurological behavioral	Animals	36.25	319.09	1.17
24	rs1713985	chr4	57786450	Aging	Age-related macular degeneration	360.55	55.25	-1.17
25	rs5998432	chr22	32745916	Aging	Alzheimers T-tau	232.88	0.00	-1.16
26	rs6139030	chr20	3187733	Viral disease	Response to hepatitis C treatment	23.26	279.21	1.14
27	rs10889353	chr1	63118196	Serum metabolites	Cholesterol	693.19	213.72	-1.13
28	rs10889353	chr1	63118196	Serum metabolites	LDL cholesterol	693.19	213.72	-1.13
29	rs10889353	chr1	63118196	Serum metabolites	Triglycerides	693.19	213.72	-1.13
30	rs7315438	chr12	115891403	Cancer	Colorectal cancer	20.29	266.98	1.12
31	rs2893923	chr10	65261184	Hematological parameters	Platelet aggregation-epinephrine	40.82	311.56	1.12
32	rs12459897	chr19	31596778	Serum metabolites	Serum polyunsaturated fatty acids	0.00	221.96	1.12
33	rs1859962	chr17	69108753	Cancer	Prostate cancer	503.23	131.36	-1.11
34	rs7758229	chr6	160840252	Cancer	Colorectal cancer	51.73	329.81	1.11
35	rs3790268	chr20	19571581	Serum metabolites	Serum matrix metalloproteinase	212.56	0.00	-1.09
36	rs1539019	chr1	247600301	Serum metabolites	Fibrinogen	0.00	211.01	1.08
37	rs1223271	chr20	13296912	Aging	Parkinsons disease	211.00	0.00	-1.08
38	rs6719977	chr2	42985803	Neurological behavioral	ADHD-Hyperactive-impulsive symptoms	208.13	0.00	-1.07
39	rs4704970	chr5	155500992	Autoimmune disease	Multiple sclerosis	0.00	204.04	1.06
40	rs157350	chr5	156139569	Quantitative traits	Brachial circumference	200.23	0.00	-1.04
41	rs157350	chr5	156139569	Quantitative traits	Hip circumference	200.23	0.00	-1.04
42	rs12913832	chr15	28365618	Quantitative traits	Eye color	191.23	0.00	-1.01
43	rs12913832	chr15	28365618	Quantitative traits	Eye color-green eyes	191.23	0.00	-1.01
44	rs12913832	chr15	28365618	Quantitative traits	Hair color	191.23	0.00	-1.01
45	rs12913832	chr15	28365618	Quantitative traits	Hair color-Black vs. blond hair color	191.23	0.00	-1.01
46	rs12913832	chr15	28365618	Quantitative traits	Hair color-Black vs. red hair color	191.23	0.00	-1.01
47	rs7931342	chr11	68994497	Cancer	Prostate cancer	0.00	189.83	1.00
