# Supplementary material for: Hidden modes of DNA binding by human nuclear receptors
Source: Nat Commun. 2023 Jul 13;14:4179. doi: 10.1038/s41467-023-39577-0 (PMC10345098; doi:10.1038/s41467-023-39577-0)
Supplement: Supplementary file 15 — Reporting Summary [file 41467_2023_39577_MOESM15_ESM.pdf]

Corresponding author(s): Aseem Z. Ansari
Parameswaran Ramanathan
Devesh Bhimsaria

Last updated by author(s): Jun 8, 2023

Reporting Summary

Nature Portfolio wishes to improve the reproducibility of the work that we publish. This form provides structure for consistency and transparency in reporting. For further information on Nature Portfolio policies, see our Editorial Policies and the Editorial Policy Checklist.

Statistics

For all statistical analyses, confirm that the following items are present in the figure legend, table legend, main text, or Methods section.

n/a	Confirmed	
☒	☐	The exact sample size (*n*) for each experimental group/condition, given as a discrete number and unit of measurement
☒	☐	A statement on whether measurements were taken from distinct samples or whether the same sample was measured repeatedly
☒	☐	The statistical test(s) used AND whether they are one- or two-sided<br>*Only common tests should be described solely by name; describe more complex techniques in the Methods section.*
☒	☐	A description of all covariates tested
☒	☐	A description of any assumptions or corrections, such as tests of normality and adjustment for multiple comparisons
☒	☐	A full description of the statistical parameters including central tendency (e.g. means) or other basic estimates (e.g. regression coefficient) AND variation (e.g. standard deviation) or associated estimates of uncertainty (e.g. confidence intervals)
☒	☐	For null hypothesis testing, the test statistic (e.g. *F*, *t*, *r*) with confidence intervals, effect sizes, degrees of freedom and *P* value noted<br>*Give P values as exact values whenever suitable.*
☒	☐	For Bayesian analysis, information on the choice of priors and Markov chain Monte Carlo settings
☒	☐	For hierarchical and complex designs, identification of the appropriate level for tests and full reporting of outcomes
☐	☒	Estimates of effect sizes (e.g. Cohen's *d*, Pearson's *r*), indicating how they were calculated

Our web collection on statistics for biologists contains articles on many of the points above.

Software and code

Policy information about availability of computer code

Data collection	No software was used for data collection.
Data analysis	Custom code with instructions to run MinSeq Find algorithm is available at GitHub: https://github.com/dev11ume/MinSeq-Find

For manuscripts utilizing custom algorithms or software that are central to the research but not yet described in published literature, software must be made available to editors and reviewers. We strongly encourage code deposition in a community repository (e.g. GitHub). See the Nature Portfolio guidelines for submitting code & software for further information.

Data

Policy information about availability of data

All manuscripts must include a data availability statement. This statement should provide the following information, where applicable:

- Accession codes, unique identifiers, or web links for publicly available datasets
- A description of any restrictions on data availability
- For clinical datasets or third party data, please ensure that the statement adheres to our policy

The Sequencing data generated in this study are available in the National Center for Biotechnology Information (NCBI) database under BioProject PRJNA729962. The HT-SELEX data used in this study from Jolma et al. 2013 and Yin et al. 2017 are available in the European Nucleotide Archive (ENA) under accession code ERP001824, ERP001826, and PRJEB9797. The SelexGLM data used in this study are available in the Sequence Read Archive (SRA) under BioProject PRJNA379022. The SMiLE-seq data used in this study are available in the Sequence Read Archive (SRA) under BioProject PRJNA318578. The SNP data used in this study are from

Maurano et al. 2012 Supplementary Table S2, the NCBI-supported ClinVar database and NHGRI-supported GWAS Catalog. LoVo cell line ChIP-seq peak data used in this study are available in the NCBI database under GEO accession code GSE49402. ENCODE ChIP-seq peak data used in this study are available on the UCSC ftp server (ftp://hgdownload.cse.ucsc.edu/goldenPath/hg19/encodeDCC/wgEncodeAwgTfbsUniform). AR and GR ChIP Exo peak data from U2OS cell line used in this study are available in the European Bioinformatics Institute (EBI) data base under accession code E-MTAB-9616. RXRA ChIP-seq peak data for the HEPG2 and H1-hESCs cell lines used in this study are available in the CistromeDB under id 46242 and 46192. The crystal structure data used in this study are available in the Protein Data Bank (PDB) database under accession codes 3g6q and 3g9i. Source data are provided with this paper.

Human research participants

Policy information about studies involving human research participants and Sex and Gender in Research.

Reporting on sex and gender	Not applicable
Population characteristics	Not applicable
Recruitment	Not applicable
Ethics oversight	Not applicable

Note that full information on the approval of the study protocol must also be provided in the manuscript.

Field-specific reporting

Please select the one below that is the best fit for your research. If you are not sure, read the appropriate sections before making your selection.

☒ Life sciences ☐ Behavioural & social sciences ☐ Ecological, evolutionary & environmental sciences

For a reference copy of the document with all sections, see nature.com/documents/nr-reporting-summary-flat.pdf

Life sciences study design

All studies must disclose on these points even when the disclosure is negative.

Sample size	All 48 known human nuclear receptors were attempted, 45 expressed and 38 provided high resolution DNA Protein Interactome datasets.
Data exclusions	No data excluded.
Replication	Out of 214 samples, 70 samples didn't work. We compared 2 direct replicates of HNF4G and NURR1, which were successful.
Randomization	Randomization was not relevant to this study because we were mapping binding sites for all expressed NRs amongst a fully randomized library of 10e12 (Trillion) 20-mer sequences. Parallel sequencing of the original DNA library as well as the absence of any detectable binding preference for DAX, a NR member that does not bind DNA serve as two independent controls for any systemic bias in our binding data for the 83 NR datasets that we report in this manuscript.
Blinding	Blinding was not relevant for this study as the majority of the NRs interactomes provided here have not been previously been reported in the literature, as shown in Fig. 2b. Moreover, we identified unexpected binding modes of several well-studied NRs in our data as well as publicly available data (as highlighted in Fig. 2 and rest of the figures). In essence, blinding would not have altered the reported outcomes or altered the underlying data for subsequent analysis by others.

Reporting for specific materials, systems and methods

We require information from authors about some types of materials, experimental systems and methods used in many studies. Here, indicate whether each material, system or method listed is relevant to your study. If you are not sure if a list item applies to your research, read the appropriate section before selecting a response.

Materials & experimental systems

n/a	Involved in the study
☒	☐ Antibodies
☐	☒ Eukaryotic cell lines
☒	☐ Palaeontology and archaeology
☒	☐ Animals and other organisms
☒	☐ Clinical data
☒	☐ Dual use research of concern

Methods

n/a	Involved in the study
☒	☐ ChIP-seq
☒	☐ Flow cytometry
☒	☐ MRI-based neuroimaging

Eukaryotic cell lines

Policy information about cell lines and Sex and Gender in Research

Cell line source(s)	HEK293T cells to express and purify Halo-tagged NRs.
Authentication	None
Mycoplasma contamination	Not tested
Commonly misidentified lines (See ICLAC register)	No commonly misidentified cell lines were used by us or included in our analysis of data from the literature.
