# Supplementary material for: Hidden modes of DNA binding by human nuclear receptors
Source: Nat Commun. 2023 Jul 13;14:4179. doi: 10.1038/s41467-023-39577-0 (PMC10345098; doi:10.1038/s41467-023-39577-0)
Supplement: Supplementary file 4 — Supplementary Data 2 [file 41467_2023_39577_MOESM4_ESM.pdf]

Supplementary Data 2: PWM logos for all the factors

Contents

1 AR-Jolma2013-Rnd2 (ERR193971) 16

2 Ar-Jolma2013-Rnd2 (ERR194680) 19

3 AR-Jolma2013-Rnd3 (ERR193680) 21

4 AR-Jolma2013-Rnd3 (ERR193972) 23

5 Ar-Jolma2013-Rnd3 (ERR194681) 25

6 AR-Jolma2013-Rnd4 (ERR193681) 27

7 AR-SelexGLM-Rnd2 (SRR5340731) 29

8 AR-SelexGLM-Rnd3 (SRR5340730) 32

9 AR-SelexGLM-Rnd4 (SRR5340729) 35

10 AR-SelexGLM-Rnd5 (SRR5340728) 38

11 AR-SelexGLM-Rnd6 (SRR5340727) 41

12 AR-SelexGLM-Rnd7 (SRR5340726) 44

13 AR-SelexGLM-Rnd8 (SRR5340725) 47

14 MR-1-Bhimsaria2022-Rnd2 (S07-005) 50

15 MR-1-Bhimsaria2022-Rnd3 (S07-126) 53

16 MR-Bhimsaria2022-Rnd2 (S07-046) 54

17 MR-Bhimsaria2022-Rnd3 (S07-167) 57

18 MR-Jolma2013-Rnd2 (ERR188787) 58

19 MR-Jolma2013-Rnd3 (ERR188788) 61

20 MR-Jolma2013-Rnd4 (ERR188789) 63

21 MR-Yin2017-Rnd4 (ERR1005249) 66

22 PGR-Bhimsaria2022-Rnd2 (S07-047) 68

23 PGR-Bhimsaria2022-Rnd3 (S07-168) 71

24 PGR-2-Bhimsaria2022-Rnd2 (S07-006) 72

25 PGR-2-Bhimsaria2022-Rnd3 (S07-127) 75

26 GR-Bhimsaria2022-Rnd2 (S07-045) 76

27 GR-Bhimsaria2022-Rnd3 (S07-166) 79

28 GR-Jolma2013-Rnd2 (ERR194205) 80

29 **GR-Jolma2013-Rnd3 (ERR194206)** **83**

30 **GR-Jolma2013-Rnd4 (ERR194207)** **85**

31 **GR-3-Bhimsaria2022-Rnd3 (S07-125)** **87**

32 **GR-Yin2017-Rnd2 (ERR1005236)** **88**

33 **GR-Yin2017-Rnd3 (ERR1005238)** **91**

34 **GR-Yin2017-Rnd3 (ERR1005239)** **94**

35 **GR-Yin2017-Rnd4 (ERR1005240)** **97**

36 **GR-Yin2017-Rnd4 (ERR1005241)** **100**

37 **GR-SelexGLM-Rnd3 (SRR5340721)** **103**

38 **GR-SelexGLM-Rnd4 (SRR5340720)** **106**

39 **GR-SelexGLM-Rnd5 (SRR5340719)** **109**

40 **GR-SelexGLM-Rnd6 (SRR5340718)** **112**

41 **GR-SelexGLM-Rnd7 (SRR5340717)** **115**

42 **GR-SelexGLM-Rnd8 (SRR5340716)** **118**

43 **ESRRA:RXRA-Bhimsaria2022-Rnd3 (S13-136)** **121**

44 **ESRRA-Bhimsaria2022-Rnd2 (S07-037)** **124**

45 **ESRRA-Bhimsaria2022-Rnd3 (S07-157)** **127**

46 **ESRRA-Jolma2013-Rnd2 (ERR195578)** **129**

47 **Esrra-Jolma2013-Rnd3 (ERR188728)** **132**

48 **ESRRA-Jolma2013-Rnd3 (ERR194308)** **135**

49 **ESRRA-Jolma2013-Rnd3 (ERR195579)** **138**

50 **Esrra-Jolma2013-Rnd4 (ERR188729)** **141**

51 **ESRRA-Jolma2013-Rnd4 (ERR194309)** **144**

52 **ESRRA-Jolma2013-Rnd4 (ERR195580)** **147**

53 **ESRRA-Jolma2013-Rnd5 (ERR194310)** **150**

54 **ESRRA-Jolma2013-Rnd6 (ERR194311)** **153**

55 **ESRRA-4-Bhimsaria2022-Rnd2 (S07-001)** **156**

56 **ESRRA-4-Bhimsaria2022-Rnd3 (S07-122)** **159**

57 **ESRRA-Yin2017-Rnd2 (ERR1003021)** **161**

58 **ESRRA-Yin2017-Rnd3 (ERR1003022)** **164**

59 **ESRRA-Yin2017-Rnd3 (ERR1003023)** **167**

60 **ESRRA-Yin2017-Rnd4 (ERR1003024)** **170**

61 **ESRRA-Yin2017-Rnd4 (ERR1003025)** **173**

62 **ESRRA-Yin2017-Rnd2 (ERR1003028)** **176**

63 **ESRRA-Yin2017-Rnd2 (ERR1003029)** **179**

64 **ESRRA-Yin2017-Rnd3 (ERR1003030)** **182**

65 **ESRRA-Yin2017-Rnd3 (ERR1003031)** **185**

66 **ESRRA-Yin2017-Rnd4 (ERR1003032)** **188**

67 **ESRRA-Yin2017-Rnd4 (ERR1003033)** **191**

68 **ESRRB:RXRA-Bhimsaria2022-Rnd3 (S13-137)** **194**

69 **ESRRB-Bhimsaria2022-Rnd2 (S07-038)** **197**

70 **ESRRB-Bhimsaria2022-Rnd3 (S07-158)** **200**

71 **ESRRB-Jolma2013-Rnd2 (ERR194874)** **203**

72 **ESRRB-Jolma2013-Rnd3 (ERR194875)** **206**

73 **ESRRB-Jolma2013-Rnd4 (ERR194876)** **209**

74 **ESRRB-4-Bhimsaria2022-Rnd2 (S07-002)** **212**

75 **ESRRB-4-Bhimsaria2022-Rnd3 (S07-123)** **215**

76 **ESRRB-Yin2017-Rnd2 (ERR1003036)** **218**

77 **ESRRB-Yin2017-Rnd2 (ERR1003037)** **221**

78 **ESRRB-Yin2017-Rnd3 (ERR1003038)** **224**

79 **ESRRB-Yin2017-Rnd3 (ERR1003039)** **227**

80 **ESRRB-Yin2017-Rnd4 (ERR1003040)** **230**

81 **ESRRB-Yin2017-Rnd4 (ERR1003041)** **233**

82 **ESRRG:RXRA-Bhimsaria2022-Rnd3 (S13-155)** **236**

83 **ESRRG-Bhimsaria2022-Rnd2 (S07-039)** **239**

84 **ESRRG-Bhimsaria2022-Rnd3 (S07-159)** **242**

85 **ESRRG-Jolma2013-Rnd3 (ERR193996)** **245**

86 **ESRRG-Jolma2013-Rnd4 (ERR193997)** **248**

**87**	**ESRRG-4-Bhimsaria2022-Rnd2 (S07-003)**	**251**
**88**	**ESRRG-4-Bhimsaria2022-Rnd3 (S07-124)**	**254**
**89**	**ESRRG-Yin2017-Rnd3 (ERR1003046)**	**256**
**90**	**ESRRG-Yin2017-Rnd3 (ERR1003047)**	**259**
**91**	**ESRRG-Yin2017-Rnd4 (ERR1003048)**	**262**
**92**	**ESRRG-Yin2017-Rnd4 (ERR1003049)**	**265**
**93**	**ESRRG-Yin2017-Rnd2 (ERR1003052)**	**268**
**94**	**ESRRG-Yin2017-Rnd3 (ERR1003054)**	**271**
**95**	**ESRRG-Yin2017-Rnd4 (ERR1003056)**	**274**
**96**	**ESR1-5-Bhimsaria2022-Rnd2 (S04-142)**	**277**
**97**	**ESR1-5-Bhimsaria2022-Rnd3 (S04-143)**	**280**
**98**	**ESR1-Jolma2013-Rnd3 (ERR194971)**	**283**
**99**	**ESR1-Jolma2013-Rnd4 (ERR194972)**	**286**
**100**	**ESR1-Yin2017-Rnd2 (ERR1003004)**	**287**
**101**	**ESR1-Yin2017-Rnd2 (ERR1003005)**	**290**
**102**	**ESR1-Yin2017-Rnd3 (ERR1003006)**	**293**
**103**	**ESR1-Yin2017-Rnd3 (ERR1003007)**	**296**
**104**	**ESR1-Yin2017-Rnd4 (ERR1003008)**	**298**
**105**	**ESR1-Yin2017-Rnd4 (ERR1003009)**	**300**
**106**	**ESR1-Yin2017-Rnd2 (ERR1003012)**	**303**
**107**	**ESR1-Yin2017-Rnd2 (ERR1003013)**	**306**
**108**	**ESR1-Yin2017-Rnd3 (ERR1003014)**	**309**
**109**	**ESR1-Yin2017-Rnd3 (ERR1003015)**	**312**
**110**	**ESR1-Yin2017-Rnd4 (ERR1003016)**	**315**
**111**	**ESR1-Yin2017-Rnd4 (ERR1003017)**	**318**
**112**	**THRA:RXRA-Bhimsaria2022-Rnd3 (S13-163)**	**321**
**113**	**THRA:RXRA-7-Bhimsaria2022-Rnd2 (S13-124)**	**324**
**114**	**THRA-Bhimsaria2022-Rnd2 (S07-034)**	**327**
**115**	**THRA-Bhimsaria2022-Rnd3 (S07-154)**	**330**

116 **THRA-Jolma2013-Rnd2 (ERR194565)** **333**

117 **THRA-Jolma2013-Rnd3 (ERR194566)** **336**

118 **THRA-Jolma2013-Rnd4 (ERR194567)** **338**

119 **THRA-Jolma2013-Rnd5 (ERR194568)** **339**

120 **THRA-7-Bhimsaria2022-Rnd3 (S07-102)** **341**

121 **THRB:RXRA-Bhimsaria2022-Rnd3 (S13-164)** **344**

122 **THRB:RXRA-7-Bhimsaria2022-Rnd2 (S13-127)** **346**

123 **THRB-Bhimsaria2022-Rnd2 (S07-035)** **349**

124 **THRB-Bhimsaria2022-Rnd3 (S07-155)** **352**

125 **THRB-Jolma2013-Rnd3 (ERR194572)** **354**

126 **THRB-Jolma2013-Rnd4 (ERR194573)** **357**

127 **THRB-Jolma2013-Rnd5 (ERR194574)** **360**

128 **THRB-Yin2017-Rnd2 (ERR1006596)** **363**

129 **THRB-Yin2017-Rnd2 (ERR1006597)** **366**

130 **THRB-Yin2017-Rnd3 (ERR1006598)** **369**

131 **THRB-Yin2017-Rnd3 (ERR1006599)** **372**

132 **THRB-Yin2017-Rnd4 (ERR1006600)** **374**

133 **THRB-Yin2017-Rnd4 (ERR1006601)** **377**

134 **THRB-Yin2017-Rnd2 (ERR1006604)** **380**

135 **THRB-Yin2017-Rnd2 (ERR1006605)** **383**

136 **THRB-Yin2017-Rnd3 (ERR1006606)** **386**

137 **THRB-Yin2017-Rnd3 (ERR1006607)** **389**

138 **THRB-Yin2017-Rnd4 (ERR1006608)** **392**

139 **THRB-Yin2017-Rnd4 (ERR1006609)** **395**

140 **THRB-7-Bhimsaria2022-Rnd3 (S07-103)** **398**

141 **RARA:RXRA-Bhimsaria2022-Rnd3 (S13-149)** **400**

142 **RARA-RXRA-SMiLE-seq-Rnd1 (SRR3405127)** **403**

143 **RARA-RXRG-SMiLE-seq-Rnd1 (SRR3405128)** **406**

144 **RARA-Bhimsaria2022-Rnd2 (S07-031)** **409**

145 **RARA-Bhimsaria2022-Rnd3 (S07-151)** 412
146 **RARA-Jolma2013-Rnd2 (ERR193727)** 415
147 **Rara-Jolma2013-Rnd2 (ERR194710)** 418
148 **RARA-Jolma2013-Rnd2 (ERR194785)** 421
149 **RARA-Jolma2013-Rnd3 (ERR193728)** 424
150 **Rara-Jolma2013-Rnd3 (ERR194711)** 427
151 **RARA-Jolma2013-Rnd3 (ERR194786)** 430
152 **RARA-Jolma2013-Rnd4 (ERR193729)** 433
153 **Rara-Jolma2013-Rnd4 (ERR194712)** 436
154 **Rara-Jolma2013-Rnd5 (ERR194713)** 439
155 **RARA-8-Bhimsaria2022-Rnd2 (S07-072)** 442
156 **RARA-8-Bhimsaria2022-Rnd3 (S07-097)** 445
157 **RARA-SMiLE-seq-Rnd1 (SRR3405076)** 448
158 **RARB:RXRA-Bhimsaria2022-Rnd3 (S13-150)** 451
159 **RARB-Bhimsaria2022-Rnd3 (S07-152)** 454
160 **Rarb-Jolma2013-Rnd2 (ERR194715)** 457
161 **Rarb-Jolma2013-Rnd3 (ERR194716)** 460
162 **Rarb-Jolma2013-Rnd4 (ERR194717)** 463
163 **RARB-Jolma2013-Rnd4 (ERR195056)** 466
164 **Rarb-Jolma2013-Rnd5 (ERR194718)** 469
165 **RARB-Yin2017-Rnd2 (ERR1005800)** 472
166 **RARB-Yin2017-Rnd2 (ERR1005801)** 475
167 **RARB-Yin2017-Rnd3 (ERR1005802)** 478
168 **RARB-Yin2017-Rnd3 (ERR1005803)** 481
169 **RARB-Yin2017-Rnd4 (ERR1005804)** 483
170 **RARB-Yin2017-Rnd4 (ERR1005805)** 485
171 **RARB-8-Bhimsaria2022-Rnd3 (S07-098)** 488
172 **RARG-Bhimsaria2022-Rnd2 (S07-033)** 491
173 **RARG-Bhimsaria2022-Rnd3 (S07-153)** 494

174 RARG-Jolma2013-Rnd2 (ERR195454) 497

175 Rarg-Jolma2013-Rnd2 (ERR195722) 500

176 RARG-Jolma2013-Rnd3 (ERR193908) 503

177 RARG-Jolma2013-Rnd3 (ERR195455) 506

178 Rarg-Jolma2013-Rnd3 (ERR195723) 509

179 RARG-Jolma2013-Rnd4 (ERR193909) 512

180 RARG-8-Bhimsaria2022-Rnd2 (S07-074) 515

181 CAR-Bhimsaria2022-Rnd3 (S07-165) 518

182 CAR-Yin2017-Rnd2 (ERR1005180) 520

183 CAR-Yin2017-Rnd2 (ERR1005181) 523

184 CAR-Yin2017-Rnd3 (ERR1005182) 526

185 CAR-Yin2017-Rnd3 (ERR1005183) 529

186 PXR-Bhimsaria2022-Rnd2 (S07-043) 531

187 PXR-Bhimsaria2022-Rnd3 (S07-164) 534

188 PXR-Yin2017-Rnd2 (ERR1005173) 536

189 PXR-Yin2017-Rnd3 (ERR1005174) 539

190 PXR-Yin2017-Rnd3 (ERR1005175) 542

191 PXR-Yin2017-Rnd4 (ERR1005176) 544

192 PXR-Yin2017-Rnd4 (ERR1005177) 547

193 PXR-9-Bhimsaria2022-Rnd2 (S07-067) 550

194 PXR-9-Bhimsaria2022-Rnd3 (S07-092) 553

195 VDR:RXRA-Bhimsaria2022-Rnd3 (S13-165) 555

196 VDR-RXRA-Rep2-SMiLE-seq-Rnd1 (SRR3405131) 556

197 VDR-Bhimsaria2022-Rnd2 (S07-054) 558

198 VDR-Bhimsaria2022-Rnd3 (S07-173) 561

199 VDR-Jolma2013-Rnd2 (ERR188863) 564

200 VDR-Jolma2013-Rnd3 (ERR188864) 566

201 Vdr-Jolma2013-Rnd3 (ERR195763) 569

202 VDR-Jolma2013-Rnd4 (ERR188865) 571

203	**Vdr-Jolma2013-Rnd4 (ERR195764)**	574
204	**VDR-SMiLE-seq-Rnd1 (SRR3405095)**	577
205	**FXR-Bhimsaria2022-Rnd3 (S07-163)**	580
206	**FXR-11-Bhimsaria2022-Rnd3 (S07-091)**	583
207	**LXRA-Bhimsaria2022-Rnd3 (S07-143)**	585
208	**LXRA-12-Bhimsaria2022-Rnd3 (S07-142)**	587
209	**LXRB:RXRA-Bhimsaria2022-Rnd3 (S13-142)**	589
210	**PPARa-RXRG-SMiLE-seq-Rnd1 (SRR3405126)**	592
211	**PPARD-Bhimsaria2022-Rnd3 (S07-148)**	595
212	**PPARD-Yin2017-Rnd2 (ERR1005720)**	596
213	**PPARD-Yin2017-Rnd2 (ERR1005721)**	597
214	**PPARD-Yin2017-Rnd3 (ERR1005722)**	598
215	**PPARD-Yin2017-Rnd3 (ERR1005723)**	600
216	**PPARD-Yin2017-Rnd4 (ERR1005724)**	603
217	**PPARD-Yin2017-Rnd4 (ERR1005725)**	606
218	**PPARD-13-Bhimsaria2022-Rnd2 (S07-027)**	609
219	**PPARD-13-Bhimsaria2022-Rnd3 (S07-147)**	612
220	**PPARD-14-Bhimsaria2022-Rnd3 (S07-095)**	614
221	**PPARG-Bhimsaria2022-Rnd2 (S07-030)**	616
222	**PPARG-Bhimsaria2022-Rnd3 (S07-150)**	619
223	**PPARG-14-Bhimsaria2022-Rnd3 (S07-096)**	620
224	**PPARG-15-Bhimsaria2022-Rnd3 (S07-149)**	621
225	**Rev-ErbA-Alpha-Yin2017-Rnd2 (ERR1005156)**	623
226	**Rev-ErbA-Alpha-Yin2017-Rnd2 (ERR1005157)**	625
227	**Rev-ErbA-Alpha-Yin2017-Rnd3 (ERR1005158)**	627
228	**Rev-ErbA-Alpha-Yin2017-Rnd3 (ERR1005159)**	630
229	**Rev-ErbA-Alpha-Yin2017-Rnd4 (ERR1005160)**	633
230	**Rev-ErbA-Alpha-Yin2017-Rnd4 (ERR1005161)**	636
231	**Rev-Erb-Beta-Yin2017-Rnd2 (ERR1005164)**	639

232 Rev-Erb-Beta-Yin2017-Rnd2 (ERR1005165) 642
233 Rev-Erb-Beta-Yin2017-Rnd3 (ERR1005166) 645
234 Rev-Erb-Beta-Yin2017-Rnd3 (ERR1005167) 648
235 Rev-Erb-Beta-Yin2017-Rnd4 (ERR1005168) 651
236 Rev-Erb-Beta-Yin2017-Rnd4 (ERR1005169) 654
237 RORA-Jolma2013-Rnd3 (ERR194476) 657
238 RORA-Jolma2013-Rnd4 (ERR194477) 660
239 RORB-Yin2017-Rnd2 (ERR1005892) 663
240 RORB-Yin2017-Rnd2 (ERR1005893) 666
241 RORB-Yin2017-Rnd3 (ERR1005894) 669
242 RORB-Yin2017-Rnd3 (ERR1005895) 672
243 RORB-Yin2017-Rnd4 (ERR1005896) 675
244 RORB-Yin2017-Rnd4 (ERR1005897) 677
245 RORB-Rep2-SMiLE-seq-Rnd1 (SRR3405080) 680
246 RORC:RXRA-Bhimsaria2022-Rnd3 (S13-157) 683
247 RORC-Bhimsaria2022-Rnd2 (S07-048) 685
248 RORC-Bhimsaria2022-Rnd3 (S07-169) 688
249 RORC-Yin2017-Rnd2 (ERR1005901) 691
250 RORC-Yin2017-Rnd3 (ERR1005903) 694
251 RORC-Yin2017-Rnd4 (ERR1005904) 696
252 RORC-Yin2017-Rnd4 (ERR1005905) 699
253 RORC-SMiLE-seq-Rnd1 (SRR3405081) 701
254 RORC-16-Bhimsaria2022-Rnd2 (S07-076) 704
255 RORC-16-Bhimsaria2022-Rnd3 (S07-101) 707
256 TR2-Bhimsaria2022-Rnd3 (S07-107) 710
257 TR2-Yin2017-Rnd4 (ERR1005192) 711
258 TR4-Bhimsaria2022-Rnd2 (S07-083) 714
259 TR4-Bhimsaria2022-Rnd3 (S07-108) 717
260 TR4-Jolma2013-Rnd2 (ERR195698) 719

261 **TR4-Jolma2013-Rnd3 (ERR195699)** 722

262 **TR4-Jolma2013-Rnd4 (ERR195700)** 725

263 **LRH1:RXRA-Bhimsaria2022-Rnd3 (S13-140)** 728

264 **LRH1-Bhimsaria2022-Rnd2 (S07-013)** 729

265 **LRH1-Bhimsaria2022-Rnd3 (S07-134)** 732

266 **LRH1-Yin2017-Rnd2 (ERR1005280)** 734

267 **LRH1-Yin2017-Rnd2 (ERR1005281)** 737

268 **LRH1-Yin2017-Rnd3 (ERR1005282)** 740

269 **LRH1-Yin2017-Rnd3 (ERR1005283)** 743

270 **LRH1-Yin2017-Rnd4 (ERR1005284)** 746

271 **LRH1-Yin2017-Rnd4 (ERR1005285)** 749

272 **SF1:RXRA-Bhimsaria2022-Rnd3 (S13-159)** 752

273 **SF1-Bhimsaria2022-Rnd2 (S07-012)** 754

274 **SF1-Bhimsaria2022-Rnd3 (S07-133)** 757

275 **TLX:RXRA-Bhimsaria2022-Rnd3 (S13-145)** 760

276 **TLX-Bhimsaria2022-Rnd2 (S07-084)** 763

277 **TLX-Bhimsaria2022-Rnd3 (S07-109)** 766

278 **Tlx-Jolma2013-Rnd3 (ERR185061)** 768

279 **TLX-Jolma2013-Rnd3 (ERR195035)** 771

280 **Tlx-Jolma2013-Rnd4 (ERR185062)** 774

281 **TLX-Jolma2013-Rnd4 (ERR195036)** 776

282 **TLX-Yin2017-Rnd3 (ERR1005202)** 779

283 **TLX-Yin2017-Rnd4 (ERR1005204)** 782

284 **TLX-Yin2017-Rnd4 (ERR1005205)** 785

285 **PNR-Bhimsaria2022-Rnd2 (S07-115)** 788

286 **PNR-Bhimsaria2022-Rnd3 (S07-110)** 791

287 **COUP-TF1:RXRA-Bhimsaria2022-Rnd3 (S13-133)** 793

288 **COUP-TF1-17-Bhimsaria2022-Rnd2 (S07-024)** 795

289 **COUP-TF1-17-Bhimsaria2022-Rnd3 (S07-144)** 798

290 COUP-TF1-Jolma2013-Rnd2 (ERR194433) 801
291 COUP-TF1-Jolma2013-Rnd3 (ERR193872) 804
292 COUP-TF1-Jolma2013-Rnd3 (ERR194434) 806
293 COUP-TF1-Jolma2013-Rnd4 (ERR193873) 809
294 COUP-TF1-Jolma2013-Rnd4 (ERR194435) 812
295 COUP-TF1-Jolma2013-Rnd5 (ERR194436) 815
296 COUP-TF1-Yin2017-Rnd2 (ERR1005208) 818
297 COUP-TF1-Yin2017-Rnd2 (ERR1005209) 821
298 COUP-TF1-Yin2017-Rnd3 (ERR1005210) 822
299 COUP-TF1-Yin2017-Rnd3 (ERR1005211) 825
300 COUP-TF1-Yin2017-Rnd4 (ERR1005212) 827
301 COUP-TF1-Yin2017-Rnd4 (ERR1005213) 830
302 COUP-TF1-Yin2017-Rnd2 (ERR1005216) 833
303 COUP-TF1-Yin2017-Rnd2 (ERR1005217) 836
304 COUP-TF1-Yin2017-Rnd3 (ERR1005218) 839
305 COUP-TF1-Yin2017-Rnd3 (ERR1005219) 842
306 COUP-TF1-Yin2017-Rnd4 (ERR1005220) 845
307 COUP-TF1-Yin2017-Rnd4 (ERR1005221) 848
308 COUP-TF2:RXRA-Bhimsaria2022-Rnd3 (S13-134) 851
309 COUP-TF2-17-Bhimsaria2022-Rnd2 (S07-025) 853
310 COUP-TF2-17-Bhimsaria2022-Rnd3 (S07-145) 856
311 COUP-TF2-Bhimsaria2022-Rnd2 (S07-117) 858
312 COUP-TF2-Bhimsaria2022-Rnd3 (S07-112) 861
313 EAR2:RXRA-Bhimsaria2022-Rnd3 (S13-135) 864
314 EAR2-Bhimsaria2022-Rnd2 (S07-118) 867
315 EAR2-Bhimsaria2022-Rnd3 (S07-113) 870
316 EAR2-Jolma2013-Rnd2 (ERR193875) 872
317 EAR2-Jolma2013-Rnd2 (ERR195350) 875
318 EAR2-Jolma2013-Rnd3 (ERR193876) 878

319 EAR2-Jolma2013-Rnd3 (ERR195351) 881
320 Ear2-Jolma2013-Rnd3 (ERR195703) 884
321 EAR2-Jolma2013-Rnd4 (ERR193877) 887
322 EAR2-Yin2017-Rnd2 (ERR1895322) 890
323 EAR2-Yin2017-Rnd2 (ERR1895323) 893
324 EAR2-Yin2017-Rnd3 (ERR1895325) 895
325 EAR2-Yin2017-Rnd4 (ERR1005224) 897
326 EAR2-Yin2017-Rnd4 (ERR1005225) 899
327 EAR2-Yin2017-Rnd4 (ERR1895327) 901
328 EAR2-Yin2017-Rnd2 (ERR1005228) 903
329 EAR2-Yin2017-Rnd2 (ERR1005229) 906
330 EAR2-Yin2017-Rnd3 (ERR1005230) 909
331 EAR2-Yin2017-Rnd3 (ERR1005231) 912
332 HNF4A-Bhimsaria2022-Rnd3 (S07-161) 915
333 HNF4A-Jolma2013-Rnd2 (ERR194035) 917
334 HNF4A-Jolma2013-Rnd2 (ERR194355) 920
335 Hnf4a-Jolma2013-Rnd2 (ERR195634) 922
336 HNF4A-Jolma2013-Rnd3 (ERR194036) 925
337 HNF4A-Jolma2013-Rnd3 (ERR194356) 928
338 Hnf4a-Jolma2013-Rnd3 (ERR195635) 931
339 HNF4A-Jolma2013-Rnd4 (ERR194037) 934
340 HNF4A-Jolma2013-Rnd4 (ERR194357) 937
341 Hnf4a-Jolma2013-Rnd4 (ERR195636) 940
342 HNF4A-Jolma2013-Rnd5 (ERR194358) 943
343 HNF4A-Jolma2013-Rnd6 (ERR194359) 946
344 HNF4A-Yin2017-Rnd2 (ERR1003748) 949
345 HNF4A-Yin2017-Rnd2 (ERR1003749) 952
346 HNF4A-Yin2017-Rnd3 (ERR1003750) 955
347 HNF4A-Yin2017-Rnd3 (ERR1003751) 958

348 HNF4A-Yin2017-Rnd4 (ERR1003752) 961
349 HNF4A-Yin2017-Rnd4 (ERR1003753) 964
350 HNF4A-18-Bhimsaria2022-Rnd2 (S07-008) 967
351 HNF4A-18-Bhimsaria2022-Rnd3 (S07-129) 970
352 HNF4G-Bhimsaria2022-Rnd2 (S07-009) 972
353 HNF4G-Bhimsaria2022-Rnd3 (S07-130) 975
354 HNF4G-Bhimsaria2022-Rnd3 (S07-162) 976
355 RXRA:RXRA-Bhimsaria2022-Rnd3 (S13-158) 979
356 RXRA-17-Bhimsaria2022-Rnd1 (S04-147) 982
357 RXRA-17-Bhimsaria2022-Rnd2 (S04-148) 985
358 RXRA-17-Bhimsaria2022-Rnd2 (S07-014) 988
359 RXRA-17-Bhimsaria2022-Rnd3 (S04-149) 991
360 RXRA-17-Bhimsaria2022-Rnd3 (S07-135) 994
361 RXRA-Bhimsaria2022-Rnd2 (S07-049) 997
362 RXRA-Bhimsaria2022-Rnd3 (S07-170) 1000
363 RXRA-Jolma2013-Rnd2 (ERR194225) 1003
364 RXRA-Jolma2013-Rnd2 (ERR194493) 1005
365 RXRA-Jolma2013-Rnd3 (ERR194226) 1008
366 RXRA-Jolma2013-Rnd3 (ERR194494) 1010
367 Rxra-Jolma2013-Rnd3 (ERR194856) 1012
368 RXRA-Jolma2013-Rnd4 (ERR194227) 1015
369 RXRA-Jolma2013-Rnd4 (ERR194495) 1018
370 Rxra-Jolma2013-Rnd4 (ERR194857) 1021
371 RXRA-Jolma2013-Rnd5 (ERR194496) 1024
372 RXRA-Yin2017-Rnd2 (ERR1005924) 1027
373 RXRA-Yin2017-Rnd2 (ERR1005925) 1030
374 RXRA-Yin2017-Rnd3 (ERR1005926) 1032
375 RXRA-Yin2017-Rnd3 (ERR1005927) 1034
376 RXRA-Yin2017-Rnd4 (ERR1005928) 1037

**377**	**RXRA-Yin2017-Rnd4 (ERR1005929)**	**1039**
**378**	**RXRA-SMiLE-seq-Rnd1 (SRR3405082)**	**1041**
**379**	**RXRB-17-Bhimsaria2022-Rnd3 (S07-136)**	**1044**
**380**	**RXRB-Bhimsaria2022-Rnd3 (S07-171)**	**1047**
**381**	**RXRB-Jolma2013-Rnd2 (ERR195462)**	**1050**
**382**	**Rxrb-Jolma2013-Rnd3 (ERR194859)**	**1053**
**383**	**RXRB-Jolma2013-Rnd3 (ERR195463)**	**1056**
**384**	**Rxrb-Jolma2013-Rnd4 (ERR194860)**	**1059**
**385**	**RXRB-Jolma2013-Rnd4 (ERR195464)**	**1061**
**386**	**RXRB-Yin2017-Rnd2 (ERR1005932)**	**1064**
**387**	**RXRB-Yin2017-Rnd2 (ERR1005933)**	**1066**
**388**	**RXRB-Yin2017-Rnd3 (ERR1005934)**	**1067**
**389**	**RXRB-Yin2017-Rnd3 (ERR1005935)**	**1069**
**390**	**RXRB-Yin2017-Rnd4 (ERR1005936)**	**1070**
**391**	**RXRB-Yin2017-Rnd4 (ERR1005937)**	**1073**
**392**	**RXRG:RXRA-Bhimsaria2022-Rnd3 (S13-162)**	**1076**
**393**	**RXRG-17-Bhimsaria2022-Rnd2 (S07-016)**	**1079**
**394**	**RXRG-17-Bhimsaria2022-Rnd3 (S07-137)**	**1082**
**395**	**RXRG-Bhimsaria2022-Rnd2 (S07-051)**	**1085**
**396**	**RXRG-Bhimsaria2022-Rnd3 (S07-172)**	**1088**
**397**	**RXRG-Jolma2013-Rnd2 (ERR195466)**	**1091**
**398**	**RXRG-Jolma2013-Rnd3 (ERR195467)**	**1094**
**399**	**RXRG-Jolma2013-Rnd4 (ERR195468)**	**1097**
**400**	**RXRG-Yin2017-Rnd4 (ERR1005945)**	**1100**
**401**	**NOR1-Bhimsaria2022-Rnd2 (S07-011)**	**1103**
**402**	**NOR1-Bhimsaria2022-Rnd3 (S07-132)**	**1106**
**403**	**NURR1-Bhimsaria2022-Rnd1 (S04-144)**	**1109**
**404**	**NURR1-Bhimsaria2022-Rnd2 (S04-145)**	**1112**
**405**	**NURR1-Bhimsaria2022-Rnd3 (S04-146)**	**1115**

406 NURR1-Bhimsaria2022-Rnd3 (S07-131) 1118
407 NURR1-Rep2-SMiLE-seq-Rnd1 (SRR3405072) 1120
408 NURR1-RXRA-Rep1-SMiLE-seq-Rnd1 (SRR3405118) 1123
409 NURR1-Jolma2013-Rnd2 (ERR195038) 1126
410 NURR1-Jolma2013-Rnd3 (ERR195039) 1129
411 NURR1-Jolma2013-Rnd4 (ERR195040) 1132
412 NURR1-Yin2017-Rnd4 (ERR1005269) 1135
413 NURR77-Yin2017-Rnd2 (ERR1005253) 1138
414 NURR77-Yin2017-Rnd3 (ERR1005257) 1141
415 NURR77-Yin2017-Rnd4 (ERR1005259) 1144
416 NURR77-Yin2017-Rnd4 (ERR1005260) 1147
417 NURR77-Yin2017-Rnd4 (ERR1005261) 1150
418 GCNF-Yin2017-Rnd2 (ERR1005288) 1153
419 GCNF-Yin2017-Rnd2 (ERR1005289) 1156
420 GCNF-Yin2017-Rnd3 (ERR1005290) 1158
421 GCNF-Yin2017-Rnd3 (ERR1005291) 1161
422 GCNF-Yin2017-Rnd4 (ERR1005292) 1164
423 GCNF-Yin2017-Rnd4 (ERR1005293) 1167

1 AR-Jolma2013-Rnd2 (ERR193971)

PWM	Seed Sequence	Seed Seq Enrichment	Repeat	Rank
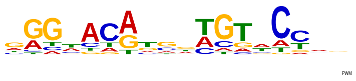	*NNGTACNNNNNGTTCCNN*	8.01	M	1
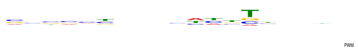	*NCCCCNNNNNNNNNTTTTN*	2.03	M	2
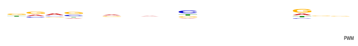	*GGAGNNNNNNNNNNCGGG*	2.11	M	3
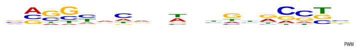	*NNNNNACACNGTGTNNNNN*	2.24	M	4
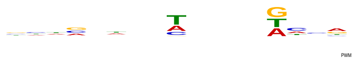	*NNNNATGANACGGNNNN*	1.35	M	5
	NNNGGGGNNNNNGGGGNNN	1.91	M	6
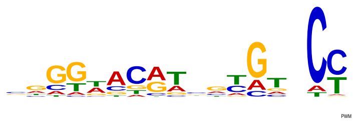	*NNNGTACNNCGTGTNNN*	5.00	M	7
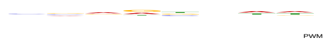	*TAGGGAAA*	1.30	M	8
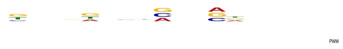	*NGGGGNNNNNNNNNATATN*	1.56	M	9

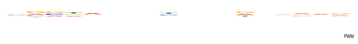	$\overrightarrow{GGGTN}NNNNNNNNNNAAAG$	1.96	M	10
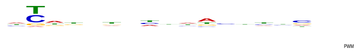	NNNTTTCNNNNCTTTNNN	1.72	M	11
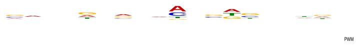	$NNGAGANNNNNN\overrightarrow{NGGTTN}N$	1.53	M	12
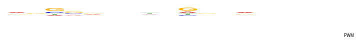	$NGGGANNNNNNNNA\overrightarrow{AGGN}$	1.84	M	13
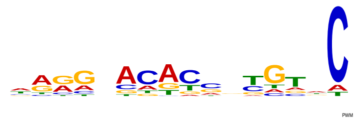	$NN\overrightarrow{AGTACN}NNGTGTNN$	4.69	M	14
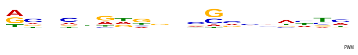	$NNNNNG\overrightarrow{TGGNCG}CANNNNN$	1.97	M	15
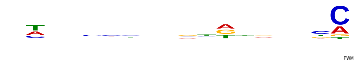	NNCTCCNNNNNNTGTGNN	1.52	M	16
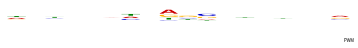	NNTTTANNNNNNTCTTNN	1.50	M	17
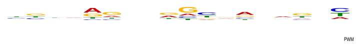	NNTAAGNNNNNNACAGNN	1.73	M	18

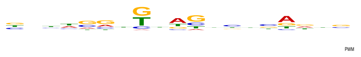	$NNN\overrightarrow{TGGTNN}N\overrightarrow{NNCTCA}NNNN$	2.04	DR:1	19
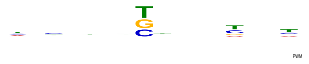	$NNNNTTTTNNNTTTTNNNN$	1.87	M	20

2 Ar-Jolma2013-Rnd2 (ERR194680)

PWM	Seed Sequence	Seed Seq Enrichment	Repeat	Rank
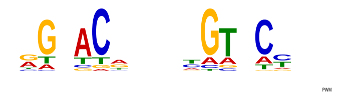	$NN\overleftarrow{NGAAC}NNNN\overrightarrow{NGTTC}NNN$	33.40	ER:3	1
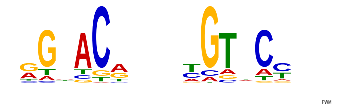	$N\overrightarrow{NGGTAN}NNNNN\overleftarrow{NTACC}NN$	17.80	IR:5	2
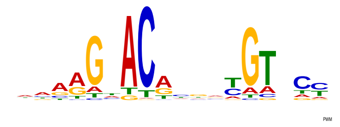	$NNNAAAGNNNNNN\overrightarrow{ATGTNN}N$	13.02	M	3
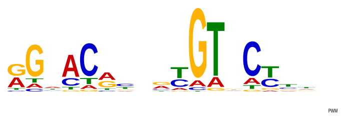	*NGGAANNNNNNNNNCTTTN*	9.26	M	4
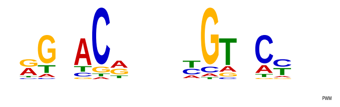	*NNAGTANNNNNNNTACTNN*	11.63	M	5
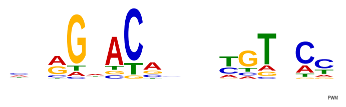	*NNNAGAANNNTCTGTNNN*	16.20	M	6
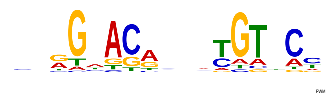	*NNNNGGAANNNAATGNNNN*	10.25	M	7
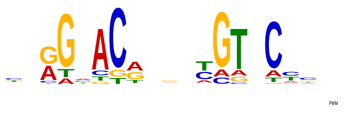	$N\overrightarrow{NGGGAA}NNNNNNNTACTNN$	17.80	M	8
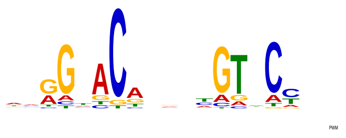	$NN\overrightarrow{AGGTN}NNNNNN\overleftarrow{NTACC}NN$	9.96	IR:5	9

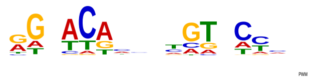	$NN\overrightarrow{AAGTNN}NNNNNNTTCCNN$	6.95	M	10
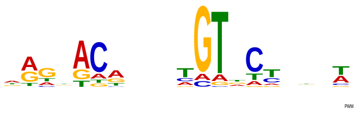	$NNNTACANNNNNNNTCTTNNN$	8.28	M	11
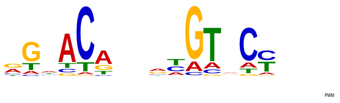	$\overrightarrow{NGGTAN}NNNNNNNNCTTTN$	7.29	M	12

3 AR-Jolma2013-Rnd3 (ERR193680)

PWM	Seed Sequence	Seed Seq Enrichment	Repeat	Rank
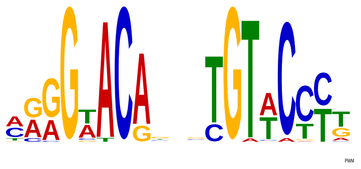	NNGGTACANNNNGTACCNN	218.72	IR:5	1
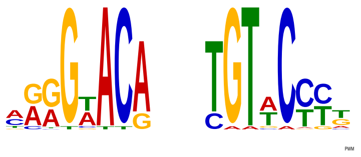	NNGGTACNNNNNGTACCNN	77.38	IR:5	2
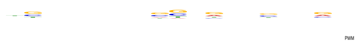	NNGGGGNNNNNNNGGGGNN	6.69	M	3
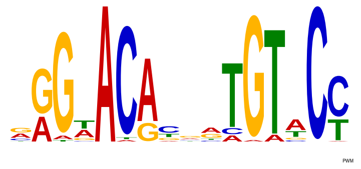	NGGTACACNNNGTACN	162.82	M	4
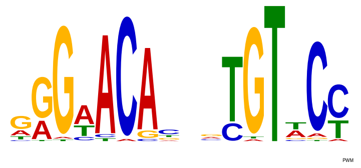	NGGAACACNNNGTTCN	158.95	DR:3	5
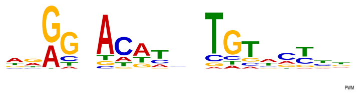	NNNGTACNNNNNGTGCNNN	4.55	M	6
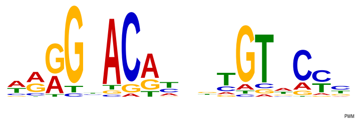	NNGGTACNNNNNGTGCNN	11.07	M	7
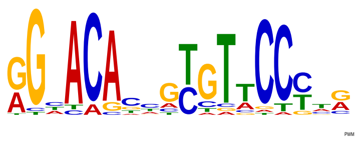	NNNNCACCGNGTTCNNNN	11.39	ER:0	8
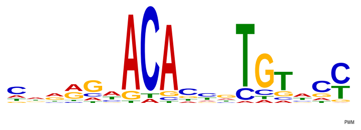	NAAAGNNNNNNNNGTTCN	3.63	M	9

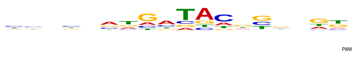	NNNNNATGATACGNNNNN	3.42	M	10
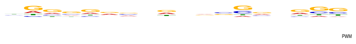	NGGGGNNNNNNNGGTGGN	7.88	M	11
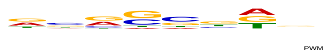	ACGGCGTG	2.73	M	12
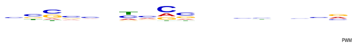	NCCCCNNNNNNNCCCCCN	10.25	M	13
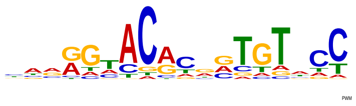	NAAGGNNNNNNNNGTACN	5.63	M	14
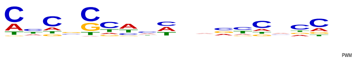	NNCGCCNNNNNNNTCACNN	4.11	M	15
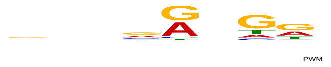	GGCGGCGA	2.36	M	16

4 AR-Jolma2013-Rnd3 (ERR193972)

PWM	Seed Sequence	Seed Seq Enrichment	Repeat	Rank
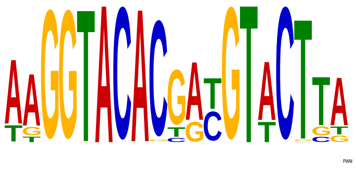	NNGGTACACNNCGTACTNN	11527.78	IR:4	1
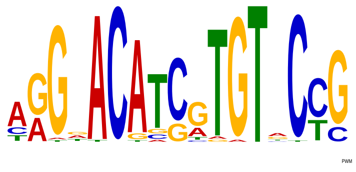	NAGGACANNNTGTACTN	443.85	IR:3	2
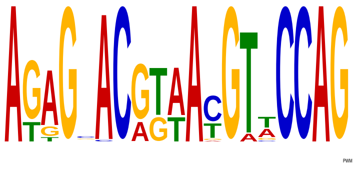	NNAGCACANNNCGTTCCNN	582.48	DR:3	3
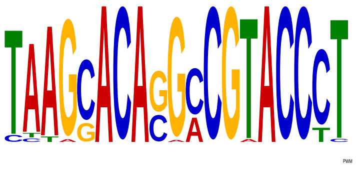	NNAGCACACNCCGTACCNN	1580.15	IR:4	4
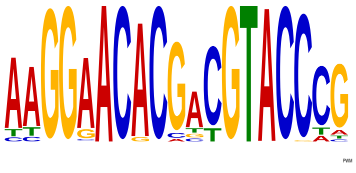	NNGGAACACNNCGTACCNN	7744.59	IR:4	5
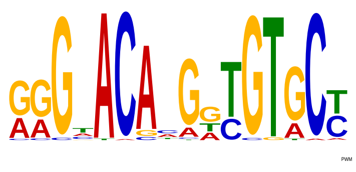	GGGCACANNNNGTGCT	341.02	IR:5	6
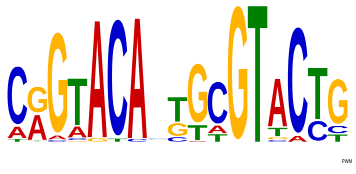	NGGAACANNNCGTTCCN	312.52	DR:3	7
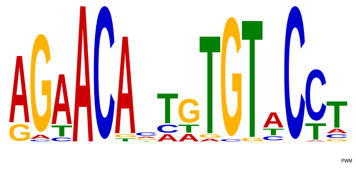	NGAACAGNNTGTCCCN	224.72	IR:3	8
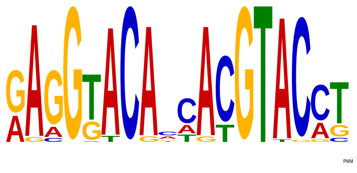	NNGGTACAGANNGTACNN	425.19	M	9

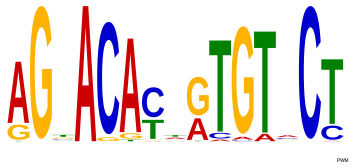	$\overrightarrow{AGGACA}NNN\overleftarrow{NGTCCT}$	108.33	IR:3	10
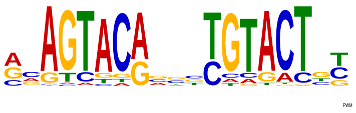	$N\overleftarrow{NGGAAC}NNNN\overrightarrow{NGTTCC}NN$	36.14	ER:3	11
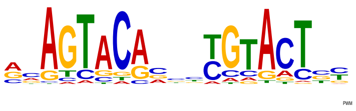	$NNGGAANNNNN\overrightarrow{NGTTCC}NN$	9.77	M	12
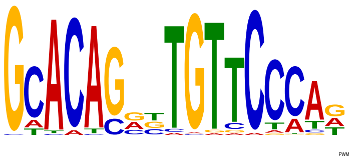	$NCACANNN\overrightarrow{NGTTCC}AAN$	70.90	M	13
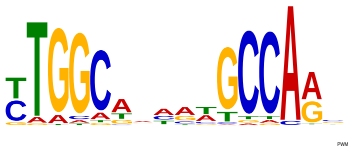	$\overrightarrow{NTGGCA}NN\overrightarrow{NNGCCA}AN$	24.30	DR:2	14
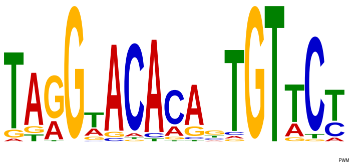	$NN\overrightarrow{GGGACA}NNCTGTTNN$	55.29	M	15
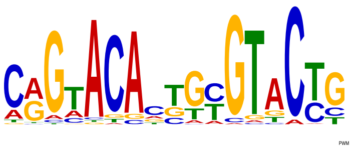	$NGGAANNNNN\overrightarrow{CGTTCC}N$	30.84	M	16
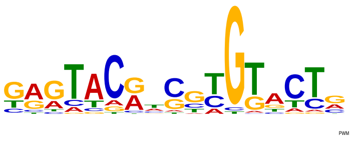	$N\overrightarrow{GGGACN}NNNNNTTCCN$	5.64	M	17
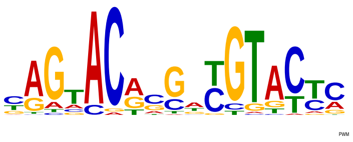	$N\overleftarrow{GGAACN}NNN\overrightarrow{NGTCCC}N$	20.20	ER:2	18

5 Ar-Jolma2013-Rnd3 (ERR194681)

PWM	Seed Sequence	Seed Seq Enrichment	Repeat	Rank
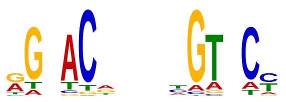	NNNGAACNNNNNGTTCNNN	45.13	ER:3	1
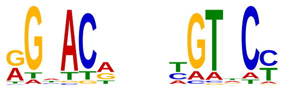	NNGGAANNNNNNNTTCCNN	33.45	M	2
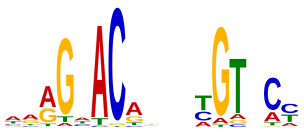	NNNAAAGNNNNNNATGTNNN	26.25	M	3
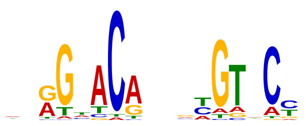	NNAGGTNNNNNNNNTACCNN	18.42	IR:5	4
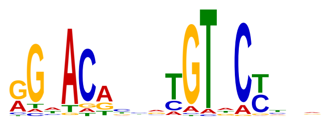	NNGGAANNNNNNNNGCTTNN	15.77	M	5
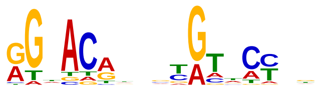	NNNNCAGANNNTTCTNNNN	12.97	M	6
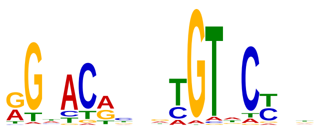	NGGAANNNNNNNNNCTCAN	10.83	M	7
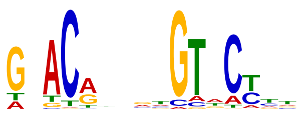	NNNCATTNNNNNCTTTNNN	14.06	M	8
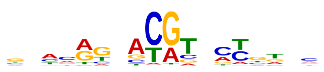	NNNACGGNNNNNNCCGTNNN	14.35	M	9

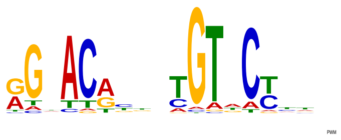	*NAGAANNNNNNNNNCTTTN*	14.24	M	10

6 AR-Jolma2013-Rnd4 (ERR193681)

PWM	Seed Sequence	Seed Seq Enrichment	Repeat	Rank
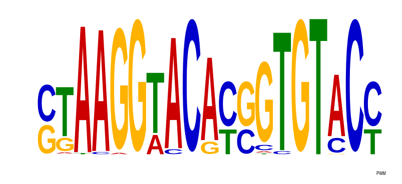	NTAAGGTACNNNGTGTACN	30468.13	IR:4	1
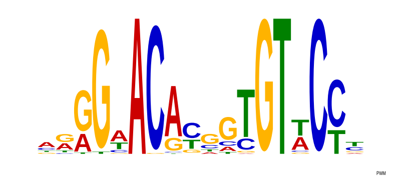	NNGGTACNNNNNGTTCCNN	740.36	DR:4	2
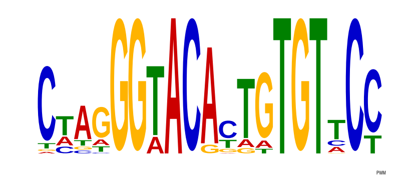	NTAGGGTACNNNNTGTTCN	5027.28	DR:5	3
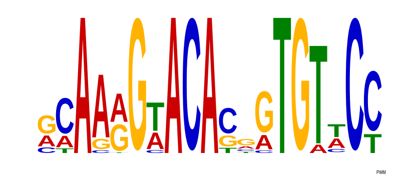	NAAAAGTACNNNNTGTTCN	3871.68	DR:4	4
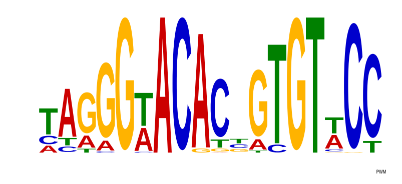	TAGGGNNNNNNNNGTTCC	309.38	DR:5	5
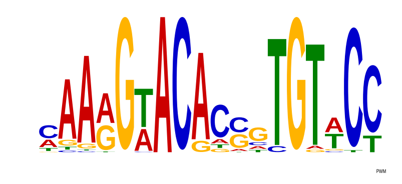	AAAAGNNNNNNNNGTTCC	251.86	M	6
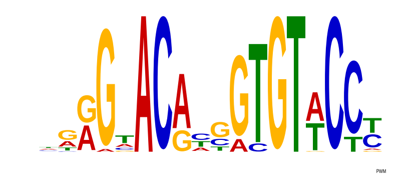	NGAGTACNNNNNGTACCN	641.49	IR:4	7
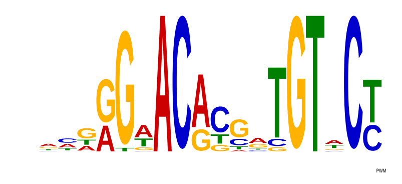	NTGGGAACNNNNNGTACN	481.69	M	8
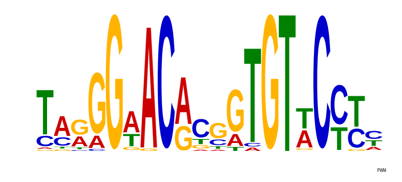	NNGGGTANNNNNNNTTCCNN	118.34	M	9

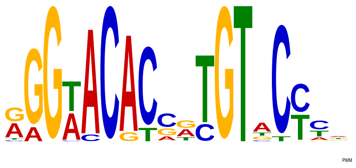	$\overrightarrow{NGGTAC}NNNNNNTACTCN$	179.45	M	10
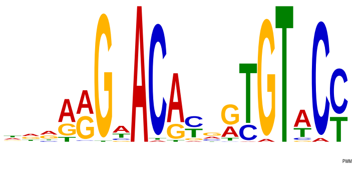	$NNNA\overrightarrow{AGAAC}NNN\overrightarrow{ATGTN}NN$	256.13	DR:2	11
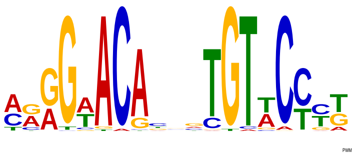	$N\overrightarrow{NGGTAN}NNNNN\overleftarrow{NTACCN}N$	50.88	IR:5	12
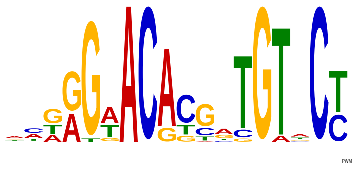	$N\overrightarrow{TGGGAA}NNNNN\overleftarrow{TGTACN}$	307.91	IR:5	13
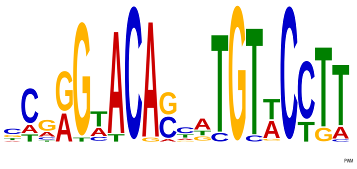	$N\overrightarrow{NGGGTA}NNNNNN\overleftarrow{NTACCN}N$	100.63	IR:6	14

7 AR-SelexGLM-Rnd2 (SRR5340731)

PWM	Seed Sequence	Seed Seq Enrichment	Repeat	Rank
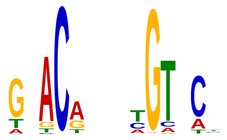	*NNNNNGTACANNNNGTACNNNNN*	551.70	M	1
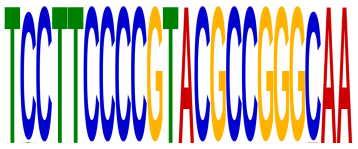	*NNCTTCCCCGNNCGCCGGGCNN*	59908.81	ER:6	2
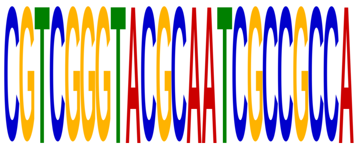	*CGTCGGGTNNNNNNNCGCCGCCA*	42937.68	DR:12	3
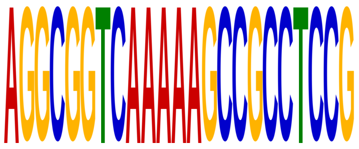	*AGGCGGTCNNNNNNNCGCCTCCG*	45789.97	IR:5	4
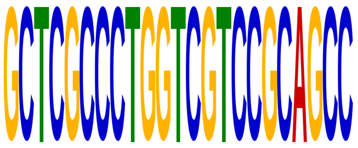	*GCTCGCCCNNNNNNNCCGCAGCC*	43433.94	M	5
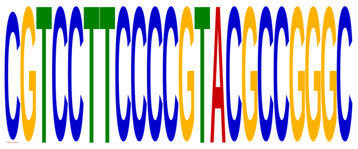	*CGTCCTTCNNNNNNCGCCGGGC*	44677.65	ER:11	6
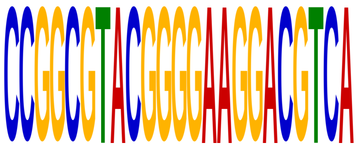	*CCGGCGTANNNNNNNGGACGTCA*	44123.36	ER:10	7
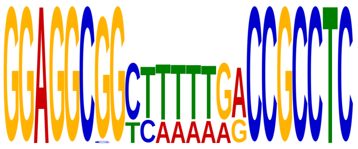	*GGAGGCGGNNNNNNNNCCGCCTC*	21707.84	IR:8	8
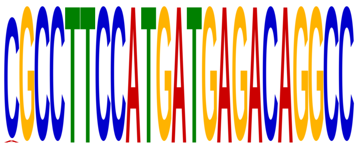	*CGCCTTCCNNNNNNNGACAGGCC*	37065.02	ER:13	9

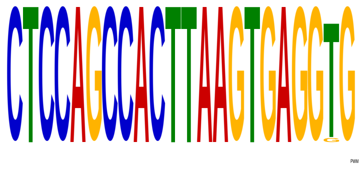	*CTCCAGCCNNNNNNGTGAGGTG*	41133.56	M	10
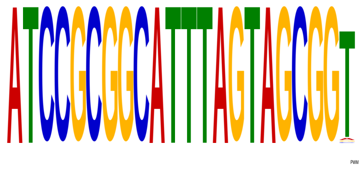	*NTCCGCGGCNNNNAGTAGCGGN*	38568.87	M	11
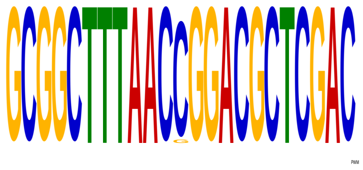	*GCGGCTTTNNNNNNNCGCTCGAC*	35033.79	ER:8	12
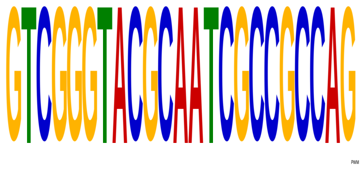	*GTCGGGTANNNNNNNGCCGCCAG*	39006.90	DR:7	13
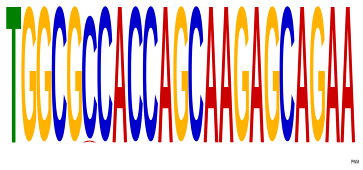	*NGGCGCCACNNNNNAGAGCAGAN*	42809.89	DR:6	14
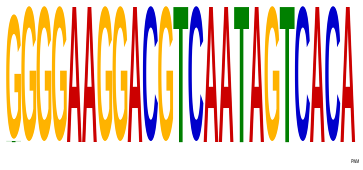	*GGGGAAGGNNNNNNTAGTCACA*	35226.46	M	15
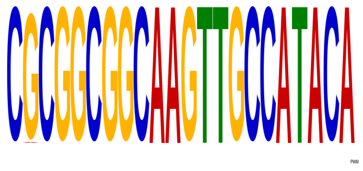	*CGCGGCGGNNNNNNGCCATACA*	41950.62	M	16
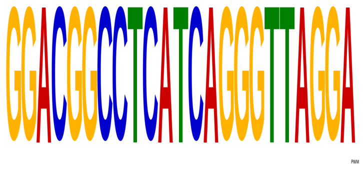	*GGACGGCCNNNNNNNGGTTAGGA*	34715.83	DR:9	17
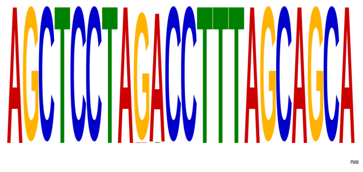	*NGCTCCTAGNNNNTTAGCAGCN*	37548.04	IR:7	18

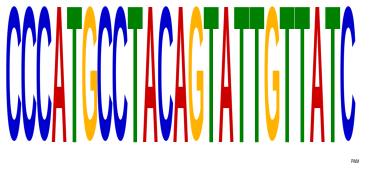	$CCC\overrightarrow{ATGCCN}NNNNNN\overrightarrow{TTGTTA}TC$	37625.87	DR:6	19
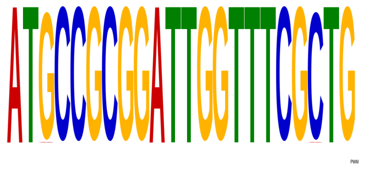	$NNGCCGCGGAN\overrightarrow{NGGTTT}CGCNN$	36777.78	M	20

8 AR-SelexGLM-Rnd3 (SRR5340730)

PWM	Seed Sequence	Seed Seq Enrichment	Repeat	Rank
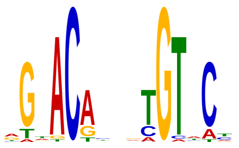	*NNNNNGTACANNNTGTACNNNNN*	4279.43	IR:3	1
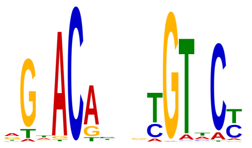	*NNNNGTACANNNNGTTCCNNNN*	2621.91	DR:3	2
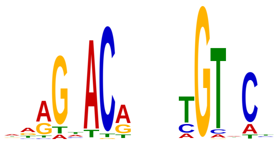	*NNNAAAGAACNNNNTGTACNNN*	5679.98	IR:3	3
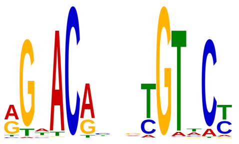	*NNNNGGAACNNNNNGTTCCNNNN*	1363.80	ER:3	4
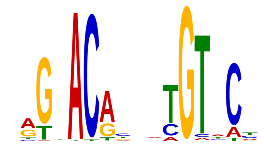	*NNNNAGCACACNNTGTACNNNN*	5405.55	IR:3	5
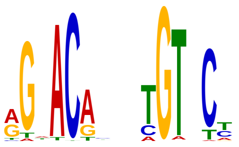	*NNNNAGAACANNNTGTCCTNNNN*	6544.50	IR:3	6
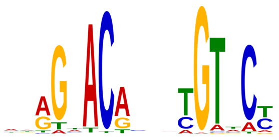	*NNNAAGAACNNNNNGTTCCNNN*	2036.04	DR:3	7
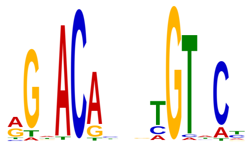	*NNNNAGAACATNNNGTACNNNN*	3241.79	M	8
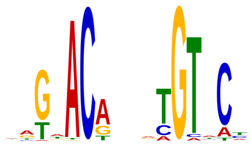	*NNNNTAGTACNNNNTGTACNNNN*	3056.32	IR:3	9

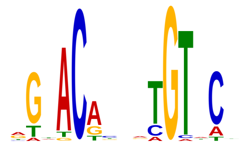 PWM	NNNNNGTACACAATGTACNNNNN	8583.73	DR:2	10
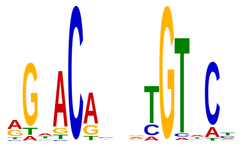 PWM	NNNNAGGTACNNNNTGTACNNNN	2490.65	IR:4	11
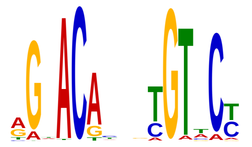 PWM	NNNNAGGACANNNNGTTCCNNNN	2414.90	DR:3	12
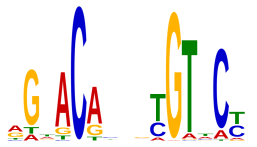 PWM	NNNAGGTACANNNNGTTCCNNN	3970.61	DR:4	13
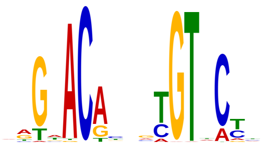 PWM	NNNNAGTACANNNNGTGCTNNNN	2243.51	IR:3	14
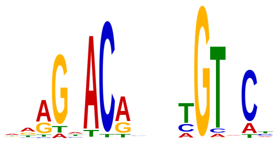 PWM	NNNAAAGAACANNNNGTACNNN	5407.24	M	15
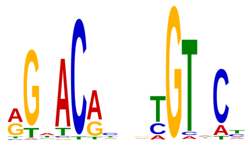 PWM	NNNNGGAACACGNNGTACNNNN	4228.00	M	16
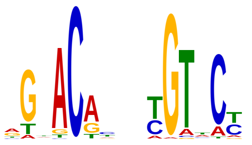 PWM	NNNNGTACACGNNGTTCCNNNN	4084.02	DR:3	17
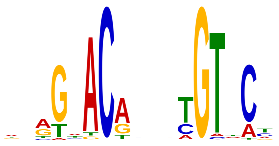 PWM	NNNAAGTACNNNNNGTACTNNN	2226.98	IR:4	18

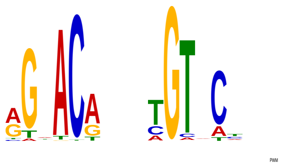	$NNNCT\overrightarrow{AGAACA}NNN\overleftarrow{TGTAC}NNN$	8029.28	IR:3	19
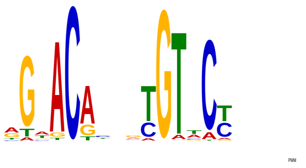	$NNN\overrightarrow{NGGTAC}NNNN\overrightarrow{NGTTCC}NNNN$	1259.75	DR:4	20

9 AR-SelexGLM-Rnd4 (SRR5340729)

PWM	Seed Sequence	Seed Seq Enrichment	Repeat	Rank
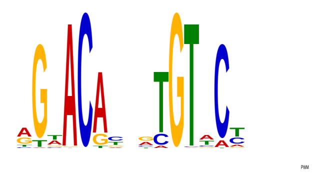	NNNNNGTACANNNTGTACNNNNN	8317.25	IR:3	1
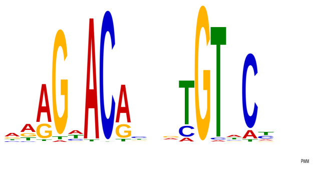	NNNAAAGAACANNNTGTACNNN	39581.63	IR:3	2
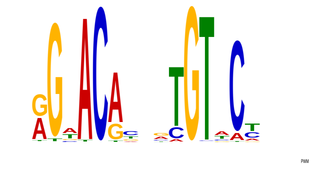	NNNNGGGAACANNNTGTACNNNN	16183.31	IR:5	3
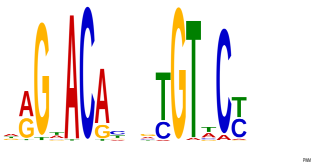	NNNNAGAACACNNNGTTCCNNNN	13933.02	DR:3	4
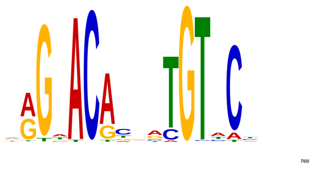	NNNNAGAACACAATGTACNNNN	44394.81	DR:2	5
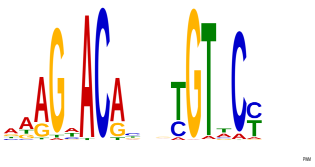	NNNAAAGAACANNNNGTTCCNNN	24142.42	DR:3	6
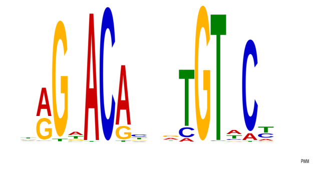	NNNCTAGAACANNNTGTACNNN	19684.86	IR:3	7
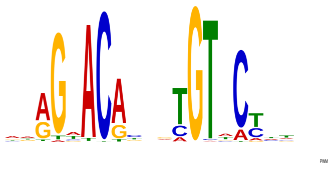	NNNAAGAACANNNNGTACTTNNN	19761.34	IR:3	8
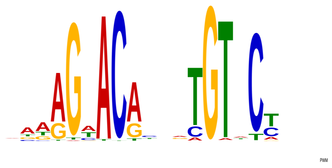	NNNAAAGAACANNNTGTCCTNNN	34783.69	IR:3	9

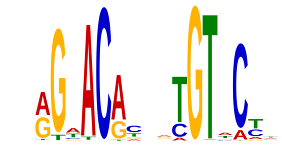 PWM	NNNNGGAACACGNNGTACTNNNN	19378.29	IR:3	10
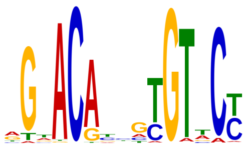 PWM	NNNNGTACATCGNGTTCCNNNN	18543.98	DR:3	11
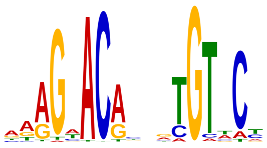 PWM	NNNAAAGAACNNNNTGTACCNNN	20771.66	IR:3	12
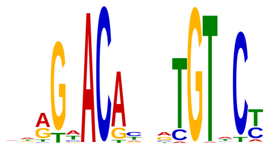 PWM	NNNNAGTACACNNTGTCCTNNNN	20907.52	IR:3	13
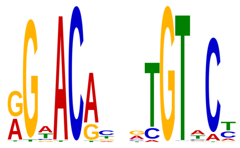 PWM	NNNNGGGAACNNNNTGTACNNNN	6323.24	IR:5	14
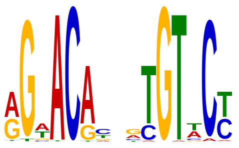 PWM	NNNNGGAACNNNNNGTTCCNNNN	2297.84	ER:3	15
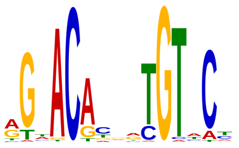 PWM	NNNNNGTACACGATGTACNNNNN	24984.55	IR:0	16
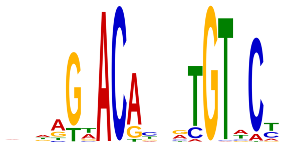 PWM	NNNNTAGTACNNNNTGTACNNNN	5950.92	IR:3	17
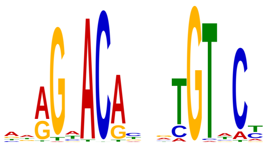 PWM	NNNNAAGAACACNNNGTACNNNN	12474.37	M	18

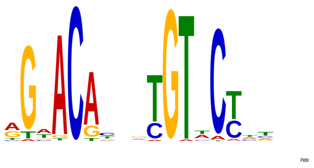	$NN\overrightarrow{NGGTAC}ACNNN\overrightarrow{NGTTCT}TNNN$	17215.91	DR:4	19
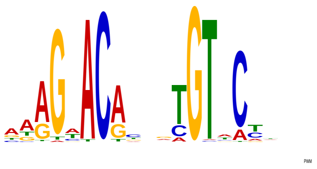	$NNNAA\overrightarrow{AGAACN}NNN\overleftarrow{NGTACT}NNN$	9710.87	IR:3	20

10 AR-SelexGLM-Rnd5 (SRR5340728)

PWM	Seed Sequence	Seed Seq Enrichment	Repeat	Rank
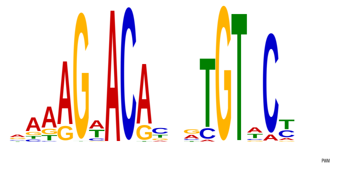	NNNAAAGAACANNNTGTACNNN	108952.05	IR:3	1
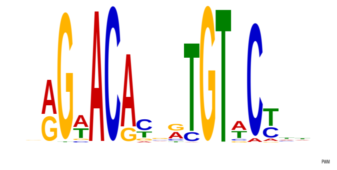	NNNNGGAACACNNTGTACNNNN	40447.85	IR:3	2
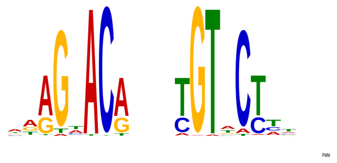	NNAAAGAACANNNTGTTCTTTNN	472148.19	IR:3	3
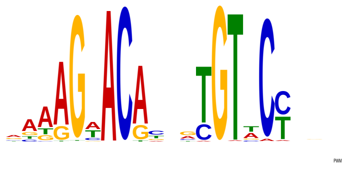	NNAAAGAACANNNTGTTCCCNN	222771.26	DR:3	4
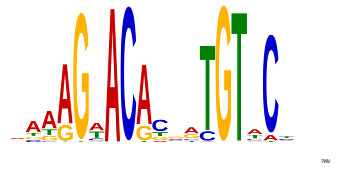	NNNAAAGAACACAATGTACNNN	377127.41	DR:2	5
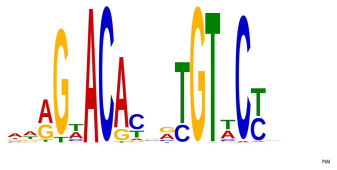	NNNNAGTACACGNNGTTCCNNNN	42332.69	DR:3	6
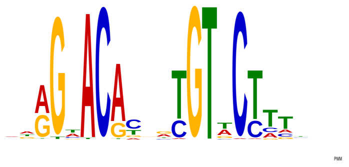	NNNAGAACACGNNGTTCTTTNNN	166828.95	DR:3	7
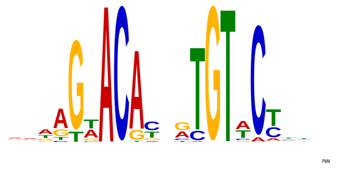	NNNNTAGTACANNNTGTACNNNN	30643.09	IR:3	8
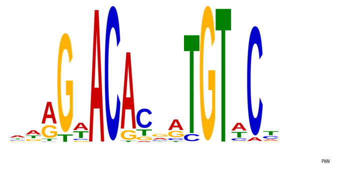	NNNNAGTACACAATGTACNNNN	96453.87	DR:2	9

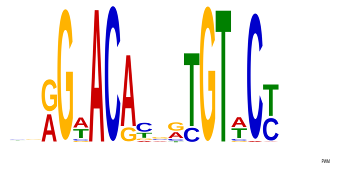	NNNGGGAACACNNTGTTCCNNN	77443.32	DR:5	10
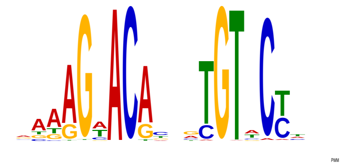	NNNAAAGAACANNNTGTTCTNNN	205757.11	IR:3	11
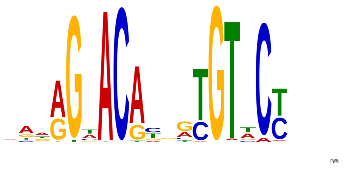	NNNAAGAACACCGTGTTCCNNN	272500.45	DR:3	12
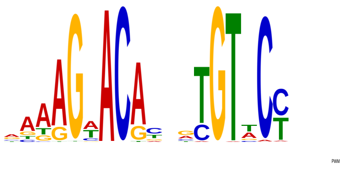	NNAAAGAACANNNNGTTCCCNN	82704.39	DR:3	13
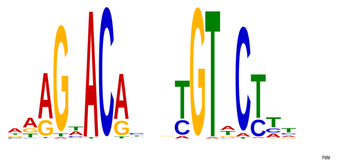	NNAAAGAACANNNNGTTCTTTNN	189876.35	DR:3	14
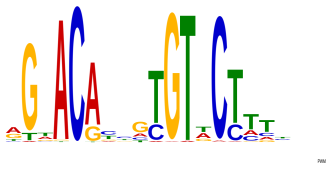	NNNGTACATCGNGTTCTTTNNN	150904.49	DR:3	15
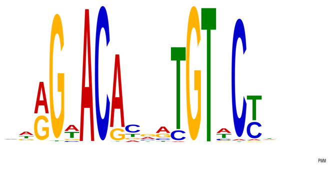	NNNNAGAACACAATGTTCTNNNN	176689.76	IR:3	16
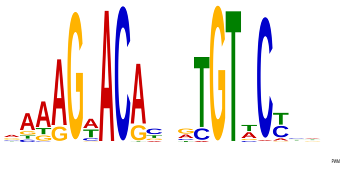	NNAAAGAACNNNNTGTACCCNN	70692.90	IR:3	17
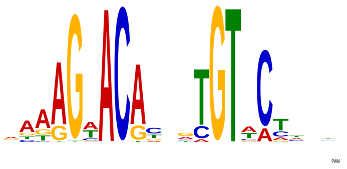	NNAAAGAACANNNNGTACTANN	83457.15	IR:3	18

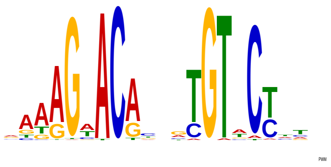	$NNAAA\overrightarrow{AGAACN}NNN\overleftarrow{TGTTCT}TNN$	153987.49	IR:3	19
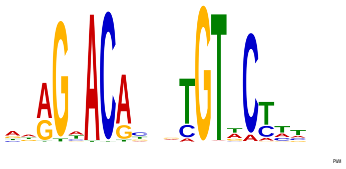	$NNA\overrightarrow{AGAACA}CNN\overleftarrow{NGTACT}TTNN$	166410.43	IR:3	20

11 AR-SelexGLM-Rnd6 (SRR5340727)

PWM	Seed Sequence	Seed Seq Enrichment	Repeat	Rank
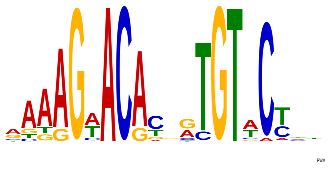	NNNAAAGAACANNNTGTACNNN	210416.26	IR:3	1
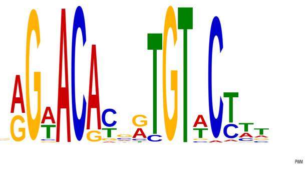	NNNNGGAACACNNTGTACTNNNN	150409.54	IR:3	2
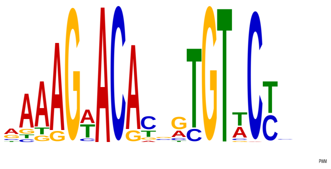	NNNAAAGAACANNNNGTTCCNNN	109100.33	DR:3	3
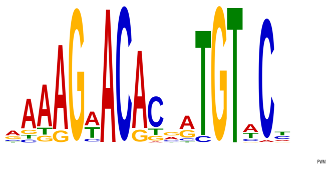	NNNAAAGAACACAATGTACNNN	920189.34	DR:2	4
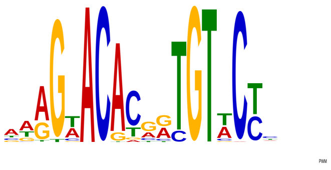	NNNNAGTACACGATGTTCCNNNN	378444.24	IR:0	5
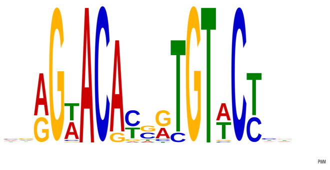	NNNNGGAACACNGTGTTCCNNNN	141253.68	DR:3	6
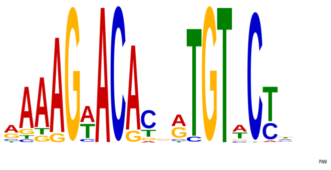	NNNAAAGAACANNNTGTCCTNNN	118757.98	IR:3	7
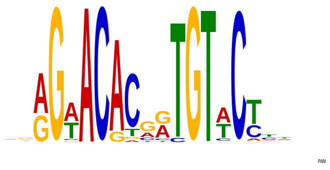	NNNNGGAACACGATGTACCNNNN	221727.85	IR:0	8
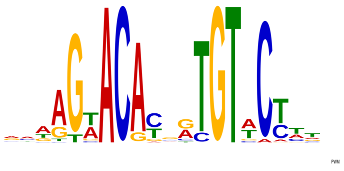	NNNTAGTACACNNTGTACTNNN	223967.73	IR:3	9

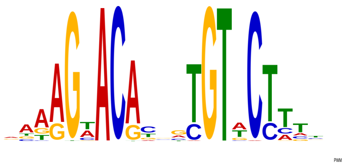	NNAAAGAACANNNNGTTCTTTNN	465774.01	DR:3	10
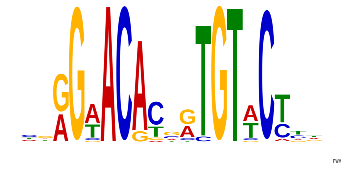	NNNGGGAACACNNTGTACCNNN	114282.07	IR:5	11
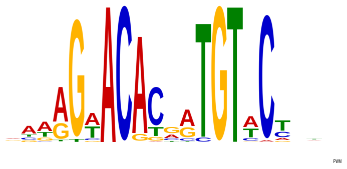	NNNNAGTACACAATGTACNNNN	153881.87	DR:2	12
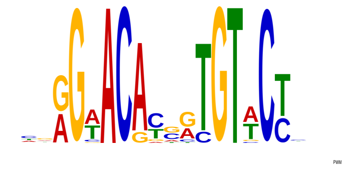	NNNGGGAACACNGTGTTCCNNN	215401.14	DR:5	13
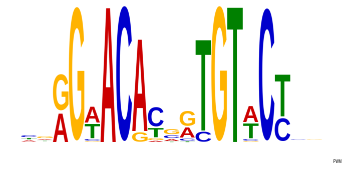	NNNGGGAACACNNTGTTCCNNN	105700.20	DR:5	14
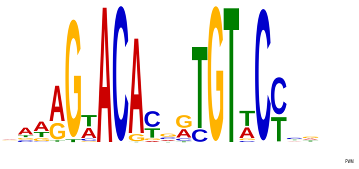	NNNAGTACACNNNGTTCCCNNN	69451.22	DR:3	15
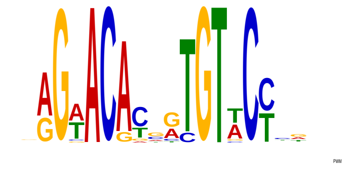	NNNGGAACACNNTGTTCCCNNN	104167.00	DR:3	16
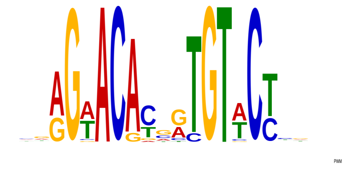	NNNNGGAACACNNTGTTCCNNNN	67635.87	DR:3	17
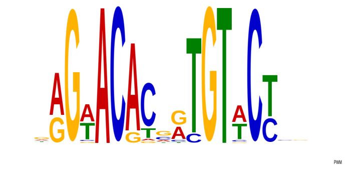	NNNNGGAACACGNTGTTCCNNNN	120423.25	DR:3	18

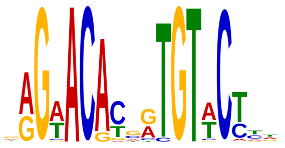	$NNNN\overrightarrow{GGAACA}CNN\overleftarrow{TGTACC}NNNNN$	73559.63	IR:3	19

12 AR-SelexGLM-Rnd7 (SRR5340726)

PWM	Seed Sequence	Seed Seq Enrichment	Repeat	Rank
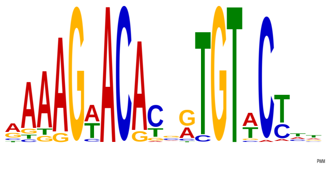	NNNAAAGAACANNNTGTACNNN	381818.79	IR:3	1
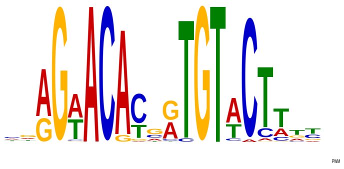	NNNGGAACACNNTGTACTANNN	191087.41	IR:3	2
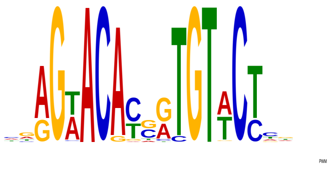	NNNNGGAACACNGTGTTCCNNNN	143755.91	DR:3	3
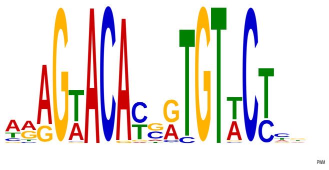	NNNAGGTACACNGTGTTCCNNN	253321.46	DR:4	4
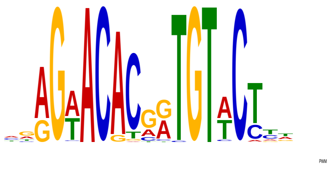	NNNNGGAACACGATGTACCNNNN	246817.81	IR:0	5
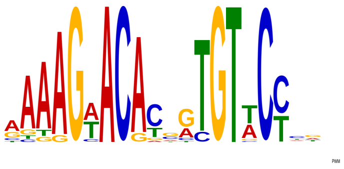	NNAAAGAACANNNNGTTCCCNN	281590.12	DR:3	6
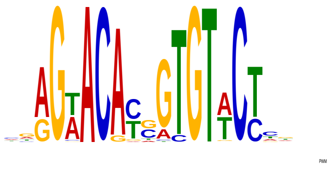	NNNNGGAACACCGTGTTCCNNNN	254538.50	DR:3	7
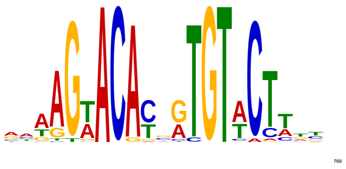	NNNTAGTACANNNTGTACTANNN	185946.90	IR:3	8
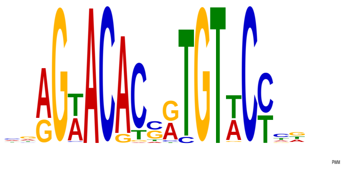	NNNGGAACACGGTGTTCCCNNN	426300.54	DR:0	9

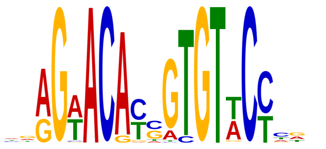 PWM	NNNGGAACACCGTGTTCCCNNN	450992.50	DR:3	10
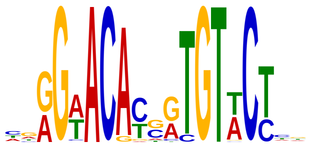 PWM	NNNGGGAACACNGTGTTCCNNN	247617.56	DR:5	11
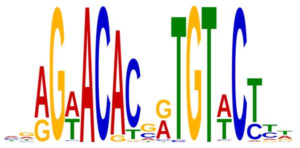 PWM	NNNNGGAACACGNTGTACCNNNN	136380.80	IR:3	12
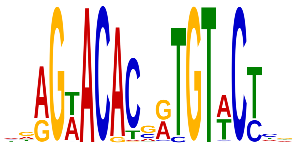 PWM	NNNNGGAACACGNTGTTCCNNNN	122931.29	DR:3	13
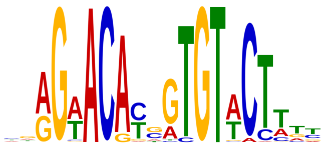 PWM	NNNGGAACACCGTGTACTANNN	589927.15	IR:3	14
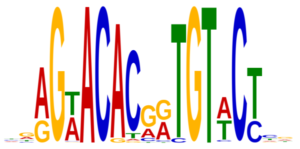 PWM	NNNNGGAACACGATGTTCCNNNN	221756.84	IR:0	15
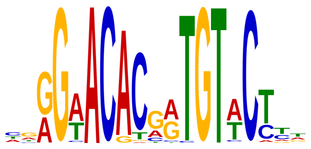 PWM	NNNGGGAACACGATGTACCNNN	463085.93	IR:2	16
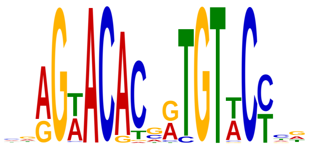 PWM	NNNGGAACACGNTGTTCCCNNN	201862.14	DR:3	17
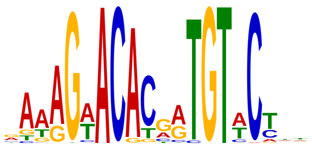 PWM	NNNNAAGTACACAATGTACNNNN	445814.93	DR:2	18

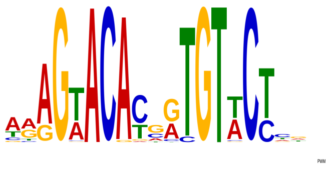	$NNN\overrightarrow{AGGTAC}ACNN\overrightarrow{TGTTCC}NNN$	115096.48	DR:4	19
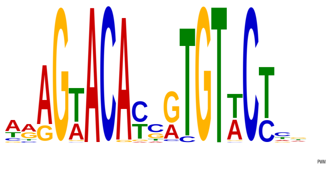	$NNN\overrightarrow{AGGTAC}ANNG\overrightarrow{TGTTCC}NNN$	122515.46	DR:4	20

13 AR-SelexGLM-Rnd8 (SRR5340725)

PWM	Seed Sequence	Seed Seq Enrichment	Repeat	Rank
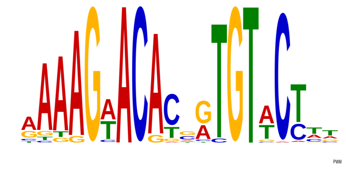	NNNAAAGAACANNNTGTACNNN	616373.31	IR:3	1
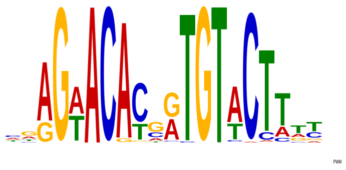	NNNGGAACACNNTGTACTANNN	191736.16	IR:3	2
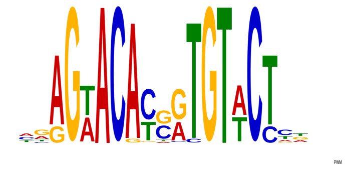	NNNNGGAACACNGTGTTCCNNNN	110489.91	DR:3	3
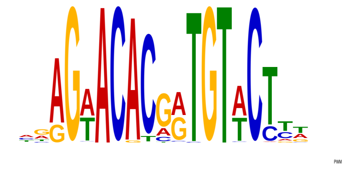	NNNNGGAACACGATGTACCNNNN	245493.58	IR:0	4
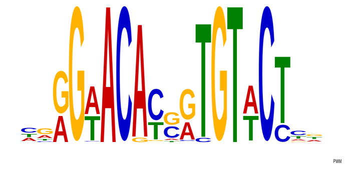	NNNGGGAACACNGTGTTCCNNN	211644.81	DR:5	5
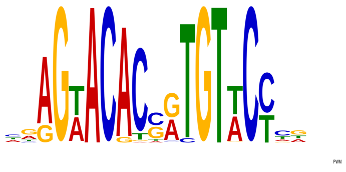	NNNGGAACACGGTGTTCCCNNN	382674.37	DR:0	6
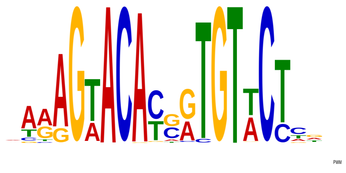	NNNAGGTACACNGTGTTCCNNN	257387.10	DR:4	7
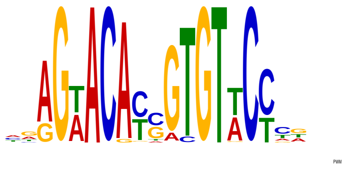	NNNGGAACACCGTGTTCCCNNN	418116.61	DR:3	8
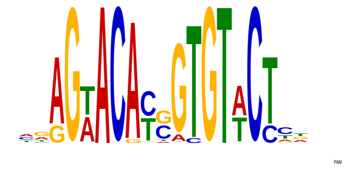	NNNNGGAACACCGTGTTCCNNNN	212501.02	DR:3	9

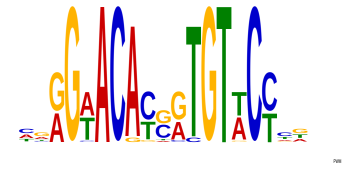	NNNGGGAACACNGTGTTCCCNNN	372039.61	DR:5	10
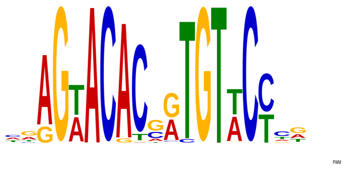	NNNGGAACACGNTGTTCCCNNN	176027.80	DR:3	11
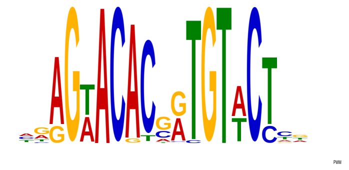	NNNNGGAACACGNTGTTCCNNNN	100532.07	DR:3	12
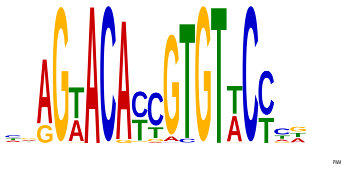	NNNGGAACATCGTGTTCCCNNN	336025.10	DR:3	13
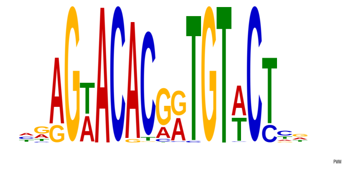	NNNNGGAACACGATGTTCCNNNN	191268.85	IR:0	14
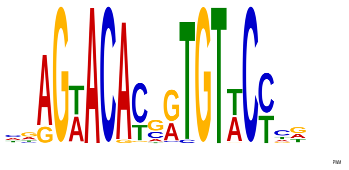	NNNGGAACACNNTGTTCCCNNN	86268.69	DR:3	15
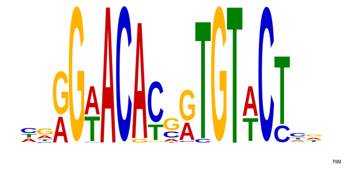	NNNGGGAACACNNTGTTCCNNN	87703.94	DR:5	16
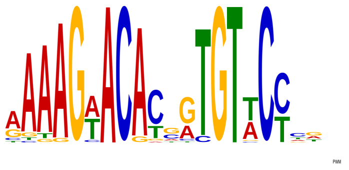	NNAAAGAACANNNNGTTCCCNN	420220.68	DR:3	17
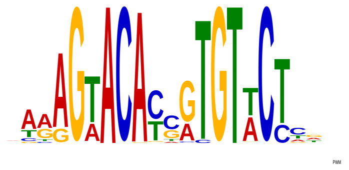	NNNAGGTACANNGTGTTCCNNN	116281.44	DR:4	18

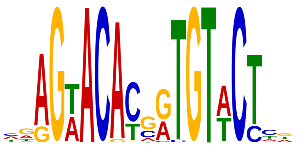	$NNNN\overrightarrow{GGAACA}CNN\overrightarrow{TGTTCC}NNNN$	46347.14	DR:3	19
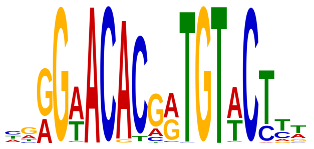	$NN\overrightarrow{NGGGAA}CA\overleftarrow{CGATGT}ACCNNN$	497121.73	IR:2	20

14 MR-1-Bhimsaria2022-Rnd2 (S07-005)

PWM	Seed Sequence	Seed Seq Enrichment	Repeat	Rank
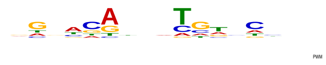	NNNGTACNNNNNGTACNNN	3.31	M	1
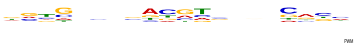	NNNNNNTGACGTCANNNNNN	2.22	M	2
	GGGGNNNNNNNNNNNGGGG	1.95	M	3
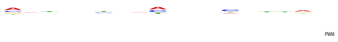	CAATNNNNNNNNNNNTTAT	1.84	M	4
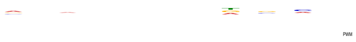	ACTANNNNNNNNNNNACGT	1.82	M	5
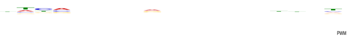	TTCANNNNNNNNNNNTTCT	1.77	M	6
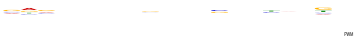	GAGTNNNNNNNNNNNACGG	1.82	M	7
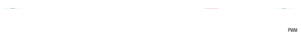	CAATNNNNNNNNNNNATGT	1.71	M	8
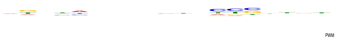	CAGGNNNNNNNNNNNTTGT	1.85	M	9

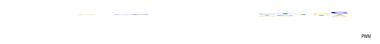	*ATTGNNNNNNNNNNNTGCT*	1.80	M	10
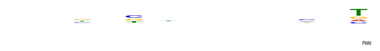	$NATGGNNNNNNNNNN\overrightarrow{ACGTN}$	1.71	M	11
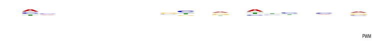	*NCAGTNNNNNNNNNNCTCTN*	1.85	M	12
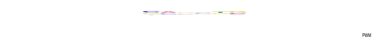	*NAACANNNNNNNNNNNTGTTN*	1.65	M	13
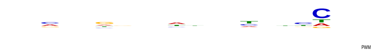	*NNNNGTACNNNGCGTNNNN*	1.90	M	14
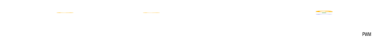	$\overrightarrow{GGGTN}NNNNNNNNNNNCGGG$	1.82	M	15
	NAGTGNNNNNNNNNTTGTN	1.58	M	16
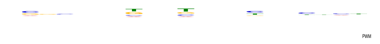	*CGCANNNNNNNNNNNNCTCT*	1.88	M	17
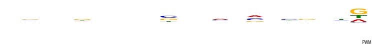	*GTAGNNNNNNNNNNNNGATG*	1.58	M	18

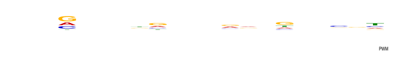	*AGCCNNNNNNNNNNNGCGT*	1.65	M	19
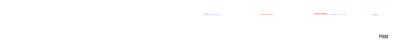	*CGCGNNNNNNNNNNN*$\overrightarrow{ACGT}$	1.75	M	20

15 MR-1-Bhimsaria2022-Rnd3 (S07-126)

PWM	Seed Sequence	Seed Seq Enrichment	Repeat	Rank
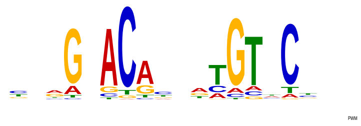	NNNGGACANNNTGTACNNN	63.67	IR:3	1
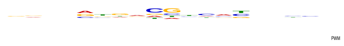	NNNNNNTGACGTCANNNNNN	4.24	M	2
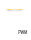	GGGGNNNNNNNNNNNNNGGGG	5.01	M	3
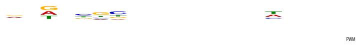	ATGTNNNNNNNNNNNNNTGTA	3.58	M	4
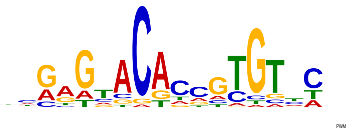	NNNAGTACNCCGTGTNNN	53.78	M	5
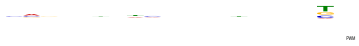	CAGGNNNNNNNNNNNNNCTG	4.29	M	6
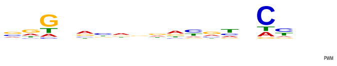	NNNNNACAGNACGTNNNNN	4.88	M	7

16 MR-Bhimsaria2022-Rnd2 (S07-046)

PWM	Seed Sequence	Seed Seq Enrichment	Repeat	Rank
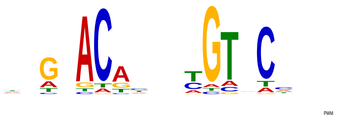	NNNGGACANNNTGTACNNN	29.25	IR:3	1
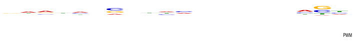	GAATNNNNNNNNNNNNGAGT	2.17	M	2
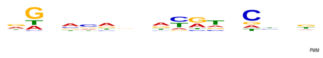	NNNNNACAGNACGTNNNNN	3.06	M	3
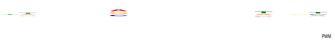	ATTTNNNNNNNNNNTCGT	2.00	M	4
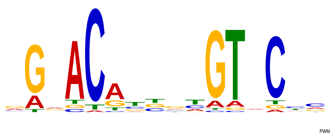	NGGGACANNNNGTACTN	28.06	IR:3	5
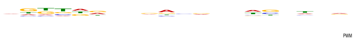	NNNNNNAGAACATTNNNNNN	2.00	M	6
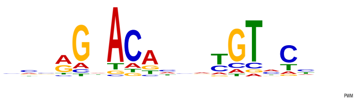	NNNAGAACNNNNNGTCCNNN	11.83	DR:2	7
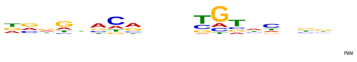	NNNGTACNNNNNNTACGNNN	2.70	M	8
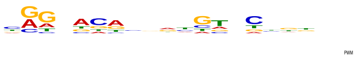	NNNNNCACGNNGTCCNNNNN	2.94	M	9

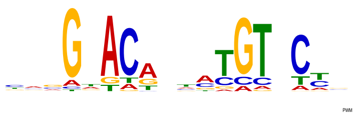	*NNCGTACANNNTGTACNN*	31.33	IR:3	10
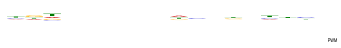	*ATGTNNNNNNNNNNNTTCG*	2.08	M	11
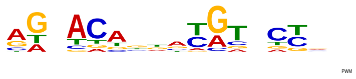	*NNNACAGNACGTTCNNN*	8.27	M	12
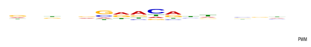	*NNNNNNCAGAACATNNNNNN*	2.82	M	13
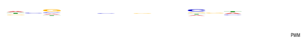	*NNNNNGTACNGTACNNNNN*	1.99	M	14
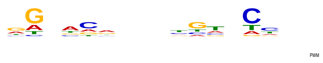	*NNNNGACANNNTGTCNNNN*	2.86	DR:2	15
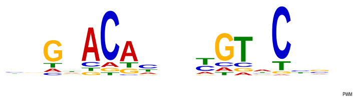	*NNGGACANNNNGTACGNN*	11.42	M	16
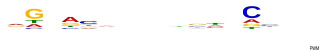	*NNNNCACANNNTGTCNNNN*	2.32	M	17
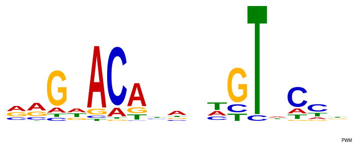	*NNGGACAANNTGTGCNN*	13.86	IR:3	18

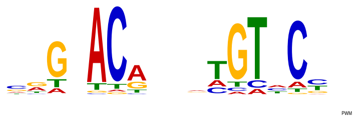	$NN\overrightarrow{GCACA}TNN\overleftarrow{TGTCC}NN$	21.26	IR:3	19
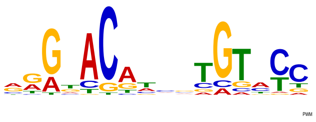	$NNN\overrightarrow{GGGACA}NCGTGTNNN$	15.08	M	20

17 MR-Bhimsaria2022-Rnd3 (S07-167)

PWM	Seed Sequence	Seed Seq Enrichment	Repeat	Rank
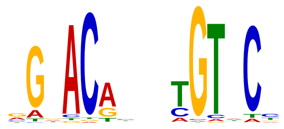	$NNN\overrightarrow{GGACA}NNN\overleftarrow{TGTAC}NNN$	213.60	IR:3	1
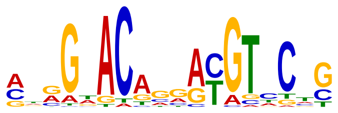	$NNN\overrightarrow{GTACA}GN\overrightarrow{ACGTC}CNNN$	299.80	DR:2	2

18 MR-Jolma2013-Rnd2 (ERR188787)

PWM	Seed Sequence	Seed Seq Enrichment	Repeat	Rank
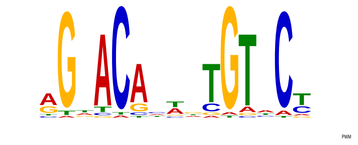	*NNNGTACANNNTGTACNNN*	33.49	IR:3	1
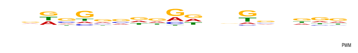	*NNNGGGGNNNNNGGGGNNN*	5.89	M	2
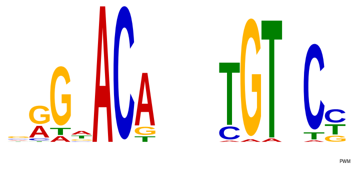	*NGGAACANNNTGTTCN*	51.28	DR:3	3
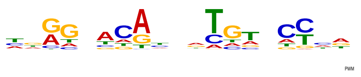	*NNNGAACNNNNNGTTCNNN*	4.02	ER:3	4
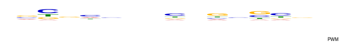	*NGCGCNNNNNNCGCGN*	2.34	M	5
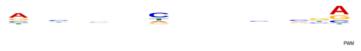	*NNNGCGCNNNCGCGNNN*	1.54	M	6
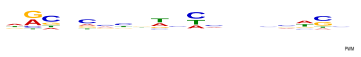	*NNNCCCCNNNNNCTCANNN*	2.40	M	7
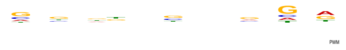	*NGCGGNNNNNNNNGTGTN*	2.43	M	8
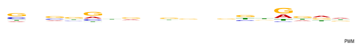	*NTGGGNNNNNNNNTGGAN*	2.41	M	9

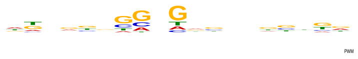	$NNTGGGNNNNNNN\overrightarrow{AGGTNN}$	2.17	M	10
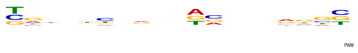	$NGTGTNNNNNNNNNT\overrightarrow{AGGN}$	2.13	M	11
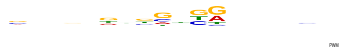	$NNTGGGNNNNNNNAGTGNN$	1.74	M	12
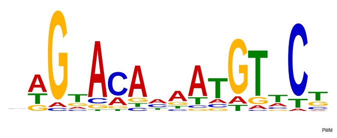	$NNNGTACNAA\overrightarrow{ATGTNN}N$	11.22	M	13
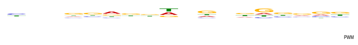	$NT\overrightarrow{AGGNNN}NNNNNGGGGN$	2.84	M	14
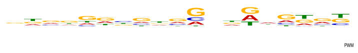	$NTGGGNNNNNNNNNAGTGN$	2.45	M	15
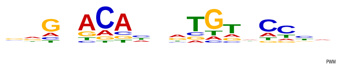	$NNNGTACNNNNN\overrightarrow{NGTTCN}NN$	4.85	M	16
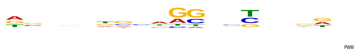	$NGGATNNNNNNNNNN\overrightarrow{GGGTN}$	1.66	M	17
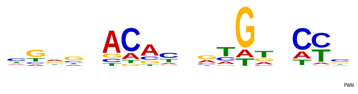	$NN\overrightarrow{AGTACN}NNNN\overrightarrow{NGTTCN}N$	5.55	DR:3	18

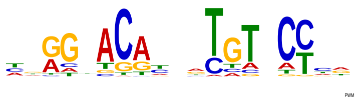	$NN\overrightarrow{NGAACA}NNN\overrightarrow{NGTTCN}NN$	8.90	DR:3	19
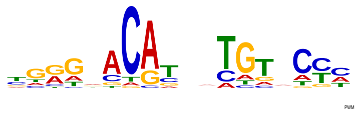	$NN\overleftarrow{GGAACN}NNNN\overrightarrow{NGTTCN}N$	7.35	ER:3	20

19 MR-Jolma2013-Rnd3 (ERR188788)

PWM	Seed Sequence	Seed Seq Enrichment	Repeat	Rank
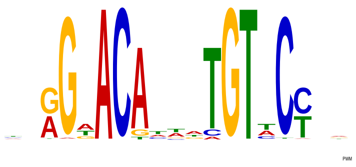	NNNGAACANNNTGTTCNNN	1775.68	DR:3	1
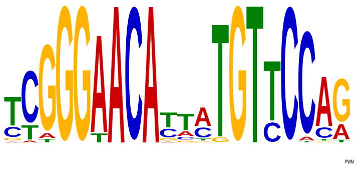	NCGGGAACANNNNGTTCCAN	25362.91	DR:5	2
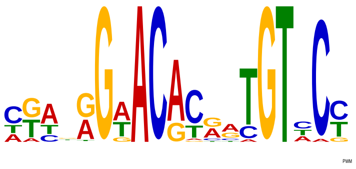	NNNGGGTACACGATGTNNN	3020.82	DR:2	3
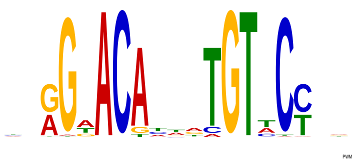	NNNGAACNNNNNGTTCNNN	254.07	ER:3	4
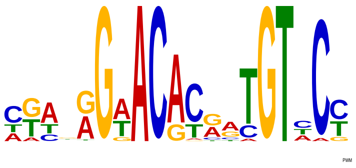	NNNGGGTNCACGATGTNNN	1301.33	DR:2	5
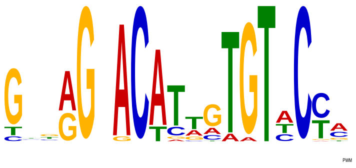	NCTAGGACANNNNGTTCCN	2465.37	DR:3	6
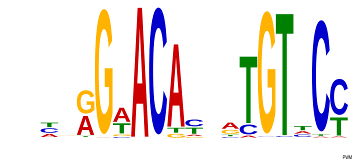	NNNGGGTACNNNGTGTNNN	308.52	M	7
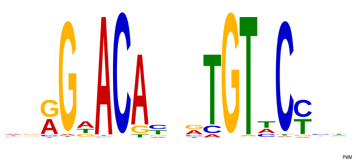	NNNGTACNNNNNGTACNNN	127.48	M	8
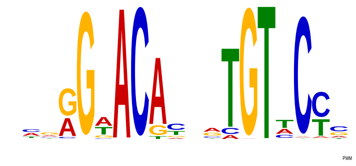	NNGGGTACNNNNNGTACNN	365.66	M	9

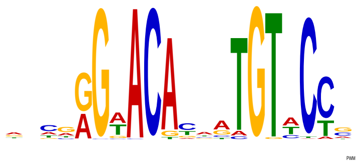	$NN\overrightarrow{NGGGTA}CNNNNTGTANNN$	304.68	M	10
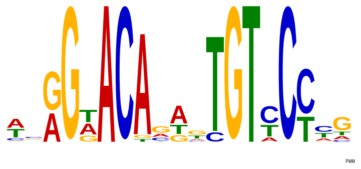	$\overrightarrow{NGGGTA}CANNN\overrightarrow{NGTTCT}AN$	1962.49	DR:5	11
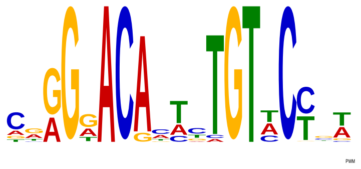	$NT\overrightarrow{AGAACA}NNNN\overleftarrow{GTACCC}N$	1618.93	IR:4	12

20 MR-Jolma2013-Rnd4 (ERR188789)

PWM	Seed Sequence	Seed Seq Enrichment	Repeat	Rank
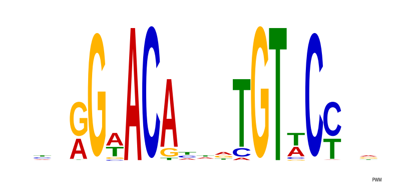	*NNGGAANNNNNNNTTCCNN*	79.66	M	1
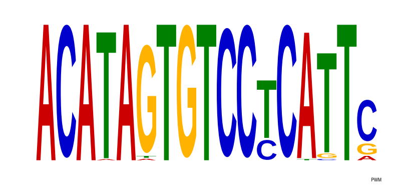	*NCATAGNN*$\overrightarrow{NCCTCA}$*TTN*	470.62	M	2
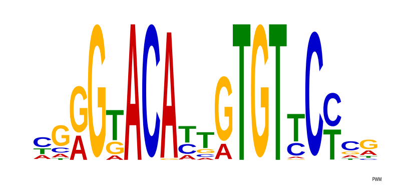	*CGGGNNNNNNNNNNNCTCG*	29.38	M	3
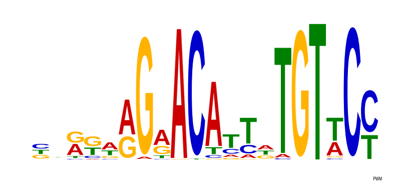	*NNNNTAGANNNNTTTGNNNN*	32.37	M	4
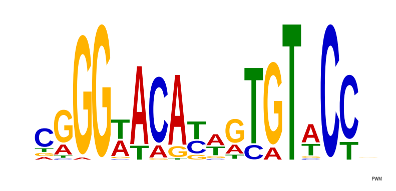	$\overrightarrow{CGGGTN}$*NNNNNNNTACTG*	46.74	M	5
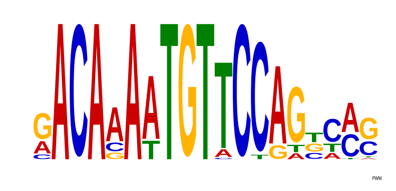	*NNCAAANNNNTC*$\overrightarrow{CAGTCN}$*N*	90.95	M	6
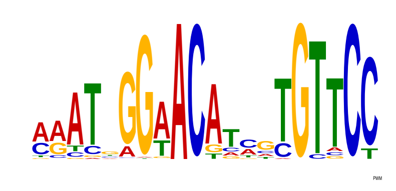	*NNNNCGGANNNNCGTGNNNN*	24.32	M	7
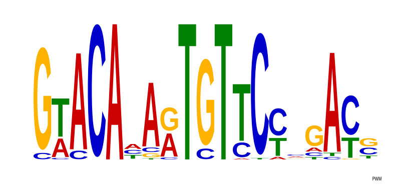	*NNNCAAANNNNTCTGGNNN*	21.08	M	8
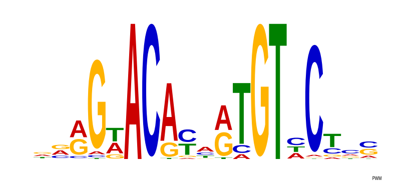	*N*$\overrightarrow{GGGTN}$*NNNNNNNNACTAN*	8.42	M	9

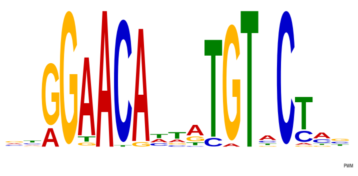	*NTGGANNNNNNNNNTACTAN*	42.36	M	10
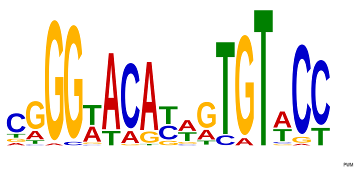	$C\overrightarrow{GGGTN}NNNNNNNNACTG$	19.73	M	11
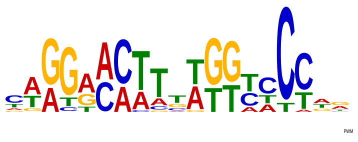	$C\overrightarrow{AGGAN}NNNNNN\overleftarrow{NNNCCT}G$	16.12	IR:5	12
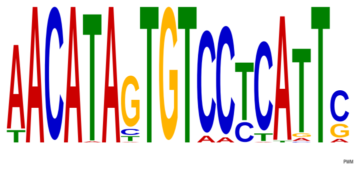	$NNCATAGNN\overrightarrow{NCCTCA}TNN$	147.15	M	13
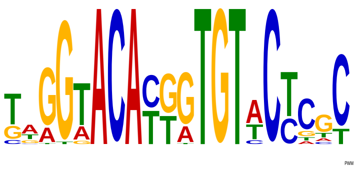	$N\overrightarrow{TGGTN}NNNNNNNNNNCTCGN$	9.33	M	14
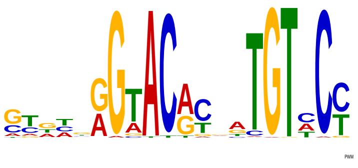	$NNNN\overrightarrow{GGGTN}NNNGATGNNNN$	43.79	M	15
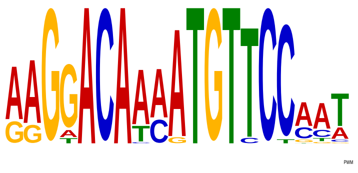	$A\overrightarrow{AGGACN}NNNNNNNNCCAAT$	85.72	M	16
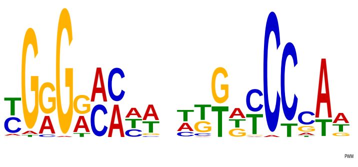	*CGGGNNNNNNNNNNNCCCAA*	43.29	M	17
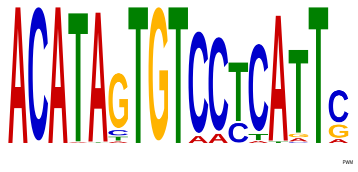	$NNATAGNN\overrightarrow{NCCTCA}TNN$	49.33	M	18

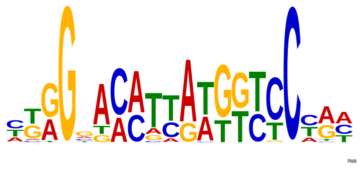	$NT\overrightarrow{AGGANN}NNNNNNNCCCGN$	26.62	M	19
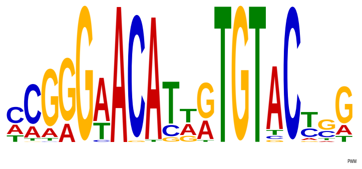	$NCGGGNNNNNNNNNNACTGN$	22.73	M	20

21 MR-Yin2017-Rnd4 (ERR1005249)

PWM	Seed Sequence	Seed Seq Enrichment	Repeat	Rank
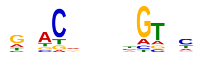 PWM	NNNNNNGAACNNNNNGTCCNNNNNN	8.20	ER:2	1
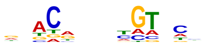 PWM	NNNNNNTACANNNNNTTCCNNNNNN	5.39	M	2
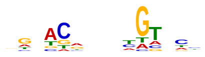 PWM	NNNNNNGGAANNNNNNNTACTNNNNNN	4.54	M	3
PWM	NNNNNNAGAANNTTCTNNNNNN	2.23	M	4
PWM	NNNNNNCGTANNNTACGNNNNNN	2.63	M	5
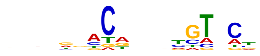 PWM	NNNNNNGACAGNNNNTACTNNNNNN	7.15	M	6
PWM	NNNNNNGGAANNNACTGNNNNNN	3.09	M	7
PWM	NNNNNNCAGANNNTTCTNNNNNN	3.27	M	8
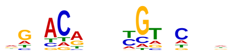 PWM	NNNNNNAGGANNNNNNNTTCTNNNNNN	4.36	M	9

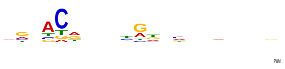	*NNNNNNTGGANNNNNNNNTTCCNNNNNN*	2.67	M	10
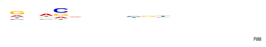	*NNNNNNCGTANNNNCCGTNNNNNN*	2.63	M	11
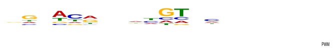	$NNNNNNC\overrightarrow{AGGNNN}TTCCNNNNNN$	2.79	M	12
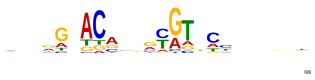	$NNNNNN\overrightarrow{AGGTAN}NNNNNNTTCCNNNNNN$	6.59	M	13
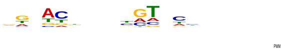	*NNNNNNGGAANNNNNNNTTCTNNNNNN*	4.14	M	14
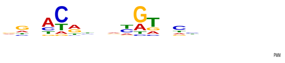	$NNNNNN\overrightarrow{AGGANN}NNN\overleftarrow{NNTCCT}NNNNNN$	2.95	IR:3	15

22 PGR-Bhimsaria2022-Rnd2 (S07-047)

PWM	Seed Sequence	Seed Seq Enrichment	Repeat	Rank
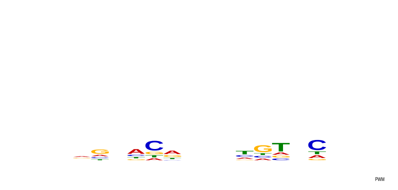	NNNGGACNNNNNGTACNNN	4.61	M	1
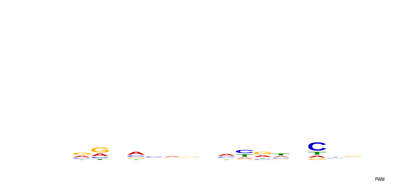	NNNNNACAGNACGTNNNNN	3.27	M	2
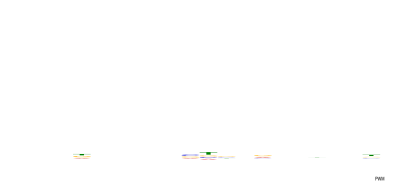	ATTTNNNNNNNNNNNTCTT	1.82	M	3
	TCCCNNNNNNNNNNNNCCCC	1.95	M	4
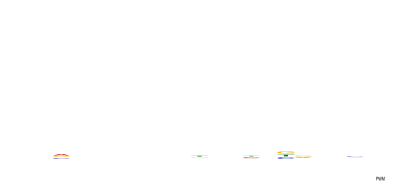	CACANNNNNNNNNNNNTACT	1.92	M	5
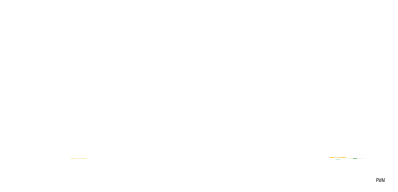	GAGANNNNNNNNNNNNTGTG	2.09	M	6
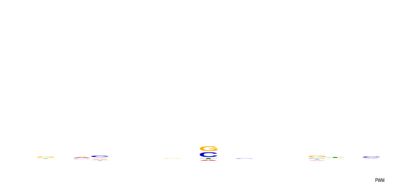	GTACNNNNNNNNNNNGTAC	2.66	M	7
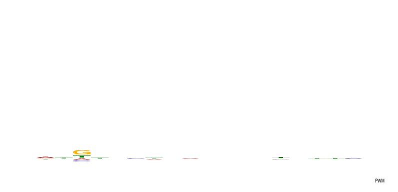	ATGTNNNNNNNNNNNTTCC	2.00	M	8
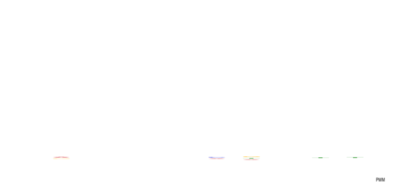	CACGNNNNNNNNNNNNTGTG	1.70	M	9

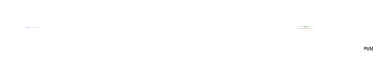	*CAGGNNNNNNNNNNNNTACT*	1.80	M	10
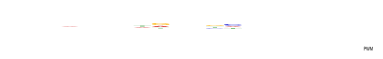	*TCATNNNNNNNNNNNNTTCT*	1.38	M	11
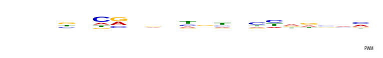	*NNNNNNCGATGTCCNNNNNN*	2.21	M	12
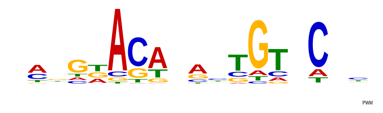	*NNGGACAANNTGTGCNN*	12.78	IR:3	13
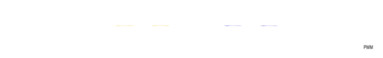	*NGTACNNNNNNNNNGTACN*	1.73	M	14
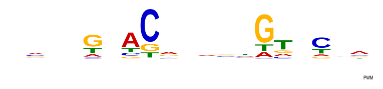	*NNAGTACANNNNGTCCNN*	9.18	DR:2	15
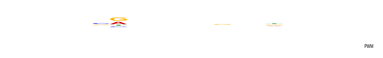	*TACTNNNNNNNNNNNNTCCC*	1.54	M	16
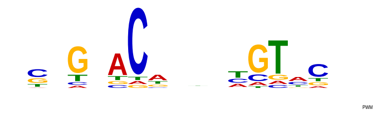	*NNGCACATNNTGTCCNN*	10.94	IR:3	17
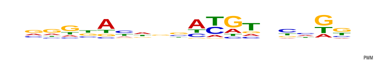	*NNNNACAGNACGTCCNNNNN*	6.34	M	18

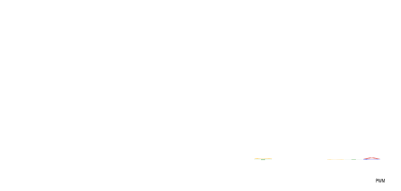	$NAGAANNNNNNNNN\overrightarrow{ATGTN}$	1.80	M	19
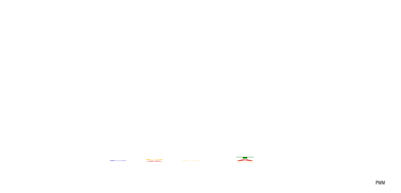	*AGTANNNNNNNNNNNCATT*	1.72	M	20

23 PGR-Bhimsaria2022-Rnd3 (S07-168)

PWM	Seed Sequence	Seed Seq Enrichment	Repeat	Rank
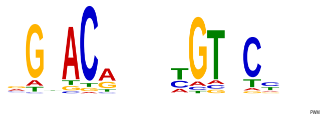	NNNGTACANNNNNGTCCNNN	70.42	DR:2	1
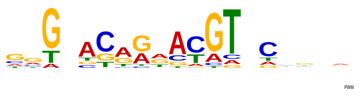	NNNNACAGGACGTCCNNNN	97.75	M	2
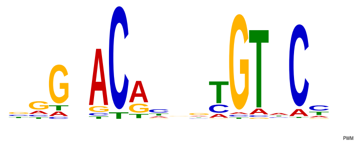	NNGGACACNGTGTACNN	260.65	IR:3	3
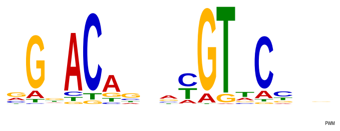	NNGGACANNACGTTCCNN	289.37	DR:3	4
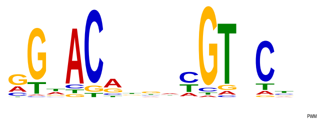	NGGGAACNNNNNGTCCTN	104.95	IR:5	5

24 PGR-2-Bhimsaria2022-Rnd2 (S07-006)

PWM	Seed Sequence	Seed Seq Enrichment	Repeat	Rank
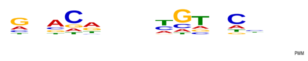	NNNGGACNNNNNGTACNNN	5.28	M	1
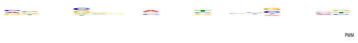	NNNNNNGGACGTCCNNNNNN	2.54	M	2
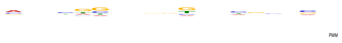	CACTNNNNNNNNNNNNCACT	1.91	M	3
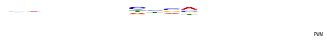	CACANNNNNNNNNNNNCCAT	1.86	M	4
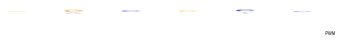	NNNNGTACNNGTACNNNN	2.22	M	5
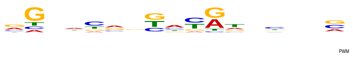	NNNACAGNACGTCCNNN	6.52	M	6
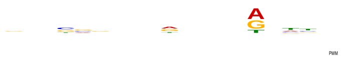	NNNNNNGGACACGGNNNNNN	1.85	M	7
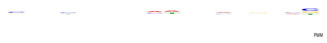	CACCNNNNNNNNNNNNGTCC	1.98	ER:9	8
	TCCCNNNNNNNNNNNNCCCC	2.02	M	9

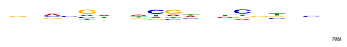	NGGACNNNNNNNNNNGTCCN	2.45	DR:7	10
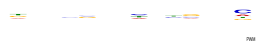	TCCCNNNNNNNNNNNNGTAC	2.04	M	11
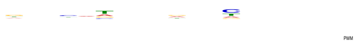	GTACNNNNNNNNNNNTGTG	2.09	M	12
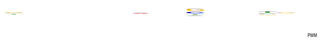	AGGCNNNNNNNNNNNCTGT	1.89	M	13
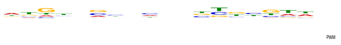	NATGTNNNNNNNNNNCGTTN	2.28	M	14
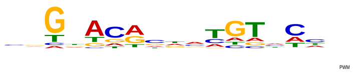	NCGTACNNNNNGTCCCN	6.46	M	15
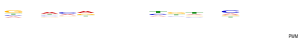	NNNNGACANNNTGTCNNNN	2.51	DR:2	16
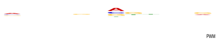	CGGGNNNNNNNNNNNNTGCG	1.81	M	17
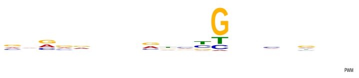	NNNGGACNNNNNNTGCANNN	1.88	M	18

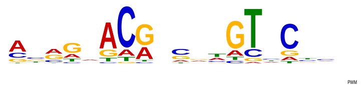	$NN\overrightarrow{AGAAC}NNNNN\overleftarrow{GTCCT}NN$	11.52	IR:3	19
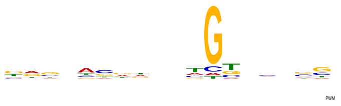	$NN\overrightarrow{AGGAC}NNNNNN\overleftarrow{TGCC}NN$	2.88	IR:5	20

25 PGR-2-Bhimsaria2022-Rnd3 (S07-127)

PWM	Seed Sequence	Seed Seq Enrichment	Repeat	Rank
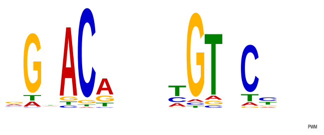	NNNGTACANNNNGTCCNNN	122.15	DR:2	1
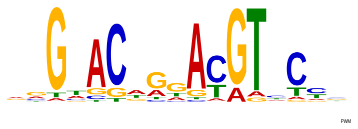	NNGTACNGGACGTCCNN	406.93	M	2
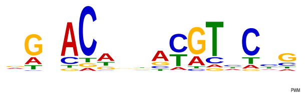	NNNNGGACAGNACGTNNNN	51.02	IR:0	3
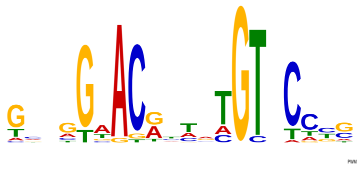	NNNGGAACGNNATGTCCNNN	394.55	ER:2	4
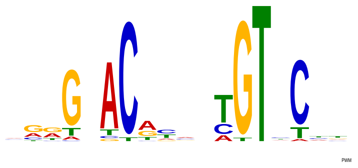	NNAGTACGCNNTGTCCNN	234.89	IR:3	5
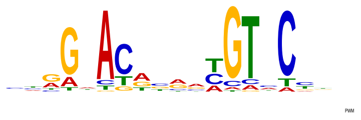	NNAGAACGNNNNGTCCTNN	119.53	IR:3	6

26 GR-Bhimsaria2022-Rnd2 (S07-045)

PWM	Seed Sequence	Seed Seq Enrichment	Repeat	Rank
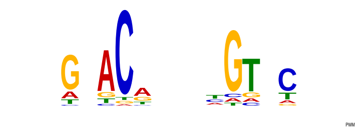	NNNGTACANNNNGTCCNNN	74.86	DR:2	1
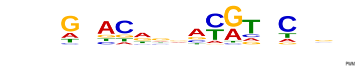	NNNNNACAGNACGTNNNNN	18.86	M	2
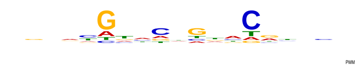	NNNNNGTACNGTACNNNNN	11.40	M	3
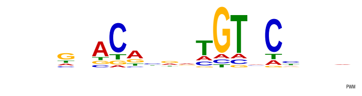	NNNNNNCACGATGTNNNNNN	12.62	M	4
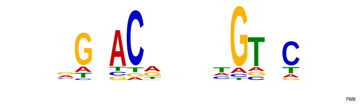	NNNAGAACNNNNNGTCCNNN	45.85	DR:2	5
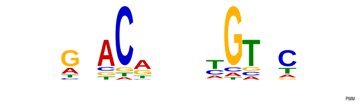	NNNNGACANNNTGTCNNNN	16.57	DR:2	6
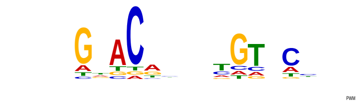	NNNNGTACNNNNTGTGNNNN	19.12	M	7
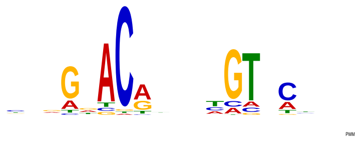	NNNGGACATNNNGTGCNNN	60.73	M	8
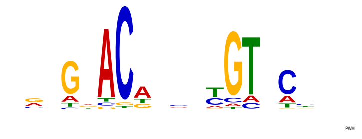	NNNGGACAANNTGTGCNNN	72.98	IR:3	9

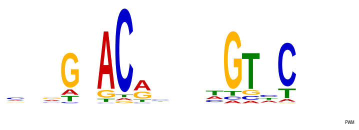	$NN\overrightarrow{AGCACA}NNNN\overleftarrow{NGTCCT}NN$	76.39	IR:3	10
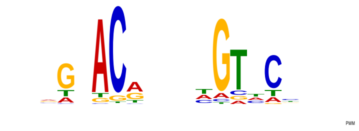	$NNN\overrightarrow{GCACA}NNNN\overrightarrow{GTTCC}NNN$	64.82	DR:3	11
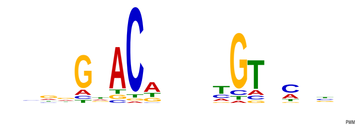	$NNN\overrightarrow{NGTACA}NNGTGTGNNNN$	41.86	M	12
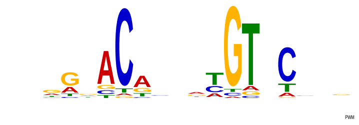	$NNN\overrightarrow{AGACA}NNN\overleftarrow{TGTCC}NNN$	40.04	IR:3	13
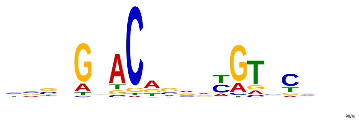	$NNN\overrightarrow{NGTACA}NN\overrightarrow{ACGTCN}NNN$	51.97	DR:2	14
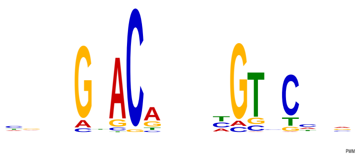	$NNN\overrightarrow{AGTACA}NN\overrightarrow{NCGTCC}NNN$	119.32	DR:2	15
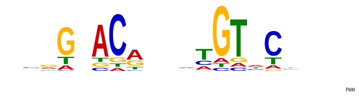	$NNNNCACANNN\overrightarrow{NGTCC}NNNN$	14.55	M	16
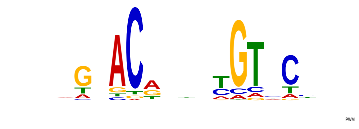	$NNN\overrightarrow{NGCACA}NN\overrightarrow{NTGTCN}NNN$	33.07	DR:2	17
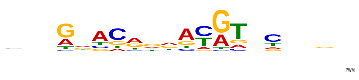	$NNNNNACAGN\overrightarrow{ACGTCN}NNNN$	22.54	M	18

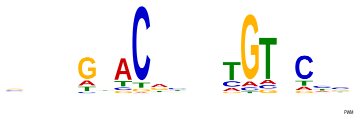	$NNNNGTACNCG\overrightarrow{ATGTN}NNN$	43.89	M	19
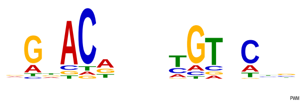	$NNN\overrightarrow{AGGAC}NNNNTGTGNNN$	17.75	M	20

27 GR-Bhimsaria2022-Rnd3 (S07-166)

PWM	Seed Sequence	Seed Seq Enrichment	Repeat	Rank
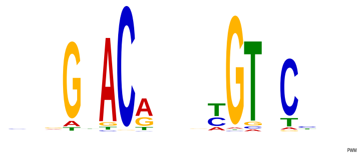	$NNN\overrightarrow{GTACA}NNN\overrightarrow{NNGTCC}NNN$	1203.72	DR:2	1
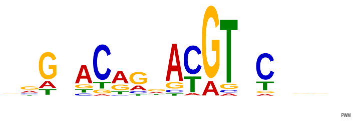	$NNNNAC\overrightarrow{AGGACG}TCCNNNNN$	1389.59	M	2

28 GR-Jolma2013-Rnd2 (ERR194205)

PWM	Seed Sequence	Seed Seq Enrichment	Repeat	Rank
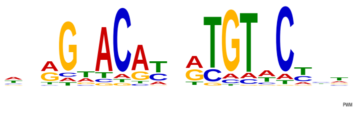	NNNGTACANNNNGTACNNN	16.93	M	1
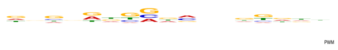	NGGGGNNNNNNNNGGGTN	2.89	M	2
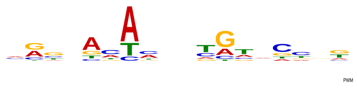	NNGGACNNNNNNTACCNN	2.47	IR:4	3
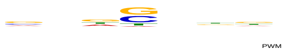	CGGGGCGG	1.83	M	4
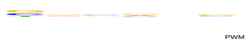	ACGGCGAC	1.56	M	5
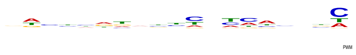	NNCTCANNNNNNNNCACANN	2.18	M	6
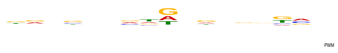	NNGGTTNNNNNNGTGGNN	1.72	M	7
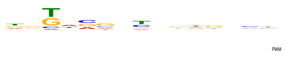	NNNTTGTNNNNNGGTGNNN	1.81	M	8
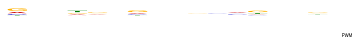	NGTTGNNNNNNNGTTGN	1.79	M	9

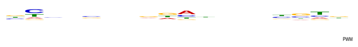	$NNN\overrightarrow{GGGTNN}NNNGATNNN$	1.80	M	10
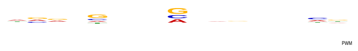	$NN\overrightarrow{GGGTNN}NNNGATANN$	1.76	M	11
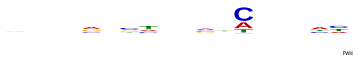	$\overrightarrow{NATCA}NNNNNNNNCCAAN$	1.56	M	12
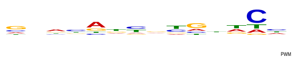	NNNGTACNNNGCGTNNN	2.50	M	13
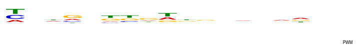	$NNT\overrightarrow{GGTNN}NNNNAGAANN$	1.71	M	14
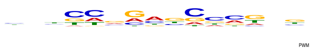	NNNNTCCGNAGCCNNNN	2.34	M	15
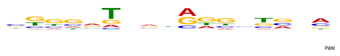	NNNGGATNNNNGGCTNNN	2.08	M	16
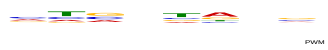	$\overrightarrow{ATGATA}CG$	1.88	M	17
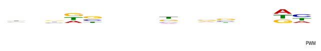	NNTTGGNNNNNNGGGANN	1.82	M	18

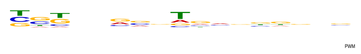	$NNNTCGGNNN\overrightarrow{AGGGN}N$	1.84	M	19
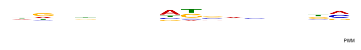	$N\overleftarrow{NNTCCT}\,\overrightarrow{NNGACA}NNN$	1.55	ER:0	20

29 GR-Jolma2013-Rnd3 (ERR194206)

PWM	Seed Sequence	Seed Seq Enrichment	Repeat	Rank
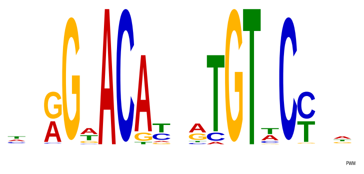	NNGGAACANNNTGTTCCNN	2212.65	DR:3	1
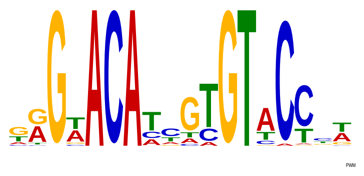	NNGTACATCGNGTACCNN	2270.13	IR:4	2
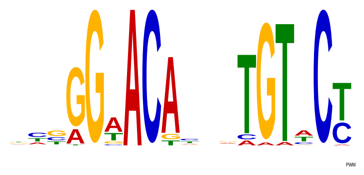	NCGGGAACANNNTGTACN	2196.63	IR:5	3
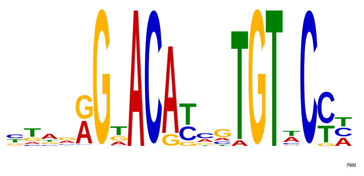	NNAAAGTACANNNTGTACNN	1734.08	IR:3	4
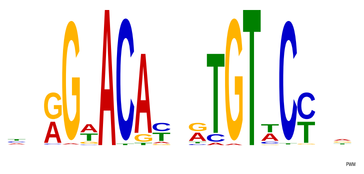	NNGGAACNNNNNGTTCCNN	319.89	ER:3	5
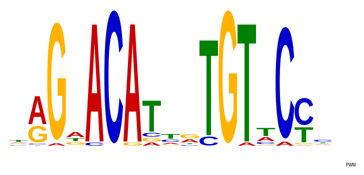	NNGGACATTNNGTTCCNN	610.69	DR:3	6
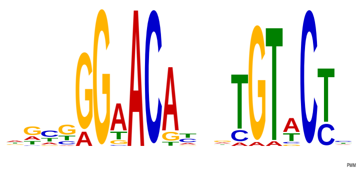	NNCGGGAACNNNNNGTACNN	402.64	M	7
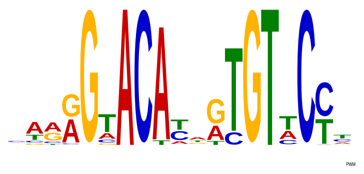	NAAGGTACNNNNNGTTCCN	680.24	DR:4	8
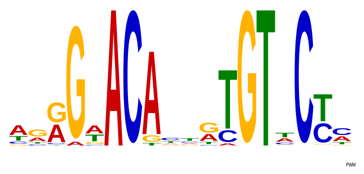	NGGGTACANNNNGTCCTN	453.77	IR:5	9

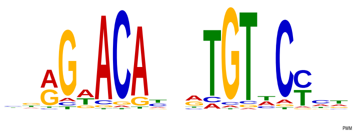	NN$\overrightarrow{NGGACN}$NNNNGTACNNN	28.57	M	10
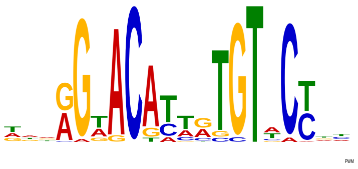	NNA$\overrightarrow{AGAACN}$NNN$\overrightarrow{NGTTCC}$NN	268.77	DR:3	11
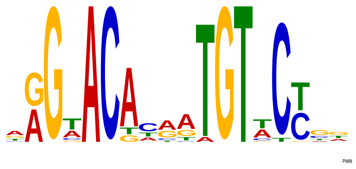	NN$\overrightarrow{GTACA}$$\overleftarrow{CGANGT}$TCCNN	1484.28	IR:0	12
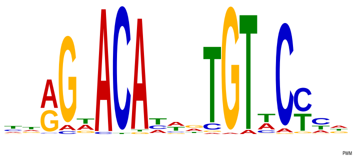	NNA$\overrightarrow{GGACN}$NNN$\overrightarrow{NGTTCC}$NN	128.46	DR:3	13
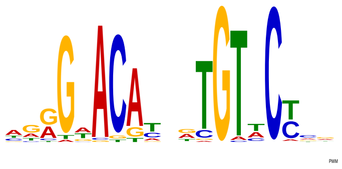	NN$\overrightarrow{NGGTACN}$NNN$\overrightarrow{NNGTCC}$NNN	65.80	DR:3	14

30 GR-Jolma2013-Rnd4 (ERR194207)

PWM	Seed Sequence	Seed Seq Enrichment	Repeat	Rank
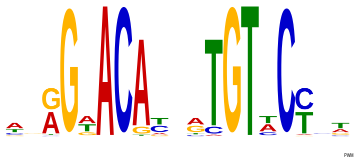	NNNGTACNNNNNGTACNNN	178.70	M	1
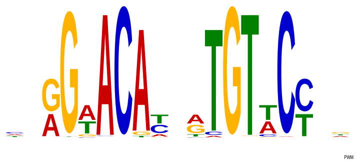	NNGGAANNNNNNNTTCCNN	98.53	M	2
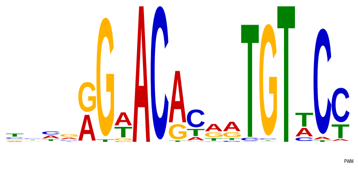	NNN$\overrightarrow{NGGTAN}$NCGATGNNNN	89.15	M	3
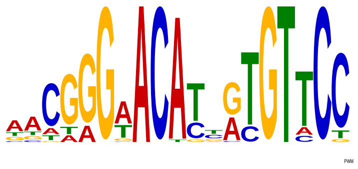	AACGGGNNNNNNN$\overrightarrow{NGTTCC}$	393.61	M	4
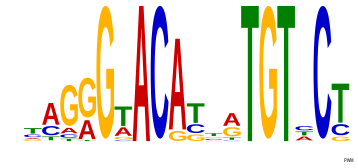	NT$\overrightarrow{AGGGTA}$NNNNN$\overrightarrow{NGTCC}$N	262.80	DR:4	5
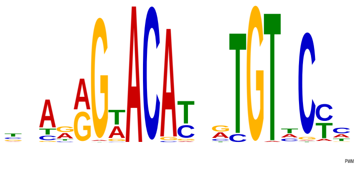	NNA$\overrightarrow{AAGTNN}$NNNNNNGTACNN	86.87	M	6
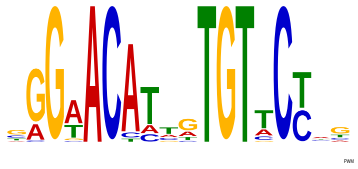	$\overrightarrow{GGGAA}$NNNNNNNTTCCAG	281.17	M	7
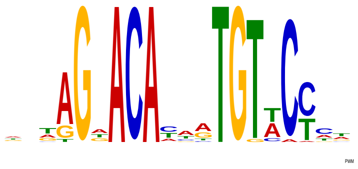	NNT$\overrightarrow{AGGACN}$NNNNNTTCCNN	189.25	M	8
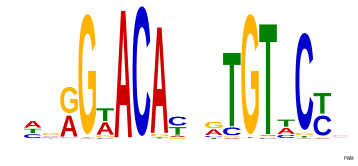	NN$\overrightarrow{GGGTNN}$NNNNNGTACNN	62.15	M	9

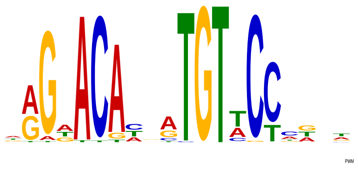	$NNGTACNNNNNNNTCCCGNN$	93.76	M	10
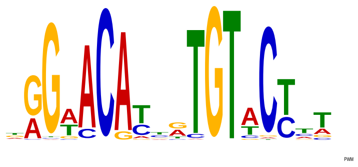	$NGGAANNNNNNNNNCTTTN$	46.17	M	11
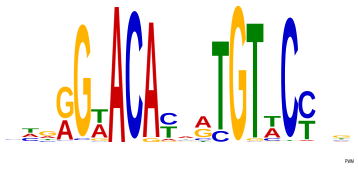	$NN\overrightarrow{GGGTN}NNNNNNNTTCCNN$	46.77	M	12
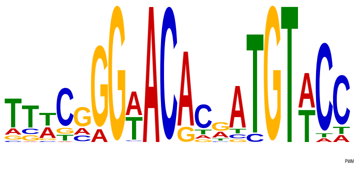	$NNNN\overrightarrow{GGGTAN}NCGATGNNNN$	153.80	M	13
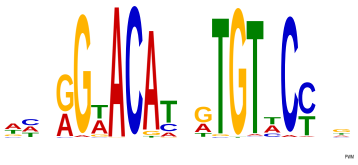	$NNGGGANNNNNNNNTTCCNN$	45.92	M	14

31 GR-3-Bhimsaria2022-Rnd3 (S07-125)

PWM	Seed Sequence	Seed Seq Enrichment	Repeat	Rank
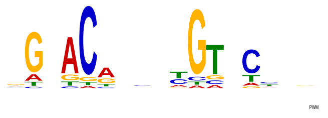	*NNNGTACANNNNGTCCNNN*	59.60	DR:2	1
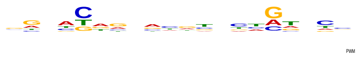	*NNNNNNGGACGTCCNNNNNN*	7.76	M	2

32 GR-Yin2017-Rnd2 (ERR1005236)

PWM	Seed Sequence	Seed Seq Enrichment	Repeat	Rank
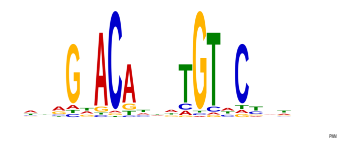	NNNNNNGTACANNNTGTACNNNNNN	34.64	IR:3	1
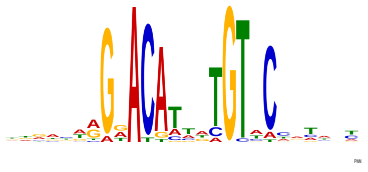	NNNNNNAGAACATTNTGTACNNNNNN	187.17	IR:3	2
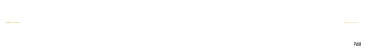	NNNNNNGGGGNNNNNGGGGNNNNNN	3.18	M	3
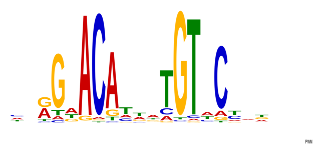	NNNNNNGGAACANNNTGTACNNNNNN	56.27	IR:3	4
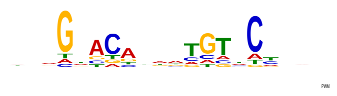	NNNNNNTACANNATGTNNNNNN	5.26	M	5
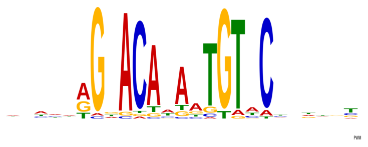	NNNNNNGGACAAANTGTACNNNNNN	56.96	IR:3	6
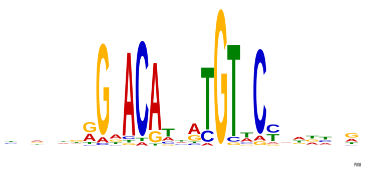	NNNNNNGGAACATNNNGTTCCNNNNNN	63.50	DR:3	7
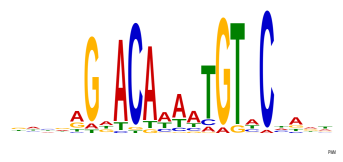	NNNNNNAGAACAAAATGTNNNNNN	59.98	DR:2	8
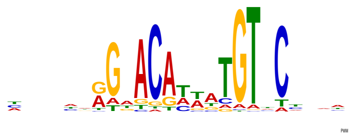	NNNNNNAAGAACANNATGTNNNNNN	28.19	DR:2	9

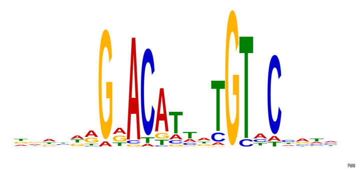	*NNNNNNAGAACATTNTGTANNNNNN*	61.53	M	10
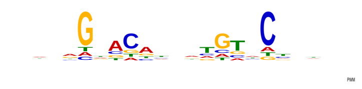	*NNNNNNTACANNNTGTANNNNNN*	4.22	M	11
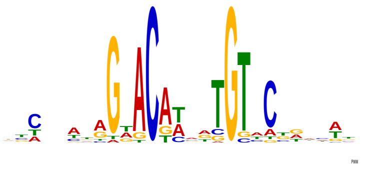	*NNNNNNGAGTACANNNTGTACNNNNNN*	57.07	IR:3	12
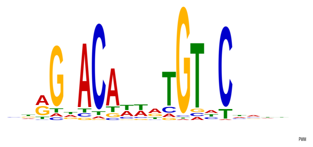	*NNNNNNAGAACANNATGTANNNNNN*	31.09	DR:2	13
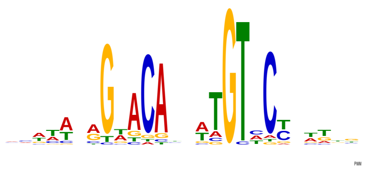	*NNNNNNGGTACACNNTGTACNNNNNN*	47.68	IR:4	14
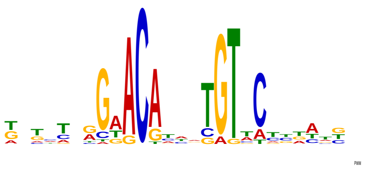	*NNNNNNGGAACATNNNGTACCNNNNNN*	44.29	IR:4	15
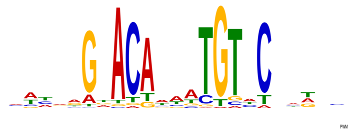	*NNNNNNGGACANAATGTANNNNNN*	23.89	DR:2	16
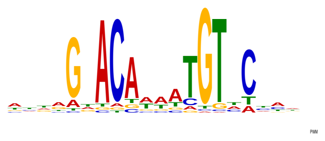	*NNNNNNAGGACANAATGTNNNNNN*	28.79	DR:2	17
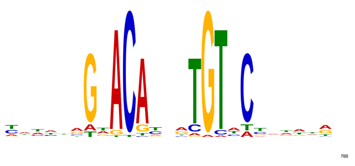	*NNNNNNAGTACANNNTGTACTNNNNNN*	99.52	IR:3	18

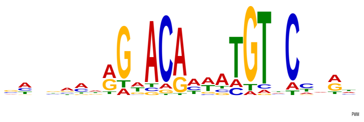	$NNNNNNA\overrightarrow{AGAACN}NA\overrightarrow{ATGTNN}NNNN$	17.60	DR:2	19
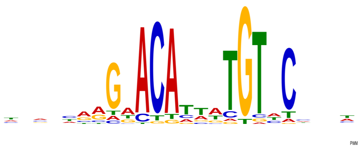	$NNNNNN\overrightarrow{AGAANA}TT\overrightarrow{ATGTNN}NNNN$	22.27	DR:2	20

33 GR-Yin2017-Rnd3 (ERR1005238)

PWM	Seed Sequence	Seed Seq Enrichment	Repeat	Rank
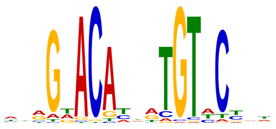	NNNNNNGTACANNNTGTACNNNNNN	63.87	IR:3	1
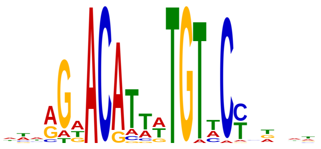	NNNNNNGAACATTNTGTTCCNNNNNN	419.79	DR:3	2
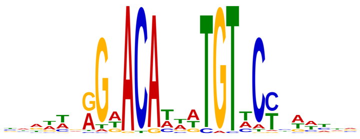	NNNNNNGGAACANNNTGTTCCNNNNNN	192.31	DR:3	3
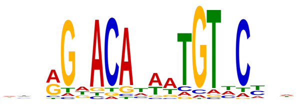	NNNNNNAGAACNNNATGTNNNNNN	19.02	DR:2	4
	NNNNNNGGGGNNNNNGGGGNNNNNN	5.72	M	5
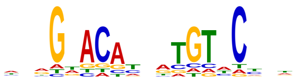	NNNNNNTACANNNTGTANNNNNN	7.90	M	6
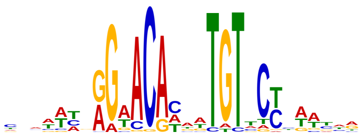	NNNNNNGGGAACNNNNNGTTCCNNNNNN	31.74	DR:5	7
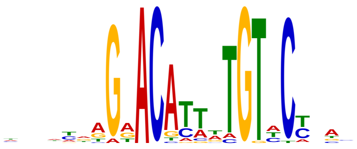	NNNNNNAGAANATTTTGTNNNNNN	53.47	M	8
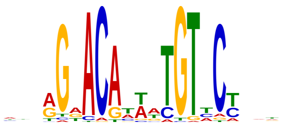	NNNNNNAGAACNNNNGTTCTNNNNNN	30.86	DR:3	9

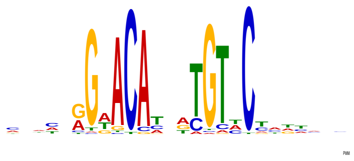	$NNNNNN\overrightarrow{GGAACA}NNNN\overleftarrow{NGTACT}NNNNNN$	66.02	IR:3	10
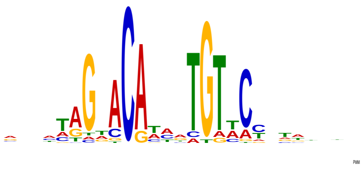	$NNNNN\overrightarrow{NGTACA}NNNN\overrightarrow{NGTTCC}GNNNNNN$	32.11	DR:3	11
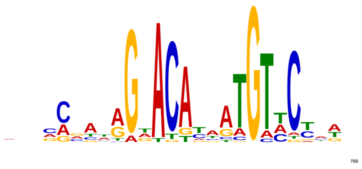	$NNNNNNA\overrightarrow{AAGTAC}NNN\overrightarrow{ATGTN}NNNNN$	37.60	DR:3	12
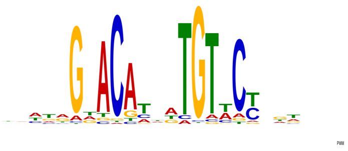	$NNNNN\overrightarrow{NGTACA}NNN\overrightarrow{NGTTCC}NNNNNN$	36.42	DR:3	13
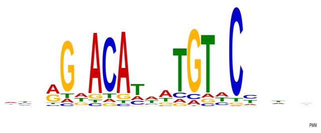	$NNNNNN\overrightarrow{AGAACN}NNNTGTANNNNNN$	13.92	M	14
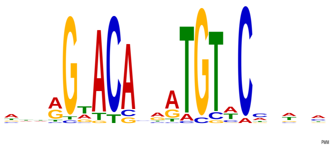	$NNNNN\overrightarrow{NGTACA}CA\overrightarrow{ATGTAN}NNNNN$	69.79	DR:2	15
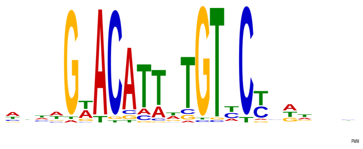	$NNNN\overleftarrow{NNACAT}TT\overrightarrow{NGTTCT}NNNNNN$	51.58	ER:2	16
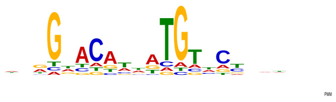	$NNNNNNTACANNNNNNTTCTNNNNNN$	4.70	M	17
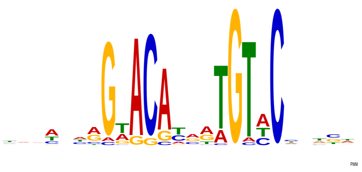	$NNNNNN\overrightarrow{AGTACA}CN\overrightarrow{ATGTAN}NNNNN$	77.95	DR:2	18

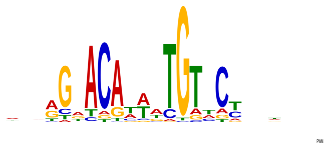	$NNNNNN\overrightarrow{AGAAC}NNNNNNTTCTNNNNNN$	9.10	M	19
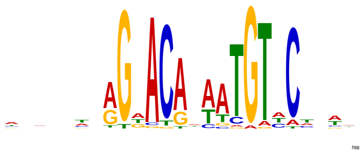	$NNNNNNT\overrightarrow{AGAAC}NNA\overrightarrow{ATGT}NNNNNN$	48.54	DR:2	20

34 GR-Yin2017-Rnd3 (ERR1005239)

PWM	Seed Sequence	Seed Seq Enrichment	Repeat	Rank
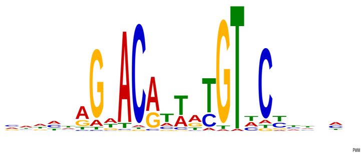	NNNNNNGAACATNNTGTACNNNNNN	64.38	IR:3	1
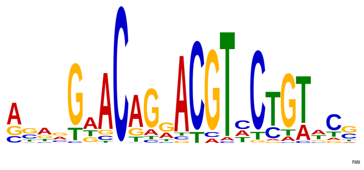	NNNNNNACAGGACGTCCNNNNNN	25.64	M	2
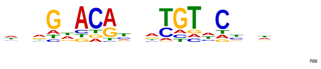	NNNNNNGTACNNNNNGTACNNNNNN	6.22	M	3
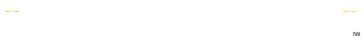	NNNNNNGGGGNNNNNGGGGNNNNNN	2.94	M	4
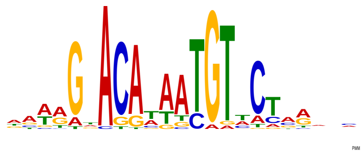	NNNNNNACAAAATGTTCNNNNNN	34.38	M	5
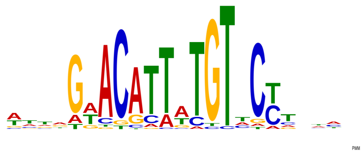	NNNNNNACATTTTGTTCNNNNNN	31.00	ER:2	6
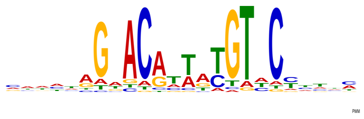	NNNNNNGAACATNNTGTANNNNNN	19.33	M	7
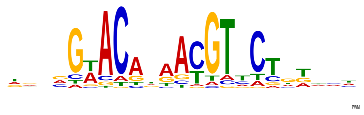	NNNNNNACAGAANGTTCNNNNNN	10.85	M	8
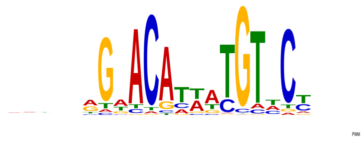	NNNNNNGAACATNATGTNNNNNN	29.09	IR:0	9

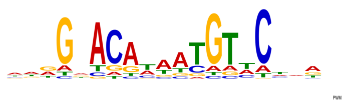	$NNNNNNACAAA\overrightarrow{ATGTN}NNNNN$	6.74	M	10
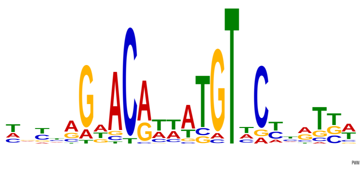	$NNNNN\overleftarrow{NGACAT}TN\overrightarrow{TGTTCN}NNNNNN$	22.33	ER:2	11
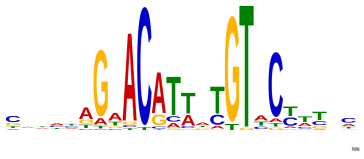	$NNNNN\overrightarrow{NGAACA}TTNTGTANNNNNN$	45.17	M	12
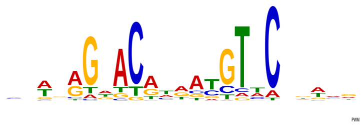	$NNNNNNTACANA\overrightarrow{ATGTAN}NNNNNN$	8.83	M	13
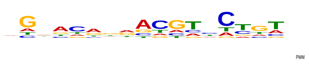	$NNNNNNNAC\overrightarrow{AGNACG}TNNNNNN$	3.38	M	14
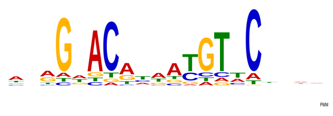	$NNNNNNTACANA\overrightarrow{ATGTN}NNNNN$	7.26	M	15
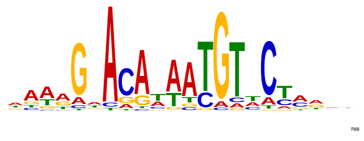	$NNNNNNACAAA\overrightarrow{ATGTTN}NNNNNN$	10.79	M	16
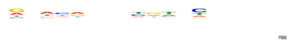	$NNNNNNTACGNNNCGTANNNNNN$	2.12	M	17
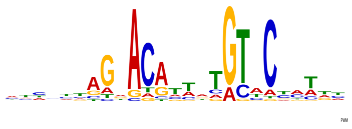	$NNNNNNT\overrightarrow{AGGACN}NNNTGTANNNNNN$	8.76	M	18

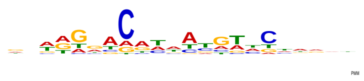	$NNNNNNACAAA\overrightarrow{NTGTC}NNNNNN$	2.81	M	19
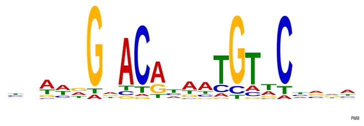	$NNNNNNTACAAA\overrightarrow{ATGTN}NNNNN$	6.96	M	20

35 GR-Yin2017-Rnd4 (ERR1005240)

PWM	Seed Sequence	Seed Seq Enrichment	Repeat	Rank
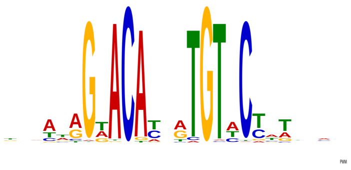	NNNNNNAGTACANNNTGTACTNNNNNN	500.38	IR:3	1
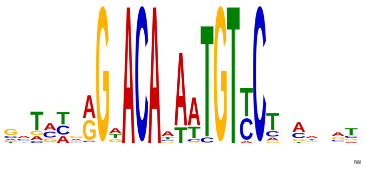	NNNNNNAGAACAAAATGTTCTNNNNNN	2245.22	IR:3	2
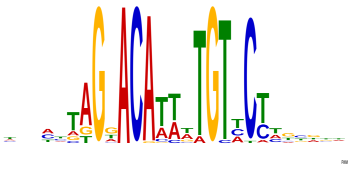	NNNNNNTAGGACATNNNGTTCTNNNNNN	591.07	DR:3	3
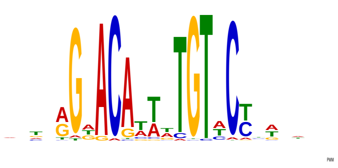	NNNNNNAGAACATNNNGTTCTNNNNNN	261.30	DR:3	4
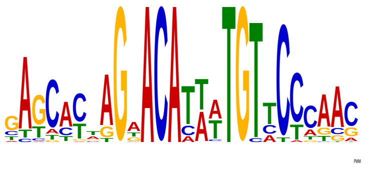	NNNNNNTAGAANATTATGTTNNNNNN	340.08	DR:2	5
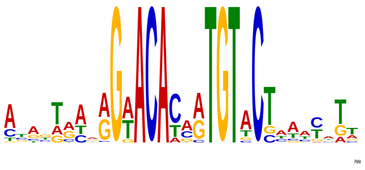	NNNNNNAAAGTACNNNNTGTACTANNNNNN	881.90	IR:4	6
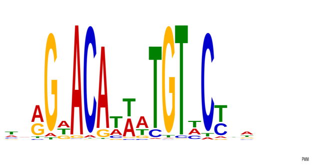	NNNNNNAGAACNNNNNGTTCTNNNNNN	62.45	DR:3	7
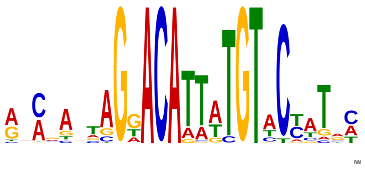	NNNNNNTAGGANATTATGTANNNNNN	341.95	DR:2	8
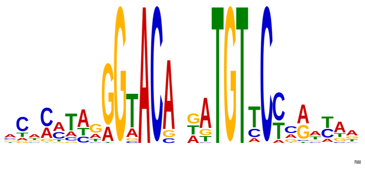	NNNNNNAGGGTACNNNATGTTCCNNNNNN	687.93	DR:4	9

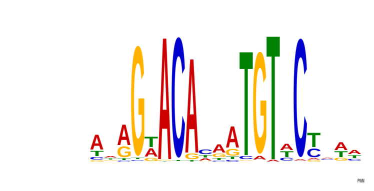	$NNNNNNA\overrightarrow{AAGTAC}NNN\overrightarrow{ATGTN}NNNNN$	104.33	DR:3	10
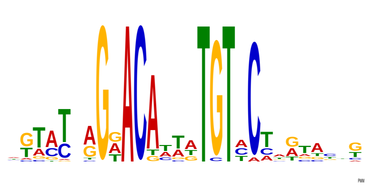	$NNNNNN\overrightarrow{AGGACA}TNN\overleftarrow{NGTCCT}ANNNNNN$	319.10	IR:3	11
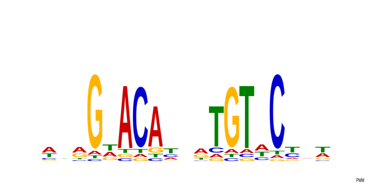	$NNNNNNTACANNNTGTANNNNNN$	16.17	M	12
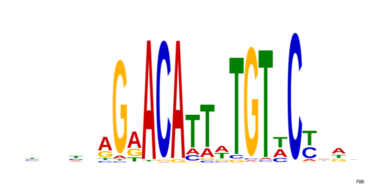	$NNNNNNAGAANNTTTTGTNNNNNN$	48.03	M	13
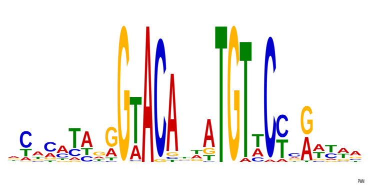	$NNNNNN\overrightarrow{AGGGTA}CANNNN\overrightarrow{NGTTCC}NNNNNN$	347.18	DR:5	14
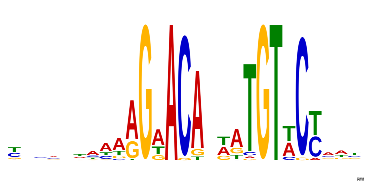	$NNNNNNAAA\overrightarrow{AGAACN}NN\overrightarrow{ATGTN}NNNNN$	157.34	DR:2	15
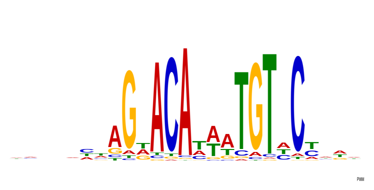	$NNNNNNT\overrightarrow{AGGACN}NN\overrightarrow{ATGTN}NNNNN$	39.59	DR:2	16
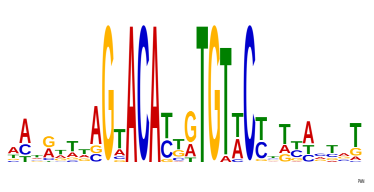	$NNNNNNT\overrightarrow{AGTACA}NNNN\overleftarrow{NTACTT}TNNNNNN$	286.20	IR:4	17
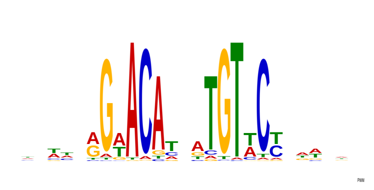	$NNNNNN\overleftarrow{GGAACN}NNNN\overrightarrow{NGTTCC}NNNNNN$	43.13	ER:3	18

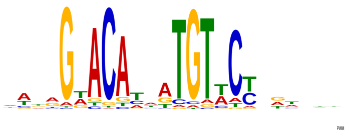	$NNNNNNTACANNNN\overrightarrow{NGTTCC}NNNNNNN$	24.90	M	19
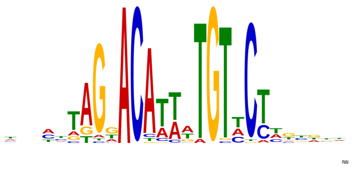	$NNNNNNT\overrightarrow{AGGACA}TNNNNTTCTNNNNNN$	198.56	M	20

36 GR-Yin2017-Rnd4 (ERR1005241)

PWM	Seed Sequence	Seed Seq Enrichment	Repeat	Rank
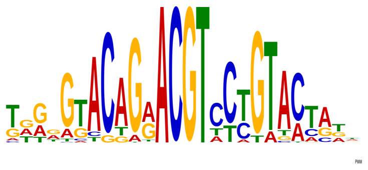	NNNNNNACAGGACNTTCTGTNNNNNN	943.29	M	1
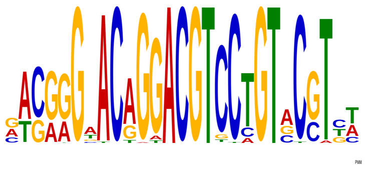	NNNNNNAACAGGACNTCCTGTNNNNNN	6047.11	M	2
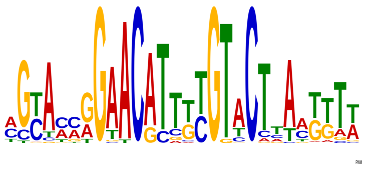	NNNNNNGGAACATTNNGTACTTNNNNNN	2330.44	IR:3	3
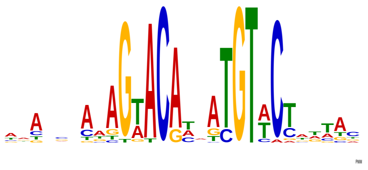	NNNNNNAAAGTACANNNTGTACNNNNNN	605.48	IR:3	4
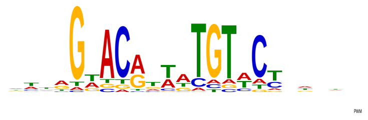	NNNNNNACATNNNGTTCNNNNNN	12.58	ER:2	5
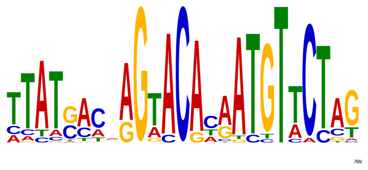	NNNNNNAAAGTACACAATGNNNNNN	277.97	M	6
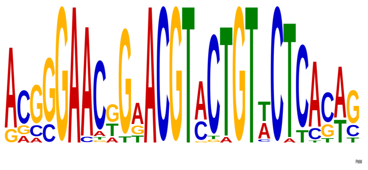	NNNNNNACGGTACGNNCTGTTCNNNNNN	832.80	IR:0	7
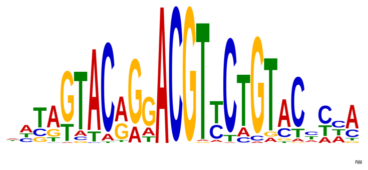	NNNNNNACAGAANNTCCTGTNNNNNN	317.84	M	8
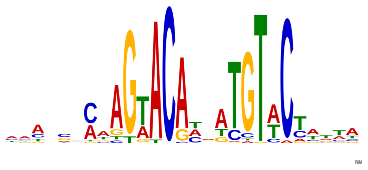	NNNNNNAAAGTACANNNTGTANNNNNN	200.26	M	9

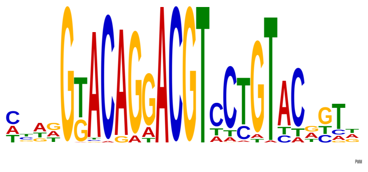	NNNNNNACAGGACNTACTGTNNNNNN	747.54	M	10
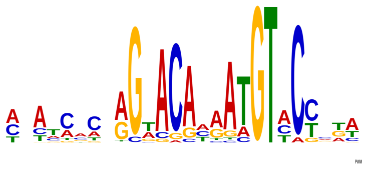	NNNNNNAAAGNNCACAATGTNNNNNN	92.80	DR:2	11
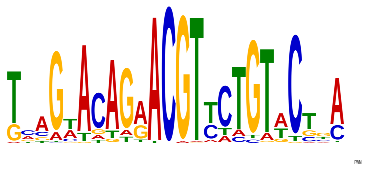	NNNNNNCAGAANNTTCTGTNNNNNN	132.62	M	12
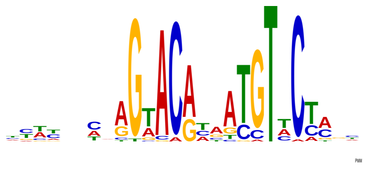	NNNNNNAAAGTACNNAATGTNNNNNN	171.93	DR:3	13
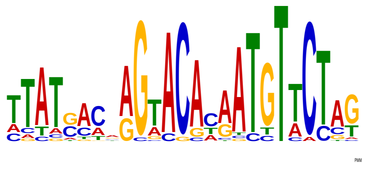	NNNNNNAAAGTNCACAATGNNNNNN	95.57	M	14
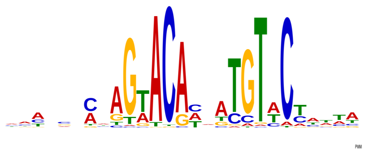	NNNNNNAAAGTACNNNNTGTANNNNNN	66.13	M	15
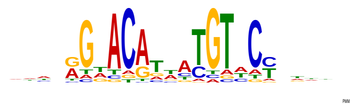	NNNNNNGAACNNNNNGTTCNNNNNN	13.33	ER:3	16
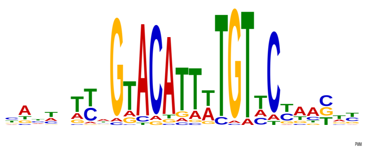	NNNNNNTTGTACATNNNGTTCNNNNNN	121.69	DR:3	17
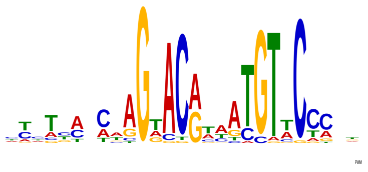	NNNNNNGAAAGTACNNNATGTNNNNNN	80.38	DR:3	18

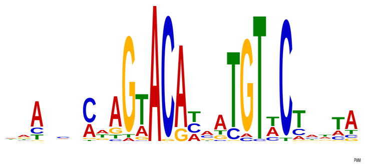	NNNNNNA$\overrightarrow{AAGTAC}$NNN$\overrightarrow{ATGTAN}$NNNNN	144.45	DR:3	19
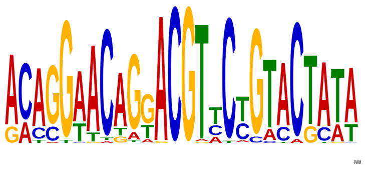	NNNNNNAC$\overrightarrow{AGGANN}\overleftarrow{TTCCGT}$NNNNNN	208.32	IR:0	20

37 GR-SelexGLM-Rnd3 (SRR5340721)

PWM	Seed Sequence	Seed Seq Enrichment	Repeat	Rank
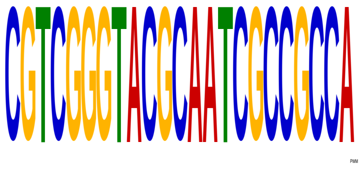	CGTCGGGTNNNNNNNCGCCGCCA	103858.30	DR:12	1
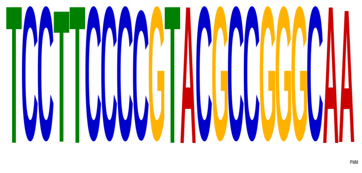	NNCTTCCCCGNNCGCCGGGCNN	92511.77	ER:6	2
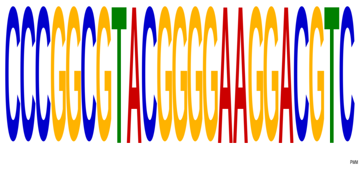	CCCGGCGTNNNNNNNAGGACGTC	87485.33	ER:7	3
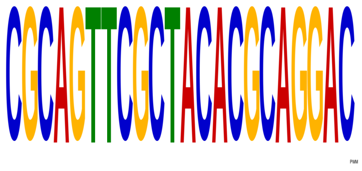	CGCAGTTCNNNNNNCGCAGGAC	82308.86	DR:8	4
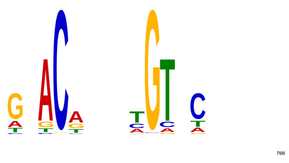	NNNNNGTACANNNTGTACNNNNN	1311.91	IR:3	5
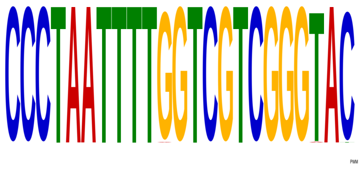	CCCTAATTNNNNNNNTCGGGTAC	66928.35	ER:12	6
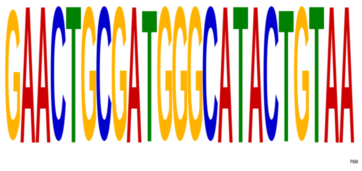	GAACTGCGNNNNNNNTACTGTAA	74605.35	ER:12	7
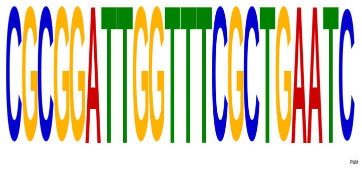	CGCGGATTNNNNNNGCTGAATC	70889.89	M	8
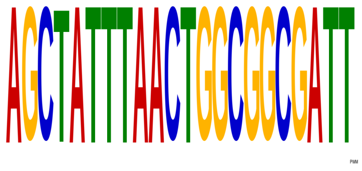	NNCTATTTAANNGGCGGCGANN	76753.52	DR:4	9

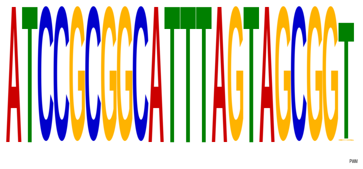	NTCCGCGGCNNNNAGTAGCGGN	67677.20	M	10
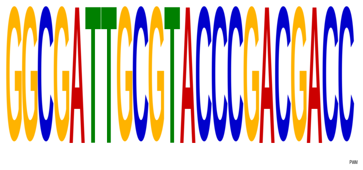	GGCGATTGNNNNNNCGACGACC	62049.42	IR:12	11
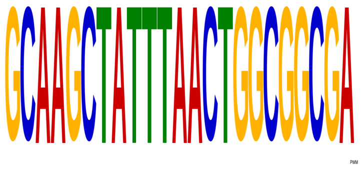	GCAAGCTANNNNNNNGGCGGCGA	58056.35	ER:10	12
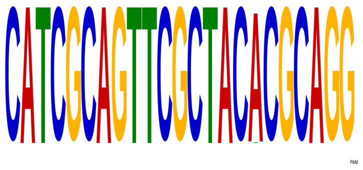	CATCGCAGNNNNNNNCACGCAGG	65233.05	M	13
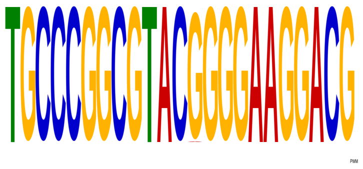	NGCCCGGCGNNNNNGGAAGGACN	79771.10	ER:11	14
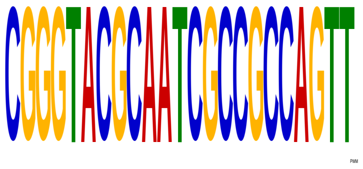	NGGGTACGCNNNNNCCGCCAGTN	81348.32	DR:7	15
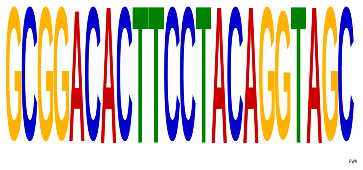	GCGGACACNNNNNNCAGGTAGC	71249.60	DR:8	16
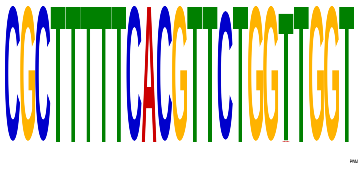	NGCTTTTTCNNNNNCTGGTTGGN	55018.98	M	17
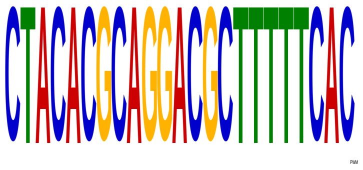	CTACACGCNNNNNNNTTTTTCAC	59835.79	M	18

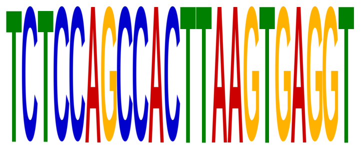 PWM	$NCTCCAGCCNNNN\overrightarrow{AAGTGA}GGN$	58683.53	M	19
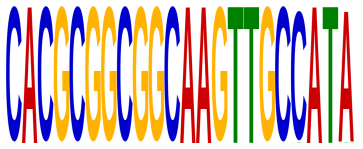 PWM	$NNCGCGG\overrightarrow{CGGNNA}G\overrightarrow{TTGCCA}NN$	73186.43	DR:1	20

38 GR-SelexGLM-Rnd4 (SRR5340720)

PWM	Seed Sequence	Seed Seq Enrichment	Repeat	Rank
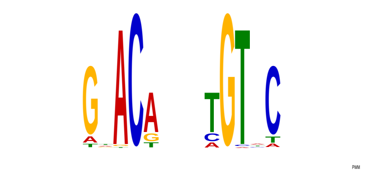	NNNNNGTACANNNTGTACNNNNN	4731.76	IR:3	1
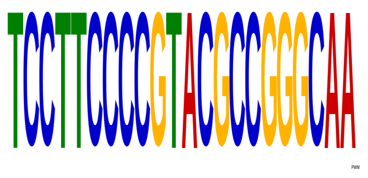	NNCTTCCCCGNNCGCCGGGCNN	108519.63	ER:6	2
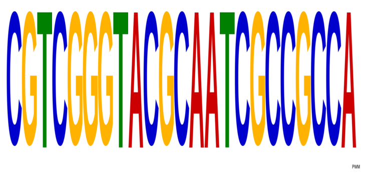	CGTCGGGTNNNNNNNCGCCGCCA	81699.01	DR:12	3
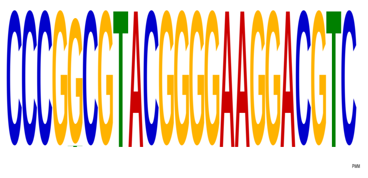	CCCGGCGTNNNNNNNAGGACGTC	60770.13	ER:7	4
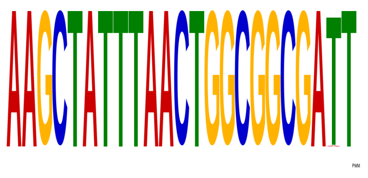	NNGCTATTTANNNGGCGGCGANN	65291.79	M	5
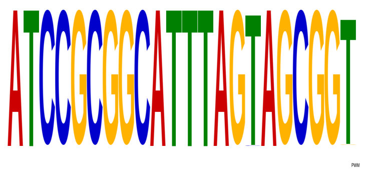	NTCCGCGGCNNNNAGTAGCGGN	60986.04	M	6
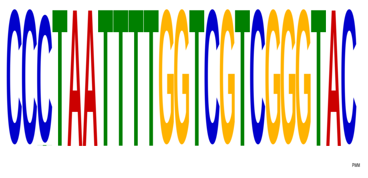	CCCTAATTNNNNNNNTCGGGTAC	69714.59	ER:12	7
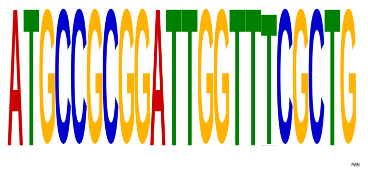	NNGCCGCGGANNGGTTTCGCNN	60871.95	M	8
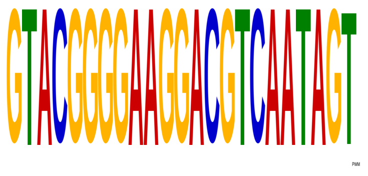	GTACGGGGNNNNNNNTCAATAGT	63022.29	M	9

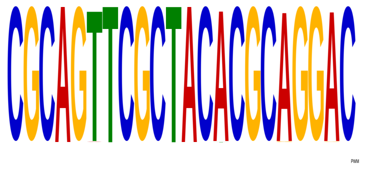	$CGC\overrightarrow{AGTTC}NNNNNNCGC\overrightarrow{AGGAC}$	51922.00	DR:8	10
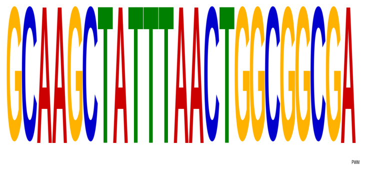	$G\overleftarrow{CAAGCT}ANNNNNNNGG\overrightarrow{CGGCGA}$	64608.84	ER:10	11
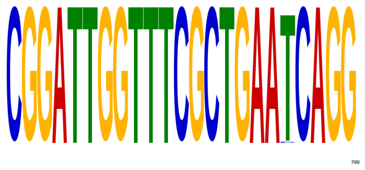	$CGGATTGGNNNNNNN\overrightarrow{GAATCA}GG$	54257.34	M	12
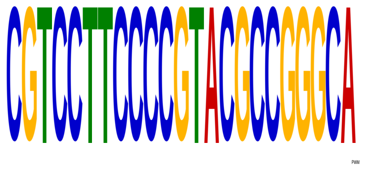	$\overleftarrow{CGTCC}TTCNNNNNNNGC\overrightarrow{CGGGCA}$	58103.40	ER:11	13
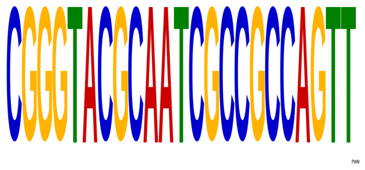	$N\overrightarrow{GGGTAC}GCNNNNN\overrightarrow{CCGCCA}GTN$	65702.11	DR:7	14
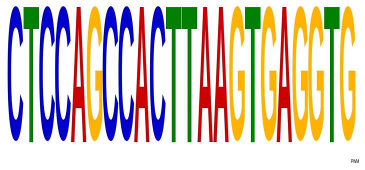	$CTCCAGCCNNNNNNGTG\overrightarrow{AGGTG}$	46429.14	M	15
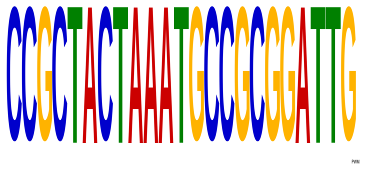	$CCGCTACTNNNNNNNGCGGATTG$	57576.90	M	16
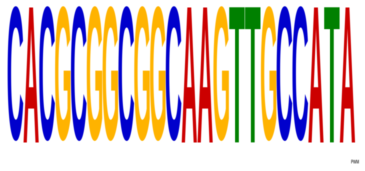	$NNCGCGG\overrightarrow{CGGNNA}\overrightarrow{GTTGCCA}NN$	60986.56	DR:1	17
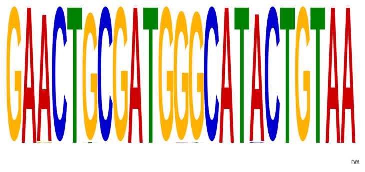	$\overleftarrow{GAACT}GCGNNNNNNNTA\overrightarrow{CTGTAA}$	47435.27	ER:12	18

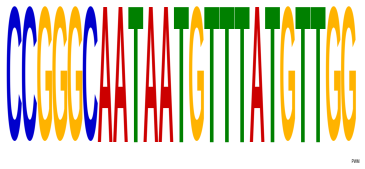	$C\overrightarrow{CGGGCA}ANNNNNNNT\overrightarrow{ATGTTG}G$	55613.09	DR:9	19
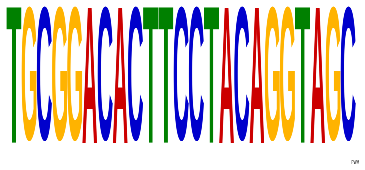	$TG\overrightarrow{CGGACA}NNNNNNN\overrightarrow{CAGGTAG}C$	50381.68	DR:8	20

39 GR-SelexGLM-Rnd5 (SRR5340719)

PWM	Seed Sequence	Seed Seq Enrichment	Repeat	Rank
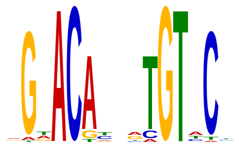	NNNNNGTACANNNTGTACNNNNN	9360.40	IR:3	1
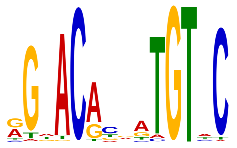	NNNNGGAACACAATGTACNNNN	40315.53	DR:2	2
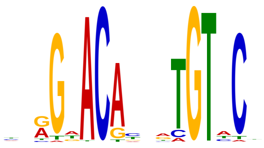	NNNNGGGAACANNNTGTACNNNN	14519.11	IR:5	3
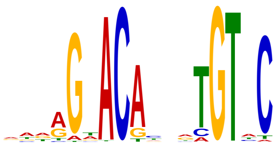	NNNAAAGAACANNNTGTACNNN	17477.38	IR:3	4
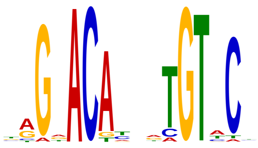	NNNCTAGGACANNNTGTACNNN	13902.77	IR:3	5
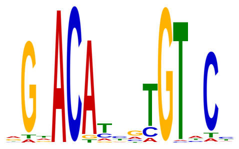	NNNNNGAACATTGTGTACNNNNN	25179.22	IR:3	6
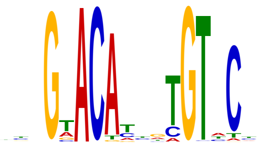	NNNNCTGTACATNNTGTACNNNN	15175.91	IR:3	7
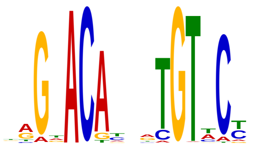	NNNNAGGACANNNTGTTCCNNNN	13115.72	DR:3	8
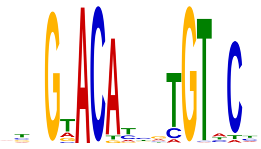	NNNNTCGTACATNNTGTACNNNN	17139.10	IR:3	9

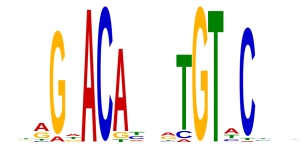 PWM	*NNNNAGGACANNNTGTACNNNN*	10003.37	IR:3	10
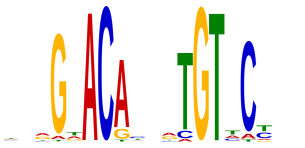 PWM	*NNNGGGTACANNNTGTTCCNNN*	19790.76	DR:5	11
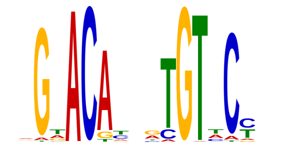 PWM	*NNNNGTACANNNNGTTCCNNNN*	4135.46	DR:3	12
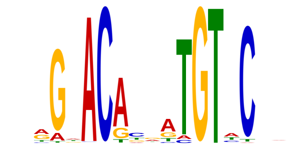 PWM	*NNNNAGGACACAATGTACNNNN*	30285.68	DR:2	13
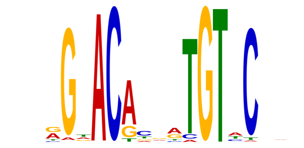 PWM	*NNNNGAGTACNNNATGTACNNNN*	7337.03	DR:2	14
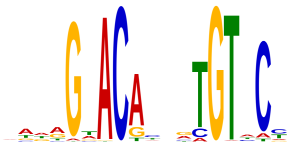 PWM	*NNNAAAGTACANNNTGTTCNNN*	15171.69	DR:3	15
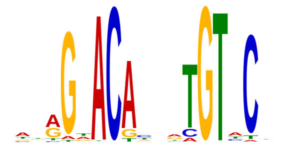 PWM	*NNNNAAGAACANNNTGTACNNNN*	10501.64	IR:3	16
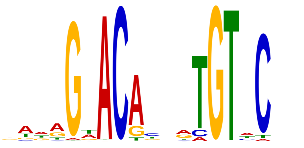 PWM	*NNNAAAGTACNNNNTGTACNNN*	6605.23	IR:4	17
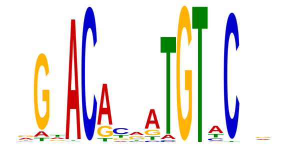 PWM	*NNNNGTACACAATGTACANNNN*	29052.85	DR:2	18

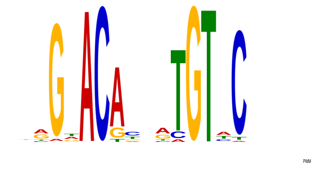	$NNNNG\overrightarrow{AGTAC}NNNN\overleftarrow{TGTAC}NNNN$	4235.13	IR:3	19
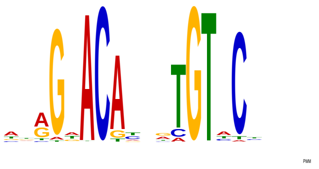	$NNNNA\overrightarrow{AGAAC}ANNNNGTACNNNN$	3358.69	M	20

40 GR-SelexGLM-Rnd6 (SRR5340718)

PWM	Seed Sequence	Seed Seq Enrichment	Repeat	Rank
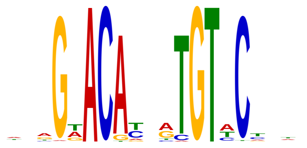 PWM	NNNNNGTACANNNTGTACNNNNN	13459.90	IR:3	1
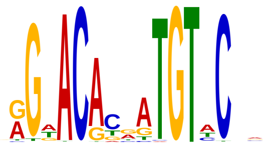 PWM	NNNNGGAACACAATGTACNNNN	85071.18	DR:2	2
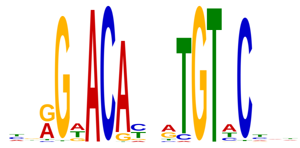 PWM	NNNNGGGAACANNNTGTACNNNN	22545.96	IR:5	3
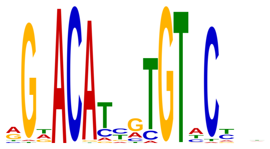 PWM	NNNNNGAACATTGTGTACNNNNN	52792.42	IR:3	4
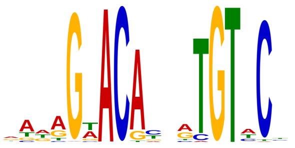 PWM	NNNAAAGTACANNNTGTACNNN	35409.51	IR:3	5
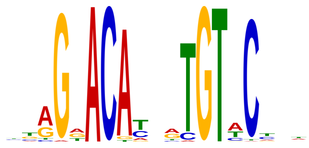 PWM	NNNCTAGGACANNNTGTACNNN	20606.95	IR:3	6
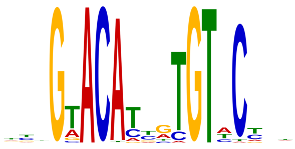 PWM	NNNNCTGTACATNNTGTACNNNN	23190.81	IR:3	7
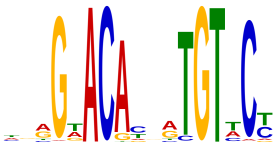 PWM	NNNGGGTACANNNTGTTCCNNN	32941.51	DR:5	8
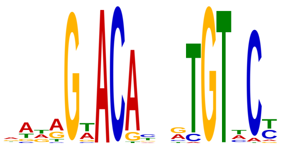 PWM	NNNAAAGTACANNNTGTTCCNNN	50508.66	DR:3	9

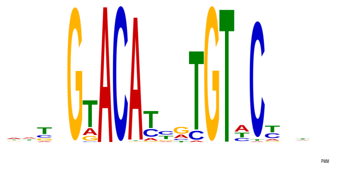	NNNNTCGTACATNNTGTACNNNN	26660.77	IR:3	10
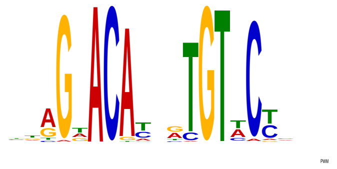	NNNTAGGACANNNTGTTCCNNN	26650.72	DR:3	11
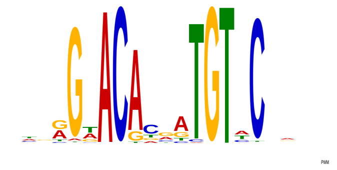	NNNNGGGTACANGATGTACNNNN	64732.76	IR:2	12
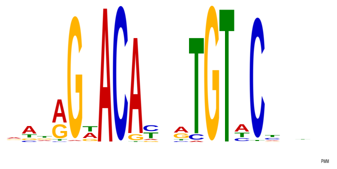	NNNNAAGAACANNNTGTACNNNN	17317.81	IR:3	13
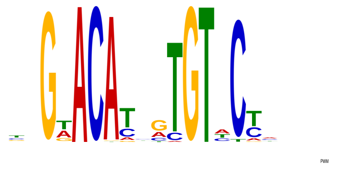	NNNNGTACATTNTGTCCTNNNN	27427.24	IR:3	14
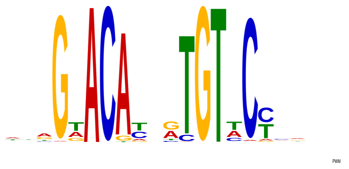	NNNNGTACANNNNGTTCCNNNN	5666.50	DR:3	15
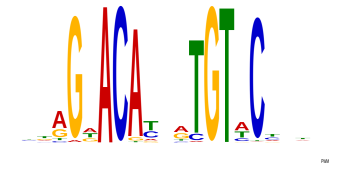	NNNNTAGGACANNNTGTACNNNN	19284.00	IR:3	16
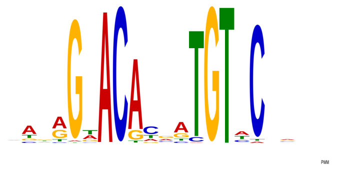	NNNNAAGTACANGATGTACNNNN	47380.38	IR:0	17
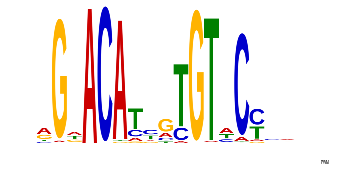	NNNNAGAACATTGTGTTCCNNNN	110520.68	IR:1	18

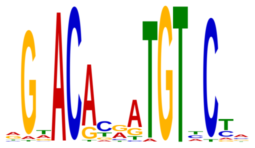	$NNNN\overrightarrow{GTACA}CAA\overleftarrow{TGTCCT}ANNNNN$	92447.99	IR:3	19
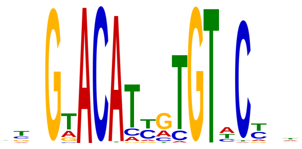	$NNNN\overrightarrow{CGTACA}TTG\overleftarrow{TGTAC}NNNN$	50196.28	IR:3	20

41 GR-SelexGLM-Rnd7 (SRR5340717)

PWM	Seed Sequence	Seed Seq Enrichment	Repeat	Rank
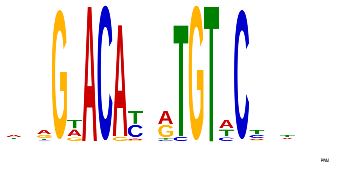	NNNNNGTACANNNTGTACNNNNN	16142.26	IR:3	1
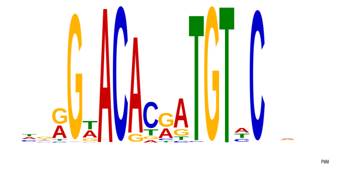	NNNNGGGTACACGATGTACNNNN	290374.11	IR:2	2
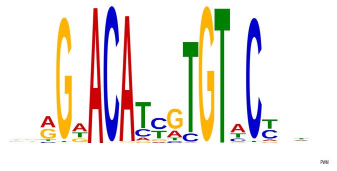	NNNNGGAACATCGTGTACNNNN	155334.58	IR:3	3
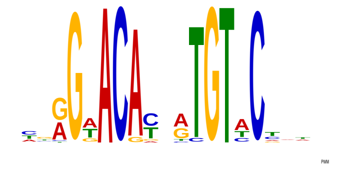	NNNNGGGAACANNNTGTACNNNN	29240.65	IR:5	4
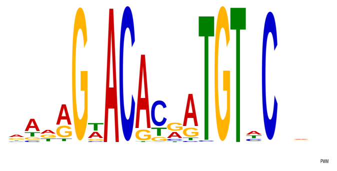	NNNAAAGTACACAATGTACNNN	283324.94	DR:2	5
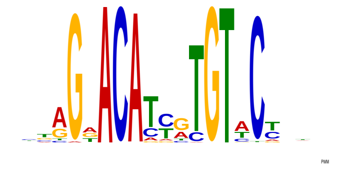	NNNNTAGGACATNGTGTACNNNN	97103.92	IR:3	6
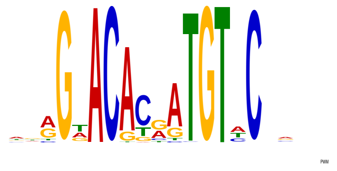	NNNNAGTACACAATGTACNNNN	148276.91	DR:2	7
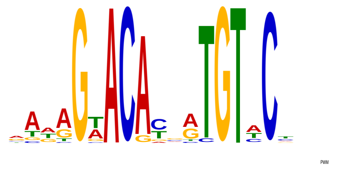	NNNAAAGTACANNNTGTACNNN	50085.40	IR:3	8
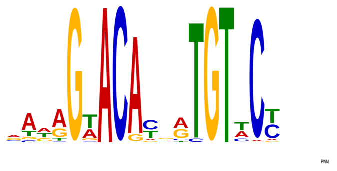	NNNAAAGTACANNNTGTTCCNNN	78527.56	DR:3	9

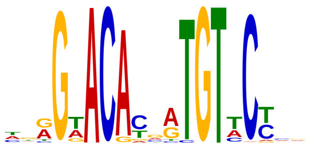 PWM	NNNGGGTACANNNTGTTCCNNN	43800.51	DR:5	10
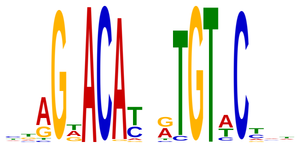 PWM	NNNNTAGGACANNNTGTACNNNN	22699.57	IR:3	11
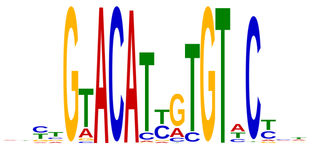 PWM	NNNNCTGTACATTGTGTACNNNN	145469.30	IR:3	12
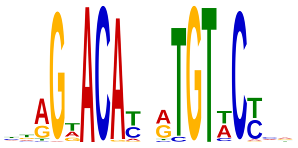 PWM	NNNNAGGACANNNTGTTCCNNNN	21010.85	DR:3	13
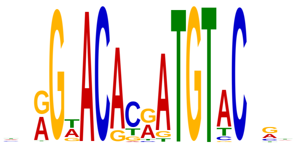 PWM	NNNNGGAACACAATGTACANNNN	132542.27	DR:2	14
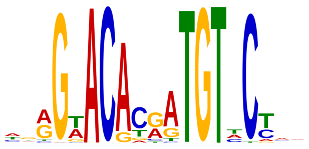 PWM	NNNGGGTACACNATGTCCTNNN	177959.64	IR:5	15
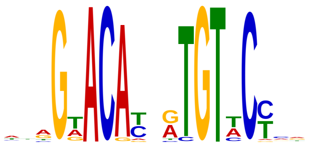 PWM	NNNNGTACANNNNGTTCCNNNN	6522.68	DR:3	16
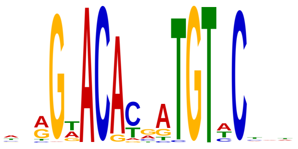 PWM	NNNNNGTACACGNTGTACNNNNN	47742.67	IR:3	17
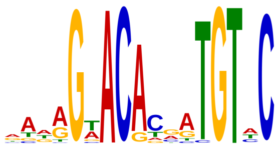 PWM	NNNAAAGTACANGATGTACNNN	164823.04	IR:0	18

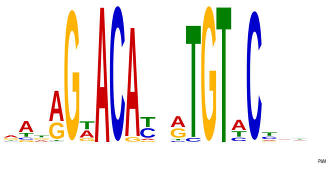	$NNNNA\overrightarrow{AGAAC}ANNN\overleftarrow{TGTAC}NNNN$	20777.74	IR:3	19
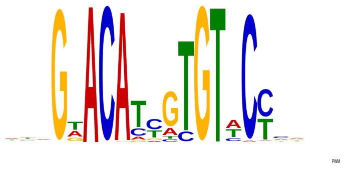	$NNN\overrightarrow{NGTAC}ATCG\overrightarrow{NGTTC}CNNNN$	53280.53	DR:3	20

42 GR-SelexGLM-Rnd8 (SRR5340716)

PWM	Seed Sequence	Seed Seq Enrichment	Repeat	Rank
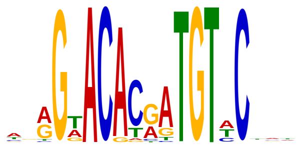	NNNNNGTACACGATGTACNNNNNN	183032.82	IR:0	1
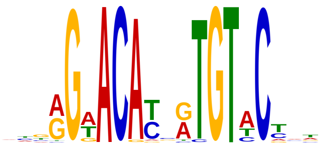	NNNNGGAACANNNTGTACNNNN	24019.96	IR:3	2
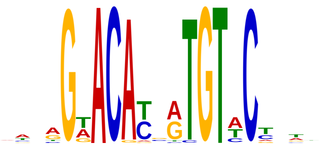	NNNNNGTACANNNTGTACNNNNN	17971.75	IR:3	3
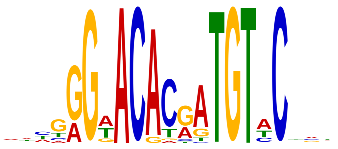	NNNCGGGAACACGATGTACNNN	496844.43	IR:2	4
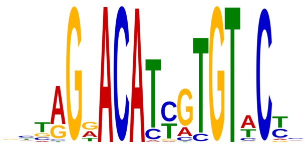	NNNCTAGGACATTGTGTACNNN	331982.72	IR:3	5
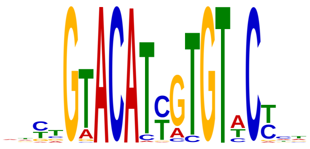	NNNNCTGTACATTGTGTACNNNN	190520.38	IR:3	6
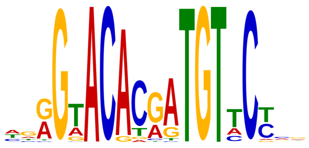	NNNGGGTACACNATGTTCCNNN	416169.14	DR:4	7
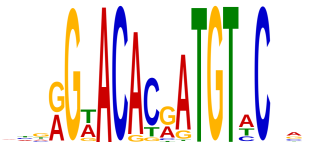	NNNNGGAACACAATGTACNNNN	142468.13	DR:2	8
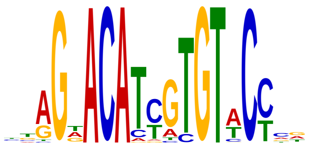	NNNAGGACATCGTGTTCCCNNN	481719.86	DR:3	9

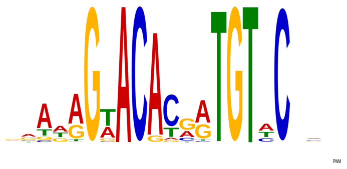	NNNAAAGTACANNATGTACNNN	136941.67	DR:2	10
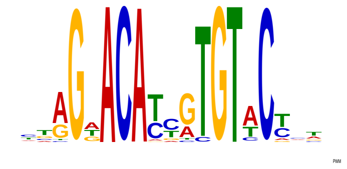	NNNNAGGACATNNTGTACNNNN	36101.61	IR:3	11
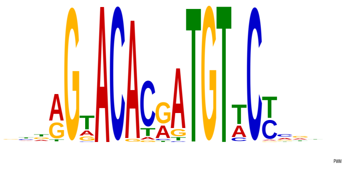	NNNNAGGACACGATGTTCCNNNN	216129.46	IR:0	12
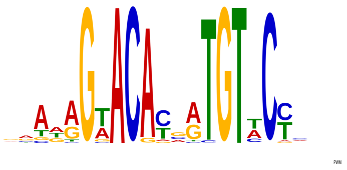	NNNAAAGTACANNNTGTTCCNNN	101096.24	DR:3	13
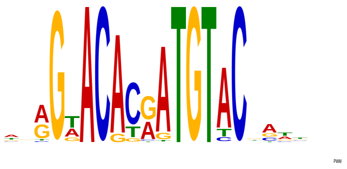	NNNNGTACACAATGTACGANNNN	174116.42	DR:2	14
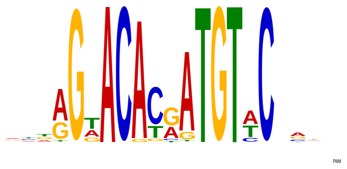	NNNNAGGACACAATGTACNNNN	79636.83	DR:2	15
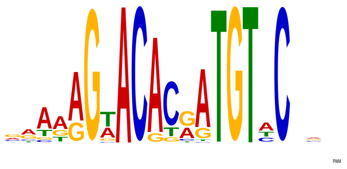	NNNAAAGAACACAATGTACNNN	332945.71	DR:2	16
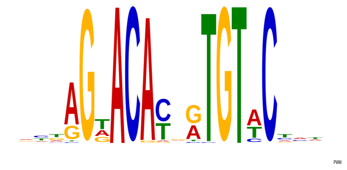	NNNNTAGGACANNATGTACNNNN	37917.23	DR:2	17
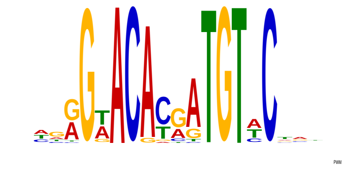	NNNNGGGTACACNNTGTACNNNN	79591.13	IR:5	18

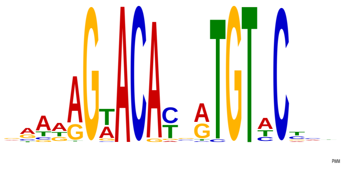	$NNNAA\overrightarrow{AGAACA}NNN\overleftarrow{TGTAC}NNN$	45903.76	IR:3	19
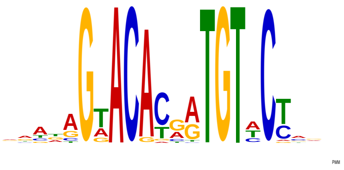	$NNNNT\overrightarrow{AGTACA}NN\overrightarrow{ATGTCC}NNNN$	36226.71	DR:2	20

43 ESRRA:RXRA-Bhimsaria2022-Rnd3 (S13-136)

PWM	Seed Sequence	Seed Seq Enrichment	Repeat	Rank
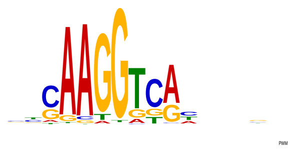	NNNNNNCAAGGTCANNNNNN	389.94	M	1
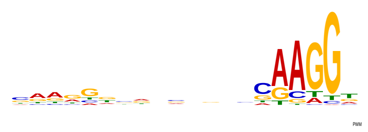	CAAGGNNNNNNNNNCAAGGT	1555.15	DR:8	2
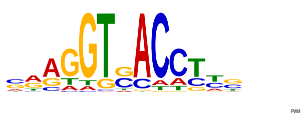	NNNNAAGGTNACCTTNNNN	810.56	M	3
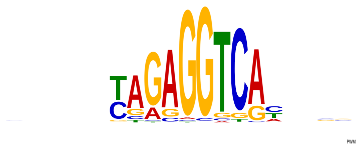	NNNNNNTAGAGGTCNNNNNN	86.85	M	4
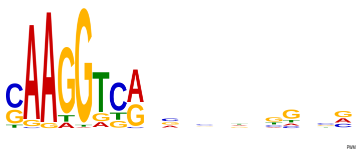	CAAGGTCNNNNNNNNNGTCG	1238.07	DR:6	5
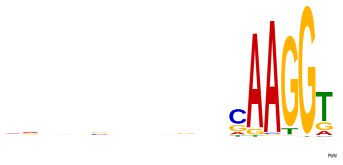	AGGGNNNNNNNNNNCAAGGT	548.61	DR:10	6
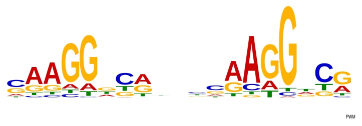	CAAGGNNNNNNNAAGGTCG	1599.81	DR:5	7
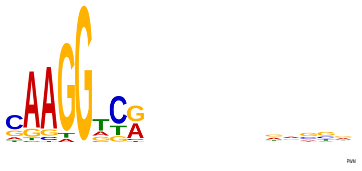	CAAGGTCNNNNNNNNNAGGG	1271.37	DR:8	8
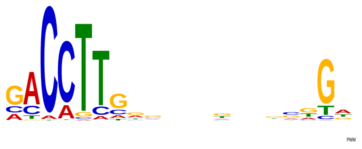	GACCTTGNNNNNNNNNGGGT	919.89	ER:11	9

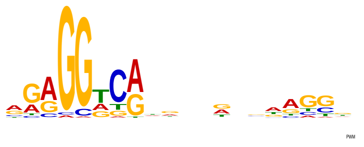	*AGAGGTCNNNNNNNNNAGGG*	569.06	DR:8	10
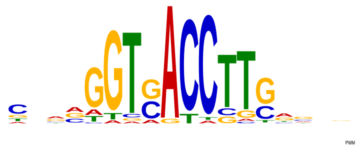	*NNNCGGTCACCTTGANNN*	1270.66	IR:3	11
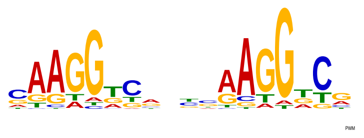	*CAAGGNNNNNNAAGGTCG*	1189.82	DR:4	12
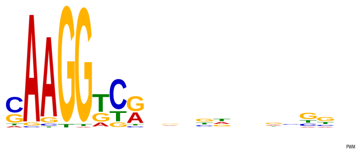	*CAAGGTCNNNNNNNNNCGGT*	989.91	DR:8	13
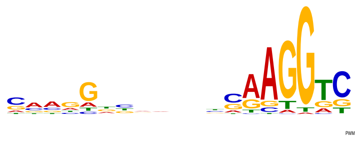	*CAAGNNNNNNNNNAAGGTC*	302.37	M	14
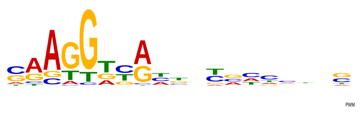	*NAAGGTCANNNNNNNCCTTN*	596.17	IR:3	15
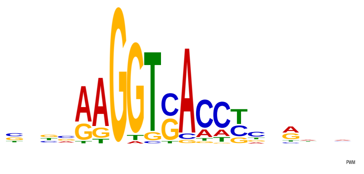	*NNNNAAGGTNACCCCTNNNN*	672.26	M	16
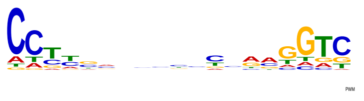	*CCTTNNNNNNNNNNAGGTC*	174.36	M	17
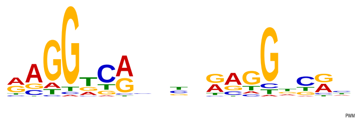	*NNGGTCNNNNNNNGGTCNN*	80.72	DR:5	18

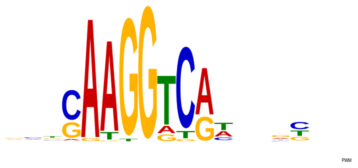	$NNNCA\overrightarrow{AGGTCA}TTCGNNN$	1096.03	M	19
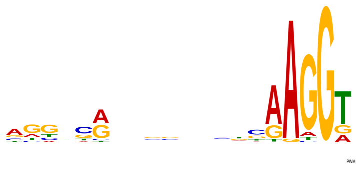	$\overrightarrow{AGGTCN}NNNNNNNNNA\overrightarrow{AGGT}$	286.48	DR:10	20

44 ESRRA-Bhimsaria2022-Rnd2 (S07-037)

PWM	Seed Sequence	Seed Seq Enrichment	Repeat	Rank
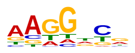	NNNNNNCAAGGTCGNNNNNN	9.15	M	1
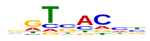	NNNNNNAGGTGACCNNNNNN	7.07	M	2
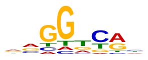	NNNNNNGAGGTCATNNNNNN	7.32	M	3
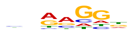	GGGGNNNNNNNNNNNNAGGG	5.27	M	4
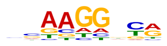	NNNNNNCAAGGGCANNNNNN	7.25	M	5
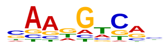	NNNNNNCAATGTCANNNNNN	5.86	M	6
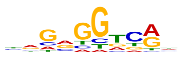	NNNNNNTAGGGGTCNNNNNN	4.60	M	7
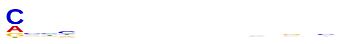	ACCCNNNNNNNNNNNNGGTC	4.12	ER:11	8
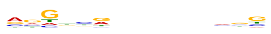	NNGGTCNNNNNNNNGGTCNN	3.63	DR:6	9

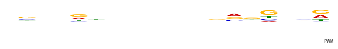	$\overrightarrow{NGGTCN}NNNNNNNN\overrightarrow{NGGTCN}$	2.84	DR:8	10
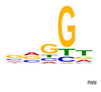	$GGGGNNNNNNNNNNNG\overrightarrow{GGGT}$	7.61	M	11
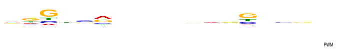	$N\overrightarrow{NGGTCN}NNNNN\overrightarrow{NGGTCN}N$	4.13	DR:5	12
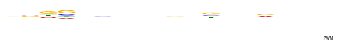	$NN\overrightarrow{NNGTCA}NN\overrightarrow{NGGTCN}NNN$	3.13	DR:2	13
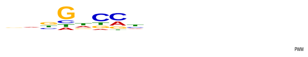	$NNNN\overrightarrow{NNGTCA}TTGGNNNNNN$	3.49	M	14
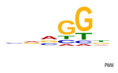	$CGTANNNNNNNNNNN\overrightarrow{AGGT}$	3.69	M	15
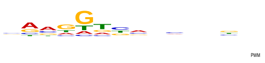	$NNNNNCA\overrightarrow{AGGTGA}CNNNNN$	8.46	M	16
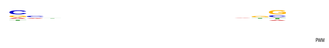	$\overleftarrow{GACCN}NNNNNNNNNN\overrightarrow{NGGTC}$	2.48	ER:10	17
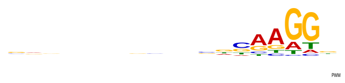	$AGAGNNNNNNNNNNCA\overrightarrow{AGGG}$	13.66	M	18

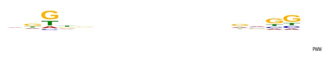	$CAAGNNNNNNNNNNNN\overrightarrow{CGGT}$	3.56	M	19
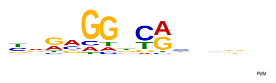	$NNNNNTAG\overrightarrow{AGGGC}ANNNNN$	5.85	M	20

45 ESRRA-Bhimsaria2022-Rnd3 (S07-157)

PWM	Seed Sequence	Seed Seq Enrichment	Repeat	Rank
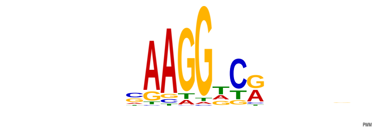	NNNNNNCAAGGTCGNNNNNN	84.61	M	1
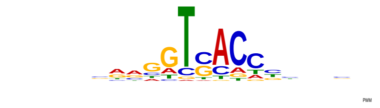	NNNNNNAGGTGACCNNNNNN	69.78	M	2
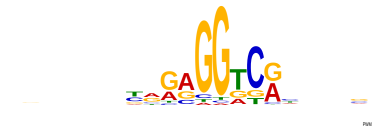	NNNNNNTAGAGGTCNNNNNN	38.49	M	3
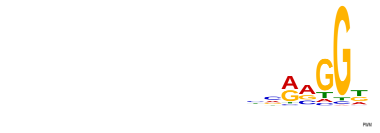	GGGGNNNNNNNNNNNNAGGT	41.88	M	4
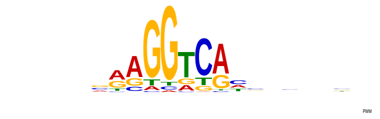	NNNNNNAGGTCACGNNNNNN	63.13	M	5
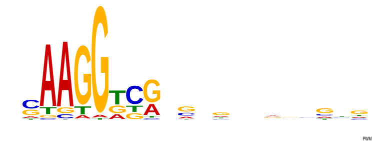	CAAGGTCNNNNNNNNNCGTG	225.34	M	6
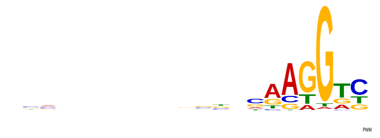	CCCCNNNNNNNNNCAAGGTC	207.06	M	7
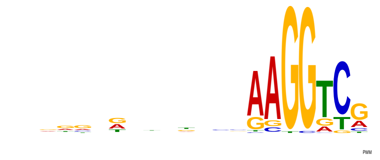	CGGGNNNNNNNNNAAGGTCG	185.66	M	8
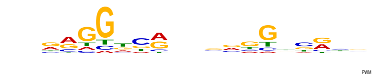	NNNGGTCNNNNNGGTCNNN	28.67	DR:3	9

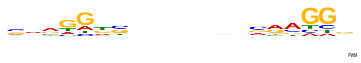	*CAAGGNNNNNNNNNNNAAGGG*	103.12	DR:8	10
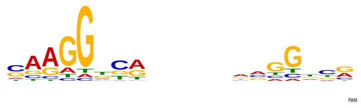	*CAAGGNNNNNNNNNNNGGTCG*	89.49	DR:6	11
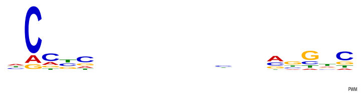	*ACCCNNNNNNNNNNNNNGGTC*	25.33	ER:11	12
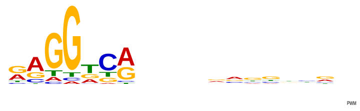	*NNGGTCNNNNNNNGGGCNN*	26.81	DR:5	13
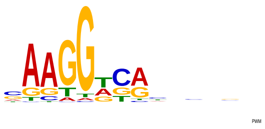	*NNNNCCAAGGNCACANNNN*	79.32	M	14
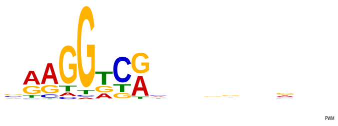	*NNNAGGTCNNNNGGGCNNN*	36.56	DR:2	15
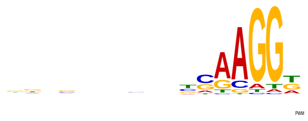	*GGGGNNNNNNNNNNGAAGGG*	92.84	M	16
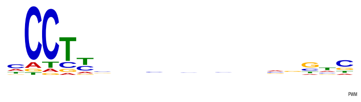	*CCCTTNNNNNNNNNNNGGTC*	70.84	ER:11	17
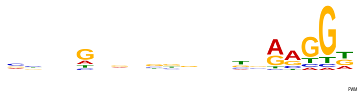	*CCTTNNNNNNNNNNNNNAGGA*	7.58	M	18

46 ESRRA-Jolma2013-Rnd2 (ERR195578)

PWM	Seed Sequence	Seed Seq Enrichment	Repeat	Rank
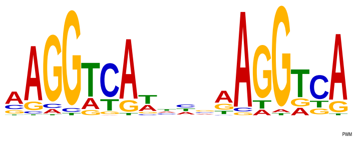	*NAGGTCNNNNNNAGGTCN*	58.41	DR:5	1
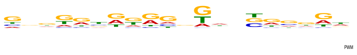	*NGGGGNNNNNNNNNNGGGGN*	8.67	M	2
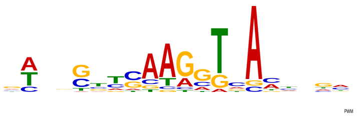	*NNNNNNTCAAGGTCNNNNNN*	5.40	DR:0	3
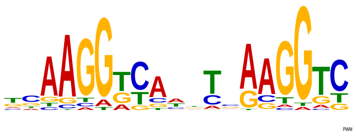	*NCAAGGNNNNNNNNAGGTN*	22.52	DR:5	4
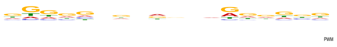	*NNGGGGNNNNNNNGGGGNN*	9.33	M	5
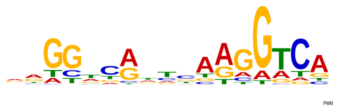	*NNAGGTNNNNNAAGGTNN*	6.80	DR:4	6
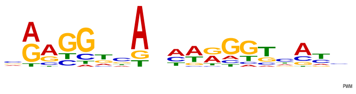	*NNNGGTCNNNNNGGTCNNN*	4.49	DR:3	7
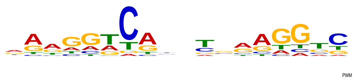	*NGAGGNNNNNNNNAGGTN*	5.69	DR:5	8
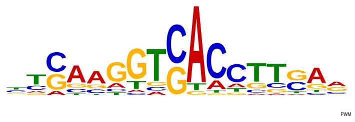	*NNNAAGGTNNCCTTNNN*	6.41	M	9

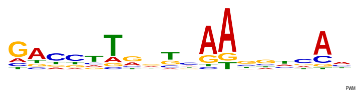	$\overleftarrow{NNCCT}TNNNNN\overrightarrow{NGGTCN}N$	2.64	ER:6	10
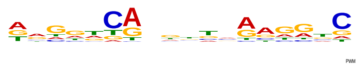	$N\overrightarrow{AGGTNN}NNNNNN\overrightarrow{AGGTN}$	3.54	DR:6	11
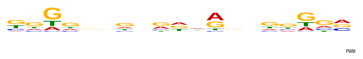	$NGGGANNNNNNNNNGGGGN$	6.02	M	12
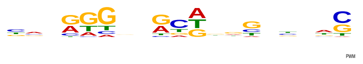	$NNN\overrightarrow{NGGTCN}NNGGGGNNNN$	4.15	M	13
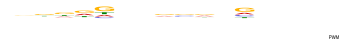	$N\overrightarrow{AGGGNN}NNNNNGTGGN$	3.19	M	14
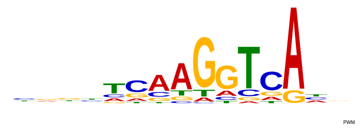	$NN\overrightarrow{NCTCA}\overrightarrow{AGGTCN}NN$	6.83	DR:0	15
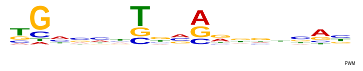	$\overleftarrow{NNACCT}NNNN\overrightarrow{NGGTCN}N$	1.96	ER:4	16
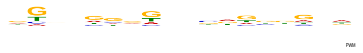	$NNG\overrightarrow{AGGNNN}NNNGGGGNN$	4.88	M	17
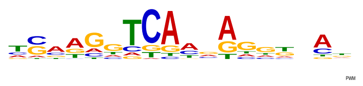	$NNA\overrightarrow{AGGNNN}NN\overrightarrow{NGGTCN}N$	2.88	DR:2	18

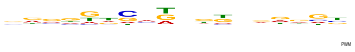	*NGGGGNNNNNNNNA*$\overrightarrow{AGGN}$	4.03	M	19
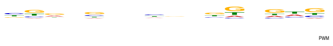	*NNGGAGNNNNNGTAGNN*	2.45	M	20

47 Esrra-Jolma2013-Rnd3 (ERR188728)

PWM	Seed Sequence	Seed Seq Enrichment	Repeat	Rank
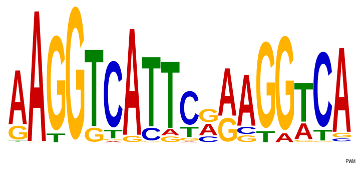	*AAGGTCANNNNNNGGTCA*	2627.49	DR:5	1
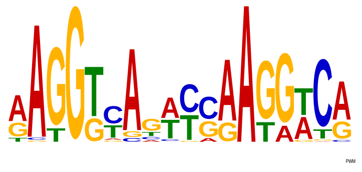	*AAGGNNNNNNCAAGGTCA*	1377.15	DR:5	2
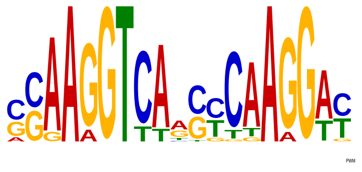	*NCAAGGTCANNNNAAGGTN*	3010.37	DR:5	3
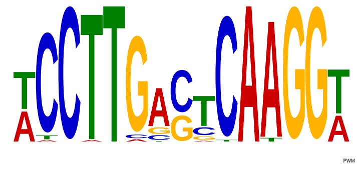	*NCCTTGANNCAAGGN*	210.91	M	4
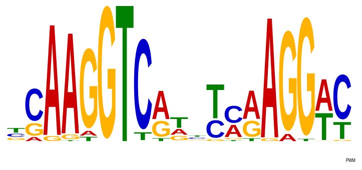	*NCAAGGTCNNNNNNAGGTN*	453.60	DR:5	5
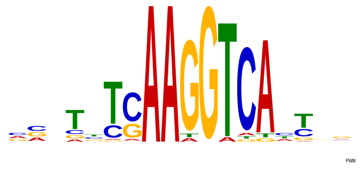	*NNNNTTCAAGGTCANNNN*	44.57	DR:0	6
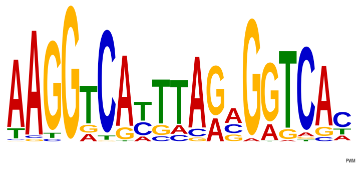	*AAGGNNNNNNNNNGGTCAT*	88.81	DR:5	7
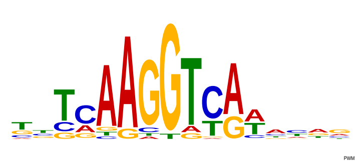	*NNNCAAGGTCAAANNN*	19.88	M	8
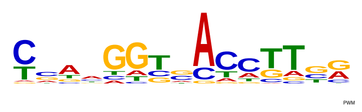	*NNAAGGTNNCCTTNN*	4.41	M	9

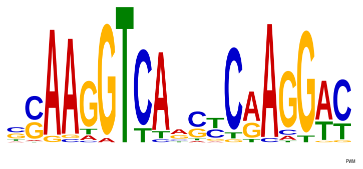	*NCAAGGNNNNNNNNAAGGTN*	58.81	DR:5	10
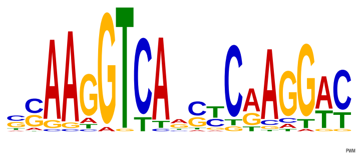	*NCAAGNNNNNNNNNAAGGTN*	18.81	M	11
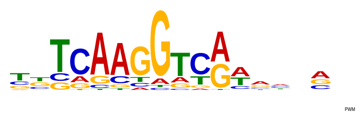	*NNNNAAGGTCAAANNNN*	8.10	M	12
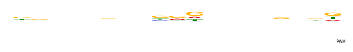	*NNNNGGGGNNNNGGGGNNNN*	4.21	M	13
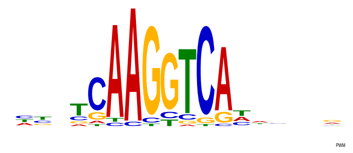	*NNNNNCAAGGTCATNNNNN*	20.19	M	14
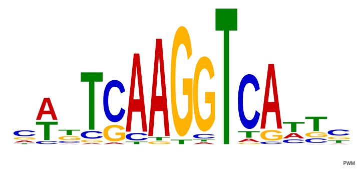	*NNTTCAANGTCATNN*	27.46	DR:0	15
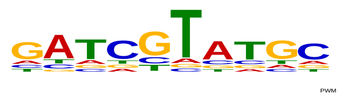	*GATCNNATGC*	4.56	M	16
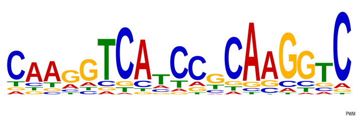	*NAAGGNNNNNNNNNAGGTN*	6.67	DR:6	17
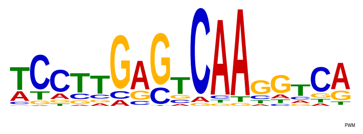	*NCCTTNNNNNNNGGTCN*	4.72	ER:7	18

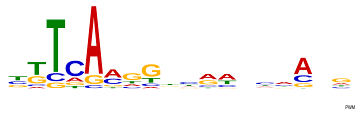	$NNNNN\overrightarrow{AGGTCA}AANNNNN$	2.87	M	19
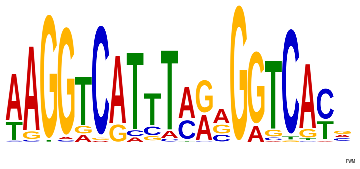	$N\overrightarrow{AGGTCN}NNNNNN\overrightarrow{NNNTCA}TN$	72.27	DR:5	20

48 ESRRA-Jolma2013-Rnd3 (ERR194308)

PWM	Seed Sequence	Seed Seq Enrichment	Repeat	Rank
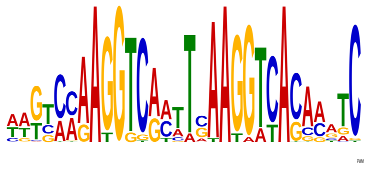	NNNNNNAAGGTCANNNCAAGGTCANNNNNN	35298.45	DR:5	1
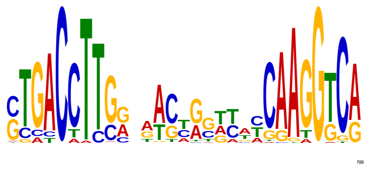	NTGACCTTGNNNNNNNNNNNCAAGGTCN	27324.20	ER:16	2
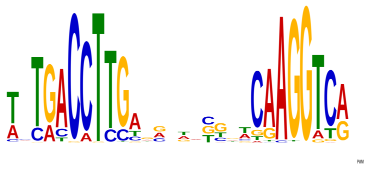	NNTGACCTTGNNNNNNNNNNAAGGTCNN	14322.74	ER:14	3
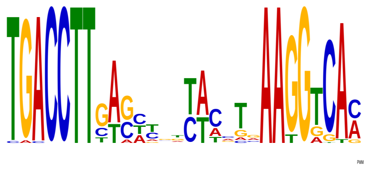	TGACCTTGNNNNNNNNNNNAAGGTCAA	52276.92	ER:15	4
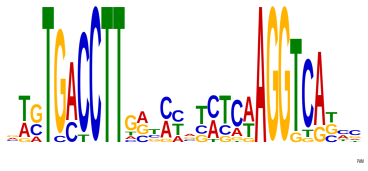	NNNTGACCTTGNNNNNNNNAAGGTCANNN	11982.40	ER:12	5
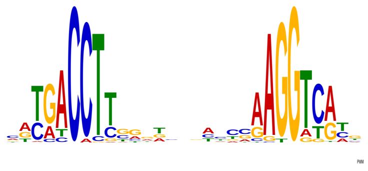	NNTGACCTTNNNNNNNNNNAAGGTCANN	7925.00	ER:13	6
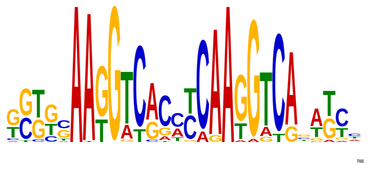	NNNNCAAGGTCNNNNNAAGGTCATNNNN	17074.90	DR:5	7
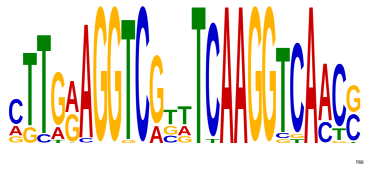	NNTGGAGGTCNNNNNAAGGTCAANN	16894.62	DR:5	8
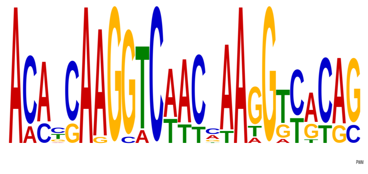	NNNCCAAGGTCNNNCAAGGTCANNN	13427.74	DR:4	9

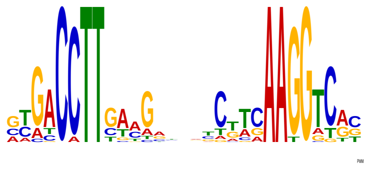	*NNGACCTTGNNNNNNNNNNNCAAGGTCNN*	9110.05	ER:15	10
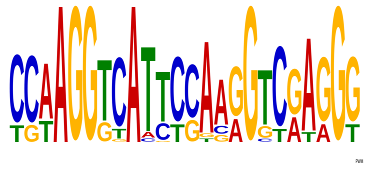	*NNNAGGTCATTNNAAGGTCGANNN*	19975.67	DR:5	11
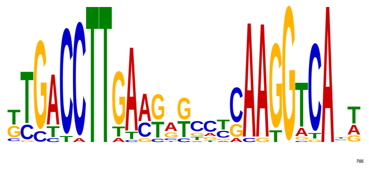	*NTGACCTTGNNNNNNNNNAAGGTCAAN*	16147.04	ER:12	12
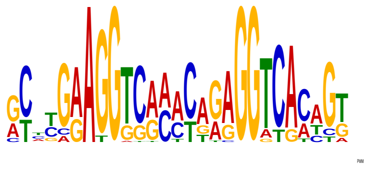	*NNNNNAAGGTCANNNAGAGGTCANNNNN*	10967.32	DR:5	13
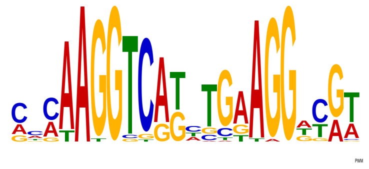	*NNCAAGGTCANNTGAAGGGCNN*	14956.07	DR:5	14
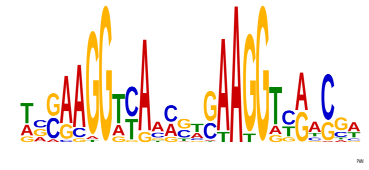	*NNNNAAGGTCANNNNCAAGGTCANNNN*	6840.16	DR:6	15
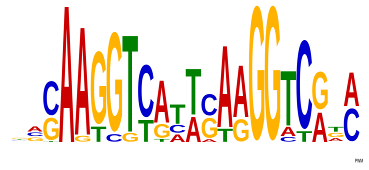	*NNCAAGGTCGNNCAAGGTCANN*	7796.99	DR:4	16
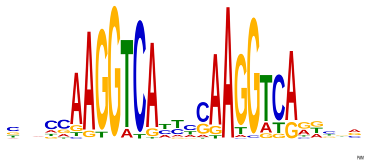	*NNNNNNAGGTCATNNNAAGGTCNNNNNN*	4075.72	DR:5	17
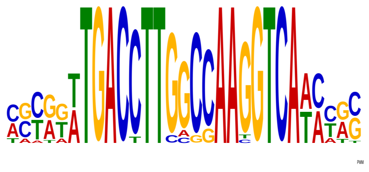	*NNNNNNTGACCTTGNNCAAGGTCNNNNNN*	12315.78	M	18

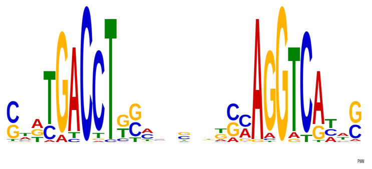	$NNN\overleftarrow{TGACCT}TNNNNNNNNNA\overrightarrow{AGGTCA}NNN$	4103.19	ER:11	19
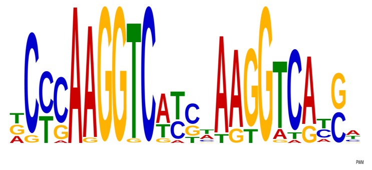	$NNCCA\overrightarrow{AGGTCN}NNNA\overrightarrow{AGGTCA}TNN$	14313.17	DR:4	20

49 ESRRA-Jolma2013-Rnd3 (ERR195579)

PWM	Seed Sequence	Seed Seq Enrichment	Repeat	Rank
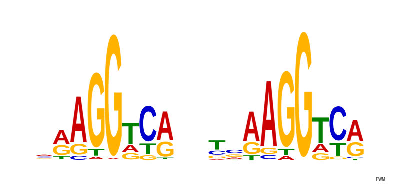	NNAGGTCNNNNNNAGGTCNN	3061.93	DR:5	1
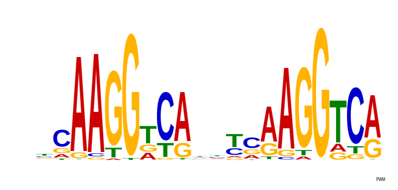	NCAAGGNNNNNNNNNGGTCN	883.32	DR:5	2
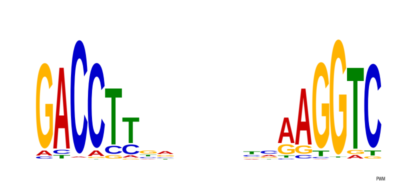	GACCNNNNNNNNNNNNGGTC	375.47	ER:10	3
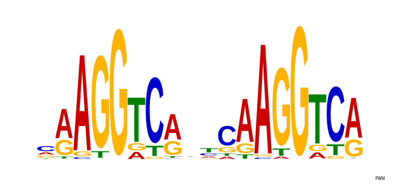	NNAGGTCNNNNNAGGTCNN	1320.27	DR:4	4
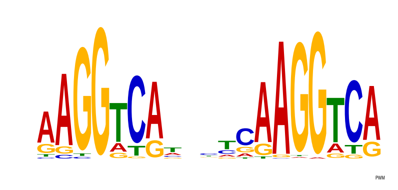	NAGGTCNNNNNNAGGTCN	768.53	DR:6	5
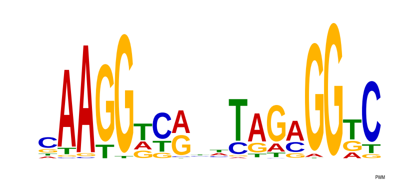	CAAGGNNNNNTAGAGGTC	12303.39	DR:5	6
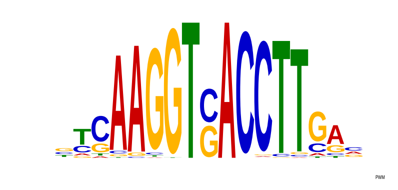	NNNCAAGGTNNCCTTGNNN	1830.00	M	7
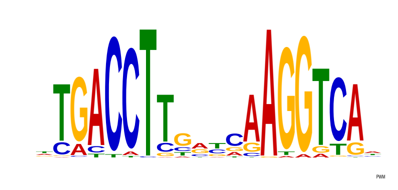	NNGACCTNNNNNNAGGTCNN	743.72	ER:6	8
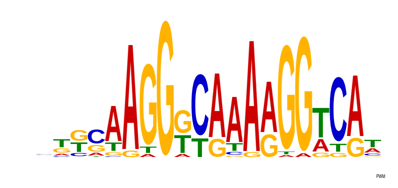	NNNCAAGGNCAAAAGGTNNN	8198.63	DR:2	9

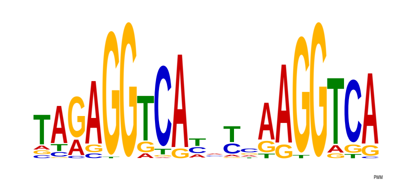	NAGAGGTCNNNNNNNGGTCN	3183.57	DR:5	10
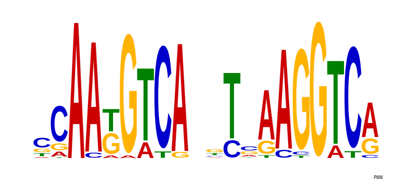	NCAATGTCANNNNNNNGGTCN	4680.13	DR:5	11
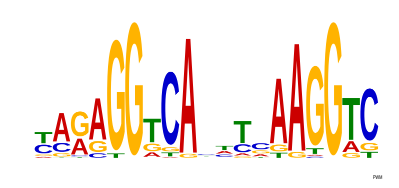	CAGAGGTCNNNNNNNGGTC	4471.58	DR:5	12
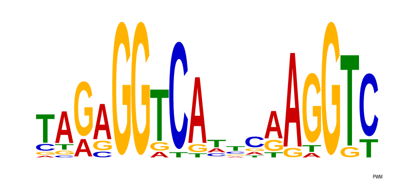	TAGAGGTCNNNNNAGGTC	7991.60	DR:4	13
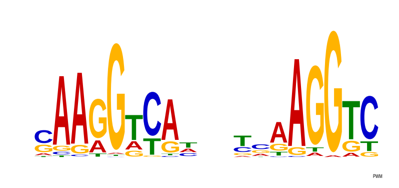	CAAGGNNNNNNNNNAGGTC	814.45	DR:6	14
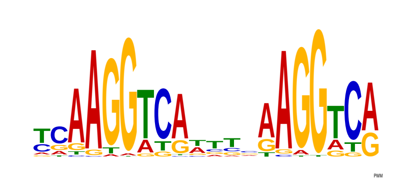	NGAAGGNNNNNNNNNNGGTCN	285.58	DR:5	15
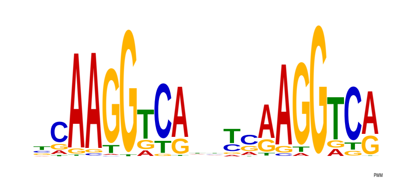	NCAAGNNNNNNNNNNGGTCN	271.08	M	16
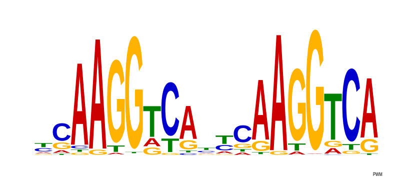	NCAAGGNNNNNNNNGGTCN	283.67	DR:4	17
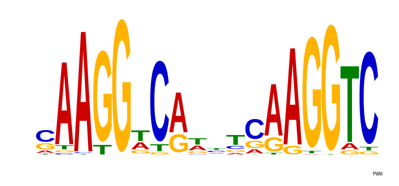	GAAGGACNNNNNNNGGTC	1604.24	DR:5	18

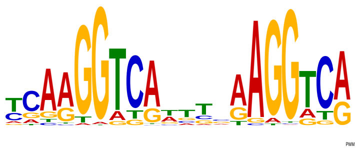	$NGAAGNNNNNNNNNN\overrightarrow{NGGTCN}$	79.13	M	19
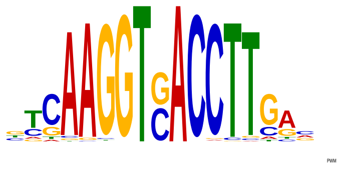	$NNNCA\overrightarrow{AGGNNN}CCTTGNNN$	793.62	M	20

50 Esrra-Jolma2013-Rnd4 (ERR188729)

PWM	Seed Sequence	Seed Seq Enrichment	Repeat	Rank
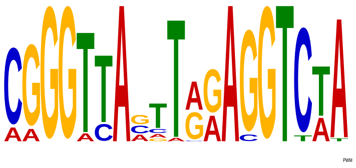	NNGGTCACANNNAGGTCNN	1057.41	DR:5	1
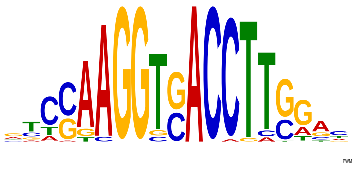	NNNNAAGGTNNCCTTNNNN	57.06	M	2
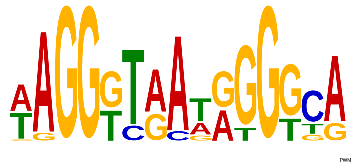	NAGGTCAANAGGTCN	447.42	DR:2	3
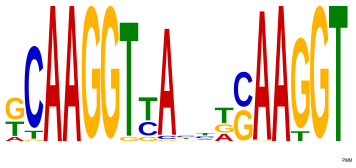	NCAAGGTTANNNNAAGGN	2751.72	DR:5	4
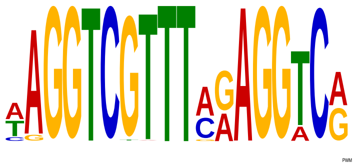	NNGGTCGTTTNNAGGTNN	6167.70	DR:5	5
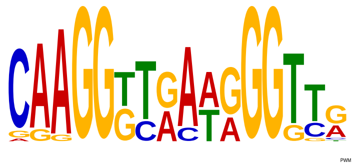	NNAGGTCNAAAGGTNN	180.90	DR:2	6
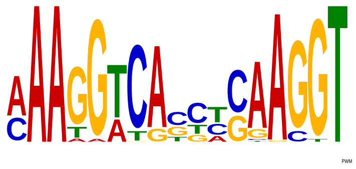	CAATGNNNNNNNAAGGT	169.35	M	7
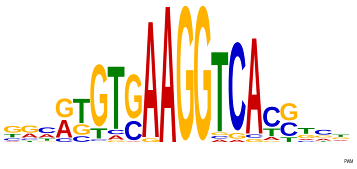	NNNNNTTCANNGTCANNNNN	34.71	DR:0	8
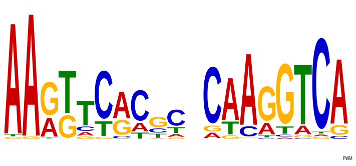	AAGGNNNNNNNNNNGTCA	51.78	DR:6	9

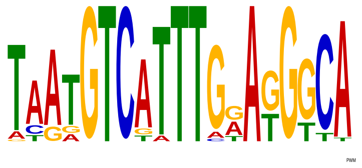	*NNNGGTCNTTTGAAGGNNN*	1126.03	DR:5	10
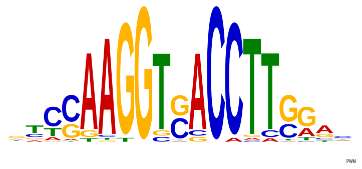	*NNNNAAGGNNNCCTTNNNN*	22.55	M	11
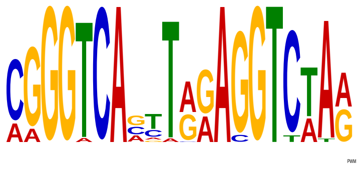	*NNNGTCACANNNAGGTCNNN*	817.98	DR:5	12
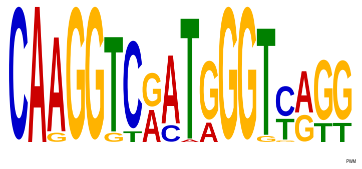	*NNNGGTCAANAGGTCNNN*	188.08	DR:2	13
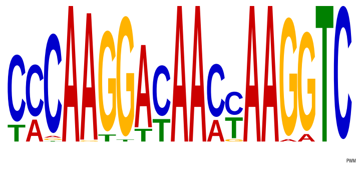	*NNCAAGGNNNNNCAAGGNN*	330.48	DR:4	14
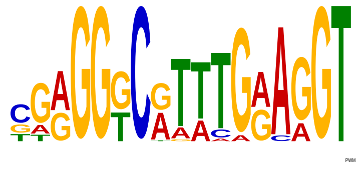	*NNNGGTCGTTTGAANNN*	267.30	IR:2	15
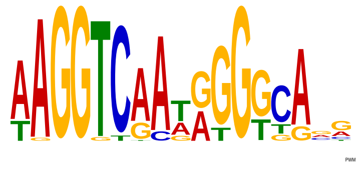	*NNNGTCAANAGGTCNNN*	163.14	DR:2	16
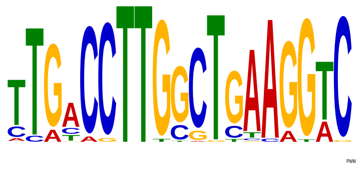	*NNGACCTNNNNNNAAGGNN*	47.04	ER:7	17
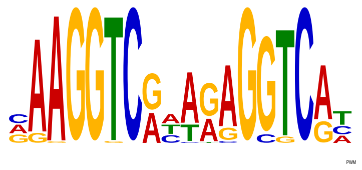	*NNAGGTCNNNAGGTCNN*	137.57	DR:3	18

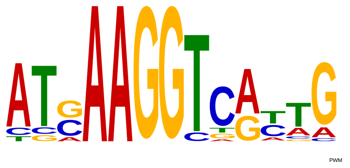	$NTGA\overrightarrow{AGNTC}ATAN$	33.02	M	19
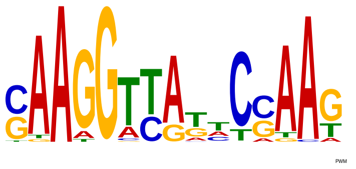	$NA\overrightarrow{AGGTN}NNA\overleftarrow{TGAA}N$	65.81	IR:2	20

51 ESRRA-Jolma2013-Rnd4 (ERR194309)

PWM	Seed Sequence	Seed Seq Enrichment	Repeat	Rank
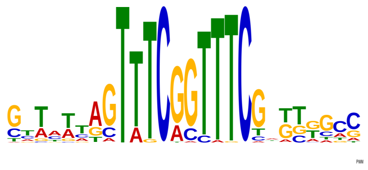	NNNNNNAGTTTCGGNTTCGANNNNNN	932.51	M	1
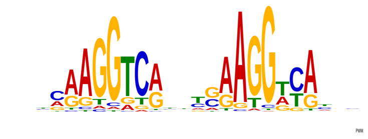	NNNNNNGGTCNNNNNAAGGNNNNNN	72.50	DR:5	2
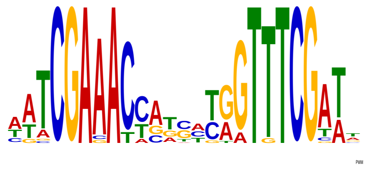	NNTCGAAANNNNNNNNNGTTTCGANN	480.07	M	3
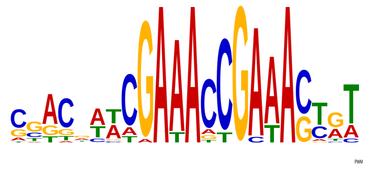	NNNNGATCGAANCCGAAANNNN	318.82	M	4
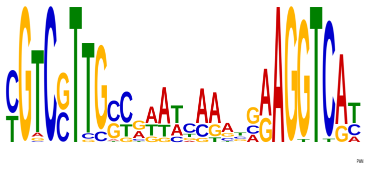	NGTCCTTGCNNNNNNNNNNNNNNGTCAN	170.10	ER:15	5
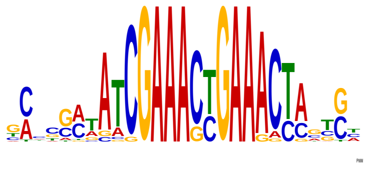	NNNNNNTATCGAAANTGAAACNNNNNN	904.83	M	6
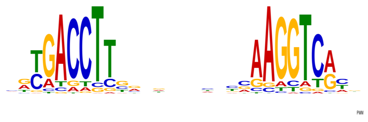	NNNNACCTNNNNNNNNNNNNNNAGGTNNNN	37.72	ER:13	7
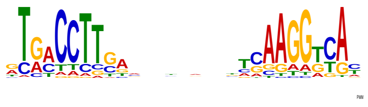	NNNNCCTTNNNNNNNNNNNNNNAAGGNNNN	30.23	ER:15	8
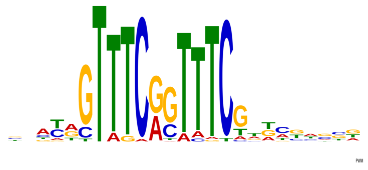	NNNNNNTTTCGGNTTCGATNNNNNN	179.70	M	9

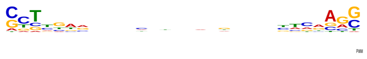	NCTTGNNNNNNNNNNNNNNNNNNNNCAAGN	17.37	M	10
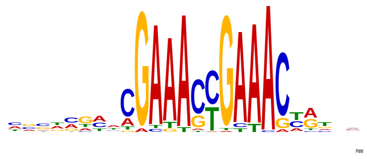	NNNNNNATCGANNCCGAAANNNNNN	65.83	M	11
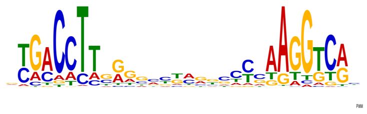	N$\overleftarrow{\text{NNACCT}}$NNNNNNNNNNNNNNNNN$\overrightarrow{\text{AGGTNN}}$N	24.92	ER:16	12
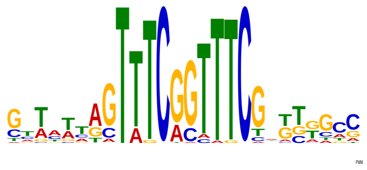	NNNNNN$\overrightarrow{\text{AGTTTC}}$GGNNTCGANNNNNN	300.66	M	13
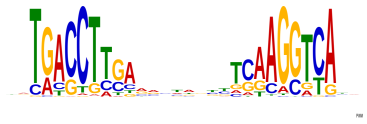	NN$\overleftarrow{\text{NNACCT}}$NNNNNNNNNNNNNN$\overrightarrow{\text{AGGTNN}}$NN	44.55	ER:14	14
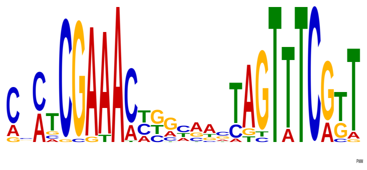	NNNTCGAAACNNNNNNNN$\overrightarrow{\text{AGTTTC}}$NNN	436.66	M	15
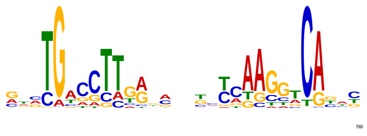	NNN$\overleftarrow{\text{NGACCN}}$NNNNNNNNNNNNN$\overrightarrow{\text{NGGTCN}}$NNNN	33.65	ER:12	16
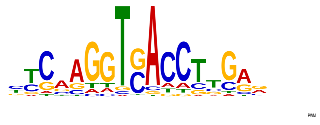	NNNNNNA$\overrightarrow{\text{AGGNNN}}$CCTTNNNNNN	10.98	M	17
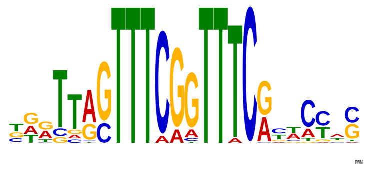	NNNNN$\overrightarrow{\text{AGTTTC}}G\overrightarrow{\text{GNTTCA}}$CNNNNN	225.48	DR:1	18

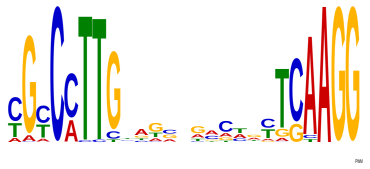	$\overleftarrow{NGTCCT}TGNNNNNNNN\overrightarrow{NNTTCA}AGN$	282.72	ER:10	19
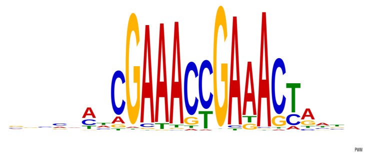	$NNNNNNTCGANNCCGAAANNNNNN$	46.56	M	20

52 ESRRA-Jolma2013-Rnd4 (ERR195580)

PWM	Seed Sequence	Seed Seq Enrichment	Repeat	Rank
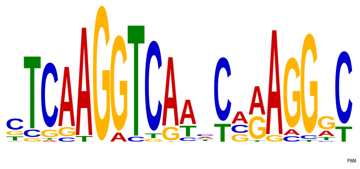	CTCANNNNNNNNNNNNGGGC	24.02	DR:11	1
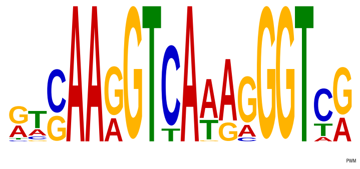	NTCAANNNNAAAGGGTCN	199.35	DR:8	2
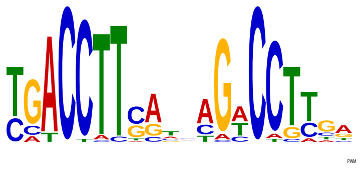	NNNNCTTCNNNNGTCCNNNN	20.48	M	3
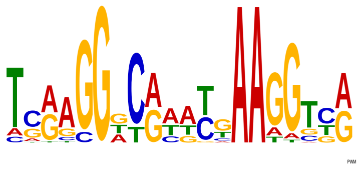	NGAAGNNNNNNNNNNGGTTN	8.44	M	4
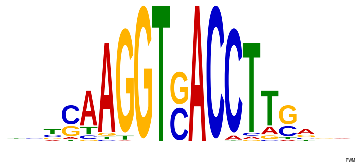	NNNCAAGNNNNNCTTGNNN	14.56	M	5
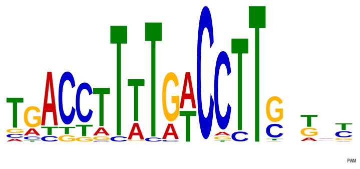	NNNNCTTTNNNNCTTGNNNN	14.65	M	6
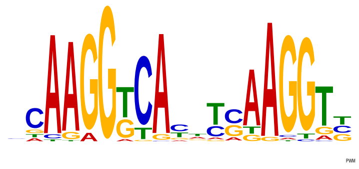	CCAANNNNNNNNNNNGGTT	12.96	M	7
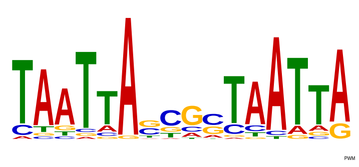	NAATTNNNNNTAATTN	14.37	M	8
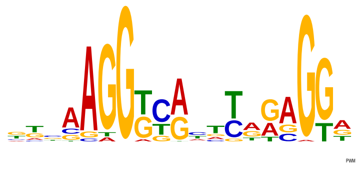	NNGAAGNNNNNNAGAGNN	14.24	M	9

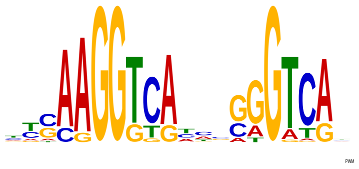	$NNCAAGNNNNNNN\overrightarrow{GGGTCN}N$	14.16	M	10
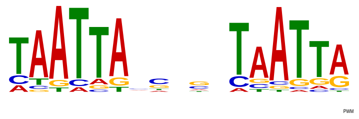	$NAATTNNNNNNTAATTN$	17.73	M	11
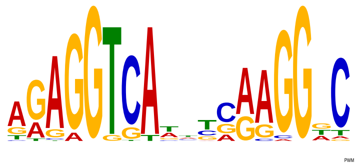	$AGAGNNNNNNNNN\overrightarrow{NGGGC}$	14.29	M	12
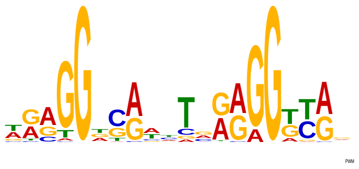	$AGAGNNNNNNNNN\overrightarrow{NNNTCA}A$	12.45	M	13
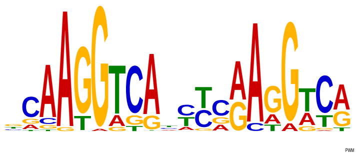	$GCAANNNNNNNNNN\overrightarrow{NNGTCA}$	17.10	M	14
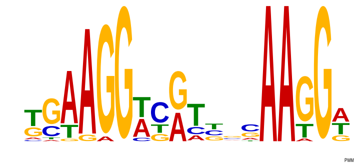	$NNGAAGNNNNNNGCAAGNN$	19.07	M	15
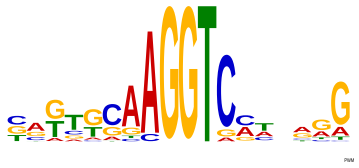	$NN\overrightarrow{NTTCA}NNNNNCATTNNN$	3.35	M	16
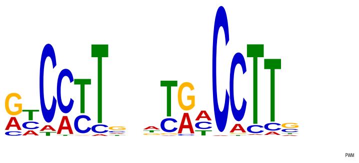	$NNNCTTGNNNNNNCTTGNNN$	16.19	M	17
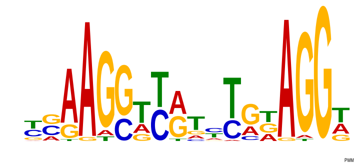	$NNGAAGNNNNNTGTAGNN$	21.61	M	18

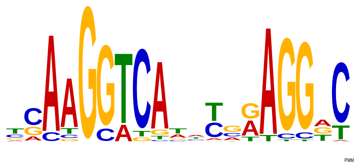	$CCAANNNNNNNNNNN\overrightarrow{NGGGC}$	11.56	M	19
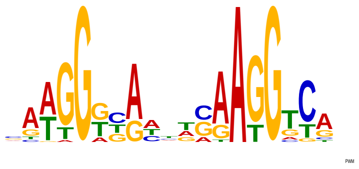	$GAAGNNNNNNNNN\overrightarrow{NNTCA}G$	11.26	M	20

53 ESRRA-Jolma2013-Rnd5 (ERR194310)

PWM	Seed Sequence	Seed Seq Enrichment	Repeat	Rank
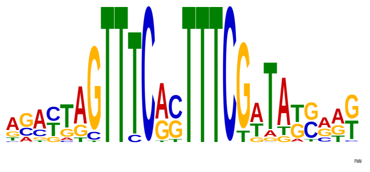	NNNNNNGTTTCAGTNTCGATNNNNNN	934.23	M	1
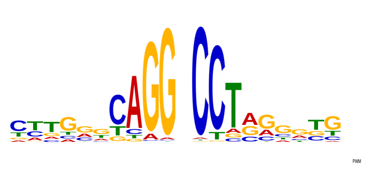	NNNNNNCAGGNCCTANNNNNN	74.67	M	2
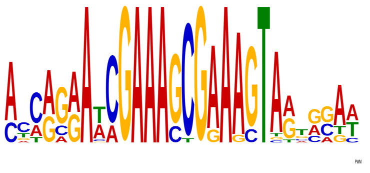	NNNNNNATCGANNNCGAAAGTANNNNNN	1866.47	M	3
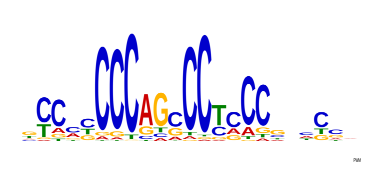	NNNNNNCCCAGNNCTCCCNNNNNN	143.69	M	4
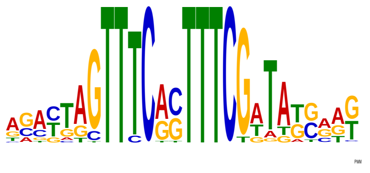	NNNNNNGTTTCAGTNNCGATNNNNNN	479.43	M	5
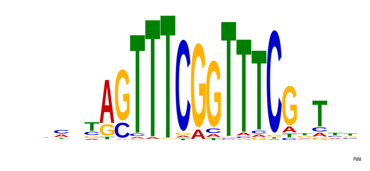	NNNNNNAGTTNNGGTTTNNNNNN	109.14	M	6
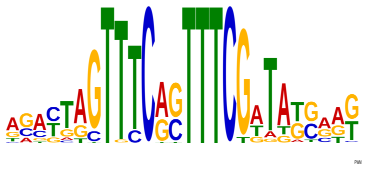	NNNNNNGTTTCAGNNNCGATNNNNNN	214.18	M	7
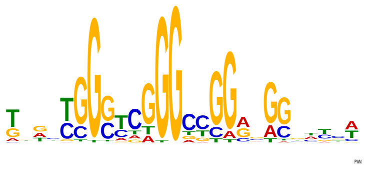	NNNNNGGGTCGGGNNNGGGGGNNNNN	814.71	M	8
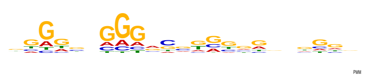	NNNNNNGGGANNNGGGGNNNNNN	27.08	M	9

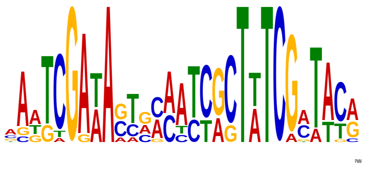	NNNTCGAAANNNNNNNNNNTTTCGATNNN	871.44	M	10
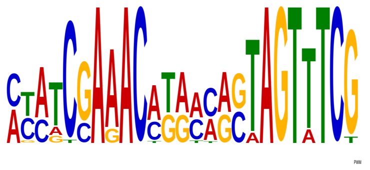	NNATCGAAACNNNNNNNNTAGTTTNN	1447.94	M	11
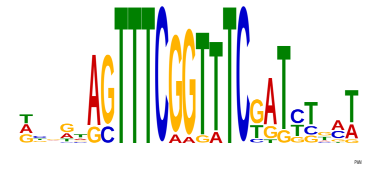	NNNNNNAGTTNNGGTTTCTANNNNNNN	265.63	M	12
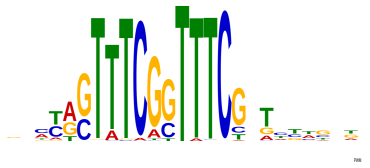	NNNNNNTTTCGNNNTCGATNNNNNN	116.30	M	13
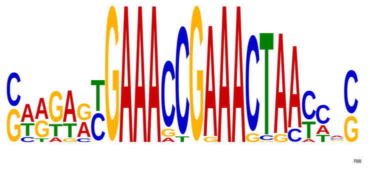	NNNNNGTGAAACCNNAACTANNNNN	982.81	IR:4	14
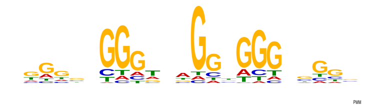	NNNNNNGGGTNNGGTGGNNNNNN	45.53	M	15
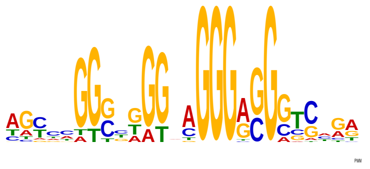	NNNNNGGGCGGNNAGGGAGGGNNNNN	870.18	DR:3	16
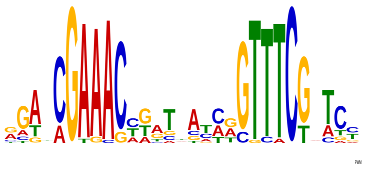	NNNNNNAAACTNNNNNNNAGTTTNNNNNN	32.73	ER:7	17
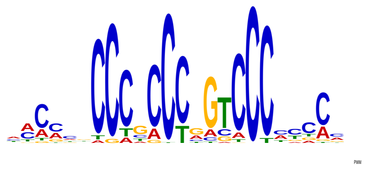	NNNNNNCCCGCCNNGTCCCNNNNNN	367.09	M	18

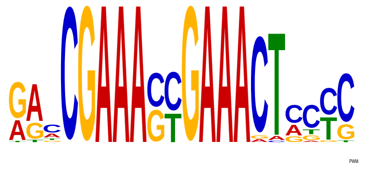	$NNCCGAANNNGA\overrightarrow{AACTCC}NN$	363.54	M	19
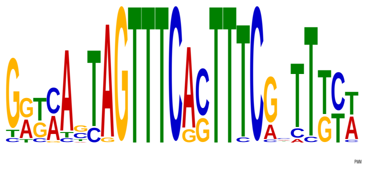	$NNNNNGT\overrightarrow{AGTTTN}NNTTTCGCNNNNN$	790.43	M	20

54 ESRRA-Jolma2013-Rnd6 (ERR194311)

PWM	Seed Sequence	Seed Seq Enrichment	Repeat	Rank
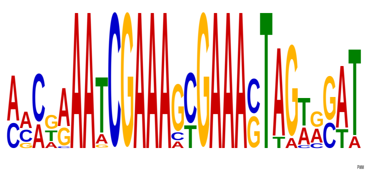	NNNNNNATCGAAAGNNAAAGTANNNNNN	6708.18	DR:0	1
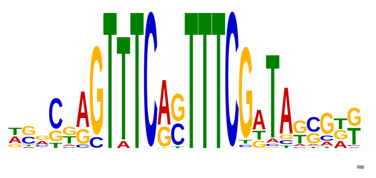	NNNNNNGTTTCAGNNNCGATNNNNNN	383.55	M	2
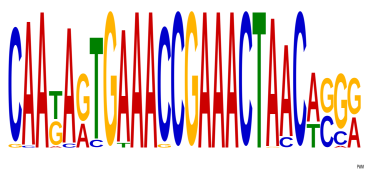	NNNNNNGTGAAACCNNAACTAANNNNNN	4979.76	IR:4	3
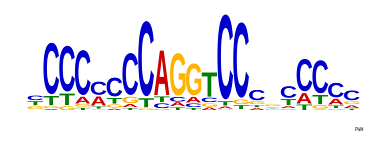	NNNNNNCCCAGNTCCCNNNNNN	100.84	M	4
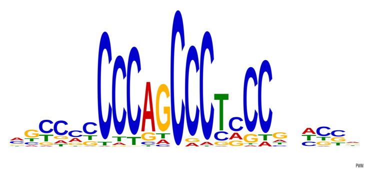	NNNNNNCCCAGNNCTCCCNNNNNN	313.43	M	5
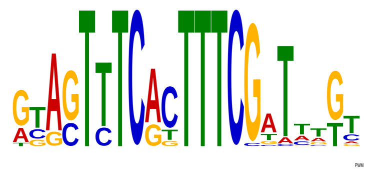	NNNGTTTCACTNNCGATTNNN	1372.03	M	6
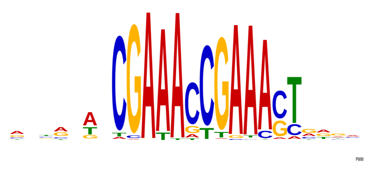	NNNNNNCCGANNNCGAAANNNNNN	98.01	M	7
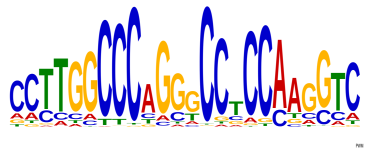	NNNNNNCCCAGNNCCTCCNNNNNN	262.51	M	8
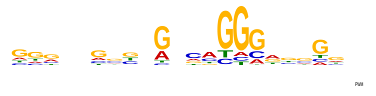	NNNNNNGGGGNNAGGGNNNNNN	33.51	M	9

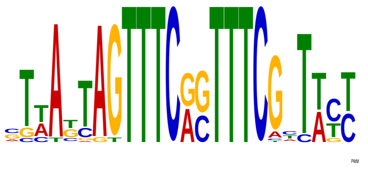	*NNNNGTAGTTTNNNTTTCGCNNNN*	970.06	M	10
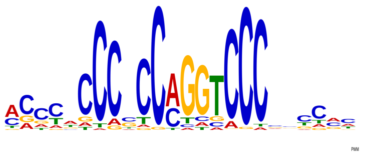	*NNNNNNCCGCCNNGTCCCNNNNNN*	290.83	M	11
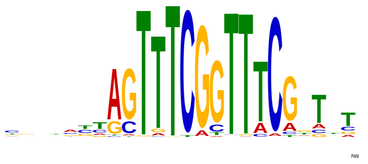	*NNNNNNTAGTTNNNGTTTNNNNNN*	98.72	M	12
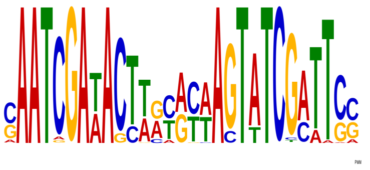	*NNATCGANNNNNNNNNNNNNNTCGATTNN*	701.45	M	13
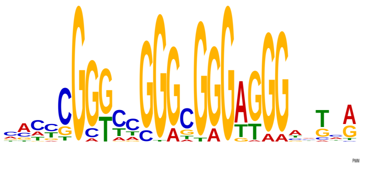	*NNNNNGGGCCGGNNNNGAGGGNNNNN*	498.36	DR:7	14
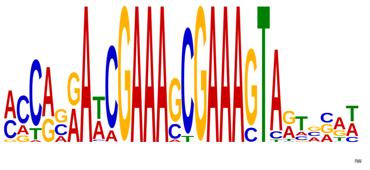	*NNNNNNATCGANNNCGAAAGTANNNNNN*	3098.18	M	15
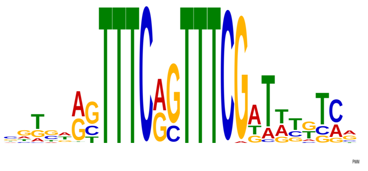	*NNNNNNGTTTCAGNNNCGTTNNNNNN*	178.15	M	16
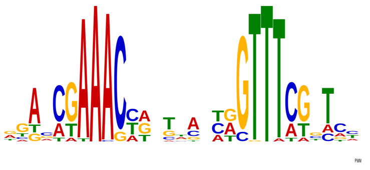	*NNNNNNAAACTNNNNNNNAGTTTNNNNNN*	60.84	ER:7	17
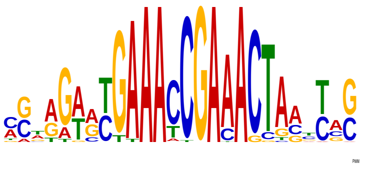	*NNNNNNNGTGAAACCNNAACTNNNNNN*	537.12	IR:4	18

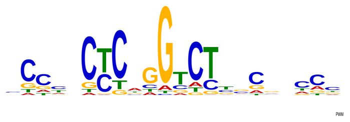	$NNNNNNCCC\overrightarrow{AGNNC}CCCNNNNNN$	57.51	M	19
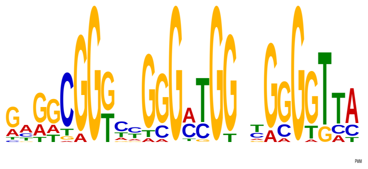	$NNNN\overrightarrow{NGGGCC}GGNNN\overrightarrow{NGGTGG}NNNNN$	238.41	DR:5	20

55 ESRRA-4-Bhimsaria2022-Rnd2 (S07-001)

PWM	Seed Sequence	Seed Seq Enrichment	Repeat	Rank
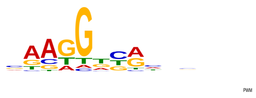	NNNNNNCAAGGTCANNNNNN	20.78	M	1
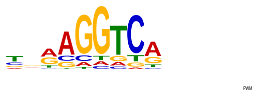	NNNNNNTGGAGGTCNNNNNN	10.36	M	2
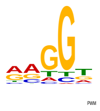	GGGGNNNNNNNNNNNNAGGT	10.51	M	3
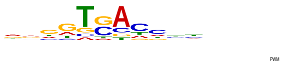	NNNNNAGGTNACCTNNNNN	12.53	M	4
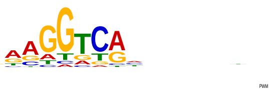	NNNNNNAGGTCATGNNNNNN	11.53	M	5
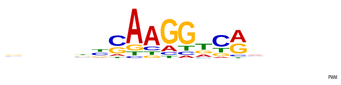	NNNNNNTCAAGGGCNNNNNN	8.75	DR:0	6
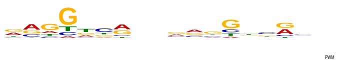	NNNGGTCNNNNNGGTCNNN	6.55	DR:3	7
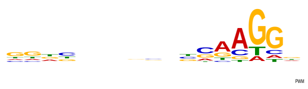	CAAGNNNNNNNNNNNNAAGGG	12.04	M	8
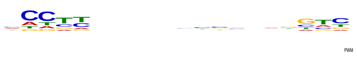	CCCTNNNNNNNNNNNNGGTC	7.59	ER:11	9

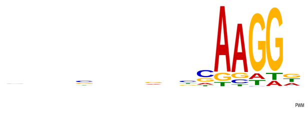	$GGGGNNNNNNNNNNNCA\overrightarrow{AGGG}$	27.65	M	10
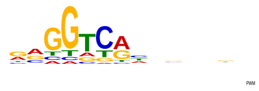	$NNNNNNG\overrightarrow{GGGTCA}TNNNNNN$	9.17	M	11
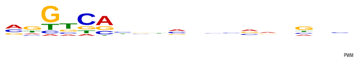	$\overrightarrow{AGGTCN}NNNNNNNNN\overrightarrow{NGGTC}$	6.74	DR:8	12
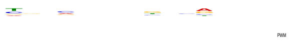	$\overleftarrow{ACCT}NNNNNNNNNNNNN\overrightarrow{AGGT}$	6.57	ER:12	13
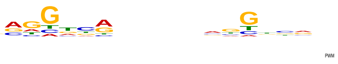	$N\overrightarrow{NGGTCN}NNNNN\overrightarrow{NGGTCN}N$	6.91	DR:5	14
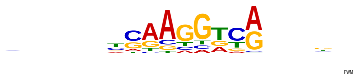	$NNNNNNTGA\overrightarrow{AGGGCN}NNNNN$	5.26	M	15
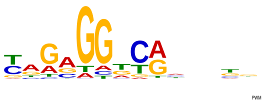	$NNNNNTAG\overrightarrow{AGGGCA}NNNNN$	9.02	M	16
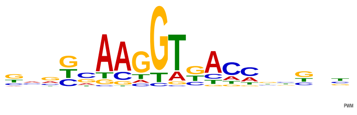	$NNNNCA\overrightarrow{AGGTGA}CCGNNNN$	50.68	M	17
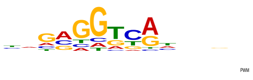	$NNNNNNTAG\overrightarrow{GGGTCN}NNNNN$	4.84	M	18

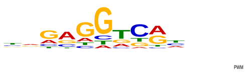	NNNNNTTAG$\overrightarrow{NGGTC}$NNNNN	5.35	M	19
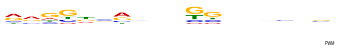	NNN$\overrightarrow{GGTCN}$NNNN$\overrightarrow{NGTCA}$NNN	5.15	DR:2	20

56 ESRRA-4-Bhimsaria2022-Rnd3 (S07-122)

PWM	Seed Sequence	Seed Seq Enrichment	Repeat	Rank
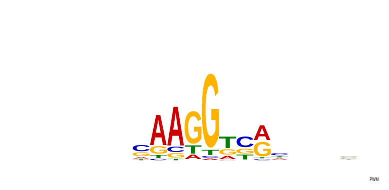	NNNNNNCAAGGTCANNNNNN	70.56	M	1
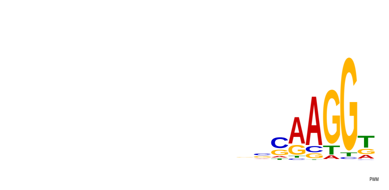	GGGGNNNNNNNNNNCAAGGT	191.87	M	2
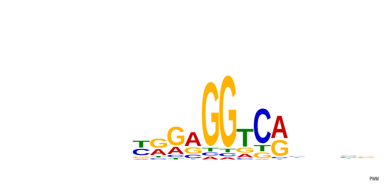	NNNNNNTAGAGGTCNNNNNN	29.17	M	3
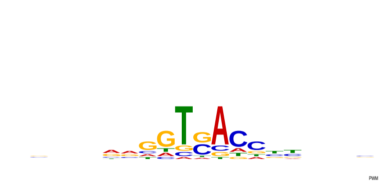	NNNNNAGGTNACCTNNNNN	47.43	M	4
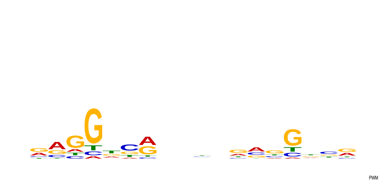	NNGGTCNNNNNNNGGTCNN	22.81	DR:5	5
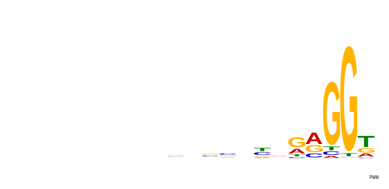	GGGGNNNNNNNNNNAGAGGT	74.34	M	6
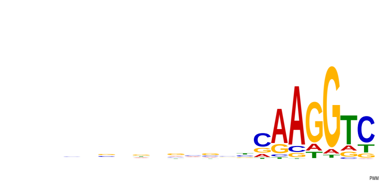	CCCCNNNNNNNNNNCAAGGTC	167.73	M	7
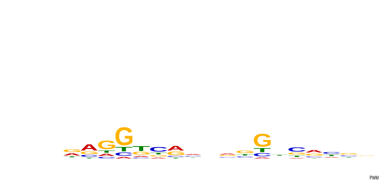	NNNNGGTCNNNNGGTCNNNN	17.16	DR:2	8
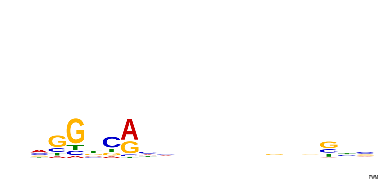	AGGTCNNNNNNNNNGGTC	19.00	DR:8	9

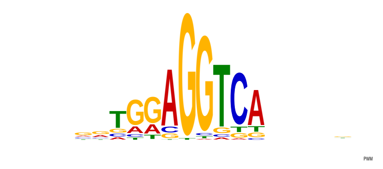	NNNNNTGGANGTCAGNNNNNN	50.59	M	10
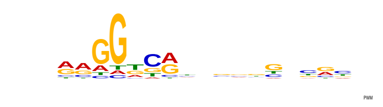	NNNAGGTCNNNNNGGTCNNN	29.48	DR:3	11
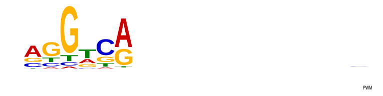	AGGTCNNNNNNNNNNCCCC	21.45	M	12
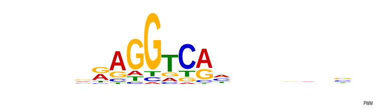	NNNNNNGGTCACGGNNNNNN	23.96	M	13
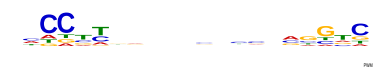	CCCTNNNNNNNNNNNNGGTC	23.17	ER:11	14
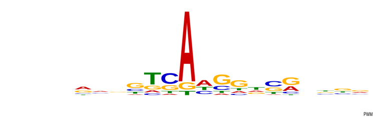	NNNNNGGTCAAGGGCNNNNN	55.08	DR:0	15
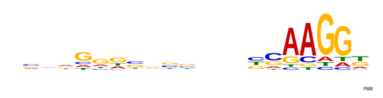	CAAGNNNNNNNNNNCAAGGG	65.79	M	16
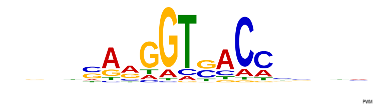	NNNCAAGGTGACCCCNNN	298.45	M	17

57 ESRRA-Yin2017-Rnd2 (ERR1003021)

PWM	Seed Sequence	Seed Seq Enrichment	Repeat	Rank
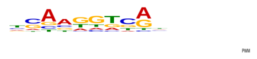	NNNNNNNTTCANNGTCANNNNNNN	5.79	DR:0	1
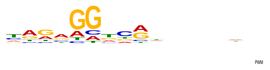	NNNNNNTAGANNTCATNNNNNN	4.19	M	2
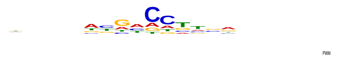	NNNNNNAACGNNNTTCANNNNNNN	3.87	M	3
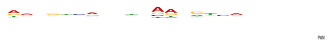	NNNNNNGTCANNNNNNNNGTCANNNNNNN	3.29	DR:5	4
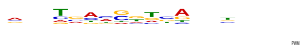	NNNNNNTTGANNNTCAANNNNNNN	4.18	M	5
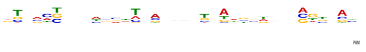	NNNNNNTTGANNNNNNNNNNNNNNNNNTCAANNNNNNN	2.43	ER:10	6
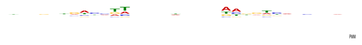	NNNNNNTGACNNNNNNNNNNNNNNNNGTCANNNNNNN	3.17	ER:11	7
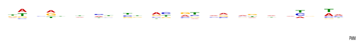	NNNNNNTGACNNNNNNNNNNNNNNNNNNGTCANNNNNNN	2.55	ER:13	8
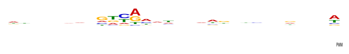	NNNNNNCAAGNNNNNNNNNNNNNNGTCANNNNNNN	2.53	M	9

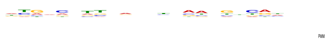	NNNNNNACCTNNNNNNNNAGGTNNNNNN	2.94	ER:8	10
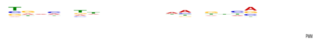	NNNNNNACCTNNNNNNNAGGTNNNNNN	2.59	ER:7	11
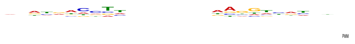	NNNNNNACCTNNNNNNNNNAGGTNNNNNN	3.29	ER:9	12
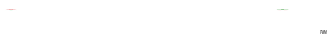	NNNNNNGTCANNNNNNNNNNNTGACNNNNNN	2.13	IR:10	13
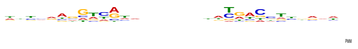	NNNNNNAGGTNNNNNNNNNNNNNNACCTNNNNNN	3.01	IR:10	14
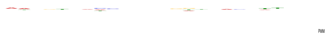	NNNNNNGTCANNNNNNNNNTGACNNNNNN	2.57	IR:8	15
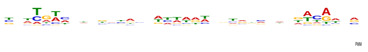	NNNNNNCCTTNNNNNNNNNNNNNNNNNNNAAGGNNNNNN	2.00	ER:20	16
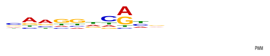	NNNNNNCAAGNNCGCGNNNNNN	3.06	M	17
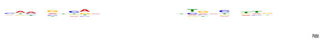	NNNNNNAGGTNNNNNNNNNNNNNNACCTNNNNNN	2.36	IR:9	18

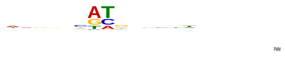	NNNNNNAAGGNNNNNNCCTTNNNNNN	2.36	IR:0	19
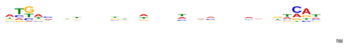	NNNNNNCCTTNNNNNNNNNNNNNNNNNAAGGNNNNNN	2.54	ER:19	20

58 ESRRA-Yin2017-Rnd3 (ERR1003022)

PWM	Seed Sequence	Seed Seq Enrichment	Repeat	Rank
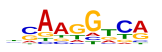	NNNNNNNTCAANNTCATNNNNNN	5.41	DR:0	1
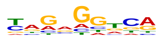	NNNNNNTAGANGTCANNNNNN	3.56	M	2
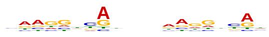	NNNNNNAAGGNNNNNNNAAGGNNNNNNN	3.64	DR:5	3
	NNNNNNTGACNNNNNNNNNNNNNGTCANNNNNN	3.25	ER:9	4
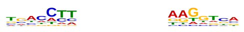	NNNNNNTGACNNNNNNNNNNNNNNNGTCANNNNNN	3.36	ER:11	5
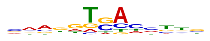	NNNNNNAAGGNNNCCTTNNNNNN	2.88	M	6
	NNNNNNTGACNNNNNNNNNNNNNNGTCANNNNNN	2.93	ER:10	7
	NNNNNNTTGANNNTCAANNNNNN	3.48	M	8
	NNNNNNGGGGNNNNNGGGGNNNNNN	2.64	M	9

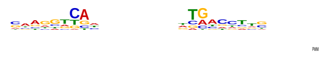	NNNNNNAAGGNNNNNNNNNNNNNNNNNNCCTTNNNNNN	2.96	IR:10	10
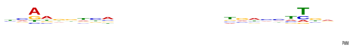	NNNNNNGTCANNNNNNNNNTGACNNNNNNN	2.86	IR:9	11
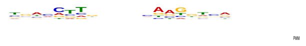	NNNNNNTGACNNNNNNNNNNNNGTCANNNNNN	2.78	ER:8	12
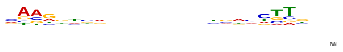	NNNNNNGTCANNNNNNNNTGACNNNNNNN	2.78	IR:8	13
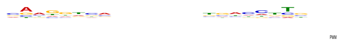	NNNNNNGTCANNNNNNNTGACNNNNNNN	2.78	IR:7	14
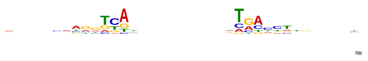	NNNNNNAAGGNNNNNNNNNNNNNNNNNNNCCTTNNNNNN	2.53	IR:11	15
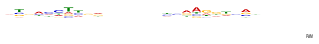	NNNNNNTGACNNNNNNNNNNNNNNNNGTCANNNNNN	2.60	ER:12	16
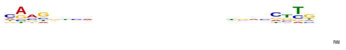	NNNNNNGTCANNNNNNNNNNNNTGACNNNNNNN	2.46	IR:12	17
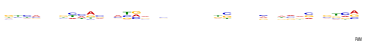	GTCANNNNNNNNNNNNNNNNNNNNNNNNNNNNNNNGTCA	2.33	DR:29	18

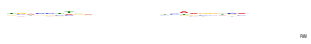	$NNNNNN\overleftarrow{TGACNN}NNNNNNNNNNNNN\overrightarrow{NNGTCA}NNNNNN$	2.49	ER:13	19
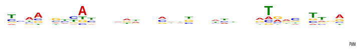	$NNNA\overrightarrow{AGGNNN}NNNNNNNNNNNNNNNNNNNN\overleftarrow{NNNCCT}TNNN$	2.55	IR:20	20

59 ESRRA-Yin2017-Rnd3 (ERR1003023)

PWM	Seed Sequence	Seed Seq Enrichment	Repeat	Rank
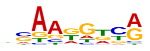	NNNNNNTCAANNTCATNNNNNN	8.12	DR:0	1
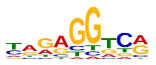	NNNNNNTAGANNTCATNNNNNN	6.45	M	2
	NNNNNNGTCANNNNNNNNGTCANNNNNN	5.68	DR:5	3
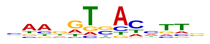	NNNNNNAAGGNNNCCTTNNNNNN	5.24	M	4
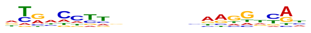	NNNNNNCCTTNNNNNNNAAGGNNNNNN	5.46	ER:9	5
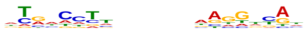	NNNNNNCCTTNNNNNNAAGGNNNNNN	5.67	ER:8	6
	NNNNNNGGTCNNGACCNNNNNN	4.18	IR:0	7
	NNNNNNTGACNNNNNNNNNNNNNNGTCANNNNNN	3.89	ER:10	8
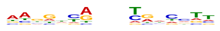	NNNNNNAAGGNNNNNNNNNCCTTNNNNNN	4.63	IR:3	9

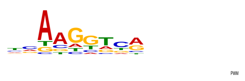	NNNNNNGTTCNNNGTCANNNNNNN	4.70	DR:0	10
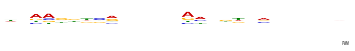	NNNNNNGTCANNNNNNNNGTCANNNNNN	3.96	DR:6	11
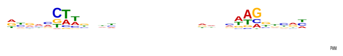	NNNNTGACNNNNNNNNNNNNNNNNNNNNNNNNGTCANNNN	3.36	ER:19	12
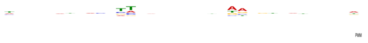	NNNNNNTGACNNNNNNNNNNNNNNNGTCANNNNNN	3.38	ER:11	13
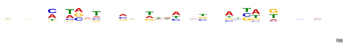	NNNTGACNNNNNNNNNNNNNNNNNNNNNNNNNNNGTCANNN	2.77	ER:22	14
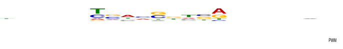	NNNNNNTTGANNNTCAANNNNNNN	5.04	M	15
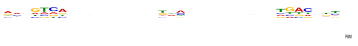	NNNNGTCANNNNNNNNNNNNNNNNNNNNNNNTGACNNNN	4.49	IR:23	16
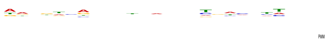	NNNNNNGTCANNNNNNNNNTGACNNNNNN	3.93	IR:9	17
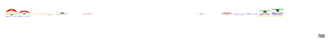	NNNNNNGTCANNNNNNNNTGACNNNNNN	3.20	IR:8	18

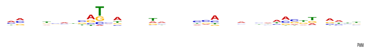	$N\overrightarrow{NNGTCA}NNNNNNNNNNNNNNNNNNNNNNNN\overrightarrow{NNGTCA}NNN$	3.45	DR:24	19
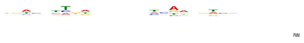	$NNNNNN\overleftarrow{TGACNN}NNNNNNNNNNNN\overrightarrow{NNGTCA}NNNNNN$	3.75	ER:12	20

60 ESRRA-Yin2017-Rnd4 (ERR1003024)

PWM	Seed Sequence	Seed Seq Enrichment	Repeat	Rank
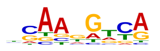	NNNNNNTCAANNTCATNNNNNN	6.14	DR:0	1
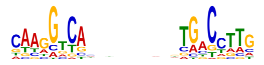	NNNNNNCAAGNNNNNNNNNNNNNNNNNNCTTGNNNNNN	4.80	M	2
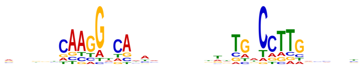	NNNNNNCAAGNNNNNNNNNNNNNNNNNNNCTTGNNNNNN	6.13	M	3
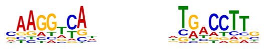	NNNNNNAAGGNNNNNNNNNNNNNNNCCTTNNNNNN	5.47	IR:9	4
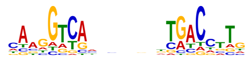	NNNNNNCAAGNNNNNNNNNNNNNNNNCTTGNNNNNN	4.21	M	5
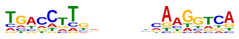	NNNNNNTGACNNNNNNNNNNNNNNGTCANNNNNN	5.08	ER:10	6
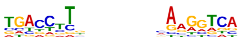	NNNNNNTGACNNNNNNNNNNNNNNNGTCANNNNNN	5.32	ER:11	7
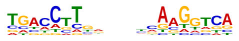	NNNNNNTGACNNNNNNNNNNNNNGTCANNNNNN	4.89	ER:9	8
	NNNNNNGGGGNNNNNGGGGNNNNNN	3.81	M	9

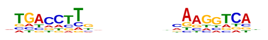	NNNNNNTGACNNNNNNNNNNNNNNNNNGTCANNNNNNN	5.02	ER:12	10
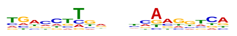	NNNNNNTGACNNNNNNNNNNNNNGTCANNNNNNN	3.73	ER:8	11
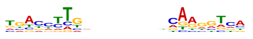	NNNNNNTGACNNNNNNNNNNNNNNNNNNGTCANNNNNNN	3.87	ER:13	12
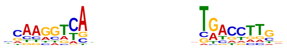	NNNNNNAAGGNNNNNNNNNNNNNNNNNCCTTNNNNNN	3.56	IR:12	13
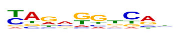	NNNNNNCAGANGTCANNNNNNN	3.21	M	14
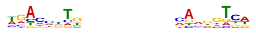	NNNNNNTGACNNNNNNNNNNNNNNNNNNNGTCANNNNNNN	3.96	ER:14	15
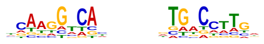	NNNNNNCAAGNNNNNNNNNNNNNNNNNCTTGNNNNNN	3.99	M	16
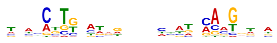	NNNNNTGACNNNNNNNNNNNNNNNNNNNNNNNGTCANNNNNN	2.82	ER:18	17
	NNNNNNGTCANNNNNNNNNNNNNNNTGACNNNNNN	3.71	IR:15	18

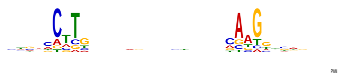	$NNNN\overleftarrow{TGACNN}NNNNNNNNNNNNNNNNNNN\overrightarrow{NNGTCA}NNNN$	2.98	ER:19	19
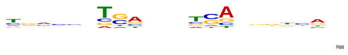	$NNNNNNTTGA\overrightarrow{NNNTCA}ANNNNNNN$	4.66	M	20

61 ESRRA-Yin2017-Rnd4 (ERR1003025)

PWM	Seed Sequence	Seed Seq Enrichment	Repeat	Rank
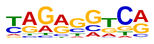	NNNNNNTAGANNTCATNNNNNN	6.86	M	1
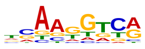	NNNNNNCTCANNNTCATNNNNNN	5.60	DR:0	2
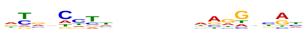	NNNNNNCCTTNNNNNNAAGGNNNNNN	4.87	ER:8	3
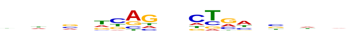	NNNNNNTCAGNNCTGANNNNNN	4.20	IR:4	4
	NNNNNNAGGTNNNNNNNNGTCANNNNNN	4.34	DR:5	5
	NNNNNNAAGGNNNCCTTNNNNNN	4.16	M	6
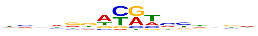	NNNNNNAGGANNTCCTNNNNNN	3.26	M	7
	NNNNNNGGTCNNGACCNNNNNN	3.81	IR:0	8
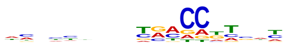	NNNNNNATTTNNNNTTCANNNNNN	3.74	M	9

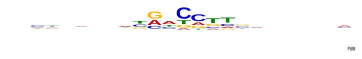	*NNNNNNGAATNNNNNTTCANNNNNN*	3.96	M	10
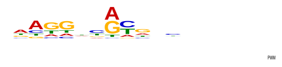	*NNNNNNAAGGNNNCGACNNNNNN*	4.10	IR:0	11
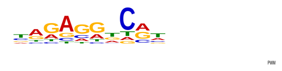	*NNNNNNTAGANNNCGTTNNNNNN*	4.65	M	12
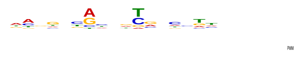	*NNNNNNAAGGNNNNNNNNNCCTTNNNNNN*	3.59	IR:3	13
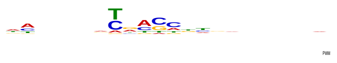	*NNNNNNTCGTNNNNNTTCANNNNNN*	2.56	M	14
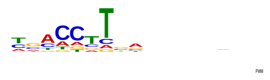	*NNNNNNGTGANNNNNGAACNNNNNN*	3.45	IR:3	15
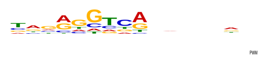	*NNNNNNTTAGNNNTCAANNNNNN*	4.37	M	16
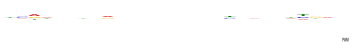	*NNNNNNGTCANNNNNNNNNTGACNNNNNN*	3.04	IR:9	17
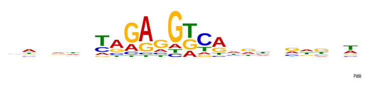	*NNNNNNTAGANNNCAAGTNNNNNN*	5.96	M	18

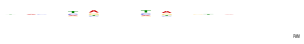	NNNNNNTGACNNNNNNNNNNNNNNGTCANNNNNN	2.97	ER:10	19
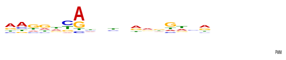	NNNNNNCAAGNNNNNNNNNNNGTCANNNNNNN	4.15	M	20

62 ESRRA-Yin2017-Rnd2 (ERR1003028)

PWM	Seed Sequence	Seed Seq Enrichment	Repeat	Rank
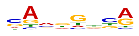	NNNNNNCTCANNGTCANNNNNN	4.14	DR:0	1
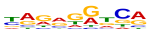	NNNNNNTAGANNTCATNNNNNN	3.91	M	2
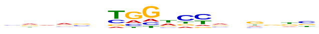	NNNNNNGTGGTCCANNNNNN	3.07	M	3
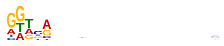	GGTCNNNNNNNNNNNNNNNNNNNNNNNNNNNNNNNNGATG	2.91	M	4
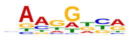	NNNNNNCAAGNNNAAACNNNNNN	3.12	M	5
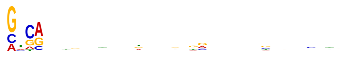	NTCATNNNNNNNNNNNNNNNNNNNNNNNNNNNNNGTGGN	2.70	M	6
	NNNNNNCAGGNCCTANNNNNN	1.95	M	7
	NNNNNNATGANNNTCATNNNNNN	2.93	M	8
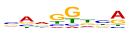	NNNNNNCCAANNNCACGNNNNNN	2.65	M	9

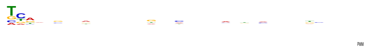	TCAANNNNNNNNNNNNNNNNNNNNNNNNNNNNNNGGTA	2.39	M	10
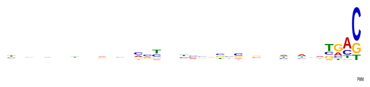	NGCCANNNNNNNNNNNNNNNNNNNNNNNNNNNNNATGAN	2.87	IR:30	11
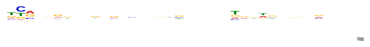	TCATNNNNNNNNNNNNNNNNNNNNNNNNNNNNNNGTGG	2.21	M	12
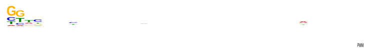	GGTCNNNNNNNNNNNNNNNNNNNNNNNNNNNNNNNATGG	2.67	M	13
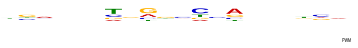	NNNNNNTGGGCCCANNNNNN	2.59	M	14
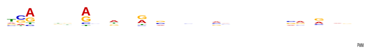	TCAANNNNNNNNNNNNNNNNNNNNNNNNNNNNNNTAGG	2.30	M	15
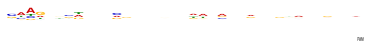	CAAGNNNNNNNNNNNNNNNNNNNNNNNNNNNNNGCCA	2.18	M	16
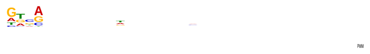	GTCANNNNNNNNNNNNNNNNNNNNNNNNNNNNNNNGCAG	2.49	M	17
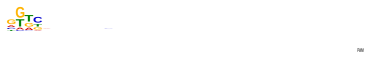	GGTCNNNNNNNNNNNNNNNNNNNNNNNNNNNNNNNGCGA	2.78	M	18

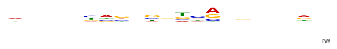	NNNNNNGCAG$\overrightarrow{NNNTC}$AANNNNNN	2.38	M	19
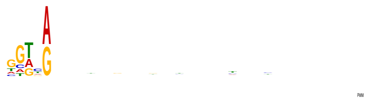	$\overrightarrow{GGTC}$NNNNNNNNNNNNNNNNNNNNNNNNNNNNNNNNGAAT	2.83	M	20

63 ESRRA-Yin2017-Rnd2 (ERR1003029)

PWM	Seed Sequence	Seed Seq Enrichment	Repeat	Rank
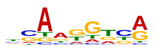	NNNNNNCTCANNGTCANNNNNN	6.30	DR:0	1
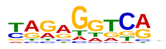	NNNNNNTAGANNTCATNNNNNN	6.55	M	2
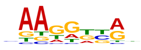	NNNNNNCAAGNNNNATTTNNNNNN	4.31	M	3
	NNNNNNGTGANNNTCACNNNNNN	4.21	DR:0	4
	NNNNNNTCGGTCCGNNNNNN	2.65	M	5
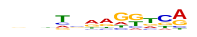	NNNNNNGTTTNNNNGTCANNNNNN	4.40	M	6
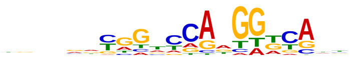	NNNNNNCGGTCCAAGNNNNNN	6.30	M	7
	NNNNNNGTCANNNNNAGGTNNNNNN	3.35	DR:5	8
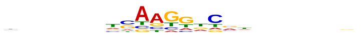	NNNNNNTTCANNNNCGTTNNNNNN	3.22	M	9

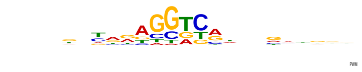	$NNNNNNTAGANNNNATTTNNNNNN$	2.89	M	10
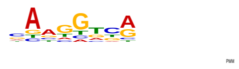	$NNNNN\overrightarrow{NGCCA}\overrightarrow{NNGTCA}NNNNNN$	4.64	DR:0	11
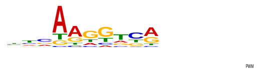	$NNNNN\overrightarrow{NGTTCN}\overrightarrow{NNGTCA}NNNNNN$	4.39	DR:0	12
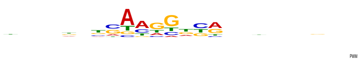	$NNN\overrightarrow{NNNTCA}ANNNNAAATNNNNNN$	4.02	M	13
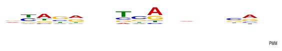	$NNNNNNC\overrightarrow{AGGNNT}CAANNNNNN$	3.01	M	14
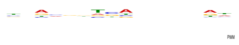	$NNNNNNGTATNN\overrightarrow{NNNTCA}ANNNNNN$	2.72	M	15
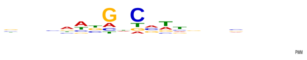	$NNNNNNCCAANNNNNTTGGNNNNNN$	2.64	M	16
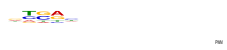	$NNNNNNCAAGNNNNNCTTGNNNNNN$	2.65	M	17
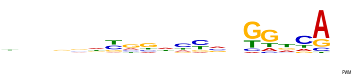	$NNNNNN\overrightarrow{TGGTCC}AANNNNNN$	2.96	M	18

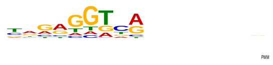	*NNNNNNTAG*$\overrightarrow{ANNNC}$*ACGNNNNNN*	3.04	M	19
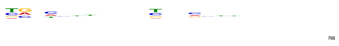	*NNNNNNCTTGNNNNNNNNCTTGNNNNNN*	2.87	M	20

64 ESRRA-Yin2017-Rnd3 (ERR1003030)

PWM	Seed Sequence	Seed Seq Enrichment	Repeat	Rank
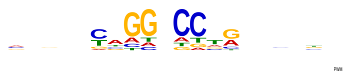	NNNNNNCAGGNCCTGNNNNNN	6.50	M	1
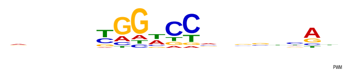	NNNNNNTGGTCCAANNNNNNN	5.09	M	2
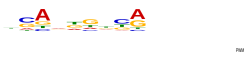	NNNNNNNCTCANNGTCANNNNNNN	3.89	DR:0	3
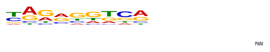	NNNNNNTAGANNTCATNNNNNN	3.46	M	4
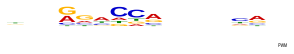	NNNNNNCAGACCAANNNNNNN	3.53	M	5
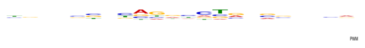	NNNNNNNTCAGNNCTGANNNNNNN	2.66	IR:4	6
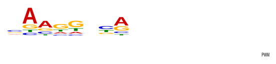	NNNNNNCAAGNNNAAACNNNNNN	3.19	M	7
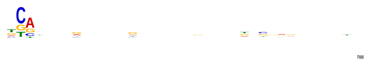	TCATNNNNNNNNNNNNNNNNNNNNNNNNNNNNNNNNGATG	2.49	M	8
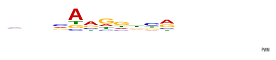	NNNNNNAGTCNNNGTCANNNNNNN	2.78	DR:0	9

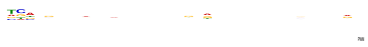	TCACNNNNNNNNNNNNNNNNNNNNNNNNNNNNNNGTAA	2.23	ER:26	10
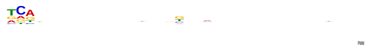	TCATNNNNNNNNNNNNNNNNNNNNNNNNNNNNNNTAGG	2.65	M	11
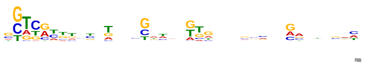	NNTCGTNNNNNNNNNNNNNNNNNNNNNNNNNNNTTAGNN	1.77	M	12
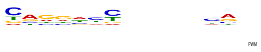	NNNNNNCAGGNCCAANNNNNNN	3.17	M	13
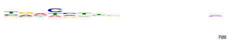	NNNNNNCAATNNNNTTCANNNNNNN	2.28	M	14
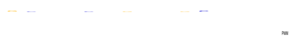	CACGNNNNNNNNNNNNNNNNNNNNNNNNNNNNNCGTG	1.94	M	15
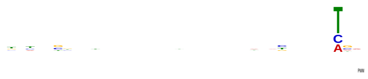	TTTANNNNNNNNNNNNNNNNNNNNNNNNNNNNNNTTGA	2.29	M	16
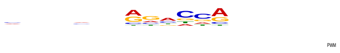	NNNNNNCGAGACCANNNNNNN	3.17	M	17
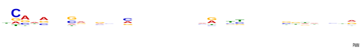	TCAANNNNNNNNNNNNNNNNNNNNNNNNNNNNNNACTA	2.26	M	18

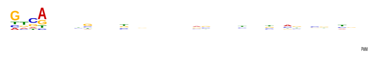	$\overrightarrow{NTCA}$GNNNNNNNNNNNNNNNNNNNNNNNNNNNNNGATGN	2.37	M	19
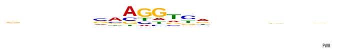	NNNN$\overrightarrow{NNCTCA}$NNNNCACGNNNNNN	2.56	M	20

65 ESRRA-Yin2017-Rnd3 (ERR1003031)

PWM	Seed Sequence	Seed Seq Enrichment	Repeat	Rank
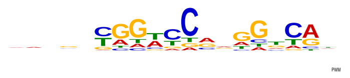	NNNNNNCGGTCCAANNNNNN	6.39	M	1
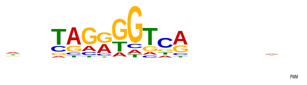	NNNNNNTTAGNNNTCATNNNNNN	5.87	M	2
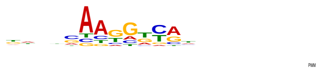	NNNNNNTCAANNNNATTCNNNNNN	4.77	M	3
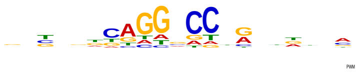	NNNNNNCAGGNCCTANNNNNN	5.66	M	4
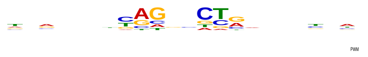	NNNNNNTCAGNNCTGANNNNNN	4.43	IR:4	5
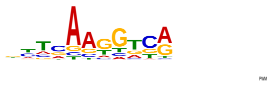	NNNNNNGCTCNNNGTCANNNNNN	5.71	DR:0	6
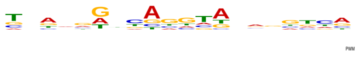	NNNNNNTCAGGTAGNNNNNN	3.61	M	7
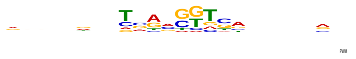	NNNNNNGTTTNNNNNTCAGNNNNNN	3.27	M	8
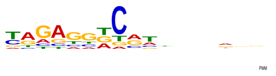	NNNNNNTAGANNNCGTTNNNNNN	4.39	M	9

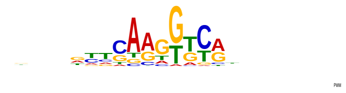	NNNNNNGTTCANNNNCATTNNNNNN	7.76	M	10
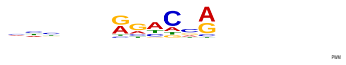	NNNNNNCAGACCACNNNNNN	3.25	M	11
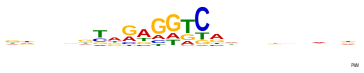	NNNNNNTAGANNNNAAACNNNNNN	2.76	M	12
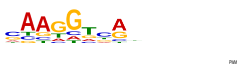	NNNNNNGCCANNNTCACNNNNNN	3.43	DR:0	13
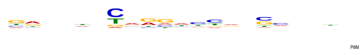	NNNNNNCAGGNCCGTNNNNNN	2.34	M	14
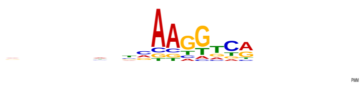	NNNNNNAGTCNNNNTCATNNNNNN	4.23	DR:0	15
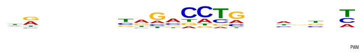	NNNNNNCTAGNNCTGCNNNNNN	2.79	M	16
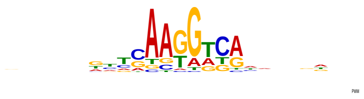	NNNNNNGTTCANNNNCAAANNNNNN	7.32	M	17
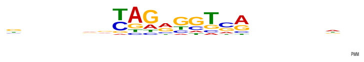	NNNNNNGCAGNNNTCACNNNNNN	3.51	M	18

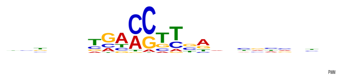	$NNNNNNTTTGNNNNNNNAAACNNNNNN$	2.84	M	19
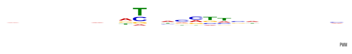	$NNNNNNTAAANNNN\overrightarrow{NNTTC}ANNNNNNN$	3.02	M	20

66 ESRRA-Yin2017-Rnd4 (ERR1003032)

PWM	Seed Sequence	Seed Seq Enrichment	Repeat	Rank
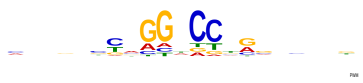	NNNNNNCAGGNCCTGNNNNNN	7.12	M	1
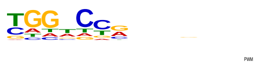	NNNNNNTGGTCCGTNNNNNN	5.82	M	2
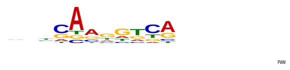	NNNNNNCTCANNNTCATNNNNNN	4.43	DR:0	3
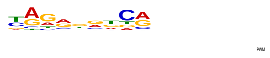	NNNNNNTAGANNTCATNNNNNN	3.60	M	4
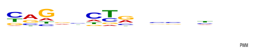	NNNNNNCCAGNNCTGANNNNNN	3.37	M	5
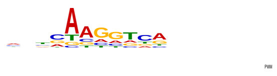	NNNNNNAGTCNNNNTCATNNNNNN	3.61	DR:0	6
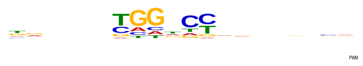	NNNNNNTGGTCCCANNNNNN	4.61	M	7
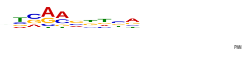	NNNNNNAGTTNNNNNTCATNNNNNN	2.63	DR:0	8
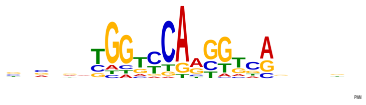	NNNNNNTGGTCCNNNGTCANNNNNN	11.89	DR:1	9

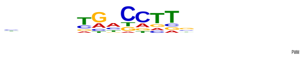	*NNNNNNGTTTNNNNTTGGNNNNNN*	2.78	M	10
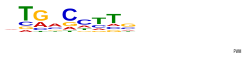	*NNNNNNCGATNNNNTTGGNNNNNN*	3.07	M	11
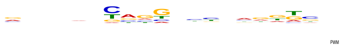	*NNNNNNGCAGNTCCANNNNNN*	2.42	M	12
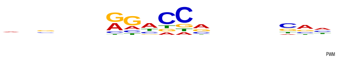	*NNNNNNCAGACCAANNNNNN*	4.25	M	13
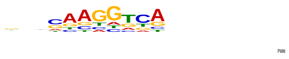	*NNNNNNNCTCANNNNCACGNNNNNN*	3.49	M	14
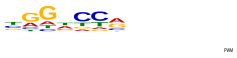	*NNNNNNTCGGTCCANNNNNN*	4.29	M	15
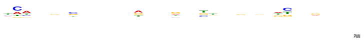	*TCATNNNNNNNNNNNNNNNNNNNNNNNNNNNNNNTCTG*	2.01	M	16
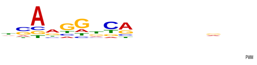	*NNNNNNNCTCANNNNCAAANNNNNN*	3.14	M	17
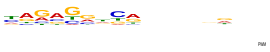	*NNNNNNTAGANNNCAAANNNNNN*	2.51	M	18

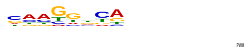	$NNNNN\overrightarrow{NGCCA}NNNNNCACGNNNNNNN$	3.03	M	19
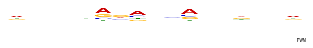	$NNNNNNAA\overrightarrow{AGACCA}NNNNNNN$	1.91	M	20

67 ESRRA-Yin2017-Rnd4 (ERR1003033)

PWM	Seed Sequence	Seed Seq Enrichment	Repeat	Rank
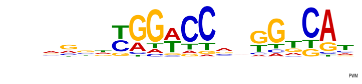	NNNNNNCGGTCCAANNNNNN	10.42	M	1
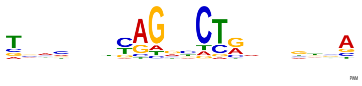	NNNNNNTCAGNNCTGANNNNNN	8.66	IR:4	2
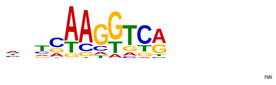	NNNNNNAGTCNNNNTCATNNNNNN	6.60	DR:0	3
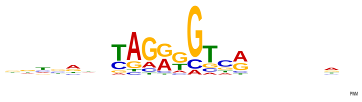	NNNNNNTTAGNNNTCATNNNNNN	6.39	M	4
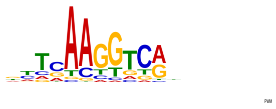	NNNNNNGCTCNNNNTCATNNNNNN	6.19	DR:0	5
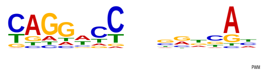	NNNNNNCAGGNCCAANNNNNN	7.03	M	6
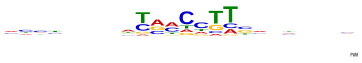	NNNNNNAAATNNNNTTCANNNNNN	6.03	M	7
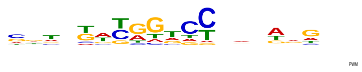	NNNNNNCGGTCTGGNNNNNN	3.52	M	8
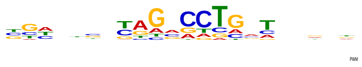	NNNNNNCTAGNNCTGCNNNNNN	4.78	M	9

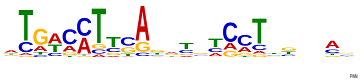	*NNNNNNTTCAGNNNTCCTNNNNNN*	7.23	IR:2	10
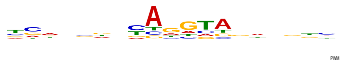	*NNNNNNGTCAGGTANNNNNN*	4.06	M	11
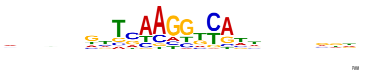	*NNNNNNGTTCNNNNNNATTTNNNNNN*	4.29	M	12
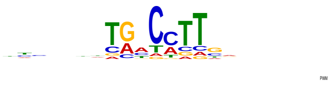	*NNNNNNGTTTNNNNTTGGNNNNNN*	3.56	M	13
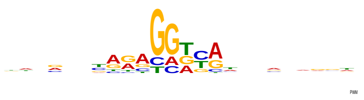	*NNNNNNTAGANNNNATTTNNNNNN*	4.09	M	14
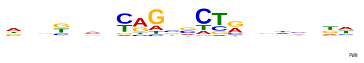	*NNNNNNACAGNNCTACNNNNNN*	3.77	M	15
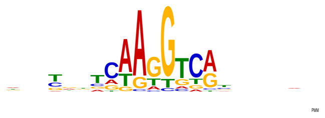	*NNNNNNTGGTCNNNNTCATNNNNNN*	7.63	DR:0	16
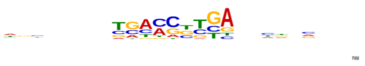	*NNNNNNTTTGNNNNNNAAACNNNNNN*	3.24	M	17
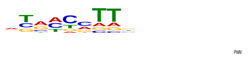	*NNNNNNGAATNNNNTTGTNNNNNN*	3.72	M	18

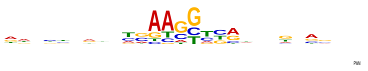	*NNNNNNGTTTNNNNNNNNATTTNNNNNN*	2.72	M	19
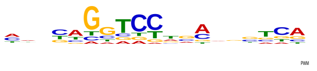	$NNNNNNC\overrightarrow{AGGNNC}TCANNNNNNN$	3.57	M	20

68 ESRRB:RXRA-Bhimsaria2022-Rnd3 (S13-137)

PWM	Seed Sequence	Seed Seq Enrichment	Repeat	Rank
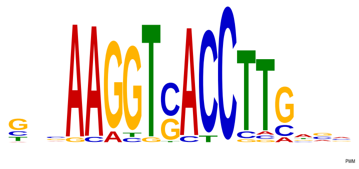	NNNAAGGTCACCTTGNNN	439.32	M	1
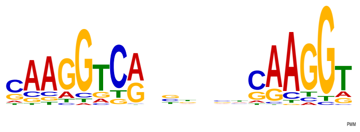	CAAGGNNNNNNNNNNAAGGT	97.99	DR:8	2
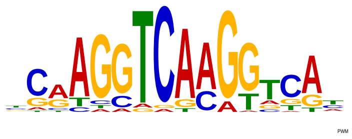	NNAAGGTCAAGGTCNN	124.74	DR:0	3
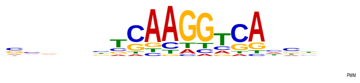	NNNNNNTCAAGGTCNNNNNN	13.49	DR:0	4
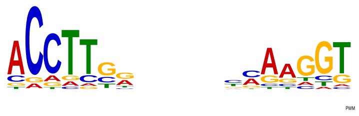	ACCTTNNNNNNNNNNAGGT	28.47	ER:11	5
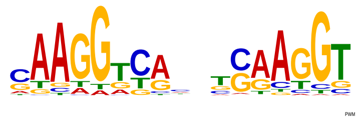	CAAGGTNNNNNNAAGGT	109.89	DR:5	6
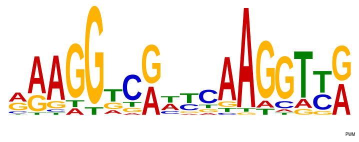	NNAGGTCNNNCAAGGTNN	71.61	DR:4	7
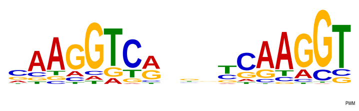	CAAGGNNNNNNNNAAGGT	35.07	DR:6	8
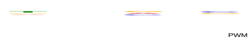	TAGAGGTC	2.17	M	9

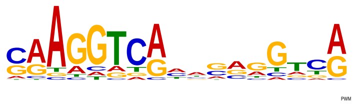	$NA\overrightarrow{AGGTCN}NNNN\overrightarrow{NGGTCN}$	24.03	DR:3	10
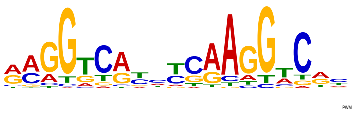	$N\overrightarrow{NGGTCN}NNNNA\overrightarrow{AGGTCN}N$	33.49	DR:5	11
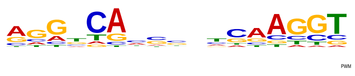	$\overrightarrow{AGGTN}NNNNNNNNA\overrightarrow{AGGT}$	10.87	DR:7	12
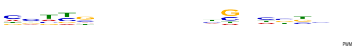	*CCTTNNNNNNNNNNNNCTCT*	5.98	M	13
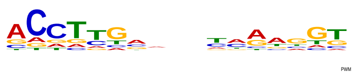	$\overleftarrow{ACCT}TNNNNNNNN\overrightarrow{AGGT}$	10.03	ER:9	14
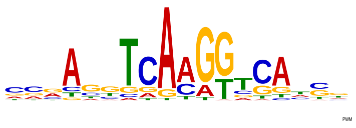	$NNN\overrightarrow{NGGTCA}\overrightarrow{AGGTCN}NNNN$	26.53	DR:0	15
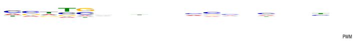	*CCTTNNNNNNNNNNNNTCTG*	5.33	M	16
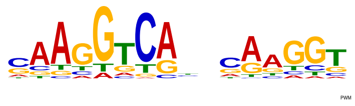	$CA\overrightarrow{AGGTN}NNNNN\overrightarrow{AGGT}$	20.95	DR:4	17
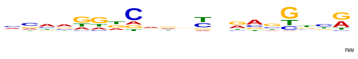	$NCAAGNNNNNNNNNN\overrightarrow{NGGTCN}$	7.15	M	18

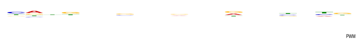	*CATGNNNNNNNNNNNTTTG*	5.08	M	19
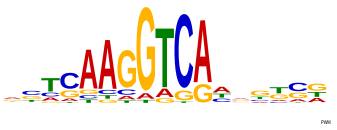	*NNNNCA*$\overrightarrow{AGGTCA}$*AGNNNN*	33.27	M	20

69 ESRRB-Bhimsaria2022-Rnd2 (S07-038)

PWM	Seed Sequence	Seed Seq Enrichment	Repeat	Rank
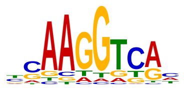	NNNNNNCAAGGTCANNNNNN	20.04	M	1
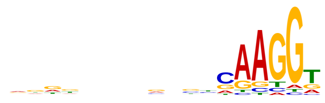	CAAGNNNNNNNNNNNCAAGGT	34.67	M	2
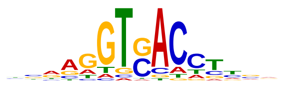	NNNNAAGGTNACCTTNNNN	28.29	M	3
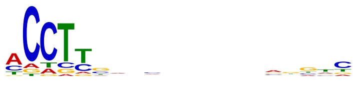	ACCTTNNNNNNNNNNNGGTC	18.11	ER:11	4
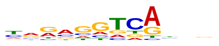	NNNNNNTAGAGGTCNNNNNN	3.74	M	5
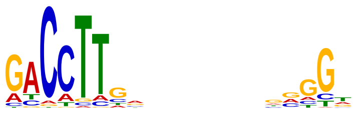	GACCTTGNNNNNNNNNGGGT	67.40	ER:11	6
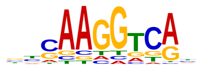	NNNNNNTCAAGGTCNNNNNN	12.25	DR:0	7
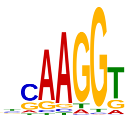	AGGGNNNNNNNNNNCAAGGT	32.57	DR:10	8
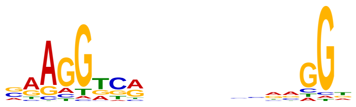	GAAGGTCNNNNNNNNNAGGT	48.49	DR:8	9

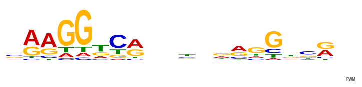	NN$\overrightarrow{AGGTCN}$NNNNN$\overrightarrow{NGGTCN}$N	9.20	DR:5	10
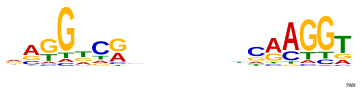	A$\overrightarrow{AGGTCN}$NNNNNNNNA$\overrightarrow{AGGT}$	43.03	DR:9	11
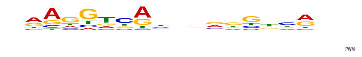	NN$\overrightarrow{NGGTCN}$NNN$\overrightarrow{NGGTCN}$NN	3.51	DR:3	12
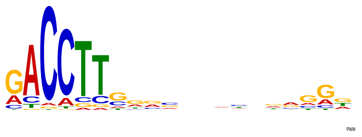	$\overleftarrow{GACCT}$TGNNNNNNNNNN$\overrightarrow{AGGG}$	52.88	ER:11	13
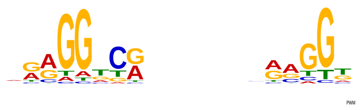	AG$\overrightarrow{AGGTCN}$NNNNNNNNN$\overrightarrow{AGGT}$	36.14	DR:8	14
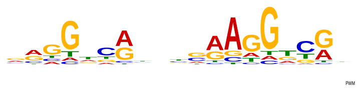	NN$\overrightarrow{GGTCN}$NNNNA$\overrightarrow{AGGTCN}$N	16.15	DR:5	15
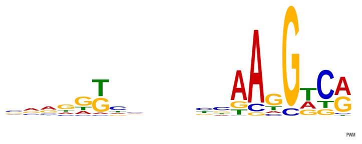	NA$\overrightarrow{AGGNN}$NNNNNCA$\overrightarrow{AGGTCN}$	27.53	DR:6	16
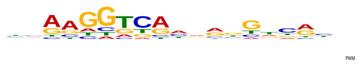	NNN$\overrightarrow{AGGTCN}$NN$\overrightarrow{NGGTCN}$NN	5.48	DR:2	17
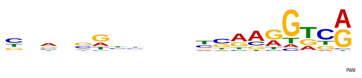	NCAAGNNNNNNNNNA$\overrightarrow{AGGTCN}$	9.02	M	18

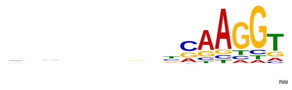	CGCANNNNNNNNNNNA$\overrightarrow{AGGT}$	8.90	M	19
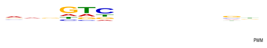	NNNNCA$\overrightarrow{AGNNNA}$CAGNNNN	1.83	M	20

70 ESRRB-Bhimsaria2022-Rnd3 (S07-158)

PWM	Seed Sequence	Seed Seq Enrichment	Repeat	Rank
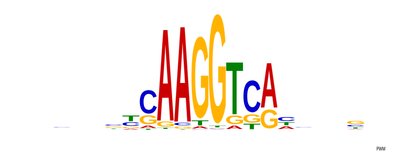	NNNNNNCAAGGTCANNNNNN	254.00	M	1
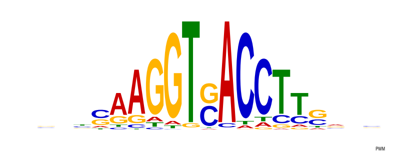	NNNNAAGGTNACCTTNNNN	850.78	M	2
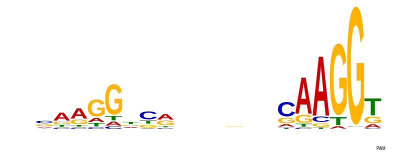	CAAGGNNNNNNNNNCAAGGT	1615.88	DR:8	3
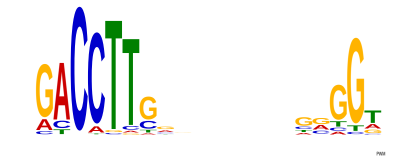	GACCTTGNNNNNNNNGGGGT	2993.23	ER:11	4
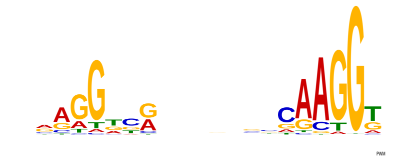	AAGGTCNNNNNNNNCAAGGT	2055.05	DR:9	5
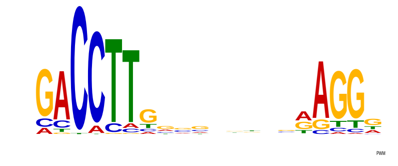	GACCTTGGNNNNNNNAAGGG	4216.06	ER:11	6
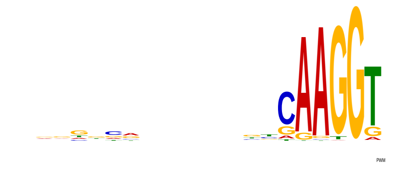	AGGTNNNNNNNNNNCAAGGT	472.35	DR:10	7
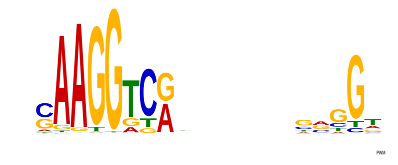	CAAGGTCNNNNNNNNGGGGT	1867.01	DR:8	8
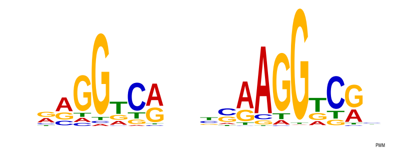	NNGGTCNNNNNAAGGTCNN	389.17	DR:5	9

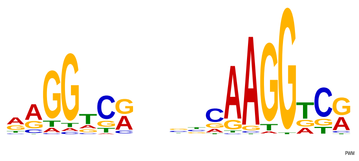	NAGGTCNNNNNCAAGGTCN	1165.77	DR:6	10
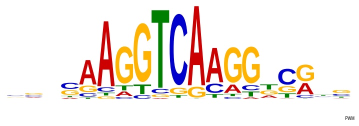	NNNCAAGGTCAAGGTCNNN	2670.86	DR:0	11
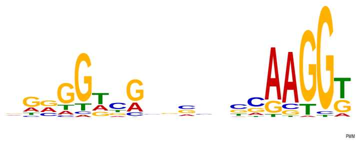	AGGGGTCNNNNNNNCAAGGT	2880.28	DR:8	12
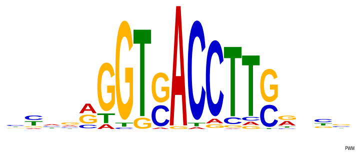	NNNGGGGTCACCTTGANNN	2339.23	IR:3	13
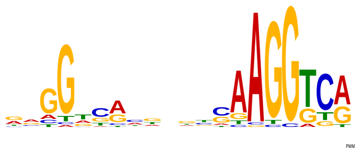	NAGGTCNNNNNNCAAGGTCN	704.22	DR:7	14
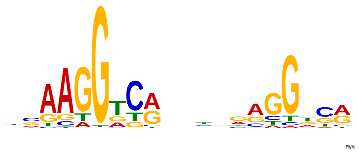	NCAAGGTCNNNNNNNGGTCN	483.65	DR:5	15
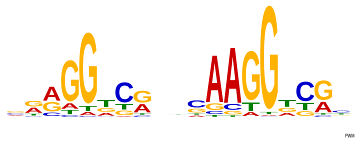	NNAGGTCNNNCAAGGTCNN	1155.14	DR:4	16
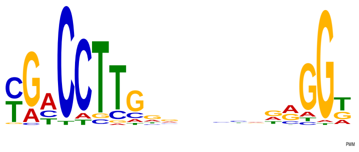	CGACCTTGNNNNNNNNNAGGT	964.90	ER:10	17
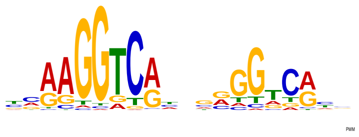	NNNAGGTCNNNNNGGTCNNN	101.11	DR:3	18

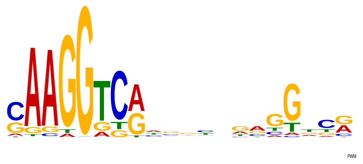	$CA\overrightarrow{AGGTCN}NNNNNN\overrightarrow{NGGTCG}$	1140.96	DR:6	19
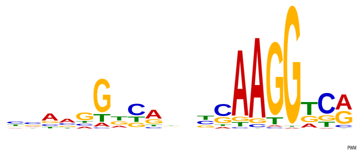	$NCAAGNNNNNNNCA\overrightarrow{AGGTCN}$	395.21	M	20

71 ESRRB-Jolma2013-Rnd2 (ERR194874)

PWM	Seed Sequence	Seed Seq Enrichment	Repeat	Rank
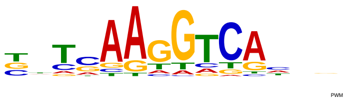	*NNNCAAGGTCANNN*	8.68	M	1
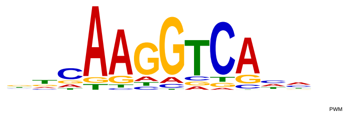	*NNCAAGGTCAANN*	12.79	M	2
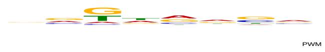	*GGGAAAGA*	2.52	M	3
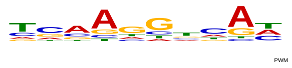	*NNCCAAGGTCNN*	3.29	M	4
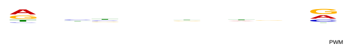	*NTCTTNNATTTN*	1.58	M	5
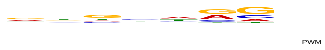	*GTGTAGGG*	2.02	M	6
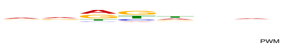	*AGAGGTCA*	1.48	M	7
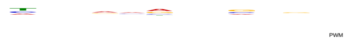	*NGGAANNGGTGN*	1.89	M	8
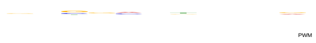	*NGGGGNNTATTN*	1.52	M	9

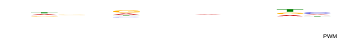	*NNGTGTGAGGNN*	1.70	M	10
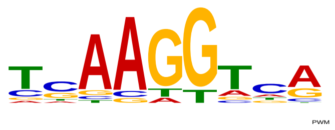	*CCAAGGTCA*	8.45	M	11
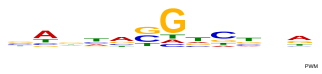	*NNAGTANGTCTNN*	2.61	M	12
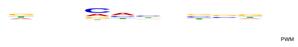	*NGGGTNNNGGGGN*	1.98	M	13
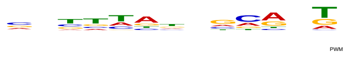	*NNTTTANAGCANN*	2.18	M	14
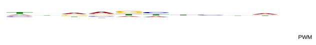	*NTTAANNTCTAN*	1.71	M	15
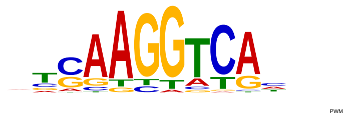	*NNCAAGGTCATNN*	10.93	M	16
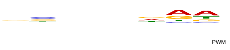	*NNGGGGGGAANN*	1.41	M	17
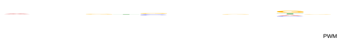	*NTAGTNNGGTGN*	1.58	M	18

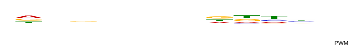	$NNGAG\overrightarrow{TGGTTN}N$	1.57	M	19
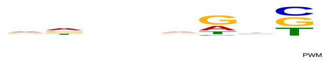	$GAA\overrightarrow{AGGTG}$	1.78	M	20

72 ESRRB-Jolma2013-Rnd3 (ERR194875)

PWM	Seed Sequence	Seed Seq Enrichment	Repeat	Rank
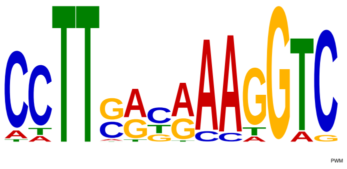	*CCTTGNNNAAGGTC*	239.85	M	1
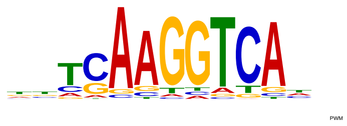	*NNTCAANGTCANN*	18.01	DR:0	2
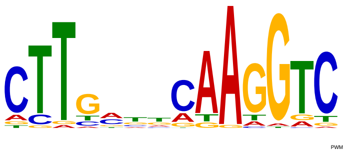	*CTTGNNNNNAGGTC*	60.71	M	3
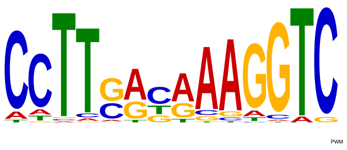	*CCTTGNNNNNGGTC*	59.84	M	4
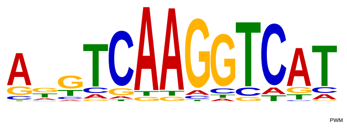	*NGGTCNNGGTCAN*	17.98	DR:0	5
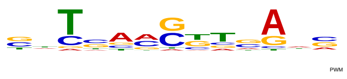	*NTTGANNNTCAAN*	3.74	M	6
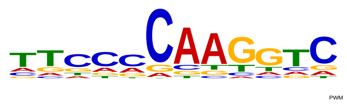	*TTCCNNNNGGTC*	6.09	M	7
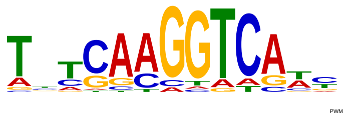	*NCTCANNGTCATN*	13.65	DR:0	8
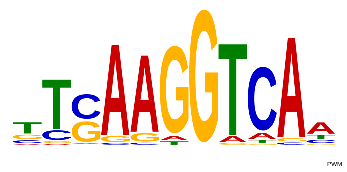	*TTGAAGNTCAA*	32.75	M	9

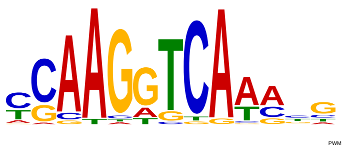	$NCA\overrightarrow{AGNTCA}AATN$	34.60	M	10
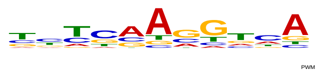	$NCTTCN\overrightarrow{NGGTCN}$	3.06	M	11
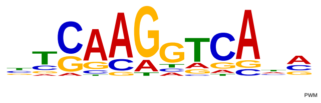	$\overrightarrow{NGTCA}N\overrightarrow{NGTCA}AN$	9.50	DR:0	12
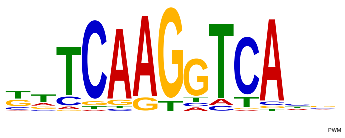	$\overrightarrow{NTTCA}N\overrightarrow{NGTCA}CN$	18.63	DR:0	13
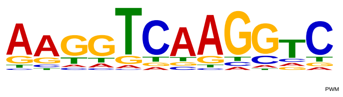	$A\overrightarrow{AGGTN}N\overrightarrow{NGGTC}$	10.97	DR:0	14
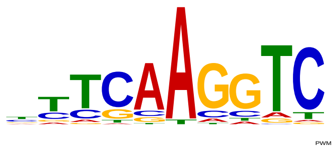	$\overrightarrow{CTTCA}\overrightarrow{NGGTC}$	14.60	DR:0	15
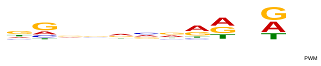	$NNGGG\overrightarrow{ANGAAA}NN$	2.21	M	16
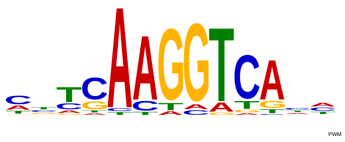	$\overrightarrow{NTTCA}N\overrightarrow{NGTCA}AN$	13.90	DR:0	17
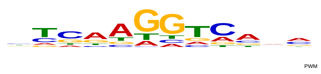	$\overrightarrow{NGTCA}N\overrightarrow{NNTCA}AN$	3.96	DR:0	18

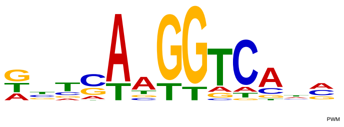	$\overrightarrow{NGTCA}\overrightarrow{ANNTCA}AN$	8.10	DR:0	19
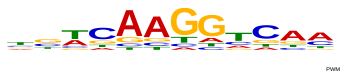	$\overrightarrow{TGTCN}\overrightarrow{NNNTCA}A$	4.37	DR:0	20

73 ESRRB-Jolma2013-Rnd4 (ERR194876)

PWM	Seed Sequence	Seed Seq Enrichment	Repeat	Rank
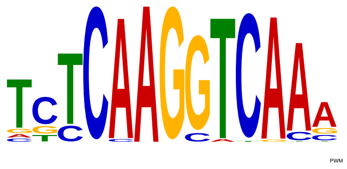	TCTCAANNTCAAA	233.27	DR:0	1
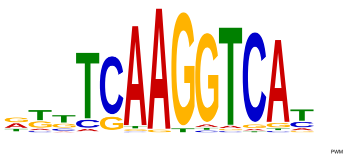	NNTTCANNGTCANN	35.65	DR:0	2
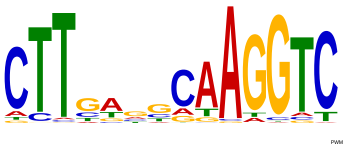	CTTGNNNNNNGGTC	64.62	M	3
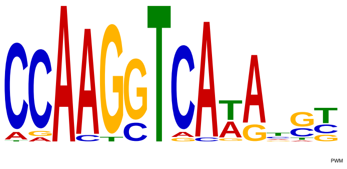	NCAAGNNCAAATGN	46.06	M	4
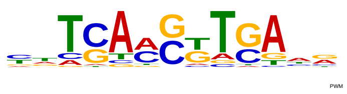	NTTGANNNTCAAN	8.07	M	5
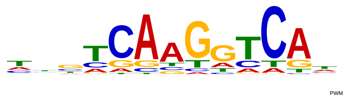	NACTCNNNGTCAN	5.33	M	6
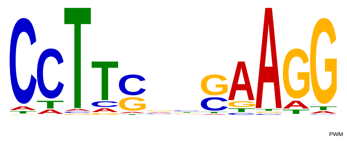	CCTTNNNNAAGG	47.88	M	7
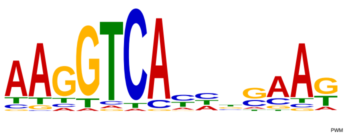	AAGGNNNNNNCAAG	7.58	M	8
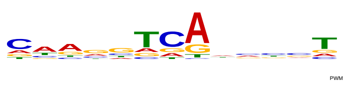	NAAGGNNNAATAN	2.26	M	9

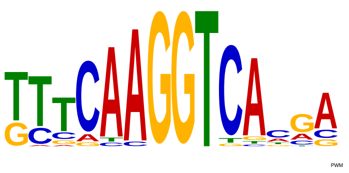	NTTCAANNNCACGN	22.30	DR:0	10
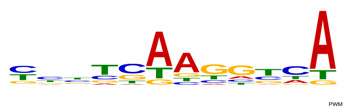	NCCTGNNGGTCN	3.00	ER:2	11
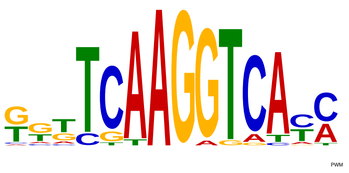	NGTTCNNNGTCACN	29.25	DR:0	12
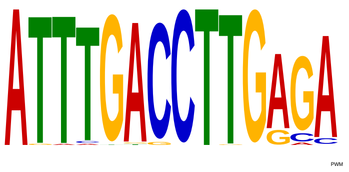	ATTTGANNNTGAGA	601.22	IR:3	13
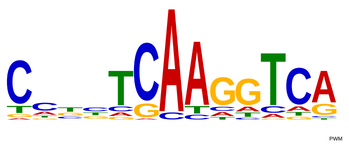	NCTCTNNNGGTCN	5.89	M	14
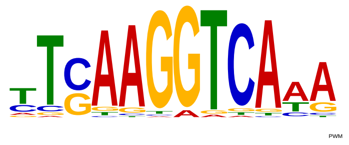	TTGAAGNNCAAA	36.46	M	15
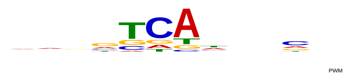	AAGGNNNNATAA	1.92	M	16
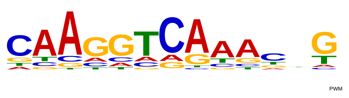	NAAGGNNNAACTN	4.70	M	17
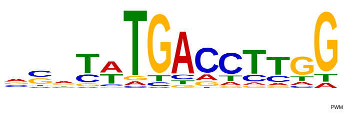	NCATTNNNNCTTGN	5.31	M	18

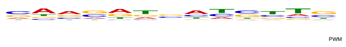	$NA\overrightarrow{AGGNNN}CCTTN$	1.87	M	19
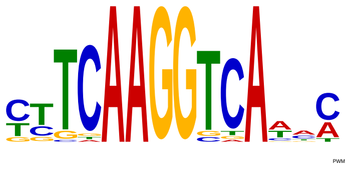	$\overrightarrow{NTTCA}\overrightarrow{NNGTCA}ACN$	49.08	DR:0	20

74 ESRRB-4-Bhimsaria2022-Rnd2 (S07-002)

PWM	Seed Sequence	Seed Seq Enrichment	Repeat	Rank
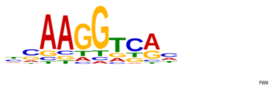	NNNNNNCAAGGTCANNNNNN	12.28	M	1
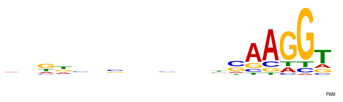	AAGGNNNNNNNNNNNAAGGT	12.09	DR:9	2
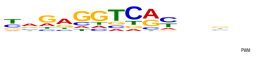	NNNNNNTAGAGGTCNNNNNN	4.04	M	3
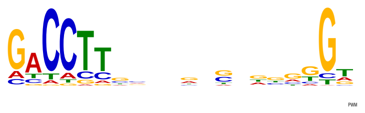	GACCTTNNNNNNNNNNGGGT	26.93	ER:11	4
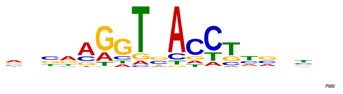	NNNNAAGGTNACCTTNNNN	19.63	M	5
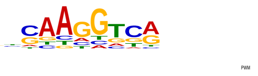	NNNNNNTCAAGGTCNNNNNN	8.55	DR:0	6
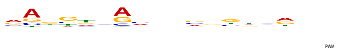	NNNGGTCNNNNNGGTCNNN	2.50	DR:3	7
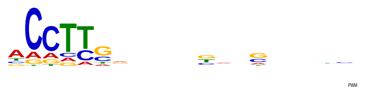	ACCTTNNNNNNNNNNNTGTC	8.85	ER:11	8
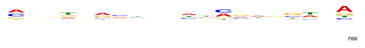	NGGTCNNNNNNNNNNGGTCN	1.96	DR:8	9

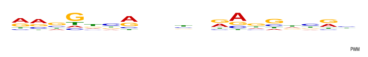	$NN\overrightarrow{GGTCN}NNNNNN\overrightarrow{GGTCN}N$	2.93	DR:5	10
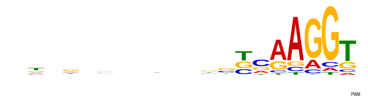	$CGTGNNNNNNNNNNNNA\overrightarrow{AGGT}$	8.31	M	11
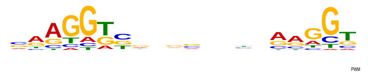	$GA\overrightarrow{AGGTN}NNNNNNNNNN\overrightarrow{AGGT}$	14.26	DR:8	12
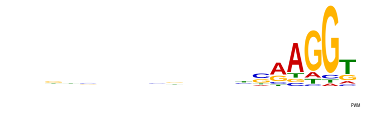	$\overrightarrow{AGGTN}NNNNNNNNNNNA\overrightarrow{AGGT}$	9.31	DR:10	13
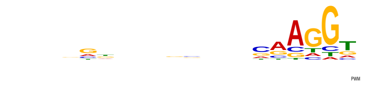	$CAAGNNNNNNNNNNNNA\overrightarrow{AGGT}$	10.17	M	14
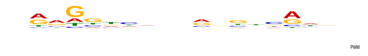	$NN\overrightarrow{NNGTCA}NNN\overrightarrow{NGGTCN}NNN$	2.89	DR:2	15
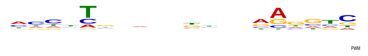	$\overleftarrow{CCCT}NNNNNNNNNNN\overrightarrow{NGGTC}$	3.23	ER:11	16
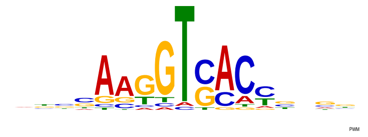	$NNNCA\overrightarrow{AGGTGA}CCCNNN$	21.89	M	17
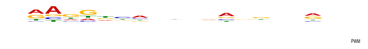	$NN\overrightarrow{NGGTCN}NNNNN\overrightarrow{NGGTCN}NN$	2.38	DR:4	18

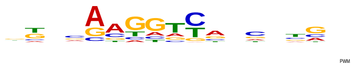	*NNNCAAGGTNACAGNNN*	6.12	M	19
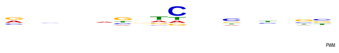	*NNNNCAAGGNNACAGNNNN*	2.20	M	20

75 ESRRB-4-Bhimsaria2022-Rnd3 (S07-123)

PWM	Seed Sequence	Seed Seq Enrichment	Repeat	Rank
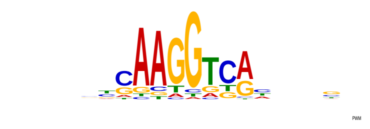	NNNNNNCAAGGTCANNNNNN	49.72	M	1
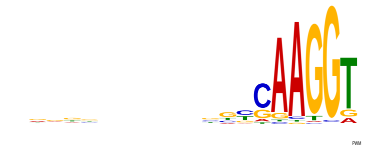	AGGGNNNNNNNNNNCAAGGT	138.12	DR:10	2
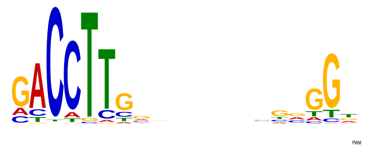	GACCTTGNNNNNNNNNGGGT	205.72	ER:11	3
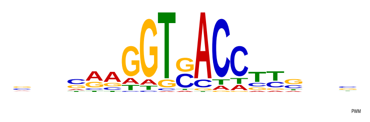	NNNNAAGGTNACCTTNNNN	91.20	M	4
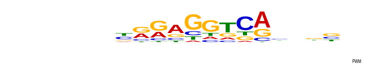	NNNNNNTAGAGGTCNNNNNN	8.66	M	5
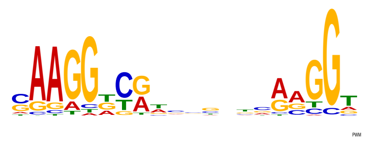	CAAGGGCNNNNNNNNNAGGT	132.96	DR:8	6
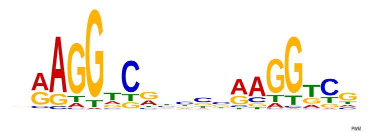	NAAGGTCNNNNNAAGGTCN	254.55	DR:5	7
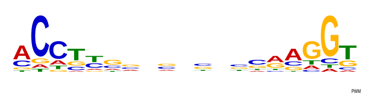	ACCTTNNNNNNNNNAAGGT	71.69	ER:11	8
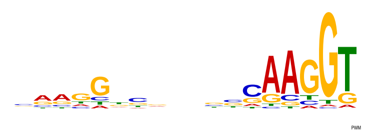	CAAGGNNNNNNNCAAGGT	100.22	DR:6	9

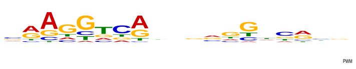	*NNNGGTCNNNNNGGTCNNN*	9.63	DR:3	10
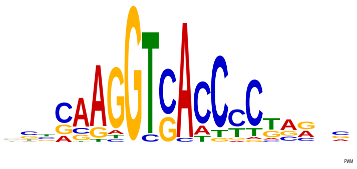	*NNNNAAGGTNACCCCTNNNN*	120.30	M	11
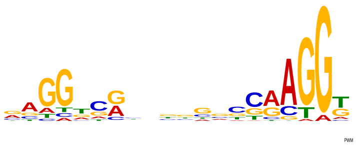	*AAGGTCNNNNNNNNNAAGGT*	137.74	DR:9	12
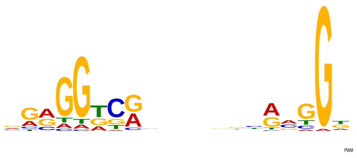	*AGAGGTCNNNNNNNNNAGGT*	103.88	DR:8	13
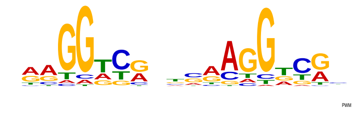	*NNAGGTCNNNNAAGGTCNN*	75.61	DR:4	14
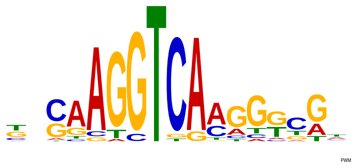	*NNCAAGGTCAAGGGCNN*	417.43	DR:0	15
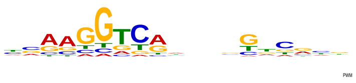	*NNNAGGTCNNNNGGTCNNN*	15.81	DR:2	16
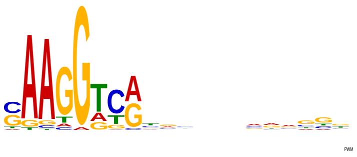	*CAAGGTCNNNNNNNNNAGGG*	154.14	DR:8	17
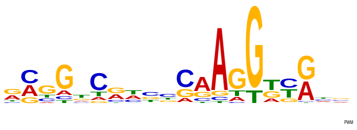	*NNGGTCNNNNNAAGGTCANN*	69.44	DR:5	18

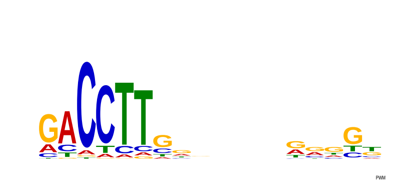	$\overleftarrow{GACCT}TGNNNNNNN\overrightarrow{GGGT}$	78.68	ER:9	19
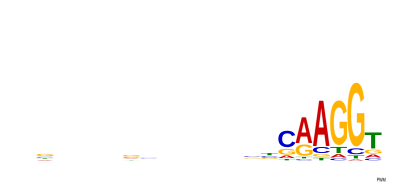	$\overrightarrow{GTCA}NNNNNNNNNNNA\overrightarrow{AGGT}$	29.89	DR:12	20

76 ESRRB-Yin2017-Rnd2 (ERR1003036)

PWM	Seed Sequence	Seed Seq Enrichment	Repeat	Rank
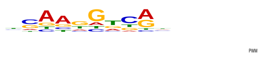	NNNNNNTCAANGTCANNNNNN	4.14	DR:0	1
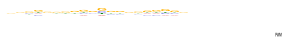	NNGGGGNNNNNNNNNNNNNNNNNNNNNNNNNNNNNGGGGNN	3.58	M	2
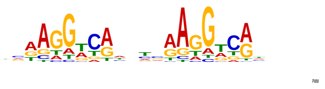	NNNNNNAGGTCNNNNNNAGGTCNNNNNN	13.05	DR:5	3
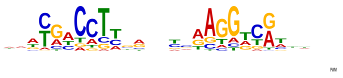	NNNNNNGACCTNNNNNNNNAGGTCNNNNNN	10.34	ER:8	4
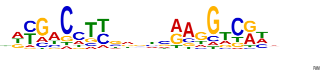	NNNNNNGACCTNNNNNNNAGGTCNNNNNN	8.32	ER:7	5
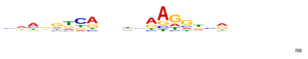	NNNNNNAAGGNNNNNNNNNNGTCANNNNNN	2.61	DR:5	6
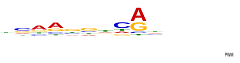	NNNNNNCTCANGGTCNNNNNN	2.60	DR:0	7
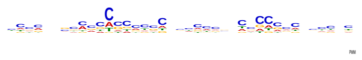	TCCCNNNNNNNNNNNNNNNNNNNNNNNNNNNNNNNNCCGC	3.07	M	8
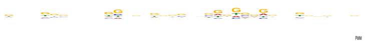	NNNNGGGANNNNNNNNNNNNNNNNNNNNNNNNAGGGNNNN	2.75	M	9

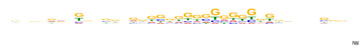	*NNCGGGNNNNNNNNNNNNNNNNNNNNNNNNNNNAGGGNN*	2.42	M	10
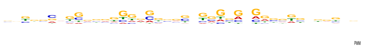	*GGTGNNNNNNNNNNNNNNNNNNNNNNNNNNNNNNNNGGAG*	2.63	M	11
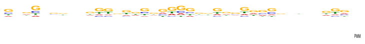	*GAGGNNNNNNNNNNNNNNNNNNNNNNNNNNNNNNNGGGC*	2.71	DR:27	12
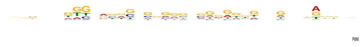	*TGGGNNNNNNNNNNNNNNNNNNNNNNNNNNNNNNNNGGAG*	2.58	M	13
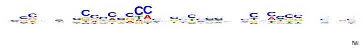	*TCCCNNNNNNNNNNNNNNNNNNNNNNNNNNNNNNNCGCC*	2.89	M	14
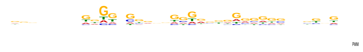	*TGGGNNNNNNNNNNNNNNNNNNNNNNNNNNNNNNNNGGGA*	2.69	M	15
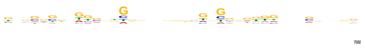	*GAGGNNNNNNNNNNNNNNNNNNNNNNNNNNNNNNNNNTGGG*	2.53	M	16
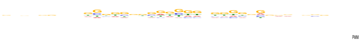	*NCGGGNNNNNNNNNNNNNNNNNNNNNNNNNNNNGGGTN*	2.10	M	17
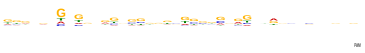	*GGGANNNNNNNNNNNNNNNNNNNNNNNNNNNNNNNNTGGG*	2.47	M	18

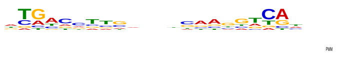	*NNNNNNCCTTNNNNNNAAGGNNNNNN*	2.66	ER:8	19
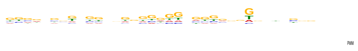	*NNGGGTNNNNNNNNNNNNNNNNNNNNNNNNNNNNGGGTNN*	2.48	DR:26	20

77 ESRRB-Yin2017-Rnd2 (ERR1003037)

PWM	Seed Sequence	Seed Seq Enrichment	Repeat	Rank
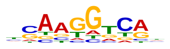	NNNNNNTCAANNTCATNNNNNN	4.56	DR:0	1
	NNNNNNGGGGNNNNNGGGGNNNNNN	3.93	M	2
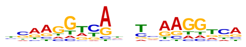	NNNNNNAAGGNNNNNNNNNNNGTCANNNNNN	3.96	DR:5	3
	NNNNNNACCTNNNNNNNNAGGTNNNNNN	2.50	ER:8	4
	NNNNGGGGNNNNNNNNNNNNNNNNNNNNNNNGGGGNNNN	3.30	M	5
	NNNNNNACCTNNNNNNNAGGTNNNNNN	2.27	ER:7	6
	NNNNNNTAGANGTCANNNNNNN	2.94	M	7
	NNNNNNACCTNNNNNNNNNNGGTCNNNNNN	2.41	ER:9	8
	NCGCANNNNNNNNNNNNNNNNNNNNNNNNNNNNNTTCGN	1.71	M	9

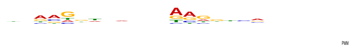	*NNNNNNNGTCANNNNNNNGTCANNNNNNN*	2.13	DR:4	10
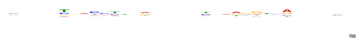	*NNNNNNTGACNNNNNNNNNNNNNNNNGTCANNNNNN*	2.16	ER:11	11
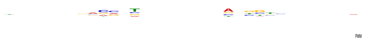	*NNNNNNTGACNNNNNNNNNNNNNNNGTCANNNNNN*	2.24	ER:10	12
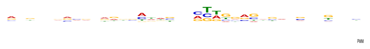	*NGGAANNNNNNNNNNNNNNNNNNNNNNNNNNNNNAGGTN*	1.75	M	13
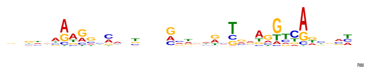	*NNNNNNAAGGNNNNNNNNNNNNNNNNNNNNNNGTCANNNNNN*	3.25	DR:15	14
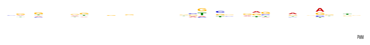	*CGCGNNNNNNNNNNNNNNNNNNNNNNNNNNNNNNNNNNGGTG*	1.96	M	15
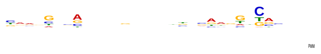	*NNNNNNAAGGNNNNNNNNNNNNNNNNNNNNAGGTNNNNNN*	2.24	DR:14	16
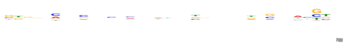	*NCGTGNNNNNNNNNNNNNNNNNNNNNNNNNNNNNNACGTN*	2.10	M	17
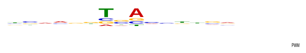	*NNNNNNAAGGNNNCCTTNNNNNN*	1.85	M	18

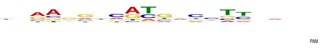	$NNNNNN\overrightarrow{NGGTCN}\overleftarrow{NGACCN}NNNNNN$	2.15	IR:0	19
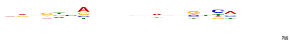	$NNNNNNA\overrightarrow{AGGNNN}NNNNNNN\overrightarrow{AGGTNN}NNNN$	2.13	DR:6	20

78 ESRRB-Yin2017-Rnd3 (ERR1003038)

PWM	Seed Sequence	Seed Seq Enrichment	Repeat	Rank
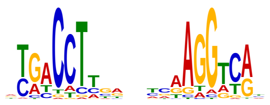	*NNNNNNGACCTNNNNNNNNNAGGTCNNNNNN*	34.64	ER:8	1
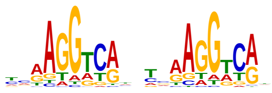	*NNNNNNAGGTCNNNNNNAGGTCNNNNNN*	34.66	DR:5	2
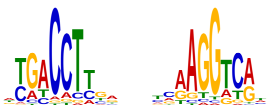	*NNNNNNGACCTNNNNNNNNNNAGGTCNNNNNN*	30.55	ER:9	3
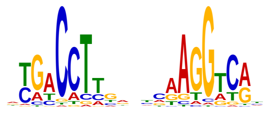	*NNNNNNGACCTNNNNNNNAGGTCNNNNNN*	23.53	ER:7	4
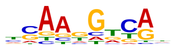	*NNNNNNTCAANGTCANNNNNN*	6.59	DR:0	5
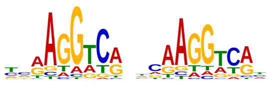	*NNNNNNAGGTCNNNNNAGGTCNNNNNN*	17.24	DR:4	6
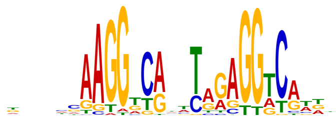	*NNNNNNAAGGNNNNNNTAGAGGTCNNNNNN*	85.38	DR:5	7
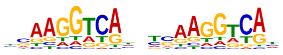	*NNNNNNAAGGNNNNNNNNAAGGNNNNNN*	7.85	DR:5	8
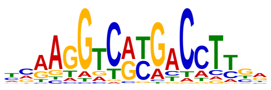	*NNNNNNAGGTCNNGACCTNNNNNN*	15.97	IR:0	9

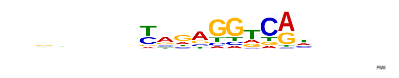	NNNNNNTAGAGGTCNNNNNN	4.07	M	10
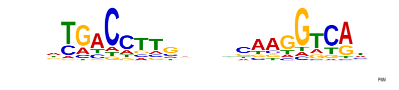	NNNNNNCTTGNNNNCAAGNNNNNN	4.93	M	11
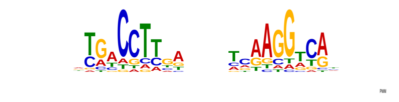	NNNNNNGACCTNNNNNNNNNNAGGTCNNNNNN	10.64	ER:10	12
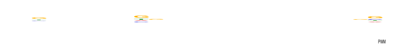	NNNNNNGGGCNNNNGGGGNNNNNN	3.10	M	13
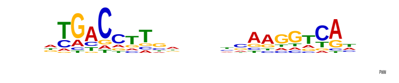	NNNNNNCTTGNNNNNNAAGGNNNNNN	4.33	M	14
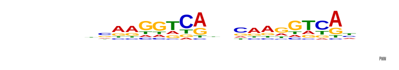	NNNNNNAAGGNNNNNNAAGGNNNNNN	4.39	DR:4	15
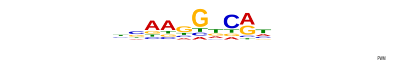	NNNNNNCAAGNNCGTTNNNNNN	3.46	M	16
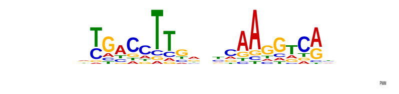	NNNNNNGACCNNNNNNNNNGGTCNNNNNN	4.04	ER:7	17
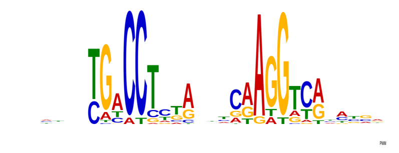	NNNNNNGACCTCTNNNNNNAGGTCNNNNNN	51.30	ER:8	18

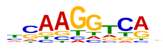	*NNNNNNTTCANNGTCANNNNNN*	5.62	DR:0	19
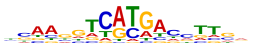	*NNNNNNAAGGNNNNNNCCTTNNNNNN*	2.91	IR:0	20

79 ESRRB-Yin2017-Rnd3 (ERR1003039)

PWM	Seed Sequence	Seed Seq Enrichment	Repeat	Rank
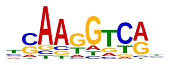	NNNNNNTCAANNTCATNNNNNN	7.41	DR:0	1
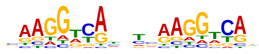	NNNNNNAAGGNNNNNNNNNAGGTNNNNNN	6.98	DR:5	2
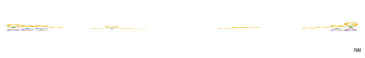	NNNNNNGGGGNNNNNGGGGNNNNNN	5.46	M	3
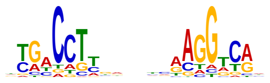	NNNNNNGACCTNNNNNNNNAGGTCNNNNNN	18.09	ER:8	4
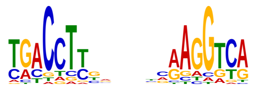	NNNNNNGACCTNNNNNNNNNAGGTCNNNNNN	18.80	ER:9	5
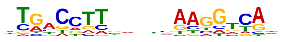	NNNNNNACCTNNNNNNNAGGTNNNNNN	4.04	ER:7	6
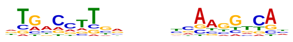	NNNNNNACCTNNNNNNNNNNAGGTNNNNNN	3.23	ER:10	7
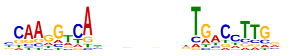	NNNNNNAAGGNNNNNNNNNNNNNNNNNCCTTNNNNNN	3.69	IR:10	8
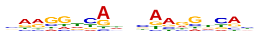	NNNNNNAAGGNNNNNNNNAGGTNNNNNN	3.42	DR:4	9

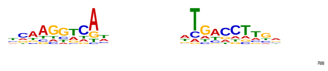	*NNNNNNAAGGNNNNNNNNNNNNNNNCCTTNNNNNN*	3.50	IR:9	10
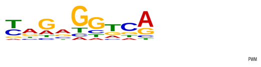	*NNNNNNTAGANGTCANNNNNNN*	3.60	M	11
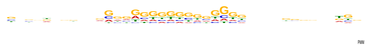	*NNNNNGGGGNNNNNNNNNNNNNNNNNNNNNNGGGGNNNNN*	4.05	M	12
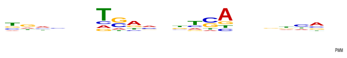	*NNNNNNTTGANNNTCAANNNNNNN*	3.97	M	13
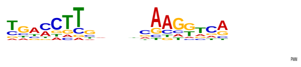	*NNNNNNTGACNNNNNNNNNNNNGTCANNNNNN*	4.07	ER:8	14
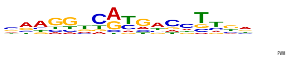	*NNNNNNAAGGNNNNGACCNNNNNN*	3.16	IR:0	15
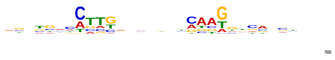	*NNNNNNTGACNNNNNNNNNNNNNNNGTCANNNNNN*	2.43	ER:11	16
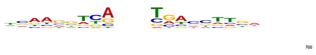	*NNNNNNAAGGNNNNNNNNNCCTTNNNNNN*	2.69	IR:3	17
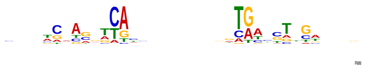	*NNNNNNAAGGNNNNNNNNNNNNNNNNNCCTTNNNNNN*	2.13	IR:11	18

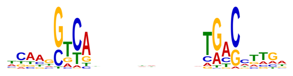	*NNNNNCAAGNNNNNNNNNNNNNNNNNNNNCTTGNNNNN*	2.92	M	19
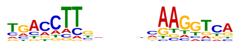	$NNNNNN\overleftarrow{TGACNN}NNNNNNNNN\overrightarrow{NNGTCA}NNNNNNN$	4.01	ER:9	20

80 ESRRB-Yin2017-Rnd4 (ERR1003040)

PWM	Seed Sequence	Seed Seq Enrichment	Repeat	Rank
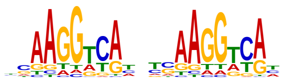	NNNNNNAAGGNNNNNNNNAAGGNNNNNNN	19.85	DR:5	1
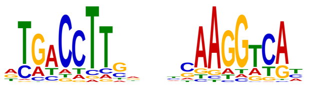	NNNNNNCCTTNNNNNNAAGGNNNNNNN	16.73	ER:8	2
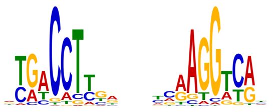	NNNNNNGACCTNNNNNNNNNAGGTCNNNNNN	50.67	ER:9	3
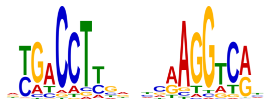	NNNNNNGACCTNNNNNNNAGGTCNNNNNN	44.94	ER:7	4
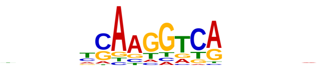	NNNNNNTTCANNGTCANNNNNNN	12.24	DR:0	5
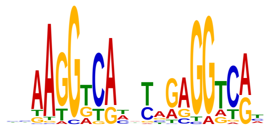	NNNNNNAAGGNNNNNNTAGAGGTCNNNNNN	177.31	DR:5	6
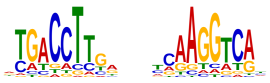	NNNNNNGACCNNNNNNNNNNNGGTCNNNNNN	12.58	ER:9	7
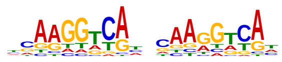	NNNNNNAAGGNNNNNNNAAGGNNNNNNN	8.70	DR:4	8
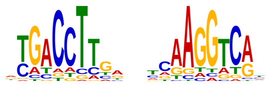	NNNNNNGACCNNNNNNNNNNGGTCNNNNNN	15.17	ER:8	9

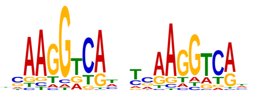	NNNNNNCAAGNNNNNNNNNNNGGTCNNNNNN	13.75	M	10
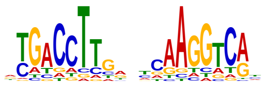	NNNNNNGACCNNNNNNNNNGGTCNNNNNN	11.02	ER:7	11
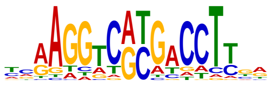	NNNNNNAGGTCNNGACCTNNNNNN	27.71	IR:0	12
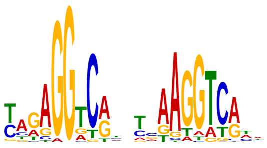	NNNNNNAGAGGTCNNNNNNNGGTCNNNNNN	66.84	DR:5	13
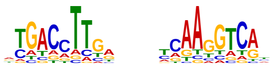	NNNNNNGACCNNNNNNNNNNNNGGTCNNNNNN	7.83	ER:10	14
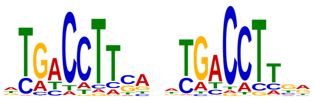	NNNNNNCTTCANNNGACCNNNNNN	26.17	IR:2	15
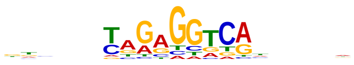	NNNNNNTAGANGTCANNNNNN	7.22	M	16
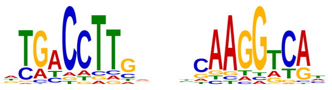	NNNNNNCTTGNNNNCAAGNNNNNN	14.81	M	17
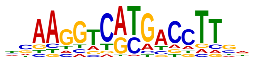	NNNNNNAAGGNNNNNNNCCTTNNNNNN	6.85	IR:0	18

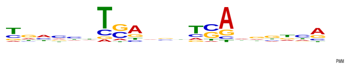	$NNNNNNTTGA\overrightarrow{NNNTC}AANNNNNNN$	7.94	M	19
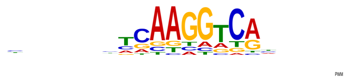	$NNNNN\overrightarrow{NGCTC}N\overrightarrow{NGGTC}NNNNNN$	7.65	DR:0	20

81 ESRRB-Yin2017-Rnd4 (ERR1003041)

PWM	Seed Sequence	Seed Seq Enrichment	Repeat	Rank
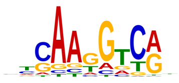	NNNNNNTCAANNTCATNNNNNN	9.97	DR:0	1
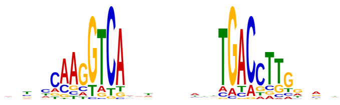	NNNNNNCAAGNNNNNNNNNNNNNNNNNNCTTGNNNNNN	7.48	M	2
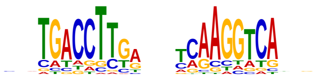	NNNNNNGACCNNNNNNNNNNNGGTCNNNNNN	10.63	ER:9	3
	NNNNNNGGGGNNNNNGGGGNNNNNN	8.06	M	4
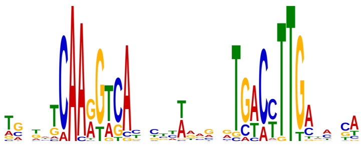	NNNNNTCAAGNNNNNNNNNNNNNNNNNNNNNCTTGANNNNN	36.20	IR:23	5
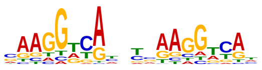	NNNNNNAAGGNNNNNNNNNAGGTNNNNNN	7.97	DR:5	6
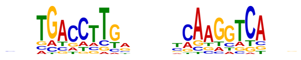	NNNNNNTGACNNNNNNNNNNNNNNNGTCANNNNNN	9.29	ER:10	7
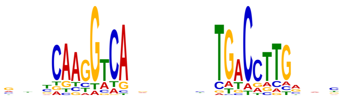	NNNNNNCAAGNNNNNNNNNNNNNNNNNNCTTGNNNNNN	8.29	M	8
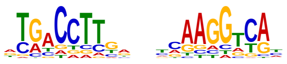	NNNNNNACCTNNNNNNNNAGGTNNNNNN	7.76	ER:8	9

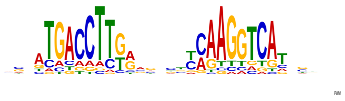	NNNNNNTGACNNNNNNNNNNNNNNNNGTCANNNNNNN	11.41	ER:11	10
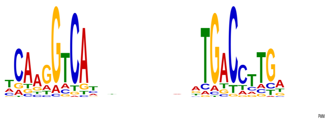	NNNNNCAAGNNNNNNNNNNNNNNNNNNNNNNCTTGNNNNN	6.72	M	11
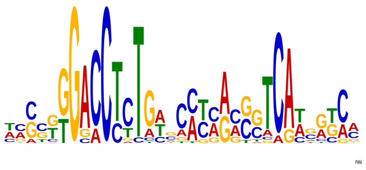	NNNNNNGACCTCTGNNNNNNNNNNGTCANNNNNNN	95.34	ER:10	12
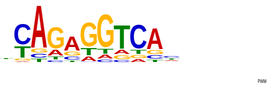	NNNNNNCAGANGTCANNNNNNN	6.64	M	13
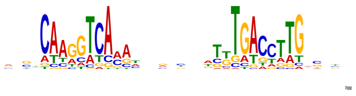	NNNNNCAAGNNNNNNNNNNNNNNNNNNNNNNNNCTTGNNNNN	7.20	M	14
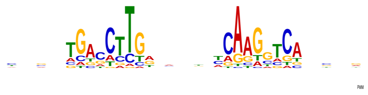	NNNNNNTGACNNNNNNNNNNNNNNNNNGTCANNNNNNN	6.58	ER:12	15
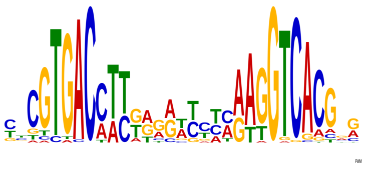	NNCGTGACNNNNNNNNNNNNNNNNNTCACGNN	47.45	ER:11	16
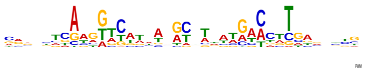	NNNNNTCAANNNNNNNNNNNNNNNNNNNNNTTGANNNNNN	4.28	IR:22	17
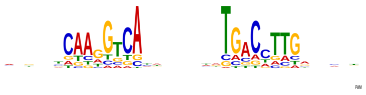	NNNNNNCAAGNNNNNNNNNNNNNNNNNNCTTGNNNNNNN	5.01	M	18

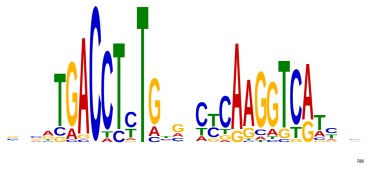	*NNNNNNACCTCTGNNNNNNNAGGTNNNNNN*	36.13	ER:10	19
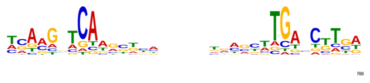	*NNNNNNTCAANNNNNNNNNNNNNNNTTGANNNNNN*	7.08	IR:17	20

82 ESRRG:RXRA-Bhimsaria2022-Rnd3 (S13-155)

PWM	Seed Sequence	Seed Seq Enrichment	Repeat	Rank
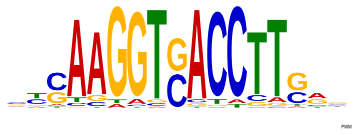	NNCAAGGTNACCTTGNN	737.06	M	1
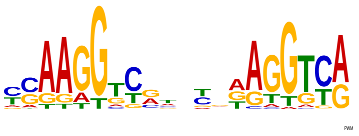	NCAAGGTCNNNNNNNGGTCN	255.24	DR:5	2
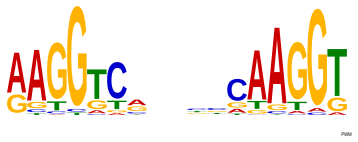	AAGGTCNNNNNCAAGGT	564.29	DR:6	3
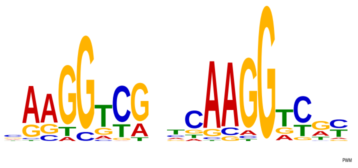	NNAGGTCNNNCAAGGTCNN	646.46	DR:4	4
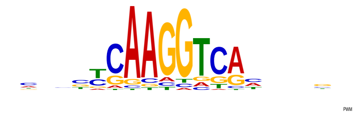	NNNNNNCAAGGTCANNNNNN	33.56	M	5
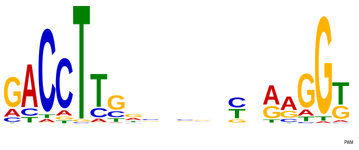	GACCTTNNNNNNNNNNAGGT	146.56	ER:11	6
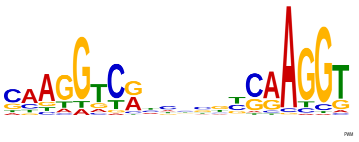	CAAGGNNNNNNNNNNAAGGT	88.13	DR:8	7
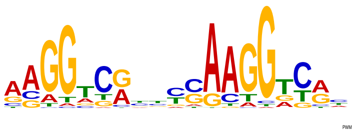	NNGGTCNNNNCAAGGTCNN	260.86	DR:5	8
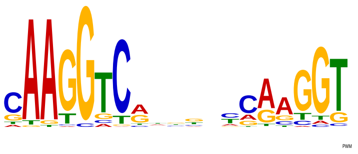	CAAGGTCNNNNNNNAGGT	157.70	DR:7	9

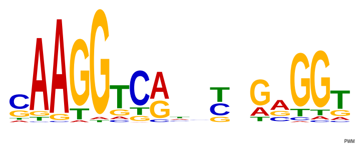	CAAGGTCNNNNNNCGGT	138.06	DR:5	10
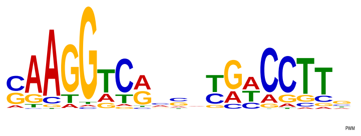	NAAGGTCNNNNNNNCCTTN	72.96	IR:3	11
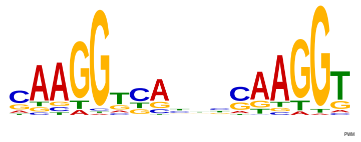	CAAGGTNNNNNCAAGGT	596.68	DR:5	12
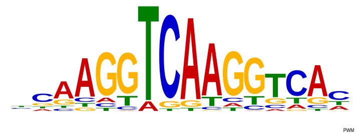	NNAAGGTCAAGGTCNN	160.56	DR:0	13
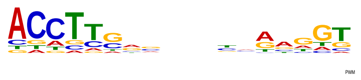	ACCTTNNNNNNNNNNAGGT	23.00	ER:10	14
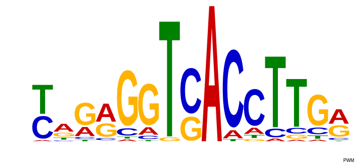	NNAGAGGTNACCTTNN	73.86	M	15
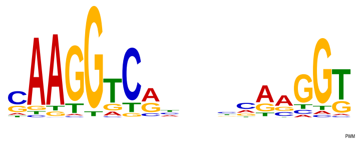	CAAGGTCNNNNNNNAGGT	209.79	DR:6	16
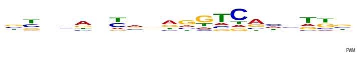	NNNNNNTAGAGGTCNNNNNN	5.91	M	17
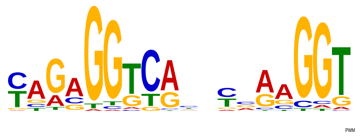	CAGAGGTNNNNNNNAGGT	83.89	DR:5	18

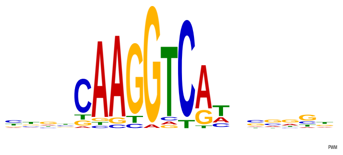	$NNNNNCA\overrightarrow{AGGTCA}TTNNNNN$	65.56	M	19
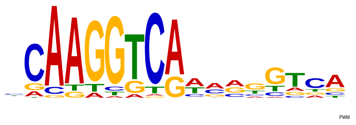	$NCA\overrightarrow{AGGTCN}NNN\overrightarrow{NGGTCN}$	56.31	DR:2	20

83 ESRRG-Bhimsaria2022-Rnd2 (S07-039)

PWM	Seed Sequence	Seed Seq Enrichment	Repeat	Rank
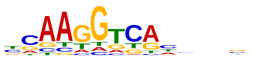	NNNNNNCAAGGTCANNNNNN	7.63	M	1
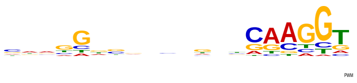	CAAGNNNNNNNNNNNNAAGGT	7.44	M	2
	NNNNNNTAGAGGTCNNNNNN	2.44	M	3
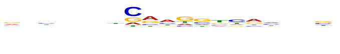	NNNNNNTGAAGGTCNNNNNN	2.48	M	4
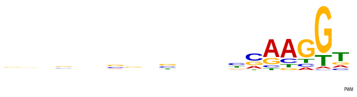	GGTGNNNNNNNNNNNNAAGGT	6.64	DR:11	5
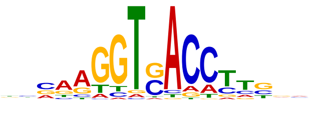	NNNNAAGGTNACCTTNNNN	16.81	M	6
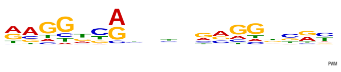	NNAGGTCNNNNNNNGGTCNN	5.89	DR:5	7
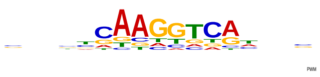	NNNNNGTCAAGGTCNNNNN	6.11	DR:0	8
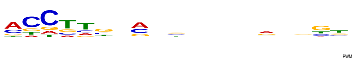	ACCTTNNNNNNNNNNNAGGT	5.00	ER:11	9

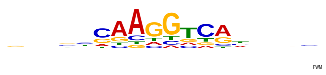	*NNNNNNTCAAGGTCNNNNNN*	5.15	DR:0	10
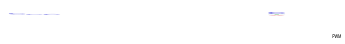	*TCCCNNNNNNNNNNNNCCCG*	1.85	M	11
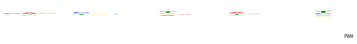	*CAGTNNNNNNNNNNNNCGTG*	1.87	M	12
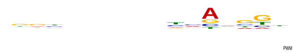	*CAGANNNNNNNNNNNNAGGT*	2.09	M	13
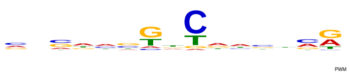	*NNCAAGGTNACAGNN*	2.44	M	14
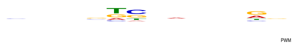	*NNNCAAGGNNACAGNNN*	1.53	M	15
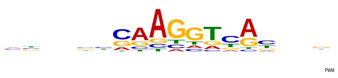	*NNNNNTTCAAGGTCNNNNN*	5.45	DR:0	16
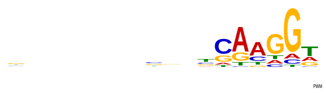	*GGGTNNNNNNNNNNNNAAGGT*	5.08	DR:10	17
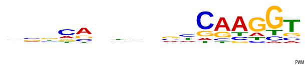	*AGAGNNNNNNNNNNNNAAGGT*	5.23	M	18

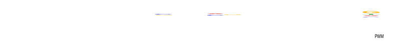	*GGGGNNNNNNNNNNNNTGCG*	1.93	M	19
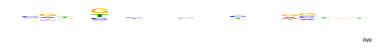	*CGTANNNNNNNNNNNNGTGT*	1.74	M	20

84 ESRRG-Bhimsaria2022-Rnd3 (S07-159)

PWM	Seed Sequence	Seed Seq Enrichment	Repeat	Rank
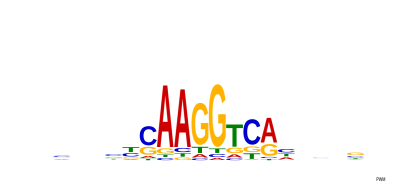	NNNNNNCAAGGTCANNNNNN	34.77	M	1
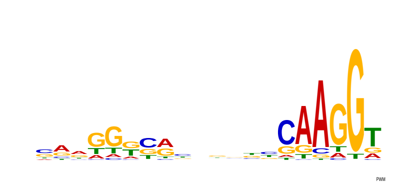	CAAGGNNNNNNNNNCAAGGT	217.52	DR:8	2
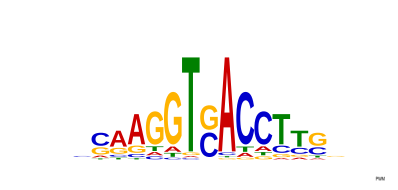	NNNNAAGGTNACCTTNNNN	85.03	M	3
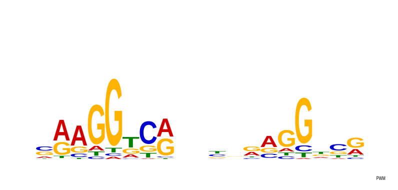	NNAGGTCNNNNNNNGGTCNN	42.28	DR:5	4
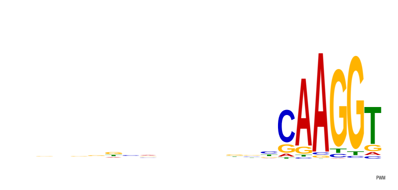	GGGGNNNNNNNNNNCAAGGT	135.41	M	5
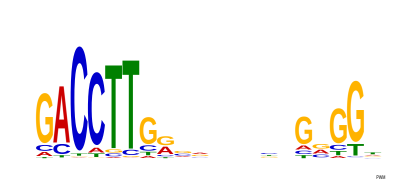	GACCTTGNNNNNNNNNGGGT	133.62	ER:11	6
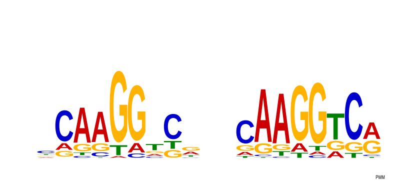	NCAAGGNNNNNCAAGGTCN	223.78	DR:4	7
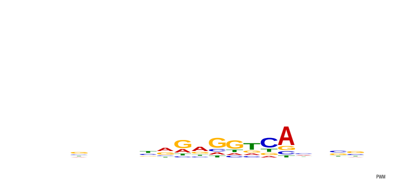	NNNNNNTAGAGGTCNNNNNN	7.62	M	8
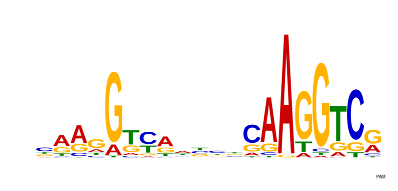	NAAGGNNNNNNCAAGGTCN	124.55	DR:6	9

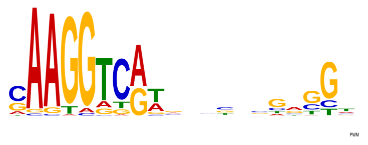	CAAGGTCNNNNNNNNGGGGT	218.38	DR:8	10
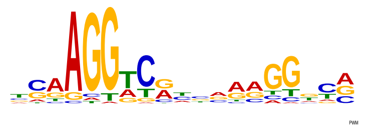	NGAAGGTCNNNNNNGGTCN	40.39	DR:4	11
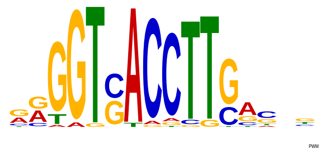	NNNGGGTCACCTTGANNN	216.37	IR:3	12
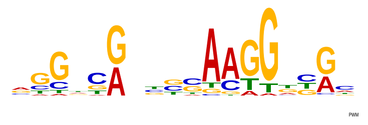	NGGTCNNNNCAAGGTCAN	110.88	DR:5	13
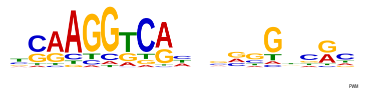	NNAAGGTCNNNNNGGTCNN	22.42	DR:3	14
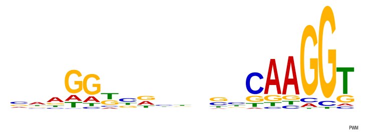	CAAGGNNNNNNNNCAAGGT	83.15	DR:7	15
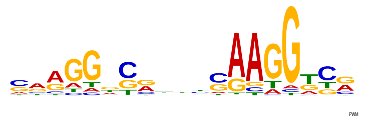	NAAGGNNNNNNNCAAGGTCN	117.00	DR:5	16
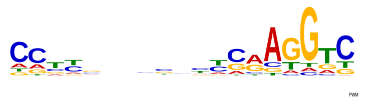	CCTTNNNNNNNNNAAGGTC	38.56	M	17
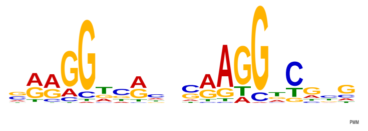	NNNGGTCANNNAAGGTCNNN	76.95	DR:4	18

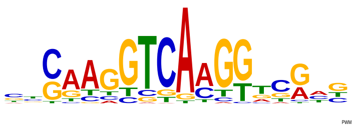	$NNNA\overrightarrow{AGGTCA}\overrightarrow{AGGTCN}NN$	109.35	DR:0	19
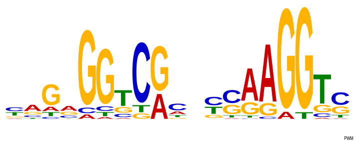	$NAG\overrightarrow{AGGTCN}NNNNA\overrightarrow{AGGTN}$	105.74	DR:5	20

85 ESRRG-Jolma2013-Rnd3 (ERR193996)

PWM	Seed Sequence	Seed Seq Enrichment	Repeat	Rank
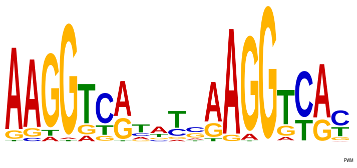	NAGGTCANNNNNAAGGTCAN	5023.47	DR:5	1
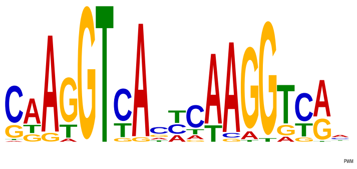	NAAGGTCANNCAAGGTCAN	39055.82	DR:4	2
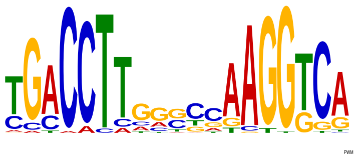	TGACCTTNNNNNNAGGTCA	5067.51	ER:7	3
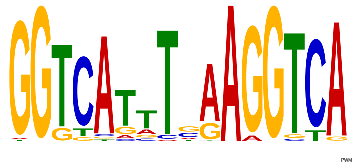	GGTCATTTNAAGGTCA	27282.20	DR:5	4
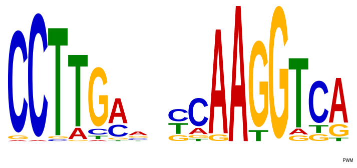	CCTTGANNNCAAGGTCA	11174.80	DR:0	5
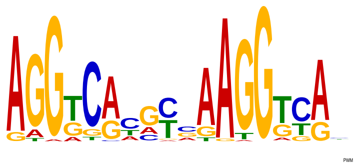	AGGTCNNNNNAAGGTCAA	2956.41	DR:5	6
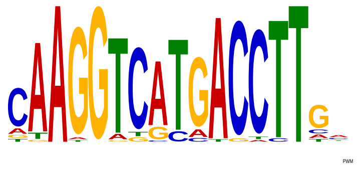	NAAGGTCATGACCTTGN	28544.51	IR:0	7
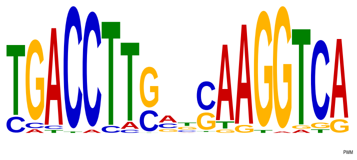	TGACCTTNNNNNAGGTCA	3314.68	ER:6	8
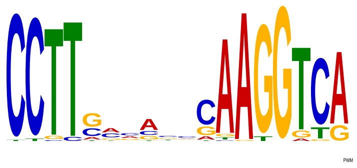	CCTTGNNNNNCAAGGTCA	5288.09	M	9

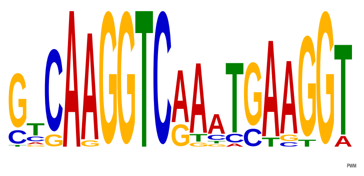	NTCAAGGTCNNNNNAAGGN	728.82	M	10
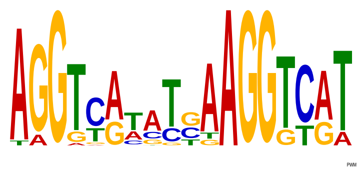	NGGTCATNNGAAGGTCAN	5573.12	DR:5	11
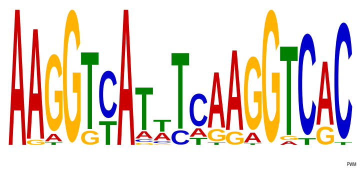	NAGGTCATTNNNNGGTCAN	1832.05	DR:5	12
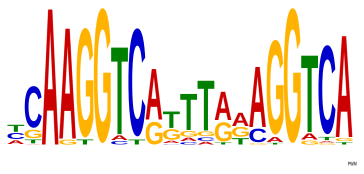	NCAAGGTCANNNNNAGGTCN	8309.44	DR:5	13
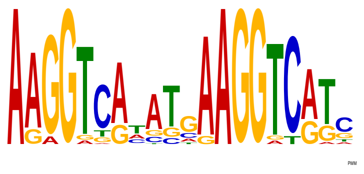	NNGGTCANNNGAAGGTCANN	4727.69	DR:5	14
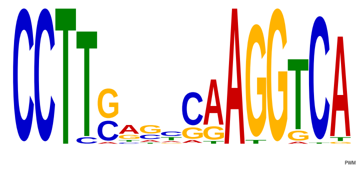	CCTTGAGNNAAGGTCA	6490.91	ER:1	15
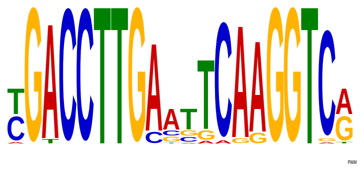	NGACCTTGNNNNNNAGGTCN	3376.64	ER:8	16
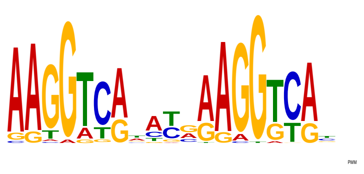	NNGGTCANNNNNAGGTCANN	797.52	DR:5	17
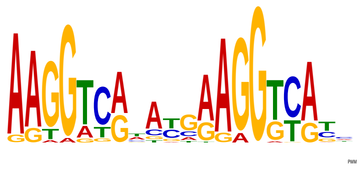	NNGGTCANNNNAAGGTCANN	1953.06	DR:5	18

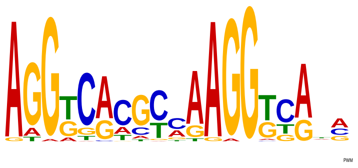	$\overrightarrow{NGGTCA}NNNNA\overrightarrow{AGGTCA}AN$	2866.37	DR:5	19
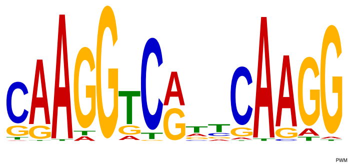	$CA\overrightarrow{AGGTCN}TTCAAGG$	5594.33	M	20

86 ESRRG-Jolma2013-Rnd4 (ERR193997)

PWM	Seed Sequence	Seed Seq Enrichment	Repeat	Rank
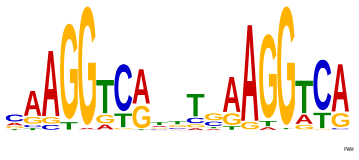	NNNGGTCNNNNNAAGGNNN	103.39	DR:5	1
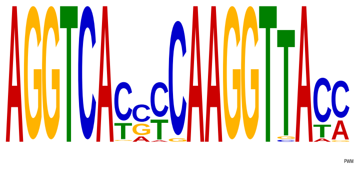	NGGTCACNNNNNGGTTACN	946.08	DR:5	2
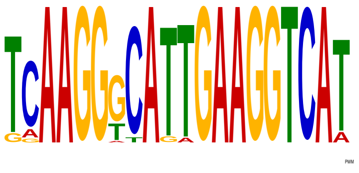	NCAAGGGCANNNNNGGTCAN	1942.05	DR:4	3
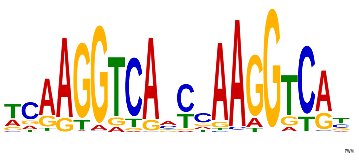	NNNNGGTCNNNNAAGGNNNN	94.67	DR:4	4
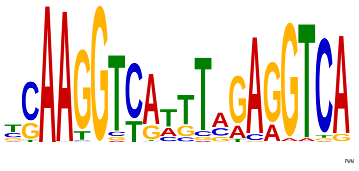	TCAAGNNNNNNNNNNNGTCA	288.08	M	5
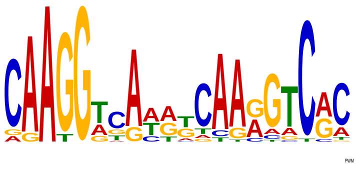	NNNGGTCNNNNNNAGGGNNN	12.17	DR:5	6
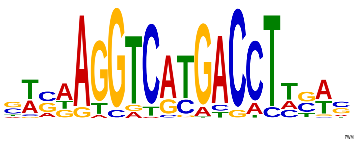	NNNNNGGTCNNGACCNNNNN	25.84	IR:0	7
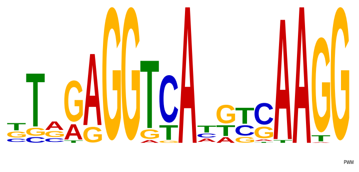	NTAGAGGTNNNNNCAAGN	313.32	M	8
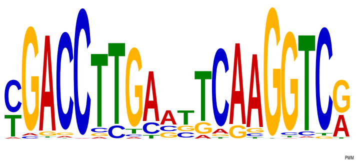	NNACCTNNNNNNNNAGGTNN	33.80	ER:8	9

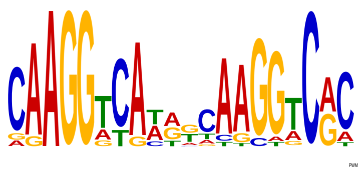	$NN\overrightarrow{NGGTCN}NNNNA\overrightarrow{AGGGNN}N$	33.73	DR:5	10
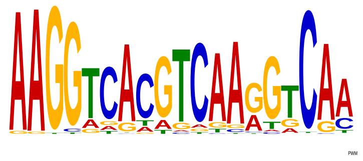	$N\overrightarrow{NNGTCA}NNNNNN\overrightarrow{AGGGNN}N$	22.71	DR:5	11
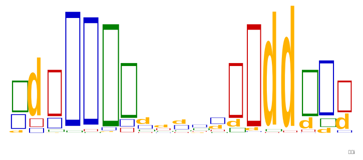	$\overleftarrow{NNACCT}NNNNNNN\overrightarrow{AGGTNN}$	34.92	ER:7	12
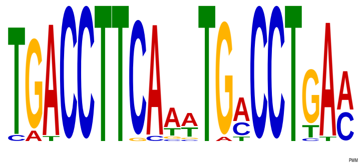	$NNN\overrightarrow{NCTTCA}TN\overleftarrow{NGACCN}NNN$	150.06	IR:2	13
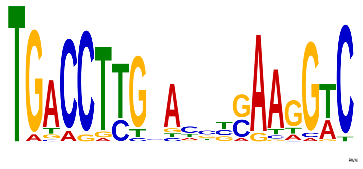	$\overleftarrow{NNACCT}NNNNNNNNA\overrightarrow{AGGNN}$	7.52	ER:9	14
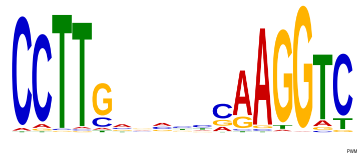	$NCTTGNNNNNNN\overrightarrow{AGGTN}$	39.68	M	15
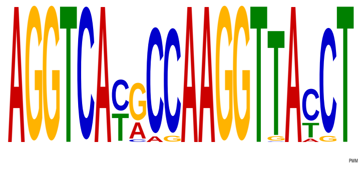	$\overrightarrow{NNGTCA}CNNNNN\overrightarrow{NGGTTA}CNN$	483.79	DR:5	16
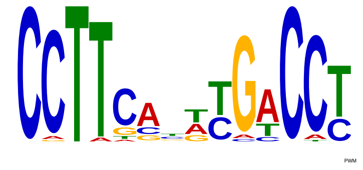	$\overrightarrow{NCTTCA}TN\overleftarrow{CGACCN}$	229.37	IR:2	17
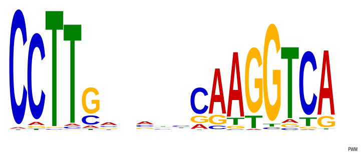	$NCTTGNNNNNNN\overrightarrow{NNGTCA}N$	55.27	M	18

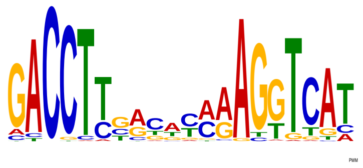	$\overleftarrow{NACCT}NNNNNNNN\overrightarrow{NNGTCA}N$	27.19	ER:8	19
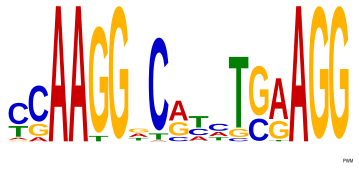	$NCA\overrightarrow{AGGACN}NN\overleftarrow{TGAAG}N$	644.21	IR:2	20

87 ESRRG-4-Bhimsaria2022-Rnd2 (S07-003)

PWM	Seed Sequence	Seed Seq Enrichment	Repeat	Rank
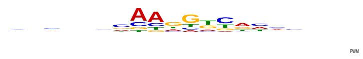	NNNNNNCAAGGTCANNNNNNN	3.69	M	1
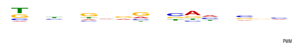	NNNNNNTAGAGGTCNNNNNN	2.05	M	2
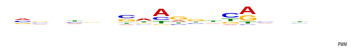	NNNNNNTCAAGGTCNNNNNN	2.82	DR:0	3
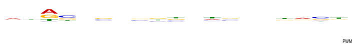	ATACNNNNNNNNNNNTACT	1.78	M	4
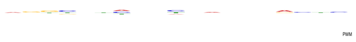	AGGCNNNNNNNNNNNACTC	2.06	M	5
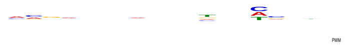	CACGNNNNNNNNNNNNCATT	1.85	M	6
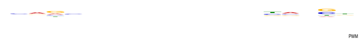	CAGCNNNNNNNNNNNNAAGT	1.81	M	7
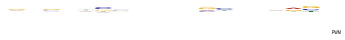	TGGGNNNNNNNNNNNNAAGG	1.68	M	8
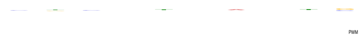	CGTTNNNNNNNNNNNCTGG	1.69	M	9

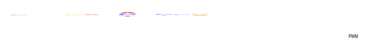	*CCGGNNNNNNNNNNNNGTGT*	1.63	M	10
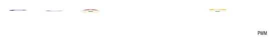	$\overrightarrow{AGGCN}$*NNNNNNNNNNNTGCG*	1.68	M	11
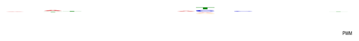	*AAATNNNNNNNNNNNNTTTT*	1.62	M	12
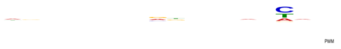	*TAGTNNNNNNNNNNNNGCAT*	1.79	M	13
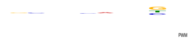	*CAGTNNNNNNNNNNNN*$\overrightarrow{AGGT}$	1.69	M	14
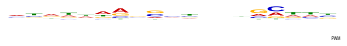	*NATATNNNNNNNNNNGCTTN*	2.43	M	15
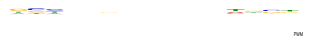	*GTAGNNNNNNNNNNNNTGCT*	1.76	M	16
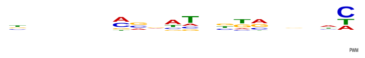	*NCATANNNNNNNNNNTGTTN*	1.30	M	17
	GGGGNNNNNNNNNNNNGGCG	1.76	M	18

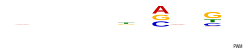	*CGAGNNNNNNNNNNNN*$\overrightarrow{AGGT}$	1.78	M	19
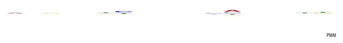	*CAAGNNNNNNNNNNNGCTT*	1.78	M	20

88 ESRRG-4-Bhimsaria2022-Rnd3 (S07-124)

PWM	Seed Sequence	Seed Seq Enrichment	Repeat	Rank
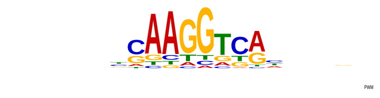	NNNNNNCAAGGTCANNNNNN	27.90	M	1
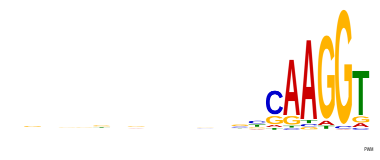	GGGGNNNNNNNNNNCAAGGT	95.93	M	2
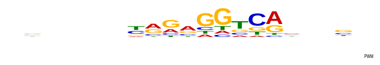	NNNNNNTAGAGGTCNNNNNN	8.28	M	3
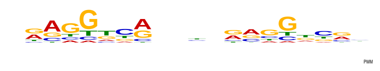	NNGGTCNNNNNNNGGTCNN	9.94	DR:5	4
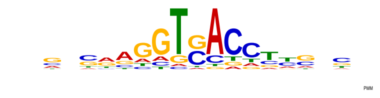	NNNNAAGGTNACCTTNNNN	43.31	M	5
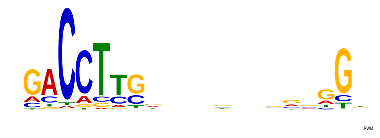	GACCTTNNNNNNNNNNGGGT	33.33	ER:11	6
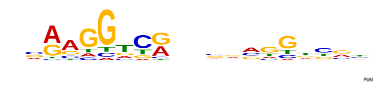	NNAGGTCNNNNNNGGTCNN	14.27	DR:4	7
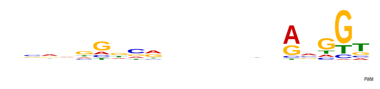	CAAGNNNNNNNNNNNGAGGT	13.40	M	8
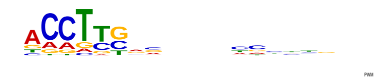	ACCTTNNNNNNNNNNNTGGT	13.84	ER:12	9

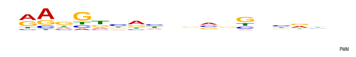	*NNNGGTCNNNNNGGTCNNN*	5.65	DR:3	10
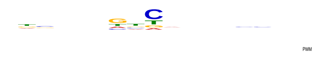	*NNNNCAAGNNNNACAGNNNN*	3.54	M	11
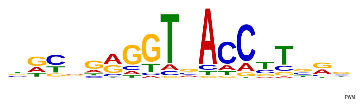	*NNNAGAGGTNACCTTNNN*	40.54	M	12
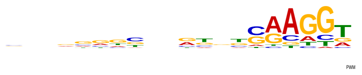	*CCAANNNNNNNNNNNNAAGGT*	14.30	M	13
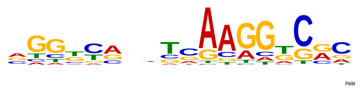	*NNGTCANNNCAAGGTCNN*	47.63	DR:5	14
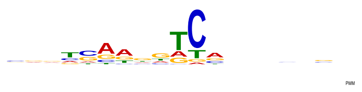	*NNNNCAAGGNNACAGNNNN*	6.32	M	15
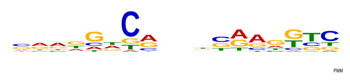	*NCAAGGNNNNNNNAAGGTN*	13.90	DR:5	16
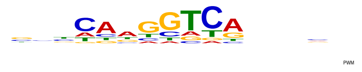	*NNCCAAGGNNACAGNN*	7.46	M	17
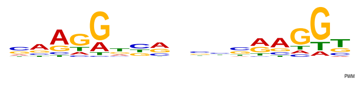	*CAAGGTNNNNNNAAGGT*	49.07	DR:5	18

89 ESRRG-Yin2017-Rnd3 (ERR1003046)

PWM	Seed Sequence	Seed Seq Enrichment	Repeat	Rank
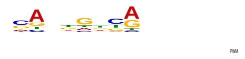	NNNNNNCTCANNGTCANNNNNN	4.25	DR:0	1
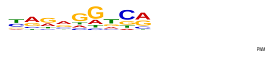	NNNNNNTAGANNTCATNNNNNN	3.35	M	2
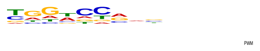	NNNNNNTGGTCCATNNNNNN	3.22	M	3
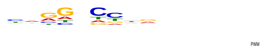	NNNNNNCAGGNCCTGNNNNNN	3.11	M	4
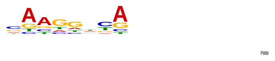	NNNNNNCAAGNNNATCGNNNNNN	3.49	M	5
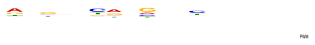	NNNNNNGTCANGTCCNNNNNN	2.70	M	6
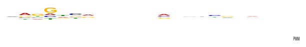	NNNNNNAGGTNNNNNNNNGGACNNNNNN	2.37	DR:5	7
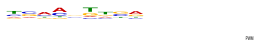	NNNNNNATGANNNTCATNNNNNN	3.14	M	8
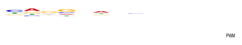	NNNNNNGCAGGTAGNNNNNN	2.01	M	9

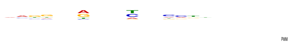	NNNNNNAAGGNNNNNNNNCCTTNNNNNN	2.67	IR:3	10
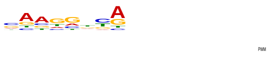	NNNNNNCAAGNNNNACGCNNNNNN	3.24	M	11
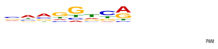	NNNNNNAGTCNNGGTCNNNNNN	2.79	DR:0	12
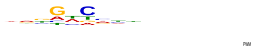	NNNNNNCAATNNNATTGNNNNNN	2.43	M	13
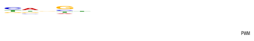	NNNNNNCAGGTACANNNNNN	2.12	M	14
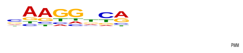	NNNNNNGCCANNNTCATNNNNNN	2.87	DR:0	15
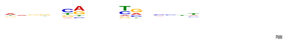	NNNNNNCAAGNNNNNNNNNNNCTTGNNNNNN	2.46	M	16
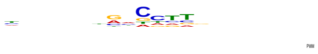	NNNNNNATTTNNNNTTGTNNNNNN	2.06	M	17
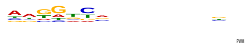	NNNNNNGCAANNNNATTTNNNNNN	1.85	M	18

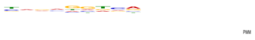	NNNNNNGTAGNNNTCAGNNNNNN	1.87	M	19
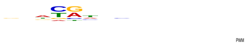	NNNNNNAGGANNTCCTNNNNNN	1.96	M	20

90 ESRRG-Yin2017-Rnd3 (ERR1003047)

PWM	Seed Sequence	Seed Seq Enrichment	Repeat	Rank
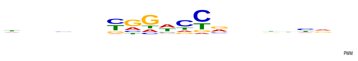	NNNNNNTGGTCCAANNNNNN	4.00	M	1
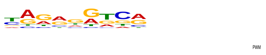	NNNNNNTAGANNTCATNNNNNN	4.12	M	2
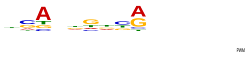	NNNNNNCTCANNGTCANNNNNN	4.11	DR:0	3
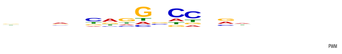	NNNNNNCAGGNCCTANNNNNN	3.19	M	4
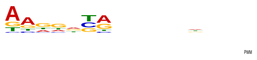	NNNNNNCAAGNNNAAATNNNNNN	3.31	M	5
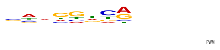	NNNNNNAGTCNNGGTCNNNNNN	2.99	DR:0	6
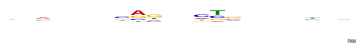	NNNNNNTCAGNNCTGANNNNNN	2.76	IR:4	7
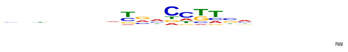	NNNNNNAAATNNNNTTCANNNNNN	3.58	M	8
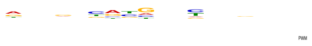	NNNNNNGTCANGTCCNNNNNN	2.74	M	9

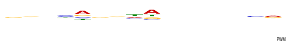	NNNNNNTCAGGTAGNNNNNN	2.34	M	10
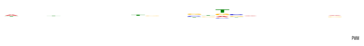	NNNNNNATTTNNNNNTCAANNNNNN	2.49	M	11
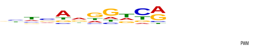	NNNNNNGCTCNNGGTCNNNNNN	3.37	DR:0	12
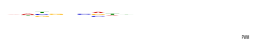	NNNNNNGCAANNNNNTTGCNNNNNN	2.19	M	13
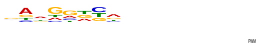	NNNNNNTCAANNNNAAAANNNNNN	2.35	M	14
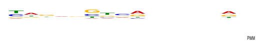	NNNNNNACAGNNNTCAANNNNNN	2.19	M	15
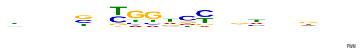	NNNNNNTGGTCCCGNNNNNN	2.90	M	16
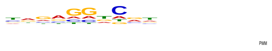	NNNNNNTAGANNNCGTTNNNNNN	2.75	M	17
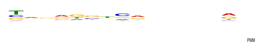	NNNNNNCTAGNNNTCAANNNNNN	2.44	M	18

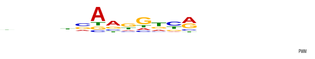	$NNNN\overrightarrow{NNCTCA}\overrightarrow{NNNTCA}TNNNNNN$	3.65	DR:0	19
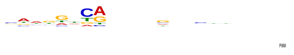	$NNNNNNCAAGNNNNNN\overrightarrow{NNGTCC}NNNNNN$	2.31	M	20

91 ESRRG-Yin2017-Rnd4 (ERR1003048)

PWM	Seed Sequence	Seed Seq Enrichment	Repeat	Rank
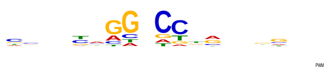	NNNNNNCAGGNCCTGNNNNNN	5.07	M	1
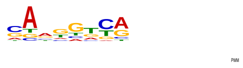	NNNNNNCTCANNNTCATNNNNNN	4.45	DR:0	2
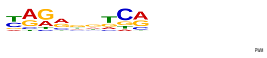	NNNNNNTAGANNTCATNNNNNN	4.16	M	3
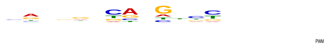	NNNNNNGTCANGTCCNNNNNN	3.60	M	4
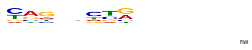	NNNNNNGCAGNNCTGCNNNNNN	2.45	M	5
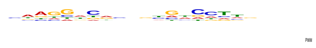	NNNNNNAGGANNNNNNNTCCTNNNNNN	2.67	IR:3	6
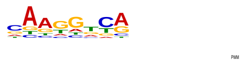	NNNNNNGCCANNNTCATNNNNNN	3.80	DR:0	7
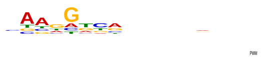	NNNNNNGCAANNNNAAATNNNNNN	2.48	M	8
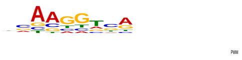	NNNNNNAGTCNNNNTCACNNNNNN	2.81	DR:0	9

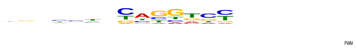	*NNNNNNCCAGNTCCCNNNNNN*	2.24	M	10
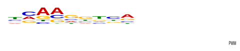	*NNNNNNAGTTNNNNNTCATNNNNNN*	2.22	DR:0	11
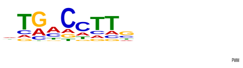	*NNNNNNCGATNNNNTTGGNNNNNN*	3.15	M	12
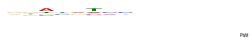	*NNNNNNAGGANNTCCTNNNNNN*	2.20	M	13
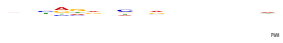	*NNNNNNACAGNNCTAGNNNNNN*	1.91	M	14
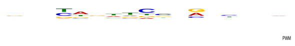	*NNNNNNCTAGNTCCTNNNNNN*	2.14	M	15
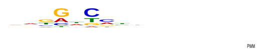	*NNNNNNCAATNNNATTGNNNNNN*	2.40	M	16
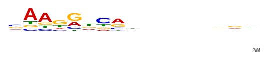	*NNNNNNGCAANNNNATTTNNNNNN*	2.48	M	17
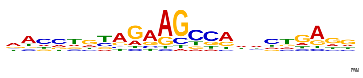	*NNNNNNTAGANNNCAAANNNNNN*	3.71	M	18

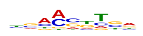 PWM	$NNNN\overrightarrow{NNGTGA}NNNNNAAACNNNNNN$	2.18	M	19
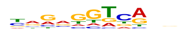 PWM	$NNNNNNTAG\overrightarrow{ANNNCA}CGNNNNNN$	2.59	M	20

92 ESRRG-Yin2017-Rnd4 (ERR1003049)

PWM	Seed Sequence	Seed Seq Enrichment	Repeat	Rank
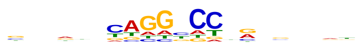	NNNNNNCAGGNCCTANNNNNNN	5.28	M	1
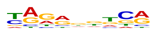	NNNNNNTAGANNTCATNNNNNN	4.15	M	2
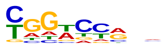	NNNNNNACGGTCCGNNNNNN	4.99	M	3
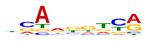	NNNNNNNCTCANNNTCATNNNNNN	4.23	DR:0	4
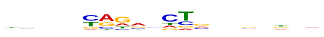	NNNNNNACAGNNCTGANNNNNN	3.37	M	5
	NNNNNNATTTNNNNNTCATNNNNNN	3.15	M	6
	NNNNNNAGCCNNGGTCNNNNNN	3.08	M	7
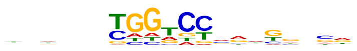	NNNNNNTGGTCCCANNNNNN	5.41	M	8
	NNNNNNGTCANGTCCNNNNNN	3.22	M	9

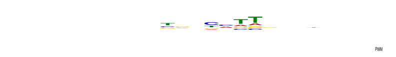	NNNNNNGAATNNNNTTGTNNNNNN	2.64	M	10
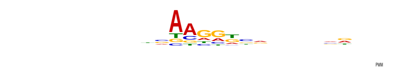	NNNNNNGTTCNNNNNCAAANNNNNN	1.71	M	11
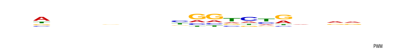	NNNNNNATCGGTCTNNNNNN	2.69	M	12
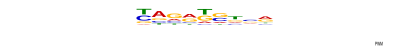	NNNNNNGCAGNNNTCACNNNNNN	2.48	M	13
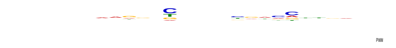	NNNNNNAGGANNNNNNGACCNNNNNN	2.28	IR:3	14
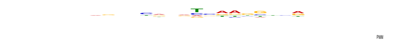	NNNNNNGGACNNNNNNNNGTCANNNNNN	2.23	DR:5	15
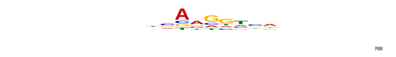	NNNNNNAGTCNNNNTCACNNNNNN	2.69	DR:0	16
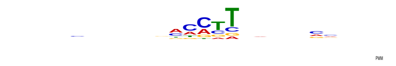	NNNNNNTTTGNNNNNGAGANNNNNN	1.35	M	17
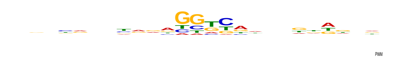	NNNNNNTAGANNNNAAATNNNNNN	2.06	M	18

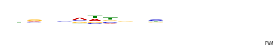	*NNNNNNCGTCNNNGACGNNNNNN*	2.13	IR:1	19
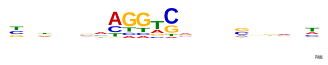	*NNNNNNCTCANNNNNNAAAANNNNNN*	1.34	M	20

93 ESRRG-Yin2017-Rnd2 (ERR1003052)

PWM	Seed Sequence	Seed Seq Enrichment	Repeat	Rank
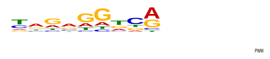	NNNNNNTAGANGTCANNNNNNN	5.04	M	1
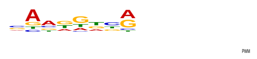	NNNNNNNTCAANNTCATNNNNNN	4.88	DR:0	2
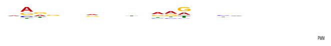	NNNNNNGTCANNNNNNNGTCANNNNNNN	2.87	DR:5	3
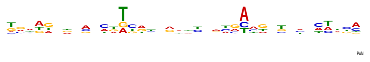	NNNNNGTCANNNNNNNNNNNNNNNNNNNNNTGACNNNNN	2.42	IR:20	4
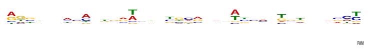	NNNNTGACNNNNNNNNNNNNNNNNNNNNNNNGTCANNNN	2.56	ER:18	5
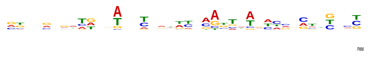	NGTCANNNNNNNNNNNNNNNNNNNNNNNNNNNNNNNGTCAN	2.59	DR:28	6
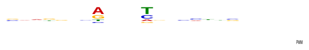	NNNNNNAAGGNNNNNNNNNCCTTNNNNNN	2.70	IR:3	7
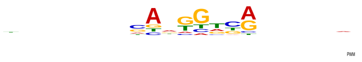	NNNNNNTCCANGGTCANNNNNNN	5.16	M	8
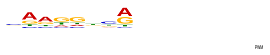	NNNNNNCAAGNNCACGNNNNNNN	3.47	M	9

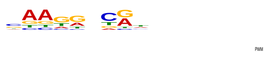	NNNNNNCAAGNNCGTTNNNNNN	4.20	M	10
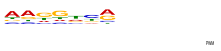	NNNNNNGCCANNGTCANNNNNN	3.20	DR:0	11
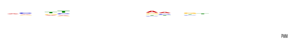	NNNNNNACCTNNNNNNNNAGGTNNNNNN	2.64	ER:8	12
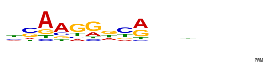	NNNNNNTCAAGNGCAANNNNNN	5.42	DR:0	13
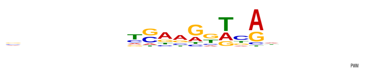	NNNNNNTTTGANNGTCANNNNNN	5.50	M	14
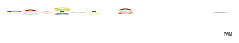	NNNNNNGTCANNGACANNNNNN	2.76	DR:0	15
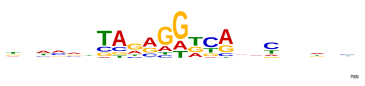	NNNNNNTAGAGNTCAAANNNNNN	8.24	M	16
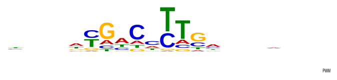	NNNNNNACGACNNTGAGNNNNNN	5.33	IR:1	17
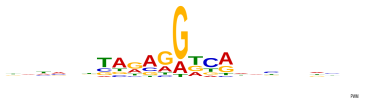	NNNNNNTAGAGNNCAAANNNNNN	5.63	M	18

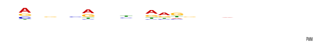	$NNNNN\overrightarrow{NGGTCN}NNNNN\overrightarrow{NNGTCA}NNNNNN$	2.57	DR:5	19
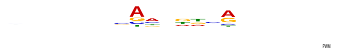	$NNNNN\overrightarrow{NTGTCA}\overrightarrow{NNGTCA}NNNNNN$	4.87	DR:0	20

94 ESRRG-Yin2017-Rnd3 (ERR1003054)

PWM	Seed Sequence	Seed Seq Enrichment	Repeat	Rank
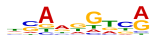	NNNNNNCTCANNGTCANNNNNN	5.54	DR:0	1
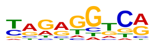	NNNNNNTAGANNTCATNNNNNN	5.28	M	2
	NNNNNNCAAGNNNNNNNNAAGGNNNNNN	3.55	M	3
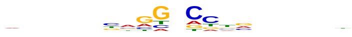	NNNNNNCAGGNCCTGNNNNNN	3.21	M	4
	NNNNNNAAGGNNNNNNNNNCCTTNNNNNN	3.62	IR:3	5
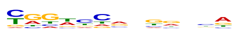	NNNNNNCGGTCCAANNNNNN	3.39	M	6
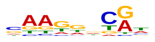	NNNNNNCAAGNNCGTTNNNNNN	4.53	M	7
	NNNNNNAAGGNNNCCTTNNNNNN	3.49	M	8
	NGTCANNNNNNNNNNNNNNNNNNNNNNNNNNNNNNTGACN	3.50	IR:30	9

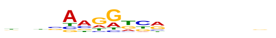	NNNNNNNTCAANNNNNNAAATNNNNNN	3.08	M	10
	NNNNNNNTCAANNNNNNATTGNNNNNN	3.22	M	11
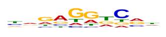	NNNNNNTAGANNNCGTTNNNNNN	3.03	M	12
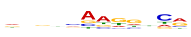	NNNNNNCGTCNNNGTCGNNNNNN	3.25	DR:0	13
	NTCAANNNNNNNNNNNNNNNNNNNNNNNNNNNNTTAGN	2.04	M	14
	NNNNNNTGAANNNNNNTTCANNNNNN	2.26	ER:1	15
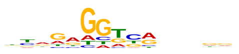	NNNNNNTAGANNNCAANNNNNN	2.96	M	16
	NNNNNNGCAANNNNNNNNNNNNGTCANNNNNN	2.74	M	17
	NNNNNNNTCAANNNNNACGANNNNNN	2.68	M	18

	$NNNNN\overrightarrow{NGTCA}NNN\overleftarrow{TGACN}NNNNN$	3.34	IR:3	19
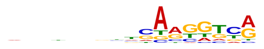	$NNNNN\overrightarrow{NGTTCA}\overrightarrow{NNNTCA}TNNNNNN$	5.92	DR:0	20

95 ESRRG-Yin2017-Rnd4 (ERR1003056)

PWM	Seed Sequence	Seed Seq Enrichment	Repeat	Rank
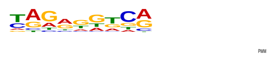	NNNNNNTAGANNTCATNNNNNN	4.66	M	1
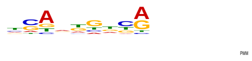	NNNNNNCTCANNGTCANNNNNN	4.29	DR:0	2
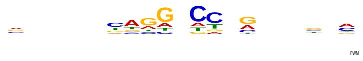	NNNNNNCAGGNCCTANNNNNN	4.24	M	3
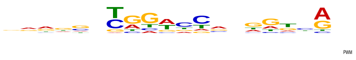	NNNNNNCGGTCCAANNNNNN	3.89	M	4
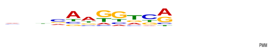	NNNNNNAGTCNNNGTCANNNNNN	3.85	DR:0	5
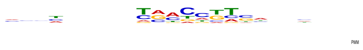	NNNNNNATTTNNNNTTCANNNNNN	3.50	M	6
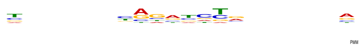	NNNNNNTCAGNNCTGANNNNNN	2.88	IR:4	7
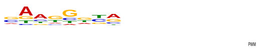	NNNNNNTCAANNNNNATTGNNNNNN	3.19	M	8
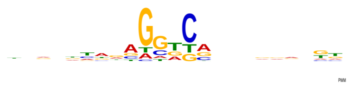	NNNNNNTAGANNNNATTTNNNNNN	2.41	M	9

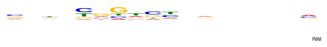	NNNNNNCGGTCTCGNNNNNN	2.36	M	10
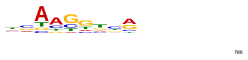	NNNNNNGTTCNNNNTCATNNNNNN	3.02	DR:0	11
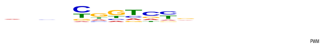	NNNNNNCGGTCTGCNNNNNN	2.49	M	12
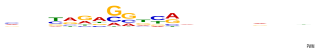	NNNNNNTAGANNNNAAATNNNNNN	2.54	M	13
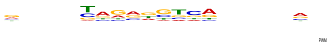	NNNNNNCTAGNNNTCAGNNNNNN	2.69	M	14
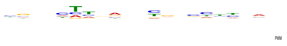	NNNNNNCGTCNNNNNNNCGACNNNNNN	2.03	IR:6	15
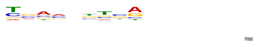	NNNNNNGTGANNNTCACNNNNNN	3.09	DR:0	16
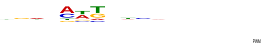	NNNNNNCGTCNNNGACGNNNNNN	1.91	IR:1	17
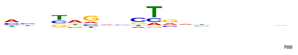	NNNNNNCTAGNNCTGANNNNNN	3.14	M	18

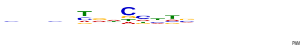	*NNNNNNGCGTNNNNTTGCNNNNNN*	2.25	M	19
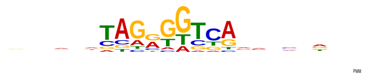	$NNNNNNTAG\overrightarrow{GNNTC}AAANNNNNN$	4.95	M	20

96 ESR1-5-Bhimsaria2022-Rnd2 (S04-142)

PWM	Seed Sequence	Seed Seq Enrichment	Repeat	Rank
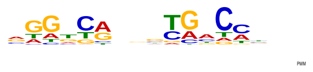	NNNNGGTCNNNNTGACNNNN	21.32	IR:3	1
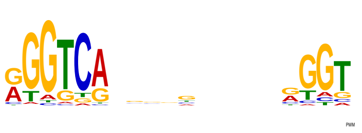	AGGTCANNNNNNNNNNGGGT	85.91	DR:10	2
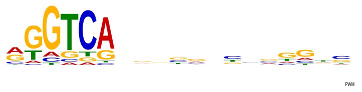	AGGTCANNNNNNNNNGGTC	18.03	DR:8	3
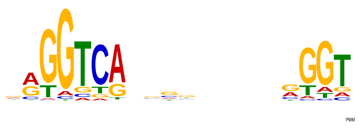	AAGGTCANNNNNNNNNGGGT	95.71	DR:9	4
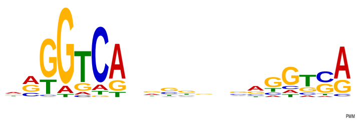	NAGGTCANNNNNNNNGGTCN	21.47	DR:7	5
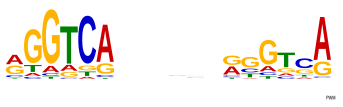	NAGGTCANNNNNNNGGTCN	22.14	DR:6	6
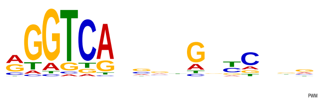	NNNGGTCANNNNGGTCNNN	22.77	DR:3	7
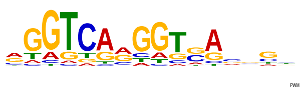	NNNNGGTCANGGTCANNNN	39.62	DR:0	8
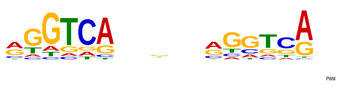	NNGGTCANNNNNNGGTCNN	12.41	DR:5	9

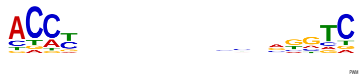	ACCTNNNNNNNNNNNNGGTC	11.87	ER:11	10
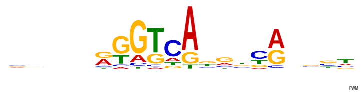	NNNNNAGGTCAGGTCNNNNN	16.69	M	11
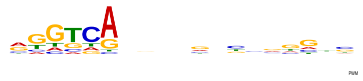	AGGTCNNNNNNNNNNGGTC	6.86	DR:8	12
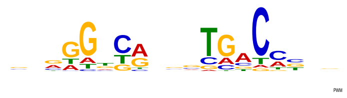	NNNNGGTCGNNGTGACNNNN	47.50	IR:3	13
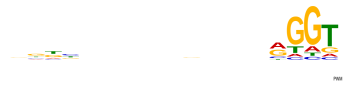	AGGGNNNNNNNNNNNNAGGT	8.35	DR:10	14
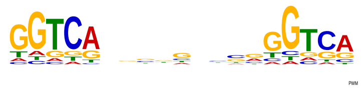	GGTCANNNNNNNNNGGTCA	30.74	DR:8	15
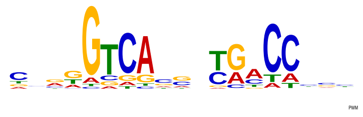	NNNTGTCACNNCGACCNNN	59.84	IR:3	16
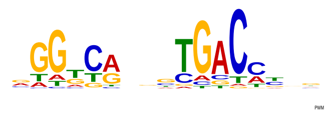	NNNGGTCGNNNTGACANNN	33.03	IR:3	17
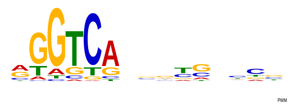	NNNNAGGTCANGGTGNNNN	38.03	DR:0	18

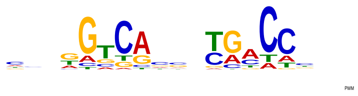	$NNN\overrightarrow{TGTCA}CNNN\overleftarrow{NGACC}NNN$	43.97	IR:3	19
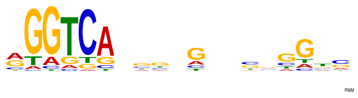	$\overrightarrow{GGGTCA}NNNNNNNN\overrightarrow{NGGTC}$	17.47	DR:8	20

97 ESR1-5-Bhimsaria2022-Rnd3 (S04-143)

PWM	Seed Sequence	Seed Seq Enrichment	Repeat	Rank
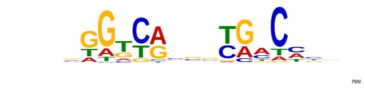	*NNNNGGTCANNNTGACNNNN*	381.53	IR:3	1
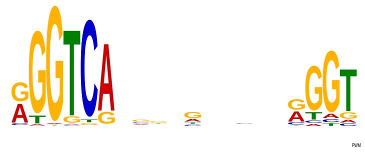	*AGGTCANNNNNNNNNNGGGT*	426.25	DR:10	2
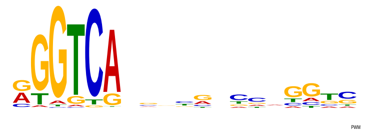	*AGGTCANNNNNNNNGGTC*	84.61	DR:8	3
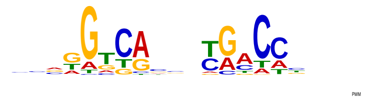	*NNNNGTCACNNNGACCNNNN*	330.62	IR:3	4
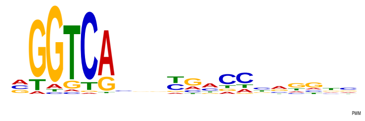	*AGGTCANNNNNNNNNGGTC*	59.99	DR:9	5
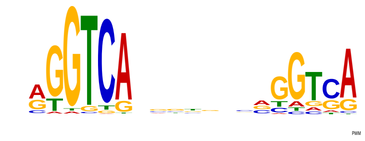	*NAGGTCANNNNNNNGGTCN*	101.48	DR:7	6
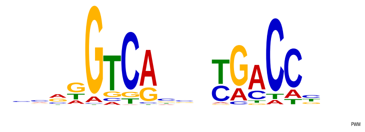	*NNNTGTCACNNNGACCNNN*	390.26	IR:3	7
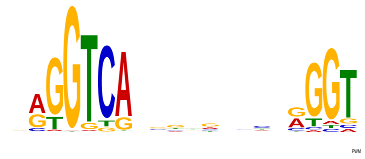	*AAGGTCANNNNNNNNNGGGT*	420.14	DR:9	8
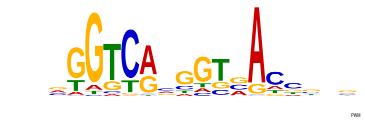	*NNNGGTCANGGTCACCNNN*	1028.31	DR:0	9

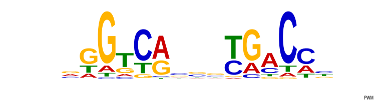	NNNNGTCANNNTGACNNNN	175.74	IR:3	10
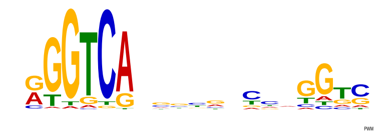	AGGTCANNNNNNNNGGGTC	84.68	DR:8	11
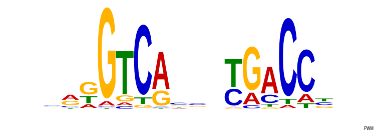	NNNTGTCANNNNGACCNNN	229.70	IR:3	12
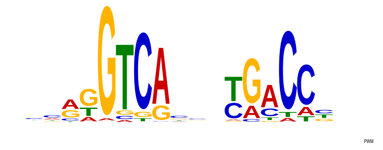	NNNTGTCACNNCGACCNNN	527.30	IR:3	13
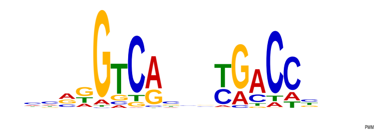	NNNTGTCACNNNGACCCNNN	505.31	IR:3	14
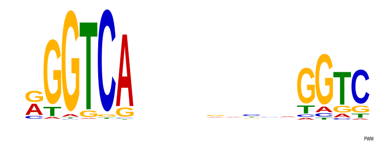	AGGTCANNNNNNNNAGGTC	123.73	DR:8	15
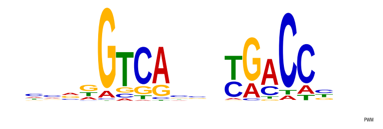	NNNTGTCACCNNGACCNNN	558.13	IR:3	16
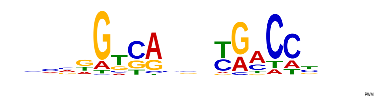	NNNNGTCACCNNGACCNNNN	453.24	IR:3	17
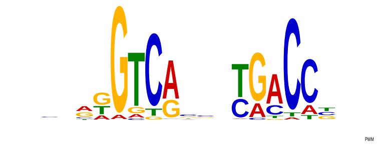	NNNGTGTCANNNNGACCNNN	305.86	IR:3	18

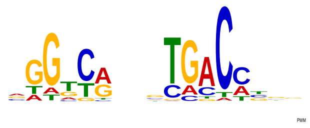	*NNNNGGTCGNNGTGACNNNN*	473.51	IR:3	19
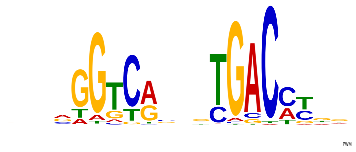	*NNNGGGTCGNNGTGACANNN*	672.76	IR:3	20

98 ESR1-Jolma2013-Rnd3 (ERR194971)

PWM	Seed Sequence	Seed Seq Enrichment	Repeat	Rank
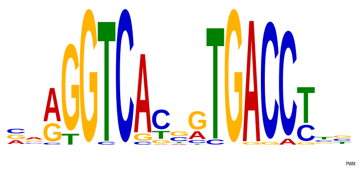	NNAGGTCANNNNGACCTNN	401.12	IR:3	1
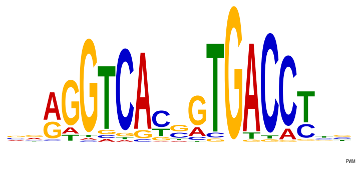	NNAGGTCNNNNNNACCTNN	27.09	IR:3	2
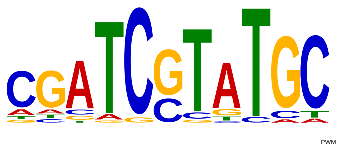	CGATCNTATGC	15.64	M	3
	NNNNGGGGNNNNGGGGNNNN	3.49	M	4
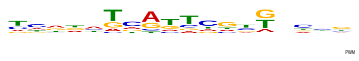	NNNNNTCATTCGTNNNNN	2.97	M	5
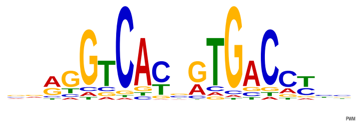	NNAGGTNNNNNNNNACCTNN	6.61	IR:3	6
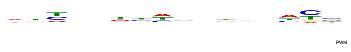	NNNATTTNNTTTTNNN	1.49	M	7
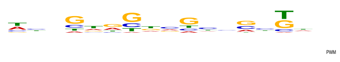	NNNNNTGGTCGCCNNNNN	1.91	M	8
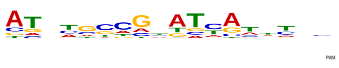	NNNNNGCCGNATCANNNNN	3.24	M	9

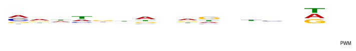	$NNNTGTAN\overrightarrow{NGCTCN}NN$	1.68	M	10
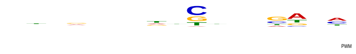	$NNN\overrightarrow{AGGANN}NGAAGNNN$	1.53	M	11
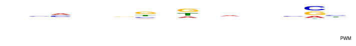	$NNNAC\overrightarrow{GGNNCA}TGNNN$	1.50	M	12
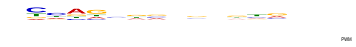	$NNCAGCNNNNNGTGTNN$	2.02	M	13
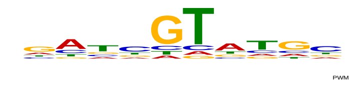	$\overrightarrow{GATCN}NATGC$	2.98	M	14
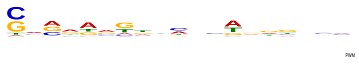	$NNNAAAGNNNNN\overrightarrow{AGGGNN}N$	2.24	M	15
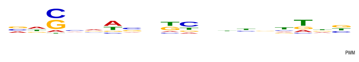	$NNNCAACNNNNNTTGTNNN$	2.16	M	16
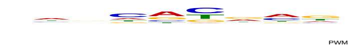	$\overrightarrow{AGCACG}AG$	2.06	M	17
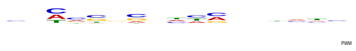	$NNNCCGCNNNCTGTNNN$	1.75	M	18

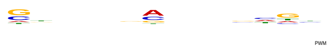	$NNNCG\overrightarrow{CGNGCA}GNNN$	1.31	M	19
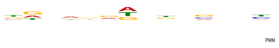	$NA\overrightarrow{AGGNNN}NNNN\overrightarrow{NGGTTN}$	1.46	DR:4	20

99 ESR1-Jolma2013-Rnd4 (ERR194972)

PWM	Seed Sequence	Seed Seq Enrichment	Repeat	Rank
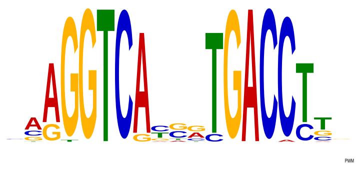	NNAGGTCANNNTGACCTNN	18527.22	IR:3	1
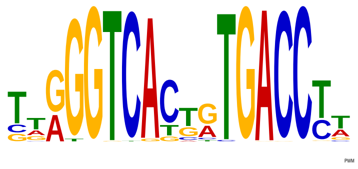	NTGGGTCANNNTGACCTN	8803.58	IR:3	2
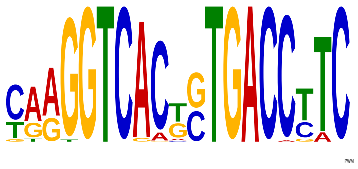	NNGGGTCACGNNGACCTNN	11244.78	IR:3	3
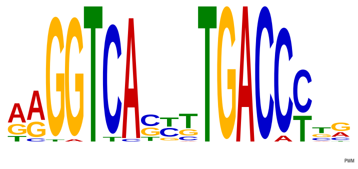	NAGGTCANNNNGACCTAN	1712.71	IR:3	4
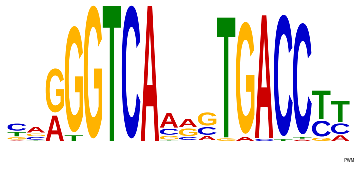	NTAGGTCNNNNNGACCTN	576.23	IR:3	5
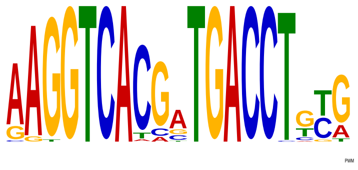	NNGGTCACGNNGACCTGNN	23135.75	IR:3	6
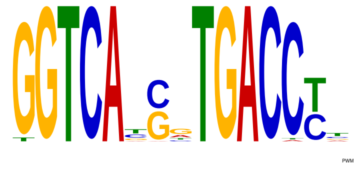	GGTCATNNNGACCTA	1107.87	IR:3	7
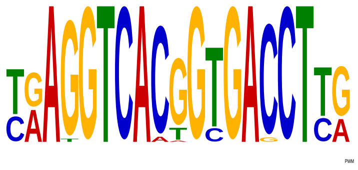	NNNGGTCACGNCGACCNNN	1266.64	IR:3	8

100 ESR1-Yin2017-Rnd2 (ERR1003004)

PWM	Seed Sequence	Seed Seq Enrichment	Repeat	Rank
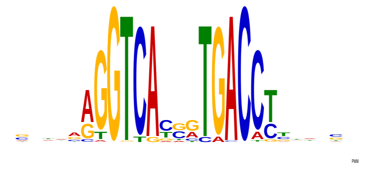	NNNNNNAGGTCANNNTGACCTNNNNNN	537.09	IR:3	1
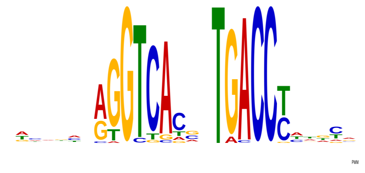	NNNNNNTAGGTCACNNTGACCNNNNNN	321.85	IR:3	2
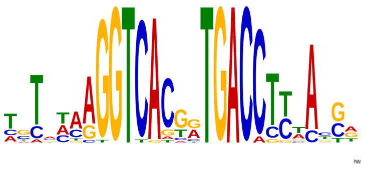	NNNNNNAGGTCACNATGACCTNNNNNN	1819.99	IR:3	3
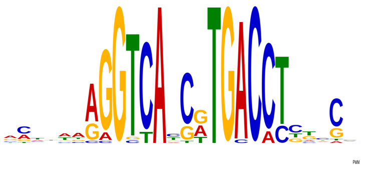	NNNNNNAGGTCAGCNTGACCNNNNNN	196.66	IR:3	4
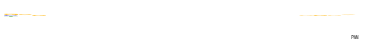	NNNNNNGGGGNNNNNGGGGNNNNNN	2.80	M	5
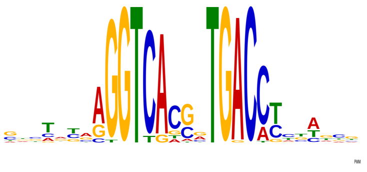	NNNNNNCAGGTCANNNTGACCTNNNNNN	411.73	IR:3	6
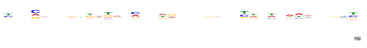	NGACANNNNNNNNNNNNNNNNNNNNNNNNNNNNNNATGCN	1.81	M	7
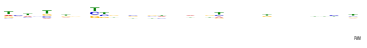	NCTCTNNNNNNNNNNNNNNNNNNNNNNNNNNNNTTCTN	1.92	M	8
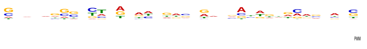	NNATAGNNNNNNNNNNNNNNNNNNNNNNNNNNNAGGANN	1.69	M	9

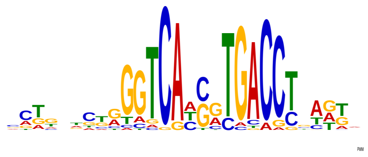	NNNNNNCTGGGTNNNNNTGACCNNNNNN	16.54	IR:3	10
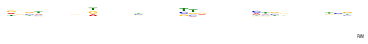	GGGTNNNNNNNNNNNNNNNNNNNNNNNNNNNNNNNNTCGC	1.86	M	11
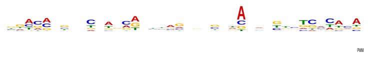	NGACANNNNNNNNNNNNNNNNNNNNNNNNNNNNNNACACN	2.36	M	12
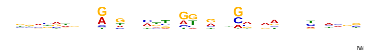	NNGACANNNNNNNNNNNNNNNNNNNNNNNNNNNNTGACNN	1.88	IR:27	13
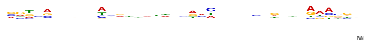	NGTCANNNNNNNNNNNNNNNNNNNNNNNNNNNNNNAACGN	2.27	M	14
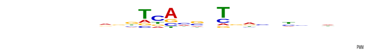	NNNNNNCAGGNNNNNNGACCNNNNNN	1.90	IR:3	15
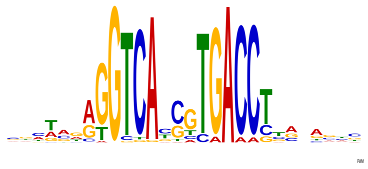	NNNNNNAGGTCANNNNNGACCTGNNNNNN	112.80	IR:3	16
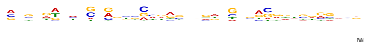	NAGGGNNNNNNNNNNNNNNNNNNNNNNNNNNNNNNGGACN	2.11	DR:27	17
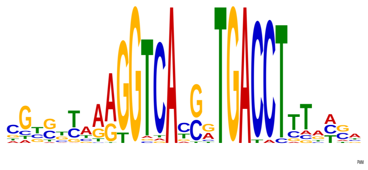	NNNNNNAAAGGTCNNNNNGACCTNNNNNN	132.10	IR:3	18

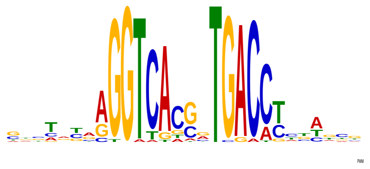	NNNNNNCAGGTCANNNNGACCTNNNNNN	103.86	IR:3	19
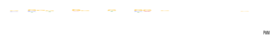	NNNNNGGGGNNNNNNNNNNNNNNNNNNNNNNGGGGNNNNN	1.83	M	20

101 ESR1-Yin2017-Rnd2 (ERR1003005)

PWM	Seed Sequence	Seed Seq Enrichment	Repeat	Rank
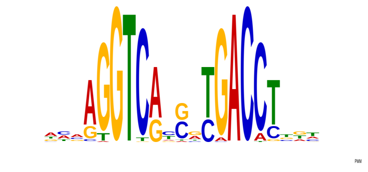	NNNNNNAGGTCANNNTGACCTNNNNNN	367.94	IR:3	1
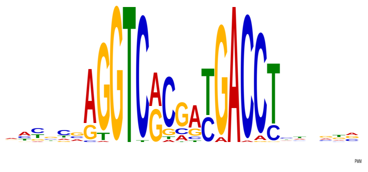	NNNNNNAGGTCGCGNNGACCTNNNNNN	446.12	IR:3	2
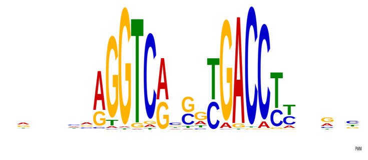	NNNNNNGAGGTCNNNNNGACCTNNNNNN	56.94	IR:3	3
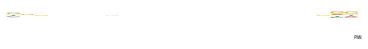	NNNNNNGGGGNNNNNGGGGNNNNNN	4.92	M	4
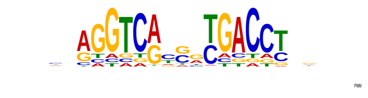	NNNNNNGGTCNNNNNGACCNNNNNN	8.94	IR:3	5
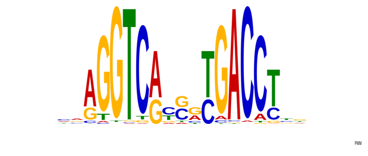	NNNNNNAGGTCNNNNNGACCTNNNNNN	63.24	IR:3	6
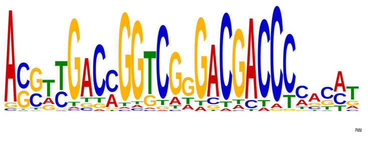	NNNNNNACCGGTCNNNNNGACCNNNNNN	32.60	IR:3	7
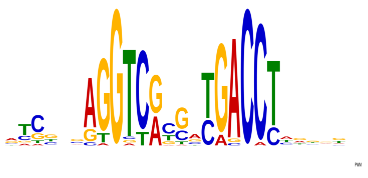	NNNNNNAGGTCGCNNCGACCTNNNNNN	255.00	IR:3	8
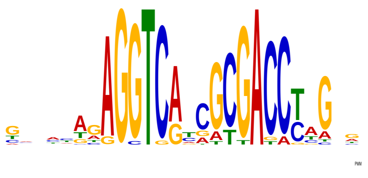	NNNNNNGAGGTCANCGCGACNNNNNN	170.36	IR:3	9

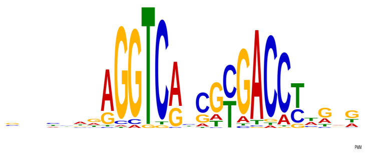	NNNNNNGAGGTCNNNGCGACNNNNNN	29.29	IR:3	10
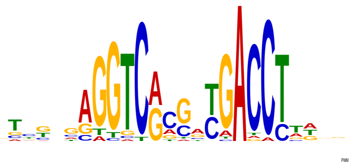	NNNNNNAGGTCGCGNCGACNNNNNN	52.04	IR:3	11
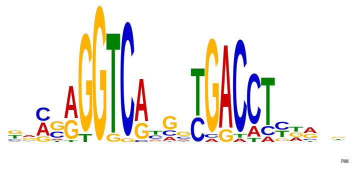	NNNNNNGGTCAANGTGACCNNNNNN	44.51	IR:3	12
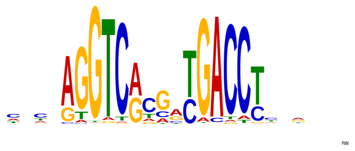	NNNNNNGGTCGCNNNGACCTNNNNNN	63.61	IR:3	13
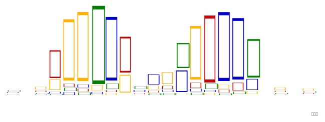	NNNNNNAGGTCNNNGCGACNNNNNN	20.19	IR:3	14
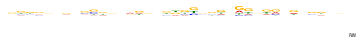	AGGGNNNNNNNNNNNNNNNNNNNNNNNNNNNNNNNGTGG	2.43	M	15
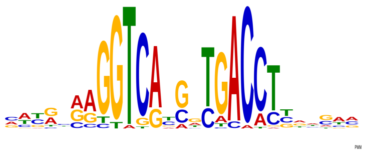	NNNNNNAGGTCAANNNGACCTNNNNNN	72.79	IR:3	16
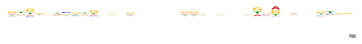	CGTGNNNNNNNNNNNNNNNNNNNNNNNNNNNNNNNGTGG	2.28	M	17
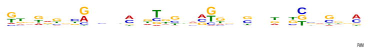	NTGGANNNNNNNNNNNNNNNNNNNNNNNNNNNNNAGGGN	2.27	M	18

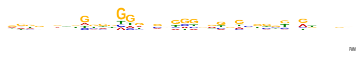	*CGGGNNNNNNNNNNNNNNNNNNNNNNNNNNNNNNNGGGG*	2.87	M	19
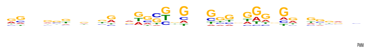	*NNNCGGGNNNNNNNNNNNNNNNNNNNNNNNNNAGGGNNN*	2.76	M	20

102 ESR1-Yin2017-Rnd3 (ERR1003006)

PWM	Seed Sequence	Seed Seq Enrichment	Repeat	Rank
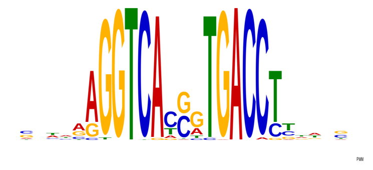	NNNNNNAGGTCANNNTGACCTNNNNNN	3168.96	IR:3	1
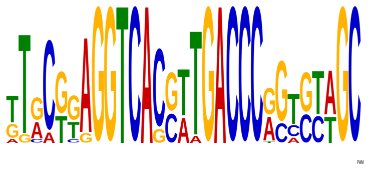	NNNNNNAGGTCACGNTGACCCAGNNNNNN	11194.02	IR:3	2
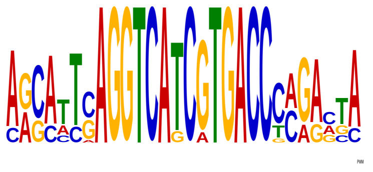	NNNNNNCAGGTCATCGTGACCCNNNNNN	16317.64	IR:3	3
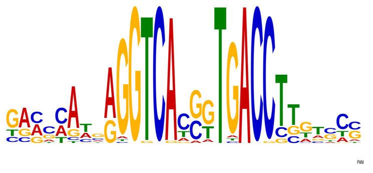	NNNNNNTTAGGTCANNNTGACCTNNNNNN	1914.29	IR:3	4
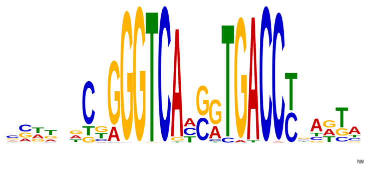	NNNNNNCTGGGTCANNNTGACCNNNNNN	1098.56	IR:3	5
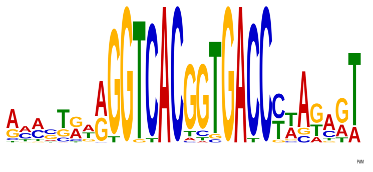	NNNNNNTAGGTCACNGTGACCCNNNNNN	4220.09	IR:3	6
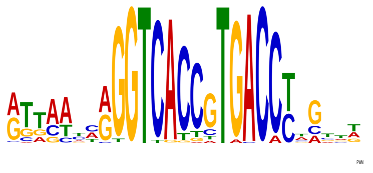	NNNNNNCGGGTCANCGTGACCNNNNNN	2020.30	IR:3	7
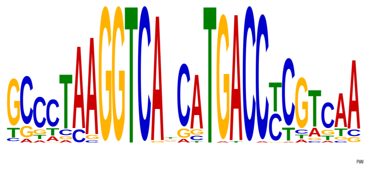	NNNNNNAGGTCAGCNTGACCCNNNNNN	1921.85	IR:3	8
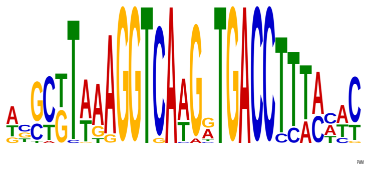	NNNNNNAAAGGTCANNCTGACCTNNNNNN	3958.58	IR:3	9

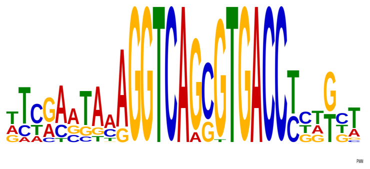	NNNNNNGAAAGGTCNNNGTGACCNNNNNN	1720.16	IR:3	10
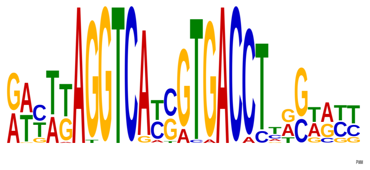	NNNNNNGGTCAACGNGACCTTNNNNNN	1087.65	IR:3	11
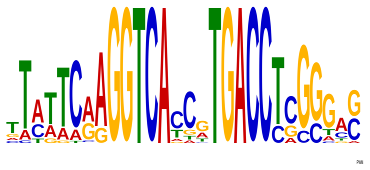	NNNNNNGAGGTCAGNGTGACCTNNNNNN	2388.81	IR:3	12
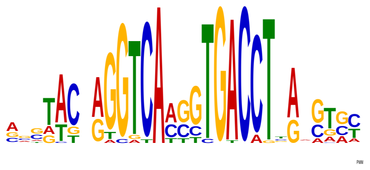	NNNNNNTGGGTCANNNNGACCTTNNNNNN	389.68	IR:3	13
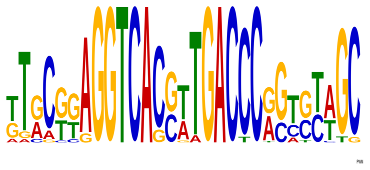	NNNNNNAGGTCACGNNGACCCAGNNNNNN	3406.37	IR:3	14
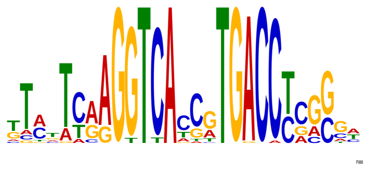	NNNNNNGAGGTCAGNGTGACCNNNNNN	938.65	IR:3	15
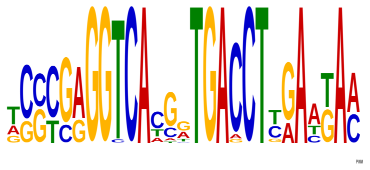	NNNNNNGGTCACGCNGACCTCNNNNNN	672.51	IR:3	16
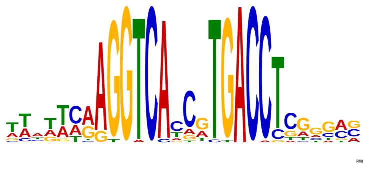	NNNNNNGAGGTCAGNNNGACCTNNNNNN	416.32	IR:3	17
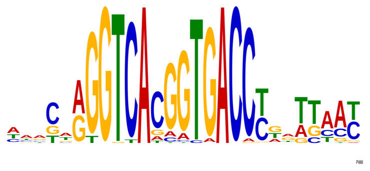	NNNNNNGGTCACGNNGACCCGNNNNNN	593.83	IR:3	18

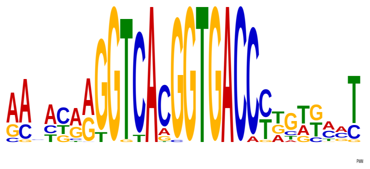	$NNNNNN\overrightarrow{AGGTCA}CGN\overleftarrow{NGACCC}GNNNNNN$	1305.40	IR:3	19
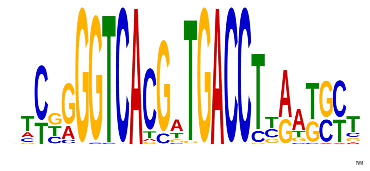	$NNNN\overrightarrow{NNGTCA}CGA\overleftarrow{NGACCT}GNNNNNN$	516.78	IR:3	20

103 ESR1-Yin2017-Rnd3 (ERR1003007)

PWM	Seed Sequence	Seed Seq Enrichment	Repeat	Rank
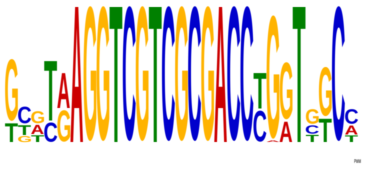	NNNNNAGGTCGTCNCGACCCGGNNNNN	25001.82	IR:3	1
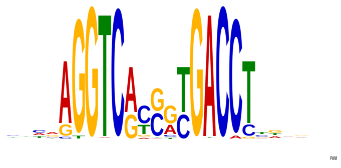	NNNNNNAGGTCNNNNNGACCTNNNNNN	868.56	IR:3	2
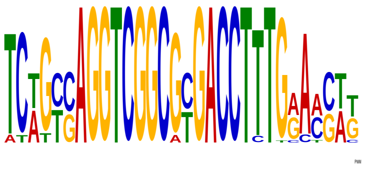	NNNNNNAGGTCGGCNNGACCTTTGNNNNNN	24168.90	IR:3	3
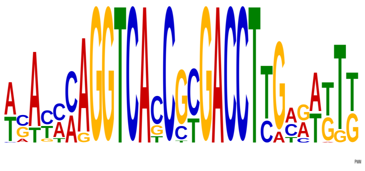	NNNNNNAGGTCAGCNCGACCTTGNNNNNN	15116.11	IR:3	4
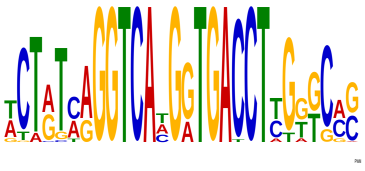	NNNNNNAGGTCAAGGTGACCTCNNNNNN	18053.22	IR:3	5
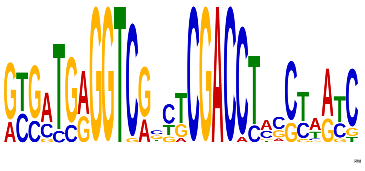	NNNNNNAGGTCACNNNGACCCAGNNNNNN	1370.21	IR:3	6
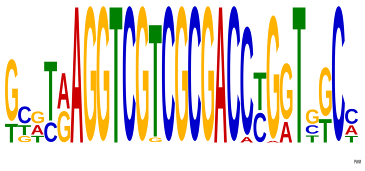	NNNNNAGGTCGTCNNGACCCGGNNNNN	9493.82	IR:3	7
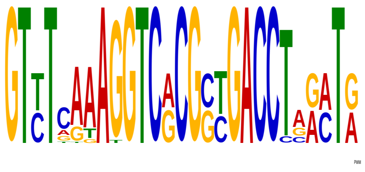	NNNNCAAAGGTCNNNNCGACCTCNNNN	3946.88	IR:3	8
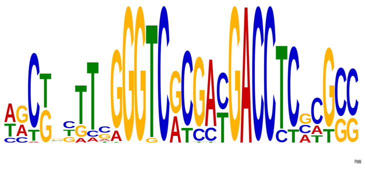	NNNNNNTTCGGGTCNNNNNGACCTNNNNNN	933.45	IR:3	9

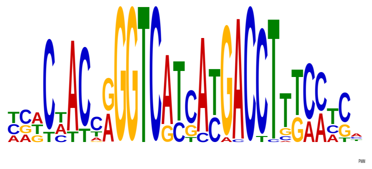	$NNNNNNCC\overrightarrow{GGGTCN}NNNN\overleftarrow{NGACCT}TNNNNNN$	794.13	IR:3	10
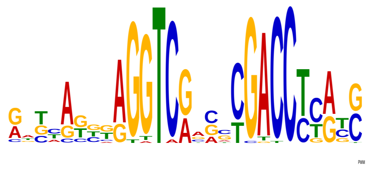	$NNNNNNCT\overrightarrow{GGGTCN}NNG\overleftarrow{TGACN}NNNNN$	128.58	IR:3	11
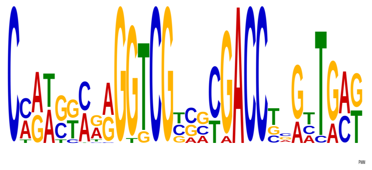	$NNNNNNCA\overrightarrow{AGGTCN}NNNN\overleftarrow{NGACCC}GNNNNNN$	360.79	IR:3	12
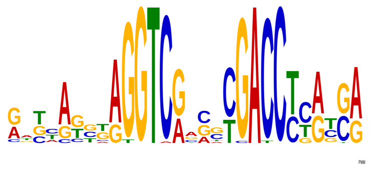	$NNNNNNCT\overrightarrow{GGGTCN}NNG\overleftarrow{TGACN}NNNNN$	564.55	IR:3	13
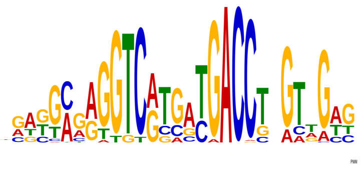	$NNNNNNA\overrightarrow{AGGTCN}NNNN\overleftarrow{NGACCC}GNNNNNN$	164.64	IR:3	14

104 ESR1-Yin2017-Rnd4 (ERR1003008)

PWM	Seed Sequence	Seed Seq Enrichment	Repeat	Rank
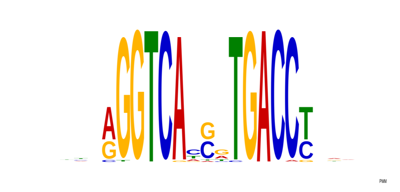	NNNNNNGGTCANNNTGACCNNNNNN	736.76	IR:3	1
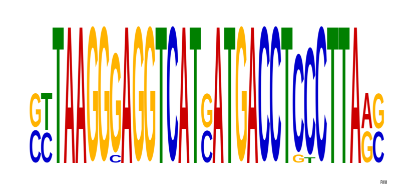	NNNNNNGGAGGTCANNNNGACCTCCNNNNNN	1103.31	IR:3	2
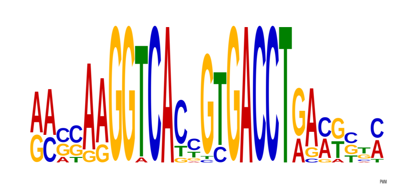	NNNNNNGGTCACTGNGACCTGNNNNNN	984.56	IR:3	3
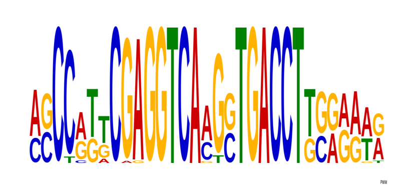	NNNNNNGCGAGGTCNNNNNGACCTGNNNNNN	1144.69	IR:3	4
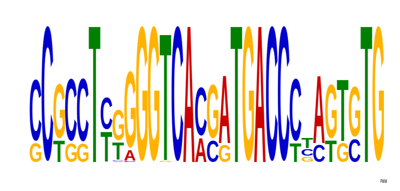	NNNNNNCTGGGTCNNNNNGACCTGNNNNNN	819.90	IR:3	5
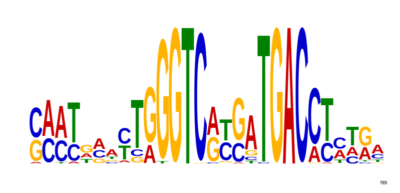	NNNNNNCCTGGGTCNNNNTGACNNNNNN	233.81	M	6
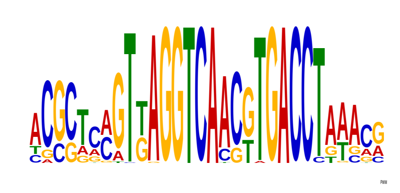	NNNNNNAGTTAGGTNNNNNTGACCNNNNNN	1024.58	IR:3	7
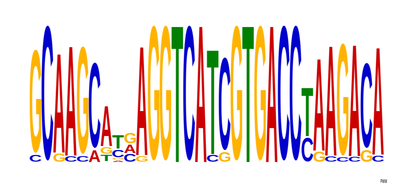	NNNNNNACCAGGTNNNNNTGACCCNNNNNN	463.08	IR:3	8
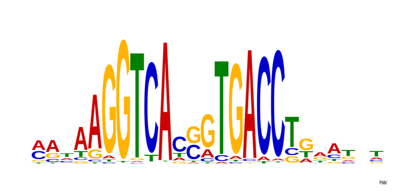	NNNNNNGTCACTNNGACCTNNNNNN	74.27	IR:3	9

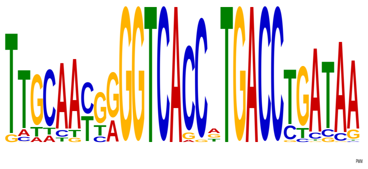	$NNNNNNCG\overrightarrow{GGGTC}NNNT\overleftarrow{TGACC}NNNNNN$	493.75	IR:3	10

105 ESR1-Yin2017-Rnd4 (ERR1003009)

PWM	Seed Sequence	Seed Seq Enrichment	Repeat	Rank
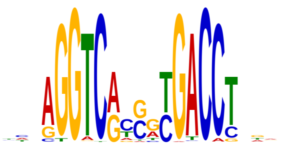	NNNNNNAGGTCNNNNNGACCTNNNNNN	501.28	IR:3	1
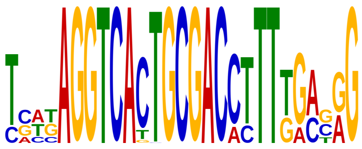	NNNNNNGTCACTGCNACCTTTNNNNNN	1275.74	IR:3	2
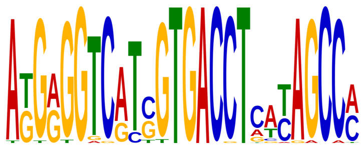	NNNNNNTCGTCGNNACCTAANNNNNN	89.39	IR:0	3
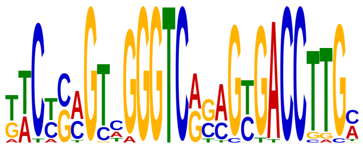	NNNNNNGTCGGGTCNNNGCGANNNNNN	49.69	M	4
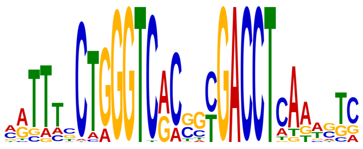	NNNNNNCTGGGTCNNNNNGACCTCNNNNNN	1972.47	IR:3	5
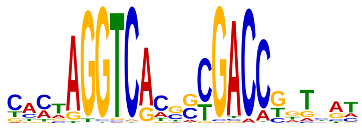	NNNNNNGTCACTNNGACCNNNNNN	57.35	IR:3	6
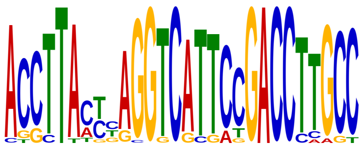	NNNNNNTCCGGGTCNNNNCGACNNNNNN	90.65	IR:3	7
	NNNNNNGGGGNNNNNGGGGNNNNNN	4.62	M	8
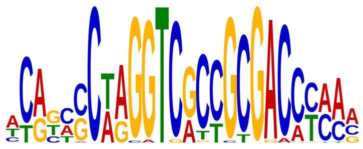	NNNNNNCTGGGTNNTCGCGANNNNNN	73.22	M	9

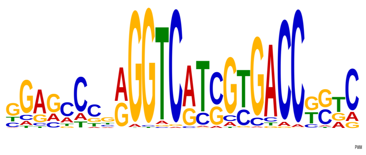	NNNNNNCGGGGTCNNNGTGANNNNNN	64.26	DR:0	10
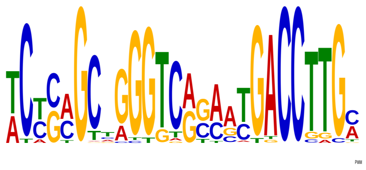	NNNNNNTCGGGTCNNNGCGANNNNNN	17.66	M	11
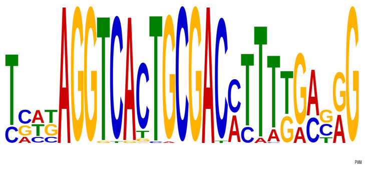	NNNNNNGTCACTGNNNCCTTTNNNNNN	181.96	IR:3	12
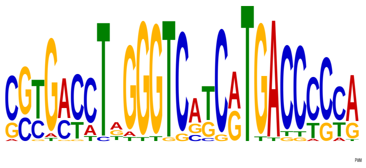	NNNNNNATTGGGTCNNNGTGANNNNNN	59.29	DR:0	13
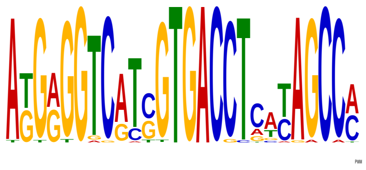	NNNNNNTCGTCGNNNCCTAANNNNNN	32.11	IR:0	14
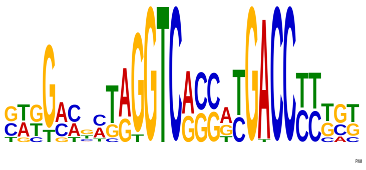	NNNNNNTCTGGGTCNNNNTGACNNNNNN	130.35	IR:3	15
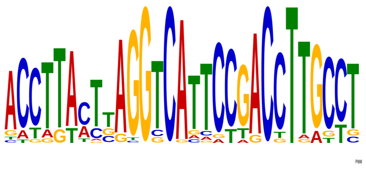	NNNNNNTCCGGGTNNNNNNGACCNNNNNN	25.01	IR:3	16
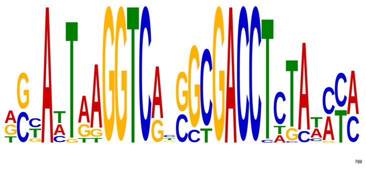	NNNNNNAAGGTCGANNNNACCTCNNNNNN	165.22	IR:3	17
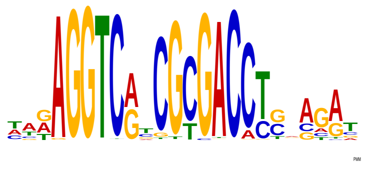	NNNNNNTCGTCGCNACCTNNNNNN	138.38	IR:0	18

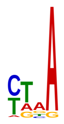	$NNNNNTAA\overrightarrow{GGGTC}NNNNN\overleftarrow{NACCT}NNNNN$	45.55	IR:3	19

106 ESR1-Yin2017-Rnd2 (ERR1003012)

PWM	Seed Sequence	Seed Seq Enrichment	Repeat	Rank
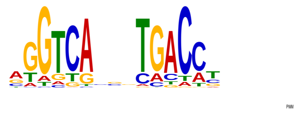	NNNNNNGGTCANNNTGACCNNNNNN	56.41	IR:3	1
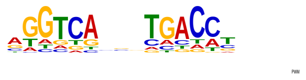	NNNNNNGTCANNNTGACNNNNNN	10.90	IR:3	2
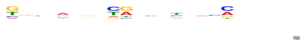	NNNNNNGTCANNNNNNNNTGACNNNNNN	2.23	IR:8	3
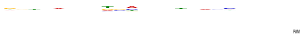	NNNNNNGTCANNNNNNNNNTGACNNNNNN	2.02	IR:9	4
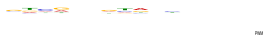	NNNNNNGTCGNNGTATNNNNNN	2.24	M	5
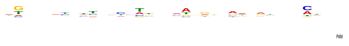	NNNNGTCANNNNNNNNNNNNNNNNNNNNNNTGACNNNN	1.67	IR:22	6
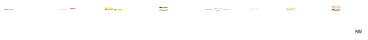	CGTGNNNNNNNNNNNNNNNNNNNNNNNNNNNNNCGTG	1.69	M	7
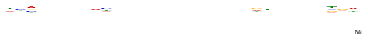	NNNNNNTGACNNNNNNNNNNNNNNGTCANNNNNN	2.00	ER:9	8
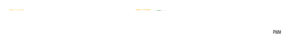	NNNNNNGTCGNNCGTANNNNNN	1.69	M	9

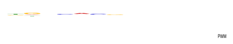	NNNNNNAATGNCACGNNNNNNN	2.03	M	10
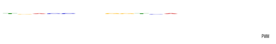	NNNNNNTGACNNNNGTCANNNNNNN	2.17	ER:0	11
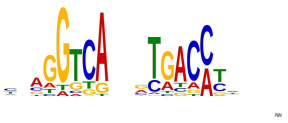	NNNNNNCAAGGTCNNNNTGACNNNNNNN	30.58	IR:3	12
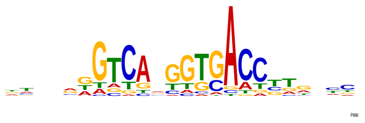	NNNNNNNGTCANGGTCACCNNNNNNN	19.26	DR:0	13
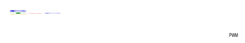	NNNNNNACACGACGNNNNNNN	1.83	M	14
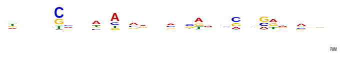	NNATGTNNNNNNNNNNNNNNNNNNNNNNNNNNNNATAGNN	1.68	M	15
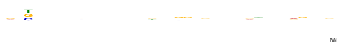	TGGGNNNNNNNNNNNNNNNNNNNNNNNNNNNNNNNNNNNGTGG	1.69	M	16
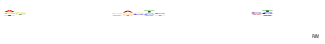	NNNNNNGTCANNNNNNNNNNNNNNNTGACNNNNNN	1.59	IR:13	17
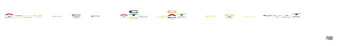	NNNNNNGTCANNNNNNNNNNNNNNNNNNNTGACNNNNNN	1.81	IR:17	18

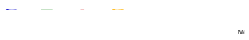	$NNNN\overrightarrow{NNGTCA}NNNNNNNNNN\overleftarrow{TGACNN}NNNN$	1.68	IR:10	19
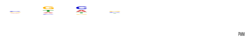	$NNNN\overrightarrow{NNGTCA}NNNNNNNNNNNN\overleftarrow{TGACNN}NNNN$	1.60	IR:12	20

107 ESR1-Yin2017-Rnd2 (ERR1003013)

PWM	Seed Sequence	Seed Seq Enrichment	Repeat	Rank
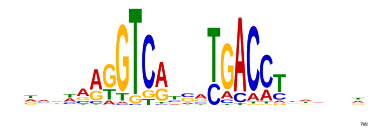	NNNNNNAGGTCANNNNGACCTNNNNNN	42.94	IR:3	1
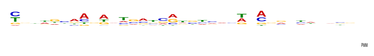	NNNTGCANNNNNNNNNNNNNNNNNNNNNNTATANNN	1.99	M	2
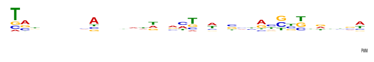	NNTTCANNNNNNNNNNNNNNNNNNNNNNNNNTGTTNN	1.53	M	3
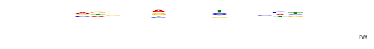	NNNNNNGTCGNNNCGACNNNNNN	1.78	IR:3	4
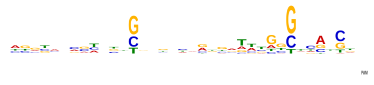	NNNTATCNNNNNNNNNNNNNNNNNNNNNNNTCATNNN	1.90	M	5
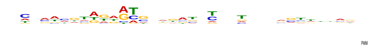	NNNAACGNNNNNNNNNNNNNNNNNNNNNNNTTTTNNN	1.87	M	6
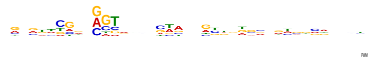	NNNTTCGNNNNNNNNNNNNNNNNNNNNNNNNNNNGCACNNN	2.25	M	7
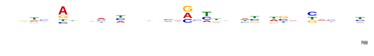	NGTCGNNNNNNNNNNNNNNNNNNNNNNNNNNNNNACCAN	1.71	IR:25	8
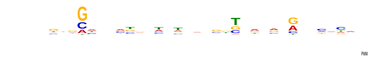	NAAGGNNNNNNNNNNNNNNNNNNNNNNNNNNNNCACAN	1.75	M	9

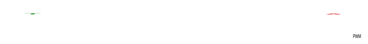	NNNNNNTGTCNNGACANNNNNN	1.59	IR:0	10
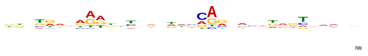	NNNTGAANNNNNNNNNNNNNNNNNNNNNNGTAANNN	1.81	ER:17	11
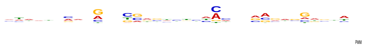	NNNNTCCANNNNNNNNNNNNNNNNNNNNAGGANNNN	1.65	M	12
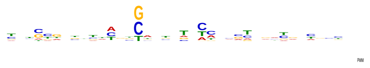	NGTCCNNNNNNNNNNNNNNNNNNNNNNNNNNNNNGCCAN	1.68	DR:27	13
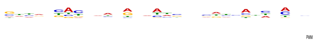	NNNNNGTTANNNNNNNNNNNNNNNNNNTCATNNNNN	1.84	DR:15	14
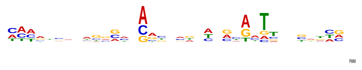	NNNNATCANNNNNNNNNNNNNNNNNNNNNNTACANNNNN	1.90	M	15
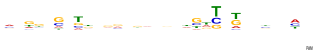	NNGACANNNNNNNNNNNNNNNNNNNNNNNACGANN	1.45	M	16
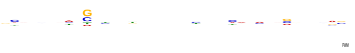	TGCANNNNNNNNNNNNNNNNNNNNNNNNNNNNNNNNCCAC	1.63	M	17
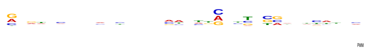	NNGTACNNNNNNNNNNNNNNNNNNNNNNNNTCATNN	1.90	M	18

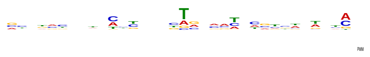	$NNNNNNTTTCNNNNNNNNNNNNN\overrightarrow{NNGTCT}NNNNNN$	1.64	M	19
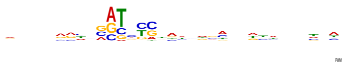	*NNNTACGNNNNNNNNNNNNNNNNNNNNNTCTTNNN*	1.39	M	20

108 ESR1-Yin2017-Rnd3 (ERR1003014)

PWM	Seed Sequence	Seed Seq Enrichment	Repeat	Rank
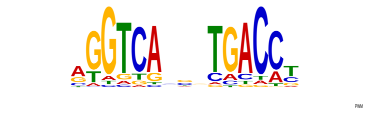	NNNNNNGTCANNNTGACNNNNNN	25.26	IR:3	1
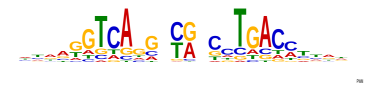	NNNNNNGTCAGNNNNNNCTGACNNNNNN	15.36	IR:8	2
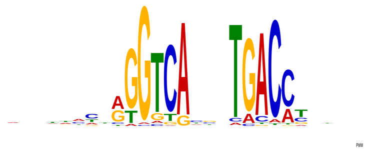	NNNNNNCTGGGTCANNNTGACNNNNNN	122.58	IR:3	3
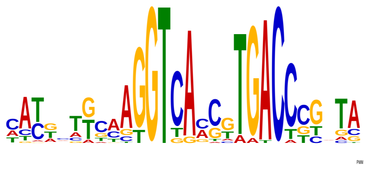	NNNNNNGCAAGGTCNNCGTGACNNNNNN	278.41	IR:3	4
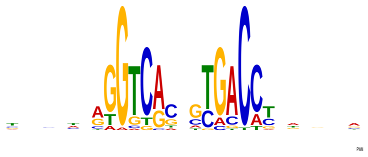	NNNNNNCAGGTCNNNNNGACCTGNNNNNN	91.83	IR:3	5
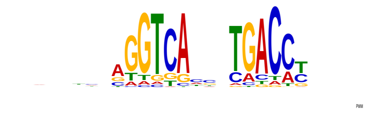	NNNNNNCAAGGTNNNNNTGACNNNNNN	26.82	IR:3	6
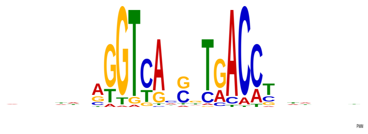	NNNNNNAAGGTCNNNNNGACCTTNNNNNN	123.39	IR:3	7
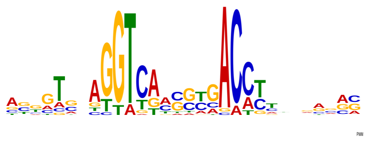	NNNNNNAAGGTCNNNNNNACCTTANNNNNN	60.65	IR:3	8
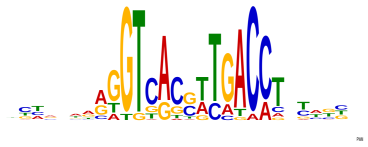	NNNNNTAAGGTCNNNNNGACCTTNNNNN	166.63	IR:3	9

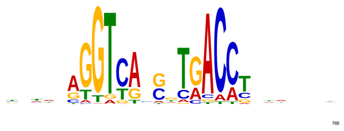	NNNNNNAAGGTCNNNNNNACCTTNNNNNN	51.87	IR:3	10
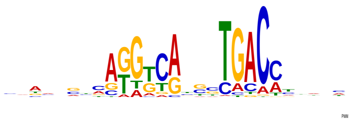	NNNNNNGTCAGGTCNNNNTGACNNNNNN	74.12	IR:3	11
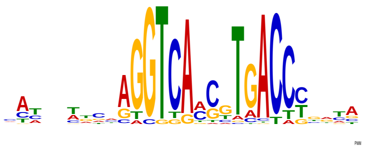	NNNNNNGCAAGGTCNNGTGACNNNNNN	127.01	IR:3	12
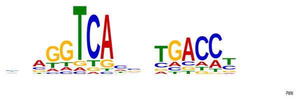	NNNNNNAAGGNNNNNNTGACNNNNNN	6.85	IR:3	13
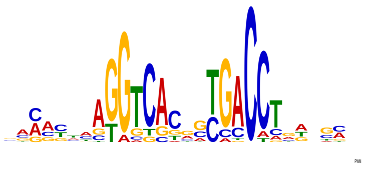	NNNNNTCAGGTCNNNNNGACCTGNNNNN	152.50	IR:3	14
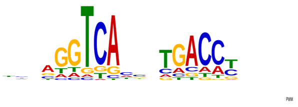	NNNNNNCAAGGNNNNNNTGACNNNNNN	8.61	IR:3	15
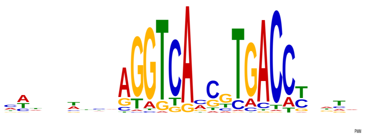	NNNNNNGCAAGGTCNNNNTGACNNNNNN	69.59	IR:3	16
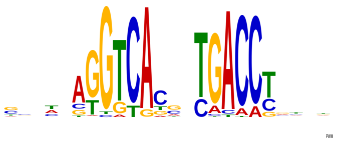	NNNNNNAAGGTCNNGCTGACNNNNNN	74.04	IR:3	17
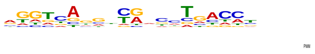	NNNNNNGTCANNNNNNNNNTGACNNNNNN	4.32	IR:8	18

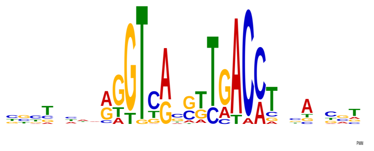	NNNNNNTAAGGTCNNNNNNACCTTNNNNNN	53.19	IR:3	19
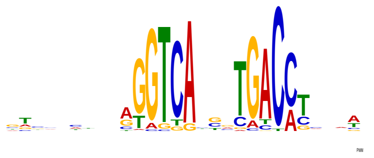	NNNNNNACAAGGTCNNNNTGACNNNNNN	67.17	IR:3	20

109 ESR1-Yin2017-Rnd3 (ERR1003015)

PWM	Seed Sequence	Seed Seq Enrichment	Repeat	Rank
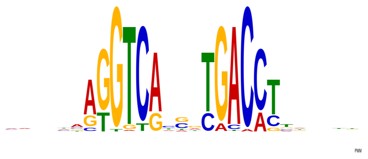	NNNNNNAGGTCANNNTGACCTNNNNNN	465.07	IR:3	1
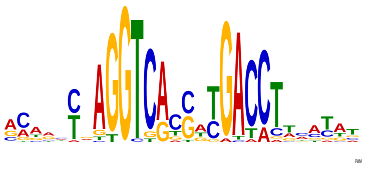	NNNNNNGAGGTCACNNNGACCTNNNNNN	381.72	IR:3	2
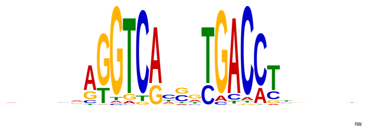	NNNNNNAGGTCNNNNNGACCTNNNNNN	73.03	IR:3	3
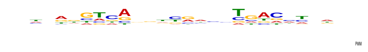	NNNNNNGTCANNNNNNNNNTGACNNNNNN	3.52	IR:8	4
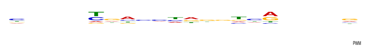	NNNNNNGACCNNGGTCNNNNNN	2.33	ER:0	5
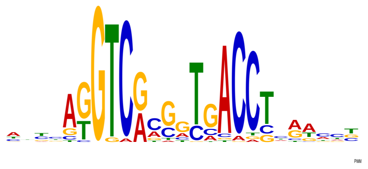	NNNNNNGTCGCGGTNACCTNNNNNN	86.72	IR:3	6
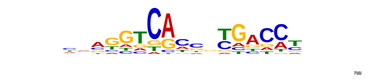	NNNNNNAGGTNNNNNCGACNNNNNN	4.80	IR:3	7
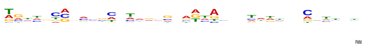	NNTTGCNNNNNNNNNNNNNNNNNNNNNNNNNCATTNN	2.13	M	8
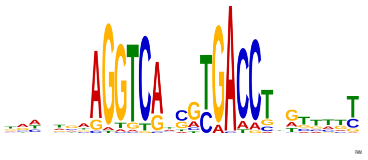	NNNNNNAAGGTCNNNNNGACCTCNNNNNN	129.48	IR:3	9

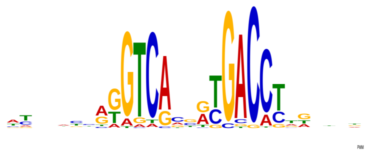	*NNNNNNCGGGTCNNNNNGACCTNNNNNN*	41.91	IR:3	10
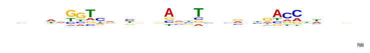	*NNNNNNGGTCNNNNNNNNNNNNNNNNGACCNNNNNN*	2.76	IR:14	11
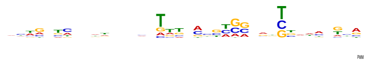	*NNNGGTCNNNNNNNNNNNNNNNNNNNNNNNNNNAAACNNN*	1.96	M	12
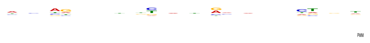	*NNNNNNNGTCANNNNNNNNNNNNNNTGACNNNNNNN*	1.90	IR:13	13
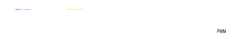	*NNNNNNNGTCGCGACNNNNNNN*	1.97	IR:0	14
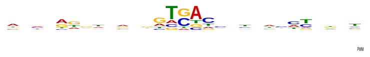	*NNNNNNNGTCANNNNNNNNNNTGACNNNNNNN*	2.36	IR:9	15
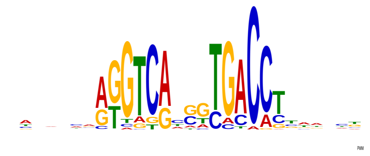	*NNNNNNTAGGTCNNNNNGACCTNNNNNN*	53.67	IR:3	16
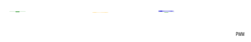	*NNNNNNNGTCGNCGTANNNNNNN*	1.98	M	17
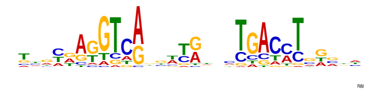	*NNNNNNAGGTCNNNNNNNNNTGACCNNNNNNN*	9.19	IR:8	18

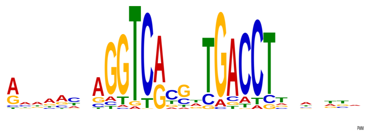	NNNNNNGAGGTCNNNNNNACCTTNNNNNN	41.19	IR:3	19
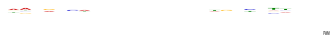	NNNNNNGTCANNNNNNNNNNTGACNNNNNN	2.08	IR:10	20

110 ESR1-Yin2017-Rnd4 (ERR1003016)

PWM	Seed Sequence	Seed Seq Enrichment	Repeat	Rank
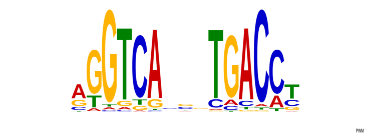	NNNNNNGTCANNNTGACNNNNNN	52.04	IR:3	1
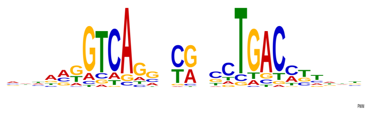	NNNNNNGTCAGNNNNNNCTGACNNNNNN	40.91	IR:8	2
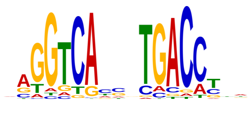	NNNNNNCAGGTNNNNNTGACNNNNNN	36.21	IR:3	3
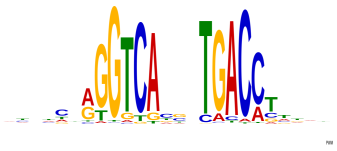	NNNNNNCTGGGTCNNNNTGACNNNNNN	109.55	IR:3	4
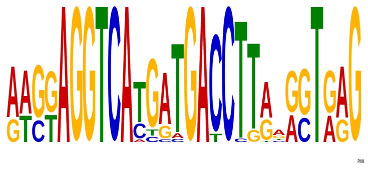	NNNNNNGTCACGATNNCCTTAANNNNNN	547.47	IR:3	5
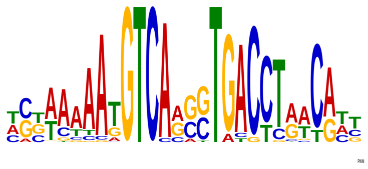	NNNNNAAATGTCANNNNGACCTGNNNNN	797.31	IR:3	6
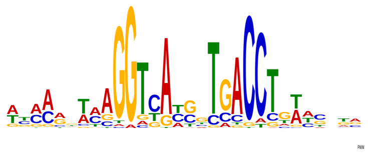	NNNNNNTCAGGTNNNNNNNACCTGNNNNNN	66.23	IR:3	7
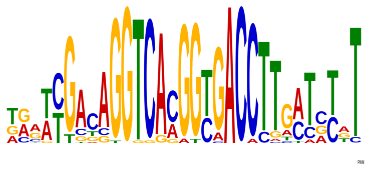	NNNNNNACAGGTCNNNNNGACCTTGNNNNNN	763.12	IR:3	8
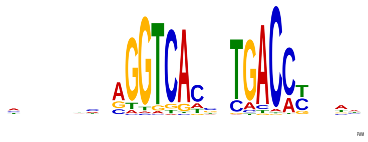	NNNNNNCGAGGTNNNNNTGACNNNNNN	44.05	IR:3	9

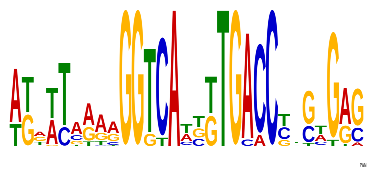	NNNNNAAGAGGTCNNNNNGACCTTNNNNNN	474.10	IR:3	10
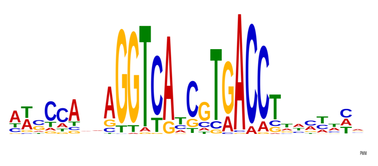	NNNNNNAAAGGTNNNNNNNACCTTNNNNNNN	62.73	IR:3	11
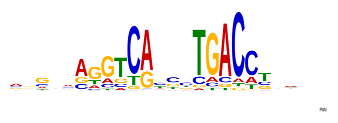	NNNNNNTCAGGNNNNNNTGACNNNNNN	18.06	IR:8	12
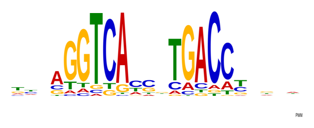	NNNNNNCAAGGNNNNNNTGACNNNNNN	22.30	IR:3	13
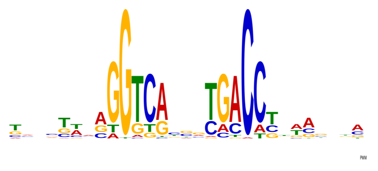	NNNNNNCAGGTCNNNNNGACCTGNNNNNN	158.63	IR:3	14
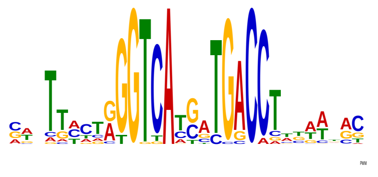	NNNNNNCTGGGTCNNNNNGACCTANNNNNN	378.12	IR:3	15
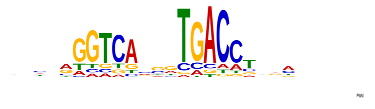	NNNNNNGTCANNNNNNNNCTTGNNNNNN	8.22	M	16
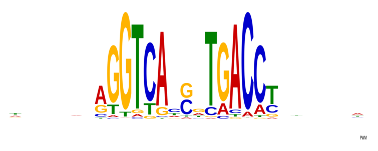	NNNNNNAAGGTNNNNNNNACCTTNNNNNN	51.93	IR:3	17
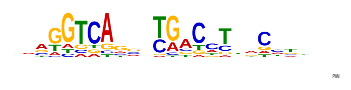	NNNNNNGTCANNNNNNNNCTGANNNNNN	7.83	IR:8	18

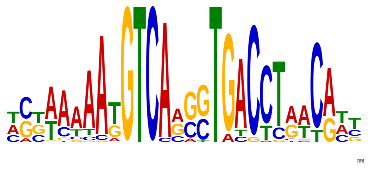	$NNNNNAA\overrightarrow{ATGTCN}NNNN\overleftarrow{NGACCT}GNNNNN$	304.12	IR:3	19
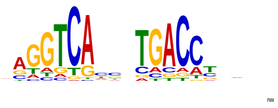	$NNNNNNC\overrightarrow{AGGNNN}NNN\overleftarrow{TGACN}NNNNNN$	12.57	IR:3	20

111 ESR1-Yin2017-Rnd4 (ERR1003017)

PWM	Seed Sequence	Seed Seq Enrichment	Repeat	Rank
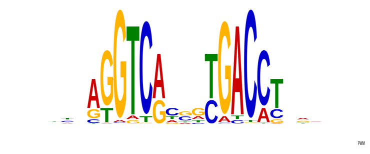	NNNNNNAGGTCNNNNNGACCTNNNNNN	180.56	IR:3	1
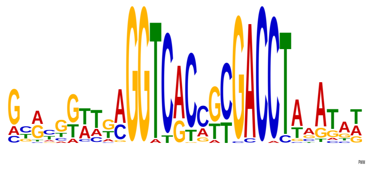	NNNNNNTTGAGGTCNNNNNGACCTNNNNNN	759.43	IR:3	2
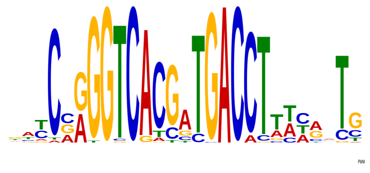	NNNNNNGGTCACGANGACCTTNNNNNN	2093.47	IR:3	3
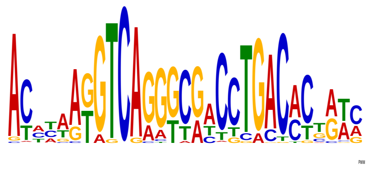	NNNNNNGGTCAGGNNNNCCTGACNNNNNN	487.64	IR:8	4
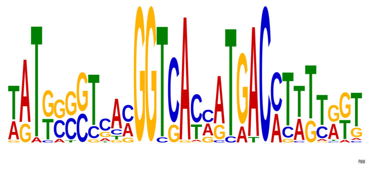	NNNNNNGTCAAGGTNNNNNNGACCTNNNNNN	369.04	M	5
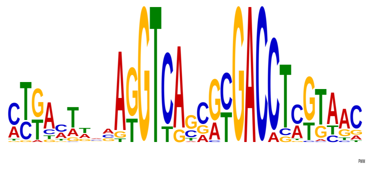	NNNNNNTAAAGGTCNNNNNGACCTNNNNNN	502.46	IR:3	6
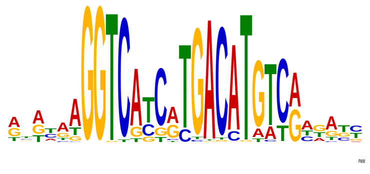	NNNNNNGGTCANNNNGACATGTCNNNNNN	491.65	DR:7	7
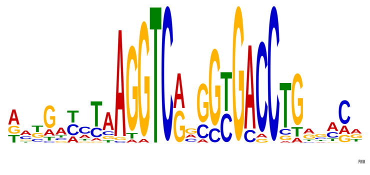	NNNNNNCTAAGGTCNNNNNGACCTNNNNNN	692.43	IR:3	8
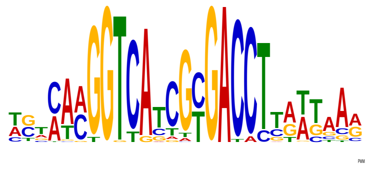	NNNNNNGGTCATCGNNACCTTNNNNNN	550.83	IR:3	9

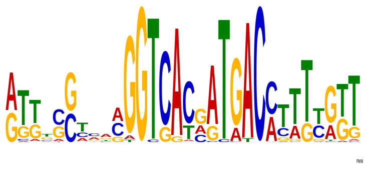	*NNNNNNNTCAAGGTNNNNNNGACCTNNNNNN*	181.10	M	10
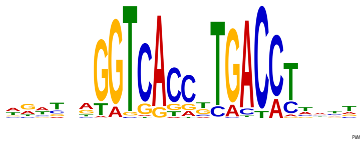	*NNNNNNGGTCACCTTGACNNNNNN*	251.47	IR:3	11
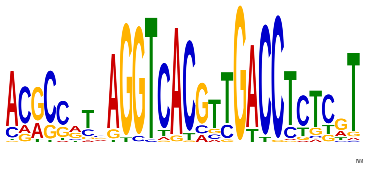	*NNNNNATCAGGTNNNNNNGACCTNNNNN*	178.74	IR:3	12
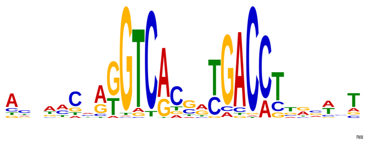	*NNNNNNCGGGTCNNNNNGACCTNNNNNN*	82.54	IR:3	13
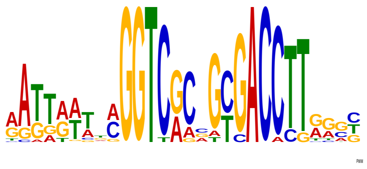	*NNNNNTTGAGGTCNNNNTGACCTNNNNN*	861.55	IR:3	14
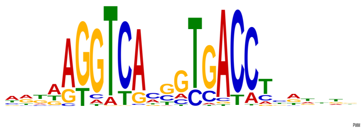	*NNNNNNNGTCAAGNNGACCTNNNNNN*	87.19	IR:3	15
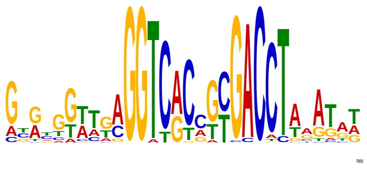	*NNNNNNTTGAGGTCNNNNNNACCTNNNNNN*	225.40	IR:3	16
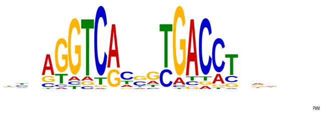	*NNNNNNAGGTNNNNNNNACCTNNNNNN*	16.26	IR:3	17
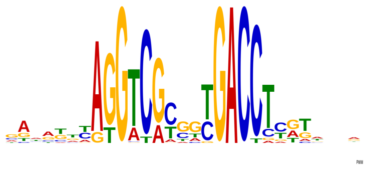	*NNNNNNCAGGTCGNNNNGACCTCNNNNNN*	392.53	IR:3	18

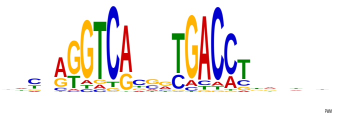	$NNNNNN\overrightarrow{GGGTCN}NNNN\overleftarrow{NACCT}NNNNNN$	22.22	IR:3	19
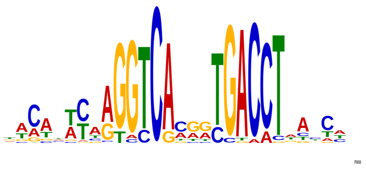	$NNNNNNTA\overrightarrow{GGGTCN}NNN\overleftarrow{NGACCT}NNNNNN$	160.01	IR:3	20

112 THRA:RXRA-Bhimsaria2022-Rnd3 (S13-163)

PWM	Seed Sequence	Seed Seq Enrichment	Repeat	Rank
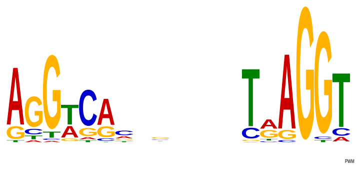	AGGTCNNNNNNNNTAAGGT	242.04	DR:9	1
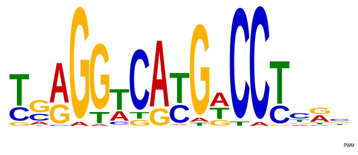	NNAGGTCATGNCCTCNN	243.09	IR:0	2
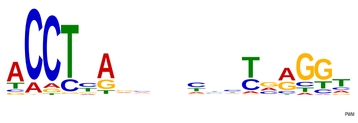	ACCTTANNNNNNNNNAGGT	68.22	ER:11	3
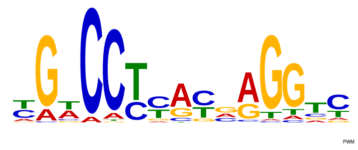	NGTCCTCANNAGGTN	86.13	ER:4	4
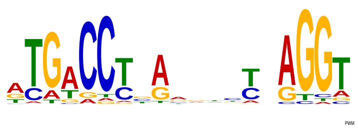	ATGACCNNNNNNNNNAGGT	55.82	ER:8	5
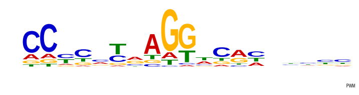	NNNNNNTAAGGTCANNNNNN	13.77	M	6
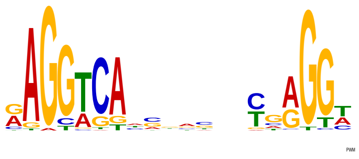	AAGGTCANNNNNNNNNGGGT	107.04	DR:9	7
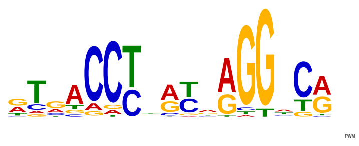	NTGACCNNNNNNGGACAN	24.30	ER:4	8
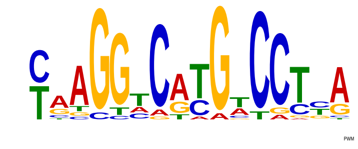	NNAAGGTCNNGTCCTNN	68.57	IR:0	9

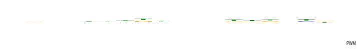	$NNNTTTTNNNTTTTNNNN$	7.07	M	10
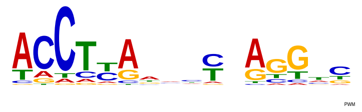	$\overleftarrow{ACCT}TANNNNNN\overrightarrow{NGGTC}$	27.32	ER:7	11
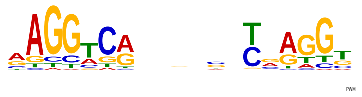	$A\overrightarrow{AGGTCN}NNNNNNNN\overrightarrow{AGGT}$	31.05	DR:8	12
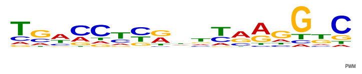	$\overleftarrow{NGACCT}NNNNNN\overrightarrow{AGGTN}$	7.31	ER:6	13
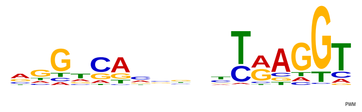	$\overrightarrow{AGGTNN}NNNNNTA\overrightarrow{AGGT}$	29.58	DR:7	14
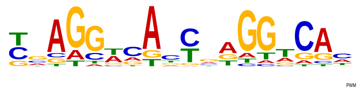	$NN\overrightarrow{AGGTCN}NNN\overrightarrow{AGGTCN}N$	13.54	DR:3	15
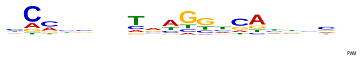	$NNNNNNTTA\overrightarrow{AGGTCN}NNNNN$	6.24	M	16
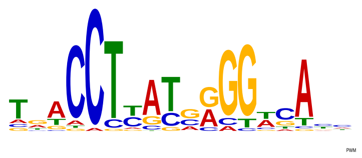	$\overleftarrow{NNACCT}TANN\overrightarrow{NGGTCA}NN$	76.65	ER:4	17
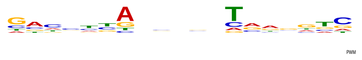	$\overleftarrow{NACCT}NNNNNNNNN\overrightarrow{AGGTN}$	7.30	ER:9	18

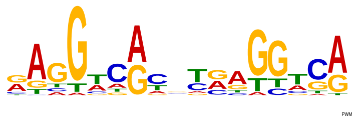	$N\overrightarrow{AGGTCN}NNNN\overrightarrow{AGGTCN}$	17.33	DR:4	19
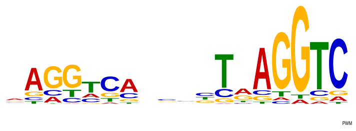	$N\overrightarrow{AGGTNN}NNNNTA\overrightarrow{AGGTN}$	16.73	DR:6	20

113 THRA:RXRA-7-Bhimsaria2022-Rnd2 (S13-124)

PWM	Seed Sequence	Seed Seq Enrichment	Repeat	Rank
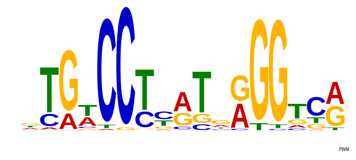	NTTGTCCTNNNNNGGTCN	164.26	ER:4	1
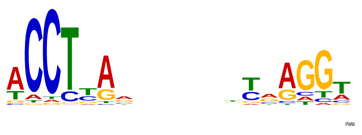	ACCTTANNNNNNNNNAGGT	136.48	ER:11	2
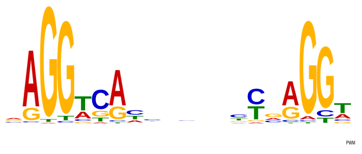	AAGGTCANNNNNNNNNAGGT	272.45	DR:9	3
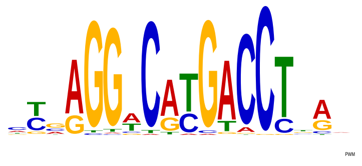	NNNAGGACATGACCTNNN	687.28	IR:0	4
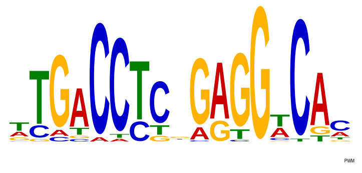	NNGACCTCNGAGGACNN	248.34	ER:3	5
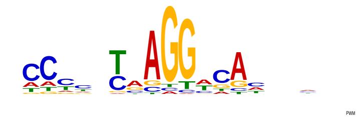	NNNNNNTAAGGTCANNNNNNN	32.54	M	6
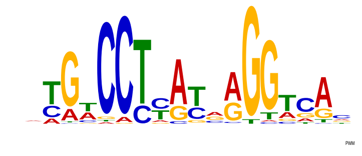	NNNGTCCTTANNAGGTNNN	194.72	ER:4	7
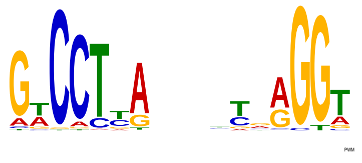	GTCCTTANNNNNNAGGT	192.70	ER:8	8
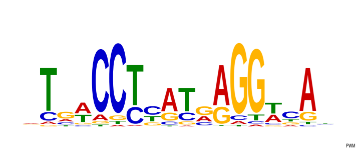	NNNNACCTNNNNAGGTNNNN	20.85	ER:4	9

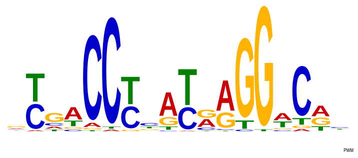	*NNGACCTCNNNAGGACNN*	128.41	ER:4	10
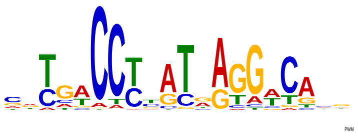	*NNNGACCTNNNNNGGACNNN*	32.99	ER:4	11
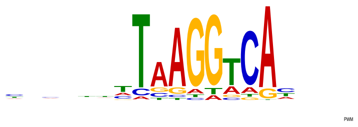	*NNNNNTTTAAGGTCNNNNN*	28.17	M	12
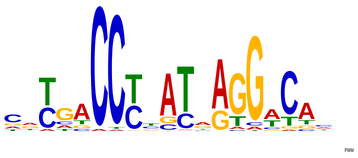	*NNNGACCTNNNNAGGACNNN*	95.83	ER:4	13
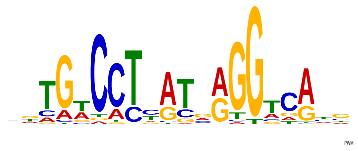	*NNNGTCCTNNNNNGGTCNNN*	45.59	ER:4	14
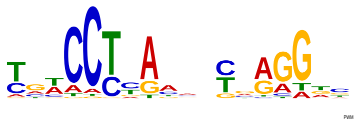	*NGACCTTANNNNNAGGTN*	77.81	ER:7	15
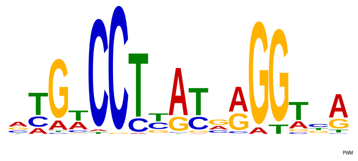	*NNGTCCTTANGAGGTNN*	363.48	ER:4	16
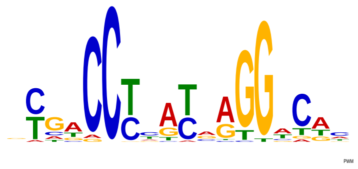	*NNGACCTCANNAGGACNN*	336.13	ER:4	17
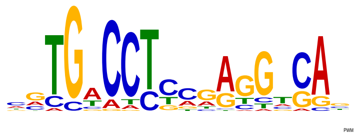	*NNNGACCTCNGGGGTNNN*	50.30	ER:3	18

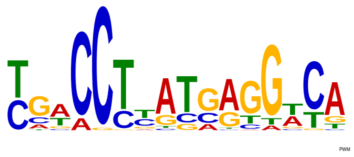	$\overleftarrow{NGACCT}CANN\overrightarrow{GGGACN}$	228.47	ER:4	19
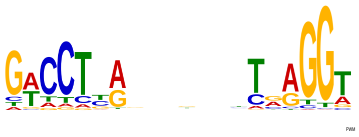	$\overleftarrow{GTCCT}NNNNNNNNNNN\overrightarrow{AGGT}$	57.30	ER:11	20

114 THRA-Bhimsaria2022-Rnd2 (S07-034)

PWM	Seed Sequence	Seed Seq Enrichment	Repeat	Rank
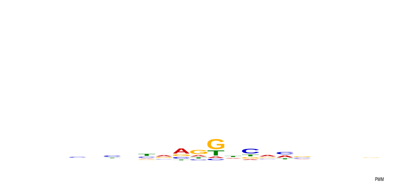	NNNNNNTAAGGTCANNNNNN	2.78	M	1
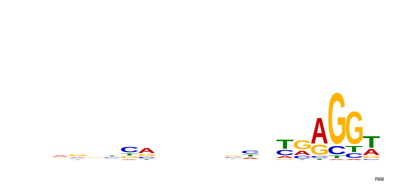	GAGGNNNNNNNNNNNTGAGGT	8.89	DR:9	2
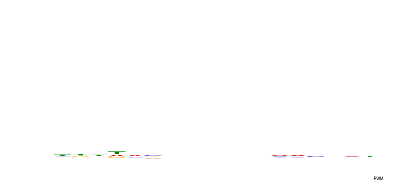	ATTTNNNNNNNNNNNCAAT	1.90	M	3
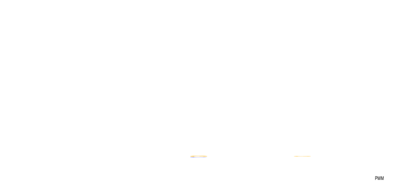	GGGGNNNNNNNNNNNNAGTG	1.78	M	4
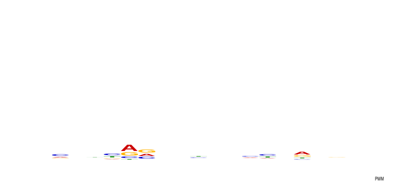	ACCTNNNNNNNNNNNNGGTC	1.85	ER:11	5
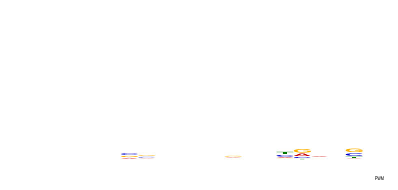	CGTGNNNNNNNNNNNNAGGT	1.84	M	6
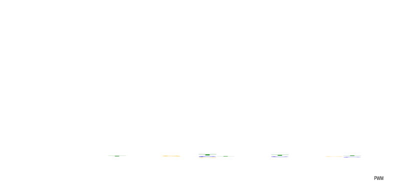	ATATNNNNNNNNNNNTGTT	1.54	M	7
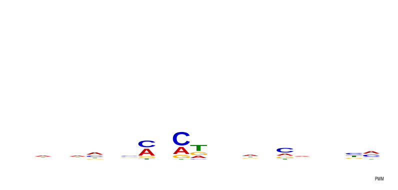	NGAATNNNNNNNNNNNAATTN	1.58	M	8
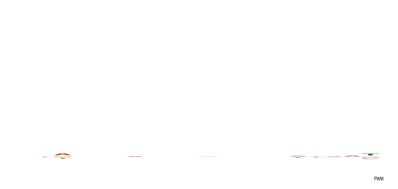	TACTNNNNNNNNNNNTAAT	1.54	M	9

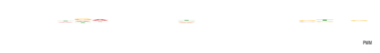	*ATTGNNNNNNNNNN*$\overrightarrow{NNGTCG}$	1.66	M	10
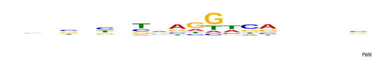	*NNNNNTTA*$\overrightarrow{AGGTCA}$*NNNNNN*	3.02	M	11
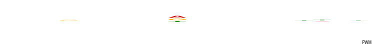	$\overrightarrow{ATGTN}$*NNNNNNNNNNTTAT*	1.61	M	12
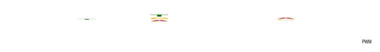	*TTTTNNNNNNNNNNNNTTTG*	1.75	M	13
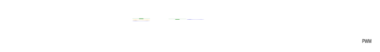	*ACAGNNNNNNNNNNNNAATT*	1.48	M	14
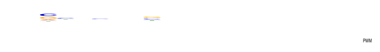	*TCCCNNNNNNNNNNNNTCGT*	1.58	M	15
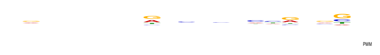	*GCGTNNNNNNNNNNNN*$\overrightarrow{AGGA}$	1.68	M	16
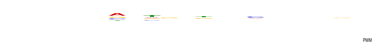	*CATGNNNNNNNNNNNNGCGT*	1.54	M	17
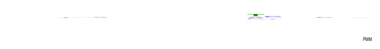	*CGTGNNNNNNNNNNNNCCGG*	1.67	M	18

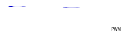	$\overrightarrow{CGTCN}NNNNNNN\overrightarrow{NNNTCA}T$	1.52	DR:7	19
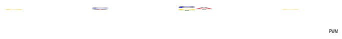	$\overrightarrow{AGGTNN}NNNNNNNNNN\overrightarrow{NGGTC}$	1.71	DR:9	20

115 THRA-Bhimsaria2022-Rnd3 (S07-154)

PWM	Seed Sequence	Seed Seq Enrichment	Repeat	Rank
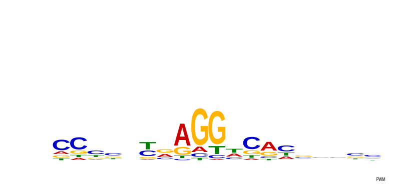	NNNNNNTGAGGTCANNNNNN	15.67	M	1
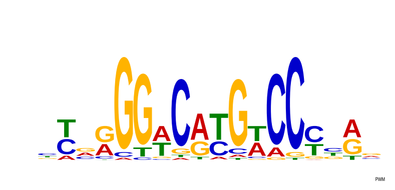	NNNAGGTCATGACCTNNN	158.39	IR:0	2
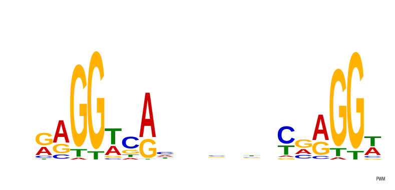	GAGGTCANNNNNNNNNGAGGT	268.89	DR:9	3
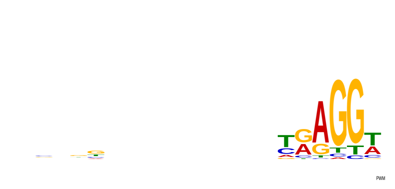	CGGGNNNNNNNNNNNTGAGGT	56.50	M	4
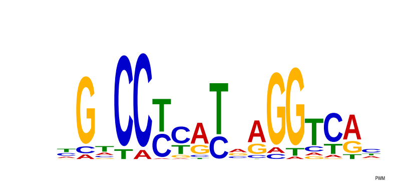	NNGTCCTCANNAGGTCNN	164.00	ER:4	5
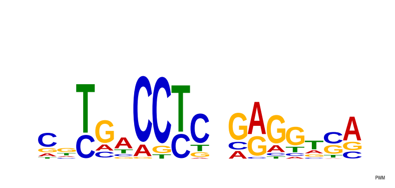	NNNGACCTCNGAGGTNNN	51.50	ER:3	6
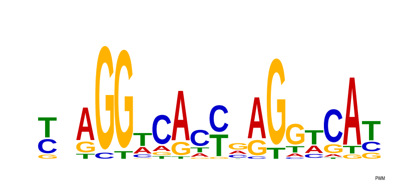	NNAGGTCACNNAGGTCNN	100.87	DR:3	7
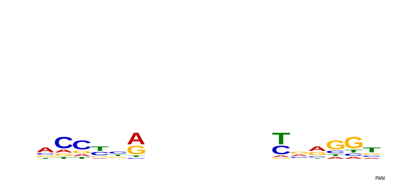	ACCTNNNNNNNNNNNAGGT	11.17	ER:11	8
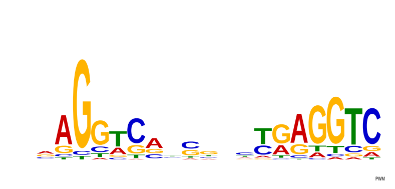	NAGGTCNNNNNNTGAGGTN	46.67	DR:7	9

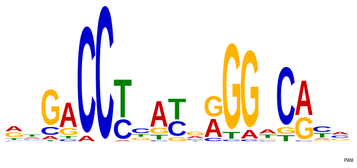	*NNGACCTCNNNNGGACANN*	58.67	ER:4	10
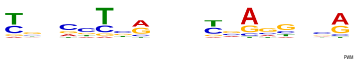	*NGACCNNNNNNNNNGGTCN*	5.07	ER:7	11
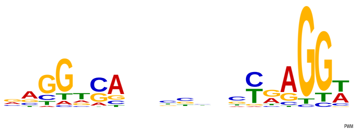	*AAGGTNNNNNNNNNTGAGGT*	110.06	DR:9	12
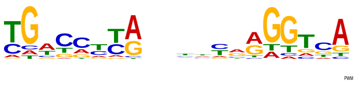	*NGTCCTNNNNNNNNNGGTCN*	10.96	ER:8	13
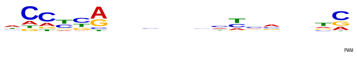	*ACCTNNNNNNNNNNNNGGAC*	6.28	ER:11	14
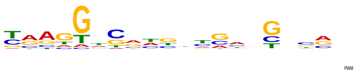	*NNAGGTCNNNNNNNNGGTCNN*	8.61	DR:5	15
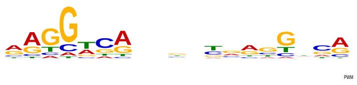	*NAGGTCNNNNNNNNGGTCN*	12.79	DR:6	16
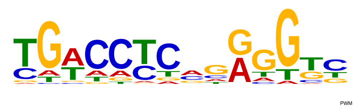	*NGTCCTCNGGGGTN*	28.92	ER:3	17
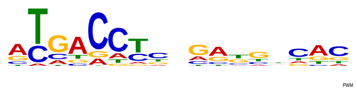	*NNGACCTCNNNGGACNN*	11.51	ER:3	18

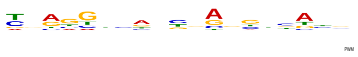	$NN\overrightarrow{NGGTCN}NNN\overrightarrow{NGGTCN}NN$	5.28	DR:3	19
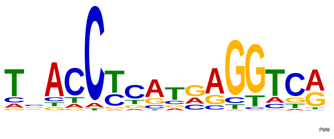	$N\overleftarrow{TCACCT}NNNN\overrightarrow{AGGTCN}$	42.95	ER:4	20

116 THRA-Jolma2013-Rnd2 (ERR194565)

PWM	Seed Sequence	Seed Seq Enrichment	Repeat	Rank
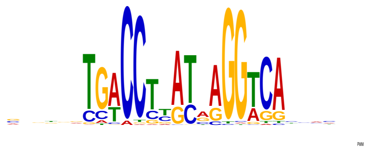	NNNNNNTGACCTNNNNNGGTCANNNNNN	63.85	ER:4	1
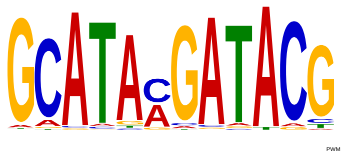	GCATANGATACG	42.96	M	2
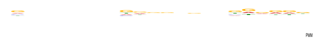	NNNNNNGGGGNNNNNNGGGGNNNNNN	3.54	M	3
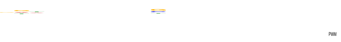	NNNNNNGGCANNNGATANNNNNN	1.18	M	4
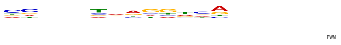	NNNNNNTAAGGTCANNNNNN	2.59	M	5
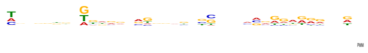	NNNGGGNNNNNNNNNNNNNNNNNNNNNNNNNGGGGNNN	3.40	M	6
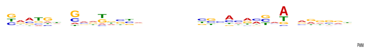	NAATGNNNNNNNNNNNNNNNNNNNNNNNNNNNNNGGGTN	2.30	M	7
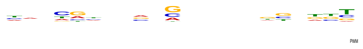	NNNNNNGCATNCGATANNNNNN	1.03	M	8
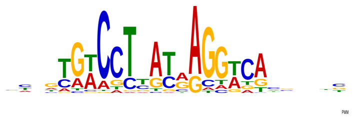	NNNNNNGTCCTNNNNNGGACANNNNNN	9.19	ER:4	9

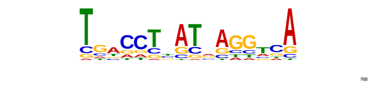	NNNNNNGACCNNNNNNGGTCNNNNNN	3.97	ER:4	10
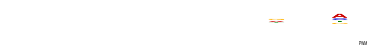	NNNNNNGGCANNCGATNNNNNN	0.92	M	11
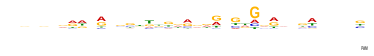	NGGGANNNNNNNNNNNNNNNNNNNNNNNNNNGGGTN	2.09	M	12
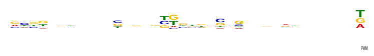	NNGGGGNNNNNNNNNNNNNNNNNNNNNNNNNGGATNN	2.39	M	13
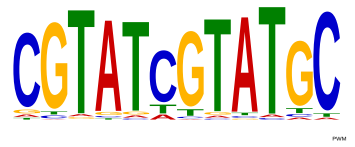	CGTATNGTATGC	37.17	M	14
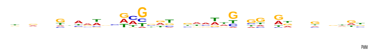	NNATGGNNNNNNNNNNNNNNNNNNNNNNNNNNGGGTNN	2.19	M	15
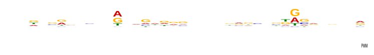	NGGGGNNNNNNNNNNNNNNNNNNNNNNNNNNNNTGGTN	2.05	M	16
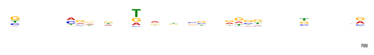	NNGGGGNNNNNNNNNNNNNNNNNNNNNNNNNNNGTTGNN	1.84	M	17
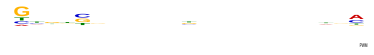	NNNNNNGTATNNTATGCNNNNNN	0.81	M	18

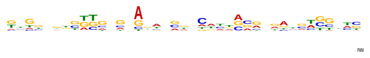	*NNGGCGNNNNNNNNNNNNNNNNNNNNNNNNNNNTGGTNN*	2.08	M	19
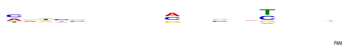	*NNNNNNGCATANGATANNNNNN*	0.63	M	20

117 THRA-Jolma2013-Rnd3 (ERR194566)

PWM	Seed Sequence	Seed Seq Enrichment	Repeat	Rank
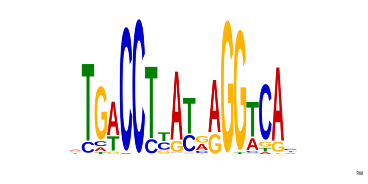	NNNNNNTGACCTTANNAGGTCANNNNNN	24126.75	ER:4	1
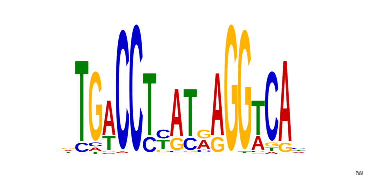	NNNNNNGACCTNNNNAGGTCNNNNNN	930.23	ER:4	2
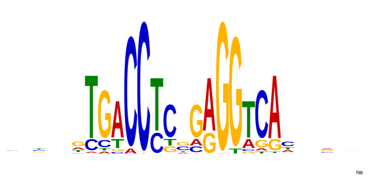	NNNNNNTGACCTCNGAGGTCANNNNNN	6476.24	ER:3	3
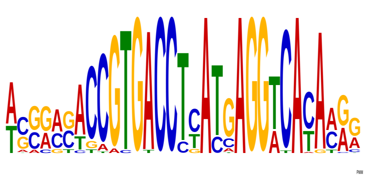	NNNNNNACCGTGACNNNATGAGGTCNNNNNN	33623.59	M	4
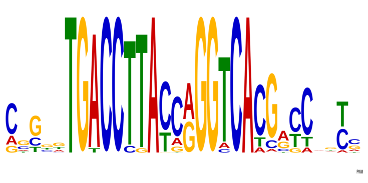	NNNNNNGACCTTACNNGGTCACGANNNNNN	44405.85	ER:4	5
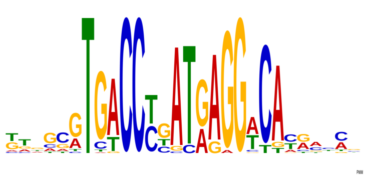	NNNNNNTGACCCGATGAGGACANNNNNN	26276.98	ER:4	6
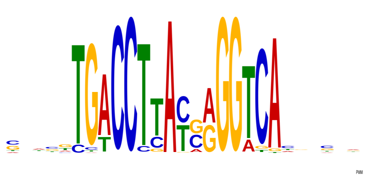	NNNNNNGACCTTACNGGGTCANNNNNN	7600.18	ER:4	7
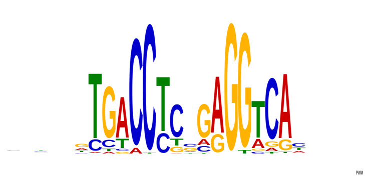	NNNNNNTGACCTCNGAGGTCNNNNNN	2733.24	ER:3	8
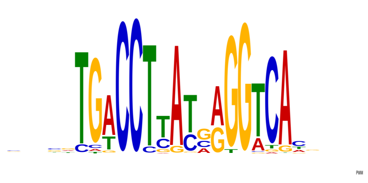	NNNNNNGACCTTACNNGGTCNNNNNN	1650.75	ER:4	9

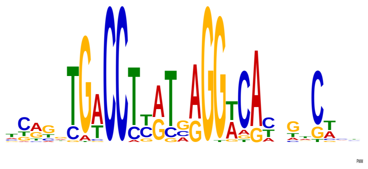	$NNNNN\overleftarrow{NGTCCT}CATN\overrightarrow{NGGTCA}CGNNNNNN$	9593.10	ER:4	10
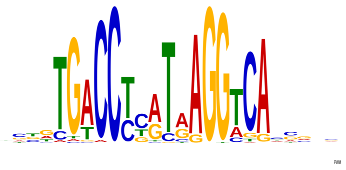	$NNNNN\overleftarrow{NGACCT}CGNA\overrightarrow{AGGTCN}NNNNN$	2700.92	ER:4	11
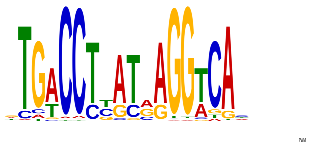	$NNNNN\overleftarrow{NGACCN}NNNN\overrightarrow{NGGTCN}NNNNN$	117.79	ER:4	12

118 THRA-Jolma2013-Rnd4 (ERR194567)

PWM	Seed Sequence	Seed Seq Enrichment	Repeat	Rank
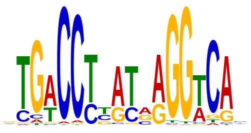	NNNNNNGACCNNNNNNGGTCNNNNNN	84.69	ER:4	1
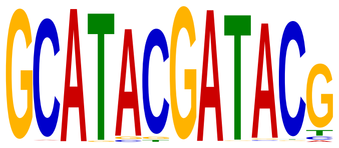	GCATNNGATACG	308.78	M	2
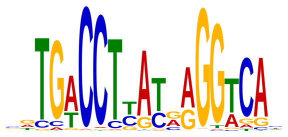	NNNNNNCTTANNNNNGTCANNNNNN	49.91	M	3
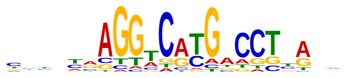	NNNNNNAGGTNNNGACCNNNNNN	13.72	IR:0	4
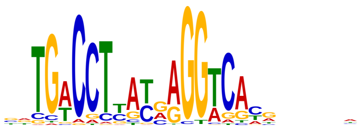	NNNNNNCTTANNNNNNCACGNNNNNN	18.04	M	5
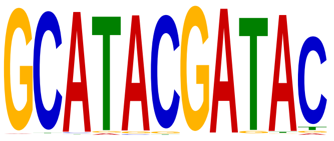	GCATNNGATAC	115.03	M	6
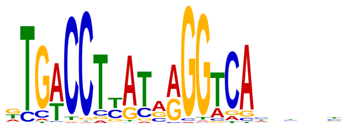	NNNNNNTTATNNNNNTCAANNNNNN	16.09	M	7
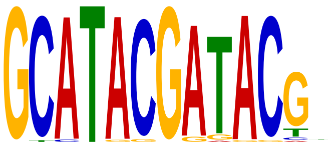	GCATNNNATACGA	94.05	M	8
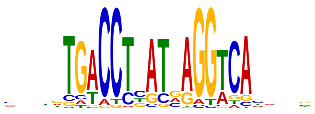	NNNNNNGTGANNNNNNNNNNTCACNNNNNN	18.60	DR:7	9

119 THRA-Jolma2013-Rnd5 (ERR194568)

PWM	Seed Sequence	Seed Seq Enrichment	Repeat	Rank
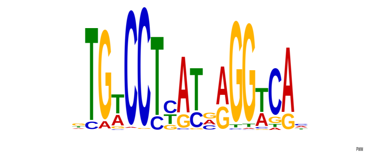	NNNNNNTGTCCNNNNNNGGTCNNNNNN	45.64	ER:4	1
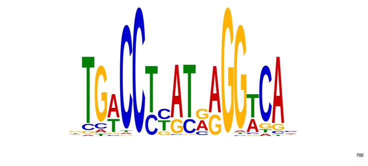	NNNNNNTGACNNNNNNNNNGTCANNNNNN	19.93	ER:4	2
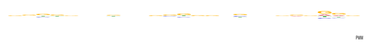	NNNNNNGGGGNNNNNGGGGNNNNNN	6.83	M	3
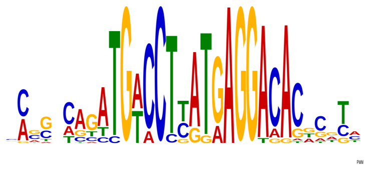	NNNNNNAGATGNNNNNNNGAGGACANNNNNN	136.77	DR:7	4
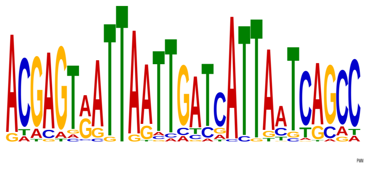	NNNNNNAATTAANNNNNNATTAANNNNNN	36.27	M	5
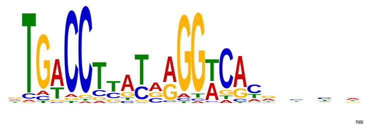	NNNNNNTTATNNNNNCACGNNNNNN	8.42	M	6
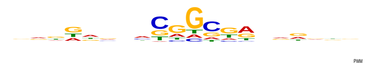	NNNNNNTACGGCGANNNNNN	4.55	M	7
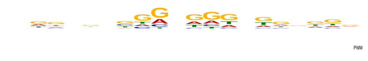	NNNNNNGGGCGGGANNNNNN	4.92	M	8
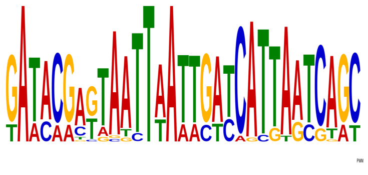	NNNNNNAGTAATNNNNNNNNNATTANNNNNN	18.23	M	9

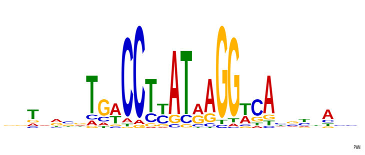	$NNNNNNT\overleftarrow{TGANN}NNNN\overrightarrow{NNNTCA}ANNNNNN$	3.42	ER:4	10
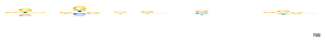	$NNNNNNTGGGNNNNNNNGGGGNNNNNN$	5.46	M	11

120 THRA-7-Bhimsaria2022-Rnd3 (S07-102)

PWM	Seed Sequence	Seed Seq Enrichment	Repeat	Rank
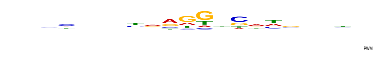	NNNNNNTAAGGTCANNNNNN	6.29	M	1
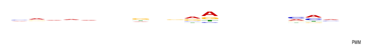	CAAANNNNNNNNNNNNCAAT	4.90	M	2
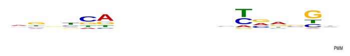	GAGGNNNNNNNNNNNNAGGT	5.23	DR:9	3
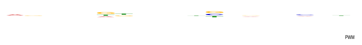	AGTTNNNNNNNNNNNNCCAT	4.60	M	4
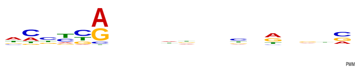	ACCTNNNNNNNNNNNNGGAC	4.14	ER:11	5
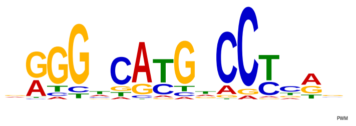	NNGGTCATGNCCTCNN	38.28	IR:0	6
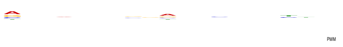	AACANNNNNNNNNNNNTTTT	4.47	M	7
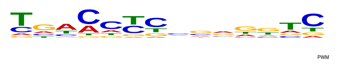	NNGACCTCNGAGGNN	8.54	ER:3	8
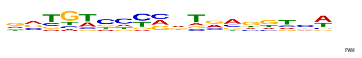	NNNGTCCCNNNNAGGTNNN	6.17	DR:5	9

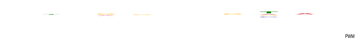	CTTCNNNNNNNNNNNNCAAT	3.38	M	10
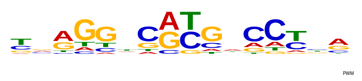	NNAGGTCNNGACCTNN	13.31	IR:0	11
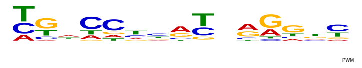	NGTCCTCNNNGGGTN	8.33	ER:4	12
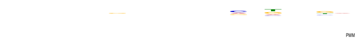	AAGTNNNNNNNNNNNNGTGA	3.77	DR:8	13
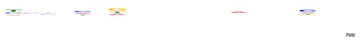	TCCCNNNNNNNNNNNNTCAT	3.91	M	14
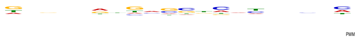	NNNNNNTGACGTCANNNNNN	4.11	M	15
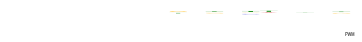	TATTNNNNNNNNNNNNTTCT	4.44	M	16
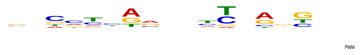	GACCTNNNNNNNNNNGGTC	4.67	ER:8	17
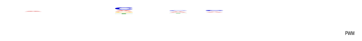	GAAGNNNNNNNNNNNNAATG	3.77	M	18

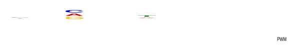	*GAGTNNNNNNNNNNNCAAT*	3.39	M	19
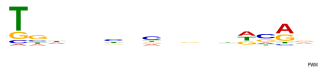	$NN\overleftarrow{NNACCT}CNG\overrightarrow{AGGNNN}N$	3.02	ER:3	20

121 THRB:RXRA-Bhimsaria2022-Rnd3 (S13-164)

PWM	Seed Sequence	Seed Seq Enrichment	Repeat	Rank
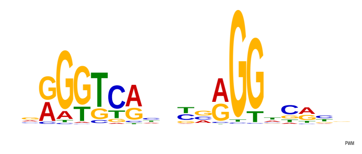	NNNGGTCNNNNNNGGTCNNN	114.31	DR:4	1
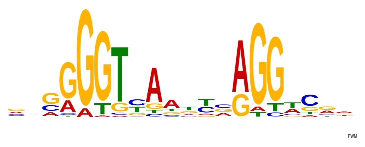	NNGGGGTTANNNNNGGTCNN	457.86	DR:4	2
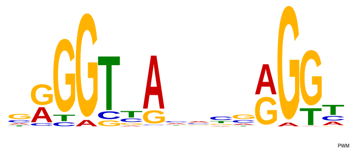	GGGGTTANNCGGGGT	1872.25	DR:4	3
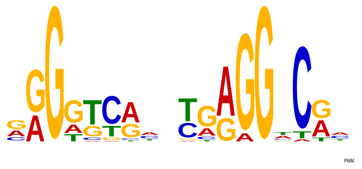	NNGGGCNNNTGAGGACNN	298.34	DR:4	4
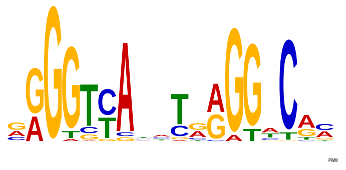	NNGGGTTANNNNGGGACNN	303.07	DR:4	5
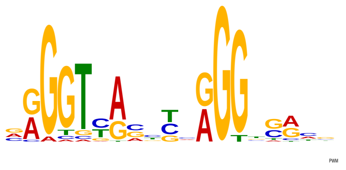	NNGGGTTANNNNNNGTCANN	142.77	DR:4	6
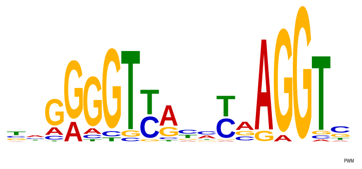	NAGGGGTNNNNCAAGGTN	637.91	DR:4	7
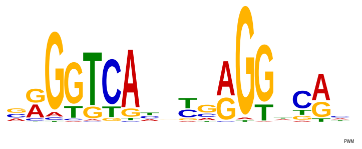	NNGGTCAANNNNGGCCNN	148.65	DR:4	8
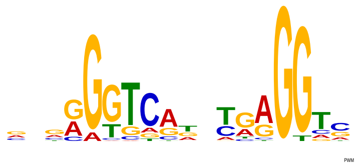	NCGGGGTNNNNTGAGGTN	691.90	DR:4	9

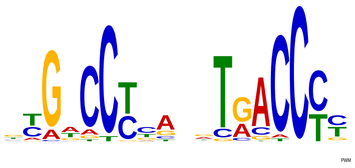	*NNGTCCCCNNNTAACCNN*	224.25	IR:5	10
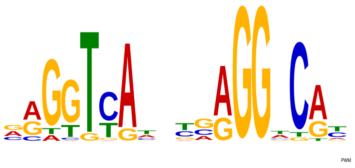	*NNGGTCAACNNNGGACNN*	317.66	DR:4	11
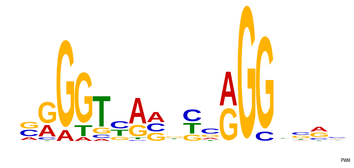	*NGGGGTTANNNNNNNGTCAN*	225.54	DR:4	12
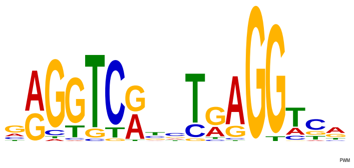	*NNGGTCGNCTGAGGTNN*	1047.38	DR:4	13
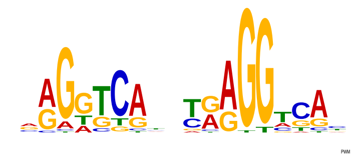	*NNNGGGCNNNTGAGGTNNN*	145.15	DR:4	14
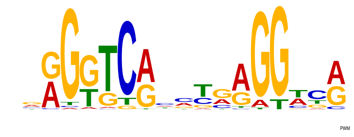	*NGAGTTCNNNNNNGGTCN*	151.00	DR:4	15
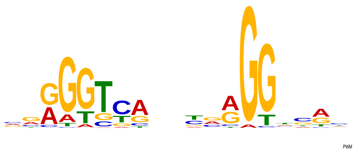	*NGGGGTNNNNNNNNGTCAN*	82.06	DR:4	16

122 THRB:RXRA-7-Bhimsaria2022-Rnd2 (S13-127)

PWM	Seed Sequence	Seed Seq Enrichment	Repeat	Rank
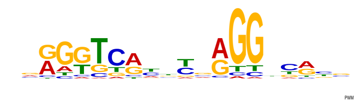	NNNGGTCNNNNNNGGTCNNN	13.45	DR:4	1
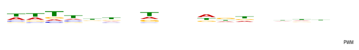	TTTTNNNNNNNNNNNTTTC	5.01	M	2
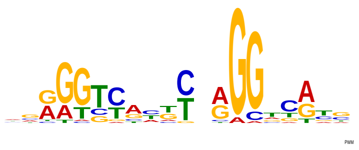	NNNGGTCATNNNNGGTCNNN	42.71	DR:4	3
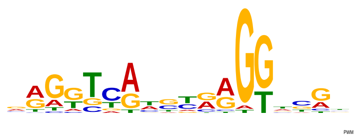	NNGGTCNNNTGAGGTCNN	73.39	DR:4	4
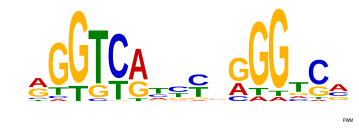	NNGGTCATNCGGGGTNN	111.68	DR:4	5
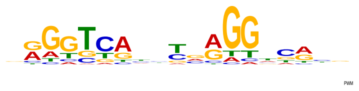	NNNGTCANNNNNGGTCNNN	11.56	DR:4	6
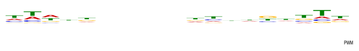	TTATNNNNNNNNNNNNTTTT	7.42	M	7
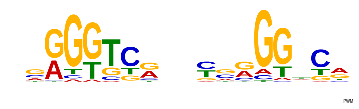	NGGGGTCNNNNNNGGGCN	20.32	DR:4	8
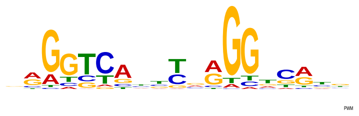	NNNGTCATNNNNGGTCNNN	17.76	DR:4	9

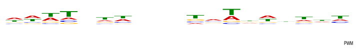	AATTNNNNNNNNNNNTTTT	7.48	M	10
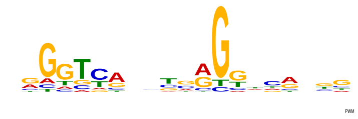	N$\overrightarrow{NNGTCA}$NNNN$\overrightarrow{NNGTCA}$NNN	7.29	DR:4	11
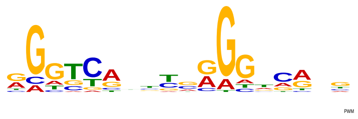	$\overrightarrow{NNGTCA}$TNNN$\overrightarrow{NNGTCA}$NN	9.83	DR:4	12
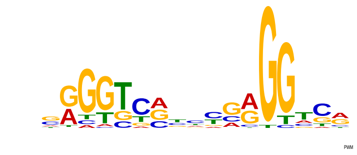	NNG$\overrightarrow{GGGTCN}$NNNG$\overrightarrow{AGGTNN}$	63.89	DR:4	13
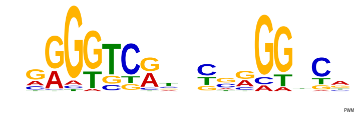	NG$\overrightarrow{GGGTCN}$NNNN$\overrightarrow{GGGGCN}$	31.46	DR:4	14
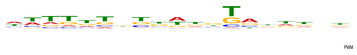	NTTTTNNNNNNNNNTTTCN	5.24	M	15
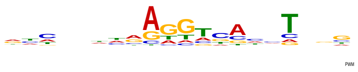	NNNNNNTA$\overrightarrow{AGGTCA}$NNNNNN	4.19	M	16
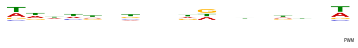	NNTTTTNNNNNNNTTTCNN	3.99	M	17
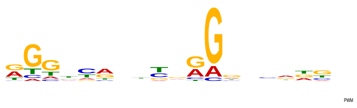	$\overrightarrow{NNNTCA}$TNNNN$\overrightarrow{NNGTCA}$NNN	3.62	DR:4	18

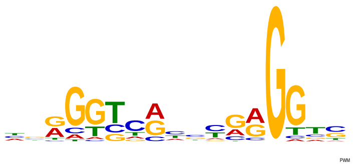	$NGG\overrightarrow{GGTCN}NNTG\overrightarrow{AGGTN}$	88.69	DR:4	19
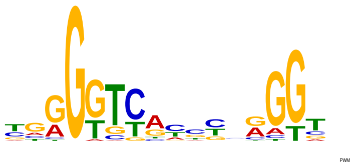	$NGG\overrightarrow{GGTCA}CNNN\overrightarrow{GGGTN}$	101.02	DR:4	20

123 THRB-Bhimsaria2022-Rnd2 (S07-035)

PWM	Seed Sequence	Seed Seq Enrichment	Repeat	Rank
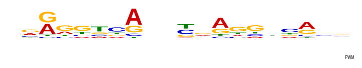	NNNGGTCNNNNNNGGTCNNN	3.45	DR:4	1
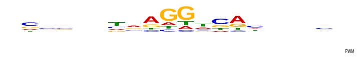	NNNNNNTAAGGTCANNNNNN	4.39	M	2
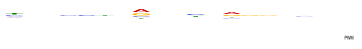	NNACCTNNNNNNNGGTCNN	1.86	ER:6	3
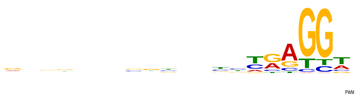	GAGGNNNNNNNNNNTGAGGT	10.85	DR:9	4
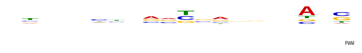	NNNACCTNNNNNNGGTCNNN	1.85	ER:5	5
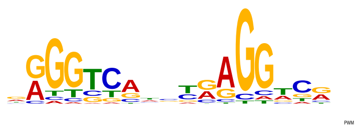	NNGGTCNNNTGAGGTCNN	27.31	DR:4	6
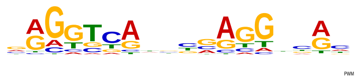	NNGGTCACNNNNGGTCNN	7.93	DR:4	7
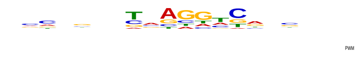	NNNNNNTTAAGGTCNNNNNN	3.93	M	8
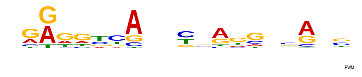	NNNGGTCNNNNNGGGTCNNN	3.39	DR:4	9

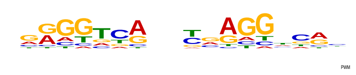	NNGGGTCNNNNNNGGTCNN	5.79	DR:4	10
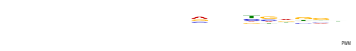	AGTTNNNNNNNNNNNNAGGT	2.30	DR:10	11
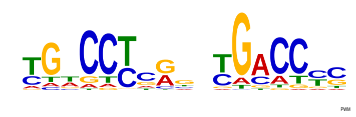	NNGTCCCCANNNGACCNN	11.36	IR:5	12
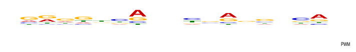	NNNGGTCNNNNNGGGTNNN	2.21	DR:4	13
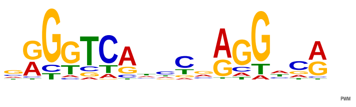	NNGGTCATNNNGGGTCNN	13.81	DR:4	14
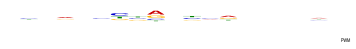	NNGACCNNNNNNNGGACNN	1.82	ER:5	15
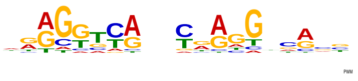	NNNGGTCATNNNNGGTCNNN	8.50	DR:4	16
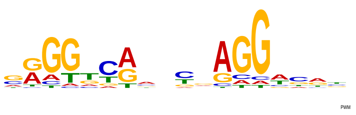	NNGGTCNNNTGGGGTCNN	8.77	DR:4	17
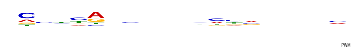	ACCTNNNNNNNNNNNNNGGAC	2.02	ER:11	18

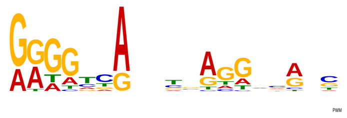	$NN\overrightarrow{NGGTCN}NNNG\overrightarrow{GGGTCN}NN$	4.56	DR:4	19
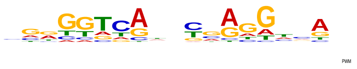	$NN\overrightarrow{NGGTCA}TNNN\overrightarrow{GGGTN}NN$	5.88	DR:4	20

124 THRB-Bhimsaria2022-Rnd3 (S07-155)

PWM	Seed Sequence	Seed Seq Enrichment	Repeat	Rank
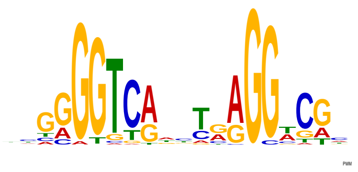	NNGGGGTCNNNTGAGGTCNN	1741.26	DR:4	1
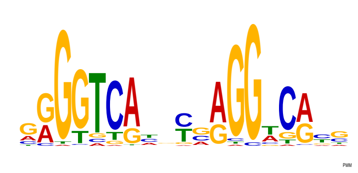	NNNGGTCACNNNNGGTCNNN	149.52	DR:4	2
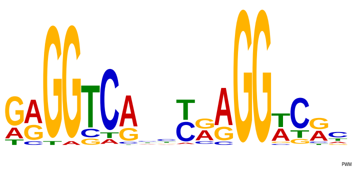	NNGGTCGTNTGAGGTCNN	467.81	DR:4	3
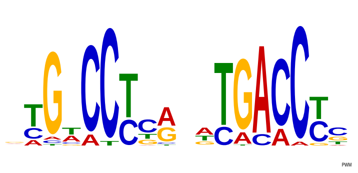	NNGTCCCCANATGACCNN	586.55	IR:5	4
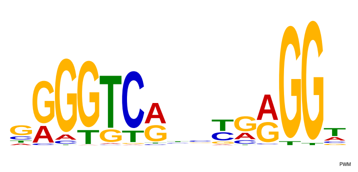	GGGGTCNTCTGGGGT	1756.23	DR:4	5
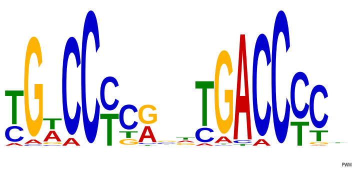	NGTCCCCGNNNGACCCCN	845.51	IR:5	6
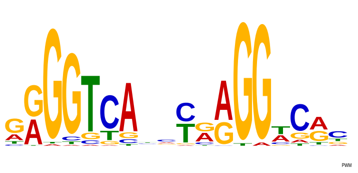	NNGGTTANNTGAGGTCNN	206.73	DR:4	7
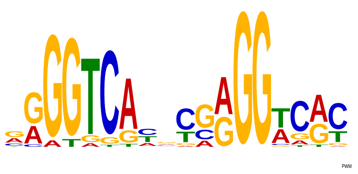	NNGGTCACNCGGGGTCNN	795.73	DR:4	8
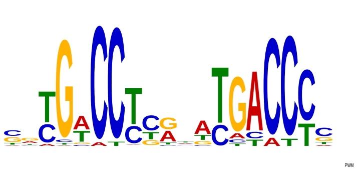	NNNGTCCCCANNNGACCNNN	131.37	IR:5	9

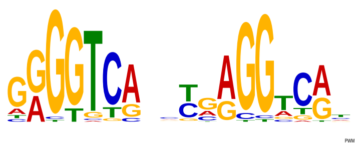	NNGGTCNNCTGGGGTCNN	266.92	DR:4	10
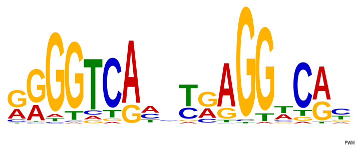	NNGGTCNNNTGAGGCCNN	96.72	DR:4	11
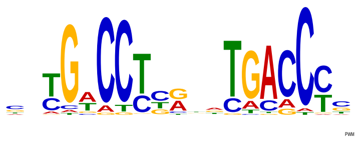	NNNGTCCCCANNTGACNNN	96.79	IR:5	12
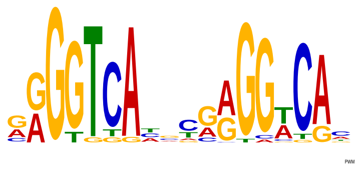	NNGGTCACNNGGGGACNN	243.40	DR:2	13
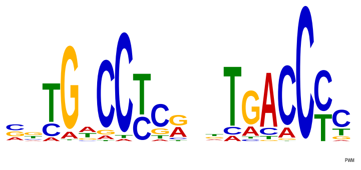	NNNGTCCCCGNNTGACNNN	105.64	IR:5	14
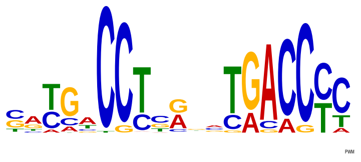	NNNGACCCCAGNTGACNNN	200.23	ER:1	15
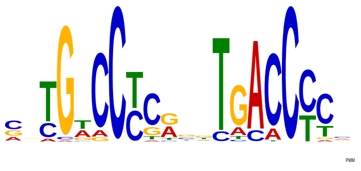	NNNGTCCCCGNNNGACCNNN	142.60	IR:5	16
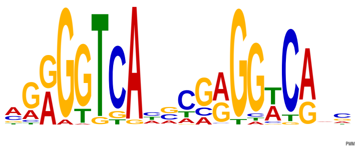	NNNGGTCACNNNGGGACNNN	148.67	DR:4	17

125 THRB-Jolma2013-Rnd3 (ERR194572)

PWM	Seed Sequence	Seed Seq Enrichment	Repeat	Rank
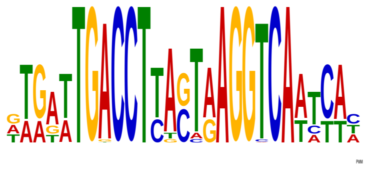	NNNNNTGACCTTANTAAGGTCANNNNN	213164.29	ER:5	1
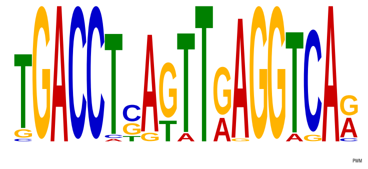	NGACCTGAGNTGAGGTCAN	52305.22	ER:6	2
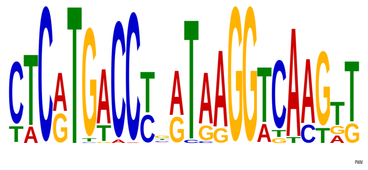	NNNNTGACCTGATAAGGTCANNNN	111848.04	ER:4	3
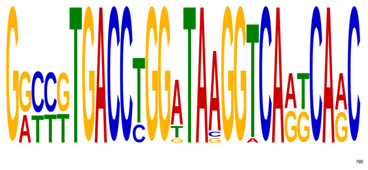	NNNNNNGACCTGGATAAGGTCANNNNNN	100821.88	ER:5	4
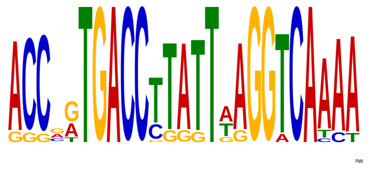	NNNCATGACCTNNNTAAGGTCANNN	34165.96	ER:5	5
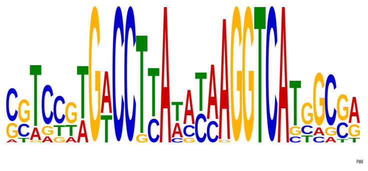	NNNNNNTGTCCTTANNNNAGGTCANNNNNN	19133.97	ER:6	6
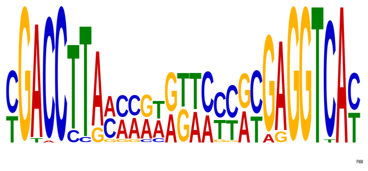	NGACCTTANNNNNNNNNNNNNGAGGTCAN	18455.00	ER:16	7
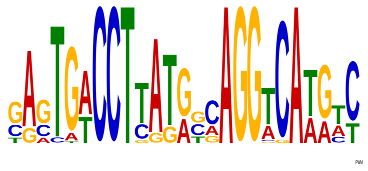	NNNTGTCCTCANNNNAGGTCAANNN	13686.08	ER:6	8
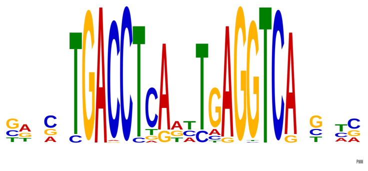	NNNNNNGACCTCANNTGAGGTCNNNNNN	43789.97	ER:6	9

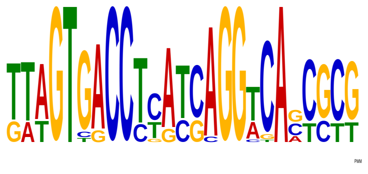	$NNNN\overleftarrow{TGACCT}CANN\overrightarrow{AGGTCA}GNNNN$	15246.73	ER:4	10
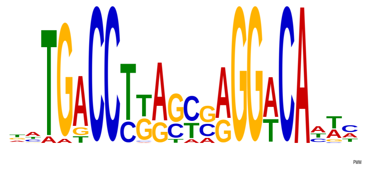	$NN\overleftarrow{TGACCT}TANNN\overrightarrow{AGGTCA}GNN$	6720.62	ER:5	11
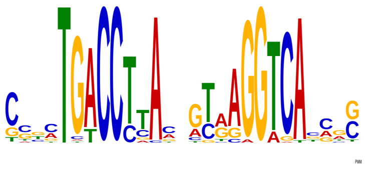	$NNNN\overleftarrow{TGACCT}TANNNNN\overrightarrow{AGGTCA}NNNN$	8907.52	ER:7	12
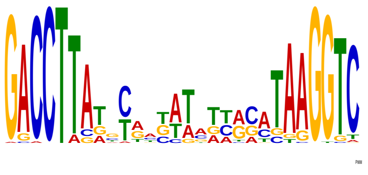	$\overleftarrow{GACCT}TANNNNNNNNNNNNNNNN\overrightarrow{AGGTC}$	1148.89	ER:18	13
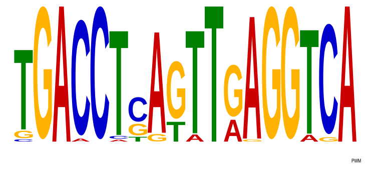	$\overleftarrow{NGACCT}GAGNTG\overrightarrow{AGGTCN}$	18011.24	ER:6	14
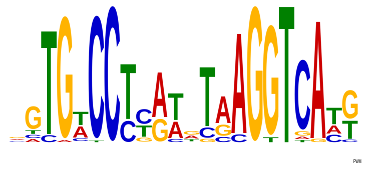	$NN\overleftarrow{TGTCCT}CGNNNN\overrightarrow{AGGTCA}NN$	4599.63	ER:6	15
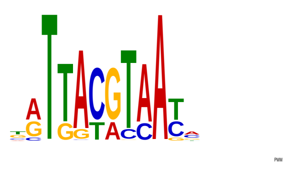	$NNNNNNATT\overrightarrow{ACGTAA}TNNNNNN$	240.99	M	16
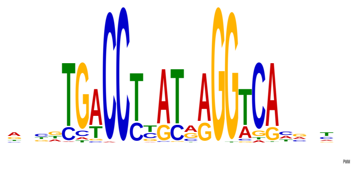	$NNNNN\overleftarrow{NGACCT}NNNN\overrightarrow{AGGTCN}NNNNN$	514.05	ER:4	17
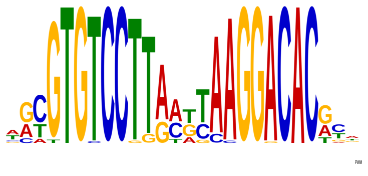	$NNNG\overleftarrow{TGTCCT}TNNNNN\overrightarrow{AGGACA}CNNN$	37729.36	ER:6	18

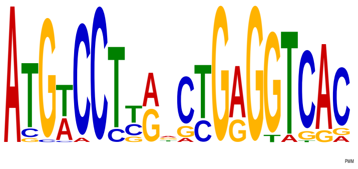	$NN\overleftarrow{GACCT}CACNNG\overrightarrow{AGGTC}NN$	2557.98	ER:6	19
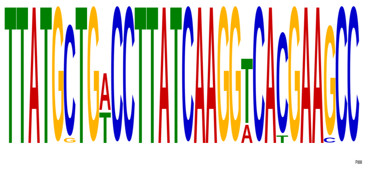	$NNNNNNT\overleftarrow{GTCCT}TANNN\overrightarrow{AGGTC}ACNNNNNN$	48908.23	ER:5	20

126 THRB-Jolma2013-Rnd4 (ERR194573)

PWM	Seed Sequence	Seed Seq Enrichment	Repeat	Rank
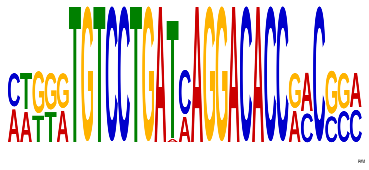	NNNNNNGTCCTGATNNNGACACCNNNNNN	9991.35	ER:4	1
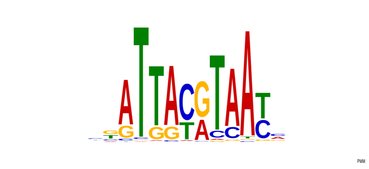	NNNNNNGATTNNNTAATNNNNNN	19.73	M	2
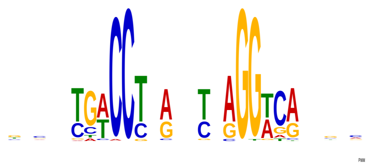	NNNNNNGACCNNNNNNNNGGTCNNNNNN	120.87	ER:6	3
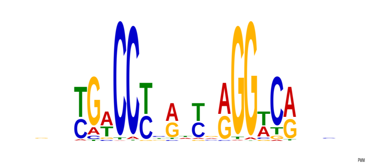	NNNNNNGACCNNNNNNNGGTCNNNNNN	80.47	ER:5	4
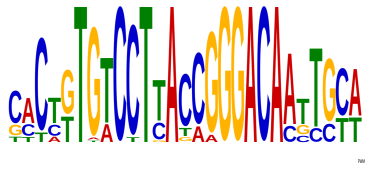	NNNNNNGTCCTTACNGGGACANNNNNN	4676.68	ER:4	5
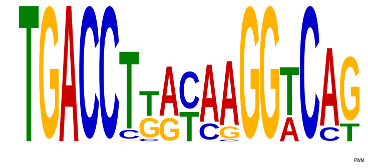	NGACCCGANCGGGTCAN	470.45	ER:4	6
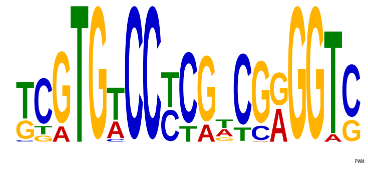	GCGTGNNNNNNNCGGGGTC	158.41	M	7
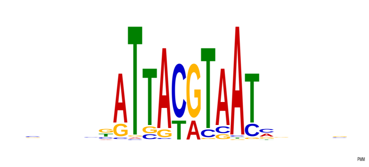	NNNNNNGATTNNNNAATCNNNNNN	16.88	M	8
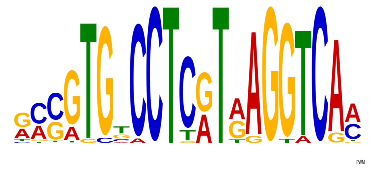	NNCATGCCCTNNNNNGGTCNN	123.71	ER:4	9

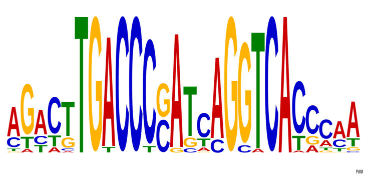	NNNNNNGACCCGAGNNGGTCNNNNNN	235.21	ER:4	10
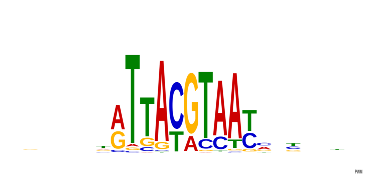	NNNNNNTGTTNNNNAATANNNNNN	11.13	M	11
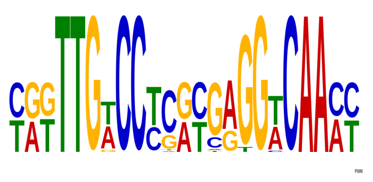	NNNNTGACCCCNNGGGGTCNNNN	262.86	ER:4	12
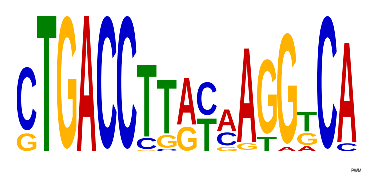	NNGACCCGANCGGGTNN	80.50	ER:4	13
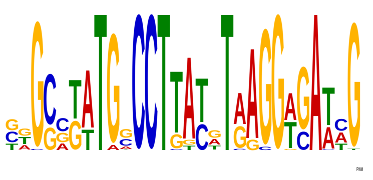	NNNNNNATGGCCTTNNNNAAGGNNNNNN	337.53	ER:6	14
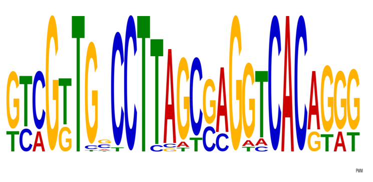	NNNNNNGCCCTTAGNNNGGTCNNNNNN	268.84	ER:5	15
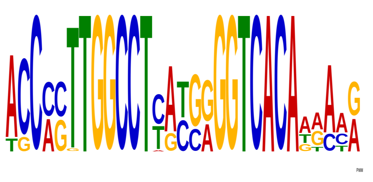	NNNNNTTGGCCTNNNNNNGTCACANNNNN	2033.47	ER:4	16
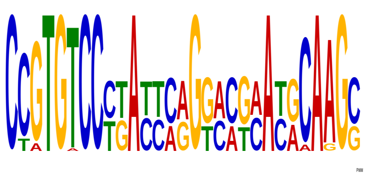	NCGTGTCNNNNNNNNNNNNNNNNNCAAGN	21.08	M	17
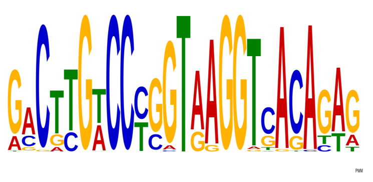	NNNNNGACCCGNNNAGGTGANNNNN	232.28	ER:4	18

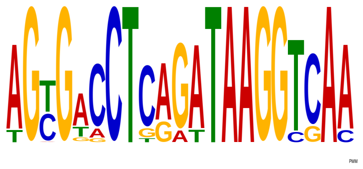	$NN\overleftarrow{CGGCCT}CNNNNNN\overrightarrow{GGTC}NN$	30.34	ER:6	19
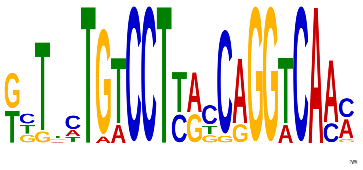	$NNNAT\overleftarrow{TGTCC}NNNNN\overrightarrow{GGGAC}NNN$	68.16	ER:4	20

127 THRB-Jolma2013-Rnd5 (ERR194574)

PWM	Seed Sequence	Seed Seq Enrichment	Repeat	Rank
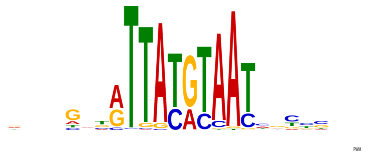	NNNNNNTGTTNNNNAATCNNNNNN	32.19	M	1
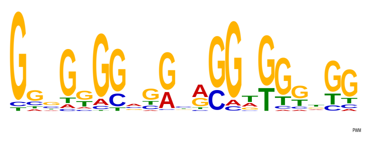	NNNNGGGAGGNNGGTGGNNNN	200.41	M	2
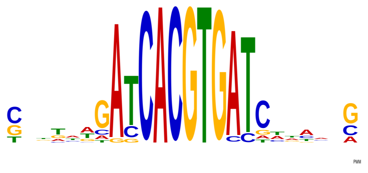	NNNNNNGATCACGNGATCNNNNNN	170.55	DR:2	3
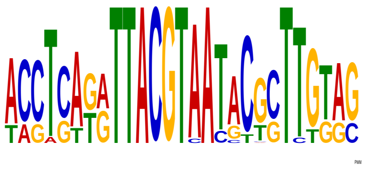	NNNNNNTGTTNNNNAATACGGNNNNNN	141.08	M	4
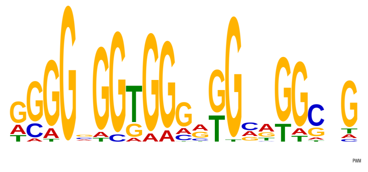	NNGGCGGNNNNNNGGAGGGNN	99.12	M	5
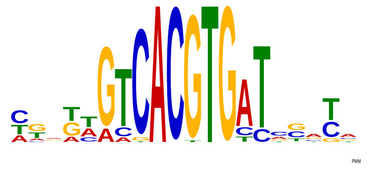	NNNNTGTCACGNGATCNNNN	77.75	DR:2	6
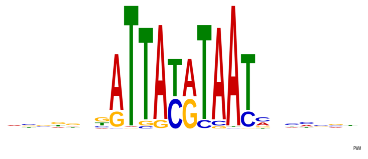	NNNNNNGATTNNNNAATCNNNNNN	58.53	M	7
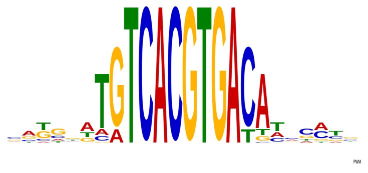	NNNNNNTGTCACGTGACANNNNNN	362.55	IR:2	8
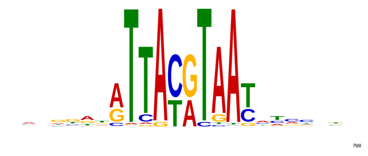	NNNNNNTGTTNNNNAACANNNNNN	14.37	M	9

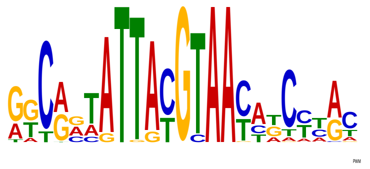	*NNNNATATTNNNNAACAGCNNNN*	102.33	M	10
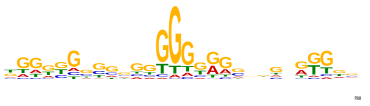	*NNNNNNGGGGNNNNNNNGGGGGNNNNNN*	41.95	M	11
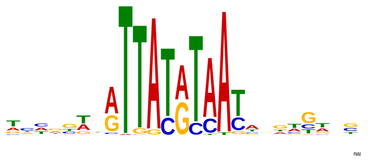	*NNNNNNTGTTNNNNAATCCNNNNNN*	18.04	M	12
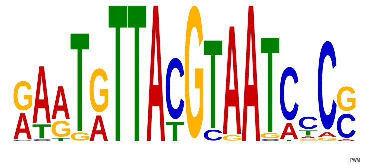	*NA$\overrightarrow{ATGTTN}$NNNAATCCCN*	230.35	M	13
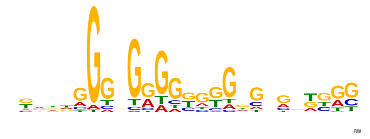	*NNNNNGGGAGNNNNNNGGGGGNNNNN*	49.40	M	14
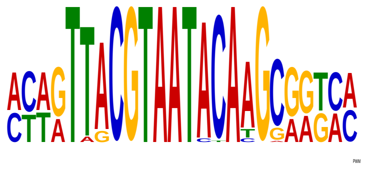	*NNNNNTACGNNNTACAAGCNNNNN*	148.32	M	15
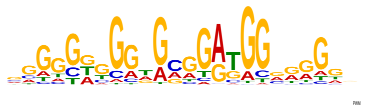	*NNNNNN$\overrightarrow{GGGTGN}$$\overrightarrow{NGGTGG}$NNNNNN*	147.75	DR:0	16
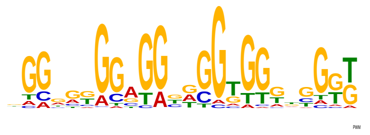	*NNNNNNGGAGGN$\overrightarrow{NGGTGG}$NNNNNN*	209.21	M	17
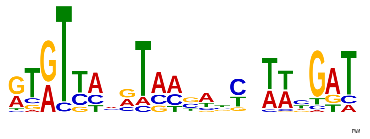	*NNNNNNCGT$\overrightarrow{ANNTCC}$GNNNNNN*	8.51	M	18

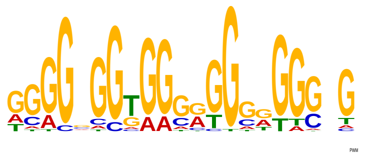	$NNGGCGGNNNNNNNG\overrightarrow{AGGGNN}$	32.49	M	19
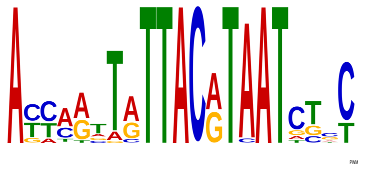	$NNN\overrightarrow{TGGTGT}TNNNNAATCNNN$	54.26	M	20

128 THRB-Yin2017-Rnd2 (ERR1006596)

PWM	Seed Sequence	Seed Seq Enrichment	Repeat	Rank
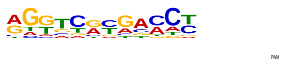	NNNNNNGGTCNNGACCNNNNNN	7.61	IR:0	1
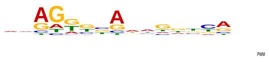	NNNNNNAAAGNNNAAAGNNNNNN	6.99	M	2
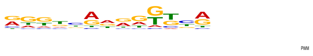	NNNNNNTCAANNGTCANNNNNN	6.37	DR:1	3
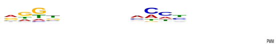	NNNNNNAGGTNNNNNACCTNNNNNN	5.42	IR:1	4
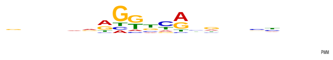	NNNNNNAAAGNNNACGANNNNNN	4.32	M	5
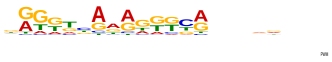	NNNNNNTCAAANNNCAAGNNNNNN	6.62	DR:1	6
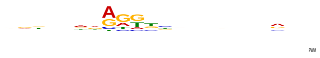	NNNNNNTCAANNNNNNAAAGNNNNNN	2.82	M	7
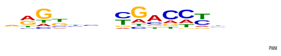	NNNNNNGGTTNNCGACNNNNNN	4.19	IR:1	8
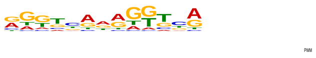	NNNNNNTCAANNNTCAGNNNNNN	5.08	DR:1	9

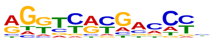	NNNNNNAGGTNNCGACNNNNNN	5.85	IR:0	10
	NNNNNNTCAGNNNTCGCNNNNNN	3.04	M	11
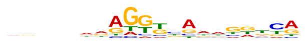	NNNNNNGAAANNNNAAAGNNNNNN	4.57	M	12
	NNNNNNTTAANNNTTAANNNNNN	3.55	M	13
	NNNNNNGGTCNNNNACACNNNNNN	2.91	M	14
	NNNNNNAAGGNNNNNNNNCCTTNNNNNN	4.07	IR:1	15
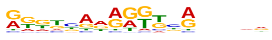	NNNNNNTCAANNNTCAAGNNNNNN	6.00	DR:1	16
	NNNNNNGTCGNCGACNNNNNN	2.88	IR:1	17
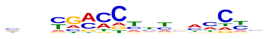	NNNNNNTACGNNNTTTGNNNNNN	3.27	M	18

PWM	$NNNNNNA\overrightarrow{AGGNNN}NN\overleftarrow{NNNCCT}TNNNNNN$	2.96	IR:2	19
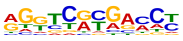 PWM	$NNNNNN\overrightarrow{AGGTNN}\overleftarrow{NNACCT}NNNNNN$	5.61	IR:0	20

129 THRB-Yin2017-Rnd2 (ERR1006597)

PWM	Seed Sequence	Seed Seq Enrichment	Repeat	Rank
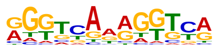	NNNNNNTCAANNGTCANNNNNN	11.65	DR:1	1
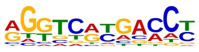	NNNNNNGGTCNNGACCNNNNNN	8.51	IR:0	2
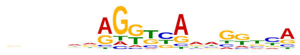	NNNNNNAAAGNNNAAAGNNNNNN	7.21	M	3
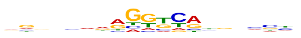	NNNNNNAAAGNNNATGANNNNNN	4.17	IR:0	4
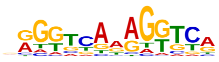	NNNNNNNTCAANNNTCATNNNNNN	8.16	DR:1	5
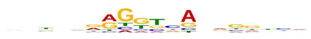	NNNNNNCAGGNNTCAANNNNNN	3.38	M	6
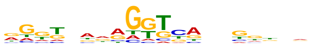	NNNNNNCAAANNNCAAANNNNNN	3.33	M	7
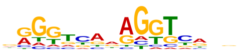	NNNNNNNTCAANNNNNCACGNNNNNN	4.05	M	8
	NNNNNNGGGGNNACGANNNNNN	2.55	M	9

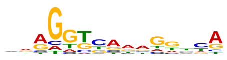 PWM	*NNNNNNGAAANNNNAAAGNNNNNN*	3.71	M	10
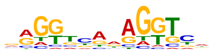 PWM	*NNNNNNTCAANNNNCATTNNNNNN*	3.64	M	11
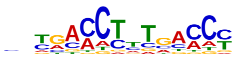 PWM	*NNNNNNGTGANNNTTGANNNNNN*	6.45	IR:4	12
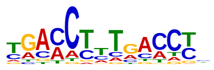 PWM	*NNNNNNGATGANNNTTGANNNNNN*	7.75	IR:4	13
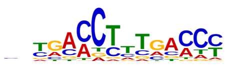 PWM	*NNNNNNTGTGANNNTTGANNNNNN*	6.51	IR:4	14
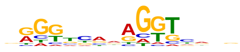 PWM	*NNNNNNTCAANNNNCACANNNNNN*	2.65	M	15
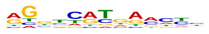 PWM	*NNNNNNGTCGNNNCCCCNNNNNN*	2.39	M	16
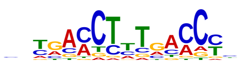 PWM	*NNNNNNAGTGANNNTTGANNNNNN*	6.34	IR:4	17
PWM	*NNNNNNAAGGNNNNNNACCCNNNNNN*	3.37	IR:1	18

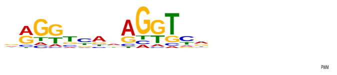	$NNN\overrightarrow{NNNTCA}ANNNNNCATCNNNNNN$	3.03	M	19
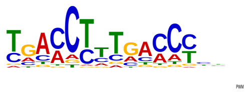	$NNNNN\overrightarrow{NGCTGA}NNNNT\overleftarrow{TGANNNN}NNN$	7.07	IR:4	20

130 THRB-Yin2017-Rnd3 (ERR1006598)

PWM	Seed Sequence	Seed Seq Enrichment	Repeat	Rank
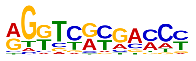	NNNNNNAGGTNNNGACCNNNNNN	8.77	IR:0	1
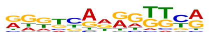	NNNNNNTCAANNNTCATNNNNNN	7.75	DR:1	2
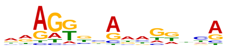	NNNNNNGAAANNNNAAAGNNNNNN	6.75	M	3
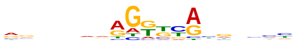	NNNNNNAAAGNNNACGANNNNNN	5.93	M	4
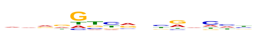	NNNNNNGTCANCGACNNNNNN	3.39	IR:1	5
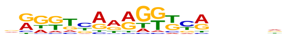	NNNNNNTCAANNNNCAAGNNNNNN	5.40	M	6
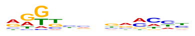	NNNNNNAGGTNNNNACCCNNNNNN	4.64	IR:1	7
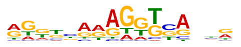	NNNNNNTCAAANNNNNAACGNNNNNN	7.43	M	8
	NNNNNNGTCANNNNNCTTTNNNNNN	3.87	M	9

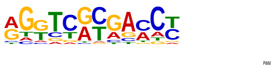	*NNNNNNAGGTNNNNACCTNNNNNN*	7.61	IR:0	10
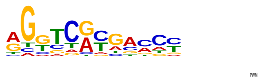	*NNNNNNAAGGNNNNGACCNNNNNN*	6.15	IR:0	11
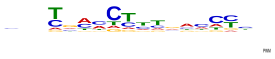	*NNNNNNCTGTNNNNTTTGNNNNNN*	3.61	M	12
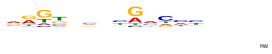	*NNNNNNAGGTNNNCGACNNNNNN*	4.69	IR:1	13
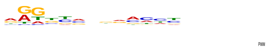	*NNNNNNAAGGNNNNNNACCCNNNNNN*	4.20	IR:1	14
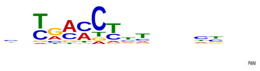	*NNNNNNTCGTNNNNTTTGNNNNNN*	4.43	M	15
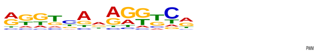	*NNNNNNTCAANNNNCGTANNNNNN*	3.89	M	16
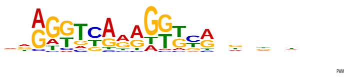	*NNNNNNTCAANNNNCAAGCNNNNNN*	5.36	M	17
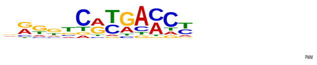	*NNNNNNGTCGNNNNCTCTNNNNNN*	3.67	M	18

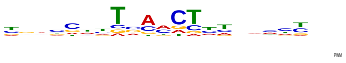	*NNNNNNTTTCNNNTTTGNNNNNN*	4.59	M	19
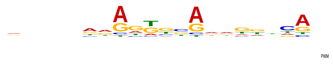	*NNNNNNGAAANNNCAAANNNNNN*	5.24	M	20

131 THRB-Yin2017-Rnd3 (ERR1006599)

PWM	Seed Sequence	Seed Seq Enrichment	Repeat	Rank
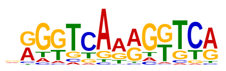	NNNNNNNTCAANNNTCATNNNNNN	15.46	DR:1	1
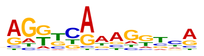	NNNNNNAAAGNNNNAAGGNNNNNN	6.85	M	2
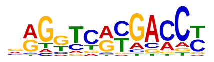	NNNNNNGGGTNNNGACCNNNNNN	6.85	IR:0	3
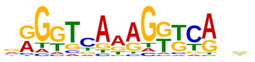	NNNNNNCAAANNNCACGNNNNNN	8.26	M	4
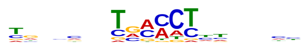	NNNNNNCTTTNNNNTTTGNNNNNN	6.20	M	5
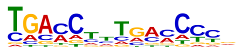	NNNNNNTTTGNNNCCGTNNNNNN	4.06	M	6
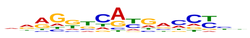	NNNNNNAGAGNNNATGANNNNNN	4.25	IR:0	7
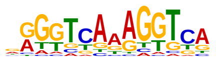	NNNNNNNTCAANNNNCAATNNNNNN	7.33	M	8
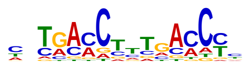	NNNNNNGCGTNNNNTTTGNNNNNN	4.41	M	9

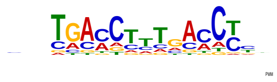	NNNNNNCGATNNNNTTTGNNNNNN	4.93	M	10
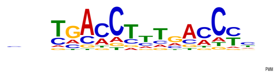	NNNNNNCAGTNNNNTTTGNNNNNN	4.09	M	11
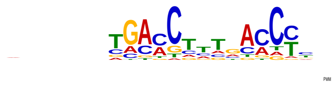	NNNNNNGAATNNNNTTTGNNNNNN	3.38	M	12
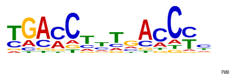	NNNNNNGCATNNNNTTTGNNNNNN	3.76	M	13
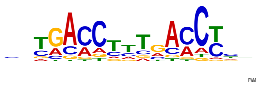	NNNNNNGCTTNNNNTTTGNNNNNN	3.88	M	14
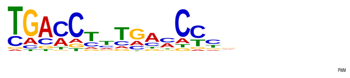	NNNNNNTTTGNNNCCGCNNNNNN	4.02	M	15

132 THRB-Yin2017-Rnd4 (ERR1006600)

PWM	Seed Sequence	Seed Seq Enrichment	Repeat	Rank
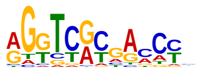	NNNNNNAGGTNNNNACCCNNNNNN	7.52	IR:0	1
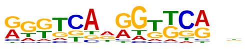	NNNNNNTCAANNTTCANNNNNN	7.08	DR:1	2
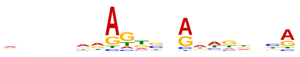	NNNNNNGAAANNNNAAAGNNNNNN	6.60	M	3
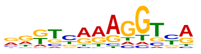	NNNNNNGGTCNNNNGTTANNNNNN	4.88	DR:1	4
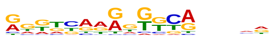	NNNNNNTCAANNNNCAAGNNNNNN	5.41	M	5
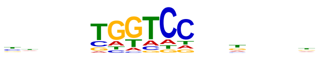	NNNNNNTTGGTCCGNNNNNN	6.65	M	6
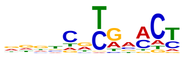	NNNNNNGTCGNNNNCTTTNNNNNN	4.63	M	7
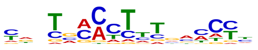	NNNNNNCGTTNNNNTTTGNNNNNN	6.05	M	8
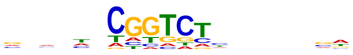	NNNNNNCGGTCTCANNNNNN	4.69	M	9

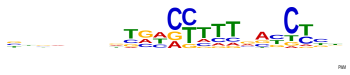	NNNNNNTCTTNNNNTTTANNNNNN	3.55	M	10
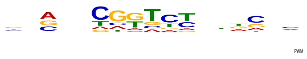	NNNNNNGACGGTCTNNNNNN	3.54	M	11
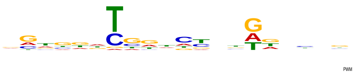	NNNNNNCGGTCCGGNNNNNN	2.75	M	12
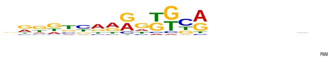	NNNNNNTCAANNNNCAGANNNNNN	4.16	M	13
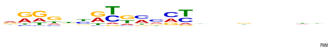	NNNNNNTCATNNNNTTTTNNNNNN	3.32	M	14
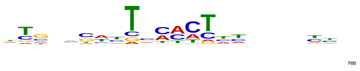	NNNNNNTTTCNNNTTTGNNNNNN	5.14	M	15
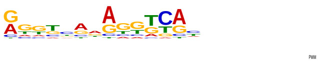	NNNNNNTCAANNNNNNATGGNNNNNN	3.65	M	16
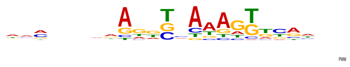	NNNNNNAAAANNNNNAAGTNNNNNN	4.33	M	17
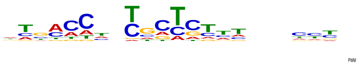	NNNNNNTTCGNNNTTTCNNNNNN	3.09	M	18

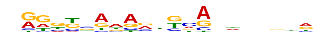 PWM	*NNNNNNCAAANNNNAAATNNNNNN*	4.19	M	19
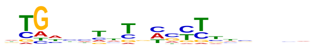 PWM	*NNNNNNTTTGNNNNTTCTNNNNNN*	3.04	M	20

133 THRB-Yin2017-Rnd4 (ERR1006601)

PWM	Seed Sequence	Seed Seq Enrichment	Repeat	Rank
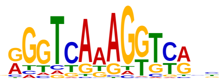	NNNNNNGGGTNNNNNGTCANNNNNN	11.16	DR:1	1
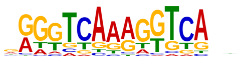	NNNNNNTCAANNNNCAATNNNNNN	9.92	M	2
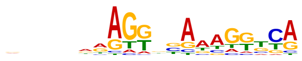	NNNNNNGAAANNNNAAAGNNNNNN	6.81	M	3
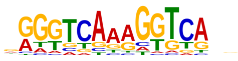	NNNNNNTCAANNNNCACANNNNNN	7.77	M	4
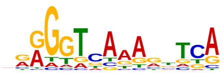	NNNNNNCAAANNNNAAATNNNNNN	5.42	M	5
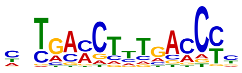	NNNNNNGCGTNNNNTTTGNNNNNN	6.09	M	6
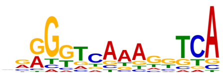	NNNNNNCAAANNNNATCANNNNNN	5.13	M	7
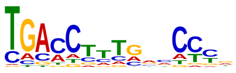	NNNNNNTTTGNNNCCGCNNNNNN	4.45	M	8
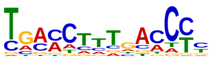	NNNNNNCTGTNNNNTTTGNNNNNN	5.14	M	9

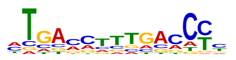	*NNNNNNGGCTNNNNTTTGNNNNNN*	3.58	M	10
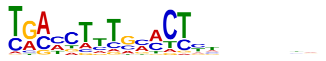	*NNNNNNCTTTNNNNTTTGTNNNNNN*	6.41	M	11
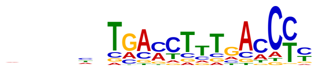	*NNNNNNGATTNNNNTTTGNNNNNN*	5.14	M	12
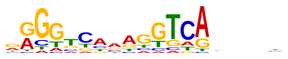	*NNNNNNTCAANNNNNCGTCNNNNNN*	3.17	DR:4	13
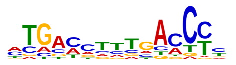	*NNNNNNGGATNNNNTTTGNNNNNN*	3.67	M	14
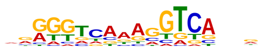	*NNNNNNTCAANNNNNNAACANNNNNN*	3.89	M	15
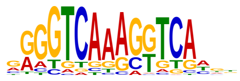	*NNNNNNTCAANNNNNCAAGTCNNNNNN*	16.53	DR:6	16
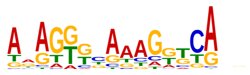	*NNNNNNGTAAANNNNAAAGNNNNNN*	6.75	M	17
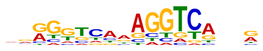	*NNNNNNTCAANNNNNNACCANNNNNN*	3.03	IR:4	18

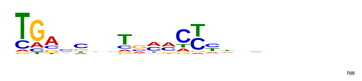	*NNNNNNTTTGNNNNNTTGCNNNNNN*	3.08	M	19
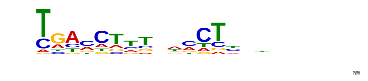	*NNNNNNCTTTNNNNNTTCTNNNNNN*	3.72	M	20

134 THRB-Yin2017-Rnd2 (ERR1006604)

PWM	Seed Sequence	Seed Seq Enrichment	Repeat	Rank
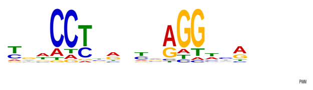	NNNNNNACCTNNNNNAGGTNNNNNN	14.74	ER:5	1
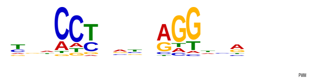	NNNNNNACCTNNNNAGGTNNNNNN	13.75	ER:4	2
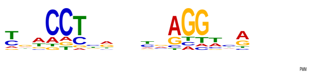	NNNNNNACCTNNNNNNAGGTNNNNNN	9.46	ER:6	3
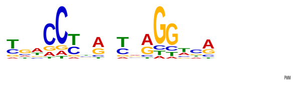	NNNNNNTGACNNNNNNNNNGTCANNNNNN	10.93	ER:5	4
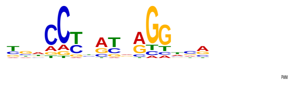	NNNNNNTGACNNNNNNNNGTCANNNNNN	9.94	ER:4	5
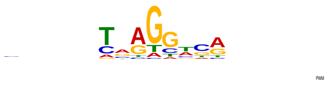	NNNNNNTTTANNGTCANNNNNN	6.20	M	6
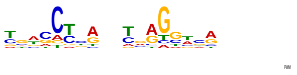	NNNNNNTGACNNNNNNNNNNGTCANNNNNN	6.82	ER:6	7
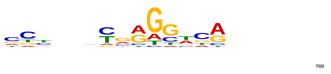	NNNNNNAACGNNGTCANNNNNN	6.74	M	8
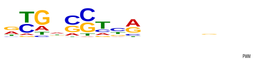	NNNNNNCGCGNNNTCATNNNNNN	4.90	M	9

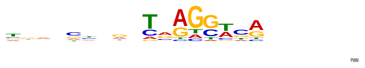	NNNNNNCGCTNNNGTCANNNNNNN	5.47	M	10
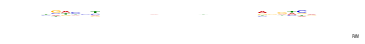	NNNNNNACCTNNNNNNNNNNNNNNNNAGGTNNNNNN	4.05	ER:16	11
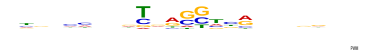	NNNNNNTCGTNNNNTCATNNNNNN	4.62	M	12
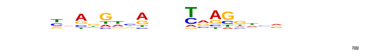	NNNNNNAGGTNNNNNNNNNGTCANNNNNNN	4.28	DR:5	13
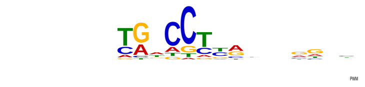	NNNNNNTTGTNNTCATNNNNNN	4.94	M	14
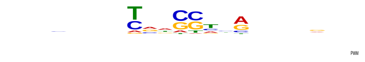	NNNNNNATTGNNNTCATNNNNNN	4.57	M	15
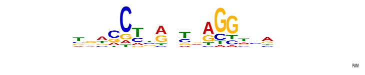	NNNNNNTGACNNNNNNNNNNNTCAANNNNNNN	4.98	ER:5	16
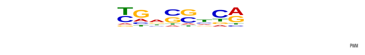	NNNNNNACGANNTCGTNNNNNN	4.63	M	17
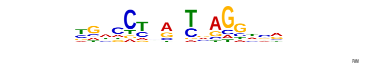	NNNNNNCGTCNNNNNNNNNGTCANNNNNNN	4.17	DR:6	18

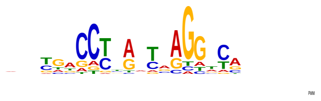	NNNNNNA$\overleftarrow{TGACNN}$NNNNN$\overrightarrow{NNGACA}$NNNNNN	8.99	ER:5	19
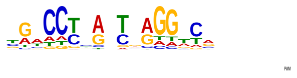	NNNNN$\overrightarrow{NTGTC}$NNNNNNN$\overrightarrow{NNGACA}$NNNNNN	4.77	DR:6	20

135 THRB-Yin2017-Rnd2 (ERR1006605)

PWM	Seed Sequence	Seed Seq Enrichment	Repeat	Rank
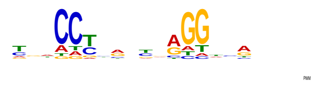	NNNNNNACCTNNNNNAGGTNNNNNN	9.43	ER:5	1
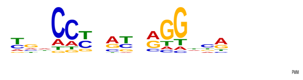	NNNNNNGACCNNNNNNGGTCNNNNNN	8.96	ER:4	2
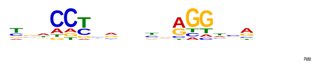	NNNNNNACCTNNNNNNAGGTNNNNNN	6.00	ER:6	3
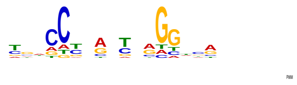	NNNNNNTGACNNNNNNNNNGTCANNNNNN	7.52	ER:5	4
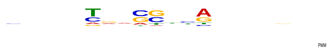	NNNNNNATGANNTCATNNNNNN	5.32	M	5
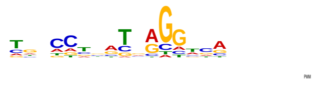	NNNNNNGCCTNNNNNNGTCANNNNNN	4.61	ER:4	6
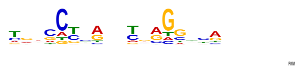	NNNNNNTGACNNNNNNNNNNGTCANNNNNNN	5.21	ER:6	7
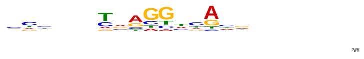	NNNNNNTAAGNNNACGTNNNNNN	4.30	M	8
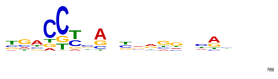	NNNNNNATGANNNNNNNNAGGCNNNNNN	2.80	ER:5	9

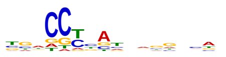	*NNNNNNATGANNNNNNNNAGGCNNNNNN*	2.52	ER:4	10
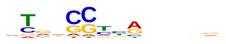	*NNNNNNCGTGNNNNTCATNNNNNN*	4.73	M	11
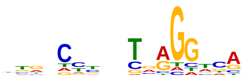	*NNNNNNCCCGNNNNNNGTCANNNNNN*	5.88	M	12
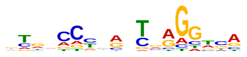	*NNNNNNGCCTNNNNNNNGTCANNNNNN*	4.63	ER:5	13
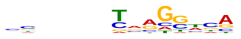	*NNNNNNATTTNNNGTCANNNNNN*	3.88	M	14
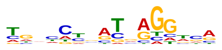	*NNNNNNGGCCNNNNNNNGTCANNNNNN*	4.28	DR:4	15
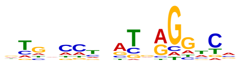	*NNNNNNGCCTNNNNNNGACANNNNNN*	2.94	ER:4	16
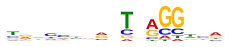	*NNNNNNGCCTNNNNNNNNTCAANNNNNN*	2.11	ER:5	17
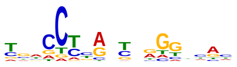	*NNNNNNGTGACNNNNNNNAGGCNNNNNN*	4.69	ER:5	18

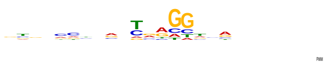	$NNNN\overleftarrow{NNGCCT}NNNNN\overrightarrow{NNNTCAC}NNNNNN$	2.32	ER:5	19
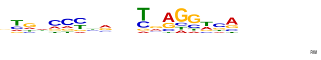	$NNNN\overleftarrow{NNGCCT}NNNNNN\overrightarrow{NNGTCA}NNNNNN$	3.18	ER:6	20

136 THRB-Yin2017-Rnd3 (ERR1006606)

PWM	Seed Sequence	Seed Seq Enrichment	Repeat	Rank
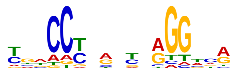	NNNNNNGACCNNNNNNGGTCNNNNNN	10.26	ER:5	1
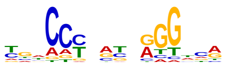	NNNNNNGACCNNNNNGGTCNNNNNN	9.02	ER:4	2
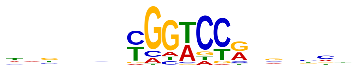	NNNNNNATGGTCCANNNNNN	8.10	M	3
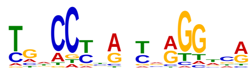	NNNNNNCTTANNNNNGTCANNNNNN	8.34	M	4
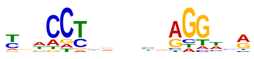	NNNNNNACCTNNNNNNAGGTNNNNNN	5.52	ER:6	5
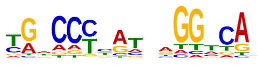	NNNNNNTGTCNNNATAANNNNNN	6.53	M	6
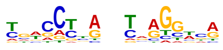	NNNNNNTGACNNNNNNNNNNGTCANNNNNN	5.07	ER:6	7
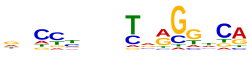	NNNNNNAATANNNTCATNNNNNN	5.20	M	8
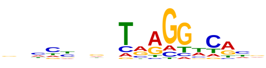	NNNNNNGCTANNNNCACGNNNNNN	5.01	M	9

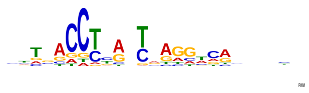	$NNNN\overrightarrow{NNTTCA}NNNNNNNNN\overrightarrow{NNGTCA}NNNNNNN$	3.80	DR:8	10
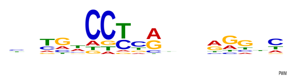	NNNNNNGTGTNNNCGTTNNNNNN	3.95	M	11
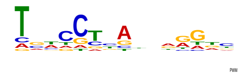	NNNNNNTGTTNNNNTTATNNNNNN	4.14	M	12
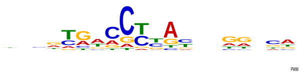	NNNNNNCGTGNNNNTATGNNNNNN	4.98	M	13
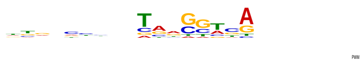	$NNNNNNCGATN\overrightarrow{NNNTCA}ANNNNNN$	4.74	M	14
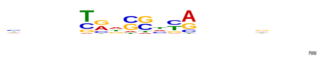	NNNNNNGATGNNNNCATCNNNNNN	4.05	M	15
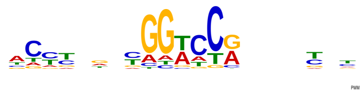	$NNNNNNT\overrightarrow{CGGTCC}ACNNNNNN$	9.55	M	16
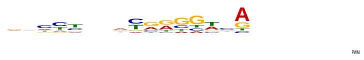	$NNNNNNAACG\overrightarrow{NNNTCA}ANNNNNN$	3.86	M	17
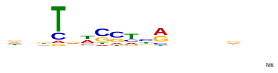	$NNNNNNGCTTN\overrightarrow{NNNTCA}TNNNNNN$	3.88	M	18

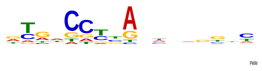	*NNNNNNCATGNNNNTAATNNNNNN*	4.18	M	19
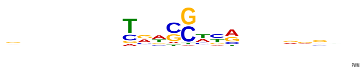	*NNNNNNATTGNNNNCATTNNNNNN*	3.94	M	20

137 THRB-Yin2017-Rnd3 (ERR1006607)

PWM	Seed Sequence	Seed Seq Enrichment	Repeat	Rank
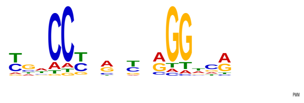	NNNNNNGACCNNNNNNNGGTCNNNNNN	10.86	ER:5	1
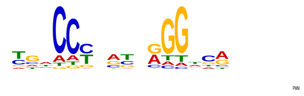	NNNNNNGACCNNNNNGGTCNNNNNN	9.32	ER:4	2
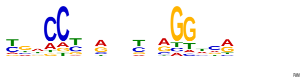	NNNNNNGACCNNNNNNNNGGTCNNNNNN	5.32	ER:6	3
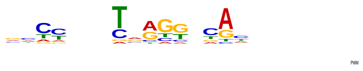	NNNNNNATAANNNCACGNNNNNN	5.00	M	4
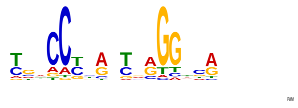	NNNNNNTGACNNNNNNNNNGTCANNNNNN	8.62	ER:5	5
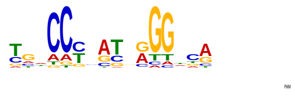	NNNNNNTGACNNNNNNNNGTCANNNNNN	8.03	ER:4	6
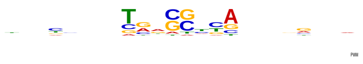	NNNNNNGATGNNNNCATCNNNNNN	4.15	M	7
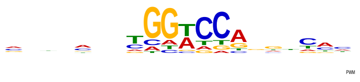	NNNNNNCTGGTCCANNNNNN	6.20	M	8
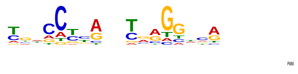	NNNNNNTGACNNNNNNNNNNGTCANNNNNN	4.28	ER:6	9

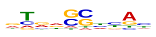	*NNNNNNCGTGNNNNNCACGNNNNNN*	4.33	M	10
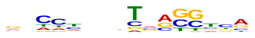	*NNNNNNATTCNNNNTCATNNNNNN*	3.67	M	11
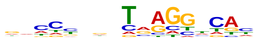	*NNNNNNGCTANNNNNCACGNNNNNN*	4.31	M	12
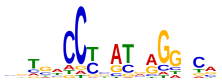	*NNNNNNCGTGNNNNNNNNNNNGACANNNNNN*	4.02	M	13
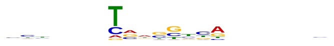	*NNNNNNATTTNNNNNTCATNNNNNN*	3.45	M	14
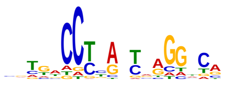	*NNNNNNCGTGNNNNNNNNNNNNGACANNNNNN*	4.43	M	15
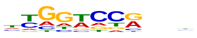	*NNNNNNCGGTCCATNNNNNN*	3.77	M	16
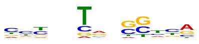	*NNNNNNTGGTNNNNNTCATNNNNNN*	3.36	M	17
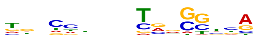	*NNNNNNGAATNNNNNTCATNNNNNN*	3.54	M	18

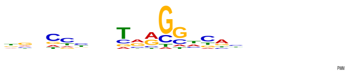	NNNNNNTGGTNNNNNCACGNNNNNN	3.14	M	19
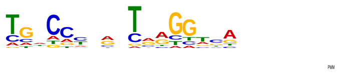	NNNNNNCTCGNNNNNTCAANNNNNN	3.39	M	20

138 THRB-Yin2017-Rnd4 (ERR1006608)

PWM	Seed Sequence	Seed Seq Enrichment	Repeat	Rank
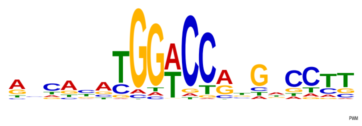	NNNNNNTGGTCCATNNNNNN	22.76	M	1
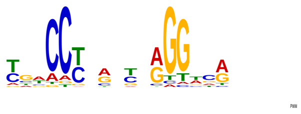	NNNNNNGACCNNNNNNNGGTCNNNNNN	10.32	ER:5	2
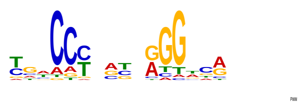	NNNNNNGACCNNNNNNGGTCNNNNNN	8.06	ER:4	3
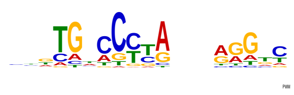	NNNNNNTGTGNNNNTATTNNNNNN	8.84	M	4
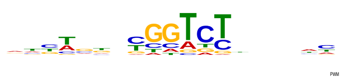	NNNNNNTTCGGTCTNNNNNN	5.43	M	5
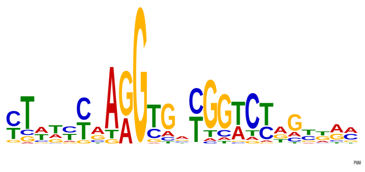	NNNNNNAAGGNNNCGGTCTNNNNNN	27.73	DR:0	6
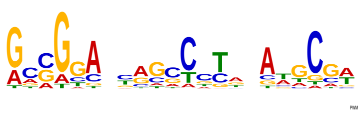	NNNNNNATAGNNCTAGNNNNNN	3.80	M	7
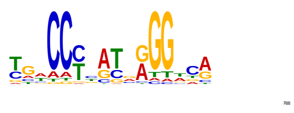	NNNNNNTGACNNNNNNNNGTCANNNNNN	7.66	ER:4	8
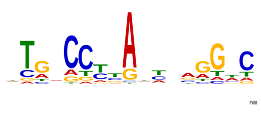	NNNNNNGATGNNNNTAATNNNNNN	6.13	M	9

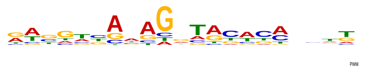	NNNNNNAAAGNTACANNNNNN	5.78	M	10
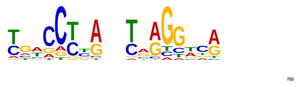	NNNNNNTGACNNNNNNNNNNGTCANNNNNN	4.62	ER:6	11
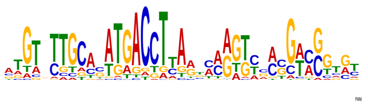	ATGTNNNNNNNNNNNNNNNNNNNNNNNNNNNNNNNGTGT	3.75	M	12
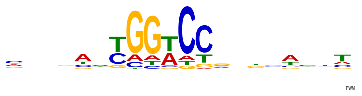	NNNNNNTGGTCCGANNNNNN	10.66	M	13
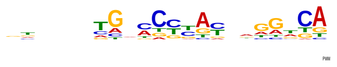	NNNNNNGATGNNNNTATGNNNNNN	4.25	M	14
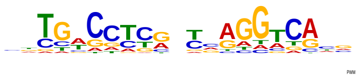	NNNNNNCTCGNCCAGNNNNNN	4.98	M	15
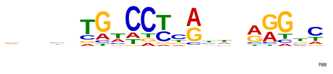	NNNNNNGTGTNNNCGTTNNNNNN	5.17	M	16
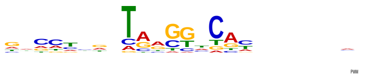	NNNNNNGCTANNNNCATGNNNNNN	5.45	M	17
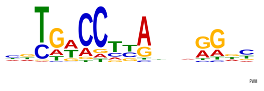	NNNNNNGCGTNNNNTTATNNNNNN	5.31	M	18

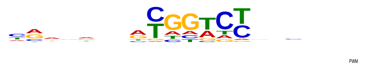	*NNNNNNCACGGTCTNNNNNN*	6.86	M	19
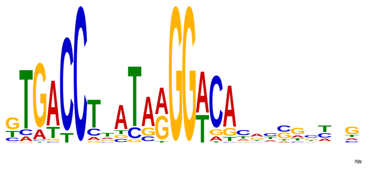	*NNNNNNTTATANNNNCACACNNNNNN*	17.31	M	20

139 THRB-Yin2017-Rnd4 (ERR1006609)

PWM	Seed Sequence	Seed Seq Enrichment	Repeat	Rank
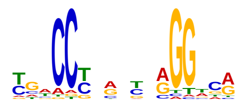	NNNNNNGACCNNNNNNNGGTCNNNNNN	13.70	ER:5	1
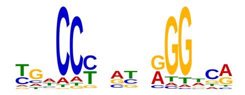	NNNNNNGACCNNNNNNGGTCNNNNNN	10.02	ER:4	2
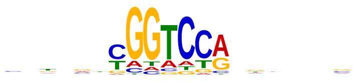	NNNNNNCGGTCCACNNNNNN	11.72	M	3
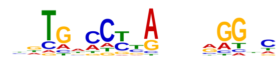	NNNNNNCGTGNNNNTATTNNNNNN	6.26	M	4
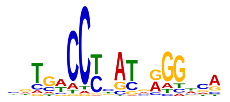	NNNNNNCGTGNNNNNNNNNNGTCANNNNNN	5.17	M	5
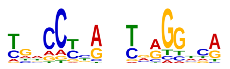	NNNNNNTGACNNNNNNNNNNGTCANNNNNN	4.94	ER:6	6
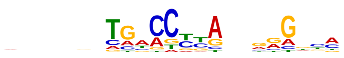	NNNNNNGCTGNNNNTATCNNNNNN	4.06	M	7
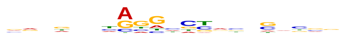	NNNNNNCTAGNNCTACNNNNNN	3.11	M	8
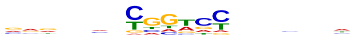	NNNNNNACGGTCTGNNNNNN	3.97	M	9

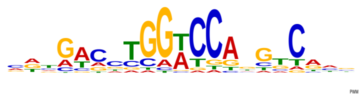	$NNNNNNC\overrightarrow{TGGTCC}AGNNNNNN$	16.14	M	10
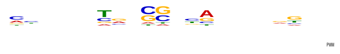	$NNNNNNAATGNNNNCATTNNNNNN$	3.88	M	11
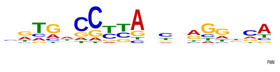	$NNNNNNCGTGNNNNNAGCGNNNNNN$	4.26	M	12
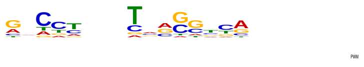	$NNNNNNATTC\overrightarrow{NNNNTCA}TNNNNNN$	3.94	M	13
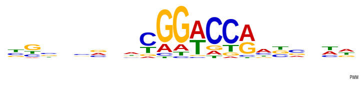	$NNNNNNGA\overrightarrow{CGGTCC}GNNNNNN$	8.58	M	14
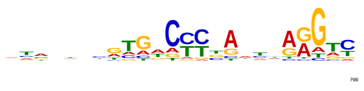	$NNNNNNCGTGNNNNGAATNNNNNN$	4.53	M	15
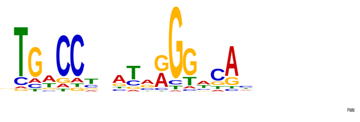	$NNNNNNTTATNNNNNCACGNNNNNN$	6.31	M	16
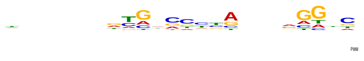	$NNNNNNCATGNNNNTACTNNNNNN$	3.63	M	17
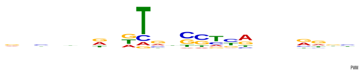	$NNNN\overleftarrow{NNACTT}\overrightarrow{NNNNTCA}TNNNNNN$	3.62	ER:1	18

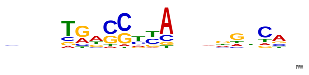	$NNNNNNGATGNNN\overrightarrow{NNATC}ANNNNNN$	4.04	M	19
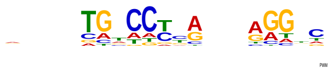	$NNNNNNGTGTNNNCATTNNNNNN$	4.97	M	20

140 THRB-7-Bhimsaria2022-Rnd3 (S07-103)

PWM	Seed Sequence	Seed Seq Enrichment	Repeat	Rank
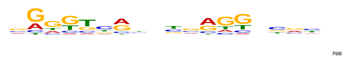	NNNGGTCNNNNNNGGTCNNN	7.28	DR:4	1
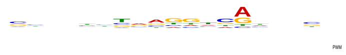	NNNNNNTTAAGGTCNNNNNN	5.24	M	2
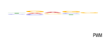	GGGGNNNNNNNNNNNGAGG	4.96	M	3
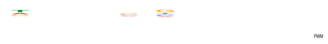	ATTTNNNNNNNNNNNTCTT	3.93	M	4
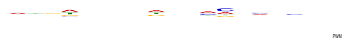	CATGNNNNNNNNNNNNCCGT	3.86	M	5
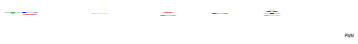	TCCCNNNNNNNNNNNNCACT	4.08	M	6
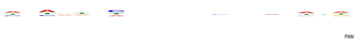	NCAGTNNNNNNNNNNATATN	3.98	M	7
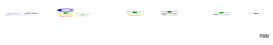	AAGTNNNNNNNNNNNNTTGT	3.85	M	8
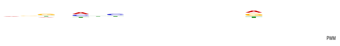	CAGGNNNNNNNNNNNNTCTT	3.90	M	9

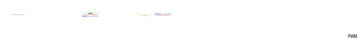	*AGTGNNNNNNNNNNNNAATT*	3.89	M	10
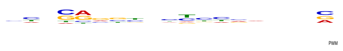	$\overleftarrow{ACCT}NNNNNNNNNNN\overrightarrow{NGGAC}$	3.12	ER:11	11
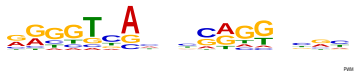	$N\overrightarrow{NGGTC}AANNNN\overrightarrow{NGGTC}NN$	15.37	DR:4	12
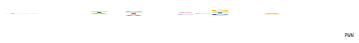	$N\overrightarrow{AAGGN}NNNNNNNNNCCATN$	3.42	M	13
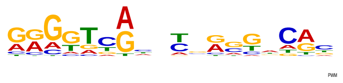	$NN\overrightarrow{AGGTC}NNNNN\overrightarrow{GGGAC}NN$	12.10	DR:4	14
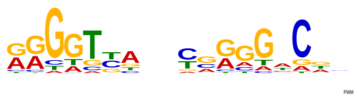	$NN\overrightarrow{GGTTA}NNNN\overrightarrow{GGGAC}NN$	13.35	DR:4	15
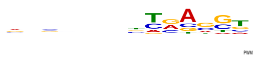	$GGGGNNNNNNNNNNNNNA\overrightarrow{AGGT}$	7.11	M	16

141 RARA:RXRA-Bhimsaria2022-Rnd3 (S13-149)

PWM	Seed Sequence	Seed Seq Enrichment	Repeat	Rank
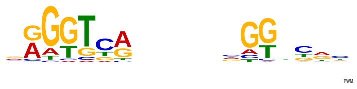	NNGGTCNNNNNNNGGTCNN	41.26	DR:5	1
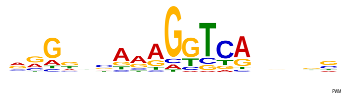	NNNNNNCAAAGGTCNNNNNN	24.59	M	2
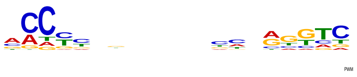	ACCTNNNNNNNNNNNNGGTC	28.13	ER:11	3
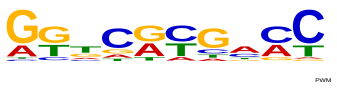	GGTCATGACC	93.53	IR:0	4
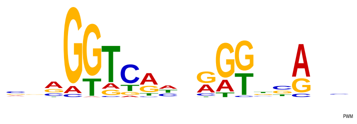	NNNGGTCACNNGGTCNNN	47.95	DR:2	5
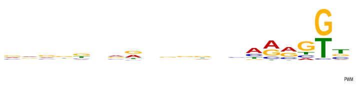	GGGGNNNNNNNNNNNNNAGGT	14.37	M	6
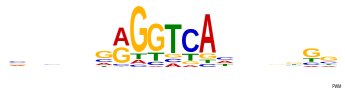	NNNNNNGGGGTCAANNNNNN	26.78	M	7
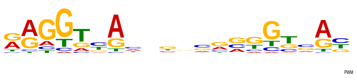	NNGGTCNNNNNNNNGGTCNN	12.80	DR:6	8
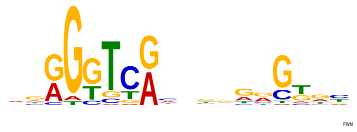	NGGGGTCNNNNNGAGTGN	106.20	M	9

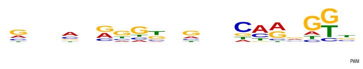	GTCANNNNNNNNNNNNAGGT	14.63	DR:12	10
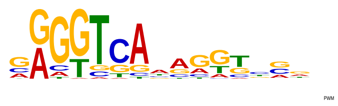	NNNGGTCAAAGGGCNNN	93.02	DR:1	11
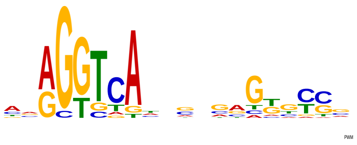	NNNGGTCATNNNNAGTGNNN	78.38	M	12
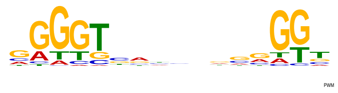	CGGGGTNNNNNNGGGGT	128.62	DR:5	13
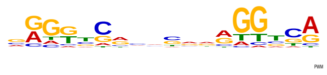	NCGGGNNNNNNNNNNGGTCN	14.93	M	14
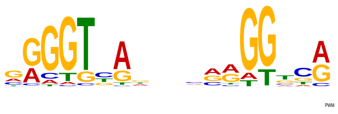	NGGGGTTNNNNNNNGGTCN	71.37	DR:5	15
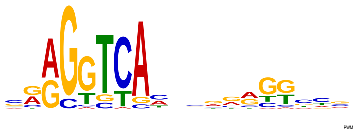	NNNGGTCACNNNAGGTNNN	57.67	DR:4	16
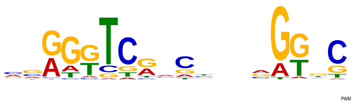	NGGGGTCNNNNNNAGTTN	44.66	DR:5	17
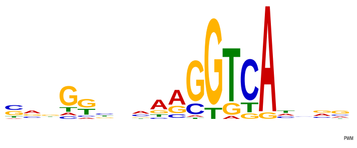	NNNNNCTAAAGGTCNNNNN	13.10	M	18

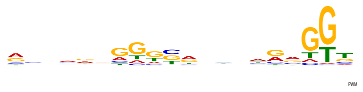	$ACGANNNNNNNNNNNN\overrightarrow{AGGT}$	14.08	M	19
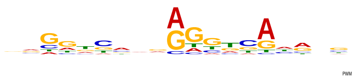	$NNNNNCAAG\overrightarrow{NGGTC}NNNNN$	10.36	M	20

142 RARA-RXRA-SMiLE-seq-Rnd1 (SRR3405127)

PWM	Seed Sequence	Seed Seq Enrichment	Repeat	Rank
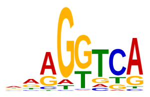	*NNNNNNAAAGGTCANNNNNN*	29.47	M	1
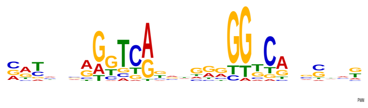	*NNNNNNAGGTCNNNNNNNGGTCANNNNNN*	90.01	DR:5	2
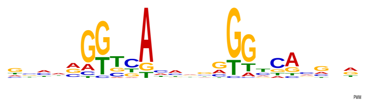	*NNNNAGGTCANNNNNNGGTCANNNNN*	97.37	DR:4	3
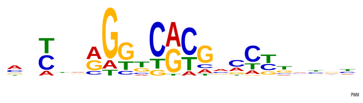	*NNNNNAGGTCANGACCTNNNNN*	81.80	IR:0	4
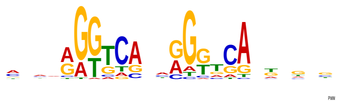	*NNNNNNAGGTCANNNGGTCANNNNNN*	130.09	DR:2	5
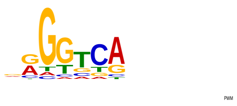	*NNNNNNCGGGGTCANNNNNN*	12.33	M	6
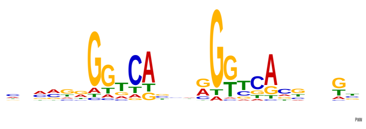	*NNNNNNGGTCANNNNGGTCANNNNNN*	53.23	DR:3	7
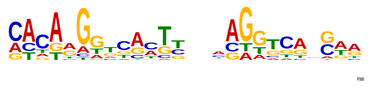	*NNNNAGGTCNNNNNNNNGGTCANNNNN*	31.31	DR:6	8
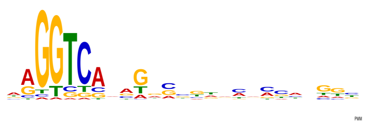	*NAGGTCANNNNNNNNNNNNNAGGGN*	31.11	DR:13	9

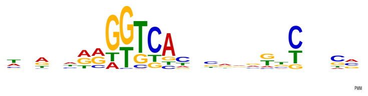	$NNNNNN\overrightarrow{AGGTCA}NNNA\overrightarrow{AGGNNN}NNN$	31.07	DR:4	10
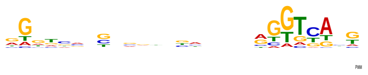	$\overrightarrow{NNGTCA}NNNNNNNNNNNNN\overrightarrow{NGGTCA}NN$	14.64	DR:13	11
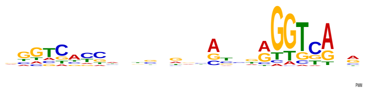	$\overrightarrow{NNGTCA}NNNNNNNNNNNNNN\overrightarrow{NGGTCA}NN$	20.87	DR:14	12
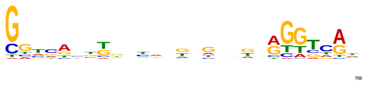	$\overrightarrow{NGTCA}NNNNNNNNNNNNNNN\overrightarrow{NGGTCA}N$	14.16	DR:15	13
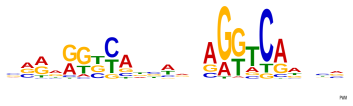	$NNNN\overrightarrow{AGGTNN}NNNNN\overrightarrow{AGTTCA}NNNN$	28.69	DR:5	14
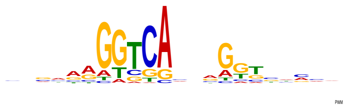	$NNNNNN\overrightarrow{AGGTCA}NA\overrightarrow{AGGNNN}NNN$	45.29	DR:2	15
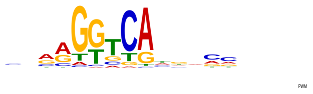	$NNNNNN\overrightarrow{AGGTCA}\overleftarrow{TGANNN}NNN$	22.59	IR:0	16
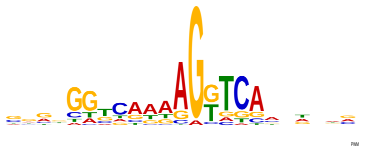	$NNNN\overrightarrow{NNTCAA}N\overrightarrow{AGTTCA}NNNNNN$	34.10	DR:2	17
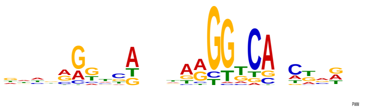	$NNNN\overrightarrow{NNGTCA}NNNN\overrightarrow{AGGTCA}NNNNNN$	42.06	DR:4	18

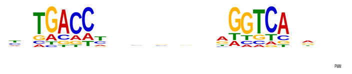	$NNNN\overleftarrow{TGACNN}NNNNNNNNN\overrightarrow{NNGTCA}NNNN$	11.56	ER:9	19
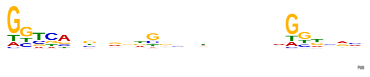	$\overrightarrow{NGTCA}NNNNNNNNNNNNNNNN\overrightarrow{NNGTCA}N$	6.20	DR:16	20

143 RARA-RXRG-SMiLE-seq-Rnd1 (SRR3405128)

PWM	Seed Sequence	Seed Seq Enrichment	Repeat	Rank
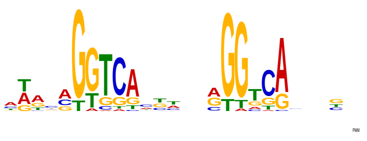	NNNNNGGTCANNNNNNGGTCANNNNN	130.22	DR:5	1
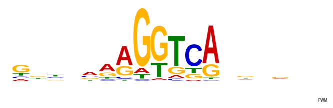	NNNNNNAAAGGTCANNNNNN	22.72	M	2
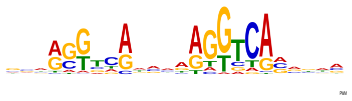	NNNNNGGTCNNNNNNGGTCANNNNN	23.39	DR:4	3
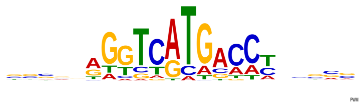	NNNNNNGGTCANGACCNNNNNN	26.55	IR:0	4
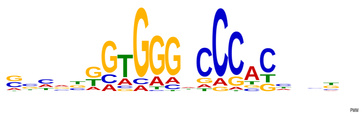	NNNNNNGTGGGNCCCANNNNNN	16.15	M	5
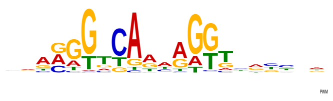	NNNNNNGGTCANNAGGTNNNNNN	23.56	DR:2	6
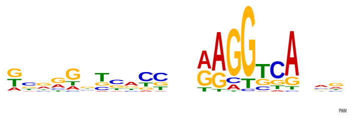	NNNGGGGNNNNNNNNNGGTCANNN	19.09	M	7
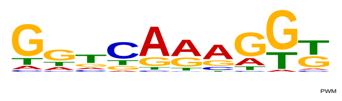	GGTCAAAGGT	37.39	DR:1	8
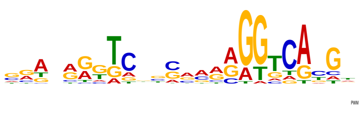	NNNAAGGNNNNNNNNNGGTCANNN	16.35	DR:5	9

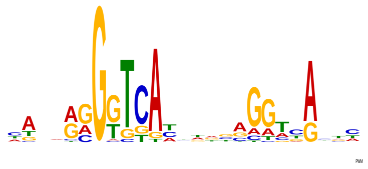	NNNNNGGGTCANNNNNNAGGTNNNNN	59.04	DR:5	10
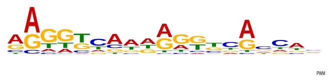	NNNNGGTCANNNNGTCANNNN	14.25	DR:2	11
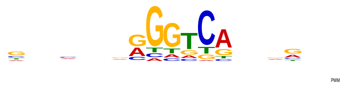	NNNNNNTGGGGTCANNNNNN	9.34	M	12
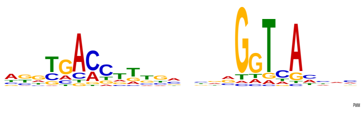	NNNNACCTNNNNNNNNGGTCNNNNN	6.94	ER:7	13
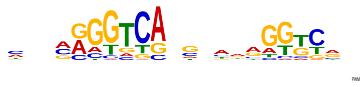	NNNNNGGTCANNNAAAGNNNNN	16.82	M	14
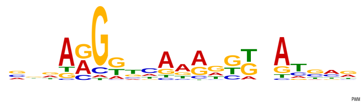	NNNNNGGTCANNGGTCNNNNN	12.22	DR:1	15
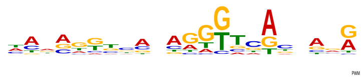	NNNNNGTCANNNGGTCANNNNN	14.94	DR:2	16
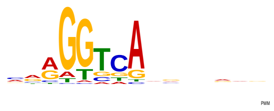	NNNNNNGAGGTCAANNNNNN	12.04	M	17
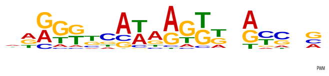	NNNNNGGTCANNNGGTCNNNNN	13.44	DR:2	18

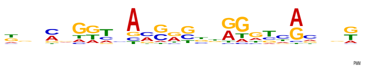	$NNNN\overrightarrow{NGGTC}NNNNNNN\overrightarrow{NGGTC}NNNNN$	5.46	DR:6	19
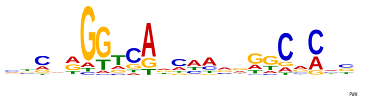	$NNNN\overrightarrow{NGGTCA}NNNN\overrightarrow{GGGTN}NNNN$	12.70	DR:4	20

144 RARA-Bhimsaria2022-Rnd2 (S07-031)

PWM	Seed Sequence	Seed Seq Enrichment	Repeat	Rank
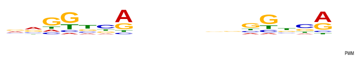	NNGGTCNNNNNNNGGTCNN	3.97	DR:5	1
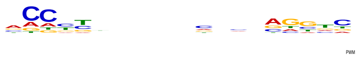	ACCTNNNNNNNNNNNNGGTC	5.04	ER:11	2
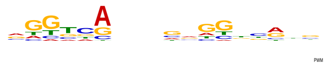	NNNGGTCNNNNNNGGTCNNN	3.71	DR:4	3
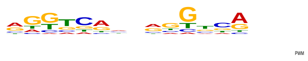	NNNNGGTCNNNNGGTCNNNN	4.68	DR:2	4
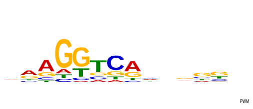	NNNNNNAAAGGTCANNNNNN	6.68	M	5
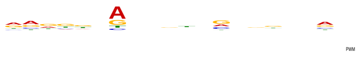	NNGGTCNNNNNNNNGGTCNN	2.04	DR:6	6
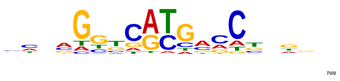	NNNNNGGTCATGACCNNNNN	16.64	IR:0	7
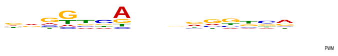	NNNGGTCNNNNNGGTCNNN	3.30	DR:3	8
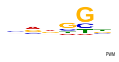	GGGGNNNNNNNNNNNNAGGT	3.34	M	9

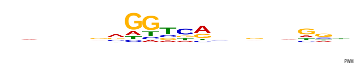	*NNNNNNGGGTCACGNNNNNN*	4.55	M	10
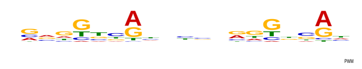	*NNGGGTCNNNNNNNGGTCNN*	4.46	DR:5	11
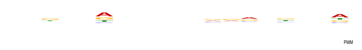	*NGGTCNNNNNNNNNGGTCN*	1.74	DR:7	12
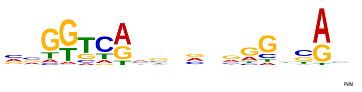	*NNGGTCACNNNNNGGTCNN*	8.83	DR:5	13
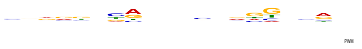	*NNGGGTNNNNNNNNGGTCNN*	2.47	DR:5	14
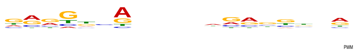	*NGGGTCNNNNNNNNGGTCN*	3.01	DR:6	15
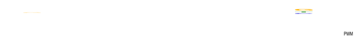	*GGTCNNNNNNNNNNNGGTC*	1.82	DR:9	16
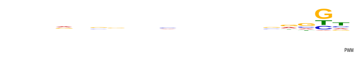	*CCAANNNNNNNNNNNNNGGGT*	2.07	M	17
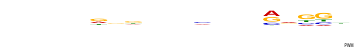	*CGTANNNNNNNNNNNNNAGGT*	2.22	M	18

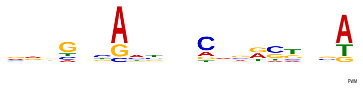	$N\overrightarrow{AGGTCN}NNNNNNN\overrightarrow{NGGTCN}$	2.41	DR:7	19
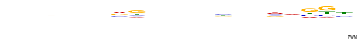	$\overrightarrow{ATGTNN}NNNNNNNNNN\overrightarrow{AGGT}$	2.39	DR:10	20

145 RARA-Bhimsaria2022-Rnd3 (S07-151)

PWM	Seed Sequence	Seed Seq Enrichment	Repeat	Rank
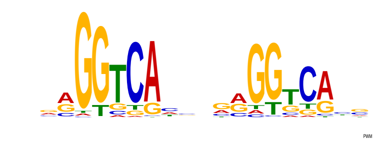	NNNGGTCANNNNNGGTCNNN	194.12	DR:4	1
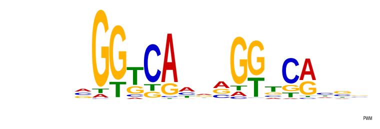	NNNNGGTCAANNGGTCNNNN	365.67	DR:2	2
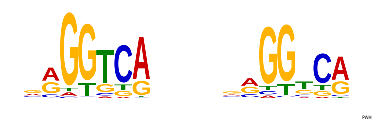	NNGGTCNNNNNNNGGTCNN	74.67	DR:5	3
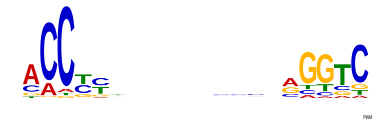	ACCTNNNNNNNNNNNNGGTC	116.87	ER:11	4
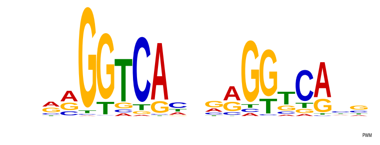	NNNGGTCACNNNGGTCNNN	307.93	DR:3	5
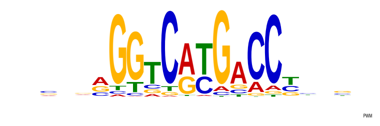	NNNNNGGTCATGACCNNNNN	627.56	IR:0	6
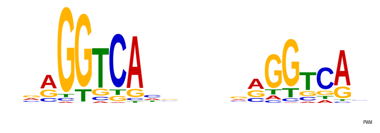	NNGGTCANNNNNNNGGTCNN	82.39	DR:6	7
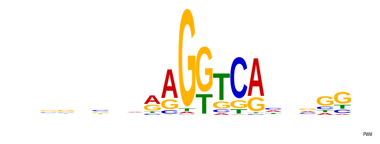	NNNNNNAAAGGTCANNNNNN	94.40	M	8
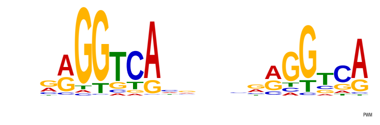	NGGGGTCNNNNNNNNGGTCN	107.75	DR:6	9

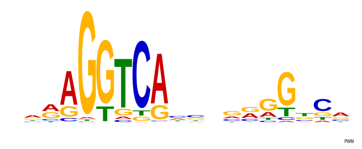	NNAAGGTCNNNNNGGGTNN	134.32	DR:4	10
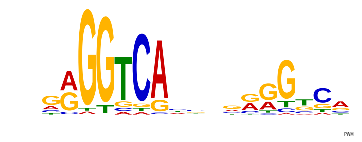	NNGGGGTCNNNNNGGGTNN	115.10	DR:4	11
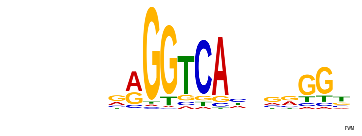	NNNNNNGGGGTCAANNNNNN	50.85	M	12
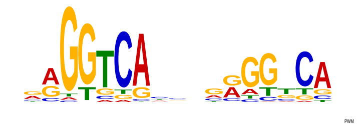	NNNGGTCNNNNGGGGTNNN	74.22	DR:4	13
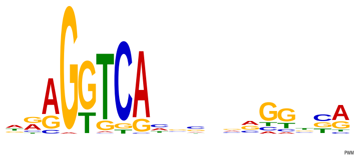	NAAGGTCACNNNNNGGGCN	472.98	DR:5	14
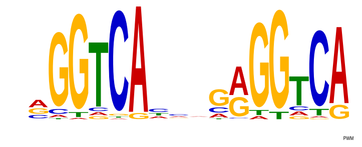	NCGGTCACNNGGGGTCN	766.87	DR:4	15
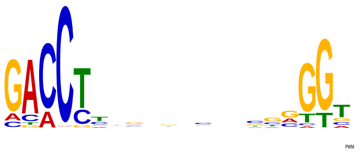	GACCTTTNNNNNNNNGGGGT	1259.73	ER:11	16
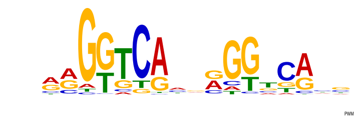	NNNAGGTCNNGGGGTCNNN	201.73	DR:2	17
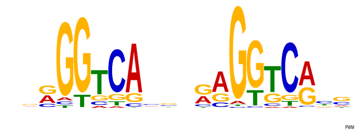	NNNGGTCNNNNAAGGTCNNN	187.95	DR:4	18

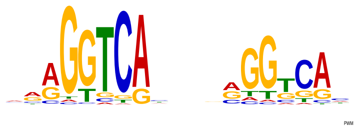	$NN\overrightarrow{AGGTCN}NNNN\overrightarrow{CGGTCN}N$	99.75	DR:4	19
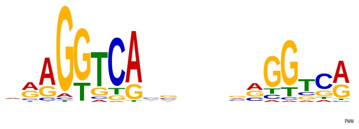	$NA\overrightarrow{AGGTCN}NNNNNN\overrightarrow{NGGTCN}$	172.22	DR:6	20

146 RARA-Jolma2013-Rnd2 (ERR193727)

PWM	Seed Sequence	Seed Seq Enrichment	Repeat	Rank
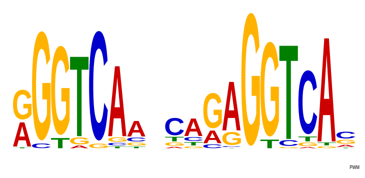	NGGTCANNNNNAGGTCAN	135.72	DR:5	1
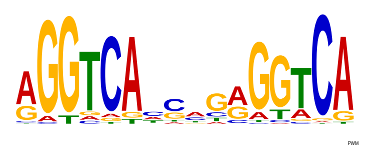	AGGTCNNNNNNGGTCA	35.41	DR:4	2
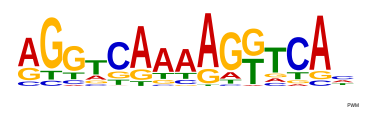	NGGTCANNGGTCAN	11.02	DR:2	3
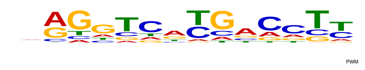	NNGGTCANGACCNN	4.30	IR:0	4
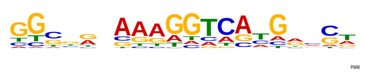	NNNNNNAAAGGTCANNNNNN	5.81	M	5
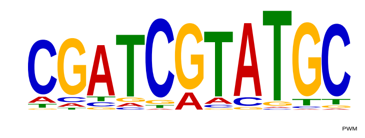	CGATCNTATGC	15.39	M	6
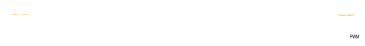	NNNGGGGNNNNNGGGGNNN	2.34	M	7
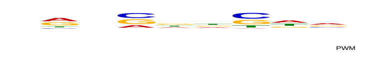	GAGGTCAA	1.77	M	8
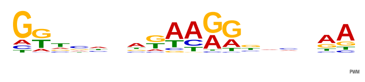	NNTCATNNNNNNGTCANN	1.89	DR:5	9

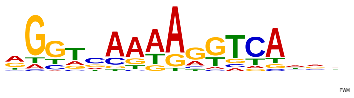	$\overrightarrow{NNNTCA}AN\overrightarrow{NGGTCA}NNN$	6.19	DR:2	10
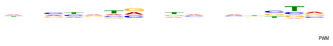	*NNNCTATNNNAAATNNN*	1.77	M	11
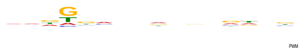	*TGTANNNNNNNNNNNGTGG*	1.80	M	12
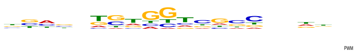	$NNNNNNG\overrightarrow{TGGTCG}CNNNNNN$	2.64	M	13
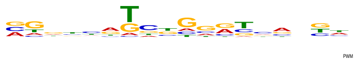	$\overrightarrow{NNGTCA}NNNN\overrightarrow{NNNTCA}GNN$	1.77	DR:3	14
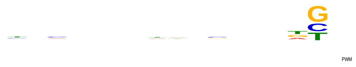	$NN\overrightarrow{NTTCA}N\overrightarrow{NNGTAA}NNN$	1.23	DR:1	15
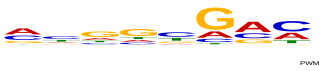	$\overrightarrow{ACGGCG}AC$	2.70	M	16
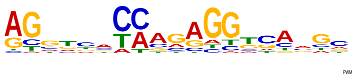	$\overrightarrow{NNGTCA}NNNN\overrightarrow{NNNTCA}ANN$	2.69	DR:4	17
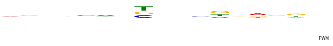	$N\overrightarrow{AGTTNN}NNNNNNG\overrightarrow{AGGN}$	1.71	DR:7	18

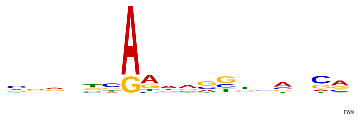	$NN\overrightarrow{GGTCN}NN\overrightarrow{NNNTCA}CNN$	1.74	DR:2	19
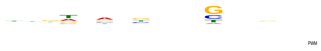	$TGTTNNNNNNNNNNNTGGG$	1.60	M	20

147 Rara-Jolma2013-Rnd2 (ERR194710)

PWM	Seed Sequence	Seed Seq Enrichment	Repeat	Rank
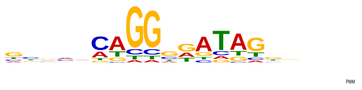	NNNNNCAGGNNATAGNNNNN	8.47	M	1
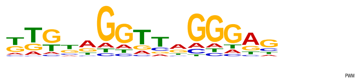	NNNNAGGTNNNGGAGNNNN	7.12	M	2
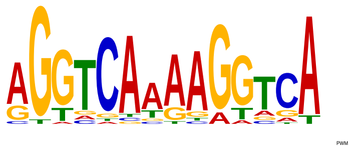	NNGTCAANAGGTCNN	18.61	DR:2	3
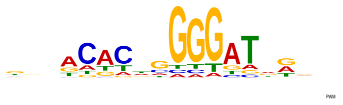	NNNNACACNNGGGATNNNN	15.33	M	4
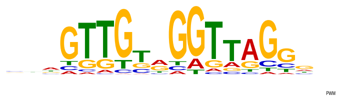	NNNAGTTGNNNNTTAGNNN	14.36	M	5
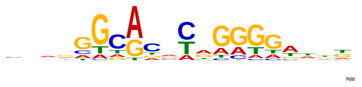	NNNNNGCACNNAGGGNNNNN	6.48	M	6
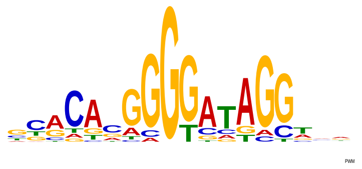	NNNCACGGNNATAGGNNN	14.77	DR:1	7
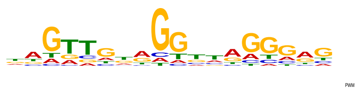	NAGTTNNNNNNNNNGGAGN	4.83	M	8
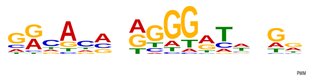	NNNNGCACNNNNGGATNNNN	5.40	M	9

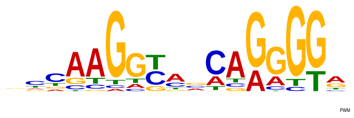	$NNCA\overrightarrow{AGGNNN}C\overrightarrow{AGGGNN}$	15.67	DR:1	10
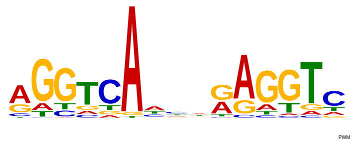	$\overrightarrow{AGGTCN}NNNNN\overrightarrow{NGGTC}$	7.08	DR:4	11
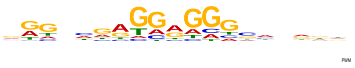	$NNNNNG\overrightarrow{AGGNGG}CNNNNN$	4.12	M	12
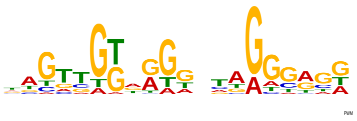	$N\overrightarrow{AGTTNN}NNNNNNNNGGAGN$	4.71	M	13
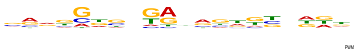	NNNNNTGCGNNAGTGNNNNN	2.85	M	14
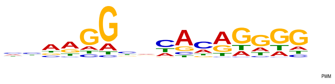	$NNNA\overrightarrow{AGGNNN}AC\overrightarrow{AGGNNN}$	8.75	DR:2	15
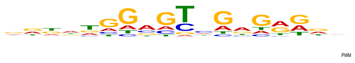	NNNNTGGANNCGAGNNNN	3.58	M	16
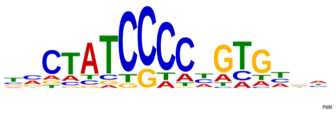	NNNCTATCNNCCGTGNNN	15.00	M	17
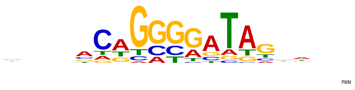	$NNNNAC\overrightarrow{AGNNGA}TAGNNNN$	13.81	M	18

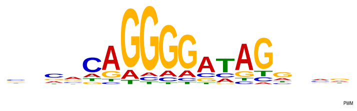	$NNNGC\overrightarrow{AGNNNA}T\overrightarrow{AGGNNN}$	6.76	DR:1	19
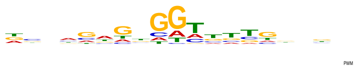	$NNNNGAGTNNNTTTGNNNN$	2.54	M	20

148 RARA-Jolma2013-Rnd2 (ERR194785)

PWM	Seed Sequence	Seed Seq Enrichment	Repeat	Rank
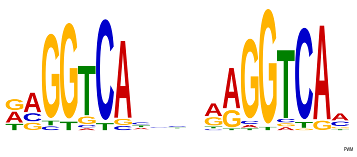	NNGGTCANNNNNAGGTCNN	43.24	DR:5	1
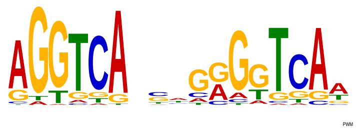	NGGTCANNNNNGGTCAN	32.01	DR:4	2
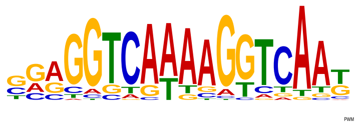	NNNGGTCANNAGGTCNNN	33.13	DR:2	3
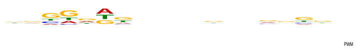	NGGGGNNNNNNNNNGGGGN	3.58	M	4
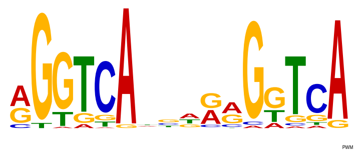	NGGTCANNNNAGGTCN	20.73	DR:4	5
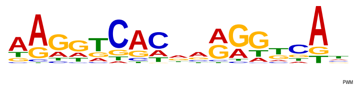	NNGGTCANNNNGGTCNN	5.72	DR:3	6
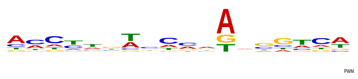	NCCTTNNNNNNNNGGTCN	2.92	ER:8	7
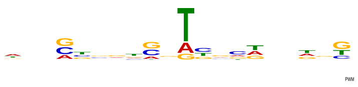	NNNNNNGTGGTCGCNNNNNN	2.63	M	8
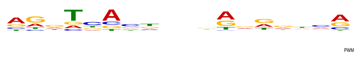	NGGTCNNNNNNNNGGTCN	2.66	DR:6	9

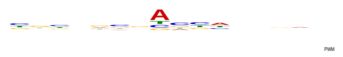	NNNNGGCGNCCACNNNN	2.18	M	10
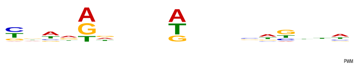	NNNNNGGGANAGAGNNNNN	1.82	M	11
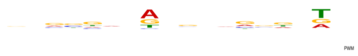	NNGGGANNNNNNGGGCNN	2.57	M	12
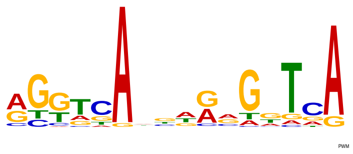	NGGTCNNNNNAGGTCN	6.01	DR:4	13
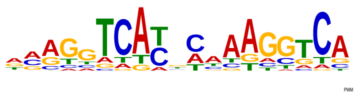	NNNGGTCNNNNAAAGGNNN	7.63	DR:5	14
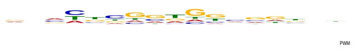	NNNNTCGGNGGCGNNNN	2.56	M	15
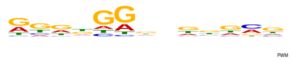	NNTGGGNNNGAGGGNN	3.25	M	16
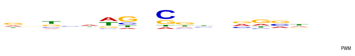	NGTCANNNNNNNNGGTCN	2.09	DR:7	17
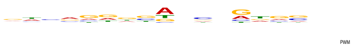	NNCAGGNNNNNNGTGGNN	2.49	M	18

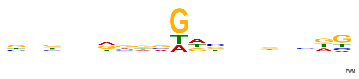	$NNNTGGGNNNNN\overrightarrow{AGGGNN}N$	1.96	M	19
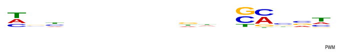	$NNNNNTCG\overrightarrow{GGGTCN}NNNN$	1.41	M	20

149 RARA-Jolma2013-Rnd3 (ERR193728)

PWM	Seed Sequence	Seed Seq Enrichment	Repeat	Rank
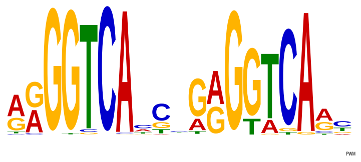	NNGGTCANNNNNGGTCANN	478.63	DR:4	1
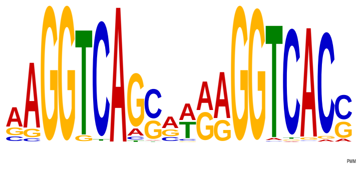	NNGGTCANNNNNNAGGTCANN	1769.83	DR:5	2
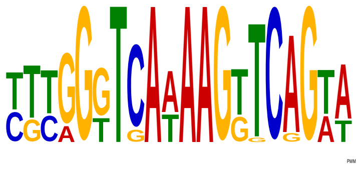	NNNAGTTCAANNGGTCANNN	548.12	DR:2	3
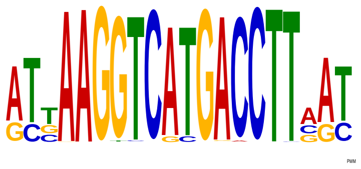	NNNNNGGTCATGACCNNNNN	727.68	IR:0	4
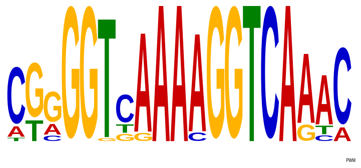	NNNGGTTAAAANGTCANNN	216.80	DR:2	5
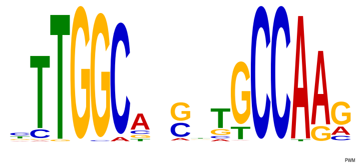	NTTGGCANNNNGCCAAN	122.18	DR:2	6
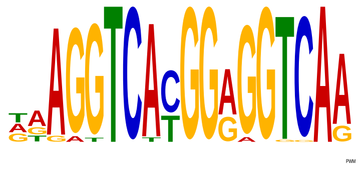	NTAGGTCANNNNGGTCAN	211.18	DR:3	7
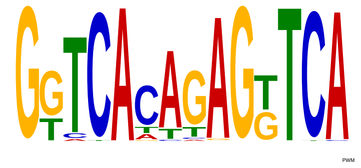	GGTCACNNAGTTCA	831.80	DR:3	8
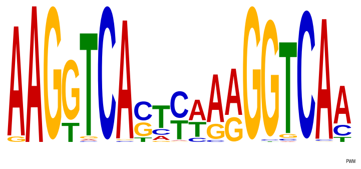	NAGTTCANNNNNNGGTCAN	422.71	DR:5	9

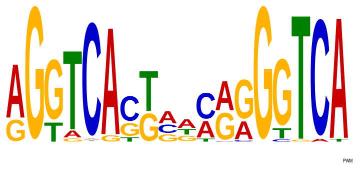	AGGTCNNNNNNNAGGTCA	94.19	DR:6	10
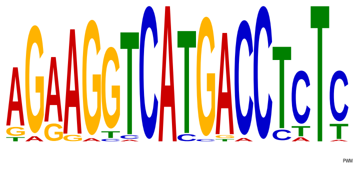	NNNAGGTCATGACCCNNN	373.93	IR:0	11
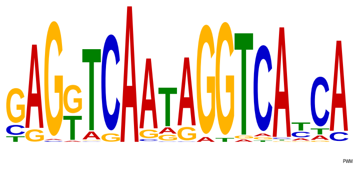	NNGGTCANNNGGTCACNN	226.02	DR:2	12
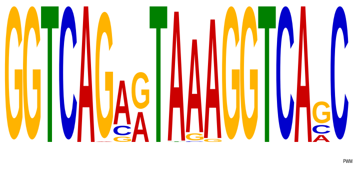	NNTCAGNNNAAAGGTCANN	996.21	DR:1	13
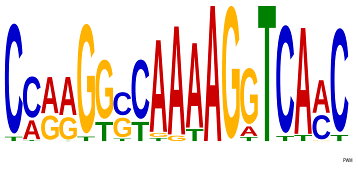	NNAGGGNNNAAAGGTCANN	132.14	DR:3	14
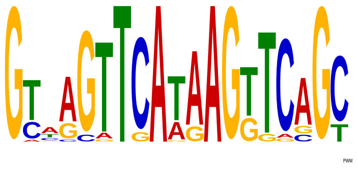	NNAAGTTCNNNNGGTCANN	82.44	DR:2	15
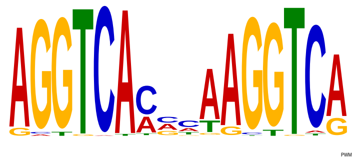	AGGTCNNNNNAGGTCG	132.84	DR:4	16
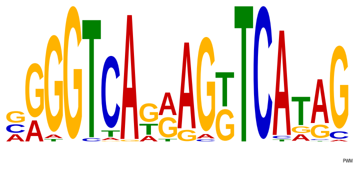	NNGGTCANNNGTTCAANN	109.04	DR:2	17
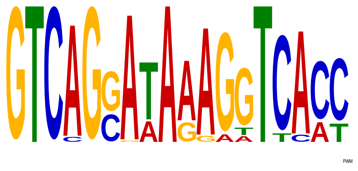	NNCAGGANNNAGGTCANN	54.32	DR:1	18

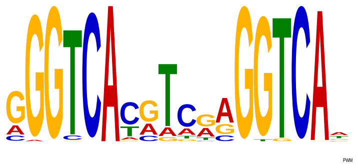	$\overrightarrow{GGGTCA}NNNNN\overrightarrow{NGGTCA}T$	639.50	DR:5	19
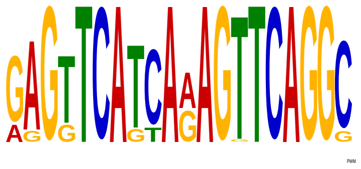	$N\overrightarrow{NNGTCA}NNAG\overrightarrow{AGTTCA}NNN$	1608.50	DR:4	20

150 Rara-Jolma2013-Rnd3 (ERR194711)

PWM	Seed Sequence	Seed Seq Enrichment	Repeat	Rank
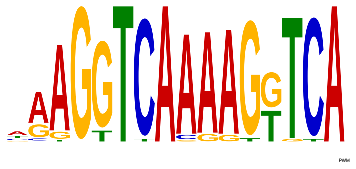	NAAGGTCNAAAGGTCN	2812.86	DR:2	1
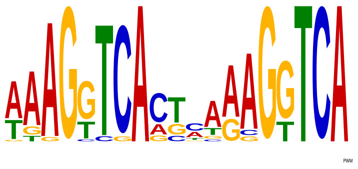	NAAGGTCANNNNNAGGTCN	750.81	DR:5	2
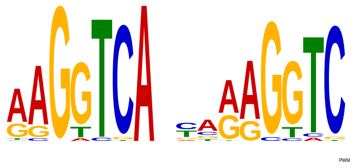	AAGGTCANNNNAGGTC	500.03	DR:4	3
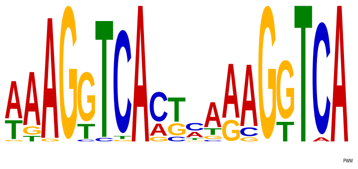	NAAGGTCNNNNNNAGGTCN	281.83	DR:5	4
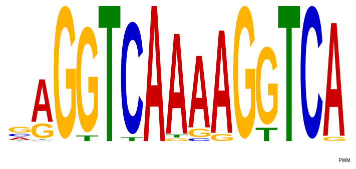	NGGGTCAANAGGTCN	360.32	DR:2	5
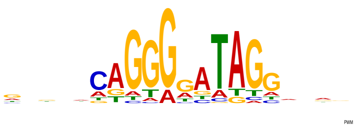	NNNNNCAGGNNATAGNNNNN	12.90	M	6
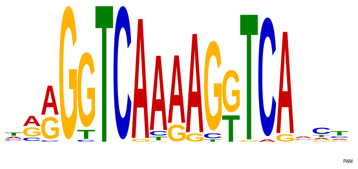	NNNNGTCAANAGGTCNNNN	137.97	DR:2	7
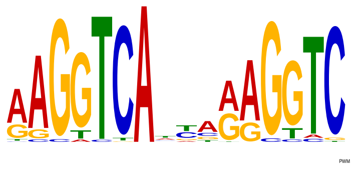	AAGGTCNNNNNAGGTC	200.47	DR:4	8
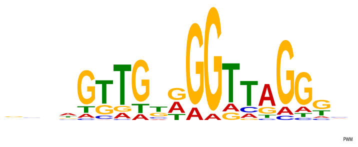	NNNAGTTGNNNNTTAGNNN	13.30	M	9

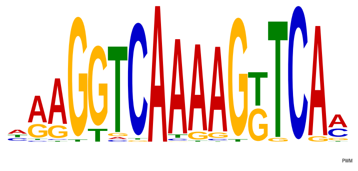	$NN\overrightarrow{AGGTCN}NN\overrightarrow{AGGTCN}N$	74.20	DR:2	10
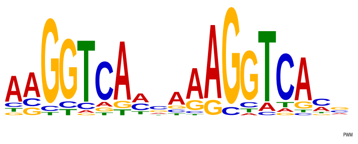	$N\overrightarrow{NGGTCA}NNNNN\overrightarrow{NNGTCA}NN$	18.11	DR:4	11
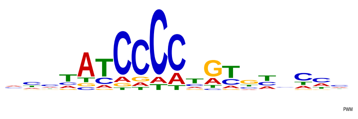	NNNNATCCNNNGTGTNNNN	7.66	M	12
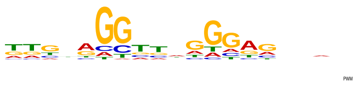	$NNNN\overrightarrow{AGGTNN}NGGAGNNNN$	6.03	M	13
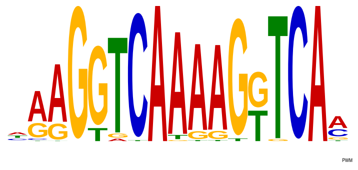	$NN\overrightarrow{AGGTCN}NA\overrightarrow{AGGTCN}N$	273.11	DR:2	14
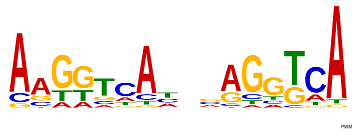	$N\overrightarrow{AGGTCN}NNNN\overrightarrow{NGGTCN}$	5.83	DR:3	15
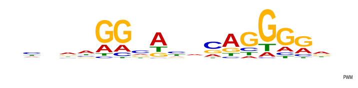	$NNNN\overrightarrow{AGGCNN}NC\overrightarrow{AGGNNN}N$	5.14	DR:2	16
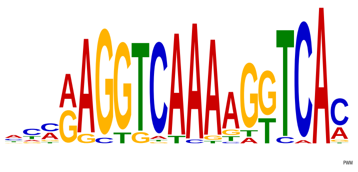	$NNNN\overrightarrow{AGGTCN}AA\overrightarrow{AGGNNN}N$	76.01	DR:2	17
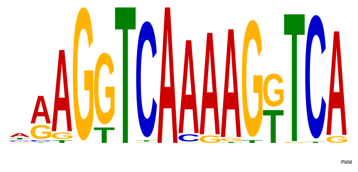	$NA\overrightarrow{AGGTCN}NN\overrightarrow{AGGTCN}$	196.23	DR:2	18

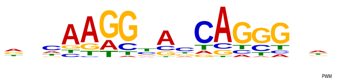	$NNNCA\overrightarrow{AGGNNN}C\overrightarrow{AGGNNN}$	8.80	DR:1	19
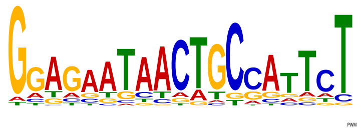	*NGAGANNNNNNNNNATTCN*	5.83	M	20

151 RARA-Jolma2013-Rnd3 (ERR194786)

PWM	Seed Sequence	Seed Seq Enrichment	Repeat	Rank
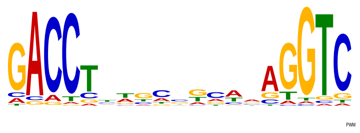	GACCTNNNNNNNNNNGGTC	24.03	ER:9	1
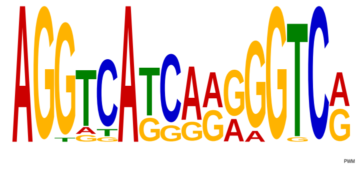	NGGTCATNGGAGGTCN	255.40	DR:4	2
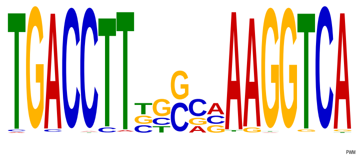	NGACCTNNNNNNNAGGTCN	259.60	ER:7	3
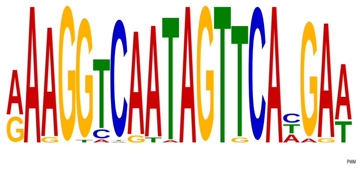	NNNGGTCAANAGTTCAANNN	3378.19	DR:2	4
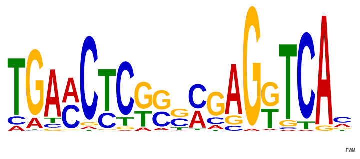	NGACCTNNNNNNGGTCAN	34.09	ER:6	5
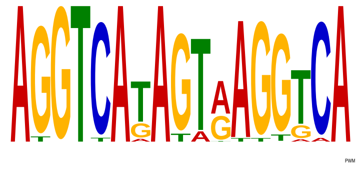	NGGTCATNNNAAGTTCN	69.29	DR:5	6
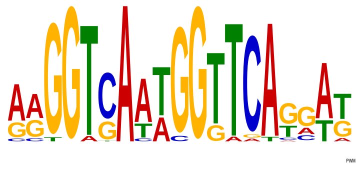	NNGGTCANNNGGTCATCNN	219.51	DR:2	7
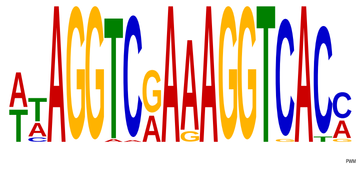	NNAGGTCGANAGGTCANN	924.90	DR:2	8
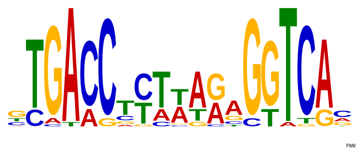	NNGACCTNNNNNAGGTCNN	20.21	ER:5	9

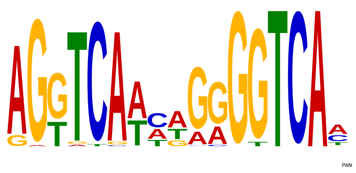	$\overrightarrow{AGTTCA}NNNN\overrightarrow{NGGTCA}A$	353.04	DR:4	10
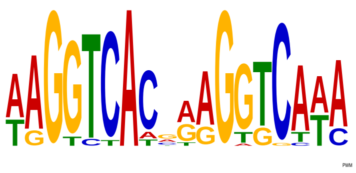	$N\overrightarrow{NGGTCA}CNN\overrightarrow{GGGTCA}NN$	233.11	DR:3	11
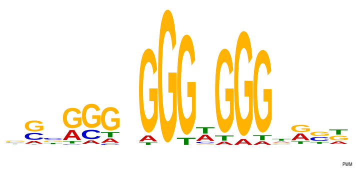	$NN\overrightarrow{NGGGCG}GGN\overrightarrow{GGGTNN}N$	36.44	DR:3	12
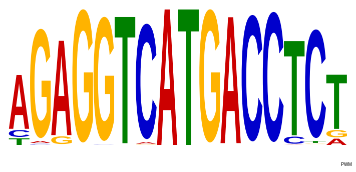	$NG\overrightarrow{AGGTCN}\overleftarrow{NNACCT}CN$	341.27	IR:0	13
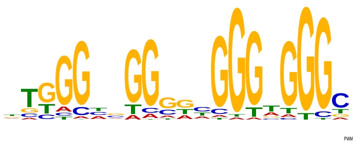	$NTGGGNNNNNN\overrightarrow{GGGTGG}GN$	93.62	M	14
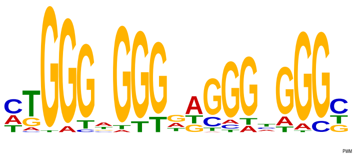	$NTGGGNNNNN\overrightarrow{NGGGCG}GGN$	91.25	M	15
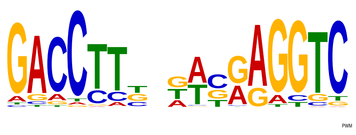	$\overleftarrow{GACCT}CNNNNNN\overrightarrow{NGGTC}$	28.26	ER:7	16
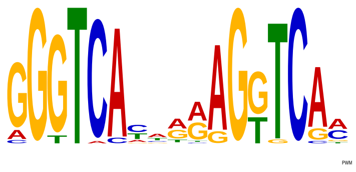	$\overrightarrow{NGGTCA}GNNN\overrightarrow{AGTTCA}N$	138.40	DR:4	17
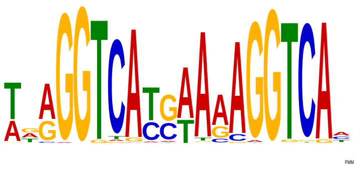	$NN\overrightarrow{GGGTCA}NNNNN\overrightarrow{NGGTCN}N$	184.79	DR:5	18

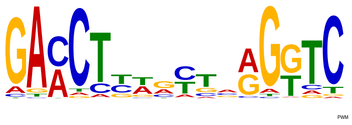	$\overleftarrow{GACCT}TNNNNN\overrightarrow{NGGTC}$	27.69	ER:6	19
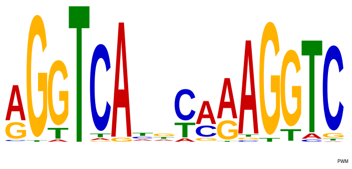	$\overrightarrow{GGGTCA}NNNNN\overrightarrow{AGTTC}$	48.05	DR:5	20

152 RARA-Jolma2013-Rnd4 (ERR193729)

PWM	Seed Sequence	Seed Seq Enrichment	Repeat	Rank
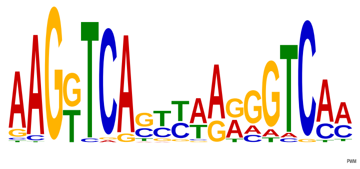	NAGTTCNNNNNNNNGTCAN	33.12	DR:5	1
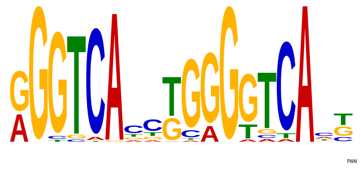	NNGTCAGNNNNNNGTCANN	16.59	DR:4	2
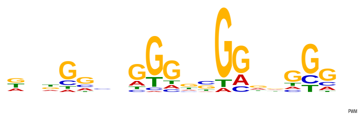	NTGGGNNNNNNNNNNAGGGN	4.45	M	3
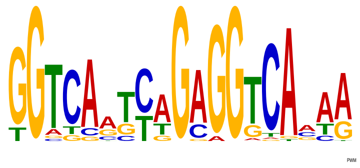	NNTCAANNNNNNNTCAGNN	10.36	DR:5	4
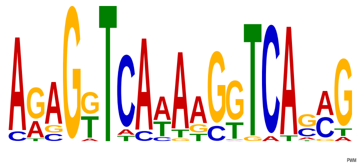	NNNGTTCANNNNTTCANNN	20.47	DR:2	5
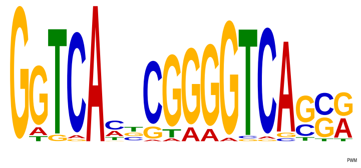	NNTCAANNNNNGTCAGNN	26.32	DR:4	6
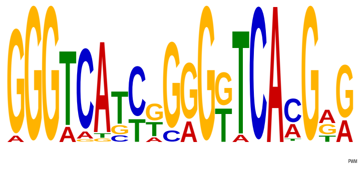	NNNTCAGNNNNNNNTCAANNN	12.45	DR:4	7
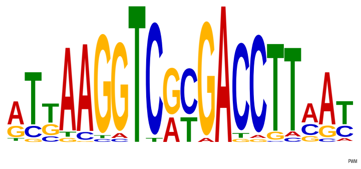	NNNNNGGTCNNGACCNNNNN	9.15	IR:0	8
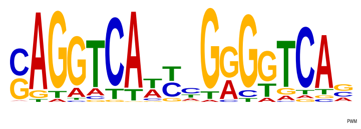	GAGGNNNNNNNNNNTCAG	8.33	DR:4	9

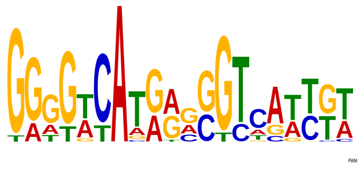	NNNNTCATNNNNGTCANNNNN	4.98	DR:3	10
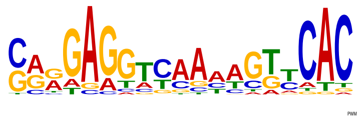	NNNNNGGTTNAAAGNNNNN	4.44	M	11
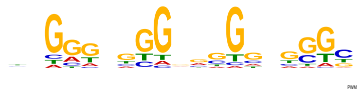	NTGGGNNNNNNNNNCGGGN	4.47	M	12
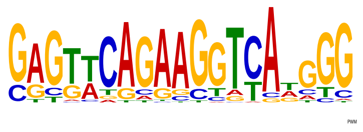	NNNGTCANNNNNTCATNNN	11.52	DR:2	13
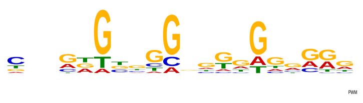	NTGGGNNNNNNNNNNGAGGN	3.46	M	14
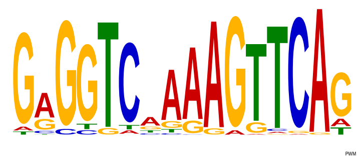	NGGGTCNNNNNTTCAN	15.08	DR:2	15
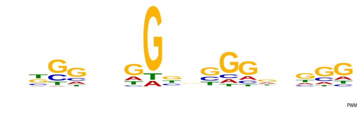	TGGGNNNNNNNNNNCGGG	3.43	M	16
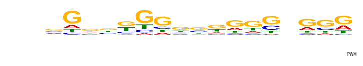	TGGGNNNNNNNNNNNAGGG	3.77	M	17
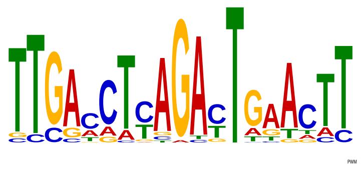	NNNNCCTTNNNGTGANNNN	5.04	ER:2	18

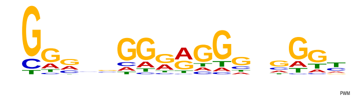	*NGGGANNNNNNNNCGGGN*	3.82	M	19
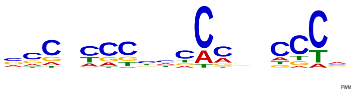	*CCCCNNNNNNNNNTCCCA*	5.92	M	20

153 Rara-Jolma2013-Rnd4 (ERR194712)

PWM	Seed Sequence	Seed Seq Enrichment	Repeat	Rank
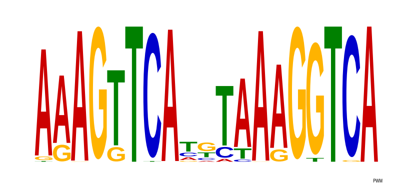	AAAGTTCANNNNNNGGTCA	5080.53	DR:5	1
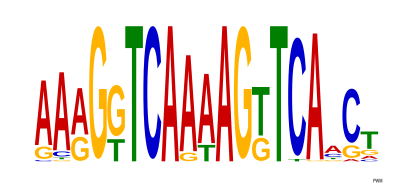	NNAGGTCNNNAGTTCACNN	995.07	DR:2	2
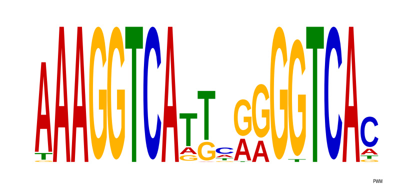	AAAGGTCNNNNNGGGTCAC	2569.42	DR:4	3
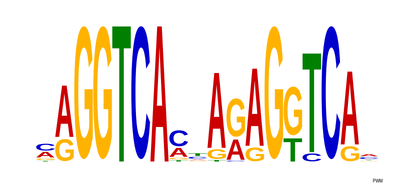	NNGGTCACNAGAGGTCNN	703.18	DR:4	4
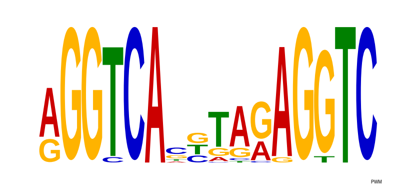	GGGTCANNTAGAGGTC	1600.08	DR:5	5
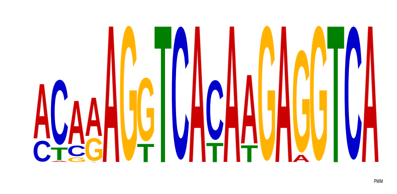	NCAGAGGTCNNNNNNGGTCN	362.15	DR:4	6
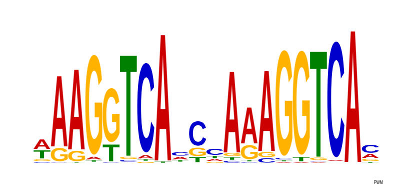	NNAGGTCNNNNNNNGGTCNN	52.83	DR:5	7
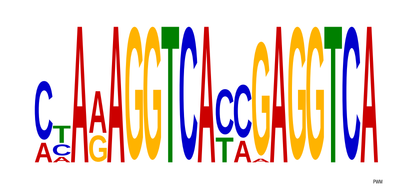	NTAAAGGTCNNNGAGGTCN	963.84	DR:3	8
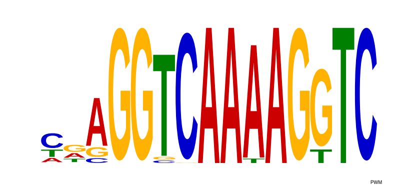	CTAGGTCNNNAGGTC	220.31	DR:2	9

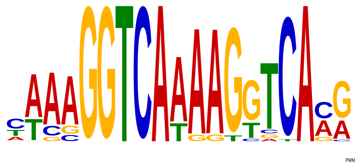	NNN$\overrightarrow{AGGTCN}$AA$\overrightarrow{GGGTCN}$NN	239.60	DR:2	10
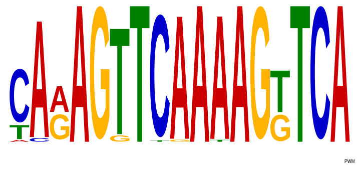	NAG$\overrightarrow{AGTTCN}$NN$\overrightarrow{AGTTCN}$	447.69	DR:2	11
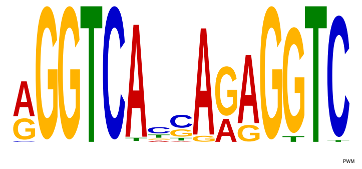	$\overrightarrow{GGGTCA}$CCNN$\overrightarrow{AGGTC}$	525.70	DR:4	12
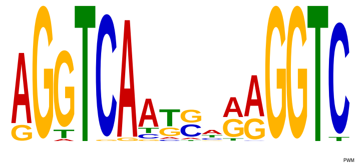	$\overrightarrow{AGGTCN}$NNNCG$\overrightarrow{AGGTC}$	219.31	DR:5	13
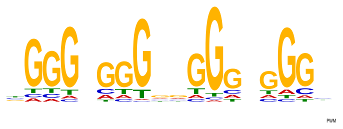	NTGGGNNNNNNNNNCGGGN	13.53	M	14
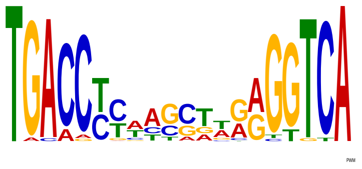	$\overleftarrow{NGACCT}$NNNNNNNN$\overrightarrow{AGGTCN}$	29.38	ER:8	15
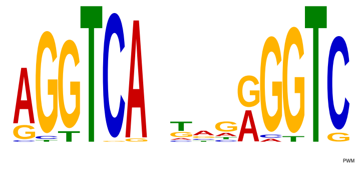	$\overrightarrow{AGGTCN}$NNCG$\overrightarrow{AGGTC}$	192.10	DR:4	16
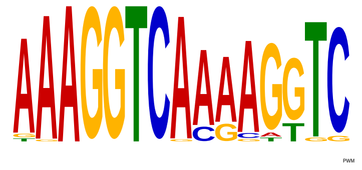	AA$\overrightarrow{AGGTNN}$CA$\overrightarrow{AGGTC}$	290.98	DR:2	17
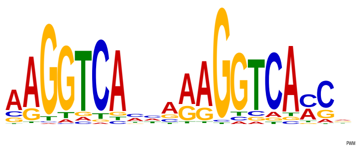	N$\overrightarrow{NNGTCA}$NNNNN$\overrightarrow{NNGTCA}$NNN	13.50	DR:4	18

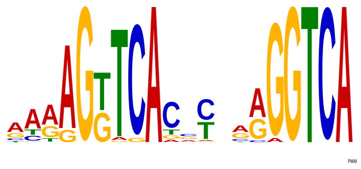	$NAG\overrightarrow{AGGTC}NNNNNN\overrightarrow{NGGTC}N$	161.65	DR:5	19
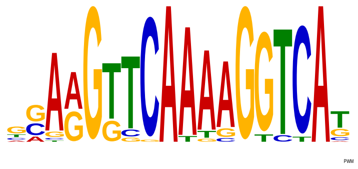	$NNG\overrightarrow{AGGTC}NNN\overrightarrow{AGTTC}NN$	40.94	DR:2	20

154 Rara-Jolma2013-Rnd5 (ERR194713)

PWM	Seed Sequence	Seed Seq Enrichment	Repeat	Rank
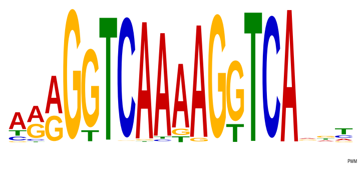	NNNNGTCANNNGGTCNNNN	135.97	DR:2	1
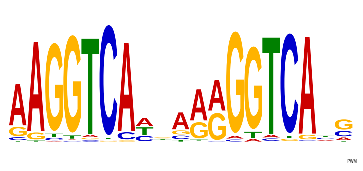	NNGGTCNNNNNNNGTCANN	20.65	DR:4	2
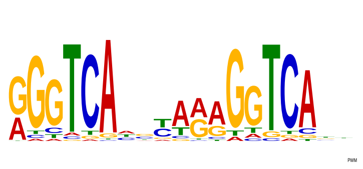	NNGTCANNNNNNNGTCANN	14.35	DR:5	3
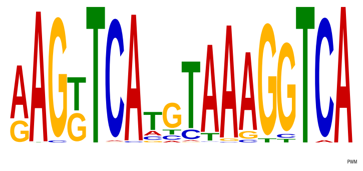	AAGTTCNNNNNNNNGTCA	120.00	DR:5	4
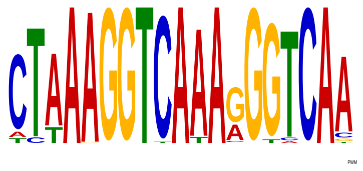	NNAAAGNNNNAAGGGTCNN	377.44	M	5
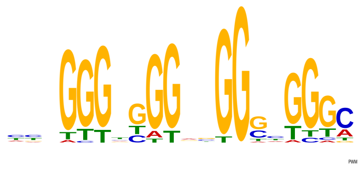	NCTGGNNNNNNNNNNCGGGN	7.00	M	6
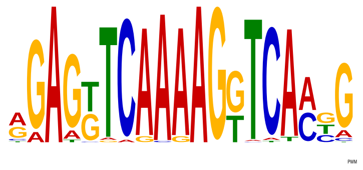	NNAGTTCNNNNNNTCACNN	43.34	DR:2	7
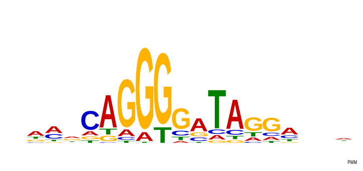	NNNNCAGGNNNTAGGNNNN	6.36	DR:1	8
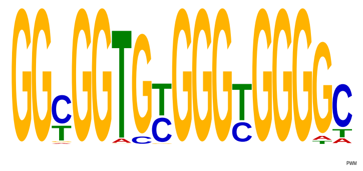	GGCGGTGNNNNTGGGGC	289.92	DR:4	9

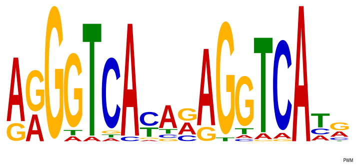	NNNGTCACNNNGGTCNNN	15.76	DR:3	10
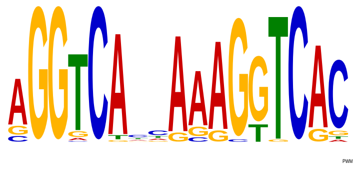	NNGTCACCNNNGGTCNN	72.79	DR:4	11
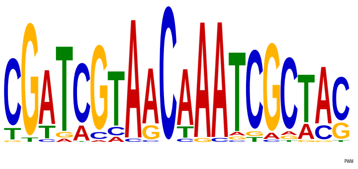	NNNTCGTNNNNNNTCGCNNN	18.96	M	12
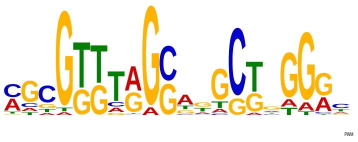	AGCGNNNNNNNNNNNNGGGC	10.48	M	13
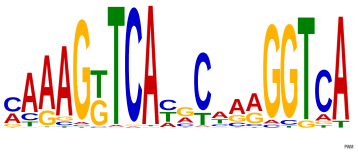	NAGAGNNNNNNNNNNGGTCN	13.28	M	14
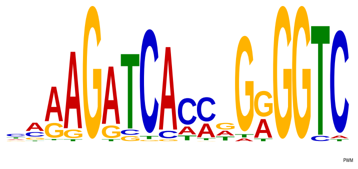	CAGAGGNNNNNNNNGGTC	27.20	DR:4	15
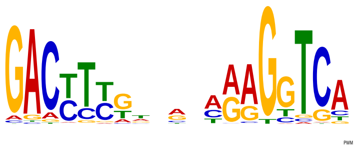	GACCNNNNNNNNNNNGTCA	12.20	ER:8	16
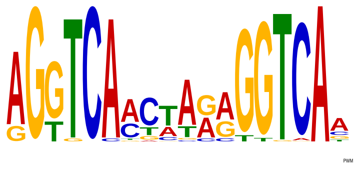	NNGTCAACNNNNGGTCNN	80.51	DR:5	17
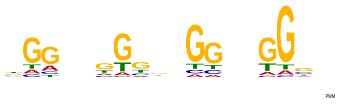	TGGGNNNNNNNNNNNGGGT	5.38	M	18

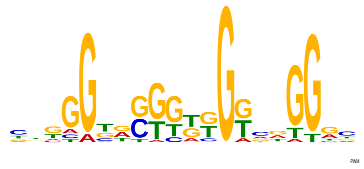	$CTGGNNNNNNNNNNNN\overrightarrow{NGGGC}$	8.00	M	19
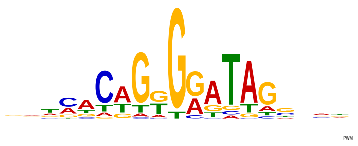	$NNNNAC\overrightarrow{AGNNNA}TAGNNNN$	7.61	M	20

155 RARA-8-Bhimsaria2022-Rnd2 (S07-072)

PWM	Seed Sequence	Seed Seq Enrichment	Repeat	Rank
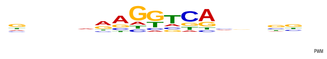	NNNNNNAAAGGTCANNNNNN	5.97	M	1
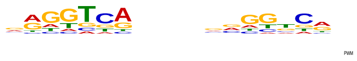	NNGGTCNNNNNNNGGTCNN	4.45	DR:5	2
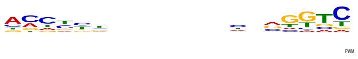	ACCTNNNNNNNNNNNGGTC	5.17	ER:11	3
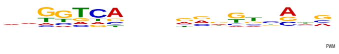	NNNGGTCNNNNNNGGTCNNN	2.96	DR:4	4
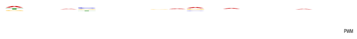	ATAANNNNNNNNNNNNAATT	3.00	M	5
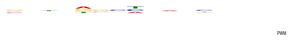	NNNNNNTGACGTCANNNNNN	1.68	M	6
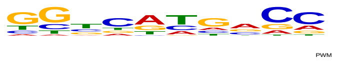	GGTCATGACC	9.62	IR:0	7
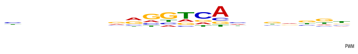	NNNNNNGGGGTCAANNNNNN	3.23	M	8
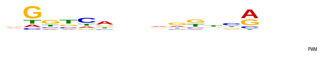	NNNNGTCANNNGGTCNNNN	3.24	DR:2	9

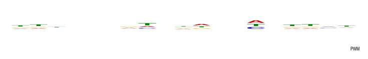	*TTTTNNNNNNNNNNNTTCT*	3.15	M	10
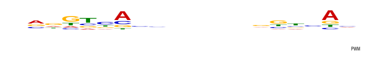	*NNGGTCNNNNNNNNGGTCNN*	2.15	DR:6	11
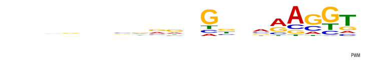	*AGGGNNNNNNNNNNNAAGGT*	4.33	DR:10	12
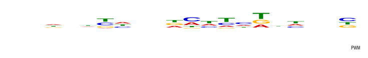	*NTATTNNNNNNNNNNTTTTN*	2.97	M	13
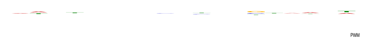	*AATTNNNNNNNNNNNGATT*	2.64	M	14
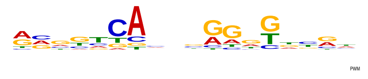	*NNGGTCATNNNNGGTCNN*	5.85	DR:4	15
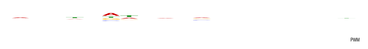	*ACGTNNNNNNNNNNNAATT*	2.42	M	16
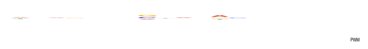	*ATAGNNNNNNNNNNNATTT*	2.49	M	17
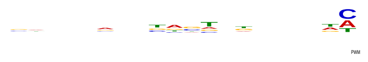	*NATGTNNNNNNNNNNATATN*	2.25	M	18

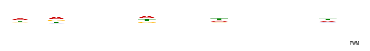	*ATATNNNNNNNNNNNCATT*	2.95	M	19
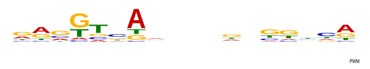	$N\overrightarrow{AGTTC}NNNNNNN\overrightarrow{GGTCN}$	3.52	DR:5	20

156 RARA-8-Bhimsaria2022-Rnd3 (S07-097)

PWM	Seed Sequence	Seed Seq Enrichment	Repeat	Rank
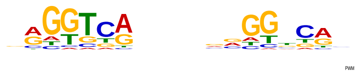	NNGGTCNNNNNNNGGTCNN	15.45	DR:5	1
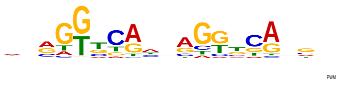	NNNNGGTCANNNGGTCNNNN	20.02	DR:2	2
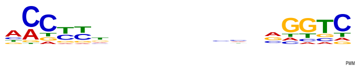	ACCTNNNNNNNNNNNNGGTC	18.45	ER:11	3
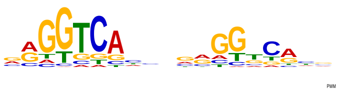	NNNGGTCANNNNNGGTCNNN	25.28	DR:4	4
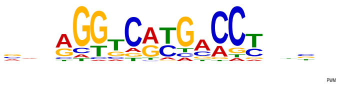	NNNNNGGTCATGACCNNNNN	57.59	IR:0	5
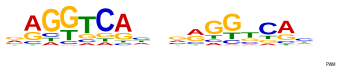	NNNGGTCANNNNGGTCNNN	16.99	DR:3	6
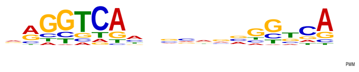	NNGGTCANNNNNNNGGTCNN	13.38	DR:6	7
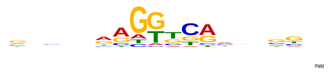	NNNNNNAAAGGTCANNNNNN	14.27	M	8
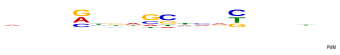	NNNNNNTGACGTCANNNNNN	3.95	M	9

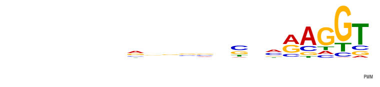	$GGGGNNNNNNNNNNNNA\overrightarrow{AGGT}$	16.81	M	10
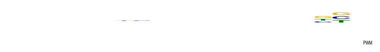	$NGGGGNNNNNNNNN\overrightarrow{GGGTN}$	4.49	M	11
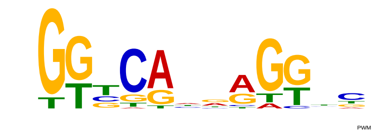	$\overrightarrow{GGTCA}AG\overrightarrow{GGGTC}$	63.40	DR:2	12
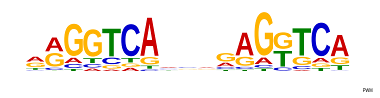	$NN\overrightarrow{GGTCN}NNNG\overrightarrow{AGTTCN}N$	23.62	DR:4	13
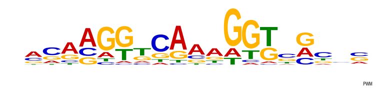	$NNNN\overrightarrow{GGTCA}A\overrightarrow{AGGTCN}NNNN$	37.38	DR:1	14
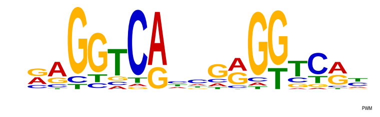	$NN\overrightarrow{GGTCA}TNG\overrightarrow{GGGTCN}N$	82.45	DR:3	15
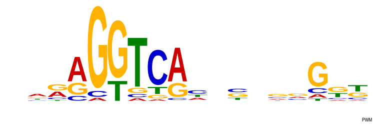	$NA\overrightarrow{AGGTCA}CNNNGGGGN$	111.65	M	16
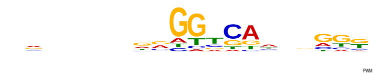	$NNNNNCG\overrightarrow{GGGTCA}ANNNNN$	14.58	M	17
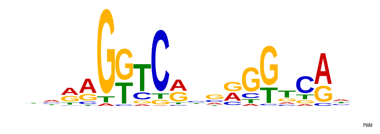	$NNN\overrightarrow{AGGTCN}NG\overrightarrow{GGGTCN}NN$	38.86	DR:2	18

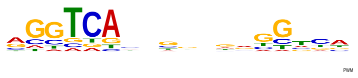	$\overrightarrow{NGGTCA}TNNNNN\overrightarrow{NGGTCN}$	15.01	DR:6	19
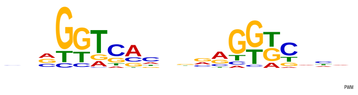	$NN\overrightarrow{NGGTCN}NNNA\overrightarrow{AGGTCN}NN$	20.62	DR:4	20

157 RARA-SMiLE-seq-Rnd1 (SRR3405076)

PWM	Seed Sequence	Seed Seq Enrichment	Repeat	Rank
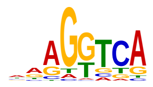	NNNNNNAAAGGTCANNNNNNN	17.32	M	1
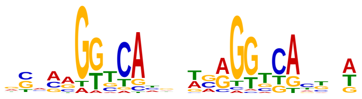	NNNNAGGTCANNNNNAGGTCANNNN	176.55	DR:5	2
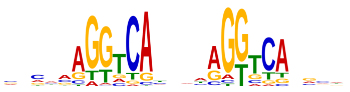	NNNNNAGGTCANNNNNGGTCANNNNN	97.57	DR:4	3
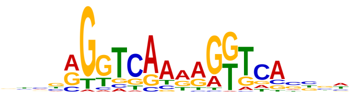	NNNNAGGTCANNAGGTCANNNN	130.16	DR:2	4
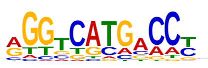	NNNNNNGGTCATGACCNNNNNN	36.89	IR:0	5
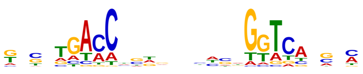	NNNNTGACCNNNNNNNNNNGGTCANNNNN	31.02	ER:8	6
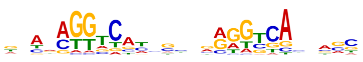	NNNNNGGTCANNNNNNNNGGTCANNNNN	23.95	DR:6	7
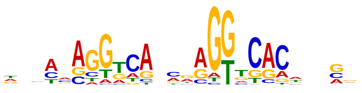	NNNNNNGGTCANNNAGGTCANNNNNNN	64.02	DR:3	8
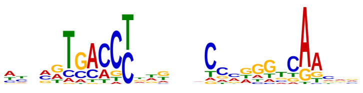	NNNNNTGACCNNNNNNNNNNNGGTCNNNNN	19.73	ER:9	9

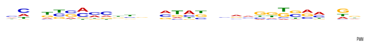	$NNNN\overleftarrow{NGACCN}NNNNNNNNNN\overrightarrow{NGGTCN}NNNN$	5.42	ER:10	10
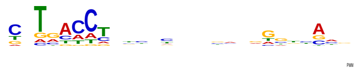	$NNN\overleftarrow{TGACCN}NNNNNNNNNNN\overrightarrow{NGGTCN}NN$	13.57	ER:11	11
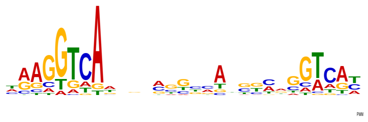	$NN\overrightarrow{AGGTCA}NNNNNNNNNNNNNN\overrightarrow{NGGTCN}N$	36.28	DR:14	12
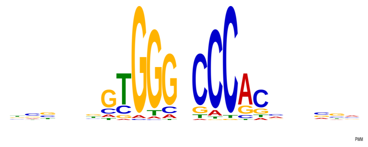	$NNNNNNGT\overrightarrow{GGGNCC}CACNNNNNN$	22.09	M	13
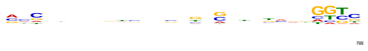	$\overleftarrow{ACCT}NNNNNNNNNNNNNNNNNNNN\overrightarrow{NGGTC}$	6.11	ER:20	14
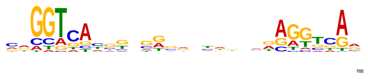	$N\overrightarrow{NGGTCA}NNNNNNNNNNNNNNN\overrightarrow{NGGTCN}N$	15.50	DR:15	15
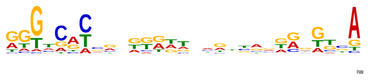	$\overrightarrow{NGGTCA}NNNNNNNNNNNNNNNNN\overrightarrow{NGGTCN}$	7.85	DR:17	16
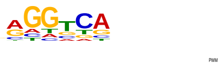	$NNNNNNCG\overrightarrow{GGGTCA}NNNNNN$	5.98	M	17
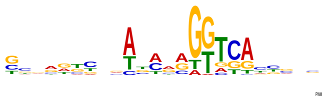	$NNNNN\overrightarrow{AGGGNN}NNNNN\overrightarrow{AGGTCA}NNNNN$	15.54	DR:5	18

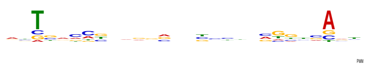	$NN\overleftarrow{NGACC}NNNNNNNNNNNN\overrightarrow{NGGTC}NNN$	5.39	ER:12	19
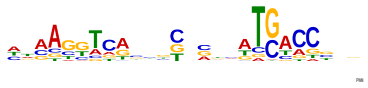	$NNN\overrightarrow{AGGTCN}NNNNNNNNN\overleftarrow{TGACC}NNN$	15.91	IR:9	20

158 RARB:RXRA-Bhimsaria2022-Rnd3 (S13-150)

PWM	Seed Sequence	Seed Seq Enrichment	Repeat	Rank
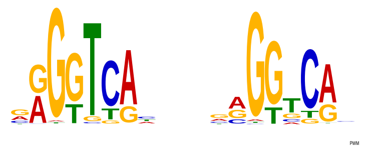	NNGGTCANNNNNNGGTCNN	1188.71	DR:5	1
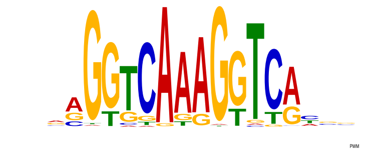	NNNNGGTCAAAGGTCNNNN	2101.09	DR:1	2
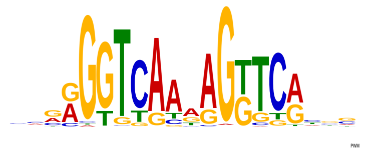	NNNNGGTCAANAGGTCNNNN	2600.61	DR:2	3
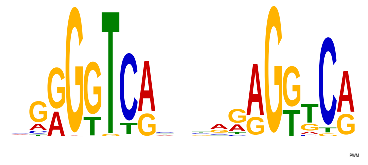	NGGGGTCNNNNNGAGTTCN	3978.38	DR:5	4
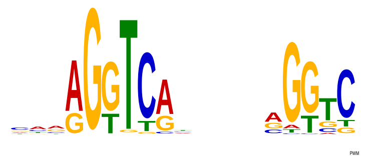	CAAAGGTCNNNNNNNGGTC	2925.95	DR:5	5
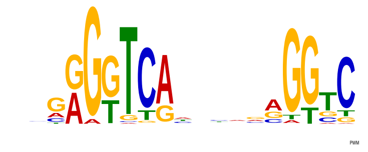	NAGGGGTCNNNNNNGGGTN	549.59	DR:5	6
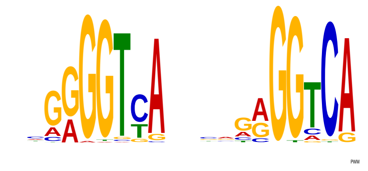	NNGGGGTTANNNNGGGGTNN	1196.00	DR:5	7
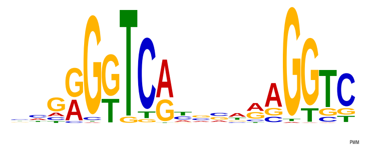	NAGGGGTCNNNNNAAGGTN	1635.15	DR:5	8
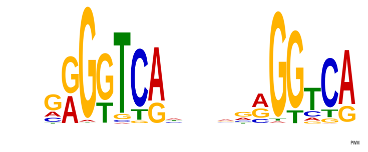	NNGGGGTCNNNNNNGGGTNN	872.00	DR:5	9

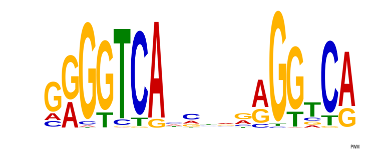	NCGGGGTCNNNNNNNGGGCN	719.89	DR:5	10
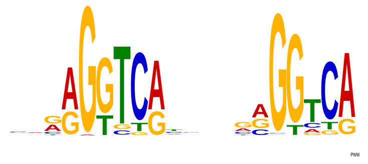	NNAAGGTCNNNNNGGGGTNN	930.96	DR:5	11
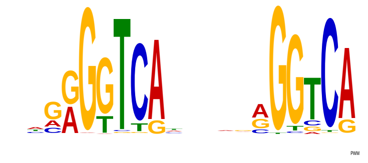	NNGGGGTCNNNNNNCGGTNN	402.54	DR:5	12
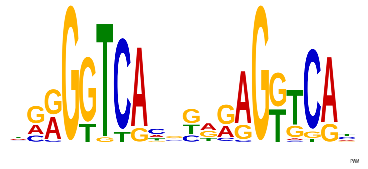	NNAGGTCACNNNGAGTTCNN	6674.11	DR:5	13
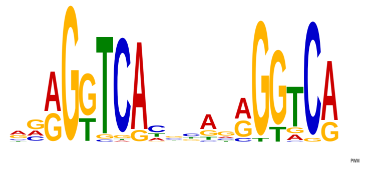	NNAGTTCACGNNNNGGTCNN	2372.68	DR:5	14
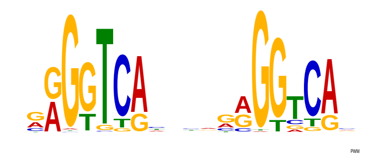	NNNGGTCNNNNNGGGGTNNN	444.23	DR:5	15
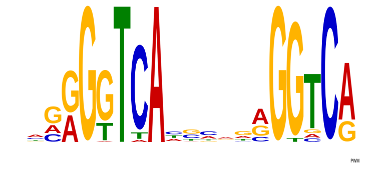	NNGGGGTCACNNNNCGGTNN	1521.81	DR:5	16
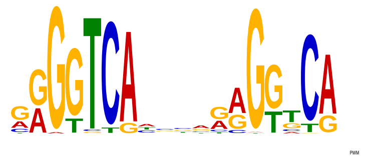	NNGGTCATNNNNAGGGCNN	1387.34	DR:5	17
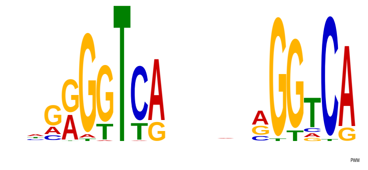	NCGGGGTCNNNNNNCGGTCN	1969.16	DR:5	18

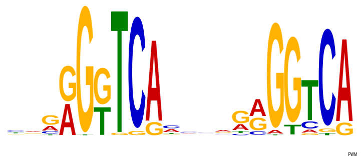	$NNG\overrightarrow{AGTTCA}NNNNN\overrightarrow{NGGGTN}N$	926.41	DR:5	19
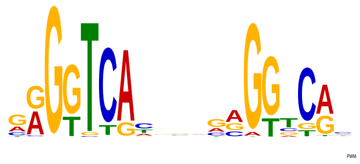	$N\overrightarrow{NGGTCA}CGGNN\overrightarrow{NGGTCN}N$	3474.28	DR:5	20

159 RARB-Bhimsaria2022-Rnd3 (S07-152)

PWM	Seed Sequence	Seed Seq Enrichment	Repeat	Rank
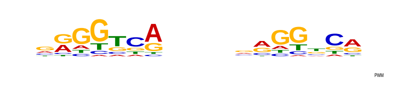	NNGGTCNNNNNNNGGTCNN	11.96	DR:5	1
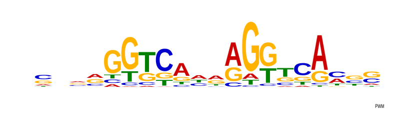	NNNNGGTCAANNGGTCNNNN	31.79	DR:2	2
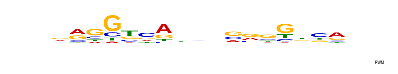	NNNGGTCNNNNNNGGTCNNN	7.28	DR:4	3
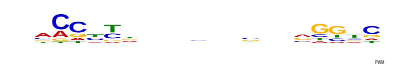	ACCTNNNNNNNNNNNNGGTC	9.77	ER:11	4
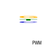	GGGGNNNNNNNNNNNGGGG	7.24	M	5
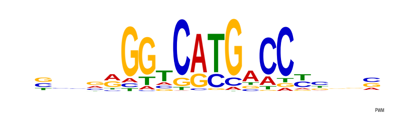	NNNNNGGTCATGACCNNNNN	58.23	IR:0	6
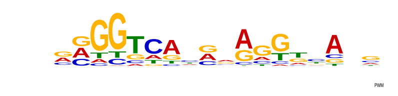	NNNGGTCACNNNGGTCNNN	19.30	DR:3	7
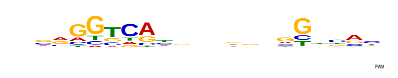	NNGGTCANNNNNNNGGTCNN	9.47	DR:6	8
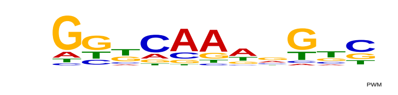	GGTCAAAGGTC	24.94	DR:1	9

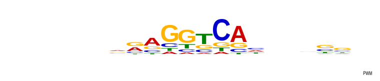	*NNNNNNAAGGTCAANNNNNN*	10.76	M	10
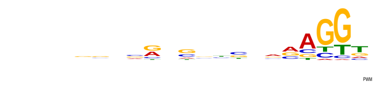	*AGGGNNNNNNNNNNNNAAGGT*	10.85	DR:10	11
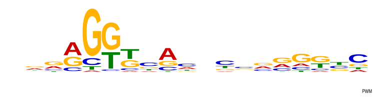	*NAAGGTCNNNNNGGGGTN*	15.95	DR:5	12
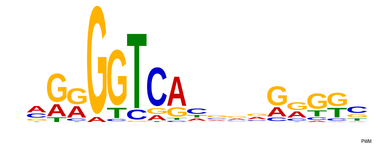	*NGGGGTCACNNNGGGTN*	67.84	DR:4	13
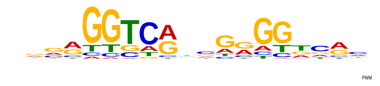	*NNAGGTCNNNGGGGTCNN*	22.71	DR:3	14
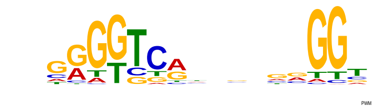	*CGGGGTCNNNNNGGGGT*	99.43	DR:5	15
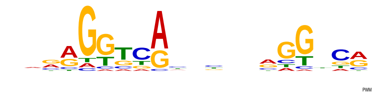	*NAAGGTCNNNNNNNGGTCN*	22.28	DR:5	16
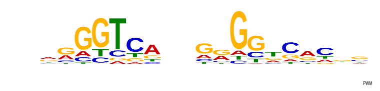	*NNNNGTCANNGGGGTCNNNN*	15.51	DR:3	17
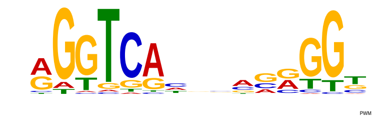	*AGGTCACGNNNGGGT*	82.79	DR:5	18

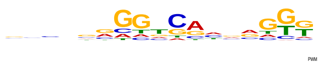	$NNNNNCG\overrightarrow{GGGTC}AANNNNNN$	11.14	M	19
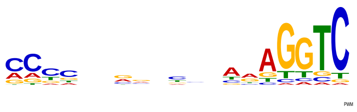	$CCCCNNNNNNNNNNA\overrightarrow{AGGTC}$	29.33	M	20

160 Rarb-Jolma2013-Rnd2 (ERR194715)

PWM	Seed Sequence	Seed Seq Enrichment	Repeat	Rank
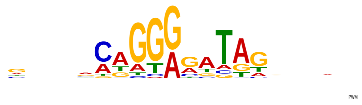	NNNNNCAGGNNATAGNNNNN	9.44	M	1
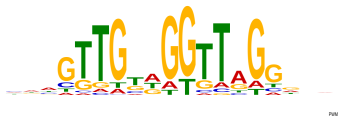	NNNNGTTGNNNNTTAGNNNN	11.67	M	2
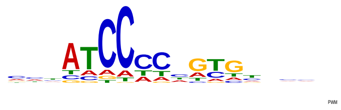	NNNNATCCNNNGTGTNNNN	9.90	M	3
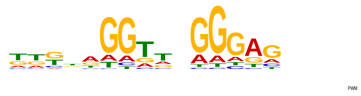	NNNNAGGTNNNGGAGNNNN	8.29	M	4
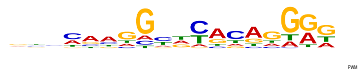	NNNNAAGGNNNACAGNNNN	6.38	M	5
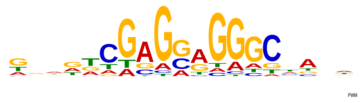	NNNNTCGAGNNGGGCNNNN	11.85	M	6
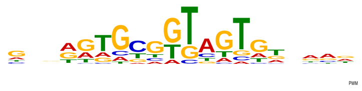	NNNNNTGCGNNAGTGNNNNN	6.61	M	7
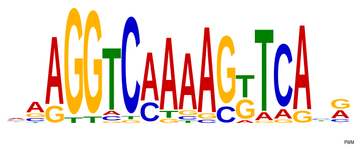	NNNGGTCANNAGGTCNNN	23.45	DR:2	8
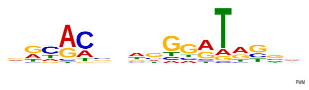	NNNNGCACNNNNGGATNNNN	5.99	M	9

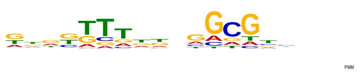	*NNNNTTTTNNNGCGTNNNN*	5.03	M	10
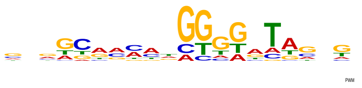	*NNGGCANNNNNNNNNATAGNN*	3.75	M	11
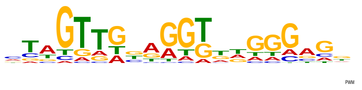	*NTAGTNNNNNNNNNNGGAGN*	6.54	M	12
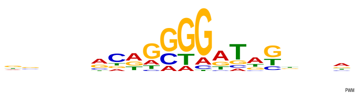	*NNNNNACAGNNGATANNNNN*	7.36	M	13
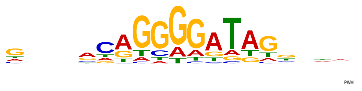	*NNNNACAGNNNATAGNNNN*	9.72	M	14
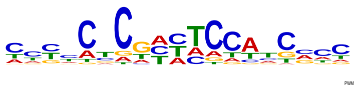	*NNNNCTCGNNNCCATNNNN*	5.46	M	15
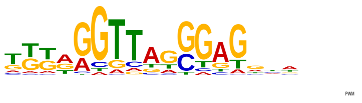	*NNNNNGTTANNGAGGNNNNN*	8.39	DR:3	16
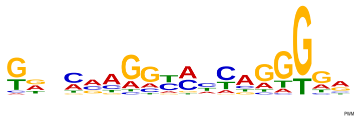	*NNNCAAGGNNNCAGGNNN*	8.00	DR:1	17
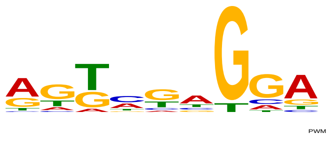	*AGTCGAGGA*	9.14	DR:0	18

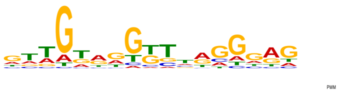	*NNTTGTNNNNNNNNGGAGNN*	4.23	M	19
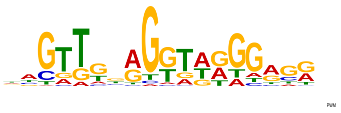	*NNAGTTNNNNNNNNGGAGNN*	5.56	M	20

161 Rarb-Jolma2013-Rnd3 (ERR194716)

PWM	Seed Sequence	Seed Seq Enrichment	Repeat	Rank
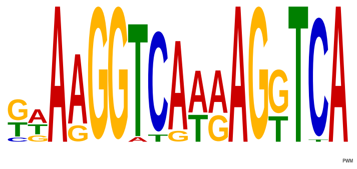	NAAAGGTCNAAAGGTCN	19914.82	DR:2	1
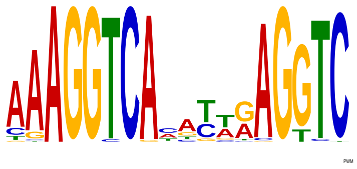	AAAGGTCANNNNNAGGTC	5716.08	DR:5	2
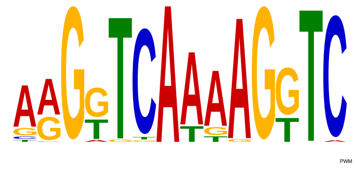	GAGGTCAANAGGTC	2038.21	DR:2	3
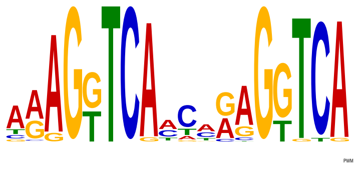	NAAGGTCANNNNAGGTCN	653.96	DR:4	4
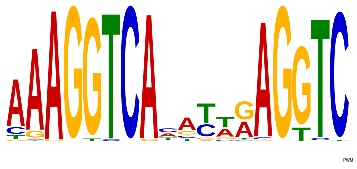	AAAGGTCNNNNNNAGGTC	2169.43	DR:5	5
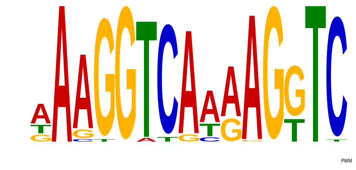	NAAAGGTCNAAAGGTN	4552.28	DR:2	6
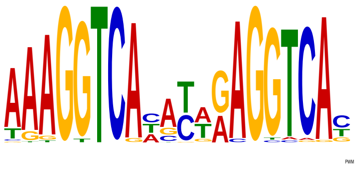	NAAGGTCNNNNNNNNGTCAN	430.22	DR:5	7
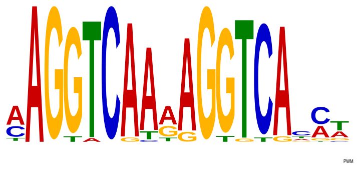	NNGGTCAAANNNTCAANN	374.12	DR:2	8
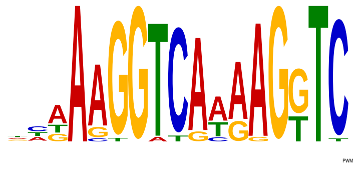	NNAAAGGTCNAAAGGNN	1163.69	DR:2	9

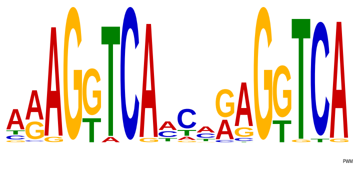	$N\overrightarrow{AAGGTCN}NNNN\overrightarrow{AGGTCN}$	267.43	DR:4	10
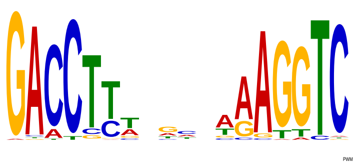	$\overleftarrow{GACCT}NNNNNNNN\overrightarrow{NGGTC}$	49.65	ER:8	11
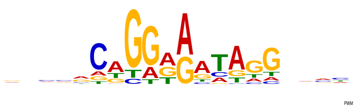	$NNNNNC\overrightarrow{AGGNNA}TAGNNNNN$	9.44	M	12
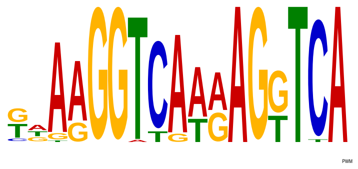	$NAA\overrightarrow{AGNNNA}AA\overrightarrow{AGGTCN}$	1137.09	DR:2	13
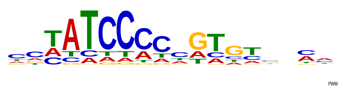	$NNNNATCCNNNGTGTNNNN$	12.44	M	14
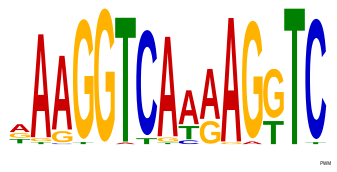	$NAA\overrightarrow{AGNNNA}AA\overrightarrow{AGGTN}$	328.42	DR:2	15
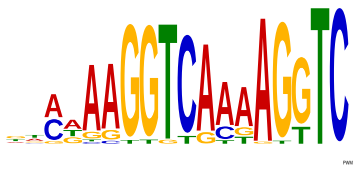	$NNNAA\overrightarrow{AGGTCN}AAAGNNN$	525.01	M	16
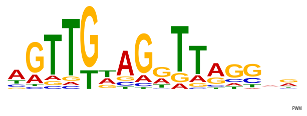	$NNN\overrightarrow{AGTTNN}NNNTTAGNNN$	8.50	M	17
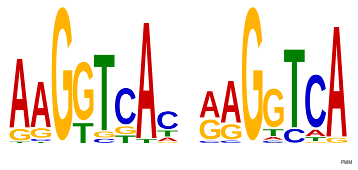	$N\overrightarrow{AGGTCA}NNNN\overrightarrow{NGGTCN}$	24.75	DR:3	18

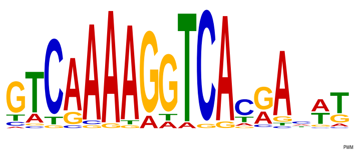	$NNNNAA\overrightarrow{AGGTCA}CGNNNN$	90.44	M	19
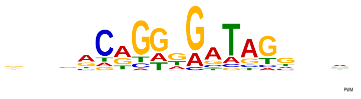	$NNNNAC\overrightarrow{AGNNNA}TAGNNNN$	12.07	M	20

162 Rarb-Jolma2013-Rnd4 (ERR194717)

PWM	Seed Sequence	Seed Seq Enrichment	Repeat	Rank
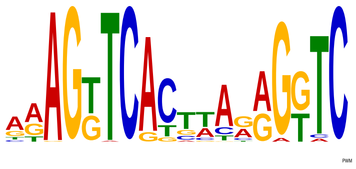	AAAGTTCANNNNNNNGGTC	499.84	DR:5	1
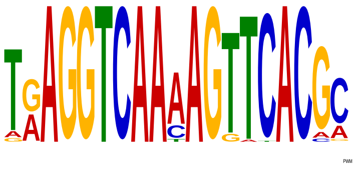	NAAGGTCNNNAGTTCACGN	6901.15	DR:2	2
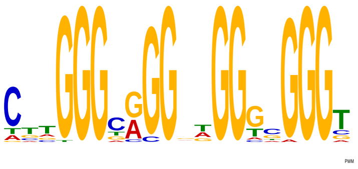	CTTGGGCNNNNNNNNNGGGT	192.60	DR:8	3
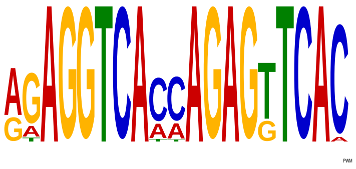	NNAGGTCACNAGAGTTCNN	2809.30	DR:4	4
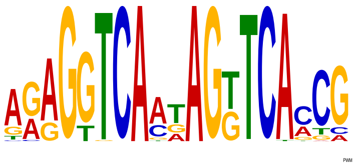	NNAGGTCNNNAGTTCAANN	861.82	DR:2	5
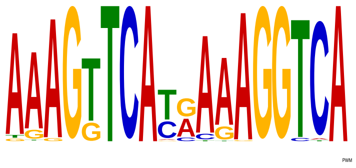	NAAGTTCANNNNAGGTCN	654.97	DR:4	6
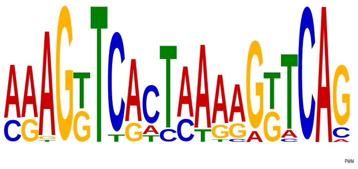	NAAGTTCANNNNNNNNGTCAN	229.34	DR:5	7
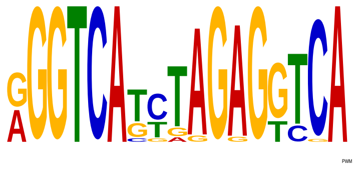	NGGTCAGNTAGAGGTCN	1313.18	DR:5	8
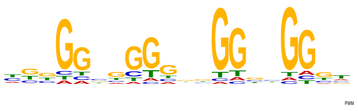	TGGGNNNNNNNNNNNNGGGT	12.95	M	9

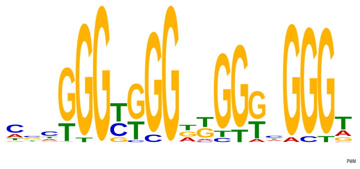	*CCCGGGNNNNNNNNNNGGGT*	52.87	M	10
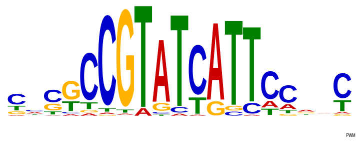	*NNNNCCGTATCATTCNNNN*	163.70	M	11
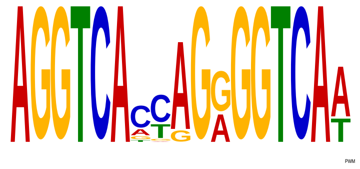	*NGGTCACTNGGGGTCAN*	771.21	DR:4	12
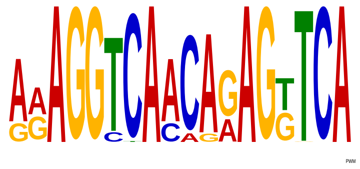	*NAAGGTCNNNAGAGTTCN*	1183.82	DR:4	13
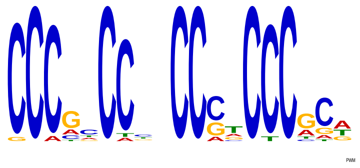	*CCCGNNNNNNNTCCCGCA*	89.25	M	14
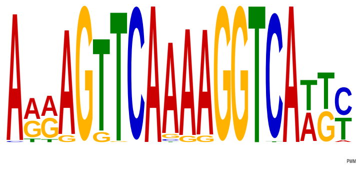	*NNNAGTTCAAANNGTCANNN*	1958.53	DR:2	15
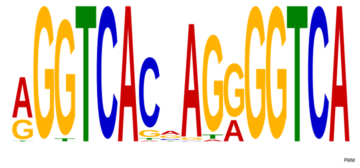	*NGGTCACGANGGGTCN*	960.58	DR:4	16
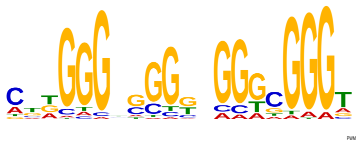	*CTTGNNNNNNNNNNNCGGGT*	24.90	M	17
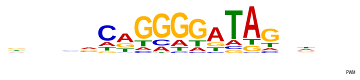	*NNNNACAGNNNATAGNNNN*	7.77	M	18

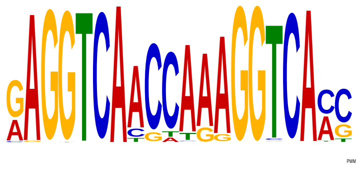	$N\overrightarrow{AGGTCN}NNNNNN\overrightarrow{NGTCA}AN$	302.75	DR:5	19
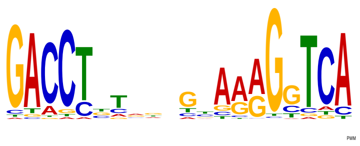	$\overleftarrow{GACCN}NNNNNNNNN\overrightarrow{NNGTCA}$	15.17	ER:9	20

163 RARB-Jolma2013-Rnd4 (ERR195056)

PWM	Seed Sequence	Seed Seq Enrichment	Repeat	Rank
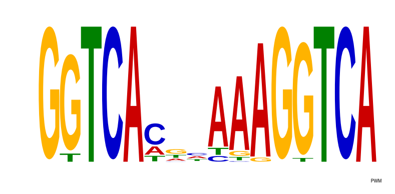	GGTCANNNAAAGGTCA	4821.39	DR:5	1
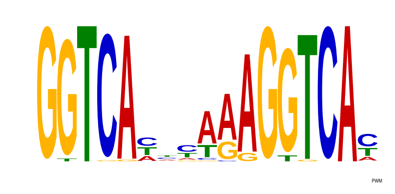	GGTCANNNNNAGGTCAC	1377.69	DR:5	2
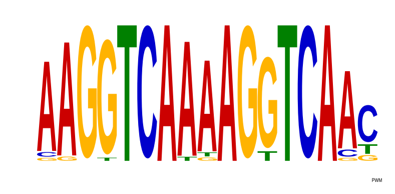	NNGGTCAAAAGGTCANN	1587.87	DR:2	3
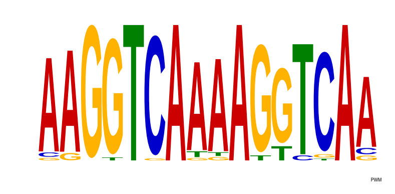	NNGGTCAANAGGTCNN	150.37	DR:2	4
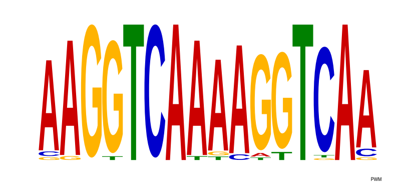	NNGGTCNAAAGGTCNN	141.61	DR:2	5
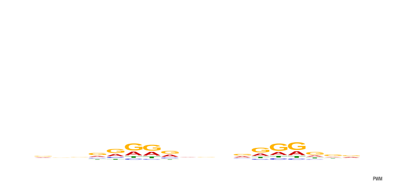	NNNGGGGNNNNNGGGGNNN	10.55	M	6
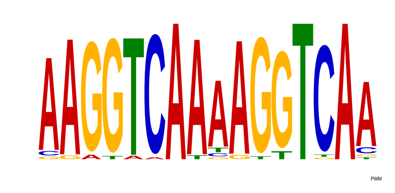	NNGGTCANAAGGTCNN	142.24	DR:2	7
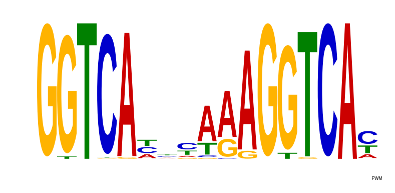	GGTCNNNNNNAGGTCAC	427.58	DR:5	8
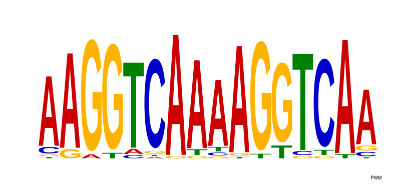	NNGGTCANNAGGTCNN	44.06	DR:2	9

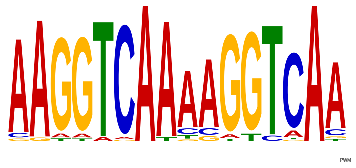	NNGGTCNNAAGGTCNN	41.36	DR:2	10
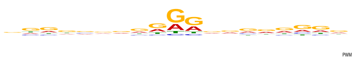	GGGGNNNNNNNNNNNGGGG	7.27	M	11
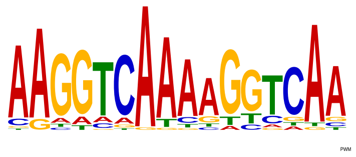	NNGGTCNNNAGGTCNN	12.89	DR:2	12
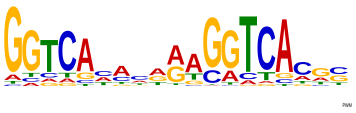	NGTCANNNNNNNNNCACGN	9.42	M	13
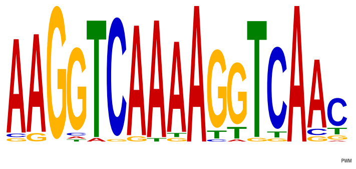	NNNGTCAANAGGTCNNN	46.30	DR:2	14
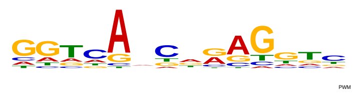	GGTCNNNNNNGGTC	3.15	DR:4	15
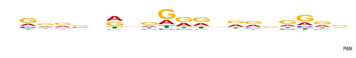	GGGGNNNNNNNNNNNNGGGG	6.75	M	16
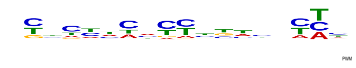	NNTCTTNNNNNNTCCCNN	2.80	M	17
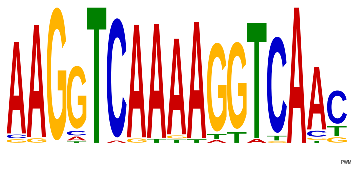	NNNGTCANAAGGTCNNN	44.67	DR:2	18

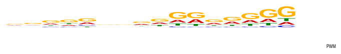	$NNGGGGNNNNNNN\overrightarrow{AGGGNN}$	5.79	M	19
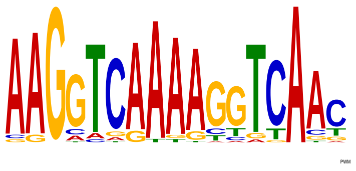	$N\overrightarrow{NNGTCA}NN\overrightarrow{AGGTCN}NN$	13.11	DR:2	20

164 Rarb-Jolma2013-Rnd5 (ERR194718)

PWM	Seed Sequence	Seed Seq Enrichment	Repeat	Rank
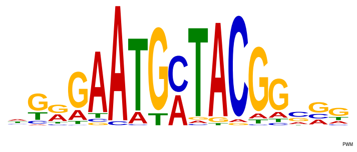	*NNNGAATNNTACGGNNN*	33.79	M	1
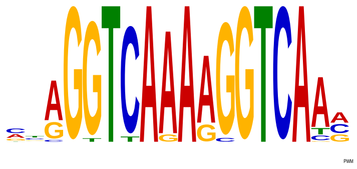	*NCAGGTCNNNNNNTCAAN*	100.68	DR:2	2
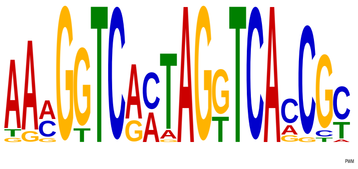	*NNNGGTCNNNNGTTCAANNN*	64.87	DR:2	3
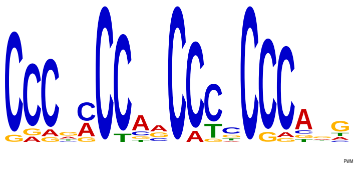	*CCCGNNNNNNNNNNCCAGG*	21.97	M	4
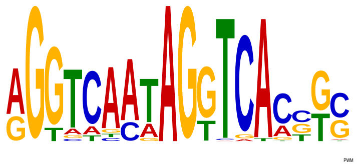	*NNNTCAANNNTTCAANNN*	27.90	DR:2	5
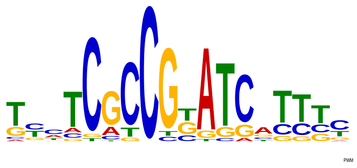	*NNNTCGCNNNATCATNNN*	21.32	M	6
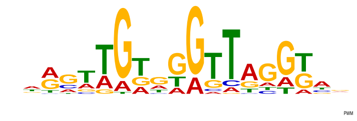	*NNNNTTGTNNNTTAGNNNN*	8.04	M	7
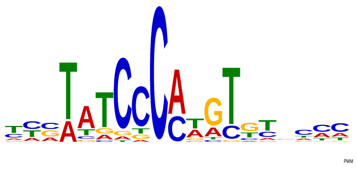	*NNNNATCCNNNGTGTNNNN*	13.08	M	8
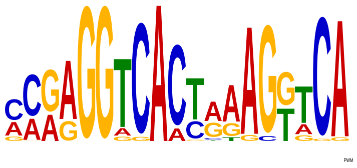	*NNNNGGTCNNCAGAGNNNN*	20.01	M	9

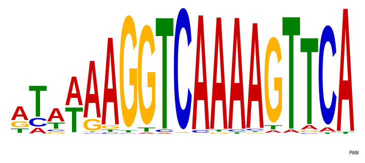	NNATAANNNNNNNAGTTNN	40.03	M	10
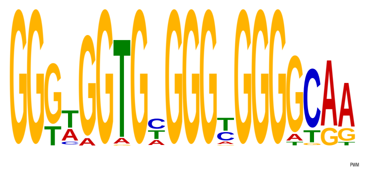	NNNTGGTGTNNNTGGGGNNN	57.67	M	11
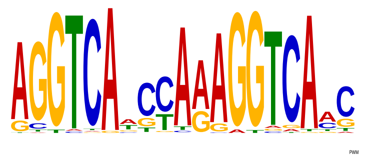	NGGTCNNNNNNNNNNTCAAN	25.22	DR:5	12
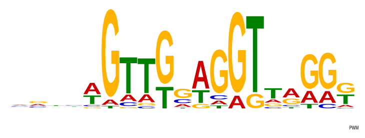	NNNNAGTTNNAGGTTNNNN	21.60	DR:0	13
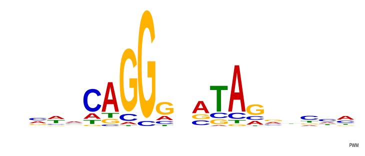	NNNNCAGGNNNTAGGNNNN	7.43	DR:1	14
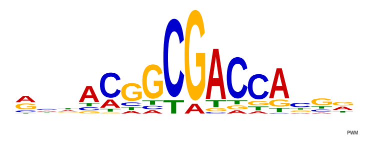	NNNACGGNNACCANNN	8.57	M	15
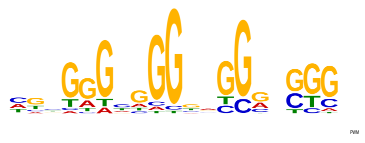	AGCGNNNNNNNNNNNNGGGT	5.45	M	16
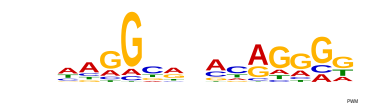	NNNAGGCNNACAGNNN	6.55	M	17
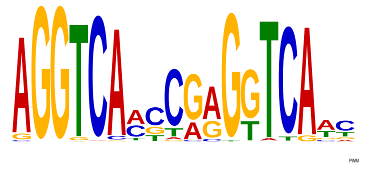	NNNTCAACNNNGGTCNNN	71.04	DR:4	18

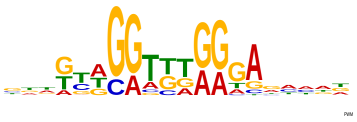	$NNNNT\overrightarrow{AGGTNN}NGGAGNNNN$	8.50	M	19
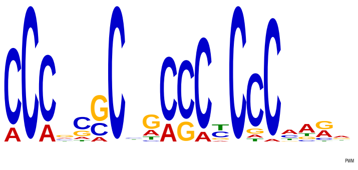	*NCCGCNNNNNNNNNCCAAAN*	13.08	M	20

165 RARB-Yin2017-Rnd2 (ERR1005800)

PWM	Seed Sequence	Seed Seq Enrichment	Repeat	Rank
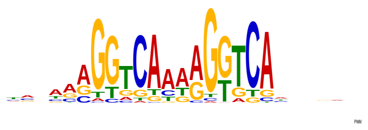	NNNNNNGGTCAAANGGTCANNNNNN	80.98	DR:2	1
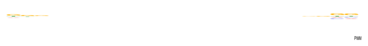	NNNNNNGGGGNNNNNGGGGNNNNNN	3.41	M	2
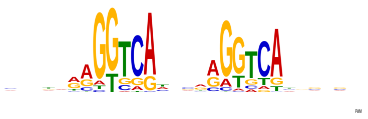	NNNNNNAGGTCANNNNNGGTCANNNNNN	23.54	DR:4	3
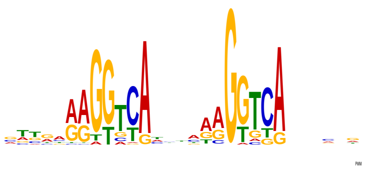	NNNNNNAGGTCANNNNNAGGTCANNNNNN	48.50	DR:5	4
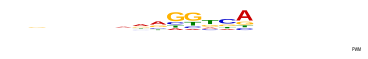	NNNNNNAAAGGTCANNNNNN	3.14	M	5
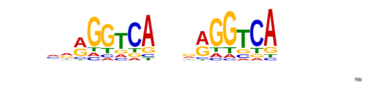	NNNNNNGGTCANNNNGGTCANNNNNN	10.11	DR:3	6
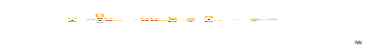	GGGGNNNNNNNNNNNNNNNNNNNNNNNNNNNNNNNNGGGG	3.26	M	7
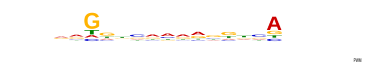	NNNNNNGTCANNGGTCNNNNNN	2.50	DR:2	8
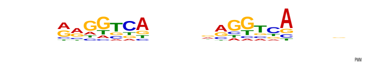	NNNNNNGGTCANNNNNNGGTCNNNNNN	4.53	DR:5	9

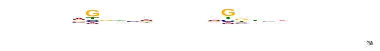	NNNNNNGTCANNNNNNNGTCANNNNNNN	2.13	DR:4	10
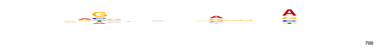	NNNNNNGTCANNNNNGGTCNNNNNN	2.01	DR:3	11
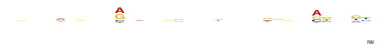	NNNNNNGGTCNNNNNNNNNNNNNNNNNNGGTCNNNNNN	1.75	DR:14	12
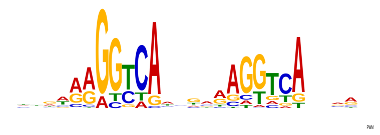	NNNNNNGGTCANNNNAAGGTCNNNNNN	15.31	DR:5	13
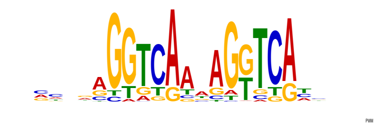	NNNNNNGGTCAANAGTTCNNNNNN	20.71	DR:2	14
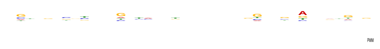	GTTGNNNNNNNNNNNNNNNNNNNNNNNNNNNNNNNNNNNNNTGTG	2.28	M	15
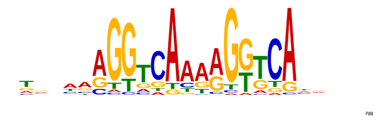	NNNNNNGGTCNAAAGGTCNNNNNN	21.53	DR:2	16
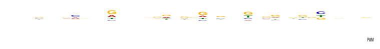	CGGGNNNNNNNNNNNNNNNNNNNNNNNNNNNNNNNNNNNNNGCGG	2.01	M	17
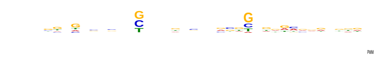	NTCGGNNNNNNNNNNNNNNNNNNNNNNNNNNNNNNNNNNNTGGGN	2.11	M	18

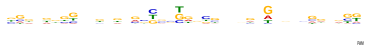	$NNGTGGNNNNNNNNNNNNNNNNNNNNNNNNNNN\overrightarrow{NGGTGN}N$	2.66	M	19
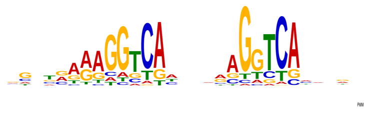	$NNNNNNA\overrightarrow{AGGTCA}NNNNN\overrightarrow{NGGTCN}NNNNN$	16.72	DR:5	20

166 RARB-Yin2017-Rnd2 (ERR1005801)

PWM	Seed Sequence	Seed Seq Enrichment	Repeat	Rank
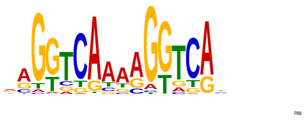	NNNNNNGGTCAAANGGTCANNNNNN	121.24	DR:2	1
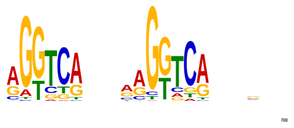	NNNNNNAGGTCANNNNAGGTCANNNNNN	77.48	DR:4	2
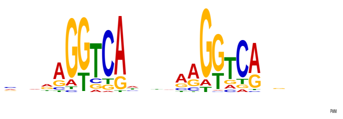	NNNNNNAGGTCANNNNNAGGTCANNNNNN	74.64	DR:5	3
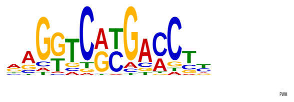	NNNNNNAGGTCNNGACCTNNNNNN	18.73	IR:0	4
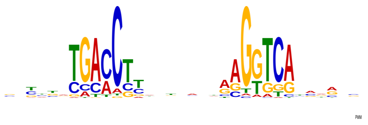	NNNNNNTGACCTNNNNNNNNNAGGTCANNNNNN	47.17	ER:9	5
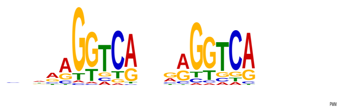	NNNNNNAGGTCANNNNGGTCANNNNNN	29.00	DR:3	6
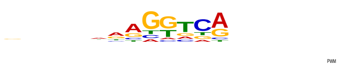	NNNNNNAAAGGTCANNNNNN	5.05	M	7
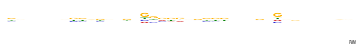	GGGGNNNNNNNNNNNNNNNNNNNNNNNNNNNNNNNNNNGGGG	3.03	M	8
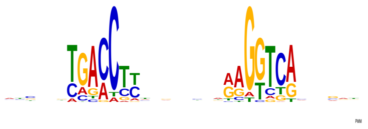	NNNNNNTGACCTNNNNNNNNNNAGGTCANNNNNN	55.48	ER:10	9

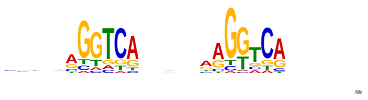	NNNNNNGGTCANNNNNNAGGTCANNNNNNN	20.41	DR:6	10
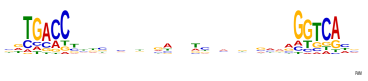	NNTGACCNNNNNNNNNNNNNNNNNNNNNNNNGGTCANN	12.91	ER:22	11
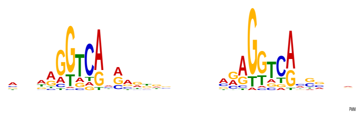	NNNNNNGGTCANNNNNNNNNNNNNNAGGTCANNNNNN	20.78	DR:14	12
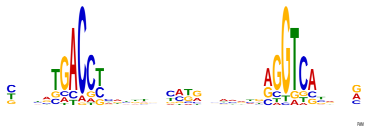	NNNNNTGACCNNNNNNNNNNNNNNNNNNNNGGTCANNNNN	20.97	ER:18	13
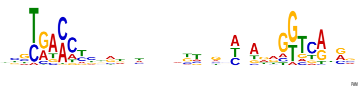	NNNTGACCTNNNNNNNNNNNNNNNNNNNNGGTCANNN	14.59	ER:19	14
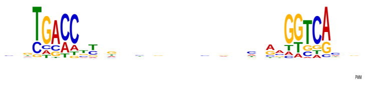	NNNTGACCNNNNNNNNNNNNNNNNNNNNNNGGTCANNN	13.64	ER:20	15
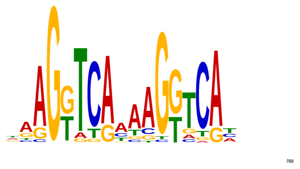	NNNNNNAAGTTCANAAGGTCNNNNNN	148.25	DR:2	16
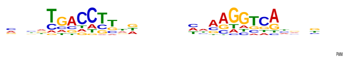	NNNNNNTGACCNNNNNNNNNNNNNGGTCANNNNNN	7.74	ER:11	17
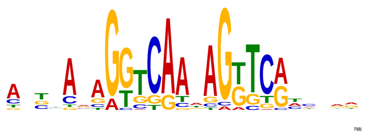	NNNNNNAGGTCAATAGTTCNNNNNN	78.70	DR:2	18

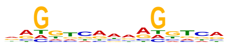	$NNNN\overrightarrow{NNGTCA}NN\overrightarrow{NNGTCA}NNNNNNN$	3.64	DR:2	19
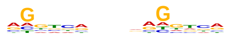	$NNNN\overrightarrow{NNGTCA}NNNN\overrightarrow{NNGTCA}NNNNNNN$	3.10	DR:4	20

167 RARB-Yin2017-Rnd3 (ERR1005802)

PWM	Seed Sequence	Seed Seq Enrichment	Repeat	Rank
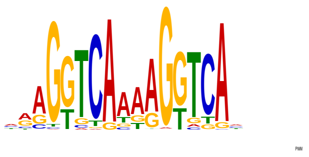	NNNNNNAGGTCNAAAGGTCNNNNNN	611.17	DR:2	1
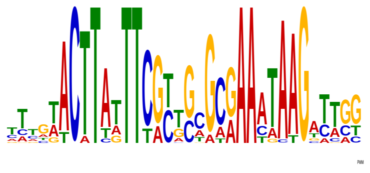	NNNNNNCTTATTTNNNNNNNNNAAATAANNNNNN	975.67	ER:9	2
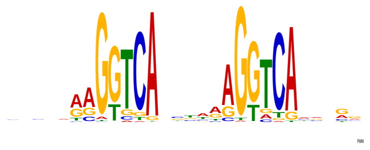	NNNNNNAGGTCANNNNNAGGTCNNNNNN	176.61	DR:5	3
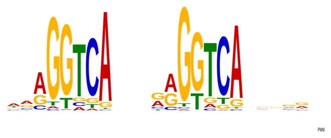	NNNNNNGGTCANNNNNGGTCANNNNNN	74.14	DR:4	4
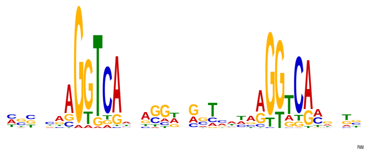	NNNNNNAGGTCANNNNNNNNNNNNNNAGGTCNNNNNN	70.23	DR:14	5
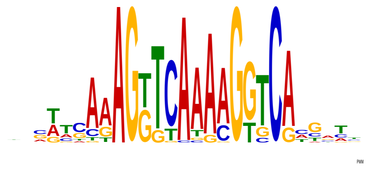	NNNNNNAAAGTTNAAAAGGTCNNNNNN	1832.84	DR:2	6
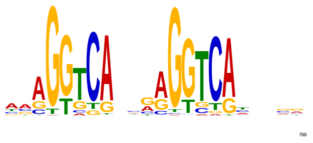	NNNNNNAGGTCANNNAGGTCNNNNNN	93.14	DR:3	7
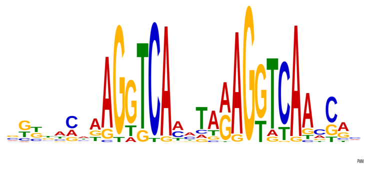	NNNNNNAAAGGTCNNNNAAAGGTCNNNNNN	917.65	DR:5	8
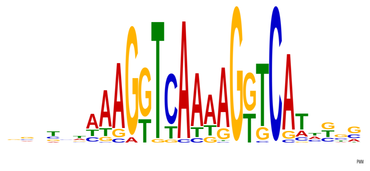	NNNNNNAAAGGTNAAAAGTTCNNNNNN	1316.43	DR:2	9

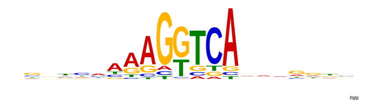	*NNNNNNAAAGNTCAANNNNNN*	10.79	M	10
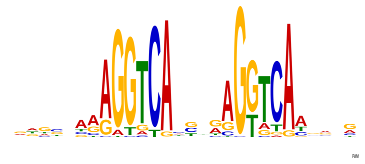	*NNNNNNAAAGGTCNNNNNAGGTCNNNNNN*	199.20	DR:4	11
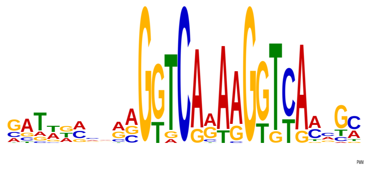	*NNNNNNAAAAGGTCNAAAGGTNNNNNN*	677.91	DR:2	12
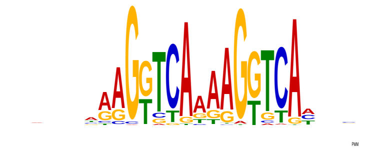	*NNNNNNAAAGGNNAAAAGGTNNNNNN*	226.05	DR:2	13
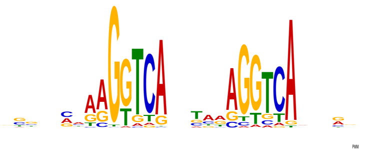	*NNNNNNAAAGGTCNNNNNNNGGTCNNNNNN*	87.04	DR:5	14
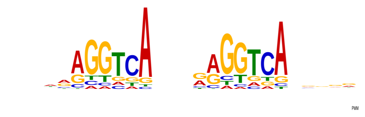	*NNNNNNGGTCNNNNNNGGTCNNNNNN*	10.03	DR:4	15
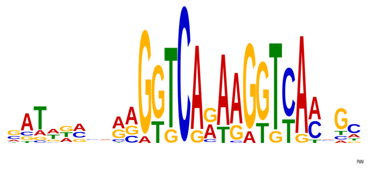	*NNNNNNAAAAGGNCAAAAGGTNNNNNN*	617.58	DR:2	16
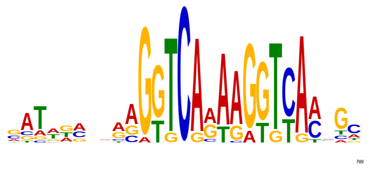	*NNNNNNAAAAGGNNAAAAGGTNNNNNN*	207.78	DR:2	17
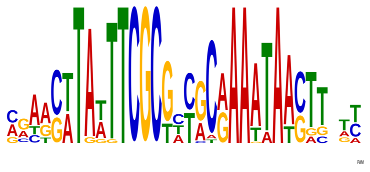	*NNNNNNTAATTCGNNNNNNNAAATAANNNNNN*	766.65	DR:7	18

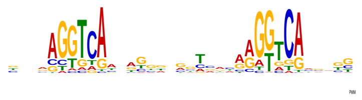	$NNNNN\overrightarrow{NGGTCA}NNNNNNNNNNNNNN\overrightarrow{AGGTCN}NNNNN$	30.72	DR:14	19
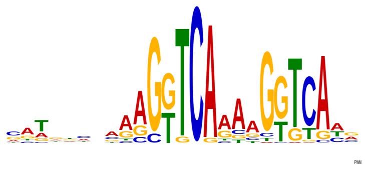	$NNNNNNAAA\overrightarrow{AGNTCA}AAAGNNNNNN$	133.53	M	20

168 RARB-Yin2017-Rnd3 (ERR1005803)

PWM	Seed Sequence	Seed Seq Enrichment	Repeat	Rank
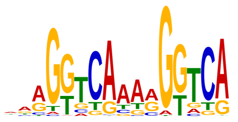	NNNNNNGGTCAAANGGTCANNNNNN	509.17	DR:2	1
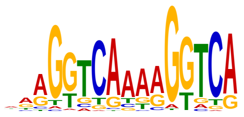	NNNNNNGTCAAANNGTCANNNNNN	109.09	DR:2	2
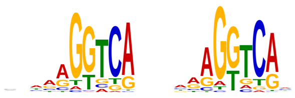	NNNNNGGTCANNNNNAGGTCNNNNNN	71.20	DR:5	3
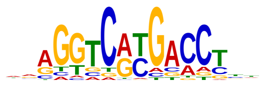	NNNNNNGGTCNNGACCNNNNNN	16.67	IR:0	4
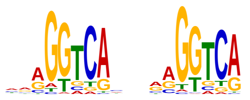	NNNNNNGGTCANNNNNGGTCANNNNNN	65.04	DR:4	5
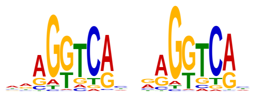	NNNNNNAGGTCNNNNAGGTCNNNNNN	51.61	DR:3	6
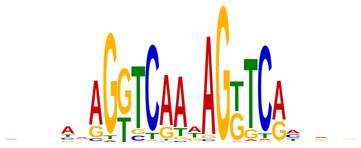	NNNNNNAGGTCNATAGTTCNNNNNN	206.23	DR:2	7
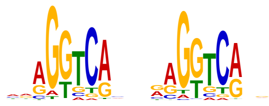	NNNNNNAGGTCNNNNNAGGTCNNNNNN	68.95	DR:4	8
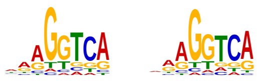	NNNNNNGTCANNNNNNNGTCANNNNNN	12.83	DR:5	9

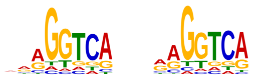	NNNNNNNGTCANNNNNNNGTCANNNNNNN	12.88	DR:4	10
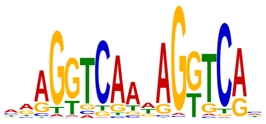	NNNNNNNGTCAANAGTTCNNNNNN	58.80	DR:2	11
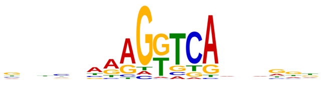	NNNNNNAAAGNTCAANNNNNNN	12.14	M	12
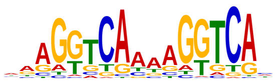	NNNNNNAGGTNNAAAGNNNNNN	14.53	M	13
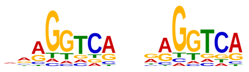	NNNNNNNGTCANNNNNNGTCANNNNNN	9.40	DR:3	14
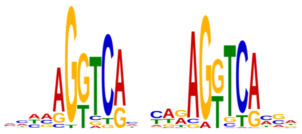	NNNNNNAGGTCNNNNAAAGTTCANNNNNN	287.78	DR:5	15
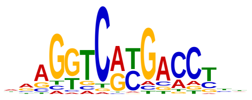	NNNNNNAGGTNNNGACCNNNNNN	13.23	IR:0	16
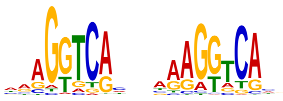	NNNNNNAGGTCNNNNAAAGGNNNNNN	43.61	DR:5	17
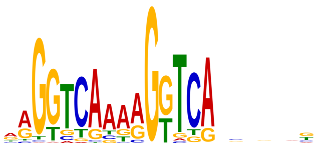	NNNNNNNGTCAAAANNTCAGNNNNNN	65.32	DR:2	18

169 RARB-Yin2017-Rnd4 (ERR1005804)

PWM	Seed Sequence	Seed Seq Enrichment	Repeat	Rank
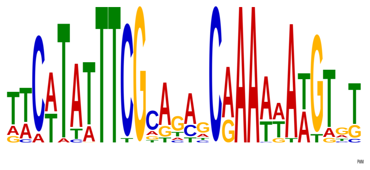	NNNTTAATTCGNNNNNNGAATTAAGNNN	45147.66	DR:7	1
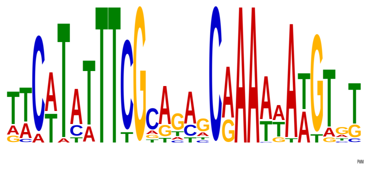	NNNTTAATTCNNNNNNNGAATTAAGNNN	14199.01	DR:7	2
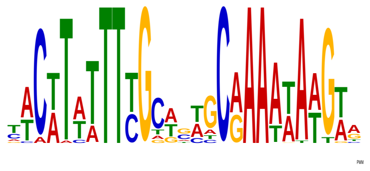	NNNTTAATTCNNNNNNCGAATTAANNN	18647.73	DR:7	3
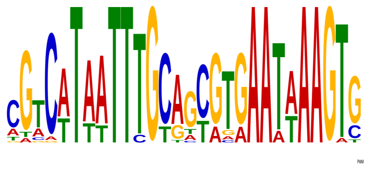	NNNGTTATTTNNNNNNNNNAAATAANNN	414.39	DR:12	4
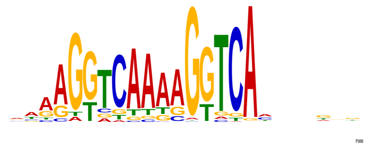	NNNNNNTCAAANNNTCAANNNNNN	67.91	DR:2	5
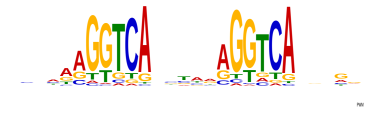	NNNNNNGGTCNNNNNNNGGTCNNNNNN	21.98	DR:5	6
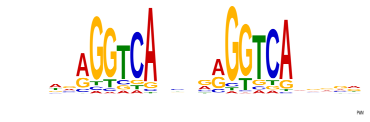	NNNNNNGGTCNNNNNNGGTCNNNNNN	19.62	DR:4	7
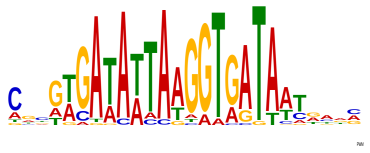	NNNNNNATAATANGGTGATANNNNNN	256.61	M	8
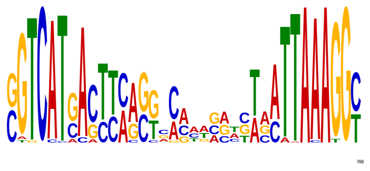	NGTCATNNNNNNNNNNNNNNNNNNNNNTTAAAGGN	151.11	DR:26	9

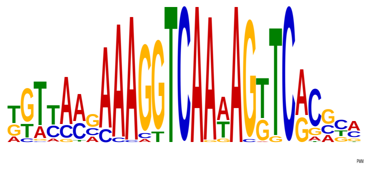	$NNNNNNGAA\overrightarrow{AGNNN}AAT\overrightarrow{AGTTN}NNNNN$	109.62	DR:2	10
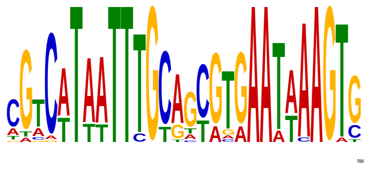	$N\overrightarrow{NNGTTA}TTNNNNNNNNNN\overrightarrow{AAATAA}NNN$	92.37	DR:12	11
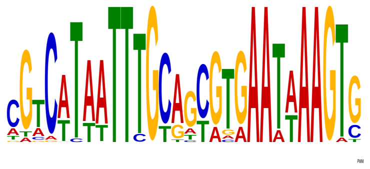	$N\overrightarrow{NNGTTA}TTTNNNNNNNNNNAATAANNN$	91.46	M	12
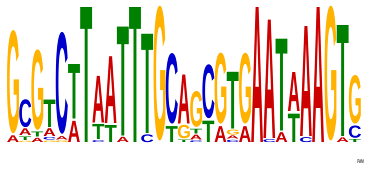	$NN\overrightarrow{NNGTTA}TTTNNNNNNNNNAAATANNNN$	112.68	M	13
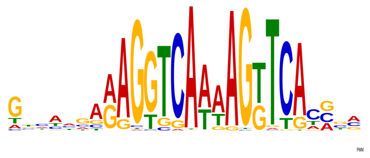	$NNNNNNAA\overrightarrow{AGNNN}AAT\overrightarrow{AGTTN}NNNNN$	75.44	DR:2	14

170 RARB-Yin2017-Rnd4 (ERR1005805)

PWM	Seed Sequence	Seed Seq Enrichment	Repeat	Rank
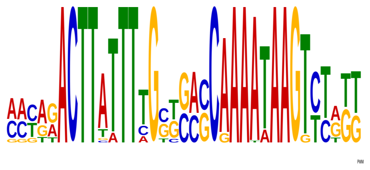	NNNNNNCTTATTTTNNNNNNNNNAAATAAGNNNNNN	70779.50	ER:9	1
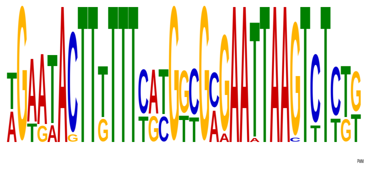	NNNNNNCTTTTTTNNNNNNNNGAATTAAGNNNNNN	18198.21	M	2
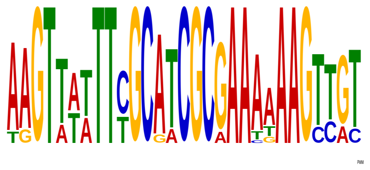	NNNNTTTTTCGNNNNNNGAAATAAGNNNN	7357.20	M	3
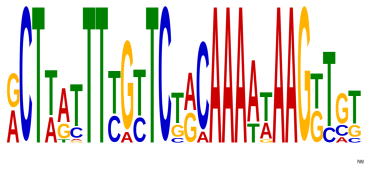	NNNTATTTCGNNNNNNNAATTAAGTNNN	6420.55	DR:11	4
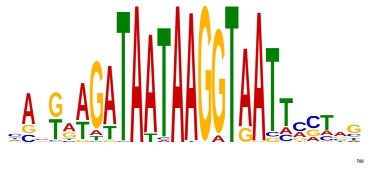	NNNNNNGATAATAAGGTAATNNNNNN	4321.44	DR:2	5
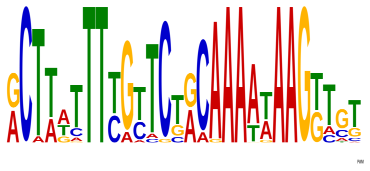	NNNTATTTCNNNNNNNNAATTAAGTNNN	2364.46	DR:11	6
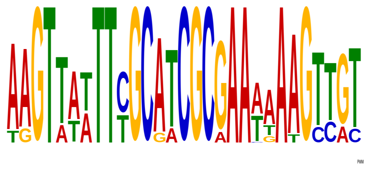	NNNNTTTTTCNNNNNNNGAAATAAGNNNN	2487.01	M	7
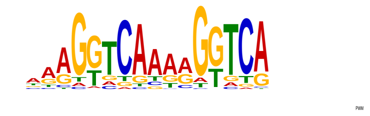	NNNNNNTCAANNNGTCANNNNNN	35.66	DR:2	8
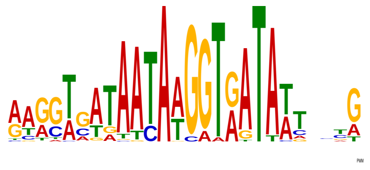	NNNNNNATAATAAGGTGATANNNNNN	1445.90	M	9

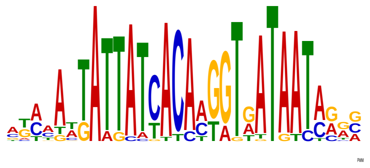	NNNNNNTATTATCANNNNNNTGATANNNNNN	545.55	IR:5	10
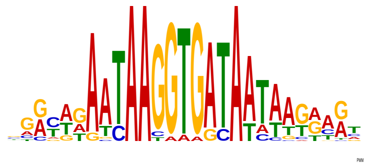	NNNNNNAATAAGGNGATAATANNNNNN	1233.55	M	11
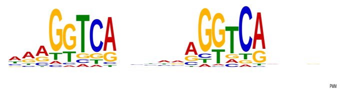	NNNNNNGTCANNNNNNGGTCNNNNNN	16.05	DR:5	12
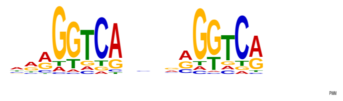	NNNNNNGTCANNNNNGGTCNNNNNN	15.05	DR:4	13
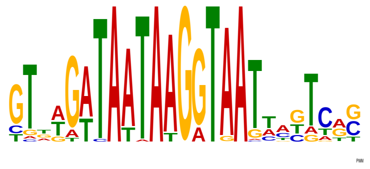	NNNNNNTAATAAGGTAATCNNNNNN	339.27	M	14
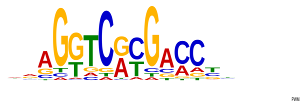	NNNNNNAGGTNNNGACCNNNNNN	13.16	IR:0	15
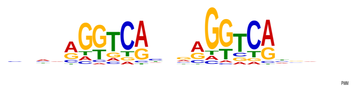	NNNNNNGGTCNNNNNGGTCNNNNNN	9.94	DR:3	16
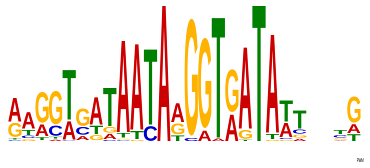	NNNNNNATAATAAGNTGATANNNNNN	611.83	M	17
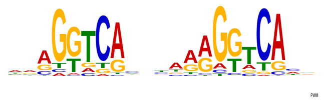	NNNNNNGGTCNNNNAAAGNNNNNN	14.91	M	18

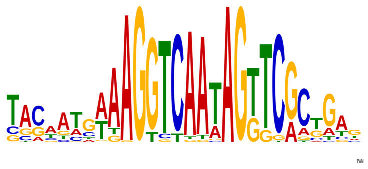	$NNNNNNGAA\overrightarrow{AGNNNA}AT\overrightarrow{AGTTCN}NNNNN$	478.00	DR:2	19
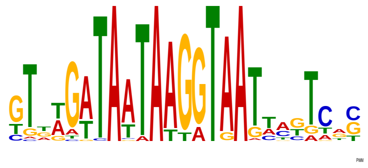	$NNNNNNTAATA\overrightarrow{AGNTAA}TCNNNNNN$	120.66	M	20

171 RARB-8-Bhimsaria2022-Rnd3 (S07-098)

PWM	Seed Sequence	Seed Seq Enrichment	Repeat	Rank
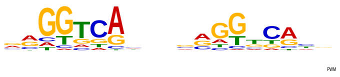	NNNGGTCNNNNNNGGTCNNN	16.47	DR:4	1
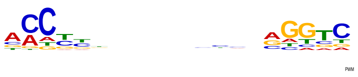	ACCTNNNNNNNNNNNNGGTC	21.54	ER:11	2
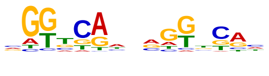	NNNNGGTCANNNGGTCNNNN	28.77	DR:2	3
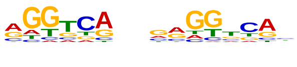	NNNGGTCNNNNNGGTCNNN	15.19	DR:3	4
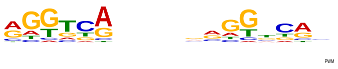	NNGGTCNNNNNNNGGTCNN	13.02	DR:5	5
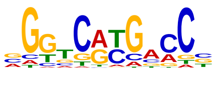	NNNNNGGTCATGACCNNNNN	73.36	IR:0	6
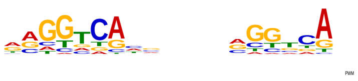	NNGGTCANNNNNNNGGTCNN	15.63	DR:6	7
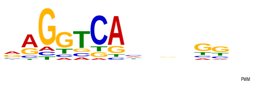	NNNNNNAAGGTCAANNNNNN	19.61	M	8
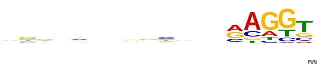	AAGGNNNNNNNNNNNNAAGGT	14.98	DR:9	9

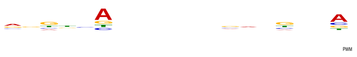	NGGTCNNNNNNNNNGGTCN	4.27	DR:7	10
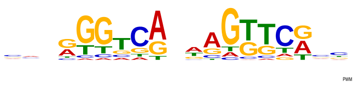	NNNGGGTCNNNAGTTCNNN	37.33	DR:2	11
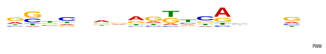	NNNNNNAAGAGTTCNNNNNN	6.09	M	12
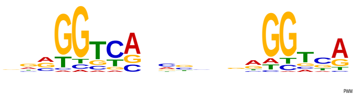	NGGGGTCNNNNNNNNGGTCN	25.21	DR:6	13
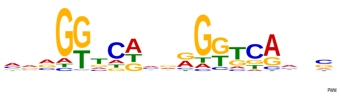	NNNNGGTCNNGGGGTCNNNN	24.75	DR:2	14
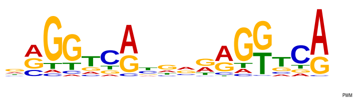	NNGGTCNNNNGAGTTCNN	25.16	DR:4	15
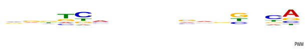	NNGGGGNNNNNNNNGGGTNN	5.17	M	16
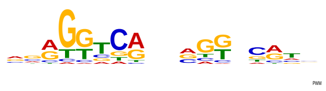	NNNGGGGTCNNNNGGTCNNN	26.05	DR:2	17
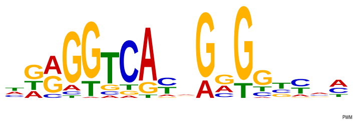	NNNGGTCACAGGGGTNNN	83.67	DR:3	18

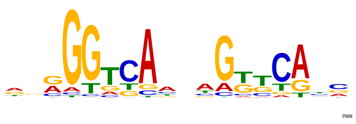	$NN\overrightarrow{NGGTCA}AN\overrightarrow{AGTGCN}NN$	58.04	DR:2	19
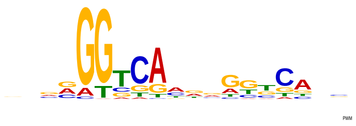	$NNN\overrightarrow{NGGTCA}AG\overrightarrow{GGGTNN}NN$	43.48	DR:2	20

172 RARG-Bhimsaria2022-Rnd2 (S07-033)

PWM	Seed Sequence	Seed Seq Enrichment	Repeat	Rank
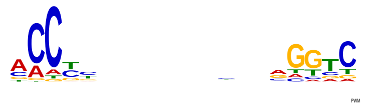	ACCTNNNNNNNNNNNNGGTC	28.17	ER:11	1
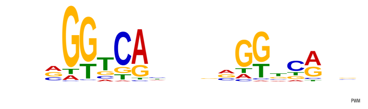	NNNGGTCANNNNNGGTCNNN	37.88	DR:4	2
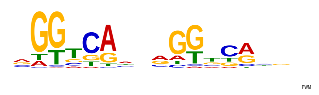	NNNNGGTCANNNGGTCNNNN	39.20	DR:2	3
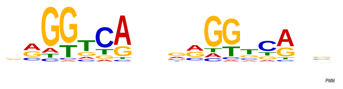	NNNGGTCNNNNNGGTCNNN	16.73	DR:3	4
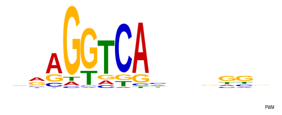	NNNNNNAAGGTCAANNNNNN	24.79	M	5
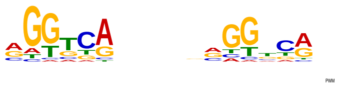	NNGGTCNNNNNNNGGTCNN	13.26	DR:5	6
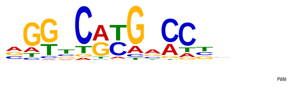	NNNNNGGTCATGACCNNNNN	68.16	IR:0	7
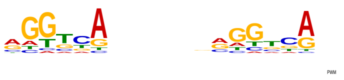	NNGGTCNNNNNNNNGGTCNN	7.11	DR:6	8
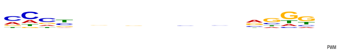	GACCNNNNNNNNNNNNGGTC	3.76	ER:10	9

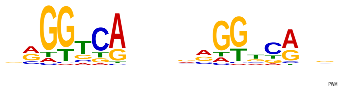	NNNGGTCNNNNNNNGGTCNNN	17.16	DR:4	10
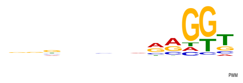	CGGGNNNNNNNNNNNNNAGGT	8.52	M	11
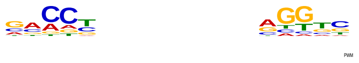	GACCNNNNNNNNNNNNGGTC	8.62	ER:9	12
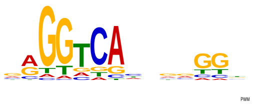	NNNNNNGGGGTCAANNNNNN	16.84	M	13
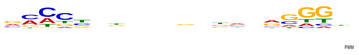	GACCTNNNNNNNNNNNGGTC	5.88	ER:10	14
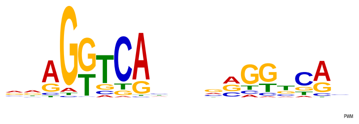	NNAGTTCANNNNNNGGTCNN	33.71	DR:4	15
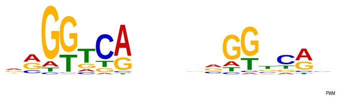	NNAGGTCNNNNNNGGTCNN	26.80	DR:4	16
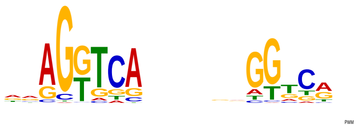	NNAGTTCANNNNNNNGGTCNN	37.49	DR:5	17
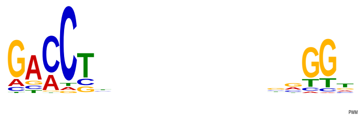	GACCTTNNNNNNNNNNGGGT	77.86	ER:11	18

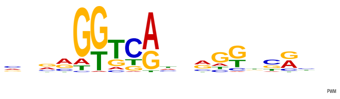	$NNNA\overrightarrow{AGGTCN}NN\overrightarrow{NGGTCN}NN$	24.41	DR:2	19
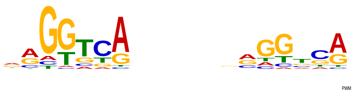	$N\overrightarrow{AGGTCN}NNNNNN\overrightarrow{NGGTCN}$	15.23	DR:6	20

173 RARG-Bhimsaria2022-Rnd3 (S07-153)

PWM	Seed Sequence	Seed Seq Enrichment	Repeat	Rank
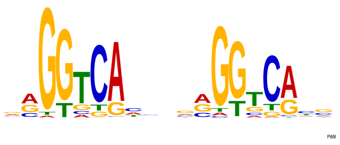	NNNGGTCANNNNNGGTCNNN	424.32	DR:4	1
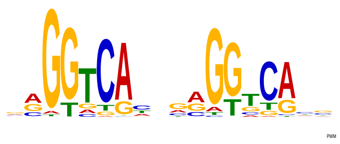	NNNGGTCACNNNGGTCNNN	764.82	DR:3	2
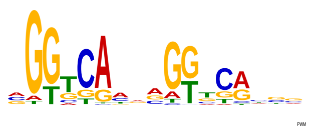	NNNNGGTCAANNGGTCNNNN	717.89	DR:2	3
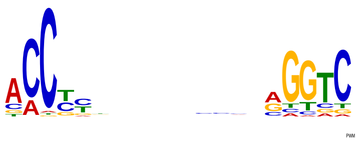	ACCTNNNNNNNNNNNGGTC	211.98	ER:11	4
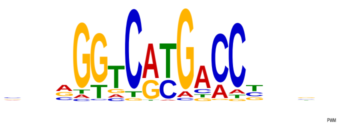	NNNNNGGTCATGACCNNNNN	1014.74	IR:0	5
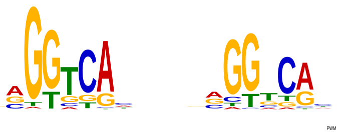	NNGGTCANNNNNNGGTCNN	228.51	DR:5	6
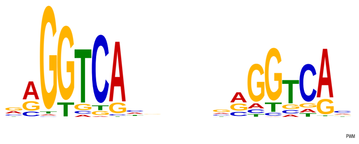	NNGGTCANNNNNNNGGTCNN	112.63	DR:6	7
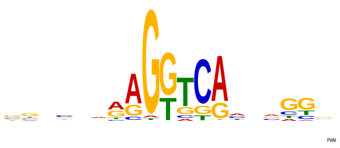	NNNNNNAAAGGTCANNNNNN	144.56	M	8
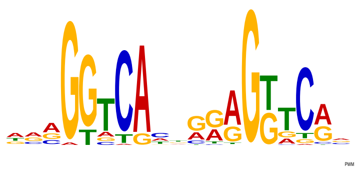	NNAGGTCACNNGAGTTCNN	2286.24	DR:4	9

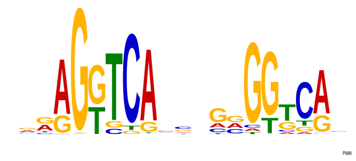	$NNG\overrightarrow{AGTTCA}NNNNN\overrightarrow{NGGTCN}N$	503.49	DR:4	10
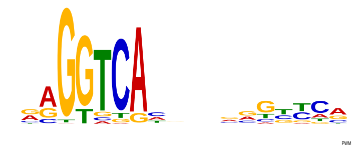	$NNN\overrightarrow{NGGTCA}CNNNGGGGNNN$	238.00	M	11
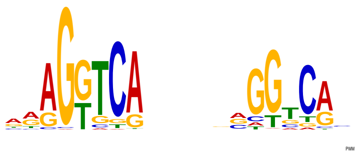	$NN\overrightarrow{AGTTCA}NNNNNN\overrightarrow{NGGTCN}N$	227.63	DR:5	12
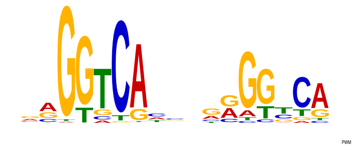	$NN\overrightarrow{NGGTCA}TNNG\overrightarrow{GGGTNN}N$	468.94	DR:4	13
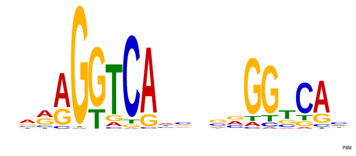	$NNA\overrightarrow{AGGTCN}NNNN\overrightarrow{CGGTCN}N$	402.45	DR:4	14
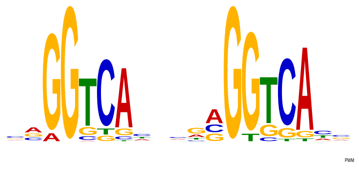	$N\overrightarrow{CGGTCA}CNNNN\overrightarrow{NGGTCA}AN$	1797.86	DR:4	15
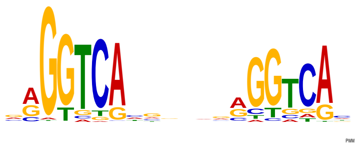	$N\overrightarrow{NGGTCA}CNNNNN\overrightarrow{NGGTCN}N$	179.10	DR:6	16
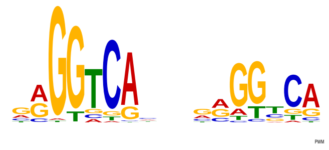	$NNG\overrightarrow{GGGTCN}NNNN\overrightarrow{GGGTNN}$	208.76	DR:4	17
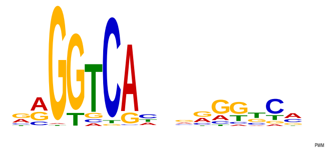	$NNN\overrightarrow{NGGTCA}TNGGGGNNNN$	285.98	M	18

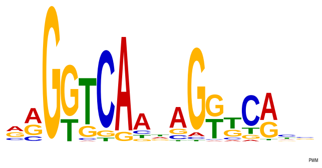	$NNN\overrightarrow{AGGTCA}AN\overrightarrow{AGTGCN}NN$	896.06	DR:2	19
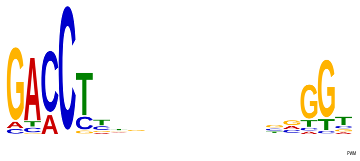	$\overleftarrow{GACCT}TTGNNNNNNNN\overrightarrow{GGGT}$	1584.36	ER:11	20

174 RARG-Jolma2013-Rnd2 (ERR195454)

PWM	Seed Sequence	Seed Seq Enrichment	Repeat	Rank
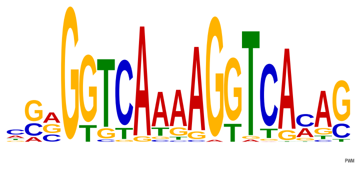	NNNGGTCAAAAGGTCANNN	8149.83	DR:2	1
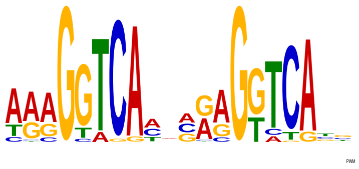	NNAGGTCANNNNNAGGTCANN	2063.03	DR:4	2
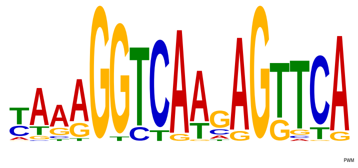	NAAAGGTCANNAGTTCN	2182.33	DR:2	3
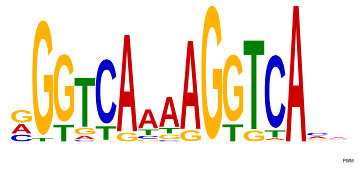	NGGTCAAAAGGTCAGN	5903.71	DR:2	4
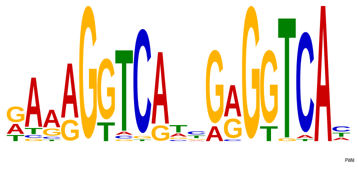	NAAAGGTCANNNNGGTCAN	3240.23	DR:3	5
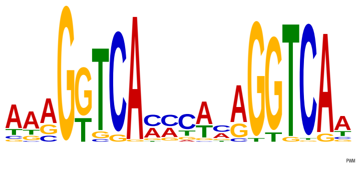	NNAGGTCANNNNNNAGGTCNN	543.54	DR:5	6
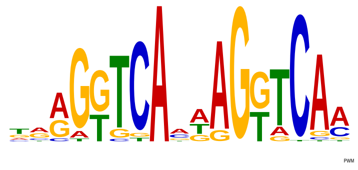	NGAGGTCANNAGTTCAN	2949.78	DR:2	7
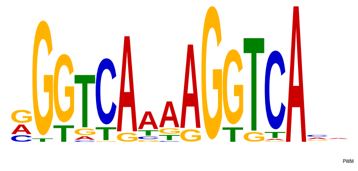	NGGTCAAAANGTCAGN	2131.29	DR:2	8
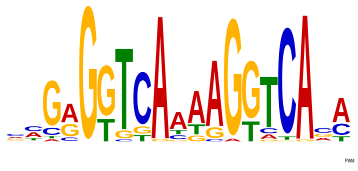	NNNAGGTCAAANGGTCNNN	1495.79	DR:2	9

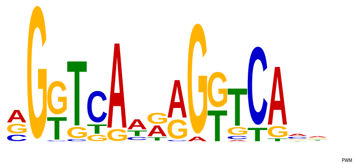	NNGTCAAAAGNTCATNN	490.45	DR:2	10
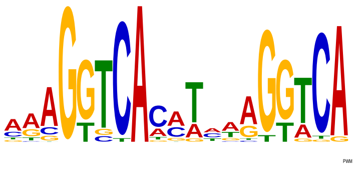	NGAGGTCANNNNNAGGTCN	590.08	DR:5	11
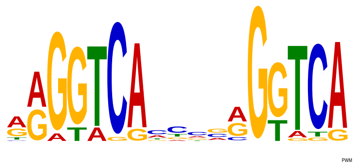	NAGGTCAGNNNAGGTCN	306.78	DR:4	12
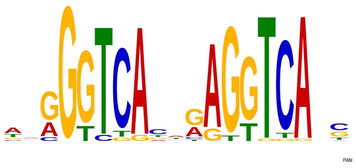	NNNGGTCACNNAGGTCNNN	437.42	DR:3	13
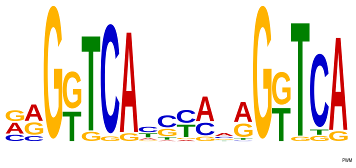	NAGGTCANNNNGAGGTCN	369.57	DR:5	14
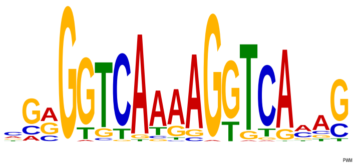	NNNNNGTCAAAAGGTCNNNN	924.30	DR:2	15
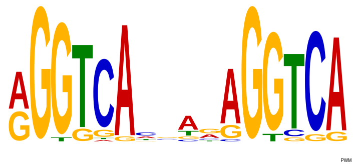	NGGTCACGNNAGGTCN	484.95	DR:4	16
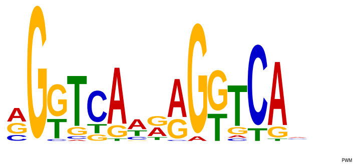	NNGTCAAAANNTCATNN	138.53	DR:2	17
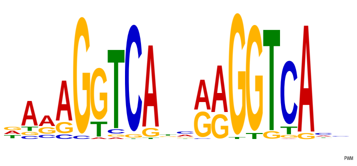	NNNAGGTCANNNNGGTCNNN	206.89	DR:3	18

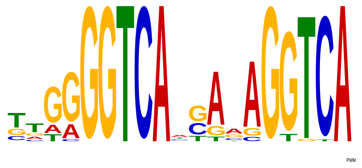	$NTG\overrightarrow{GGGTCA}NNNN\overrightarrow{AGGTCN}$	2063.38	DR:4	19
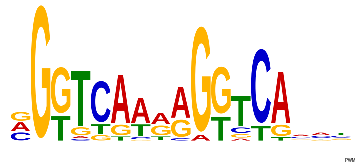	$\overrightarrow{NNGTCA}AA\overrightarrow{NNGTCA}TNN$	142.32	DR:2	20

175 Rarg-Jolma2013-Rnd2 (ERR195722)

PWM	Seed Sequence	Seed Seq Enrichment	Repeat	Rank
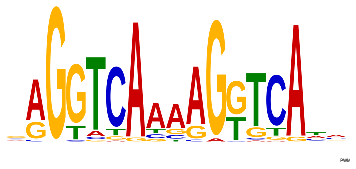	NNGGTCANNAGGTCANN	146.09	DR:2	1
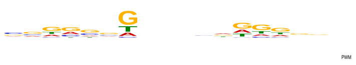	NNGGGGNNNNNNGGGGNN	5.76	M	2
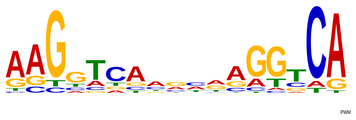	NNGGTCANNNNAGGTNN	7.58	DR:4	3
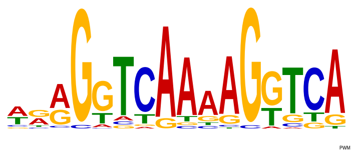	NNAGGTCNNAAGGTNN	29.58	DR:2	4
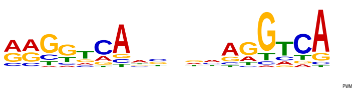	NNGGTCNNNNNNNGGTCNN	4.79	DR:5	5
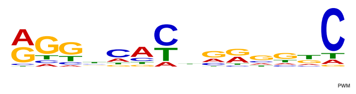	NGGTCANNNAGGTN	4.01	DR:3	6
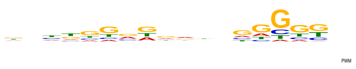	NNTTGGNNNNNNGGGGNN	4.12	M	7
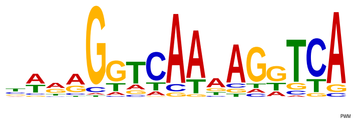	NNNAGGTCNNAAGGNNN	10.27	DR:2	8
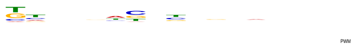	NTTTGNNNNNNNAAAGN	1.73	M	9

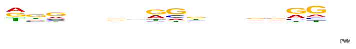	$NNN\overrightarrow{AGGGNN}NNGGGANNN$	1.99	M	10
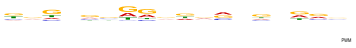	$\overrightarrow{NGGTGN}NNNNNNNGTGGN$	2.94	M	11
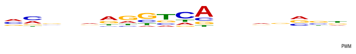	$NNNNN\overrightarrow{AGGTCA}TGNNNNN$	3.15	M	12
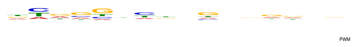	$NN\overrightarrow{AGGTNN}NNGGGANN$	2.05	M	13
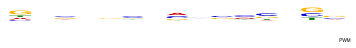	$NNNGG\overrightarrow{CGNCCA}CNNN$	1.77	M	14
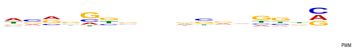	$NN\overrightarrow{GGGTNN}NNNN\overrightarrow{AGGTNN}$	2.74	DR:4	15
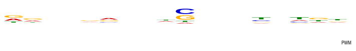	$NGTTGNNNNNNNNTATGN$	1.77	M	16
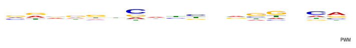	$NN\overrightarrow{AGGTNN}NNN\overrightarrow{AGGTNN}$	2.18	DR:3	17
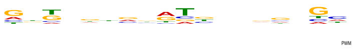	$NNTTGTNNNNNNGGGANN$	1.86	M	18

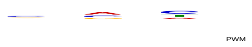	*TCCCAGCC*	1.31	M	19
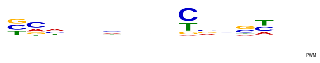	*NNNNN*$\overrightarrow{CGCCCA}$*CCNNNNNN*	2.18	M	20

176 RARG-Jolma2013-Rnd3 (ERR193908)

PWM	Seed Sequence	Seed Seq Enrichment	Repeat	Rank
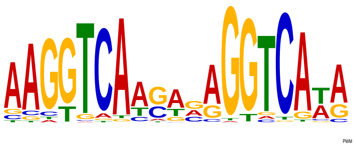	NNGGTCANNNNNGGTCANN	134.20	DR:4	1
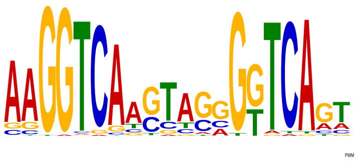	NNGGTCANNNNNNGGTCANN	123.90	DR:5	2
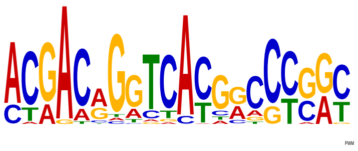	NNNNNAGGTCNTGACNNNNN	15.45	IR:0	3
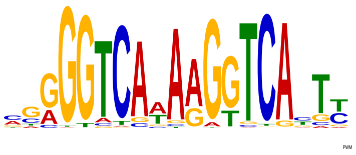	NNNGGTCANNNGGTCANNN	331.99	DR:2	4
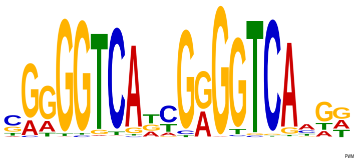	NNNGGTCANNNNGGTCANNN	233.25	DR:3	5
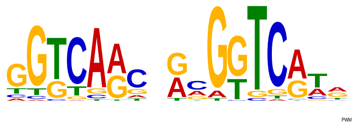	NGTCANNNNNGGTCACN	19.79	DR:4	6
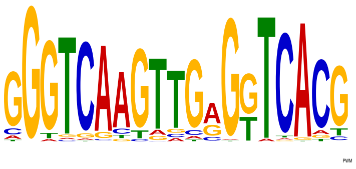	NNGTCANNNNNAGGTCANN	110.58	DR:5	7
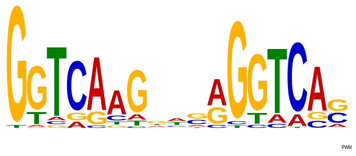	NGTCACNNNNNGGTCAN	20.68	DR:5	8
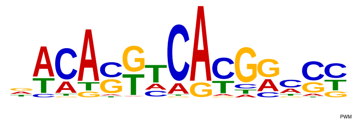	NNAAGGTCNTGACNN	11.05	IR:0	9

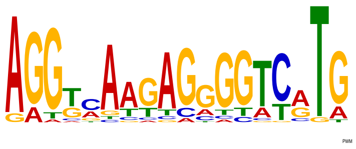	NNGTCACNNNNGGTCANN	17.85	DR:4	10
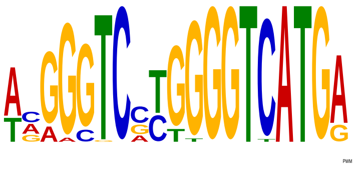	NNGGGTCNNGGGGTCATNN	623.75	DR:2	11
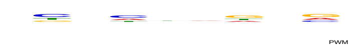	GTCATGAC	1.30	IR:0	12
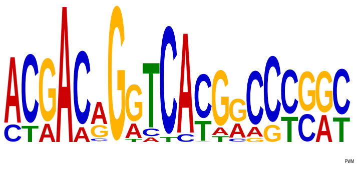	NNNNNAGGTCATGACNNNNN	35.22	IR:0	13
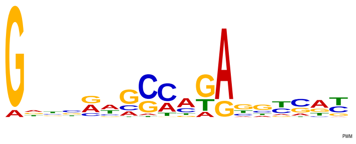	NGTCANNNNNNNGGTCAN	3.06	DR:6	14
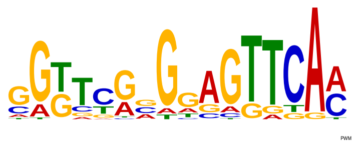	NNGTCANNAAGGTCNN	15.44	DR:3	15
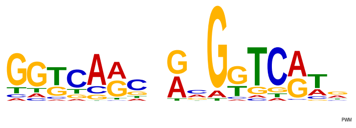	NGTCANNNNNNGTCACN	6.39	DR:4	16
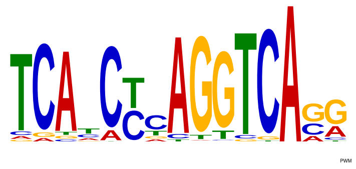	NCATCNNAGGTCACN	103.21	M	17
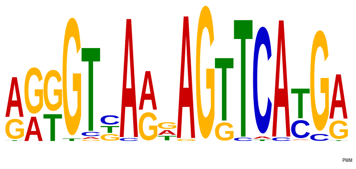	NNNGTTAANAGTTCANNN	84.94	DR:1	18

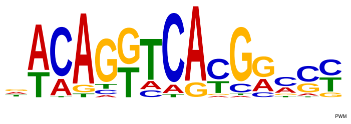	$NNA\overrightarrow{AGGTCA}\overleftarrow{TGACNN}$	24.19	IR:0	19
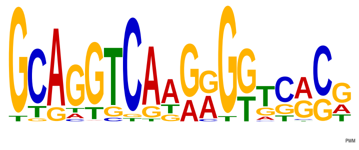	$NN\overrightarrow{AGTTCA}NN\overrightarrow{NGGTCA}NN$	43.45	DR:2	20

177 RARG-Jolma2013-Rnd3 (ERR195455)

PWM	Seed Sequence	Seed Seq Enrichment	Repeat	Rank
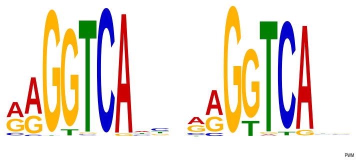	NNNGTCANNNNNGGTCNNN	131.13	DR:4	1
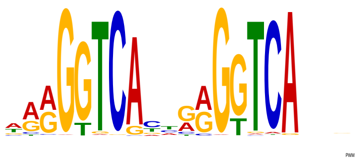	NNNNGTCANNNNGGTCNNNN	73.05	DR:3	2
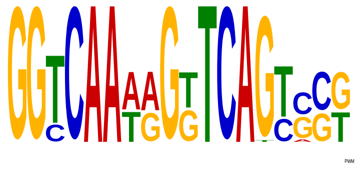	NGTCAANNNNNTCAGTGCN	690.60	DR:7	3
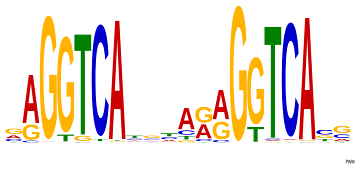	NNNGTCANNNNNNGGTCNNN	66.07	DR:5	4
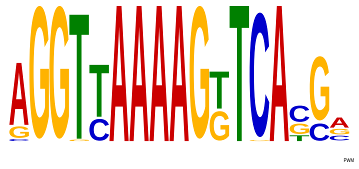	NGGTTAAAANNNCAGGN	549.47	DR:2	5
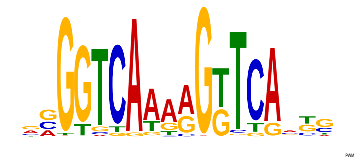	NNNNGTCANNNNGTCANNNN	140.79	DR:2	6
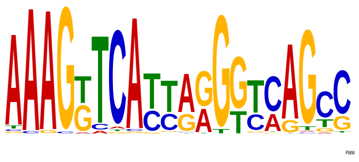	AAAGNNNNNNNNNNNNAGTC	16.93	M	7
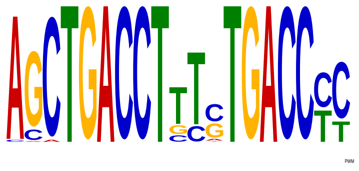	AGCTGANNNNNNNGACCCC	288.28	IR:6	8
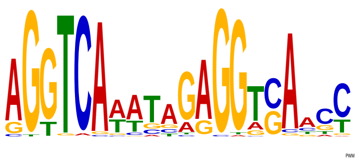	NNGTCANNNNNNNNTCAANN	39.54	DR:5	9

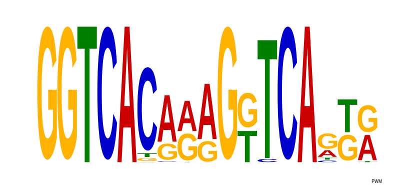	NGTCACGNNNNTCAGTN	161.98	DR:3	10
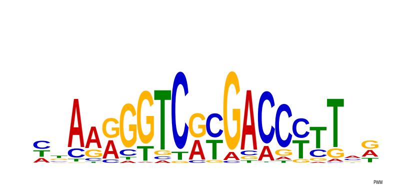	NNNNNGGTCNNGACCNNNNN	14.78	IR:0	11
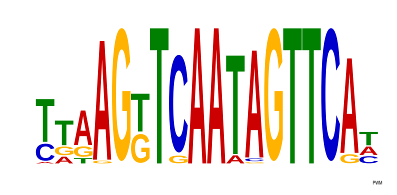	NNAAGTTNNATAGTTCNN	962.81	DR:2	12
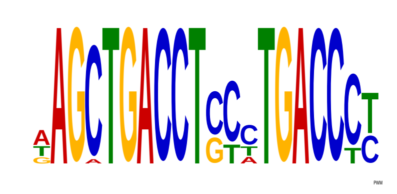	NAGCTGANNNNNNNGACCCN	185.20	IR:6	13
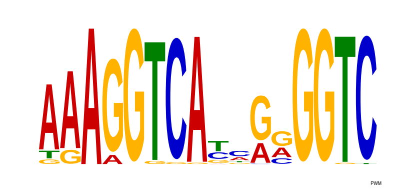	AAAGGNNNNNGCGGTC	95.80	DR:3	14
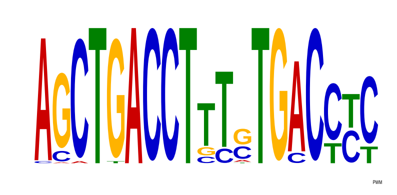	AGCTGANNNNNNNNACCCC	124.23	IR:6	15
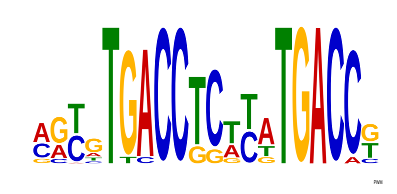	NGCGTGANNNNNNNNGACCN	97.52	IR:7	16
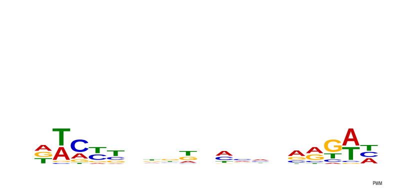	GACCNNNNNNNNNNGGTC	11.07	ER:9	17
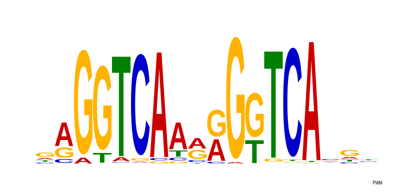	NNNGTCACNNNGTCANNN	45.22	DR:2	18

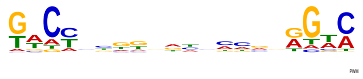	$\overleftarrow{GACC}NNNNNNNNNNN\overrightarrow{NGGTC}$	10.18	ER:10	19
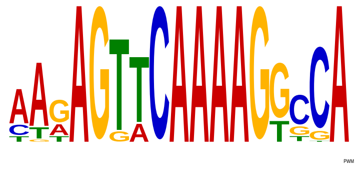	$NAA\overrightarrow{AGNNNA}AA\overrightarrow{AGGGCN}$	126.01	DR:2	20

178 Rarg-Jolma2013-Rnd3 (ERR195723)

PWM	Seed Sequence	Seed Seq Enrichment	Repeat	Rank
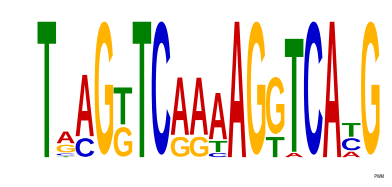	NTAGGTCAAAAGTTCAAN	32597.53	DR:2	1
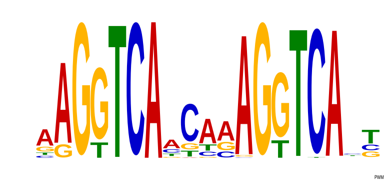	NAGGTCANNNNAGGTCACN	9797.77	DR:4	2
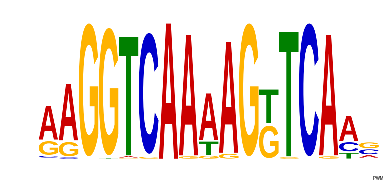	NGGGTCAANAGTTCATN	15274.68	DR:2	3
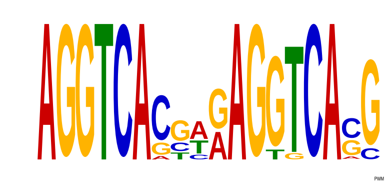	NGGTCACGNGAGGTCACN	23658.04	DR:4	4
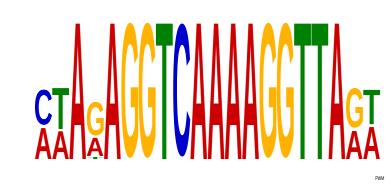	NNAGAGGTCNAAAGGTTANN	62791.79	DR:2	5
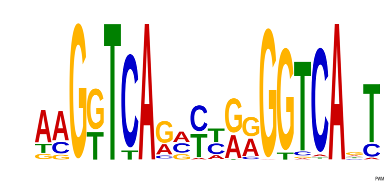	NAGGTCANNNNNNGGTCAAN	2803.29	DR:5	6
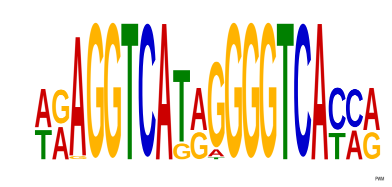	NNAGGTCATNGGGGTCACNN	84924.72	DR:3	7
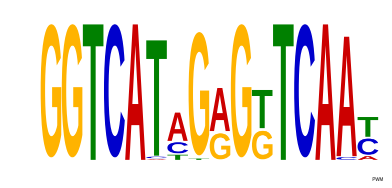	GGTCATAGNGGTCAAT	18061.16	DR:3	8
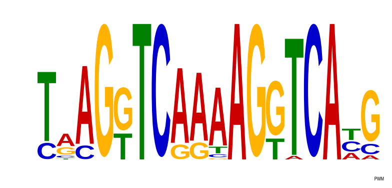	NTAGGTCNNAAGTTCAAN	6947.77	DR:2	9

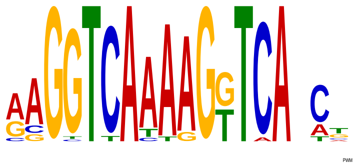	$N\overrightarrow{NGGTCA}TA\overrightarrow{NGGTCA}TNN$	2654.34	DR:2	10
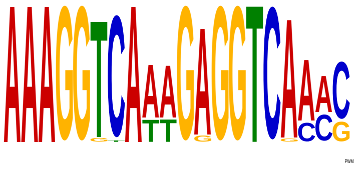	$NA\overrightarrow{AGGTCA}NNN\overrightarrow{AGGTCA}AAN$	61694.83	DR:3	11
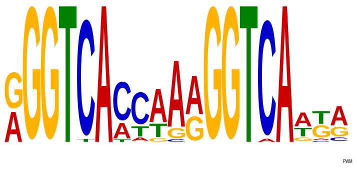	$\overrightarrow{NGGTCA}NNNN\overrightarrow{GGGTCA}TGN$	2423.85	DR:4	12
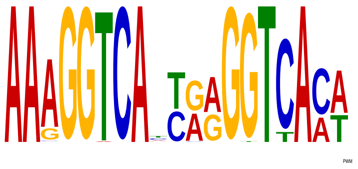	$NN\overrightarrow{AGGTCA}CNG\overrightarrow{AGGTCA}NN$	20376.58	DR:3	13
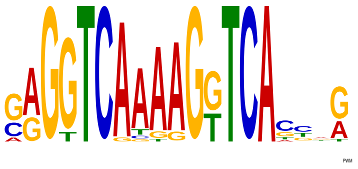	$N\overrightarrow{NGGTCA}NN\overrightarrow{GGGTCA}CGNN$	2269.10	DR:2	14
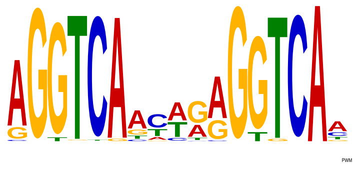	$\overrightarrow{AGGTCN}NNNG\overrightarrow{AGGTCA}A$	11348.50	DR:4	15
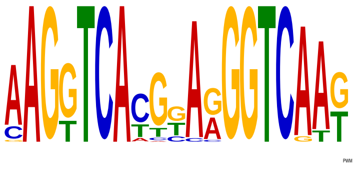	$N\overrightarrow{AGGTCA}TGNN\overrightarrow{NGGTCA}AN$	6680.71	DR:4	16
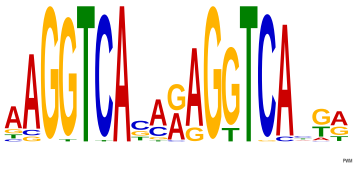	$N\overrightarrow{NGGTCA}CNN\overrightarrow{NGGTCA}TNN$	1420.41	DR:3	17
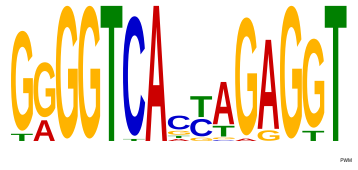	$G\overrightarrow{GGGTCA}NTAG\overrightarrow{AGGT}$	4503.30	DR:4	18

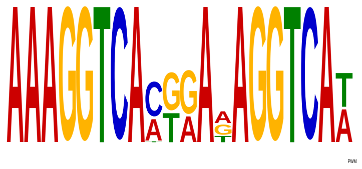	$NA\overrightarrow{AGGTCA}NNNAT\overrightarrow{AGGTCA}N$	30282.57	DR:5	19
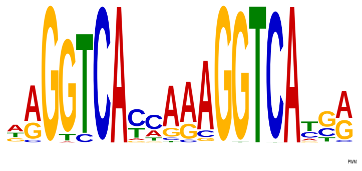	$N\overrightarrow{NGGTCA}TNNN\overrightarrow{NGGTCA}TNN$	3000.72	DR:4	20

179 RARG-Jolma2013-Rnd4 (ERR193909)

PWM	Seed Sequence	Seed Seq Enrichment	Repeat	Rank
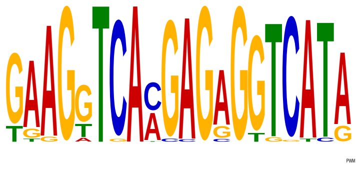	NNAGGTCNNNNNNNGTCANN	60.13	DR:4	1
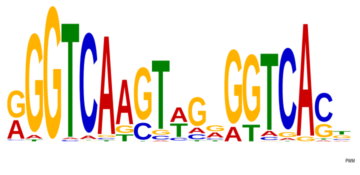	NNGTCANNNNNNNGTCANN	28.32	DR:5	2
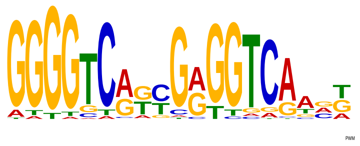	NNNGTCANNNNNGTCANNN	16.69	DR:3	3
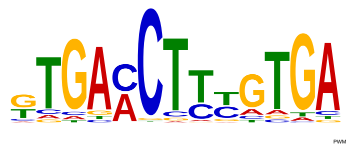	GTGANNNNNGTGA	15.08	DR:3	4
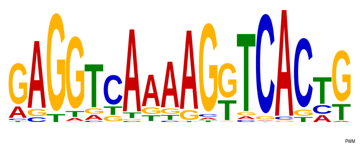	NNNGTCANNNNTTCANNN	23.77	DR:2	5
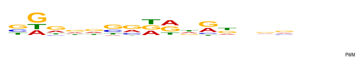	NGGAGNNNNNNNNGGGGN	2.57	M	6
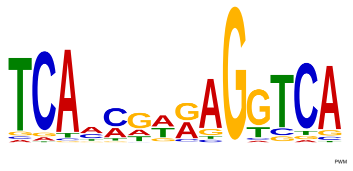	TCACNNNNNNGTCA	12.72	ER:2	7
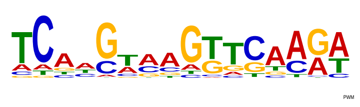	NNAACTNNNNTCACNN	4.75	ER:1	8
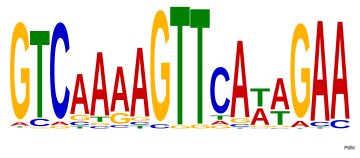	NNNAAAANNNCATANNN	11.33	M	9

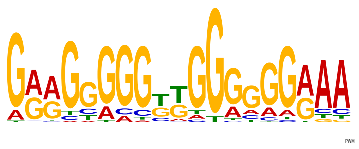	*NNNGGGGGNNGGGGGGGNNN*	52.70	M	10
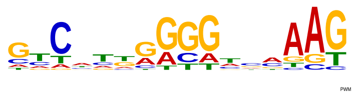	*NNNATGGNNGTCANNN*	2.59	M	11
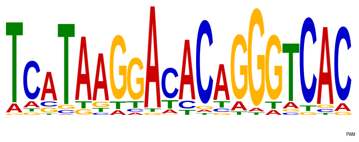	*NNNNAAGGNNNNAGGGNNNN*	13.36	DR:1	12
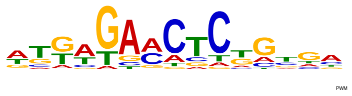	*GTGTNNNNNNNGTGA*	2.97	M	13
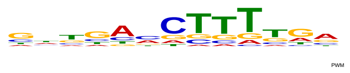	*GTTGNNNNNGTGA*	2.34	M	14
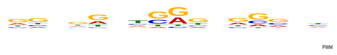	*NNNAGGGNNNGCGGNNN*	2.40	M	15
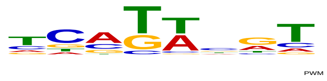	*TCATTCGT*	3.34	M	16
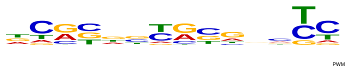	*NNAGGTNNTGACNN*	1.32	IR:0	17
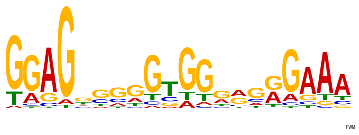	*NNNNGGGGNNNGGGGGNNNN*	9.68	M	18

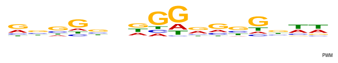	$NN\overrightarrow{NGGGCN}NGGGGGNNN$	5.28	M	19
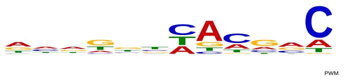	$NA\overrightarrow{AGGNNA}\overleftarrow{TGA}N$	2.34	IR:0	20

180 RARG-8-Bhimsaria2022-Rnd2 (S07-074)

PWM	Seed Sequence	Seed Seq Enrichment	Repeat	Rank
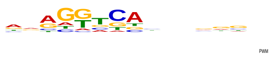	NNNNNNAAGGTCAANNNNNN	5.83	M	1
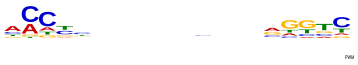	ACCTNNNNNNNNNNNNGGTC	6.68	ER:11	2
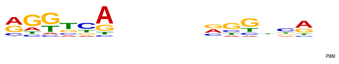	NNGGTCNNNNNNNGGTCNN	4.09	DR:5	3
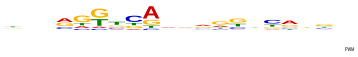	NNNNGGTCNNNNGGTCNNNN	4.23	DR:2	4
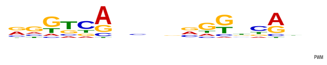	NNNGGTCNNNNNNGGTCNNN	4.23	DR:4	5
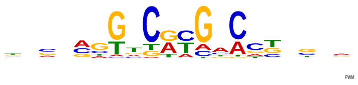	NNNNNGGTCATGACCNNNNN	15.05	IR:0	6
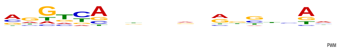	NNGGTCNNNNNNNNGGTCNN	2.62	DR:6	7
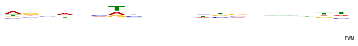	AGCANNNNNNNNNNNNTTTT	2.48	M	8
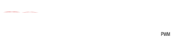	AATTNNNNNNNNNNNNCATT	2.39	M	9

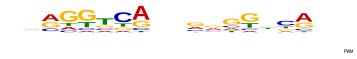	NNNGGTCNNNNNGGTCNNN	4.35	DR:3	10
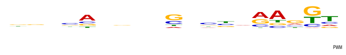	AGGTNNNNNNNNNNNAAGGT	5.30	DR:10	11
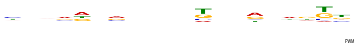	CGAANNNNNNNNNNNNNAGTT	2.57	M	12
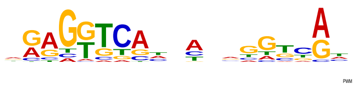	NNAGTTCANNNNNGGTCNN	9.90	DR:4	13
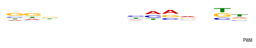	GTCANNNNNNNNNNNNAGGT	2.30	DR:12	14
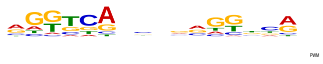	NNAGGTCNNNNNGGTCNN	6.25	DR:4	15
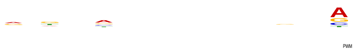	NGGTCNNNNNNNNGGTCN	1.53	DR:7	16
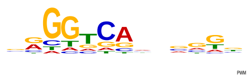	NNNNNCGGGGTCAANNNNN	6.30	M	17
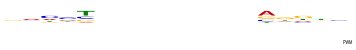	GACCNNNNNNNNNNGGTC	2.39	ER:9	18

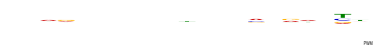	*AAGANNNNNNNNNNNTGTT*	2.33	M	19
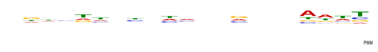	*NTCTTNNNNNNNNNNAAATN*	2.52	M	20

181 CAR-Bhimsaria2022-Rnd3 (S07-165)

PWM	Seed Sequence	Seed Seq Enrichment	Repeat	Rank
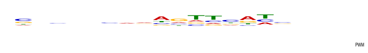	NNNNNNAAAGTTCANNNNNN	6.83	M	1
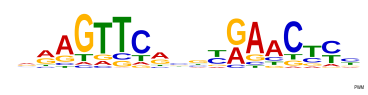	NNAGTTCANNNNNAACTNN	22.01	IR:2	2
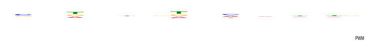	CATTNNNNNNNNNNNNTCTG	4.45	M	3
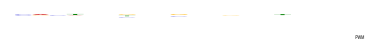	CACTNNNNNNNNNNNNCCGT	3.97	M	4
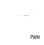	CCAGNNNNNNNNNNNNACGT	3.65	M	5
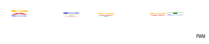	CAATNNNNNNNNNNNNAGTT	4.20	M	6
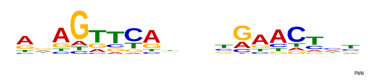	NNAGTTCNNNNNNAACTNN	10.82	IR:3	7
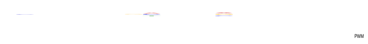	CTCANNNNNNNNNNNNAAAC	3.28	M	8
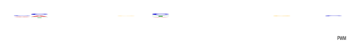	CCCANNNNNNNNNNNNGTTC	3.64	M	9

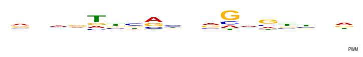	$NN\overrightarrow{AGTTCN}NNNN\overrightarrow{AGTTNN}$	6.07	DR:4	10
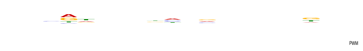	$\overrightarrow{ATCA}NNNNNNNNNNNNGGGA$	2.96	M	11
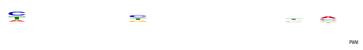	$\overleftarrow{NNAACT}NNNNNNN\overrightarrow{NGTTCN}N$	3.09	ER:7	12
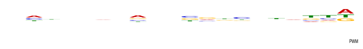	NATAGNNNNNNNNNNTATTN	4.58	M	13
PWM	GGGGNNNNNNNNNNNNGGGG	3.10	M	14
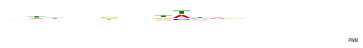	GTTTNNNNNNNNNNNATTT	4.03	M	15
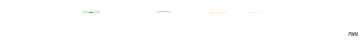	CAAANNNNNNNNNNNAATT	3.74	M	16
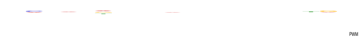	ACGANNNNNNNNNNNNTTGT	3.92	M	17
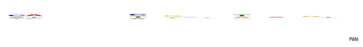	CAGTNNNNNNNNNNNNCGTT	4.13	M	18

182 CAR-Yin2017-Rnd2 (ERR1005180)

PWM	Seed Sequence	Seed Seq Enrichment	Repeat	Rank
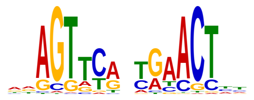	NNNNNNAGTTCNNNGAACTNNNNNN	63.06	IR:1	1
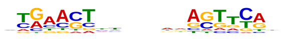	NNNNNNAACTNNNNNNNAGTTNNNNNN	5.43	ER:7	2
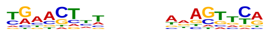	NNNNNNAACTNNNNNNNNNAGTTNNNNNN	4.75	ER:9	3
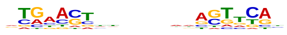	NNNNNNAACTNNNNNNNNAGTTNNNNNN	4.61	ER:8	4
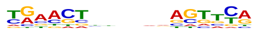	NNNNNNAACTNNNNNNAGTTNNNNNN	4.89	ER:6	5
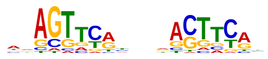	NNNNNNAGTTCNNNNNAGTTNNNNNN	11.39	DR:4	6
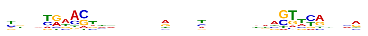	NNNNNNAACTNNNNNNNNNNNNNNNNNNNAGTTNNNNNN	3.39	ER:19	7
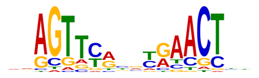	NNNNNNAGTTCNNNGAACTNNNNNN	27.83	IR:2	8
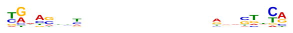	NNNNNNAACTNNNNNNNNNNNNNNNNNNAGTTNNNNNN	2.98	ER:18	9

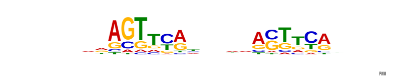	NNNNNNAGTTCNNNNNNAGTTNNNNNN	8.67	DR:5	10
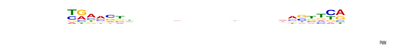	NNNNNNAACTNNNNNNNNNNNNNNNNNAGTTNNNNNN	3.48	ER:17	11
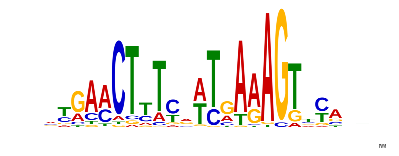	NNNNNNCTTTNNATGAAAGTNNNNNN	56.79	M	12
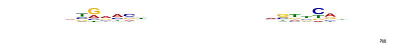	NNNNNNAACTNNNNNNNNNNAGTTNNNNNN	2.74	ER:10	13
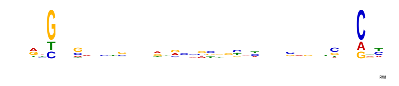	NNNNNNAACTNNNNNNNNNNNNNNNNNNNNAGTTNNNNNN	1.68	ER:20	14
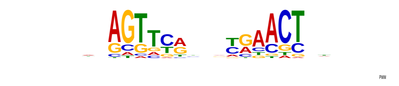	NNNNNNAGTTCNNNNNGAACTNNNNNN	19.52	IR:3	15
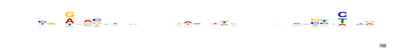	NNNNNAACTNNNNNNNNNNNNNNNNNNNNNAGTTNNNNN	1.85	ER:21	16
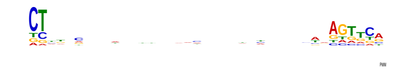	CTTTNNNNNNNNNNNNNNNNNNNNNNNNNNNNNNGTTCA	8.17	M	17
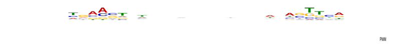	NNNNNNAACTNNNNNNNNNNNNNNNNAGTTNNNNNN	3.22	ER:16	18

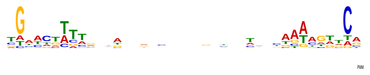	$\overleftarrow{NNAACT}NNNNNNNNNNNNNNNNNNNNNNNNNNNN\overrightarrow{AGTTNN}$	2.59	ER:28	19
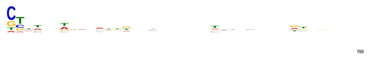	$CTTTNNNNNNNNNNNNNNNNNNNNNNNNNNNNNNNN\overrightarrow{NNGTCA}$	3.70	M	20

183 CAR-Yin2017-Rnd2 (ERR1005181)

PWM	Seed Sequence	Seed Seq Enrichment	Repeat	Rank
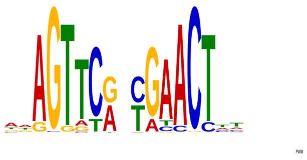	NNNNNNAGTTCNNNGAACTNNNNNN	296.32	IR:1	1
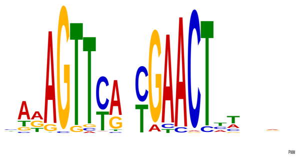	NNNNNNAAAGTTTANNGAACTNNNNNN	635.34	IR:1	2
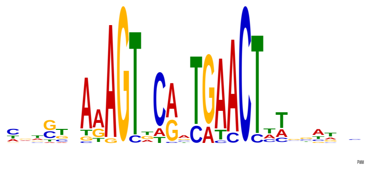	NNNNNNAAAGTGCANNGAACTTTNNNNNN	1198.30	IR:1	3
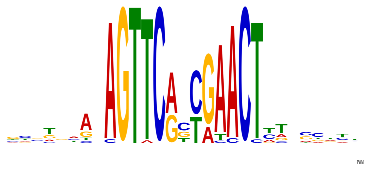	NNNNNNACAGTTCNNNGAACTCTNNNNNN	628.46	IR:1	4
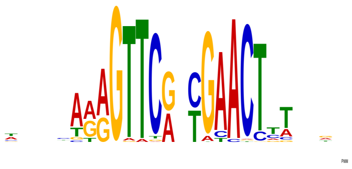	NNNNNNAAGGTTCGNNGAACTNNNNNN	373.92	IR:2	5
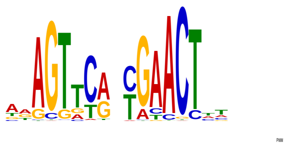	NNNNNNAAAGTNNNNNGAACTNNNNNN	91.35	IR:2	6
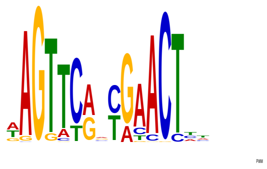	NNNNNNATAAGTTCNNNGAACTNNNNNN	428.26	IR:1	7
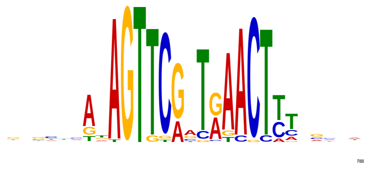	NNNNNNATAGTTCGNTAAACTTNNNNNN	1642.91	IR:1	8
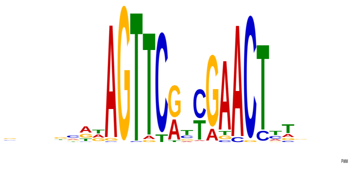	NNNNNNCGTAGTTCNNNGAACTNNNNNN	474.00	IR:1	9

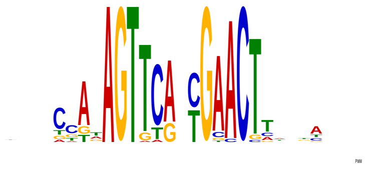	NNNNNNATAGTTCANNGAACTTANNNNNN	2093.94	IR:1	10
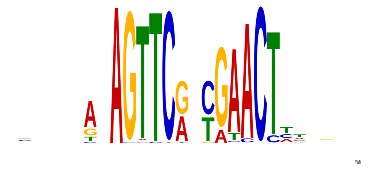	NNNNNNACAGTTCGNNGAACTNNNNNN	487.96	IR:1	11
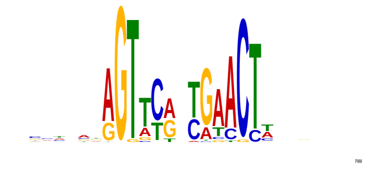	NNNNNNAGAGTTCNNNGAACTTANNNNNN	800.90	IR:1	12
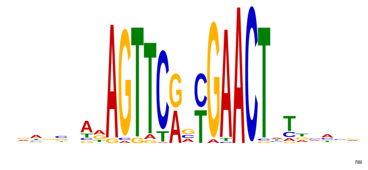	NNNNNNAGAGTTCNNNGAACTGNNNNNN	274.62	IR:1	13
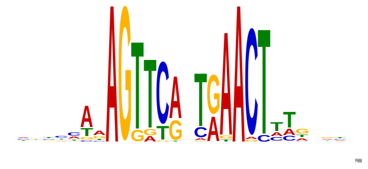	NNNNNNAAAGTTCANTAAACTTNNNNNN	1680.74	IR:1	14
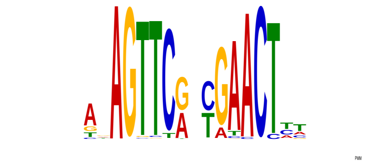	NNNNNNACAGTTCNNNGAACTNNNNNN	260.66	IR:1	15
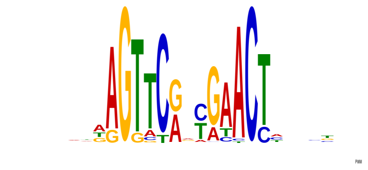	NNNNNNTAAGTTCGNNGAACTCNNNNNN	836.76	IR:1	16
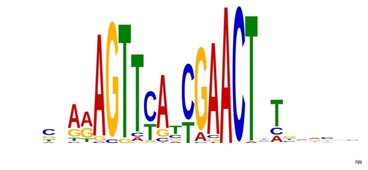	NNNNNNAAGTTTANNGAACTATNNNNNN	697.83	IR:1	17
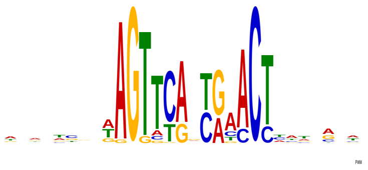	NNNNNNATAAGTTCNNNGAACTTNNNNNN	553.99	IR:1	18

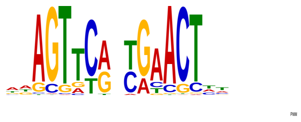	$NNNNNNA\overrightarrow{AGTTN}NN\overleftarrow{NNAACT}TNNNNNN$	58.88	IR:2	19
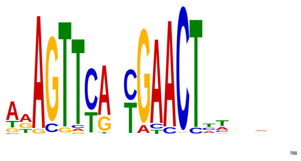	$NNNNNNA\overrightarrow{AGTTTA}N\overleftarrow{NGAACT}TNNNNNN$	446.47	IR:1	20

184 CAR-Yin2017-Rnd3 (ERR1005182)

PWM	Seed Sequence	Seed Seq Enrichment	Repeat	Rank
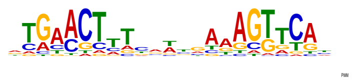	*NNNNNNCTTTNNNNNNAAAGNNNNNN*	9.90	M	1
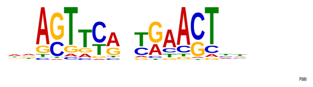	*NNNNNNAGTTNNNNNAACTNNNNNN*	21.56	IR:1	2
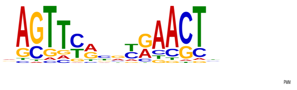	*NNNNNNAGTTNNNNNNAACTNNNNNN*	28.36	IR:2	3
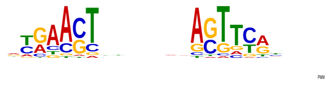	*NNNNNNAACTNNNNNNNAGTTNNNNNN*	19.57	ER:7	4
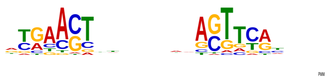	*NNNNNNAACTNNNNNNNNAGTTNNNNNN*	14.51	ER:8	5
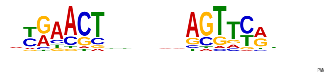	*NNNNNNAACTNNNNNNAGTTNNNNNN*	15.23	ER:6	6
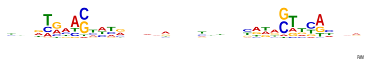	*NNNNNNAACTNNNNNNNNNNNNNNNNNNNAGTTNNNNNN*	6.24	ER:19	7
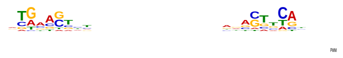	*NNNNNNAACTNNNNNNNNNNNNNNNNNNAGTTNNNNNN*	7.73	ER:18	8
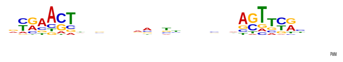	*NNNNNNAACTNNNNNNNNNNNNNNNNNAGTTNNNNNN*	10.90	ER:17	9

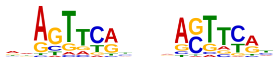	*NNNNNNGTTCNNNNNAGTTNNNNNN*	15.86	DR:4	10
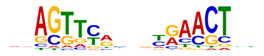	*NNNNNNAGTTNNNNNNNAACTNNNNNN*	16.52	IR:3	11
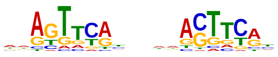	*NNNNNNGTTCNNNNNNAGTTNNNNNN*	12.82	DR:5	12
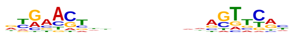	*NNNNNNAACTNNNNNNNNNNAGTTNNNNNN*	7.47	ER:10	13
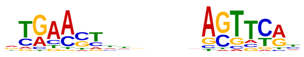	*NNNNNNCTTTNNNNNAGTTNNNNNN*	9.47	M	14
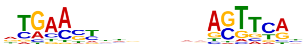	*NNNNNNCTTTNNNNNNAGTTNNNNNN*	7.35	M	15
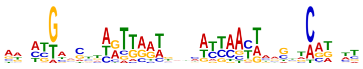	*NNNNNNAACTNNNNNNNNNNNNNNNNNNNNAGTTNNNNNN*	2.94	ER:20	16
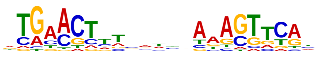	*NNNNNNCTTTNNNNNNAAGTNNNNNN*	8.99	M	17
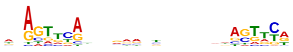	*NNNNNNGTTCNNNNNNNNNNNNNNNNAGTTNNNNNN*	8.17	DR:15	18

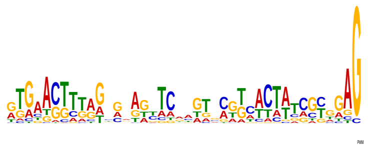	$\overrightarrow{GTGA}$ANNNNNNNNNNNNNNNNNNNNNNNNNNNNNNNGGAG	15.62	M	19
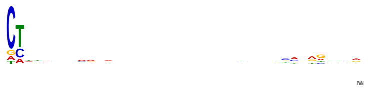	CTTTNNNNNNNNNNNNNNNNNNNNNNNNNNNNNN$\overrightarrow{NNTTCA}$	8.19	M	20

185 CAR-Yin2017-Rnd3 (ERR1005183)

PWM	Seed Sequence	Seed Seq Enrichment	Repeat	Rank
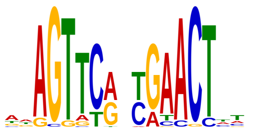	NNNNNNAGTTCNNNGAACTNNNNNN	797.02	IR:1	1
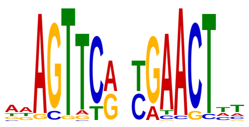	NNNNNNAGTTNNNNNAACTNNNNNN	143.08	IR:1	2
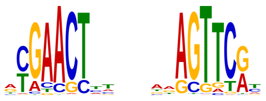	NNNNNNGAACTNNNNNNNAGTTCNNNNNN	150.79	ER:7	3
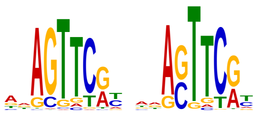	NNNNNNAGTTCNNNNNAGTTCNNNNNN	147.58	DR:4	4
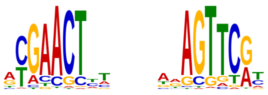	NNNNNNGAACTNNNNNNNNAGTTCNNNNNN	102.35	ER:8	5
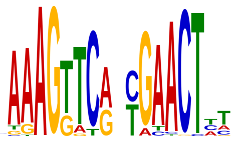	NNNNNNAAAGGTCNNNGAACTNNNNNN	539.51	IR:1	6
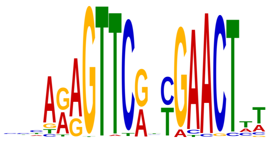	NNNNNNAGGGTTCNNNGAACTNNNNNN	454.15	IR:3	7
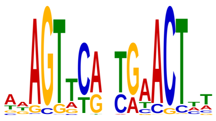	NNNNNNAAAGTNNNNNNACTTTNNNNNN	187.01	IR:3	8
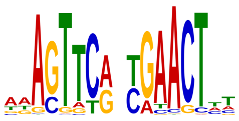	NNNNNNAAGTNNNNNNAACTNNNNNN	81.71	IR:2	9

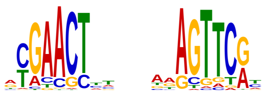 PWM	NNNNNNGAACTNNNNNNNNGTTCNNNNNN	62.78	ER:7	10
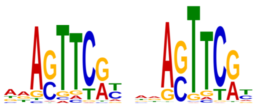 PWM	NNNNNNAGTTNNNNNNAGTTCNNNNNN	63.16	DR:4	11
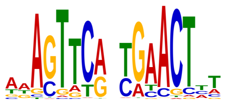 PWM	NNNNNNAAGTNNNNNNNNCTTTNNNNNN	30.91	M	12
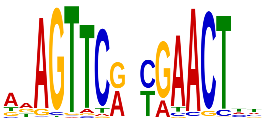 PWM	NNNNNNTTCGGNNAACTNNNNNN	85.03	M	13

186 PXR-Bhimsaria2022-Rnd2 (S07-043)

PWM	Seed Sequence	Seed Seq Enrichment	Repeat	Rank
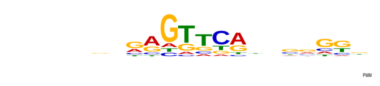	NNNNNNGAGTTCATNNNNNN	6.85	M	1
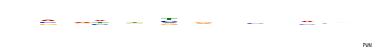	CAAANNNNNNNNNNNNATAT	2.00	M	2
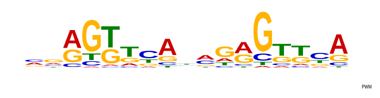	NNAGTTCNNNNAGTTCNN	9.69	DR:3	3
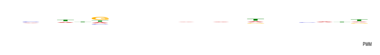	CGTTNNNNNNNNNNNNCATT	1.98	M	4
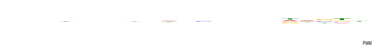	ATTANNNNNNNNNNNNAGTT	2.14	M	5
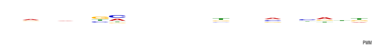	ATAGNNNNNNNNNNNNCATT	2.01	M	6
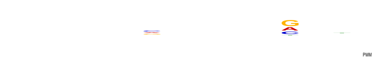	CAACNNNNNNNNNNNNAGTT	1.81	M	7
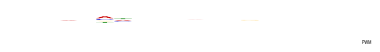	TCAANNNNNNNNNNNNAAAT	1.66	M	8
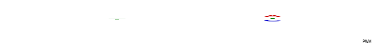	CATTNNNNNNNNNNNNACTG	1.75	M	9

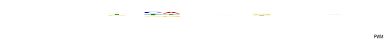	$NN\overrightarrow{AGTTNN}NNNNNNTTCGNN$	1.77	M	10
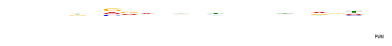	$CCTTNNNNNNNNNNNNAGTG$	1.72	M	11
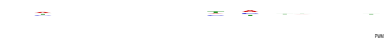	$\overrightarrow{ACGTNN}NNNNNNNNNNATTT$	1.89	M	12
	$CACANNNNNNNNNNNNCACT$	1.59	M	13
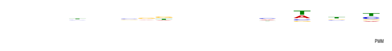	$AATCNNNNNNNNNNNN\overrightarrow{ATGT}$	1.97	M	14
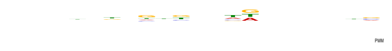	$GTTTNNNNNNNNNNNNGTTG$	1.60	M	15
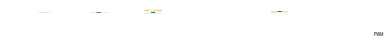	$\overrightarrow{AGTTNN}NNNNNNNNNNTTCT$	1.85	M	16
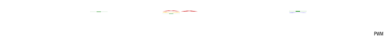	$\overrightarrow{AGTTNN}NNNNNNNNNNCATT$	1.78	M	17
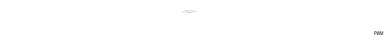	$\overrightarrow{AAGTNN}NNNNNNNNNNTTGT$	1.70	M	18

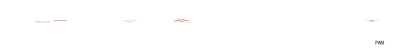	*CAAANNNNNNNNNNNNGCTT*	1.69	M	19
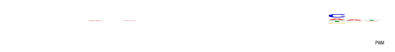	*CGAANNNNNNNNNNNNGCAT*	1.66	M	20

187 PXR-Bhimsaria2022-Rnd3 (S07-164)

PWM	Seed Sequence	Seed Seq Enrichment	Repeat	Rank
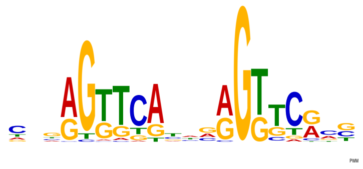	NNNAGTTCNNNGAGTTCNNN	372.61	DR:3	1
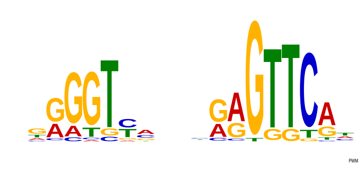	NNGGGTCNNNNGAGTTCNN	132.23	DR:4	2
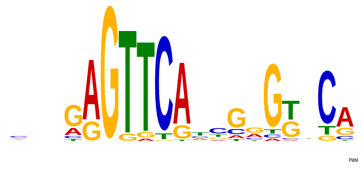	NNNGAGTTCANCGGGGNNN	342.42	M	3
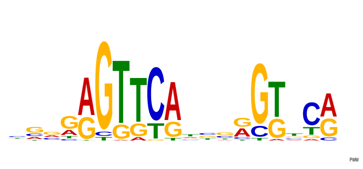	NNNGAGTTCNNNGGGGTNNN	112.58	DR:3	4
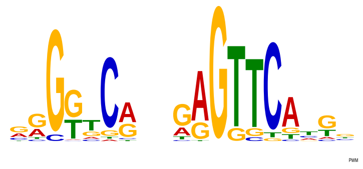	NNGGTCNNNGAGTTCATNN	686.42	DR:3	5
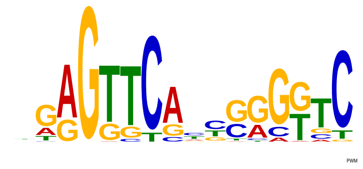	NGAGTTCACNGGGGTN	466.84	DR:3	6
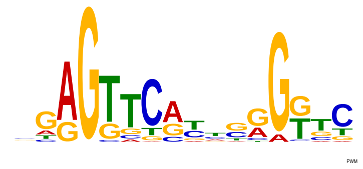	NGAGTTCNTTGGGGTN	341.05	DR:3	7
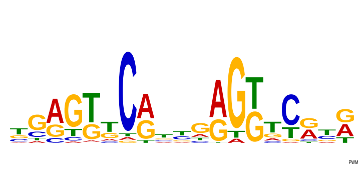	NNNGGTCNNCGGGTTCNNN	85.01	DR:3	8
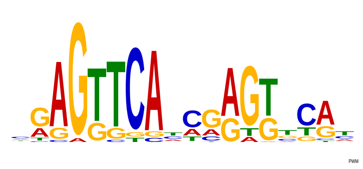	NNAGTTCACNNNGGGCNN	92.90	DR:3	9

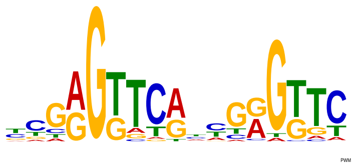	NCGGGTTCNNNGGGTTN	179.05	DR:3	10

188 PXR-Yin2017-Rnd2 (ERR1005173)

PWM	Seed Sequence	Seed Seq Enrichment	Repeat	Rank
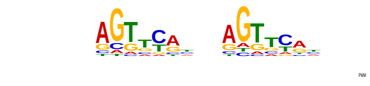	NNNNNNAGTTNNNNNAGTTNNNNNN	10.48	DR:3	1
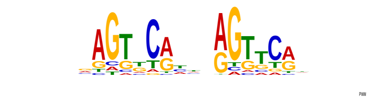	NNNNNNAGTGCNNNNNGTTCNNNNNN	16.34	DR:3	2
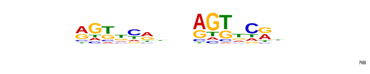	NNNNNNGTTCNNNNNNNTCGTNNNNNN	5.10	M	3
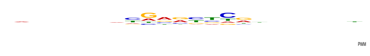	NNNNNNACGANNTCGTNNNNNN	3.86	M	4
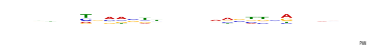	NNNNNNTGAANNNNNNNNNNTTCANNNNNN	3.64	ER:6	5
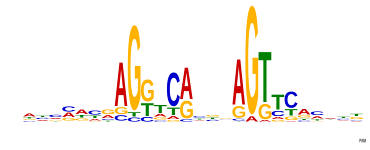	NNNNNNCGAGGTCNNNNAGTTNNNNNN	33.26	DR:3	6
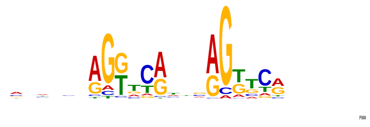	NNNNNNAGGTCANNNNNTTCANNNNNN	19.49	DR:3	7
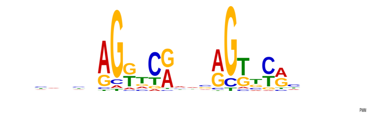	NNNNNNGAGGTCNNNNNNTTCANNNNNN	12.70	DR:3	8
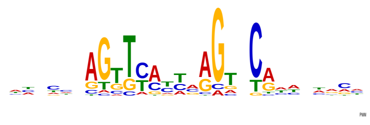	NNNNNNAGTTNNNNNAGTGCAANNNNNN	27.20	DR:3	9

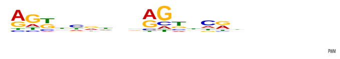	*NNNNNNCGTTNNNNTCGTNNNNNN*	3.50	M	10
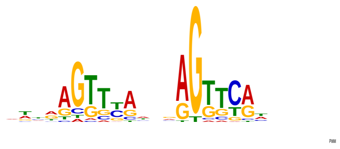	*NNNNNNAGTTTANNNNNTTCANNNNNN*	19.86	DR:3	11
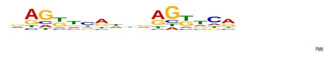	*NNNNNNGTCANNNNNGTCANNNNNN*	3.87	DR:3	12
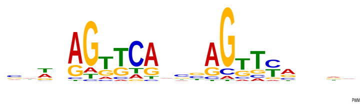	*NNNNNNTTCAACNAGTTNNNNN*	12.77	DR:3	13
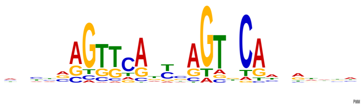	*NNNNNNGTTCANNNNGTGCAANNNNNN*	29.11	DR:3	14
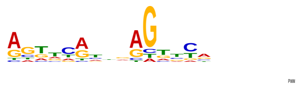	*NNNNNNGTTCNNNNNNTTTANNNNNN*	4.20	M	15
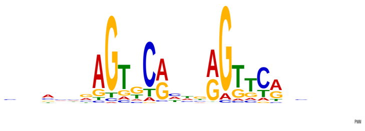	*NNNNNNGAGTGCNNNNNNTTCANNNNNN*	19.00	DR:3	16
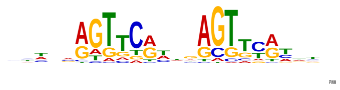	*NNNNNNCGGTTCNNNNAGTTNNNNNN*	10.31	DR:4	17
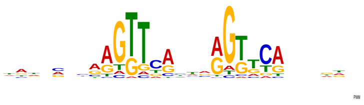	*NNNNNNCAAGTTNNNNNNNTTCANNNNNN*	14.63	DR:4	18

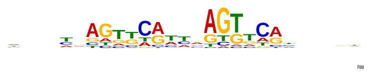	$NNNNNN\overrightarrow{AGTGCN}NNNN\overrightarrow{NNGTCA}NNNNNNN$	6.85	DR:3	19
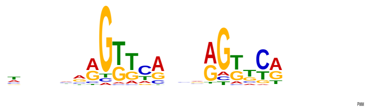	$NNNNNN\overrightarrow{GGTTCA}NNNN\overrightarrow{NNTTCA}NNNNNNN$	15.04	DR:3	20

189 PXR-Yin2017-Rnd3 (ERR1005174)

PWM	Seed Sequence	Seed Seq Enrichment	Repeat	Rank
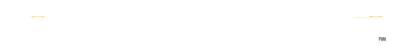	NNNNNNGGGGNNNNNGGGGNNNNNN	3.11	M	1
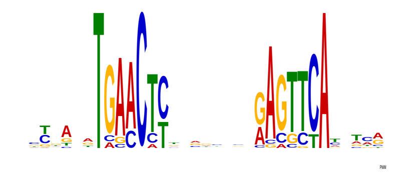	NNNNNNTGAACTNNNNNNNNNNAGTTCNNNNNN	22.97	ER:10	2
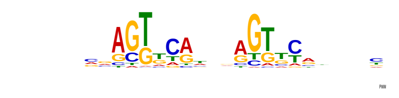	NNNNNNAGTTCNNNNAGTTCNNNNNN	11.39	DR:3	3
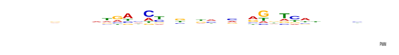	NNNNNNATGANNNNNNNNNNNNNTCATNNNNNN	2.44	ER:8	4
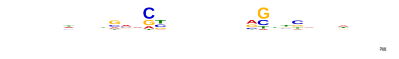	NNNNNNTGAANNNNNNNNNNNTTCANNNNNN	1.95	ER:7	5
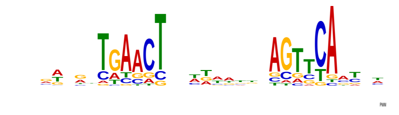	NNNNNNTGAACTNNNNNNNNNAGTTNNNNNN	8.25	ER:9	6
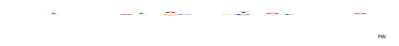	NNNNNNATCANNNNNTGATNNNNNN	1.80	IR:4	7
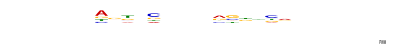	NNNNNNGTTCNNNNNNNNTCATNNNNNN	2.20	DR:3	8
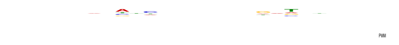	NNNNNNATCANNNNNNTGATNNNNNN	1.86	IR:5	9

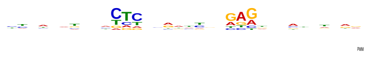	*NNNNNNTGAANNNNNNNNNNNNTTCANNNNNN*	1.93	ER:10	10
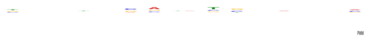	*NNNNNNTACANNNNNNNNNTGTANNNNN*	1.49	M	11
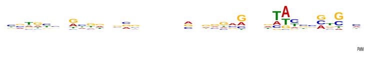	*NGTGCNNNNNNNNNNNNNNNNNNNNNNNNNNNNNNGTGTN*	2.08	M	12
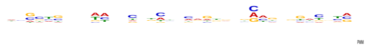	*NNGCTGNNNNNNNNNNNNNNNNNNNNNNNNNNNACGTNN*	2.02	M	13
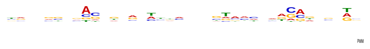	*NNAAGGNNNNNNNNNNNNNNNNNNNNNNNNNGTGANN*	1.66	DR:21	14
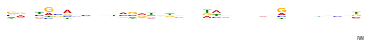	*NGATGNNNNNNNNNNNNNNNNNNNNNNNNNNTGCGN*	2.06	M	15
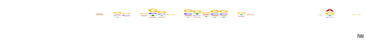	*NNNNNGGGGNNNNNNNNNNNNNNNNNNNNNNGGGGNNNNN*	2.21	M	16
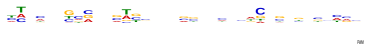	*NNACCTNNNNNNNNNNNNNNNNNNNNNNNNNTCAANN*	1.36	ER:22	17
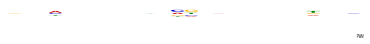	*NNNNNNTGTCNNNNNNGACANNNNNN*	1.33	DR:3	18

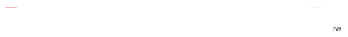	$NNNNNN\overrightarrow{ACGTNN}N\overrightarrow{ACGTNN}NNNN$	1.77	DR:1	19
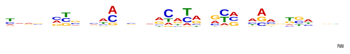	$NNN\overrightarrow{AAGTNN}NNNNNNNNNNNNNNNNNNNN\overrightarrow{NNGTGA}NNN$	1.83	DR:20	20

190 PXR-Yin2017-Rnd3 (ERR1005175)

PWM	Seed Sequence	Seed Seq Enrichment	Repeat	Rank
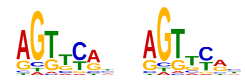	NNNNNNAGTTNNNNNAGTTNNNNNN	16.28	DR:3	1
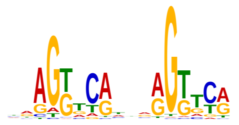	NNNNNNAGGTCNNNNNNTTCANNNNNN	19.79	DR:3	2
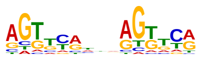	NNNNNNAGTTNNNNNNNNTCATNNNNNN	8.66	DR:3	3
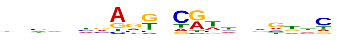	NNNNNNTGAGNNCGTTNNNNNN	5.36	M	4
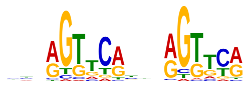	NNNNNNGGTTCNNNNNNTTCANNNNNN	14.03	DR:4	5
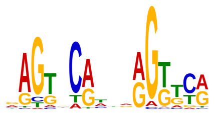	NNNNNNGAGTGCNNNNNNTTCANNNNNN	20.79	DR:3	6
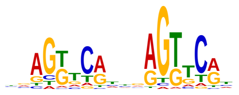	NNNNNNAGGTCNNNNNNNTCGTNNNNNN	11.82	M	7
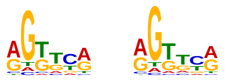	NNNNNNTTCANNNNNTTCANNNNNN	10.13	DR:3	8
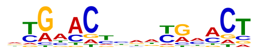	NNNNNNTCAANNNACTCNNNNNN	4.61	M	9

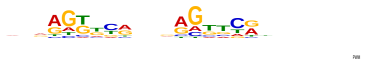	NNNNNNTCATNNNNNNTCGTNNNNNN	5.19	M	10
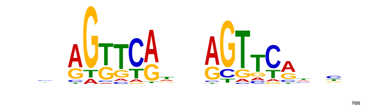	NNNNNNTTCANGCAGTTNNNNN	17.14	DR:3	11
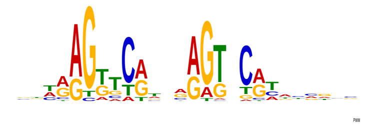	NNNNNGTTCNNNNAGTCCATNNNNNN	32.99	DR:3	12
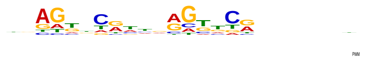	NNNNNNCGTTNNNNTCGTNNNNNN	5.14	M	13
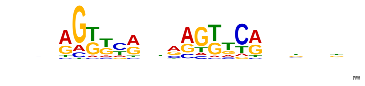	NNNNNTTCANNNNNGTCATNNNNNN	8.61	DR:3	14
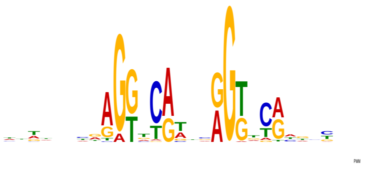	NNNNNNCGAGGTCNNNNNNTTCANNNNNN	31.21	DR:3	15
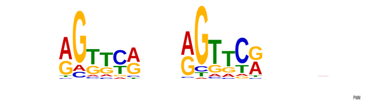	NNNNNNTTCANNNNNNTTCGCNNNNNN	10.33	M	16
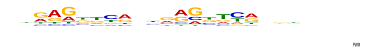	NNNNNNTCAANNNNNTCATNNNNNN	4.52	DR:3	17
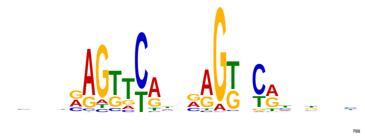	NNNNNAGTTNNNNNAGTCCANNNNNN	19.79	DR:3	18

191 PXR-Yin2017-Rnd4 (ERR1005176)

PWM	Seed Sequence	Seed Seq Enrichment	Repeat	Rank
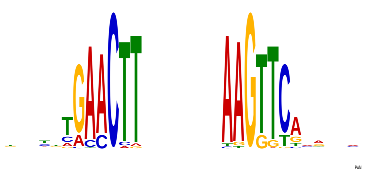	NNNNNNGAACTTNNNNNNNAAGTTCNNNNNN	171.07	ER:9	1
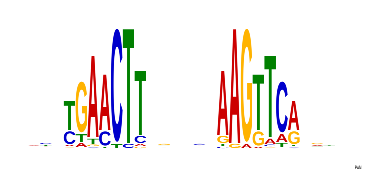	NNNNNNGAACTTNNNNNNAAGTTCNNNNNN	112.44	ER:8	2
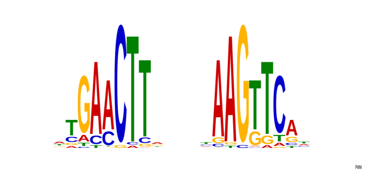	NNNNNNGAACTTNNNNNAAGTTCNNNNNN	147.48	ER:7	3
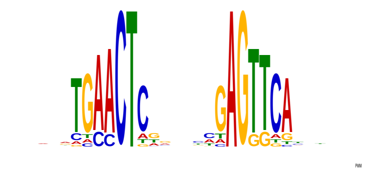	NNNNNNTGAACTCNNNNNNGAGTTCANNNNNN	368.44	ER:8	4
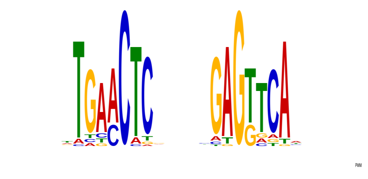	NNNNNNTGAACTCNNNNNGAGTTCANNNNNN	374.40	ER:7	5
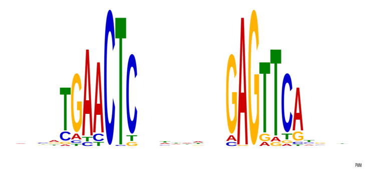	NNNNNNGAACTCNNNNNNNNGAGTTCNNNNNN	114.64	ER:10	6
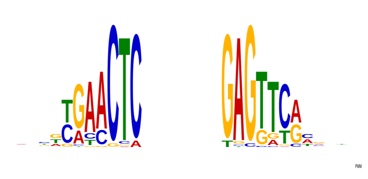	NNNNNNGAACTCNNNNNNNGAGTTCNNNNNN	90.86	ER:9	7
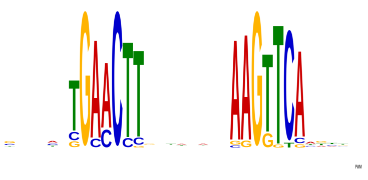	NNNNNNTGAACTTNNNNNNNNAAGTTCNNNNNN	201.09	ER:10	8
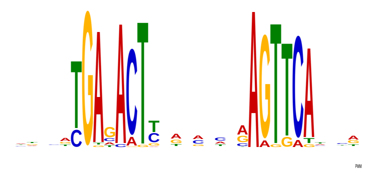	NNNNNNTGAGACTNNNNNNNNNAGTTNNNNNN	45.59	ER:10	9

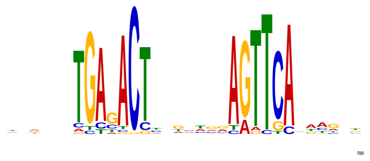	NNNNNNTGAGACTNNNNNNNNNNTTCANNNNNNN	47.95	ER:8	10
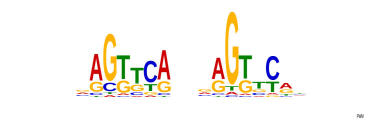	NNNNNNAGTTCNNNNAGTTCNNNNNN	18.38	DR:3	11
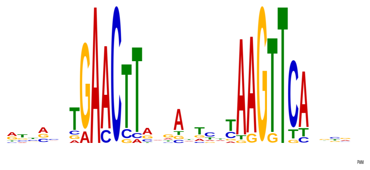	NNNNNNTGAACTTNNNNNNNNNAAGTTCNNNNNN	160.01	ER:11	12
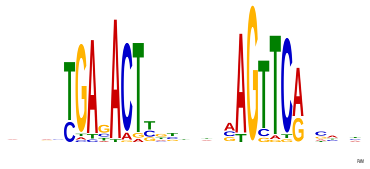	NNNNNNGAGACTNNNNNNNNAGTTCNNNNNN	39.58	ER:7	13
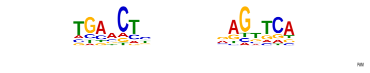	NNNNNNTGAANNNNNNNNNNNNTTCANNNNNNN	4.42	ER:8	14
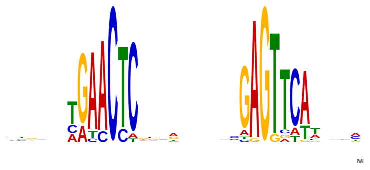	NNNNNNTGAACTCNNNNNNNNNNGAGTTCNNNNNN	139.68	ER:12	15
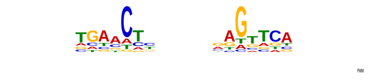	NNNNNNTGAANNNNNNNNNNNNTTCANNNNNNN	4.22	ER:7	16
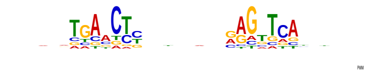	NNNNNNTGAANNNNNNNNNNNNNNTTCANNNNNNN	4.33	ER:10	17
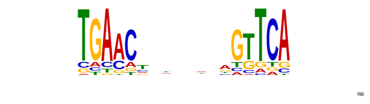	NNNNNNTGAACNNNNNNNNGTTCANNNNNNN	14.17	ER:6	18

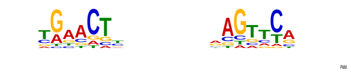	*NNNNNNAACTNNNNNNNNNNAGTTNNNNNN*	5.05	ER:9	19
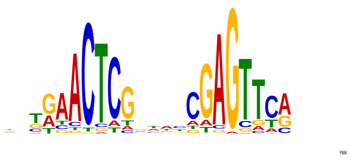	*NNNNNNACTCGNNNNCGAGTTNNNNNN*	40.93	M	20

192 PXR-Yin2017-Rnd4 (ERR1005177)

PWM	Seed Sequence	Seed Seq Enrichment	Repeat	Rank
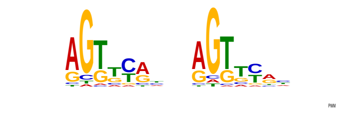	NNNNNNAGTTNNNNNAGTTNNNNNN	20.72	DR:3	1
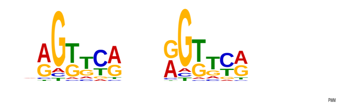	NNNNNNTTCANNNNNTTCANNNNNN	15.38	DR:3	2
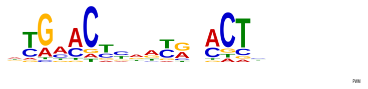	NNNNNNTCAANNNACTCNNNNNN	7.06	M	3
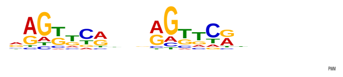	NNNNNNTCATNNNNNTCGTNNNNNN	7.23	M	4
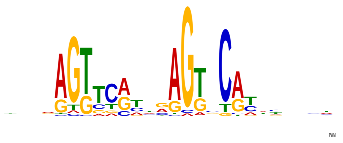	NNNNNNAGTTNNNNNAGTCCATNNNNNN	50.11	DR:3	5
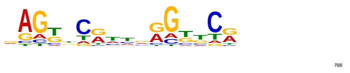	NNNNNNCGTTNNNNTCGTNNNNNN	6.40	M	6
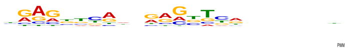	NNNNNNTCAANNNNNTCAANNNNNN	5.95	DR:3	7
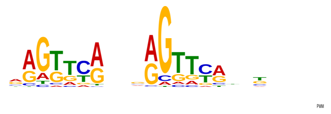	NNNNNNTTCANNNNNTTTATNNNNNN	11.22	M	8
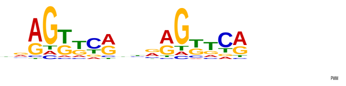	NNNNNNTCATNNNNGTCANNNNNN	7.05	DR:3	9

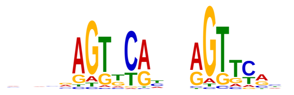	NNNNNNCGAGTNNNNNCAGTTNNNNNN	20.64	M	10
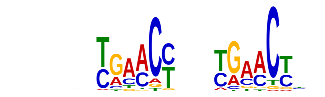	NNNNNNCGTGAANNNNNTGAANNNNNN	23.21	IR:6	11
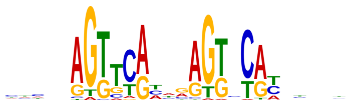	NNNNNNAGTTNNNNGAGTCCANNNNNN	48.76	DR:3	12
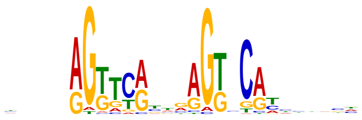	NNNNNNGTTCNNNNAGTCCATNNNNNN	42.85	DR:3	13
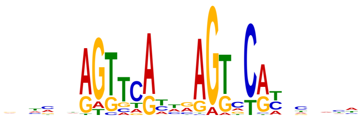	NNNNNNAGTTNNNNGAGTCCATNNNNNN	77.26	DR:3	14
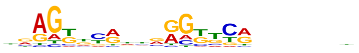	NNNNNNCATTNNNNTCATNNNNNN	6.49	M	15
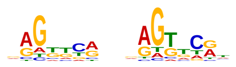	NNNNNNTTCANNNNNNNNCGTTNNNNNN	5.73	M	16
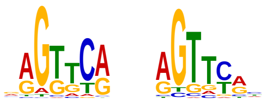	NNNNNNTTCANGCAGTTNNNNNN	24.51	DR:3	17
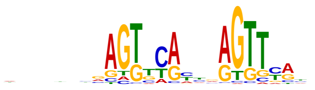	NNNNNNCGAGTNNNNNNAGTTTNNNNNN	19.38	M	18

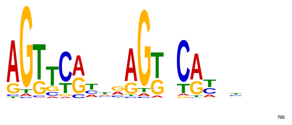	$NNNNNN\overrightarrow{AGTTNN}NNN\overrightarrow{AGTCCA}NNNNNN$	29.74	DR:3	19
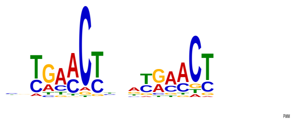	$NNNNN\overrightarrow{NCGTGA}NNNNNN\overleftarrow{NNAACT}NNNNNN$	13.22	IR:6	20

193 PXR-9-Bhimsaria2022-Rnd2 (S07-067)

PWM	Seed Sequence	Seed Seq Enrichment	Repeat	Rank
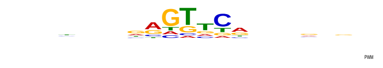	NNNNNNGAGTTCATNNNNNN	5.22	M	1
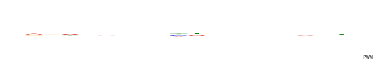	AGATNNNNNNNNNNNATTT	2.16	M	2
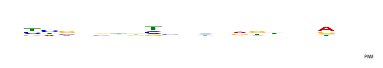	NNNAGTTNNNNNNGTTCNNN	2.46	DR:3	3
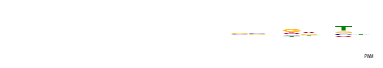	CATGNNNNNNNNNNNNAGTT	2.28	M	4
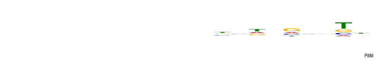	AGGANNNNNNNNNNNNAGTT	2.33	DR:10	5
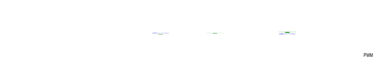	ATTANNNNNNNNNNNCATT	1.81	M	6
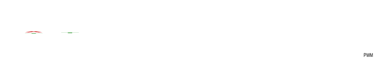	AGTGNNNNNNNNNNNTTCT	2.01	M	7
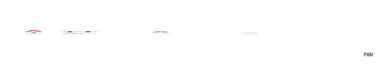	AGTTNNNNNNNNNNNTACT	2.13	M	8
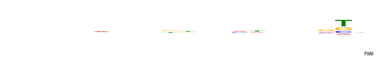	AGCTNNNNNNNNNNNNGGTT	1.88	DR:9	9

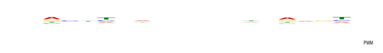	$\overleftarrow{NACTT}NNNNNNNNNN\overrightarrow{AAGTN}$	2.43	ER:9	10
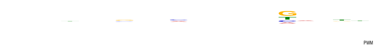	$CATTNNNNNNNNNNNN\overrightarrow{AGTT}$	2.07	M	11
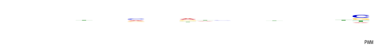	$NAGTGNNNNNNNNNNCTGTN$	1.86	M	12
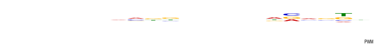	$AAACNNNNNNNNNNNN\overrightarrow{AGTT}$	2.17	M	13
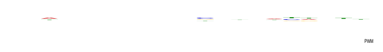	$AATGNNNNNNNNNNNNTGTT$	1.99	M	14
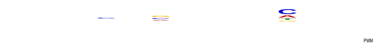	$\overrightarrow{GGTTN}NNNNNNNNNNN\overrightarrow{AAGT}$	1.42	DR:10	15
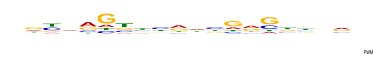	$NNN\overrightarrow{AGTTCN}NNN\overrightarrow{GGTTN}NN$	3.48	DR:2	16
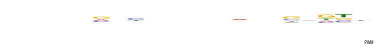	$ATGGNNNNNNNNNNNN\overrightarrow{AGTT}$	2.17	M	17
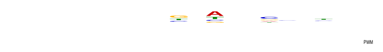	$TTTANNNNNNNNNNNNTTTT$	1.91	M	18

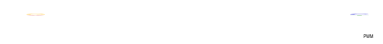	$NATTTNNNNNNNNAAATN$	1.59	M	19
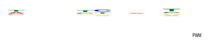	$\overrightarrow{AAGT}NNNNNNNNNNNN\overrightarrow{AGTT}$	2.26	DR:10	20

194 PXR-9-Bhimsaria2022-Rnd3 (S07-092)

PWM	Seed Sequence	Seed Seq Enrichment	Repeat	Rank
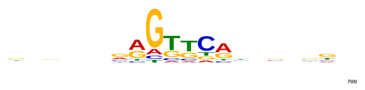	NNNNNNGAGTTCATNNNNNN	14.09	M	1
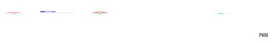	AAGANNNNNNNNNNNNTTTT	3.79	M	2
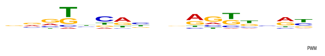	NNGGGTTNNNNNNGTTCNN	7.81	DR:4	3
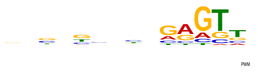	GGGGNNNNNNNNNNNGAGTT	12.95	M	4
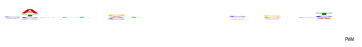	CACTNNNNNNNNNNNNGCTT	4.36	M	5
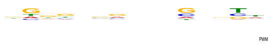	AGCANNNNNNNNNNNNAGTT	4.35	M	6
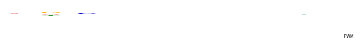	ATGTNNNNNNNNNNNCTCT	3.80	M	7
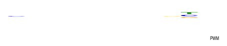	CAGGNNNNNNNNNNNNCGTT	3.94	M	8
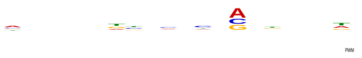	NNCAAGNNNNNNNNGTTCNN	2.86	M	9

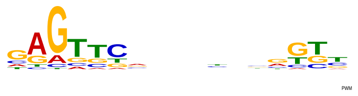	$G\overrightarrow{AGTTC}NNNNNN\overrightarrow{NGGTT}$	16.65	DR:5	10
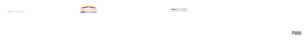	$CATGNNNNNNNNNNNGCTT$	3.52	M	11

195 VDR:RXRA-Bhimsaria2022-Rnd3 (S13-165)

PWM	Seed Sequence	Seed Seq Enrichment	Repeat	Rank
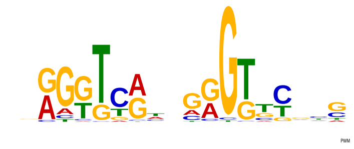	NNNGGTCNNNGGGTTCNNN	142.51	DR:3	1
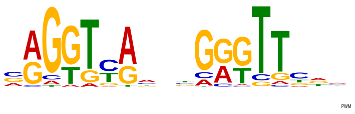	NNGGTCNNNNGGGTTCNN	57.13	DR:4	2
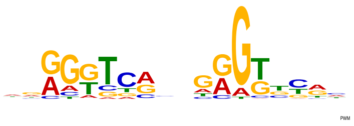	NGGGGTNNNNNNNTTCAN	18.78	DR:3	3
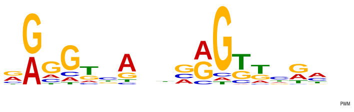	NNGGTTANNNNNNTTCANN	13.07	DR:3	4
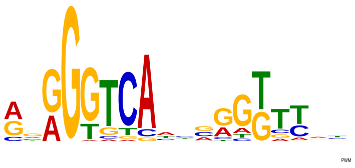	NNNGGTCAACNGGGTNNN	81.31	DR:3	5
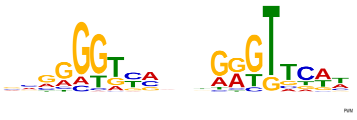	NNGGGGTNNNNNNNGTTCNN	22.44	DR:4	6

196 VDR-RXRA-Rep2-SMiLE-seq-Rnd1 (SRR3405131)

PWM	Seed Sequence	Seed Seq Enrichment	Repeat	Rank
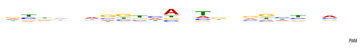	NNNNNAGGTNNNNGGGGNNNNNN	2.40	M	1
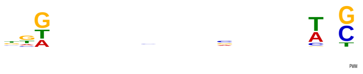	NNNNGGGGNNNNNNNGGGGNNNN	1.64	M	2
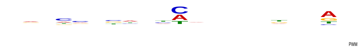	NNNNNTTAANNNATTTNNNNN	1.53	M	3
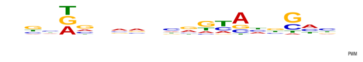	NNNNNNAAACGGTANNNNNNN	2.13	M	4
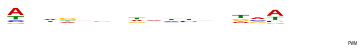	NNNNNNGTATTATTNNNNNN	1.50	M	5
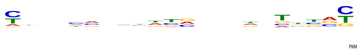	NNNNNNTTTTNNTTTANNNNNN	1.27	M	6
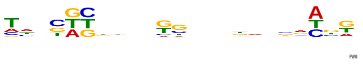	NNNNNNTTATNNNTTTANNNNNN	1.58	M	7
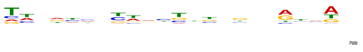	NNNNNNGTTANNNTTGTNNNNNN	1.66	M	8
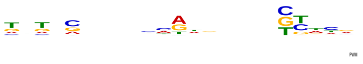	NNNNNGGGGNNNNNGGGGNNNNN	1.60	M	9

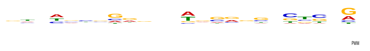	*NNNNNNGGGGNNNNGGGGNNNNNN*	2.51	M	10
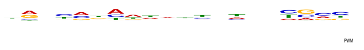	$NNNNNNA\overrightarrow{ATATT}ATNNNNNN$	1.88	M	11

197 VDR-Bhimsaria2022-Rnd2 (S07-054)

PWM	Seed Sequence	Seed Seq Enrichment	Repeat	Rank
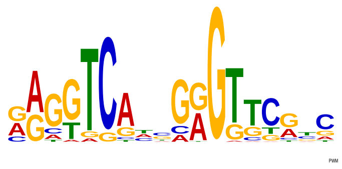	NNNGGTCANNGGGTTCNNN	22.03	DR:3	1
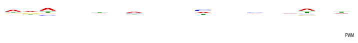	AAAANNNNNNNNNNNNNAAGT	3.17	M	2
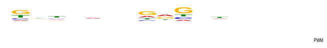	NNNGTCANNNNNTTCANNN	2.19	DR:3	3
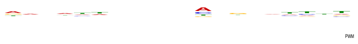	AACANNNNNNNNNNNNTTTT	3.02	M	4
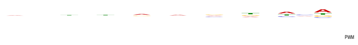	AAGTNNNNNNNNNNNACAT	2.40	IR:7	5
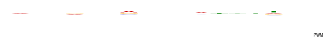	NGAATNNNNNNNNNTTTTN	2.60	M	6
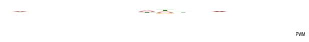	ATCANNNNNNNNNNNAATT	2.54	M	7
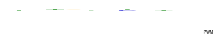	ATTTNNNNNNNNNNTTGT	2.54	M	8
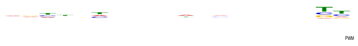	AGTTNNNNNNNNNNNNGGTT	2.21	DR:9	9

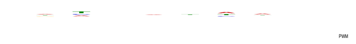	*TCATNNNNNNNNNNNAATT*	2.17	M	10
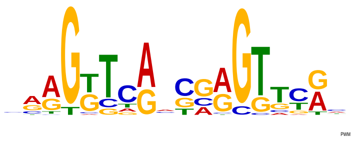	*NNGGGTCNNNGGGTTCNN*	11.80	DR:3	11
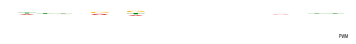	*ATTTNNNNNNNNNNNAGTT*	2.74	M	12
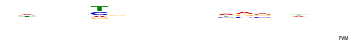	*GATANNNNNNNNNNNATTT*	2.35	M	13
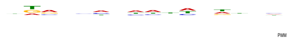	*NAAGANNNNNNNNNNNTTTTN*	2.57	M	14
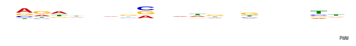	*CAGANNNNNNNNNNNNTGTT*	2.37	M	15
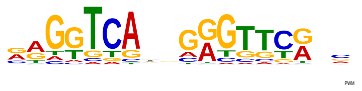	*NNNGTCANNGGGTTCNNN*	10.94	DR:3	16
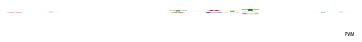	*ATTGNNNNNNNNNNNTTTT*	2.98	M	17
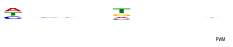	*ATAGNNNNNNNNNNNATTT*	2.53	M	18

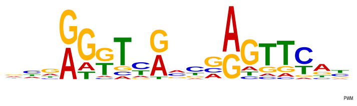	$NNG\overrightarrow{GGGTCN}NNN\overrightarrow{NGTTCN}N$	6.63	DR:3	19
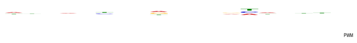	$ATAANNNNNNNNNNNTTTT$	3.05	M	20

198 VDR-Bhimsaria2022-Rnd3 (S07-173)

PWM	Seed Sequence	Seed Seq Enrichment	Repeat	Rank
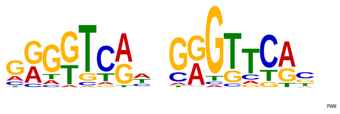	NNNGGTCNNNGGGTTCNNN	30.80	DR:3	1
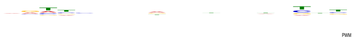	AGTTNNNNNNNNNNNNGTTT	4.89	M	2
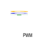	GGGGNNNNNNNNNNNNGGGG	4.09	M	3
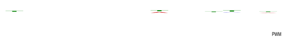	ATTGNNNNNNNNNNNNTTGT	4.42	M	4
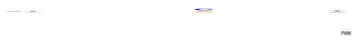	ATTANNNNNNNNNNNNTTAT	4.27	M	5
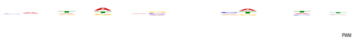	CAGTNNNNNNNNNNNNATGT	4.11	M	6
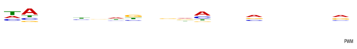	NAGCTNNNNNNNNNNGTTTN	3.73	M	7
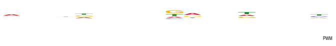	CATGNNNNNNNNNNNNCATT	3.84	M	8
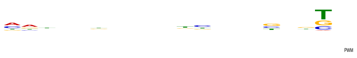	AATGNNNNNNNNNNNNGGTT	4.10	M	9

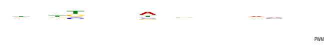	$AGTGNNNNNNNNNNN\overrightarrow{AAGT}$	3.81	M	10
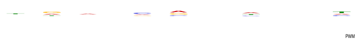	$TCGANNNNNNNNN\overrightarrow{NNNTCAT}$	3.42	M	11
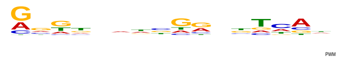	$N\overrightarrow{NGGTCA}NNNNGTGCNN$	4.20	M	12
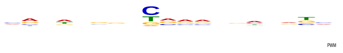	$NCAGANNNNNNNNNNAAATN$	5.15	M	13
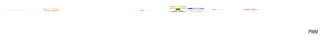	$\overrightarrow{AGGANN}NNNNNNNNNACTG$	3.66	M	14
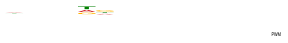	$CATANNNNNNNNNNNNTTGT$	3.96	M	15
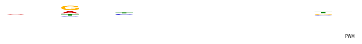	$ATCGNNNNNNNNNNNAATT$	3.55	M	16
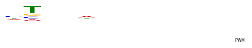	$\overrightarrow{NAGTCN}NNNNNNNNNTCGTN$	3.29	M	17
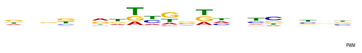	$NCGTANNNNNNNNNNCATGN$	3.74	M	18

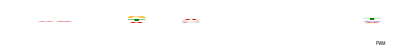	$AATCNNNNNNNN\overrightarrow{NNNTCA}T$	3.62	M	19
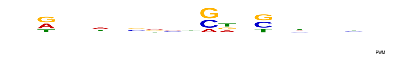	$NATTCNNNNNNNNNTAATN$	3.73	M	20

199 VDR-Jolma2013-Rnd2 (ERR188863)

PWM	Seed Sequence	Seed Seq Enrichment	Repeat	Rank
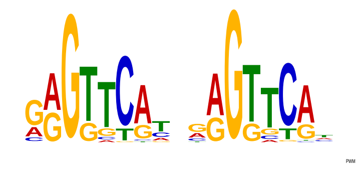	NNAGTTCNNNNNGTTCANN	818.55	DR:3	1
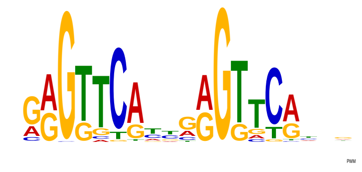	NNNNTTCANNNGGTTCNNNN	266.87	DR:3	2
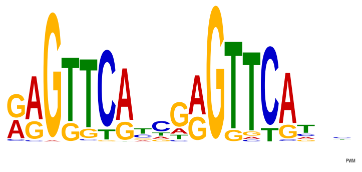	NAGTTCNNNNNNTTCATTN	517.88	DR:3	3
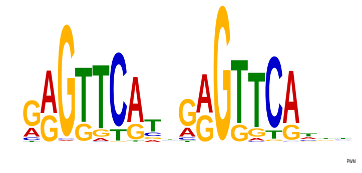	NNAGTTCNNNNNNNNTCATNN	186.37	DR:3	4
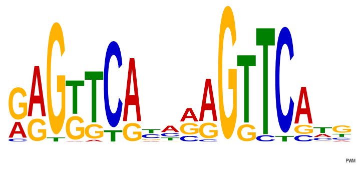	NAGGTCACNNNGTTCATN	1462.50	DR:3	5
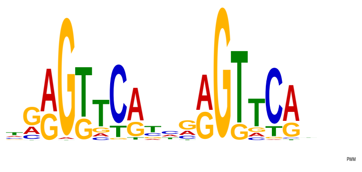	NNGGTTCNNNNNNNNTCATNN	117.13	DR:4	6
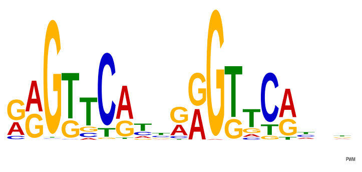	NNNNTCATTNGGTTCNNNN	228.96	DR:3	7
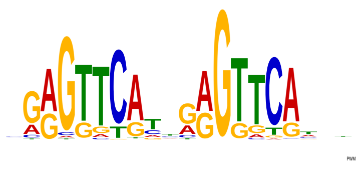	NNAGTTNNNNNNNNNTCATNN	54.61	DR:3	8
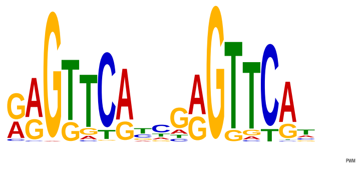	NAGTTCNNNNNNNNTCATTN	170.06	DR:3	9

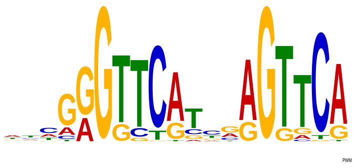	$NTC\overrightarrow{GGGTTC}NNNN\overrightarrow{NGTTCN}$	1224.16	DR:4	10
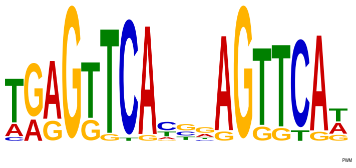	$NN\overrightarrow{AGTTCA}CGG\overrightarrow{AGTTCN}N$	4376.08	DR:3	11

200 VDR-Jolma2013-Rnd3 (ERR188864)

PWM	Seed Sequence	Seed Seq Enrichment	Repeat	Rank
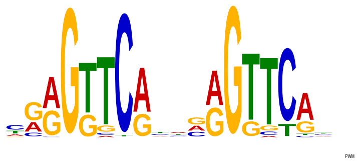	NNNGGTCNNNNNGTTCNNN	49.84	DR:3	1
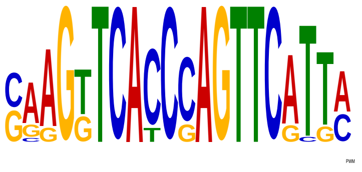	NAAGGTCACNNNNNNCATTN	180.16	M	2
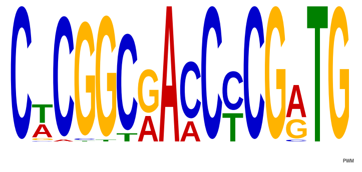	CACGGCNNNNNCGATG	80.53	M	3
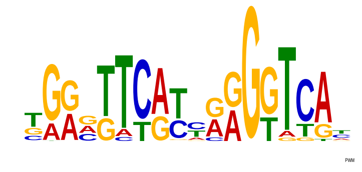	ATGAGNNNNNNNNNNTCAG	17.80	ER:5	4
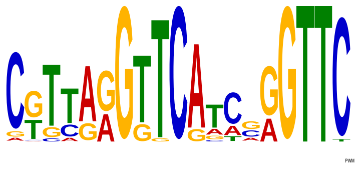	CTTTAGGNNNNNNNNGTTC	121.00	DR:4	5
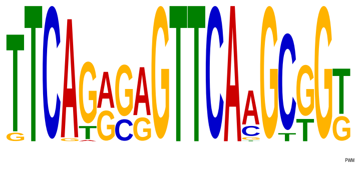	NTCAGNNNNNNCAAGCGGN	96.66	M	6
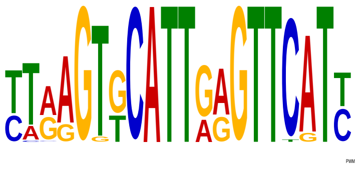	NTAAGTGCNNNNNNNNTCATN	190.49	DR:4	7
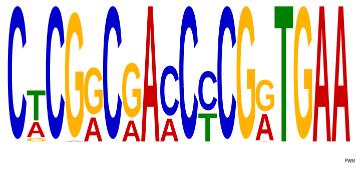	CACGGCNNNNNNGATGAA	136.11	DR:4	8
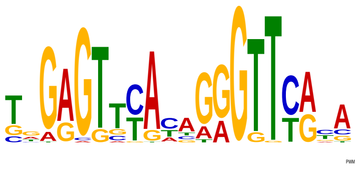	NAGAGNNNNNNNNNTTCAGN	17.92	M	9

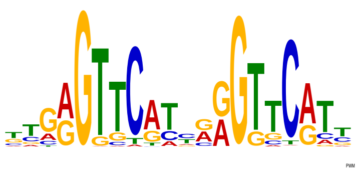	NNNAGTTNNNNNNGTGCNNN	14.50	M	10
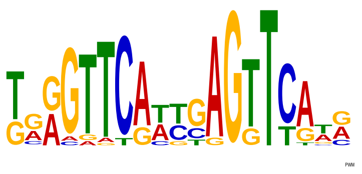	NCAGTTNNNNNNNTTCAGN	17.35	DR:3	11
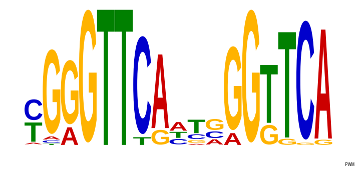	ACGGGNNNNNNNNNNNTCAG	23.24	M	12
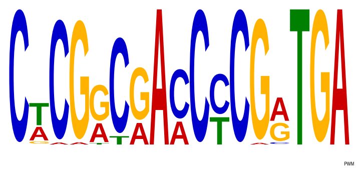	CACGGCNNNNNNGATGA	76.87	DR:4	13
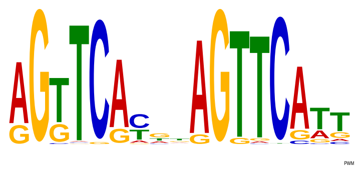	NNNTCACGTNGTTCNNN	69.29	ER:0	14
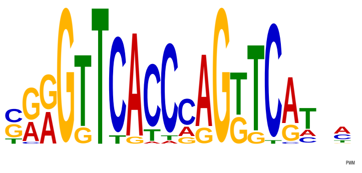	NAAGGTCNNNNNNNNCATTN	31.10	M	15
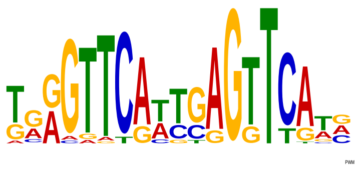	NCAGTTNNNNNNNTCAGN	8.27	DR:3	16
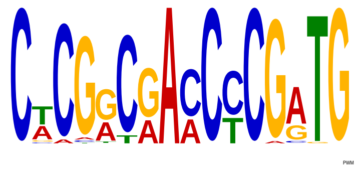	CACGGCNNNNNNGATG	29.65	M	17
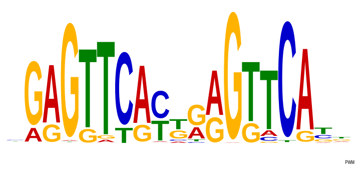	AGAGNNNNNNNNNNNCATT	25.00	M	18

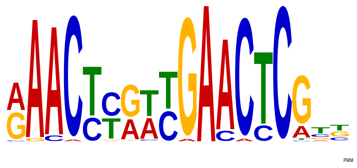	*AAACNNNNNNNNNCTCGTT*	34.38	M	19
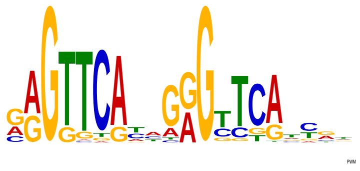	$N\overrightarrow{NNNTCA}GNN\overrightarrow{NNNTTCA}NNNN$	16.53	DR:3	20

201 Vdr-Jolma2013-Rnd3 (ERR195763)

PWM	Seed Sequence	Seed Seq Enrichment	Repeat	Rank
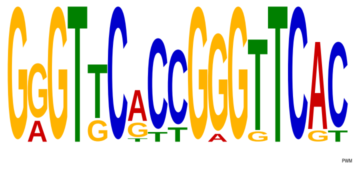	NGGTTCANTGGGTTCAN	6396.57	DR:3	1
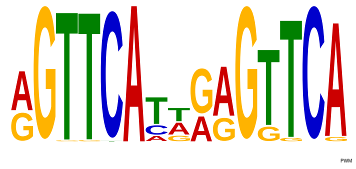	AGTTCATNNNGGTCA	3130.89	DR:3	2
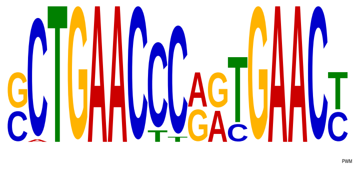	NNTGAACTCGNTGAANN	2676.94	ER:0	3
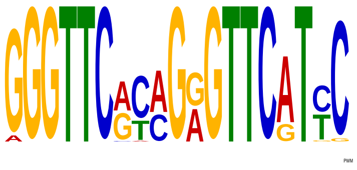	NNGTTCGCNNNGTTCATNN	3188.40	DR:3	4
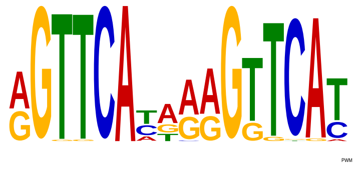	AGTTCANNNNGGTCAT	2879.97	DR:3	5
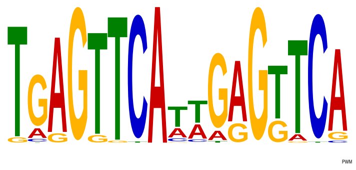	NGAGTTCANNNNGGTCN	657.00	DR:3	6
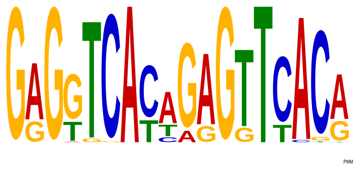	NNGGTCANNNAGTTCANN	890.35	DR:3	7
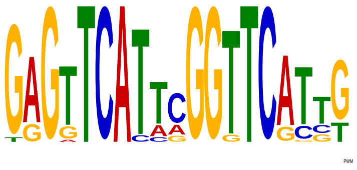	NNNTTCATNNGGTTCGNNN	561.79	DR:3	8
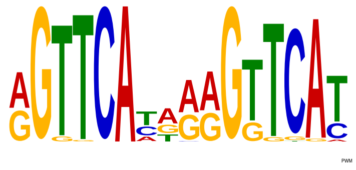	AGTTCANNNNNGTCAT	878.68	DR:3	9

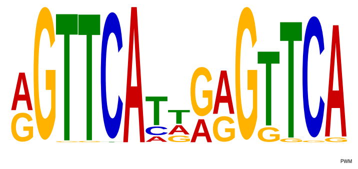	$\overrightarrow{AGTTCA}TNNN\overrightarrow{NGTCA}$	909.71	DR:3	10
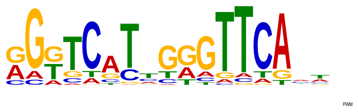	$\overrightarrow{NNNTCA}GNN\overrightarrow{NNTTCA}NNN$	11.57	DR:3	11
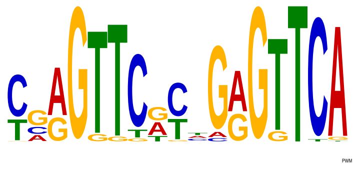	$\overleftarrow{TGGGTT}CNNNN\overrightarrow{NNTTCA}$	604.07	ER:5	12
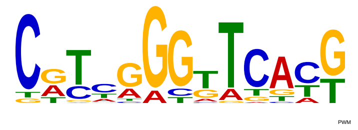	$NNGCG\overrightarrow{GGTTCA}NN$	8.70	M	13
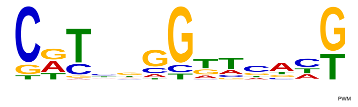	$NNGCG\overrightarrow{GNTTCA}NN$	3.23	M	14
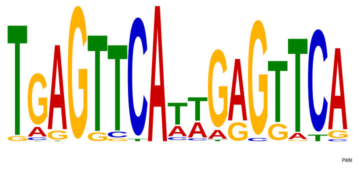	$NG\overrightarrow{AGTTCN}NNNN\overrightarrow{NGGTCN}$	164.52	DR:3	15
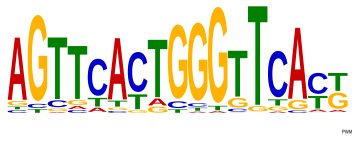	$\overrightarrow{NNTTCA}NNNNNTGCANN$	12.01	M	16
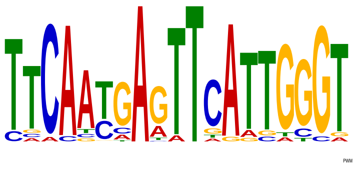	$NNNNNCG\overrightarrow{AGNTCA}TNNNNN$	20.28	M	17
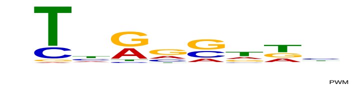	$GCG\overrightarrow{GGTTC}$	2.31	M	18

202 VDR-Jolma2013-Rnd4 (ERR188865)

PWM	Seed Sequence	Seed Seq Enrichment	Repeat	Rank
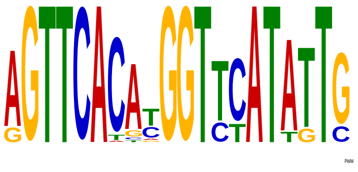	NNNNNACACNNTTCANNNNN	22.81	M	1
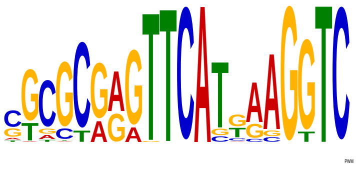	NGCGCNNNNNNNNNNAGGTN	26.36	M	2
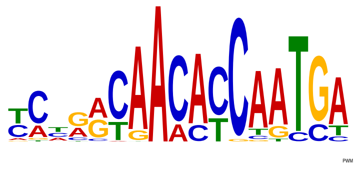	NNNGACANNNCCAANNN	24.29	M	3
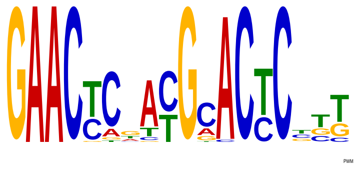	NNNNTCGANNCACTNNNN	12.08	M	4
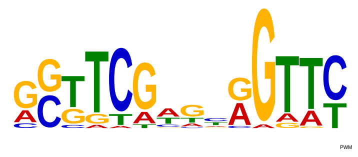	NNTTCGNNCAGTNN	8.83	M	5
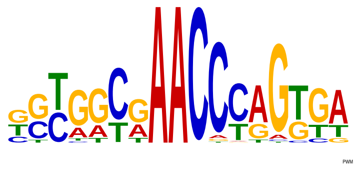	NNNGGCGNNNCCAGNNN	12.81	M	6
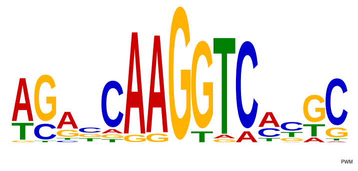	NNAGCANNNTCACNN	18.24	M	7
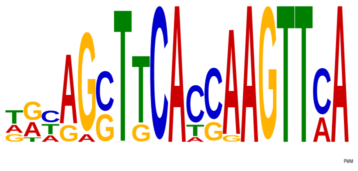	NNNAGGTTNNNCAAGTNNN	123.36	DR:3	8
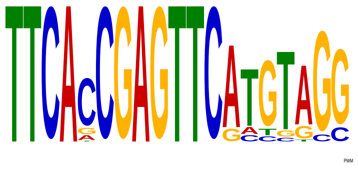	NTCAGNNNNNNNNNGTAGN	7.34	M	9

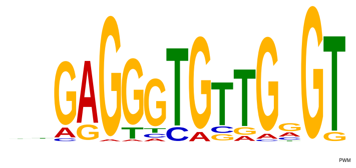	NNGAGGGNNTTGGNN	26.61	M	10
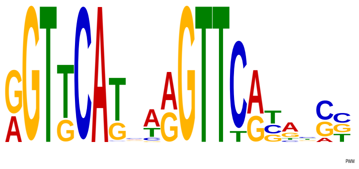	NNNNNATCTNNTTCANNNNN	9.05	ER:0	11
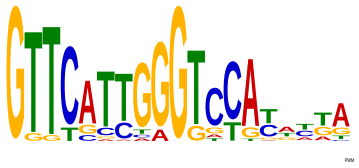	NNNNNTTGGNTCCANNNNN	10.49	M	12
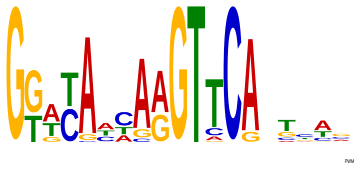	NNNNATCANNNTCAANNNN	12.54	DR:0	13
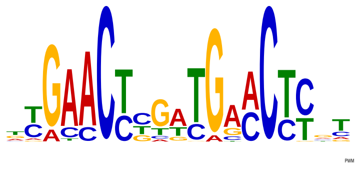	NCGAANNNNNNNNNCTCAN	10.12	M	14
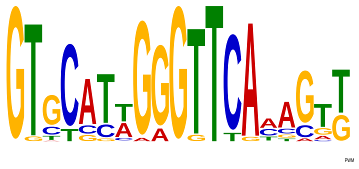	NNGCATNNNNNNNAAAGNN	11.03	M	15
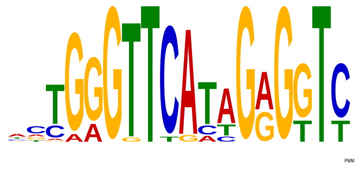	TCTGGNNNNNNNNNGGTC	12.62	M	16
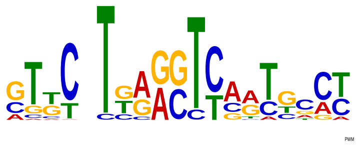	NNGCGTNNNNNNNATGCNN	6.20	M	17
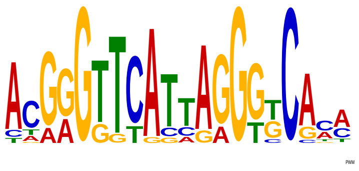	NAGGGNNNNNNNNNNTCAAN	9.00	DR:5	18

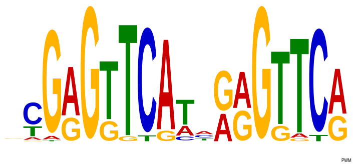	*GCGGNNNNNNNNNNTTCG*	8.05	M	19
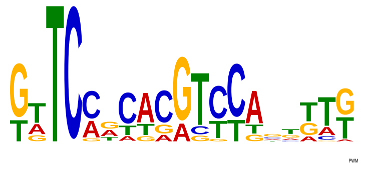	*NNNCAGCNNNNNCAGTNNN*	5.78	M	20

203 Vdr-Jolma2013-Rnd4 (ERR195764)

PWM	Seed Sequence	Seed Seq Enrichment	Repeat	Rank
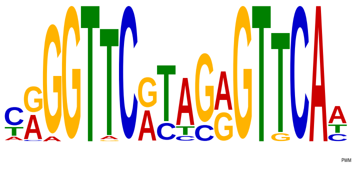	NAGGTTCNNNNNNTTCAN	600.10	DR:4	1
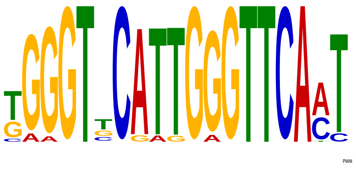	NNGGTGCANNNNNTTCANN	161.96	DR:4	2
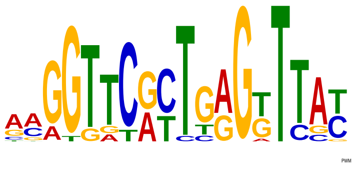	NNAGGTCNNNNNGTTCNN	31.59	DR:3	3
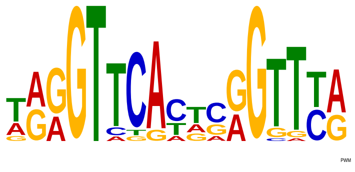	NNAGTTCNNNAGGTTNN	97.33	DR:2	4
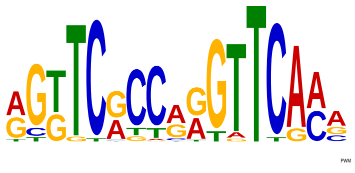	NNTTCGGNNNNNTTCANN	28.56	M	5
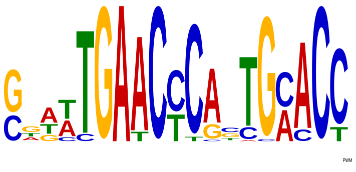	NNNNTGAANNCACTGNNNN	48.22	M	6
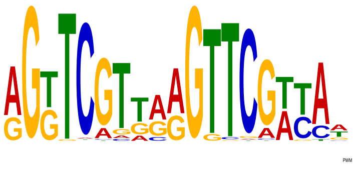	NNNTCGTNNNNNTCGTNNN	63.03	M	7
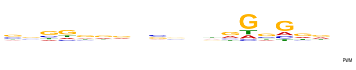	NNNNGGGGNNNGGGGNNNN	4.53	M	8
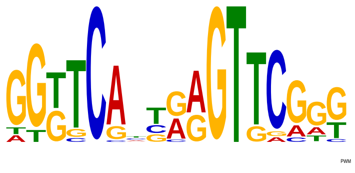	NNNTCAANNAGTTCNNN	28.13	DR:3	9

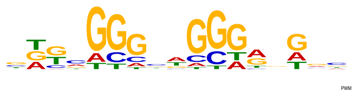	NNNNGGGCNGGGANNNN	3.92	M	10
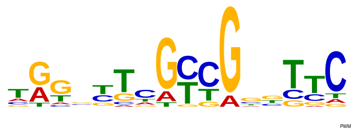	NNNNTTCGGTGGNNNN	2.16	M	11
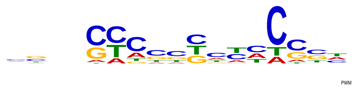	NNNTCCCNNNCTCCNNN	2.79	M	12
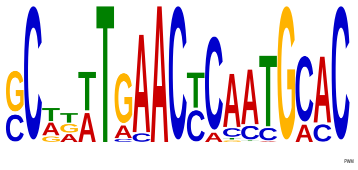	NNNNATGANNNCACTNNNN	9.04	M	13
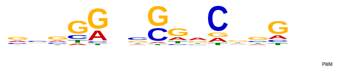	NNAGGGNNNNNAGGGNN	2.92	DR:3	14
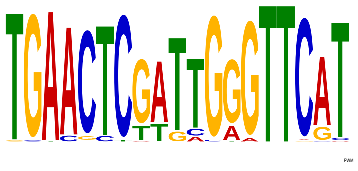	NNAACTNNNNNNNGTTCNN	63.70	ER:6	15
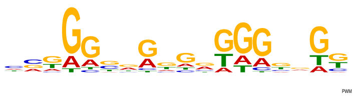	NNNNGGAGNNAGGGNNNN	3.57	M	16
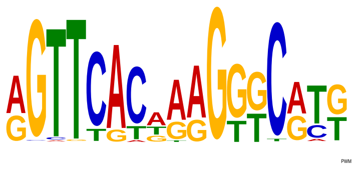	AGTTNNNNNNNNNCATT	47.72	M	17
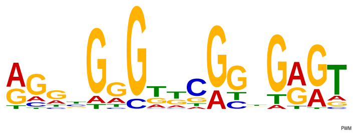	NGGTGNNNNNNGTGGGN	4.91	M	18

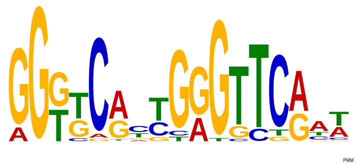	$\overrightarrow{NNNTCA}GNN\overrightarrow{NGTTCN}NN$	17.71	DR:3	19
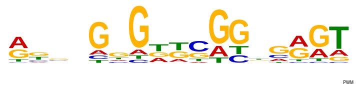	$\overrightarrow{NGGTGN}NNNNNNTGGGN$	2.79	M	20

204 VDR-SMiLE-seq-Rnd1 (SRR3405095)

PWM	Seed Sequence	Seed Seq Enrichment	Repeat	Rank
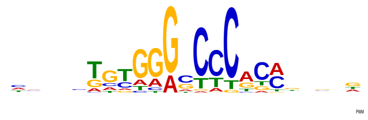	*NNNNNNGTGGNNCCCACNNNNNN*	7.62	M	1
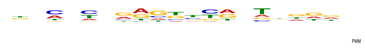	*NNNNNNGAGTTCATNNNNNN*	2.73	M	2
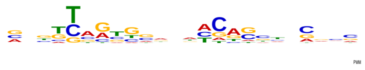	*NNNNNNGTGGNNNNCAGCNNNNNN*	2.53	M	3
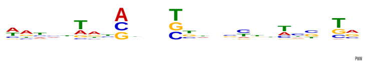	*NNNNTTATNNNNNNNNNNTTTCNNNN*	2.12	M	4
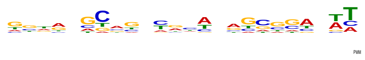	*NNNNNGCAGNNNNNNNAGCGNNNNN*	2.63	M	5
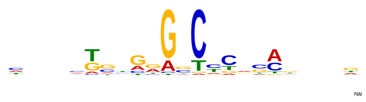	*NNNNNNGTGGNNNCCACNNNNNN*	2.56	M	6
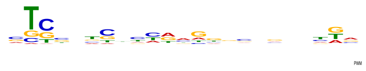	*NNNNNTCTCNNNNNGCAGNNNNN*	1.87	M	7
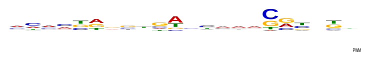	*NNNNNNGGTGNNCAAANNNNNNN*	1.95	M	8
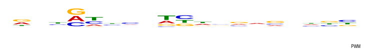	*NNNNNTCGTNTCGANNNNN*	1.57	M	9

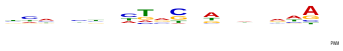	NNNNTCTTNNNNNAATCNNNN	1.46	M	10
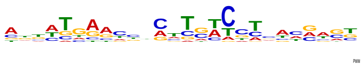	NNNNNGAACNNNNNNNNCTCANNNNN	3.07	ER:5	11
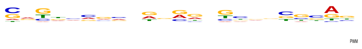	NNNNNCGCGNNNNNGCGGNNNNN	1.92	M	12
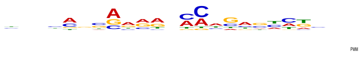	NNNNNGCAANNNNNNGAGTNNNNN	1.94	M	13
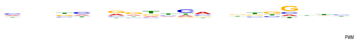	NNNNNNGGTTCACGNNNNNN	2.27	M	14
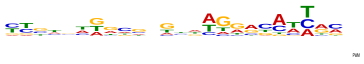	NNNNNNGGCGNNNNNGGACNNNNNN	2.63	M	15
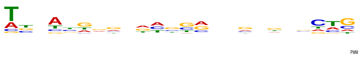	NNNNTGGGNNNNNNNNGCGANNNN	1.85	M	16
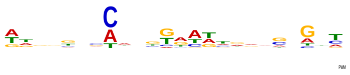	NNNGGGTNNNNNNNNNNNAAGGNNN	1.64	DR:10	17
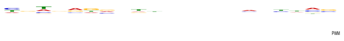	NNNNNNGAGTNNACAANNNNNN	1.76	M	18

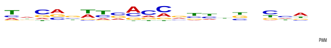	NNNNN$\overrightarrow{NGTTC}$NNNGTTTNNNNNN	1.92	M	19
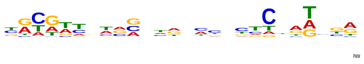	NNNNTTGTNNNNNNNNNNTCTANNNN	2.39	M	20

205 FXR-Bhimsaria2022-Rnd3 (S07-163)

PWM	Seed Sequence	Seed Seq Enrichment	Repeat	Rank
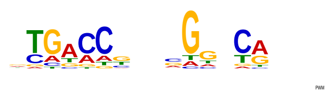	NNNNGACCNNNNNGGGCNNNN	22.23	ER:2	1
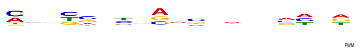	NGGTCANNNNNNNNAGCAN	2.22	M	2
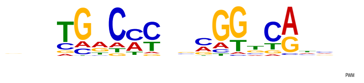	NNNNGACACNNNGGTCNNNN	19.01	DR:3	3
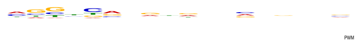	NGGTCANNNNNNNCAGCN	4.36	M	4
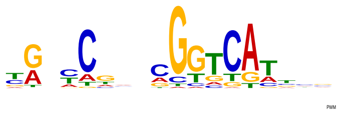	NNNCTCGNNNGGTCATNNN	21.16	M	5
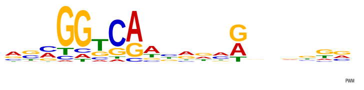	NNCGGTCANNNNNNCCAGNN	22.41	M	6
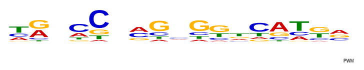	NNCTCGNNNGGTCAANN	8.56	M	7
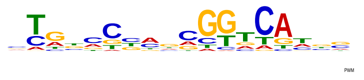	NNGCTCNNNNGGTCAANN	15.29	DR:2	8
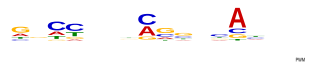	NNNNCTCGCNNGGTCNNNN	5.55	M	9

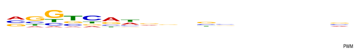	NGGTCANNNNNNNTAGCN	5.79	M	10
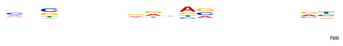	NNGGTCNNNNNNNNCAGCNN	1.66	M	11
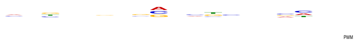	NGGTCNNNNNNNNNAGCAN	2.15	M	12
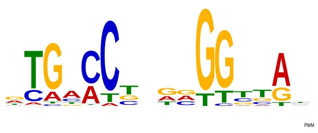	NGGTGACCNNNNGGTTAN	173.22	ER:2	13
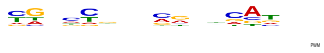	NNNNCTCGNNCGGTCNNNN	7.73	M	14
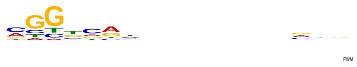	NGGTCANNNNNNNGAGCN	7.35	IR:6	15
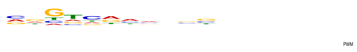	NGGTCANNNNNNNAAGCN	5.18	M	16
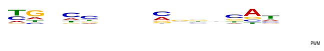	NNNNCTCGNNNGGTCNNNN	4.59	M	17
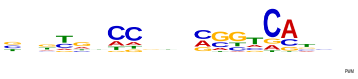	NNNNGCTCGNNNGGTCNNNN	8.95	DR:2	18

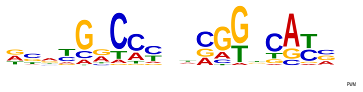	*NNNNGACACCNNGGTCNNNN*	18.41	DR:3	19
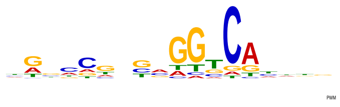	*NNNCTCTNNNGGTCATNNN*	10.69	M	20

206 FXR-11-Bhimsaria2022-Rnd3 (S07-091)

PWM	Seed Sequence	Seed Seq Enrichment	Repeat	Rank
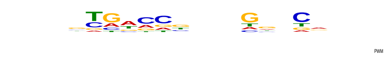	NNNNGACCNNNNGGGCNNNN	9.53	ER:2	1
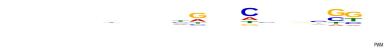	GGGGNNNNNNNNNNNNCGGT	6.71	M	2
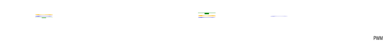	GTCANNNNNNNNNNNCCAC	3.33	M	3
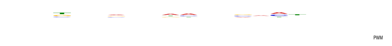	GGTCNNNNNNNNNNNGCCA	2.63	DR:8	4
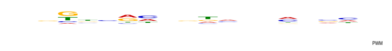	GGTCANNNNNNNNAGCA	3.95	M	5
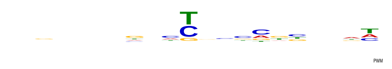	NCTGTNNNNNNNNGGTCAN	2.99	M	6
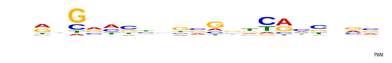	NNNNNCTAGNGGTCGNNNNN	7.17	M	7
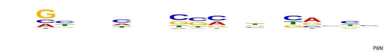	NNNCTCGNNNGGTCGNNN	3.30	M	8
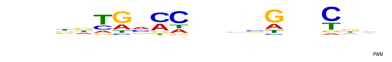	NNATGACCNNNNGGACNN	15.60	ER:2	9

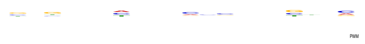	$\overrightarrow{NGGTCN}NNNNNNNN\overrightarrow{NGGTCN}$	3.79	DR:8	10
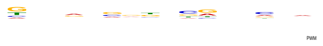	$\overrightarrow{NGGTCA}NNNNNNNCAGCN$	3.47	M	11

207 LXRA-Bhimsaria2022-Rnd3 (S07-143)

PWM	Seed Sequence	Seed Seq Enrichment	Repeat	Rank
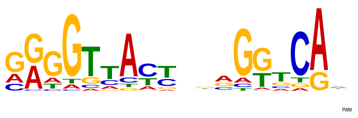	NNGGTTACNNNNGGTCNN	15.57	DR:4	1
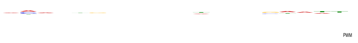	ACAGNNNNNNNNNNNNAATT	4.89	M	2
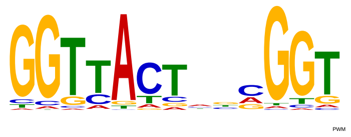	GGTTACTNNCGGT	60.46	DR:4	3
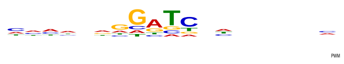	NNNNNNAGGATCAANNNNNN	7.94	M	4
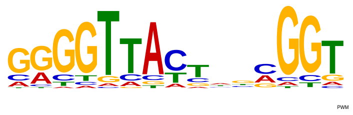	GGGGTTANNNNCGGT	41.29	DR:4	5
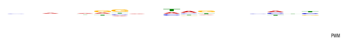	NNNNNAGGANNAGTTNNNNN	4.82	DR:0	6
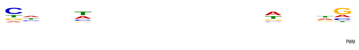	NNNNNNTGACGTCANNNNNN	1.96	M	7
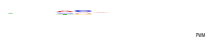	GAATNNNNNNNNNNNTTTT	3.37	M	8
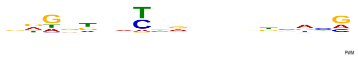	NNNGTTANNNNCGGTCNNN	4.26	DR:4	9

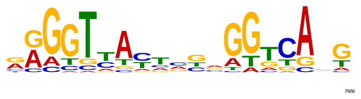	$N\overrightarrow{NNGTTA}CTNN\overrightarrow{NGGTCN}NN$	13.70	DR:4	10

208 LXRA-12-Bhimsaria2022-Rnd3 (S07-142)

PWM	Seed Sequence	Seed Seq Enrichment	Repeat	Rank
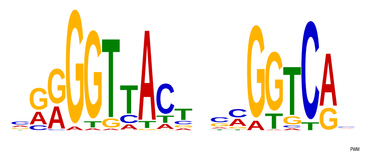	NNGGGTTACTNNNGGTCNN	195.15	DR:4	1
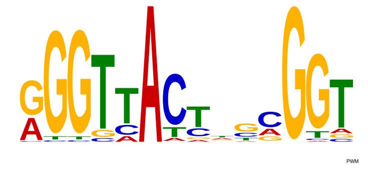	GGGTTACTNGCGGT	535.65	DR:4	2
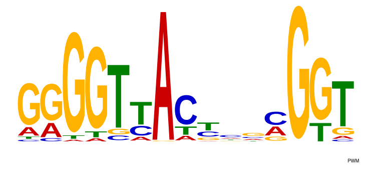	GGGGTTACNNGAGGT	329.02	DR:4	3
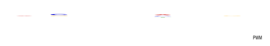	AAATNNNNNNNNNNNNATGT	3.90	M	4
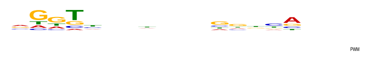	NNNNTACTNNNGGTCNNNN	3.61	M	5
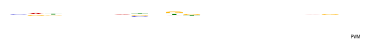	CATTNNNNNNNNNNNNGAGT	3.84	M	6
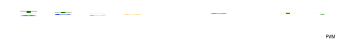	AGTCNNNNNNNNNNNNTTGT	3.87	M	7
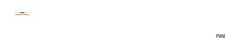	GAGTNNNNNNNNNNNNTGGG	3.27	M	8
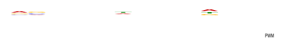	ACTGNNNNNNNNNNNNAAGT	3.38	M	9

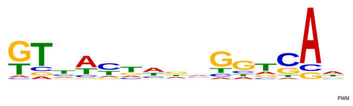	$NNTACTNG\overrightarrow{CGGTCN}N$	5.75	M	10
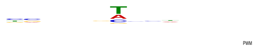	$GATTNNNNNNNNNN\overrightarrow{NNGTGA}$	3.03	M	11
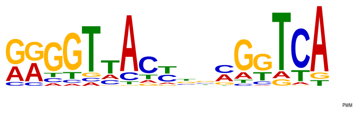	$N\overrightarrow{NGGTTA}CNNN\overrightarrow{GGGTCN}N$	26.16	DR:4	12
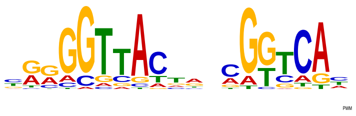	$NN\overrightarrow{GGGTTA}TNNN\overrightarrow{NGGTCN}N$	25.82	DR:4	13

209 LXRB:RXRA-Bhimsaria2022-Rnd3 (S13-142)

PWM	Seed Sequence	Seed Seq Enrichment	Repeat	Rank
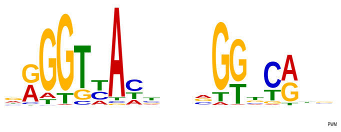	NNNGGTTACNNNNGGTCNNN	561.77	DR:4	1
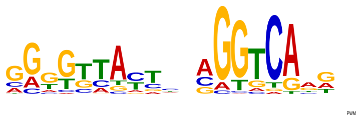	NNNNCTACTNNNGGTCNNNN	23.10	M	2
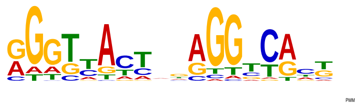	NNNNTACTAGNGGTCNNNN	76.10	M	3
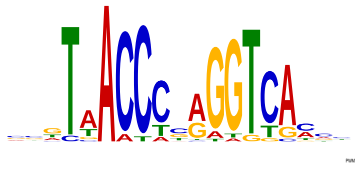	NNNTAACCCNAGGTCANNN	755.76	ER:1	4
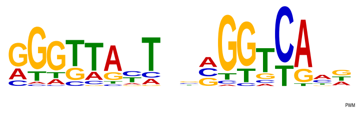	NNNCTACTANNGGTCNNN	26.37	M	5
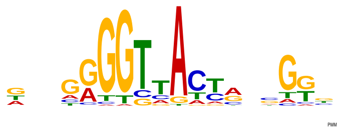	NNNNGGGGTTACTATNNNN	714.87	M	6
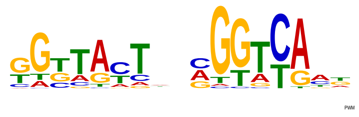	NNCTACTANNGGTCANN	79.93	M	7
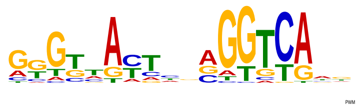	NNNCTACTNNAGGTCNNN	41.09	M	8
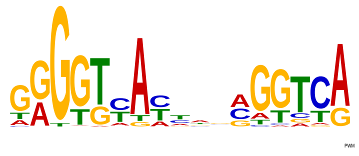	NAGTTCACNNNNGGTCN	168.08	DR:4	9

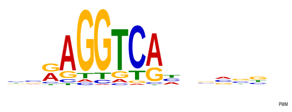	NNNNNNAAGGTCATNNNNNN	48.42	M	10
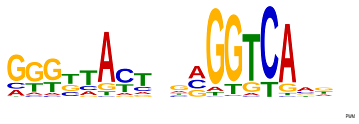	NNNCTACTNNNGGTCANNN	63.42	M	11
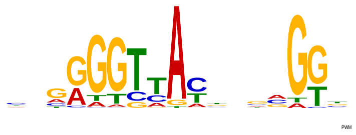	NNGGGGTTACNTGAGNN	1001.86	IR:2	12
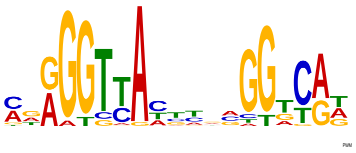	NNGGGTTAANNNCGGTCNN	593.42	DR:4	13
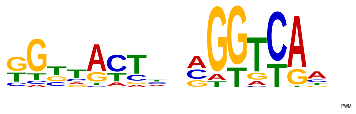	NNCTACTNNAGGTCANN	104.87	M	14
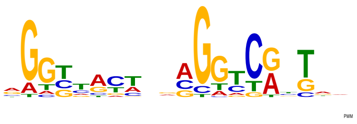	NNNNNACTAGNGGTCNNNNN	26.14	M	15
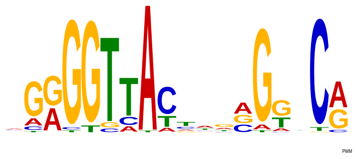	NGGGGTTACNNNNGGACN	1176.99	DR:4	16
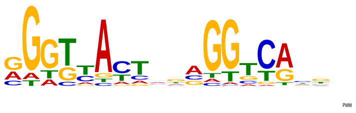	NNNNTACTANNGGTCNNNN	45.51	M	17
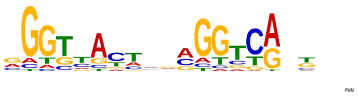	NNNNNACTANNGGTCNNNNN	18.90	M	18

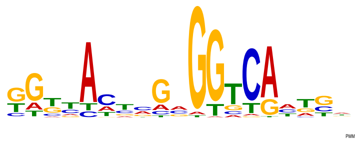	$NNNTACTNN\overrightarrow{NGGTCA}TNNN$	73.50	M	19

210 PPARa-RXRG-SMiLE-seq-Rnd1 (SRR3405126)

PWM	Seed Sequence	Seed Seq Enrichment	Repeat	Rank
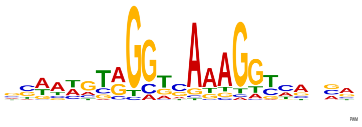	NNNNNNTAGGNNAAAGGNNNNNN	12.39	DR:1	1
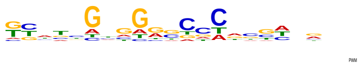	NNNNNNTGGGNNCCACNNNNNN	2.75	M	2
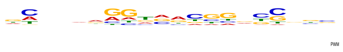	NNNNNNAGGTNACGGNNNNNN	2.79	M	3
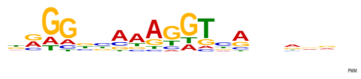	NNNNNNAAAGGTCANNNNNN	5.12	M	4
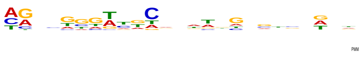	NNNNNGGTTNNNNNNNGGGTNNNNN	2.30	DR:6	5
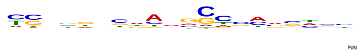	NNNNNNCAAAGCCCNNNNNN	2.14	M	6
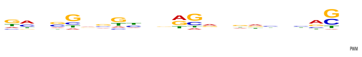	NNNNNNGGTGNNNGCGGNNNNNN	1.79	M	7
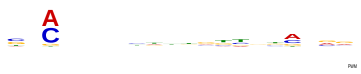	NNNNNNGTTTTGTTNNNNNN	1.50	M	8
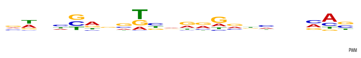	NNNNNAGGTNNNNGGTANNNNN	2.06	DR:1	9

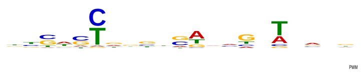	*NNNNNNGTGTNAAAGNNNNNN*	2.09	M	10
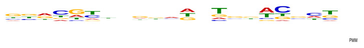	*NNNNNNTGGTNNTTGTNNNNNN*	1.32	M	11
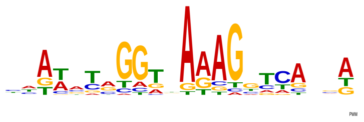	*NNNNNTAGGNNNAAGGTNNNNN*	8.80	DR:1	12
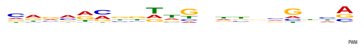	*NNNNNNGGTTGTTTNNNNNN*	1.66	M	13
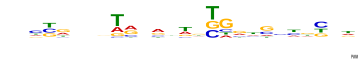	*NNNNNNTGTGNNNNNNGGTGNNNNNN*	2.20	M	14
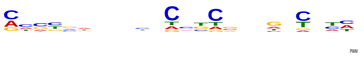	*NNNNGTGGNNNNNNNNGGGTNNNN*	1.46	M	15
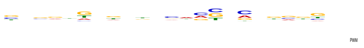	*NNGGTGNNNNNNNNNNNNGGGGNN*	2.11	M	16
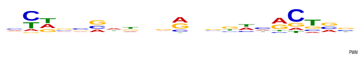	*NNNNNNATTGNGGGTNNNNNN*	1.70	M	17
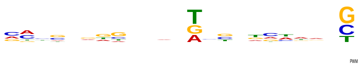	*NNNNNNGGTTNNNGGGTNNNNNN*	1.78	DR:2	18

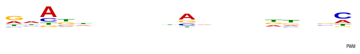	*NNNNNTTGGNNAGGGNNNNN*	1.32	M	19
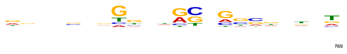	*NNNGTGGNNNNNNNNNGGGTNNN*	1.35	M	20

211 PPARD-Bhimsaria2022-Rnd3 (S07-148)

PWM	Seed Sequence	Seed Seq Enrichment	Repeat	Rank
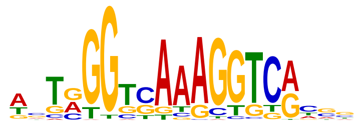	NNNNGGTCAAAGGTCNNNN	92.78	DR:1	1
	GGGGNNNNNNNNNNNNNGGGG	6.20	M	2
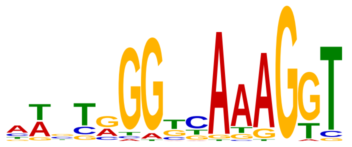	ATGTGGGNNAAAGGT	192.18	DR:5	3
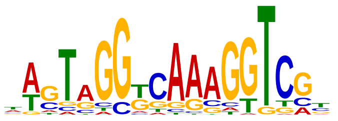	NNAGTANNNNAAAGGTCNN	34.51	M	4
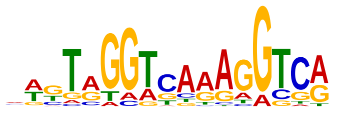	NNAGTGNNNCAAAGGTNN	19.39	M	5
	NNNNNNTGACGTCANNNNNN	2.05	M	6
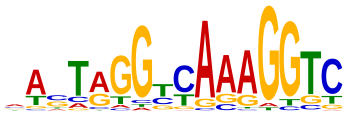	NACTAGNNNAAAGGTN	24.68	DR:1	7
	AGGTNNNNNNNNNNNNCATG	3.78	M	8

212 PPARD-Yin2017-Rnd2 (ERR1005720)

PWM	Seed Sequence	Seed Seq Enrichment	Repeat	Rank
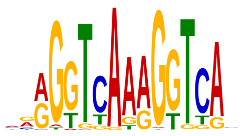 PWM	NNNNNNGGTCAANGGTCANNNNNN	1227.50	DR:1	1
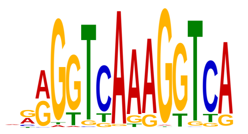 PWM	NNNNNNGTCAANNGTCANNNNNN	190.10	DR:1	2
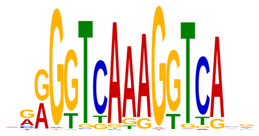 PWM	NNNNNNTCAANNGTCACGNNNNNN	110.73	DR:1	3
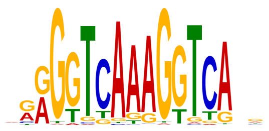 PWM	NNNNNNTCAANNGTCACNNNNNN	69.98	DR:1	4
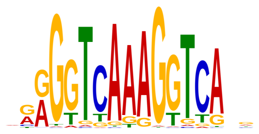 PWM	NNNNNNGTCANNNGTCACNNNNNN	64.92	DR:1	5

213 PPARD-Yin2017-Rnd2 (ERR1005721)

PWM	Seed Sequence	Seed Seq Enrichment	Repeat	Rank
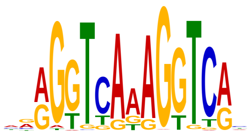	NNNNNNGGTCANAGGTCNNNNNN	590.15	DR:1	1
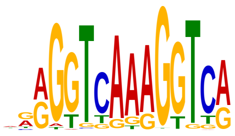	NNNNNNGTCAANNGTCANNNNNN	277.86	DR:1	2
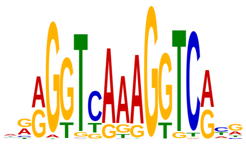	NNNNNNGTCAAANNTCGCGNNNNNN	336.61	DR:1	3
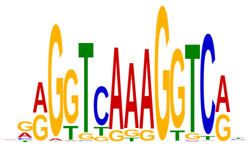	NNNNNNTCAAANGTCGTNNNNNN	220.52	DR:1	4
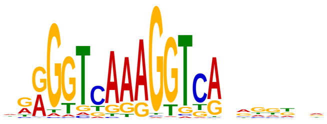	NNNNNNGTCANNNNTCAANNNNNN	35.46	DR:1	5
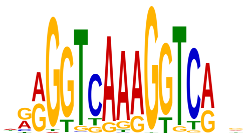	NNNNNNGTCAANNNTCATGNNNNNN	177.59	DR:1	6
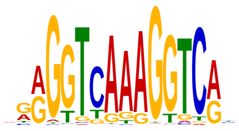	NNNNNNGTCAANNNTCGTNNNNNN	69.15	M	7
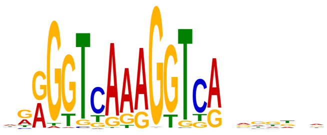	NNNNNNGTCAANNNTCAANNNNNN	112.25	DR:1	8
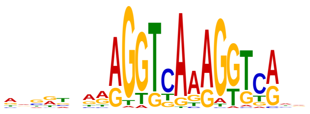	NNNNNNCAAAGNNNAAAGGTCNNNNNN	676.39	DR:1	9

214 PPARD-Yin2017-Rnd3 (ERR1005722)

PWM	Seed Sequence	Seed Seq Enrichment	Repeat	Rank
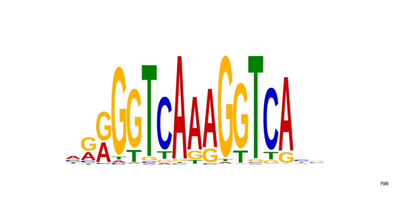	NNNNNNGTCANNNGTCANNNNNN	111.95	DR:1	1
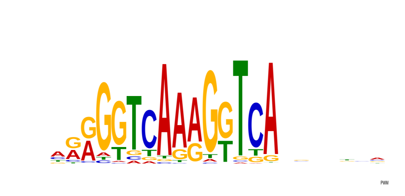	NNNNNNTCAANNNTCAANNNNNN	61.70	DR:1	2
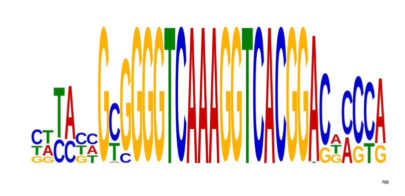	NNNNNNGCGGGGTCNNNNGTCACGGANNNNNN	14036.39	DR:1	3
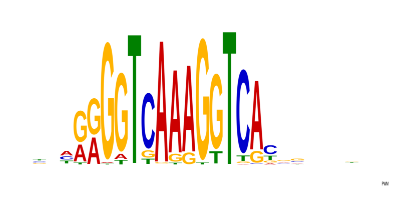	NNNNNNGTCANNNGTCACAGNNNNNN	247.96	DR:1	4
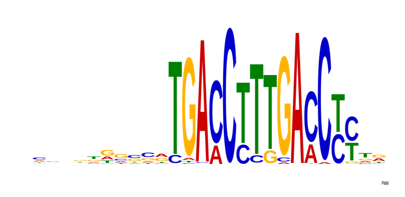	NNNNNNGCCATGANNNTTGANNNNNN	168.40	M	5
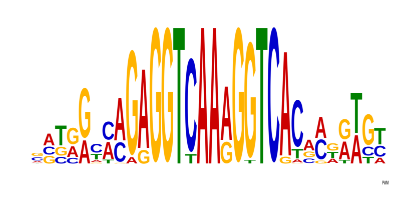	NNNNNNAAGAGGTCNNNNGTCACANNNNNN	1447.29	DR:1	6
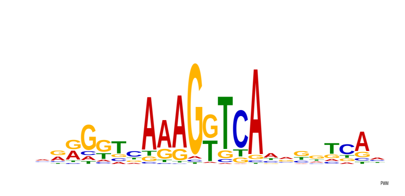	NNNNNNCAAANNNCAAANNNNNN	28.57	M	7
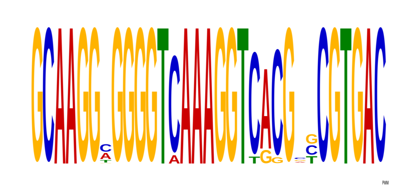	NNNNNNCGGGGTCNNNNGTCACGCTNNNNNN	2276.65	DR:1	8
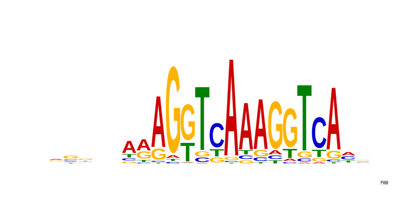	NNNNNNAAAGNNNNAAGGNNNNNN	34.89	M	9

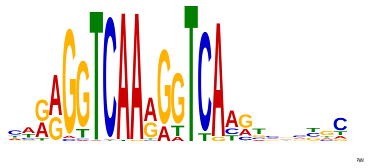	$NNN\overrightarrow{NNNTCA}ANNNNT\overrightarrow{CAAGTC}NNNNNN$	174.86	DR:6	10
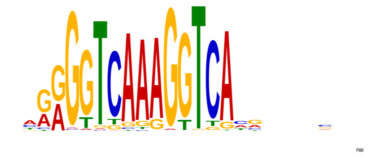	$NNN\overrightarrow{NNNTCA}ANNNNNCACGGNNNNNN$	48.28	M	11
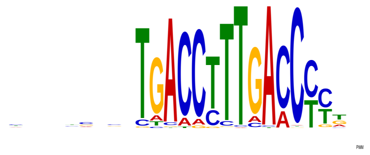	$NNNNNN\overrightarrow{CGTTGA}NNNNT\overleftarrow{TGANNN}NNNN$	79.43	IR:4	12
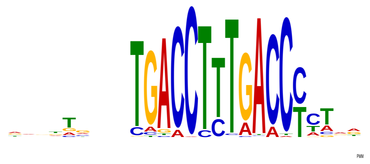	$NNNNNN\overrightarrow{CGATGA}NNNNN\overleftarrow{TGACNN}NNNN$	67.88	IR:4	13
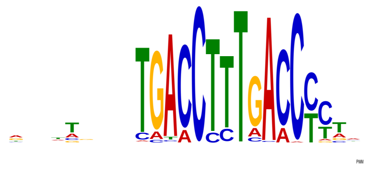	$NNNNNN\overrightarrow{CGATGA}NNNNT\overleftarrow{TGANNN}NNNN$	68.55	IR:4	14
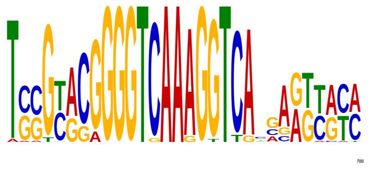	$NNNNNNCG\overrightarrow{GGGTCN}N\overrightarrow{NNGTCA}CCANNNNNN$	1353.10	DR:1	15
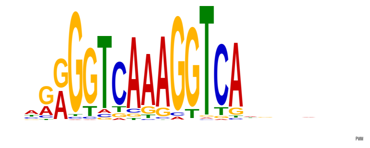	$NNN\overrightarrow{NNNTCA}ANNNNNCATCNNNNNN$	16.49	M	16

215 PPARD-Yin2017-Rnd3 (ERR1005723)

PWM	Seed Sequence	Seed Seq Enrichment	Repeat	Rank
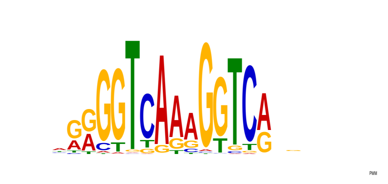	NNNNNNGTCANNNNTCATNNNNNN	67.16	DR:1	1
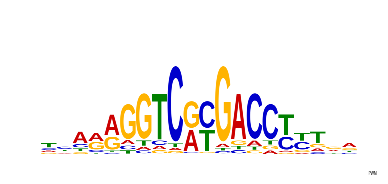	NNNNNNGGTCNNGACCNNNNNN	19.20	IR:0	2
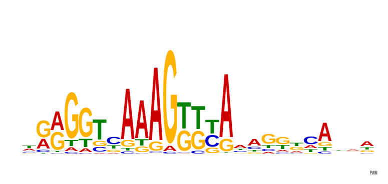	NNNNNNCAAANNNNAAAGTNNNNNN	23.96	M	3
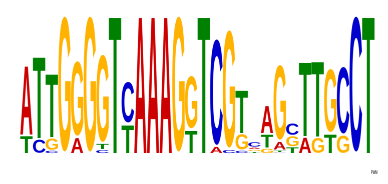	NNNNNNGTCANNNNNTCGTCAACNNNNNN	95.57	DR:4	4
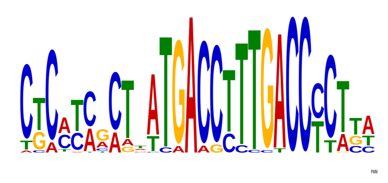	NNNNNNGCTTATGANNNNTGACNNNNNN	196.10	M	5
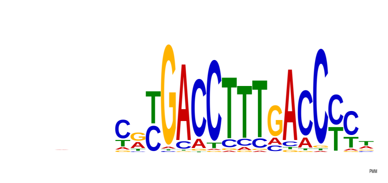	NNNNNNCGCGNNNTTTGNNNNNN	21.73	M	6
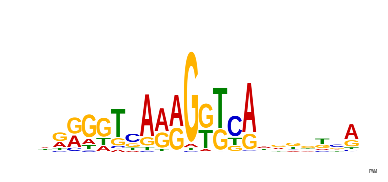	NNNNNNTCAANNNNCAAANNNNNN	28.88	M	7
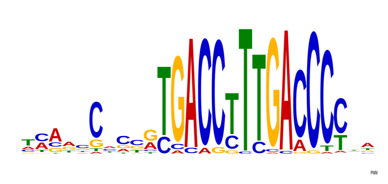	NNNNNNTCTGTGANNNTTGANNNNNN	92.32	IR:4	8
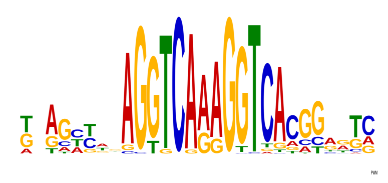	NNNNNNACAGGTCNNNNGTCACNNNNNN	156.70	DR:1	9

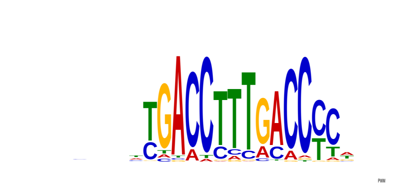	NNNNNNGCTGANNNNTGACNNNNNN	39.14	IR:4	10
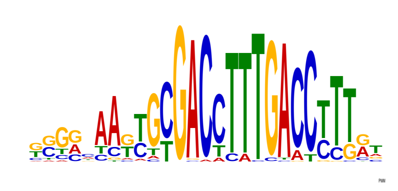	NNNNNNAGTGCGACNNNTGACNNNNNN	217.95	IR:5	11
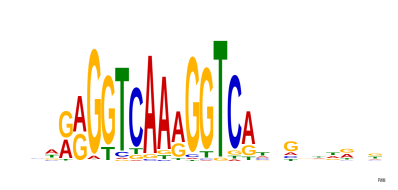	NNNNNNTCAANNNNCATTGNNNNNN	31.43	M	12
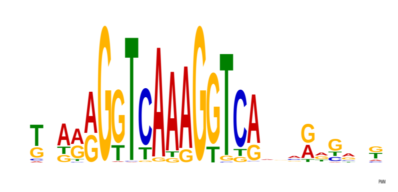	NNNNNNGTCANNNNTCAATCNNNNNN	44.61	DR:1	13
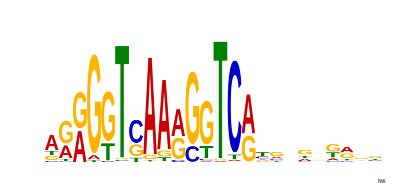	NNNNNNTCAANNNNTCATAANNNNNN	48.67	DR:1	14
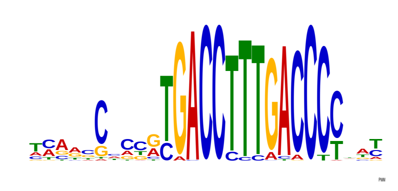	NNNNNNTCTGTGANNNNTGACNNNNNN	96.12	IR:4	15
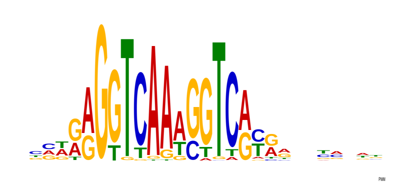	NNNNNNGTCANNNNTCGCGGCNNNNNN	78.43	M	16
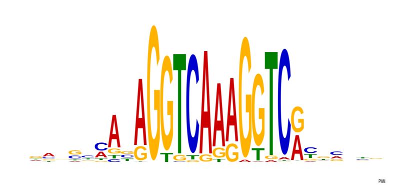	NNNNNNATAGGTCNNNNGTCGNNNNNN	107.89	DR:1	17
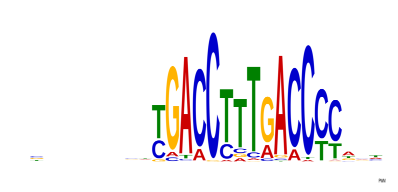	NNNNNNCGCTGANNNNTGACNNNNNN	43.96	IR:4	18

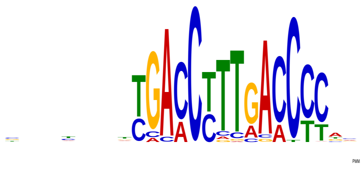	$NNNNNN\overrightarrow{CGCTGA}NNN\overleftarrow{TTGANNN}NNNN$	42.53	IR:4	19
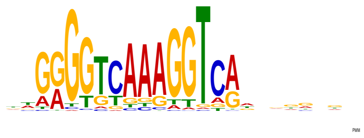	$NNN\overrightarrow{NNNTCA}ANNNNNCAGCNNNNNN$	11.47	M	20

216 PPARD-Yin2017-Rnd4 (ERR1005724)

PWM	Seed Sequence	Seed Seq Enrichment	Repeat	Rank
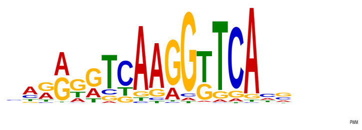	NNNNNNNTCAANNTTCANNNNNNN	19.76	DR:1	1
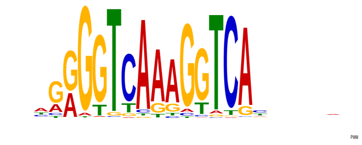	NNNNNNGTCANNNNNTCATNNNNNN	36.99	DR:1	2
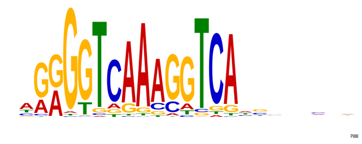	NNNNNNNTCAANNNNNCAATNNNNNN	14.85	M	3
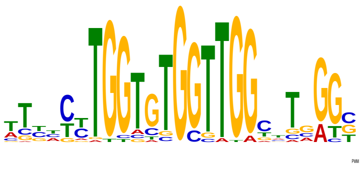	NNNNNNTGGTGTNNTTGGCNNNNNN	32.50	IR:3	4
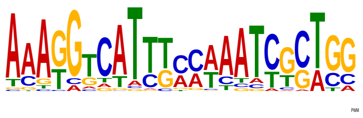	NNNNNNCATTNNAAAATNNNNNN	6.46	M	5
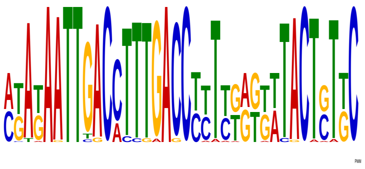	NNNNAATTGANNNNNNNNNNNNNNNNNTACTNNNN	28.50	M	6
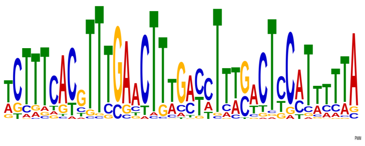	NNTTTCNNNNNNNNNNNNNNNNNNNNNNNNNNTTTTNN	4.78	M	7
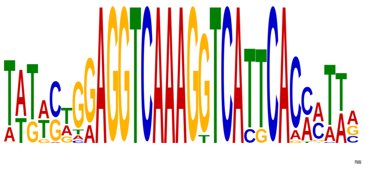	NNNNNNGGAGGTNNNNNNTCATTCANNNNNN	199.38	DR:5	8
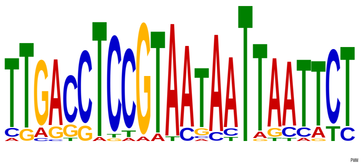	NNNNNNTCCGTNNTAATTNNNNNN	9.51	M	9

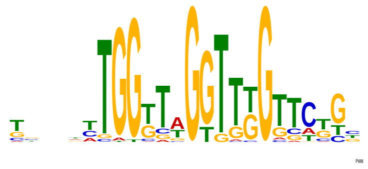	*NNNNNNTGGTNAGGTTGGNNNNNN*	25.29	M	10
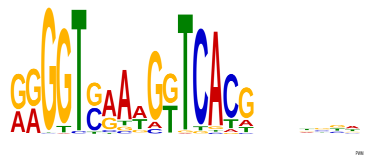	*NNNNNNAAAGTNNACGCNNNNNN*	10.60	M	11
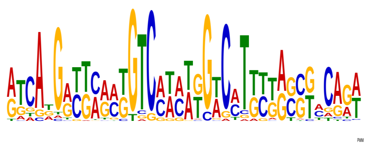	*NTCAGNNNNNNNNNNNNNNNNNNNNNNNNNNNNNACAGN*	3.01	M	12
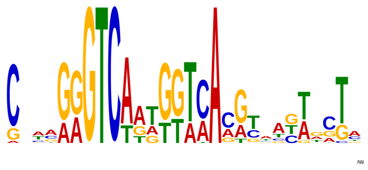	*NNNNNNGTCANNNNTCAGGTAGNNNNNN*	47.63	DR:6	13
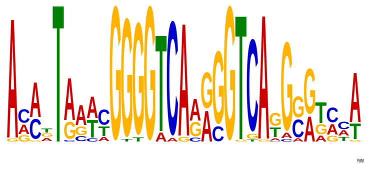	*NNNNNNATCGGGNNNNNNNNTCAGGNNNNNN*	31.97	M	14
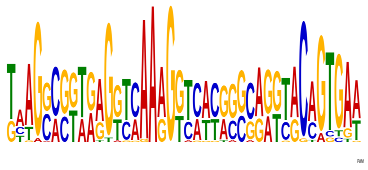	*NCAGCNNNNNNNNNNNNNNNNNNNNNNNNNNNNNNNNGTGAN*	4.43	M	15
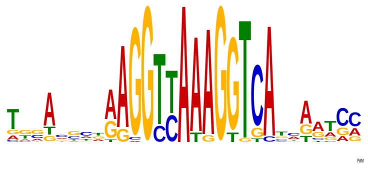	*NNNNNNCTAAGGNNNNNNNTCAGNNNNNN*	12.47	DR:1	16
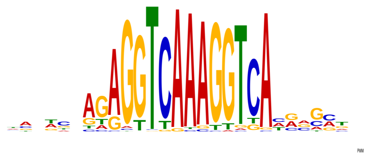	*NNNNNNATAGGTNNNNNNTCACNNNNNN*	21.02	DR:1	17
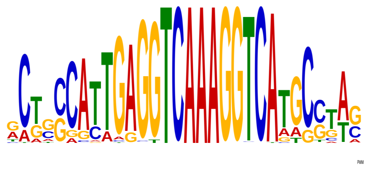	*NNNNNNATTGAGNNNNNNNNTCATNNNNNN*	94.81	ER:3	18

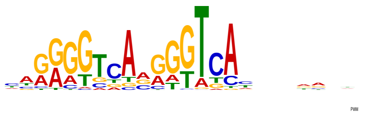	$NNN\overrightarrow{NNNTCA}ANNNNNCAGANNNNNNN$	6.56	M	19
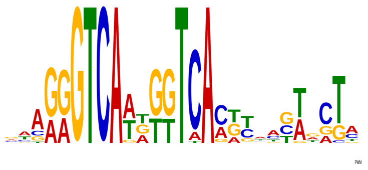	$NNN\overrightarrow{NNNTCA}ANNNNNC\overrightarrow{AGGTAG}NNNNNNN$	17.76	DR:6	20

217 PPARD-Yin2017-Rnd4 (ERR1005725)

PWM	Seed Sequence	Seed Seq Enrichment	Repeat	Rank
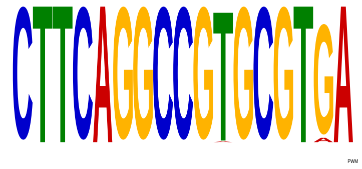	CTTCAGGNNNTGCGTGA	1850.22	DR:6	1
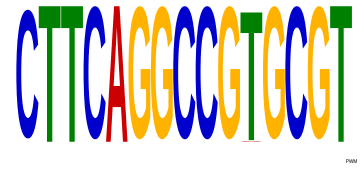	CTTCAGGNNNTGCGT	328.49	M	2
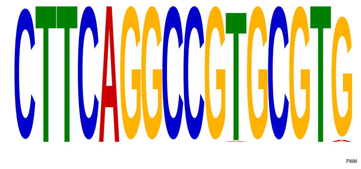	CTTCAGGNNNTGCGTG	724.65	M	3
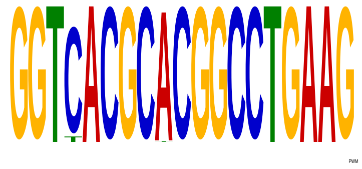	NGTCACGCANNNCCTGAAN	1067.89	IR:4	4
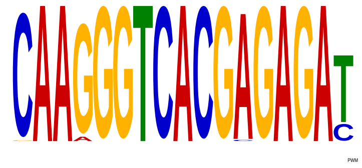	CAAGNNNNNCGAGAGAT	151.98	M	5
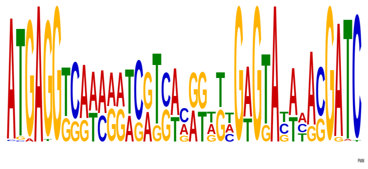	ATGAGNNNNNNNNNNNNNNNNNNNNNNNNNNNNNNGATC	47.63	ER:27	6
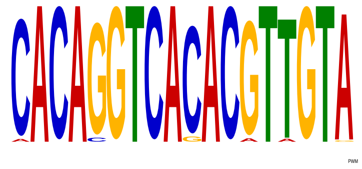	CACANNNNNNACGTTGTA	136.50	M	7
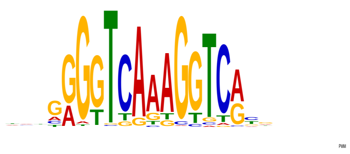	NNNNNNGGTCNNNNNTCACNNNNNN	26.96	DR:1	8
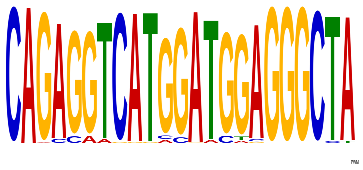	CAGANNNNNNNNNNNNAGGGCTA	67.95	M	9

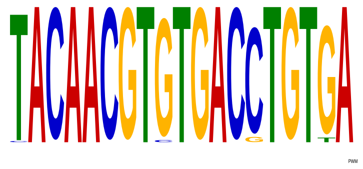	TACAACGNNNNNNNTGTGA	130.42	M	10
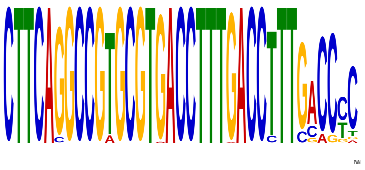	NNNNNGGCCGTGCNNNNNNNNNNNNNTTTGNNNNN	134.00	M	11
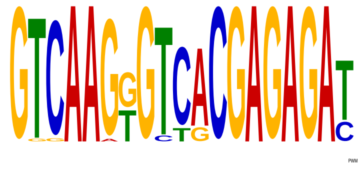	NTCAAGNNNNNCGAGAGAN	138.39	M	12
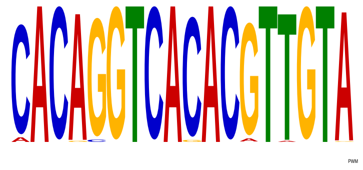	CACANNNNNNNCGTTGTA	64.01	M	13
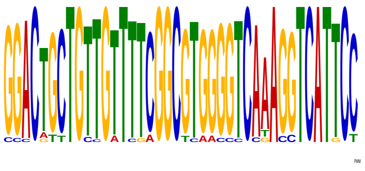	NNNCTGCTNNNNNNNNNNNNNNNNNNNNNNNNNTCATNNN	29.01	M	14
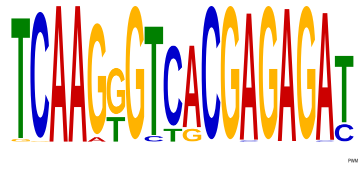	NCAAGNNNNNCGAGAGAN	76.20	M	15
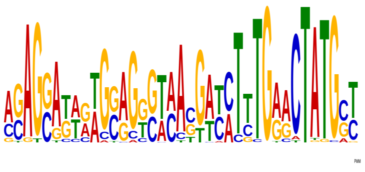	NNAGCANNNNNNNNNNNNNNNNNNNNNNNCTATGNN	14.12	M	16
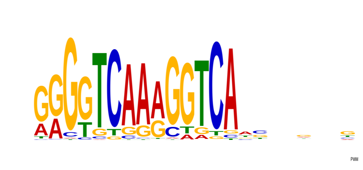	NNNNNNTCAANNNNCAACNNNNNN	21.74	M	17
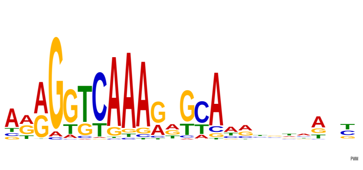	NNNNNNCAAANNNNAAATNNNNNN	12.00	M	18

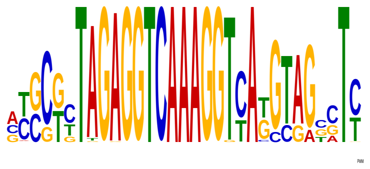	$NNNNNNTAGAGNNNNNNNNNTC\overrightarrow{AGGTN}NNNNN$	44.66	M	19
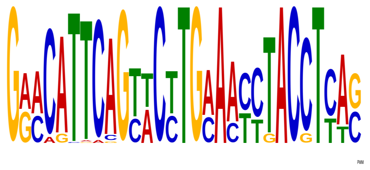	$NNN\overrightarrow{CATTCA}GNNNNNNNNNNN\overleftarrow{NNACCT}NNN$	31.62	IR:11	20

218 PPARD-13-Bhimsaria2022-Rnd2 (S07-027)

PWM	Seed Sequence	Seed Seq Enrichment	Repeat	Rank
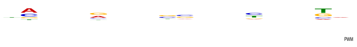	NNNNNNTGACGTCANNNNNN	1.04	M	1
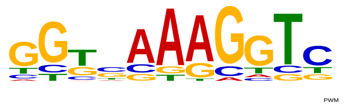	GGTCAAAGGTC	14.30	DR:1	2
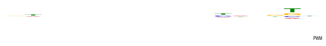	GAGTNNNNNNNNNNNNTGTT	2.09	M	3
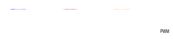	GGGGNNNNNNNNNNNNCGTG	1.89	M	4
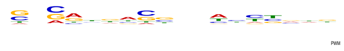	NNNNNTGACGTCACNNNNN	1.94	M	5
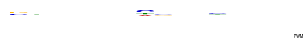	ATTGNNNNNNNNNNNCTGT	1.70	M	6
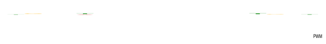	ACTGNNNNNNNNNNNNTGGT	1.54	M	7
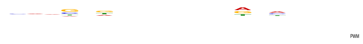	CAAGNNNNNNNNNNNNTTTG	1.80	M	8
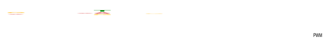	CAGGNNNNNNNNNNNNGTCG	1.59	DR:7	9

	NNGGGGNNNNNNNNGGCGNN	1.55	M	10
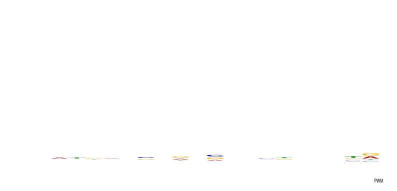	*GATGNNNNNNNNNNNNNATTG*	2.04	M	11
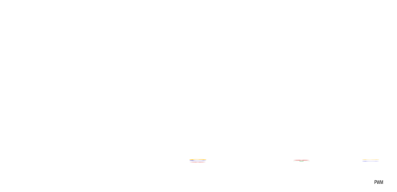	$\overrightarrow{AGGCNN}NNNNNNNNNNNTGTG$	1.49	M	12
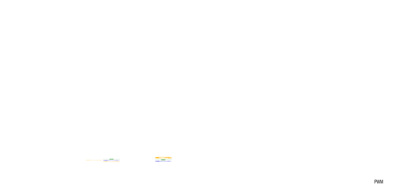	$\overrightarrow{GTCG}NNNNNNNNNNNN\overrightarrow{NGGTG}$	1.52	DR:11	13
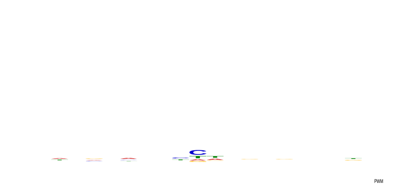	$N\overrightarrow{AAGTNN}NNNNNNNNNCACTN$	1.53	M	14
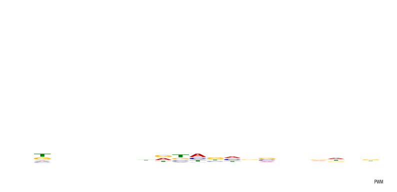	$\overrightarrow{NTCA}ANNNNNNNNNNNCAATN$	1.28	M	15
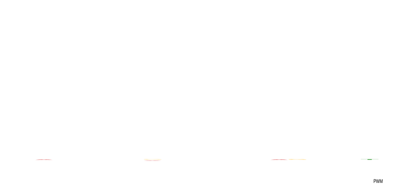	$\overrightarrow{AGTTNN}NNNNNNN\overrightarrow{NNNTCA}T$	1.62	DR:6	16
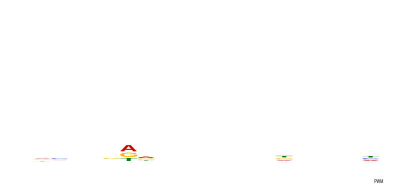	$NCAAGNNNNNNNNNNN\overrightarrow{ACGTN}$	1.58	M	17
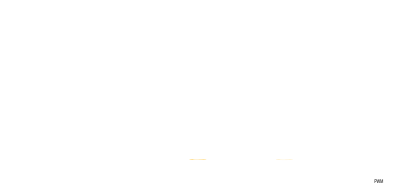	$\overrightarrow{AGGGNN}NNNNNNNNNNNGCGT$	1.74	M	18

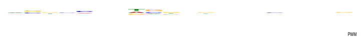	$\overrightarrow{AGGCNN}NNNNNNNNNNCGCG$	1.72	M	19
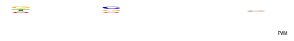	$NNNNNGTG\overrightarrow{ANGTCA}NNNNN$	1.22	M	20

219 PPARD-13-Bhimsaria2022-Rnd3 (S07-147)

PWM	Seed Sequence	Seed Seq Enrichment	Repeat	Rank
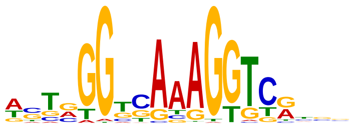 PWM	*NNNNGGTCAAAGGTCNNNN*	138.99	DR:1	1
PWM	*GGGGNNNNNNNNNNNNGGGG*	5.43	M	2
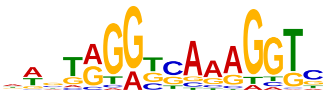 PWM	*NAAGTNNNNCAAAGGTN*	28.44	DR:5	3
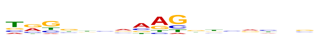 PWM	*NNNNGTCANNNGTCANNNN*	4.63	DR:1	4
PWM	*NNNNNNTGACGTCANNNNNN*	2.59	M	5
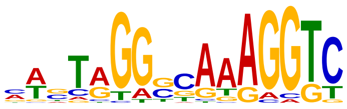 PWM	*NACTAGNNCAAAGGTN*	105.10	DR:1	6
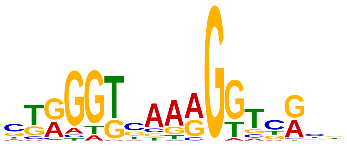 PWM	*NNAGGTGAAANGTCANN*	44.97	DR:1	7
PWM	*ATGTNNNNNNNNNNNNCTGT*	3.88	M	8
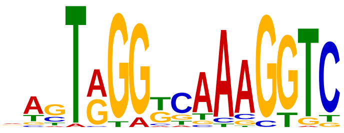 PWM	*NAGTGNNGCAAAGGTN*	51.75	M	9

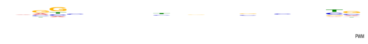	$\overrightarrow{AGGCNN}NNNNNNNNNNTCTG$	4.02	M	10

220 PPARD-14-Bhimsaria2022-Rnd3 (S07-095)

PWM	Seed Sequence	Seed Seq Enrichment	Repeat	Rank
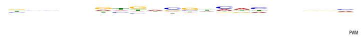	NNNNNNTGACGTCANNNNNN	4.07	M	1
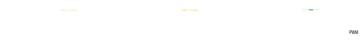	CATGNNNNNNNNNNNNTTGT	4.18	M	2
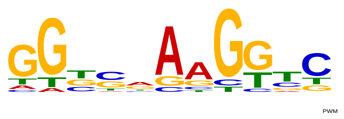	GGTCAAAGGTC	25.84	DR:1	3
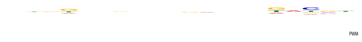	ATGGNNNNNNNNNNNNACGT	4.60	M	4
	GGGGNNNNNNNNNNNNGGGG	4.09	M	5
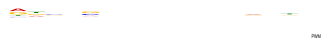	ATATNNNNNNNNNNNNAATT	3.76	M	6
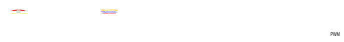	CAATNNNNNNNNNNNNTTTT	3.73	M	7
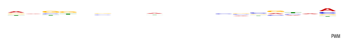	AAAGNNNNNNNNNNNNGTAA	3.87	M	8
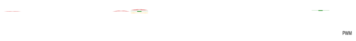	AGTTNNNNNNNNNNNNCATT	3.89	M	9

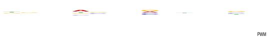	$ACAGNNNNNNNNNN\overrightarrow{NNGTGA}$	3.55	M	10
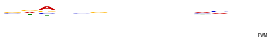	$CATGNNNNNNNNNNNN\overrightarrow{ACGT}$	4.25	M	11
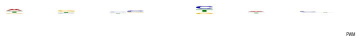	$\overrightarrow{AGGGNN}NNNNNNNNNNTCTT$	3.95	M	12
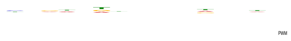	$GAAGNNNNNNNNNNNNCATG$	3.97	M	13
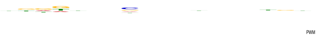	$AGTGNNNNNNNNNNNNTTGT$	4.09	M	14
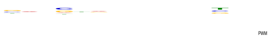	$GAAGNNNNNNNNNNNNCTGT$	4.04	M	15

221 PPARG-Bhimsaria2022-Rnd2 (S07-030)

PWM	Seed Sequence	Seed Seq Enrichment	Repeat	Rank
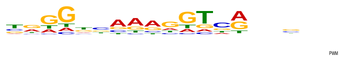	NNNNNNCAAAGGTCNNNNNN	4.06	M	1
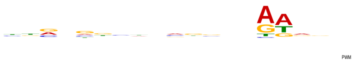	NNNNNCTAAAGGTCNNNNN	1.48	M	2
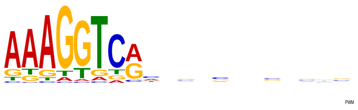	AAAGGTCNNNNNNNNNGGGG	22.27	M	3
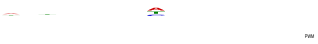	AGATNNNNNNNNNNNAATT	1.86	M	4
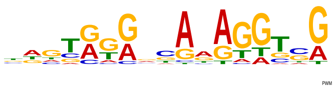	NNAGTANNNCAAAGGTNN	11.67	DR:1	5
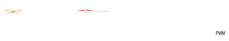	NNATGTNNNNNNNAGGTNN	1.59	DR:5	6
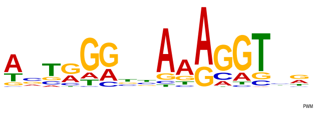	NNACTGGNNNAAAGGTNN	14.51	M	7
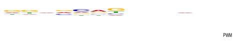	NNNNNNAGGAACAGNNNNNN	1.93	M	8
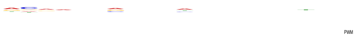	ACAANNNNNNNNNNNNGTTT	2.02	M	9

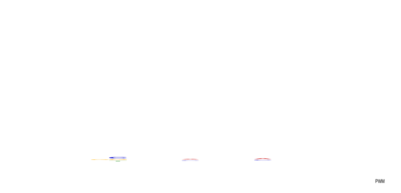	ATTGNNNNNNNNNNNNTACT	1.81	M	10
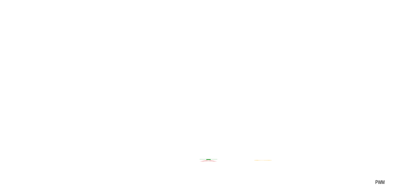	$\overrightarrow{AGTCN}$NNNNNNNNNNN$\overrightarrow{AAGT}$	1.81	DR:10	11
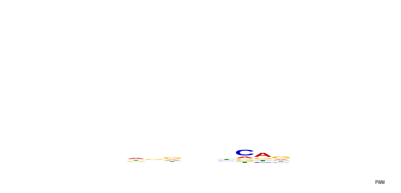	NNNNN$\overrightarrow{AGGANT}$CAGNNNNN	2.35	M	12
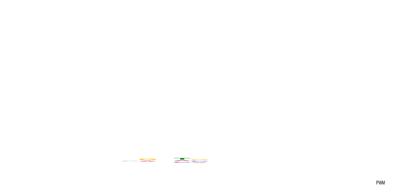	CAGANNNNNNNNNNNNTAGT	1.76	M	13
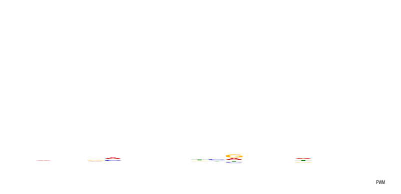	ATTGNNNNNNNNNNN$\overrightarrow{NGGTA}$	1.48	M	14
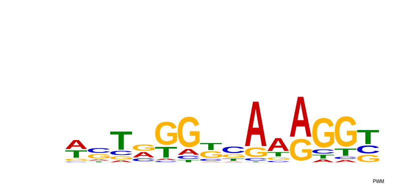	NACT$\overrightarrow{GGGNCA}$$\overrightarrow{AAGGN}$	31.39	DR:1	15
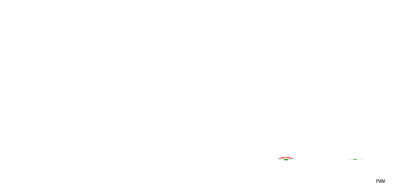	GAGTNNNNNNNNNNNNTGTT	1.61	M	16
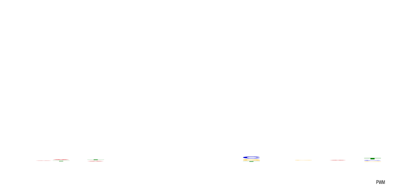	$\overrightarrow{AAGTNN}$NNNNNNNNNNGATT	1.98	M	17
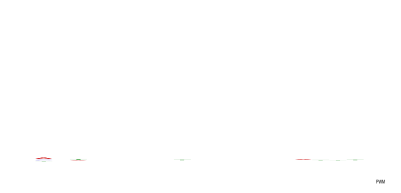	AATGNNNNNNNNNNNNTTTT	2.02	M	18

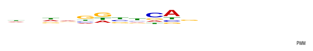	$NNNNNN\overrightarrow{AGGTTC}AGNNNNNNN$	2.40	M	19
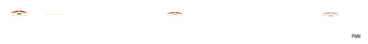	$NNNNNCTAA\overrightarrow{NNGTCA}NNNNN$	1.36	M	20

222 PPARG-Bhimsaria2022-Rnd3 (S07-150)

PWM	Seed Sequence	Seed Seq Enrichment	Repeat	Rank
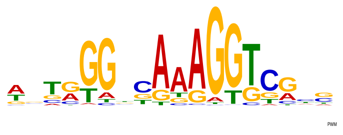	NNNTGGGNCAAAGGTCNNN	791.22	DR:1	1
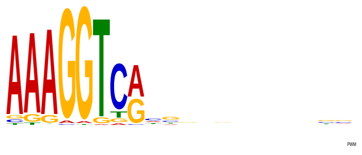	AAAGGTCNNNNNNNNNGGGG	143.74	M	2
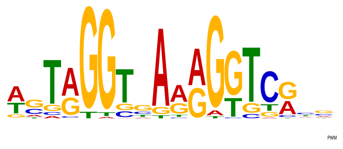	NNNTAGGTGAGNGGTCNNN	204.07	DR:1	3
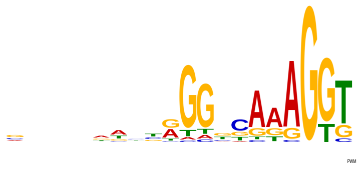	GGGGNNNNNNNNNCAAAGGT	130.06	M	4
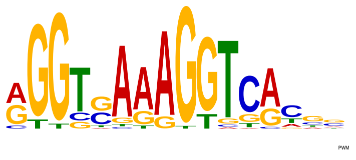	NGGTGAAAGGTCACGN	1429.16	DR:1	5
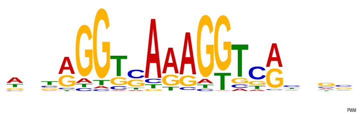	NNNNGGTGAANNGTCANNNN	48.69	DR:1	6

223 PPARG-14-Bhimsaria2022-Rnd3 (S07-096)

PWM	Seed Sequence	Seed Seq Enrichment	Repeat	Rank
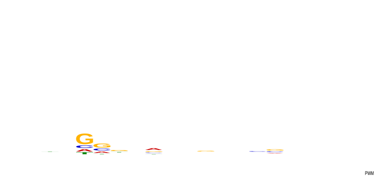	NNNNNNCAAAGGTCNNNNNN	3.87	M	1
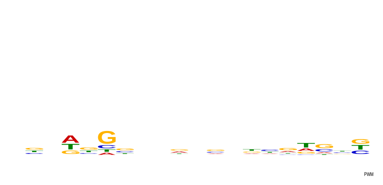	NNNNNCTAAAGGTCNNNNN	3.28	M	2
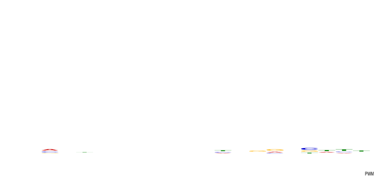	AAATNNNNNNNNNNNNCTTT	4.91	M	3
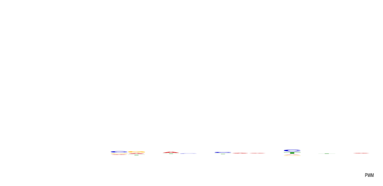	AAAANNNNNNNNNNNNGTGA	3.49	M	4
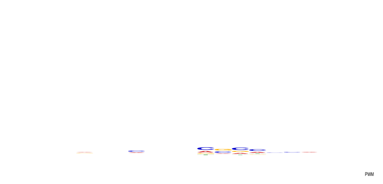	GAGGNNNNNNNNNNNNACGT	3.81	DR:9	5
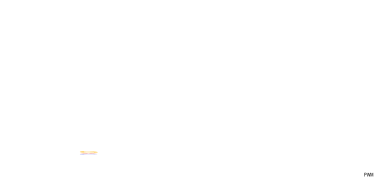	AGAGNNNNNNNNNNNNCTTT	4.90	M	6
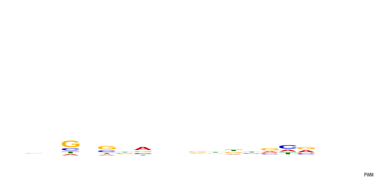	NNNNNCAAAGGTCANNNNNN	3.80	M	7
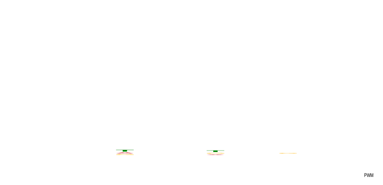	AGTTNNNNNNNNNNNNTTTT	3.96	M	8
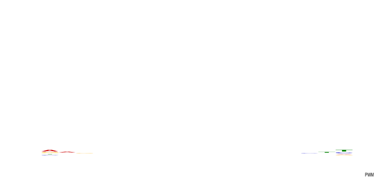	NAAGTNNNNNNNNNNACTTN	5.23	IR:6	9

224 PPARG-15-Bhimsaria2022-Rnd3 (S07-149)

PWM	Seed Sequence	Seed Seq Enrichment	Repeat	Rank
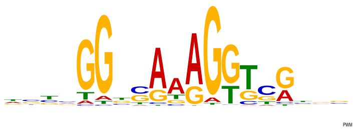	NNNNGGTCAAAGGTCNNNN	283.75	DR:1	1
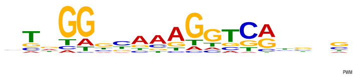	NNNNNCTAAAGGTCNNNN	9.18	M	2
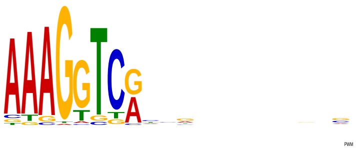	AAAGGTCNNNNNNNNNGGCG	212.03	M	3
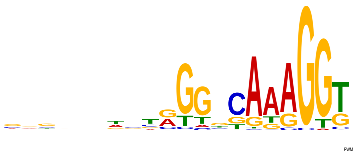	GGGGNNNNNNNNNCAAAGGT	209.95	M	4
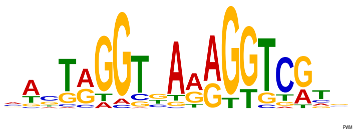	NNNTGGGTGAGNGGTCNNN	183.88	DR:1	5
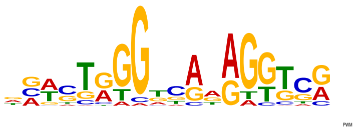	NNAGTGGGTCNNNGGTCNN	166.74	DR:1	6
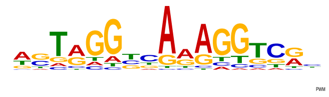	NNAGTAGNNNNNNGGTCNN	20.88	M	7
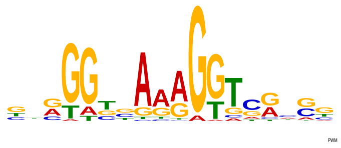	NNNGGGTGAAANGTCANNN	183.53	DR:1	8
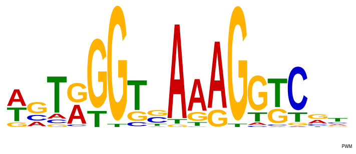	NNTGGGTGAAAGGGCNN	356.86	DR:1	9

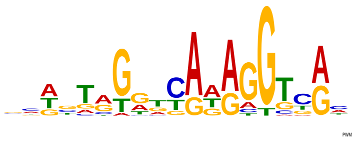	$NNAGT\overrightarrow{GGGGCN}N\overrightarrow{NGGTCN}N$	102.56	DR:1	10

225 Rev-ErbA-Alpha-Yin2017-Rnd2 (ERR1005156)

PWM	Seed Sequence	Seed Seq Enrichment	Repeat	Rank
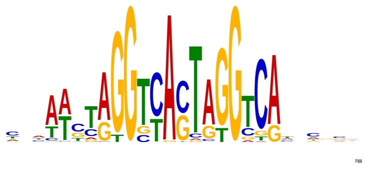	NNNNNNTAGGTCANTAGGTCANNNNNN	7862.27	DR:2	1
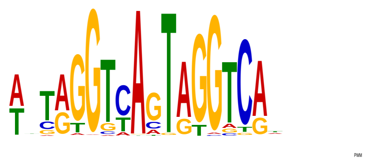	NNNNNNNTCAGTAGGTCANNNNNN	484.14	DR:2	2
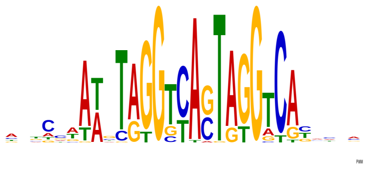	NNNNNNATGTANNNNAGTAGGTCNNNNNN	1509.80	DR:6	3
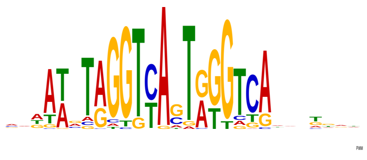	NNNNNNTAGGTCANNNNNTCAANNNNNN	203.26	DR:2	4
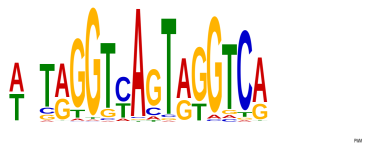	NNNNNNNTCAGTNNGTCANNNNNN	75.51	DR:2	5
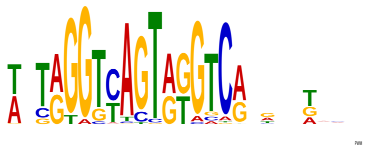	NNNNNNTCAGTNNGTCACGNNNNNN	100.38	DR:2	6
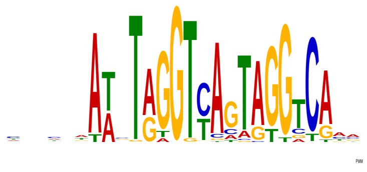	NNNNNNATGTNNNTTAGTAGNNNNNN	87.08	M	7
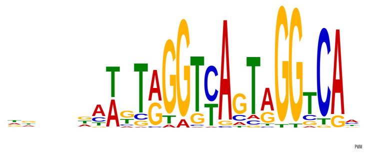	NNNNNNAAGTNNNTCAGTANNNNNN	47.47	M	8
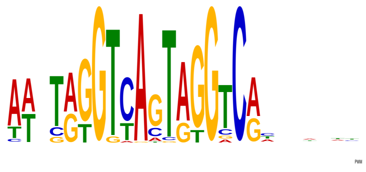	NNNNNNGTCANTAGGTCGCNNNNNN	266.02	DR:2	9

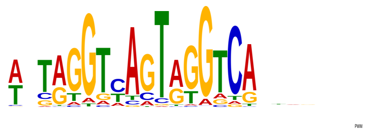	$NNN\overrightarrow{NNNTCA}GT\overrightarrow{NNNTCA}GNNNNNN$	27.59	DR:2	10
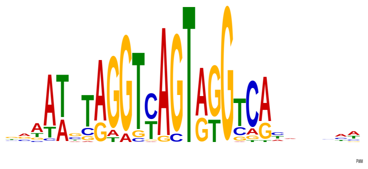	$NNNNNNT\overrightarrow{AGGTCA}NNNNNNTCGTNNNNNN$	71.24	M	11

226 Rev-ErbA-Alpha-Yin2017-Rnd2 (ERR1005157)

PWM	Seed Sequence	Seed Seq Enrichment	Repeat	Rank
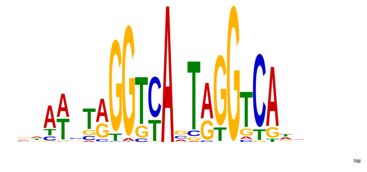	NNNNNNTAGGTCANTAGGTCANNNNNN	3034.95	DR:2	1
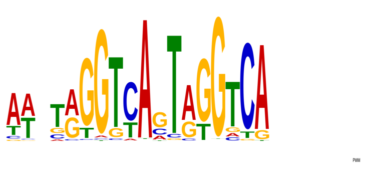	NNNNNNGTCAGTNGGTCANNNNNN	187.22	DR:2	2
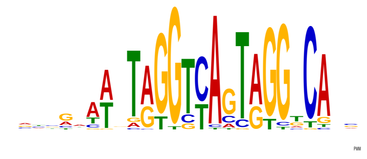	NNNNNNAAGTAGGTNAGTAGNNNNNN	257.35	M	3
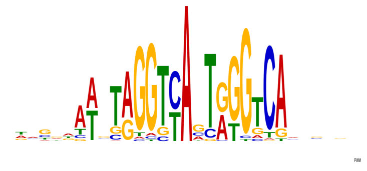	NNNNNNAAGTAGGTNNNNNNGTCANNNNNN	132.58	DR:2	4
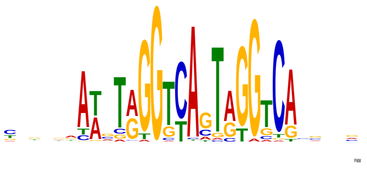	NNNNNNATGTNNNNNAGTAGGTCNNNNNN	127.89	DR:6	5
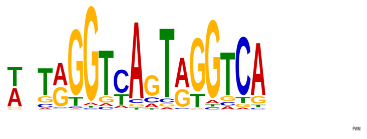	NNNNNNTCAGTNNGTCANNNNNN	22.67	DR:2	6
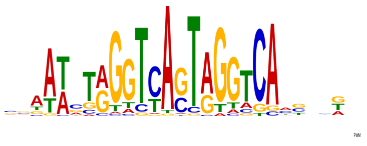	NNNNNNCAGGTCANNNNGTCANNNNNN	24.22	DR:2	7
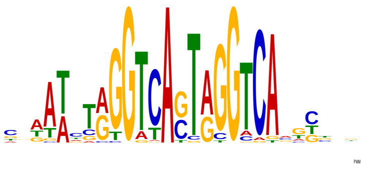	NNNNNNCGGGTCANNNGGTCANNNNNN	70.87	DR:2	8
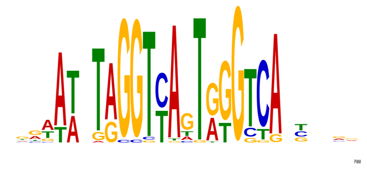	NNNNNNATAGGTTANNNNGTCANNNNNN	112.26	DR:2	9

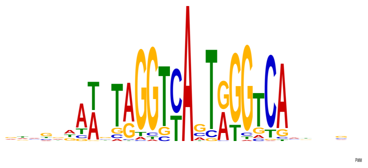	$NNNNNN\overrightarrow{AAGTAG}GNNNNNN\overrightarrow{NNGTCA}NNNNNNN$	45.99	DR:6	10
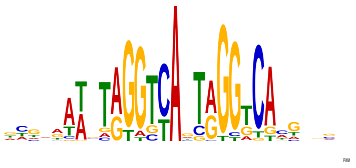	$NNNNNN\overrightarrow{AAGTAG}NNNNNN\overrightarrow{AGGTCA}NNNNNNN$	82.15	DR:6	11
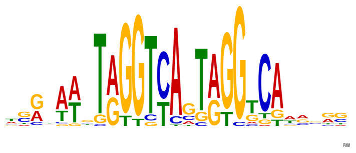	$NNNNNNAGTANNNNAGT\overrightarrow{AGGTCN}NNNNNN$	90.48	M	12

227 Rev-ErbA-Alpha-Yin2017-Rnd3 (ERR1005158)

PWM	Seed Sequence	Seed Seq Enrichment	Repeat	Rank
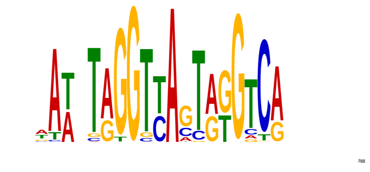	NNNNNNTAGGTTANNNNGTCANNNNNN	2665.72	DR:2	1
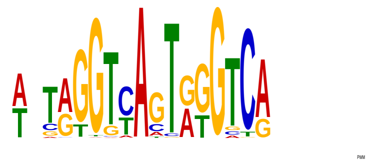	NNNNNNTCAGTNNGTCANNNNNN	832.03	DR:2	2
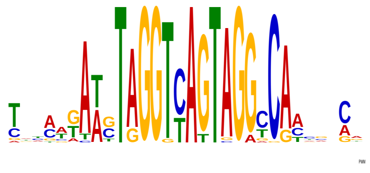	NNNNNNAAGTAGGTNNNTAGGCCANNNNNN	31799.78	DR:2	3
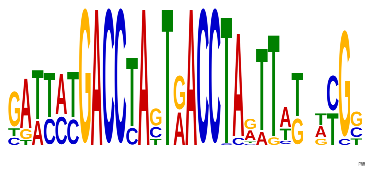	NNNNNNGACCTAGTNNCCTAGTTANNNNNN	100342.57	M	4
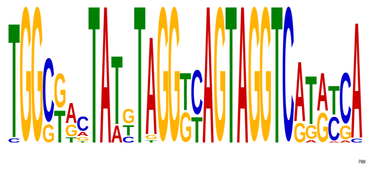	NNNNNACTATGTANNNNNNTAGGTCANNNNN	16036.71	DR:6	5
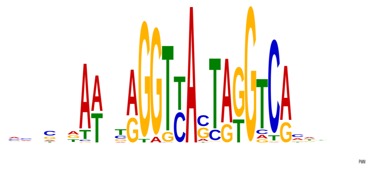	NNNNNNAATTAGGTNNNNNNGTCANNNNNN	2994.72	DR:2	6
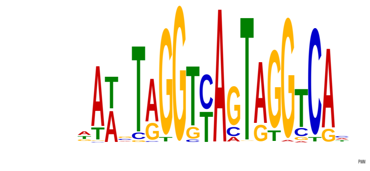	NNNNNNATGTANNNNAGTAGNNNNNN	608.49	M	7
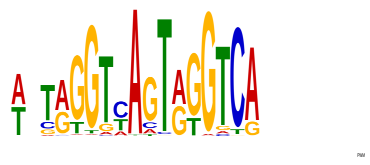	NNNNNNTCAGTNNNTCAGNNNNNN	361.24	DR:2	8
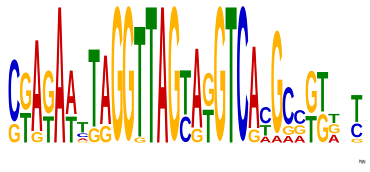	NNNNNNTTAGGTTANNNNGTCACGCNNNNNN	17503.43	DR:2	9

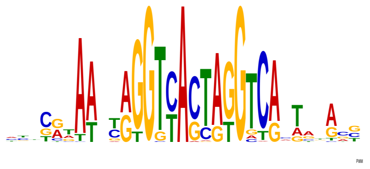	NNNNNNAATTAGGTNNNNNNNTCAANNNNNN	1728.18	DR:2	10
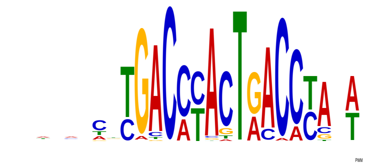	NNNNNNCGTGANNNACTGANNNNNN	634.73	IR:5	11
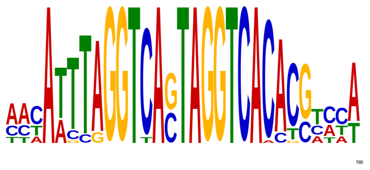	NNNNNTTAGGTCANNNNGTCACACNNNNN	18174.99	DR:2	12
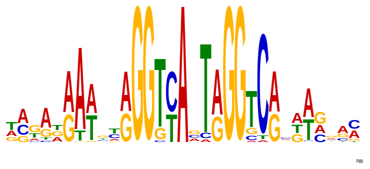	NNNNNNAATTAGGTNNNNNNNTCATNNNNNN	897.73	DR:2	13
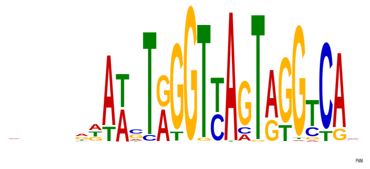	NNNNNNAATGTNNNTTAGTANNNNNN	1051.99	M	14
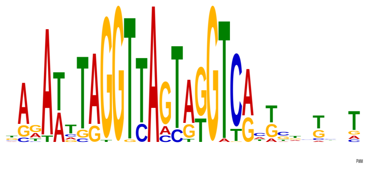	NNNNNNTAGGTTANNNNNNTCACGCNNNNNN	2736.40	DR:2	15
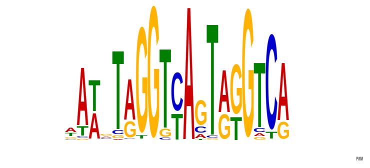	NNNNNNATGTANNNNNNNNNGTCANNNNNN	258.32	DR:6	16
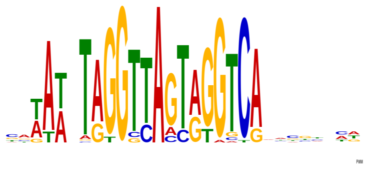	NNNNNNTAGGTTANNNNNNTCAGANNNNNN	1476.09	DR:2	17
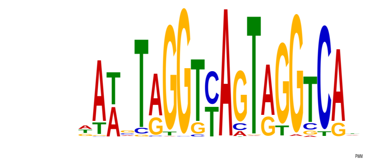	NNNNNNATGTANNNNAGTANNNNNN	275.39	M	18

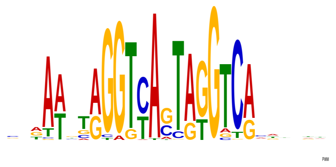	NNNNNNAATTAGNNNNNN$\overrightarrow{NNGTCA}$NNNNNN	399.09	M	19

228 Rev-ErbA-Alpha-Yin2017-Rnd3 (ERR1005159)

PWM	Seed Sequence	Seed Seq Enrichment	Repeat	Rank
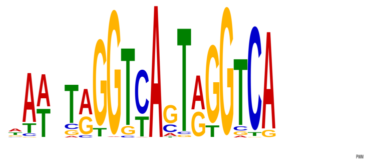	NNNNNNGGTCANNNGGTCANNNNNN	769.54	DR:2	1
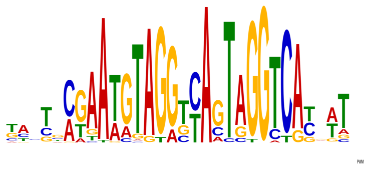	NNNNNNGAATGTAGNNNAGTAGGTCNNNNNN	43108.74	DR:6	2
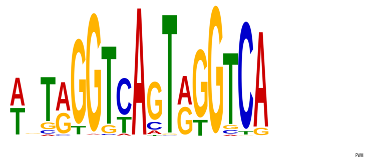	NNNNNNTCAGTNNGTCANNNNNN	279.26	DR:2	3
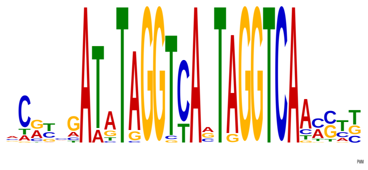	NNNNNNATATAGGNNAATAGGTCNNNNNN	14901.96	DR:2	4
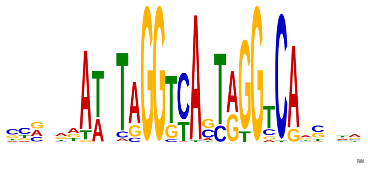	NNNNNNATGTAGGNNACTAGGTCNNNNNN	15391.97	DR:2	5
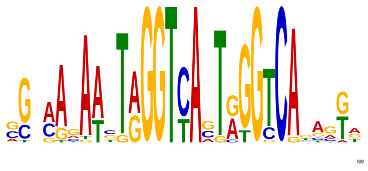	NNNNNNAACTGGGTNACTAGGTCNNNNNN	28176.03	M	6
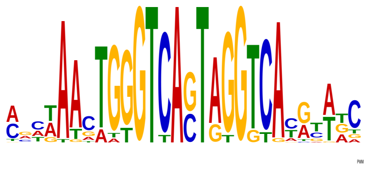	NNNNAACTGGGTNNNNAGGTCACGNNNN	14900.96	M	7
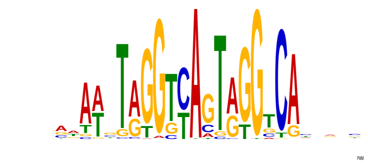	NNNNNNAAGTANNNNAGTAGGTCNNNNNN	2824.16	DR:6	8
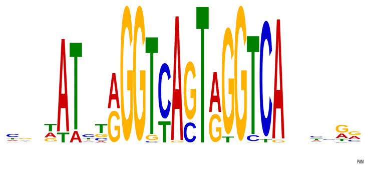	NNNNNNCCAGGTCANNNGGTCANNNNNN	916.76	DR:2	9

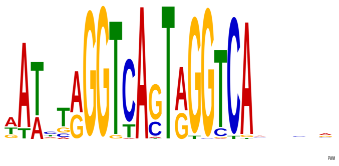	$NNNNNNC\overrightarrow{AGGTCN}NNN\overrightarrow{NGGTCA}NNNNNN$	205.25	DR:2	10
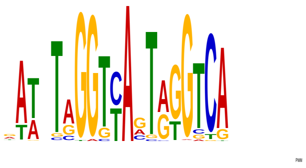	$NNNNNNATAT\overrightarrow{AGGTNN}NNNN\overrightarrow{NNGTCA}NNNNNN$	1479.94	DR:2	11
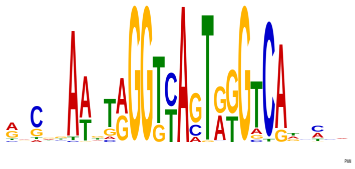	$NNNNNNAATT\overrightarrow{AGNNNA}GT\overrightarrow{GGGTCN}NNNNN$	4423.69	DR:2	12
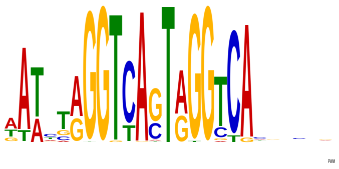	$NNNNNNC\overrightarrow{AGGTCN}NN\overrightarrow{AGGTCA}NNNNNN$	418.02	DR:2	13
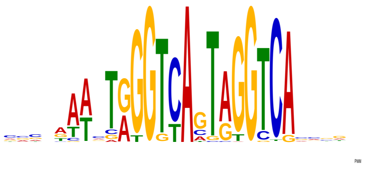	$NNNNNNACT\overrightarrow{GGGTCN}NN\overrightarrow{AGGTCN}NNNNN$	2935.18	DR:2	14
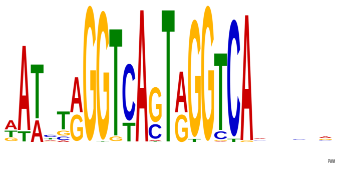	$NNNNNNC\overrightarrow{AGGTCA}NN\overrightarrow{NGGTCA}NNNNNN$	541.26	DR:2	15
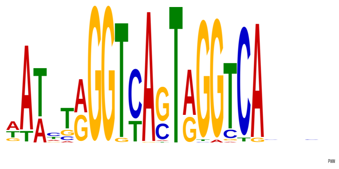	$NNNNNNC\overrightarrow{AGGTCA}NN\overrightarrow{AGGTCN}NNNNN$	404.92	DR:2	16
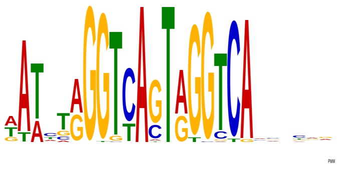	$NNNNNNC\overrightarrow{AGGTCA}GN\overrightarrow{NNGTCA}NNNNNN$	422.39	DR:2	17
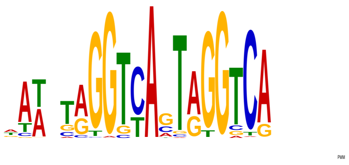	$NNNNNN\overrightarrow{AGGTCN}NN\overrightarrow{AGGTCN}NNNNN$	445.24	DR:2	18

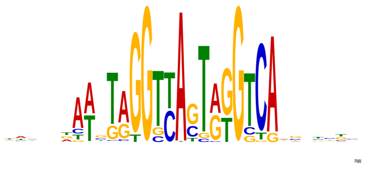	$NNNNNNAAGT\overrightarrow{AGGTNN}NN\overrightarrow{NNGTCA}ANNNNNNN$	1893.74	DR:2	19
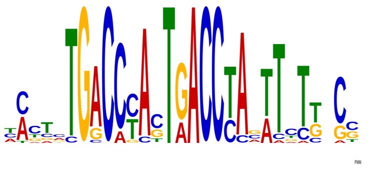	$NNNNN\overleftarrow{NGACCT}AG\overleftarrow{TNNCCC}\overrightarrow{AGTTN}NNNNN$	7924.52	M	20

229 Rev-ErbA-Alpha-Yin2017-Rnd4 (ERR1005160)

PWM	Seed Sequence	Seed Seq Enrichment	Repeat	Rank
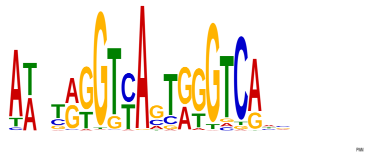	NNNNNNGTCANNNNNTCAANNNNNN	62.60	DR:2	1
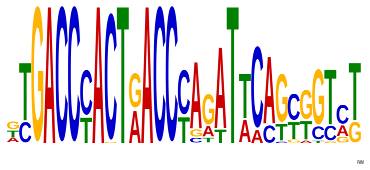	NNNNNNCACTNNNNNAGATTCAGNNNNNN	493.94	M	2
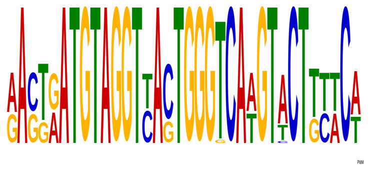	NNNNNNTGTANNNNNNNNNNNTCAAGTACNNNNNN	845.94	DR:0	3
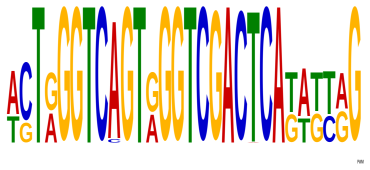	NNNNNNTCAGNNNNTCGACTCANNNNNN	482.90	DR:7	4
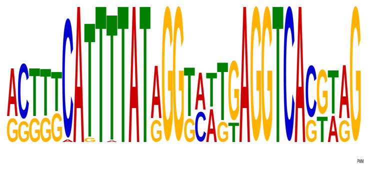	NNNNNCATTTTATNNNNNNNNNNGTCANNNNN	287.89	DR:9	5
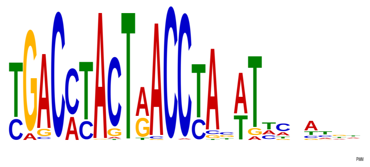	NNNNNNACTANNNNAGATTNNNNNN	82.24	M	6
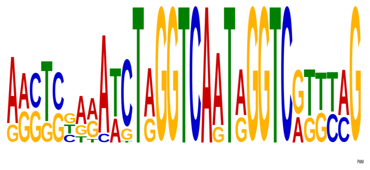	NNNNNTAAAACTNNNNNNNNNNGTCGNNNNN	95.56	ER:8	7
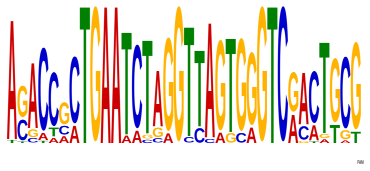	NNNNNNCTGAATCTNNNNNNNNNNGTCGNNNNNN	589.16	ER:9	8
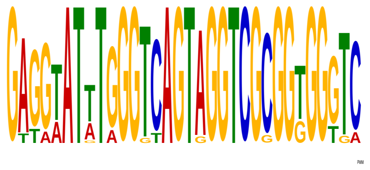	NNNNTATATNNNNNNNNNNNTCGCGGTGNNNN	269.47	M	9

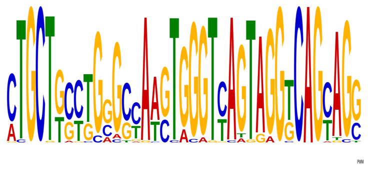	NTGCTNNNNNNNNNNNNNNNNNNNNNNNNNCAGCAGN	46.16	M	10
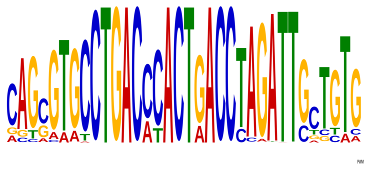	NNNNNNGCCTGANNNNNNNNNNNNAGATTNNNNNN	1095.23	ER:13	11
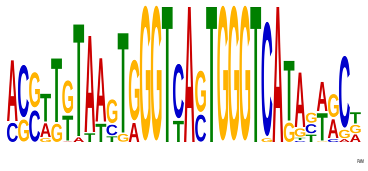	NNNNNTTAAGTNNNNNNNNNNNNTCAGANNNNN	84.23	ER:8	12
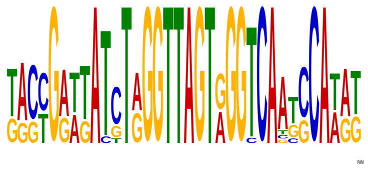	NNNNNNTGATCTNNNNNNNNNNNNTCAAGNNNNNN	115.61	ER:8	13
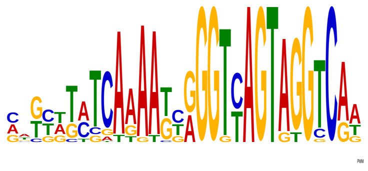	NNNNNNNATCAAAANNNNNNNAGTANNNNNN	237.43	M	14
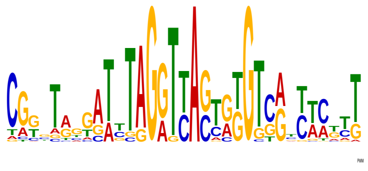	NNNNNNAAAAGTNNNNNNNNNNNTCAANNNNNNN	27.21	DR:6	15
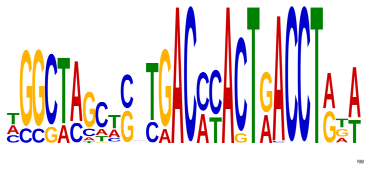	NNNNNNGCTCCTGANNNNCTAANNNNNNN	118.41	IR:0	16
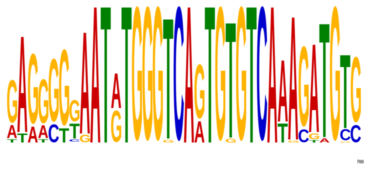	NNNNNNTAATGTGNNNNNNNNNNNTCAAANNNNNNN	156.68	ER:8	17
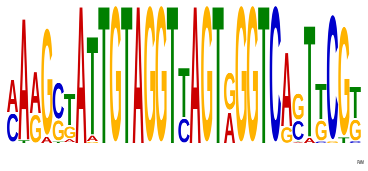	NNNNNTATTGTAGNNNNNNNNNNTCACNNNNNN	189.85	M	18

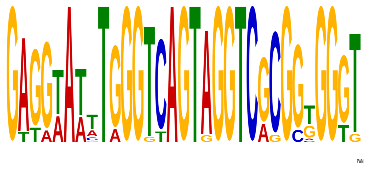	$NNNNTATATNNNNNNNNNNNTCG\overrightarrow{CGGTN}NNN$	124.70	M	19
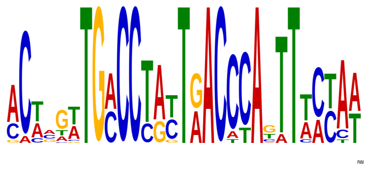	$NNNNTTTGNNNNNNNNNNCC\overrightarrow{AGTTTN}NNN$	69.71	M	20

230 Rev-ErbA-Alpha-Yin2017-Rnd4 (ERR1005161)

PWM	Seed Sequence	Seed Seq Enrichment	Repeat	Rank
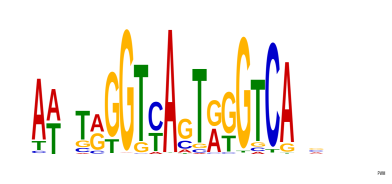	NNNNNNGTCANNNNGTCANNNNNN	134.98	DR:2	1
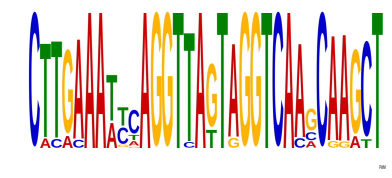	NNNNNNAATCAGGTNNNNNNGTCAAANNNNNN	2351.88	DR:2	2
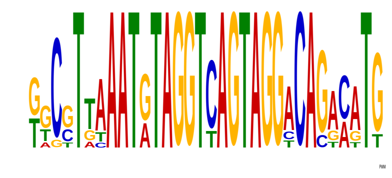	NNNNNNAAATGTAGNNNNNTAGGACANNNNNN	4148.31	DR:6	3
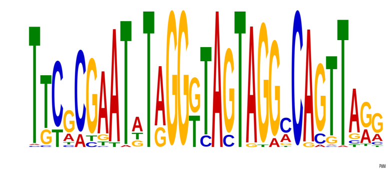	NNNNNNAATATAGGNNNNTAGGCCANNNNNN	7223.01	DR:2	4
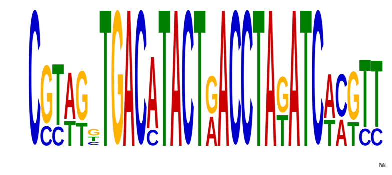	NNNNNGTGACATANNNNNCTAGATCNNNNN	3682.41	ER:8	5
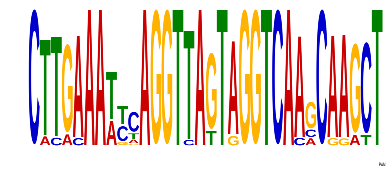	NNNNNNAATCAGGTNNNNNNNTCAAANNNNNN	1130.18	DR:2	6
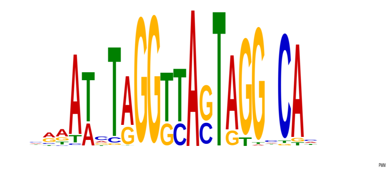	NNNNNNTAGGNNNNTAGGCCANNNNNN	347.39	DR:2	7
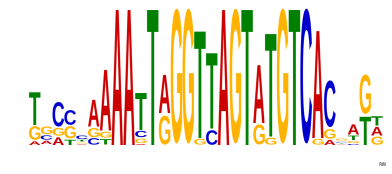	NNNNNNAAATTANNNNAGTATGTCNNNNNN	4447.25	DR:7	8
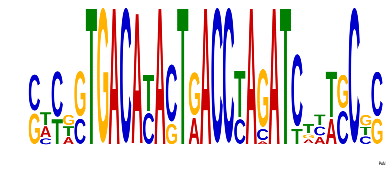	NNNNNNGACATACTNNNNNAGATCTNNNNNN	2138.30	ER:8	9

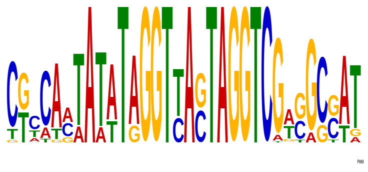	NNNNNNTATATAGNNNNNNAGGTCGANNNNNN	2462.72	M	10
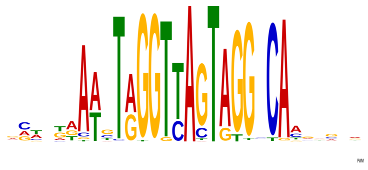	NNNNNNAAGTNNNNNNNTAGGCCANNNNNN	270.30	DR:6	11
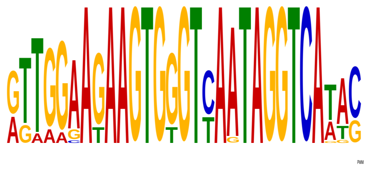	NNNNNAAGAAGTGNNNNAATAGGTNNNNN	1432.25	ER:8	12
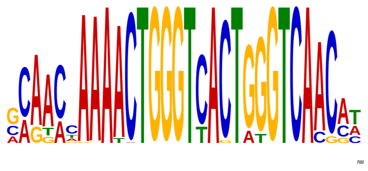	NNNNNNAAAACTGGNNNACTGGGTNNNNNN	7001.40	ER:8	13
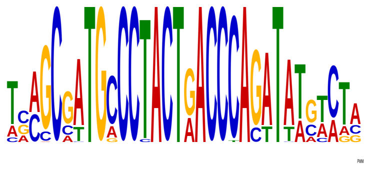	NNNNNNATGCCCTANNNNNCCAGATANNNNNN	4359.67	ER:8	14
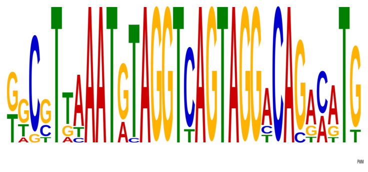	NNNNNNAAATGTANNNNNNTAGGACANNNNNN	1836.50	DR:6	15
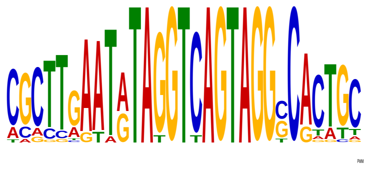	NNNNNAAATATANNNNNGTAGGCCNNNNN	861.16	M	16
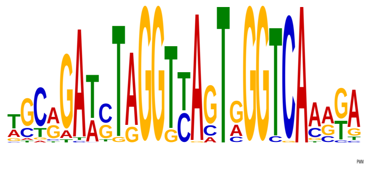	NNNNNAAGTANNNNAGTCGGTCNNNNN	265.71	DR:6	17
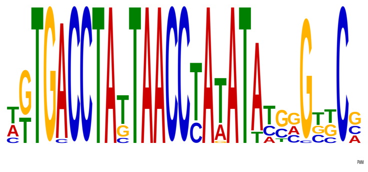	NNNNNNCTATTAACNNNNATATCNNNNNN	368.60	ER:3	18

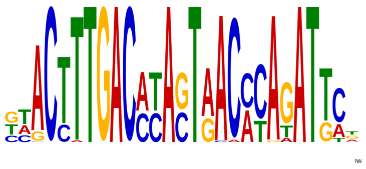	$\overleftarrow{NNACTT}\overleftarrow{TGACNN}NNNNNNNN\overrightarrow{AGATTN}N$	749.24	M	19
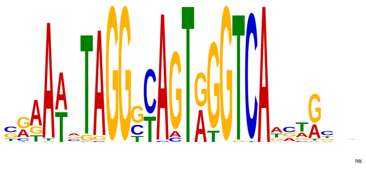	$NNNNNNT\overrightarrow{AGGCCA}GN\overrightarrow{NNNTCA}ANNNNNN$	293.66	DR:2	20

231 Rev-Erb-Beta-Yin2017-Rnd2 (ERR1005164)

PWM	Seed Sequence	Seed Seq Enrichment	Repeat	Rank
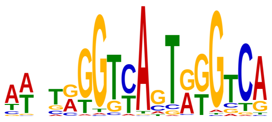 PWM	NNNNNNGTCAGNNNGTCANNNNNN	99.30	DR:2	1
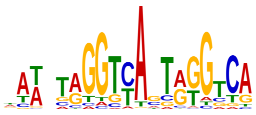 PWM	NNNNNNTAGGNNNNNNGTCANNNNNN	27.09	DR:2	2
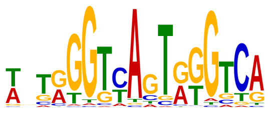 PWM	NNNNNNTCAGNNNGTCANNNNNN	36.07	DR:2	3
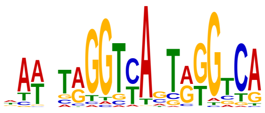 PWM	NNNNNNAGGTTNNNNNGTCANNNNNN	41.59	DR:2	4
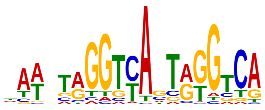 PWM	NNNNNNAGGTNNNNNNGTCANNNNNN	26.81	DR:2	5
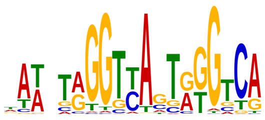 PWM	NNNNNNAGGTTANNNNGTCANNNNNN	130.66	DR:2	6
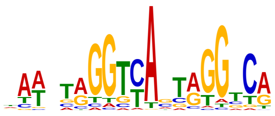 PWM	NNNNNNAGGTNNNNAGGTCNNNNNN	43.61	DR:2	7
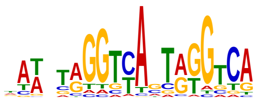 PWM	NNNNNNAGGTNNNNNNGTCACNNNNNN	27.15	DR:2	8
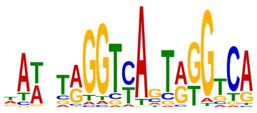 PWM	NNNNNNAGGTTNNNNNGTCACNNNNNN	40.87	DR:2	9

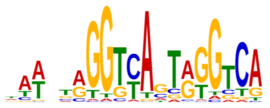 PWM	NNNNNNAGGTCNNNNNGTCANNNNNNN	60.96	DR:2	10
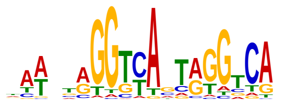 PWM	NNNNNNAGGTCNNNNGGTCNNNNNN	52.46	DR:2	11
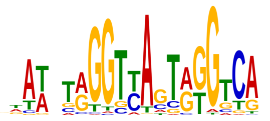 PWM	NNNNNNAGGTTANNNNGTCACNNNNNN	120.90	DR:2	12
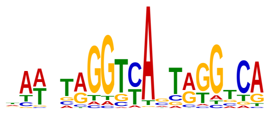 PWM	NNNNNNTAGGNNNNNNGGTCNNNNNN	21.61	DR:2	13
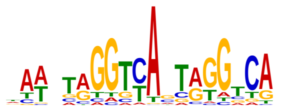 PWM	NNNNNNAGGTNNNNNGGTCNNNNNN	21.22	DR:2	14
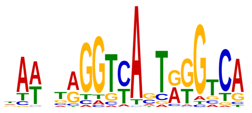 PWM	NNNNNNTTAGGNNNNNNNGTCANNNNNNN	19.54	DR:2	15
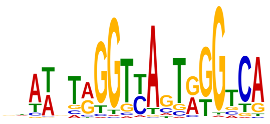 PWM	NNNNNNAGGTTANNNNNTCAGNNNNNN	48.95	DR:2	16
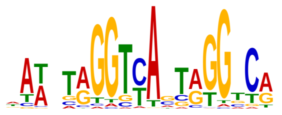 PWM	NNNNNNAGGTTNNNAGGTCNNNNNN	67.77	DR:2	17
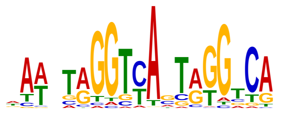 PWM	NNNNNNAGGTTNNNNGGTCNNNNNN	32.61	DR:2	18

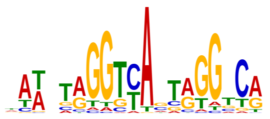 PWM	$NNNNNNT\overrightarrow{AGGNNN}NN\overrightarrow{AGGTCN}NNNNN$	42.77	DR:2	19
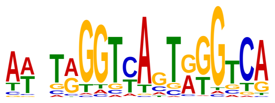 PWM	$NNNN\overrightarrow{NNGTTA}NN\overrightarrow{NNGTCA}NNNNNN$	27.04	DR:2	20

232 Rev-Erb-Beta-Yin2017-Rnd2 (ERR1005165)

PWM	Seed Sequence	Seed Seq Enrichment	Repeat	Rank
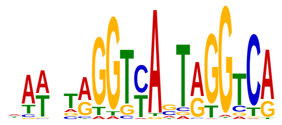	NNNNNNAGGTCANNAGGTCNNNNNN	306.52	DR:2	1
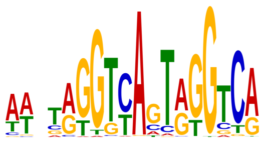	NNNNNNGTCAGTNNGTCAANNNNNN	296.32	DR:2	2
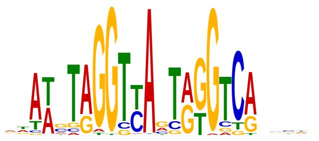	NNNNNNTAGGTTANNNNNTCAANNNNNN	209.21	DR:2	3
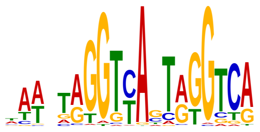	NNNNNNAGGTCANNNNGTCAANNNNNN	211.29	DR:2	4
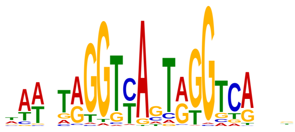	NNNNNNAGGTCANNNNNTCAANNNNNN	70.22	DR:2	5
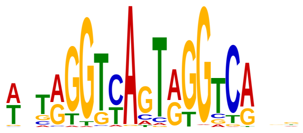	NNNNNNTCAGTNNGTCAANNNNNN	118.18	DR:2	6
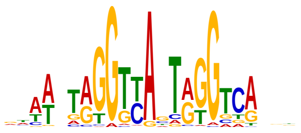	NNNNNNTAGGTCANNNNNTCAANNNNNN	179.57	DR:2	7
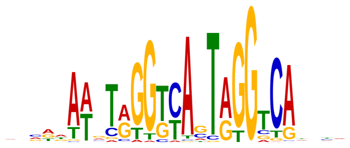	NNNNNNATGTNNNNCAGTAGGTNNNNNN	327.12	DR:6	8
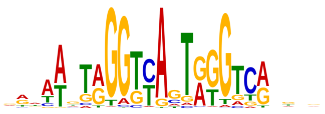	NNNNNNTAGGTCANNNNNTCACNNNNNN	130.59	DR:2	9

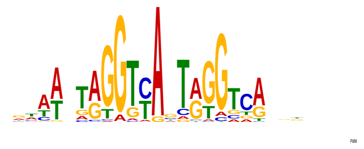	$NNNNNNT\overrightarrow{AGGTCN}NN\overrightarrow{NNNTCA}ANNNNNN$	52.69	DR:2	10
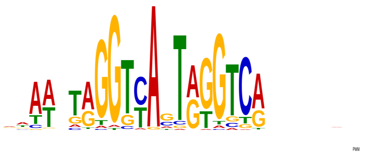	$NNNNNN\overrightarrow{AGGTCA}NN\overrightarrow{NNGTCA}CNNNNNN$	169.51	DR:2	11
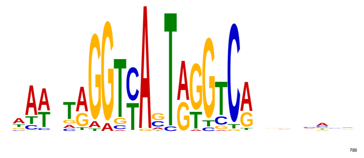	$NNNNNN\overrightarrow{AGGTCA}NN\overrightarrow{NNGTCA}CGNNNNNN$	218.28	DR:2	12
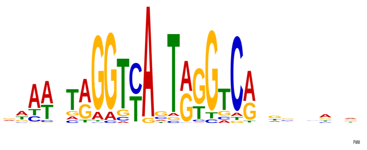	$NNNNNN\overrightarrow{AGGTCA}NN\overrightarrow{NNNTCA}CGNNNNNN$	80.42	DR:2	13
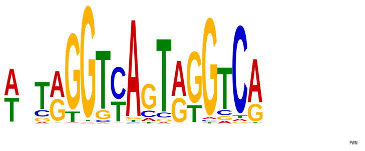	$NNN\overrightarrow{NNNTCA}GT\overrightarrow{NNGTCG}NNNNNN$	43.36	DR:2	14
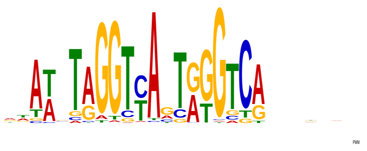	$NNNNNN\overrightarrow{AGGTTA}NN\overrightarrow{NNNTCA}ANNNNNN$	62.19	DR:2	15
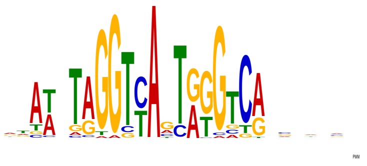	$NNNNNN\overrightarrow{AGGTTA}GN\overrightarrow{NNNTCA}ANNNNNN$	109.45	DR:2	16
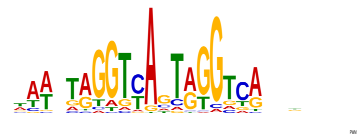	$NNNNNN\overrightarrow{AGGTCN}NN\overrightarrow{NNNTCA}ANNNNNN$	20.67	DR:2	17
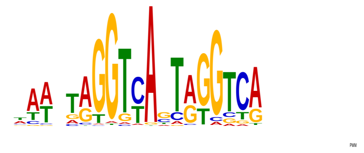	$NNNNNN\overrightarrow{AGGTCN}NN\overrightarrow{NNGTCA}ANNNNNN$	58.65	DR:2	18

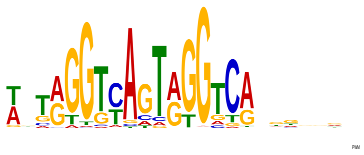	$NNN\overrightarrow{NNNTCA}GT\overrightarrow{NNNTCA}ANNNNNN$	35.96	DR:2	19
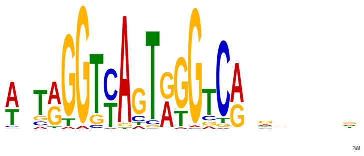	$NNN\overrightarrow{NNNTCA}GT\overrightarrow{NNGTCA}CGNNNNNN$	135.12	DR:2	20

233 Rev-Erb-Beta-Yin2017-Rnd3 (ERR1005166)

PWM	Seed Sequence	Seed Seq Enrichment	Repeat	Rank
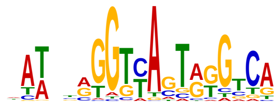	*NNNNNNGGTCNNNNNGTCANNNNNN*	26.19	DR:2	1
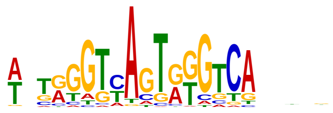	*NNNNNNTCAGNNNNTCAGNNNNNN*	24.09	DR:2	2
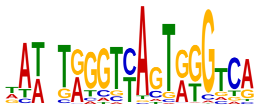	*NNNNNNGGTTNNNNNNNCACGNNNNNN*	9.73	M	3
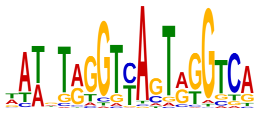	*NNNNNNGGTTNNNNNNNCAGCNNNNNN*	7.93	M	4
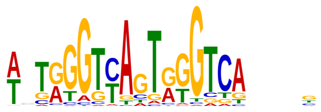	*NNNNNNTCAGNNNNNCAGGNNNNNN*	14.80	DR:7	5
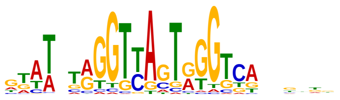	*NNNNNNAGGTNNNNNNNNCAGCNNNNNN*	8.04	M	6
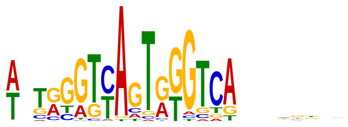	*NNNNNNTCAGNNNNNCAGGGNNNNNN*	16.54	DR:7	7
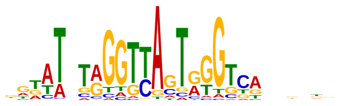	*NNNNNNTAGGNNNNNNNNNCAGCNNNNNN*	7.35	M	8
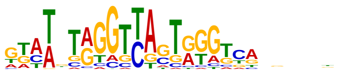	*NNNNNNAGGTNNNNNNNNCACGNNNNNN*	9.28	M	9

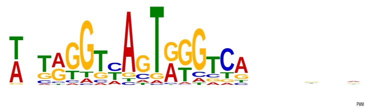	*NNNNNNTCAGNNNNNCAGTNNNNNN*	12.23	M	10
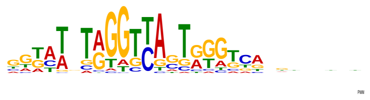	*NNNNNNTAGGNNNNNNNNNCACGNNNNNN*	9.84	M	11
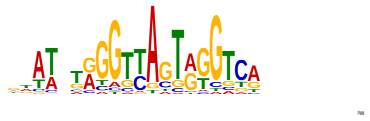	*NNNNNNAGGTNNNNNNNNCAAGNNNNNN*	9.76	M	12
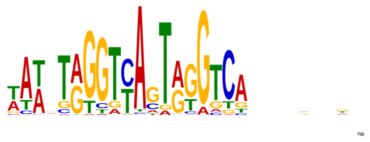	*NNNNNNGGTTNNNNNNNCAGCGNNNNNN*	10.54	M	13
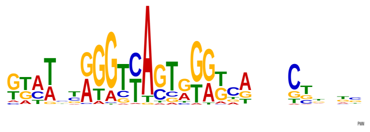	*NNNNNNAGGTNNNNNNNNCACGGNNNNNN*	9.89	M	14
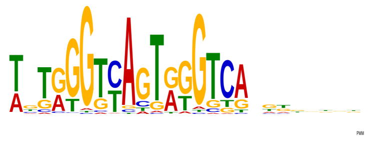	*NNNNNNTCAGNNNNNCAAGNNNNNN*	15.67	M	15
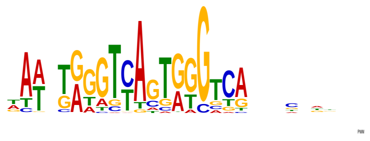	*NNNNNNGGTTNNNNNNNCACGGNNNNNN*	9.95	M	16
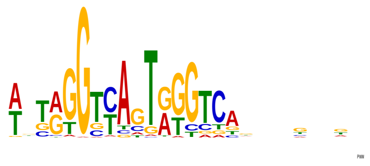	*NNNNNNTCAGNNNNNCAGTTNNNNNN*	14.29	DR:7	17
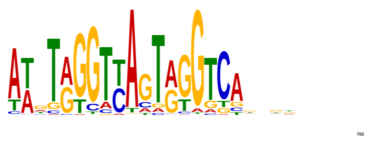	*NNNNNNGTTANNNNNNNCAGCGNNNNNN*	13.04	M	18

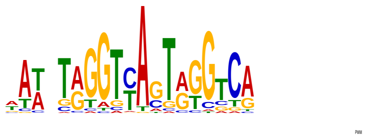	$NNNNN\overrightarrow{NGGTTN}NNNNNNCAAGNNNNNN$	10.75	M	19
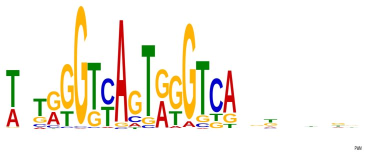	$NNN\overrightarrow{NNNTCA}GNNNNNCA\overrightarrow{AGGNNN}NNN$	18.71	DR:8	20

234 Rev-Erb-Beta-Yin2017-Rnd3 (ERR1005167)

PWM	Seed Sequence	Seed Seq Enrichment	Repeat	Rank
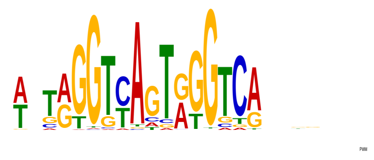	NNNNNNTCAGNNNNTCAANNNNNN	52.39	DR:2	1
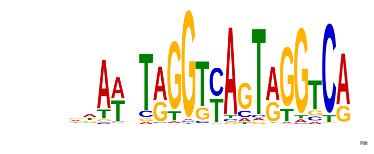	NNNNNNATGTNNNNNAGTANNNNNN	42.76	M	2
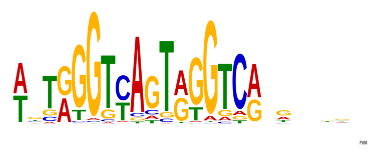	NNNNNNTCAGNNNNNTCGCNNNNNN	23.77	M	3
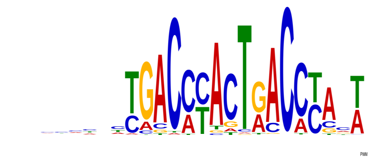	NNNNNNGCTGANNNNNCTGANNNNNN	56.11	IR:5	4
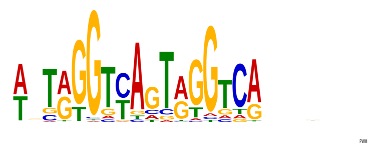	NNNNNNTCAGNNNNNTCGANNNNNN	19.18	M	5
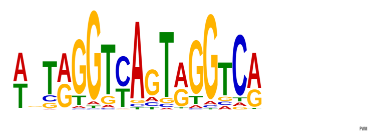	NNNNNNTCAGNNNNNTCGTNNNNNN	23.12	M	6
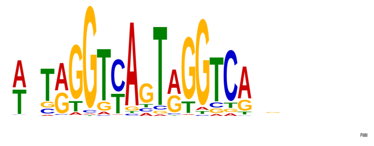	NNNNNNTCAGNNNNNNCAAGNNNNNN	21.61	M	7
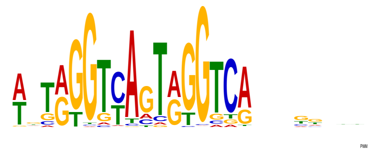	NNNNNNTCAGNNNNTCAANNNNNN	47.49	DR:2	8
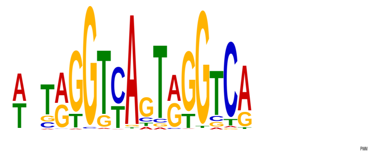	NNNNNNTCAGNNNNTCAAGNNNNNN	70.47	DR:2	9

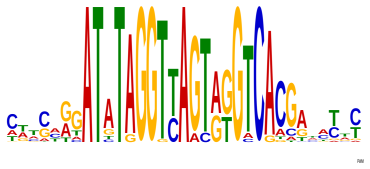	*NNNNNNGATATAGGNNNNNNNNTCACGNNNNNNN*	1342.39	M	10
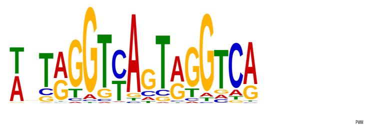	*NNNNNNTCAGNNNNTCGGNNNNNN*	16.97	M	11
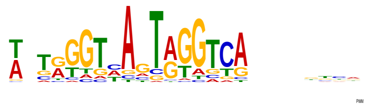	*NNNNNNTCAGNNNNNCAGTNNNNNN*	18.05	M	12
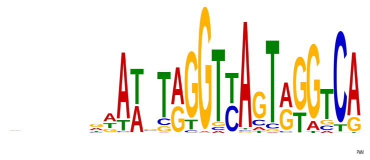	*NNNNNNGAATNNNNNTTAGTNNNNNN*	59.23	M	13
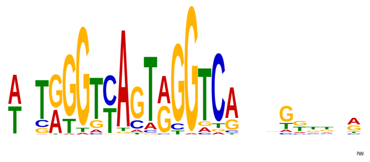	*NNNNNNTCAGNNNNTCAAAGNNNNNN*	62.12	DR:2	14
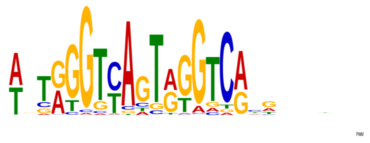	*NNNNNNTCAGNNNNTCGCGNNNNNN*	40.48	M	15
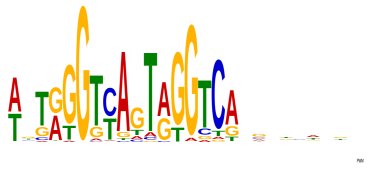	*NNNNNNTCAGNNNNNCAAGTNNNNNN*	27.57	DR:7	16
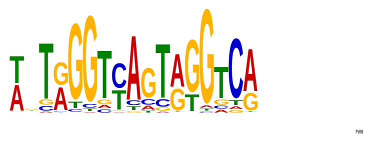	*NNNNNNTTAGNNNNTCGANNNNNN*	14.85	M	17
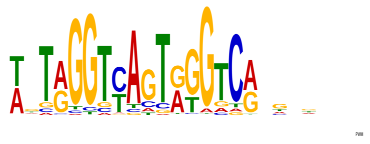	*NNNNNNTTAGNNNNTCGCNNNNNN*	16.98	M	18

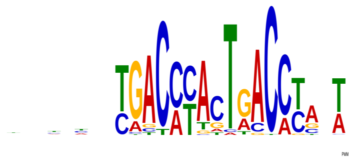	$NNNNNN\overrightarrow{CGCTGA}NNNNC\overleftarrow{TGANN}NNNN$	69.72	IR:5	19
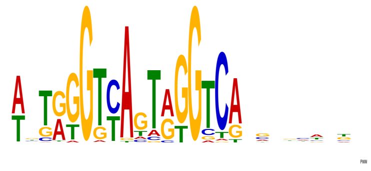	$NNN\overrightarrow{NNNTCA}GNNNNTC\overrightarrow{AAGTN}NNNNN$	93.23	DR:7	20

235 Rev-Erb-Beta-Yin2017-Rnd4 (ERR1005168)

PWM	Seed Sequence	Seed Seq Enrichment	Repeat	Rank
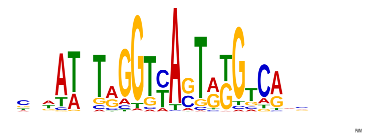	NNNNNNATAGNNNNNNNNGTCANNNNNN	7.01	M	1
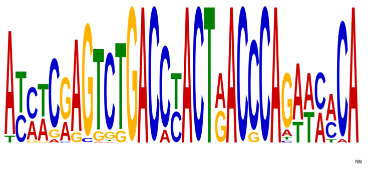	NNNNNNAGTCTGANNNNNNNNNCCAGNNNNNN	50.37	M	2
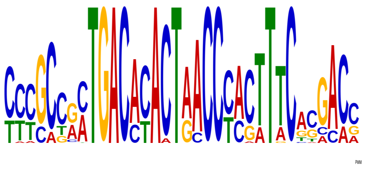	NNNNNNGATGACANNNNNNNNNNNTTTCNNNNNN	35.43	M	3
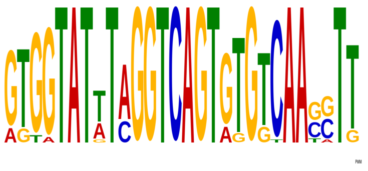	NNGGTATATNNNNNNNNNNNNCAAGGNN	42.26	DR:16	4
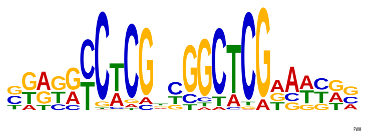	NNNNNNCTCGNNNGCTCGNNNNNN	8.35	M	5
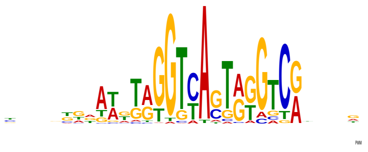	NNNNNNGAAANNNNNNNNNNNNGGTCNNNNNN	6.54	M	6
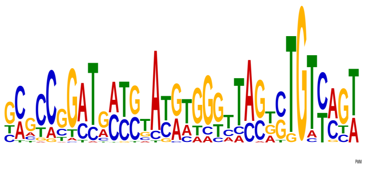	NNNCCGGANNNNNNNNNNNNNNNNNNTGTCNNN	10.67	M	7
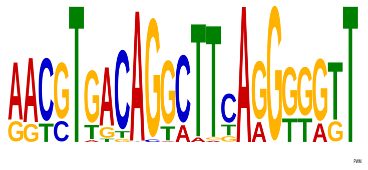	NNNNNNACAGGNTTCAGNNNNNN	11.46	M	8
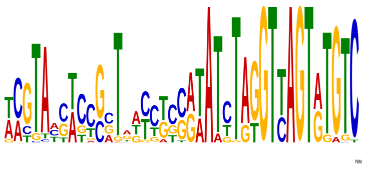	TCGTANNNNNNNNNNNNNNNNNNNNNNNNNNNNNNNTGTC	9.23	M	9

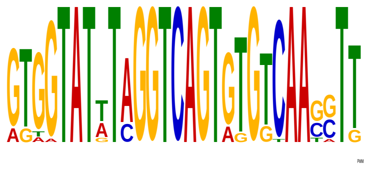	*NNGGTATANNNNNNNNNNNNNCAAGGNN*	21.54	DR:16	10
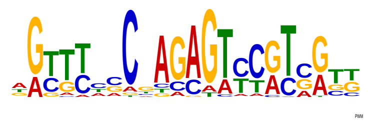	*NNNNNNGCGAGNGTCCNNNNNN*	4.49	M	11
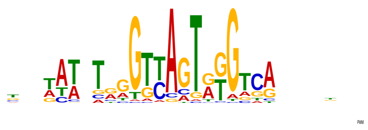	*NNNNNNATAGNNNNNNNNNNTCACNNNNNN*	3.57	M	12
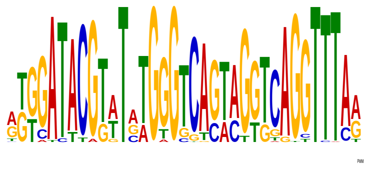	*NNNGATANNNNNNNNNNNNNNNNNNNNNCAGGTTNNN*	18.78	M	13
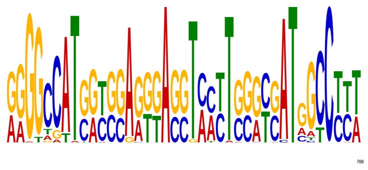	*NNNGCCANNNNNNNNNNNNNNNNNNNNNNNNNNGGCCNNN*	7.59	DR:24	14
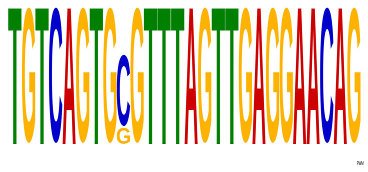	*NNNNNNTGCGNNTAGTTGAGNNNNNN*	26.51	M	15
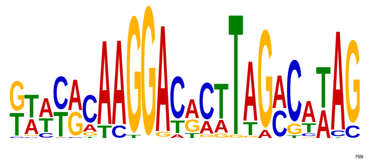	*NNNNNNAAGGANNCTTAGNNNNNN*	17.32	M	16
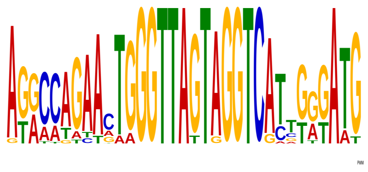	*NNNNNNGAACTGGNNNNNNNNNTCATTNNNNNN*	65.18	ER:8	17
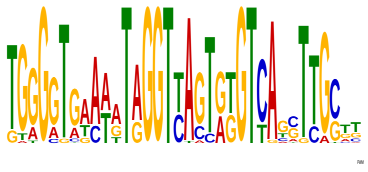	*NNNNNNGAAAGTNNNNNNNNNNNNCAGGNNNNNN*	12.38	DR:11	18

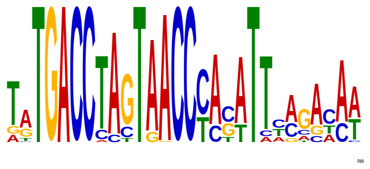	*NNNNNNCTAGTAANNNNGATTTNNNNNN*	42.39	IR:3	19
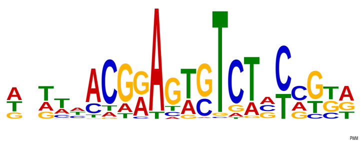	*NNNNNNCGAAGNGTCTNNNNNN*	7.39	M	20

236 Rev-Erb-Beta-Yin2017-Rnd4 (ERR1005169)

PWM	Seed Sequence	Seed Seq Enrichment	Repeat	Rank
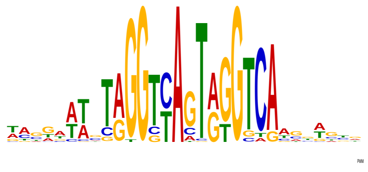	NNNNNNTCTAGNNNNNNNNNNTCAANNNNNN	29.43	M	1
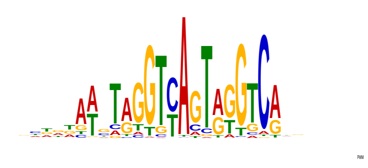	NNNNNNAAGTNNNNNNNNNNNNTCATNNNNNN	15.52	DR:6	2
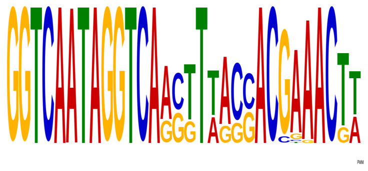	NNNCAATAGNNNNNNNNNNNNNACGAAANNN	92.26	M	3
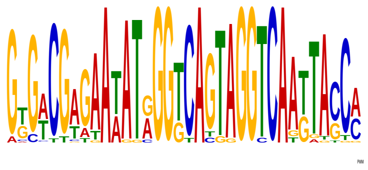	NNNNNNAGAATATNNNNNNNNNNNNTCAANNNNNN	112.03	DR:9	4
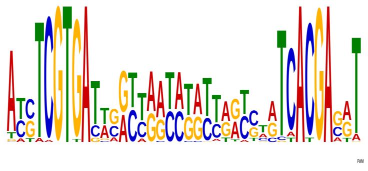	NNNTCGTGANNNNNNNNNNNNNNNNNNNNNNNACGANN	88.34	M	5
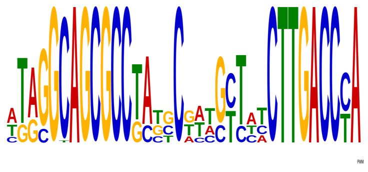	NNNNGCAGCGCCNNNNNNNNNNNNNNCTTGANNNN	435.46	IR:15	6
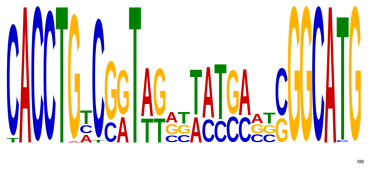	CACCTGNNNNNNNNNNNNNNNNNGGCATG	104.28	ER:16	7
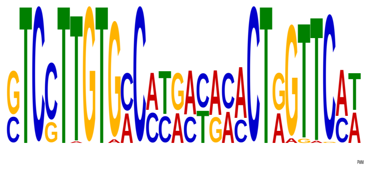	NNCCTTGTGNNNNNNNNNNNNNNGTTCNN	54.26	ER:16	8
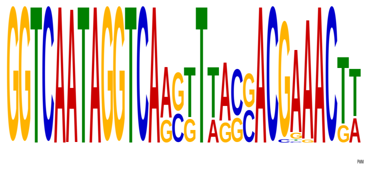	NNNCAATANNNNNNNNNNNNNNACGAAANNN	45.30	M	9

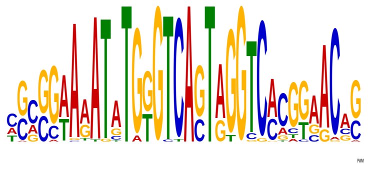	*NNNNNNAAATATNNNNNNNNNNNNTCACGNNNNNN*	77.31	M	10
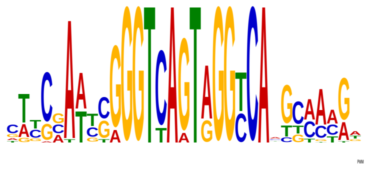	*NNNNNNATGGGGTCNNNNNNNTCAAGNNNNNN*	220.01	DR:2	11
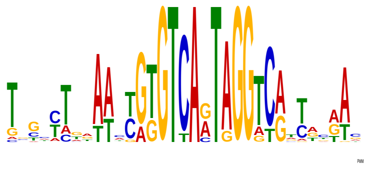	*NNNNNNGAAATTNNNNNNNNNNNNTCAANNNNNN*	21.93	ER:9	12
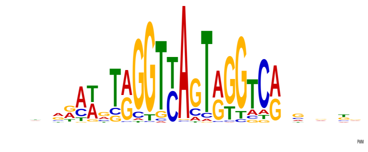	*NNNNNNATGTNNNNNNNNNNNNTCGCNNNNNN*	7.97	M	13
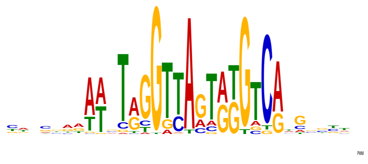	*NNNNNNAATGTNNNNNNNNNNNNTCAANNNNNN*	21.78	DR:6	14
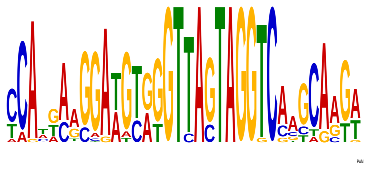	*NNNNNNAGGATGTNNNNNNNNNNNNTCAANNNNNN*	54.60	DR:9	15
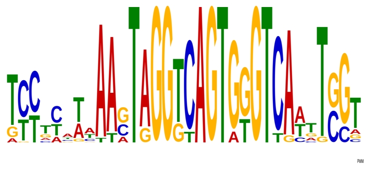	*NNNNNAATAAGTNNNNNNNNNNNNTCAANNNNN*	53.68	DR:6	16
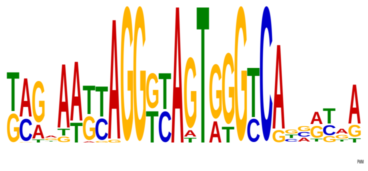	*NNNAAAGTNNNNNNNNNNNNTCAGAGNNN*	28.97	DR:6	17
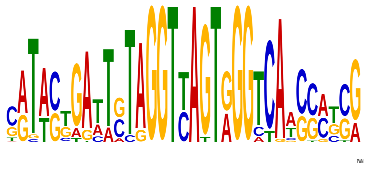	*NNNNNNGATTGTANNNNNNNNNNNTCAANNNNNN*	84.34	M	18

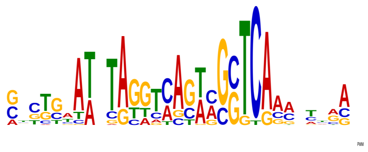	$NNNNNNATTTANNNNNNN\overrightarrow{NNNTC}AAANNNNNN$	29.77	M	19
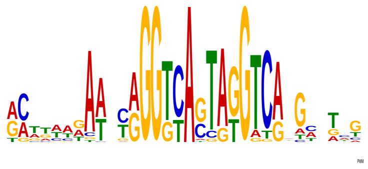	$NNNNNN\overrightarrow{AAATT}ANNNNNNN\overrightarrow{NNNTC}AANNNNNN$	24.51	DR:7	20

237 RORA-Jolma2013-Rnd3 (ERR194476)

PWM	Seed Sequence	Seed Seq Enrichment	Repeat	Rank
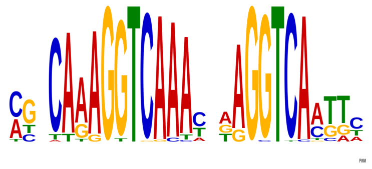	NNNNAAAGGTCANNNNNAGGTCANNNN	5798.62	DR:5	1
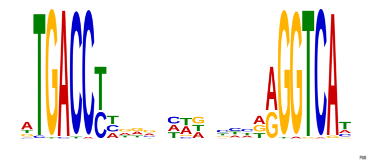	NNTGACCTNNNNNNNNNNNNNAGGTCANN	695.08	ER:13	2
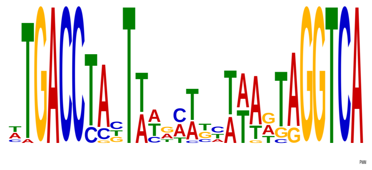	NTGACCTANNNNNNNNNNNNNTAGGTCN	1376.61	ER:15	3
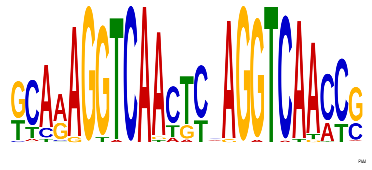	NNNAGGTCAANNNCAGGTCANNNN	3130.18	DR:5	4
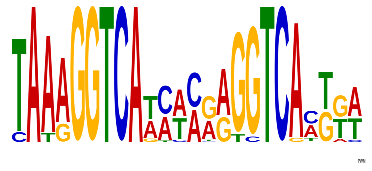	NNNAGGTCANNNNGAGGTCACNNN	1803.05	DR:5	5
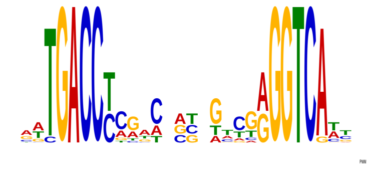	NNNTGACCTNNNNNNNNNNNAGGTCANNN	650.32	ER:12	6
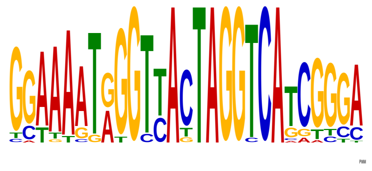	NNNNNNTAGGTCANTAGGTCANNNNNNN	2865.33	DR:2	7
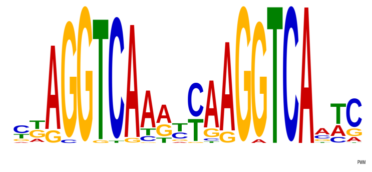	NNAGGTCAANNNNAGGTCAGNN	1682.88	DR:5	8
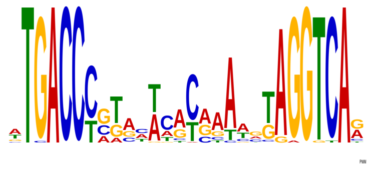	NTGACCCNNNNNNNNNNNNNNAGGTCAN	410.61	ER:14	9

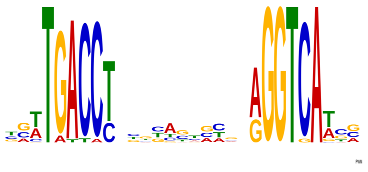	*NNNTGACCTNNNNNNNNNNNNGGTCANNN*	103.63	ER:11	10
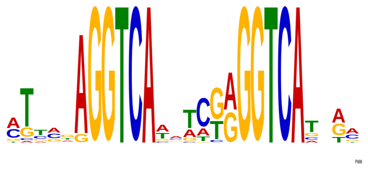	*NNNNGAGGTCANNNNNGGGTCANNNN*	641.70	DR:5	11
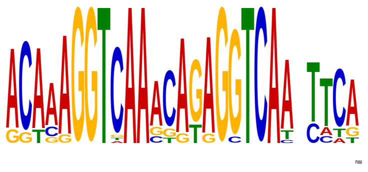	*NNNNAGGTCANNNNNAGGTCAAGNNNN*	2381.15	DR:5	12
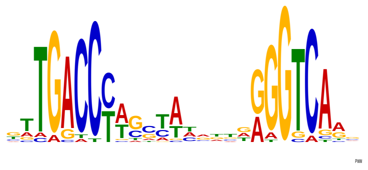	*NNTGACCTNNNNNNNNNNNGGTCANN*	34.98	ER:10	13
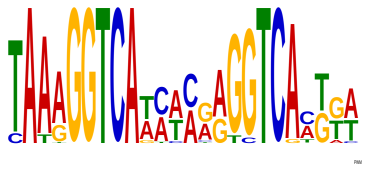	*NNNAGGTCNNNNNGAGGTCACNNN*	518.40	DR:5	14
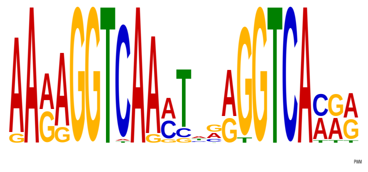	*NNNAGGTCANNNTGAGGTCANNN*	1958.23	DR:5	15
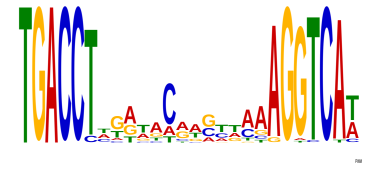	*NTGACCTANNNNNNNNNNNNAGGTCAN*	1170.38	ER:13	16
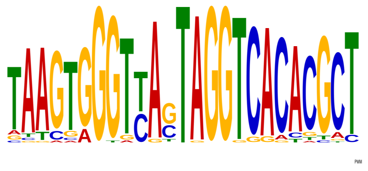	*NNNNNNGGTCAGTAGGTCANNNNNN*	698.71	DR:2	17
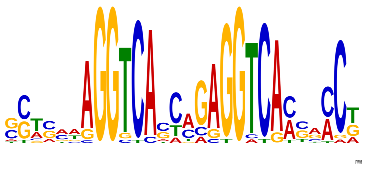	*NNNNNNAGGTCANNNNAGGTCANNNNNN*	209.02	DR:4	18

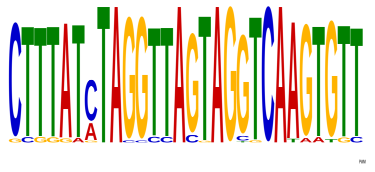	$NNNNNNCT\overrightarrow{AGGNNNN}NT\overrightarrow{AGGTCA}NNNNNN$	483.64	DR:2	19
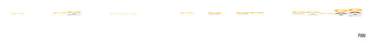	$NNNNNNGGGGNNNNNGGGGNNNNNN$	5.88	M	20

238 RORA-Jolma2013-Rnd4 (ERR194477)

PWM	Seed Sequence	Seed Seq Enrichment	Repeat	Rank
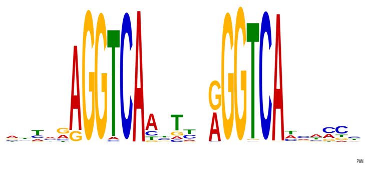	*NNNNNNGGTCANNNNNNGGTCANNNNNN*	525.93	DR:5	1
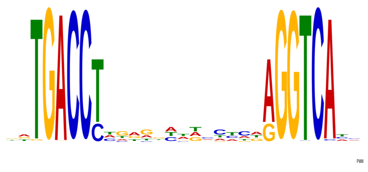	*NNTGACCNNNNNNNNNNNNNNGGTCANN*	536.30	ER:13	2
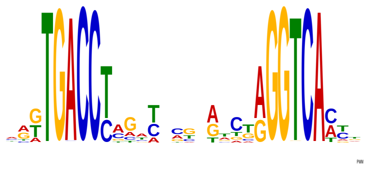	*NNNTGACCNNNNNNNNNNNNNGGTCANNN*	634.55	ER:12	3
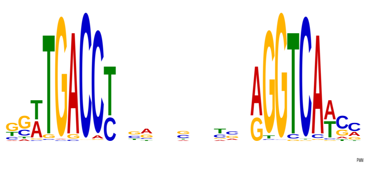	*NNNTGACCNNNNNNNNNNNNGGTCANNN*	155.97	ER:11	4
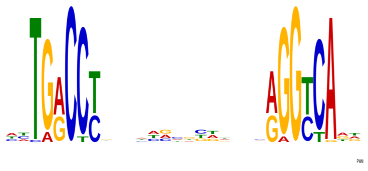	*NNTGACCNNNNNNNNNNNNNNNGGTCANN*	84.60	ER:14	5
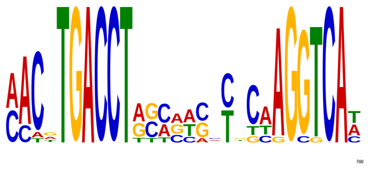	*NNCGTGACCNNNNNNNNNNNNAGGTCNN*	269.78	ER:11	6
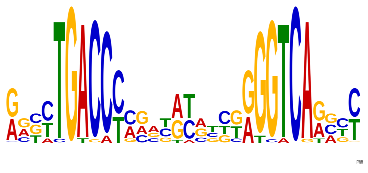	*NNNNTGACCNNNNNNNNNNNGGTCANNNN*	88.96	ER:10	7
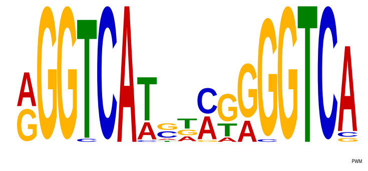	*NGGTCAACNCGAGGTCN*	2488.91	DR:5	8
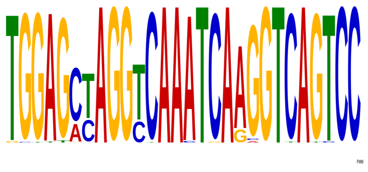	*NNNNNCTAGGTCNNNNNAAGGTCNNNNN*	3302.87	DR:5	9

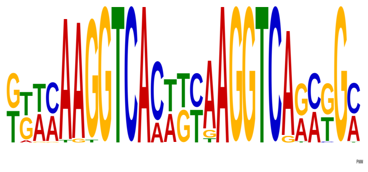	*NNNNNNGGTCACANNAAGGTCNNNNNN*	1204.57	DR:5	10
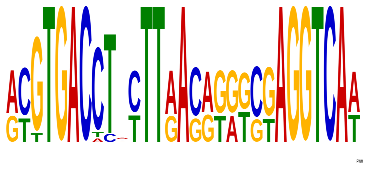	*NNGTGACCTGNNNNNNNNNNNNAGGTCNN*	901.10	ER:13	11
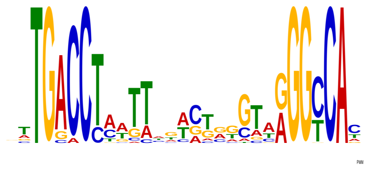	*NGTGACCNNNNNNNNNNNNNNNNGGTCAN*	76.62	ER:14	12
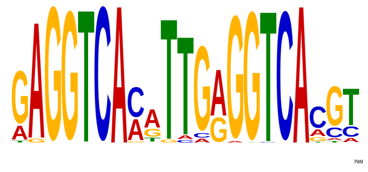	*NNGGTCACANNNNGGTCACNN*	2202.94	DR:5	13
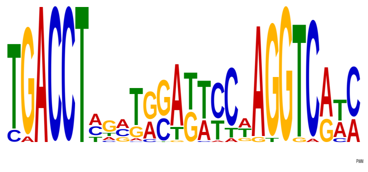	*NGACCTTANNNNNNNNNNNGGTCAAN*	479.70	ER:12	14
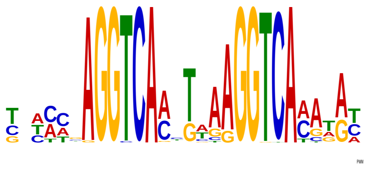	*NNNNNNAGGTCNNNNAAAGGTCNNNNNN*	1047.39	DR:5	15
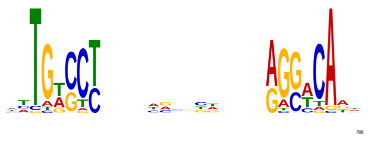	*NNNGACCNNNNNNNNNNNNNNNNGGTCNNN*	16.78	ER:14	16
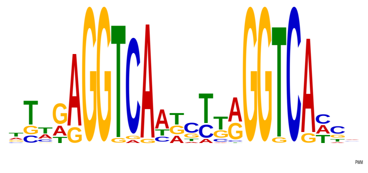	*NNNNGGGTCACNNNNAGGTCNNNN*	122.46	DR:5	17
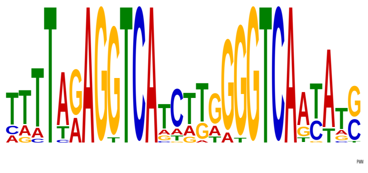	*NNNNAGAGGTCNNNNNNNGGTCAGNNNN*	1246.66	DR:5	18

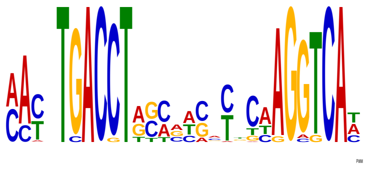	$NNCG\overleftarrow{TGACCN}NNNNNNNNNNNN\overrightarrow{NGGTCN}N$	107.09	ER:11	19
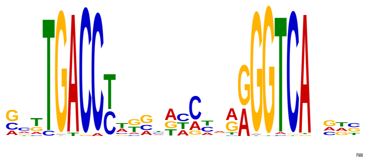	$NNN\overleftarrow{NGACCT}NNNNNNNNNN\overrightarrow{NGGTCA}NNNN$	107.62	ER:10	20

239 RORB-Yin2017-Rnd2 (ERR1005892)

PWM	Seed Sequence	Seed Seq Enrichment	Repeat	Rank
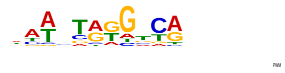	NNNNNNAAGTNNGTCANNNNNN	8.53	M	1
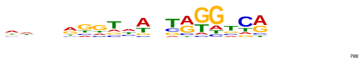	NNNNNNGTTANNNNGTCANNNNNN	5.26	DR:2	2
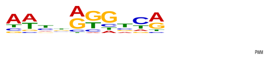	NNNNNNAATGNNGTCANNNNNN	5.09	M	3
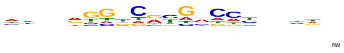	NNNNNNGGTCNNGACCNNNNNN	4.31	IR:0	4
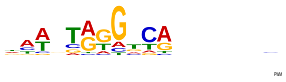	NNNNNNAAGTNNNTCAGNNNNNN	6.21	M	5
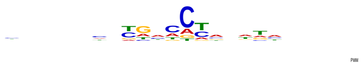	NNNNNNCGCGNNNNAGATNNNNNN	2.68	M	6
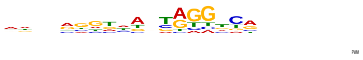	NNNNNNGTTANNNNNTCAGNNNNNN	3.51	DR:2	7
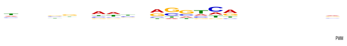	NNNNNNTATTNNNNTCATNNNNNN	3.24	M	8
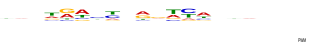	NNNNNNTAACNNNGTTANNNNNN	4.07	M	9

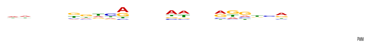	*NNNNNNGTCANNNNNNNNNGTCANNNNNN*	3.22	DR:7	10
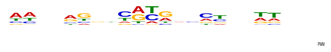	*NNNNNNTAGGNNNNNNCCTANNNNNN*	2.79	IR:0	11
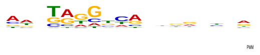	*NNNNNNGAAANNNNNNCAGTNNNNNN*	2.89	M	12
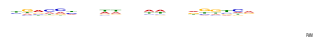	*NNNNNNGACCNNNNNNNNNNNNGGTCNNNNNN*	2.72	ER:10	13
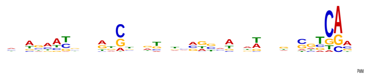	*NNAGAANNNNNNNNNNNNNNNNNNNNNNNNNNNGTCANN*	3.34	M	14
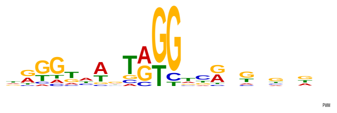	*NNNNNNTTAGTNNNTCACNNNNNN*	4.97	M	15
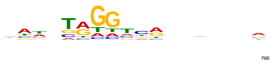	*NNNNNNAATCNNNNTCATNNNNNN*	2.94	M	16
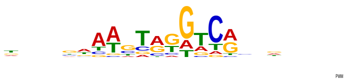	*NNNNNNAAAGTNNNTCGCNNNNNN*	6.11	M	17
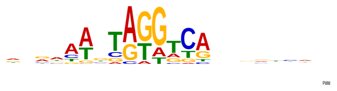	*NNNNNNAAGTNNNTCAAANNNNNN*	6.17	M	18

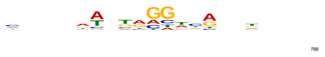	$NNNNNN\overrightarrow{AGATNN}\overrightarrow{NNNTCA}ANNNNNNN$	2.47	DR:0	19
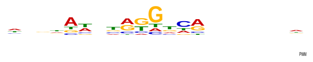	$NNNNNNTTATNN\overrightarrow{NNNTCA}ANNNNNNN$	2.97	M	20

240 RORB-Yin2017-Rnd2 (ERR1005893)

PWM	Seed Sequence	Seed Seq Enrichment	Repeat	Rank
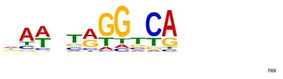	NNNNNNAACTNNGTCANNNNNN	9.89	ER:0	1
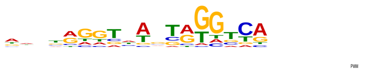	NNNNNNGTTANNNNGTCANNNNNN	6.05	DR:2	2
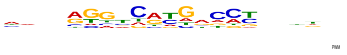	NNNNNNGGTCNNGACCNNNNNN	4.67	IR:0	3
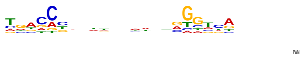	NNNNNNTGACNNNNNNNNNNNNNNGTCANNNNNN	4.34	ER:10	4
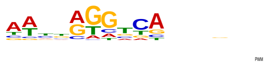	NNNNNNAATGNNGTCANNNNNN	5.18	M	5
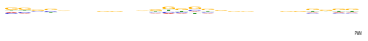	NNNNNNGGGGNNNNNNNGGGGNNNNNN	3.15	M	6
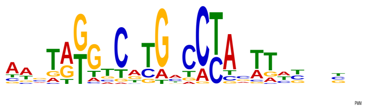	NNNNNNGTCATGNNNNACTTNNNNNN	10.75	IR:4	7
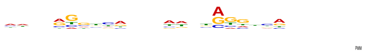	NNNNNNGTCANNNNNNNNNGTCANNNNNN	3.59	DR:7	8
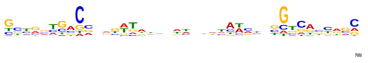	NNNNNTGACNNNNNNNNNNNNNNNNNNNNNGTCANNNNNN	5.60	ER:18	9

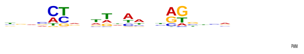	NNNNNNTGACNNNNNNNNNNNNNGTCANNNNNN	2.83	ER:9	10
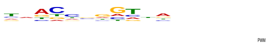	NNNNNNTGACNNNGTCANNNNNN	3.45	M	11
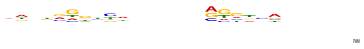	NNNNNNGTCANNNNNNNNGTCANNNNNN	3.20	DR:6	12
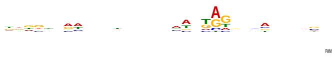	NNNNNNGTCANNNNNNNNNNNNNNNNGTCANNNNNN	2.71	DR:14	13
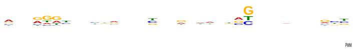	NNNNNNGTCANNNNNNNNNNNNNNNNNGTCANNNNNN	2.59	DR:15	14
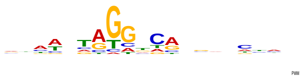	NNNNNNAAGTNNNNCATGNNNNNN	3.99	M	15
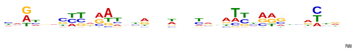	NNNNTGACNNNNNNNNNNNNNNNNNNNNNNNGTCANNNN	2.54	ER:19	16
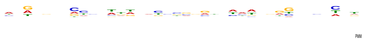	NNNTGACNNNNNNNNNNNNNNNNNNNNNNNNGTCANNN	2.25	ER:20	17
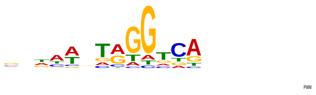	NNNNNNAAGTNNNTCAANNNNNNN	5.34	M	18

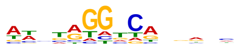	$NNNN\overleftarrow{NNATCT}\overrightarrow{NNNTCA}TNNNNNN$	4.53	ER:0	19
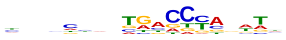	$NNNNNNACTGNNNN\overrightarrow{AGATN}NNNNN$	3.69	M	20

241 RORB-Yin2017-Rnd3 (ERR1005894)

PWM	Seed Sequence	Seed Seq Enrichment	Repeat	Rank
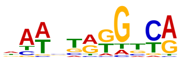	NNNNNNAACTNNGTCANNNNNN	11.17	ER:0	1
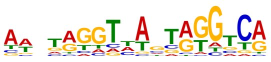	NNNNNNGTTANNNNGTCANNNNNN	9.80	DR:2	2
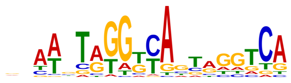	NNNNNNAAGTNNNNCAGTANNNNNN	16.30	M	3
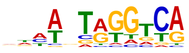	NNNNNNAAGTNNNTCAANNNNNN	9.49	M	4
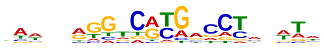	NNNNNNGGTCNNNNCCTANNNNNN	6.16	IR:0	5
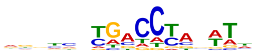	NNNNNNCATGNNNNAGATNNNNN	5.50	M	6
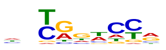	NNNNNNGTGGTCCANNNNNN	4.05	M	7
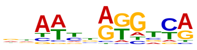	NNNNNNAATGNNGTCANNNNNN	6.52	M	8
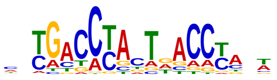	NNNNNNCGTGNNNNACTAANNNNNN	8.69	M	9

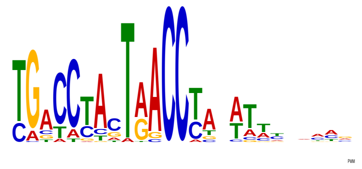	*NNNNNNTACTAANNNAGATTNNNNNN*	34.37	M	10
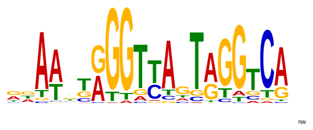	*NNNNNNAATTNNNTTAGTANNNNNN*	23.35	M	11
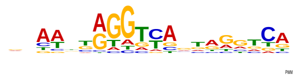	*NNNNNNGAATNNNNNCAGTNNNNNN*	4.34	M	12
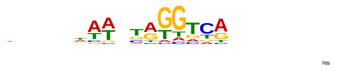	*NNNNNNGTATNNNNNTCAANNNNNN*	4.84	M	13
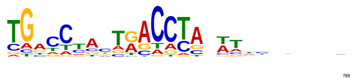	*NNNNNNTACTNNNNNNNATTCNNNNNN*	4.40	M	14
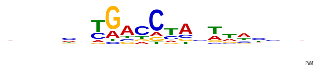	*NNNNNNTCGTNNNNTACTNNNNNN*	4.01	M	15
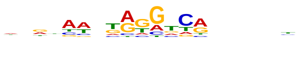	*NNNNNNGAATNNNNTCATNNNNNN*	4.03	M	16
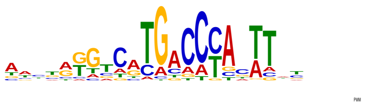	*NNNNNNGTCATGANNNACTTNNNNNN*	46.75	IR:0	17
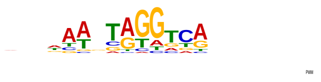	*NNNNNNAAAGNNNNTCAANNNNNN*	6.13	M	18

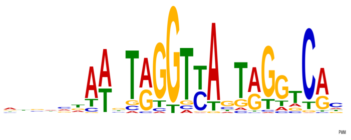	$NNNNNNT\overrightarrow{AAGTNN}NTTAGTANNNNNN$	38.88	M	19
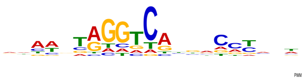	$NNNNNN\overrightarrow{AAGTNN}NNNNA\overleftarrow{TGANNN}NNN$	4.56	IR:4	20

242 RORB-Yin2017-Rnd3 (ERR1005895)

PWM	Seed Sequence	Seed Seq Enrichment	Repeat	Rank
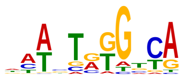	NNNNNNAAGTNNGTCANNNNNN	12.92	M	1
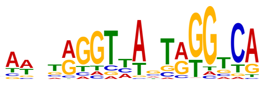	NNNNNNGGTTANNNNGTCANNNNNN	18.47	DR:2	2
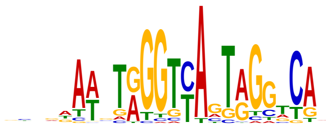	NNNNNNAAGTNNNTTAGTAGNNNNNN	47.88	M	3
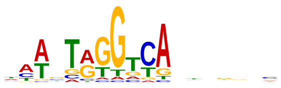	NNNNNNAAGTNNNTCAGNNNNNN	10.44	M	4
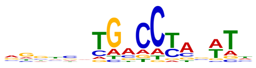	NNNNNNCATGNNNNAGATNNNNNN	6.08	M	5
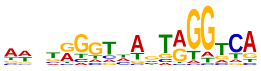	NNNNNNGTTANNNNNTCAGNNNNNN	5.97	DR:2	6
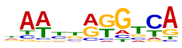	NNNNNNAATGNNGTCANNNNNN	6.74	M	7
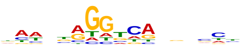	NNNNNNTAATNNNNNCATGNNNNNN	3.79	M	8
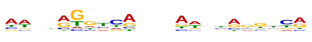	NNNNNNGTCANNNNNNNAGGTNNNNNN	4.17	DR:7	9

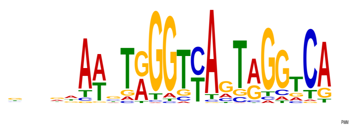	NNNNNNAAGTNNNTTAGTANNNNNN	23.56	M	10
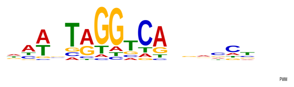	NNNNNNAAGTNNNNCATGNNNNNN	6.66	M	11
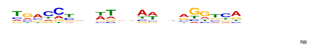	NNNNNNTGACNNNNNNNNNNNNNNGTCANNNNNN	4.11	ER:10	12
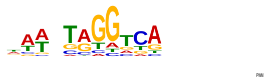	NNNNNNGTATNNNNNTCACNNNNNN	4.48	M	13
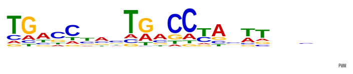	NNNNNNTACTNNNNTACTNNNNNN	5.29	M	14
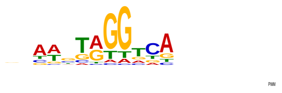	NNNNNNAAAANNNNNTCAANNNNNN	4.25	M	15
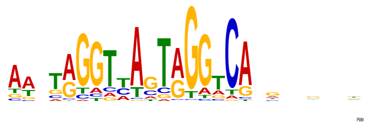	NNNNNNGTTAGTNNNNCACGNNNNNN	18.06	M	16
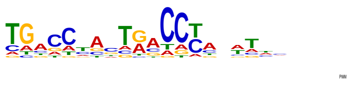	NNNNNNTACTNNNNNAGATNNNNNN	4.50	M	17
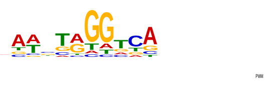	NNNNNNAAACNNNNTCACNNNNNN	4.48	M	18

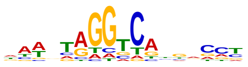	*NNNNNNAAGTNNNNNATGANNNNNNN*	4.62	IR:4	19
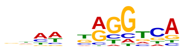	*NNNNNNTAATNNNNTCAANNNNNNN*	4.59	M	20

243 RORB-Yin2017-Rnd4 (ERR1005896)

PWM	Seed Sequence	Seed Seq Enrichment	Repeat	Rank
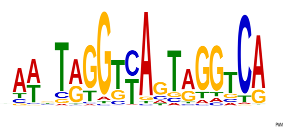	NNNNNNAAGTNNNNNAGTANNNNNN	21.76	M	1
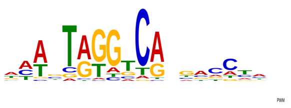	NNNNNNAAGTNNNNCATGNNNNNN	11.31	M	2
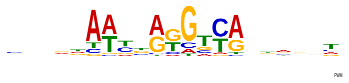	NNNNNNAATGNNGTCANNNNNN	8.30	M	3
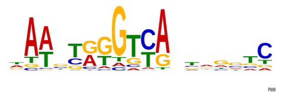	NNNNNNAAAGNNNNTCAGNNNNNN	11.50	M	4
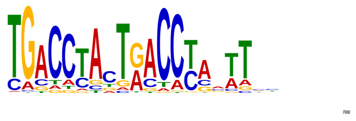	NNNNNNTACTNNNNNNATTCNNNNNN	10.10	M	5
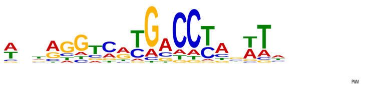	NNNNNNTCATNNNNNNAGATNNNNNN	7.27	DR:6	6
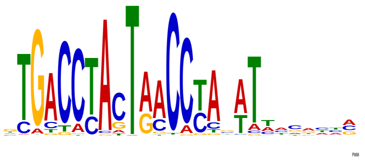	NNNNNNTACTANNNNAGATTNNNNNN	49.46	M	7
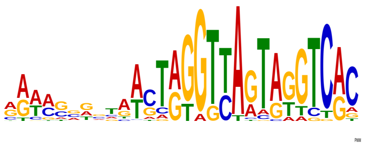	NNNNNNGCTAAGTNNNNNAGTANNNNNN	42.66	M	8
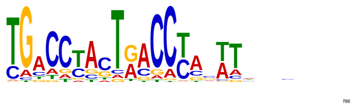	NNNNNNTACTNNNNNNNNTTTCNNNNNN	10.32	M	9

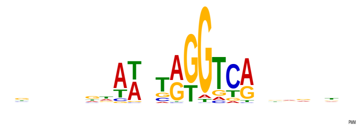	*NNNNNNGTATNNNNNTCACNNNNNN*	7.77	M	10
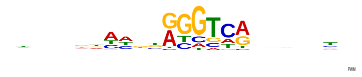	*NNNNNNTAATNNNNNTCATNNNNNN*	7.11	M	11
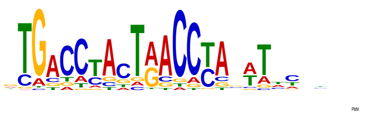	*NNNNNNTACTNNNNNATATNNNNNN*	9.61	M	12
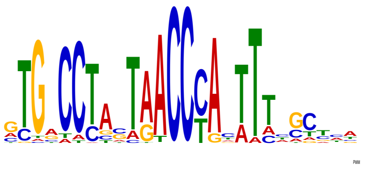	*NNNNNNTAGTAANNNACTTTNNNNNN*	60.39	IR:1	13
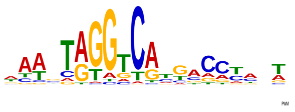	*NNNNNNAAGTNNNNNATGANNNNNN*	9.33	IR:4	14
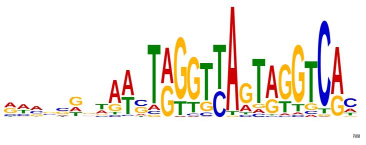	*NNNNNNCTAAGTNNNNNAGTANNNNNN*	26.41	M	15
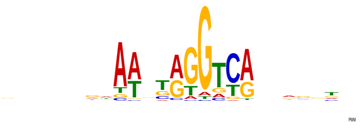	*NNNNNNGAAANNNNNTCACNNNNNN*	9.41	M	16
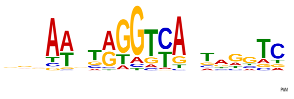	*NNNNNNAGAANNNNNTCAGNNNNNN*	7.71	M	17
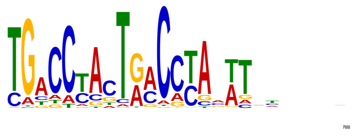	*NNNNNNTACTNNNNNACTTCNNNNNN*	19.70	M	18

244 RORB-Yin2017-Rnd4 (ERR1005897)

PWM	Seed Sequence	Seed Seq Enrichment	Repeat	Rank
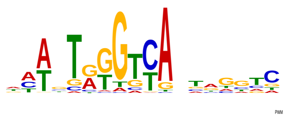	NNNNNNAAGTNNNTCAGNNNNNN	18.30	M	1
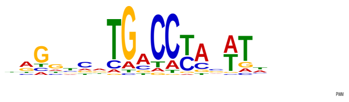	NNNNNNCATGNNNNAGTTNNNNNN	11.50	M	2
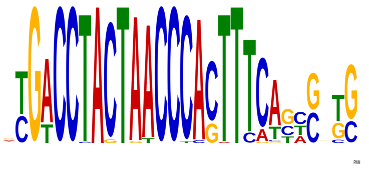	NNNNNNTACTAANNCACTTTCANNNNNN	789.57	DR:0	3
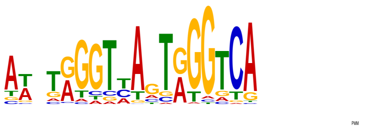	NNNNNNGTTAGNNNNTCAGNNNNNN	28.58	DR:2	4
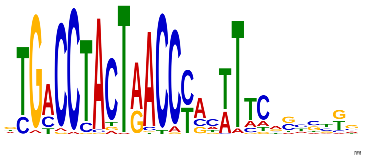	NNNNNNTACTAANNNNNTTTCANNNNNN	69.08	M	5
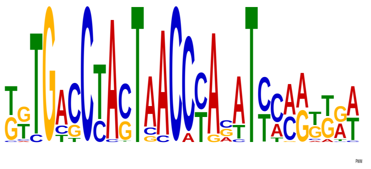	NNNNNNCTAGTAANNNACATTCNNNNNN	221.84	IR:1	6
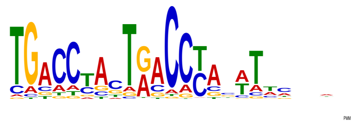	NNNNNNTACTNNNNNAGATNNNNNN	11.52	M	7
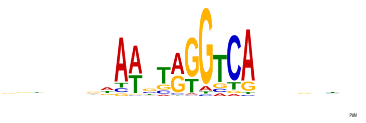	NNNNNNGAAANNNNNTCAANNNNNN	10.06	M	8
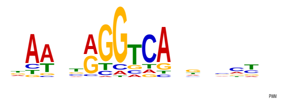	NNNNNNGAATNNNNTCATNNNNNN	5.91	M	9

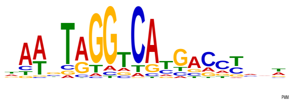	NNNNNNAAGTNNNNNATGANNNNNN	11.43	IR:4	10
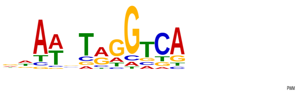	NNNNNNATATNNNNTCAANNNNNN	5.98	M	11
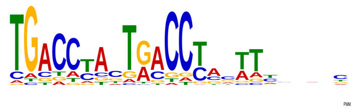	NNNNNNTACTNNNNNAATTNNNNNN	9.75	M	12
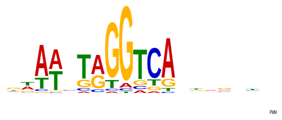	NNNNNNGTATNNNNNTCACNNNNNN	7.82	M	13
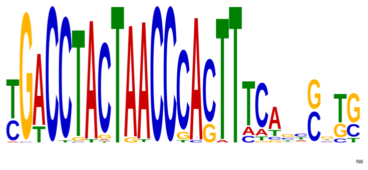	NNNNNNACTAANNCACTTTCANNNNNN	315.01	DR:0	14
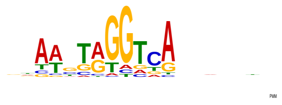	NNNNNNATAANNNNNTCACNNNNNN	6.77	M	15
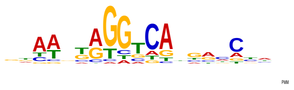	NNNNNNTAATNNNNNCATGNNNNNN	5.73	M	16
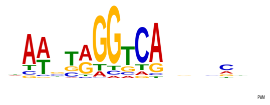	NNNNNNAAAANNNNNTCATNNNNNN	7.22	M	17
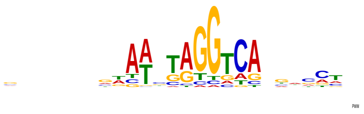	NNNNNNCGAANNNNNNTCATNNNNNN	6.41	M	18

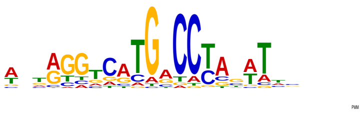	$NNN\overrightarrow{NNNTCA}TNNNNN\overrightarrow{AGATNN}NNNNN$	10.13	DR:6	19
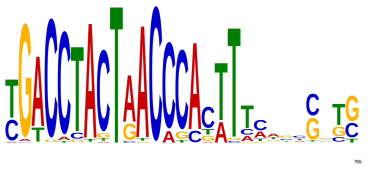	$NNNNNNACTAANNNNA\overrightarrow{CTTTCA}NNNNNNN$	121.34	M	20

245 RORB-Rep2-SMiLE-seq-Rnd1 (SRR3405080)

PWM	Seed Sequence	Seed Seq Enrichment	Repeat	Rank
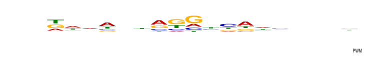	NNNNNNGTAGGTCANNNNNN	2.64	M	1
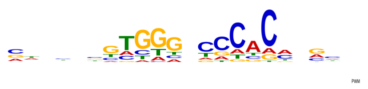	NNNNNNGTGGGNCCCANNNNNN	5.09	M	2
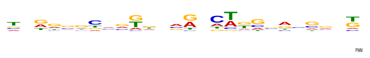	NNGGGGNNNNNNNNNNNNNNACGGNN	2.15	M	3
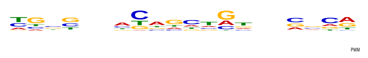	NNNNNNACAGCTGTNNNNNN	2.39	M	4
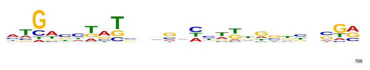	NNNNCCTANNNNNNNNNNNGGTCNNNN	2.46	ER:11	5
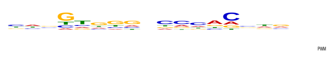	NNNNNNTGGGNCCCANNNNNN	2.59	M	6
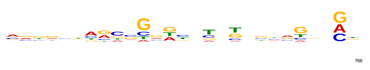	NNGGCTNNNNNNNNNNNNNNNGGGCNN	2.03	M	7
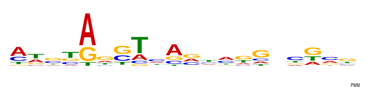	NNNNNGGTCANTAGGNNNNN	3.07	DR:2	8
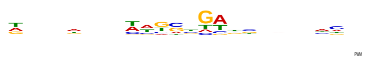	NNNTTAANNNNNNNNNNAATTNNN	1.31	M	9

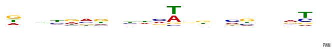	NNNNNNGAGGNNNNGGCCNNNNNN	1.87	DR:0	10
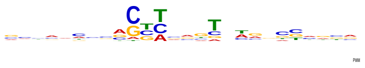	NNNNACCCNNNNNNNNNNGGCCNNNN	1.72	ER:9	11
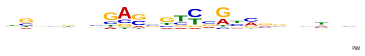	NNNCTGCNNNNNNNNNNNGGCCNNN	1.54	M	12
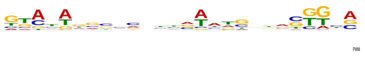	NNNNNGGCCNNNNNNNNGGTANNNNN	1.73	DR:6	13
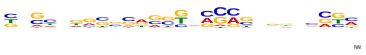	NNNCTGGNNNNNNNNNNNNNGGGCNNN	2.02	M	14
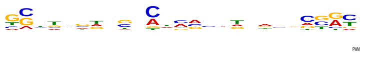	NNNNCGTGNNNNNNNNNCGCGNNNN	1.79	M	15
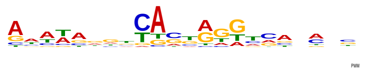	NNNNAGGTNNNNNGGTCANNNN	3.28	DR:2	16
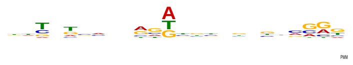	NNNNNTCAGNNNNNNNAGTGNNNNN	1.59	M	17
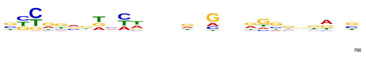	NNNGGAGNNNNNNNNNNNNNNGGGTNNN	1.99	M	18

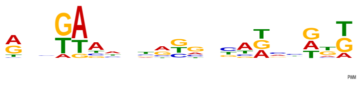	*NNNNNNAGT$\overrightarrow{AGGTCA}$NNNNNN*	3.58	M	19
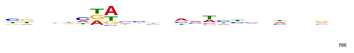	*NNTATTNNNNNNNNNNNNNATATNN*	1.65	M	20

246 RORC:RXRA-Bhimsaria2022-Rnd3 (S13-157)

PWM	Seed Sequence	Seed Seq Enrichment	Repeat	Rank
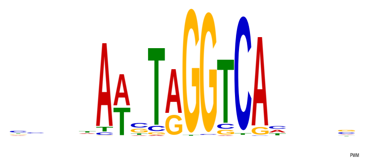	*NNNNNAACTAGGTCANNNNN*	876.27	ER:0	1
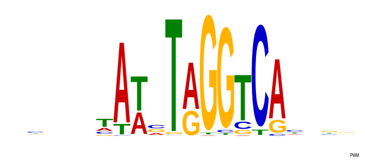	*NNNNNTATATAGGTCNNNNN*	416.32	M	2
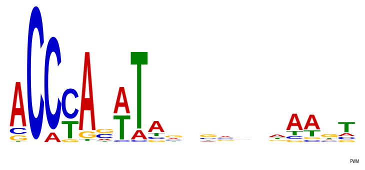	*ACCCACATNNNNNNNNNAAGT*	1255.81	ER:12	3
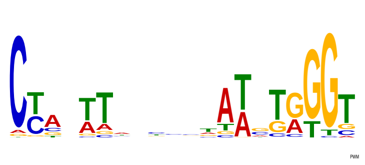	*CCAGTTNNNNNNNNNNTGGGT*	500.25	DR:8	4
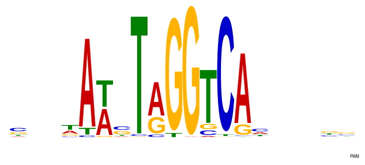	*NNNNAAGTNGGTCACGNNNN*	945.10	M	5
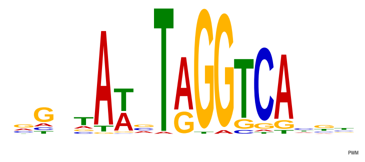	*NNCTATATNGGTCAANN*	624.59	M	6
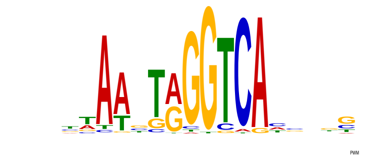	*NNNNTAATNNGGTCATNNNN*	192.98	M	7
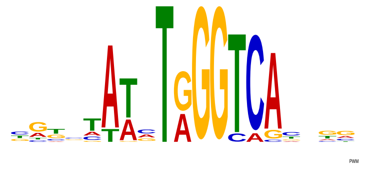	*NNNCTATATNGGTCATNNN*	812.25	M	8
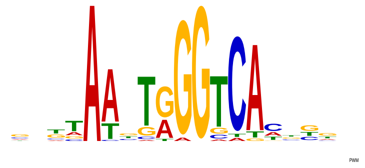	*NNNTAATTNGGTCACTNNN*	916.45	M	9

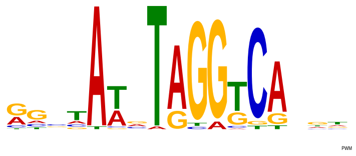	$NNCTATAT\overrightarrow{AGNTCA}ANN$	498.60	M	10
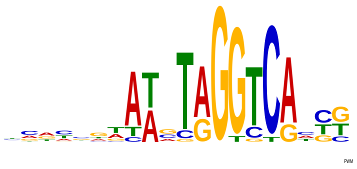	$NNNNC\overleftarrow{GTATCT}\overrightarrow{NGGTCN}NNN$	305.38	ER:0	11
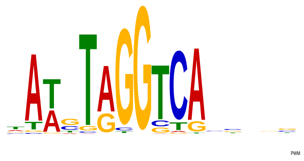	$NNNNAAGT\overrightarrow{ANGTCA}GNNNN$	239.08	M	12
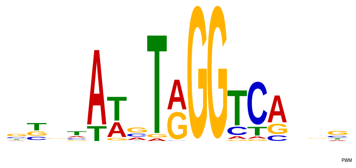	$NNCTATAT\overrightarrow{ANGTCA}TNN$	331.31	M	13
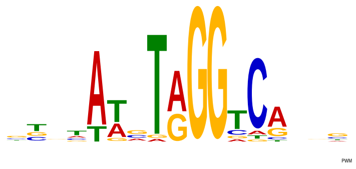	$NNCTATAT\overrightarrow{AGNTCA}TNN$	373.43	M	14

247 RORC-Bhimsaria2022-Rnd2 (S07-048)

PWM	Seed Sequence	Seed Seq Enrichment	Repeat	Rank
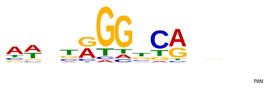	NNNNNAACTNGGTCNNNNN	9.67	ER:0	1
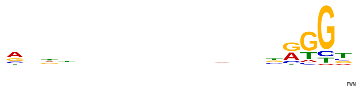	AGTTNNNNNNNNNNNNGGGT	10.77	DR:10	2
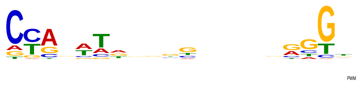	CCAGATNNNNNNNNNNGGGT	30.83	DR:8	3
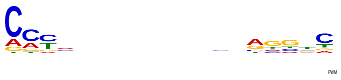	ACCCNNNNNNNNNNNNGGTC	10.02	ER:11	4
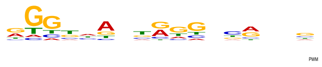	NNNNGTCANNNGGTCNNNN	5.99	DR:2	5
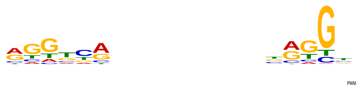	GGGTCNNNNNNNNNNNGGGT	15.72	DR:10	6
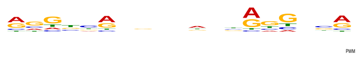	NGGTCNNNNNNNNNGGTCN	3.99	DR:7	7
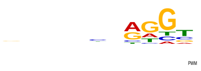	GTCANNNNNNNNNNNNAGGT	3.86	DR:12	8
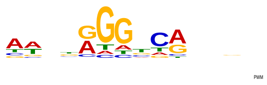	NNNNNAATGNGGTCNNNNN	6.81	M	9

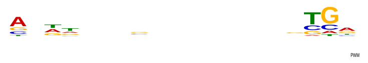	$\overrightarrow{AGTTNN}NNNNNNNN\overrightarrow{NNGTGA}$	9.24	DR:8	10
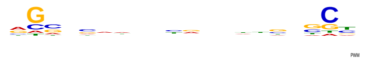	$\overrightarrow{NNGTCA}NNNNNNNN\overleftarrow{TGACNN}$	3.12	IR:8	11
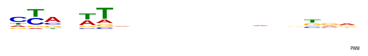	CCAGNNNNNNNNNNNNNGTGG	5.25	M	12
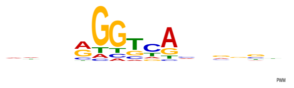	$NNNNNNCC\overrightarrow{AGGTCA}NNNNNN$	7.44	M	13
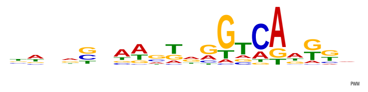	$NNNNNCTAGT\overrightarrow{NGGTCN}NNNN$	4.50	M	14
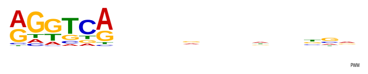	$\overrightarrow{AGGTCN}NNNNNNNNNNNGTGG$	11.00	M	15
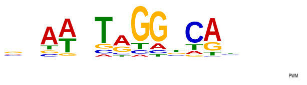	$NNNNNAAGT\overrightarrow{AGGCCN}NNNN$	10.36	M	16
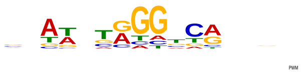	$NNNNNATAT\overrightarrow{NGGTCN}NNNN$	7.11	M	17
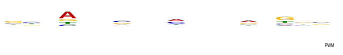	$\overrightarrow{GGTCN}NNNNNNNNN\overrightarrow{NGGTC}$	2.56	DR:9	18

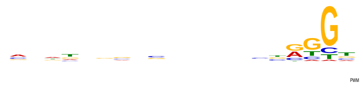	*ATATNNNNNNNNNNNN*$\overrightarrow{GGGT}$	7.78	M	19
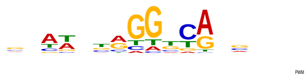	*NNNNNNACT*$\overrightarrow{GGGTC}$*NNNNNN*	6.21	M	20

248 RORC-Bhimsaria2022-Rnd3 (S07-169)

PWM	Seed Sequence	Seed Seq Enrichment	Repeat	Rank
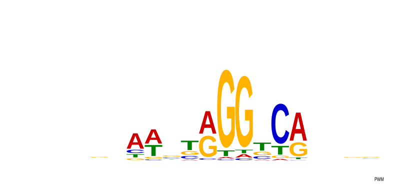	NNNNNAACTNGGTCNNNNN	44.01	ER:0	1
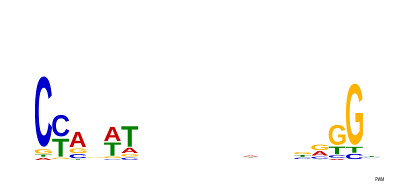	CCAGTTNNNNNNNNNNGGGT	152.17	DR:8	2
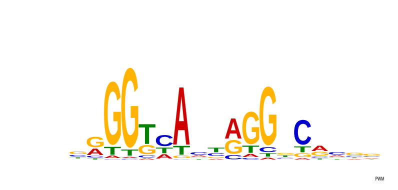	NNNNGGTCACTNGGTCNNNN	312.41	DR:2	3
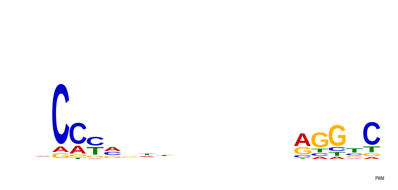	ACCCNNNNNNNNNNNNNGGTC	45.66	ER:11	4
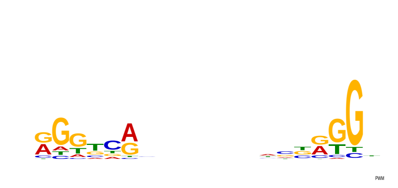	AGGTCNNNNNNNNNNNGGGT	60.07	DR:10	5
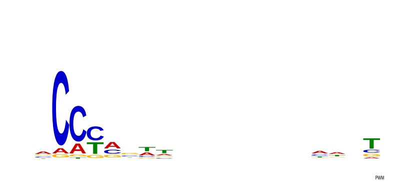	ACCCNNNNNNNNNNNNNAAGT	24.69	ER:12	6
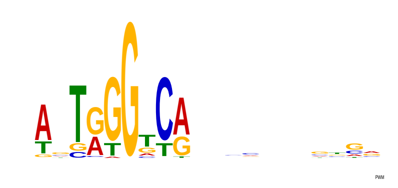	AGTGGGTCNNNNNNNNNGTGG	376.17	M	7
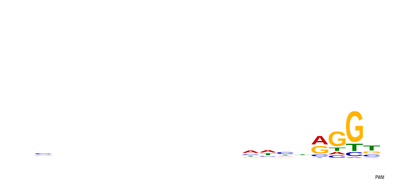	GTCANNNNNNNNNNNNNAGGT	8.49	DR:12	8
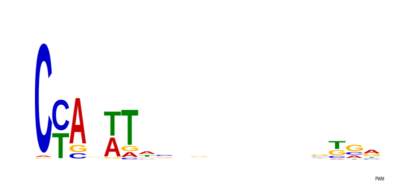	CCAGTTNNNNNNNNNNGTGG	111.50	M	9

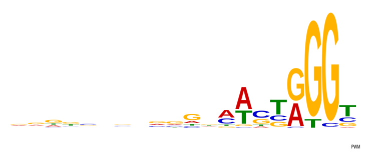	AGGGNNNNNNNNNNACTGGGT	206.43	DR:10	10
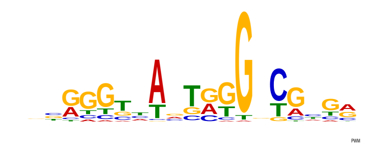	NNNNGGTTAGTGGGTCNNNN	290.78	DR:2	11
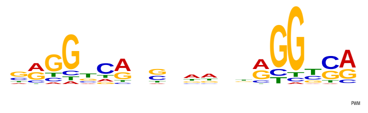	NGGGTCNNNNNNNNNGGTCN	22.34	DR:7	12
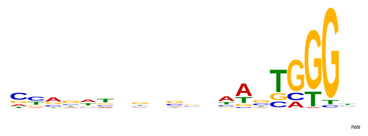	CCAGNNNNNNNNNNAGTGGGT	194.60	M	13
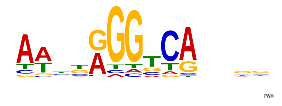	NNNNNAATGNGGTCNNNNN	24.29	M	14
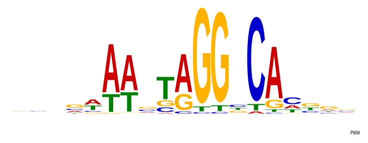	NNNNTATCTAGGCCANNNN	117.49	ER:0	15
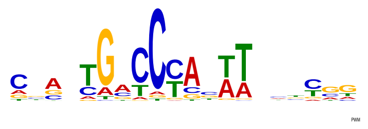	NNNNNGCCCTAGATANNNNN	52.55	ER:0	16
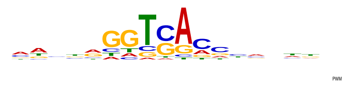	NNNNNAGGTNACCCNNNNN	32.17	M	17
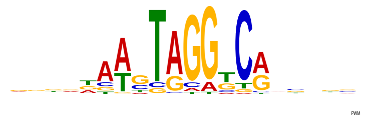	NNNNNAAGTANGGCGNNNNN	30.79	M	18

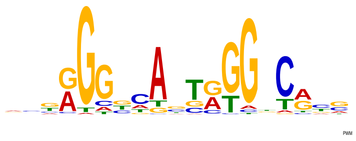	$NNN\overrightarrow{AGGGCA}NT\overrightarrow{GGGTCN}NN$	282.15	DR:2	19
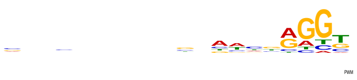	$\overrightarrow{GCCA}NNNNNNNNNNNN\overrightarrow{AGGT}$	10.23	DR:12	20

249 RORC-Yin2017-Rnd2 (ERR1005901)

PWM	Seed Sequence	Seed Seq Enrichment	Repeat	Rank
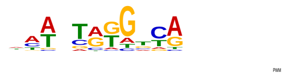	NNNNNNAAGTNNGTCANNNNNNN	10.69	M	1
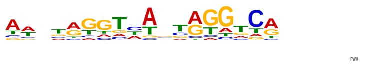	NNNNNNGTTANNNGGTCNNNNNN	7.29	DR:2	2
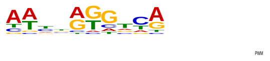	NNNNNNAATGNNGTCANNNNNN	5.86	M	3
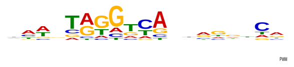	NNNNNNAAGTNNNNCAGTNNNNNN	5.69	M	4
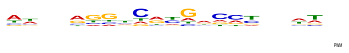	NNNNNNGGTCNNGACCNNNNNN	3.86	IR:0	5
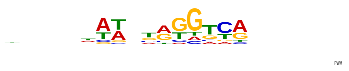	NNNNNNTATCNNNGTCANNNNNN	7.38	M	6
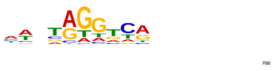	NNNNNNAAGTNNNNCATGNNNNNN	4.36	M	7
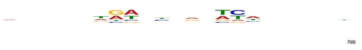	NNNNNNTAACNNNGTTANNNNNN	4.93	M	8
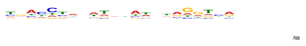	NNNNNNTGACNNNNNNNNNNNNNGTCANNNNNN	3.67	ER:10	9

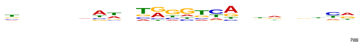	NNNNNNATATNNNNCAGTNNNNNN	3.57	M	10
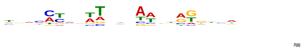	NNNNNNTGACNNNNNNNNNNNNNNGTCANNNNNN	3.22	ER:11	11
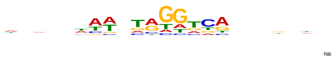	NNNNNNGTATNNNNNTCAANNNNNN	4.28	M	12
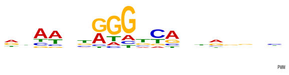	NNNNNNGAATNNNNTCAGNNNNNN	3.44	M	13
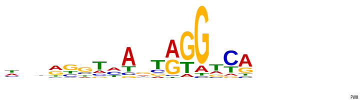	NNNNNNTTAGTNNNTCAGNNNNNN	6.76	M	14
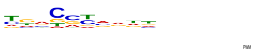	NNNNNNATGANNNCATTNNNNNN	3.72	M	15
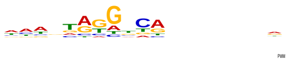	NNNNNNAAGTNNNNCAAANNNNNN	3.82	M	16
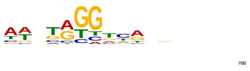	NNNNNNGAATNNNNNTCATNNNNNN	3.72	M	17
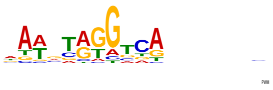	NNNNNNAATGTNNNTCAANNNNNN	8.45	M	18

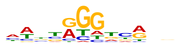	*NNNNNNGGAANNNNNTCACNNNNNN*	3.00	M	19
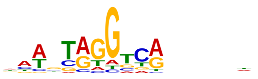	*NNNNNNGAAGT NNNTCACNNNNNN*	6.58	ER:0	20

250 RORC-Yin2017-Rnd3 (ERR1005903)

PWM	Seed Sequence	Seed Seq Enrichment	Repeat	Rank
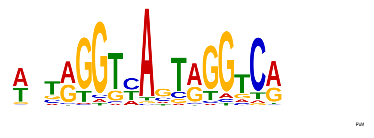	NNNNNNTCAGNNGGTCNNNNNN	20.65	DR:2	1
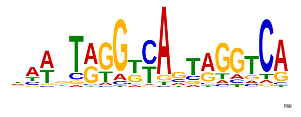	NNNNNNAAGTNNNNCAGTNNNNNN	17.91	M	2
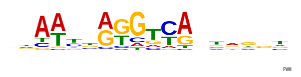	NNNNNNAATGNNGTCANNNNNN	7.63	M	3
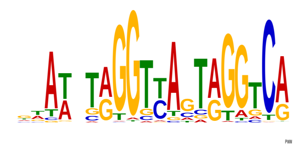	NNNNNNGTATNNNNNTTAGTNNNNNN	19.38	M	4
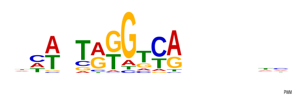	NNNNNNAAGTNNNTCATNNNNNN	8.30	M	5
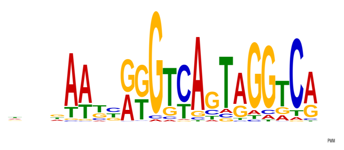	NNNNNNAATCNNNTCAGTANNNNNN	34.39	M	6
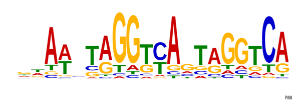	NNNNNNGAATNNNNNNCAGTNNNNNN	8.20	M	7
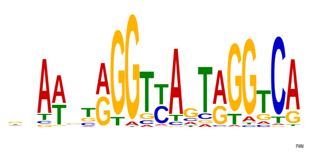	NNNNNNAATTNNNTTAGTNNNNNN	19.94	M	8
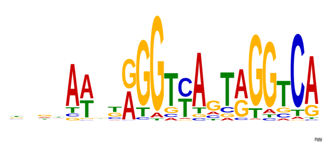	NNNNNNTAATNNNNTTAGTNNNNNN	13.69	M	9

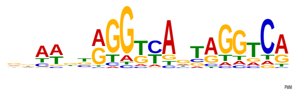	*NNNNNNTAATNNNNNCAGTNNNNNN*	7.22	M	10
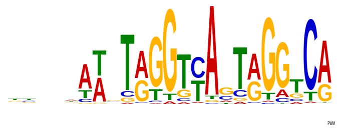	$NNNNNNT\overrightarrow{AAGTNN}NTTAGTNNNNNN$	36.28	M	11
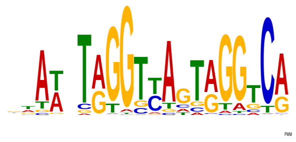	*NNNNNNTATGNNNNTTAGTNNNNNN*	15.57	M	12
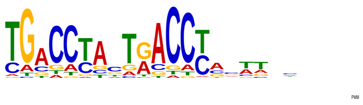	*NNNNNNACTANNNNAATTNNNNNN*	7.07	M	13
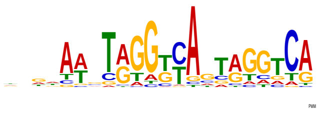	$NNNNNNA\overrightarrow{ATGTNN}NNCAGTNNNNNN$	17.14	M	14
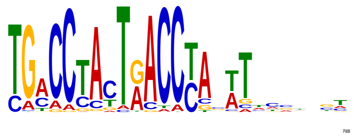	$NNNNNNTACTANNNN\overrightarrow{AGTTTN}NNNNN$	31.12	M	15

251 RORC-Yin2017-Rnd4 (ERR1005904)

PWM	Seed Sequence	Seed Seq Enrichment	Repeat	Rank
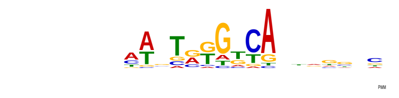	*NNNNNNAAGTNNNTCAGNNNNNN*	8.47	M	1
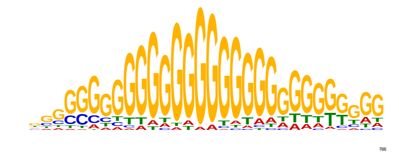	*NNNNNNGGGGGGGGNNGGGGGGGGNNNNNN*	2044.56	M	2
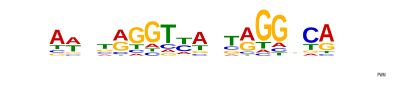	*NNNNNNGGTTNNTAGGNNNNNN*	8.24	DR:2	3
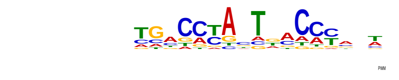	*NNNNNNGTGANNNNCTGANNNNNN*	4.68	IR:5	4
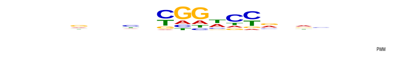	*NNNNNNACGGTCCGNNNNNN*	3.97	M	5
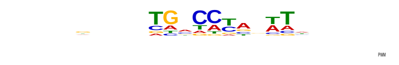	*NNNNNNCATGNNNNAGATNNNNNN*	4.19	M	6
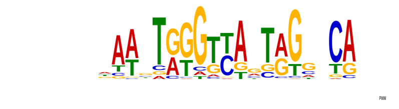	*NNNNNNAAGTNNNTTAGTAGNNNNNN*	32.19	M	7
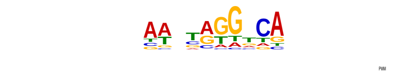	*NNNNNNAAAANNNNNTCAANNNNNN*	4.66	M	8
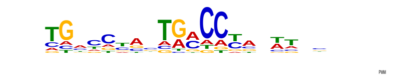	*NNNNNTACTNNNNNAGATNNNNNN*	3.91	M	9

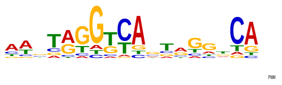	NNNNNNAAGTNNNNNNAGTANNNNNN	5.83	M	10
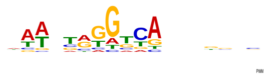	NNNNNNGTATNNNNNTCAGNNNNNN	4.32	M	11
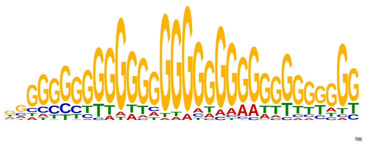	NNNNNNGGGGGGNNNNNGGGGGGGNNNNNN	555.46	M	12
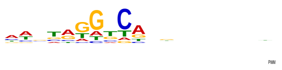	NNNNNNAAGTNNNNNNATGANNNNNN	3.23	IR:4	13
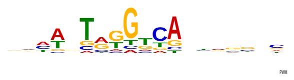	NNNNNNTAAGNNNNTCAGNNNNNN	5.41	M	14
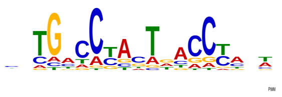	NNNNNNCGTGNNNNACTAANNNNNN	6.38	M	15
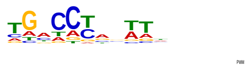	NNNNNNGTTGNNNNAGATNNNNNN	3.36	M	16
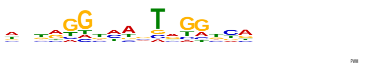	NNNNNNTTAGNNNNTCAGNNNNNN	3.33	M	17
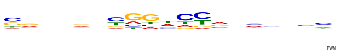	NNNNNNCCGGTCCGNNNNNN	3.14	M	18

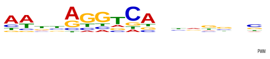	$NNNNNNAATC\overrightarrow{NNNTCA}GNNNNNN$	3.14	M	19
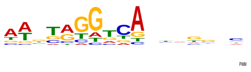	$NNNNNNAGAANN\overrightarrow{NNNTCA}GNNNNNN$	3.36	M	20

252 RORC-Yin2017-Rnd4 (ERR1005905)

PWM	Seed Sequence	Seed Seq Enrichment	Repeat	Rank
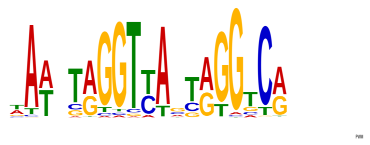	NNNNNNGGTTNNNNGGTCNNNNNN	37.62	DR:2	1
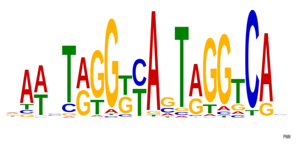	NNNNNNAAGTNNNNNAGTANNNNNN	40.32	M	2
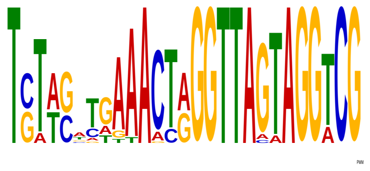	NNNNNATGAAACTNNNNTAGTANNNNN	547.07	M	3
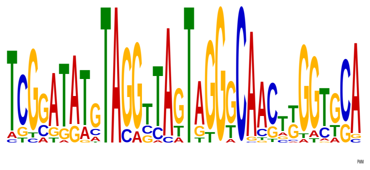	NNNNNNATGTANNNNNNNNNNNCAAGTTNNNNNN	63.03	DR:12	4
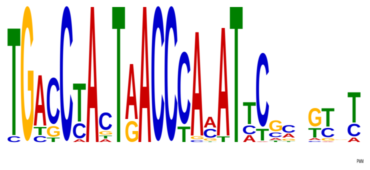	NNNNNNACTAANNNAAATTCGNNNNNN	200.40	M	5
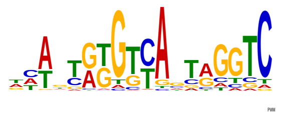	NNNNNNTAAGNNNNTCAGNNNNNN	18.79	M	6
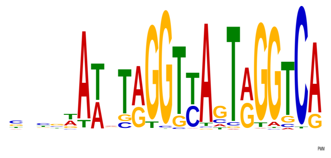	NNNNNNGTATNNNNNTTAGTNNNNNN	40.46	M	7
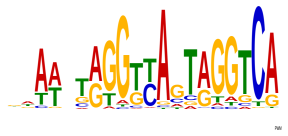	NNNNNNGTATNNNNNNCAGTNNNNNN	16.68	M	8
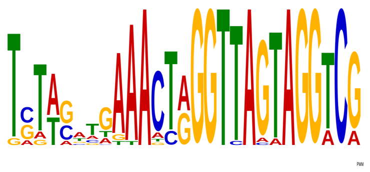	NNNNNATGAAACNNNNNTAGTANNNNN	193.64	M	9

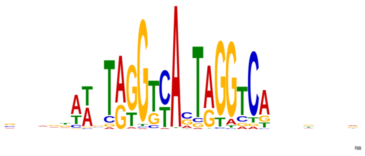	$NNNNNN\overrightarrow{AAGTN}NNNNNNNNNNNCAAGNNNNNN$	11.65	M	10
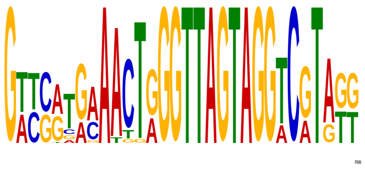	$NNNN\overrightarrow{ATGAAA}CTNNNNNNNNN\overrightarrow{NNGTCG}NNNN$	138.76	DR:10	11
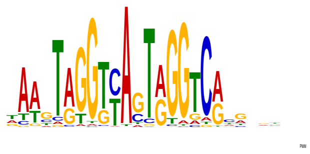	$NNNNNN\overrightarrow{AAGTN}NNNNNNNNNNTCGCNNNNNN$	14.48	M	12
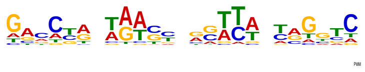	$NNNNNNCTAANNNNNTTAGNNNNNN$	11.23	M	13
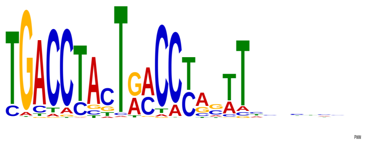	$NNNNNNACTGNNNNNNTTTTNNNNNN$	14.87	M	14
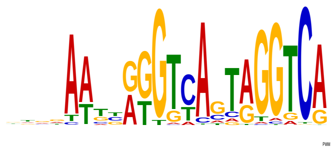	$NNNNNNAATC\overrightarrow{NNNTCA}GTNNNNNN$	24.38	M	15

253 RORC-SMiLE-seq-Rnd1 (SRR3405081)

PWM	Seed Sequence	Seed Seq Enrichment	Repeat	Rank
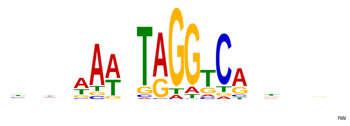	NNNNNNAAGTAGGTCANNNNNNN	42.45	M	1
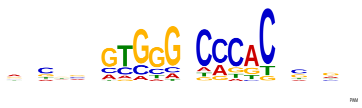	NNNNNNGTGGGNCCCANNNNNNN	6.81	M	2
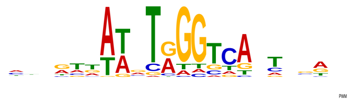	NNNNNNTATATNGGTCANNNNNNN	20.35	M	3
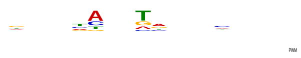	NNNNNNCCTANNTAGGNNNNNNN	3.35	ER:4	4
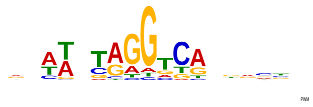	NNNNNNTCTAGGTCANNNNNNN	10.35	M	5
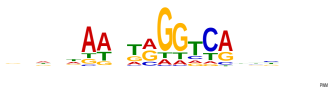	NNNNNNTAATNNGGTCANNNNNNN	9.19	M	6
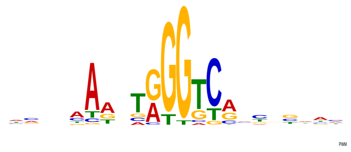	NNNNNNAATTNGGTCAANNNNNNN	16.96	M	7
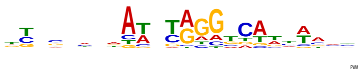	NNNNNNAGAANNNGGTCANNNNNNN	5.98	M	8
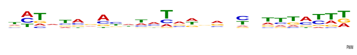	NNNTATANNNNNNNNNNNNNNNTTTANNN	2.68	M	9

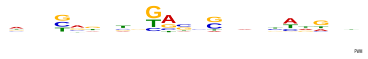	NNNNNGCTGNNNNNNCACTNNNNN	1.68	M	10
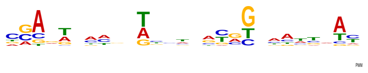	NNNGTTANNNNNNNNNNNNNNAATTNNN	2.32	M	11
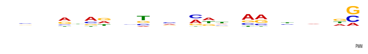	NNNTATANNNNNNNNNNNNNNTTCANNN	2.13	M	12
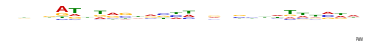	NNNNATTTNNNNNNNNNNNNNTTTTNNNN	2.41	M	13
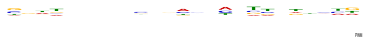	NNTTTTNNNNNNNNNNNNNGTTCNN	1.66	M	14
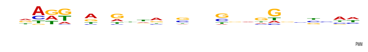	NNNNGTAGNNNNNNNNNNNGGGCNNNN	1.74	M	15
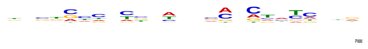	NNNTCCCNNNNNNNNNNNNTATCNNN	2.08	M	16
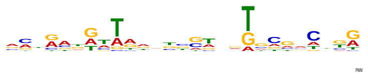	NNNNATGTNNNNNNNNNNNNGCGANNNN	2.18	M	17
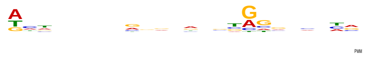	NNTAGGNNNNNNNNNNNNGTCANN	1.78	DR:7	18

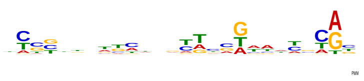	*NNNNTTCTNNNNNNNNNNAAATNNNN*	1.73	M	19
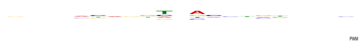	$NNNNNN\overrightarrow{AGGTNA}CCTNNNNNN$	2.46	M	20

254 RORC-16-Bhimsaria2022-Rnd2 (S07-076)

PWM	Seed Sequence	Seed Seq Enrichment	Repeat	Rank
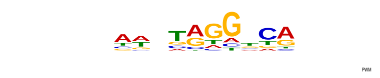	NNNNNATGTNGGTCNNNNN	5.34	M	1
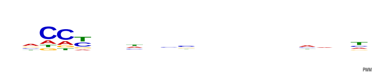	ACCTNNNNNNNNNNNNAAGT	5.21	ER:12	2
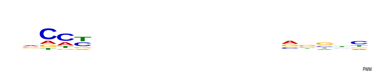	ACCTNNNNNNNNNNNNGGTC	5.05	ER:11	3
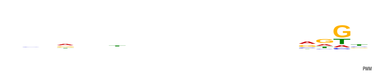	CCAGNNNNNNNNNNNNAGGT	4.24	M	4
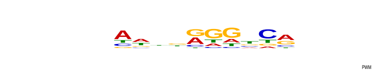	NNNNNAATGNGGTCNNNNN	3.97	M	5
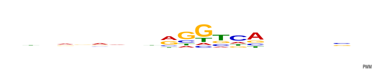	NNNNNNCCAGGTCANNNNNN	3.58	M	6
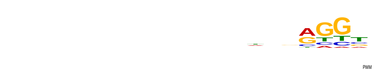	GGGGNNNNNNNNNNNNAGGT	3.85	M	7
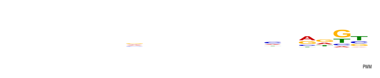	GTCANNNNNNNNNNNNAGGT	2.68	DR:12	8
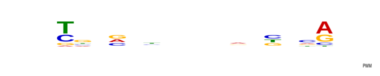	NNNNNNCTATATAGNNNNNN	2.24	M	9

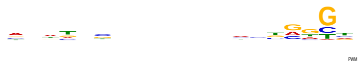	$ATATNNNNNNNNNNNNT\overrightarrow{GGGT}$	6.32	M	10
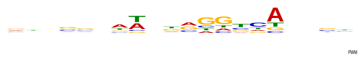	$NNNNNAATCN\overrightarrow{NGGTCN}NNNN$	3.76	M	11
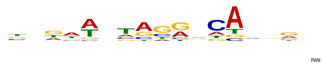	$NNNNN\overrightarrow{AAGTAG}AGCNNNNN$	3.37	M	12
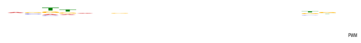	$\overrightarrow{AGTTNN}NNNNNNNNNNNATGG$	2.75	M	13
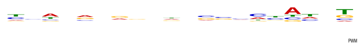	$CCAANNNNNNNNNNNNNATAT$	2.70	M	14
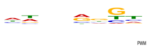	$CATTNNNNNNNNNNNNN\overrightarrow{GGGT}$	3.15	M	15
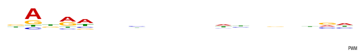	$NNNNNNCTCT\overrightarrow{AGATNN}NNNN$	1.60	M	16
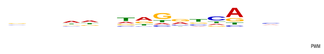	$NNNNN\overrightarrow{AAGTNG}GCCNNNNN$	2.82	M	17
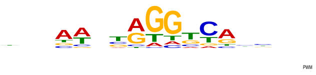	$NNN\overleftarrow{NNAACT}\overrightarrow{GGGTCN}NNNN$	7.60	ER:0	18

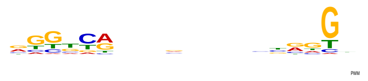	$\overrightarrow{GGGTCA}NNNNNNNNNN\overrightarrow{GGGT}$	9.54	DR:10	19
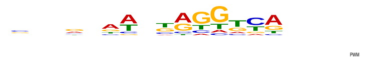	$NNN\overleftarrow{NNATCT}\overrightarrow{GGGTCN}NNNNN$	5.63	ER:0	20

255 RORC-16-Bhimsaria2022-Rnd3 (S07-101)

PWM	Seed Sequence	Seed Seq Enrichment	Repeat	Rank
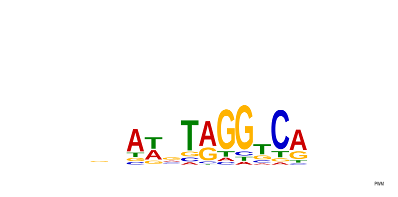	NNNNNATGTNGGTCNNNNN	25.72	M	1
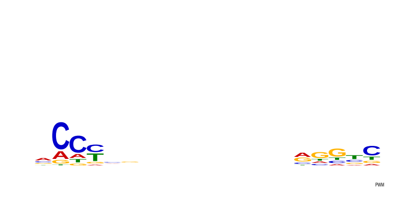	ACCTNNNNNNNNNNNNGGTC	21.33	ER:11	2
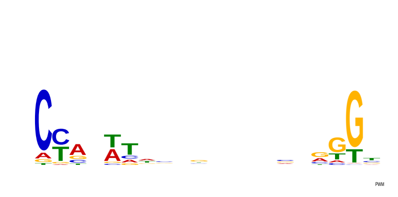	CCAGTTNNNNNNNNNNGGGT	59.44	DR:8	3
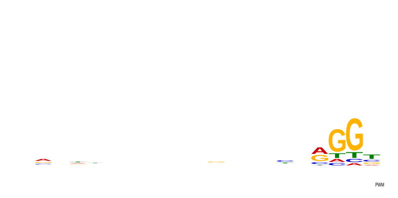	AGTTNNNNNNNNNNNNAGGT	15.61	DR:10	4
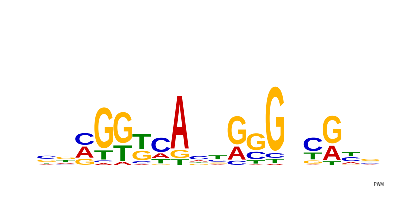	NNNGGTCACTGGGTCNNN	150.93	DR:2	5
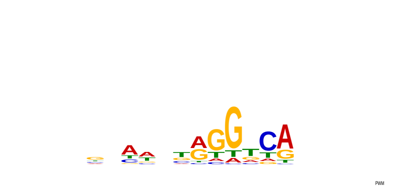	NNNNNNACTGGGTCNNNNNN	12.51	M	6
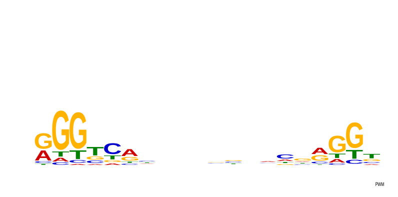	GGGTCANNNNNNNNNNAGGT	49.57	DR:10	7
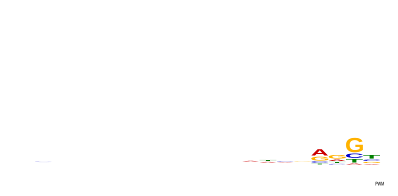	GTCANNNNNNNNNNNNAGGT	6.18	DR:12	8
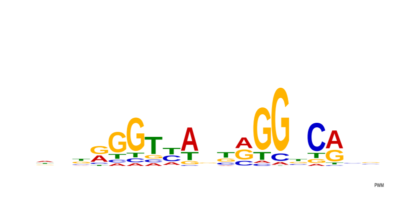	NNNGGGTTANNNGGTCNNN	40.28	DR:2	9

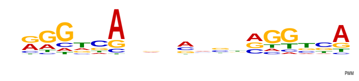	NGGGTCNNNNNNNNNNGGTCN	11.41	DR:7	10
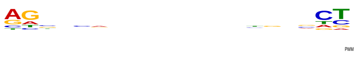	NNGTCANNNNNNNNNTGACNN	5.61	IR:8	11
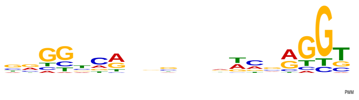	GGGGTCNNNNNNNNNNAGGT	36.29	DR:9	12
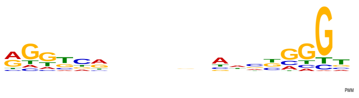	AGGTCNNNNNNNNNNTGGGT	45.78	DR:10	13
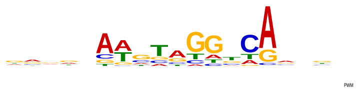	NNNNNAACTAGGCCNNNNN	14.88	ER:0	14
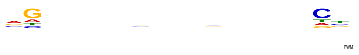	NNGTCANNNNNNNNTGACNN	3.93	IR:7	15
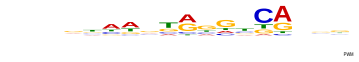	NNNNCTAGTNGGTCNNNN	7.99	M	16
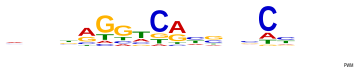	NNNNNGGTCATGACNNNNN	18.26	IR:0	17
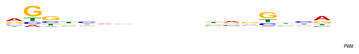	NNGTCANNNNNNNGGTCNN	6.31	DR:6	18

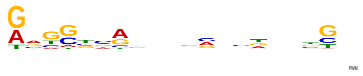	$N\overrightarrow{AGGTCN}NNNNNNNN\overrightarrow{GGGTN}$	5.75	DR:8	19
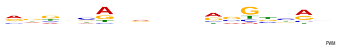	$N\overrightarrow{NGGTCN}NNNNN\overrightarrow{NGGTCN}N$	6.18	DR:5	20

256 TR2-Bhimsaria2022-Rnd3 (S07-107)

PWM	Seed Sequence	Seed Seq Enrichment	Repeat	Rank
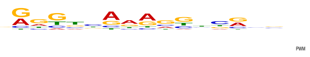	NNNNGGTCNNNGGTCNNNN	6.92	DR:1	1
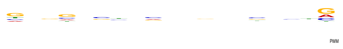	CGTGNNNNNNNNNNNNACTG	4.68	M	2
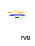	GGGGNNNNNNNNNNNNGGGG	6.06	M	3
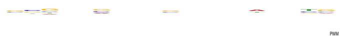	CGCGNNNNNNNNNNNNAGTG	4.56	M	4
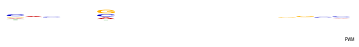	CACTNNNNNNNNNNNGGCC	4.72	M	5
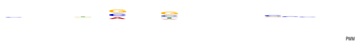	CGTANNNNNNNNNNNNCCGG	3.92	M	6
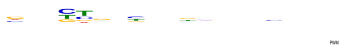	CGGANNNNNNNNNNNNCTCG	3.69	M	7
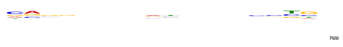	NCAGGNNNNNNNNNNCCTGN	5.44	IR:4	8

257 TR2-Yin2017-Rnd4 (ERR1005192)

PWM	Seed Sequence	Seed Seq Enrichment	Repeat	Rank
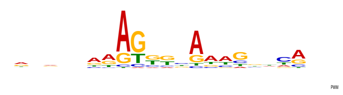	*NNNNNNGAAANNNNAAAGNNNNNN*	7.24	M	1
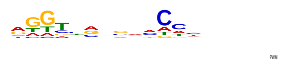	*NNNNNNAGGTNNNNAACCNNNNNN*	5.38	IR:1	2
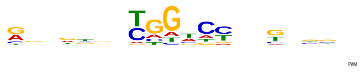	*NNNNNNATGGTCCGNNNNNN*	4.58	M	3
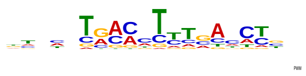	*NNNNNNGCTTNNNNTTTGNNNNNN*	4.48	M	4
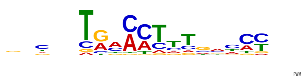	*NNNNNNCGATNNNNTTTGNNNNNN*	5.36	M	5
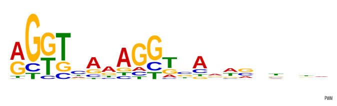	*NNNNNNCAAANNNGAATNNNNNN*	4.85	M	6
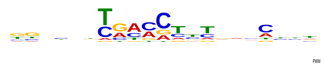	*NNNNNNCAGCNNNNTTTGNNNNNN*	2.83	M	7
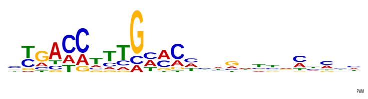	*NNNNNNTTTGNNNNNNGATTNNNNNN*	3.67	M	8
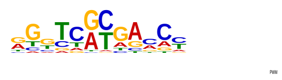	*NNNNNNAGGTNNNNACCTNNNNNN*	4.24	IR:0	9

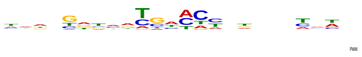	*NNNNNNTCATNNNNTTTTNNNNNN*	2.95	M	10
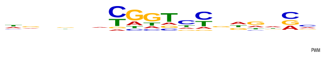	*NNNNNNTACGGTCCNNNNNN*	3.34	M	11
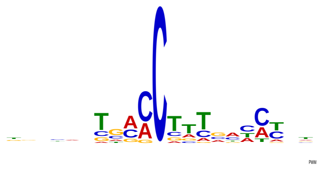	*NNNNNNTACTNNNNTTTGNNNNNN*	4.36	M	12
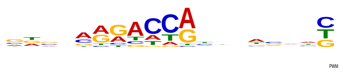	*NNNNNNAGACCACTNNNNNN*	4.25	M	13
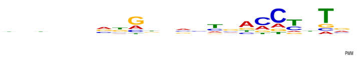	*NNNNNNATGTNNCTGANNNNNN*	2.93	IR:1	14
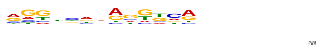	*NNNNNNTCAANNNNCGTGNNNNNN*	2.66	M	15
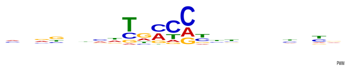	*NNNNNNTCATNNNNTTTCNNNNNN*	3.11	M	16
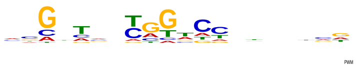	*NNNNNNGTGGTCCANNNNNN*	3.21	M	17
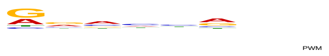	*AGACCAAC*	2.07	M	18

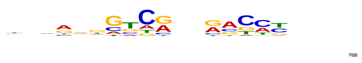	$NNNN\overrightarrow{NNGTCG}NCAACNNNNNN$	3.42	M	19
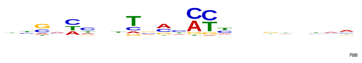	$NNNNNNTTTGNNNCTGGNNNNNN$	2.61	M	20

258 TR4-Bhimsaria2022-Rnd2 (S07-083)

PWM	Seed Sequence	Seed Seq Enrichment	Repeat	Rank
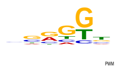	GGGGNNNNNNNNNNNNGGGT	6.88	M	1
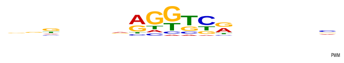	NNNNNNAAAGGTCGNNNNNN	5.33	M	2
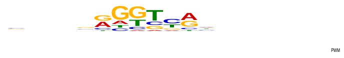	NNNNNNGGGTCACGNNNNNN	5.71	M	3
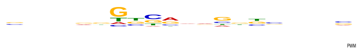	NNNNNNGTCAAAGGNNNNNN	4.45	DR:1	4
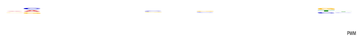	ACCCNNNNNNNNNNNNGGGT	3.90	ER:12	5
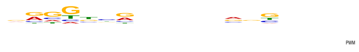	NNGGTCNNNNNNNGGTCNN	3.91	DR:5	6
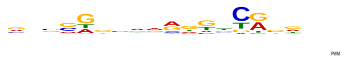	NNNNNNCTAAAGGTNNNNNN	1.84	M	7
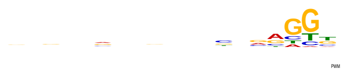	GGGGNNNNNNNNNNNAAGGT	6.80	M	8
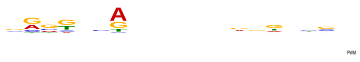	NNGGTCNNNNNNNNGGTCNN	3.20	DR:6	9

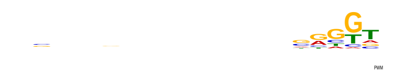	$CCTANNNNNNNNNNNNN\overrightarrow{GGGT}$	3.37	M	10
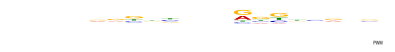	$NNN\overrightarrow{GGGTN}NNN\overrightarrow{NGGTCN}NN$	3.72	DR:2	11
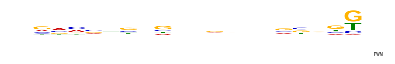	$\overleftarrow{GACCT}NNNNNNNNNNNN\overrightarrow{GGGT}$	6.31	ER:11	12
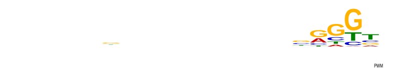	$CCCGNNNNNNNNNNNNN\overrightarrow{GGGT}$	4.34	M	13
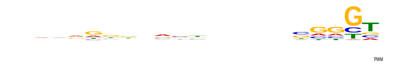	$AAAGNNNNNNNNNNNNC\overrightarrow{GGGT}$	6.25	M	14
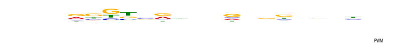	$NN\overrightarrow{NGGTCN}NNNN\overrightarrow{NGGTCN}NN$	3.68	DR:4	15
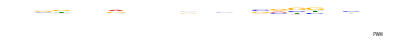	$\overrightarrow{GGTCN}NNNNNNNNN\overrightarrow{NNGTCA}$	2.04	DR:8	16
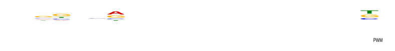	$\overrightarrow{GGTCN}NNNNNNNNNN\overrightarrow{NGGGC}$	2.11	DR:9	17
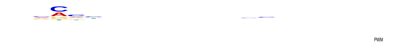	$\overleftarrow{ACCT}NNNNNNNNNNN\overrightarrow{NGGTC}$	3.64	ER:11	18

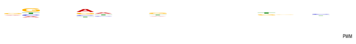	$\overrightarrow{GGTCN}NNNNNNNN\overrightarrow{NNGGCA}$	1.80	DR:8	19
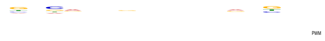	$\overrightarrow{AGGTN}NNNNNNNNN\overrightarrow{NGGTC}$	2.42	DR:8	20

259 TR4-Bhimsaria2022-Rnd3 (S07-108)

PWM	Seed Sequence	Seed Seq Enrichment	Repeat	Rank
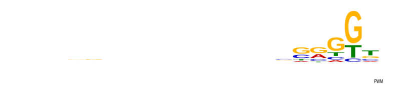	GGGGNNNNNNNNNNNNGGGT	29.16	M	1
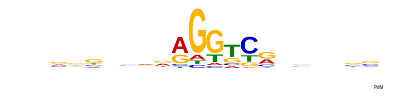	NNNNNNAAAGGTCGNNNNNN	18.22	M	2
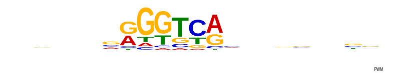	NNNNNNGGTCACGGNNNNNN	20.71	M	3
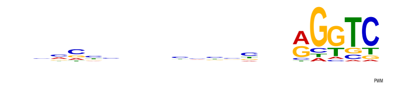	CCCCNNNNNNNNNNNAGGTC	32.87	M	4
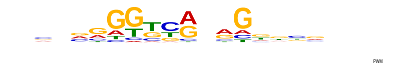	NNNNGGTCNNAGTGCNNNN	23.50	DR:1	5
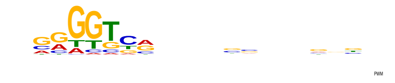	GGGGTCNNNNNNNNNNGGGG	48.75	M	6
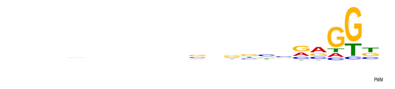	GGGGNNNNNNNNNNNAAGGT	24.93	M	7
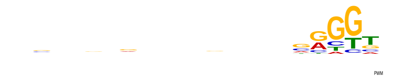	CCCANNNNNNNNNNNNGGGT	11.51	M	8
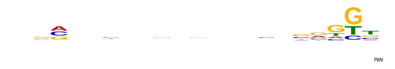	AACCNNNNNNNNNNNNGGGT	11.69	ER:11	9

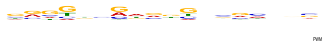	$NNN\overrightarrow{NGGTCN}NN\overrightarrow{NNGTCA}NNNN$	10.68	DR:1	10
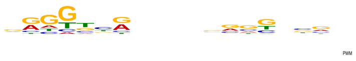	$N\overrightarrow{NGGTCN}NNNNN\overrightarrow{NGGTCN}N$	13.00	DR:5	11

260 TR4-Jolma2013-Rnd2 (ERR195698)

PWM	Seed Sequence	Seed Seq Enrichment	Repeat	Rank
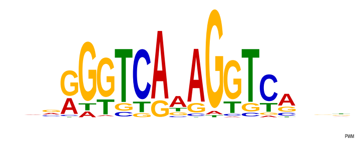	NNNNGGTCNNAGGTCNNNN	90.41	DR:1	1
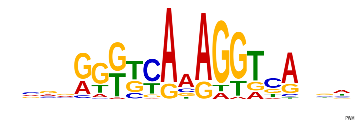	NNNNAGGTCNNNGGTCNNNN	28.71	DR:1	2
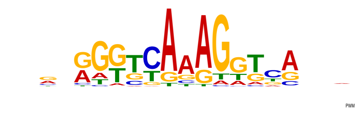	NNNNAGGTNNNNGGTCNNNN	11.85	DR:1	3
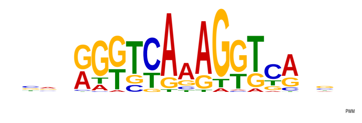	NNNNAGGTNNNAGGTCNNNN	32.90	DR:1	4
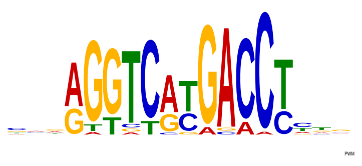	NNNAGGTCANGACCTNNN	137.73	IR:0	5
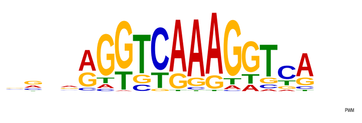	NNNAAGGTNNNNGGTCNNN	21.54	DR:1	6
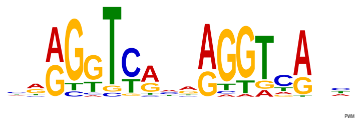	NNNGGTCANNNGGTCNNN	15.21	DR:2	7
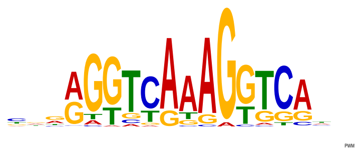	NNNAGGTCNNAGTTCNNN	54.34	DR:1	8
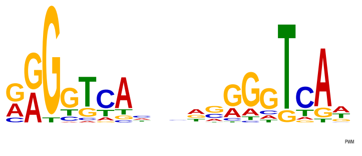	NNGGTCANNNNNNGGTCNN	14.04	DR:5	9

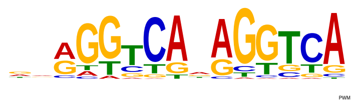	$NT\overrightarrow{AGGTCN}N\overrightarrow{NGGTCN}$	31.90	DR:1	10
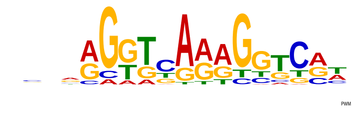	$NNAG\overrightarrow{AGGNNN}N\overrightarrow{NGGTCN}N$	11.65	DR:1	11
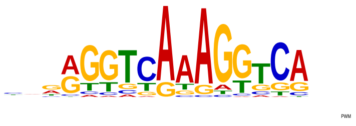	$NNN\overrightarrow{AGGTCN}N\overrightarrow{NGTTCN}NN$	16.65	DR:1	12
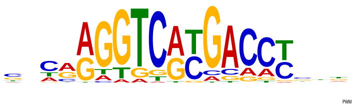	$NNNN\overrightarrow{GGGTCN}\overleftarrow{NGACC}NNNN$	31.18	IR:0	13
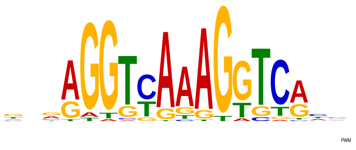	$NNN\overrightarrow{AGGTTA}N\overrightarrow{NGGTCN}NN$	52.10	DR:1	14
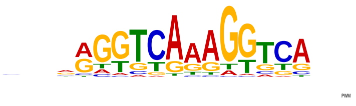	$NNNN\overrightarrow{AGGTNN}N\overrightarrow{AGGTNN}NN$	15.61	DR:1	15
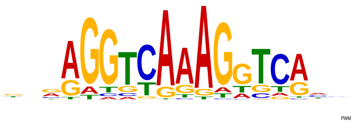	$NNN\overrightarrow{AGGTTN}N\overrightarrow{NGGTCN}NN$	16.62	DR:1	16
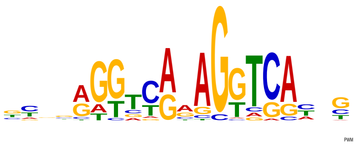	$NNNN\overrightarrow{AGGTNN}N\overrightarrow{AGTTCN}NNN$	15.43	DR:1	17
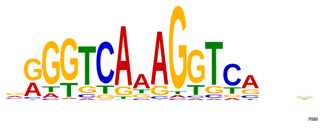	$NNN\overrightarrow{NGGTCN}N\overrightarrow{NGGTCN}NNN$	30.31	DR:1	18

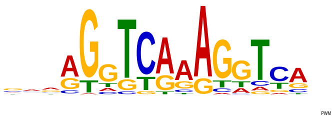	$NNAA\overrightarrow{AGGNNN}N\overrightarrow{NGGTCN}N$	13.69	DR:1	19
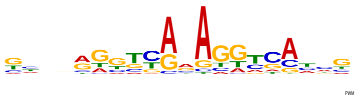	$NNNN\overrightarrow{AGGTNN}N\overrightarrow{NGTTCN}NNN$	4.98	DR:1	20

261 TR4-Jolma2013-Rnd3 (ERR195699)

PWM	Seed Sequence	Seed Seq Enrichment	Repeat	Rank
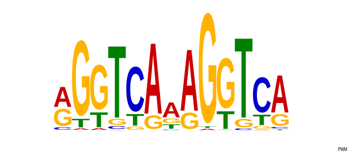	NNNNGGTCNNNGGTCNNNN	163.85	DR:1	1
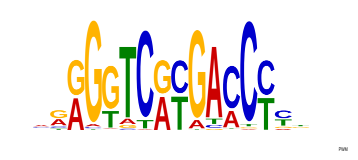	NNNNNGGTCNNGACCNNNNN	48.53	IR:0	2
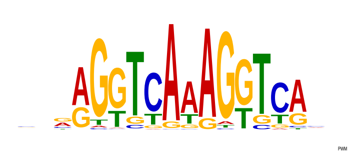	NNNAAGGNNNNNGGTCNNN	50.14	DR:1	3
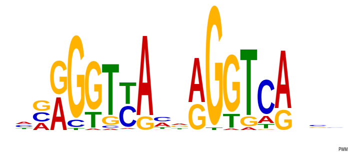	NNNNGGTCNNNNGGTCNNNN	25.45	DR:2	4
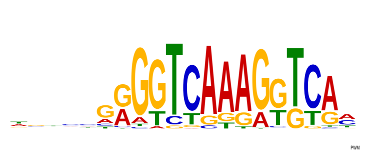	NNNNCGGGNNNNAAGGNNNN	34.52	M	5
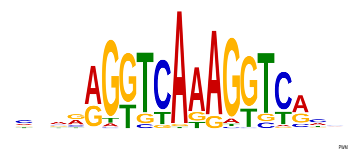	NNNAAAGNNNNNNGGTCNNN	32.50	M	6
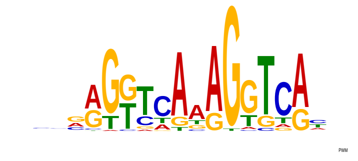	NNNAGGGNNNNNNGGTCNNN	24.91	DR:3	7
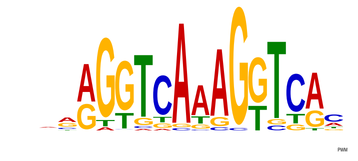	NNTAAGGNNNNNGGTCNN	48.85	DR:1	8
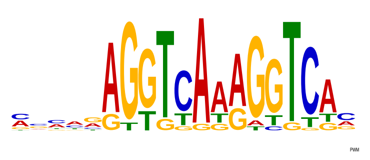	NNAAGAGGNNNNNGGTCNN	104.44	DR:1	9

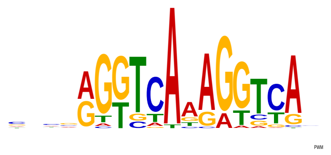	NNNNAGGGNNNNNAAGGNNNN	23.56	DR:3	10
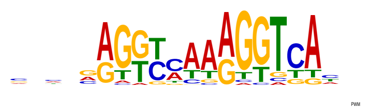	NNNAAGGNNNNNNNGGTCNNN	18.05	DR:2	11
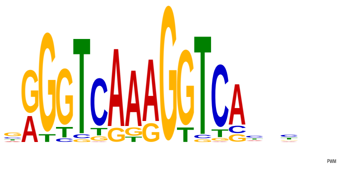	NNNGGTCNNNNNNCATGNNN	31.74	M	12
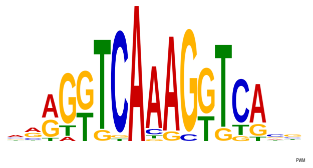	NNNTAAGNNNNNNGGTCNNN	13.84	M	13
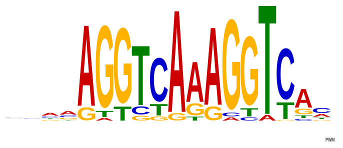	NNGAAAGNNNNNNGGTCNN	40.85	M	14
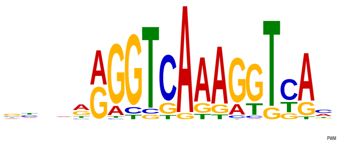	NNNNTAAGNNNNNAAGGNNNN	20.81	M	15
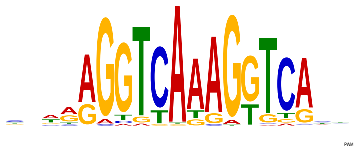	NNNAAGGNNNNNGTTCNNN	22.03	DR:1	16
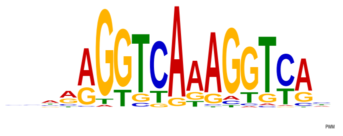	NNNAAAGNNNNNAGGTNNN	36.73	M	17
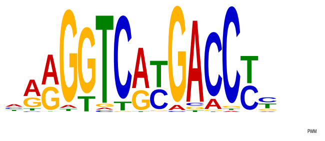	NNNNAGGTNNNGACCNNNN	35.38	IR:0	18

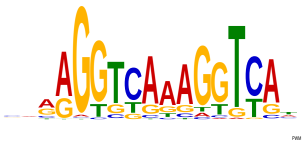	*NNNNAAAGNNNNA*$\overrightarrow{AGGNNN}$*N*	33.92	M	19
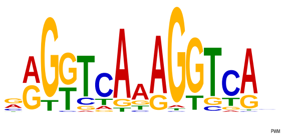	*NNN*$\overrightarrow{AGGGNN}$*NNN*$\overrightarrow{AGGTNN}$*N*	24.55	DR:3	20

262 TR4-Jolma2013-Rnd4 (ERR195700)

PWM	Seed Sequence	Seed Seq Enrichment	Repeat	Rank
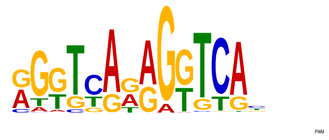	NNNGGTCNNNNNTCACNNN	26.09	DR:1	1
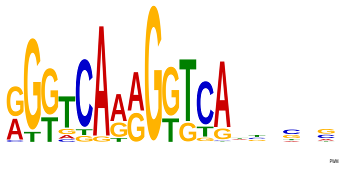	NNNNTCAANNNNNCAATNNNN	23.54	M	2
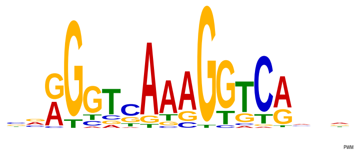	NGGGGNNNNNNNNCACGN	8.53	M	3
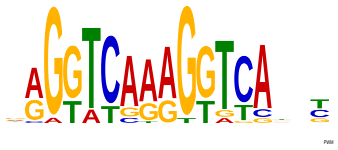	NGAGGNNNNNNNNCATCN	9.12	M	4
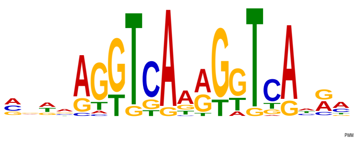	NNAAAGNNNNNNNNTCACNN	8.03	M	5
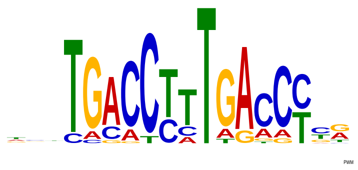	NGTTGNNNNNNNNCCCCN	8.71	M	6
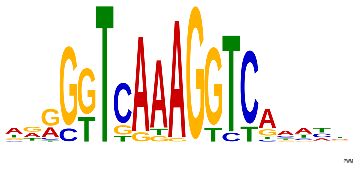	NGGGGNNNNNNNNNAAATN	6.23	M	7
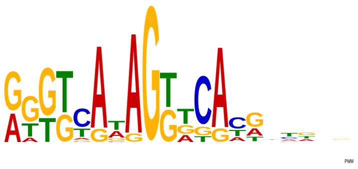	NNNNCAAANNNNACGTNNNN	12.38	M	8
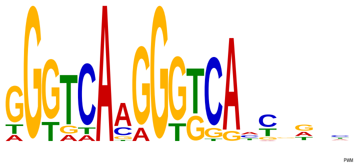	NNNTCAANNNNCAACGNNN	27.12	M	9

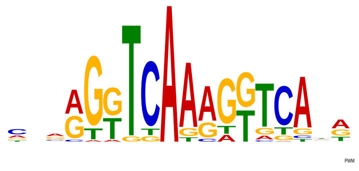	*NAGAGNNNNNNNNTCACN*	7.90	M	10
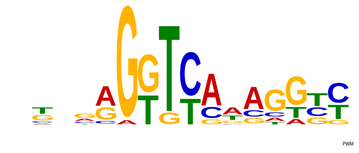	*NNAGAGNNNNTAGGNN*	6.29	M	11
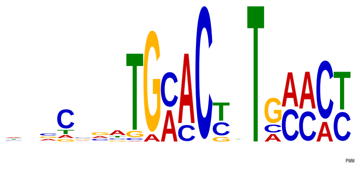	*NNNNCGATNNNNTTTGNNNN*	10.93	M	12
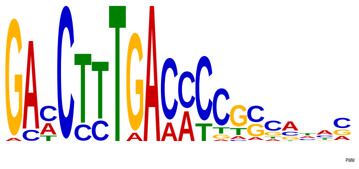	*NNNNTTTGNNNNCGCCNNNN*	14.02	M	13
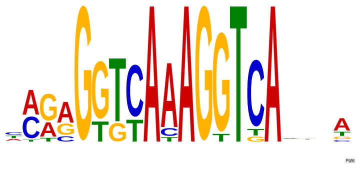	*NNGAGGNNNNNNNNCAATNN*	13.11	M	14
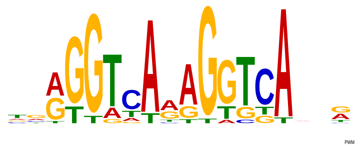	*NGAGGNNNNNNNNCACTN*	7.21	M	15
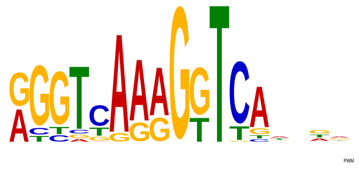	*NNGTTANNNNNNCAACNN*	6.02	M	16
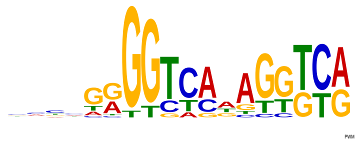	*NNNCGGGNNNNGAGGNNN*	7.28	M	17
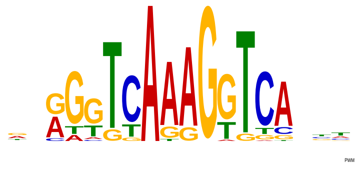	*NAAGGNNNNNNNNCAACN*	6.24	M	18

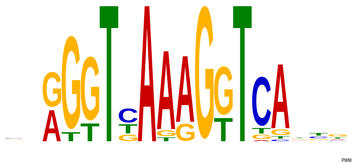	$NG\overrightarrow{AGGNNN}NNNNNCAACN$	7.61	M	19

263 LRH1:RXRA-Bhimsaria2022-Rnd3 (S13-140)

PWM	Seed Sequence	Seed Seq Enrichment	Repeat	Rank
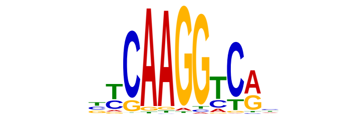	NNNNNNTCAAGGTCNNNNNN	103.70	DR:0	1
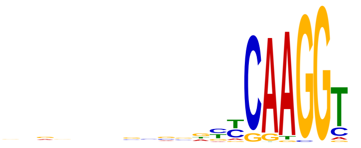	GGGGNNNNNNNNNNCAAGGT	282.81	M	2
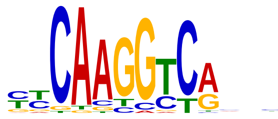	NNTCAAGGNNACAGNN	33.31	DR:0	3
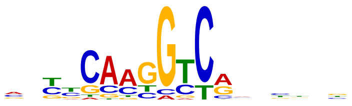	NNNTCAAGNNNACAGNNN	10.69	DR:0	4
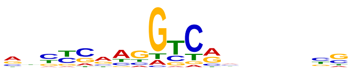	NNNNCAAGNNNACAGNNNN	6.14	M	5
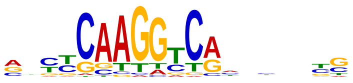	NNNNCAAGGNNACAGNNNN	17.49	M	6
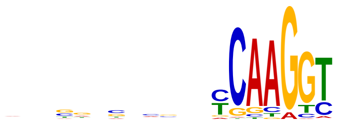	AATGNNNNNNNNNNNAAGGT	46.31	M	7
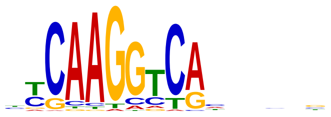	NNNCAAGGTNACAGNNN	51.69	M	8
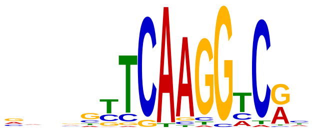	NNNNGTTCAAGGACNNNN	80.36	DR:0	9

264 LRH1-Bhimsaria2022-Rnd2 (S07-013)

PWM	Seed Sequence	Seed Seq Enrichment	Repeat	Rank
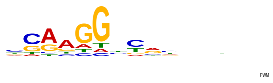	*NNNNNNCAAGGTCANNNNNN*	6.69	M	1
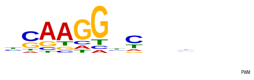	*NNNNNNTCAAGGTCNNNNNN*	7.61	DR:0	2
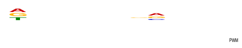	*TGGGNNNNNNNNNNNNAAGG*	1.46	M	3
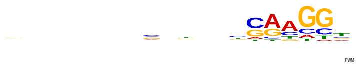	*GGTGNNNNNNNNNNNAAGGT*	5.34	DR:11	4
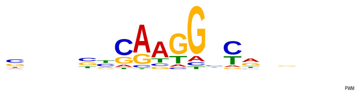	*NNNNNTCAAGGCCANNNNN*	9.89	DR:0	5
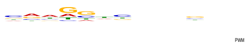	*NNNNNGCAANGTCANNNNN*	2.62	M	6
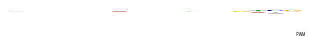	*CCGCNNNNNNNNNNNNGTCG*	1.87	M	7
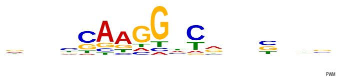	*NNNNNCAAGGTCGTNNNNN*	7.49	M	8
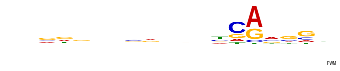	*CACGNNNNNNNNNNNNAGGT*	2.31	M	9

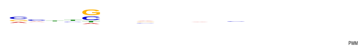	*CCTTNNNNNNNNNNNNGGCC*	2.39	M	10
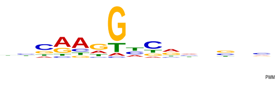	*NNNNNGCAAGGTCANNNNNN*	4.50	M	11
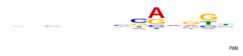	*AGTANNNNNNNNNNNNNAGGT*	2.11	M	12
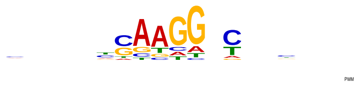	*NNNNNTCAAGGTCGNNNNNN*	9.45	DR:0	13
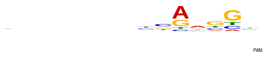	*AGGTNNNNNNNNNNNNNAGGT*	2.10	DR:10	14
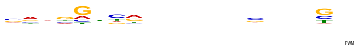	*CAAGNNNNNNNNNNNNNGGGT*	2.30	M	15
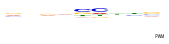	*CCACNNNNNNNNNNNNNCTTG*	2.30	M	16
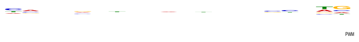	*CAGANNNNNNNNNNNNNCTTG*	2.15	M	17
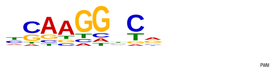	*NNNNNNCAAGGTCGNNNNNNN*	6.58	M	18

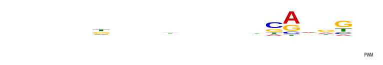	GAGTNNNNNNNNNNNN$\overrightarrow{AGGT}$	2.04	M	19
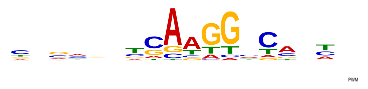	NNNN$\overrightarrow{GTTCA}\overrightarrow{AGGCC}$NNNN	8.66	DR:0	20

265 LRH1-Bhimsaria2022-Rnd3 (S07-134)

PWM	Seed Sequence	Seed Seq Enrichment	Repeat	Rank
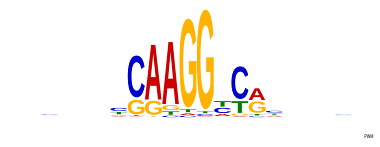	NNNNNNCAAGGTCANNNNNN	113.39	M	1
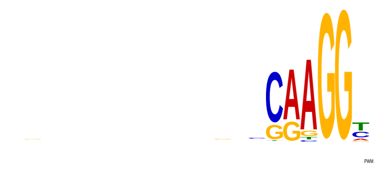	GGGGNNNNNNNNNNCAAGGT	391.36	M	2
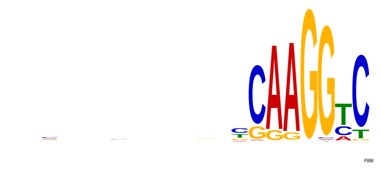	CCCCNNNNNNNNNCAAGGTC	396.40	M	3
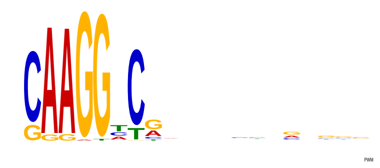	CAAGGTCGNNNNNNNNTGGG	664.34	M	4
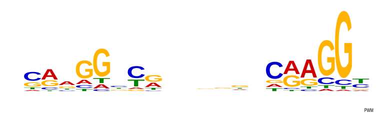	CAAGGNNNNNNNNNCGAGGT	299.80	DR:8	5
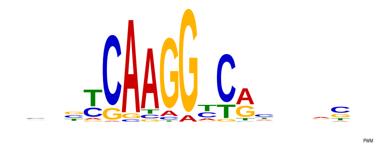	NNNTCAAGGCNACAGNNN	80.79	DR:0	6
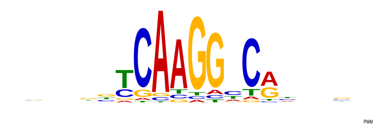	NNNNNTCAAGGGCANNNNN	45.01	DR:0	7
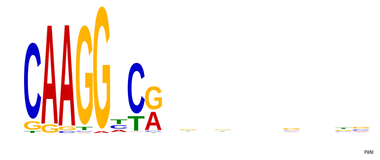	CAAGGCCNNNNNNNNNGGTG	266.13	DR:7	8
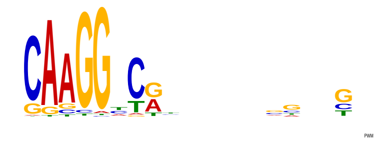	CAAGGCCNNNNNNNNNGGGT	230.69	DR:8	9

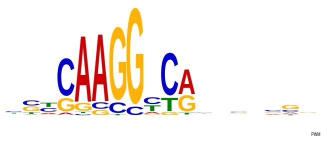	$N\overrightarrow{NNNTCA}\overrightarrow{AGGNNA}CAGNNNN$	51.45	DR:0	10
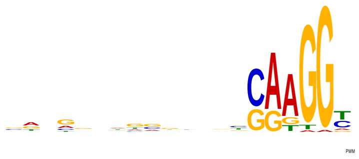	$CATGNNNNNN\overrightarrow{NNNTCA}\overrightarrow{AGGT}$	251.76	DR:0	11

266 LRH1-Yin2017-Rnd2 (ERR1005280)

PWM	Seed Sequence	Seed Seq Enrichment	Repeat	Rank
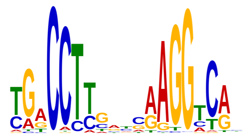	NNNNNNTGACCTNNNNNNAGGTCNNNNNN	186.00	ER:6	1
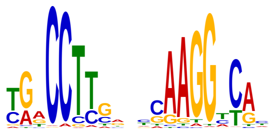	NNNNNNGACCTTNNNNCAAGGNNNNNNN	135.02	ER:7	2
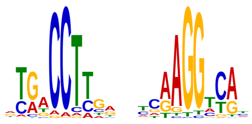	NNNNNNTGACCTNNNNNNNNAGGTCANNNNNN	151.68	ER:8	3
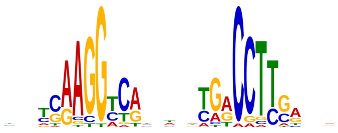	NNNNNNAAGGTCNNNNNNNNGACCTTNNNNNN	128.78	IR:5	4
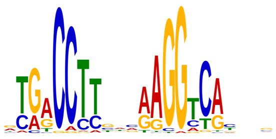	NNNNNNTGACCTTNNNNAGGTCANNNNNN	278.85	ER:5	5
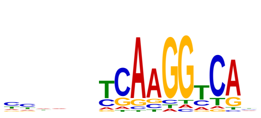	NNNNNNGTCANGGTCANNNNNN	20.10	DR:0	6
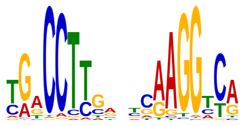	NNNNNNGACCTNNNNNNNAGGTCNNNNNN	70.46	ER:7	7
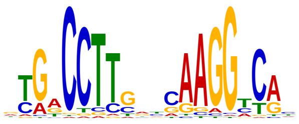	NNNNNNCCTTGNNCAAGGNNNNNN	64.93	M	8
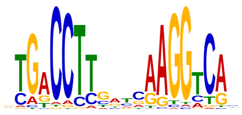	NNNNNNTGACCNNNNNNNNGGTCANNNNNN	61.93	ER:6	9

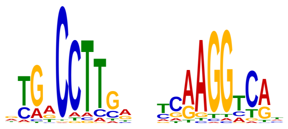	NNNNNNCCTTGNNNNNNAGGTCNNNNNN	39.28	ER:8	10
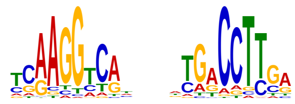	NNNNNNAGGTCNNNNNNNGACCTNNNNNN	28.93	IR:5	11
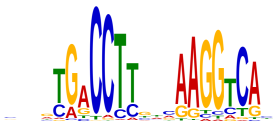	NNNNNNTGACCTNNNNNNGGTCANNNNNN	57.10	ER:5	12
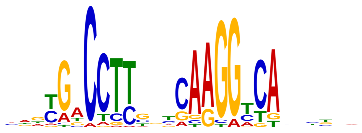	NNNNNNCCTTGGTCNNGGTCANNNNNN	270.14	M	13
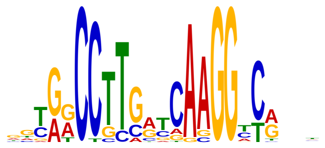	NNNNNNCCTTGATCNAGGCCNNNNNN	240.24	M	14
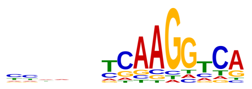	NNNNNNGTCANNGTCANNNNNN	8.71	DR:0	15
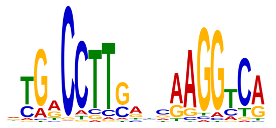	NNNNNNCTTGANNNNGGTCANNNNNN	36.90	ER:0	16
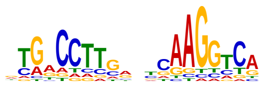	NNNNNNCCTTNNNNNNNNGTCANNNNNN	8.34	ER:7	17
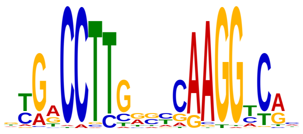	NNNNNNCCTTGGNCCAAGGNNNNNN	156.11	M	18

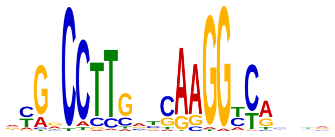 PWM	$NNNNNNCT\overrightarrow{TGATCA}\overrightarrow{NGGTCN}NNNNN$	131.25	DR:0	19
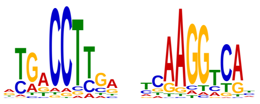 PWM	$NNNNN\overleftarrow{NGACCT}NNNNNNNN\overrightarrow{NGGTCA}NNNNNNN$	31.33	ER:8	20

267 LRH1-Yin2017-Rnd2 (ERR1005281)

PWM	Seed Sequence	Seed Seq Enrichment	Repeat	Rank
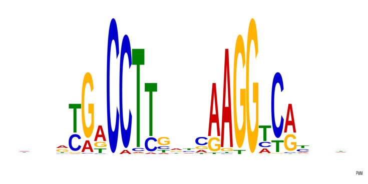	NNNNNNGACCTTNNNNAAGGTCNNNNNN	2427.53	ER:6	1
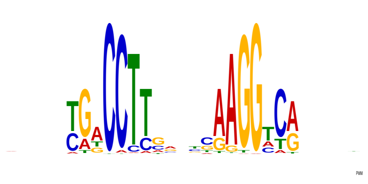	NNNNNNGACCTTNNNNNAAGGTCNNNNNN	1579.94	ER:7	2
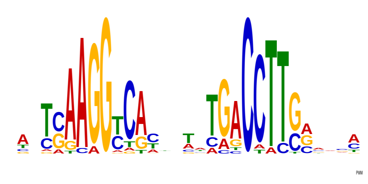	NNNNNAAGGTCANNNNNNGACCTTGNNNNN	3719.62	IR:5	3
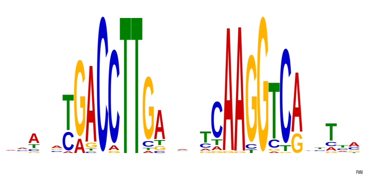	NNNNNNGACCTTGANNNNNAAGGTCNNNNNN	5675.95	M	4
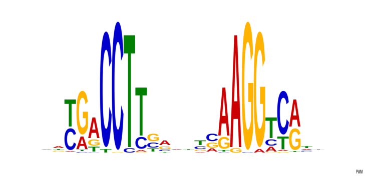	NNNNNNGACCTTNNNNNNAAGGTCNNNNNN	898.47	ER:8	5
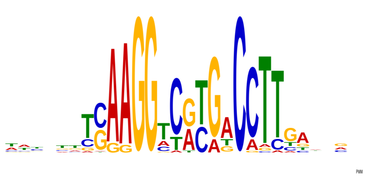	NNNNNNTCAAGGTCNNGACCTTNNNNNN	4229.07	M	6
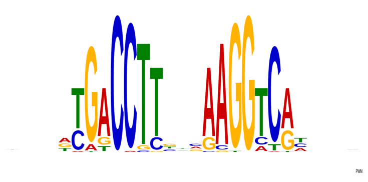	NNNNNNGACCTTNNNAAGGTCNNNNNN	743.74	ER:5	7
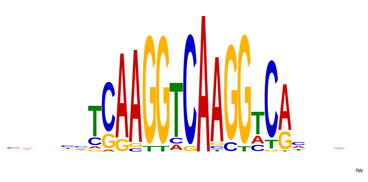	NNNNNNTCAAGGTCAAGGTCNNNNNN	2780.83	M	8
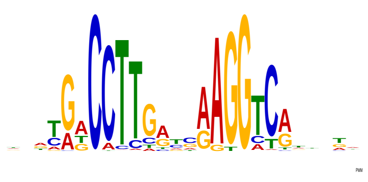	NNNNNNCCTTGATNNAGGTCNNNNNN	1134.25	M	9

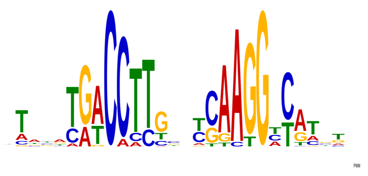	NNNNNNGACCTTGNNTCAAGGCNNNNNN	2622.96	M	10
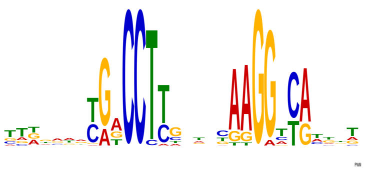	NNNNNNACGACCTTNNNNNAAGGTNNNNNN	1186.07	ER:7	11
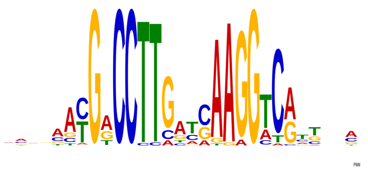	NNNNNNCGGCCTTGNNNAAGGTCNNNNNN	4856.66	ER:6	12
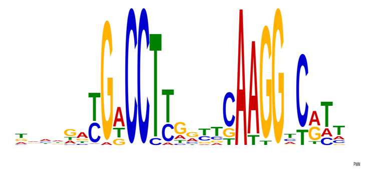	NNNNNNACGACCTNNNNTCAAGGNNNNNN	1146.68	M	13
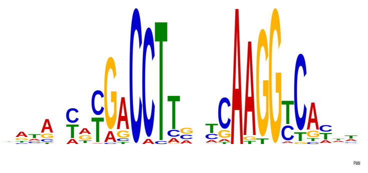	NNNNNNGCGACCTTNNTCAAGGNNNNNN	3512.54	M	14
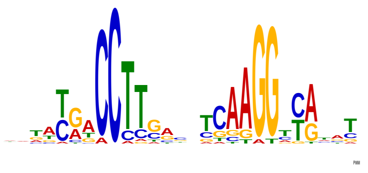	NNNNNNACCTTGANNTCAAGGNNNNNN	1024.96	M	15
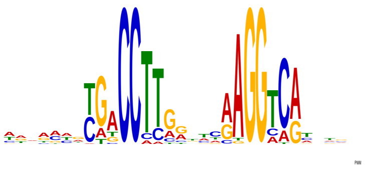	NNNNNNGCGACCTTNNNNNNAGGTCNNNNNN	1370.18	ER:7	16
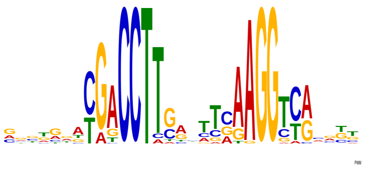	NNNNNNACGACCTTNNNNNNAAGGTNNNNNN	1477.99	ER:8	17
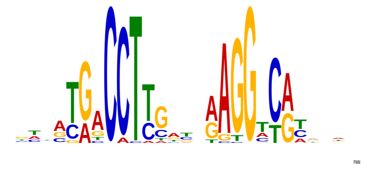	NNNNNNGACCTTCANNNAGGTCNNNNNN	1342.46	ER:6	18

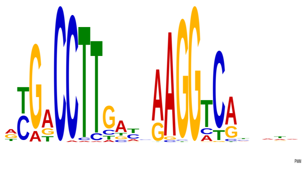	*NNNNNNCGACCTTGNNNNAGGTCNNNNNN*	3407.00	ER:6	19
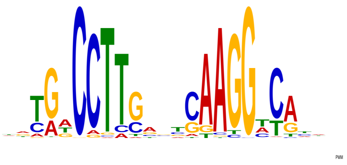	*NNNNNNNCCTTGANTCAAGGNNNNNNN*	598.09	M	20

268 LRH1-Yin2017-Rnd3 (ERR1005282)

PWM	Seed Sequence	Seed Seq Enrichment	Repeat	Rank
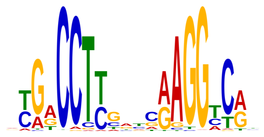	NNNNNNGACCTNNNNNNAGGTCNNNNNN	250.91	ER:6	1
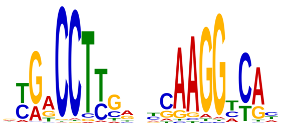	NNNNNNGACCTNNNNNNAAGGNNNNNN	96.66	ER:7	2
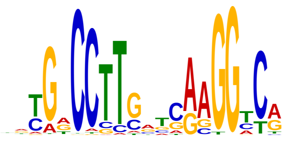	NNNNNNCTTGATCNNGGTCNNNNNN	344.46	ER:0	3
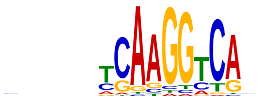	NNNNNNTTCANNGTCANNNNNN	30.77	DR:0	4
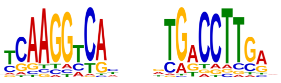	NNNNNNAAGGNNNNNNNNNNCCTTNNNNNN	19.69	IR:5	5
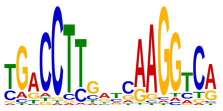	NNNNNNTGACNNNNNNNNNNGTCANNNNNN	30.68	ER:6	6
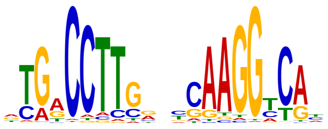	NNNNNNCTTGNNNCAAGNNNNNN	42.80	M	7
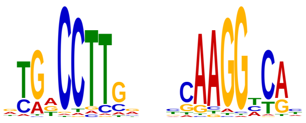	NNNNNNCCTTGNNNNCAAGGNNNNNN	95.68	ER:8	8
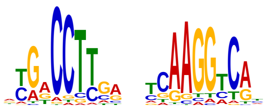	NNNNNNGACCTNNNNNNNNNGGTCNNNNNN	40.82	ER:8	9

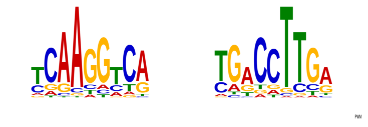	NNNNNNGGTCNNNNNNNGACCNNNNNN	16.34	IR:5	10
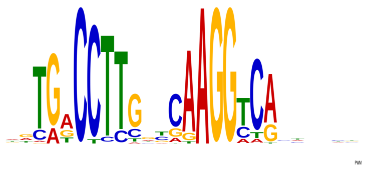	NNNNNNCTTGGCCANNGTCANNNNNNN	423.33	DR:0	11
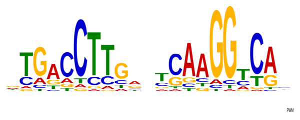	NNNNNNTGACNNNNNNTCAANNNNNNN	19.55	ER:1	12
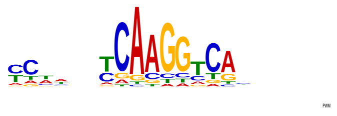	NNNNNNGGTCNNNGTCANNNNNNN	17.67	DR:0	13
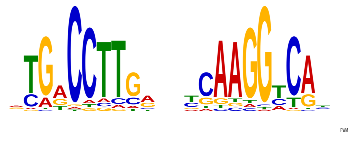	NNNNNNCTTGNNNNNCAAGNNNNNNN	22.94	M	14
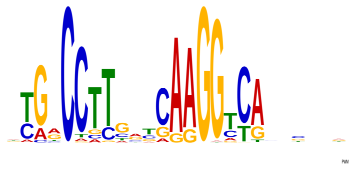	NNNNNNCTTGGTCNNNGTCANNNNNNN	236.60	DR:0	15
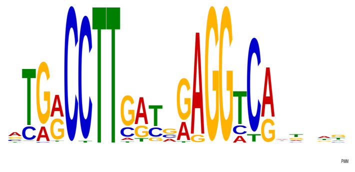	NNNNNNCTTGATGGAGGTCNNNNNN	606.29	ER:0	16
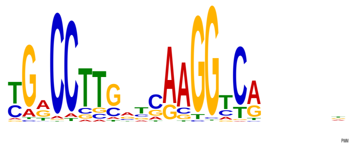	NNNNNNTTGATCNNNGTCANNNNNNN	93.52	ER:0	17
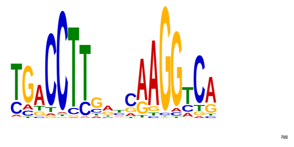	NNNNNNGTGACNNNNNNNNNNGTCANNNNNNN	36.72	ER:6	18

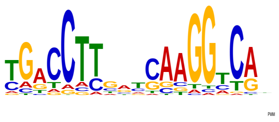	$NNNNNN\overleftarrow{TGACNN}\overrightarrow{NNNTCA}ANNNNNNN$	22.70	ER:0	19
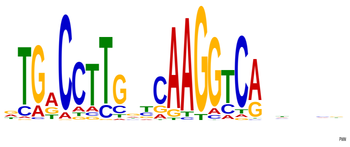	$NNNNNNCT\overleftarrow{TGGCCN}\overrightarrow{NNGTCA}NNNNNNN$	135.02	ER:0	20

269 LRH1-Yin2017-Rnd3 (ERR1005283)

PWM	Seed Sequence	Seed Seq Enrichment	Repeat	Rank
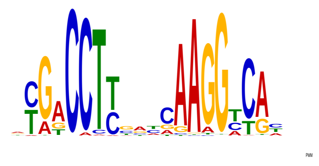	NNNNNNGACCTNNNNNAAGGNNNNNN	366.73	ER:6	1
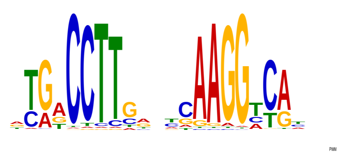	NNNNNNCCTTNNNNNAAGGNNNNNN	139.96	ER:7	2
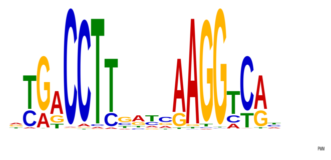	NNNNNNACCTNNNNNNAGGTNNNNNN	137.49	ER:6	3
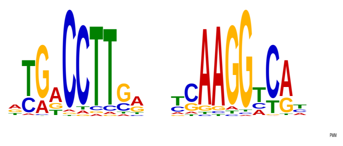	NNNNNNCCTTNNNNNNAAGGNNNNNN	65.07	ER:8	4
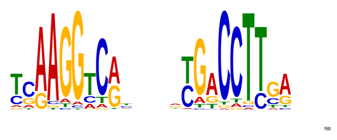	NNNNNNAGGTCNNNNNNNGACCTNNNNNN	228.27	IR:5	5
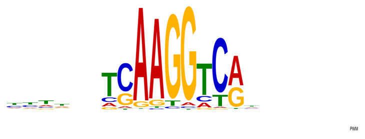	NNNNNNNTCAANNTCATNNNNNN	73.13	DR:0	6
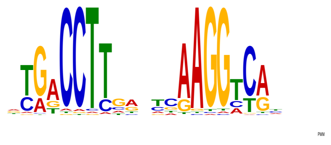	NNNNNNACCTNNNNNNNAGGTNNNNNN	95.82	ER:7	7
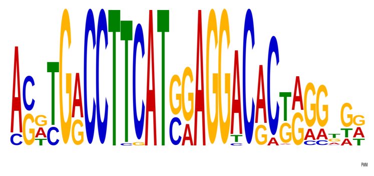	NNNNNNCCTTCATNNAGGACACTNNNNNN	10462.78	DR:3	8
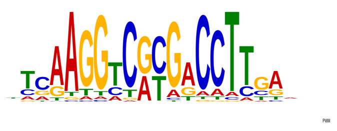	NNNNNNAGGTCNNGACCTNNNNNN	198.65	IR:0	9

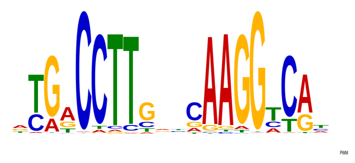	NNNNNNCTTGNNCAAGNNNNNN	136.64	M	10
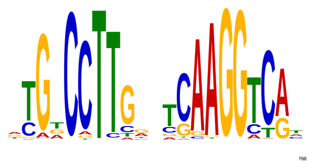	NNNNNNGTCCTTNNNTCAANNNNNN	269.19	ER:1	11
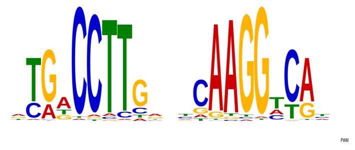	NNNNNNCTTGNNNCAAGNNNNNN	129.73	M	12
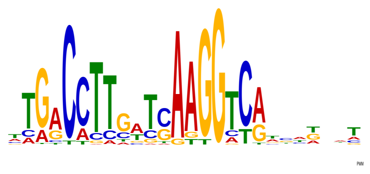	NNNNNNTTGATCANNNTCATNNNNNN	552.82	DR:0	13
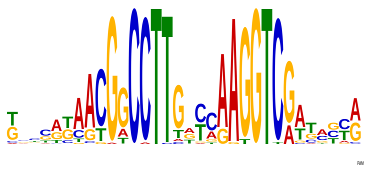	NNNNNNAACGGCCTNNNNNNAGGTCGNNNNNN	6544.21	ER:6	14
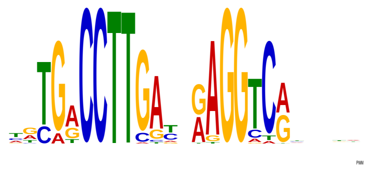	NNNNNNCTTGACNGAGGTCNNNNNN	944.80	ER:0	15
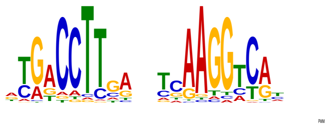	NNNNNNACCTNNNNNNNNAGGTNNNNNN	46.21	ER:8	16
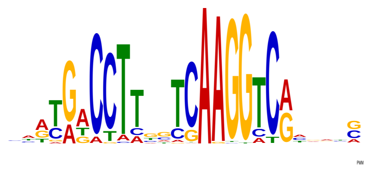	NNNNNNCCTTTGTCNNGGTCNNNNNN	946.82	M	17
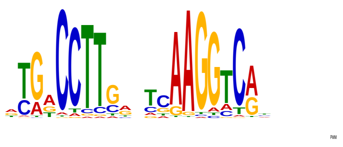	NNNNNNCCTTNNNNNNNNGTCGNNNNNN	51.11	ER:7	18

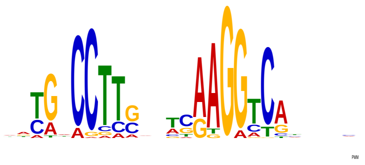	NNNNNNCTTGACTNNNGGTCNNNNNN	345.95	ER:1	19
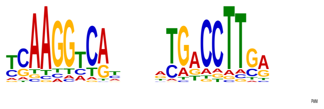	NNNNNNAAGGNNNNNNNNNNNNCCTTNNNNNN	51.41	IR:5	20

270 LRH1-Yin2017-Rnd4 (ERR1005284)

PWM	Seed Sequence	Seed Seq Enrichment	Repeat	Rank
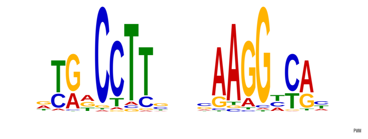	NNNNNNCCTTNNNNAAGGNNNNNN	36.07	ER:6	1
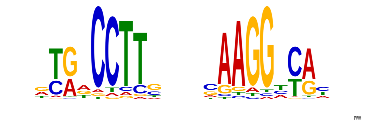	NNNNNNCCTTNNNNNAAGGNNNNNN	29.46	ER:7	2
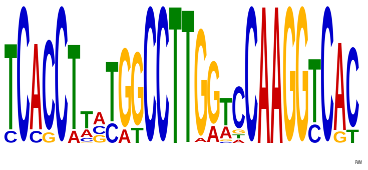	NNNNNNAATGNNNNTGGTCCAANNNNNN	190.96	M	3
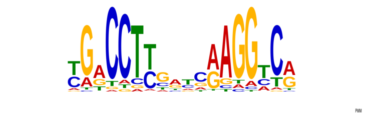	NNNNNNGACCNNNNNNNNGGTCNNNNNN	33.09	ER:6	4
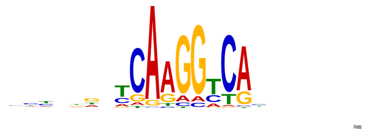	NNNNNNNTTCANNNTCATNNNNNN	20.21	DR:0	5
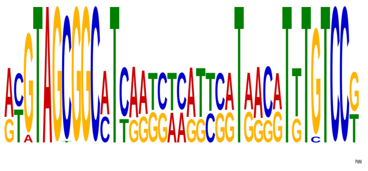	NNGTAGCGGCNNNNNNNNNNNNNNNNNNNNTGTCNN	216.98	M	6
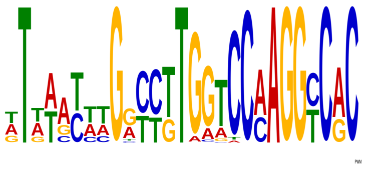	NNNNNNTTGGNNNNTGGTCCAANNNNNN	201.21	M	7
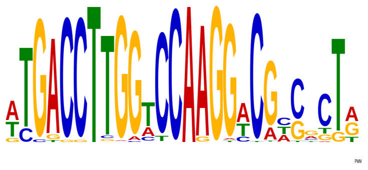	NNNNNNTTGGTCCANNGACGNNNNNN	377.24	IR:2	8
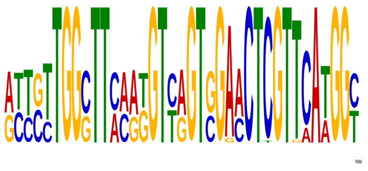	NNNNTTGGNNNNNNNNNNNNNNNNNCTCGTTCANNNN	192.78	M	9

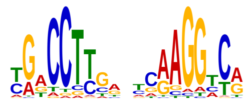	NNNNNNGACCNNNNNNNNNGGTCNNNNNN	25.22	ER:7	10
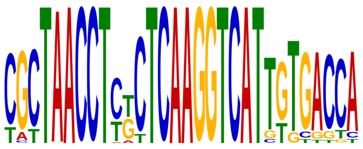	NNNNNNCCTTTCTCNNNNTCATTGNNNNNN	457.47	ER:6	11
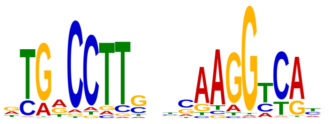	NNNNNNCTTGNNGAAGNNNNNN	18.70	M	12
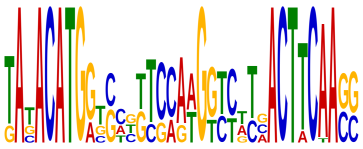	NNNACATGGNNNNNNNNNNNNNNNNACTTCANNN	203.75	ER:19	13
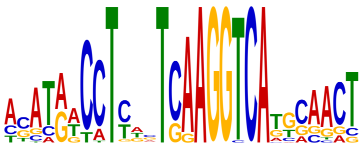	NNNNNNCCTTAGTCNNNNTCAGNNNNNN	127.91	M	14
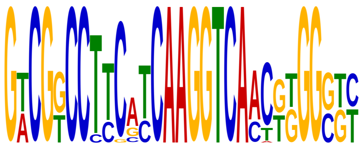	NNNNNNCTTCGTCANNNTCACCGNNNNNN	411.54	IR:3	15
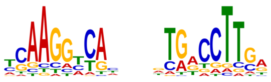	NNNNNNAAGGNNNNNNNNNNNCCTTNNNNNN	18.30	IR:5	16
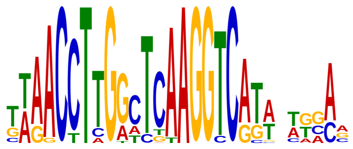	NNNNNNCTTGGCTCNNNNTCATTNNNNNN	280.76	ER:2	17
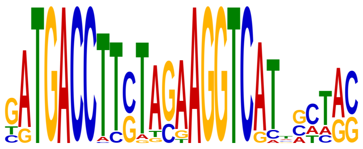	NNNNNNCTTCTAGANNNTCATNNNNNN	88.41	M	18

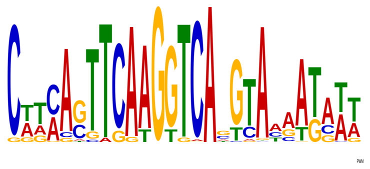	NNNNNNTTCANNNNCAGGTAANNNNNN	51.41	DR:5	19
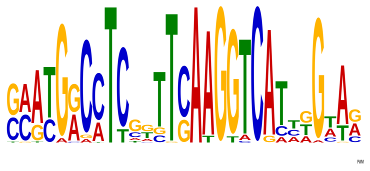	NNNNNNCCTCGGTTNNNNNTCATNNNNNN	95.35	M	20

271 LRH1-Yin2017-Rnd4 (ERR1005285)

PWM	Seed Sequence	Seed Seq Enrichment	Repeat	Rank
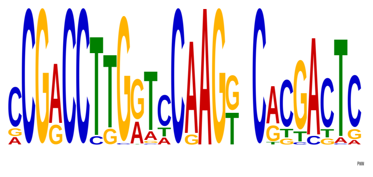	NNNNNNTTGGACCANNGACANNNNNN	234.02	ER:0	1
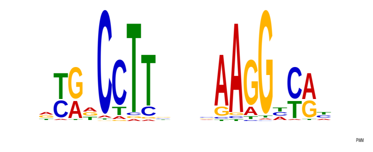	NNNNNNCCTTNNNNAAGGNNNNNN	33.31	ER:6	2
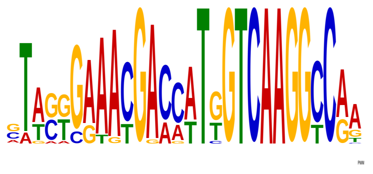	NNNNNNAAATNNNNTTTGTCAANNNNNN	129.51	M	3
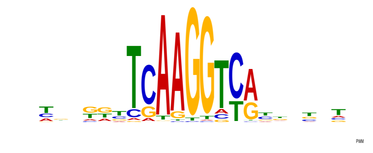	NNNNNNTTTCNNNNTCATNNNNNN	23.14	M	4
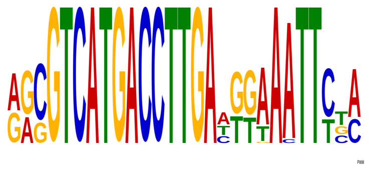	NNNGTCATGANNNNNNNNNNAAATTNNN	165.00	IR:0	5
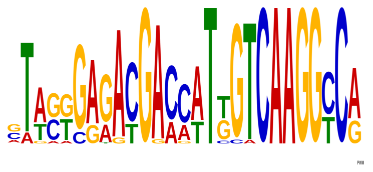	NNNNNNAAATNNNNTTTGTCANNNNNN	68.27	M	6
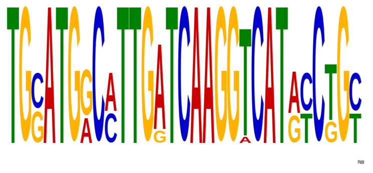	NNNNNNGCATTGATNNNNNNTCATNNNNNN	118.34	ER:0	7
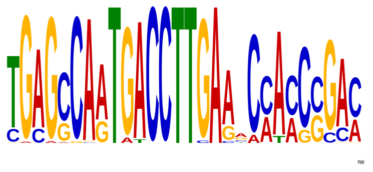	NNNNNNAATGNNNNNGAAACAANNNNNN	105.54	M	8
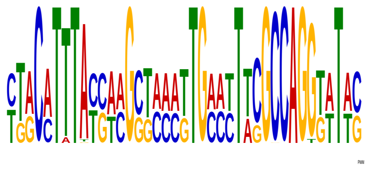	NNNNNTTTANNNNNNNNNNNNNNNNNNNGCCAGGNNNNNN	100.83	M	9

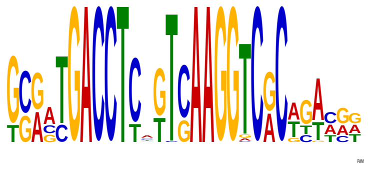	$NNNN\overleftarrow{NNACCT}CAGTNN\overrightarrow{NNGTCG}CNNNNNN$	336.83	ER:6	10
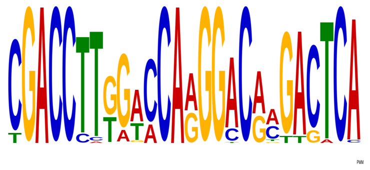	$NNNNNNT\overleftarrow{GGACCA}\overrightarrow{NNGACA}CNNNNNN$	232.45	ER:0	11
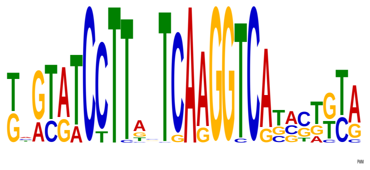	$NNN\overleftarrow{NNNCCT}TTTTCN\overrightarrow{NNNTCA}TNNNNNN$	150.35	ER:6	12
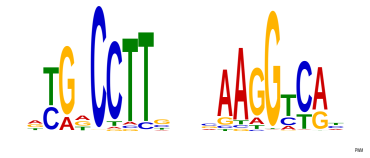	$NNNNNNCTTGNNGAAGNNNNNN$	23.59	M	13
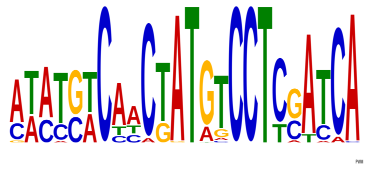	$NNNNN\overleftarrow{NCAACT}\overrightarrow{ANGTCC}TNNNNNN$	45.27	ER:0	14
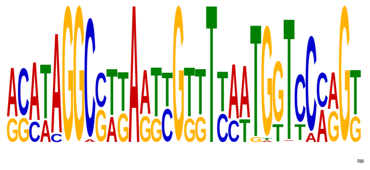	$NNNN\overrightarrow{AGGCNN}NNNNNNNNNNNN\overrightarrow{TGGTCC}NNNN$	33.66	DR:12	15
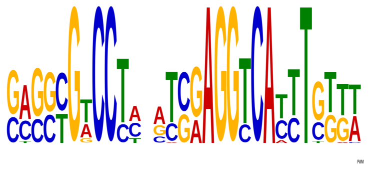	$NNNN\overleftarrow{NNACCT}CGATNN\overrightarrow{NNNTCA}TNNNNNN$	60.11	ER:6	16
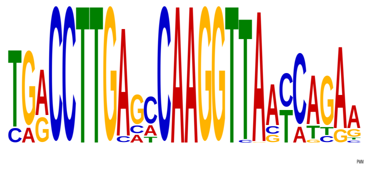	$NNNNNN\overleftarrow{TGAGCC}A\overrightarrow{ANNTTA}ANNNNNN$	167.19	ER:1	17
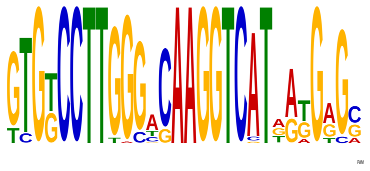	$NNNNNNTT\overrightarrow{GGGACA}NNNNCATTNNNNNN$	103.57	M	18

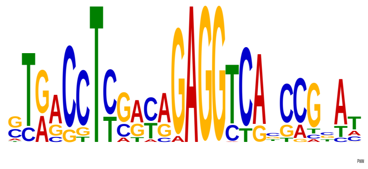	$NNNNNNT\overleftarrow{TGATAG}\overrightarrow{ANNTCA}TNNNNNN$	186.40	ER:0	19
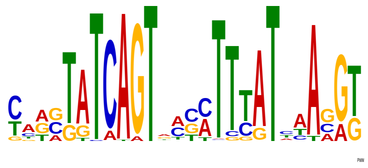	$NNN\overrightarrow{NNNTCA}GTNNNNTTTATNNNNNN$	57.13	M	20

272 SF1:RXRA-Bhimsaria2022-Rnd3 (S13-159)

PWM	Seed Sequence	Seed Seq Enrichment	Repeat	Rank
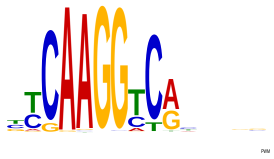	NNNNNNTCAAGGTCNNNNNN	1056.63	DR:0	1
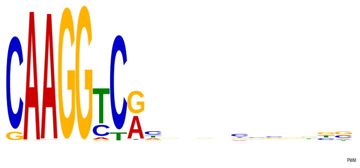	CAAGGTCNNNNNNNNNGGGG	4130.05	M	2
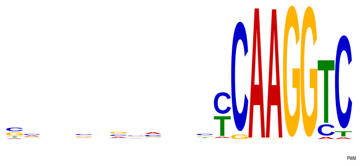	CCCCNNNNNNNNCCAAGGTC	5560.45	M	3
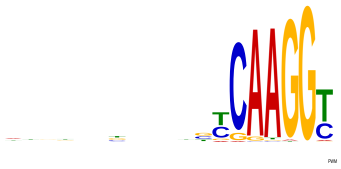	CATGNNNNNNNNNTCAAGGT	2108.34	DR:0	4
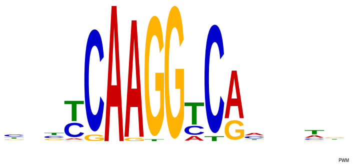	NNCCCAAGGTNACAGNN	1020.61	M	5
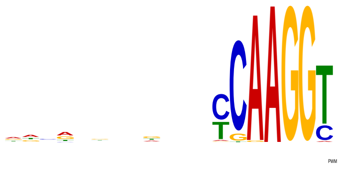	AAACNNNNNNNNNCCAAGGT	1590.91	M	6
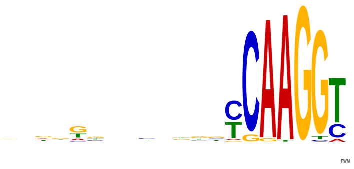	ATGGNNNNNNNNNCCAAGGT	1841.57	M	7
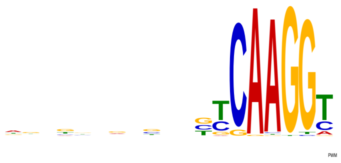	CAGCNNNNNNNNNTCAAGGT	1626.13	DR:0	8
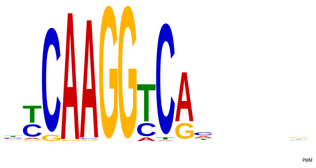	NNNCCAAGGTNACAGNNN	693.21	M	9

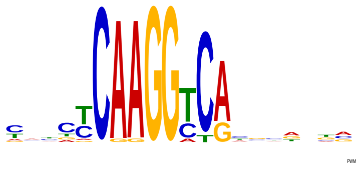	$NNNNCCA\overrightarrow{AGGNNA}CAGNNNN$	197.91	M	10
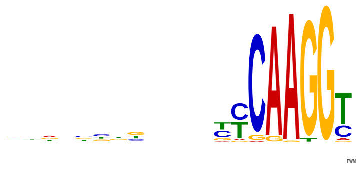	$ATATNNNNNN\overrightarrow{NNNTCA}\overrightarrow{AGGT}$	1658.19	DR:0	11
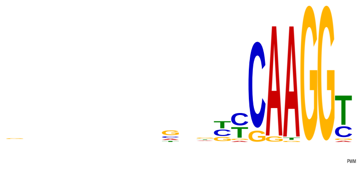	$\overrightarrow{GGGCN}NNNNN\overrightarrow{NNNTCA}\overrightarrow{AGGT}$	1731.39	M	12

273 SF1-Bhimsaria2022-Rnd2 (S07-012)

PWM	Seed Sequence	Seed Seq Enrichment	Repeat	Rank
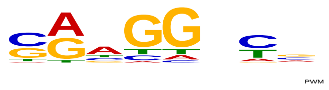	CAAGGTCG	10.00	M	1
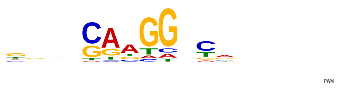	NNNNNCAAGGCCANNNNN	12.01	M	2
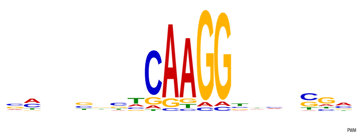	NNNNNNCTCAAGGTNNNNNN	14.99	DR:0	3
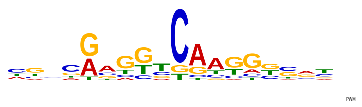	NNNNNAGGTCAAGGNNNNN	15.67	DR:0	4
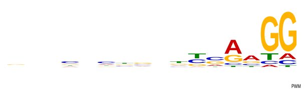	GGGGNNNNNNNNNNNAAGG	5.42	M	5
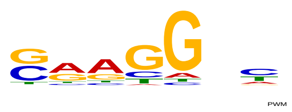	CCAAGGTC	10.09	M	6
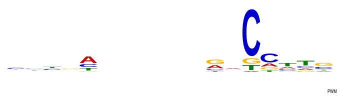	CCCCNNNNNNNNNNNCTTG	4.42	M	7
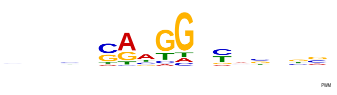	NNNNNTCAAGGCCNNNNN	9.24	DR:0	8
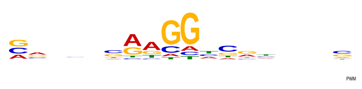	NNNNNCGAGGTCANNNNN	5.89	M	9

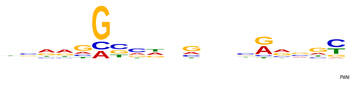	*NCAAGNNNNNNNNNGAGGN*	4.13	M	10
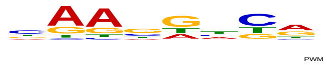	*CGAGGCCA*	4.45	M	11
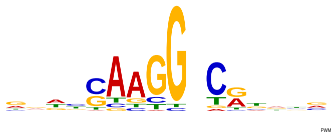	*NNNNCCAAGGACGNNNN*	17.28	M	12
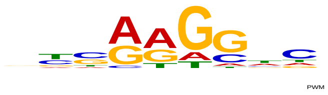	*GTCGAGGTC*	8.34	DR:0	13
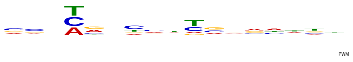	*NNNNGGCCNTGACNNNN*	4.00	IR:0	14
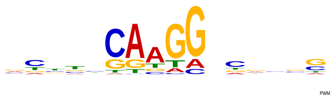	*NNNNTCCAAGGCCNNNN*	13.73	M	15
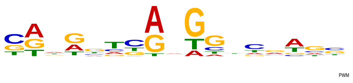	*NNNGGTCNNGGTCGNNN*	7.66	DR:0	16
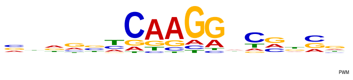	*NNNNGTCAAGGGCNNNN*	11.69	DR:0	17
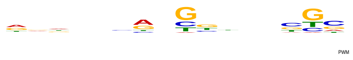	*NNNGTCANNGCCANNN*	3.25	DR:0	18

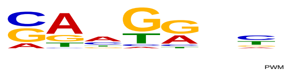	$\overrightarrow{CCAAGGCC}$	6.80	M	19
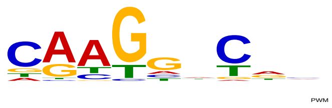	$\overrightarrow{CAAGGACAA}$	13.26	M	20

274 SF1-Bhimsaria2022-Rnd3 (S07-133)

PWM	Seed Sequence	Seed Seq Enrichment	Repeat	Rank
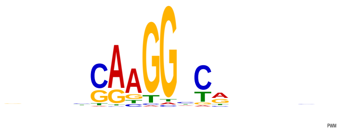	NNNNNNCAAGGCCANNNNNN	90.14	M	1
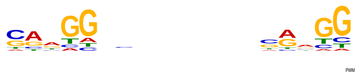	CAAGGNNNNNNNNNNNAAGG	111.28	M	2
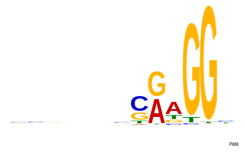	GGGGNNNNNNNNNNCAAGGT	190.93	M	3
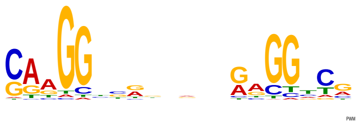	CAAGGNNNNNNNNGAGGTCG	394.81	DR:6	4
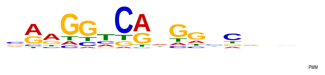	NNNNAGGTCNNGGCCNNNN	65.45	DR:0	5
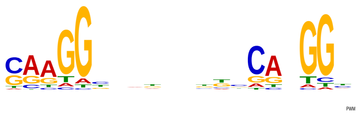	CAAGGNNNNNNNNNCATGGT	285.40	DR:8	6
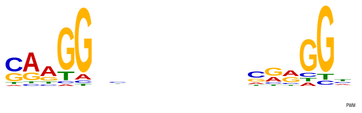	CAAGGNNNNNNNNNCGGGGT	330.99	DR:8	7
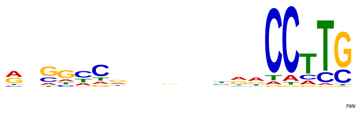	ACGGCCNNNNNNNNNCCTTG	190.67	IR:6	8
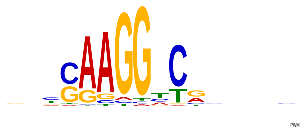	NNNNNTGAAGGTCGNNNNN	74.05	M	9

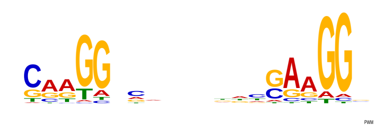	CAAGGNNNNNNNNCGAAGGG	484.87	DR:8	10
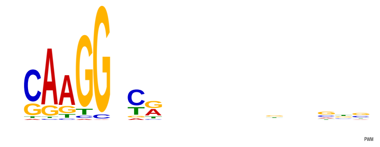	CAAGGCCNNNNNNNNNGGTG	216.16	DR:7	11
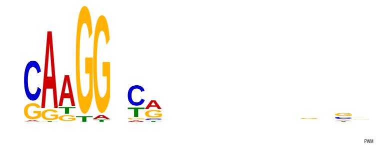	CAAGGCCNNNNNNNNNGTGG	207.13	M	12
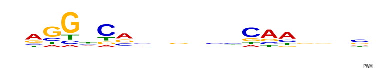	AGGTCNNNNNNNNNNGGCC	14.22	DR:8	13
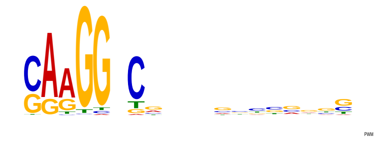	CAAGGACNNNNNNNNNGGGA	250.90	M	14
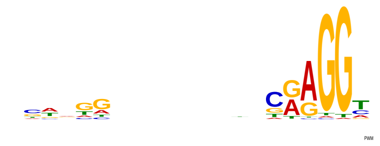	CATGGNNNNNNNNNCGAGGT	258.91	M	15
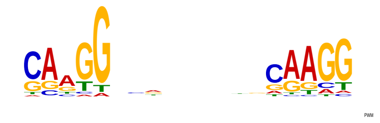	CAAGGNNNNNNNNGCAAGGG	495.70	DR:8	16
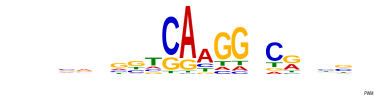	NNNNNGGTCAAGGGCNNNNN	111.51	DR:0	17
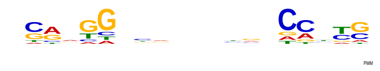	CAAGGNNNNNNNNNCCTTG	123.46	IR:3	18

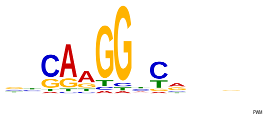	$NNN\overrightarrow{NNCTCA}\overrightarrow{NGGTCN}NNNN$	35.30	DR:0	19
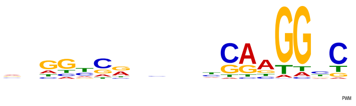	$A\overrightarrow{AGGNNN}NNNNNCA\overrightarrow{AGGCC}$	128.11	DR:7	20

275 TLX:RXRA-Bhimsaria2022-Rnd3 (S13-145)

PWM	Seed Sequence	Seed Seq Enrichment	Repeat	Rank
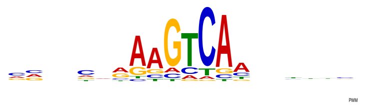	NNNNNNAAAGTCAANNNNNN	31.00	M	1
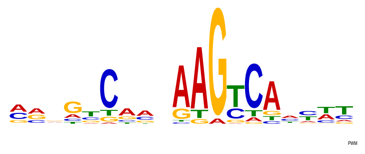	NNNGTCANNAAGTCAANNN	162.02	DR:2	2
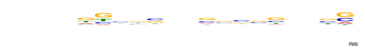	GGGGNNNNNNNNNNNNGGGG	7.63	M	3
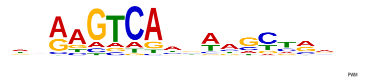	NNNAGTCANNNAGCTNNN	17.46	IR:1	4
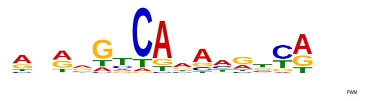	NNNAGTCAANAGTCNNN	26.74	DR:1	5
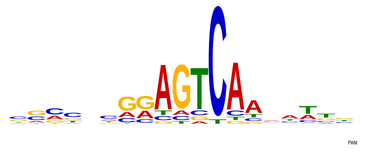	NNNNNCGGAGTCAANNNNN	26.85	M	6
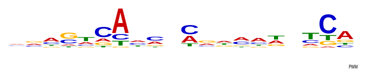	NNAGTCNNNNNNNNAAGTNN	8.20	DR:7	7
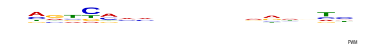	NAGTCNNNNNNNNNAAGTN	7.21	DR:8	8
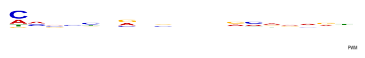	CAACNNNNNNNNNNNAAGT	6.46	M	9

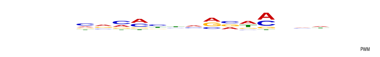	$\overrightarrow{NGACA}NNNNNNNNNNAAAAN$	4.37	M	10
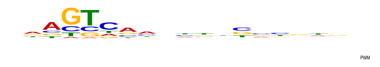	$\overrightarrow{NAGTCA}NNNNNNNNCGTGN$	11.67	M	11
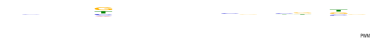	$CAATNNNNNNNNNNNNGTTG$	5.31	M	12
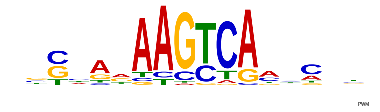	$NNNAT\overrightarrow{AAGTCA}ATNNN$	23.92	M	13
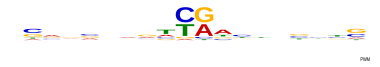	$NNNN\overrightarrow{AAGTNN}ACTTNNNN$	5.89	M	14
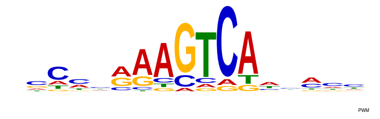	$NNNAG\overrightarrow{GAGTCA}ATNNN$	16.81	M	15
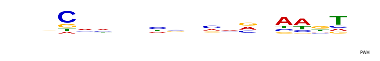	$NGCAANNNNNNNNNN\overrightarrow{AAGTN}$	5.80	M	16
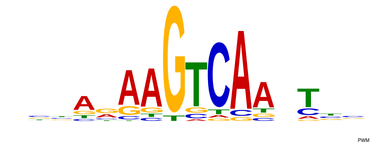	$NNAG\overrightarrow{AANTCA}AGTNN$	22.05	M	17
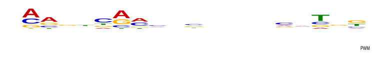	$\overrightarrow{NAGTCN}NNNNNNNNCATGN$	6.96	M	18

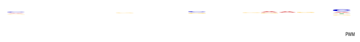	CCACNNNNNNNNNNNAGTC	6.03	M	19
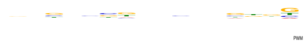	CGTGNNNNNNNNNNNGTGG	5.93	M	20

276 TLX-Bhimsaria2022-Rnd2 (S07-084)

PWM	Seed Sequence	Seed Seq Enrichment	Repeat	Rank
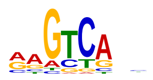	NNNNNNGAAGTCAANNNNNN	14.49	M	1
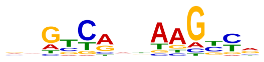	NNNNGTCANNAAGCCNNNN	21.62	DR:2	2
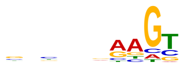	GGGGNNNNNNNNNNNNAAGT	7.66	M	3
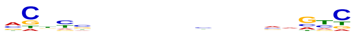	ACTCNNNNNNNNNNNNAGTC	8.84	M	4
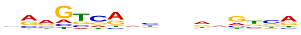	NNNAGTCNNNNNNAGTCNNN	5.67	DR:4	5
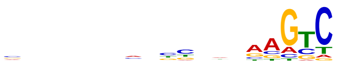	CCACNNNNNNNNNNNAAGTC	11.76	M	6
	NNNNAGTCNNNGACTNNNN	6.55	IR:1	7
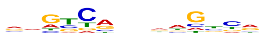	NNNAGTCNNNNNGTCANNN	8.14	DR:2	8
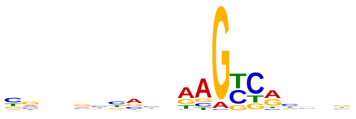	NNNNGCCANNAAGTCANNNN	30.36	DR:2	9

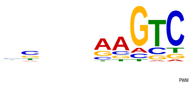	$CCGCNNNNNNNNNNN\overrightarrow{AAGTC}$	10.34	M	10
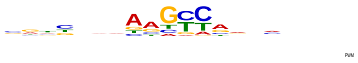	$NNNNNNAA\overrightarrow{AGCTAA}NNNNNN$	4.02	M	11
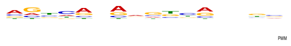	$NNN\overrightarrow{NNGTCA}NN\overrightarrow{NAGTCN}NNNNN$	6.15	DR:1	12
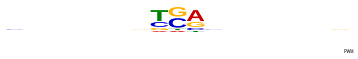	$NNNNN\overrightarrow{AAGTNA}CTTNNNNN$	6.40	M	13
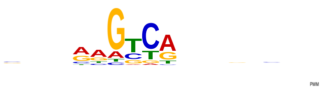	$NNNNNN\overrightarrow{AAGTCA}AANNNNNN$	11.30	M	14
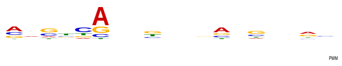	$N\overrightarrow{NAGTCN}NNNNNNN\overrightarrow{NAGTCN}N$	4.02	DR:6	15
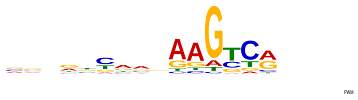	$NNNNNCAAG\overrightarrow{AAGTCN}NNNN$	15.71	M	16
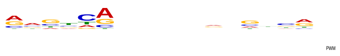	$N\overrightarrow{NAGTCN}NNNNN\overrightarrow{NAGTCN}N$	4.34	DR:5	17
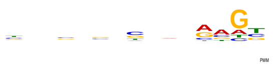	$AAAGNNNNNNNNNNNNN\overrightarrow{AAGT}$	4.52	M	18

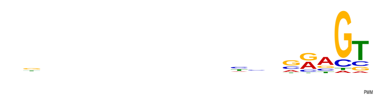	*GGCGNNNNNNNNNNNGGAGT*	9.43	M	19
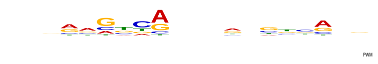	$NN\overrightarrow{NAGTCN}NNN\overrightarrow{NAGTCN}NN$	4.98	DR:3	20

277 TLX-Bhimsaria2022-Rnd3 (S07-109)

PWM	Seed Sequence	Seed Seq Enrichment	Repeat	Rank
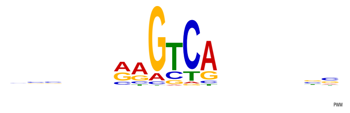	NNNNNNGAAGTCAANNNNNN	36.83	M	1
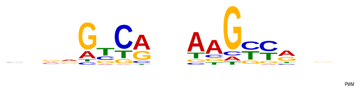	NNNNGTCANNAAGCCNNNN	65.91	DR:2	2
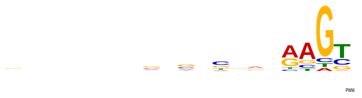	GGGGNNNNNNNNNNNNAAGT	22.24	M	3
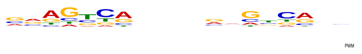	NNNGTCANNNNNAGTCNNN	12.95	DR:4	4
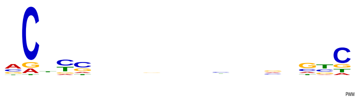	ACTCCNNNNNNNNNNNAGTC	35.08	M	5
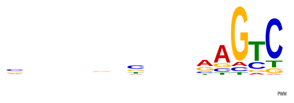	CCCCNNNNNNNNNNNAAGTC	30.93	M	6
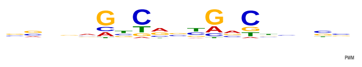	NNNNAGTCANTGACTNNNN	60.11	IR:1	7
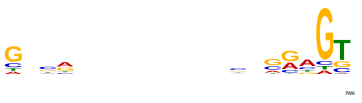	GTCANNNNNNNNNNNGGAGT	27.70	M	8
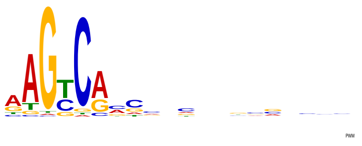	AAGTCANNNNNNNNNNCCCC	78.81	M	9

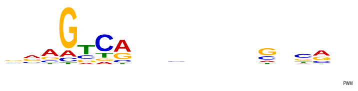	$NN\overrightarrow{AGTCA}NNNNNN\overrightarrow{NAGTC}NN$	23.76	DR:5	10
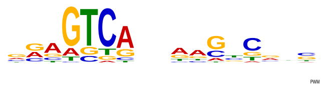	$NNG\overrightarrow{GAGTC}NNNNN\overrightarrow{NGTCG}NN$	47.10	DR:2	11
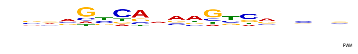	$NNN\overrightarrow{NGTCA}NN\overrightarrow{NGTCA}NNNN$	17.14	DR:1	12

278 Tlx-Jolma2013-Rnd3 (ERR185061)

PWM	Seed Sequence	Seed Seq Enrichment	Repeat	Rank
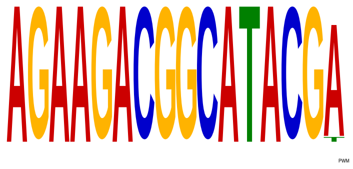	AGAAGACGGCATACGA	67503.75	M	1
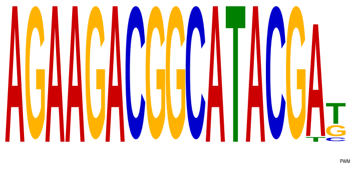	NGAAGACGGNNTACGAN	3463.65	M	2
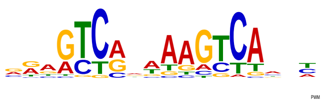	NNNNAGTCNNNAAGTCNNNN	29.62	DR:2	3
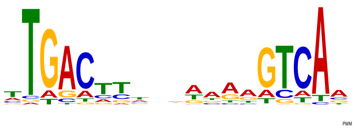	NTGACTNNNNNNNNGTCAN	14.06	ER:5	4
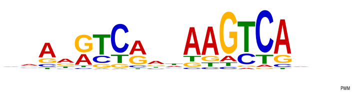	NNNNGTCAANNAGTCNNNN	24.31	DR:2	5
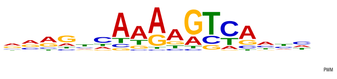	NNNNGTCANNAGTCANNNN	9.01	DR:1	6
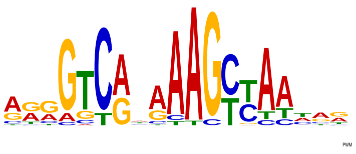	NNNGTCANNAAGCTAANNN	57.20	DR:3	7
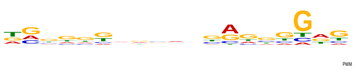	NGGGGNNNNNNNNGGGGAN	7.27	M	8
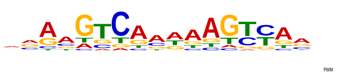	NNNNAGTCNNNAAGCNNNN	6.83	M	9

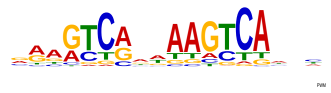	$NNN\overrightarrow{NAGTCN}NN\overrightarrow{NAGTCN}NNN$	13.96	DR:2	10
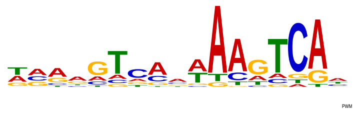	$NN\overrightarrow{CAGTCN}NN\overrightarrow{NAGTCN}N$	6.31	DR:2	11
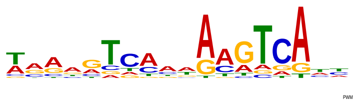	$NN\overrightarrow{CAGTCN}NNN\overrightarrow{NGTCAN}N$	6.41	DR:2	12
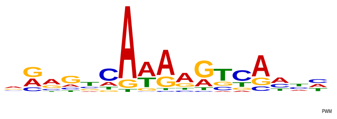	$NN\overrightarrow{NAGTCN}N\overrightarrow{NAGTCA}NN$	7.25	DR:1	13
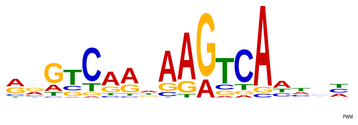	$\overrightarrow{NNNTCA}AG\overrightarrow{ANNTCA}ANNN$	16.33	DR:2	14
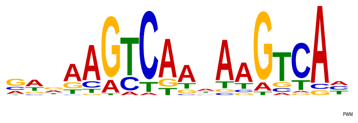	$NNT\overrightarrow{AAGTCN}NN\overrightarrow{NAGTCN}N$	19.02	DR:2	15
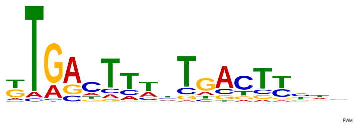	$NNNNCTTT\overrightarrow{ANGACT}NNNN$	6.89	M	16
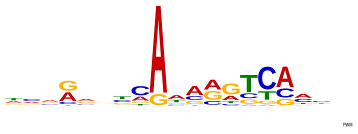	$NNN\overrightarrow{NAGTCN}N\overrightarrow{AAGTCN}NNN$	5.83	DR:1	17
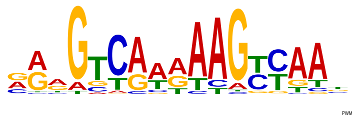	$N\overrightarrow{NAGTCN}NN\overrightarrow{AAGCTAN}N$	23.80	DR:3	18

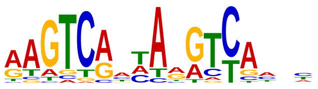	$NN\overrightarrow{AGTCN}NN\overrightarrow{NNGTCA}ACNN$	25.24	DR:2	19
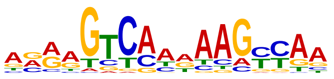	$NN\overrightarrow{NAGTCN}NNA\overrightarrow{AGCTNN}N$	9.76	DR:3	20

279 TLX-Jolma2013-Rnd3 (ERR195035)

PWM	Seed Sequence	Seed Seq Enrichment	Repeat	Rank
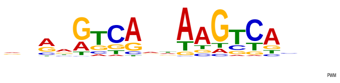	NNNNAGTCNNNNAGTCNNNN	8.65	DR:2	1
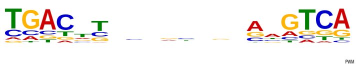	NGACTNNNNNNNNNAGTCN	7.67	ER:8	2
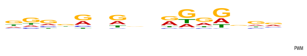	NNNGGGGNNNNNNGGGGNNN	6.65	M	3
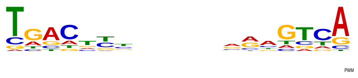	NGACTNNNNNNNNAGTCN	5.75	ER:7	4
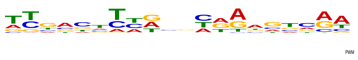	NNGACTNNNNNNNNAGTCNN	3.30	ER:6	5
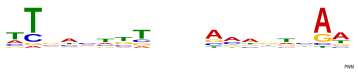	NNGACTNNNNNNNAGTCNN	2.67	ER:5	6
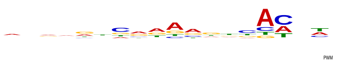	NNNNAGTCNNAGTCNNNN	2.88	DR:1	7
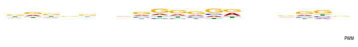	GGGGNNNNNNNNNNNGGGG	4.94	M	8
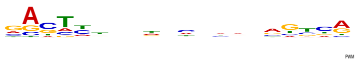	GACTNNNNNNNNNNNGTCA	3.99	ER:9	9

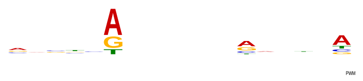	NAGTCNNNNNNNNAGTCN	2.49	DR:6	10
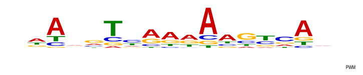	NNNAGCCNNNNAGTCNNN	3.12	M	11
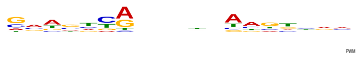	NNAGTCNNNNNNNAGTCNN	2.74	DR:5	12
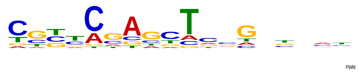	NNNNNGAGCTCCGNNNNN	3.04	M	13
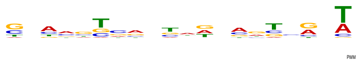	NNNAGTCNNNNNNAGTCNNN	2.87	DR:4	14
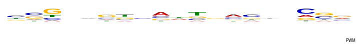	NNNNAGTCNNNNGACTNNNN	2.54	IR:1	15
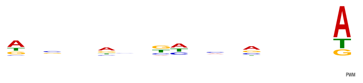	NAGTCNNNNNNNNNAGTCN	2.05	DR:7	16
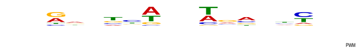	NNNAGTCNNNNGACTNNN	2.35	IR:2	17
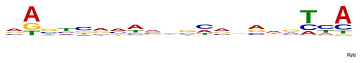	NAGTCNNNNNNNNNNAGTCN	3.35	DR:8	18

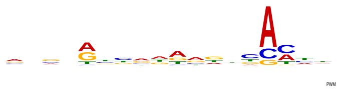	$NNN\overrightarrow{NAGTCA}N\overrightarrow{NAGTCN}NNN$	3.88	DR:1	19
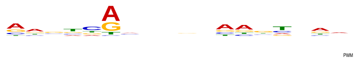	$\overrightarrow{NAGTCN}NNNNN\overrightarrow{NNGTCA}N$	2.75	DR:5	20

280 Tlx-Jolma2013-Rnd4 (ERR185062)

PWM	Seed Sequence	Seed Seq Enrichment	Repeat	Rank
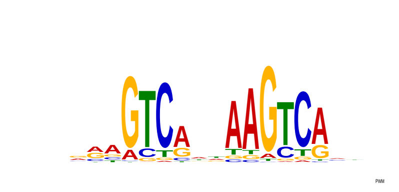	NNNNAGTCNNNNAGTCNNNN	45.01	DR:2	1
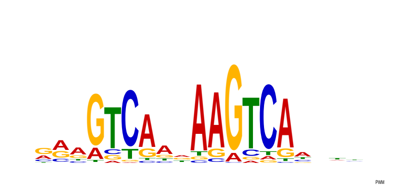	NNNNTCAANNNNTCAANNNN	21.72	DR:2	2
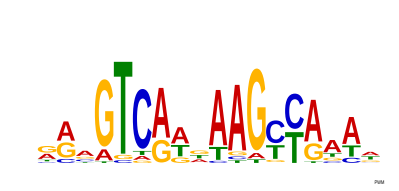	NNAGTCNNNAAGCTAANN	71.45	DR:3	3
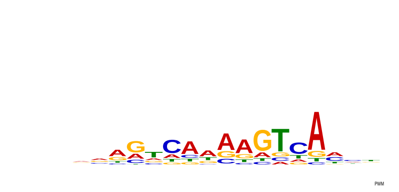	NNNNAGTCNNAGTCNNNN	6.52	DR:1	4
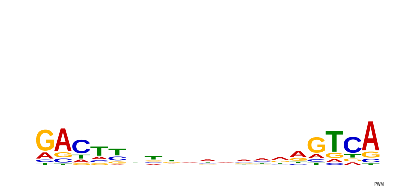	GACTNNNNNNNNNNGTCA	6.66	ER:8	5
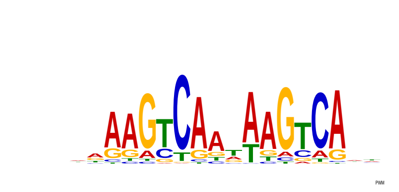	NNNAAAGNNNNNNAGTCNNN	19.26	M	6
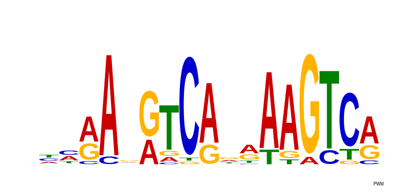	NAAAGGTCNNNNAGTCN	72.40	DR:2	7
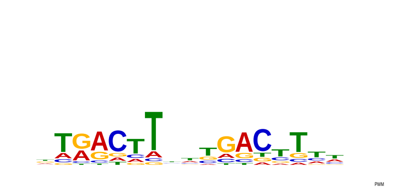	NNNGCTTNNNNNCTTTNNN	5.75	M	8
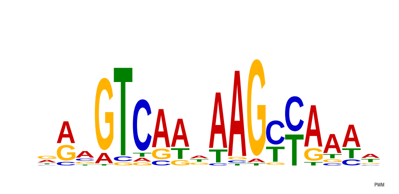	NNAGTCNNNNAGCTAANN	35.13	DR:3	9

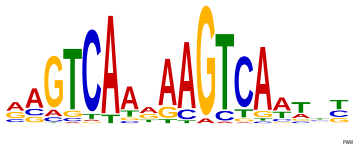	$\overrightarrow{NAGTC}NNN\overrightarrow{NNNTC}AATCN$	36.39	DR:2	10
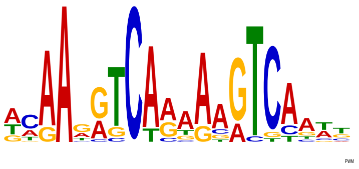	$NNA\overrightarrow{AGGTC}NNN\overrightarrow{NNNTC}AANN$	40.42	DR:2	11
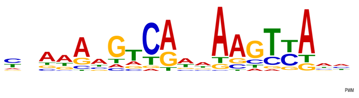	$NNNN\overrightarrow{AAGT}NNNNAAGCNNNN$	7.26	M	12
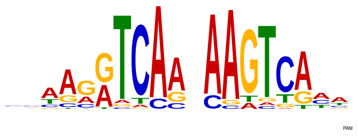	$NNN\overrightarrow{AGATC}NNN\overrightarrow{NAGTC}NNN$	21.93	DR:2	13
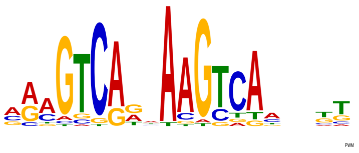	$N\overrightarrow{NAGTC}NNNNNNNCAATTNN$	19.33	M	14

281 TLX-Jolma2013-Rnd4 (ERR195036)

PWM	Seed Sequence	Seed Seq Enrichment	Repeat	Rank
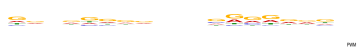	NNNGGGGNNNNNGGGGNNN	10.54	M	1
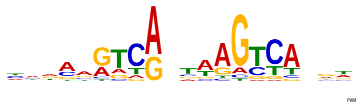	NNNNAGTCNNNNAGTCNNNN	9.21	DR:2	2
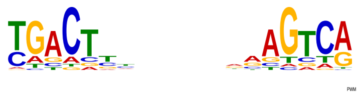	NGACTNNNNNNNNAGTCN	9.74	ER:7	3
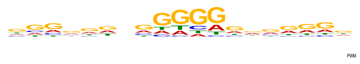	GGGGNNNNNNNNNNGGGG	9.43	M	4
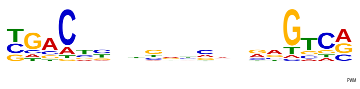	NGACTNNNNNNNNNAGTCN	5.82	ER:8	5
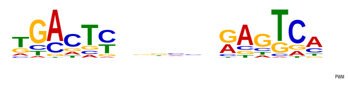	NNGACTNNNNNNNNAGTCNN	6.70	ER:6	6
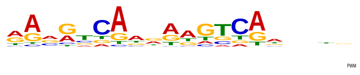	NNNNTCAANNNNTCAANNNN	5.61	DR:2	7
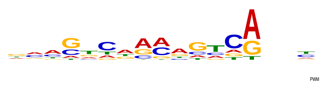	NNNNAGTCNNAGTCNNNN	3.48	DR:1	8
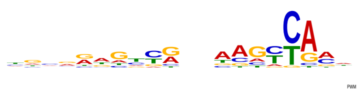	NNNNNAGTCNNAAAGNNNNN	4.15	M	9

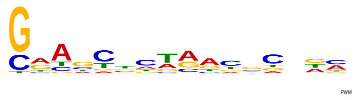	NNNGCTCNNACGCNNN	3.21	M	10
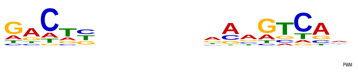	GACTNNNNNNNNNNNTCAA	4.84	ER:7	11
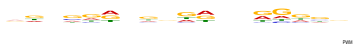	NTGGGNNNNNNNNAGGGN	3.93	M	12
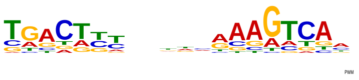	TGACNNNNNNNNNNNNTCAA	6.02	ER:7	13
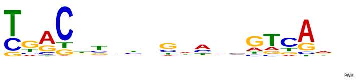	NGACTNNNNNNNNNNTCAAN	4.04	ER:6	14
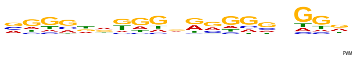	NGGGTNNNNNNNNNGTGGN	3.84	M	15
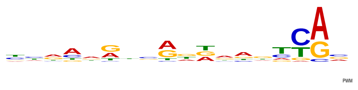	NNNNGGTCNNAAAGNNNN	2.67	M	16
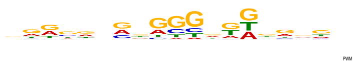	NGGGANNNNNNNNNGAGGN	3.87	M	17
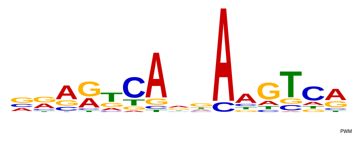	NGAATCNNNNAGTCN	6.19	M	18

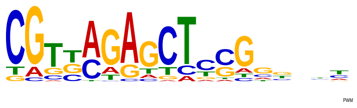	$NNNNNG\overrightarrow{AGCTCC}GNNNNN$	6.07	M	19
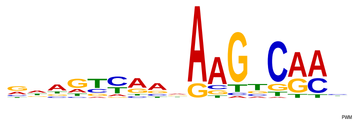	$N\overrightarrow{NNGCCA}AN\overrightarrow{NAGTCN}NN$	6.11	DR:2	20

282 TLX-Yin2017-Rnd3 (ERR1005202)

PWM	Seed Sequence	Seed Seq Enrichment	Repeat	Rank
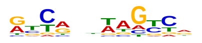 PWM	NNNNNNGTCANNNAGTCNNNNNN	7.11	DR:2	1
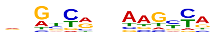 PWM	NNNNNNAAGTNNNNAAGCNNNNNN	5.12	M	2
PWM	NNNNNNNTCAANNNTCAANNNNNN	4.14	DR:1	3
PWM	NNNNNNTATTNNNTTCTNNNNNN	3.04	M	4
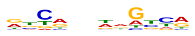 PWM	NNNNNNTCAANNGTCANNNNNN	5.48	DR:2	5
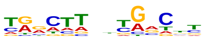 PWM	NNNNNNGCTTNNNNNCTTTNNNNNN	4.53	M	6
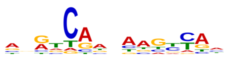 PWM	NNNNNNAGATNNNNAAGCNNNNNN	2.68	M	7
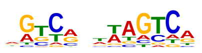 PWM	NNNNNNAGTCNNNTAGTCNNNNNN	9.09	DR:2	8
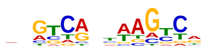 PWM	NNNNNNATGTCNNNNAGTCNNNNNN	7.25	DR:2	9

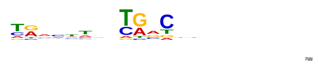	*NNNNNNGCTTNNNNNNNTTCTNNNNNN*	2.33	M	10
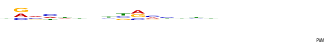	*NNNNNNTTATNNNNNTTTANNNNNN*	2.37	M	11
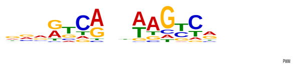	*NNNNNNGAATCNNNNAGTCNNNNNN*	5.75	M	12
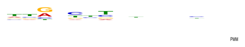	*NNNNNNAGTTNNNTTTTNNNNNN*	2.92	M	13
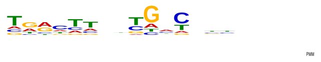	*NNNNNNCTTANNNATTTNNNNNN*	2.63	M	14
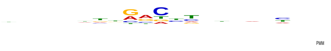	*NNNNNNGTTTNNNNTTTTNNNNNN*	2.57	M	15
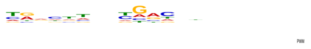	*NNNNNNGCTTNNNNNCTTGNNNNNN*	2.83	M	16
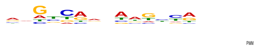	*NNNNNNTAAANNNNNNNAAGCNNNNNN*	2.47	M	17
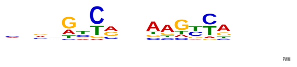	*NNNNNNCAAGTNNNNAAGCNNNNNN*	4.65	M	18

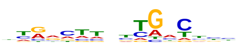	*NNNNNNGCTTNNNNATTCNNNNNN*	2.33	M	19
	NNNNNNCTTTNNNNNTTTTNNNNNN	2.57	M	20

283 TLX-Yin2017-Rnd4 (ERR1005204)

PWM	Seed Sequence	Seed Seq Enrichment	Repeat	Rank
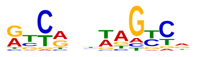	NNNNNNGTCANNNAGTCNNNNNN	9.37	DR:2	1
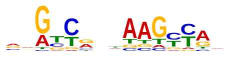	NNNNNNAAGTNNNNAAGCNNNNNN	7.79	M	2
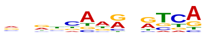	NNNNNNTCAANNNNTCAANNNNNN	5.80	DR:1	3
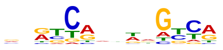	NNNNNNTCAANNNGTCANNNNNN	8.09	DR:2	4
	NNNNNNTTTTNNNTTTTNNNNNN	4.06	M	5
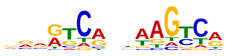	NNNNNNAATCNNNNAGTCNNNNNN	6.41	M	6
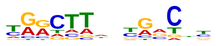	NNNNNNCTTANNNNCTTTNNNNNN	6.22	M	7
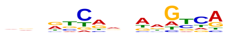	NNNNNNTCAANNNNTCAANNNNNN	5.74	DR:2	8
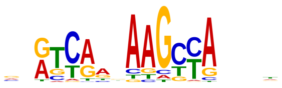	NNNNNNTCAAGNAGCTANNNNNN	16.13	DR:3	9

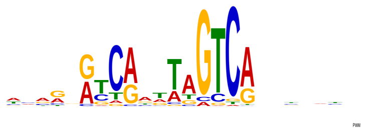	$NNN\overrightarrow{NNNTCA}AT\overrightarrow{TANTCA}CNNNNNN$	24.52	DR:2	10
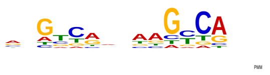	$NNNNNN\overrightarrow{ATGTNN}ATAANNNNNN$	3.59	M	11
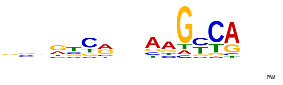	$NNNNNNC\overrightarrow{AGTNNA}TAANNNNNN$	3.16	M	12
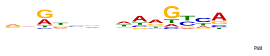	$NNNNNN\overrightarrow{AAGTNN}TAAANNNNNN$	3.57	M	13
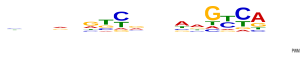	$NNNNNNCA\overrightarrow{AGNNNA}TAANNNNNN$	2.90	M	14
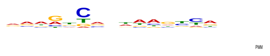	$NNNNNNTAAANNNNNAAAGNNNNNN$	3.50	M	15
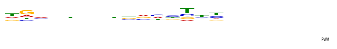	$NNNNNNTTTTNNNNTTTANNNNNN$	3.61	M	16
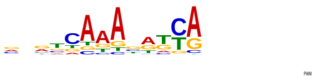	$NNN\overrightarrow{NNNTCA}A\overrightarrow{AGNTCA}ANNNNNN$	12.90	DR:1	17
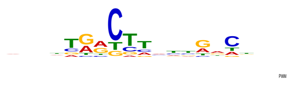	$NNNNNNTATTNNNNTATTNNNNNN$	3.60	M	18

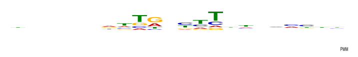	*NNNNNNAATTNNNTTTTNNNNNN*	4.41	M	19
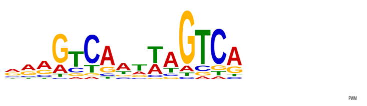	*NNNNNNAAT*$\overrightarrow{TANTCA}$*CNNNNNNN*	6.75	M	20

284 TLX-Yin2017-Rnd4 (ERR1005205)

PWM	Seed Sequence	Seed Seq Enrichment	Repeat	Rank
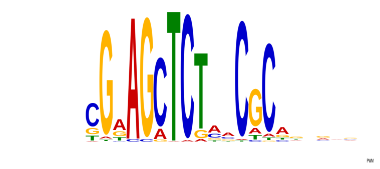	NNNNNNGAAGCTCTNNCGCANNNNNN	138.89	M	1
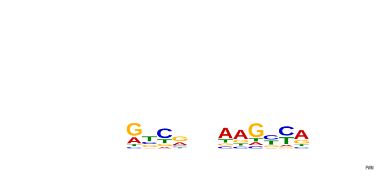	NNNNNNAGTCNNNAAGCNNNNNN	4.52	M	2
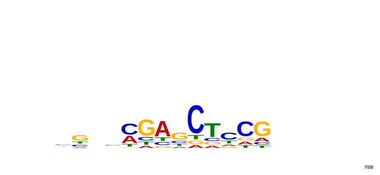	NNNNNNCGAGNTCCGNNNNNN	4.33	M	3
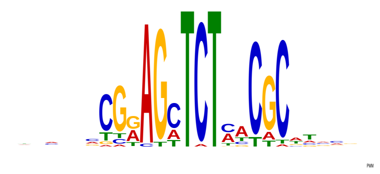	NNNNNNCGGAGNTCTCACGCNNNNNN	170.98	M	4
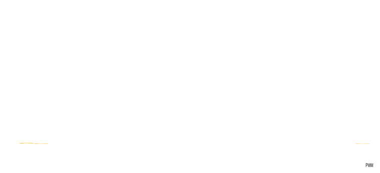	NNNNNNGGGGNNNNGGGGNNNNNN	2.78	M	5
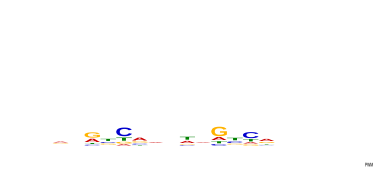	NNNNNNCAATNNGTCANNNNNN	3.43	M	6
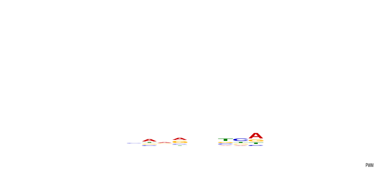	NNNNNNTCAANNNTCAANNNNNN	2.73	DR:1	7
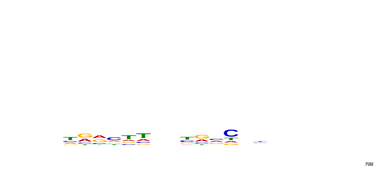	NNNNNNCTTANNNNCTTTNNNNNN	2.75	M	8
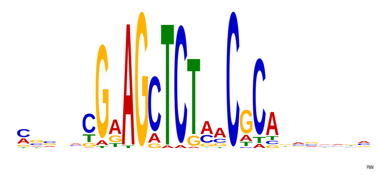	NNNNNNGAAGCTCTNNNGCAGNNNNNN	69.62	M	9

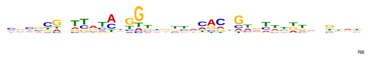	CGCGNNNNNNNNNNNNNNNNNNNNNNNNNNNNNNNNGTAT	1.98	M	10
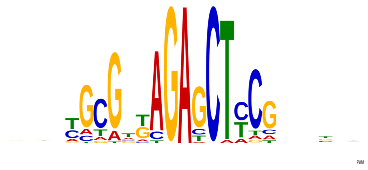	$NNNNN\overrightarrow{NCGTTA}G\overrightarrow{AGNTCC}GNNNNNN$	61.35	DR:1	11
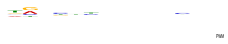	NNNNNNTATTNNNTTTTNNNNNN	2.38	M	12
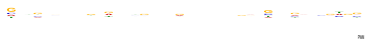	GATGNNNNNNNNNNNNNNNNNNNNNNNNNNNNNNNNNNGTGG	2.17	M	13
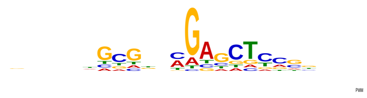	$NNNNNNGCGGNNN\overrightarrow{AGCTCN}NNNNN$	4.37	M	14
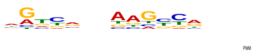	NNNNNNGAGTNNNTAAGNNNNNN	3.44	M	15
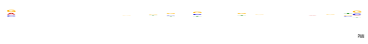	$GCGGNNNNNNNNNNNNNNNNNNNNNNNNNNNNNNNNN\overrightarrow{NGGTG}$	1.93	M	16
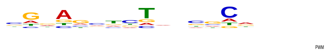	$NNNNN\overrightarrow{NGCTCN}AACGNNNNNN$	2.21	M	17
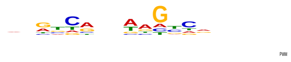	$NNNNN\overrightarrow{NGGTCN}NN\overrightarrow{NAGTCN}NNNNN$	3.55	DR:2	18

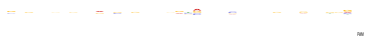	*GGGGNNNNNNNNNNNNNNNNNNNNNNNNNNNNNNN*$\overrightarrow{NGGGC}$	2.04	M	19
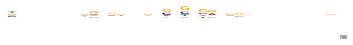	*NNNNGGGGNNNNNNNNNNNNNNNNNNNNNNNGGGGNNNN*	1.79	M	20

285 PNR-Bhimsaria2022-Rnd2 (S07-115)

PWM	Seed Sequence	Seed Seq Enrichment	Repeat	Rank
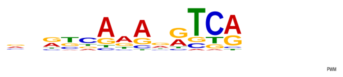	NNNNNCAAANGTCANNNNN	24.95	M	1
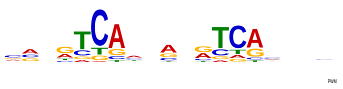	NNNNGTCANNNNNGTCANNNN	16.52	DR:2	2
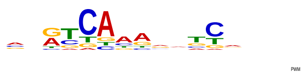	NNNNNATCAAAGAGNNNNN	6.09	M	3
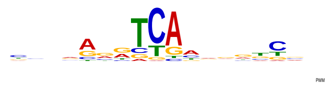	NNNNNNAGGTCAAANNNNNN	11.01	M	4
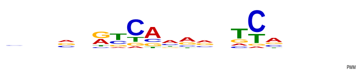	NNNNNNTCAAAGAGNNNNNN	4.04	M	5
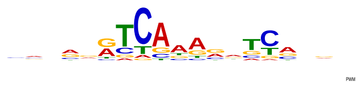	NNNNNGTCAAAGAGNNNNN	7.02	M	6
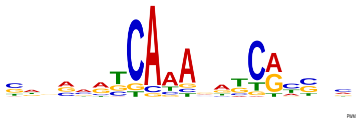	NNNNNATCAAAGAGCNNNNN	17.92	DR:1	7
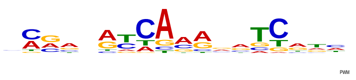	NNNNCATCAAAGAGNNNNN	5.83	M	8
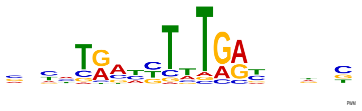	NNNNNGCTCTTTGACNNNNN	15.60	IR:1	9

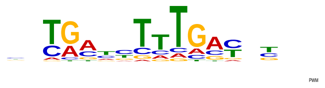	NNNNNGCTCTTTGANNNNNN	12.93	IR:1	10
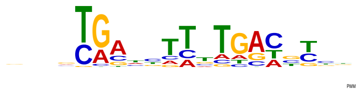	NNNNNGCTCTNTTGANNNNNN	7.56	IR:2	11
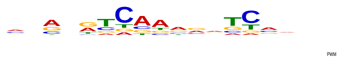	NNNNNGTCANAGAGNNNNN	3.51	M	12
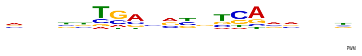	NNNNNTGACNNGTCANNNNN	9.12	M	13
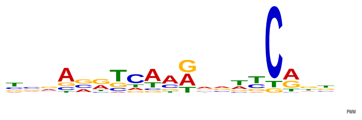	NNNNNATCAANAGAGNNNNN	3.93	M	14
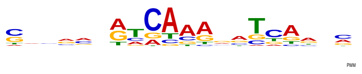	NNNNNCATCANAGAGNNNNN	3.59	M	15
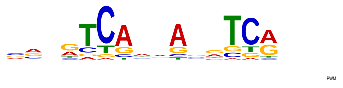	NNNNGTCANNAGGTCNNNN	20.89	DR:2	16
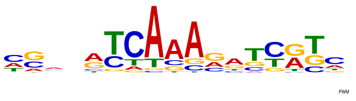	NNNCATCAAAGAGCNNN	15.58	DR:1	17
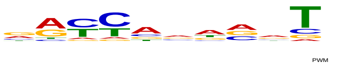	CGTCAAAGAG	3.99	M	18

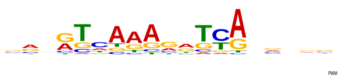	*NNNNNNCAAAGAGCNNNNNN*	5.71	M	19
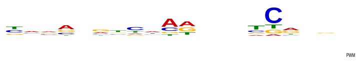	*NNNNNATCANNAGAGNNNNN*	1.69	M	20

286 PNR-Bhimsaria2022-Rnd3 (S07-110)

PWM	Seed Sequence	Seed Seq Enrichment	Repeat	Rank
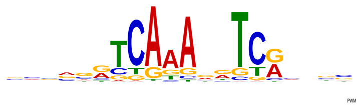	NNNNNGTCAAAGGTCNNNNN	271.27	DR:1	1
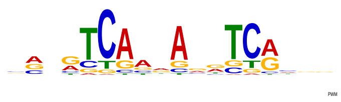	NNNNGTCANNAGGTCNNNN	77.94	DR:2	2
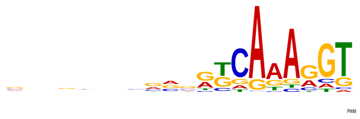	GGGGNNNNNNNNNCAAAGGT	344.06	M	3
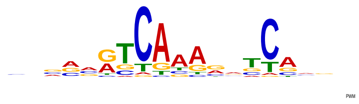	NNNNNATCAAAGAGNNNNN	17.93	M	4
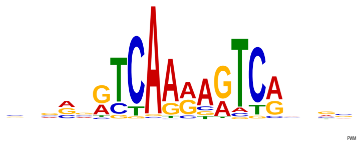	NNNNNGTCAAGAGTCNNNNN	231.16	DR:1	5
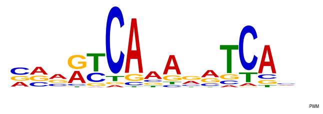	NNNNCATCAAAGAGNNNN	16.18	M	6
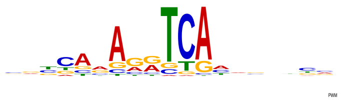	NNNNNNAAGGTCAANNNNNNN	42.15	M	7
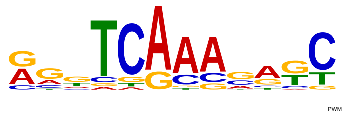	AGGTCAAAGAGC	259.84	DR:1	8
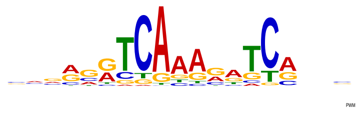	NNNNNGTCAAAGAGNNNNN	24.89	M	9

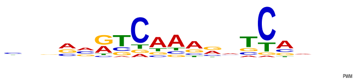	$NNNN\overrightarrow{NATCA}NAGAGNNNNN$	7.34	M	10
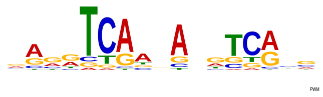	$NNN\overrightarrow{AGGTCN}NNN\overrightarrow{NGGTCN}NN$	28.84	DR:2	11
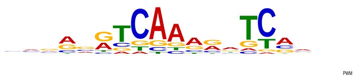	$NNNN\overrightarrow{NGTCA}NAGAGNNNNN$	10.50	M	12
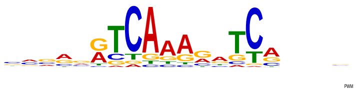	$NNN\overrightarrow{NNNTCA}AAGAGNNNNNN$	15.78	M	13
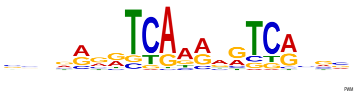	$NNNN\overrightarrow{AGGTCN}N\overrightarrow{NAGTCN}NNN$	56.81	DR:1	14

287 COUP-TF1:RXRA-Bhimsaria2022-Rnd3 (S13-133)

PWM	Seed Sequence	Seed Seq Enrichment	Repeat	Rank
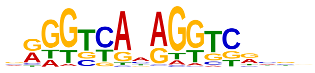	NNNNGGTCANAGGTCNNNN	73.53	DR:1	1
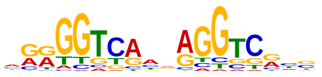	NNNNGGTCANNNGGTCNNNN	24.31	DR:2	2
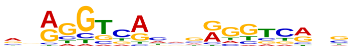	NNNGGTCNNNNNGGTCNNN	8.24	DR:3	3
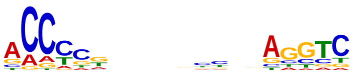	ACCCCNNNNNNNNNNNGGTC	21.14	ER:11	4
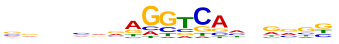	NNNNNNAAGGTCAANNNNNN	13.45	M	5
	CATANNNNNNNNNNNNGCTT	5.30	M	6
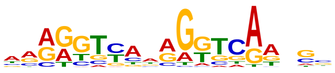	NNNNNGTCAGNGGTCNNNNN	12.68	DR:1	7
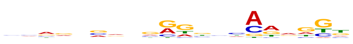	CCAGNNNNNNNNNNNNAGGT	5.62	M	8
	NNNGGTCNNNNNNGGTCNNN	4.04	DR:4	9

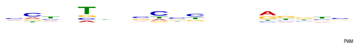	*CCTTNNNNNNNNNNNGGTC*	5.55	M	10
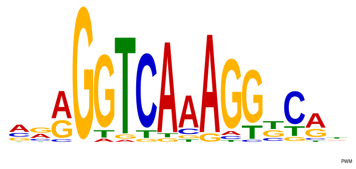	*NNAGGTCAAAGGGCNN*	156.44	DR:1	11
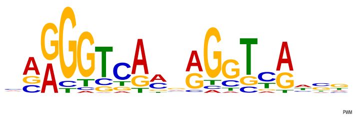	*NNNNGTCANGGGGTCNNNN*	20.57	DR:2	12
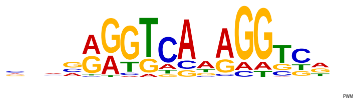	*NNNGGGGTCNGAGGTNNN*	26.38	DR:1	13
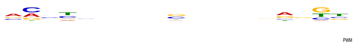	*ACCCNNNNNNNNNNNGGGT*	5.60	ER:11	14
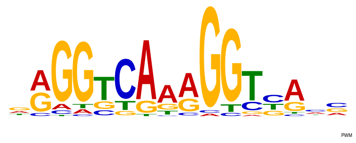	*NNGGTCNAAGGTTANN*	73.96	DR:1	15
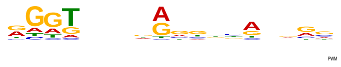	*NNNNNCAAGNGGTCNNNNN*	5.10	M	16

288 COUP-TF1-17-Bhimsaria2022-Rnd2 (S07-024)

PWM	Seed Sequence	Seed Seq Enrichment	Repeat	Rank
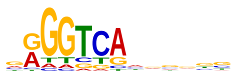	NNNNNNGGGGTCAANNNNNN	16.99	M	1
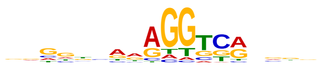	NNNNNNCAAAGGTCNNNNNN	12.75	M	2
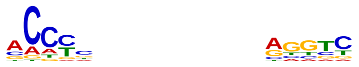	ACCCNNNNNNNNNNNNGGTC	14.65	ER:11	3
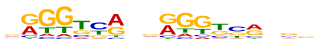	NNNNGGTCNNNNGGTCNNNN	9.24	DR:2	4
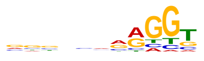	GGGGNNNNNNNNNNNNAGGT	8.63	M	5
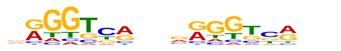	NNNGGTCNNNNNGGTCNNN	8.26	DR:3	6
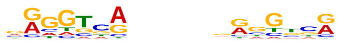	NNGGTCNNNNNNNNGGTCNN	5.77	DR:6	7
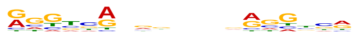	NGGTCNNNNNNNNNGGTCN	4.52	DR:7	8
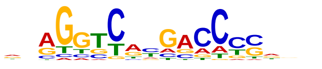	NNNNAGGTCNNGACCCNNNN	27.63	IR:0	9

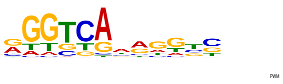	NNNNNGGTCAAAGGNNNNNN	25.10	DR:1	10
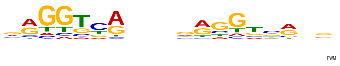	NNNGGTCNNNNNNGGTCNNN	6.15	DR:4	11
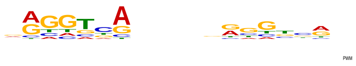	NNGGTCNNNNNNNGGTCNN	4.86	DR:5	12
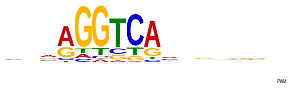	NNNNNNAAGGTCAANNNNNN	15.45	M	13
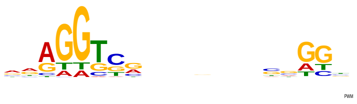	AAAGGTCNNNNNNNNNGGGT	42.20	DR:8	14
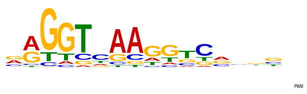	NNNNNGGTCAAGGTCNNNNN	29.14	DR:0	15
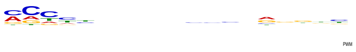	ACCTNNNNNNNNNNNGGTC	4.74	ER:10	16
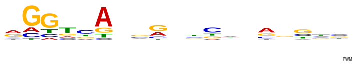	GGGTCNNNNNNNNNNGGTC	4.78	DR:8	17
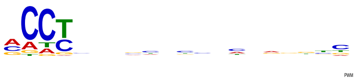	ACCTTNNNNNNNNNNNGGTC	13.93	ER:11	18

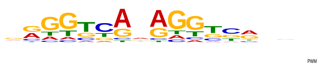	$NNN\overrightarrow{NGGTC}NN\overrightarrow{NGGTC}NNNN$	11.52	DR:1	19
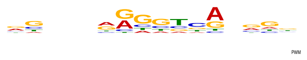	$NNNN\overrightarrow{NGTCG}\overrightarrow{NGGTC}NNNNN$	4.46	DR:0	20

289 COUP-TF1-17-Bhimsaria2022-Rnd3 (S07-144)

PWM	Seed Sequence	Seed Seq Enrichment	Repeat	Rank
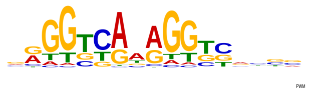	NNNNNGGTCANAGGTNNNNN	216.36	DR:1	1
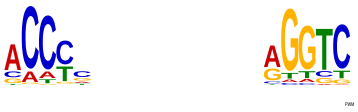	ACCCCNNNNNNNNNNNAGGTC	537.26	ER:11	2
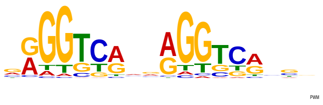	NNNNGGTCNNNNGGTCNNNN	89.78	DR:2	3
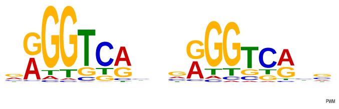	NNNGGTCNNNNNGGTCNNN	65.17	DR:3	4
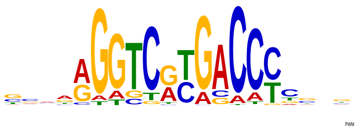	NNNNAGGTCGTGACCCNNNN	1653.05	IR:0	5
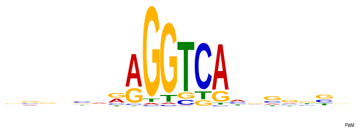	NNNNNNAAGGTCAANNNNNN	103.34	M	6
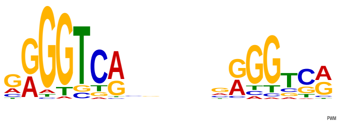	NGGGGTCNNNNNNNNGGTCN	205.26	DR:6	7
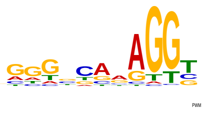	GGGGNNNNNNNNNCAAAGGT	414.41	M	8
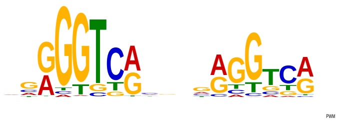	NNGGGGTCNNNNNNGGTCNN	127.31	DR:4	9

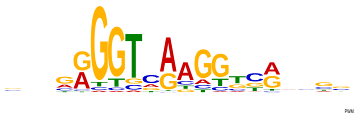	$NNN\overrightarrow{NNGGTCA}\overrightarrow{AGGTCN}NNNN$	168.36	DR:0	10
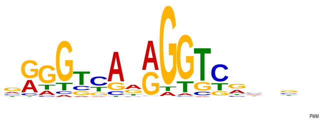	$NNN\overrightarrow{NNGTCA}G\overrightarrow{GGGTCN}NNNN$	173.36	DR:1	11
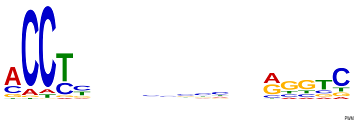	$\overleftarrow{ACCT}TNNNNNNNNNN\overrightarrow{NGGTC}$	126.72	ER:11	12
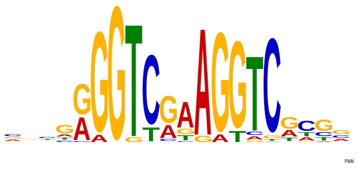	$NNNG\overrightarrow{GGGTCG}A\overrightarrow{AGGTCG}NNN$	4699.59	DR:1	13
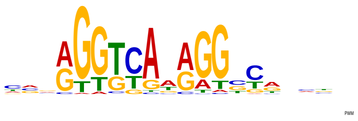	$NNN\overrightarrow{NGGTCA}A\overrightarrow{AGGCCA}NNNN$	805.23	DR:1	14
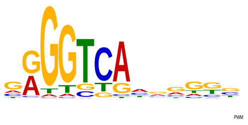	$NNNNNCG\overrightarrow{GGGTCA}ANNNNN$	127.46	M	15
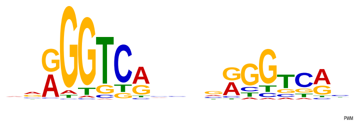	$NN\overrightarrow{AGGTCN}NNNN\overrightarrow{NGGTCN}N$	70.04	DR:4	16
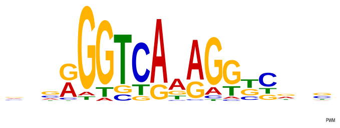	$NNNG\overrightarrow{GGGTCN}A\overrightarrow{AGGGCN}NN$	799.96	DR:1	17
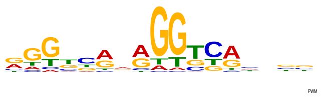	$NN\overrightarrow{NGGTCN}N\overrightarrow{NGGTCA}ANNN$	278.86	DR:1	18

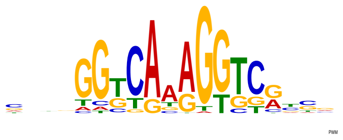	$NNNN\overrightarrow{CGGTCA}A\overrightarrow{AGGTCN}NNN$	741.89	DR:1	19

290 COUP-TF1-Jolma2013-Rnd2 (ERR194433)

PWM	Seed Sequence	Seed Seq Enrichment	Repeat	Rank
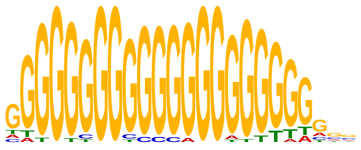	NNNNGGGGGGGGGGGGGGGGNNNN	3054.02	M	1
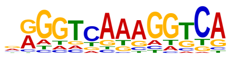	NNNNNNGGTCANAGGTNNNNNN	9.53	DR:1	2
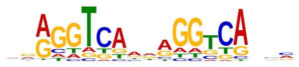	NNNNNNGGTCAANAGGTNNNNNN	11.31	DR:2	3
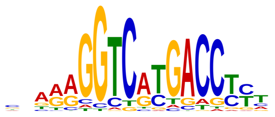	NNNNNNAGGTCANGACCTNNNNNN	35.74	IR:0	4
	NNNNNNGGGGNNNNNGGGGNNNNNN	3.42	M	5
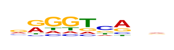	NNNNNNGGGGTCAANNNNNNN	3.77	M	6
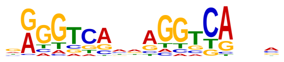	NNNNNNGGTCANAAGGTNNNNNN	9.74	DR:2	7
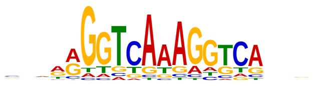	NNNNNNAGGTCNAAGGTNNNNNN	14.93	DR:1	8
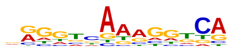	NNNNNNGGTCNNAGGTNNNNNN	3.89	DR:1	9

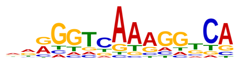	NNNNNNAGGTCNNAGGTNNNNNN	5.96	DR:1	10
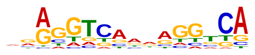	NNNNNNGGTCANNAGGTNNNNNN	6.13	DR:2	11
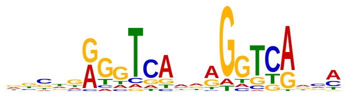	NNNNNNGGTCAAAAGGTNNNNNN	15.58	DR:2	12
	NNNNNNGGTCNNNNAGGTNNNNNN	2.25	DR:3	13
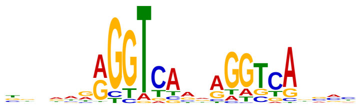	NNNNNNAGGTCAANAGGTNNNNNN	21.10	DR:2	14
	GGGGNNNNNNNNNNNNNNNNNNNNNNNGGGG	2.53	M	15
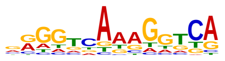	NNNNNNGGGTCNNAGGTNNNNNN	6.27	DR:1	16
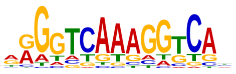	NNNNNNAGGTCANAGGTNNNNNN	14.59	DR:1	17
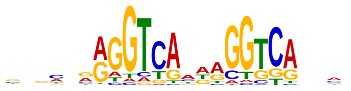	NNNNNNGGGTCAANAGGTNNNNNN	18.29	DR:2	18

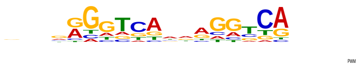	$NNNNN\overrightarrow{NGTCA}AN\overrightarrow{AGGTN}NNNNN$	5.14	DR:2	19
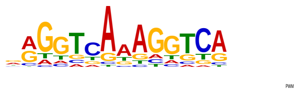	$NNNNNN\overrightarrow{GGTCN}A\overrightarrow{AGGTN}NNNNN$	8.47	DR:1	20

291 COUP-TF1-Jolma2013-Rnd3 (ERR193872)

PWM	Seed Sequence	Seed Seq Enrichment	Repeat	Rank
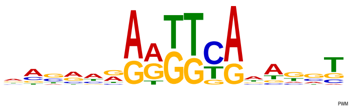	*NNNNAGAGNTCAANNNN*	5.81	M	1
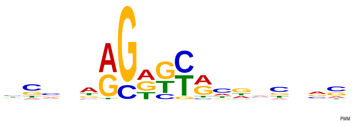	*NNNNNAGGTCACGNNNNN*	4.30	M	2
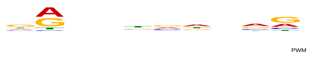	*GGGGNNNAAAG*	2.95	M	3
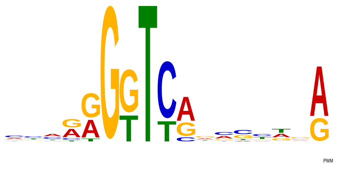	*NNNNNGGGTCAGCNNNNN*	3.19	M	4
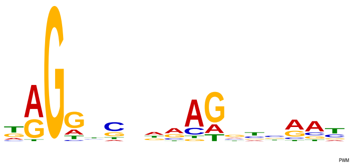	*NNNGTCANNNGGTCNNN*	3.07	DR:2	5
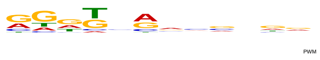	*NGGGTNNNCGGGN*	2.81	M	6
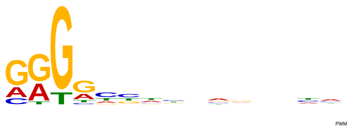	*NNNGTCANGGGGNNN*	1.69	M	7
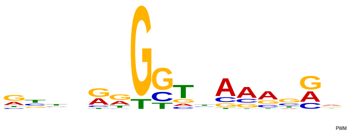	*NNNNGGGGTTAANNNN*	3.54	M	8
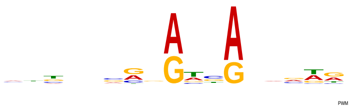	*NNNNCGGGNTCAANNNN*	3.41	M	9

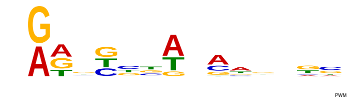	*NNNGGTCNAGGGNNN*	1.66	DR:0	10
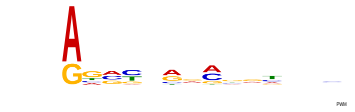	*NNNNGGTCNAAGGNNNN*	2.98	DR:1	11
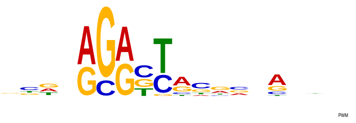	*NNNNNGGTCACGGNNNNN*	3.25	M	12
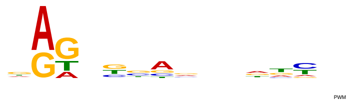	*NNGGTCNNAGGGNN*	1.78	DR:1	13
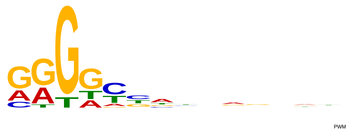	*NNGGTCANGGGGNN*	2.29	M	14
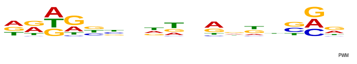	*NNNGGTCNNNGGGTNNN*	2.13	DR:2	15
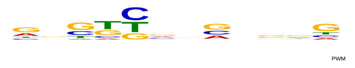	*GGGTNNNNCGGG*	2.56	M	16

292 COUP-TF1-Jolma2013-Rnd3 (ERR194434)

PWM	Seed Sequence	Seed Seq Enrichment	Repeat	Rank
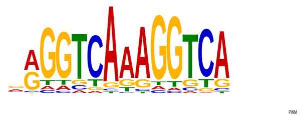	NNNNNNGGTCNNAGGTNNNNNN	27.96	DR:1	1
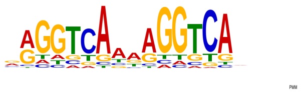	NNNNNNGGTCNNNAGGTNNNNNN	14.36	DR:2	2
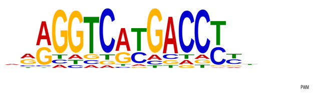	NNNNNNGGTCNNGACCNNNNNN	15.38	IR:0	3
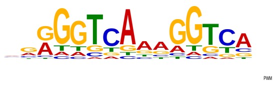	NNNNNNGGGGNCAAANNNNNN	9.58	M	4
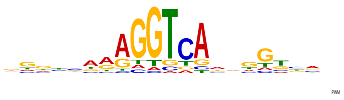	NNNNNNAAAGNNCAAANNNNNN	9.40	M	5
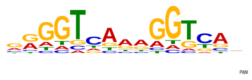	NNNNNNTGGGNNCAAANNNNNN	4.85	M	6
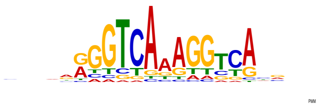	NNNNNNAAAGNNNNAAGGTNNNNNN	12.02	M	7
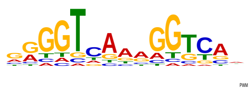	NNNNNNCGGGNNCAAANNNNNN	5.45	M	8
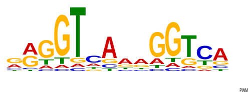	NNNNNNAGGGNNCAAANNNNNN	5.59	M	9

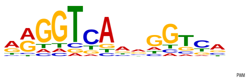	NNNNNNAGAGNNCAAANNNNNN	5.79	M	10
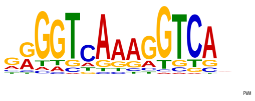	NNNNNNCGGGNNCAAAGNNNNNN	13.28	M	11
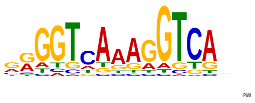	NNNNNNTGGGNNCAAAGNNNNNN	11.42	M	12
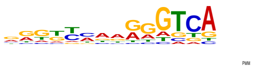	NNNNNNGGGGNNNAAAGNNNNNN	5.16	M	13
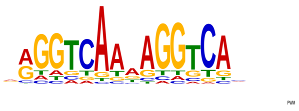	NNNNNNGGTCNNTAGGTNNNNNN	11.50	DR:2	14
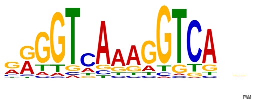	NNNNNNAGGGNNCAAAGNNNNNN	13.64	M	15
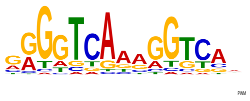	NNNNNNCGGGNTCAAANNNNNN	13.84	M	16
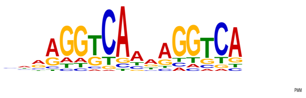	NNNNNNAAGGTNNNNAGGTNNNNNN	14.05	DR:2	17
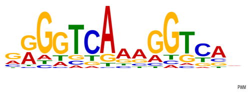	NNNNNNTGGGNTCAAANNNNNN	11.09	M	18

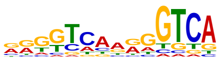 PWM	$NNNNNN\overrightarrow{AGGGNN}NAAAGNNNNNN$	5.84	M	19
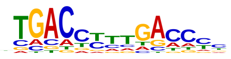 PWM	$NNNNNNCTTTNNNCCCGNNNNNN$	5.32	M	20

293 COUP-TF1-Jolma2013-Rnd4 (ERR193873)

PWM	Seed Sequence	Seed Seq Enrichment	Repeat	Rank
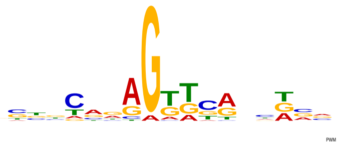	NNNNNAGAGTTCANNNNN	4.59	M	1
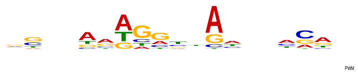	NNNNAAAGNNNATGTNNNN	3.53	M	2
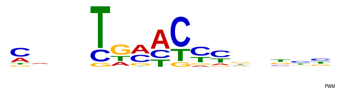	NNNTTTGNNNCCGTNNN	2.29	M	3
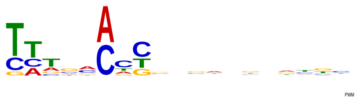	TTTGNNNNNNNNNNNATGC	3.57	M	4
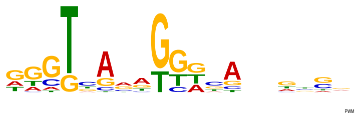	NNNNCAAANNNCAGTNNNN	3.98	M	5
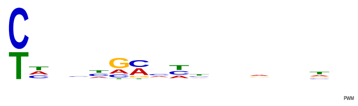	NNTTTGNNNNNTACANN	2.22	M	6
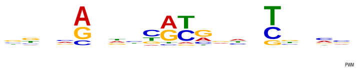	NNNNNGGTCNNGACCNNNNN	1.54	IR:0	7
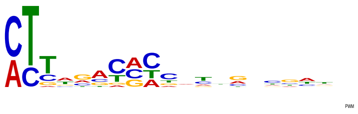	NTTTGNNNNNNNNNNCGATN	3.76	M	8
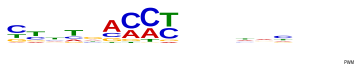	NTTTGNNNNNNNNACACN	3.10	M	9

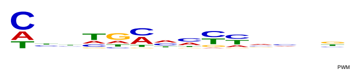	*NTTTGNNNNTACAN*	2.71	M	10
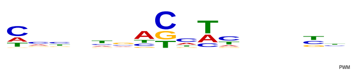	*NNTTTGNNNNTTCGNN*	1.91	M	11
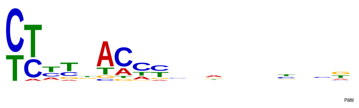	*NTTTGNNNNNNNNNATACN*	2.79	M	12
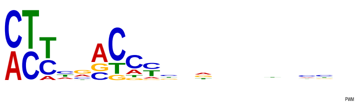	*NTTTGNNNNNNNNNTTCCN*	2.88	M	13
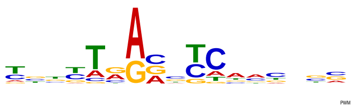	*NNTTTGNNNNCAACANN*	3.89	M	14
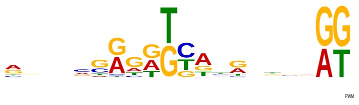	$NNNNNG\overrightarrow{GGGNNA}CGCNNNNN$	2.46	M	15
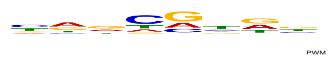	$C\overrightarrow{AGCGTA}G$	2.83	M	16
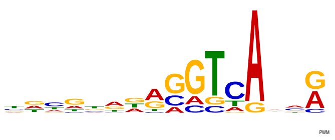	$NNGCGTAN\overrightarrow{NNNTCA}ANN$	5.39	M	17
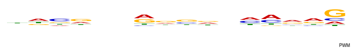	*TACGNNNNNNNNAAAG*	3.00	M	18

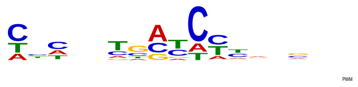	$NNNTTTGNNNCCGCNNN$	2.26	M	19
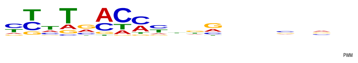	$NTTTGNNNNNNN\overrightarrow{NNGCCA}N$	2.59	M	20

294 COUP-TF1-Jolma2013-Rnd4 (ERR194435)

PWM	Seed Sequence	Seed Seq Enrichment	Repeat	Rank
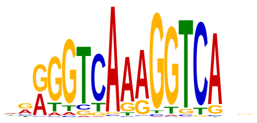	NNNNNNGTCAANNNTCACNNNNNN	48.84	DR:1	1
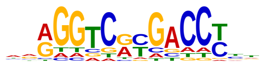	NNNNNNGGTCNNGACCNNNNNN	8.94	IR:0	2
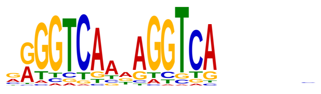	NNNNNNGTCANNNNGTCACNNNNNN	18.94	DR:2	3
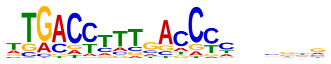	NNNNNNTTTGNNNCCGANNNNNN	5.90	M	4
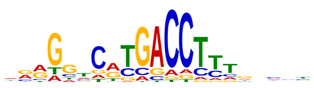	NNNNNNGTCATNNNCTTNNNNNN	11.29	IR:0	5
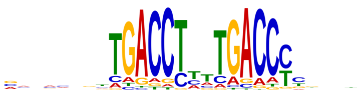	NNNNNNGTTGANNNNNNGACCNNNNNN	15.26	IR:5	6
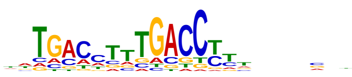	NNNNNNCTTTNNNNTTTTNNNNNN	6.30	M	7
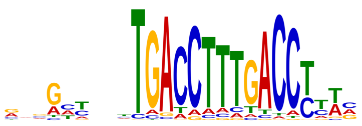	NNNNNNAGCTGANNNTTGANNNNNN	32.51	IR:4	8
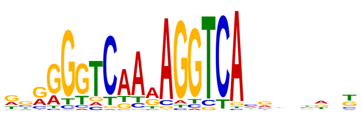	NNNNNNTCAAAANNNCACGNNNNNN	27.12	DR:2	9

	NNNNNNTTTGNNNNCAAANNNNNN	2.48	M	10
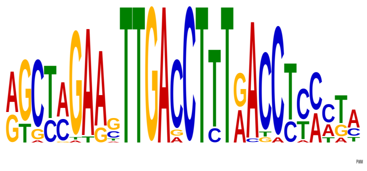	NNNNNGAAGTTGANNNNNNACCTNNNNN	127.43	IR:4	11
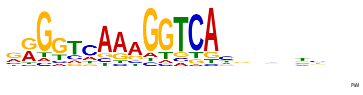	NNNNNNAAAGNNNNACGGNNNNNN	6.82	M	12
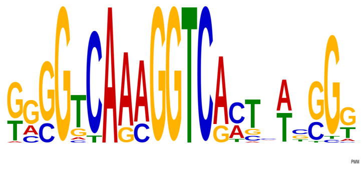	NNNGTCAANNNTCAATCTCNNN	119.26	M	13
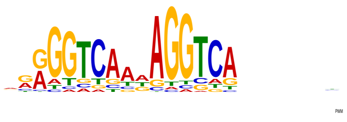	NNNNNNTCAANNNNTCATNNNNNN	9.06	DR:2	14
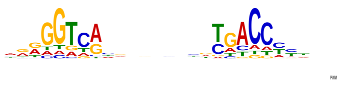	NNNNNGGTCNNNNNNNNNNNGACCNNNNN	6.42	IR:9	15
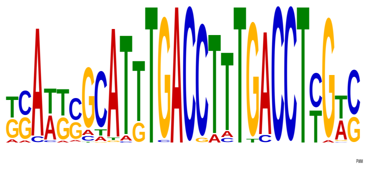	NNNNNNGCATGTGANNNNTGACNNNNNN	134.43	IR:4	16
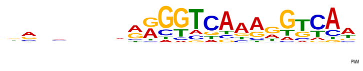	NNNNNNTAGANNNCAAANNNNNN	4.89	M	17
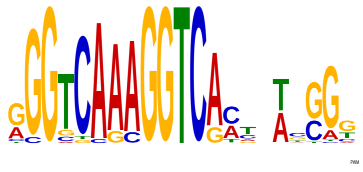	NNNTCAANNNTCAATCTCNNN	57.64	M	18

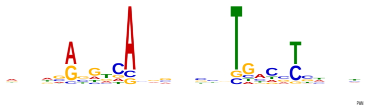	$NNNNN\overrightarrow{NGGTCN}NNNNNNNN\overleftarrow{NGACCN}NNNNN$	4.05	IR:8	19
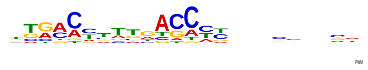	NNNNNNTTTGNNNNCTAANNNNNN	3.46	M	20

295 COUP-TF1-Jolma2013-Rnd5 (ERR194436)

PWM	Seed Sequence	Seed Seq Enrichment	Repeat	Rank
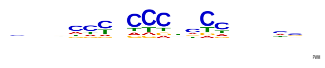	NNNNNNTCCCNNNNTCCCNNNNNN	4.19	M	1
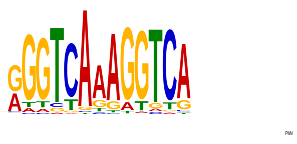	NNNNNNGGTCNNNNNTCACNNNNNN	16.96	DR:1	2
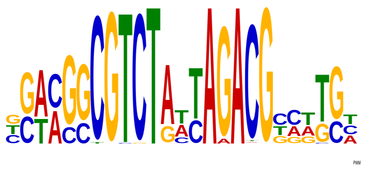	NNNNNNCGTCTANNAGACGNNNNN	106.09	IR:3	3
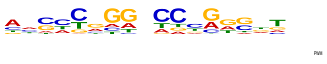	NNNNNNTAGGNCCTANNNNNN	4.06	M	4
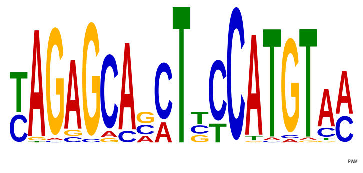	NAGAGCANNNNNNATGTAN	21.64	DR:6	5
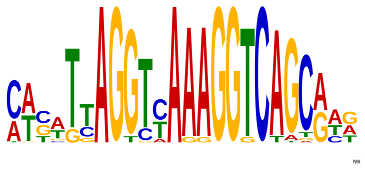	NNNATTAGGTNNNNNNTCAGCNNN	46.91	DR:1	6
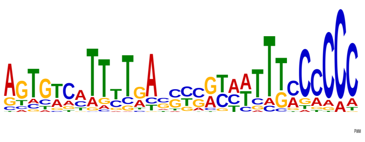	AGTGNNNNNNNNNNNNNNNNNNNNNCCCCC	6.23	M	7
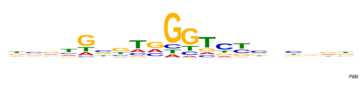	NNNNNNGTGGGTCTNNNNNN	3.85	M	8
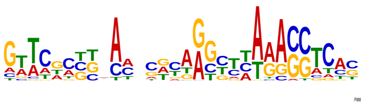	GTACNNNNNNNNNNNNNNNNNNNNNNTCAC	2.85	M	9

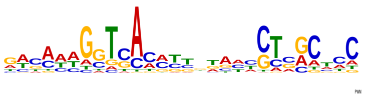	$\overrightarrow{GTCA}$NNNNNNNNNNNNNNNNNNNNNCTCC	2.99	M	10
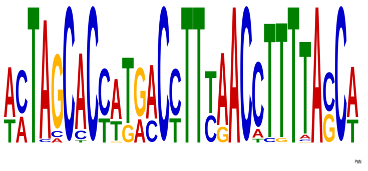	NNNAGCANNNNNNNNNNNNNNCTTTTANNN	39.86	M	11
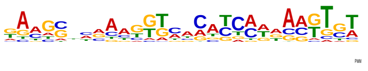	GAAGNNNNNNNNNNNNNNNNNN$\overrightarrow{NNGTCT}$	2.66	M	12
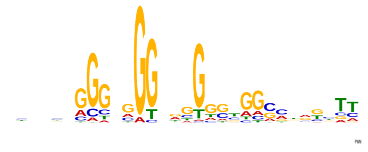	NNNNN$\overrightarrow{NGGGCG}$NNNNNNNN$\overrightarrow{NGGGCN}$NNNNN	5.64	DR:8	13
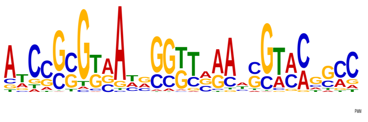	ATCCNNNNNNNNNNNNNNNNNNNNN$\overrightarrow{NGGCC}$	3.10	M	14
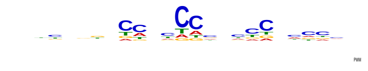	NNNNNNTCCCNNNNNNTCCCNNNNNNN	3.72	M	15
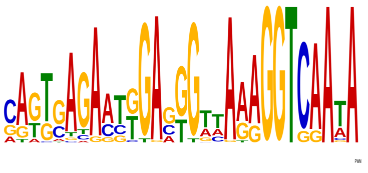	$\overrightarrow{NAGTGA}$GNNNNNNNNNNNNNNNNNCAATN	8.48	M	16
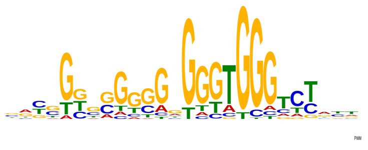	NNNGGGGNNNNNNNNNT$\overrightarrow{GGGTCT}$NNN	21.34	M	17
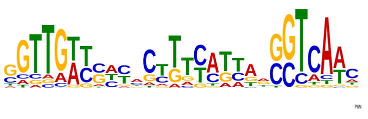	$\overrightarrow{GGTTG}$NNNNNNNNNNNNNNNNNNNNCAAC	4.83	M	18

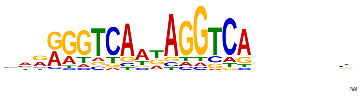	$NNN\overrightarrow{NNNTCA}AN\overrightarrow{NNNTCA}TNNNNNN$	6.37	DR:2	19
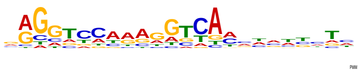	$NNNNNNAAAANNNN\overrightarrow{AAGTN}NNNNN$	4.03	M	20

296 COUP-TF1-Yin2017-Rnd2 (ERR1005208)

PWM	Seed Sequence	Seed Seq Enrichment	Repeat	Rank
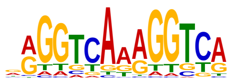	NNNNNNTCAANNGTCANNNNNNN	30.36	DR:1	1
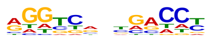	NNNNNNGGTCNNNGACCNNNNNN	13.92	IR:1	2
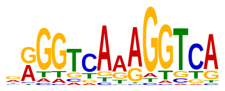	NNNNNNTCAANNNTCAANNNNNN	18.61	DR:1	3
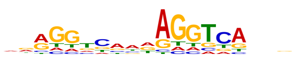	NNNNNNTCAANNNGTCANNNNNN	5.96	DR:2	4
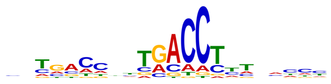	NNNNNNCTTTNNNNTTTGNNNNNNN	7.30	M	5
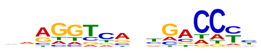	NNNNNNAGGTNNCTGANNNNNNN	6.50	IR:1	6
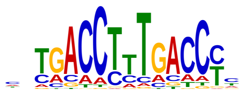	NNNNNNCGTGANNNTTGANNNNNNN	22.80	IR:4	7
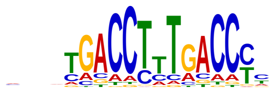	NNNNNNGATGANNNTTGANNNNNNN	15.93	IR:4	8
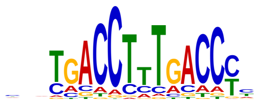	NNNNNNGCTGANNNTTGANNNNNNN	14.14	IR:4	9

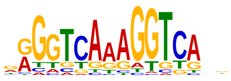 PWM	$NNN\overrightarrow{NNNTCA}AANNNNACGGNNNNNN$	12.24	M	10
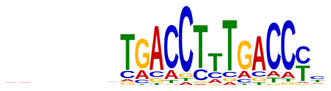 PWM	$NNNNN\overrightarrow{NGTTGA}NNNT\overleftarrow{TGANNN}NNN$	15.22	IR:4	11
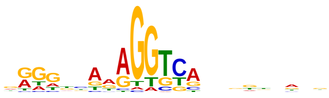 PWM	$NNN\overrightarrow{NNNTCA}AANNNNAAAGNNNNNN$	11.03	M	12
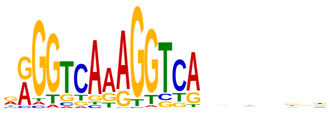 PWM	$NNN\overrightarrow{NNNTCA}A\overrightarrow{NNNTCA}AGCNNNNNN$	21.36	DR:1	13
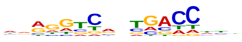 PWM	$NNNN\overrightarrow{NNGTCA}NNNNNCTTTNNNNNN$	6.94	M	14
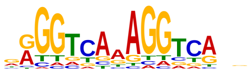 PWM	$NNN\overrightarrow{NNNTCA}ANNNTCGCNNNNNN$	5.71	M	15
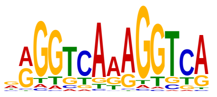 PWM	$NNN\overrightarrow{NNNTCA}A\overrightarrow{NNNTCA}AGNNNNNN$	24.79	DR:1	16
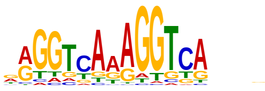 PWM	$NNN\overrightarrow{NNNTCA}A\overrightarrow{NNNTCA}GANNNNNN$	15.05	DR:1	17
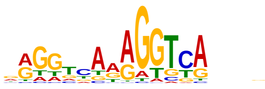 PWM	$NNN\overrightarrow{NNNTCA}A\overrightarrow{NNNTCA}AANNNNNN$	15.70	DR:1	18

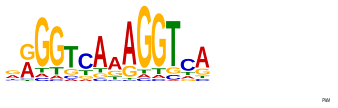	$NNN\overrightarrow{NNNTCA}ANNNNCAAGNNNNNN$	9.02	M	19
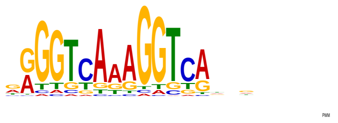	$NNN\overrightarrow{NNNTCA}A\overrightarrow{ANNNCA}GCNNNNNN$	12.16	DR:1	20

297 COUP-TF1-Yin2017-Rnd2 (ERR1005209)

PWM	Seed Sequence	Seed Seq Enrichment	Repeat	Rank
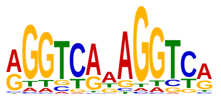	NNNNNNGGTCNNAGGTNNNNNN	33.11	DR:1	1
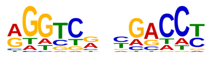	NNNNNNGGTCNNNGACCNNNNNN	26.49	IR:1	2
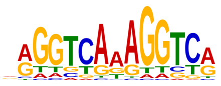	NNNNNNNGTCANNNGTCANNNNNN	26.11	DR:1	3
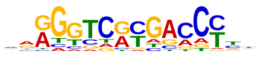	NNNNNNGGTCNNGACCNNNNNN	8.52	IR:0	4
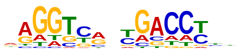	NNNNNNAAGGNNNNNTGACNNNNNN	13.94	IR:1	5
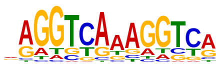	NNNNNNAAGGNNNAAGGNNNNNN	19.17	DR:1	6
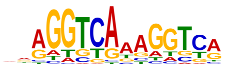	NNNNNNAAAGNNNNAAGGNNNNNN	11.54	M	7
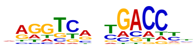	NNNNNNAAGGNNNNNNNNCTTTNNNNNN	6.99	M	8
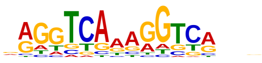	NNNNNNGGGGNNNAAGGNNNNNN	10.66	M	9

298 COUP-TF1-Yin2017-Rnd3 (ERR1005210)

PWM	Seed Sequence	Seed Seq Enrichment	Repeat	Rank
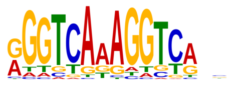	NNNNNNGGTCNNNNGTCANNNNNN	28.95	DR:1	1
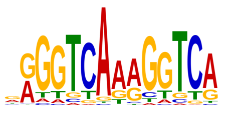	NNNNNNTCAANNNTCATNNNNNN	31.71	DR:1	2
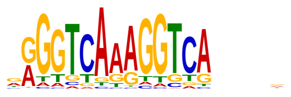	NNNNNNTCAANNNNCAAGNNNNNN	20.53	M	3
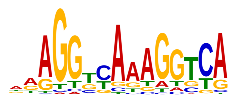	NNNNNNGAAANNNNAAAGNNNNNN	13.73	M	4
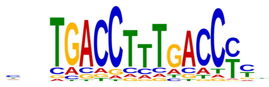	NNNNNNCGATNNNNTTTGNNNNNN	9.25	M	5
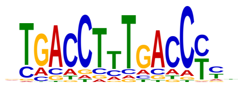	NNNNNNCCGTNNNNTTTGNNNNNN	10.49	M	6
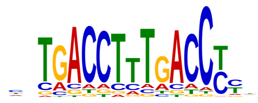	NNNNNNGCTTNNNNTTTGNNNNNN	9.88	M	7
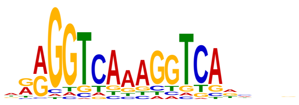	NNNNNNCAAANNNNAGCTNNNNNN	5.61	M	8
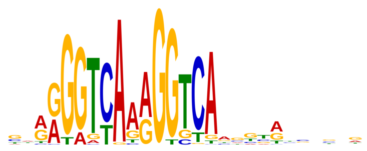	NNNNNNTCAANNNNCAAGGTANNNNNN	57.28	DR:7	9

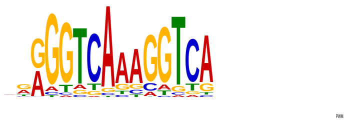	$NNN\overrightarrow{NNNTCA}ANNNNN\overrightarrow{CAGGAN}NNNNN$	15.96	DR:6	10
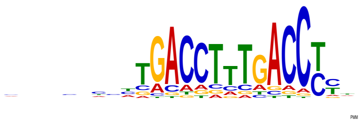	$NNNNNN\overrightarrow{AGTTN}NNNTTTGNNNNNN$	5.87	M	11
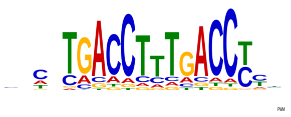	NNNNNNGCGTNNNNTTTGNNNNNN	8.71	M	12
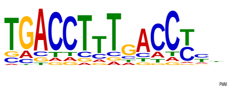	NNNNNNTATTNNNNTTTGNNNNNN	6.45	M	13
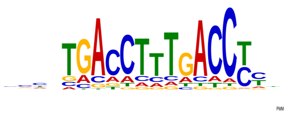	NNNNNNGTTTNNNNTTTGNNNNNN	6.29	M	14
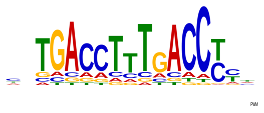	NNNNNNGATTNNNNTTTGNNNNNN	6.15	M	15
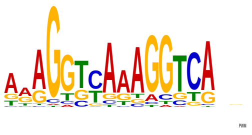	$NNNN\overrightarrow{NNGTAA}ANNNNAAAGNNNNNN$	16.40	M	16
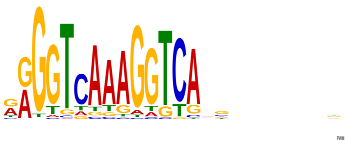	$NNNNNNCAAANNNN\overrightarrow{AAGTAN}NNNNN$	13.51	M	17
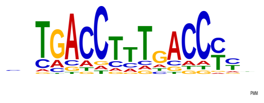	NNNNNNTCGTNNNNTTTGNNNNNN	7.44	M	18

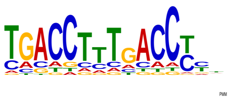	*NNNNNNCTGTNNNNNTTTGNNNNNN*	6.61	M	19
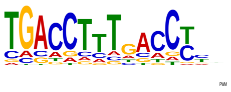	*NNNNNNCAATNNNNNTTTGNNNNNN*	9.14	M	20

299 COUP-TF1-Yin2017-Rnd3 (ERR1005211)

PWM	Seed Sequence	Seed Seq Enrichment	Repeat	Rank
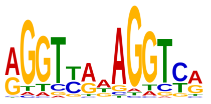	NNNNNNAGGTNNNAGGTNNNNNN	31.36	DR:1	1
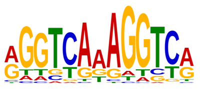	NNNNNNGGTCNNNNGTCANNNNNN	30.60	DR:1	2
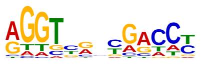	NNNNNNAGGTNNNNGACCNNNNNN	17.62	IR:1	3
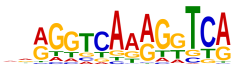	NNNNNNTCAANNNTCAANNNNNN	22.31	DR:1	4
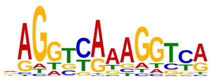	NNNNNNAAAGNNNNAAGGNNNNNN	18.37	M	5
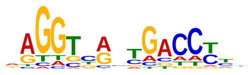	NNNNNNAAGGNNNNCTGANNNNNN	11.73	IR:1	6
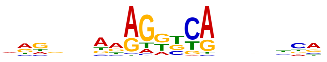	NNNNNNCAAANNNCAAANNNNNN	11.58	M	7
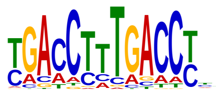	NNNNNNTGTGANNNTTGANNNNNN	24.90	IR:4	8
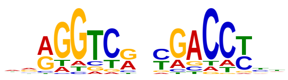	NNNNNNGTCGNCGACNNNNNN	10.38	IR:1	9

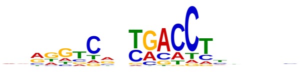 PWM	$NNNN\overrightarrow{NNGTCG}NNNNNNCTTTNNNNNN$	8.12	M	10
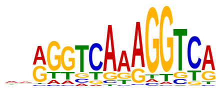 PWM	$NNN\overrightarrow{NNNTCA}ANNNNNCAACGNNNNNN$	16.76	M	11
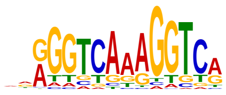 PWM	$NNN\overrightarrow{NNNTCA}ANNNNNCACGNNNNNN$	13.71	M	12
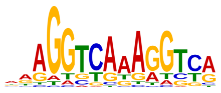 PWM	$NNNNNNGAAANNNNNA\overrightarrow{AGGNNN}NNN$	10.70	M	13
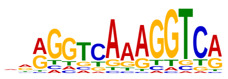 PWM	$NNN\overrightarrow{NNNTCA}ANNNNNCAGANNNNNN$	8.50	M	14
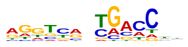 PWM	$NNNNNNA\overrightarrow{AGGNNN}NNNNNNCTTTNNNNNN$	7.42	M	15
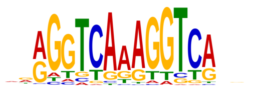 PWM	$NNN\overrightarrow{NNNTCA}ANNNNNCAAGCNNNNNN$	16.96	M	16

300 COUP-TF1-Yin2017-Rnd4 (ERR1005212)

PWM	Seed Sequence	Seed Seq Enrichment	Repeat	Rank
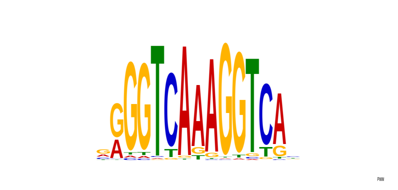	NNNNNNGGGTNNNNNNTCACNNNNNN	20.05	DR:1	1
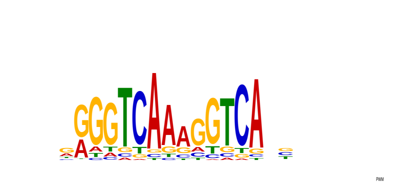	NNNNNNTCAANNNNCAACNNNNNN	15.39	M	2
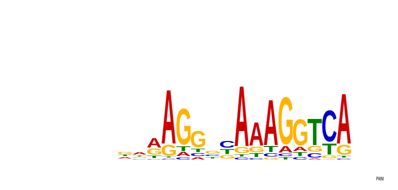	NNNNNNGAAANNNNAAAGNNNNNN	12.67	M	3
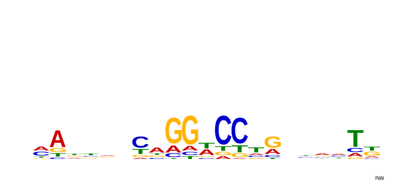	NNNNNNCAGGNCCTGNNNNNN	8.20	M	4
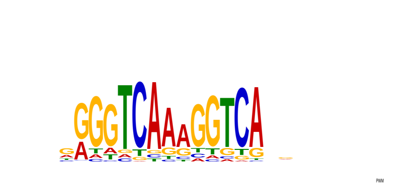	NNNNNNTCAANNNNCACGNNNNNN	19.13	M	5
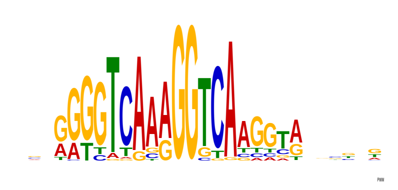	NNNNNNTCAANNNNCAAGGTANNNNNN	121.26	DR:7	6
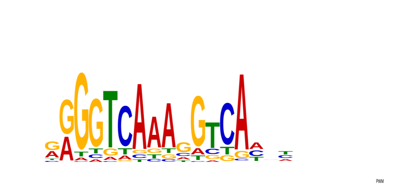	NNNNNNCAAANNNNAAGTNNNNNN	11.56	M	7
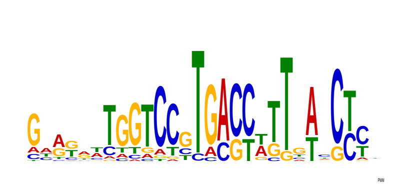	NNNNNNTGGTCCNNNNNNTTTGNNNNNN	34.58	M	8
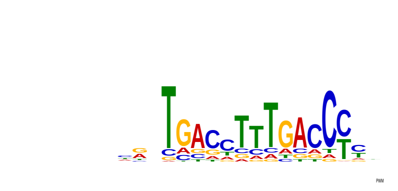	NNNNNNCGTTNNNNTTTGNNNNNN	9.56	M	9

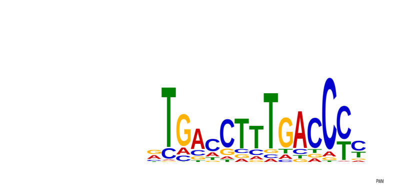	$NNNNNNCCGTNNNNTTTGNNNNNN$	9.65	M	10
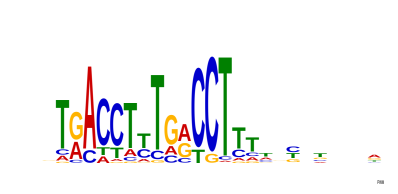	$NNNNNNCTTTNNNNTTTTGCNNNNNN$	26.91	M	11
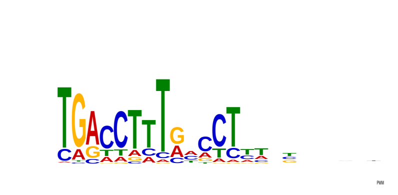	$NNNNNNCTTTNNNNTTTGTNNNNNN$	11.02	M	12
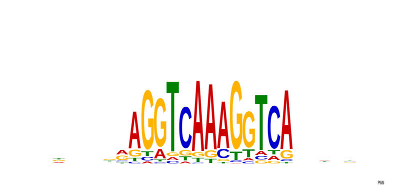	$NNNNNNGAAGNNNNN\overrightarrow{NNNTCA}GNNNNNN$	6.48	M	13
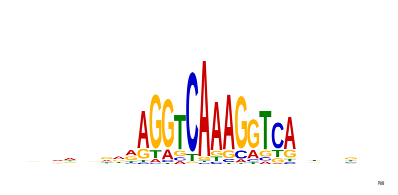	$NNNNNNGAAANNNNNNN\overrightarrow{NNNTCA}GNNNNNN$	5.66	M	14
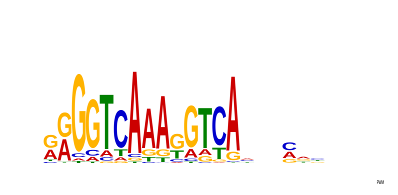	$NNNNNNCAAANNNNA\overrightarrow{AGGCN}NNNNN$	11.26	M	15
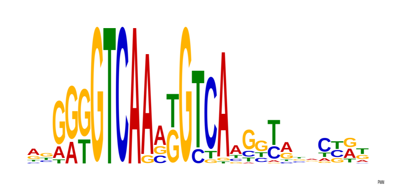	$NNN\overrightarrow{NNNTCA}ANNNNNCA\overrightarrow{AGGTGT}NNNNNN$	90.73	DR:7	16
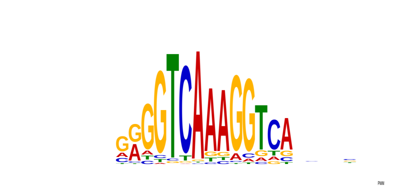	$NNNNNNCGGGNNNNN\overrightarrow{NNNTCA}GNNNNNN$	5.52	M	17
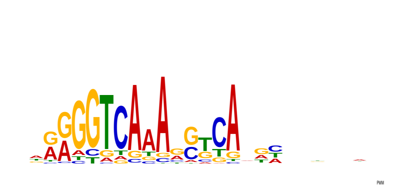	$NNNNNNCAAANNNNA\overrightarrow{AGCTN}NNNNN$	10.91	M	18

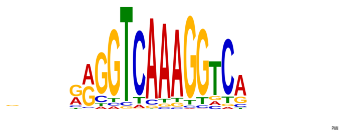	*NNNNNNCGAGNNNNNNNNTCATNNNNNN*	6.01	M	19
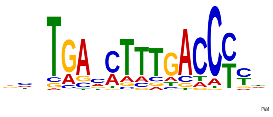	*NNNNNNGTTTNNNNTTTGNNNNNN*	7.06	M	20

301 COUP-TF1-Yin2017-Rnd4 (ERR1005213)

PWM	Seed Sequence	Seed Seq Enrichment	Repeat	Rank
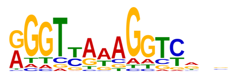	NNNNNNAGGTNNNNGGTCNNNNNN	14.24	DR:1	1
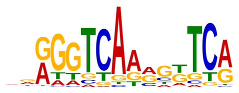	NNNNNNTCAANNNTCATNNNNNN	15.23	DR:1	2
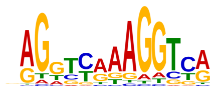	NNNNNNAGAGNNNNAAGGNNNNNN	13.62	M	3
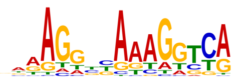	NNNNNNGAAANNNNAAAGNNNNNN	14.73	M	4
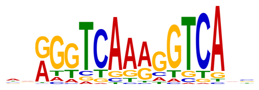	NNNNNNTCAANNNNCAACNNNNNN	14.82	M	5
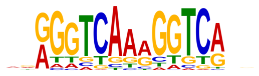	NNNNNNTCAANNNNCACGNNNNNN	14.62	M	6
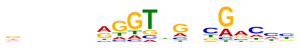	NNNNNNAGGTNNNCGACNNNNNN	5.86	IR:1	7
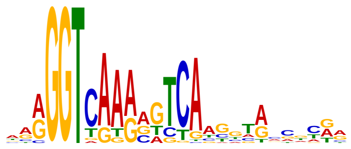	NNNNNNTCAANNNNCAAGGTANNNNNN	64.56	DR:7	8
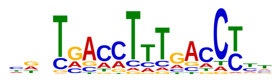	NNNNNNCGTTNNNNTTTGNNNNNN	9.68	M	9

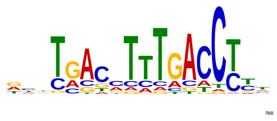	NNNNNNGCTTNNNNTTTGNNNNNN	8.87	M	10
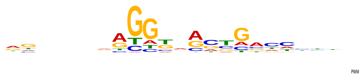	NNNNNNAAGGNNNCTGANNNNNN	5.86	IR:1	11
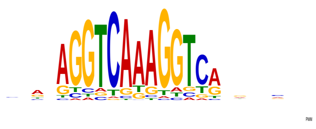	NNNNNNATAGNNNNNNNNTCACNNNNNN	6.60	M	12
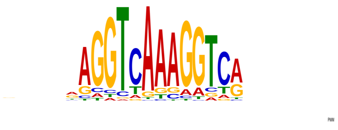	NNNNNNGAAGNNNNNNNNTCATNNNNNN	6.77	M	13
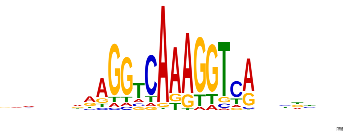	NNNNNNGAAANNNNNNNNNNTCAGNNNNNN	4.93	M	14
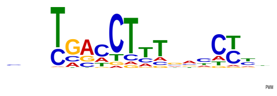	NNNNNNCAGTNNNNTTTGNNNNNN	7.14	M	15
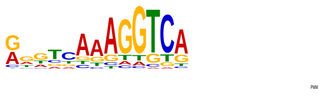	NNNNNNTCAANNNNNACTGNNNNNN	5.89	M	16
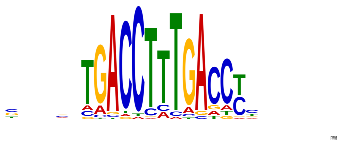	NNNNNNGTTGNNNNNNNNCCCCNNNNNN	5.06	M	17
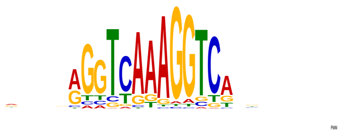	NNNNNNGAGGNNNNNNNNCAATNNNNNN	6.18	M	18

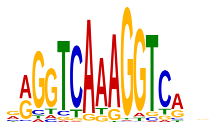	$NNNNNNGGGGNNNNNNNNNCACGNNNNNN$	5.52	M	19
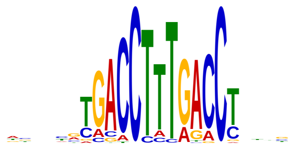	$NNNNN\overrightarrow{NTGTGA}NNNNNNNNNCTCTNNNNNN$	11.31	M	20

302 COUP-TF1-Yin2017-Rnd2 (ERR1005216)

PWM	Seed Sequence	Seed Seq Enrichment	Repeat	Rank
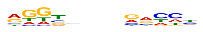	NNNNNNAGGTNNNNGACCNNNNNN	9.13	IR:1	1
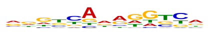	NNNNNNGTCANNNGTCANNNNNN	7.26	DR:1	2
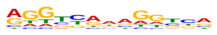	NNNNNNAGGTNNAAGGNNNNNN	7.12	DR:1	3
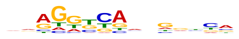	NNNNNNAAAGNNNAAAGNNNNNN	5.58	M	4
	NNNNNNGTCANNAGGTNNNNNN	3.71	DR:2	5
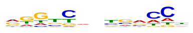	NNNNNNGTCANNNNNNCTTTNNNNNN	4.79	M	6
	NNNNNNGGTCNNGACCNNNNNN	3.28	IR:0	7
	NNNNNNGTCANNNNNNNNNTGACNNNNNN	2.77	IR:9	8
	NNNNNNGTCANNNNNNNNTGACNNNNNN	3.06	IR:8	9

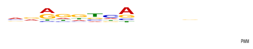	NNNNNNCAGGNNTCAANNNNNN	3.11	M	10
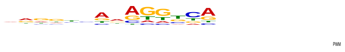	NNNNNNCAAANNNCAAGNNNNNN	3.71	M	11
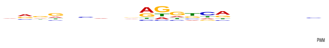	NNNNNNTCAANNNGTCANNNNNN	3.46	DR:2	12
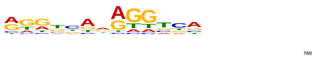	NNNNNNTCAANNNTCAGNNNNNN	4.11	DR:1	13
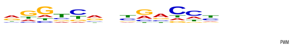	NNNNNNGTCGNCGACNNNNNN	4.33	IR:1	14
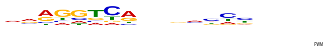	NNNNNNGTCANGCACNNNNNN	3.19	M	15
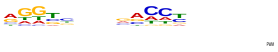	NNNNNNAGGTNNNNNACCTNNNNNN	8.69	IR:1	16
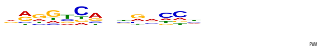	NNNNNNGTCANNNNNCTCCNNNNNN	3.35	M	17
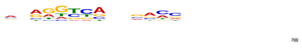	NNNNNNAAGGNNNNNNNNCTTANNNNNN	2.85	M	18

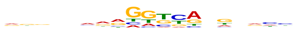	NNNNNNAAAGNNNACGCNNNNNN	2.77	M	19
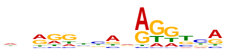	NNNNNNTCAANNNTCAAGNNNNNN	4.99	DR:1	20

303 COUP-TF1-Yin2017-Rnd2 (ERR1005217)

PWM	Seed Sequence	Seed Seq Enrichment	Repeat	Rank
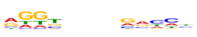 PWM	NNNNNNAGGTNNNNGACCNNNNNN	7.75	IR:1	1
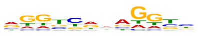 PWM	NNNNNNGGTCNNAGGTNNNNNN	6.94	DR:1	2
PWM	NNNNNNGTCANNNGTCANNNNNN	5.16	DR:1	3
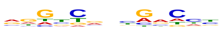 PWM	NNNNNNGTCGNCGACNNNNNN	4.76	IR:1	4
PWM	NNNNNNGTCANNNGGTCNNNNNN	3.17	DR:2	5
PWM	CGGGNNNNNNNNNNNNNNNNNNNNNNNNNNNNNNNNGGGG	2.44	M	6
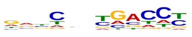 PWM	NNNNNNGGCCNNNGACCNNNNNN	5.89	IR:1	7
PWM	NNNNNNAGGTNNCGACNNNNNN	2.95	IR:0	8
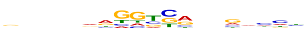 PWM	NNNNNNAAGGNNACTGNNNNNN	3.27	M	9

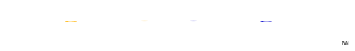	*NNNNNNGTCANNNNNNNNNTGACNNNNNN*	2.51	IR:9	10
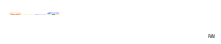	*NNNNNNGTCANNNNNNNNTGACNNNNNN*	2.41	IR:8	11
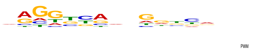	*NNNNNNAAAGNNNAAAGNNNNNN*	3.92	M	12
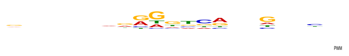	*NNNNNNCAGGNNTCAANNNNNN*	2.81	M	13
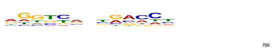	*NNNNNNAAGGNNNNNNNCCTTNNNNNN*	4.46	IR:1	14
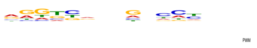	*NNNNNNAAGGNNNACGANNNNNN*	3.23	M	15
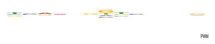	*NNNNNNGGGGNNNGAGGNNNNNN*	2.85	M	16
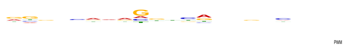	*NNNNNNAAAGNNCAGTNNNNNN*	2.48	M	17
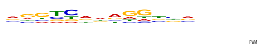	*NNNNNNTAGGNNNNAGGTNNNNNN*	3.65	DR:1	18

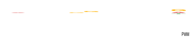	$NNNNNNGGGGNNNNG\overrightarrow{AGGNNN}NNNN$	2.33	M	19
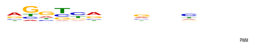	$NNNNNNA\overrightarrow{AGGNNA}TCGNNNNNN$	2.68	M	20

304 COUP-TF1-Yin2017-Rnd3 (ERR1005218)

PWM	Seed Sequence	Seed Seq Enrichment	Repeat	Rank
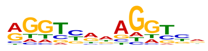	NNNNNNAGGTNNNAGGTNNNNNN	8.87	DR:1	1
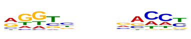	NNNNNNAGGTNNNNNACCTNNNNNN	8.13	IR:1	2
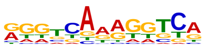	NNNNNNTCAANNNTCATNNNNNN	8.63	DR:1	3
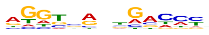	NNNNNNAAGGNNNCTGANNNNNN	5.26	IR:1	4
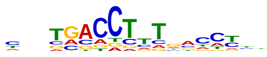	NNNNNNCGTTNNNNTTTGNNNNNN	5.23	M	5
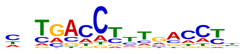	NNNNNNGCGTNNNNTTTGNNNNNN	4.02	M	6
	NNNNNNTCAANNNGTCANNNNNN	3.25	DR:2	7
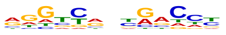	NNNNNNGTCGNCGACNNNNNN	4.66	IR:1	8
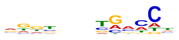	NNNNNNAGGTNNNNNNCTTTNNNNNN	5.26	M	9

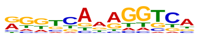	NNNNNNTCAANNNNNCAGTNNNNNN	4.60	M	10
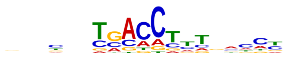	NNNNNNCAGTNNNNTTTGNNNNNN	4.15	M	11
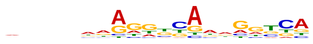	NNNNNNGAAANNNCAAANNNNNN	4.75	M	12
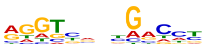	NNNNNNGGGTNNNCGACNNNNNN	5.22	IR:1	13
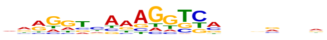	NNNNNNCAAANNNNAAATNNNNNN	3.30	M	14
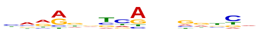	NNNNNNCAGGNNTCAANNNNNN	3.70	M	15
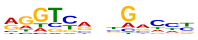	NNNNNNGAGGNNNNCGACNNNNNN	4.10	IR:1	16
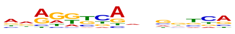	NNNNNNCAGANNNCAAANNNNNN	4.15	M	17
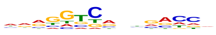	NNNNNNGTCANNNNNCTCGNNNNNN	3.57	M	18

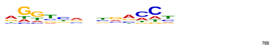	NNNNNNAAGGNNNNNNNCCTTNNNNNN	5.21	IR:1	19
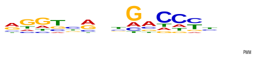	NNNNNNGAGGNNNCTGANNNNNN	4.06	IR:1	20

305 COUP-TF1-Yin2017-Rnd3 (ERR1005219)

PWM	Seed Sequence	Seed Seq Enrichment	Repeat	Rank
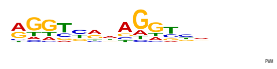	*NNNNNNAGGTNNNAGGTNNNNN*	8.91	DR:1	1
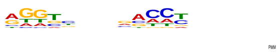	*NNNNNNAGGTNNNNNACCTNNNNNN*	8.59	IR:1	2
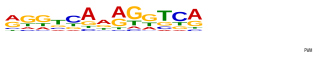	*NNNNNNTCAANNNTCAGNNNNNN*	5.81	DR:1	3
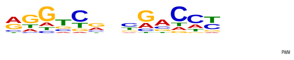	*NNNNNNGTCGNCGACNNNNNN*	6.52	IR:1	4
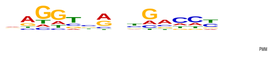	*NNNNNNAAGGNNNCTGANNNNNN*	5.36	IR:1	5
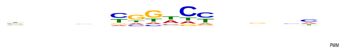	*NNNNNNCCGGTCCGNNNNNN*	3.14	M	6
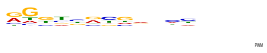	*NNNNNNAGGTNNCGACNNNNNN*	3.16	IR:0	7
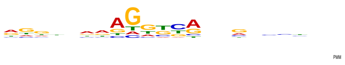	*NNNNNNAAAGNNNATTGNNNNNN*	4.33	M	8
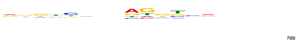	*NNNNNNGTCANNNGGTCNNNNNN*	3.24	DR:2	9

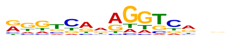 PWM	NNNNNNTCAANNNNCACGNNNNNN	4.17	M	10
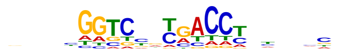 PWM	NNNNNNCGGTCCNNNACCTNNNNNN	12.88	IR:1	11
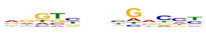 PWM	NNNNNNCAGGNNNACGANNNNNN	3.10	M	12
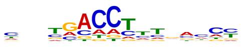 PWM	NNNNNNCGTTNNNNTTTGNNNNNN	3.50	M	13
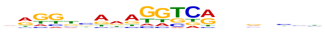 PWM	NNNNNNCAAANNNCGTTNNNNNN	3.14	M	14
PWM	NNNNNNAAGGNNNNNNNCCTTNNNNNN	5.40	IR:1	15
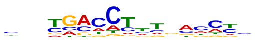 PWM	NNNNNNCAATNNNNTTTGNNNNNN	3.64	M	16
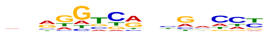 PWM	NNNNNNGTCACCGANNNNNN	3.80	M	17
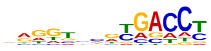 PWM	NNNNNNGGTGCNNNACCTNNNNNN	6.12	IR:1	18

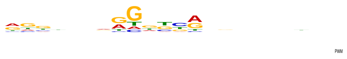	$NNNNNNAA\overrightarrow{AGNNNA}CGANNNNNN$	3.00	M	19
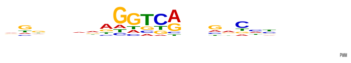	$NNNNNNAAAGNNNN\overrightarrow{NNGACA}NNNNNN$	3.72	M	20

306 COUP-TF1-Yin2017-Rnd4 (ERR1005220)

PWM	Seed Sequence	Seed Seq Enrichment	Repeat	Rank
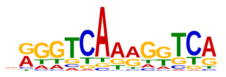	NNNNNNTCAANNNTCATNNNNNN	13.14	DR:1	1
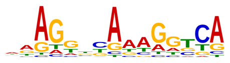	NNNNNNGAAANNNNAAAGNNNNNN	9.88	M	2
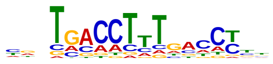	NNNNNNCGTTNNNNTTTGNNNNNN	9.42	M	3
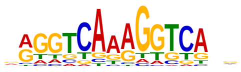	NNNNNNTCAANNNNCACGNNNNNN	11.48	M	4
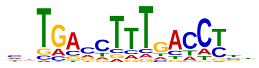	NNNNNNGATTNNNNTTTGNNNNNN	6.97	M	5
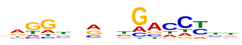	NNNNNNGAGGNNNCTGANNNNNNN	3.86	IR:1	6
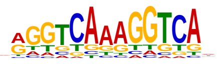	NNNNNNTCAANNNNCAGCNNNNNN	9.46	M	7
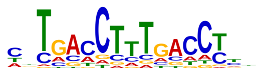	NNNNNNGCGTNNNNTTTGNNNNNN	8.34	M	8
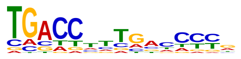	NNNNNNTTTGNNNCCGTNNNNNN	4.13	M	9

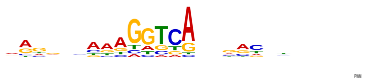	$NNNNNNAAAGNNNNN\overrightarrow{NNGACA}NNNNNN$	4.87	M	10
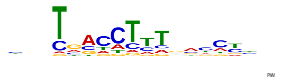	$NNNNNNCAGTNNNNNTTTGNNNNNN$	6.22	M	11
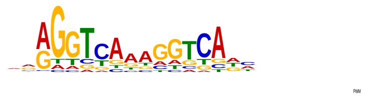	$NNNNNNCAAANNNN\overrightarrow{AGCTNN}NNNN$	4.33	M	12
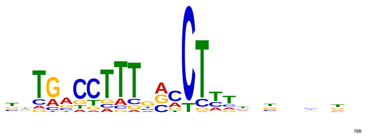	$NNNNNNCTTTNNNNTTTTGTNNNNNN$	19.29	M	13
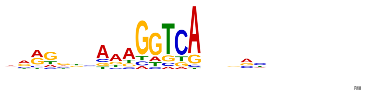	$NNNNNNCAAANNNNNN\overrightarrow{NNGACA}NNNNNN$	4.20	M	14
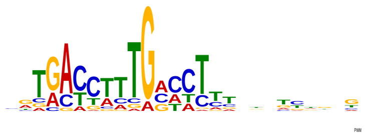	$NNNNNNCTTTNNNNTTTCTCNNNNNN$	15.55	M	15
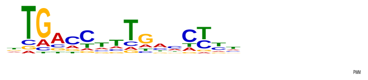	$NNNNNNTTTGNNNNTTGTNNNNNN$	4.84	M	16
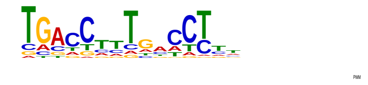	$NNNNNNTTTGNNNNTTCCNNNNNN$	4.79	M	17
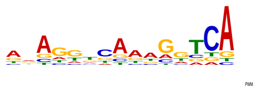	$NNNNNNAAT\overrightarrow{ANNNCA}AANNNNNN$	4.25	M	18

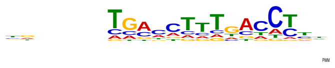	*NNNNNNTTGTNNNNTTTGNNNNNN*	4.57	M	19
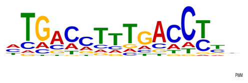	*NNNNNNGGATNNNNTTTGNNNNNN*	4.08	M	20

307 COUP-TF1-Yin2017-Rnd4 (ERR1005221)

PWM	Seed Sequence	Seed Seq Enrichment	Repeat	Rank
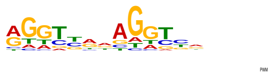	NNNNNNAGGTNNNAGGTNNNNNN	8.91	DR:1	1
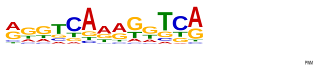	NNNNNNTCAANNNTCAGNNNNNN	7.81	DR:1	2
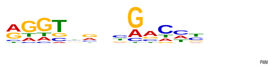	NNNNNNAGGTNNNCGACNNNNNN	7.50	IR:1	3
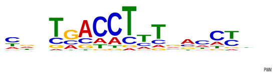	NNNNNNCGTTNNNNTTTGNNNNNN	6.86	M	4
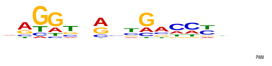	NNNNNNAAGGNNNCTGANNNNNN	5.93	IR:1	5
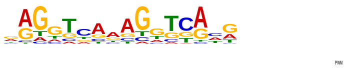	NNNNNNCAAANNNNACGTNNNNNN	4.63	M	6
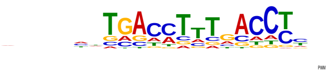	NNNNNNGATTNNNNTTTGNNNNNN	4.63	M	7
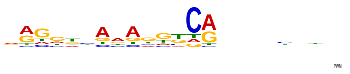	NNNNNNCAAANNNCGTTNNNNNN	4.31	M	8
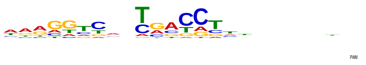	NNNNNNCAGTNNNNTTTTNNNNNN	3.13	M	9

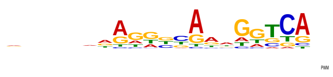	NNNNNNGAA$\overrightarrow{\text{ANNNNC}}$AAANNNNNN	5.60	M	10
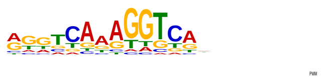	NNN$\overrightarrow{\text{NNNTC}}$AANNNNCAGCNNNNNN	5.83	M	11
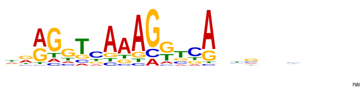	NNNNNNCAAANNNNNATTGTNNNNNN	7.89	M	12
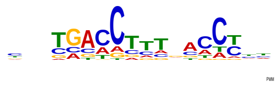	NNNNNNTACTNNNNTTTGNNNNNN	3.84	M	13
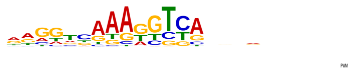	NNNNNNCAAANNNNN$\overrightarrow{\text{AAGTA}}$NNNNNN	6.19	M	14
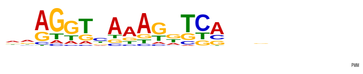	NNNNNNCAAANNNNNAAATNNNNNN	4.25	M	15
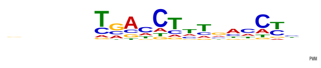	NNNNNNCTGTNNNNTTTGNNNNNN	3.79	M	16
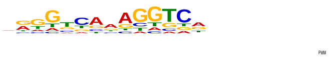	NNN$\overrightarrow{\text{NNNTC}}$AANNNNCGCTNNNNNN	3.27	M	17
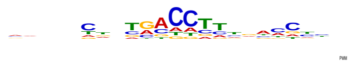	NNNNNNCGTTNNNNTCTGNNNNNN	3.23	M	18

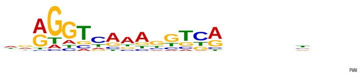	*NNNNNNCAAANNNNATATNNNNNN*	3.23	M	19

308 COUP-TF2:RXRA-Bhimsaria2022-Rnd3 (S13-134)

PWM	Seed Sequence	Seed Seq Enrichment	Repeat	Rank
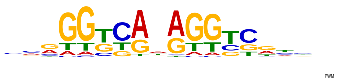	NNNNGGTCANAGGTCNNNN	63.99	DR:1	1
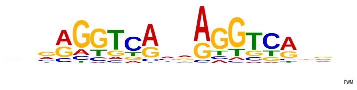	NNNNGGTCNNNNGGTCNNNN	15.28	DR:2	2
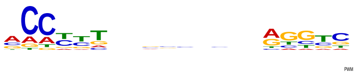	ACCTNNNNNNNNNNNNGGTC	11.90	ER:11	3
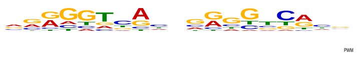	NNNGGTCNNNNNGGTCNNN	7.91	DR:3	4
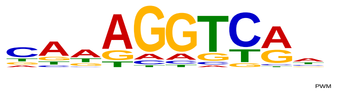	CAAAGGTCAA	54.01	M	5
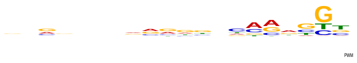	GGGGNNNNNNNNNNNNAGGT	6.92	M	6
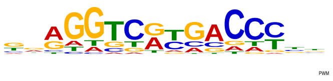	NNNAGGTCNTGACCNNN	25.79	IR:0	7
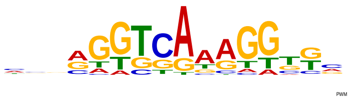	NNGAGGTCAAAGGTNN	123.31	DR:1	8
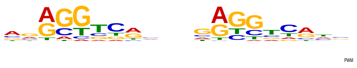	NNNGGTCNNNNNNGGTCNNN	7.82	DR:4	9

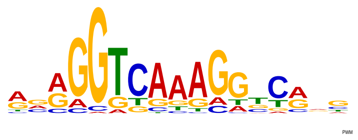	*NNNGGTCAAAGGGCNNN*	72.14	DR:1	10
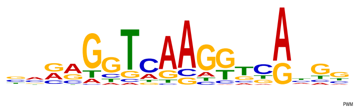	*NNNNGGTCAAGGTCNNNN*	25.81	DR:0	11
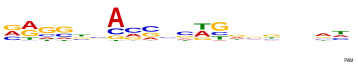	*NNGGTCNNNNNNNNGGTCNN*	3.99	DR:6	12
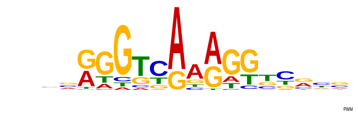	*NNNGGGGTCNNNGGTCNNN*	28.54	DR:1	13
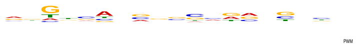	*NGGTCNNNNNNNNNGGTCN*	3.67	DR:7	14
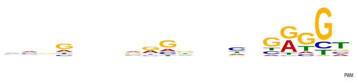	*AAGGNNNNNNNNNNNGGGGT*	10.96	DR:9	15
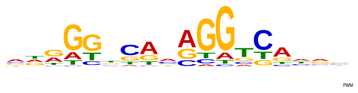	*NNGGGTNNNNGGTCAANN*	27.01	DR:1	16
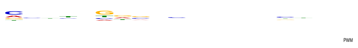	*CCTTNNNNNNNNNNNCTTG*	5.55	M	17
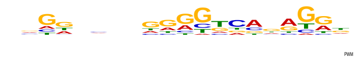	*NNNNNNACGGGGTCNNNNNN*	8.56	M	18

309 COUP-TF2-17-Bhimsaria2022-Rnd2 (S07-025)

PWM	Seed Sequence	Seed Seq Enrichment	Repeat	Rank
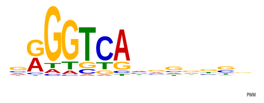	NNNNNNGGGGTCAANNNNNN	22.23	M	1
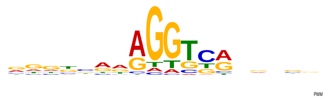	NNNNNNCAAAGGTCNNNNNN	17.27	M	2
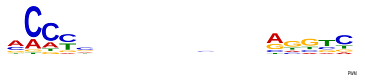	ACCCNNNNNNNNNNNNGGTC	16.75	ER:11	3
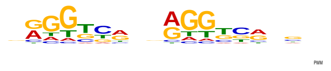	NNNNGGTCNNNNNGGTCNNNN	14.22	DR:2	4
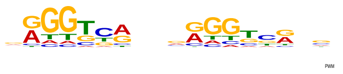	NNNGGTCNNNNNGGTCNNN	11.51	DR:3	5
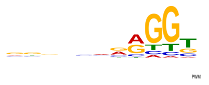	GGGGNNNNNNNNNNNNNAGGT	10.52	M	6
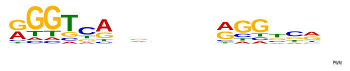	NNGGTCNNNNNNNNGGTCNN	7.89	DR:6	7
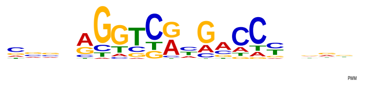	NNNNAGGTCNNGACCCNNNN	37.38	IR:0	8
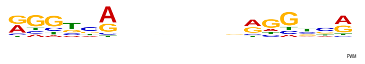	NGGTCNNNNNNNNNGGTCN	6.06	DR:7	9

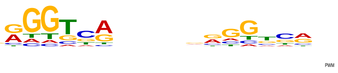	NNGGTCNNNNNNNGGTCNN	7.60	DR:5	10
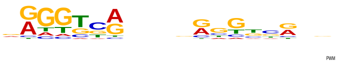	NNNGGTCNNNNNNGGTCNNN	7.82	DR:4	11
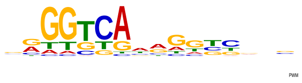	NNNNNGGTCAAAGGNNNNN	34.60	DR:1	12
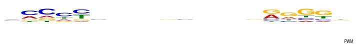	GACCNNNNNNNNNNNNGGTC	3.87	ER:10	13
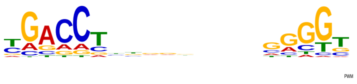	CGACCTNNNNNNNNNNGGGT	30.93	ER:10	14
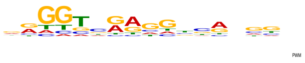	NNNNNGGTCNNGGTCNNNNN	8.16	DR:0	15
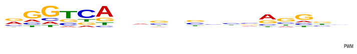	AGGTCNNNNNNNNNNGGTC	5.65	DR:8	16
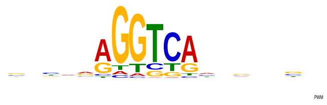	NNNNNNAAGGTCAANNNNNN	20.62	M	17
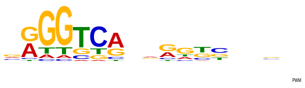	NNNNNGGTCNNAGGGNNNNN	10.70	DR:1	18

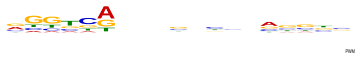	$\overrightarrow{GGGTCN}NNNNNNNN\overrightarrow{NGGTC}$	5.91	DR:8	19
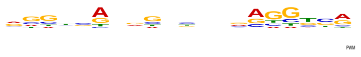	$\overrightarrow{NGGTCN}NNNNNNNN\overrightarrow{NGGTCN}$	4.46	DR:8	20

310 COUP-TF2-17-Bhimsaria2022-Rnd3 (S07-145)

PWM	Seed Sequence	Seed Seq Enrichment	Repeat	Rank
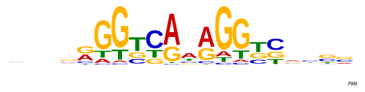	NNNNNGGTCANAGGTNNNNN	242.74	DR:1	1
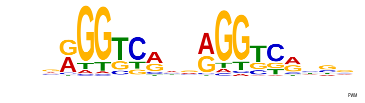	NNNNGGTCNNNNGGTCNNNN	115.45	DR:2	2
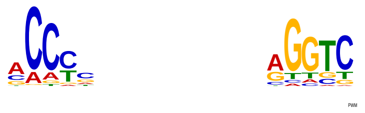	ACCCCNNNNNNNNNNAGGTC	514.85	ER:11	3
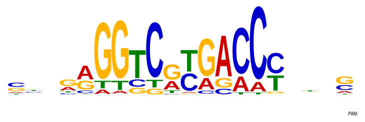	NNNNAGGTCGTGACCCNNNN	1757.51	IR:0	4
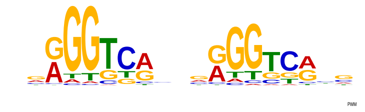	NNNGGTCNNNNNGGTCNNN	79.89	DR:3	5
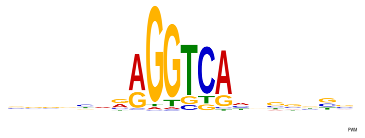	NNNNNNAAGGTCAANNNNNN	115.54	M	6
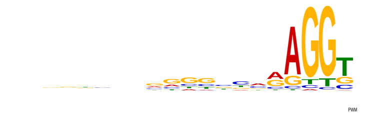	GGGGNNNNNNNNNNNAAGGT	108.75	M	7
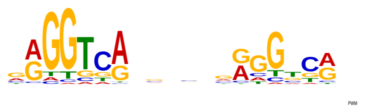	NNGGTCNNNNNNNNGGTCNN	40.76	DR:6	8
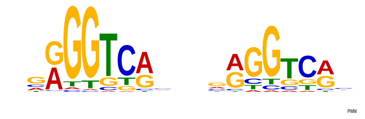	NNGGGTCNNNNNNGGTCNN	95.63	DR:4	9

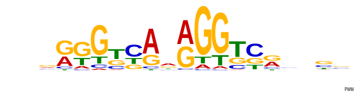	$NNN\overrightarrow{NNGTCA}G\overrightarrow{NGGTCN}NNNN$	121.44	DR:1	10
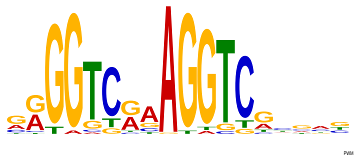	$N\overrightarrow{NGGTCG}A\overrightarrow{AGGTCG}CGNN$	3093.60	DR:1	11
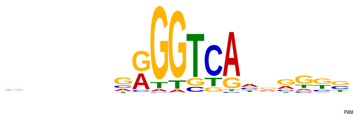	$NNNNNCG\overrightarrow{GGGTCA}ANNNNNN$	150.67	M	12
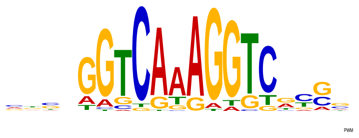	$NNN\overrightarrow{CGGTCA}A\overrightarrow{AGGTCG}NNN$	1359.25	DR:1	13
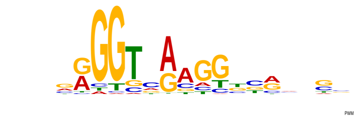	$NNNN\overrightarrow{NGGTCA}\overrightarrow{AGGTCN}NNNN$	171.26	DR:0	14
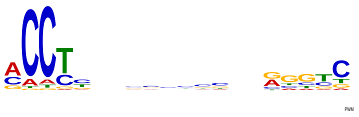	$\overleftarrow{ACCT}TNNNNNNNNNN\overrightarrow{NGGTC}$	130.05	ER:11	15
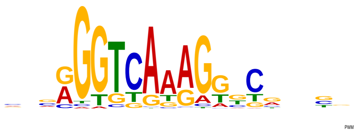	$NNN\overrightarrow{NGGTCA}A\overrightarrow{AGGGCG}NNNN$	1209.72	DR:1	16
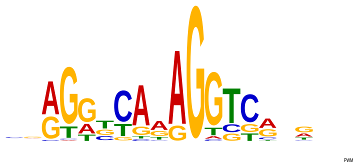	$NN\overrightarrow{NGGGCA}A\overrightarrow{AGGTCG}CNNN$	1416.95	DR:1	17
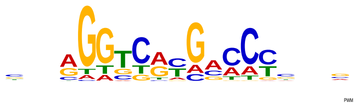	$NNN\overrightarrow{AGGTCA}\overleftarrow{TGNCCC}CNNN$	869.49	IR:0	18

311 COUP-TF2-Bhimsaria2022-Rnd2 (S07-117)

PWM	Seed Sequence	Seed Seq Enrichment	Repeat	Rank
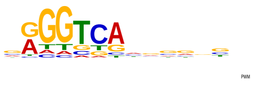	NNNNNNGGGGTCAANNNNNN	13.36	M	1
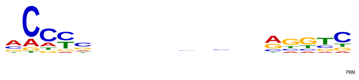	ACCCNNNNNNNNNNNNNGGTC	11.15	ER:11	2
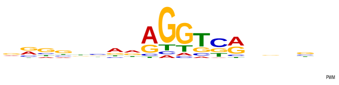	NNNNNNCAAAGGTCNNNNNN	9.41	M	3
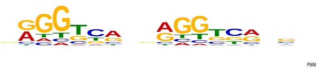	NNNNGGTCNNNNGGTCNNNN	9.47	DR:2	4
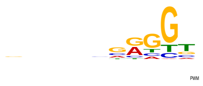	GGGGNNNNNNNNNNNNNGGGT	7.22	M	5
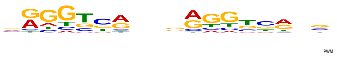	NNNGGTCNNNNNGGTCNNN	6.50	DR:3	6
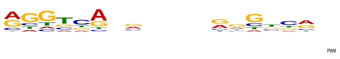	NNGGTCNNNNNNNNGGTCNN	4.53	DR:6	7
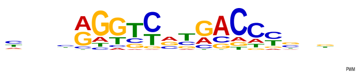	NNNNAGGTCNNGACCCNNNN	21.05	IR:0	8
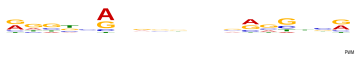	NGGTCNNNNNNNNNGGTCN	3.64	DR:7	9

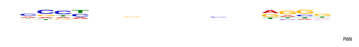	GACCNNNNNNNNNNNNGGTC	3.12	ER:10	10
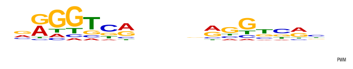	NNNGGTCNNNNNNGGTCNNN	6.06	DR:4	11
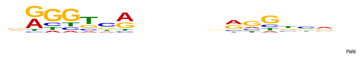	NNGGTCNNNNNNGGTCNN	4.55	DR:5	12
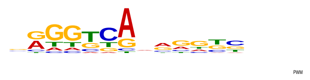	NNNNNGGTCNAAGGNNNNN	7.97	DR:1	13
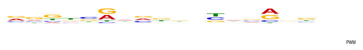	AGGTCNNNNNNNNNNGGTC	3.23	DR:8	14
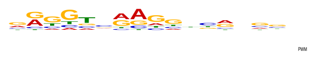	NNNNNGGTCNNGGTCNNNNN	5.03	DR:0	15
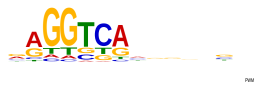	NNNNNNAAGGTCAANNNNNN	11.83	M	16
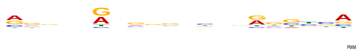	NGGTCNNNNNNNNNNGGTCN	2.65	DR:8	17
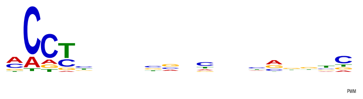	ACCTTNNNNNNNNNNNGGTC	11.12	ER:11	18

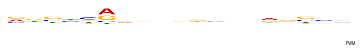	$\overrightarrow{GGGTC}NNNNNNNNNN\overrightarrow{GGTC}$	3.35	DR:8	19
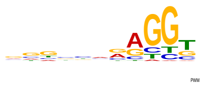	$GGGGNNNNNNNNNNNA\overrightarrow{AGGT}$	10.65	M	20

312 COUP-TF2-Bhimsaria2022-Rnd3 (S07-112)

PWM	Seed Sequence	Seed Seq Enrichment	Repeat	Rank
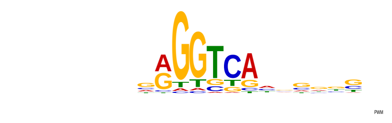	NNNNNNGGGGTCAANNNNNN	48.26	M	1
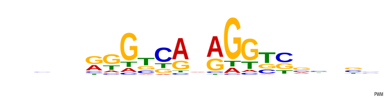	NNNNNGTCANAGGTCNNNNN	77.78	DR:1	2
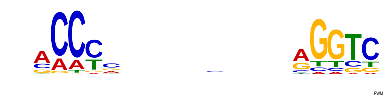	ACCCCNNNNNNNNNNAGGTC	167.99	ER:11	3
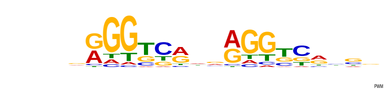	NNNNGGTCNNNNGGTCNNNN	33.98	DR:2	4
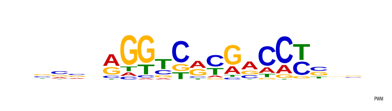	NNNNAGGTCNNGACCCNNNN	88.63	IR:0	5
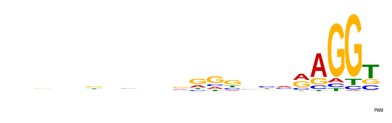	GGGGNNNNNNNNNNNAAGGT	39.83	M	6
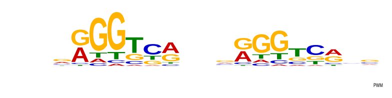	NNNGGTCNNNNNGGTCNNN	26.14	DR:3	7
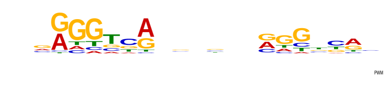	NNGGTCNNNNNNNNGGTCNN	13.11	DR:6	8
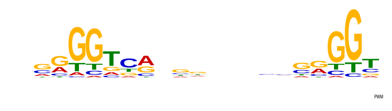	GGGGTCNNNNNNNNNGGGGT	130.74	DR:9	9

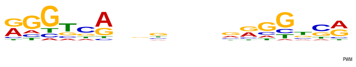	$\overrightarrow{NGGTCN}NNNNNNN\overrightarrow{NGGTCN}$	14.04	DR:7	10
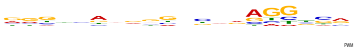	$\overrightarrow{NGGTCN}NNNNNNNN\overrightarrow{NGGTCN}$	8.70	DR:8	11
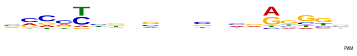	$\overleftarrow{GACCN}NNNNNNNNNN\overrightarrow{NGGTC}$	9.18	ER:10	12
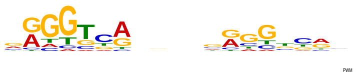	$N\overrightarrow{NGGTCN}NNNNN\overrightarrow{NGGTCN}N$	14.87	DR:5	13
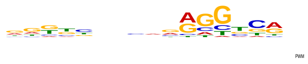	$NNGGGGNNNNNNN\overrightarrow{NGGTCN}N$	12.29	M	14
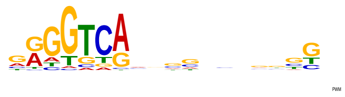	$NG\overrightarrow{GGGTCN}NNNNNNN\overrightarrow{GGGTN}$	39.42	DR:7	15
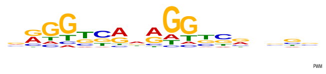	$NNN\overrightarrow{NGGTCN}N\overrightarrow{NNGTCA}NNNN$	20.70	DR:1	16
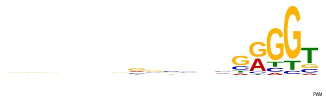	$\overrightarrow{AGGGNN}NNNNNNNNNG\overrightarrow{GGGT}$	33.26	DR:10	17
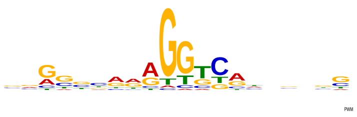	$NNNNNCAA\overrightarrow{AGGTCA}ANNNNN$	83.20	M	18

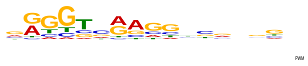	$NNNN\overrightarrow{NGGTC}N\overrightarrow{NGGTC}NNNNN$	16.21	DR:0	19
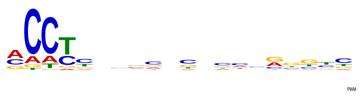	$\overleftarrow{ACCT}TNNNNNNNNNN\overrightarrow{NGGTC}$	34.73	ER:11	20

313 EAR2:RXRA-Bhimsaria2022-Rnd3 (S13-135)

PWM	Seed Sequence	Seed Seq Enrichment	Repeat	Rank
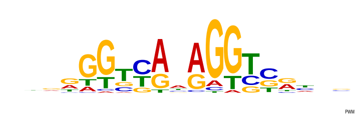	NNNNGGTCANAGGTCNNNN	82.72	DR:1	1
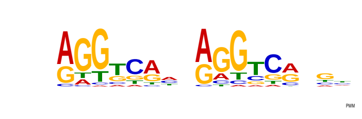	NNNNGGTCANNNGGTCNNNN	19.14	DR:2	2
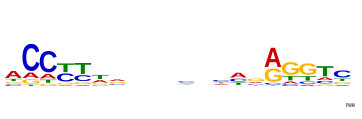	ACCTNNNNNNNNNNNNGGTC	12.58	ER:11	3
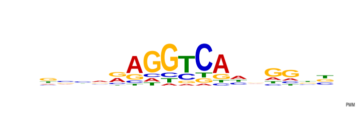	NNNNNNAAGGTCAANNNNNN	16.04	M	4
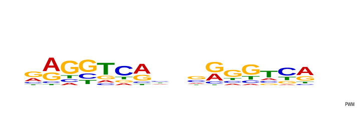	NNNGGTCNNNNNNGGTCNNN	7.07	DR:3	5
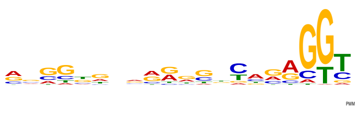	AGGGNNNNNNNNNNNGAGGT	16.76	DR:10	6
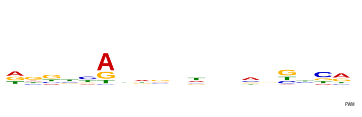	NGGTCNNNNNNNNNGGTCN	4.63	DR:7	7
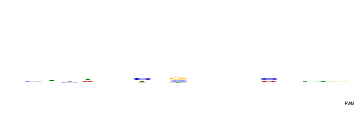	CCTTNNNNNNNNNNNTTTG	6.28	M	8
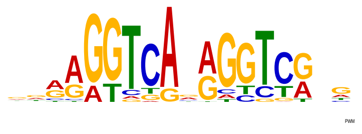	NNNAGGTCNGGGGTCNNN	45.09	DR:1	9

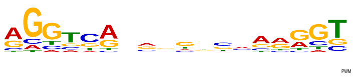	*AGGTCNNNNNNNNNAGGT*	13.40	DR:8	10
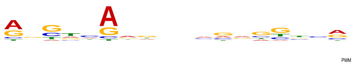	*NGGTCNNNNNNNNGGTCN*	4.56	DR:6	11
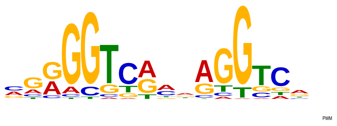	*NNGGGGTCNNNNGGTCNN*	27.03	DR:2	12
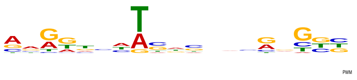	*NAGGTCNNNNNNNNGGGTN*	6.73	DR:7	13
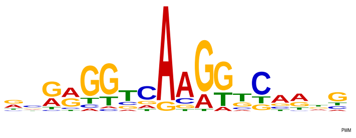	*NNNNGGTCAAGGTCNNNN*	22.68	DR:0	14
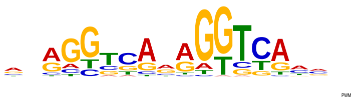	*NNAGGTNNNNGGTCAANN*	45.98	DR:1	15
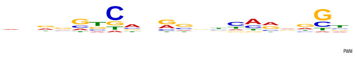	*AAAGNNNNNNNNNNNNNAGGT*	6.28	M	16
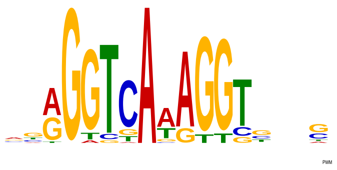	*NNNAGGTCAAAGGTGNNN*	195.71	DR:1	17
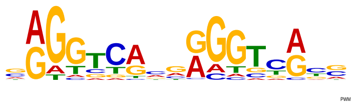	*NNNGTCANGGGGTCNNN*	25.05	DR:2	18

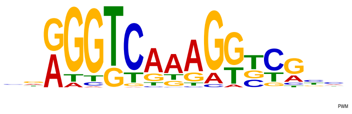	NNN$\overrightarrow{GGTCA}$A$\overrightarrow{AGGGC}$NNN	85.41	DR:1	19
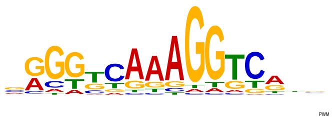	NNN$\overrightarrow{GGGCN}$A$\overrightarrow{AGGTC}$NNN	41.29	DR:0	20

314 EAR2-Bhimsaria2022-Rnd2 (S07-118)

PWM	Seed Sequence	Seed Seq Enrichment	Repeat	Rank
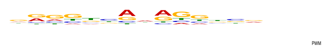	NNNNGGTCNNNGGTCNNNN	3.47	DR:1	1
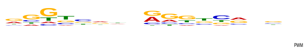	NNNNGGTCNNNNGGTCNNNN	3.44	DR:2	2
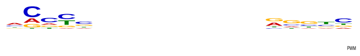	ACCCNNNNNNNNNNNNGGTC	3.60	ER:11	3
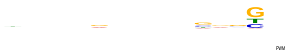	GGGGNNNNNNNNNNNNGGGT	2.74	M	4
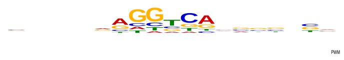	NNNNNNAAGGTCAANNNNNN	4.12	M	5
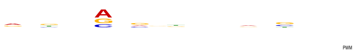	NGGTCNNNNNNNNNGGTCN	1.87	DR:7	6
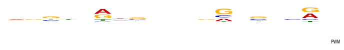	NNGGTCNNNNNNNNGGTCNN	2.06	DR:6	7
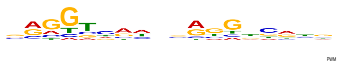	NNNGGTCANNNNGGTCNNN	4.42	DR:3	8
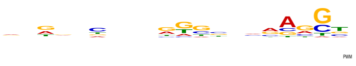	AAGGNNNNNNNNNNNAAGGT	3.53	DR:9	9

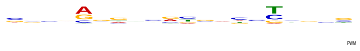	NNNNNGGTCNNGACCNNNNN	2.45	IR:0	10
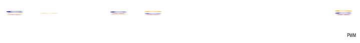	CAGGNNNNNNNNNNNNNACCG	1.95	IR:7	11
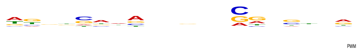	NGGTCANNNNNNNNNGGTCN	2.21	DR:8	12
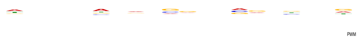	NGGTCNNNNNNNNNNGGTCN	1.40	DR:8	13
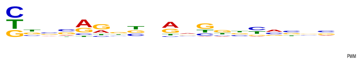	NNNNNGGTCNAGGTCNNNNN	3.01	DR:0	14
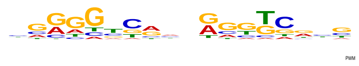	NNNGGTCACNNGGTCNNN	5.96	DR:2	15
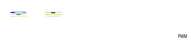	CCGGNNNNNNNNNNNNNGCTG	1.76	M	16
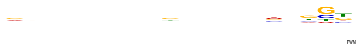	GGTCNNNNNNNNNNNNNAGGT	2.09	DR:11	17
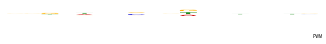	CAGGNNNNNNNNNNNNNTCTG	1.91	M	18

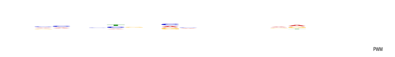	$\overleftarrow{ACCT}NNNNNNNNNN\overrightarrow{NGGTC}$	1.65	ER:10	19
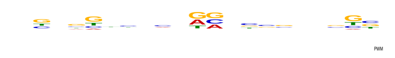	$N\overrightarrow{GGGTCN}NNNNNNNN\overrightarrow{GGGTN}$	2.72	DR:8	20

315 EAR2-Bhimsaria2022-Rnd3 (S07-113)

PWM	Seed Sequence	Seed Seq Enrichment	Repeat	Rank
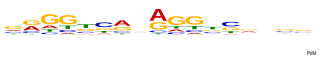	NNNNGGTCNNNGGTCNNNN	11.24	DR:1	1
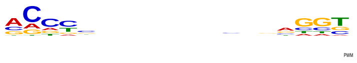	ACCCNNNNNNNNNNNAGGT	8.79	ER:11	2
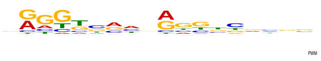	NNNNGGTCNNNNGGTCNNNN	8.92	DR:2	3
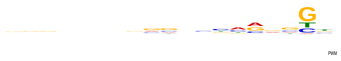	GGGGNNNNNNNNNNNNAGGT	8.02	M	4
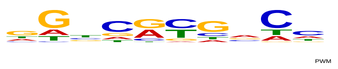	GGTCATGACC	17.36	IR:0	5
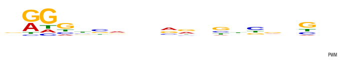	NNNNGTCANNNNGGTCNNNN	5.17	DR:3	6
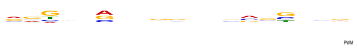	NGGTCNNNNNNNNGGTCN	5.25	DR:7	7
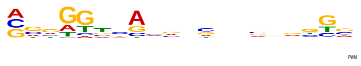	NGGGGTCNNNNNNNGGGTN	14.13	DR:7	8
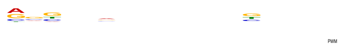	NNGGTCNNNNNNGGTCNN	4.27	DR:5	9

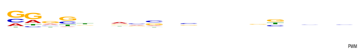	NNGGTCNNNNNNNNNGGTCNN	4.80	DR:6	10
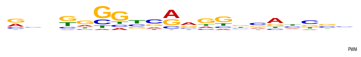	NNNNNGGTCNAGGTCNNNNN	10.17	DR:0	11
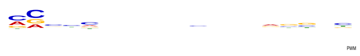	ACCCNNNNNNNNNNNGGTC	5.86	ER:10	12
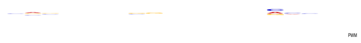	CAGGNNNNNNNNNNNNCCCC	6.17	M	13
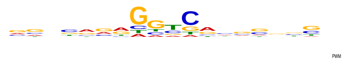	NNNNNNAAGGTCAANNNNNN	7.81	M	14
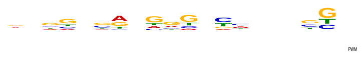	NAGGTCNNNNNNNNNGGGTN	6.65	DR:8	15
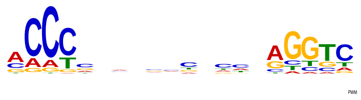	ACCCCNNNNNNNNNNAGGTC	42.03	ER:11	16
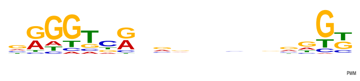	GGGGTCNNNNNNNNNGGGT	22.26	DR:8	17
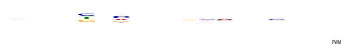	CAGGNNNNNNNNNNNNCATG	4.81	M	18

316 EAR2-Jolma2013-Rnd2 (ERR193875)

PWM	Seed Sequence	Seed Seq Enrichment	Repeat	Rank
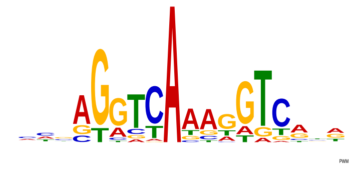	NNNNNGGTCNAAGGNNNNN	25.41	DR:1	1
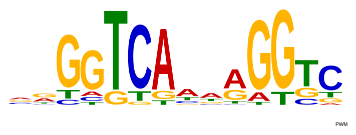	NNGGTCANAAGGNN	24.41	DR:2	2
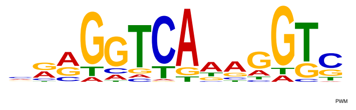	NNGGGTCNAAGGNN	28.15	DR:1	3
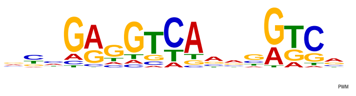	NNNNGGGTNAAAGNNNN	9.51	M	4
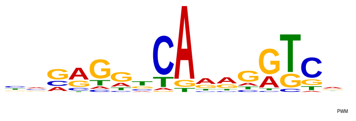	NNNGGGTNNAAGGNNN	8.37	DR:1	5
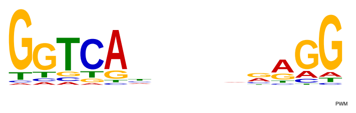	GGTCNNNNNNGAGG	14.57	M	6
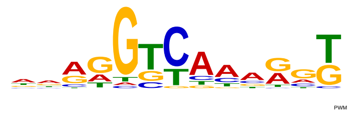	NAAGGNNNNAAGGN	12.06	DR:1	7
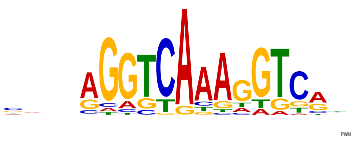	NNNNAGGTNNAAGGNNNN	20.14	DR:1	8
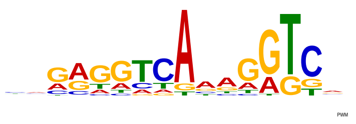	NNNGGGTNAAAGGNNN	22.16	DR:1	9

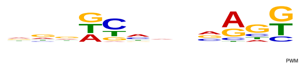	$NGGGGNNNNA\overrightarrow{AGGN}$	4.55	M	10
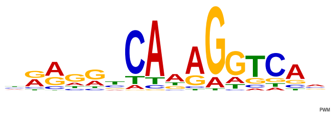	$NNN\overrightarrow{GGGTNN}N\overrightarrow{AGGTNN}N$	9.90	DR:1	11
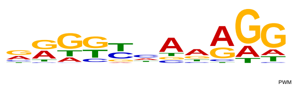	$NGGGGNNNAAAGN$	13.48	M	12
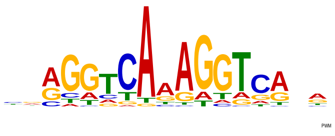	$NNN\overrightarrow{NGGTCN}N\overrightarrow{AGGTNN}NN$	28.46	DR:1	13
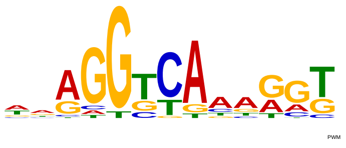	$NA\overrightarrow{AGGTNN}A\overrightarrow{AGGN}$	23.72	DR:1	14
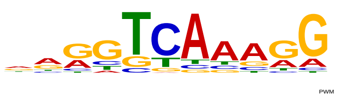	$T\overrightarrow{AGGNNN}AAGG$	12.03	M	15
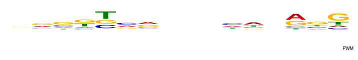	$GGGGNNNNNNNGGGG$	4.90	M	16
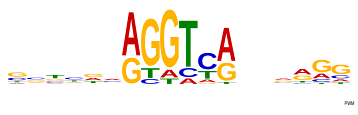	$NNNNNA\overrightarrow{AGGTCA}TNNNNN$	12.43	M	17
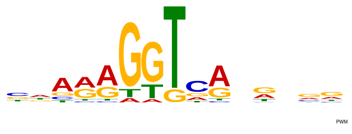	$NNNA\overrightarrow{AGGNCA}GGNNN$	9.13	M	18

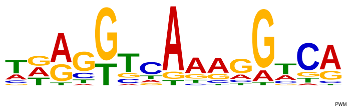	$NN\overrightarrow{NGGTCN}G\overrightarrow{AGGNNN}$	8.08	DR:1	19
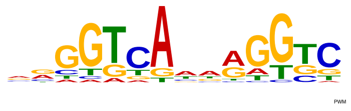	$N\overrightarrow{NGGTCN}NA\overrightarrow{AGGNN}$	10.37	DR:2	20

317 EAR2-Jolma2013-Rnd2 (ERR195350)

PWM	Seed Sequence	Seed Seq Enrichment	Repeat	Rank
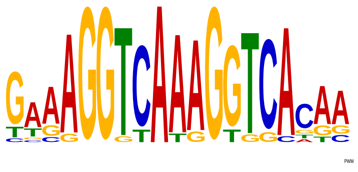	NNNAGGTCAAAGGTCANNN	13388.16	DR:1	1
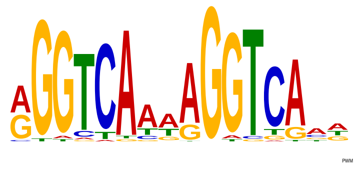	NNGTCANNAGGTCANN	146.12	DR:2	2
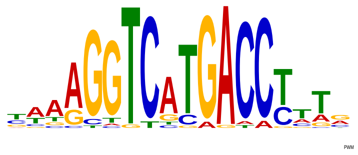	NNNNGGTCANGACCNNNN	50.72	IR:0	3
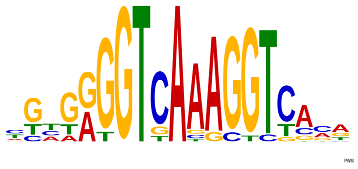	NNNNGGGTCAAAGGTNNNN	400.65	DR:1	4
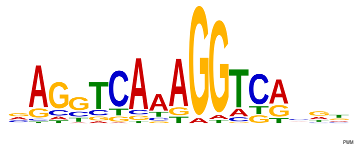	NNGGTCNNNNNTCAANN	8.13	DR:1	5
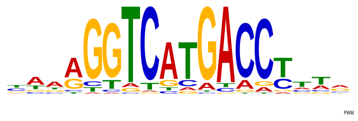	NNNNGGTCNNGACCNNNN	18.00	IR:0	6
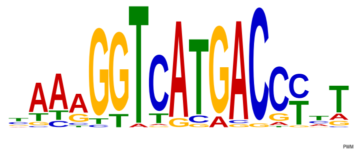	NNAGGTCNNGACCNNN	39.92	IR:0	7
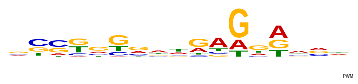	NNNNGGGANGAGGNNNN	3.00	M	8
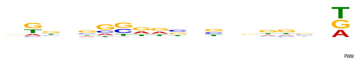	NNGGGGNNNNNNNGGGGNN	2.99	M	9

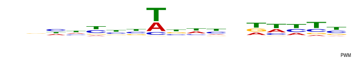	*NNNTTTTNNNTTTTNNN*	2.32	M	10
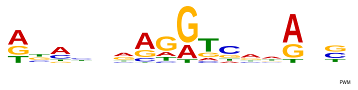	*NNNNAGGGGTCANNNN*	3.77	M	11
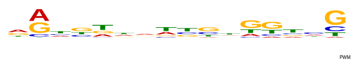	*NNNGTTANNGTGGNNN*	2.02	M	12
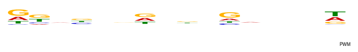	*NNNNGGGTGTGANNNN*	1.66	M	13
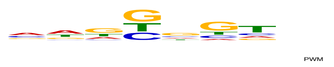	*AAGGGGTG*	2.36	M	14
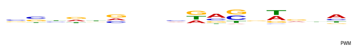	*NNTGTGNNNNNGGTGNN*	1.86	M	15
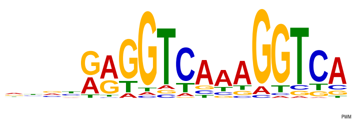	*NNNNNGGGTCAAANNNNN*	10.34	M	16
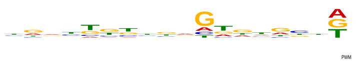	*NNNNTGTTNNGTGTNNNN*	2.14	M	17
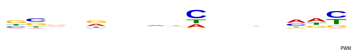	*NNNAGTGNNNGGTGNNN*	1.70	M	18

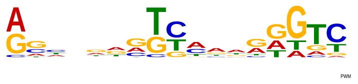	*NNNGGGGNNNAAAGNNN*	3.18	M	19
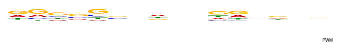	*NNGGGGNNNNNGGAGNN*	2.61	M	20

318 EAR2-Jolma2013-Rnd3 (ERR193876)

PWM	Seed Sequence	Seed Seq Enrichment	Repeat	Rank
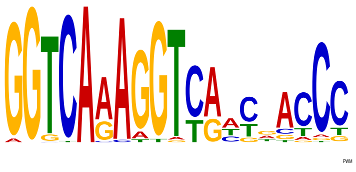	GGTCAAAGNNNNNNNACCC	1342.48	IR:8	1
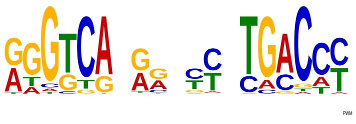	NGGTCANNNNNNNTGACCN	76.08	IR:7	2
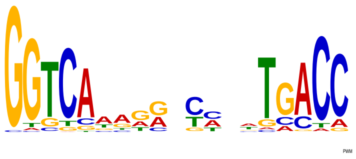	GGTCANNNNNNNNNGACC	43.26	IR:9	3
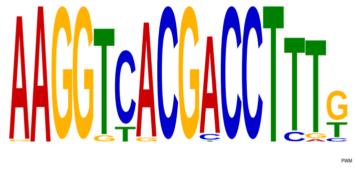	AAGGTCACGACCTTTG	8039.80	IR:0	4
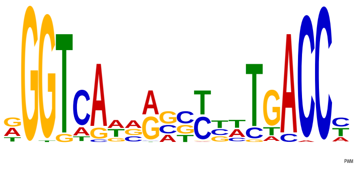	NGGTCANNNNNNNTGACCN	156.76	IR:8	5
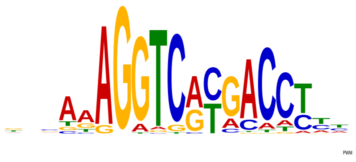	NNNAAAGGTNNNGACCNNN	98.17	IR:0	6
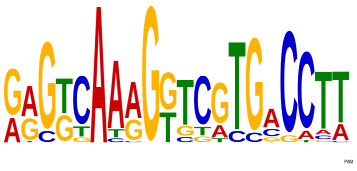	NNGTCAAAGNNNNNGACCNN	325.35	IR:7	7
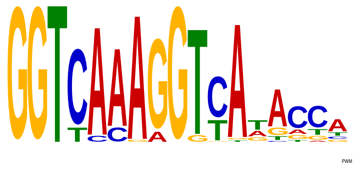	GGTCAAAGNNNNNACCA	225.99	IR:6	8
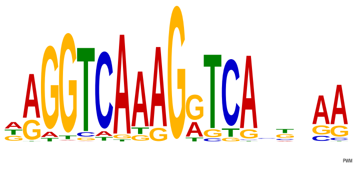	NNNGTCAAANNNCACTNNN	63.62	DR:1	9

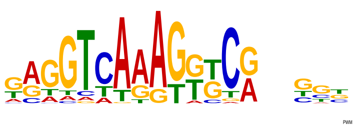	*NNNGTCAAANNNCATCNNN*	35.81	DR:1	10
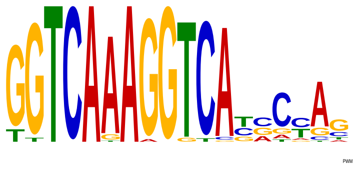	*GGTCAAAGNNNNNNCTAG*	213.74	M	11
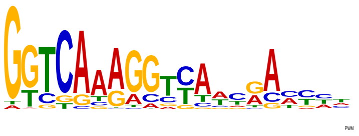	*GGTCANNNNNNNNNNNCCCC*	33.25	M	12
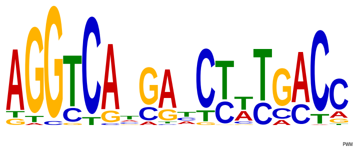	*AGGTCATNNNNNNNGACC*	112.94	IR:7	13
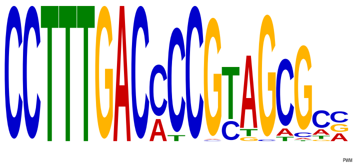	*NNTTTGNNNCCGTAGCGNN*	289.59	M	14
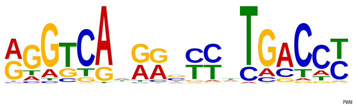	*NGGTCNNNNNNNNNGACCN*	14.74	IR:7	15
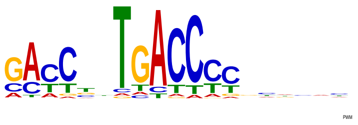	*NNNNTTTGNNNCCGCNNNN*	3.82	M	16
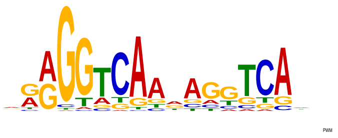	*NNNNNGTCANTAGGNNNNN*	4.75	DR:2	17
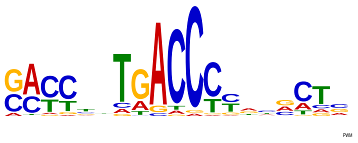	*NNNNTTTGNNNCCGTNNNN*	2.91	M	18

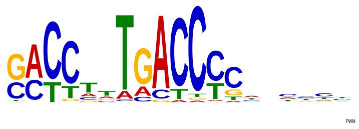	*NNNNTTTGNNNCCAGNNNN*	4.14	M	19
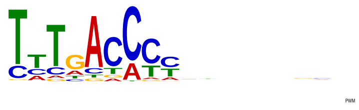	*TTTGNNNNNNNNNNCGCA*	7.15	M	20

319 EAR2-Jolma2013-Rnd3 (ERR195351)

PWM	Seed Sequence	Seed Seq Enrichment	Repeat	Rank
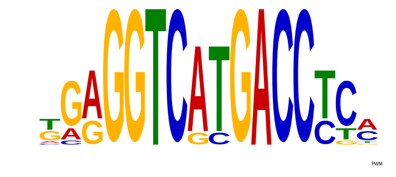	NNGGGTCATGACCCNN	1566.88	IR:0	1
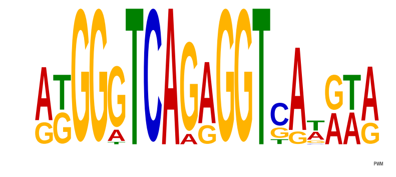	NNNGGTCNNAGGTTAANNN	480.85	DR:1	2
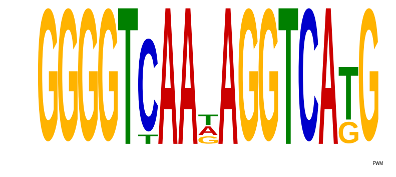	NNGGTTAATAGGTCANN	1014.25	DR:2	3
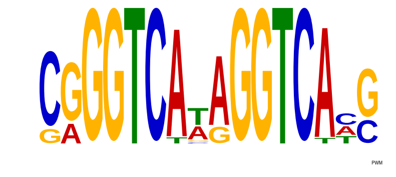	NAGGTCAGNNGTCAAN	487.48	DR:1	4
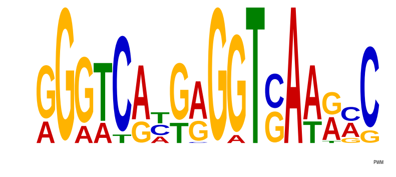	NNGTCANNAGGTCAAANN	1326.73	DR:2	5
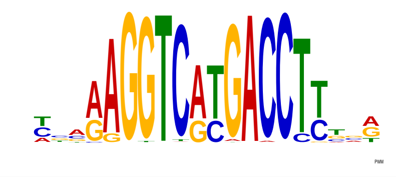	NNNNNGGTCNNGACCNNNNN	278.33	IR:0	6
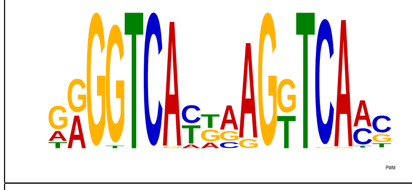	NGGGTCANNNNNGTCACN	612.92	DR:3	7
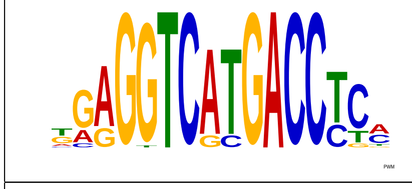	NNGGGTCANGACCCNN	630.92	IR:0	8
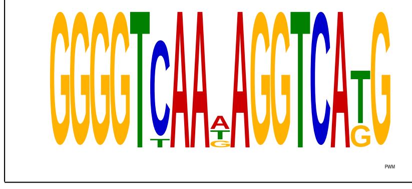	NNGGTTAATANGTCANN	536.72	DR:2	9

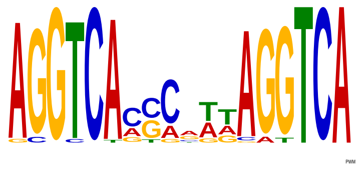	AGGTCNNNNNNNAGGTCA	336.84	DR:6	10
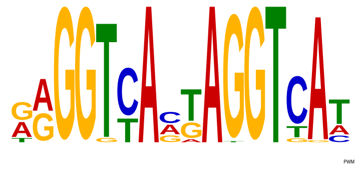	NAGGTCANNAGGTTAN	1286.58	DR:2	11
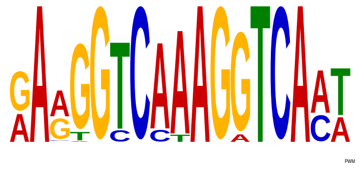	NNATGTCAANNGTCANN	160.14	DR:1	12
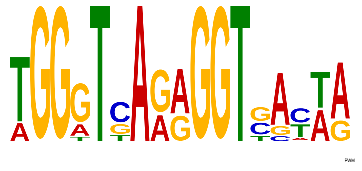	NNGGTCNAAGGTTAANN	376.38	DR:1	13
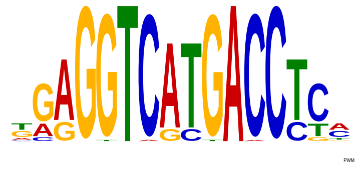	NNGGGTCNNGACCCNN	234.12	IR:0	14
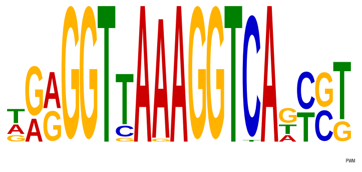	NNNGGTTAAANNTCAGNNN	803.19	DR:1	15
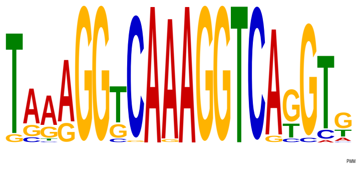	NNNNGGGCAANNGTCANNNN	232.17	DR:1	16
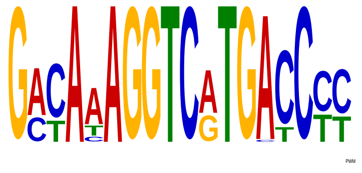	NNNATAGGTCNTGACNNN	787.26	IR:0	17
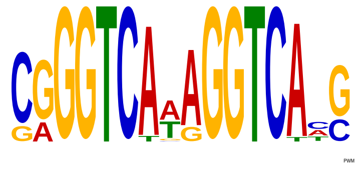	NAGGTCAGNNNTCAAN	195.68	DR:1	18

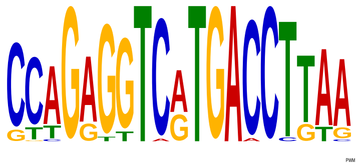	$NNNN\overrightarrow{GGGTCG}\overleftarrow{NGACC}NNNN$	275.61	IR:0	19
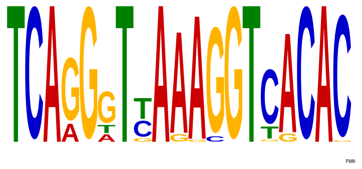	$NNN\overrightarrow{AGGTCN}N\overrightarrow{AGGTTA}NNN$	399.84	DR:1	20

320 Ear2-Jolma2013-Rnd3 (ERR195703)

PWM	Seed Sequence	Seed Seq Enrichment	Repeat	Rank
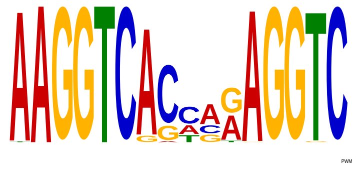	AAGGTCANNNNAGGTC	7210.32	DR:4	1
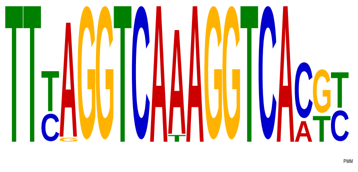	NNNAGGTCATAGGTCANNN	19924.17	DR:1	2
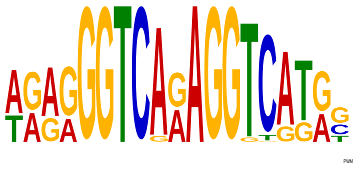	NNNGGGTCANAGGTCGNNN	4014.63	DR:1	3
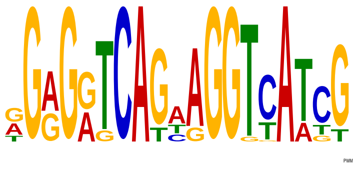	NNNGGTCANNAGGTCANNN	1142.59	DR:2	4
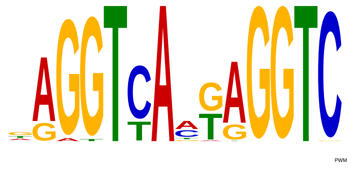	AGGTCANNNGGTC	708.18	DR:2	5
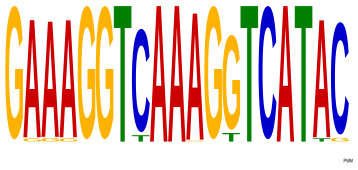	NNNNGGTCAAAGTTCNNNN	2550.62	DR:1	6
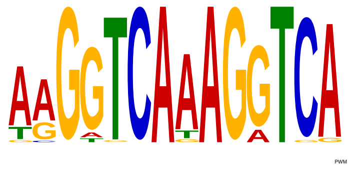	NAGGGCANAGGTCN	392.41	DR:1	7
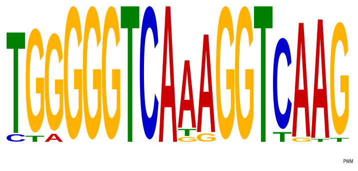	NNNGGGTCAAGGGTCNNN	1258.43	DR:1	8
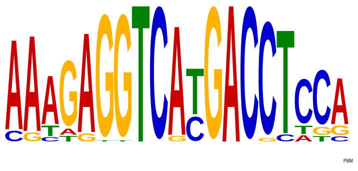	NNNNAGGTCANGACCNNNN	1143.34	IR:0	9

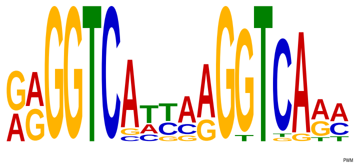	NNGGTCANNNNGGTCANN	136.52	DR:3	10
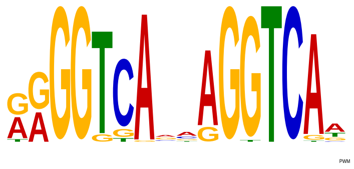	NGGGTCANNNNGTCAN	220.48	DR:2	11
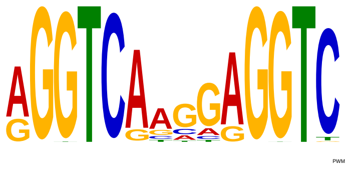	AGGTCANNNNGGTC	543.20	DR:3	12
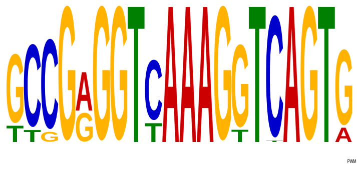	NNNNGGGTTAAAGGTCNNNN	6647.41	DR:1	13
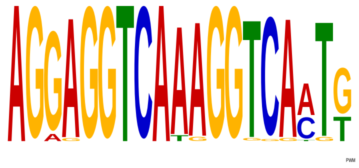	NNNNGGTCAAAGGGCNNNN	758.65	DR:1	14
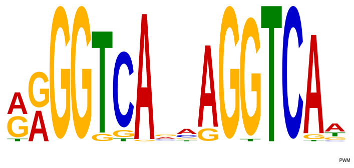	NGGGTCANNNGGTCAN	583.62	DR:2	15
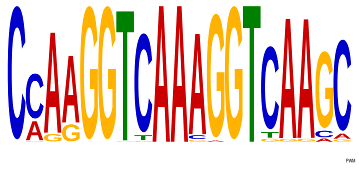	NNNNGGTCANAGGTTNNNN	745.18	DR:1	16
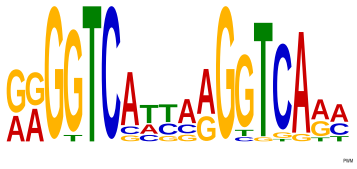	NNGGTCANNNNNGTCANN	53.32	DR:3	17
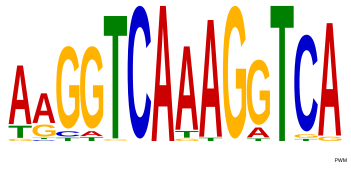	NAGGGCNNAGGTCN	96.74	DR:1	18

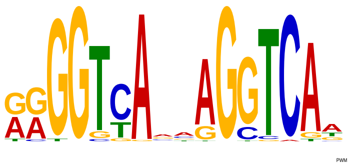	$N\overrightarrow{GGGTCN}NN\overrightarrow{NNGTCA}N$	59.38	DR:2	19
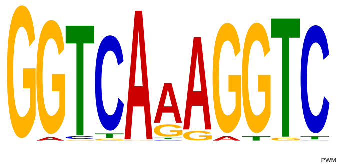	$\overrightarrow{GGTCA}G\overrightarrow{GGGTC}$	294.95	DR:1	20

321 EAR2-Jolma2013-Rnd4 (ERR193877)

PWM	Seed Sequence	Seed Seq Enrichment	Repeat	Rank
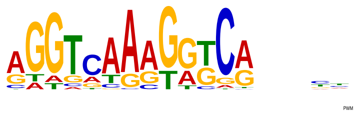	*NNNTCAANNNNCATCNNN*	7.93	M	1
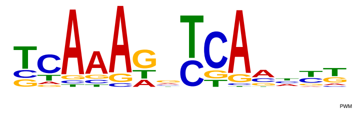	*NCAAANNNNAGATN*	3.08	M	2
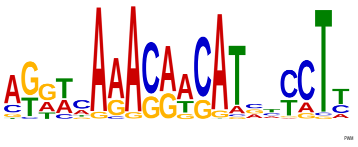	*NNNNCAAANNNNATCANNNN*	6.22	M	3
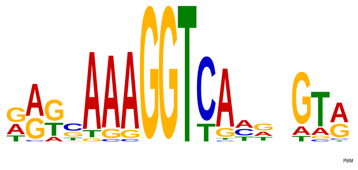	*NNNCAAANNNNACGTNNN*	5.67	M	4
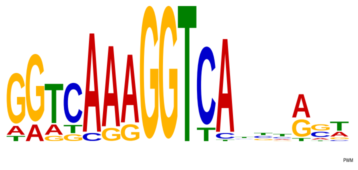	*NNNCAAANNNNATTTNNN*	5.37	M	5
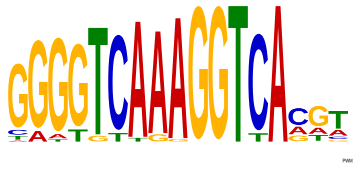	*GGGGNNNNNNNNCACGT*	14.80	M	6
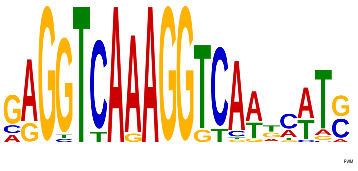	*NNNGTCANNNNNNAATCNNN*	16.52	M	7
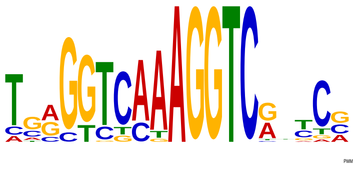	*NNGGGTNNNNNNCATCNN*	5.61	M	8
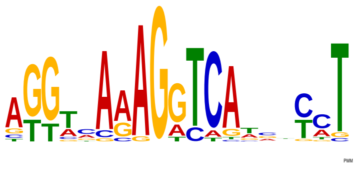	*NNNTCAANNNNNATCANNN*	3.95	DR:4	9

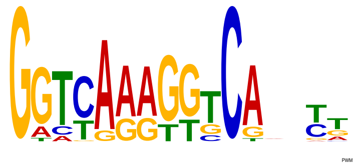	$\overrightarrow{NGTCA}NNNNNN\overrightarrow{CAGATN}$	7.30	DR:6	10
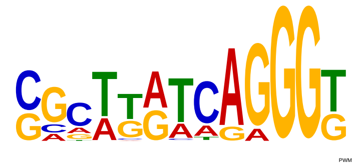	$NGCTTNTC\overrightarrow{AGGGN}$	13.27	M	11
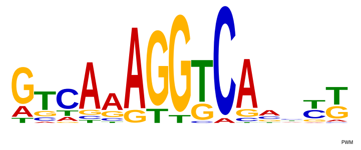	$\overrightarrow{NTCA}ANNNNN\overrightarrow{AGATN}$	2.90	DR:6	12
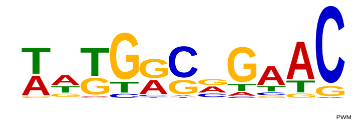	$NA\overrightarrow{TGGNA}GAAN$	3.37	M	13
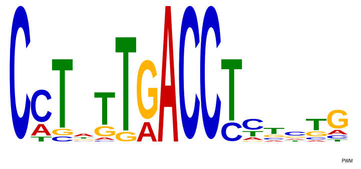	$NNTATTNNNNTCTCNN$	4.94	M	14
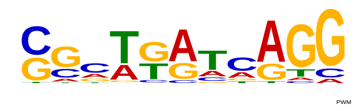	$NGC\overrightarrow{TTNTCA}GN$	3.38	M	15
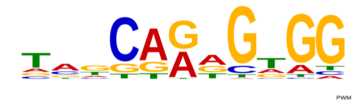	$TAGCNNNGTGG$	4.19	M	16
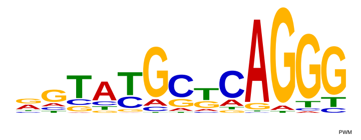	$NGTATNNNC\overrightarrow{AGGN}$	7.38	M	17
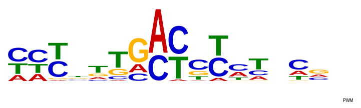	$NNTTTTNNNNNCTACNN$	4.00	M	18

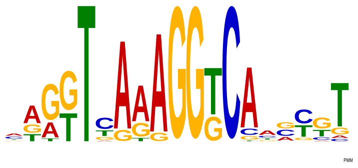	$N\overrightarrow{NGGTCN}NNNNNNNAAATNN$	7.74	M	19
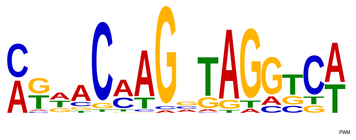	$NNNACAANNT\overrightarrow{AGGNNN}$	7.24	M	20

322 EAR2-Yin2017-Rnd2 (ERR1895322)

PWM	Seed Sequence	Seed Seq Enrichment	Repeat	Rank
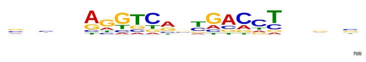	NNNNNNGGTCANTGACCNNNNNN	5.23	IR:1	1
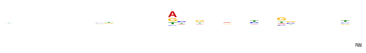	TTTGNNNNNNNNNNNNNNNNNNNNNNNNNNNNNNNAGTT	1.59	M	2
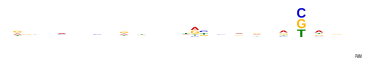	TGGTNNNNNNNNNNNNNNNNNNNNNNNNNNNNNNNNCGTT	1.60	M	3
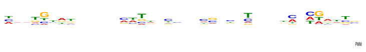	NAATGNNNNNNNNNNNNNNNNNNNNNNNNNNNNNNGATTN	1.88	M	4
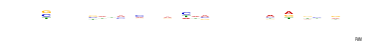	AATGNNNNNNNNNNNNNNNNNNNNNNNNNNNNNNTGTC	1.42	M	5
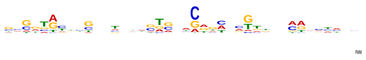	NCGGTNNNNNNNNNNNNNNNNNNNNNNNNNNNNNNCTATN	1.89	M	6
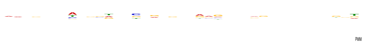	AAGGNNNNNNNNNNNNNNNNNNNNNNNNNNNNNNNAGGT	1.68	DR:28	7
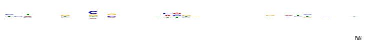	CCTTNNNNNNNNNNNNNNNNNNNNNNNNNNNNNNCGTT	1.57	M	8
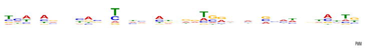	NCAAANNNNNNNNNNNNNNNNNNNNNNNNNNNNTATTN	1.97	M	9

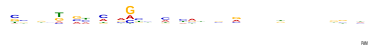	NCGGCNNNNNNNNNNNNNNNNNNNNNNNNNNNNNNCGGGN	1.53	M	10
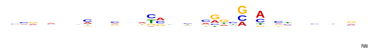	NCGGANNNNNNNNNNNNNNNNNNNNNNNNNNNNNCTCGN	1.57	M	11
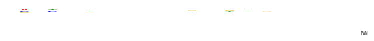	CATTNNNNNNNNNNNNNNNNNNNNNNNNNNNNNNN$\overrightarrow{AGGT}$	1.58	M	12
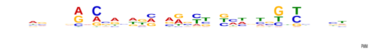	NNCATGNNNNNNNNNNNNNNNNNNNNNNNNNNNCATGNN	0.78	M	13
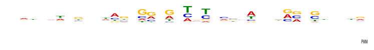	$\overrightarrow{NATCA}$NNNNNNNNNNNNNNNNNNNNNNNNNNNNNTAATN	1.63	M	14
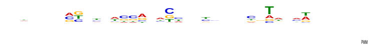	NTGCANNNNNNNNNNNNNNNNNNNNNNNNNNNNGTATN	1.14	M	15
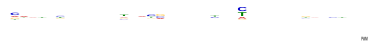	CAATNNNNNNNNNNNNNNNNNNNNNNNNNNNNN$\overrightarrow{NNCTCA}$	1.70	M	16
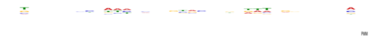	NN$\overrightarrow{ACGTNN}$NNNNNNNNNNNNNNNNNNNNNNNNN$\overrightarrow{ACGTNN}$	1.32	DR:24	17
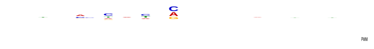	NNCTTCNNNNNNNNNNNNNNNNNNNNNNNNNNCATGNN	1.55	M	18

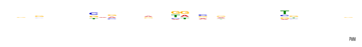	$\overrightarrow{\mathit{CAGGNN}}$NNNNNNNNNNNNNNNNNNNNNNNNNNNNNGAGG	1.41	M	19
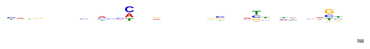	N$\overrightarrow{\mathit{ATGTN}}$NNNNNNNNNNNNNNNNNNNNNNNNNNN$\overrightarrow{\mathit{NAGTCN}}$	1.82	DR:26	20

323 EAR2-Yin2017-Rnd2 (ERR1895323)

PWM	Seed Sequence	Seed Seq Enrichment	Repeat	Rank
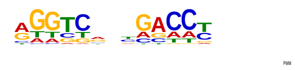	NNNNNNGGTCNNNGACCNNNNNN	15.87	IR:1	1
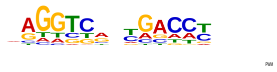	NNNNNNAAGGNNNNTGACNNNNNN	8.13	IR:1	2
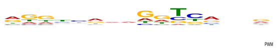	NNNNNNGGTCNAAGGNNNNNN	3.13	DR:1	3
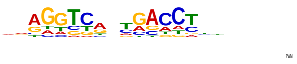	NNNNNNAAGGNNNNNNNCCTTNNNNNN	5.92	IR:1	4
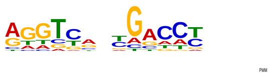	NNNNNNGGGTNNNCGACNNNNNN	6.21	IR:1	5
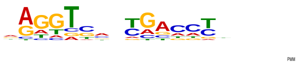	NNNNNNGGTGCNNGACCNNNNNN	4.67	IR:1	6
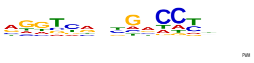	NNNNNNAAGGNNNACGANNNNNN	3.61	M	7
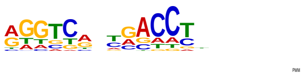	NNNNNNGTCAGNNNCCTTNNNNNN	9.65	IR:1	8
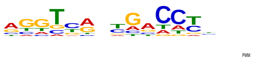	NNNNNNAAGGNNNCCGANNNNNN	3.36	M	9

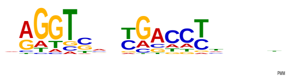	NNNNNNAGGTGCNNGACCNNNNNN	9.47	IR:1	10
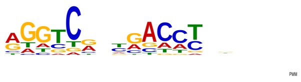	NNNNNNNGTCGTNNACCCNNNNNN	7.14	IR:1	11
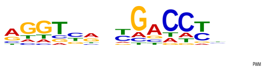	NNNNNNAGGTNNCCGANNNNNN	5.23	M	12
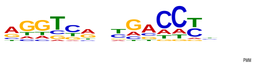	NNNNNNAAGGNNNCTGANNNNNN	4.49	IR:1	13
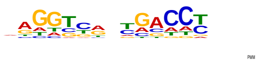	NNNNNNAAAGGNNNCTGANNNNNN	5.74	IR:1	14

324 EAR2-Yin2017-Rnd3 (ERR1895325)

PWM	Seed Sequence	Seed Seq Enrichment	Repeat	Rank
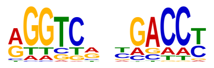	*NNNNNNGGTCNNNGACCNNNNNN*	26.39	IR:1	1
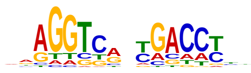	*NNNNNNAAGGNNNNTGACNNNNNN*	13.46	IR:1	2
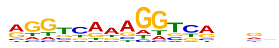	*NNNNNNGGTCNNAGGTNNNNNN*	5.80	DR:1	3
	NNNNNNGGGGNNNNNNNNNNNNNNGGGGNNNNNN	4.44	M	4
	NNNNNNGGGGNNNNNNNNNNNNNNNNNGGGGNNNNNN	4.27	M	5
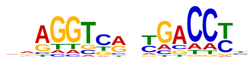	*NNNNNNAAGGNNNCTGANNNNNNN*	7.45	IR:1	6
	NNNNNGGGGNNNNNNNNNNNNNNNNNNNNNNGGGGNNNNN	4.60	M	7
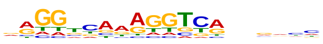	*NNNNNNNTCAANNGTCANNNNNNN*	4.94	DR:1	8
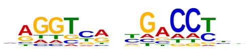	*NNNNNNAAGGNNNACGANNNNNNN*	5.10	M	9

PWM	NNNNGGGGNNNNNNNNNNNNNNNNNNNNNNNGGGGNNNN	4.87	M	10
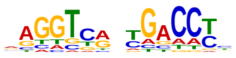 PWM	NNNNNNA$\overrightarrow{AGGNNN}$CCGANNNNNN	5.14	M	11
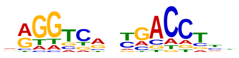 PWM	NNN$\overrightarrow{NNNTCA}$GNNNNCTTTNNNNNN	4.43	M	12
PWM	NNGGGGNNNNNNNNNNNNNNNNNNNNNNNNNNNGGGGNN	4.43	M	13
PWM	NNNGGGGNNNNNNNNNNNNNNNNNNNNNNNNNNGGGGNNN	4.25	M	14

325 EAR2-Yin2017-Rnd4 (ERR1005224)

PWM	Seed Sequence	Seed Seq Enrichment	Repeat	Rank
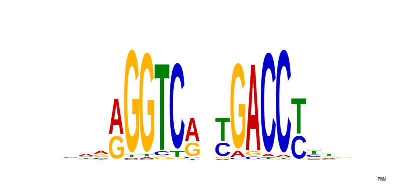	NNNNNNGGTCNNNGACCNNNNNN	100.44	IR:1	1
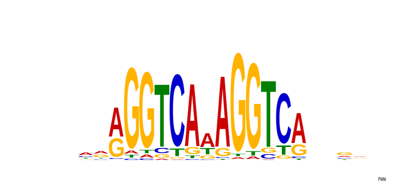	NNNNNNGGTCNNAGGTCNNNNNN	67.08	DR:1	2
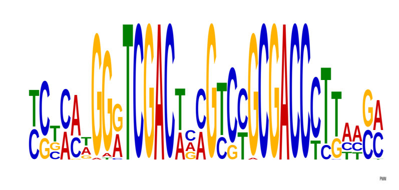	NNNNNNGGGTCGACNNNNNNNNGCGACCCNNNNNN	6671.64	IR:10	3
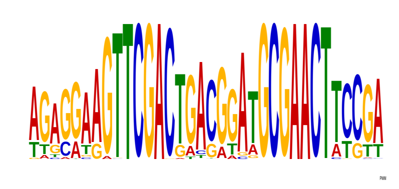	NNNNNNAGTTCGACNNNNNNNNNGCGAACNNNNNN	9784.05	IR:11	4
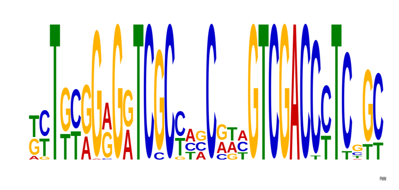	NNNNNNGAGGTCGCNNNNNNNNGTCGACCNNNNNN	3788.15	IR:10	5
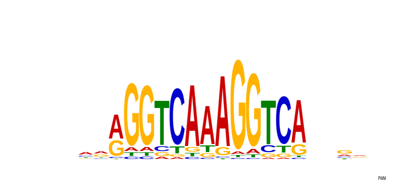	NNNNNNGGTCNNNGGTCNNNNNN	32.48	DR:1	6
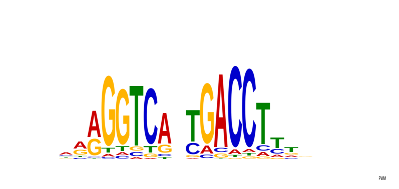	NNNNNNGTCANNNNNCTTTNNNNNN	19.03	M	7
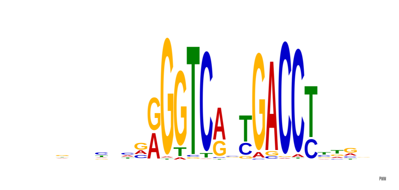	NNNNNNACGGGGTCNNNGACCNNNNNN	213.48	IR:1	8
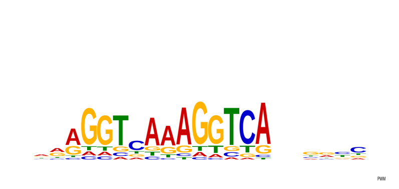	NNNNNNCAAANNTCAANNNNNN	11.25	M	9

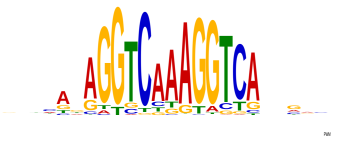	$NNNNNNAT\overrightarrow{AGGTC}NN\overrightarrow{NGGTC}NNNNNN$	77.21	DR:1	10
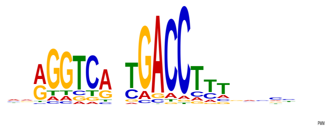	$NNNNN\overrightarrow{NGGTC}NNNNNNNTTTGNNNNNN$	11.60	M	11
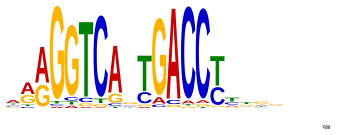	$NNNN\overrightarrow{NNGTCA}G\overleftarrow{NNNCCT}CNNNNNN$	26.51	IR:1	12
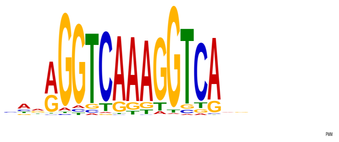	$NNNNN\overrightarrow{NGGTC}NN\overrightarrow{NNGTCA}AGNNNNNN$	43.29	DR:1	13

326 EAR2-Yin2017-Rnd4 (ERR1005225)

PWM	Seed Sequence	Seed Seq Enrichment	Repeat	Rank
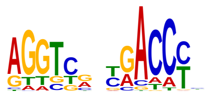	NNNNNNAGGTNNNNNACCCNNNNNN	24.75	IR:1	1
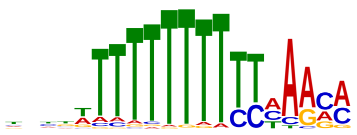	NNNNNNTTTTTTTTNNNNNN	28.18	M	2
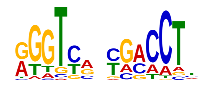	NNNNNNGGGTNNNNNNNCTTTNNNNNN	18.09	M	3
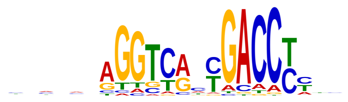	NNNNNNCAGGNNNCCGANNNNNN	12.02	M	4
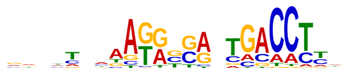	NNNNNNGAAANNNNACTGNNNNNN	8.22	M	5
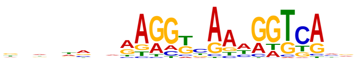	NNNNNNGAAANNNNAAAGNNNNNN	8.06	M	6
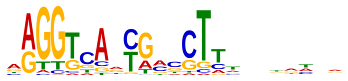	NNNNNNCAACNNNNTTTTNNNNNN	8.00	M	7
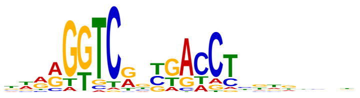	NNNNNNTCGGNNNNCTCGNNNNNN	9.33	M	8
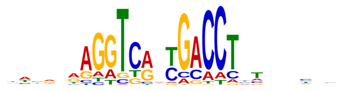	NNNNNNCAAGNNNNNNNNNCTATNNNNNN	7.09	M	9

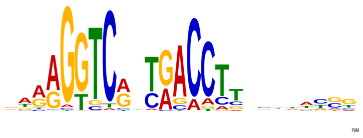	NNNNNNTCGGNNNNNCTTAGNNNNNNN	12.83	M	10
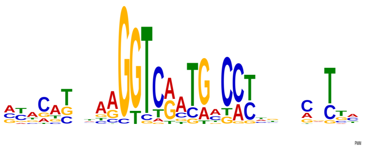	NNNN$\overrightarrow{NNGTAA}$NNNNNNNNNNNNNTTTTNNNNNN	4.14	M	11
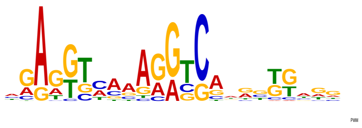	NNNNNNCAAANNNNNAAATNNNNNN	4.19	M	12
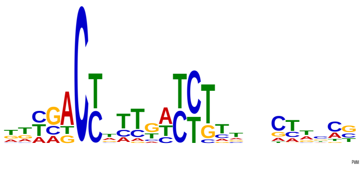	NNNNNNCTTTNNNNTTTTCNNNNNN	10.09	M	13
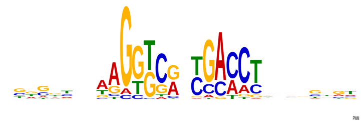	NNNNNNGAAANNNNNNNNNNNCTCTNNNNNN	5.09	M	14
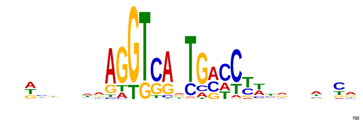	NNNNNNGAAANNNNNNNNNNNNTTTGNNNNNN	5.55	M	15
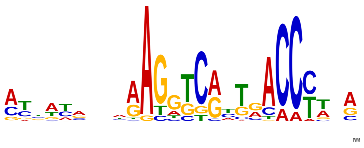	NNNNNNCAAAANNN$\overrightarrow{NGTTGA}$NNNNNN	20.17	M	16

327 EAR2-Yin2017-Rnd4 (ERR1895327)

PWM	Seed Sequence	Seed Seq Enrichment	Repeat	Rank
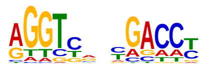	NNNNNNAGGTNNNNGACCNNNNNN	16.81	IR:1	1
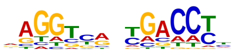	NNNNNNAGGNNNCTGANNNNNN	6.86	IR:1	2
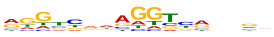	NNNNNNGGTCNNAGGTNNNNNN	4.19	DR:1	3
	NNNNNNGGGANNCGGGNNNNNN	2.95	M	4
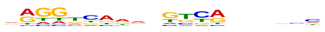	NNNNNNTCAANNGTCANNNNNN	3.53	DR:1	5
	NNNNGGGGNNNNNNNNNNNNNNNNNNNNNNNNNGGGGNNNN	2.58	M	6
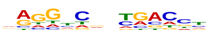	NNNNNNTCGTNNNNCTCTNNNNNN	2.62	M	7
	NNNNNNAAGGNNNAAGGNNNNNNN	3.02	DR:1	8
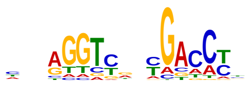	NNNNNNGCAGGTNNNCGACNNNNNN	9.63	IR:1	9

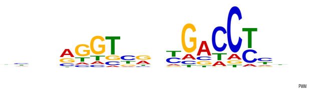	NNNNNNCAGGTNNCCGANNNNNN	5.50	M	10
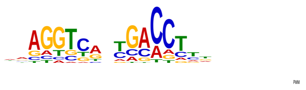	NNNNNNAAGGNNNNNNNNCTTTCNNNNNN	5.25	M	11
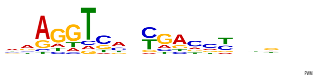	NNNNNNGGTGCNNGACCNNNNNN	4.00	IR:1	12
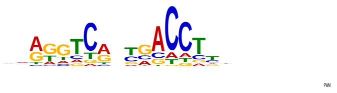	NNNNNNGTCGNNNNNCTTTCNNNNNN	4.99	M	13
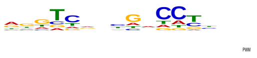	NNNNNNCAGGNNNACGANNNNNN	2.71	M	14

328 EAR2-Yin2017-Rnd2 (ERR1005228)

PWM	Seed Sequence	Seed Seq Enrichment	Repeat	Rank
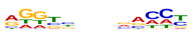	*NNNNNNAGGTNNNNNACCTNNNNNN*	10.24	IR:1	1
	NNNNNNGTCANNNGTCANNNNNN	4.43	DR:1	2
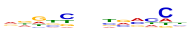	*NNNNNNGTCANNNNNCTTTNNNNNN*	5.68	M	3
	NNNNNNGTCANNNNNNNNNTGACNNNNNN	3.57	IR:9	4
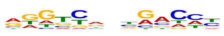	*NNNNNNGTCGNCGACNNNNNN*	5.53	IR:1	5
	NNNNNNAAGGNNNNNNNNNCCTTNNNNNN	5.04	IR:2	6
	NNNNNNGTCANNNNNNNNTGACNNNNNN	4.45	IR:8	7
	NNNNNNAGGTNNNNNNNNNNNNACCTNNNNNN	3.02	IR:10	8
	NNNNNNAAAGNNNAAAGNNNNNN	3.86	M	9

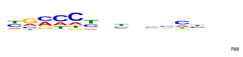	NNNNNNCACGNNNTTTGNNNNNN	2.85	M	10
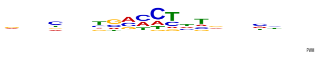	NNNNNNCAATNNNNTTTGNNNNNN	3.64	M	11
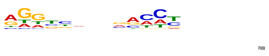	NNNNNNAAGGNNNNNNACCTNNNNNN	8.10	IR:1	12
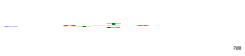	NNNNNNGTCANNNNGTCANNNNNN	2.87	DR:2	13
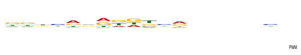	NNNNNNTCAGNNNTCAGNNNNNN	3.32	DR:1	14
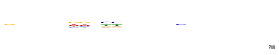	NNNNNNAGGTNNNNNNNNNNNNNACCTNNNNNN	3.43	IR:9	15
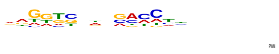	NNNNNNAGAGNNNNNNNNNCTCTNNNNNN	3.14	M	16
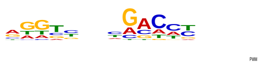	NNNNNNCAGGTNNNCGACNNNNNN	7.68	IR:1	17
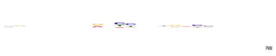	NNNNNNAAGGNNNNNNNNNNNNNTGACNNNNNN	3.34	IR:9	18

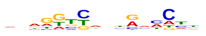	*NNNNNNATAGNNNNNNNNNNCTTTNNNNNN*	3.75	M	19
	$NNN\overrightarrow{NNNTCA}A\overrightarrow{NNNTCA}TNNNNNN$	3.95	DR:1	20

329 EAR2-Yin2017-Rnd2 (ERR1005229)

PWM	Seed Sequence	Seed Seq Enrichment	Repeat	Rank
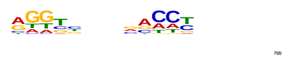	*NNNNNNAGGTNNNNNACCTNNNNNN*	12.00	IR:1	1
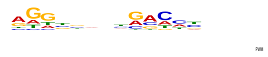	*NNNNNNAAGGNNNNTGACNNNNNN*	8.56	IR:1	2
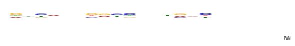	*NNNNNNGTCANNNNNNNNNTGACNNNNNN*	4.65	IR:8	3
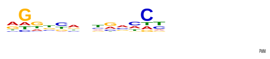	*NNNNNNAAAGNNNNNNNNNCTTTNNNNNN*	5.28	M	4
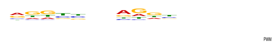	*NNNNNNAGGTNNNAGGTNNNNNN*	4.68	DR:1	5
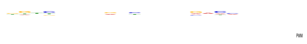	*NNNNNNGTCANNNNNNNNNNTGACNNNNNN*	3.91	IR:9	6
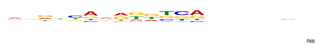	*NNNNNNTCAANNNTCAGNNNNNN*	3.99	DR:1	7
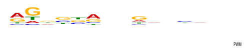	*NNNNNNAAAGNNNAAAGNNNNNN*	4.37	M	8
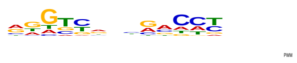	*NNNNNNGTCANCGACNNNNNN*	6.23	IR:1	9

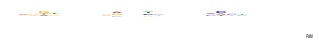	*NNNNNNAGGTNNNNNNNNNNNNNNNACCTNNNNNN*	3.35	IR:10	10
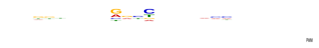	*NNNNNNAGGTNNNNNNNNNNNNNACCTNNNNNN*	3.38	IR:8	11
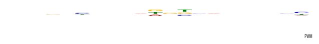	*NNNNNNNGTCANNNNNGTCANNNNNNN*	3.08	DR:2	12
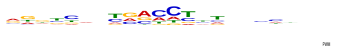	*NNNNNNCAGTNNNNTTTGNNNNNN*	3.48	M	13
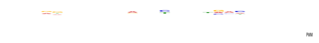	*NNNNNNAAGGNNNNNNNNNNNNNTGACNNNNNN*	3.26	IR:10	14
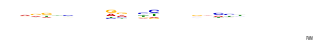	*NNNNNNAGGTNNNNNNNNNNNNNNACCTNNNNNN*	3.92	IR:9	15
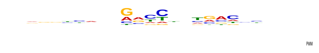	*NNNNNNAGGCNNNNNNNNNNTGACNNNNNN*	3.14	IR:8	16
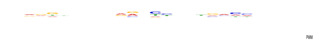	*NNNNNNAGGTNNNNNNNNNNNTGACNNNNNN*	3.96	IR:9	17
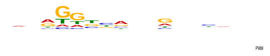	*NNNNNNTAAANNNNAAAGNNNNNN*	2.93	M	18

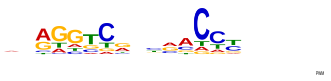	*NNNNNNGTCGTNAACCNNNNNN*	7.61	IR:0	19
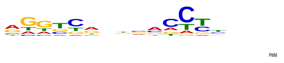	*NNNNNNGGTCNNGCACNNNNNN*	4.03	M	20

330 EAR2-Yin2017-Rnd3 (ERR1005230)

PWM	Seed Sequence	Seed Seq Enrichment	Repeat	Rank
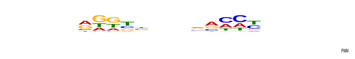	NNNNNNAGGTNNNNNACCTNNNNNN	7.94	IR:1	1
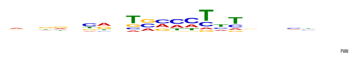	NNNNNNCACTNNNNTTTGNNNNNN	4.82	M	2
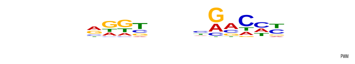	NNNNNNAGGTNNNCGACNNNNNN	7.79	IR:1	3
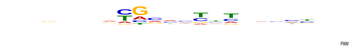	NNNNNNCACGNNNTTTGNNNNNN	3.47	M	4
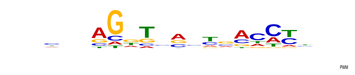	NNNNNNAGTTNNCTGANNNNNN	4.18	IR:1	5
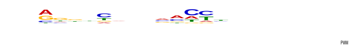	NNNNNNTCGTNNNNCTTGNNNNNN	3.02	M	6
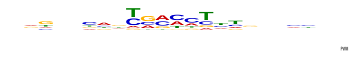	NNNNNNCAGCNNNNTTTGNNNNNN	3.54	M	7
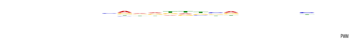	NNNNNNTCAGNNNTCAGNNNNNN	2.68	DR:1	8
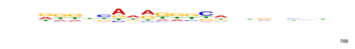	NNNNNNTCAANNNNCAGTNNNNNN	3.95	M	9

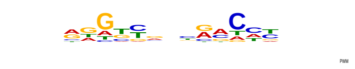	NNNNNNNGTCGNCGACNNNNNN	5.47	IR:1	10
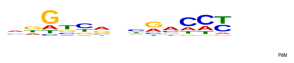	NNNNNNNGTCACCGANNNNNN	3.85	M	11
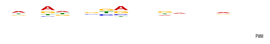	NNNNNNTAAANNNNAAAGNNNNNN	3.17	M	12
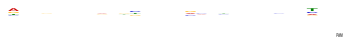	NNNNNNCAAANNNNNNNNNNNNNTTTGNNNNNN	2.87	M	13
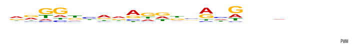	NNNNNNCAAANNNNACGTNNNNNN	2.84	M	14
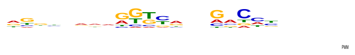	NNNNNNATAGNNNNNNGACANNNNNN	3.11	M	15
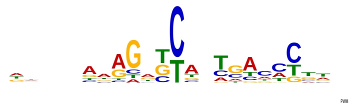	NNNNNNAAAGANNACTGANNNNNN	11.35	IR:1	16
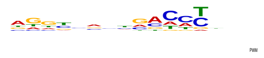	NNNNNNTAGGNNNCTGANNNNNN	3.59	IR:1	17
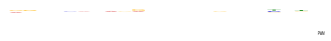	NNNNNNTCAANNNNNNNATGANNNNNN	2.69	IR:8	18

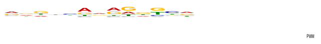	NNNNNNTCAANNNNNCATGNNNNNN	3.24	M	19
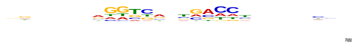	NNNNNNAGAGNNNNNNNNNNCTCTNNNNNN	3.16	M	20

331 EAR2-Yin2017-Rnd3 (ERR1005231)

PWM	Seed Sequence	Seed Seq Enrichment	Repeat	Rank
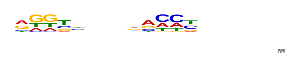	NNNNNNAGGTNNNNNACCTNNNNNN	8.42	IR:1	1
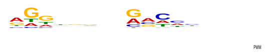	NNNNNNAAGGNNNNTGACNNNNNN	6.39	IR:1	2
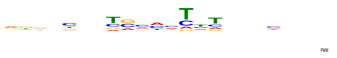	NNNNNNCAATNNNNTTTGNNNNNN	4.78	M	3
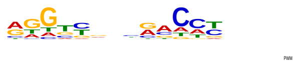	NNNNNNGTCGNCGACNNNNNN	5.48	IR:1	4
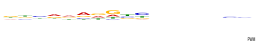	NNNNNNGGTCNNNNGTCGNNNNNN	2.80	DR:1	5
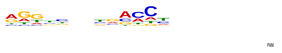	NNNNNNAAGGNNNNNNNNCTTCNNNNNN	4.11	M	6
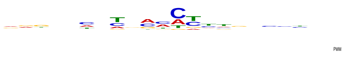	NNNNNNCGTGNNNCTTGNNNNNN	3.03	M	7
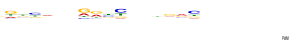	NNNNNNGTCANNNNNNNNNGACANNNNNN	3.19	DR:7	8
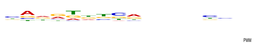	NNNNNNTCAGNNNNTCAGNNNNNN	3.14	DR:1	9

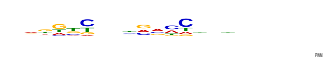	NNNNNNGTCANNNNNCTATNNNNNN	4.56	M	10
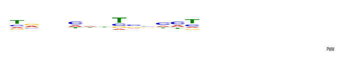	NNNNNNCTTTNNNNTTTTNNNNNN	3.13	M	11
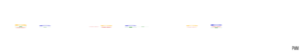	NNNNNNGTCANNNNNNNNNTGACNNNNNN	2.80	IR:9	12
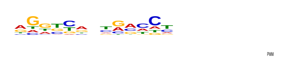	NNNNNNAGAGNNNNNNNNNCTCTNNNNNN	3.36	M	13
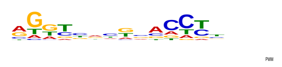	NNNNNNAGGTNNCGCACNNNNNN	5.72	M	14
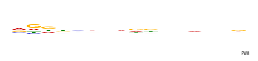	NNNNNNCGAGNNNNNAAGGNNNNNN	2.80	M	15
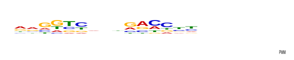	NNNNNNTAAGNNNNNNNNNNCTTANNNNNN	2.75	M	16
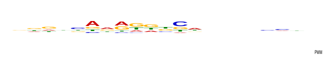	NNNNNNTCAANNNNCAGTNNNNNN	3.66	M	17
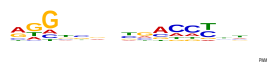	NNNNNNATGTNNCTGANNNNNN	3.13	IR:1	18

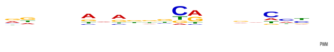	*NNNNNNCAAANNNCGTTNNNNNN*	3.11	M	19
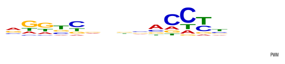	*NNNNNN*$\overrightarrow{GGTC}$*NNGCACNNNNNN*	3.86	M	20

332 HNF4A-Bhimsaria2022-Rnd3 (S07-161)

PWM	Seed Sequence	Seed Seq Enrichment	Repeat	Rank
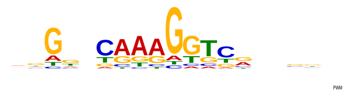	NNNNNNCAAAGGTCNNNNNN	22.30	M	1
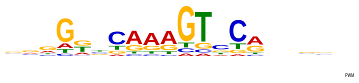	NNNNNNCAAAGTCCNNNNNN	19.10	M	2
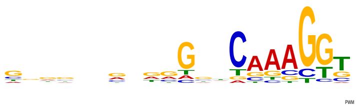	GGGGNNNNNNNNNNNAAAGGT	30.12	DR:9	3
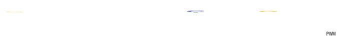	GGGGNNNNNNNNNNNGTCC	4.68	DR:7	4
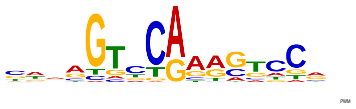	NNAAGTCCANNGTCCNN	47.13	DR:1	5
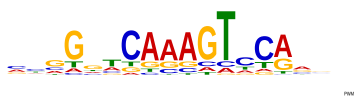	NNNNCTCAAAGTCCNNNN	15.48	DR:1	6
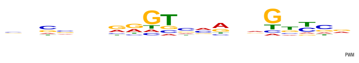	NNNNNNGGGTCCAANNNNNN	8.71	M	7
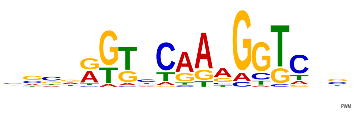	NNNGGGTCCAAAGGTNNN	108.97	DR:1	8
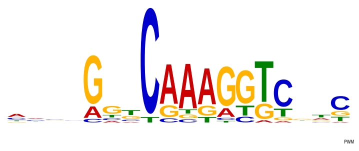	NNNCGGTCAAAGGTCNNN	137.49	DR:1	9

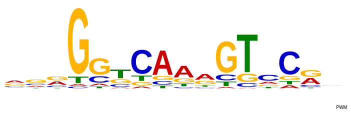	$NN\overrightarrow{AGGTCN}\overrightarrow{AGGTCC}NN$	39.79	DR:1	10
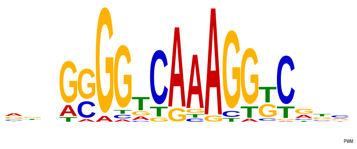	$NNNG\overrightarrow{GGGTCN}A\overrightarrow{AGGGCN}NN$	134.62	DR:1	11
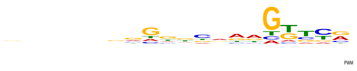	$NNNNNNG\overrightarrow{GGGGCA}ANNNNNN$	6.94	M	12

333 HNF4A-Jolma2013-Rnd2 (ERR194035)

PWM	Seed Sequence	Seed Seq Enrichment	Repeat	Rank
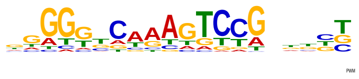	NNNNNNCAAAGTCCNNNNNN	4.66	M	1
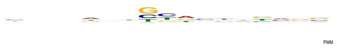	NNNNAAGTNNAGTTNNNN	2.07	DR:0	2
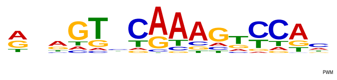	NNNNGTCCAAAGTNNNN	6.05	DR:0	3
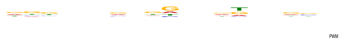	GGGGNNNNNNNNNNNNGGGG	2.06	DR:9	4
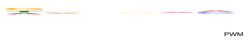	ACGGCGAC	1.61	M	5
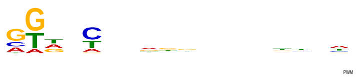	NNNNNAAAGGTCANNNNN	1.57	M	6
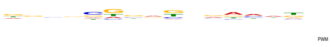	NGGCGNNNNNNNAAATN	1.80	M	7
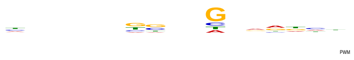	NNGTCGNNNNNGAAGNN	1.47	M	8
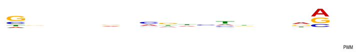	NNATCANNNNNNTATANN	1.31	M	9

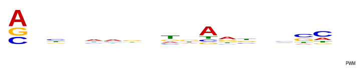	NNNAAAGNNNNAGGCNNN	1.73	M	10
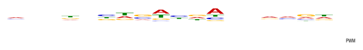	NTTTGNNNNNNNNNAAGTN	1.73	M	11
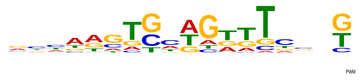	NNNAAGTNNNGTTTCNNN	4.11	DR:1	12
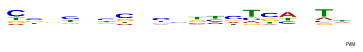	NNNCGCCNNNNNCTCANNN	2.34	M	13
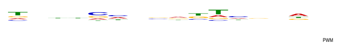	NTGTTNNNNNNNCGCAN	1.81	M	14
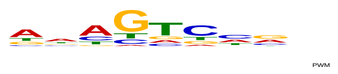	AAAGTCCAT	3.48	M	15
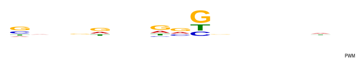	NATGGNNNNNNNGCATN	1.37	M	16
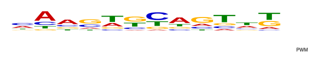	NNCAAGTNNAGTTNN	3.51	DR:0	17
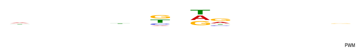	NATGTNNNNNNNTGAAN	1.35	IR:5	18

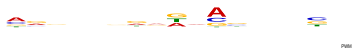	$NNA\overrightarrow{AGGNNN}NNGATANN$	1.36	M	19
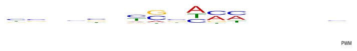	$NCGCCNNNNN\overrightarrow{NNCTCA}N$	1.55	M	20

334 HNF4A-Jolma2013-Rnd2 (ERR194355)

PWM	Seed Sequence	Seed Seq Enrichment	Repeat	Rank
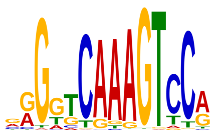	NNNNNNGGGTCNNAGTCCNNNNNN	194.67	DR:1	1
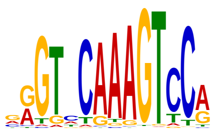	NNNNNNGGTCCNNAGTCCNNNNNN	192.40	DR:1	2
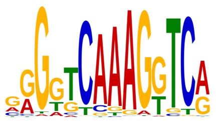	NNNNNNGGGTCANAGGTCNNNNNN	219.64	DR:1	3
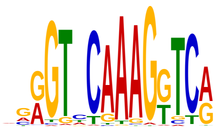	NNNNNNGGGTCCNAAGGTCNNNNNN	339.13	DR:1	4
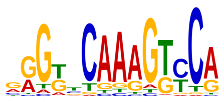	NNNNNNAGGGNNNAAAGNNNNNN	16.98	M	5
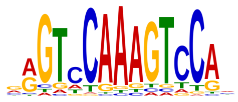	NNNNNNAGTCNNNAGTCNNNNNN	24.30	DR:1	6
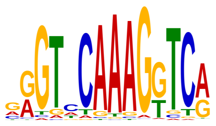	NNNNNNGGTCCNNAGGTCNNNNNN	82.07	DR:1	7
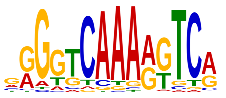	NNNNNNGGTCNNAGGTCNNNNNN	44.22	DR:0	8
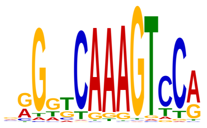	NNNNNNGGTCANNNTCCAANNNNNN	91.99	DR:1	9

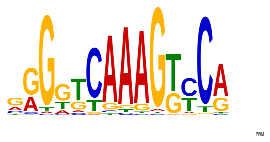	NNNNNNGGGGTNAAAGNNNNNN	49.29	M	10
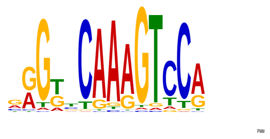	NNNNNNAGGGNNNNAAGTCNNNNNN	40.91	DR:1	11
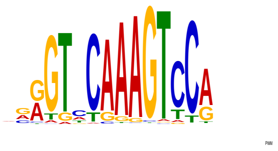	NNNNNNTGGGTCCNNAGTCNNNNNN	119.22	DR:0	12
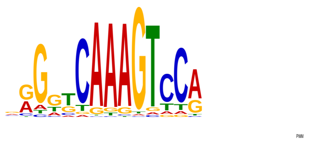	NNNNNNGGTCNNNNTCCAANNNNNN	33.54	DR:0	13
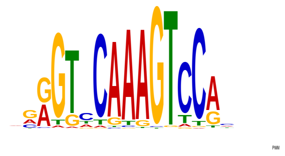	NNNNNNCGGGTCCNNAGTCNNNNNN	132.48	DR:0	14
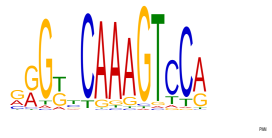	NNNNNNTGGGNNNNAAGTCNNNNNN	33.02	DR:1	15
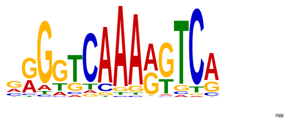	NNNNNNGGGTCNNAGGTNNNNNN	29.72	DR:1	16
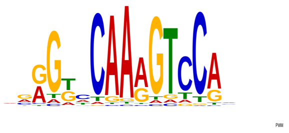	NNNNNNGGGTNNNNAGTCNNNNNN	18.29	DR:2	17

335 Hnf4a-Jolma2013-Rnd2 (ERR195634)

PWM	Seed Sequence	Seed Seq Enrichment	Repeat	Rank
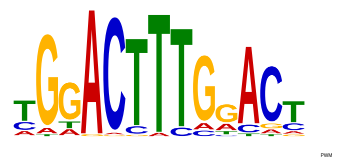	NNGGACTNTGGACNN	73.06	DR:1	1
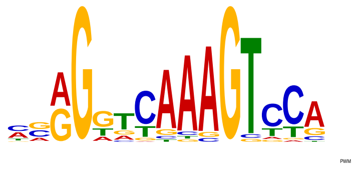	NNNGGTCAAAGTCNNN	48.83	DR:0	2
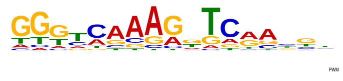	NNNNNCAAAGGTCNNNNN	5.42	M	3
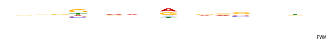	NNNGGGGNNNNNGGGGNNN	3.03	M	4
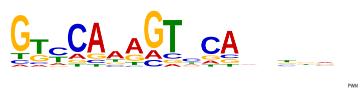	NNNNNAAGTCCAANNNNN	5.20	M	5
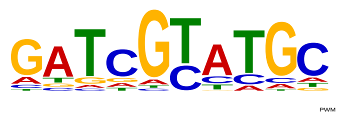	GATCGNATGC	6.41	M	6
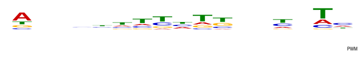	NNNNTTTTNTTTTNNNN	2.58	M	7
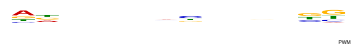	NNAAGGNNATGGNN	1.58	M	8
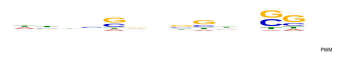	NNNTCGGNGGTCNNN	2.06	M	9

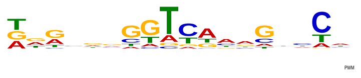	NNNTGGGNNNNAAGTNNN	2.58	M	10
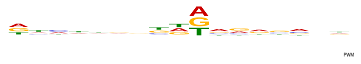	NNNTTGGNNNAGAGNNN	1.97	M	11
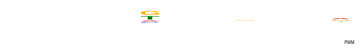	GGGGNNNNNNNNNGGGG	2.30	M	12
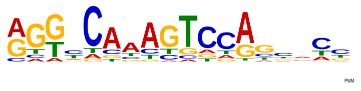	NNNNNAAAGTCCGNNNNN	5.03	M	13
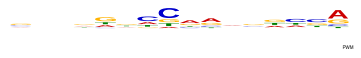	NNNGGTANNAAGTNNN	2.12	DR:1	14
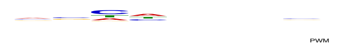	CGATCGTA	0.92	M	15
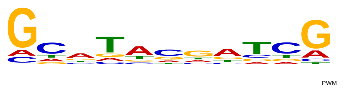	NCATANGATCN	3.09	M	16
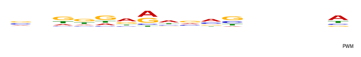	NNNGGAANNAGTGNNN	2.43	M	17
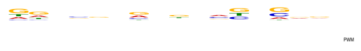	NGGGGNNNNNNNCGGGN	2.27	M	18

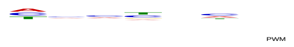	*CACACTCT*	1.46	M	19
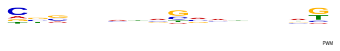	*NNGGGGNNNNNATTTNN*	1.70	M	20

336 HNF4A-Jolma2013-Rnd3 (ERR194036)

PWM	Seed Sequence	Seed Seq Enrichment	Repeat	Rank
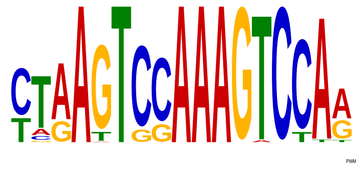	*NTAAGTCNAAAGTCCAN*	6505.86	DR:1	1
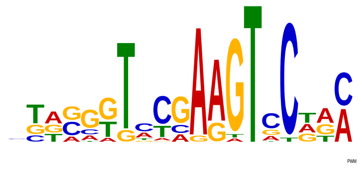	*NNNGGGTCCNAAGTCCNNN*	596.68	DR:1	2
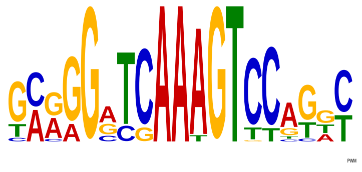	*NNNGGGTCAAAGTCCGNNN*	1070.94	DR:1	3
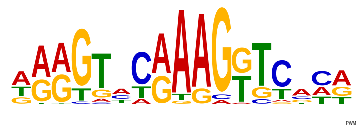	*NNGGTCCNAAGGTCANN*	236.44	DR:1	4
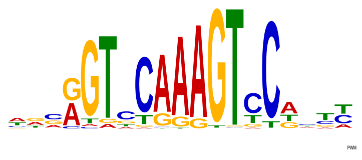	*NNNNGTCCANAGTCCNNNN*	227.90	DR:1	5
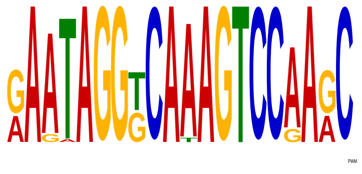	*NNATAGNNNNAAGTCCAANN*	1027.51	M	6
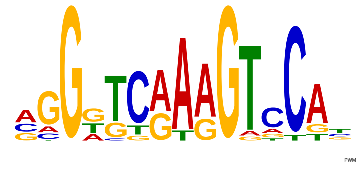	*NGGGTCAAAGTACAN*	323.85	DR:1	7
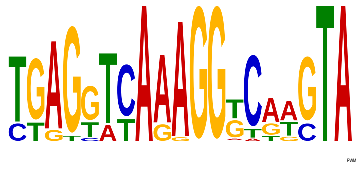	*NNNGGTCAAAGNTCAANNN*	347.16	DR:1	8
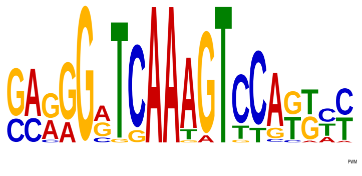	*NNNNGGTCANAGTCCGNNNN*	193.51	DR:1	9

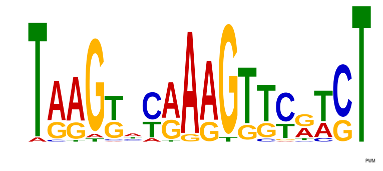	NNNGTCCANAGGTCANNN	86.99	DR:1	10
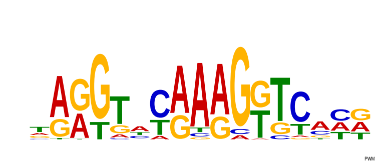	NNGGTCCAANGGTCANN	199.02	DR:1	11
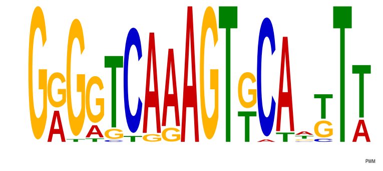	NNGGTCAAAGNNCAATNN	385.36	DR:1	12
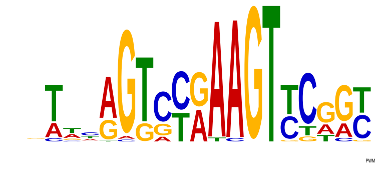	NNNAAGTCNNAAGTCCNNN	178.71	DR:2	13
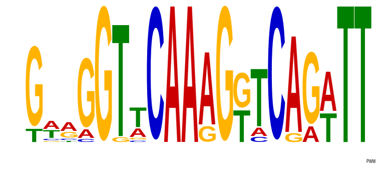	NNNGGTTCAAANNTCAGNNN	924.25	DR:1	14
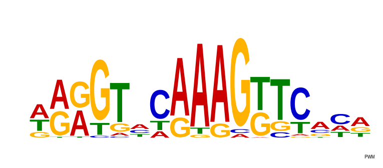	NNGGTCCNNAGGTCANN	80.82	DR:1	15
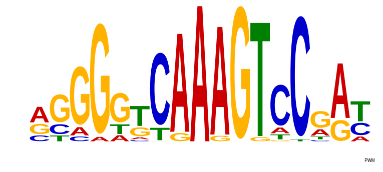	NNGGGGCAAAGTCCGNN	819.00	DR:1	16
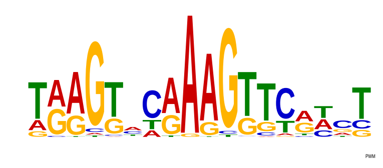	NNNGTCCNNAGGTCANNN	40.53	DR:1	17
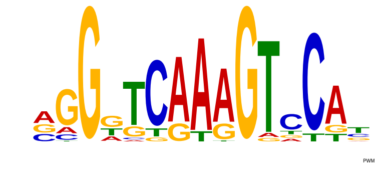	NGGGTCANAGTACAN	148.60	DR:1	18

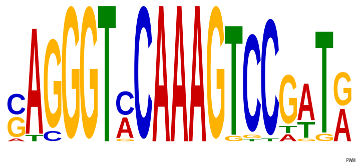	*NNNGGTACANAGTCCGNNN*	596.05	DR:1	19
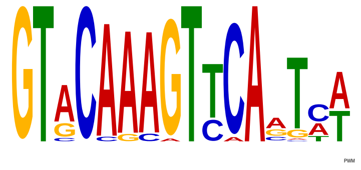	*NTACAAAGNNCAATCN*	148.78	M	20

337 HNF4A-Jolma2013-Rnd3 (ERR194356)

PWM	Seed Sequence	Seed Seq Enrichment	Repeat	Rank
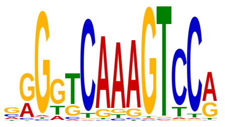	NNNNNNGGTCNNNGTCCNNNNNN	65.85	DR:0	1
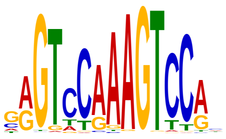	NNNNNNGAGTCNNNAGTCNNNNNN	113.86	DR:1	2
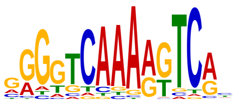	NNNNNNGGTCNNNGGTCNNNNNN	24.89	DR:1	3
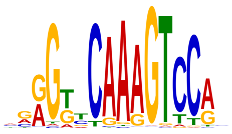	NNNNNNAGGGNNNNNAGTCNNNNNN	37.87	DR:2	4
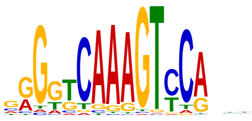	NNNNNNGTCANNNNCCAANNNNNN	33.17	M	5
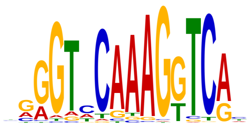	NNNNNNGGTCCNNAGGTNNNNNN	42.58	DR:1	6
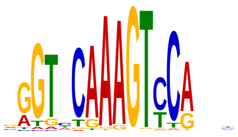	NNNNNNGGTCCNNNNNCCAANNNNNN	54.29	M	7
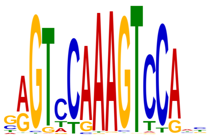	NNNNNNGAGTCNNNNNTCCAACNNNNNN	156.87	DR:2	8
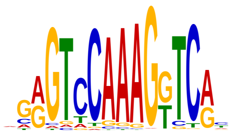	NNNNNNGAGTCNNAGGTCNNNNNN	68.15	DR:2	9

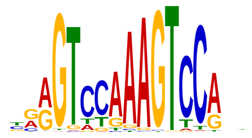	*NNNNNNTAAGTCNNNAGTCNNNNNN*	71.38	DR:1	10
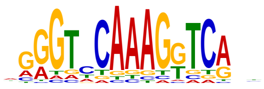	*NNNNNNNTCCANNNGTCANNNNNN*	21.91	DR:1	11
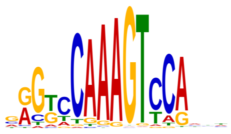	*NNNNNNGAGTNNNNNNNCCAANNNNNN*	16.44	M	12
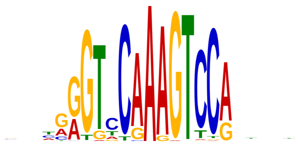	*NNNNNNCGGGTCCNNNNTCCAGNNNNNN*	233.89	DR:1	13
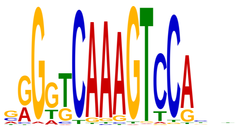	*NNNNNNGGTCNNNNTCCACANNNNNN*	83.71	DR:0	14
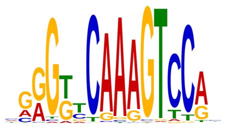	*NNNNNNACGGGNNNNNAGTCNNNNNN*	35.50	DR:1	15
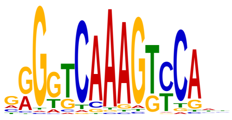	*NNNNNNGTCANNNNNCAACNNNNNN*	18.77	M	16
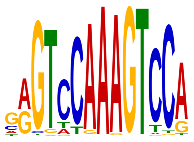	*NNNNNNGAGTCNNNNNTCCAGNNNNNN*	64.02	DR:2	17
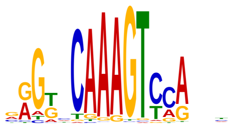	*NNNNNNGGGTNNNNNNNCCAANNNNNN*	17.70	M	18

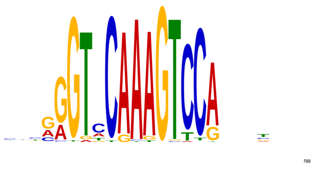	$NNNNNNTA\overrightarrow{GGTCCN}NN\overrightarrow{NNTCCA}TNNNNNN$	214.88	DR:1	19
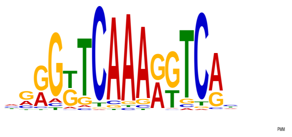	$NNNNNNG\overrightarrow{GGGNNN}N\overrightarrow{AGGTNN}NNNN$	14.18	DR:1	20

338 Hnf4a-Jolma2013-Rnd3 (ERR195635)

PWM	Seed Sequence	Seed Seq Enrichment	Repeat	Rank
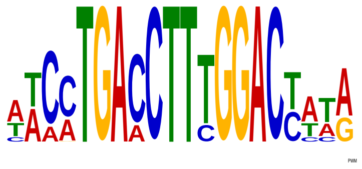	NNNCTGACCTTNGGACTNNN	12756.60	ER:1	1
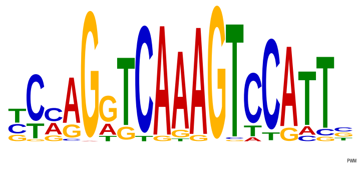	NNNAGGGCAAAGTCCANNN	6235.15	DR:0	2
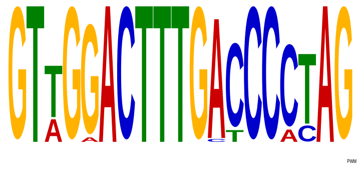	NNAGGACTTTGACCCCTNN	23359.03	IR:1	3
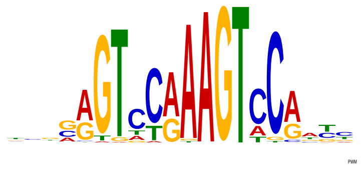	NNNNGGTCNNAAGTCCNNNN	1336.83	DR:1	4
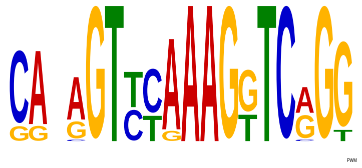	NNCAGTCCAAAGGTCANN	5262.61	DR:1	5
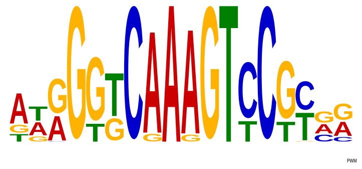	NNAGGTCAAAGTCCGCNN	12282.32	DR:0	6
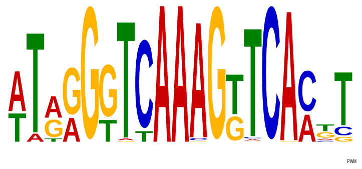	NNNAGGTCAAAGGTCANNN	6878.72	DR:1	7
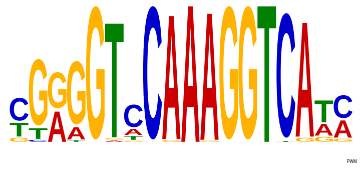	NNGGGTACAAAGGTCANN	12625.98	DR:3	8
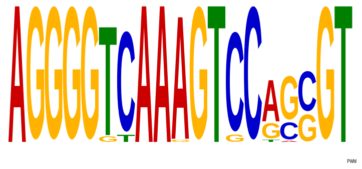	NNGGGTCAAAGTCCAGGNN	33887.82	DR:1	9

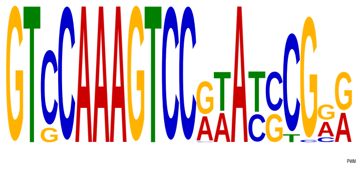	*NTCCAAAGTNNNNNNCCGGN*	2363.04	M	10
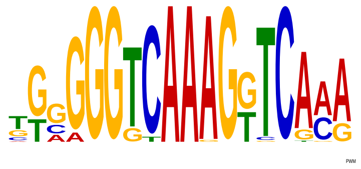	*NNCGGGTCAAAGGTCANN*	10042.84	DR:1	11
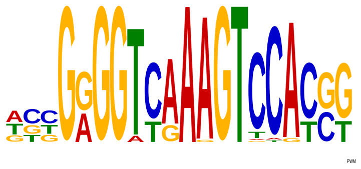	*NNNGGGGTCGAAGTCCANNN*	14911.18	DR:0	12
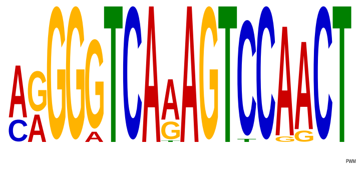	*NNGGGTCAGAGTCCAANN*	6484.27	DR:1	13
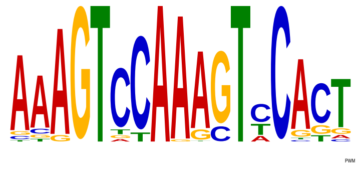	*NNAGTCCAAGGTCCANN*	2738.83	DR:0	14
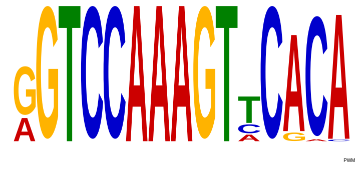	*GGTCCAAAGTACACA*	12516.33	DR:2	15
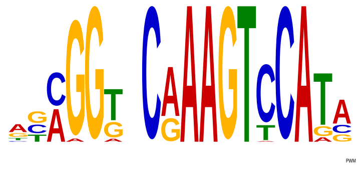	*NNAGGTGCAAAGTCCANN*	10540.94	DR:1	16
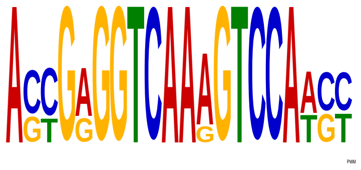	*NNNGAGGTCAAGGTCCANNN*	16855.34	DR:0	17
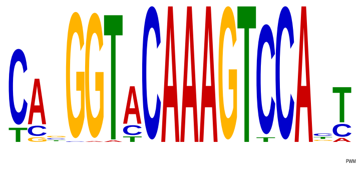	*NNTGGTACAAAGTCCANN*	16445.27	DR:1	18

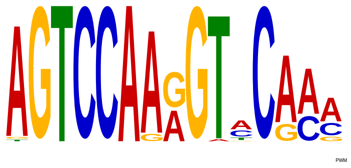	$\overrightarrow{AGTCCA}\overrightarrow{ANGTAC}AAA$	4264.58	DR:0	19
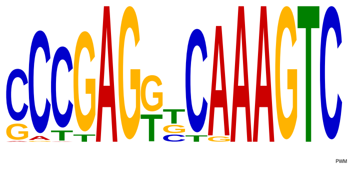	$CCCG\overrightarrow{AGGNNA}\overrightarrow{AAGTC}$	2663.99	DR:0	20

339 HNF4A-Jolma2013-Rnd4 (ERR194037)

PWM	Seed Sequence	Seed Seq Enrichment	Repeat	Rank
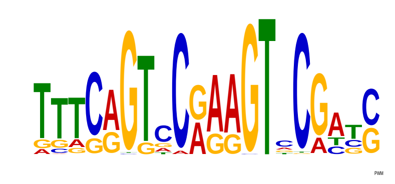	NNNNGGTCCNNNGTCCNNNN	164.22	DR:1	1
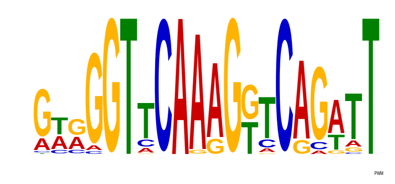	NNNGGTTCAANNNTCAGNNN	517.00	DR:1	2
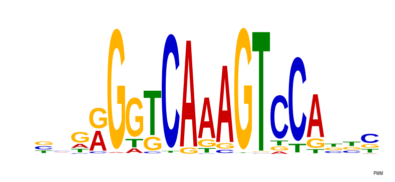	NNNNGGTCNNNGTCCNNNN	104.70	DR:0	3
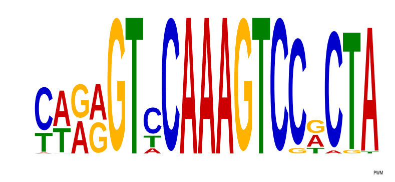	NNNGGTCCNNAGTCCTNNN	221.02	DR:1	4
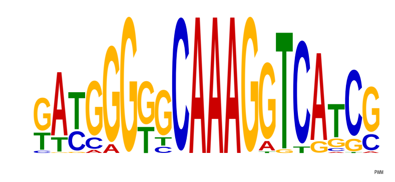	NNNNGGGGCANAGGTCNNNN	160.66	DR:1	5
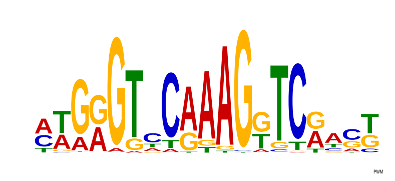	NNNNGTCCNNAGGTCNNNN	102.65	DR:1	6
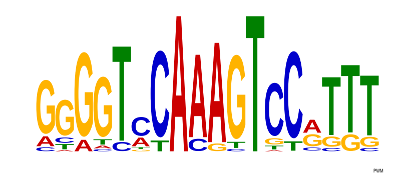	NNNGTCCNNNGTCTANNN	17.95	DR:1	7
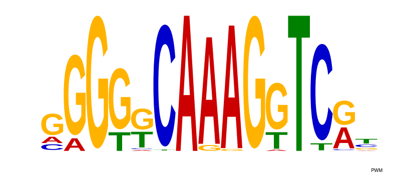	NGGGGCNNAGGTCGN	117.54	DR:1	8
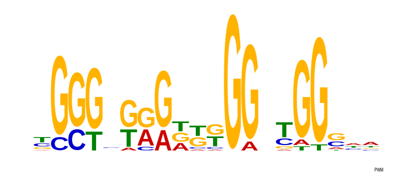	NGGGCGGGNNNNNNNGGGTN	41.73	DR:9	9

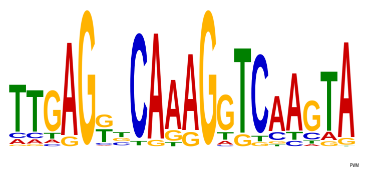	$NNNN\overrightarrow{GGTCNN}N\overrightarrow{GGTCA}NNNN$	47.94	DR:0	10
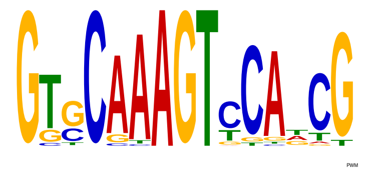	NTGCAANNNCCAGCN	32.03	M	11
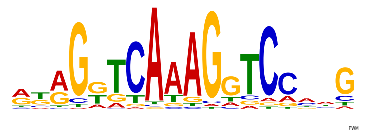	$NNN\overrightarrow{GGTCNN}N\overrightarrow{NGTCGN}NN$	9.42	DR:1	12
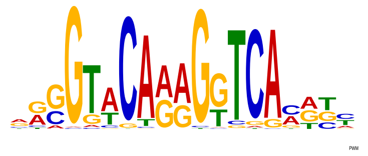	$NNNNTACAN\overrightarrow{NNGTCA}NNNN$	26.12	M	13
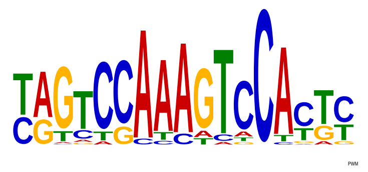	$NN\overrightarrow{TTCCA}NN\overrightarrow{NTCCA}CNN$	16.24	DR:1	14
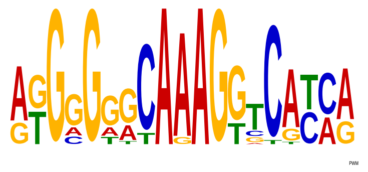	$NNN\overrightarrow{GGGGCA}AN\overrightarrow{NTTCA}NNN$	80.53	DR:1	15
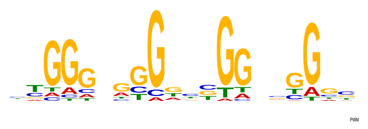	$NN\overrightarrow{GGGCNN}NNNNN\overrightarrow{GGGGGN}N$	13.53	DR:7	16
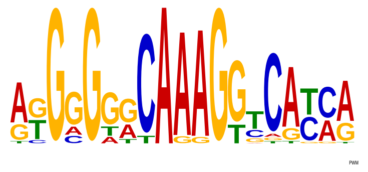	$NNN\overrightarrow{GGGGCA}NN\overrightarrow{NTTCA}NNN$	32.86	DR:1	17
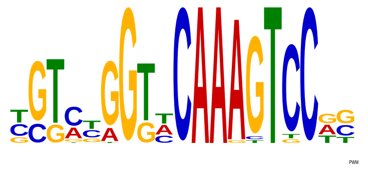	$NNNC\overrightarrow{CGGTNN}NN\overrightarrow{AGTCN}NN$	38.87	DR:1	18

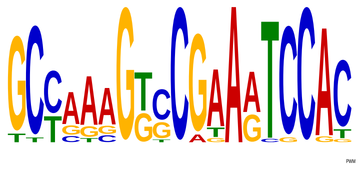	NNNAAAGNNCGAAGTCNNN	102.14	DR:0	19
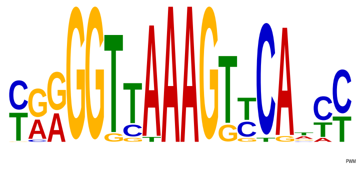	NNNGGTTAAANTTCANNN	92.73	DR:1	20

340 HNF4A-Jolma2013-Rnd4 (ERR194357)

PWM	Seed Sequence	Seed Seq Enrichment	Repeat	Rank
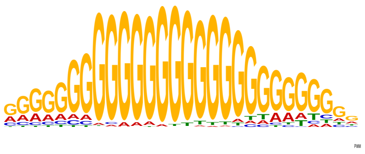	NNNNNNGGGGGGGGGGGGGGGGGGNNNNNN	7568.54	M	1
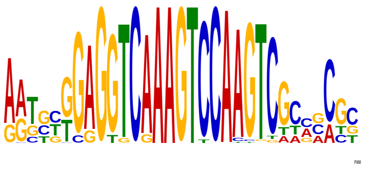	NNNNNNGAGGTNNNNNNCCAAGTCGNNNNNN	1728.99	DR:6	2
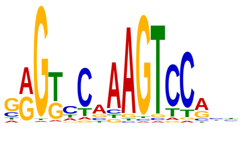	NNNNNNGAGTNNNNAGTCNNNNNN	23.66	DR:2	3
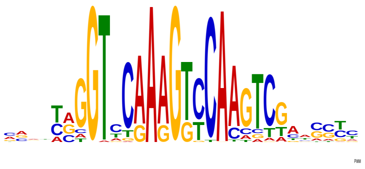	NNNNNNGGTCCNNNNNNCAAGTCGNNNNNN	309.32	DR:6	4
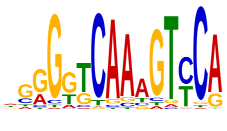	NNNNNNGTCANNNNCCATNNNNNN	18.82	M	5
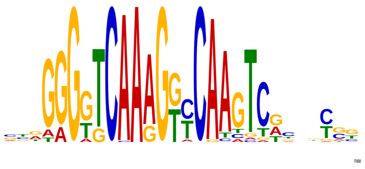	NNNNNNGTCANNNNTCAAGTCGNNNNNN	243.74	DR:6	6
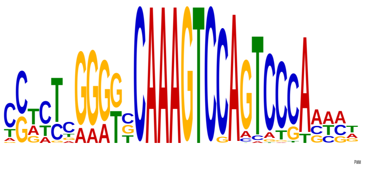	NNNNNCGGGNNNNNNNNCCAGTCCCNNNNN	201.47	DR:7	7
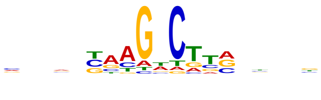	NNNNNNTAAGNCTTANNNNNN	14.00	M	8
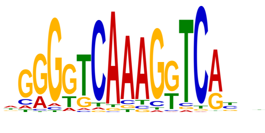	NNNNNNGTCANNNGTCATNNNNNN	21.31	DR:1	9

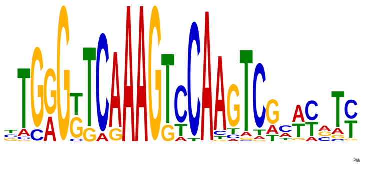	NNNN$\overrightarrow{NGTCA}$NNNNNNCA$\overrightarrow{AGTCGA}$NNNNNN	221.06	DR:7	10
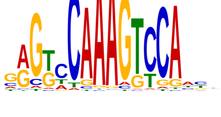	NNNNNN$\overrightarrow{GAGTN}$NNNNNNNNCAACNNNNNN	10.34	M	11
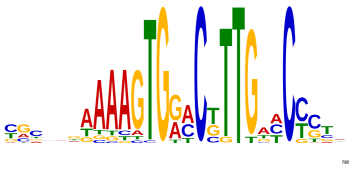	NNNNNNGAA$\overrightarrow{AAGTGN}$NNNNT$\overleftarrow{TGAN}$NNNNNN	293.23	IR:4	12
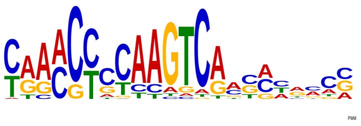	NNNNNNCC$\overrightarrow{AAGTCA}$ACNNNNNN	31.36	M	13
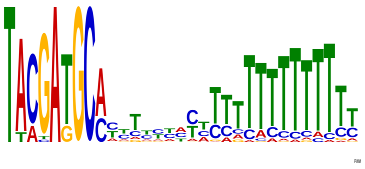	NNC$\overrightarrow{GATGCN}$NNNNNNNNNNNNNTTTTTTTNN	283.52	M	14
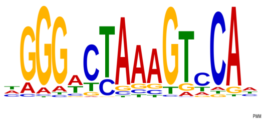	NNNNNNAG$\overrightarrow{GGNCTA}$AANNNNNNN	26.12	M	15
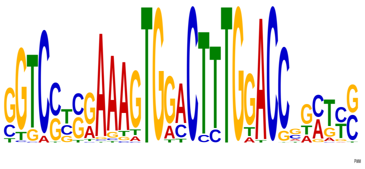	NNNNN$\overrightarrow{NGGAAA}$GTGNNNNN$\overleftarrow{NGGACC}$NNNNNN	594.17	IR:8	16
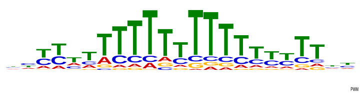	NNNNNNTTTTNNTTTTTNNNNNN	19.54	M	17
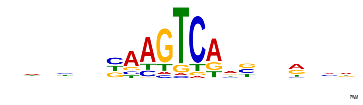	NNNNNNTA$\overrightarrow{AGNCAA}$GNNNNNN	10.17	M	18

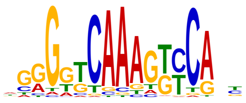 PWM	*NNNNNNGTCANNNNNNCAATNNNNNN*	16.16	M	19
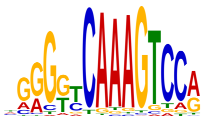 PWM	*NNNNNNCGGGNNNNNNNTCCGNNNNNN*	8.80	M	20

341 Hnf4a-Jolma2013-Rnd4 (ERR195636)

PWM	Seed Sequence	Seed Seq Enrichment	Repeat	Rank
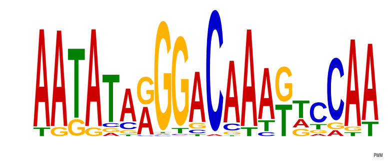	NNNNNNAGGACAAANNNNNN	23.87	M	1
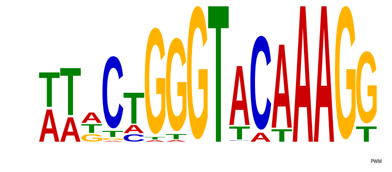	NNACTGGNNACAAANN	30.94	M	2
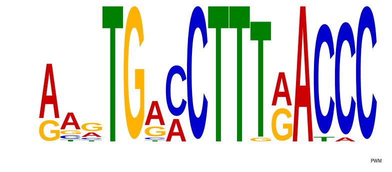	NAGTGANNNNTAACCN	33.63	IR:4	3
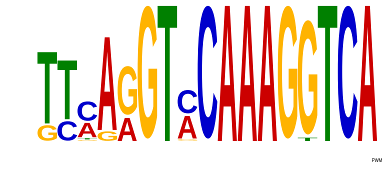	NNCAGGTACNNAGGTNN	54.85	DR:2	4
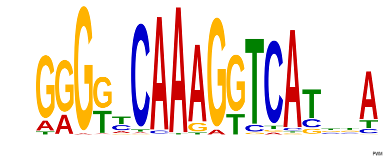	NNNTTCANNNNTCATNNN	27.47	DR:1	5
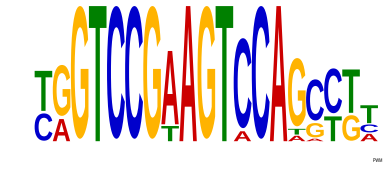	NNNTCCGANNTCCAGCNNN	93.74	M	6
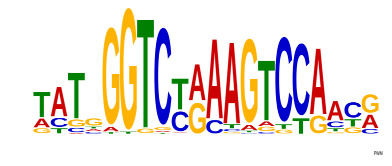	NNNNNGTCTNNAGTCNNNNN	23.61	DR:1	7
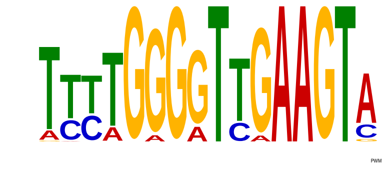	TCTTGGGGNNNNAGTC	85.00	M	8
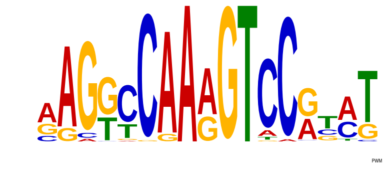	NNGTTCNNNNTCCGGNN	12.08	M	9

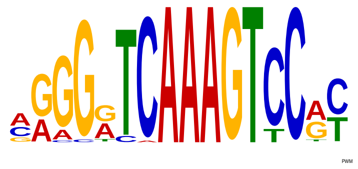	*NGGGATCNNNNTCCGN*	52.58	M	10
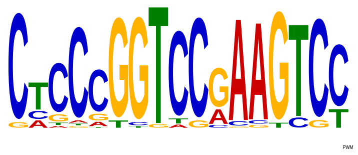	*NNNCCGGTNNGAAGNNN*	18.09	M	11
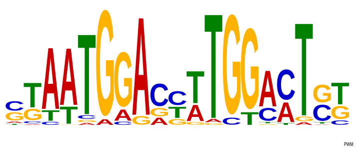	*NNNATGGNNNNTGGAANNN*	11.37	M	12
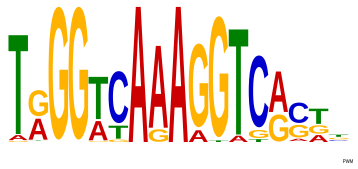	*NAGGTNNNNNGTCACAN*	22.50	DR:1	13
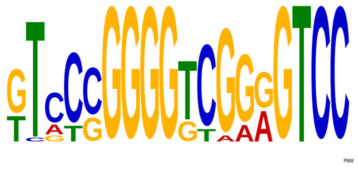	*NTACCGGGNNNNNAGTCN*	77.17	ER:6	14
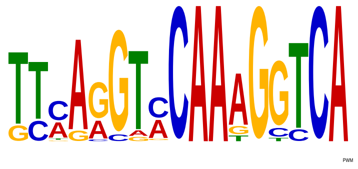	*NNCAGGTANNNAGGTNN*	22.06	DR:2	15
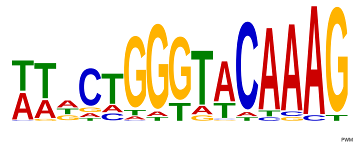	*NNACTGGNNACAANN*	14.28	M	16
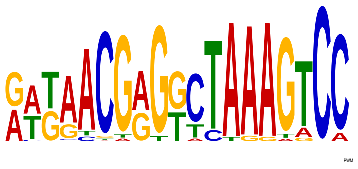	*NNNAACGNNNNTAAAGNNN*	22.00	M	17
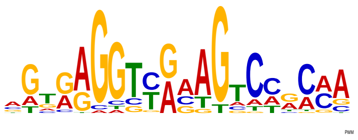	*NNNNNGGTTNNAGTCNNNNN*	5.59	DR:0	18

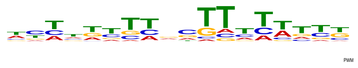	$NNNNTTTTNNTTTTNNNN$	5.23	M	19
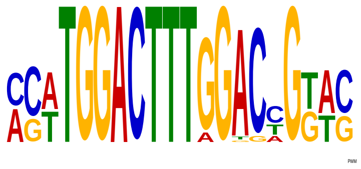	$NNNTGGANNNN\overrightarrow{NGGACA}GNNN$	51.90	M	20

342 HNF4A-Jolma2013-Rnd5 (ERR194358)

PWM	Seed Sequence	Seed Seq Enrichment	Repeat	Rank
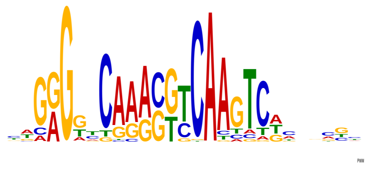	NNNNNNTCAANNNNCAAGTCGNNNNNN	181.82	DR:6	1
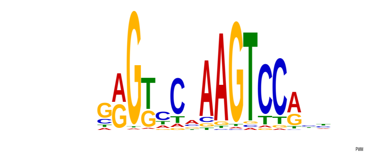	NNNNNNGAGTNNNNAGTCNNNNNN	21.72	DR:2	2
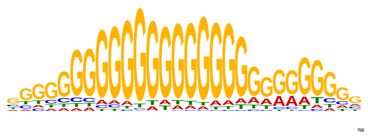	NNNNNNGGGGGGGGGGGGGGGGGGNNNNNN	3395.07	M	3
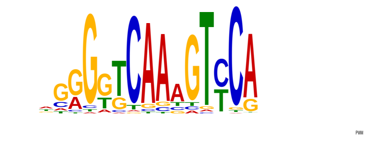	NNNNNNGTCANNNNCCATNNNNNN	18.27	M	4
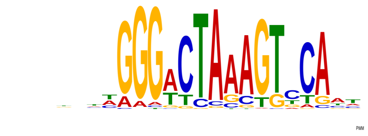	NNNNNNAGGGNCTAAAGNNNNNN	82.84	M	5
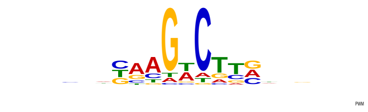	NNNNNNTAAGNCTTANNNNNN	21.12	M	6
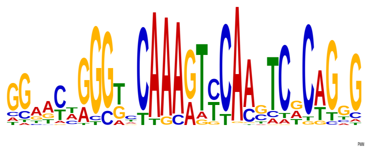	NNNNNCGGGNNNNNNNNNCAAGTCGNNNNNN	75.25	DR:7	7
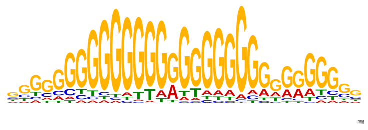	NNNNNNGGGGGGGGNNNGGGGGGGGNNNNNN	3921.98	DR:5	8
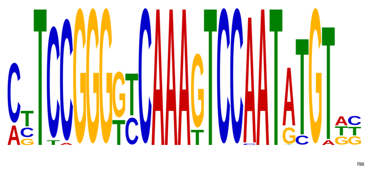	NNTCCGGGNNNNNNNNNCAATATGTNN	749.61	DR:10	9

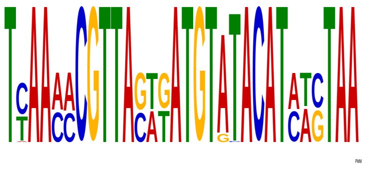	NNNNNNCGTTANNNNNGTATACATNNNNNN	468.30	M	10
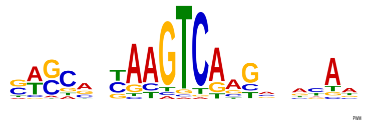	NNNNNNTAAGTCAAGNNNNNN	26.40	M	11
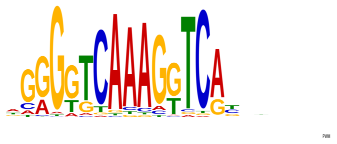	NNNNNNGTCANNNGTCATNNNNNN	21.77	DR:1	12
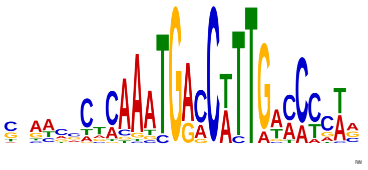	NNNNNNCTCAAATGNNNNTTGANNNNNN	170.03	IR:9	13
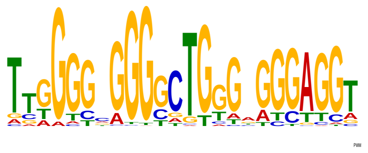	NNNNNNCGGGNCTGGGAGNNNNNN	90.11	DR:0	14
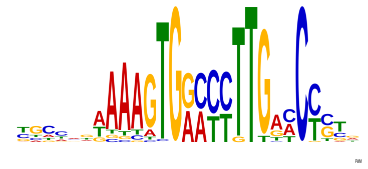	NNNNNNGAAAAGTGNNNNTTGANNNNNN	282.10	IR:4	15
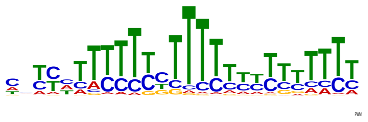	NNNNNNTTTTNNTTTTTTTTNNNNNN	132.48	M	16
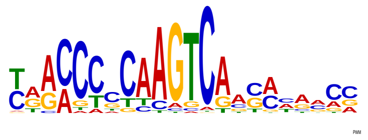	NNNNNNCCAAGTCAACNNNNNN	43.48	M	17
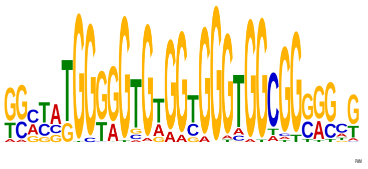	NNNNNNGGGGGTGNNNNGGGTGGCGNNNNNN	2030.61	DR:3	18

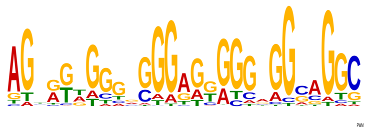	NNNNNN$\overrightarrow{GGGGGG}$GANN$\overrightarrow{GGGAG}$NNNNNN	319.60	DR:4	19
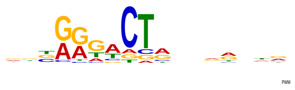	NNNNNNT$\overrightarrow{AGGNCT}$GGNNNNNN	10.69	M	20

343 HNF4A-Jolma2013-Rnd6 (ERR194359)

PWM	Seed Sequence	Seed Seq Enrichment	Repeat	Rank
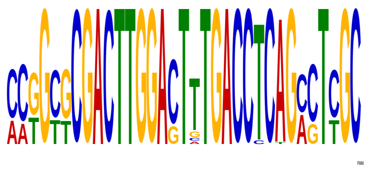	NNNNNNCGACTTGGNNNNNNNNNCTCAGNNNNNN	3208.85	ER:8	1
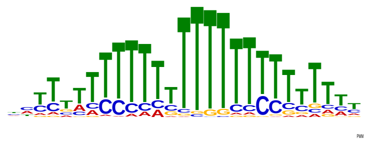	NNNNNNTTTTTNNTTTTTTTTNNNNNN	1211.39	M	2
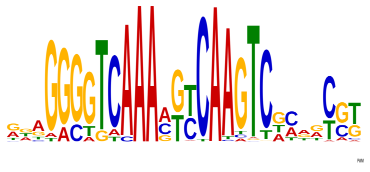	NNNNNNGTCANNNNTCAAGTCGNNNNNN	525.97	DR:6	3
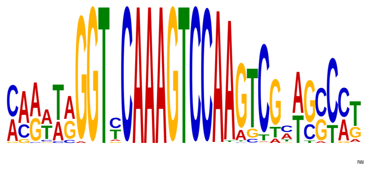	NNNNNNGGTCCNNNNNNCAAGTCGANNNNNN	652.36	DR:7	4
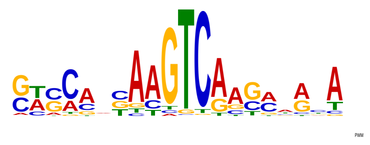	NNNNNNTAAGTCAAGNNNNNN	40.71	M	5
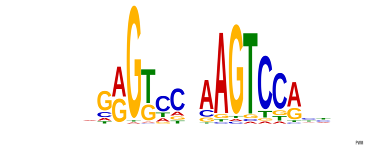	NNNNNNGAGTNNNNAGTCNNNNNN	24.00	DR:2	6
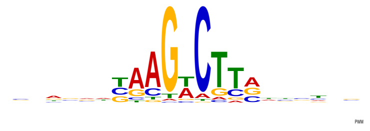	NNNNNNTAAGNCTTANNNNNN	32.34	M	7
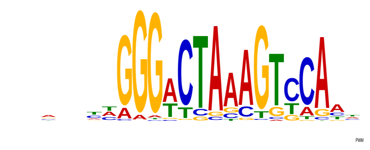	NNNNNNAGGGNCTAAAGNNNNNN	103.77	M	8
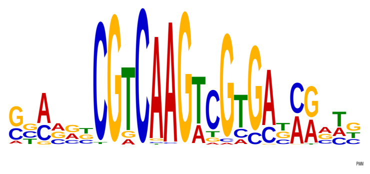	NNNNNNCGTCAAGNNGTGANNNNNN	240.60	DR:2	9

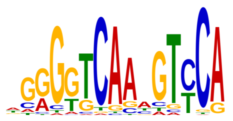	NNNNNNGTCANNNNNCCATNNNNNN	21.01	M	10
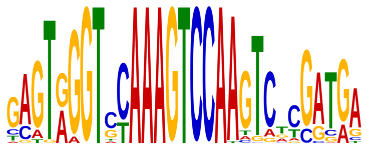	NNNNNGGTCCNNNNNNNCAAGTCTTNNNNN	446.02	DR:7	11
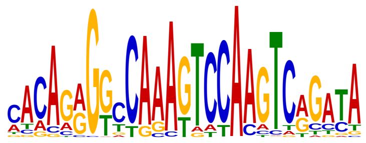	NNNNGAGTNNNNNNNNNCAAGTCGNNNN	146.34	DR:7	12
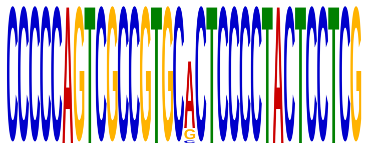	NNNNCAGTCGCNNNNNNNNNNNCTACTCNNNN	436.59	IR:12	13
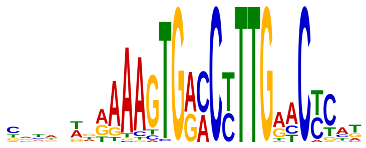	NNNNNNTAAAAGTGNNNNTTGANNNNNN	335.43	IR:4	14
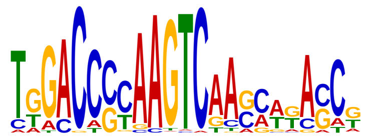	NNNNNNCCAAGTCAACCNNNNNN	108.19	M	15
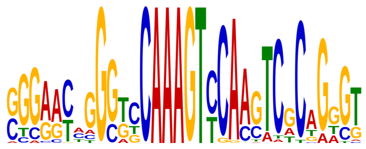	NNNNNNCGGGNNNNNNNNNCAAGTCGNNNNNN	145.33	DR:7	16
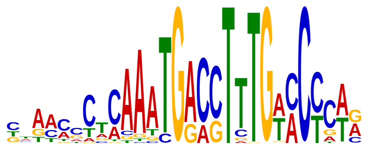	NNNNNNCTCAAATGNNNNTTGANNNNNN	239.17	IR:9	17
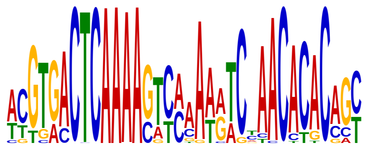	NNNNNNCTCAAAANNNNNNNNNNNNAACANNNNNN	192.06	M	18

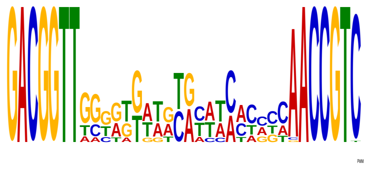	$GAC\overrightarrow{GGTNNN}NNNNNNNNNNNNNNNNNNAAC\overrightarrow{CGTC}$	344.97	DR:21	19
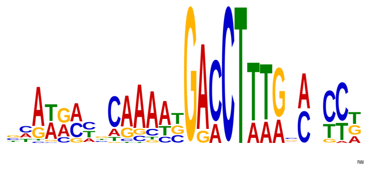	$NNNN\overrightarrow{NNCTC}AAATTNNNNTTTGNNNNNN$	73.09	M	20

344 HNF4A-Yin2017-Rnd2 (ERR1003748)

PWM	Seed Sequence	Seed Seq Enrichment	Repeat	Rank
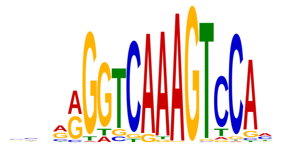	NNNNNNGGTCAAAGTCCANNNNNNN	129.37	DR:1	1
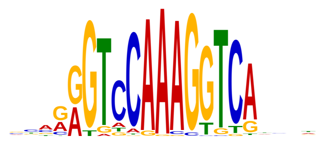	NNNNNNGTCCAAAGGTCANNNNNNN	125.80	DR:1	2
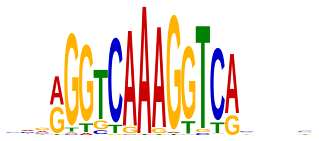	NNNNNNGGTCAAAGGTCANNNNNNN	113.71	DR:1	3
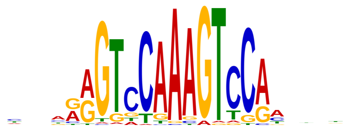	NNNNNNGTCCAAAGTCCANNNNNNN	93.60	DR:1	4
	NNNNNNGGGGNNNNNGGGGNNNNNN	2.58	DR:3	5
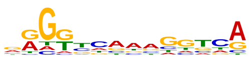	NNNNNNCAAAGGTCNNNNNN	2.96	M	6
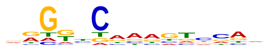	NNNNNNAAAGTCCANNNNNN	2.46	M	7
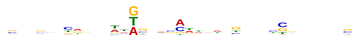	NNACGTNNNNNNNNNNNNNNNNNNNNNNNNNNTCGTNN	1.34	M	8
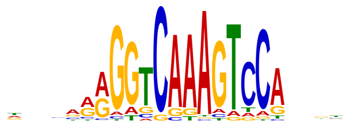	NNNNNNAGGTCNAAGTCCNNNNNN	29.01	DR:1	9

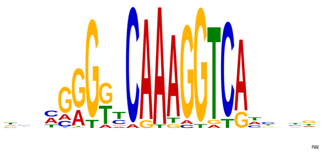	NNNNNNCGGGNNCAAAGGTCNNNNNN	44.68	DR:1	10
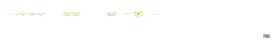	NNNNNGGGGNNNNNNNNNNNNNNNNNNNNNNGGGGNNNNN	2.10	DR:20	11
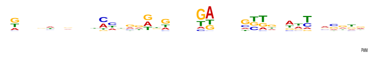	NGCGTNNNNNNNNNNNNNNNNNNNNNNNNNNNNNNACGTN	1.97	DR:28	12
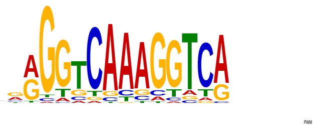	NNNNNNGTCAAAGGTCNNNNNN	15.04	DR:1	13
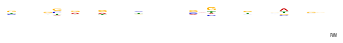	CAGGNNNNNNNNNNNNNNNNNNNNNNNNNNNNNNNGGGC	1.68	DR:27	14
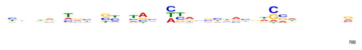	NCACTNNNNNNNNNNNNNNNNNNNNNNNNNNNNGTATN	1.15	M	15
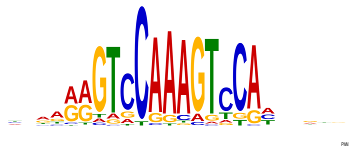	NNNNNNAGTCNNAAGTCCANNNNNN	21.89	DR:1	16
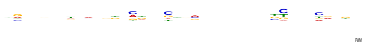	TGTGNNNNNNNNNNNNNNNNNNNNNNNNNNNNNNNNGTGG	1.66	M	17
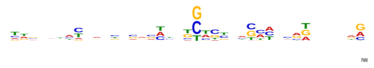	NNGCATNNNNNNNNNNNNNNNNNNNNNNNNNNNCTCTNN	1.21	M	18

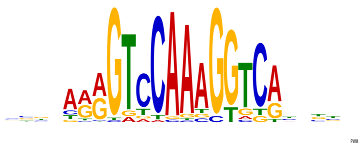	*NNNNNN$\overrightarrow{AGTCC}$NA$\overrightarrow{AGGTC}$NNNNNN*	25.67	DR:1	19
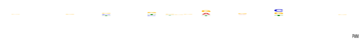	*GGCGNNNNNNNNNNNNNNNNNNNNNNNNNNNNNNNGCGG*	1.70	M	20

345 HNF4A-Yin2017-Rnd2 (ERR1003749)

PWM	Seed Sequence	Seed Seq Enrichment	Repeat	Rank
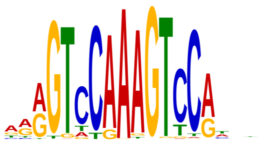	NNNNNNAGTCCAAAGTCCANNNNNN	3142.56	DR:1	1
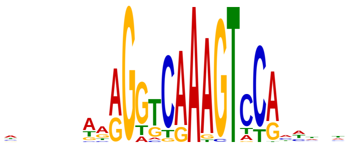	NNNNNNAAAGGTNAAAGTCCANNNNNN	4747.70	DR:1	2
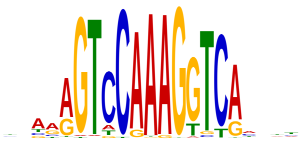	NNNNNNAGTCCANAGGTCANNNNNN	1084.13	DR:1	3
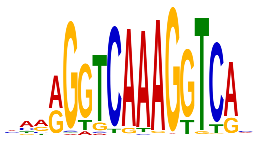	NNNNNNGGTCANAGGTCANNNNNN	362.02	DR:1	4
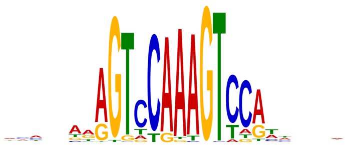	NNNNNNAAAGTCNNAAGTCCANNNNNN	1628.05	DR:2	5
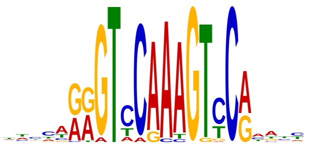	NNNNNNGGGTCCNAAGTCCACNNNNNN	1676.22	DR:1	6
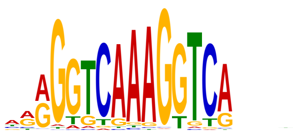	NNNNNNNGTCAANNGTCANNNNNN	71.52	DR:1	7
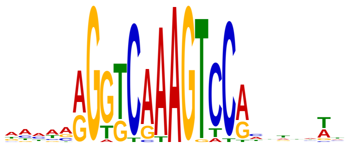	NNNNNNGGTCAAAGTCCACGNNNNNN	1421.61	DR:1	8
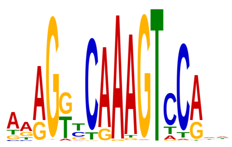	NNNNNNAAAGNNNAAAGTCCANNNNNN	1043.24	M	9

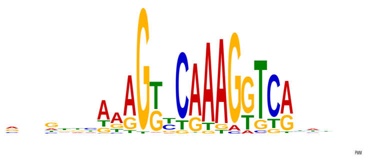	$NNNNNNCAA\overrightarrow{AGNNCA}A\overrightarrow{AGGTCN}NNNNN$	1243.89	DR:1	10
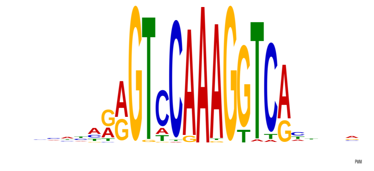	$NNNNNNAG\overrightarrow{GGTCCA}N\overrightarrow{AGGTCN}NNNNN$	766.23	DR:1	11
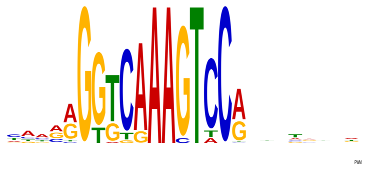	$NNNNN\overrightarrow{NNGTCA}A\overrightarrow{AGTCCA}TTNNNNNN$	692.40	DR:1	12
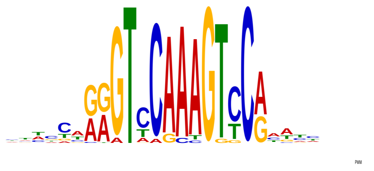	$NNNNNNG\overrightarrow{GGTCCA}N\overrightarrow{AGTCCA}CNNNNNN$	1648.56	DR:1	13
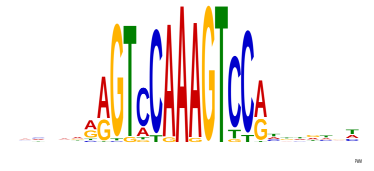	$NNNNNN\overrightarrow{AAGTCN}NA\overrightarrow{AGTCCA}CNNNNNN$	809.91	DR:2	14
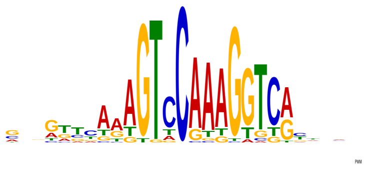	$NNNNNNCAA\overrightarrow{AGTCCN}N\overrightarrow{AGGTCN}NNNNN$	819.26	DR:1	15
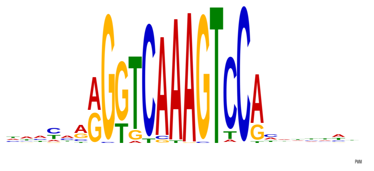	$NNNNNN\overrightarrow{GGGTCA}N\overrightarrow{AGTCCA}CNNNNNN$	915.75	DR:1	16
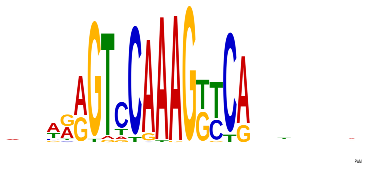	$NNNNN\overrightarrow{NGTCCA}A\overrightarrow{AGNNCA}ATNNNNNN$	394.59	DR:1	17
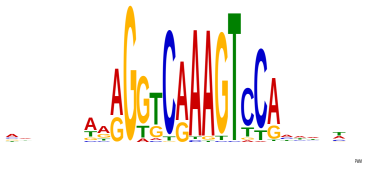	$NNNNNNAA\overrightarrow{AGGTCN}N\overrightarrow{AGTCCA}NNNNNN$	1449.74	DR:1	18

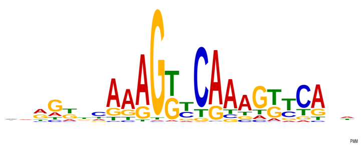	*NNNNNNCAAAGNNCAAAGNNNNNN*	87.83	M	19
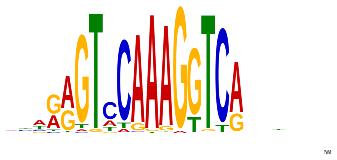	*NNNNNNGGTCCAANNGTCANNNNNN*	197.18	DR:1	20

346 HNF4A-Yin2017-Rnd3 (ERR1003750)

PWM	Seed Sequence	Seed Seq Enrichment	Repeat	Rank
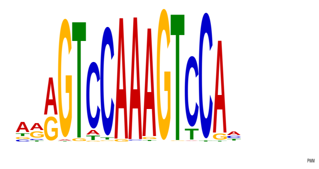	NNNNNNAGTCCAAAGTCCANNNNNN	1478.69	DR:1	1
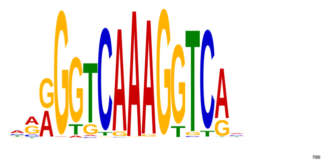	NNNNNNGGTCAAAGGTCANNNNNN	512.13	DR:1	2
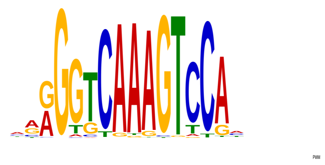	NNNNNNGGTCAAAGTCCANNNNNN	498.43	DR:1	3
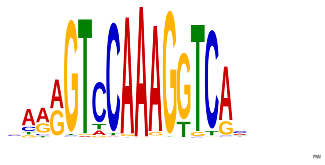	NNNNNNAGTCCAAAGGTCANNNNNN	1012.89	DR:1	4
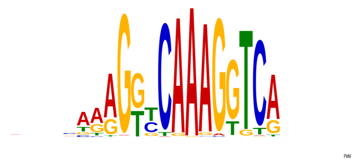	NNNNNNAAAGNNCAAAGGTCNNNNNN	367.76	DR:1	5
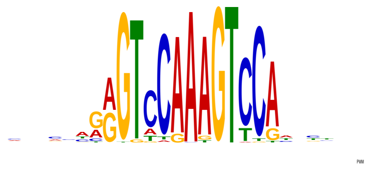	NNNNNNGGGTCCNAAGTCCANNNNNN	853.01	DR:1	6
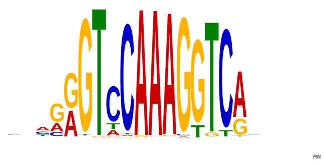	NNNNNNGGGTCCNAAGGTCNNNNNN	303.34	DR:1	7
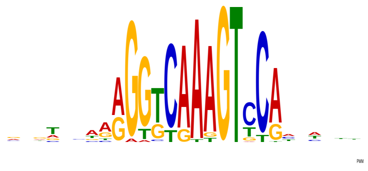	NNNNNNAAAGGTCNAAGTCCANNNNNN	1291.55	DR:1	8
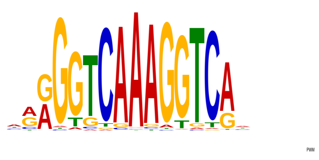	NNNNNNGGTCANAGGTCNNNNNN	87.32	DR:1	9

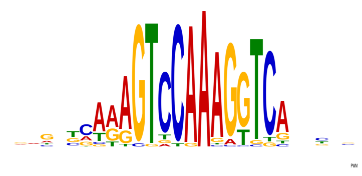	*NNNNNNCAAAGTCCNAAGGTCNNNNNN*	1318.42	DR:1	10
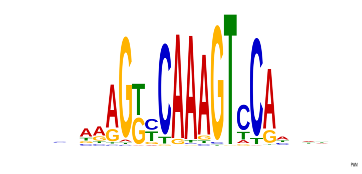	*NNNNNNAAAGNNNAAAGTCCANNNNNN*	277.09	M	11
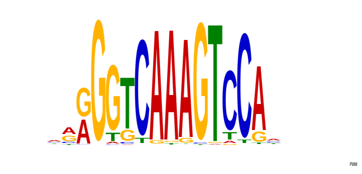	*NNNNNNGGTCAAANTCCANNNNNN*	212.49	DR:1	12
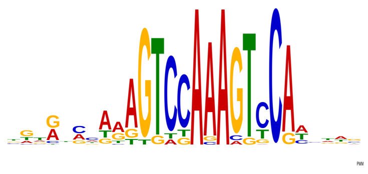	*NNNNNNCAAAGTCNAAAGTCCNNNNNN*	976.40	DR:2	13
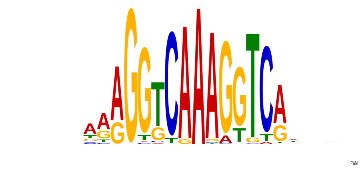	*NNNNNNAAAGGTCNAAGGTCNNNNNN*	631.37	DR:1	14
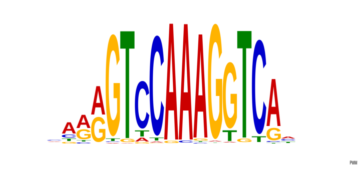	*NNNNNNAGTCCNAAGGTCNNNNNN*	155.13	DR:1	15
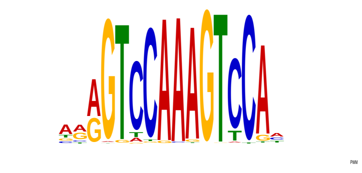	*NNNNNNAGTCCNAAGTCCANNNNNN*	583.43	DR:1	16
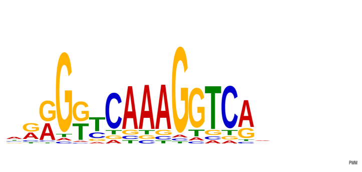	*NNNNNNCAAANGTCANNNNNN*	12.26	M	17
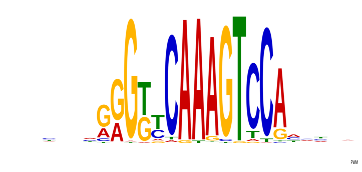	*NNNNNNCGGGNNCAAAGTCCNNNNNN*	145.57	DR:1	18

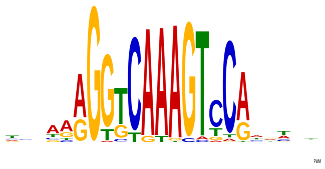	$NNNNNNAA\overrightarrow{AGGTCA}N\overrightarrow{AGTCCN}NNNNN$	466.68	DR:1	19
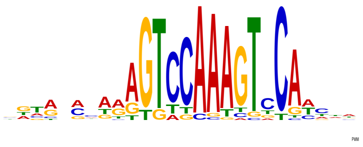	$NNNNNNCAA\overrightarrow{AGTCCN}\overrightarrow{AAGTCN}NNNNN$	267.62	DR:0	20

347 HNF4A-Yin2017-Rnd3 (ERR1003751)

PWM	Seed Sequence	Seed Seq Enrichment	Repeat	Rank
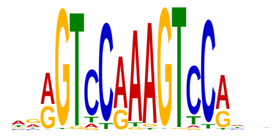	NNNNNNAGTCCNNAGTCCNNNNNN	382.83	DR:1	1
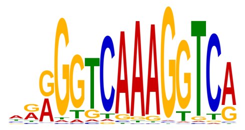	NNNNNNGTCANNNGTCANNNNNN	87.83	DR:1	2
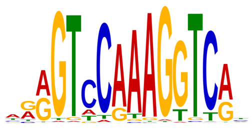	NNNNNNGTCCNNAGGTCNNNNNN	159.84	DR:1	3
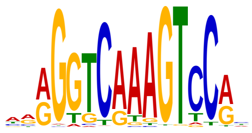	NNNNNGGTCNNAGTCCNNNNNN	147.85	DR:0	4
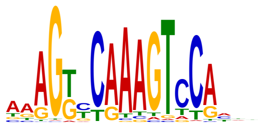	NNNNNNAAAGNNNNAAGTCNNNNNN	121.18	M	5
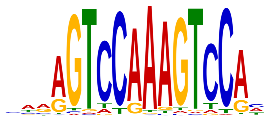	NNNNNAGTCNNNAGTCNNNNNN	68.93	DR:1	6
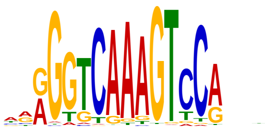	NNNNNNGGTCANNNTCCANNNNNNN	139.93	DR:1	7
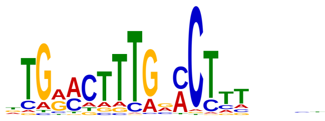	NNNNNNCTTTNNNCTTTNNNNNN	65.28	M	8
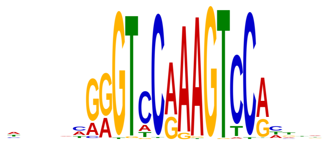	NNNNNNACGGGTCCNNAGTCCNNNNN	1160.67	DR:1	9

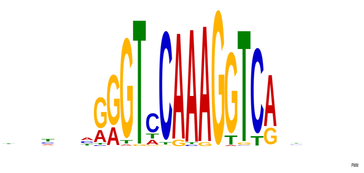	*NNNNNNCGGGTCCANNNGTCANNNNNN*	597.34	DR:1	10
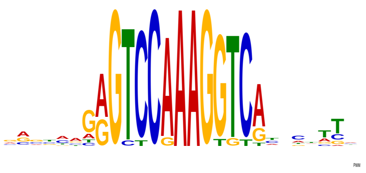	*NNNNNAGAGTCCNNNGGTCACTNNNNN*	1198.81	DR:1	11
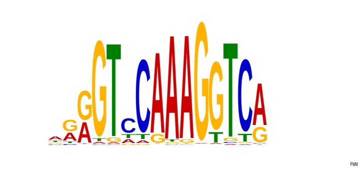	*NNNNNNGTCCANNNGTCANNNNNN*	127.79	DR:1	12
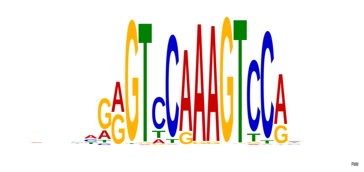	*NNNNNNAGGGTCCNNNGTCCNNNNNN*	325.85	DR:1	13
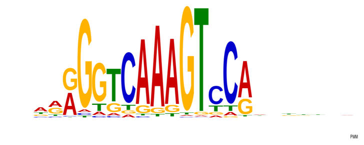	*NNNNNNGTCANNNTCCAANNNNNNN*	64.49	DR:1	14
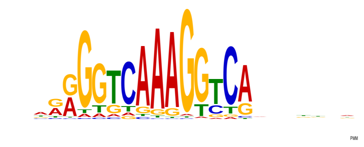	*NNNNNNGTCANNNNTCAANNNNNNN*	43.60	DR:1	15
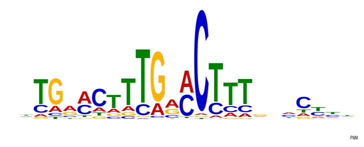	*NNNNNNCTTTNNNNTTTGNNNNNN*	40.69	M	16
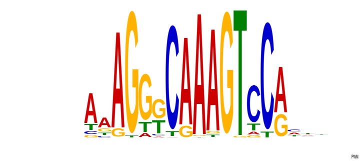	*NNNNNNAAAGGGCNNAGTCCNNNNNN*	544.03	DR:1	17
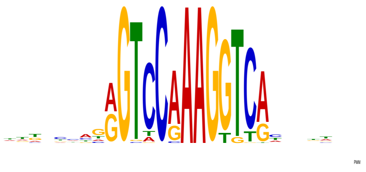	*NNNNNNAGAGTCCNNNGGTCACNNNNNN*	908.69	DR:1	18

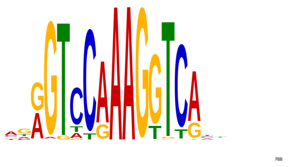	*NNNNNNAG*$\overrightarrow{AGTCC}$*NNN*$\overrightarrow{NGTCA}$*NNNNNNN*	232.29	DR:1	19
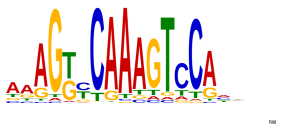	*NNNNNNAAAGNNNN*$\overrightarrow{NAGTC}$*NNNNNNN*	39.16	M	20

348 HNF4A-Yin2017-Rnd4 (ERR1003752)

PWM	Seed Sequence	Seed Seq Enrichment	Repeat	Rank
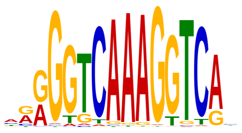	*NNNNNNGGTCANNGGTCANNNNNN*	123.00	DR:1	1
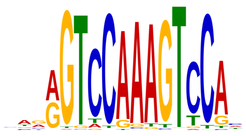	*NNNNNNAGTCCNNAGTCCANNNNNN*	236.46	DR:1	2
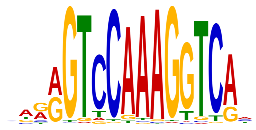	*NNNNNNAGTCCNNAGGTCNNNNNN*	103.99	DR:1	3
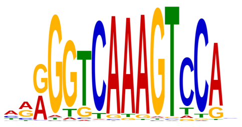	*NNNNNNGGTCANNGTCCANNNNNN*	98.43	DR:1	4
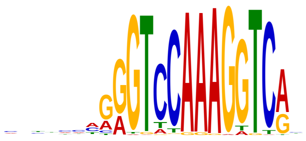	*NNNNNNCGGGTCCNNAGGTCNNNNNN*	299.56	DR:1	5
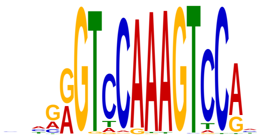	*NNNNNNGGGTCCNNAGTCCNNNNNN*	209.86	DR:1	6
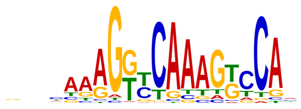	*NNNNNNAAAGNNNAAAGTNNNNNN*	27.03	M	7
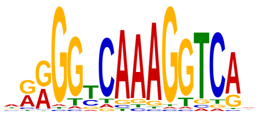	*NNNNNNTCAANNGTCANNNNNN*	22.28	DR:1	8
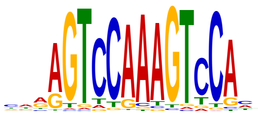	*NNNNNNAAGTCNNAAGTCNNNNNN*	62.06	DR:1	9

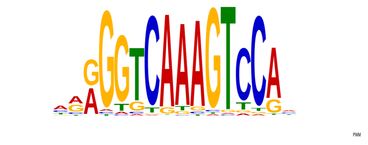	NNNNNNGGTCNNAGTCCNNNNNN	47.60	DR:0	10
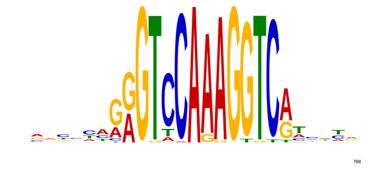	NNNNNNTAGGGTCCNNAGGTCNNNNNN	462.58	DR:1	11
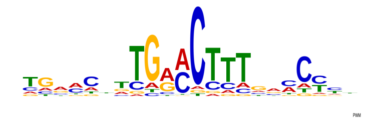	NNNNNNTTTGNNNTTTGNNNNNN	11.60	M	12
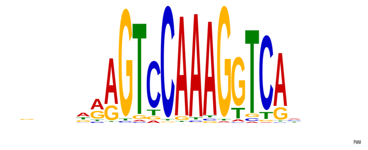	NNNNNNAAGTCNNNAGGTCNNNNNN	51.57	DR:2	13
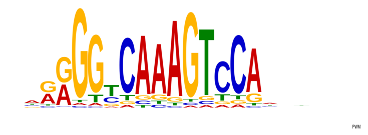	NNNNNNTCAANNTCCANNNNNN	16.00	DR:1	14
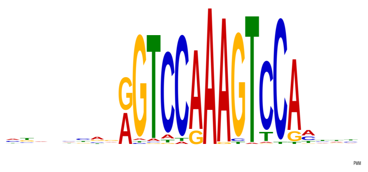	NNNNNNAGAGTCCNNAGTCNNNNNN	93.27	DR:0	15
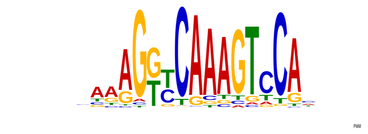	NNNNNNAAAGNNNNAAGTCNNNNNN	25.04	M	16
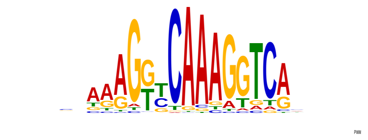	NNNNNNAAAGNNNNNAGGTCNNNNNN	23.30	M	17
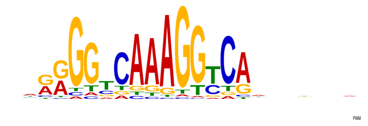	NNNNNNTCAANNNTCAANNNNNN	12.52	DR:1	18

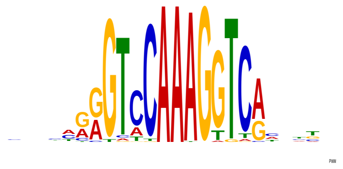	$NNNNNNTG\overrightarrow{GGTCCN}N\overrightarrow{AGGTCN}NNNNN$	183.68	DR:1	19
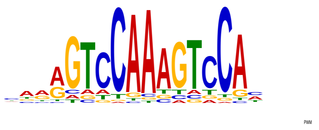	$NNNNN\overrightarrow{NAGTCN}N\overrightarrow{NAGTCN}NNNNN$	11.42	DR:1	20

349 HNF4A-Yin2017-Rnd4 (ERR1003753)

PWM	Seed Sequence	Seed Seq Enrichment	Repeat	Rank
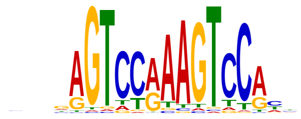	NNNNNNAGTCNNNAGTCNNNNNN	55.73	DR:1	1
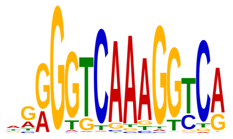	NNNNNNGTCANNNNTCATNNNNNN	48.47	DR:1	2
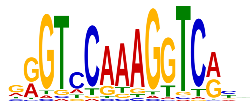	NNNNNNGTCCNNNGGTCNNNNNN	31.32	DR:2	3
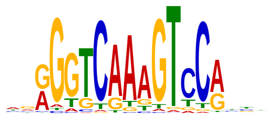	NNNNNNGGTCNNNNTCCANNNNNN	39.57	DR:1	4
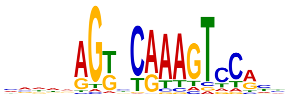	NNNNNNAAAGNNNNNAGTCNNNNNN	26.36	M	5
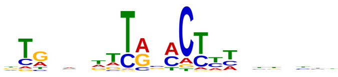	NNNNNNCTTTNNNNTTTGNNNNNN	20.29	M	6
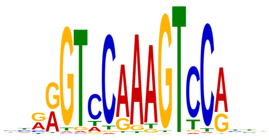	NNNNNNGGTCCNNNNTCCANNNNNN	73.33	DR:1	7
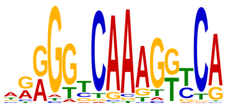	NNNNNNNTCAANNNNCAATNNNNNN	33.81	M	8
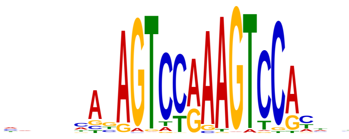	NNNNNNATAGTCNNNAGTCNNNNNN	109.49	DR:1	9

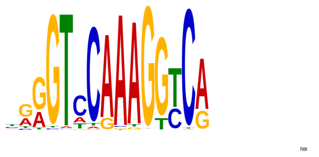	NNNNNNGGTCCNNNNNTCATNNNNNN	48.10	DR:1	10
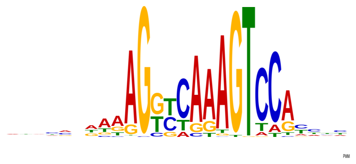	NNNNNNTAAAAGNNNNNAGTCNNNNNN	58.13	M	11
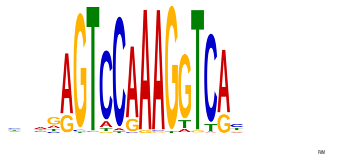	NNNNNNGAGTCNNNNNGTCACNNNNNN	81.71	DR:2	12
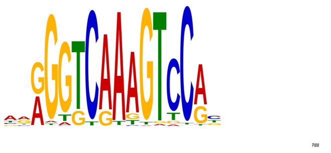	NNNNNNGGTCNNNNTCCACANNNNNN	64.66	DR:0	13
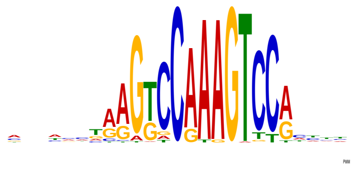	NNNNNNCTAAGTNNNNAGTCNNNNNN	69.75	DR:1	14
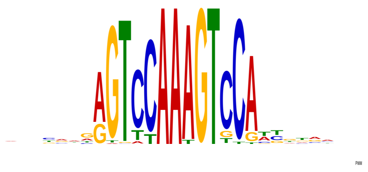	NNNNNNGAGTCNNNNNTCCAACNNNNNN	135.47	DR:2	15
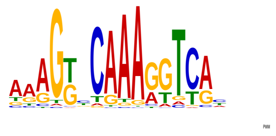	NNNNNNTAAANNNNNNAGGTNNNNNN	13.71	M	16
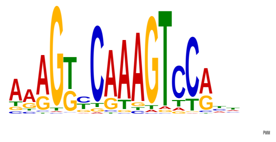	NNNNNNTAAANNNNNNAGTCNNNNNN	21.58	M	17
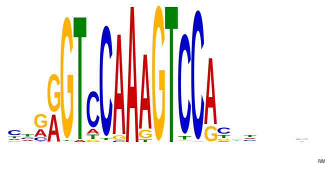	NNNNNNGGTCCNNNNTCCACANNNNNN	127.30	DR:1	18

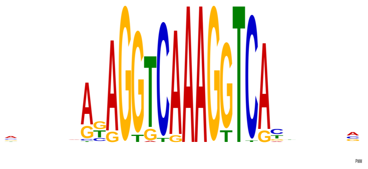	$NNNNNNAT\overrightarrow{AGGTCN}N\overrightarrow{NNGTCA}CNNNNNN$	267.65	DR:1	19
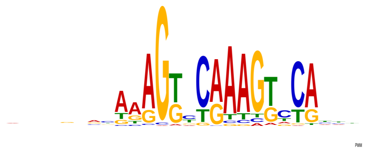	$NNNNNNACAAANNNNN\overrightarrow{AAGTN}NNNNN$	34.29	M	20

350 HNF4A-18-Bhimsaria2022-Rnd2 (S07-008)

PWM	Seed Sequence	Seed Seq Enrichment	Repeat	Rank
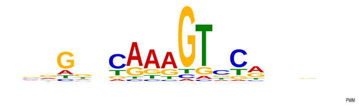	NNNNNNCAAAGTCCNNNNNN	19.05	M	1
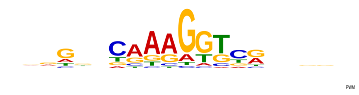	NNNNNNCAAAGGTCNNNNNN	20.21	M	2
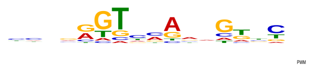	NNNNNNGGGTCCAANNNNNN	6.52	M	3
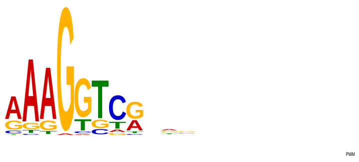	AAAGGTCNNNNNNNNNGGGG	48.39	M	4
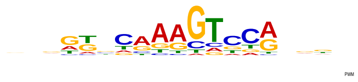	NNNNNCTAAAGTCCNNNNN	7.24	M	5
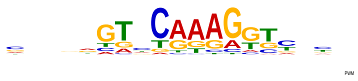	NNNNAGTCCNAAGGTNNNN	21.86	DR:1	6
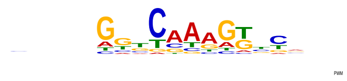	NNNNNNGGTCAAAGNNNNNN	12.04	M	7
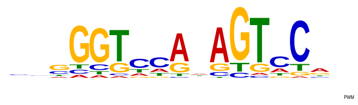	NNGGGTCCANAGTCCN	140.28	DR:1	8
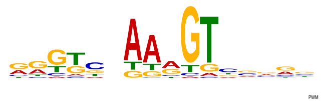	NNNGGTCNAAGGTCCNNN	54.44	DR:1	9

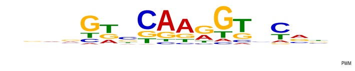	NNNAGTCCAANGTCCNNN	49.05	DR:1	10
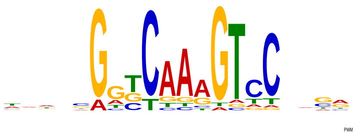	NNNNCGGTCAANGTCCNNNN	77.32	DR:1	11
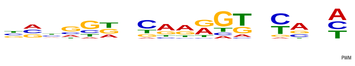	NNNAGTCCANAGTCCNNN	32.96	DR:1	12
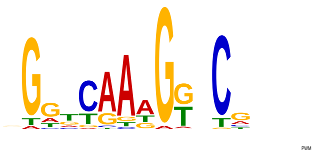	NNNGGTCAAAGTGCGNNN	90.71	DR:1	13
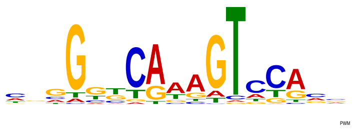	NNNGGTCATAGTCCNNN	37.88	DR:1	14
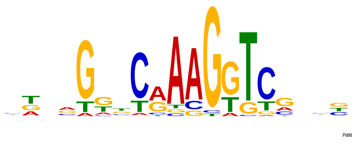	NNNNGGGCGAAGGTCNNNN	40.14	DR:0	15
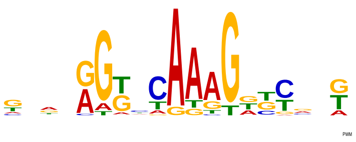	NNNGGGTNCAAAGGGCNNN	59.12	DR:1	16
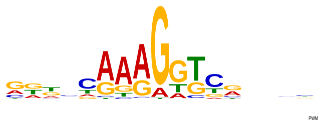	NNNNTCCAAAGGTCGNNNN	60.88	DR:1	17
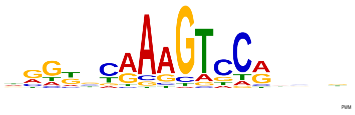	NNNNCTAAAGTCCGNNNN	12.10	M	18

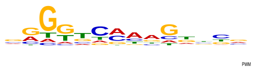	$NNNNNNG\overrightarrow{GGGTCA}ANNNNNN$	5.84	M	19
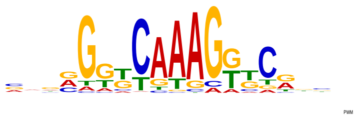	$NNNN\overrightarrow{GGGCA}A\overrightarrow{AGGGC}NNNN$	40.63	DR:1	20

351 HNF4A-18-Bhimsaria2022-Rnd3 (S07-129)

PWM	Seed Sequence	Seed Seq Enrichment	Repeat	Rank
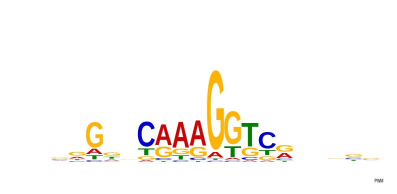	NNNNNNCAAAGGTCNNNNNN	109.55	M	1
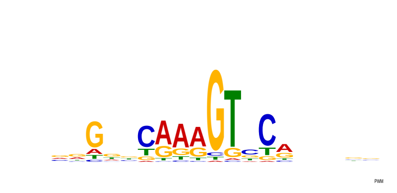	NNNNNNCAAAGTCCNNNNNN	84.81	M	2
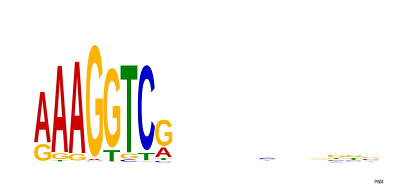	AAAGGTCGNNNNNNNNGGGG	775.27	M	3
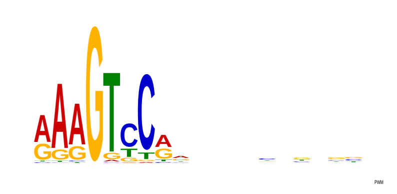	AAAGTCCNNNNNNNNNGGGG	317.77	M	4
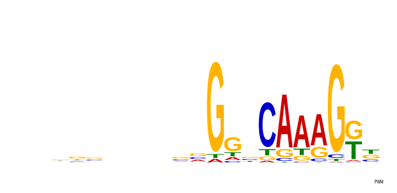	GGGGNNNNNNNNNCAAAGGG	245.41	DR:9	5
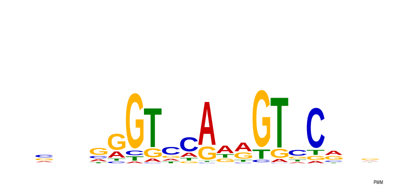	NNNGGGTCCANNGTCCNNN	479.27	DR:1	6
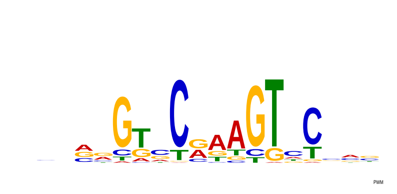	NNNAGTCCGAAGTCCNN	563.54	DR:1	7
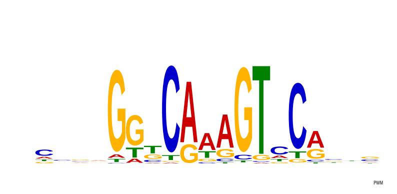	NNNNGGTCATAGTCCNNNN	218.88	DR:1	8
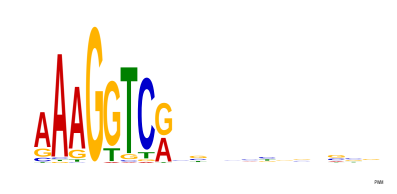	AAAGGTCNNNNNNNNTGCG	230.90	M	9

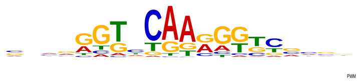	$NNNN\overrightarrow{AGTCCA}N\overrightarrow{NGGTCN}NNN$	92.56	DR:1	10
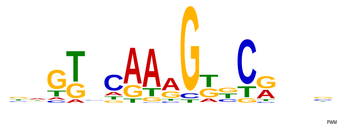	$NNN\overrightarrow{GTCCAA}A\overrightarrow{GGGCAN}NN$	327.41	DR:1	11
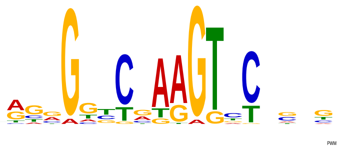	$NNN\overrightarrow{GGGTCG}A\overrightarrow{AGTCCG}NNN$	1114.63	DR:1	12
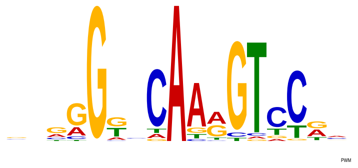	$NNG\overrightarrow{GGTGCA}A\overrightarrow{GGTCCN}N$	671.11	DR:1	13

352 HNF4G-Bhimsaria2022-Rnd2 (S07-009)

PWM	Seed Sequence	Seed Seq Enrichment	Repeat	Rank
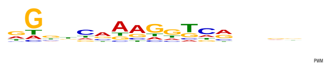	NNNNNNCAAAGGTCNNNNNN	3.78	M	1
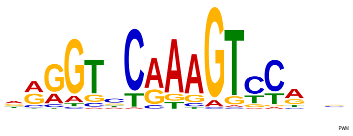	NNNGTCCAAAGTCCNNN	29.56	DR:1	2
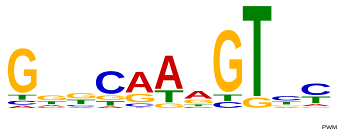	GGTCAAAGTCC	17.70	DR:1	3
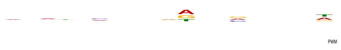	NAGATNNNNNNNNNNTTATN	1.74	M	4
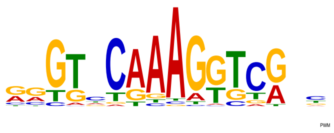	NNNGTCCAAAGGTCNNN	26.44	DR:1	5
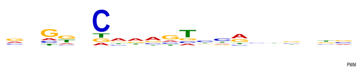	NNNNNNAAAGTCCANNNNNN	3.18	M	6
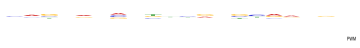	CAGTNNNNNNNNNNNNAGGG	1.77	M	7
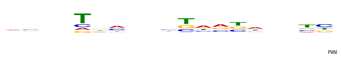	NACTTNNNNNNNNNNAGTTN	1.88	M	8
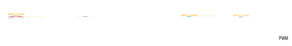	GGGGNNNNNNNNNNNNCGCG	1.60	M	9

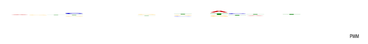	AGTCNNNNNNNNNNNTGCT	1.73	M	10
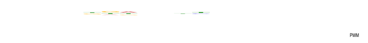	CAGGNNNNNNNNNNNCATG	1.57	M	11
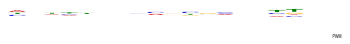	NNATTTNNNNNNNNGTTTNN	2.27	M	12
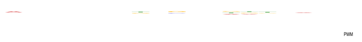	AAGGNNNNNNNNNNNAATT	1.68	M	13
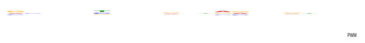	GCGTNNNNNNNNNNNNGTGG	1.61	M	14
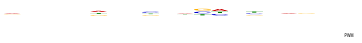	ACGANNNNNNNNNNNNAGGG	1.58	M	15
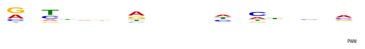	NATTANNNNNNNNNNTACTN	1.98	M	16
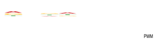	CAATNNNNNNNNNNNACGT	1.57	M	17
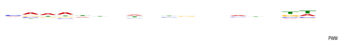	NCAAANNNNNNNNNNTGTTN	2.08	M	18

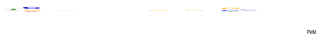	$\overrightarrow{AGTC}NNNNNNNNNNNCCGG$	1.55	M	19
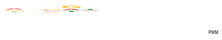	$TTCTNNNNNNNNNNATAT$	1.62	M	20

353 HNF4G-Bhimsaria2022-Rnd3 (S07-130)

PWM	Seed Sequence	Seed Seq Enrichment	Repeat	Rank
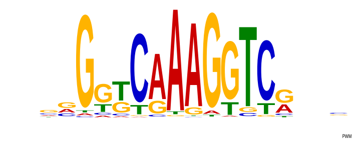	NNNNGGTCAAAGGTCNNNN	315.63	DR:1	1
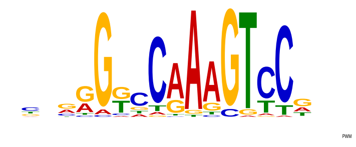	NNNGGGTNCAAAGTCCNNN	504.26	DR:1	2
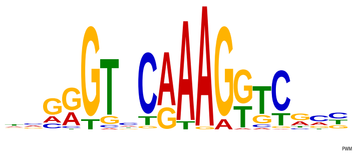	NNGGGTCCAAAGGTCGNN	1741.88	DR:1	3
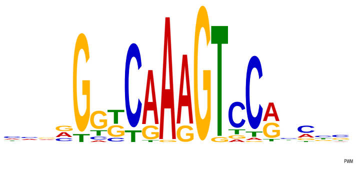	NNNNGGTCAAAGTCCANNNN	742.59	DR:1	4
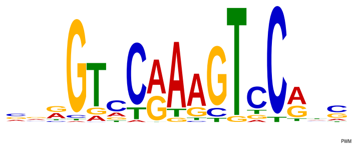	NNAGTCCAAAGTCCANN	1004.73	DR:1	5
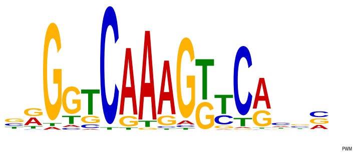	NNGGTCAAAGNNCACGNN	214.84	DR:1	6
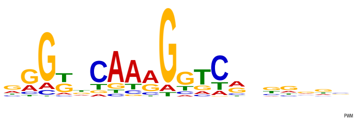	NNNNNCAAANGTCACNNNNN	12.69	M	7
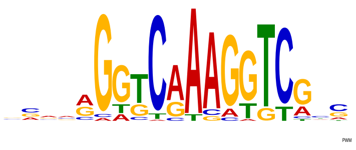	NNNAAGGTCNAAGGTCNNN	207.66	DR:1	8
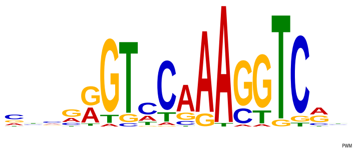	NNNAAGTCCAAAGGTNNN	127.48	DR:1	9

354 HNF4G-Bhimsaria2022-Rnd3 (S07-162)

PWM	Seed Sequence	Seed Seq Enrichment	Repeat	Rank
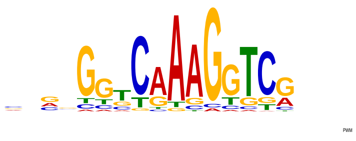	NNNNGGTCAAAGGTCNNNN	105.15	DR:1	1
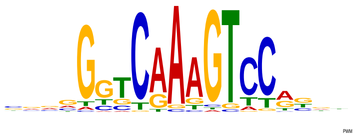	NNNNGGTCAAAGTCCNNNN	115.46	DR:1	2
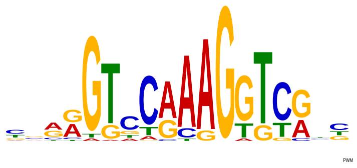	NNNGGTCCAAAGGTCNNN	152.87	DR:1	3
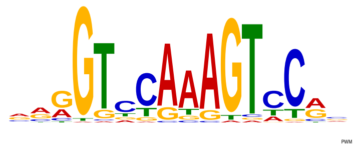	NNGGTCCAAAGTCCNN	176.77	DR:1	4
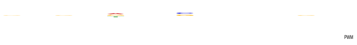	GGGGNNNNNNNNNNNNCGTG	5.74	M	5
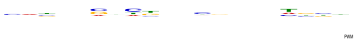	CATGNNNNNNNNNNNNTGCT	3.97	M	6
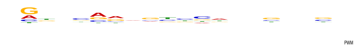	NNNNNNAAGTCCAANNNNNN	4.54	M	7
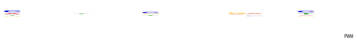	CACANNNNNNNNNNNNGCTT	3.70	M	8
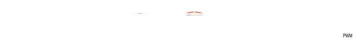	CAGCNNNNNNNNNNNCCGT	3.65	M	9

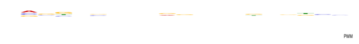	*CAGGNNNNNNNNNNNNNGTCC*	5.11	DR:7	10
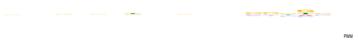	*GTCGNNNNNNNNNNNNGGGA*	4.03	M	11
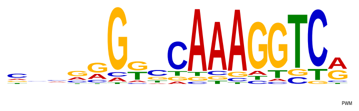	*NNCGGGNNCAAAGGTNN*	28.78	DR:1	12
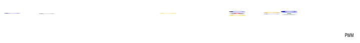	*CACANNNNNNNNNNNNCATT*	3.63	M	13
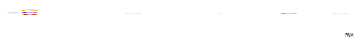	*CGCGNNNNNNNNNNNNCATG*	3.89	M	14
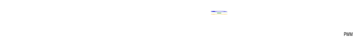	*CACGNNNNNNNNNNNNCTTG*	4.01	M	15
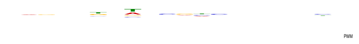	*CAGGNNNNNNNNNNNNTCCG*	3.62	M	16
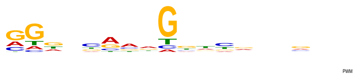	*NNNNCAAANGTCACNNNN*	5.27	M	17
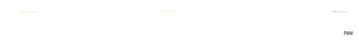	*AGGGNNNNNNNNNNNNGGCC*	4.20	IR:8	18

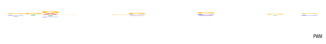	$C\overrightarrow{GGGNNN}NNNNNNNNNAGTG$	4.21	M	19
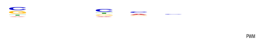	$\overrightarrow{GGCCNN}NNNNNNNNNN\overrightarrow{GGTG}$	3.87	DR:10	20

355 RXRA:RXRA-Bhimsaria2022-Rnd3 (S13-158)

PWM	Seed Sequence	Seed Seq Enrichment	Repeat	Rank
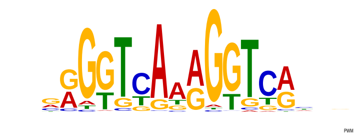	NNNNGGTCANNGGTCNNNN	223.18	DR:1	1
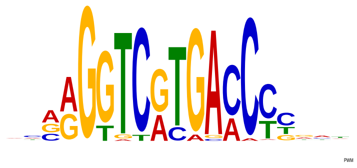	NNNAGGTCGTGACCCCNNN	4931.66	IR:0	2
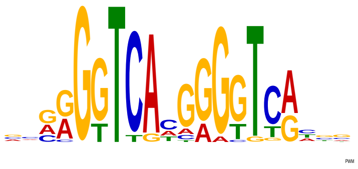	NNNNGGTCACGGGGTCNNNN	1181.17	DR:2	3
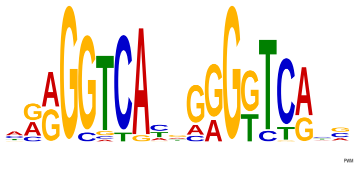	NNNGGTCACNGGGGTCNNN	427.02	DR:3	4
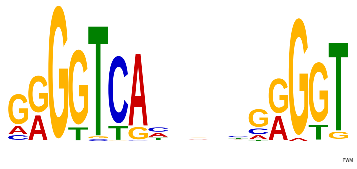	GGGGTCACNNNNGGGGT	1927.04	DR:6	5
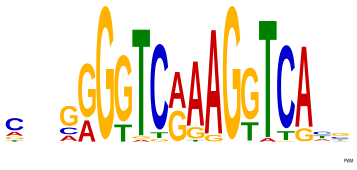	NNNGGGGTCGAAGGTCNNN	2026.02	DR:1	6
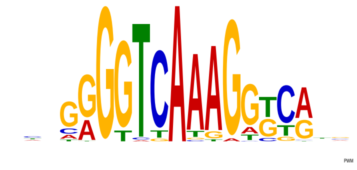	NNNGGGGTCAAAGGGCNNN	2080.77	DR:1	7
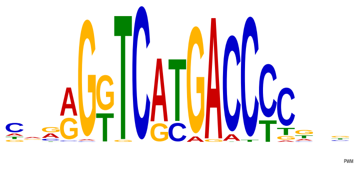	NNNAGTTCANGACCCCNNN	1308.03	IR:0	8
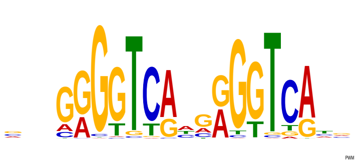	NNNGGGGTCNNNNGGTCNNN	157.27	DR:2	9

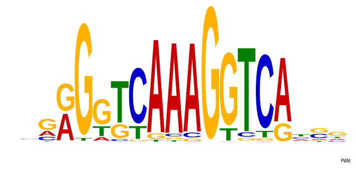	NNNNGGGCAAAGGTCNNNN	397.15	DR:1	10
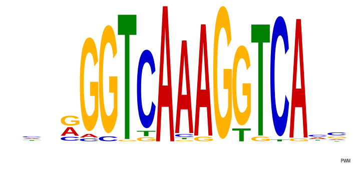	NNNCGGTCAAAGGTCNNN	605.08	DR:1	11
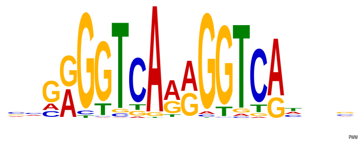	NNNNNGTCAGGGGTCNNNNN	134.11	DR:1	12
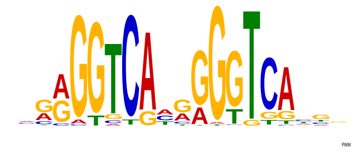	NNNNGGTCATNNGGTCNNNN	129.69	DR:2	13
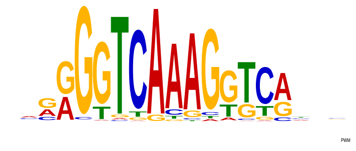	NNNNGGTCNAAGGGCNNNN	179.15	DR:1	14
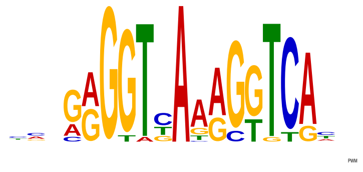	NNNGGGGTTAGAGGTCNNN	622.28	DR:1	15
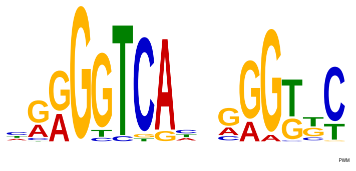	NGGGGTCACNGGGGTN	374.36	DR:3	16
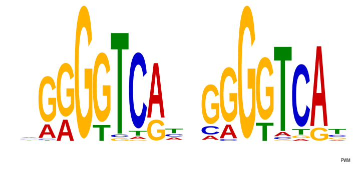	NNGGGGTCNNNGGGGTCNN	304.36	DR:3	17
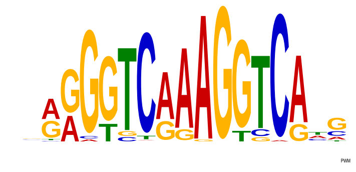	NNNAGGTCGAAGGTCNNN	507.96	DR:1	18

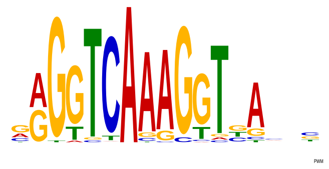	$NNN\overrightarrow{AGGTCN}A\overrightarrow{AGGTGA}NNN$	898.62	DR:1	19
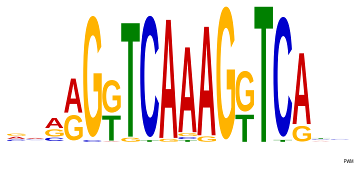	$NNN\overrightarrow{AGTTCA}A\overrightarrow{AGTTCN}NN$	797.14	DR:1	20

356 RXRA-17-Bhimsaria2022-Rnd1 (S04-147)

PWM	Seed Sequence	Seed Seq Enrichment	Repeat	Rank
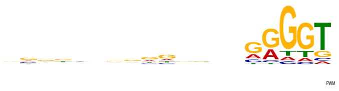	AGGGNNNNNNNNNNNNGGGT	14.51	DR:10	1
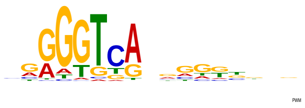	NNNNGGGGTCANGGGGNNNN	55.35	M	2
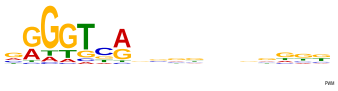	NGGGGTCANNNNNNGGGGN	51.26	M	3
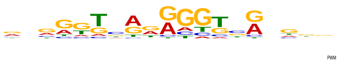	NNNNGGTCNNNGGTCANNNN	8.18	DR:1	4
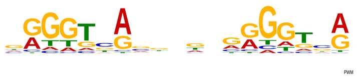	NGGGTCNNNNNNGGGTCN	13.97	DR:6	5
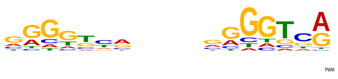	NGGGGTNNNNNNGGGTCN	14.75	DR:5	6
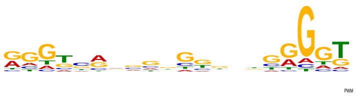	AGGTCANNNNNNNNNNGGGT	18.65	DR:10	7
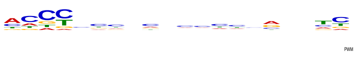	ACCTNNNNNNNNNNNNGGTC	4.85	ER:11	8
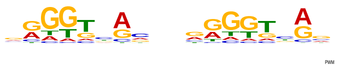	NNGGGTCNNNNNGGGTCNN	11.22	DR:4	9

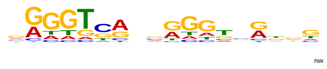	*NNNNGGTCANNNGGTCNNNN*	11.06	DR:2	10
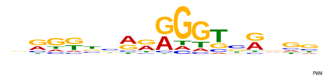	*NNNGGGTNNNNGGTCANNN*	8.93	DR:1	11
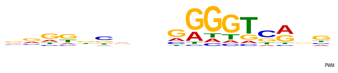	*NNGGGTNNNNNNGGTCANN*	6.72	DR:3	12
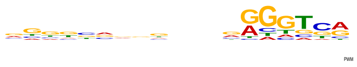	*GGGTNNNNNNNNNNGGTCA*	5.61	DR:7	13
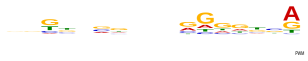	*NCGGGNNNNNNNNNNGGTCN*	2.55	M	14
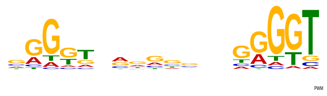	*AAGGGGTNNNNNNNNNGGGT*	38.55	DR:9	15
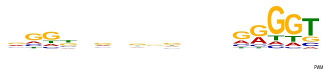	*AAAGGNNNNNNNNNNNGGGT*	13.49	DR:8	16
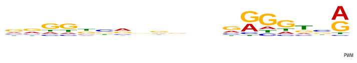	*NGGGTNNNNNNNNNGGTCN*	4.18	DR:6	17
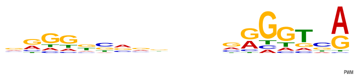	*NGGGTNNNNNNNGGGTCN*	7.81	DR:6	18

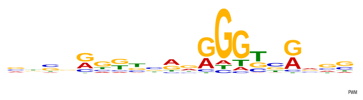	$NNNG\overrightarrow{GGGTNN}NN\overrightarrow{NGGTCA}NNN$	13.01	DR:1	19
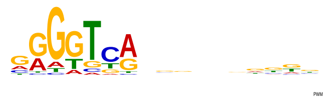	$NNG\overrightarrow{GGGTCA}NNNNNGGGGNN$	38.15	M	20

357 RXRA-17-Bhimsaria2022-Rnd2 (S04-148)

PWM	Seed Sequence	Seed Seq Enrichment	Repeat	Rank
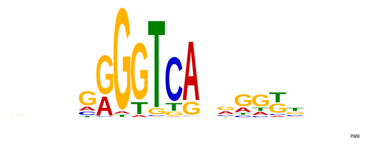	NNNNGGGGTCANGGGGNNNN	403.96	M	1
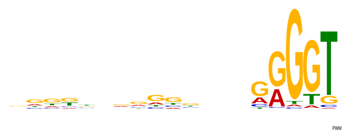	AGGGNNNNNNNNNNNGGGGT	140.19	DR:10	2
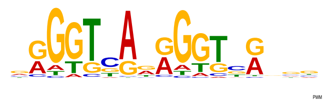	NNNNGGTCANGGGTCNNNN	217.72	DR:1	3
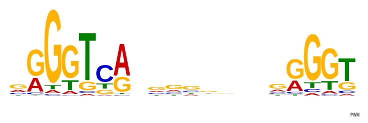	GGGGTCANNNNNNNNNGGGT	812.54	DR:9	4
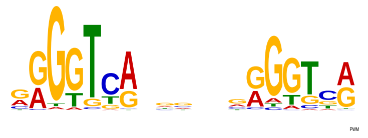	NGGGTCANNNNNNGGGTCN	344.84	DR:6	5
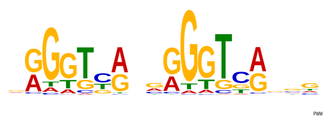	NNNGGGTCNNNGGGTCANNN	380.75	DR:2	6
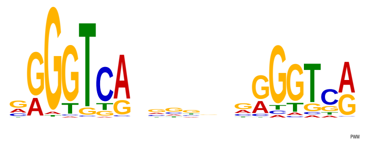	NGGGTCANNNNNNNGGGTCN	358.86	DR:7	7
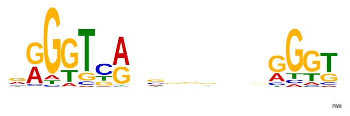	AGGGGTCANNNNNNNNGGGT	900.48	DR:8	8
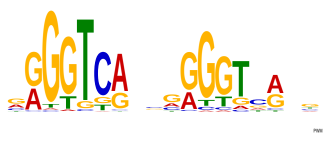	NNNGGGTCANNNGGGTCNNN	268.77	DR:3	9

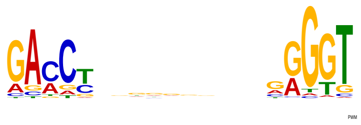	$\overleftarrow{GACCT}NNNNNNNNNNN\overrightarrow{GGGT}$	144.11	ER:11	10
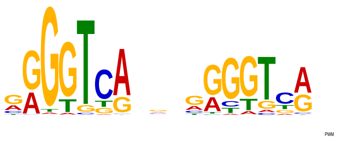	$NN\overrightarrow{GGGTCA}NNNN\overrightarrow{GGGTCN}N$	249.63	DR:4	11
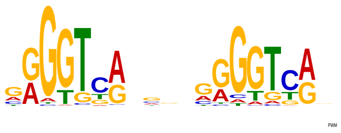	$NN\overrightarrow{GGGTCN}NNNNN\overrightarrow{GGGTCN}N$	115.42	DR:5	12
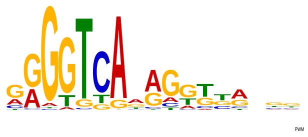	$NNNN\overrightarrow{GGGTCA}N\overrightarrow{NGGGCN}NNN$	145.59	DR:1	13
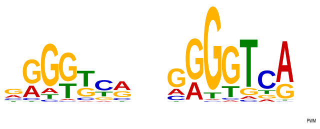	$NNG\overrightarrow{GGGTN}NNNG\overrightarrow{GGGTCN}N$	171.36	DR:3	14
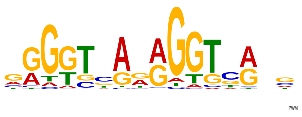	$NNNG\overrightarrow{GGGTNN}N\overrightarrow{NGGTCN}NN$	82.83	DR:1	15
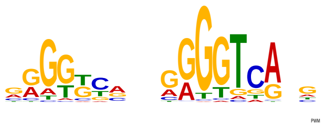	$NNN\overrightarrow{GGGTNN}NNG\overrightarrow{GGGTCN}NN$	94.42	DR:3	16
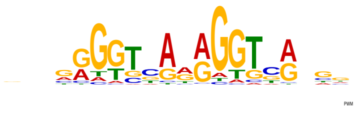	$NNNN\overrightarrow{GGGTNN}N\overrightarrow{NGGTCN}NNN$	48.20	DR:1	17
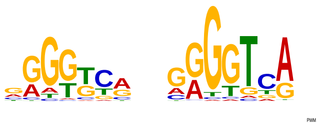	$NNG\overrightarrow{GGGTNN}NNN\overrightarrow{GGGTCN}N$	77.14	DR:3	18

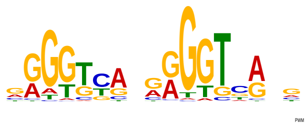	$NNNG\overrightarrow{GGGTNN}NNN\overrightarrow{GGGTCN}NN$	123.54	DR:2	19
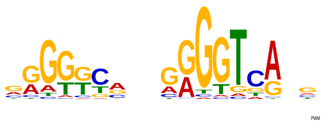	$NNN\overrightarrow{GGGTNN}NNNN\overrightarrow{GGGTCN}NN$	42.31	DR:3	20

358 RXRA-17-Bhimsaria2022-Rnd2 (S07-014)

PWM	Seed Sequence	Seed Seq Enrichment	Repeat	Rank
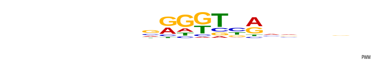	NNNNNNCGGGGTCANNNNNN	6.10	M	1
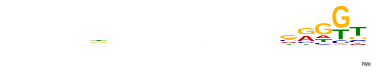	GGGGNNNNNNNNNNNNGGGT	7.34	M	2
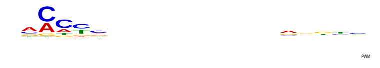	ACCCNNNNNNNNNNNNGGTC	6.40	ER:11	3
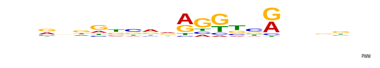	NNNNNNCAAAGGTCNNNNNN	6.39	M	4
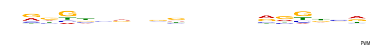	NGGTCNNNNNNNNNGGTCN	3.30	DR:7	5
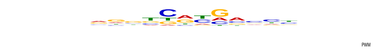	NNNNNNGTCATGACNNNNNN	4.32	IR:0	6
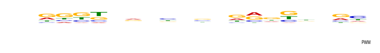	NNGGTCNNNNNNNNGGTCNN	3.07	DR:6	7
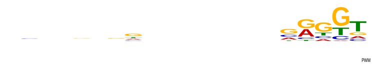	CCCGNNNNNNNNNNNNGGGT	5.39	M	8
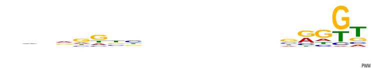	AAAGGNNNNNNNNNNNGGGT	8.36	DR:8	9

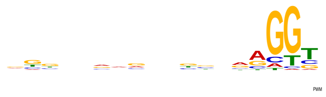	$CGGGNNNNNNNNNNNNA\overrightarrow{AGGT}$	8.23	M	10
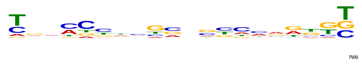	$\overleftarrow{NGACCT}NNNNNNNNN\overrightarrow{GGGTN}$	3.97	ER:9	11
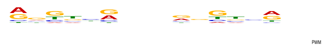	$NN\overrightarrow{NGGTCN}NNNN\overrightarrow{NGGTCN}NN$	3.32	DR:3	12
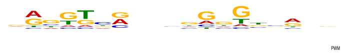	$NN\overrightarrow{NGGTCN}NNNNN\overrightarrow{NGGTCN}NN$	3.53	DR:4	13
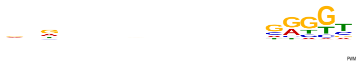	$ATGCNNNNNNNNNNNN\overrightarrow{GGGT}$	4.26	M	14
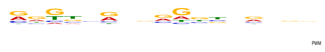	$NN\overrightarrow{NGGTCN}NNN\overrightarrow{NNGTCA}NNN$	3.57	DR:2	15
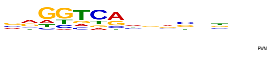	$NNNNNN\overrightarrow{GGGTCA}TGNNNNNN$	5.03	M	16
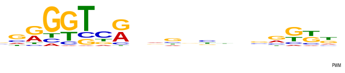	$NG\overrightarrow{GGGTCN}NNNNNNN\overrightarrow{GGGTN}$	13.19	DR:7	17
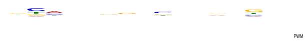	$\overrightarrow{GGGTNN}NNNNNNNNN\overrightarrow{NGGTC}$	2.07	DR:8	18

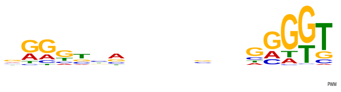	$CG\overrightarrow{GGGTN}NNNNNNNNG\overrightarrow{GGGT}$	27.23	DR:8	19
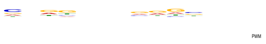	$\overrightarrow{GGTCN}NNNNNNNNN\overrightarrow{NGTCA}$	1.40	DR:8	20

359 RXRA-17-Bhimsaria2022-Rnd3 (S04-149)

PWM	Seed Sequence	Seed Seq Enrichment	Repeat	Rank
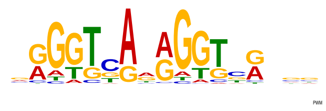	NNNNGGTCANNGGTCNNNN	265.78	DR:1	1
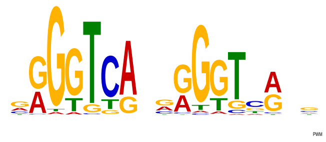	NNNGGGTCANNGGGTCNNN	874.40	DR:2	2
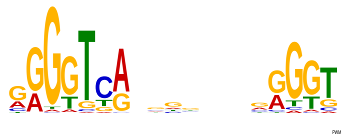	CGGGGTCANNNNNNNNGGGT	1468.61	DR:8	3
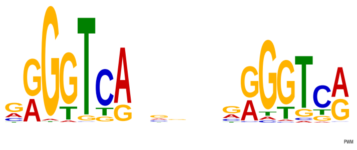	NGGGTCANNNNNNGGGTCN	627.50	DR:6	4
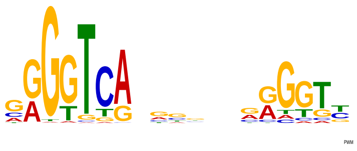	GGGGTCANNNNNNNGGGTC	549.81	DR:7	5
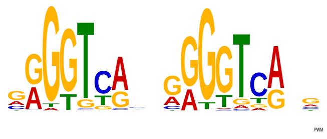	NNNGGGTCNNNGGGGTCNNN	366.92	DR:3	6
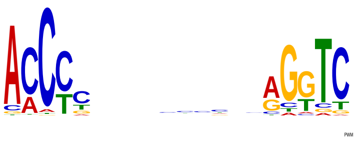	ACCCCNNNNNNNNNNAGGTC	402.46	ER:11	7
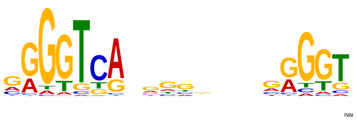	GGGGTCANNNNNNNNNGGGT	1078.24	DR:9	8
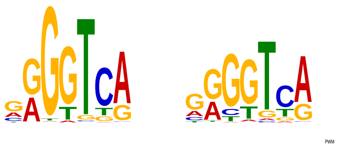	NNGGGTCANNNNGGGTCNN	325.14	DR:4	9

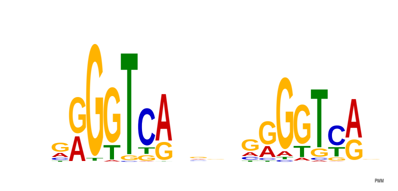	NNGGGTCANNNNNGGGTCNN	425.84	DR:5	10
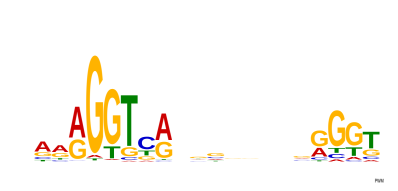	AAAGGTCNNNNNNNNNGGGT	548.36	DR:8	11
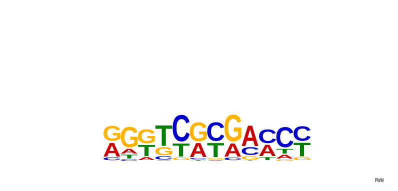	NNNNNGGTCATGACCNNNNN	180.40	IR:0	12
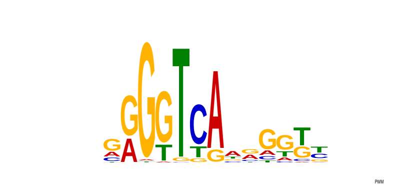	NNNNGGGGTCANAGGGNNNN	569.14	DR:1	13
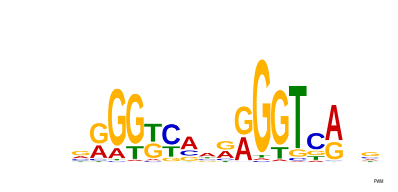	NNNGGGTNNNNNGGTCNNN	56.97	DR:2	14
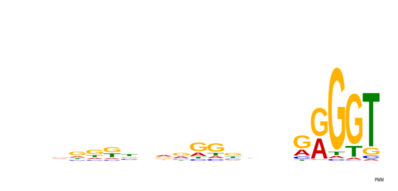	AGGGNNNNNNNNNNNNGGGT	67.16	DR:10	15
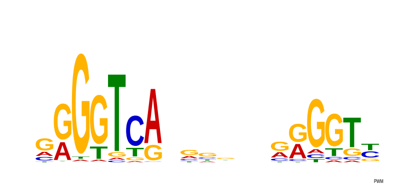	GGGGTCNNNNNNNNGGGTC	207.16	DR:7	16
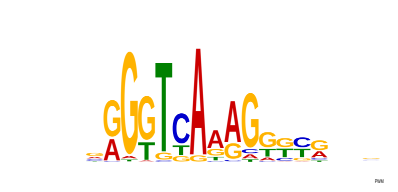	NNNNGGGTCAAAGGGCNNNN	1165.87	DR:1	17
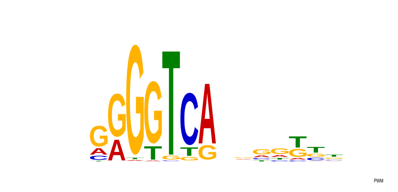	NNNGGGGTCANNGGGGNNN	462.19	M	18

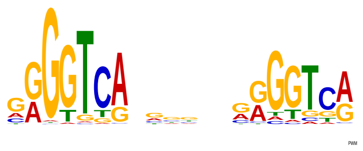	$N\overrightarrow{GGGTCA}NNNNNNN\overrightarrow{GGGTCN}$	492.16	DR:7	19
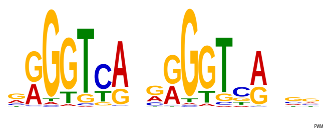	$NNN\overrightarrow{NGGTCN}NN\overrightarrow{GGGTCN}NNN$	157.15	DR:2	20

360 RXRA-17-Bhimsaria2022-Rnd3 (S07-135)

PWM	Seed Sequence	Seed Seq Enrichment	Repeat	Rank
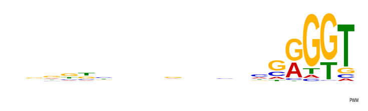	GGGGNNNNNNNNNNNGGGT	145.33	M	1
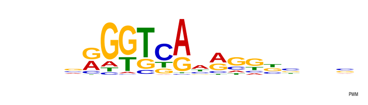	NNNNNGGTCAAAGGNNNNN	102.98	DR:1	2
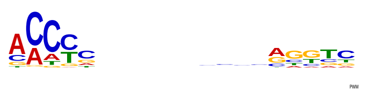	ACCCNNNNNNNNNNNNGGTC	62.28	ER:11	3
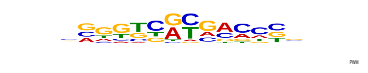	NNNNNGGTCATGACCNNNNN	172.58	IR:0	4
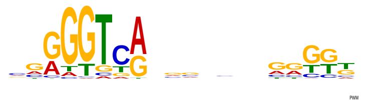	NGGGGTCNNNNNNNNGGGTN	149.65	DR:7	5
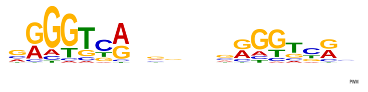	NNGGTCNNNNNNNNGGTCNN	28.06	DR:6	6
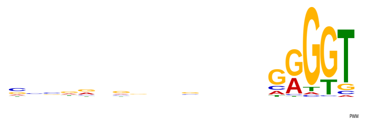	CCCANNNNNNNNNNNGGGT	67.97	M	7
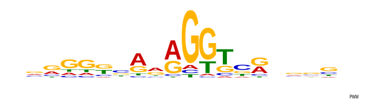	NNNNNNCAAAGGTCNNNNNN	51.71	M	8
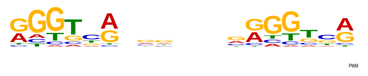	NGGTCNNNNNNNNGGTCN	23.46	DR:7	9

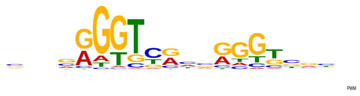	NNNGGGGTCNNNNGGTCNNN	102.39	DR:2	10
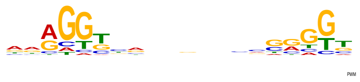	AAAGGTNNNNNNNNNNGGGT	131.09	DR:8	11
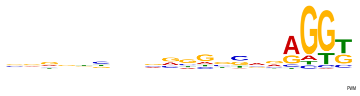	GGGGNNNNNNNNNNAAAGGT	129.38	M	12
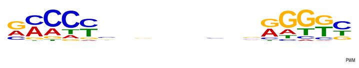	NACCCNNNNNNNNNGGGTN	26.82	ER:9	13
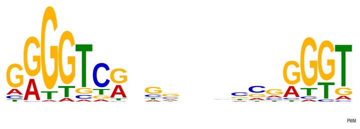	GGGGTCGCNNNNNNNNGGGT	500.24	DR:9	14
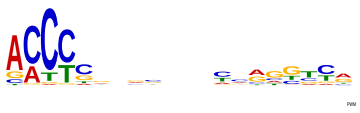	ACCCCNNNNNNNNNNGGTCG	110.62	ER:10	15
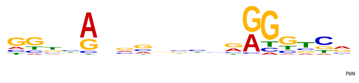	GGTCNNNNNNNNNNNGTCA	8.99	DR:8	16
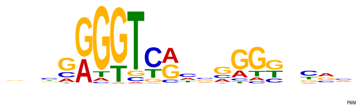	NNNGGGGTCNNNNGGGTNNN	76.45	DR:3	17
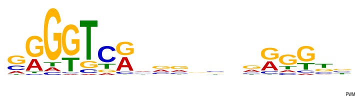	GGGGTCNNNNNNNNNGGTC	86.74	DR:7	18

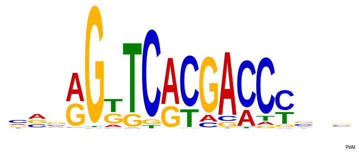	$NNN\overrightarrow{AGTTCA}\overleftarrow{CGACCC}NNN$	636.21	IR:0	19
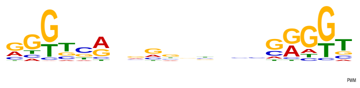	$\overrightarrow{GGGTCA}NNNNNNNNNN\overrightarrow{GGGT}$	118.38	DR:10	20

361 RXRA-Bhimsaria2022-Rnd2 (S07-049)

PWM	Seed Sequence	Seed Seq Enrichment	Repeat	Rank
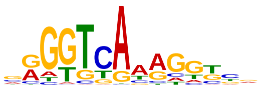	NNNNNGGTCAAAGGNNNNN	56.42	DR:1	1
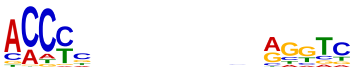	ACCCNNNNNNNNNNNNGGTC	22.77	ER:11	2
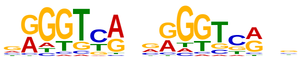	NNNNGGTCNNNNGGTCNNNN	16.54	DR:2	3
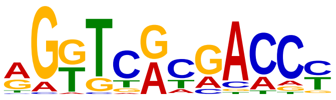	AGGTCACGACCC	265.08	IR:0	4
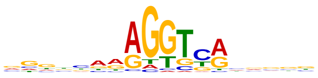	NNNNNNAAAGGTCANNNNNN	25.43	M	5
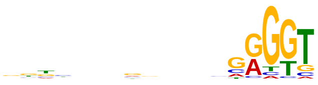	GGGGNNNNNNNNNNNGGGGT	27.73	M	6
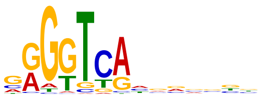	NNNNNGGGGTCACGNNNNN	29.25	M	7
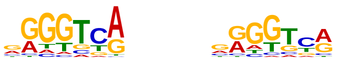	NNGGTCNNNNNNNNGGTCNN	10.17	DR:6	8
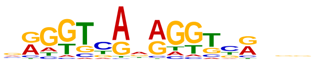	NNNNGGTCNNNGGTCNNNN	19.51	DR:1	9

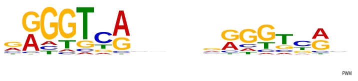	*NNGGTCNNNNNNNGGTCNN*	8.75	DR:5	10
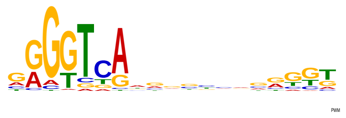	*NGGGGTCNNNNNNNNGGGTN*	29.84	DR:7	11
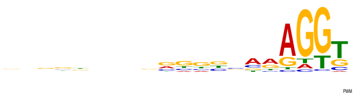	*GGGGNNNNNNNNNNNAAAGGT*	47.54	M	12
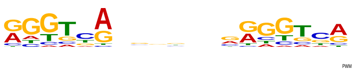	*NGGTCNNNNNNNNNGGTCN*	6.98	DR:7	13
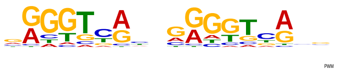	*NNNGGTCNNNNNGGTCNNN*	8.77	DR:3	14
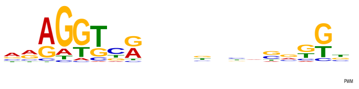	*AAAGGTCNNNNNNNNNGGGT*	83.87	DR:8	15
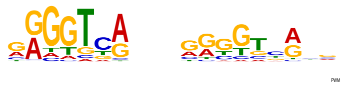	*NNNGGTCNNNNNNGGTCNNN*	8.18	DR:4	16
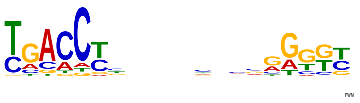	*NGACCTNNNNNNNNNGGGTN*	17.16	ER:9	17
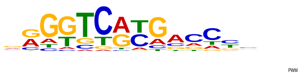	*NNNNNGGTCATGACNNNNN*	31.40	IR:0	18

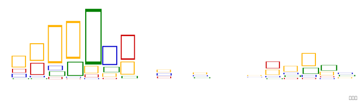	$G\overrightarrow{GGGTCN}NNNNNNN\overrightarrow{NGGTC}$	22.09	DR:7	19
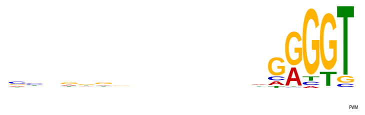	$CCCGNNNNNNNNNNNG\overrightarrow{GGGT}$	21.04	M	20

362 RXRA-Bhimsaria2022-Rnd3 (S07-170)

PWM	Seed Sequence	Seed Seq Enrichment	Repeat	Rank
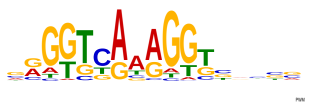	NNNNNGGTCAAAGGTNNNNN	1081.03	DR:1	1
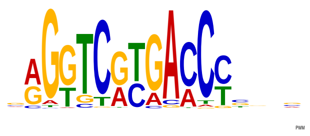	NNNNAGGTCGTGACCCNNNN	5171.56	IR:0	2
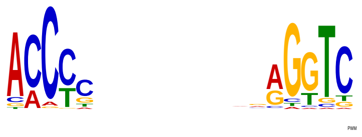	ACCCCNNNNNNNNNNAGGTC	725.01	ER:11	3
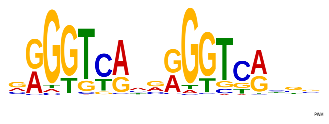	NNNNGGTCNNNNGGTCNNNN	123.73	DR:2	4
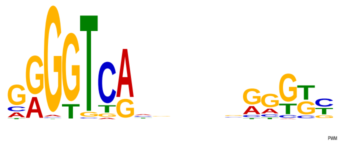	NGGGGTCNNNNNNNGGGTN	277.81	DR:6	5
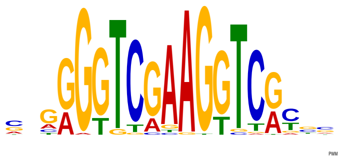	NNNGGGGTCGAAGGTCGNNN	11347.95	DR:1	6
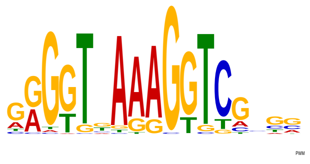	NNNGGGGTGAAAGGTCGNNN	8232.94	DR:1	7
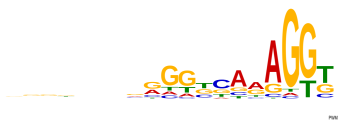	GGGGNNNNNNNNNNAAAGGT	378.55	M	8
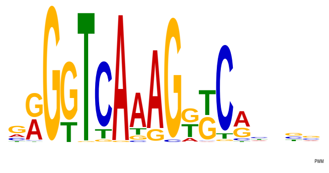	NNNNGGTCAAAGTGCANNNN	1683.69	DR:1	9

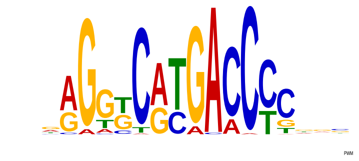	$NNN\overrightarrow{AGGGCA}\overleftarrow{TGACCC}CNNN$	3291.81	IR:0	10
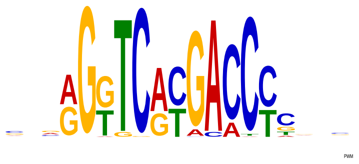	$NNN\overrightarrow{AGTTCA}\overleftarrow{CGACCC}CNNN$	5446.48	IR:0	11
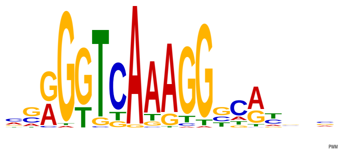	$NNN\overrightarrow{NGGTCA}A\overrightarrow{AGGCCA}NNNN$	1136.78	DR:1	12
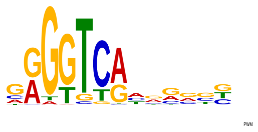	$NNNNNNG\overrightarrow{GGGTCA}TNNNNNN$	113.78	M	13
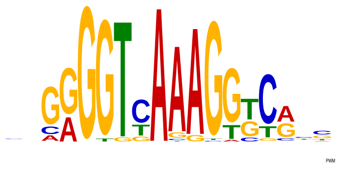	$NNNG\overrightarrow{GGGTTA}A\overrightarrow{AGGGCN}NN$	2445.88	DR:1	14
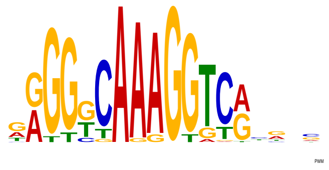	$NNN\overrightarrow{AGGGCA}A\overrightarrow{AGGTCG}CNNN$	4376.18	DR:1	15
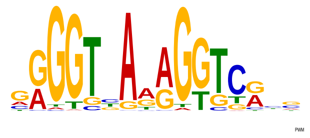	$NNNG\overrightarrow{GGGTTA}N\overrightarrow{NGGTCN}NN$	889.02	DR:1	16
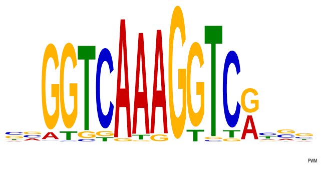	$NNN\overrightarrow{CGGTCA}A\overrightarrow{AGGTCG}NNN$	2951.08	DR:1	17
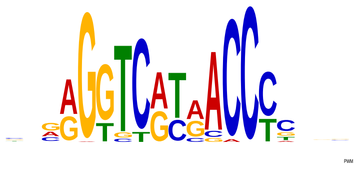	$NNN\overrightarrow{AGGTCA}\overleftarrow{TAACCC}CNNN$	4051.59	IR:0	18

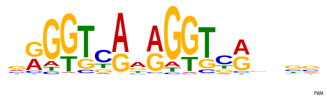	$NNN\overrightarrow{NGGTC}NNN\overrightarrow{NGTCA}NNNN$	120.32	DR:1	19
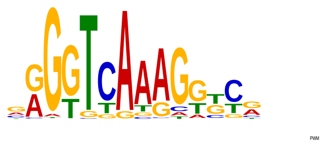	$NNN\overrightarrow{NGGTCA}A\overrightarrow{AGGGC}NNNN$	1533.13	DR:1	20

363 RXRA-Jolma2013-Rnd2 (ERR194225)

PWM	Seed Sequence	Seed Seq Enrichment	Repeat	Rank
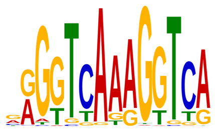	NNNNNNGGTCAANGGTCANNNNNN	451.93	DR:1	1
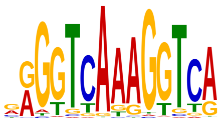	NNNNNNGGTCNNAGGTCNNNNNN	78.09	DR:1	2
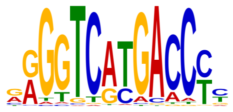	NNNNNNGGTCATGACCNNNNNN	55.57	IR:0	3
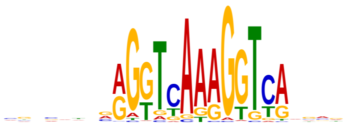	NNNNNNTGGGGTCNNNGGTCNNNNNN	73.22	DR:1	4
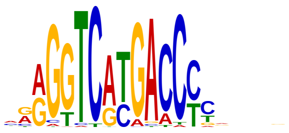	NNNNNNAGGTCANGACCCCNNNNNN	175.16	IR:0	5
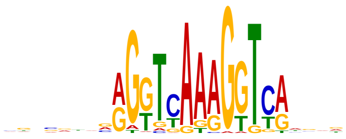	NNNNNNTGGGGTCNNAGGTCNNNNNN	194.95	DR:1	6
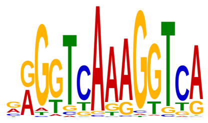	NNNNNNGGGGTCNNNGGTCNNNNNN	86.49	DR:1	7
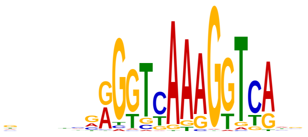	NNNNNNTGGGGTNNNAGGTCNNNNNN	74.42	ER:3	8
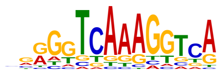	NNNNNNGGGGNNNNNGGTCNNNNNN	10.30	M	9

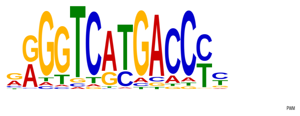	$NNNNN\overrightarrow{NGGTCA}\overleftarrow{NGACC}NNNNNN$	26.48	IR:0	10
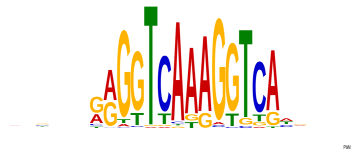	$NNNNNNTG\overrightarrow{AGGTCN}N\overrightarrow{NGGTCN}NNNNN$	91.33	DR:1	11

364 RXRA-Jolma2013-Rnd2 (ERR194493)

PWM	Seed Sequence	Seed Seq Enrichment	Repeat	Rank
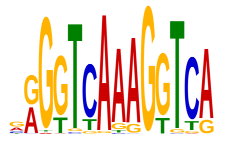	NNNNNNGGTCAAAGGTCANNNNNN	2495.54	DR:1	1
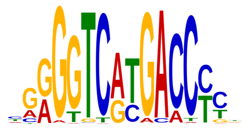	NNNNNNGGGGTCATGACCNNNNNN	575.60	IR:0	2
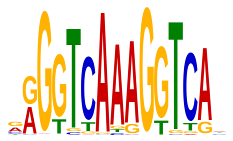	NNNNNNAGGTCNAAGGTCNNNNNN	552.47	DR:1	3
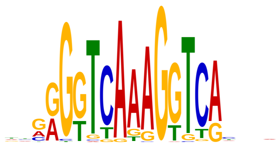	NNNNNNCGGGGTCNAAGGTCNNNNNN	1265.07	DR:1	4
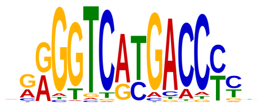	NNNNNNGGTCANGACCNNNNNN	57.95	IR:0	5
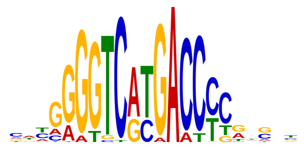	NNNNNNTGGGGTCNNGACCCCNNNNNN	781.43	IR:0	6
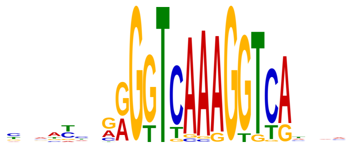	NNNNNNCGAGGTCNAAGGTCNNNNNN	828.10	DR:1	7
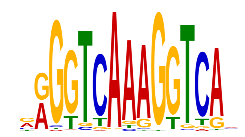	NNNNNNAGGTCNNAGGTCNNNNNN	168.53	DR:1	8
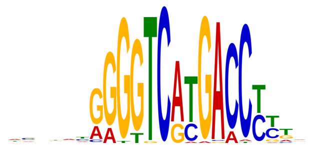	NNNNNNTGGGGTCANGACCTNNNNNN	704.46	IR:0	9

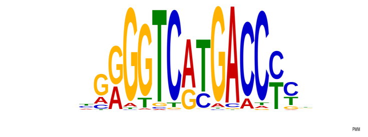	NNNNNNGGGGTCNNGACCNNNNNN	110.54	IR:0	10
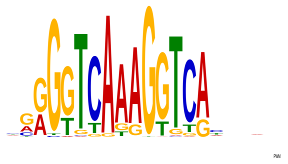	NNNNNNCGGGGTCNNAGGTCNNNNNN	397.59	DR:1	11
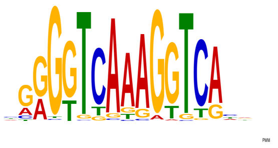	NNNNNNCGGGNTCAAAGGNNNNNN	167.00	DR:1	12
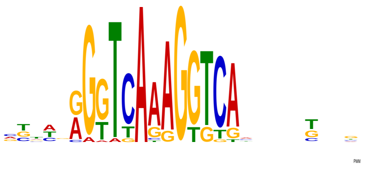	NNNNNNGGTCAAAGNTCAAATNNNNNN	1353.64	DR:1	13
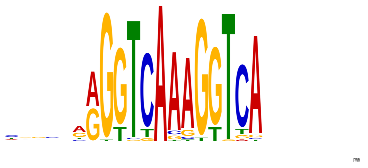	NNNNNNAGGTCNNAGGTCAGNNNNNN	299.26	DR:1	14
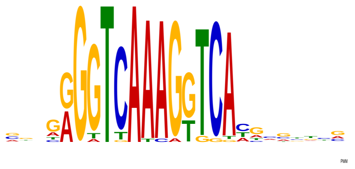	NNNNNNGGTCAAAGTTCACGNNNNNN	1635.09	DR:1	15
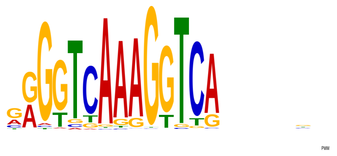	NNNNNNTCAAANGTCATNNNNNN	107.46	DR:1	16
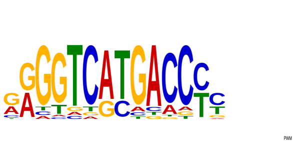	NNNNNNGGTCNNGACCNNNNNN	23.17	IR:0	17
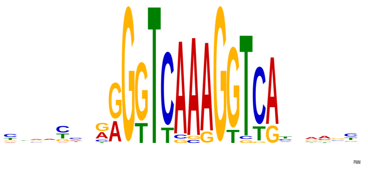	NNNNNNCGAGGTCNNAGGTCANNNNNN	626.85	DR:1	18

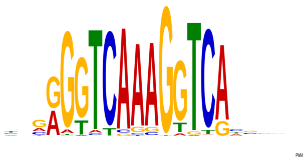	$NNNNNNC\overrightarrow{GGGTCN}A\overrightarrow{AGGTCN}NNNNN$	431.99	DR:1	19
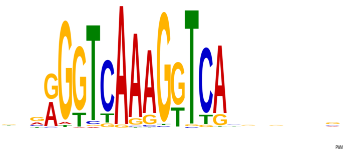	$NNNN\overrightarrow{NNGTCA}A\overrightarrow{AGNTCA}CGNNNNNN$	500.35	DR:1	20

365 RXRA-Jolma2013-Rnd3 (ERR194226)

PWM	Seed Sequence	Seed Seq Enrichment	Repeat	Rank
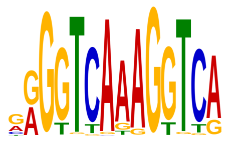	NNNNNNGGTCNNAGGTCNNNNNN	930.71	DR:1	1
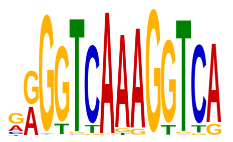	NNNNNNGGTCNNNGGTCNNNNNN	435.26	DR:1	2
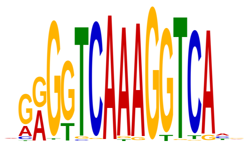	NNNNNNGAGTTCNNNGGTCNNNNNN	376.50	DR:1	3
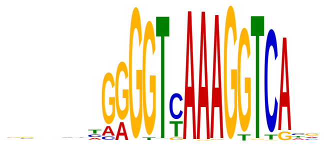	NNNNNNTGGGGTTANNGGTCNNNNNN	1208.11	DR:1	4
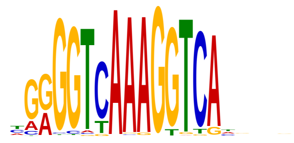	NNNNNNGGGGTTNNNGGTCNNNNNN	273.00	DR:1	5
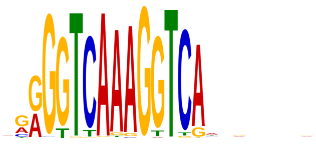	NNNNNNGGTCNNNNGTCAGCNNNNNN	258.54	DR:1	6
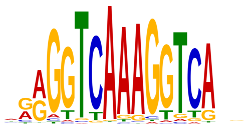	NNNNNNAAGGNNNNNGGTCNNNNNN	83.55	DR:1	7
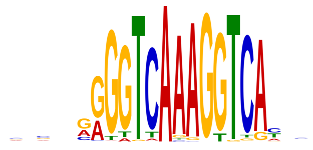	NNNNNNCCGGGTCNNNGGTCNNNNNN	526.66	DR:1	8
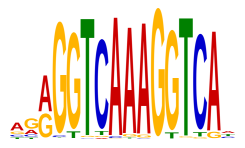	NNNNNNTAGGTCNNNGGTCNNNNNN	238.78	DR:1	9

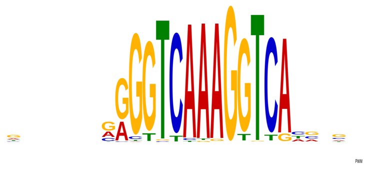	$NNNNNNTC\overrightarrow{GGGTC}NN\overrightarrow{NGGTC}NNNNNN$	463.39	DR:1	10
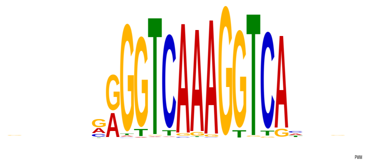	$NNNNNNC\overrightarrow{GGGTC}NN\overrightarrow{NGGTC}NNNNNN$	458.07	DR:1	11
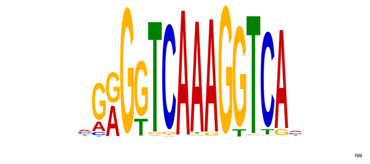	$NNNNNN\overrightarrow{GGGTT}CNN\overrightarrow{NGGTC}NNNNNN$	382.56	DR:2	12
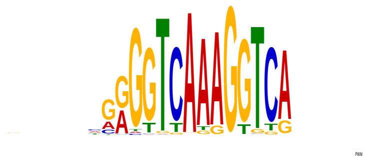	$NNNNNNCGGGGNNNN\overrightarrow{NGGTC}NNNNNN$	191.52	M	13

366 RXRA-Jolma2013-Rnd3 (ERR194494)

PWM	Seed Sequence	Seed Seq Enrichment	Repeat	Rank
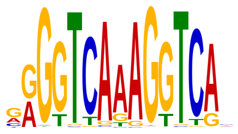	NNNNNNGGTCNNAGGTCNNNNNN	484.55	DR:1	1
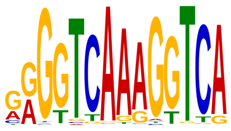	NNNNNNNTCAANNGTCANNNNNN	258.65	DR:1	2
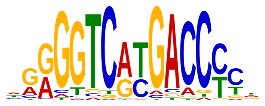	NNNNNNGGTCNNGACCNNNNNN	69.55	IR:0	3
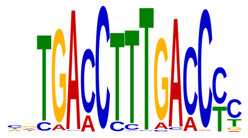	NNNNNNCGTGANNNTTGANNNNNN	199.89	IR:4	4
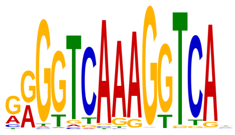	NNNNNNNTCAANNNTCAANNNNNN	130.33	DR:1	5
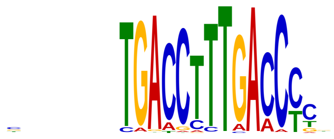	NNNNNNCGATGANNNTTGANNNNNN	180.61	IR:4	6
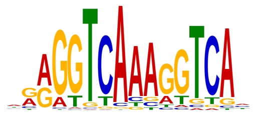	NNNNNNAAGGNNNAAGGNNNNNN	45.95	DR:1	7
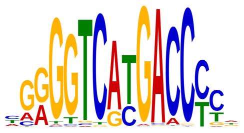	NNNNNNGGGGTNNNGACCNNNNNN	107.82	IR:0	8
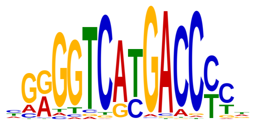	NNNNNNGGGTNNNGACCNNNNNN	48.59	IR:0	9

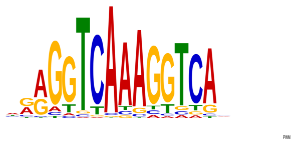	$NNNNNNA\overrightarrow{AGGNNN}N\overrightarrow{NGGTC}NNNNNN$	47.57	DR:1	10

367 Rxra-Jolma2013-Rnd3 (ERR194856)

PWM	Seed Sequence	Seed Seq Enrichment	Repeat	Rank
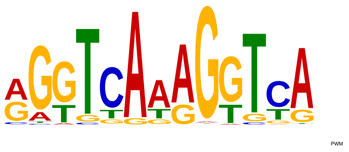	NGGTCNNGGTCAN	344.41	DR:1	1
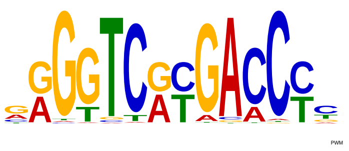	NNGGTCNNGACCNN	91.37	IR:0	2
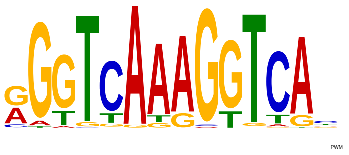	GGGTCNNNNGTCAC	377.60	DR:1	3
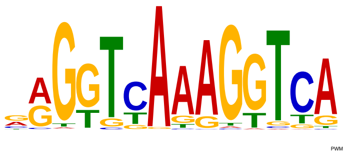	GAGGNNNNNNGTCA	54.72	DR:1	4
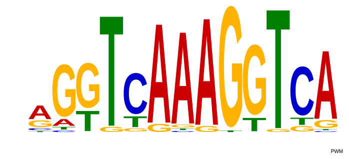	CAGGTCNNNNGTCA	182.31	DR:1	5
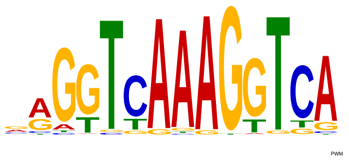	CAGGTNNNNNGTCA	68.98	DR:1	6
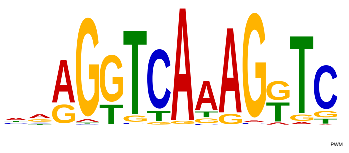	AAAGNNNNNAGTTC	57.83	M	7
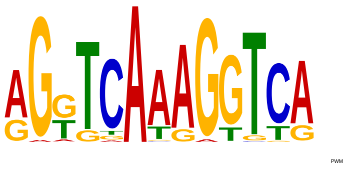	AGTTCNNNNGTCAA	183.43	DR:1	8
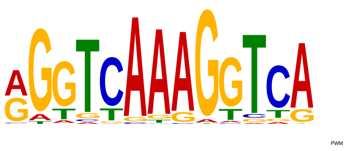	NGGTCNNNNGTCGN	33.01	DR:1	9

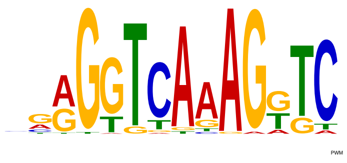	CGAGGNNNNAGTTC	116.39	DR:1	10
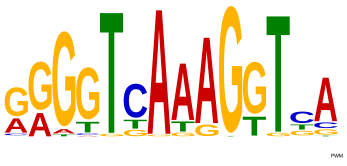	GGGGTNNNNGGTGA	117.05	DR:1	11
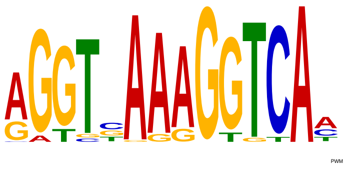	AGGTGANNNGTCAA	282.35	DR:1	12
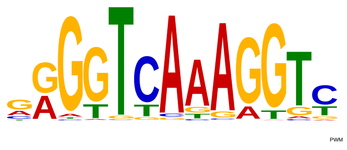	GGGGNNNNAGGTT	65.71	M	13
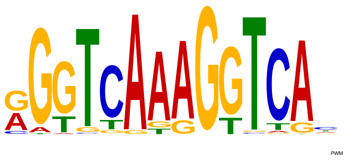	GGGTCNNNNNTCAC	140.79	DR:1	14
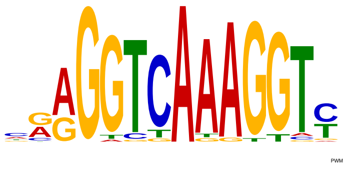	CGAGGNNNNAGGTT	111.54	DR:1	15
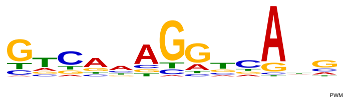	NTCAANNNTCGTN	2.99	M	16
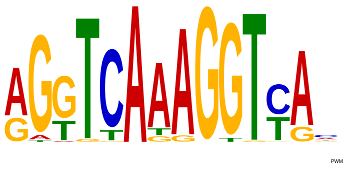	AGGTCNNNGGTTAC	421.05	DR:1	17
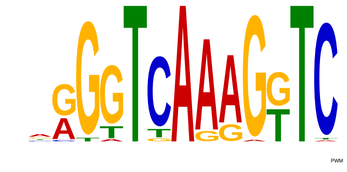	CAGGGTCNNNGTTC	209.53	DR:1	18

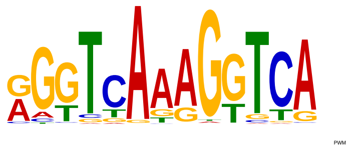	$\overrightarrow{AGGTNN}N\overrightarrow{NNNTCA}C$	46.79	DR:1	19
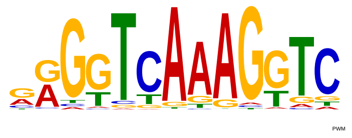	$GGGGNNNN\overrightarrow{NGTTC}$	25.01	M	20

368 RXRA-Jolma2013-Rnd4 (ERR194227)

PWM	Seed Sequence	Seed Seq Enrichment	Repeat	Rank
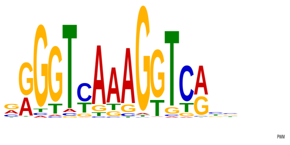	NNNNNNGGGTNNNNGGTCNNNNNN	32.65	DR:1	1
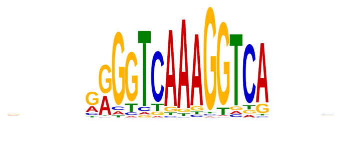	NNNNNNGAGGNNNNNNNNTCAGNNNNNN	10.87	DR:1	2
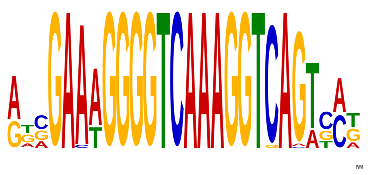	NNNGAAAGGGGNNNNNNGTCAGTNNN	746.04	DR:3	3
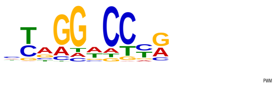	NNNNNNCAGGNCCTANNNNNN	9.54	M	4
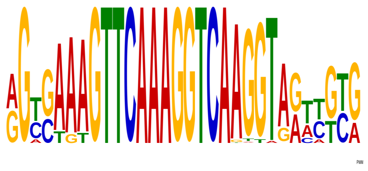	NNNNNNAGTTCNNNNNTCAAGGTANNNNNN	2574.40	M	5
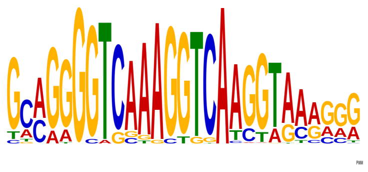	NNNNGGGTNNNNNNNCAAGGTAANNNN	275.27	DR:7	6
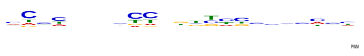	NNNNNNTCCCNNNTCCCNNNNNN	5.22	M	7
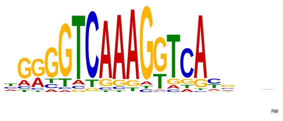	NNNNNNTGGGNNNNNNGTTCNNNNNN	6.82	M	8
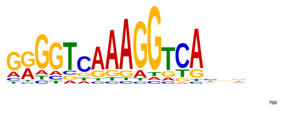	NNNNNNGGGGNNNNNGGTTNNNNNN	9.61	M	9

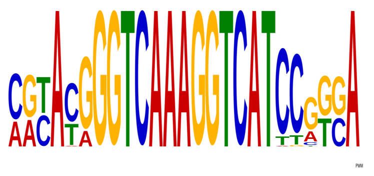	*NNNACGGGTCNNNNNTCATCCGNNN*	445.20	DR:1	10
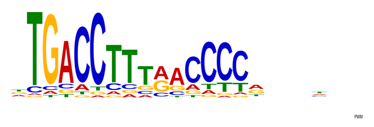	*NNNNNNTTTANNCCCANNNNNN*	9.21	M	11
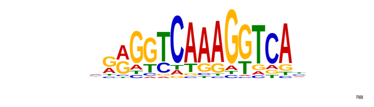	*NNNNNNAGAGNNNNNNGTTCNNNNNN*	5.31	M	12
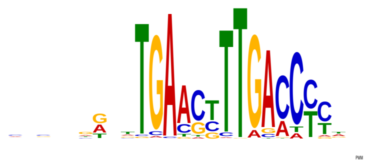	*NNNNNNATTTGAANNTTGANNNNNN*	50.78	IR:4	13
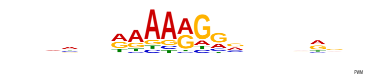	*NNNNNNAAAANGGAANNNNNN*	7.21	M	14
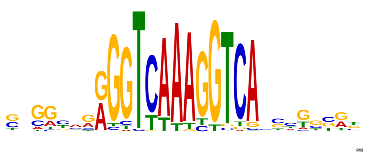	*NNNNNNCGGGTNNNNNNTCAATNNNNNN*	13.27	DR:1	15
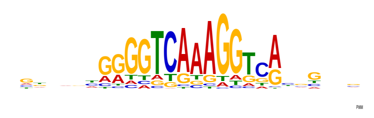	*NNNNNNTGGGNNNNNNNGTCGNNNNNN*	6.37	M	16
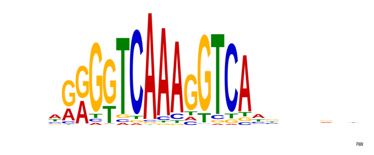	*NNNNNNGTTCNNNNNTCAATNNNNNN*	12.09	DR:1	17
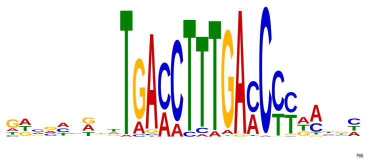	*NNNNNNATTTGANNNNNGAACCNNNNNN*	36.93	IR:4	18

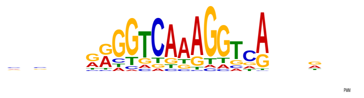	NNNNNNAGAGNNNNNN$\overrightarrow{NNGTC}$GNNNNNN	4.10	M	19
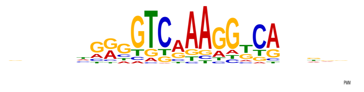	NNNNNNCGGGNNNNNN$\overrightarrow{NGGTT}$NNNNNN	4.35	M	20

369 RXRA-Jolma2013-Rnd4 (ERR194495)

PWM	Seed Sequence	Seed Seq Enrichment	Repeat	Rank
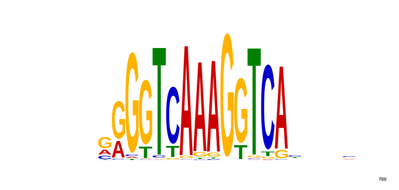	*NNNNNNGGGTNNNNNNTCACNNNNNNN*	25.68	DR:1	1
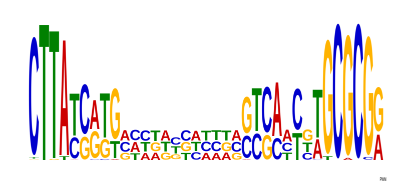	*CTTANNNNNNNNNNNNNNNNNNNNNNNNNNGCGCGG*	59.62	M	2
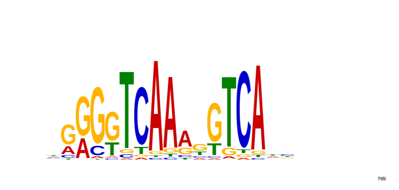	*NNNNNNTCAANNNNNCAACNNNNNN*	19.34	M	3
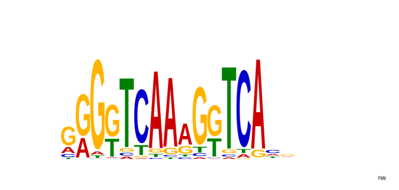	*NNNNNNTCAANNNNNCACGNNNNNN*	23.61	M	4
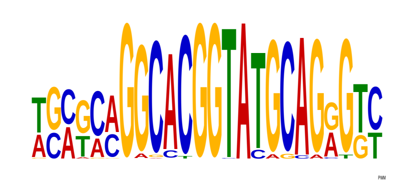	*NNNNNNGGCANGGTATGCNNNNNN*	117.46	DR:0	5
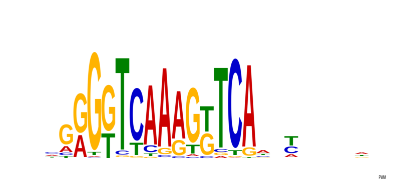	*NNNNNNTCAANNNNNCAAATNNNNNN*	29.20	M	6
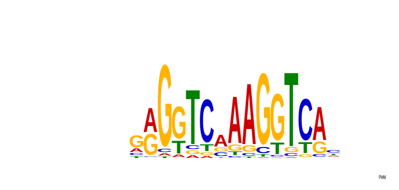	*NNNNNNAGAGNNNNNNAGGTNNNNNN*	13.80	M	7
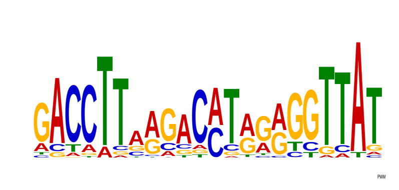	*NNNNNNAAGACNTAGGNNNNNN*	11.00	ER:0	8
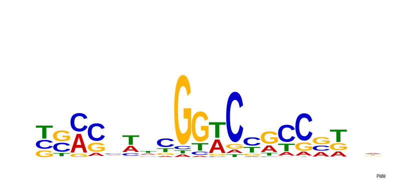	*NNNNNNTCGGTCCGNNNNNN*	8.93	M	9

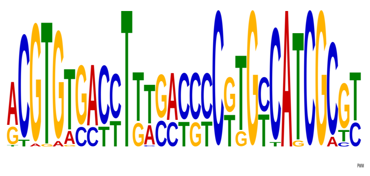	NCGTGNNNNNNNNNNNNNNNNNNCATCGCGN	38.61	M	10
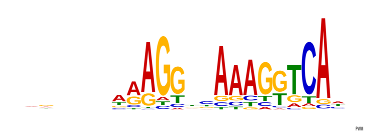	NNNNNNGAAANNNNAAAGNNNNNN	7.30	M	11
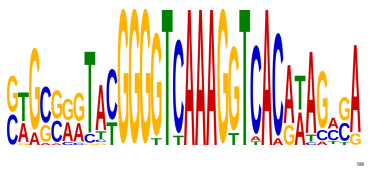	NNNNNNGTACGGNNNNNNNNNTCACANNNNNN	80.97	M	12
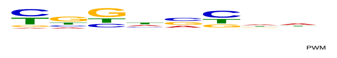	CGGTCTCG	2.06	M	13
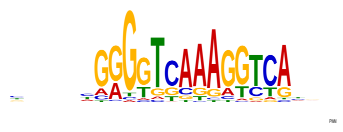	NNNNNNTCGGNNNNNNAAGGNNNNNN	11.05	M	14
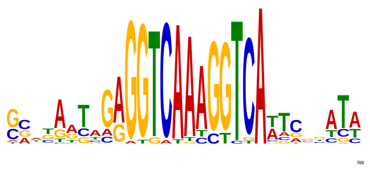	NNNNNNTAGAGNNNNNNNNNTCATTNNNNNN	28.77	M	15
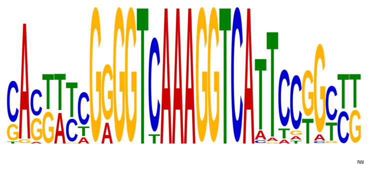	NNNNNTCGGGNNNNNNNNNTCATTCCNNNNN	128.46	M	16
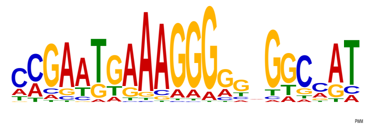	NNNNNNGAAANGGGGANNNNNN	18.44	M	17
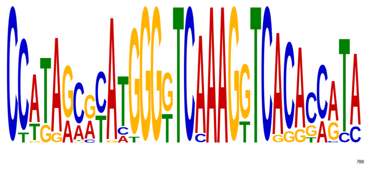	NNNNNNCGCATGGGNNNNNNNNNTCACNNNNNN	110.02	M	18

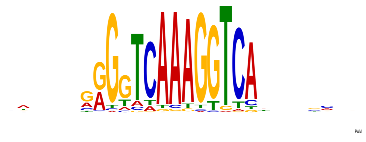	$NNNNNNG\overrightarrow{AGGNNN}NNNNNCAACNNNNNN$	8.21	M	19
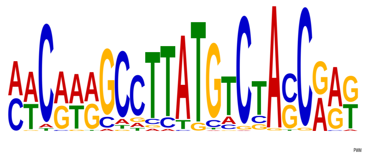	$NNNN\overleftarrow{NNGCCT}T\overrightarrow{NNGTCT}NNNNNN$	20.23	ER:1	20

370 Rxra-Jolma2013-Rnd4 (ERR194857)

PWM	Seed Sequence	Seed Seq Enrichment	Repeat	Rank
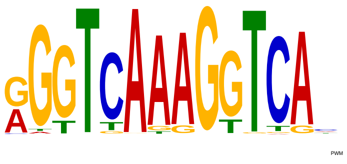	GGGTNNNNNNTCAC	72.81	DR:1	1
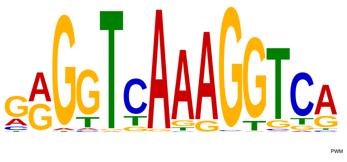	GGGGNNNNNNGTCG	21.98	M	2
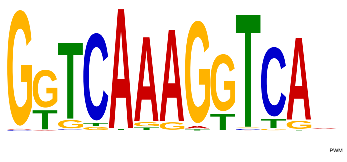	GTTCNNNNNTCAA	36.00	DR:1	3
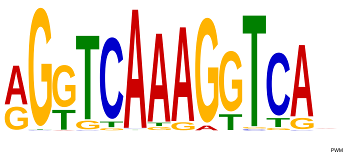	AGTTNNNNNNTCAA	33.93	DR:1	4
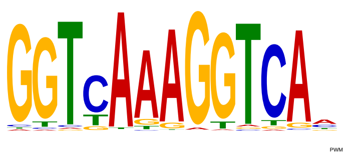	GGTTNNNNNTCAA	20.99	DR:1	5
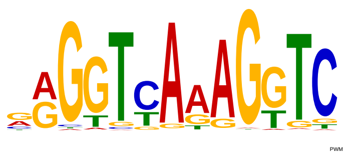	GGGGNNNNNGTTC	26.32	M	6
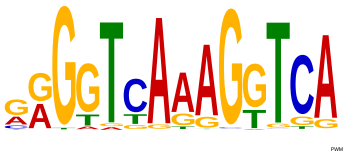	GGGGNNNNNNTTCA	22.65	M	7
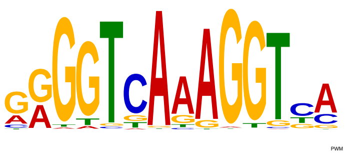	GGGGNNNNNNGTTA	25.11	M	8
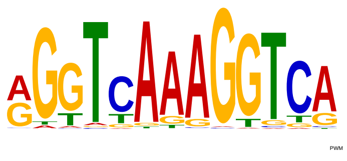	GGGTNNNNNGTCG	22.11	DR:1	9

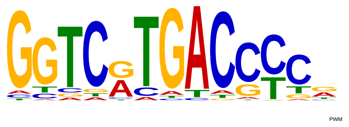	$\overrightarrow{NGTCG}NNNCCCCN$	5.29	M	10
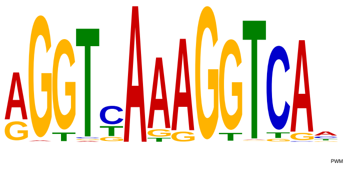	$\overrightarrow{AGGTTN}N\overrightarrow{NNNTCA}A$	54.77	DR:1	11
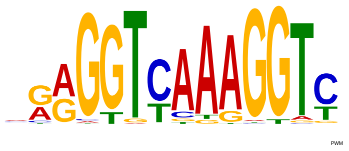	$CAAGNNNNN\overrightarrow{NGGTT}$	15.09	M	12
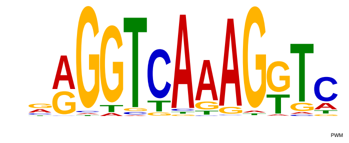	$CGGGNNNNN\overrightarrow{NGTTC}$	15.20	M	13
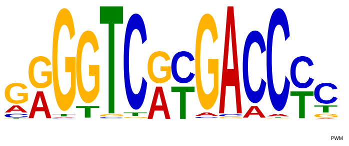	$GGGGNNNNNNCCCC$	11.91	M	14
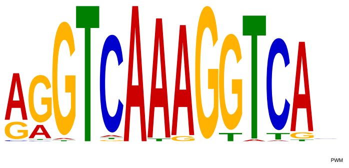	$\overrightarrow{AAGTCN}N\overrightarrow{NNNTCA}C$	24.32	DR:1	15
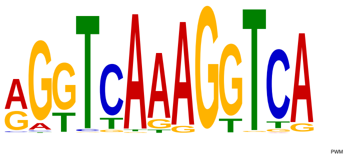	$\overrightarrow{AGGTNN}N\overrightarrow{NNNTCA}G$	42.79	DR:1	16
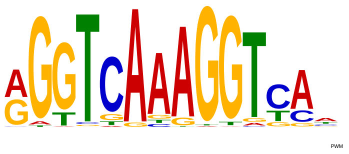	$\overrightarrow{GGGTNN}NN\overrightarrow{NGTTAA}$	31.74	DR:2	17
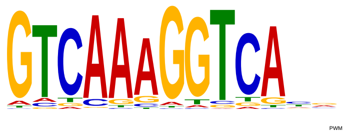	$\overrightarrow{GTCA}NNNNNCATG$	10.11	M	18

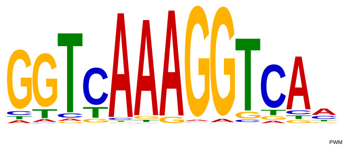	$\overrightarrow{GGTGN}N\overrightarrow{NNNTCA}A$	8.41	DR:1	19
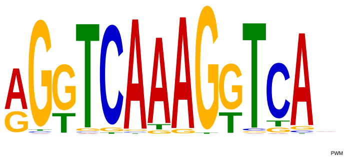	$\overrightarrow{AGTTNN}N\overrightarrow{NNNTCA}C$	24.08	DR:1	20

371 RXRA-Jolma2013-Rnd5 (ERR194496)

PWM	Seed Sequence	Seed Seq Enrichment	Repeat	Rank
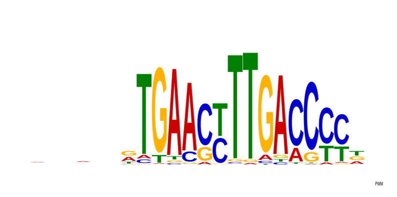	*NNNNNNGTGAANNTTGANNNNNNN*	35.21	IR:4	1
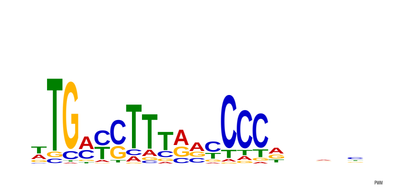	*NNNNNNTTTANNCCCGNNNNNN*	9.10	M	2
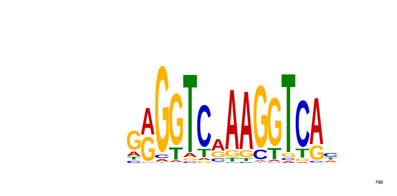	*NNNNNNAGAGNNNNNAGGTNNNNNN*	13.66	M	3
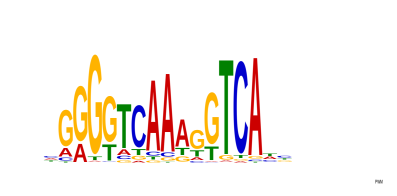	*NNNNNNTCAANNNNCAATNNNNNN*	13.95	M	4
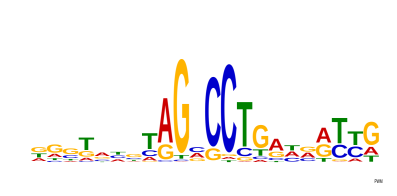	*NNNNNNGTAGNNCTGANNNNNN*	17.71	M	5
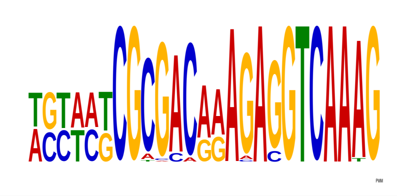	*NNNNNNCGCGACNNAGAGGNNNNNN*	76.78	ER:2	6
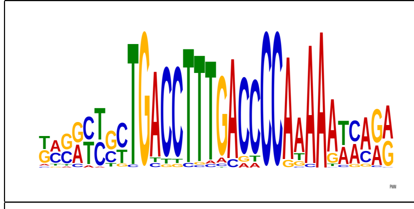	*NNNNNNGCTGNNNNNNNNNNNCCAAAANNNNNN*	51.03	M	7
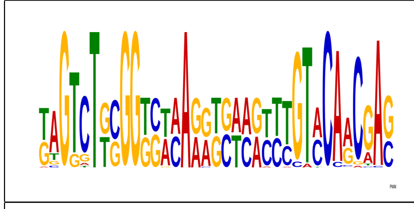	*NAGTCNNNNNNNNNNNNNNNNNNNNNNNNAACGAN*	15.28	M	8
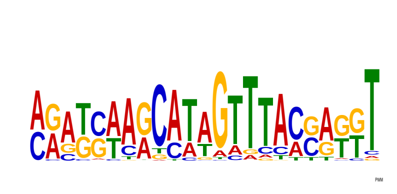	*NNNNNNAACANNGTTTANNNNNN*	16.76	M	9

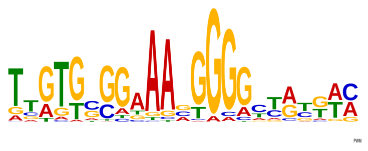	NNNNNNGGAAANNGGGCNNNNNN	29.56	DR:1	10
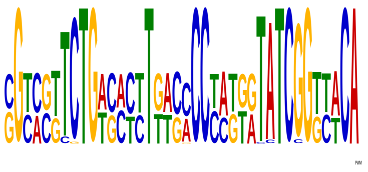	NNNNNNTCTGNNNNNNNNNNNNNNNNNTATCGNNNNNN	31.71	M	11
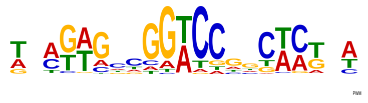	NNNNNNCCGGNCCGGNNNNNN	8.50	M	12
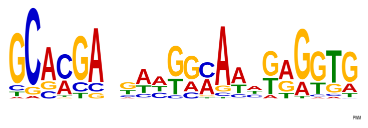	NNNNNNAGATNNAAATNNNNNN	4.19	M	13
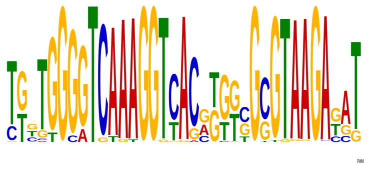	NNNTGGGNNNNNNNNNNNNNNNNNNNGTAAGANNN	62.13	M	14
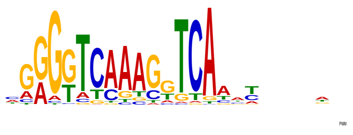	NNNNNNTCAANNNNNAAATNNNNNN	13.08	M	15
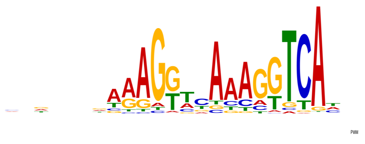	NNNNNNGAAANNNNNAAAGNNNNNN	7.80	M	16
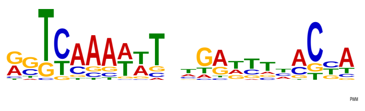	NNNNNNATTTNNGATCNNNNNN	5.15	M	17
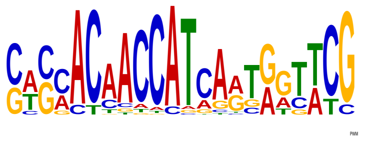	NNNNNNAACCNTCAATNNNNNN	17.99	M	18

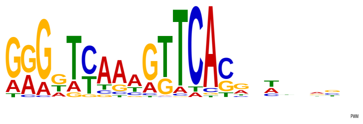	$NNNNNNA\overrightarrow{AAGTNN}ACGGNNNNNN$	10.28	M	19
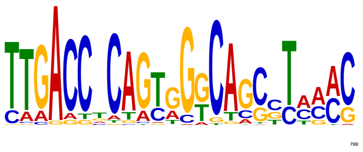	$NNN\overrightarrow{NNNTCA}GTNNNCAGCNNNNNN$	10.46	M	20

372 RXRA-Yin2017-Rnd2 (ERR1005924)

PWM	Seed Sequence	Seed Seq Enrichment	Repeat	Rank
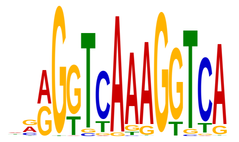	NNNNNNGGTCAAAGGTCANNNNNN	311.35	DR:1	1
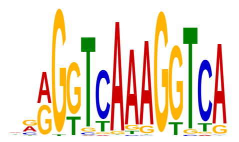	NNNNNNGGTCAAANGTCANNNNNN	131.73	DR:1	2
	NNNNNNGGGGNNNNNGGGGNNNNNN	2.86	M	3
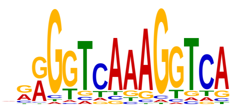	NNNNNNGTCAANNGTCANNNNNN	13.84	DR:1	4
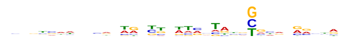	NCGGTNNNNNNNNNNNNNNNNNNNNNNNNNNNNNCTACN	1.73	M	5
	NTTTCNNNNNNNNNNNNNNNNNNNNNNNNNNNNNCGTCN	1.89	M	6
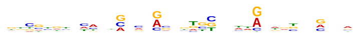	NTCGTNNNNNNNNNNNNNNNNNNNNNNNNNNNNNGCGAN	1.73	M	7
	TGCGNNNNNNNNNNNNNNNNNNNNNNNNNNNNNNNTGCG	1.86	M	8
	NNTCTTNNNNNNNNNNNNNNNNNNNNNNNNNTCTCNN	1.82	M	9

PWM	CGTGNNNNNNNNNNNNNNNNNNNNNNNNNNNNNNACGG	1.76	M	10
PWM	CCTCNNNNNNNNNNNNNNNNNNNNNNNNNNNNNNGCCA	1.85	M	11
PWM	GGGTNNNNNNNNNNNNNNNNNNNNNNNNNNNNNNAGGG	1.93	DR:30	12
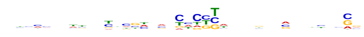 PWM	NTCCCNNNNNNNNNNNNNNNNNNNNNNNNNNNNCTCCN	1.87	M	13
PWM	GCGGNNNNNNNNNNNNNNNNNNNNNNNNNNNNNNGGCG	1.76	M	14
PWM	GAGGNNNNNNNNNNNNNNNNNNNNNNNNNNNNNNAGGG	1.75	DR:28	15
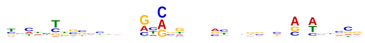 PWM	NCCTCNNNNNNNNNNNNNNNNNNNNNNNNNNNNGTCCN	2.03	ER:29	16
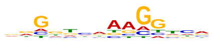 PWM	NNNNNNGTCANNNNTCATNNNNNN	2.44	DR:1	17
PWM	GGGANNNNNNNNNNNNNNNNNNNNNNNNNNNNNNAGGG	1.81	M	18

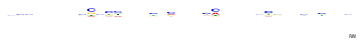	*TCCCNNNNNNNNNNNNNNNNNNNNNNNNNNNNNNNNCCCC*	1.88	M	19
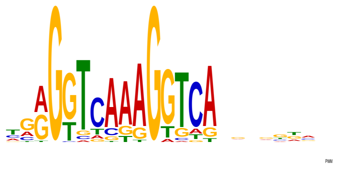	$NNNNN\overrightarrow{NNGTCA}A\overrightarrow{NNGTCA}CGNNNNNN$	19.73	DR:1	20

373 RXRA-Yin2017-Rnd2 (ERR1005925)

PWM	Seed Sequence	Seed Seq Enrichment	Repeat	Rank
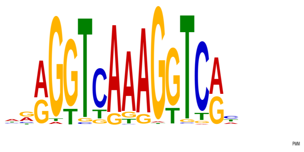	NNNNNNAGGTCAAAGGTCNNNNNN	246.24	DR:1	1
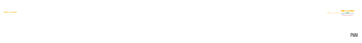	NNNNNNGGGGNNNNNGGGGNNNNNN	3.85	M	2
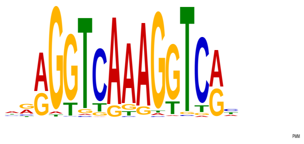	NNNNNNAGGTCNAAGGTCNNNNNN	102.88	DR:1	3
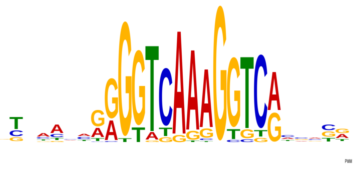	NNNNNNCGGGGTCNAAGGTCNNNNNN	173.04	DR:1	4
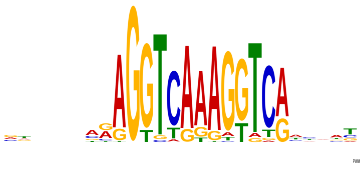	NNNNNNAAAGGTCNNAGGTCNNNNNN	91.28	DR:1	5
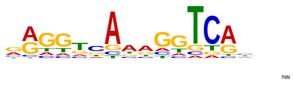	NNNNNNGGTCNAAGGNNNNNN	4.46	DR:1	6
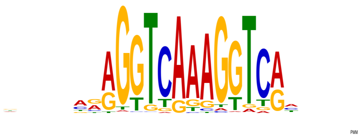	NNNNNNAAGGTCNNAGGTCNNNNNN	50.11	DR:1	7
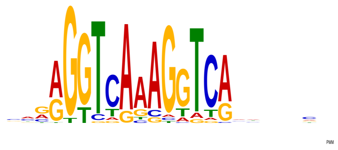	NNNNNNGGTCNAAGGTCAGNNNNNN	50.37	DR:1	8
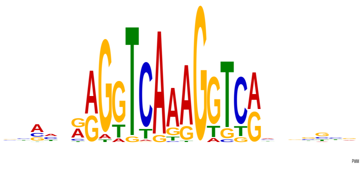	NNNNNNGGGTCNAAGGTCATNNNNNN	134.38	DR:1	9

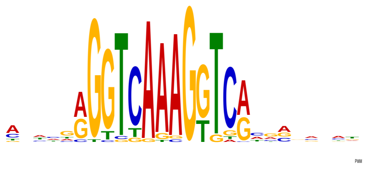	$NNNNN\overrightarrow{NGGTCA}A\overrightarrow{AGNTCA}CGNNNNNN$	199.41	DR:1	10
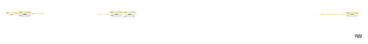	*NNNNNNCGGGNNNNNNNGGGGNNNNNN*	3.25	M	11
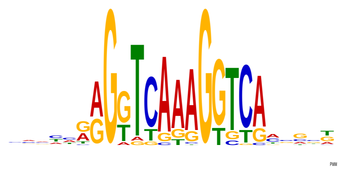	$NNNNNNTG\overrightarrow{GGGTCA}N\overrightarrow{AGGTCN}NNNNN$	128.65	DR:1	12
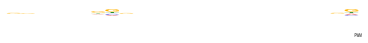	*NNNNNNGGGANNNNNGGGGNNNNNN*	3.23	M	13

374 RXRA-Yin2017-Rnd3 (ERR1005926)

PWM	Seed Sequence	Seed Seq Enrichment	Repeat	Rank
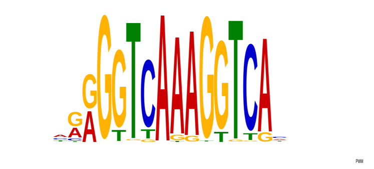 PWM	NNNNNNGGTCAAANGTCANNNNNN	582.25	DR:1	1
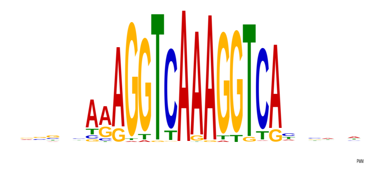 PWM	NNNNNNAAAGGTCNNAGGTCANNNNNN	1570.13	DR:1	2
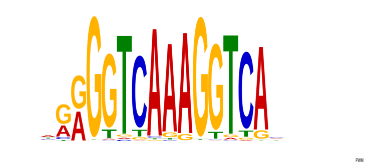 PWM	NNNNNNNGTCAANNGTCANNNNNN	77.03	DR:1	3
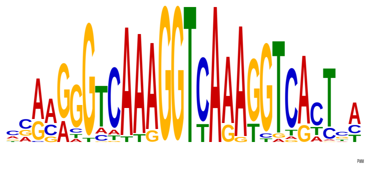 PWM	NNNNNGGTCAAAGNNNAAAGGTCNNNNN	3259.90	M	4
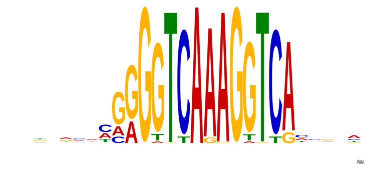 PWM	NNNNNNACGGGGTCNNAGGTCNNNNNN	642.28	DR:1	5
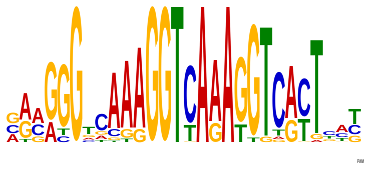 PWM	NNNNNNTCAAAGNNCAAAGGTCNNNNNN	1505.86	M	6
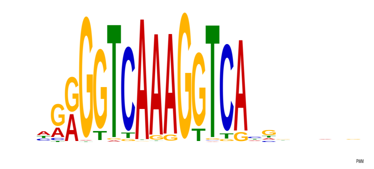 PWM	NNNNNNGTCAAANNTCACGNNNNNN	164.75	DR:1	7
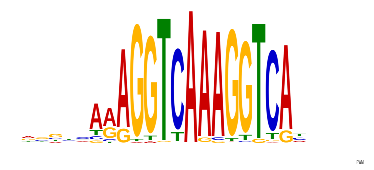 PWM	NNNNNNAAAGGTCNNAGGTCNNNNNN	563.52	DR:1	8
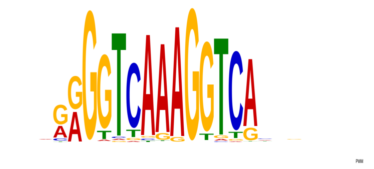 PWM	NNNNNNNGTCAANNGTCATNNNNNN	79.78	DR:1	9

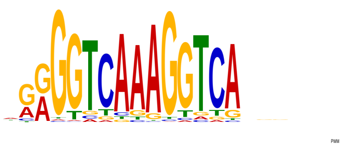	$NNN\overrightarrow{NNNTCA}A\overrightarrow{NNGTCA}NNNNNNN$	25.01	DR:1	10

375 RXRA-Yin2017-Rnd3 (ERR1005927)

PWM	Seed Sequence	Seed Seq Enrichment	Repeat	Rank
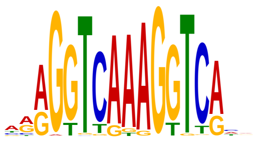	NNNNNNAGGTCNAAGGTCNNNNNN	580.32	DR:1	1
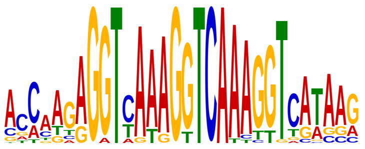	NNNNNNAGGTCAAANNTCAAAGGTNNNNNN	8732.88	M	2
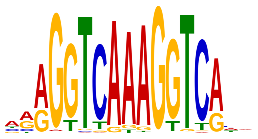	NNNNNNAGGTCNNAGGTCNNNNNN	223.50	DR:1	3
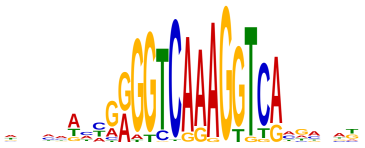	NNNNNNACGGGGTCNNAGGTCANNNNNN	1106.18	DR:1	4
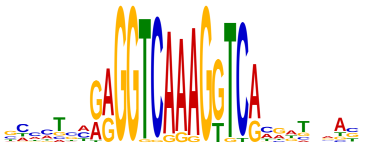	NNNNNNAGGGGTCNNAGGTCACGNNNNNN	2298.98	DR:1	5
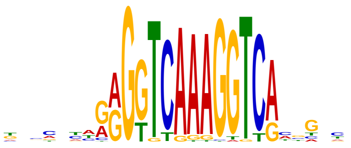	NNNNNNTAGGGGTCNNAGGTCNNNNNN	493.74	DR:1	6
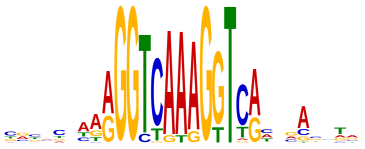	NNNNNNAAAGGTCNNAGGTCATGNNNNNN	2024.69	DR:1	7
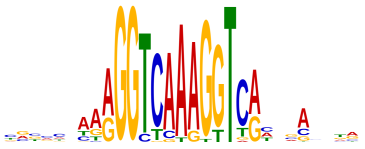	NNNNNNAAAGGTCNNNGGTCATGNNNNNN	665.78	DR:1	8
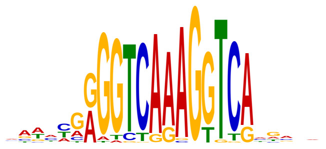	NNNNNNACGGGGTCNNAGGTCNNNNNN	470.86	DR:1	9

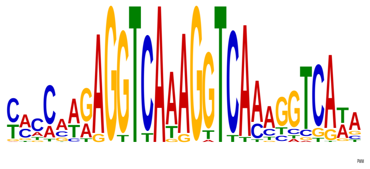	NNNNNNG$\overrightarrow{AGGTCA}$N$\overrightarrow{NGGTCA}$AAGNNNNNN	3332.23	DR:1	10
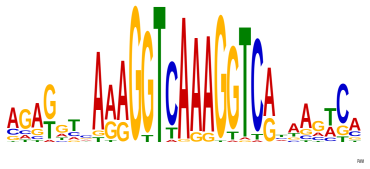	NNNNNNCAA$\overrightarrow{AGGTCN}$N$\overrightarrow{NGGTCA}$TNNNNNN	1229.26	DR:1	11
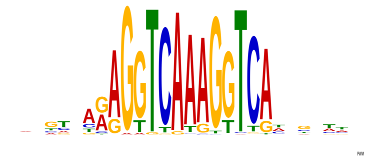	NNNNNNAA$\overrightarrow{AGGTCN}$N$\overrightarrow{NGGTCA}$ANNNNNN	707.21	DR:1	12
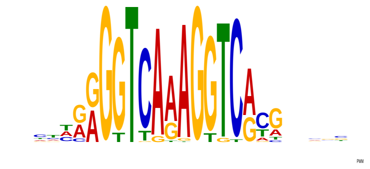	NNNNNN$\overrightarrow{GGGTCA}$N$\overrightarrow{AGGTCG}$CGNNNNNN	1040.79	DR:1	13
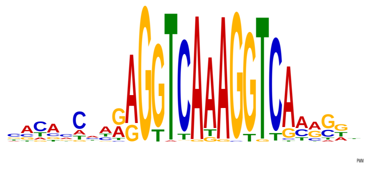	NNNNNNAAG$\overrightarrow{AGGTCN}$N$\overrightarrow{AGGTCN}$NNNNN	935.25	DR:1	14
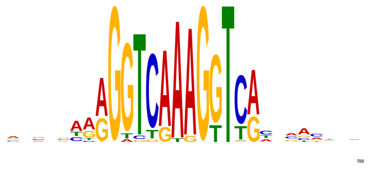	NNNNNNA$\overrightarrow{AGGTCN}$N$\overrightarrow{AGGTCA}$TGNNNNNN	998.69	DR:1	15
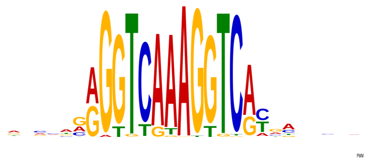	NNNNNN$\overrightarrow{AGGTCN}$N$\overrightarrow{AGGTCG}$CGNNNNNN	404.15	DR:1	16
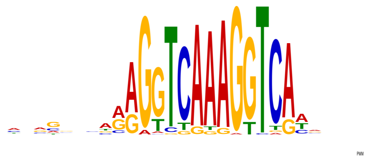	NNNNNNTAA$\overrightarrow{AGGTCN}$N$\overrightarrow{AGGTCN}$NNNNN	618.00	DR:1	17
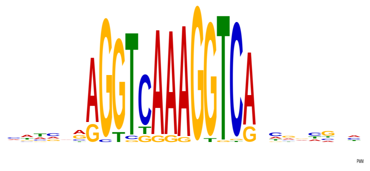	NNNNNN$\overrightarrow{AGGTCA}$A$\overrightarrow{NGGTCA}$GCNNNNNN	1026.11	DR:1	18

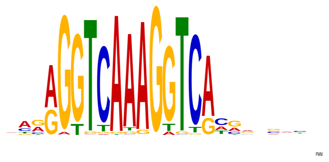	$NNNNNN\overrightarrow{AGGTCN}NN\overrightarrow{NGGTCA}CGNNNNNNN$	306.08	DR:1	19
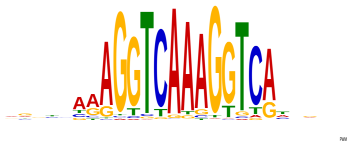	$NNNNNNNAA\overrightarrow{AGGNNN}N\overrightarrow{AGGTCN}NNNNN$	59.12	DR:1	20

376 RXRA-Yin2017-Rnd4 (ERR1005928)

PWM	Seed Sequence	Seed Seq Enrichment	Repeat	Rank
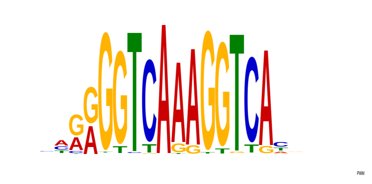	NNNNNNGGTCANNGGTCANNNNNNN	442.08	DR:1	1
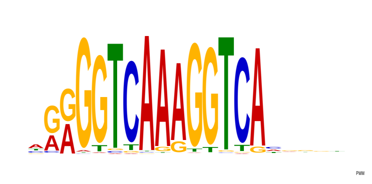	NNNNNNTCAANNGTCANNNNNNN	88.94	DR:1	2
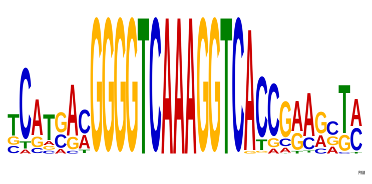	NNNNNNCGGGGTCNNNGGTCACCGNNNNNN	7086.75	DR:1	3
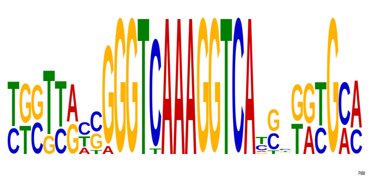	NNNNNNCGGGGTCNNNGGTCACGCNNNNNN	2141.99	DR:1	4
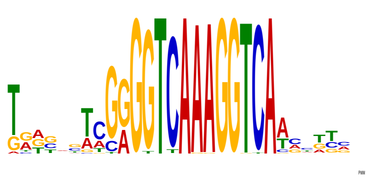	NNNNNNTCGGGGTCNNNNNTCAANNNNNN	557.80	DR:1	5
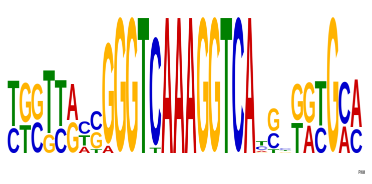	NNNNNNCGGGGTCNNNNGTCACGCNNNNNN	715.68	DR:1	6
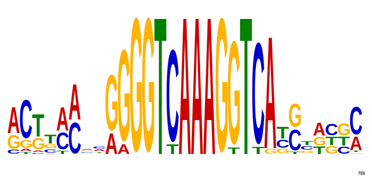	NNNNNNCCGGGGTCNNNGGTCACNNNNNN	1772.84	DR:1	7
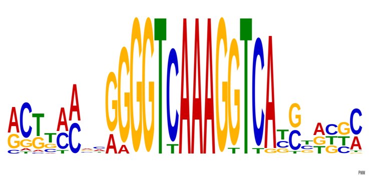	NNNNNNCCGGGGTCNNNNGTCACNNNNNN	561.77	DR:1	8
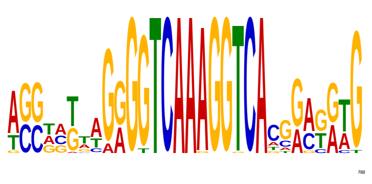	NNNNNNTAGGGGTCNNNNNTCACGNNNNNN	733.22	DR:1	9

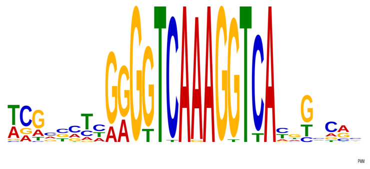	$NNNNNNTTG\overrightarrow{GGGTCN}NN\overrightarrow{NNGTCA}TNNNNNN$	694.46	DR:1	10
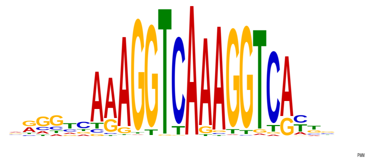	$NNNNNNAAAGNNNNN\overrightarrow{AGGTCN}NNNNN$	73.27	M	11
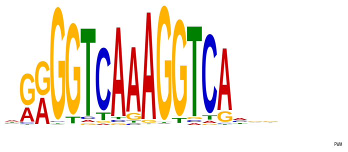	$NNN\overrightarrow{NNNTCA}A\overrightarrow{NNNTCA}TNNNNNN$	29.99	DR:1	12
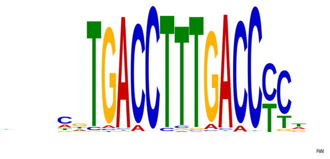	$NNNNN\overrightarrow{NCGTGA}NNNT\overleftarrow{TGANNN}NNNN$	70.03	IR:4	13

377 RXRA-Yin2017-Rnd4 (ERR1005929)

PWM	Seed Sequence	Seed Seq Enrichment	Repeat	Rank
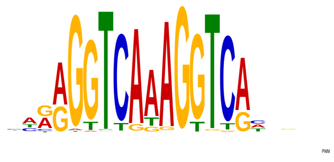	NNNNNNGGTCNNAGGTCNNNNNN	213.50	DR:1	1
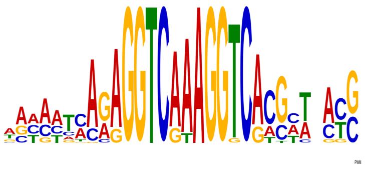	NNNNNNAAGAGGTCNNNGGTCACGNNNNNN	5306.54	DR:1	2
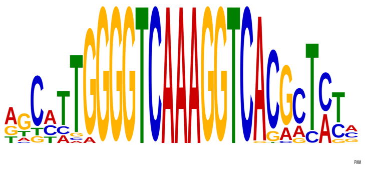	NNNNTTGGGGTCNNNGGTCACGCNNNN	8821.12	DR:1	3
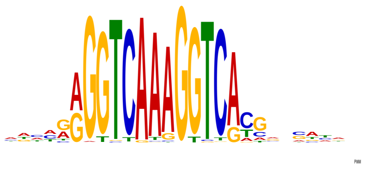	NNNNNNGGTCNNNGGTCACGANNNNNN	643.24	DR:1	4
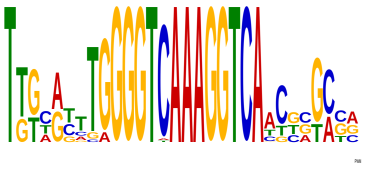	NNNNNNTTGGGGTCNNNGGTCAACNNNNNN	2965.01	DR:1	5
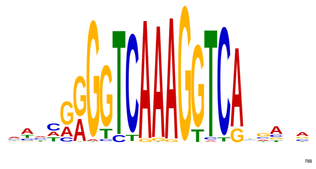	NNNNNNACGGGGTCNNNGGTCNNNNNN	375.98	DR:1	6
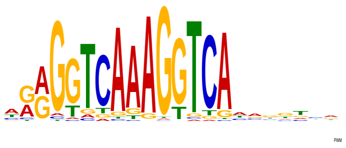	NNNNNNTCAANNNTCAANNNNNNN	33.19	DR:1	7
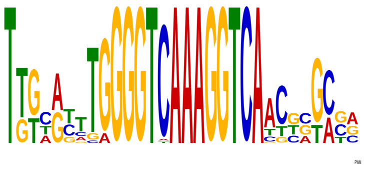	NNNNNNTTGGGGTCNNNNGTCAACNNNNNN	1354.82	DR:1	8
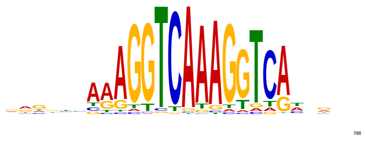	NNNNNNAAAGNNNNNNGGTCNNNNNN	23.46	M	9

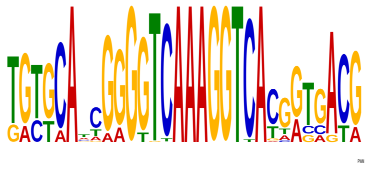	$NNNNNNTCG\overrightarrow{GGGTCN}NN\overrightarrow{NGGTCA}CGNNNNNN$	5014.34	DR:1	10
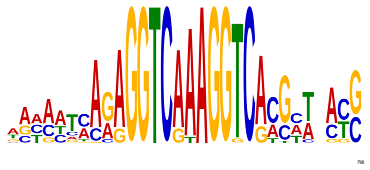	$NNNNNNAAG\overrightarrow{AGGTCN}NN\overrightarrow{NNGTCA}CGNNNNNN$	1443.40	DR:1	11
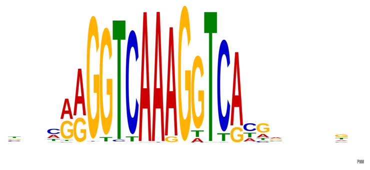	$NNNNN\overrightarrow{NGGTCN}NN\overrightarrow{NGGTCA}CGCNNNNNN$	387.64	DR:1	12
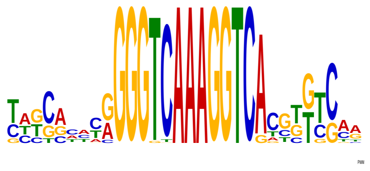	$NNNNNNATG\overrightarrow{GGGTCN}NN\overrightarrow{NGGTCA}CGNNNNNN$	2281.12	DR:1	13
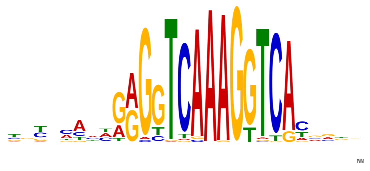	$NNNNNNTCG\overrightarrow{GGGTCN}NN\overrightarrow{NGGTCN}NNNNN$	313.17	DR:1	14
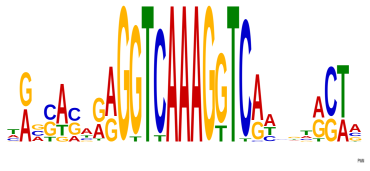	$NNNNNNAG\overrightarrow{AGGTCN}NN\overrightarrow{NNGTCA}TCNNNNNN$	522.15	DR:1	15

378 RXRA-SMiLE-seq-Rnd1 (SRR3405082)

PWM	Seed Sequence	Seed Seq Enrichment	Repeat	Rank
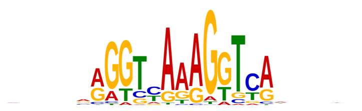	NNNNNNAGGTNAAAGGTCANNNNNN	660.98	DR:1	1
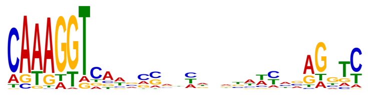	CAAAGGTNNNNNNNNNNNNNNNNNNNGGTC	216.43	DR:16	2
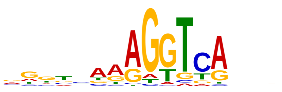	NNNNNNAAAGGTCANNNNNN	28.09	M	3
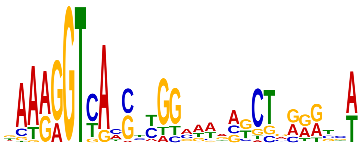	NAAAGGTCANNNNNNNNNNNNNNNNGGTCN	248.86	DR:15	4
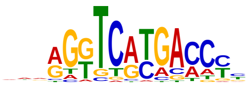	NNNNNNAGGTCATGACCNNNNNN	117.11	IR:0	5
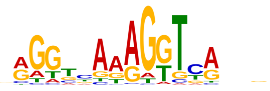	NNNNNNTCAAAGGTCANNNNNN	85.96	DR:1	6
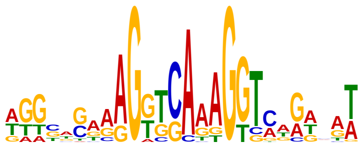	NNNNNNAAAGTTCAAAGGTCNNNNNN	879.72	DR:1	7
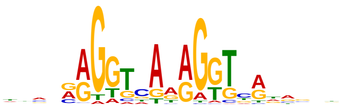	NNNNNNGTTCAAAGGTCANNNNNN	205.28	DR:1	8
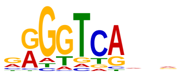	NNNNNNGGGGTCATNNNNNN	11.86	M	9

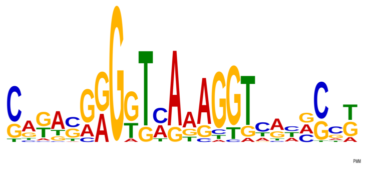	*NNNNNGGGTTCAAAGGTCANNNNNN*	706.02	DR:1	10
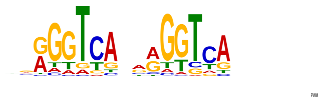	*NNNNNNGGTCANNNGGTCANNNNNN*	60.21	DR:2	11
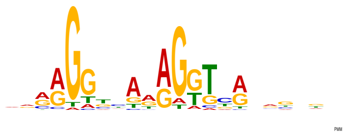	*NNNNNNNTTCAAAGGTCANNNNNN*	61.93	DR:1	12
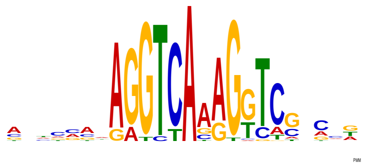	*NNNNNATAGGTCAAAGGTCNNNNN*	747.67	DR:1	13
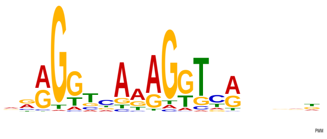	*NNNNNNNTTCAAAGGTCNNNNNN*	31.51	DR:1	14
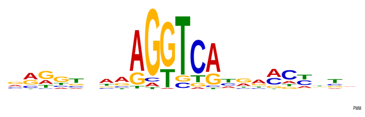	*NNNNNNAAAGGTCATGANNNNNN*	63.54	IR:0	15
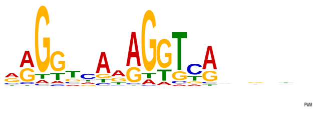	*NNNNNNNTTCANAGGTCANNNNNN*	36.58	DR:1	16
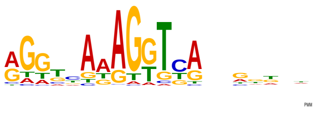	*NNNNNNNTCAAAGGTCAANNNNNN*	106.17	DR:1	17
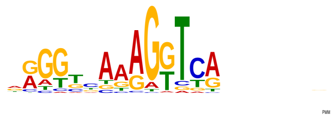	*NNNNNNNTCAAAGGTCATNNNNNN*	98.00	DR:1	18

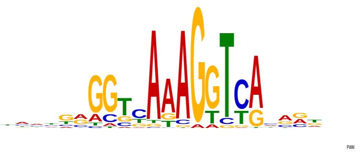	$NNNNN\overrightarrow{NAGTC}AA\overrightarrow{AGGTC}ANNNNNN$	140.46	DR:1	19
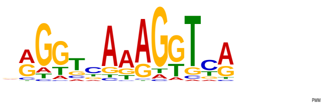	$NNN\overrightarrow{NNNTC}AA\overrightarrow{AGGTC}NNNNNN$	36.42	DR:1	20

379 RXRB-17-Bhimsaria2022-Rnd3 (S07-136)

PWM	Seed Sequence	Seed Seq Enrichment	Repeat	Rank
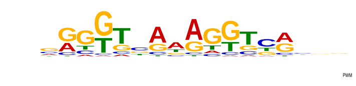	NNNNGGTCANNGGTCNNNN	20.42	DR:1	1
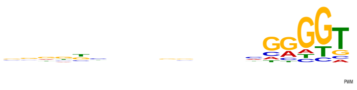	GGGGNNNNNNNNNNNGGGGT	24.14	M	2
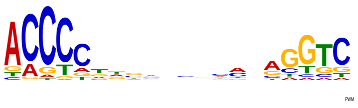	ACCCCNNNNNNNNNNAGGTC	46.57	ER:11	3
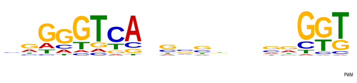	NGGGGTCNNNNNNNNGGGTN	22.07	DR:7	4
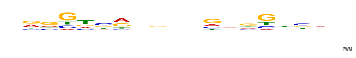	NNGGTCNNNNNNNGGTCNN	5.69	DR:5	5
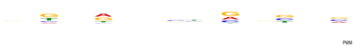	NGGTCNNNNNNNNNGGTCN	3.93	DR:7	6
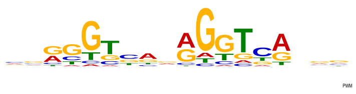	NNNNGTCAGGGGTCNNNN	19.58	DR:1	7
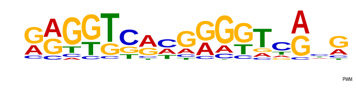	NNNGGTCANGGGGTCNNN	30.01	DR:2	8
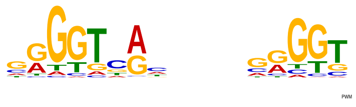	GGGGTCNNNNNNGGGGT	46.64	DR:6	9

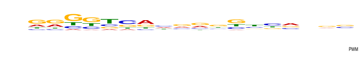	*NNN*$\overrightarrow{NGTCA}$*NNN*$\overrightarrow{NGGTC}$*NNNN*	6.22	DR:2	10
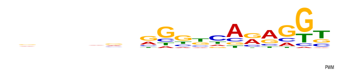	*GGGGNNNNNNNNNNNNA*$\overrightarrow{AGGT}$	11.33	M	11
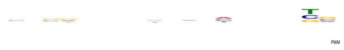	*CCCGNNNNNNNNNNNN*$\overrightarrow{NGGTG}$	5.19	M	12
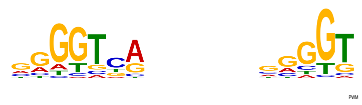	*G*$\overrightarrow{GGGTC}$*NNNNNNNG*$\overrightarrow{GGGT}$	57.11	DR:7	13
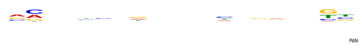	$\overleftarrow{NACCC}$*NNNNNNNNNN*$\overrightarrow{GGGTN}$	4.76	ER:10	14
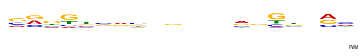	*NN*$\overrightarrow{GGTCN}$*NNNNNNN*$\overrightarrow{NGGTCN}$*N*	5.06	DR:6	15
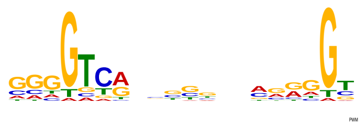	*G*$\overrightarrow{GGGTC}$*NNNNNNNNNNA*$\overrightarrow{GGT}$	30.53	DR:9	16
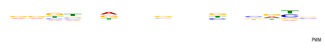	*NN*$\overrightarrow{NGGTC}$*NNNN*$\overrightarrow{NGGTC}$*NNN*	3.81	DR:3	17
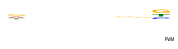	$\overrightarrow{GTC}$*GNNNNNNNNNNNNGGGG*	5.55	M	18

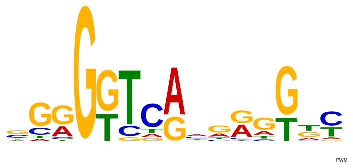	$NG\overrightarrow{GGGTCA}CN\overrightarrow{GGGTN}$	48.75	DR:2	19
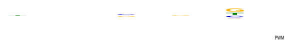	$GGGGNNNNNNNNNN\overrightarrow{NGGTC}$	4.10	M	20

380 RXRB-Bhimsaria2022-Rnd3 (S07-171)

PWM	Seed Sequence	Seed Seq Enrichment	Repeat	Rank
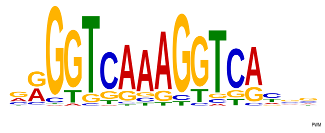	NNNNGGTCAAAGGTCNNNN	109.43	DR:1	1
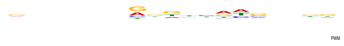	NNNNNNCGGGGTCANNNNNN	4.17	M	2
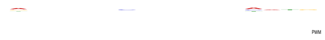	CGAANNNNNNNNNNNAATG	4.29	M	3
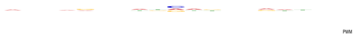	ATTANNNNNNNNNNNATAT	3.78	M	4
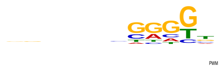	GGGGNNNNNNNNNNNGGGGT	9.44	M	5
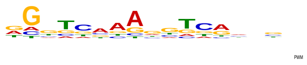	NNNNNGTCANNGGTCNNNNN	7.00	DR:1	6
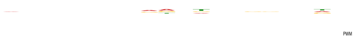	ACAANNNNNNNNNNNNTCTT	3.94	M	7
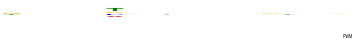	GTCGNNNNNNNNNNNNTGTG	3.93	M	8
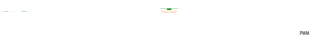	GTTTNNNNNNNNNNNCGTT	3.63	M	9

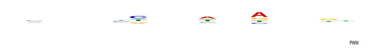	CCATNNNNNNNNNNNNAGGT	3.83	M	10
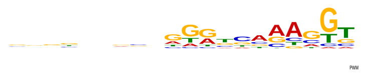	GGGGNNNNNNNNNNNNAGGT	8.47	M	11
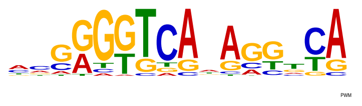	NNGGGGTCAAGGGTNN	28.62	DR:1	12
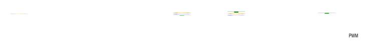	GTCTNNNNNNNNNNNTATG	3.80	M	13
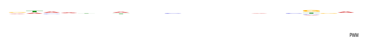	GTAANNNNNNNNNNNNCGGA	3.61	M	14
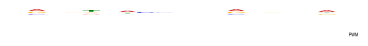	NNGGTCNNNNNNNGGTCNN	2.98	DR:5	15
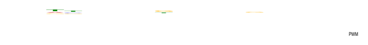	CCTTNNNNNNNNNNNGTTT	3.83	M	16
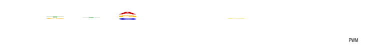	ATGTNNNNNNNNNNNTCTT	3.65	M	17
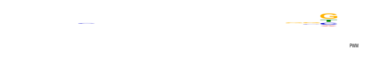	AGTCNNNNNNNNNNNNGGGT	3.89	DR:11	18

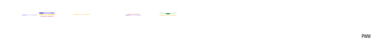	*CCCGNNNNNNNNNNNNCTTG*	3.38	M	19
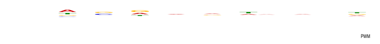	$ATATNNNNNNNNNNN\overrightarrow{AGCT}$	3.24	M	20

381 RXRB-Jolma2013-Rnd2 (ERR195462)

PWM	Seed Sequence	Seed Seq Enrichment	Repeat	Rank
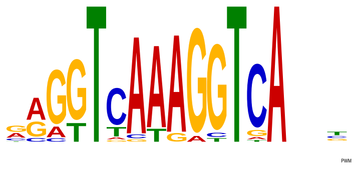	NNNGTCANAGGTCANNN	116.79	DR:1	1
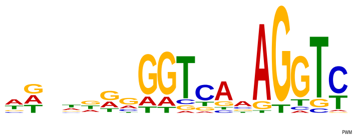	NNNNNGGGGTCAANNNNN	5.76	M	2
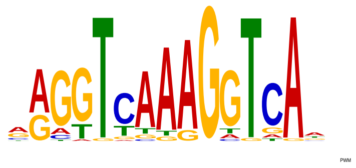	NNGGTCANNNGTCANN	29.79	DR:1	3
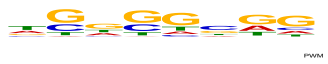	TGGGGCGG	2.56	M	4
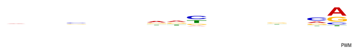	NNGGGGNNNNNGGGGNN	1.74	M	5
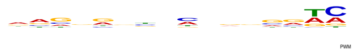	NNNGGGTNNCGGGNNN	2.03	M	6
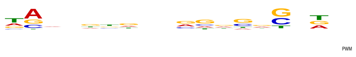	NNNNGTGTNNGGGGNNNN	2.04	M	7
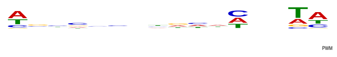	NNNCCCCNNNCACTNNN	1.84	M	8
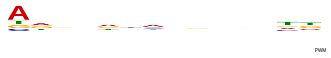	NNNGTGTNTGGTNNN	1.49	M	9

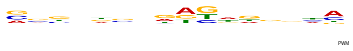	$NNGTTGNNNNTGGGNN$	1.64	M	10
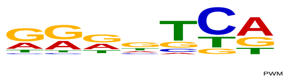	$AG\overrightarrow{GGGTCA}$	3.77	M	11
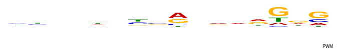	$NNN\overrightarrow{AGGGNN}NNGGGGNNN$	1.27	M	12
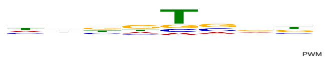	$TTGG\overrightarrow{TGGT}$	1.79	M	13
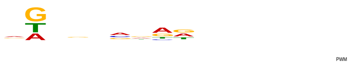	$NNNTGGGNNTGGGNNN$	1.23	M	14
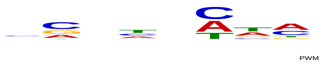	$\overrightarrow{CCTCA}CAC$	1.73	M	15
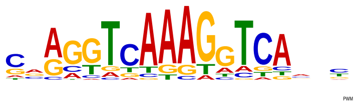	$NN\overrightarrow{NGGTCN}NN\overrightarrow{NGTCA}NNN$	8.15	DR:1	16
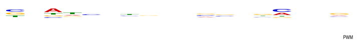	$NNNNNGTGGGGGGNNNNN$	1.76	M	17
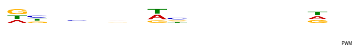	$NNGTGGNNNNNNGGGGNN$	1.38	M	18

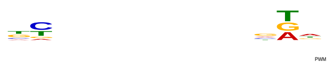	$NNN\overrightarrow{GGGTNG}GCGNNN$	1.09	M	19
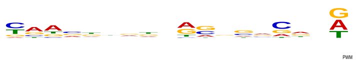	$NNNNNTGTGGGGGNNNNN$	1.85	M	20

382 Rxrb-Jolma2013-Rnd3 (ERR194859)

PWM	Seed Sequence	Seed Seq Enrichment	Repeat	Rank
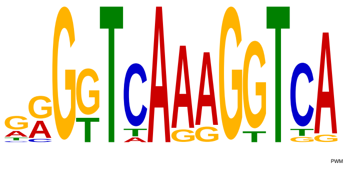	NGGGTCAAAGGTCN	516.00	DR:1	1
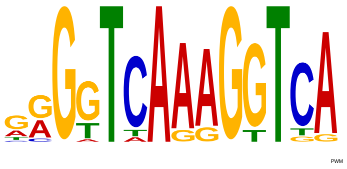	NGGGTCNAAGGTCN	205.32	DR:1	2
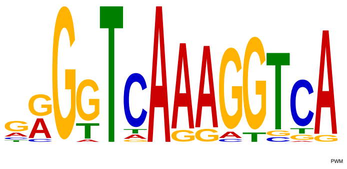	NGGGTCNNAGGTCN	67.55	DR:1	3
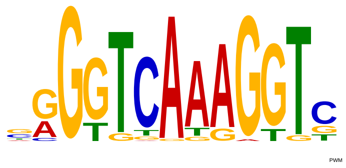	GGGGTCNNAGGTC	200.45	DR:1	4
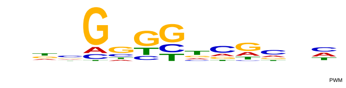	NNCGGGNTCGCNN	4.09	M	5
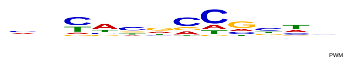	NNCACGCCGCNN	3.22	M	6
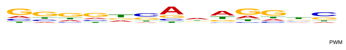	GGGGNNNNNGGTG	3.90	M	7
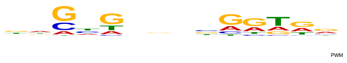	NAGTGNNNNGGTGN	3.07	M	8
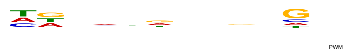	NNGATACGGCNN	2.01	M	9

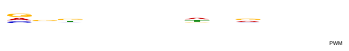	NGGGANNNGGAGN	2.07	M	10
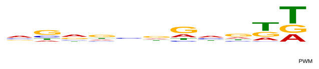	NNG$\overrightarrow{AGCGGA}$GNN	2.30	M	11
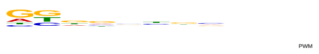	NGGGGNNGCTGN	2.10	M	12
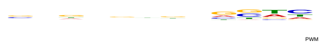	NNAGTGNG$\overrightarrow{AGGN}$N	2.16	M	13
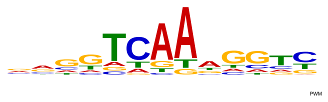	NA$\overrightarrow{AGGNNN}$N$\overrightarrow{AGGTN}$	4.52	DR:1	14
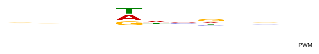	$\overrightarrow{AGGGNN}$NNGAGT	2.21	M	15
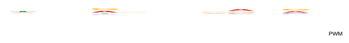	NGT$\overrightarrow{AGNNGA}$AGN	1.80	M	16
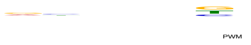	GTGTTTGG	1.54	M	17
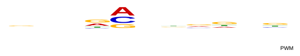	N$\overrightarrow{AGGANN}$N$\overrightarrow{GGGTN}$	1.94	DR:1	18

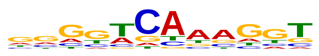 PWM	$CGGGNNNNN\overrightarrow{AGGT}$	4.37	M	19
PWM	$NTGGGNNC\overrightarrow{AGGN}$	1.65	M	20

383 RXRB-Jolma2013-Rnd3 (ERR195463)

PWM	Seed Sequence	Seed Seq Enrichment	Repeat	Rank
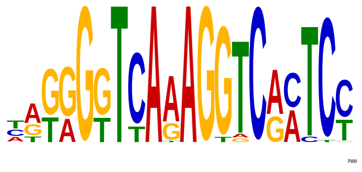	*NNNNGGTCAAAGTTCANNNN*	3390.63	DR:1	1
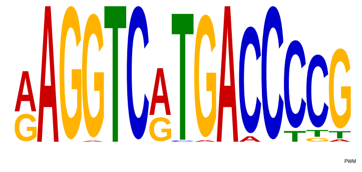	*NAGGTCATGACCCCN*	4727.26	IR:0	2
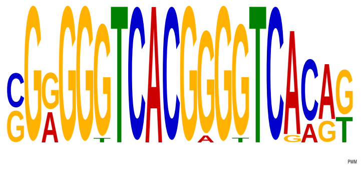	*NNNGGGTCACGGGGTCANNN*	29872.12	DR:2	3
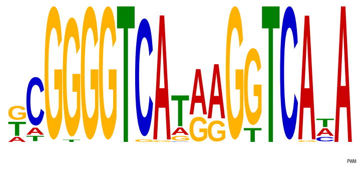	*NNGGGGTCANNAGGTCANN*	6596.64	DR:2	4
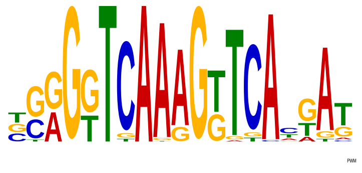	*NNNGGTCAAAGNTCAGNNN*	4473.20	DR:1	5
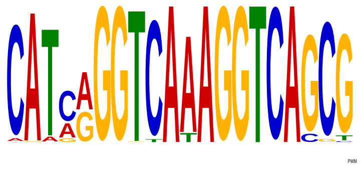	*NNNAAGGTNAAAGGTCANNN*	22770.14	DR:1	6
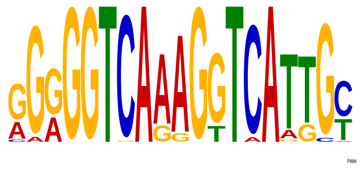	*NNNGGTCAGANGTCATNNN*	2203.20	DR:1	7
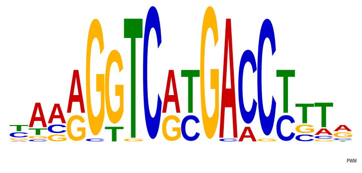	*NNNNGGTCATGACCNNNN*	164.26	IR:0	8
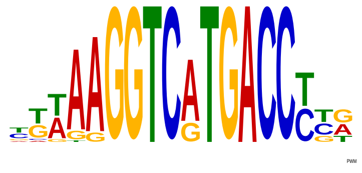	*NNNAAGGTCATGACCNNN*	2791.72	IR:0	9

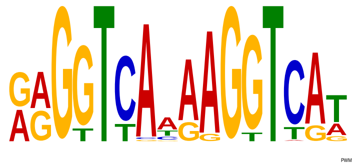	$NN\overrightarrow{GGTCA}AA\overrightarrow{AGGTCN}N$	1331.88	DR:2	10
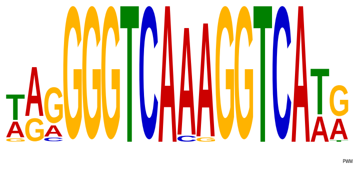	$NNA\overrightarrow{GGGTCA}N\overrightarrow{GGGTCA}NN$	2581.41	DR:1	11
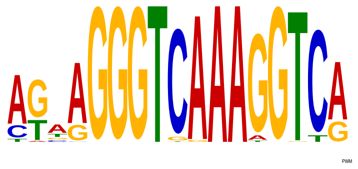	$NNCA\overrightarrow{GGGTCA}A\overrightarrow{AGGTNN}$	4292.48	DR:1	12
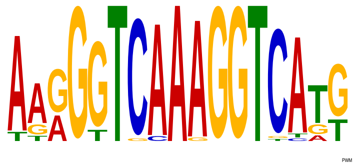	$NNA\overrightarrow{GGTCA}A\overrightarrow{ANGTTA}NN$	928.27	DR:1	13
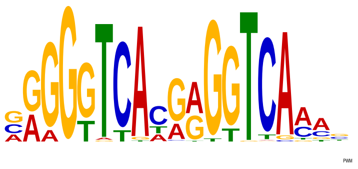	$NN\overrightarrow{NGGTCA}NN\overrightarrow{NNGTCA}NNN$	75.81	DR:2	14
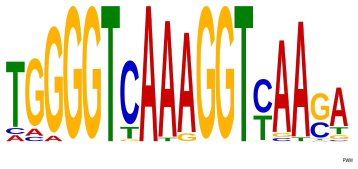	$NN\overrightarrow{GGGTNN}A\overrightarrow{AGGTTA}NN$	2323.02	DR:1	15
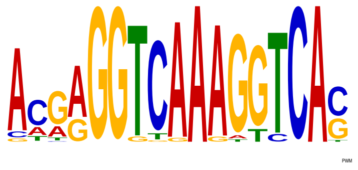	$NNA\overrightarrow{AGGTTA}N\overrightarrow{NGGTCN}N$	256.96	DR:1	16
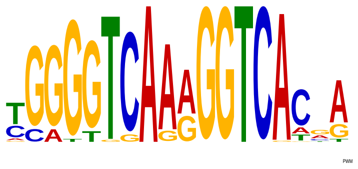	$NN\overrightarrow{NGGTCA}G\overrightarrow{GNGTCA}NNN$	316.26	DR:1	17
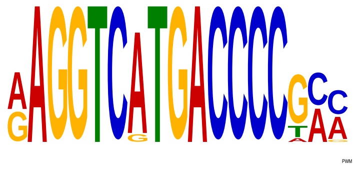	$N\overrightarrow{NGGTCA}\overleftarrow{TGNNCC}CGNN$	482.85	IR:0	18

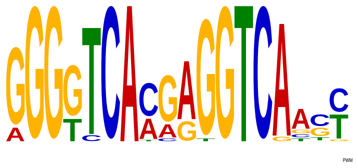	$N\overrightarrow{NGGTCA}NN\overrightarrow{NGGTCA}GNN$	247.71	DR:2	19
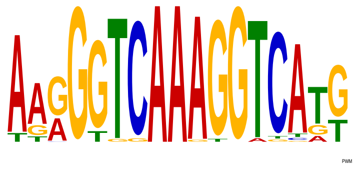	$NN\overrightarrow{AGGTCN}N\overrightarrow{AGGTTA}NN$	285.07	DR:1	20

384 Rxrb-Jolma2013-Rnd4 (ERR194860)

PWM	Seed Sequence	Seed Seq Enrichment	Repeat	Rank
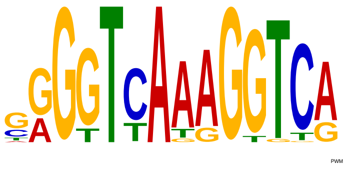	NAGGTCNNAGGTCN	280.18	DR:1	1
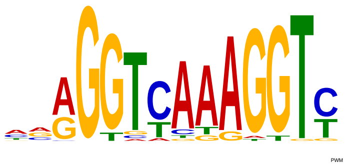	NAAGGTNNNAGGTN	54.29	DR:1	2
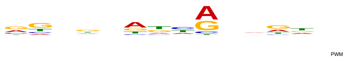	NGGGGNNNNGGGGN	3.20	M	3
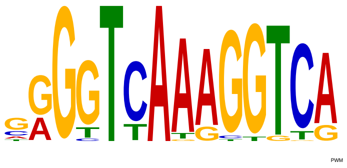	NAGGTCNNNGGTCN	106.79	DR:1	4
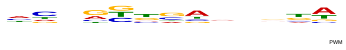	NNAGGGNAAGGNN	2.12	DR:0	5
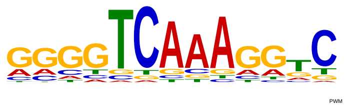	GGGGNNNNNGGTT	7.36	M	6
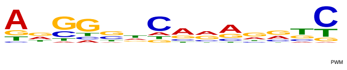	NNGGGTNNGGGGNN	4.08	M	7
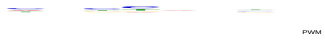	TTTTTTTT	1.40	M	8
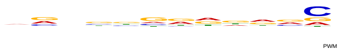	NNGGGGAGGANN	2.23	M	9

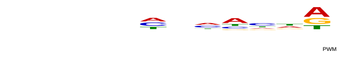	$NNC\overrightarrow{CGAACA}CNN$	1.70	M	10
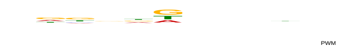	$NGTGTN\overrightarrow{AGGGN}$	1.60	M	11
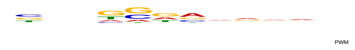	$NN\overrightarrow{AGGGAA}AANN$	1.95	M	12
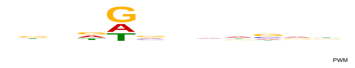	$N\overrightarrow{AGGGNA}AGAN$	2.49	M	13
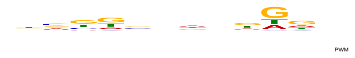	$NNGGGT\overrightarrow{AGGGNN}$	1.84	M	14

385 RXRB-Jolma2013-Rnd4 (ERR195464)

PWM	Seed Sequence	Seed Seq Enrichment	Repeat	Rank
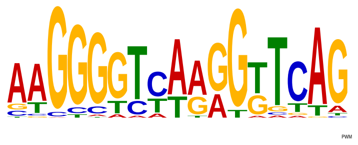	AAGGGNNNNNNNNTCAG	16.70	DR:3	1
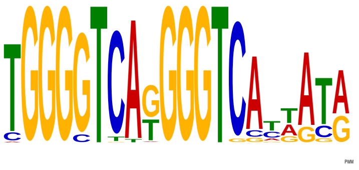	NNNNGTCAGNNNTCACNNNN	41.50	DR:1	2
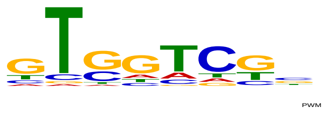	GTGGTCGC	9.79	M	3
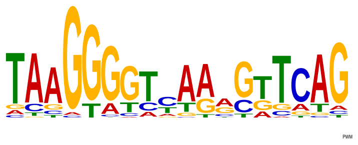	TAAGNNNNNNNNNNTCAG	7.70	M	4
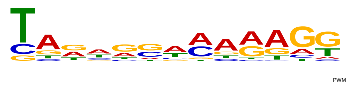	NAGAGNNNAGAGN	5.77	M	5
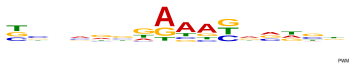	NNNGTGTNNAGGGNNN	3.55	M	6
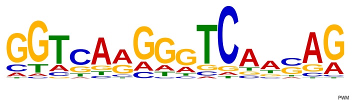	NNTCAGNNNNCAACNN	5.62	M	7
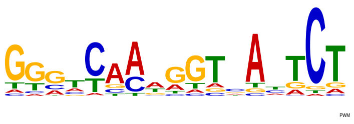	NNGTTANNNNNCATANN	3.87	M	8
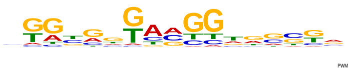	NNNTGGGNNGGTGGNNN	7.02	M	9

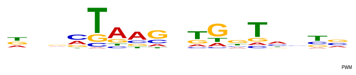	NNNNTAAGNTGGTNNNN	4.63	M	10
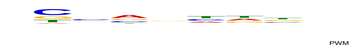	TAGGTGGT	1.63	M	11
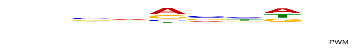	GTAGGTGG	2.23	M	12
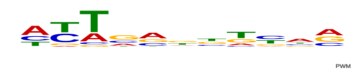	NTAGGNTTCAN	1.91	M	13
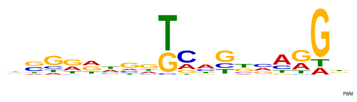	NNNNGTGGNNAGGGNNNN	5.61	M	14
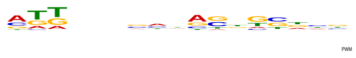	NNNGCGGNNNGTGGNNN	2.63	M	15
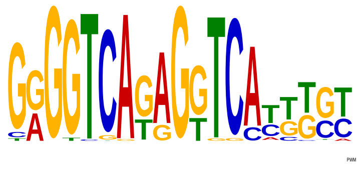	NNNNTCAGANNTCATNNNN	39.45	DR:1	16
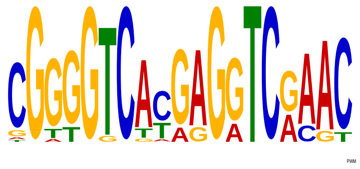	NNNNGTCATGNGGTCNNNN	30.73	DR:2	17
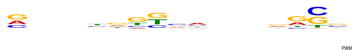	NNNGTGGNNNGGAGNNN	3.35	M	18

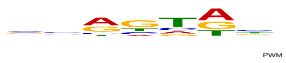	$TT\overrightarrow{AGGTGG}$	2.92	M	19
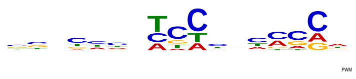	*NNCCCCNNNNCTCCCNN*	3.54	M	20

386 RXRB-Yin2017-Rnd2 (ERR1005932)

PWM	Seed Sequence	Seed Seq Enrichment	Repeat	Rank
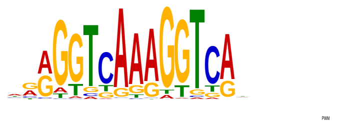	NNNNNNGTCAANNGTCANNNNNNN	73.08	DR:1	1
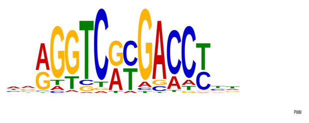	NNNNNNAGGTCNNGACCTNNNNNN	40.70	IR:0	2
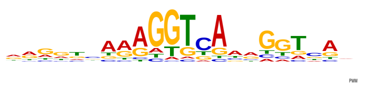	NNNNNNAAAGNNCAAANNNNNN	9.98	M	3
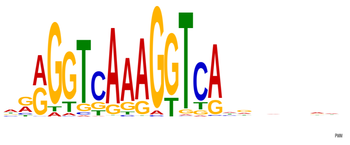	NNNNNNNTCAAANNTCAAGNNNNNN	59.26	DR:1	4
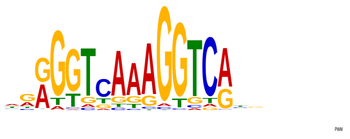	NNNNNNNTCAAANNTCGTNNNNNN	20.88	DR:1	5
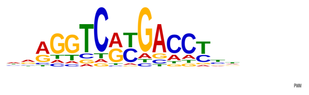	NNNNNNAGGTNNNNACCTNNNNNN	6.46	IR:0	6
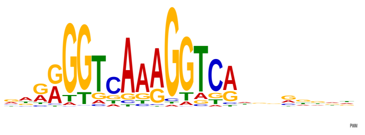	NNNNNNNTCAANNNTCATANNNNNN	14.25	DR:1	7
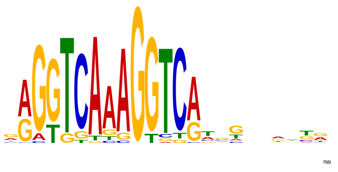	NNNNNNNTCAANNTCATCGNNNNNN	68.04	DR:1	8
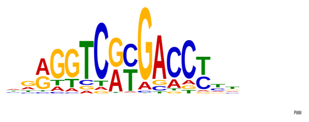	NNNNNNGGGTCNNNACCTNNNNNN	12.39	IR:0	9

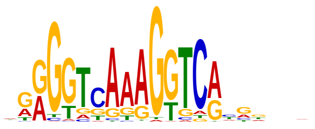	$NNN\overrightarrow{NNNTCA}A\overrightarrow{ANNTCG}CGNNNNNN$	29.72	DR:1	10

387 RXRB-Yin2017-Rnd2 (ERR1005933)

PWM	Seed Sequence	Seed Seq Enrichment	Repeat	Rank
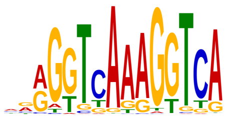	NNNNNNGGTCAANNGTCANNNNNN	189.60	DR:1	1
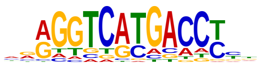	NNNNNNGGTCNNGACCNNNNNN	13.49	IR:0	2
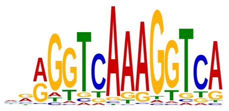	NNNNNNGGTCNNNNGTCANNNNNN	28.07	DR:1	3
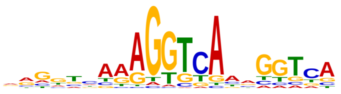	NNNNNNAAAGGNCAAANNNNNN	22.27	M	4
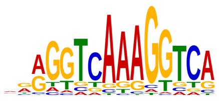	NNNNNNGGTCNNNNNTCATNNNNNN	11.86	DR:1	5

388 RXRB-Yin2017-Rnd3 (ERR1005934)

PWM	Seed Sequence	Seed Seq Enrichment	Repeat	Rank
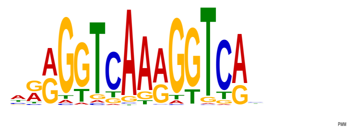	NNNNNNTCAANNGTCANNNNNN	86.50	DR:1	1
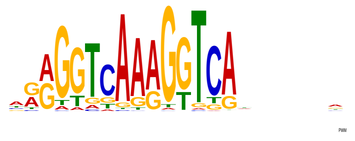	NNNNNNTCAANNNTCAANNNNNN	69.38	DR:1	2
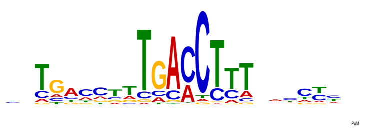	NNNNNNCTTTNNNNTTTGNNNNNN	26.46	M	3
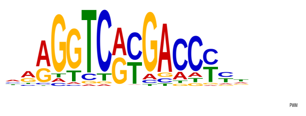	NNNNNNAGGTNNNGACCNNNNNN	19.85	IR:0	4
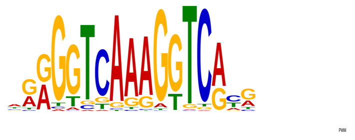	NNNNNNTCAANNNTCGCGNNNNNN	51.74	M	5
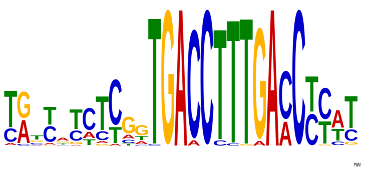	NNNNNNCTCGGTGANNNTTGANNNNNN	404.06	IR:4	6
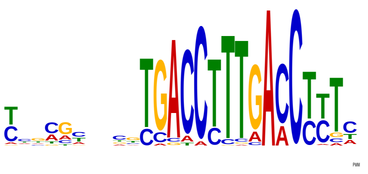	NNNNNNATCGTGANNNTTGANNNNNN	109.15	IR:4	7
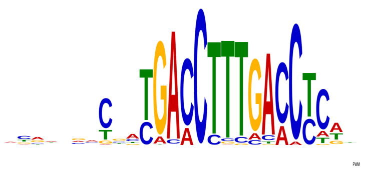	NNNNNNACGATGANNNTTGANNNNNN	175.89	IR:4	8
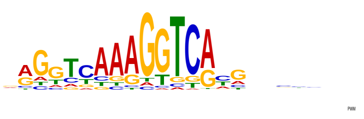	NNNNNNAAAGNNNACGANNNNNN	16.66	M	9

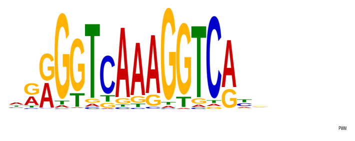	NNNNNNTCAANNNNTCGTNNNNNN	29.80	M	10
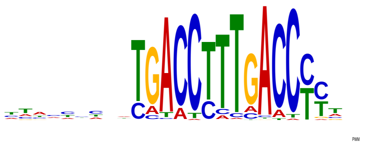	NNNNNNCGTTGANNNNTTGANNNNNN	97.94	IR:4	11
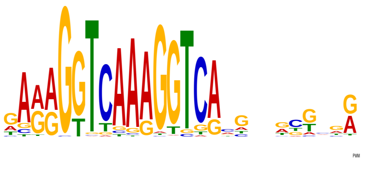	NNNNNNTCAANNNNNCACGATNNNNNN	40.30	M	12
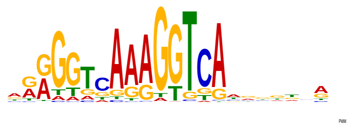	NNNNNNTCAANNNNNCAAANNNNNN	30.99	M	13
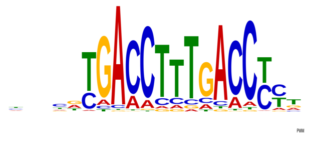	NNNNNNCCGTNNNNTTTGNNNNNN	14.36	M	14
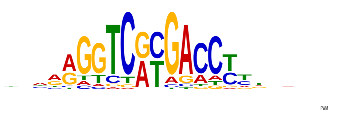	NNNNNNAGGTNNNNACCTNNNNNN	14.41	IR:0	15
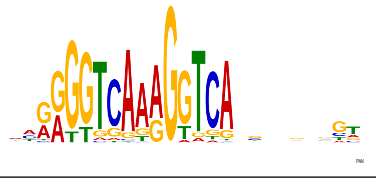	NNNNNNTCAANNNNTCAATANNNNNN	40.60	DR:1	16
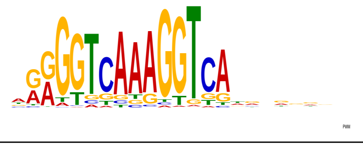	NNNNNNTCAANNNNNCAATNNNNNN	15.92	M	17
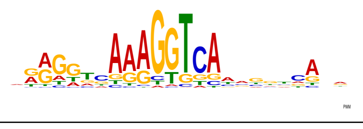	NNNNNNTCAANNNNNNAAAGNNNNNN	19.63	M	18

389 RXRB-Yin2017-Rnd3 (ERR1005935)

PWM	Seed Sequence	Seed Seq Enrichment	Repeat	Rank
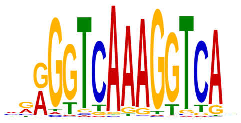	NNNNNNGGTCNNNNGTCANNNNNNN	79.77	DR:1	1
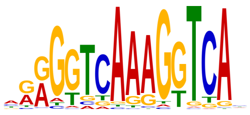	NNNNNNNTCAANNNNTCAANNNNNNN	57.64	DR:1	2
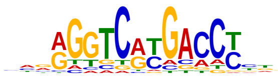	NNNNNNGGTCNNGACCNNNNNN	14.54	IR:0	3
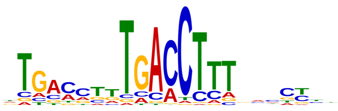	NNNNNNCTTTNNNNTTTGNNNNNN	22.06	M	4
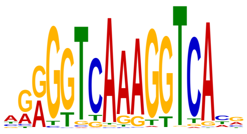	NNNNNNNTCAANNNNNCACGGNNNNNN	51.39	M	5
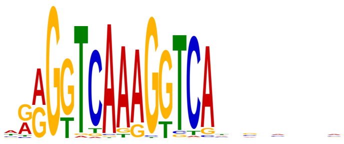	NNNNNNNTCAANNNNTCATTGNNNNNN	62.81	DR:1	6
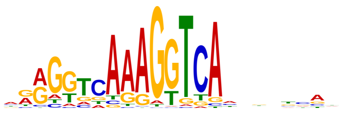	NNNNNNNTCAANNNNNCAAANNNNNNN	25.58	M	7
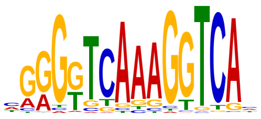	NNNNNNCGGGNNCAANNNNNNN	21.65	M	8

390 RXRB-Yin2017-Rnd4 (ERR1005936)

PWM	Seed Sequence	Seed Seq Enrichment	Repeat	Rank
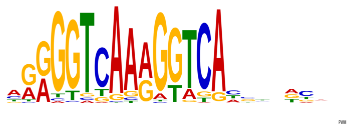	NNNNNNTCAANNNNCAAGNNNNNN	30.11	M	1
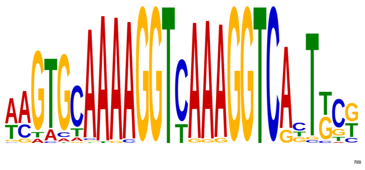	NNNNGCAAAAGNNNNNNNNTCAGNNNN	75.55	M	2
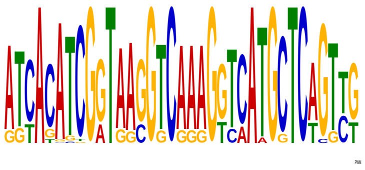	NNNACATCGNNNNNNNNNNNNNNNNNNNTCAGNNN	42.07	ER:18	3
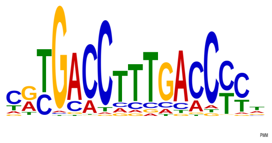	NNNNNNCGCGNNNTTTGNNNNNN	11.48	M	4
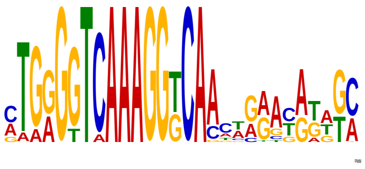	NNNNNNTCAANNNNCAACAGGANNNNN	143.15	DR:9	5
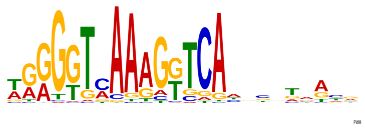	NNNNNNCAAANNNNAAATNNNNNN	10.46	M	6
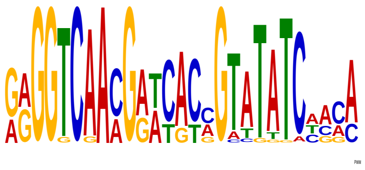	NNNNTCAANNNNNNNNGTATATCNNNN	67.66	DR:11	7
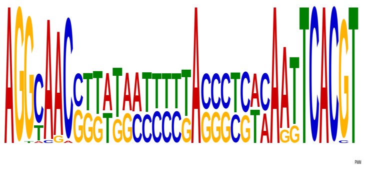	NGGCAACNNNNNNNNNNNNNNNNNNNNNTCACGN	48.11	DR:22	8
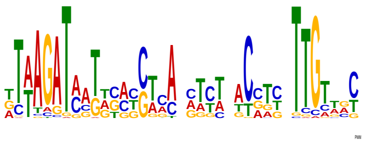	NNNAGATNNNNNNNNNNNNNNNNNNNNNNTTGTNNN	9.50	M	9

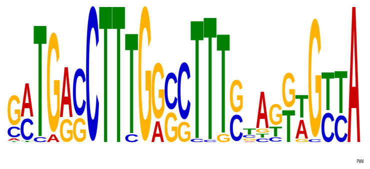	NNNNNNCTTTNNNNTTTCTAGNNNNNN	48.08	M	10
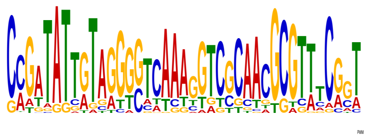	NCGATNNNNNNNNNNNNNNNNNNNNNNNNNNNNNNNTCGGN	4.81	M	11
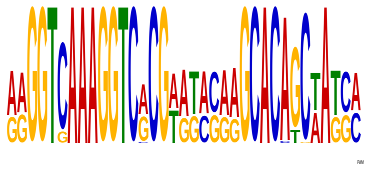	NNNNNCAAANNNNNNNNNNNNNNNGCACAGCNNNNN	61.87	M	12
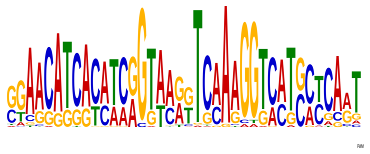	GGAANNNNNNNNNNNNNNNNNNNNNNNNNNNNNNNNNCAGT	4.80	M	13
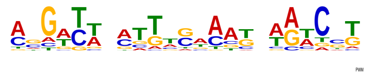	NNNNNNACTTNNNAAGTNNNNNN	1.95	ER:3	14
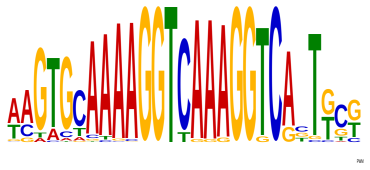	NNNNGCAAAANNNNNNNNNTCAGNNNN	34.97	M	15
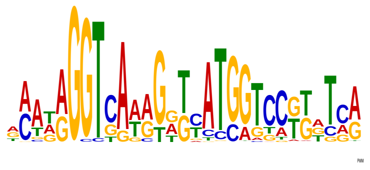	NNNNNNGTCANNNNNNNNTGGTCCNNNNNN	33.96	DR:7	16
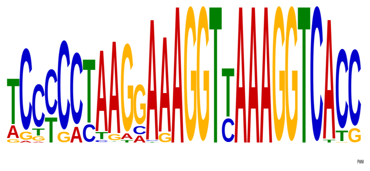	NNNNNNTAAGGAAANNNNAAAGNNNNNN	273.74	M	17
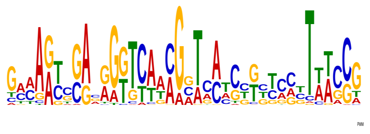	GAAANNNNNNNNNNNNNNNNNNNNNNNNNNNNNNNNNNTCGG	4.63	M	18

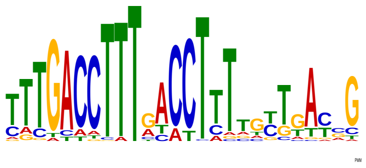	*NNNNNNCTTTNNNNTTTTGTNNNNNN*	101.46	M	19
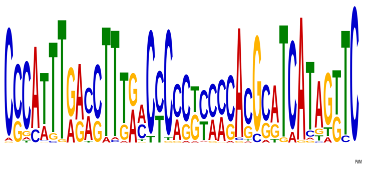	*NNCATTNNNNNNNNNNNNNNNNNNNNNNNNNNNNT*$\overrightarrow{AGGNN}$	12.38	M	20

391 RXRB-Yin2017-Rnd4 (ERR1005937)

PWM	Seed Sequence	Seed Seq Enrichment	Repeat	Rank
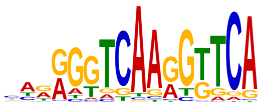	NNNNNNTCAANNTTCANNNNNN	18.51	DR:1	1
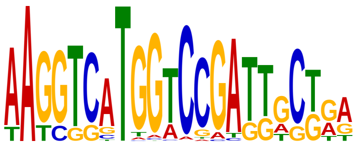	NNNNNNATGGTCCGATNNNNNN	29.63	M	2
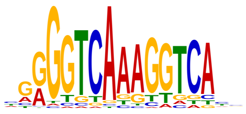	NNNNNNGGTCNNNNNTCAGNNNNNN	14.88	DR:1	3
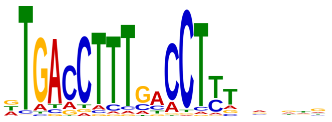	NNNNNNCTTTNNNNTTTTNNNNNN	16.46	M	4
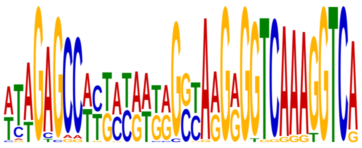	NTTGAGCCNNNNNNNNNNNNNNNNNNNNNNNGGTCN	30.50	ER:22	5
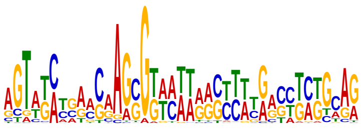	AGTTNNNNNNNNNNNNNNNNNNNNNNNNNNNNNNNNNGCAA	3.87	M	6
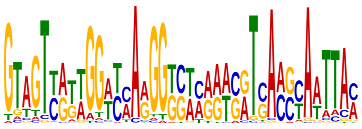	NTAGTNNNNNNNNNNNNNNNNNNNNNNNNNNNNNATTAN	4.21	M	7
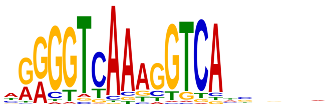	NNNNNNTCAANNNNCAATNNNNNN	14.24	M	8
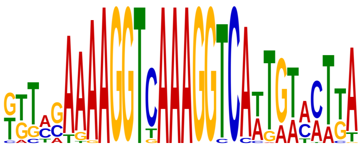	NNNNNAAAAGNNNNNNNNNTCAATGTNNNNN	86.53	DR:0	9

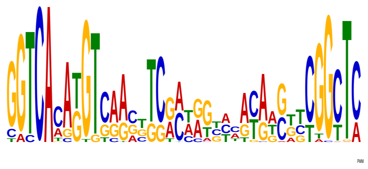	GGTCNNNNNNNNNNNNNNNNNNNNNNNNNNNNCGGCTC	18.77	DR:28	10
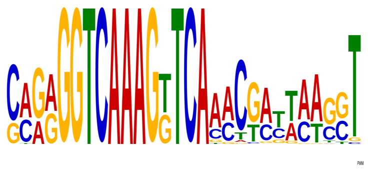	NNNNNNTCAANNNNCAAACTCTNNNNNN	52.43	DR:7	11
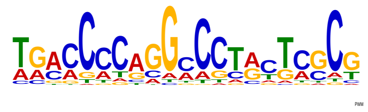	NNNNNNCAGGNCCTANNNNNN	9.33	M	12
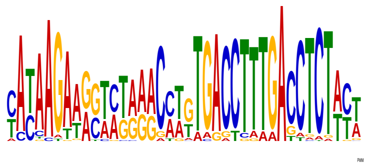	NNNAAGATNNNNNNNNNNNNNNNNNNNNNNNCTCTNNN	9.30	M	13
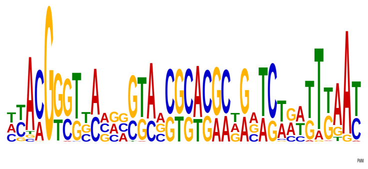	NTACGNNNNNNNNNNNNNNNNNNNNNNNNNNNTTTAAN	8.85	M	14
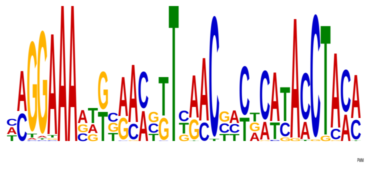	NNGGAAANNNNNNNNNNNNNNNNNNNNNACCTANN	17.88	IR:19	15
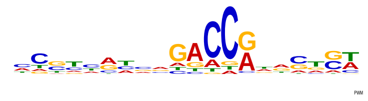	NNNNNNCCAGACCANNNNNN	5.12	M	16
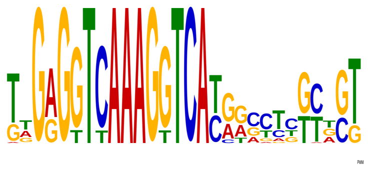	NNNNNNTCAANNNNCACAGATTNNNNNN	57.16	DR:8	17
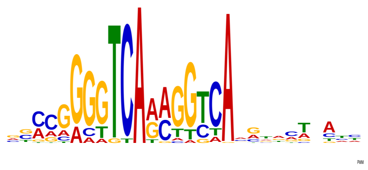	NNNNNNGGTCNNNNNTCAGGTANNNNNN	32.60	DR:6	18

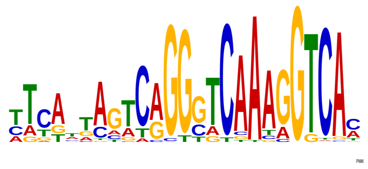	$NNNNN\overrightarrow{NAGTCA}GNNNCAAANNNNNNN$	32.55	M	19
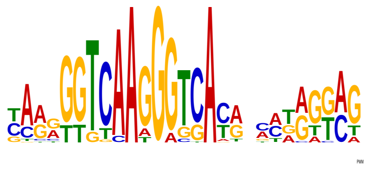	$NNN\overrightarrow{NNNTCA}ANNNNNCACAAAANNNNNN$	28.06	M	20

392 RXRG:RXRA-Bhimsaria2022-Rnd3 (S13-162)

PWM	Seed Sequence	Seed Seq Enrichment	Repeat	Rank
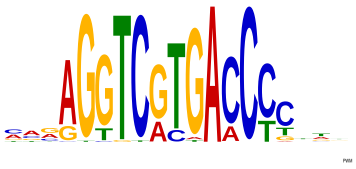	NNNAGGTCGTGACCCCNNN	8955.65	IR:0	1
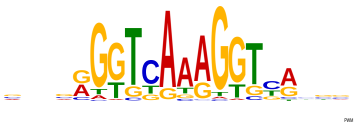	NNNNNGGTCAAAGGTNNNNN	674.31	DR:1	2
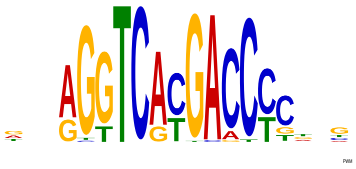	NNNAGGTCACGACCCCNNN	5987.58	IR:0	3
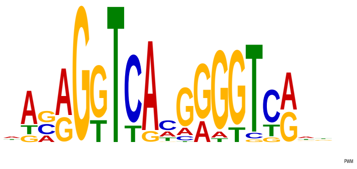	NNNNGGTCACGGGGTCNNNN	1205.81	DR:2	4
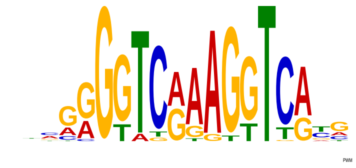	NNNGGGGTCGAAGGTCNNN	1880.45	DR:1	5
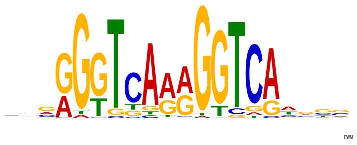	NNNNNGTCAGGGGTCNNNNN	152.40	DR:1	6
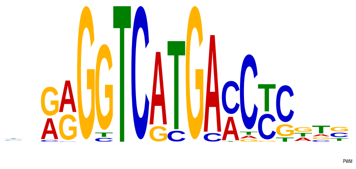	NNNGGGTCATGAACCCNNN	2700.78	IR:0	7
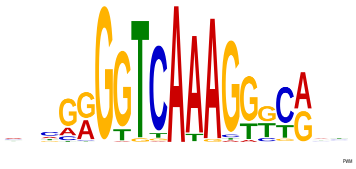	NNNGGGGTCAAAGGGCNNN	2153.27	DR:1	8
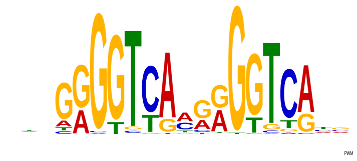	NNNGGGGTCNNNNGGTCNNN	173.83	DR:2	9

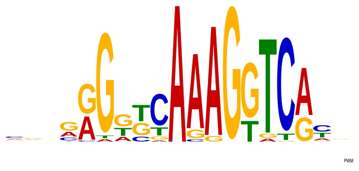	$NNNG\overrightarrow{GGGGCA}A\overrightarrow{AGGTCN}NN$	1532.63	DR:1	10
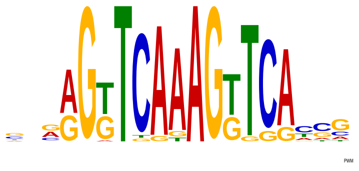	$NNN\overrightarrow{AGTTCA}A\overrightarrow{AGTTCA}NNN$	3652.00	DR:1	11
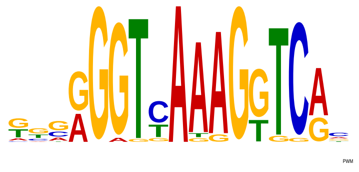	$NNG\overrightarrow{GGGTTA}A\overrightarrow{AGTTCN}N$	1104.40	DR:1	12
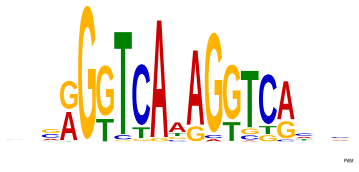	$NNN\overrightarrow{NGGTCA}T\overrightarrow{AGGTCN}NNN$	472.65	DR:1	13
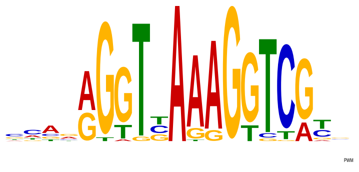	$NNNA\overrightarrow{AGGTGA}A\overrightarrow{AGGTCN}NN$	1304.42	DR:1	14
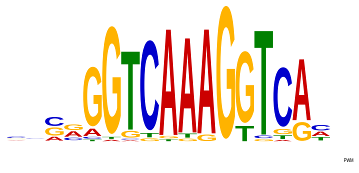	$NNN\overrightarrow{CGGTCA}A\overrightarrow{AGGTCN}NN$	802.59	DR:1	15
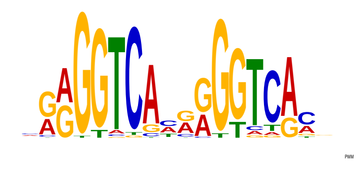	$NNN\overrightarrow{NGGTCA}TN\overrightarrow{NGGTCN}NNN$	128.03	DR:2	16
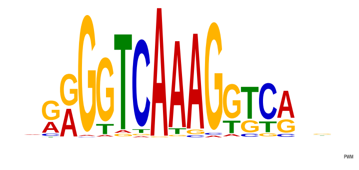	$NNN\overrightarrow{NGGTCN}A\overrightarrow{AGGGCN}NNN$	234.58	DR:1	17
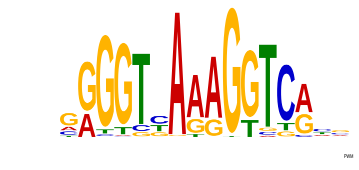	$NNNG\overrightarrow{GGGTTA}N\overrightarrow{NGGTCN}NN$	443.35	DR:1	18

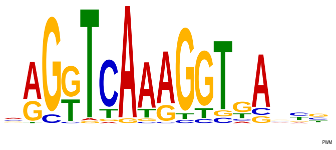	$NN\overrightarrow{AGGTCA}A\overrightarrow{AGGTGA}GNN$	3632.26	DR:1	19
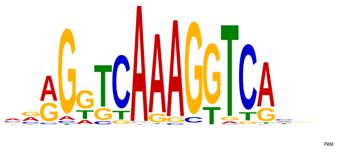	$NNNN\overrightarrow{GGGCNA}\overrightarrow{AGGTCN}NNN$	177.35	DR:0	20

393 RXRG-17-Bhimsaria2022-Rnd2 (S07-016)

PWM	Seed Sequence	Seed Seq Enrichment	Repeat	Rank
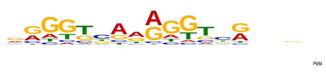	NNNNNGTCANNGGTCNNNNN	6.57	DR:1	1
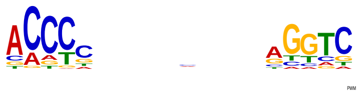	ACCCCNNNNNNNNNNAGGTC	31.23	ER:11	2
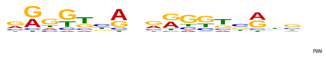	NNNNGGTCNNNNGGTCNNNN	4.20	DR:2	3
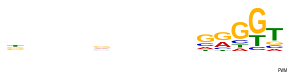	GGGGNNNNNNNNNNNGGGGT	10.26	M	4
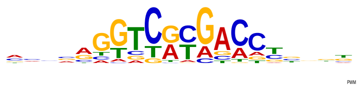	NNNNNGGTCATGACCNNNNN	22.99	IR:0	5
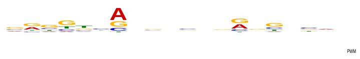	NNGGTCNNNNNNNNGGTCNN	2.63	DR:6	6
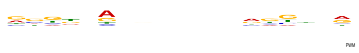	NGGTCNNNNNNNNNGGTCN	2.56	DR:7	7
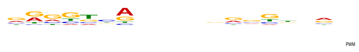	NNGGTCNNNNNNNGGTCNN	2.83	DR:5	8
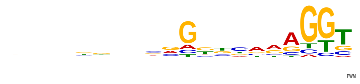	GGGGNNNNNNNNNNAAAGGT	13.73	M	9

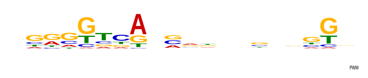	*NGGGGTCNNNNNNNNGGGTN*	8.08	DR:7	10
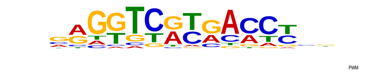	*NNNGGGTCATNACCCNNN*	24.20	IR:0	11
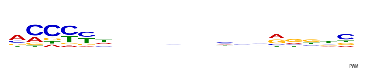	*ACCTNNNNNNNNNNNNGGTC*	4.66	ER:11	12
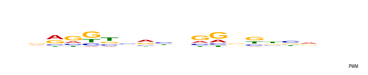	*NNNGGTCNNNNNGGTCNNN*	2.69	DR:3	13
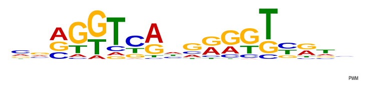	*NNNGGTCACNGGGTCNNN*	17.63	DR:2	14
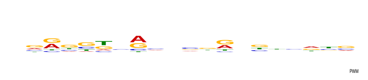	*NNNGGTCNNNNNNGGTCNNN*	2.41	DR:4	15
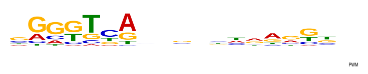	*GGGGTCNNNNNNNNNGGTC*	6.92	DR:7	16
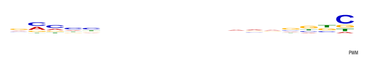	*NACCCNNNNNNNNNAGGTN*	2.42	ER:9	17
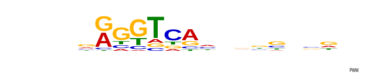	*NNNNNNGGTCACGGNNNNNN*	4.97	M	18

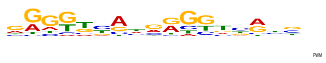	$NNN\overrightarrow{NGGTCN}NN\overrightarrow{GGGTCN}NNNN$	6.26	DR:2	19
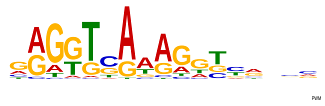	$NNN\overrightarrow{NGGTCA}A\overrightarrow{AGGGCN}NNNN$	23.47	DR:1	20

394 RXRG-17-Bhimsaria2022-Rnd3 (S07-137)

PWM	Seed Sequence	Seed Seq Enrichment	Repeat	Rank
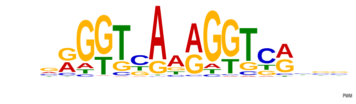	NNNNGGTCANNGGTCNNNN	187.65	DR:1	1
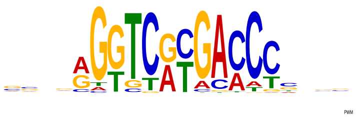	NNNNAGGTCATGACCCNNNN	1944.86	IR:0	2
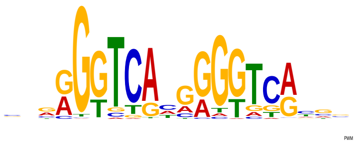	NNNNGGTCACNGGGTCNNNN	382.72	DR:2	3
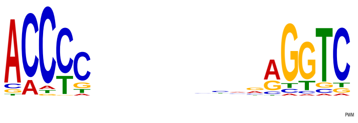	ACCCCNNNNNNNNNNAGGTC	362.76	ER:11	4
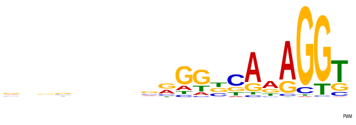	GGGGNNNNNNNNNCAAAGGT	500.72	M	5
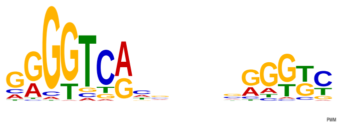	NGGGGTCNNNNNNGGGGTN	118.94	DR:6	6
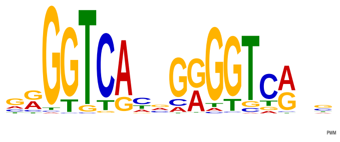	NNNGGTCACNGGGGTCNNN	500.22	DR:3	7
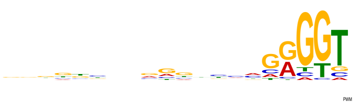	GGGGNNNNNNNNNNNGGGGT	90.45	M	8
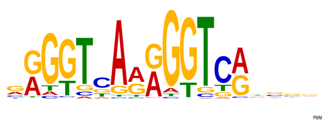	NNNNNGTCAGGGGTCNNNNN	143.53	DR:1	9

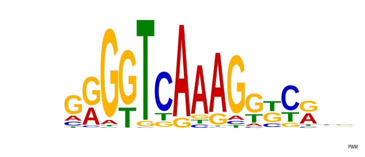	$NNNG\overrightarrow{GGGTCA}A\overrightarrow{AGGGCN}NN$	1735.42	DR:1	10
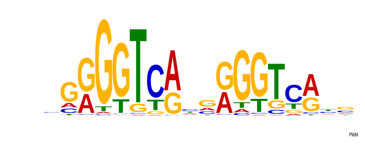	$NNNG\overrightarrow{GGGTCN}NNN\overrightarrow{NGGTCN}NN$	157.87	DR:2	11
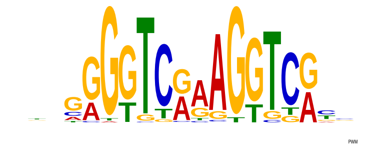	$NNNG\overrightarrow{GGGTCG}A\overrightarrow{AGGTCN}NN$	1862.19	DR:1	12
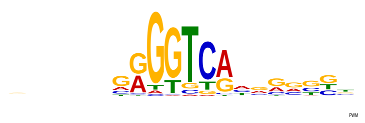	$NNNNNNG\overrightarrow{GGGTCA}TNNNNNN$	49.67	M	13
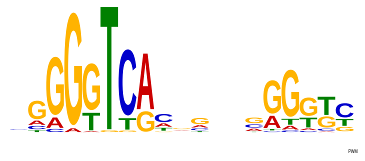	$NG\overrightarrow{GGGTCA}CNNNNG\overrightarrow{GGGTN}$	596.54	DR:6	14
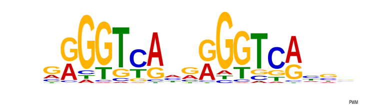	$NNN\overrightarrow{NGGTCN}NN\overrightarrow{NGGTCN}NNN$	45.48	DR:2	15
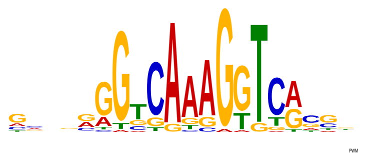	$NNNN\overrightarrow{CGGTCA}A\overrightarrow{AGGTCN}NNN$	555.12	DR:1	16
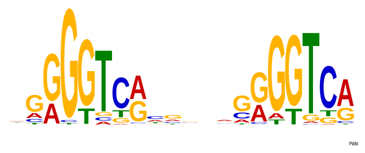	$NG\overrightarrow{GGGTCN}NNNNNG\overrightarrow{GGGTCN}$	579.41	DR:6	17
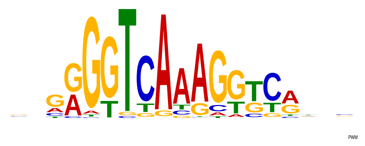	$NNN\overrightarrow{NGGTCA}A\overrightarrow{AGGGCN}NNN$	455.01	DR:1	18

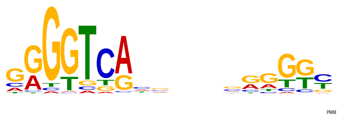	$NG\overrightarrow{GGGTCN}NNNNNN\overrightarrow{GGGTN}$	80.68	DR:6	19
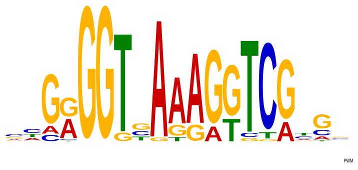	$NNG\overrightarrow{GGGTGA}A\overrightarrow{AGGTCG}CNN$	4405.25	DR:1	20

395 RXRG-Bhimsaria2022-Rnd2 (S07-051)

PWM	Seed Sequence	Seed Seq Enrichment	Repeat	Rank
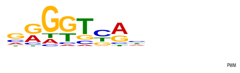	NNNNNNCGGGGTCANNNNNN	8.24	M	1
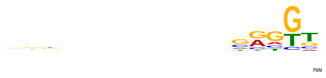	GGGGNNNNNNNNNNNNGGGT	7.96	M	2
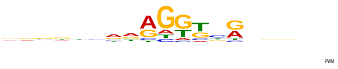	NNNNNNCAAAGGTCNNNNNN	7.10	M	3
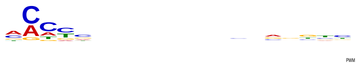	ACCCNNNNNNNNNNNNGGTC	6.67	ER:11	4
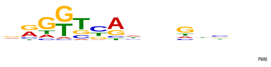	NNNNNNAGGTCAAANNNNNN	6.03	M	5
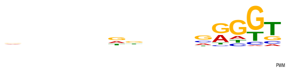	CCCANNNNNNNNNNNNGGGT	4.40	M	6
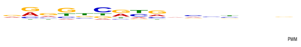	NNNNNGGTCNTGACNNNNNN	4.71	IR:0	7
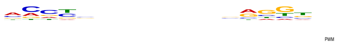	GACCNNNNNNNNNNNNGGTC	3.62	ER:9	8
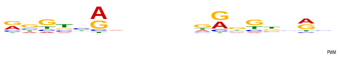	NNGGTCNNNNNNNNGGTCNN	3.81	DR:6	9

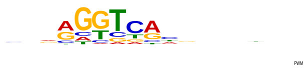	*NNNNNNAGGTCACGNNNNNN*	7.79	M	10
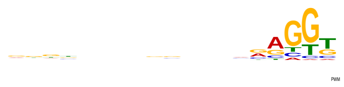	*GGGGNNNNNNNNNNNNAAGGT*	10.54	M	11
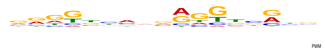	*NNNGGGTNNNNNGGTCNNN*	4.31	DR:2	12
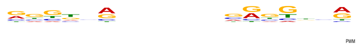	*NGGTCNNNNNNNNNGGTCN*	3.28	DR:7	13
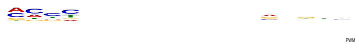	*ACCTNNNNNNNNNNNGGTC*	3.58	ER:10	14
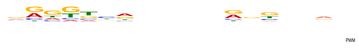	*NNGGTCNNNNNNNGGTCNN*	3.26	DR:5	15
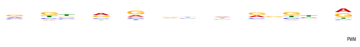	*NGGTCNNNNNNNNNNGGTCN*	2.66	DR:8	16
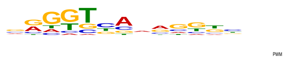	*NNNNNGGGTNAAAGNNNNN*	6.58	M	17
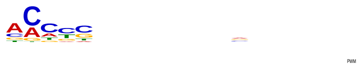	*ACCCNNNNNNNNNNNNNTCAT*	4.89	ER:9	18

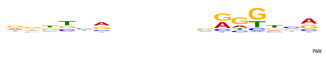	$NGGGGNNNNNNNNN\overrightarrow{NGGTCN}$	4.00	M	19
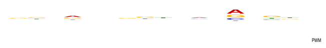	$\overrightarrow{AGGTNN}NNNNNNNN\overrightarrow{NGGTC}$	1.99	DR:8	20

396 RXRG-Bhimsaria2022-Rnd3 (S07-172)

PWM	Seed Sequence	Seed Seq Enrichment	Repeat	Rank
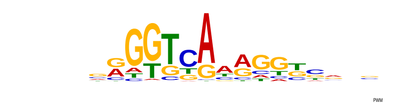	NNNNNGGTCAAAGGNNNNN	185.96	DR:1	1
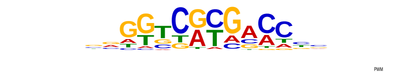	NNNNNGGTCATGACCNNNNN	353.07	IR:0	2
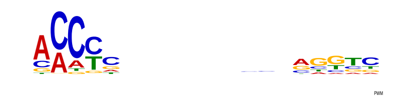	ACCCCNNNNNNNNNNNGGTC	137.23	ER:11	3
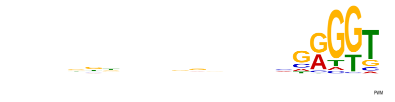	GGGGNNNNNNNNNNNGGGGT	119.94	M	4
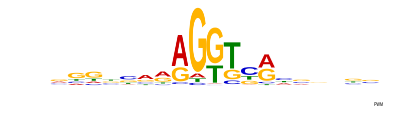	NNNNNNAAAGGTCANNNNNN	73.23	M	5
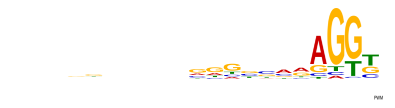	GGGGNNNNNNNNNNAAAGGT	181.08	M	6
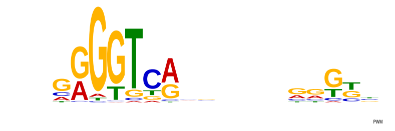	NGGGGTCNNNNNNNGGGTN	159.77	DR:6	7
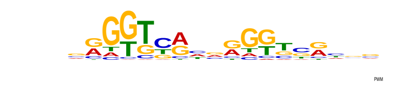	NNNNGGTCANNNGGTCNNNN	86.96	DR:2	8
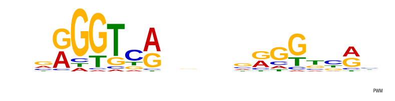	NNGGTCNNNNNNNGGTCNN	30.99	DR:5	9

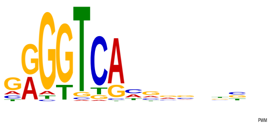<br>PWM	*NNNNNGGGGTCACGGNNNNN*	216.79	M	10
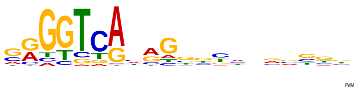<br>PWM	*GGGGTCNNNNNNNNNNGGTC*	46.50	DR:8	11
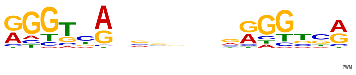<br>PWM	*NGGTCNNNNNNNNNNGGTCN*	26.01	DR:7	12
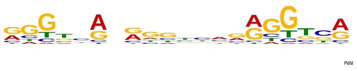<br>PWM	*NGGTCNNNNNNNNNNGGTCN*	19.32	DR:8	13
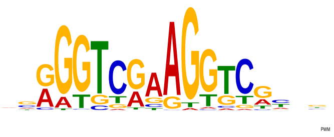<br>PWM	*NNNGGGTCGAAGGTCGNNN*	1295.08	DR:1	14
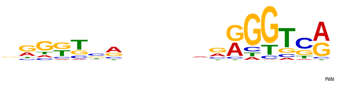<br>PWM	*NGGGGNNNNNNNNNNGGTCN*	35.88	M	15
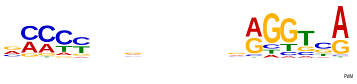<br>PWM	*NACCCNNNNNNNNNNGGTCN*	30.71	ER:9	16
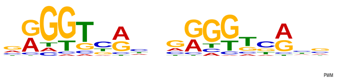<br>PWM	*NNNGGTCNNNNNGGTCNNN*	32.19	DR:3	17
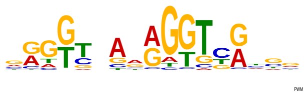<br>PWM	*NNNNGGGTNAGGGGTCNNNN*	217.57	DR:1	18

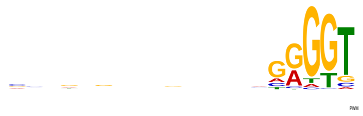	CCCGNNNNNNNNNNNG$\overrightarrow{GGGT}$	70.25	M	19
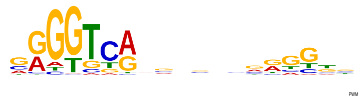	G$\overrightarrow{GGGTC}$NNNNNNNN$\overrightarrow{NGGTC}$	99.01	DR:7	20

397 RXRG-Jolma2013-Rnd2 (ERR195466)

PWM	Seed Sequence	Seed Seq Enrichment	Repeat	Rank
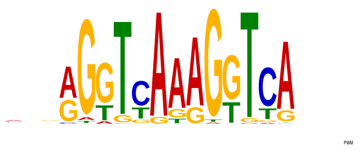	NNNNGGTCAAAGGTCNNNN	6800.59	DR:1	1
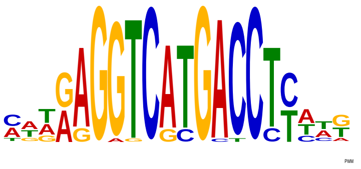	NNNAAGGTCATGACCTTNNN	41513.90	IR:0	2
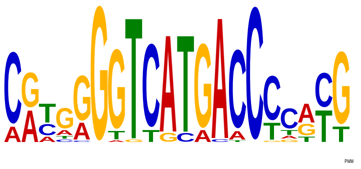	NNNGGGGTCATGACCCCNNN	32931.45	IR:0	3
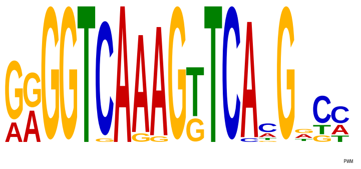	NNGGTCAAAGTTCACGGNN	60999.13	DR:1	4
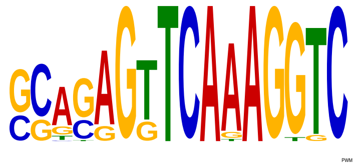	GCAGAGTTCAAAGGTC	74947.57	DR:1	5
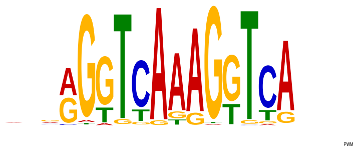	NNNNGGTCNNAGGTCNNNN	1076.98	DR:1	6
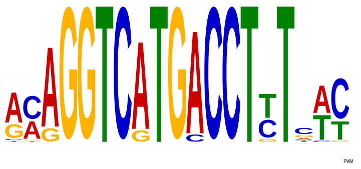	NNAGGTCATGACCTTTCNN	61467.53	IR:0	7
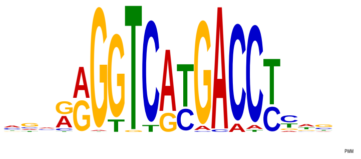	NNNNAGGTCANGACCCNNNN	2314.94	IR:0	8
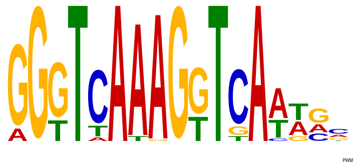	GGGTCAAANNTCAATGC	27991.84	DR:1	9

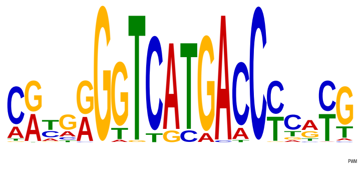	NNNGGGGTCNNGACCCCNNN	3709.83	IR:0	10
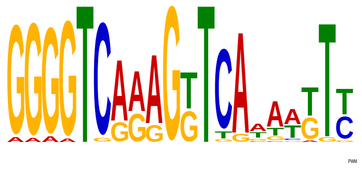	NNNGTCAAANNTCAAATNNN	4048.09	DR:1	11
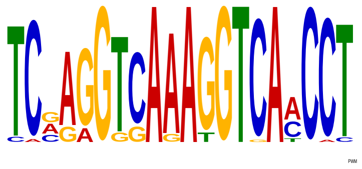	NNGAGGTCAANNGTCAACNN	39805.76	DR:1	12
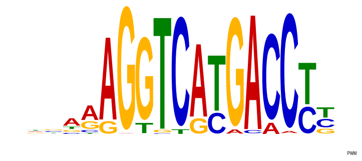	NNNAAAGGTCNNGACCNNN	2294.40	IR:0	13
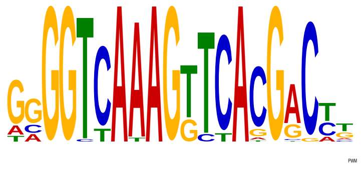	NNGGTCAAAGNNNACGACNN	15890.49	M	14
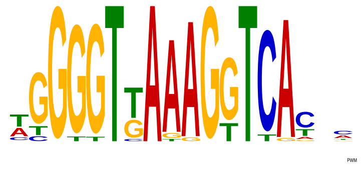	NGGGGTTAAAGGTCACTN	58673.72	DR:1	15
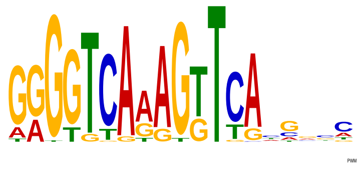	NNGGTCAAANNTCACGGNN	11781.06	DR:1	16
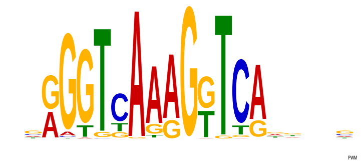	NNNNGTCAAANNTCATNNNN	2034.06	DR:1	17
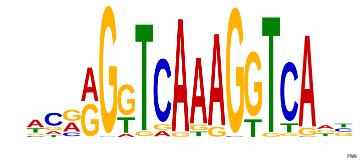	NNNGGGTTCNNAGGTCNNN	1269.47	DR:2	18

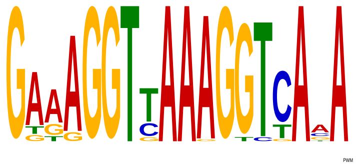	$NAA\overrightarrow{AGGTTA}A\overrightarrow{AGGTCA}AN$	138120.76	DR:1	19
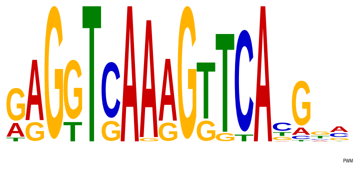	$N\overrightarrow{AGGTCA}A\overrightarrow{ANNTCA}CGTN$	12548.92	DR:1	20

398 RXRG-Jolma2013-Rnd3 (ERR195467)

PWM	Seed Sequence	Seed Seq Enrichment	Repeat	Rank
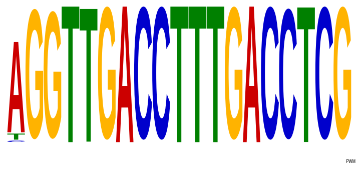	AGGTTGNNNNNNNNNCTCG	754.86	M	1
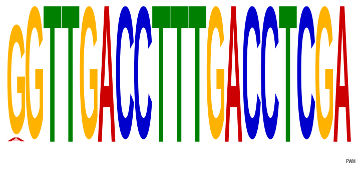	GGTTGNNNNNNNNNCTCGA	1162.26	M	2
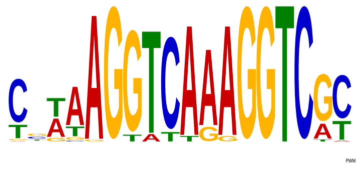	NGTAAGNNNNNNNGTCGN	11.43	DR:6	3
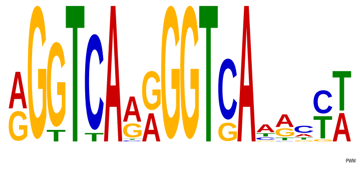	NNGTCAGNNNNCAAGCNN	23.95	M	4
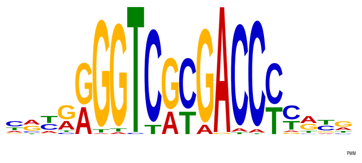	NNNNGGGTNNNNACCCNNNN	14.40	IR:0	5
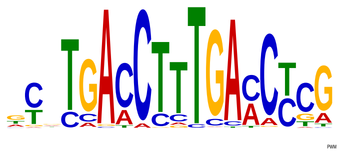	NNNGTGANNNNNGAACNNN	16.39	IR:4	6
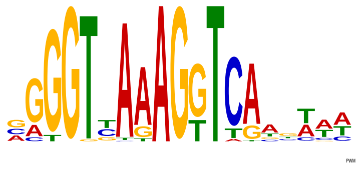	NNNGTTANNNNNCATCNNN	10.86	M	7
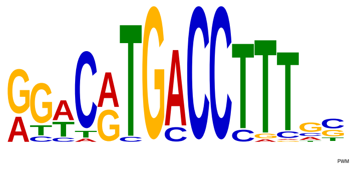	NGTCGTNNNNTTTCN	12.04	M	8
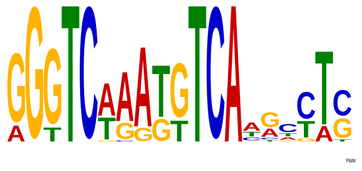	NNNTCAGNNNNCAAGCNNN	17.81	M	9

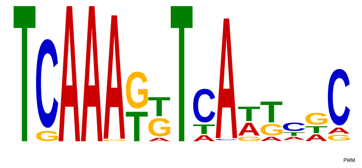	*NCAAANNNNATTCTN*	26.89	M	10
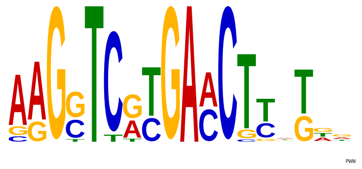	*NNNGTCGTNNNCTCTNNN*	12.00	IR:0	11
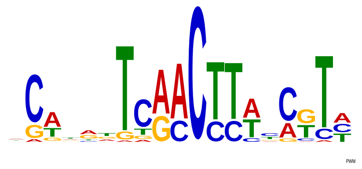	*NNNNTTTCNNNTTTGNNNN*	5.02	M	12
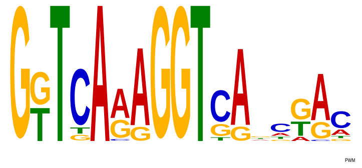	*NNNCAAGNNNCAATNNN*	3.65	M	13
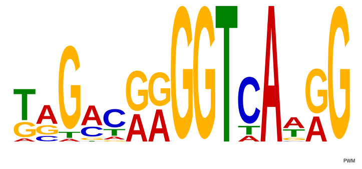	*NGGACGNNNNCAAGN*	8.90	M	14
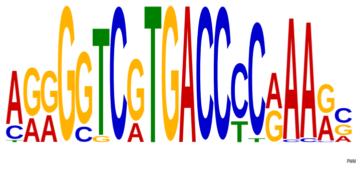	*NNNNGTCGTNNNCCCANNNN*	32.91	M	15
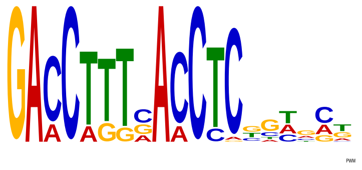	*NNNNTTTCNNNTCCCNNNN*	4.32	M	16
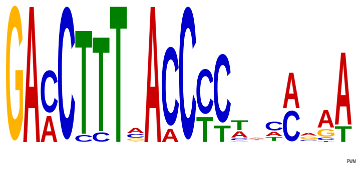	*NNNNTTTCNNNNCTACNNNN*	5.94	M	17
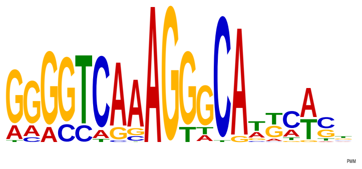	*NNNNNCAAANNGCAANNNNN*	6.52	M	18

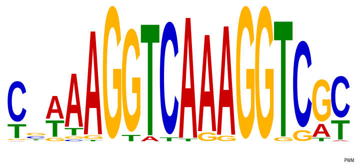	$\overrightarrow{NGTAA}NNNNNN\overrightarrow{NNGTCG}N$	4.74	DR:6	19
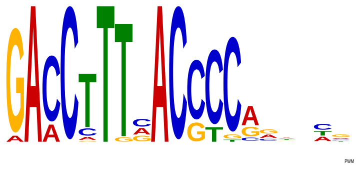	*NNNNTTTCNNNCCAGNNNN*	7.84	M	20

399 RXRG-Jolma2013-Rnd4 (ERR195468)

PWM	Seed Sequence	Seed Seq Enrichment	Repeat	Rank
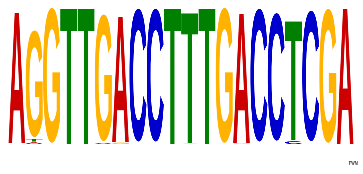	NGGTTGNNNNNNNNNCTCGN	530.14	M	1
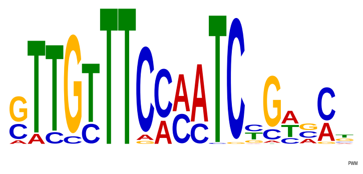	NNNNTTTCNNNTCCCNNNN	52.25	M	2
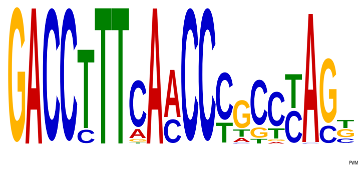	NNNNTTTCNNNNCGCCNNNN	32.40	M	3
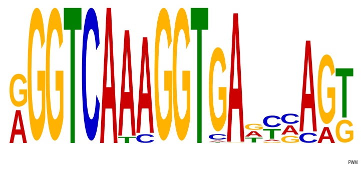	NNNNCAAANNNGAGTNNNN	133.21	M	4
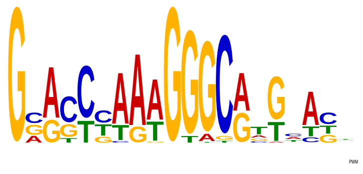	NNNNNCAAANNGCAANNNNN	20.08	M	5
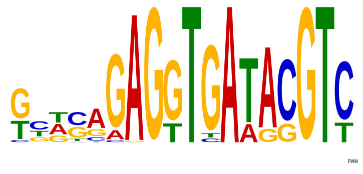	NNNGGGANNNGAAAGNNN	40.22	M	6
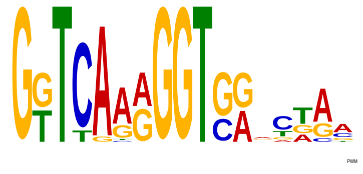	NNNCAAGNNNCAATNNN	5.77	M	7
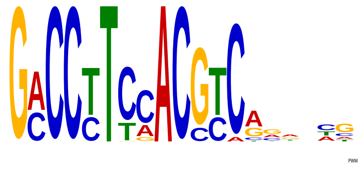	NNNNTTTCNNNCCAGNNNN	11.70	M	8
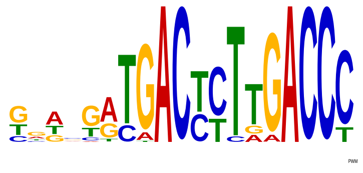	NNNNTATGNNNCTTGNNNN	14.88	M	9

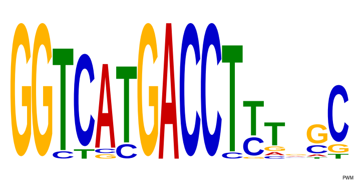	$\overrightarrow{NNTCA}TNNNNTCTCNN$	14.19	M	10
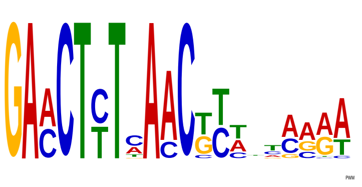	NNNNTTTCNNNNCTACNNNN	10.43	M	11
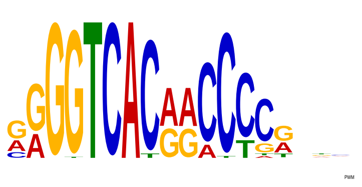	$N\overrightarrow{NNGTCA}CNNNCCCGNNN$	65.39	M	12
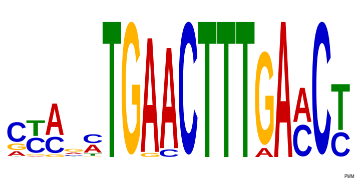	$NN\overrightarrow{NGCTGA}ANNT\overleftarrow{TGANNN}$	30.68	IR:4	13
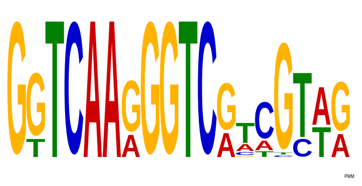	NNNCAAGNNNCATTGNNN	9.84	M	14
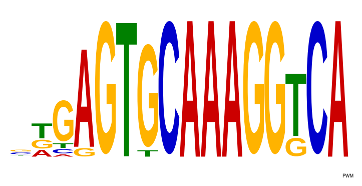	$NNGAGTGNNN\overrightarrow{AGGTNN}$	119.89	M	15
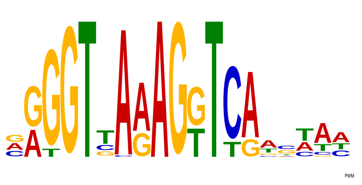	$N\overrightarrow{NNGTTA}NNNNNCATCNNN$	14.26	M	16
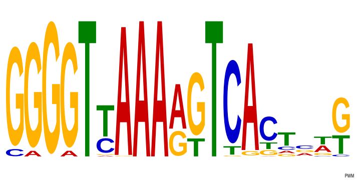	$N\overrightarrow{NNGTTA}NNNNNCACTNNN$	18.26	M	17
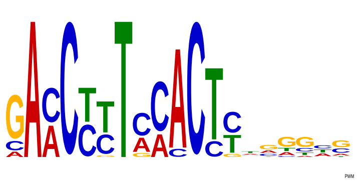	NNNNTTTCNNNCCAANNNN	7.08	M	18

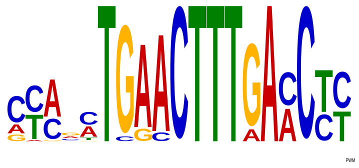	$NNN\overrightarrow{GCTGA}ANNNN\overleftarrow{TGACNN}N$	22.16	IR:4	19
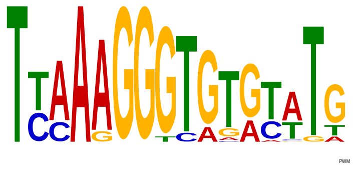	*NNAAAGNNNGTGTTNN*	21.73	M	20

400 RXRG-Yin2017-Rnd4 (ERR1005945)

PWM	Seed Sequence	Seed Seq Enrichment	Repeat	Rank
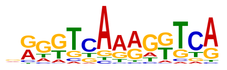	NNNNNNTCAANNNNTCATNNNNNN	12.43	DR:1	1
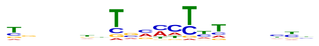	NNNNNNCTTTNNNNTTTGNNNNNN	7.00	M	2
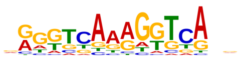	NNNNNNTCAANNNNNCACGNNNNNN	8.67	M	3
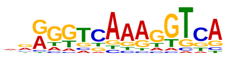	NNNNNNTCAANNNNNCAATNNNNNN	7.03	M	4
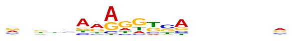	NNNNNNTCAANNNNNNAAAGNNNNNN	4.85	M	5
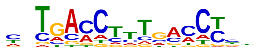	NNNNNNGCGTNNNNTTTGNNNNNN	4.84	M	6
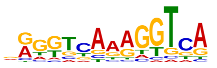	NNNNNNTCAANNNNNCAGCNNNNNN	5.23	M	7
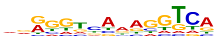	NNNNNNTCAANNNNNNATTGNNNNNN	3.40	M	8
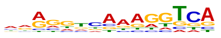	NNNNNNTCAANNNNNNACAANNNNNN	3.32	M	9

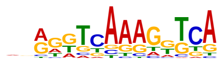	NNNNNNTCAANNNNNCAAATNNNNNN	9.71	M	10
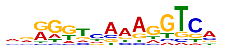	NNNNNNTCAANNNNNNAATCNNNNNN	3.58	M	11
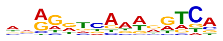	NNNNNNTCAANNNNNNAACANNNNNN	3.74	M	12
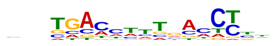	NNNNNNTGTTNNNNTTTGNNNNNN	3.92	M	13
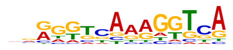	NNNNNNTCAANNNNNCAGANNNNNN	5.50	M	14
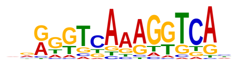	NNNNNNTCAANNNNNCATGTNNNNNN	8.93	DR:6	15
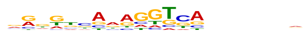	NNNNNNTCAANNNNNNAGAGNNNNNN	3.21	M	16
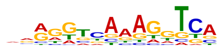	NNNNNNTCAANNNNNNAAACNNNNNN	4.19	M	17
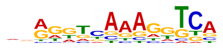	NNNNNNTCAANNNNNNAAATNNNNNN	4.55	M	18

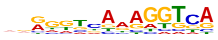 PWM	$\overrightarrow{NNNNNTCA}ANNNNNNACAGNNNNNN$	3.85	M	19
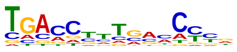 PWM	*NNNNNNTTTGNNNCCGCNNNNNN*	3.23	M	20

401 NOR1-Bhimsaria2022-Rnd2 (S07-011)

PWM	Seed Sequence	Seed Seq Enrichment	Repeat	Rank
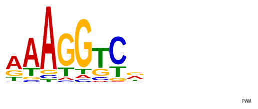	NNNNNNAAAGGTCGNNNNNN	13.49	M	1
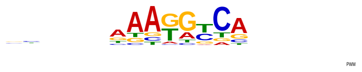	NNNNNNAAATGCCANNNNNN	7.64	M	2
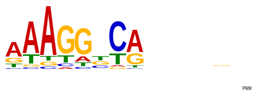	NNNNNNAAAGGGCANNNNNN	10.54	M	3
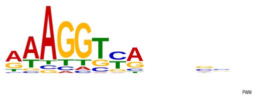	NNNNNNAAAGGTTANNNNNN	11.36	M	4
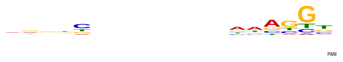	AAGGNNNNNNNNNNNNNAGGT	5.21	DR:9	5
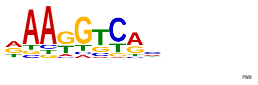	NNNNNNTAAGGTCANNNNNN	8.25	M	6
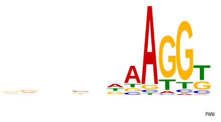	GGGCNNNNNNNNNNNNAGGT	12.10	DR:11	7
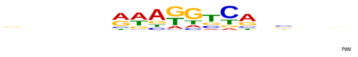	NNNNNTATANGTCANNNNNN	4.70	M	8
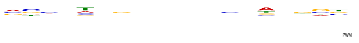	ACCTNNNNNNNNNNNNAGGT	3.74	ER:11	9

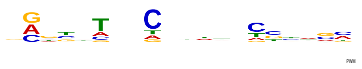	NNNNNNGCTCTTTANNNNNN	1.39	M	10
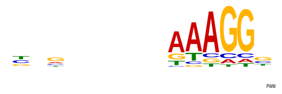	GGAGNNNNNNNNNNNAAAGGG	15.87	M	11
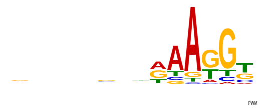	CGGGNNNNNNNNNNNNAAGGT	10.33	M	12
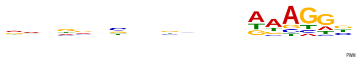	AAAGNNNNNNNNNNNNAAGGG	9.05	M	13
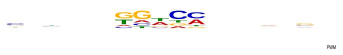	NNNNNAATGNCATTNNNNN	3.72	M	14
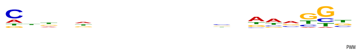	ATTTNNNNNNNNNNNNNAGGT	3.75	M	15
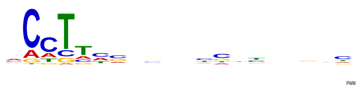	ACCTTNNNNNNNNNNNGGTC	10.14	ER:11	16
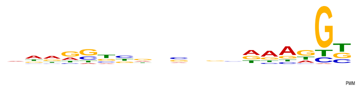	AAAGGNNNNNNNNNNAAGGT	12.07	DR:7	17
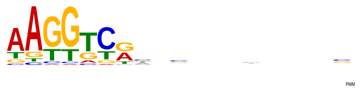	AAGGTCNNNNNNNNNGGTG	11.84	DR:7	18

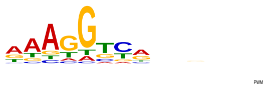	$NNNNNAA\overrightarrow{AGNTCG}CNNNNN$	5.79	M	19
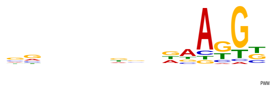	$GGAANNNNNNNNNNNA\overrightarrow{AGGT}$	8.25	M	20

402 NOR1-Bhimsaria2022-Rnd3 (S07-132)

PWM	Seed Sequence	Seed Seq Enrichment	Repeat	Rank
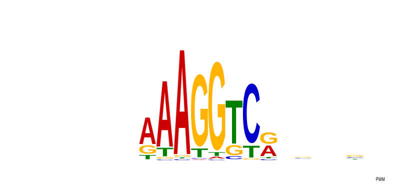	NNNNNNAAAGGTCGNNNNNN	92.46	M	1
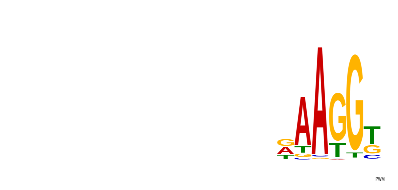	GGGGNNNNNNNNNNNNAAGGT	117.02	M	2
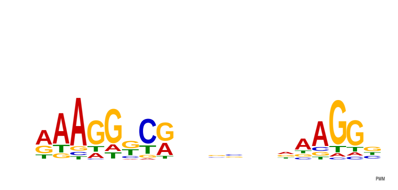	AAAGGGCNNNNNNNNAAGGG	654.60	DR:8	3
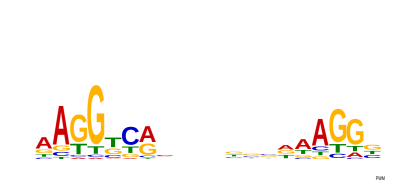	AAGGTCNNNNNNNNNAAGGG	316.08	DR:9	4
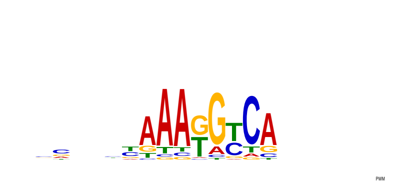	NNNNNNAAATGCCANNNNNN	36.98	M	5
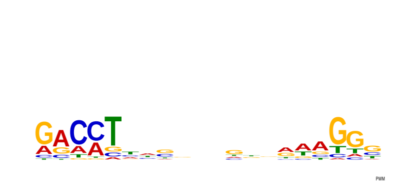	GACCTTNNNNNNNNNAAGGG	299.82	ER:11	6
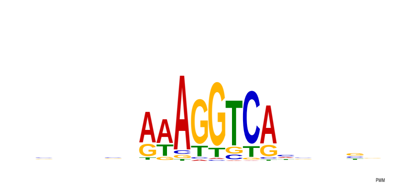	NNNNNNATAGGTCANNNNNN	42.14	M	7
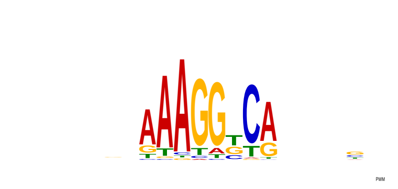	NNNNNNAAAGGGCANNNNNN	63.43	M	8
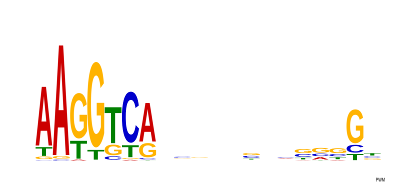	AAGGTCANNNNNNNNGGGGT	324.47	DR:9	9

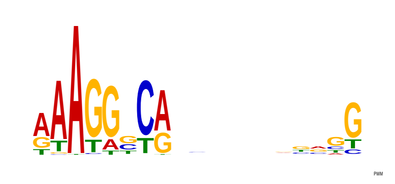	AAAGGGCANNNNNNNNGGGT	342.17	DR:8	10
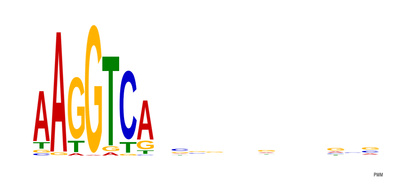	AAGGTCANNNNNNNNNCGCG	174.60	M	11
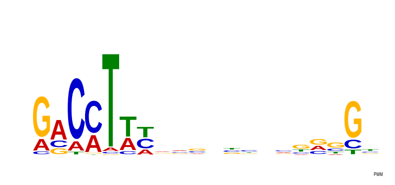	GACCTTNNNNNNNNNGGGGT	203.75	ER:11	12
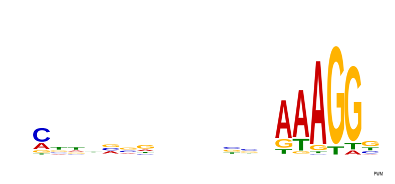	ATTTNNNNNNNNNNNAAAGGG	88.87	M	13
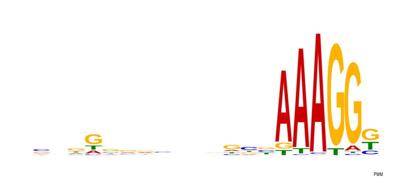	CCGGGNNNNNNNNNAAAGGG	194.49	M	14
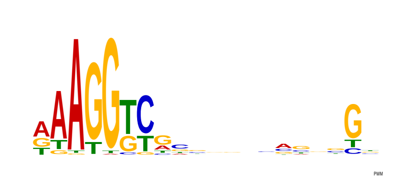	AAAGGTCGNNNNNNNNGGGT	373.67	DR:8	15
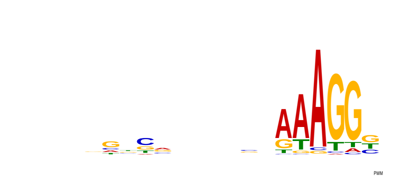	GAAGNNNNNNNNNNAAAGGG	145.59	M	16
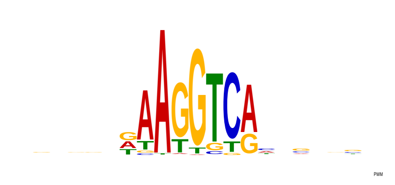	NNNNNGAAGGTCACGNNNNN	178.69	M	17
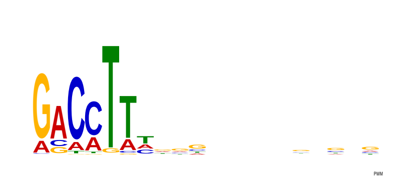	GACCTTNNNNNNNNNGGTG	100.28	ER:10	18

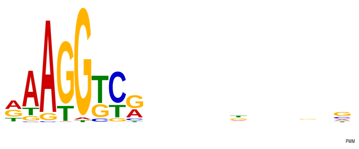	$AA\overrightarrow{AGGTC}NNNNNNNNNCGTG$	170.89	M	19
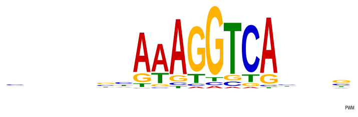	$NNNNNGCAT\overrightarrow{AGGTC}NNNNN$	32.69	M	20

403 NURR1-Bhimsaria2022-Rnd1 (S04-144)

PWM	Seed Sequence	Seed Seq Enrichment	Repeat	Rank
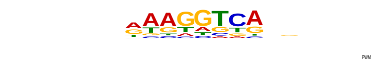	NNNNNNAAAGGTCANNNNNN	6.74	M	1
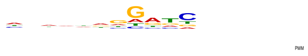	NNNNNNAAAGGATCNNNNNN	3.63	M	2
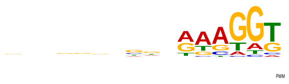	AGGGNNNNNNNNNNNAAGGT	7.04	DR:10	3
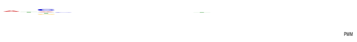	ATCCNNNNNNNNNNNNTTTT	2.34	M	4
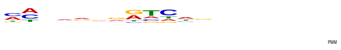	NNNNNNAGATCAGANNNNNN	2.93	M	5
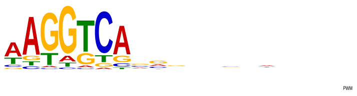	AAGGTCANNNNNNNNNGGGG	15.30	M	6
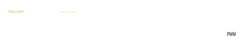	CGGGNNNNNNNNNNNNGGGG	1.80	M	7
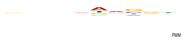	ACAGNNNNNNNNNNNNAGGT	2.34	M	8
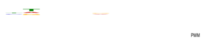	CAAANNNNNNNNNNNNGATC	1.63	M	9

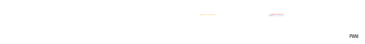	ATCANNNNNNNNNNNNGGAT	1.91	M	10
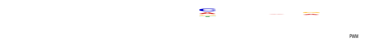	CGCGNNNNNNNNNNNNGGTC	1.53	M	11
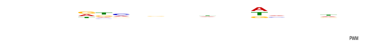	CGGCNNNNNNNNNNNNGGTC	1.48	M	12
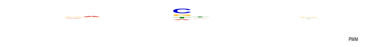	NNNNNAGGTNACAGNNNNN	1.25	M	13
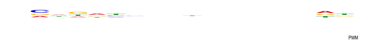	NNNGATCCNNNGTAGNNN	1.96	M	14
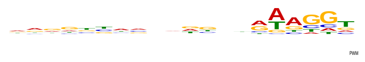	AAGGNNNNNNNNNNNTAGGT	4.04	DR:9	15
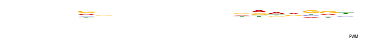	CGAANNNNNNNNNNNNAGGT	2.25	M	16
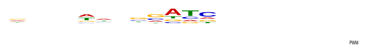	NNNNNNTGGATCACNNNNNN	2.25	M	17
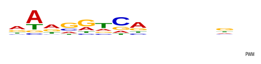	NNNNNNATAGGTCANNNNNN	3.00	M	18

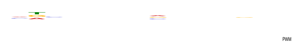	$\overrightarrow{AGGTN}NNNNNNNNNN\overrightarrow{NGGTC}$	1.41	DR:9	19
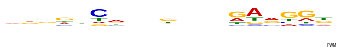	$AA\overrightarrow{AGGNN}NNNNNNNNN\overrightarrow{AGGT}$	4.79	DR:8	20

404 NURR1-Bhimsaria2022-Rnd2 (S04-145)

PWM	Seed Sequence	Seed Seq Enrichment	Repeat	Rank
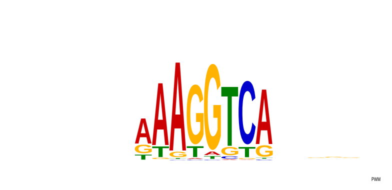	NNNNNNAAAGGTCANNNNNN	67.68	M	1
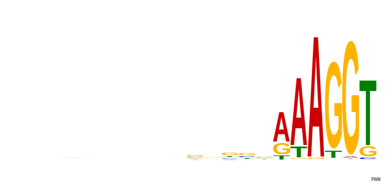	AGGGNNNNNNNNNNAAAGGT	115.43	DR:10	2
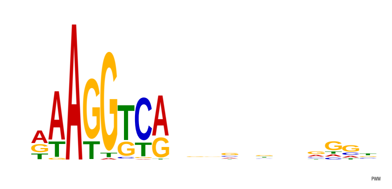	AAAGGTCANNNNNNNNGGGT	433.29	DR:8	3
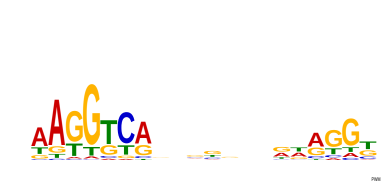	AAGGTCANNNNNNNNNAGGT	188.54	DR:9	4
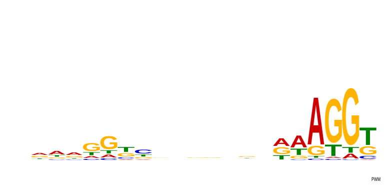	AAAGGNNNNNNNNNNAAGGT	97.84	DR:8	5
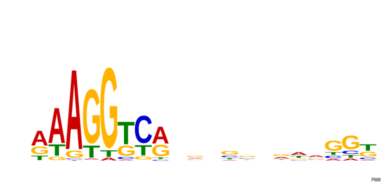	AAAGGTCNNNNNNNNNAGGG	163.43	DR:8	6
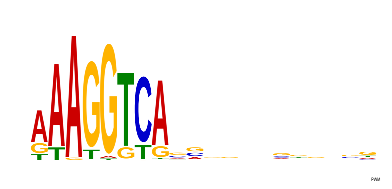	AAAGGTCANNNNNNNNGTGG	264.94	M	7
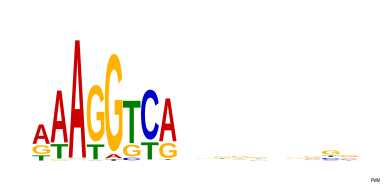	AAAGGTCANNNNNNNGGGT	251.49	DR:7	8
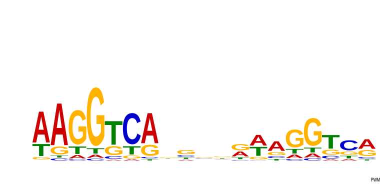	NAGGTCANNNNNNAGGTCN	68.40	DR:6	9

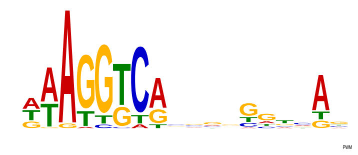	NNAAGGTCANNGGGGTCNN	171.71	DR:3	10
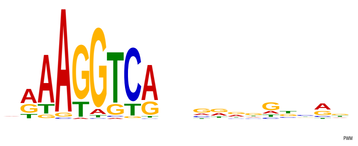	NNAAGGTCANNNNNGGTCNN	87.59	DR:4	11
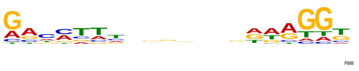	GACCTNNNNNNNNNNNAGGT	26.77	ER:11	12
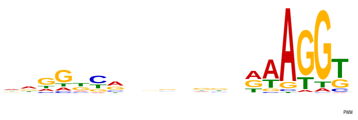	AAGGTNNNNNNNNNNAAGGT	75.90	DR:9	13
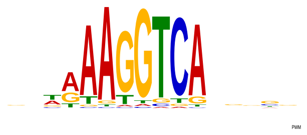	NNNNNTGAAGGTCANNNNN	39.73	M	14
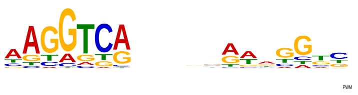	AAGGTCNNNNNNNNAGGTC	38.88	DR:7	15
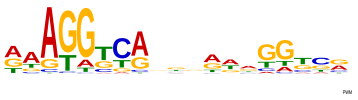	NAAGGTCNNNNNNAGGTCN	58.53	DR:5	16
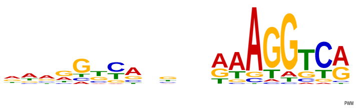	NAAGGNNNNNNNNAAGGTCN	31.00	DR:6	17
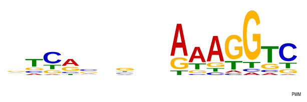	AAGGNNNNNNNNNAAGGTC	12.80	DR:7	18

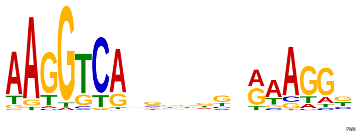	$A\overrightarrow{AGGTCA}NNNNNNNNNA\overrightarrow{AGGG}$	311.52	DR:9	19
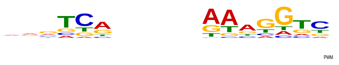	$A\overrightarrow{AGGNNN}NNNNNNN\overrightarrow{AGGTC}$	5.80	DR:7	20

405 NURR1-Bhimsaria2022-Rnd3 (S04-146)

PWM	Seed Sequence	Seed Seq Enrichment	Repeat	Rank
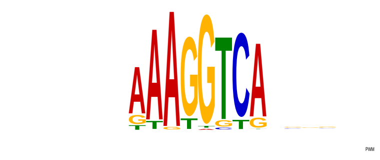	NNNNNNAAAGGTCANNNNNN	278.85	M	1
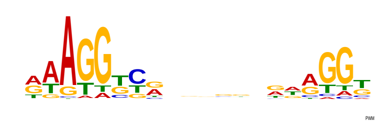	AAAGGTCNNNNNNNNNAGGT	1364.14	DR:8	2
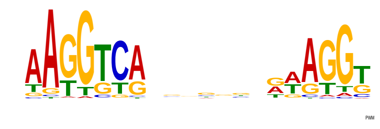	AAGGTCANNNNNNNNNAGGT	1318.63	DR:9	3
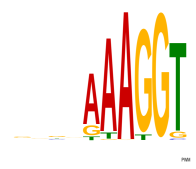	CGGGNNNNNNNNNNAAAGGT	526.79	M	4
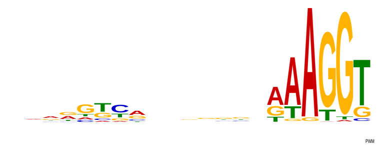	AAGGNNNNNNNNNNNAAGGT	250.03	DR:9	5
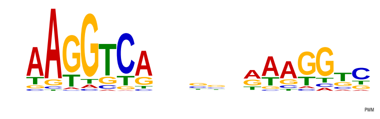	AAGGTCNNNNNNNNAGGTC	226.19	DR:7	6
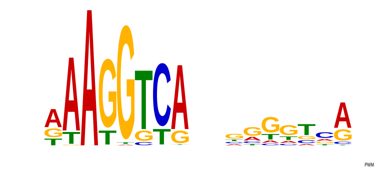	NNAAGGTCANNGGGGTCNN	1135.08	DR:3	7
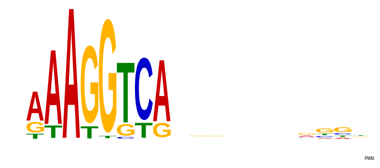	AAAGGTCANNNNNNNGGGT	1298.94	DR:7	8
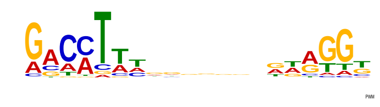	GACCTTNNNNNNNNNNAGGT	377.15	ER:11	9

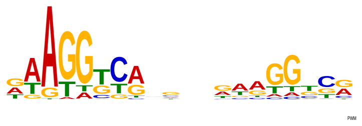	$NA\overrightarrow{AGGTCA}NNNNNN\overrightarrow{AGGTCN}$	908.00	DR:6	10
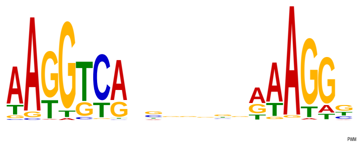	$A\overrightarrow{AGGTCA}NNNNNNNNNAA\overrightarrow{AGGG}$	3421.63	DR:9	11
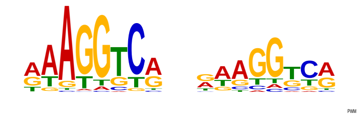	$NNA\overrightarrow{AGGTCN}NNNN\overrightarrow{AGGTCN}N$	370.38	DR:4	12
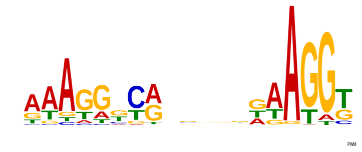	$CAA\overrightarrow{AGGGCN}NNNNNNA\overrightarrow{AGGT}$	1864.33	DR:7	13
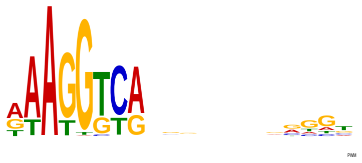	$AA\overrightarrow{AGGTCA}NNNNNNNN\overrightarrow{GGGT}$	2976.34	DR:8	14
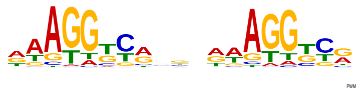	$NA\overrightarrow{AGGTCN}NNNNA\overrightarrow{AGGTCN}$	742.46	DR:5	15
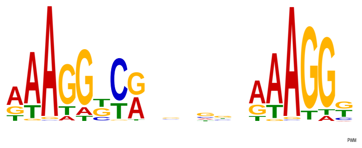	$AA\overrightarrow{AGGGCN}NNNNNNNAA\overrightarrow{AGGG}$	2688.75	DR:8	16
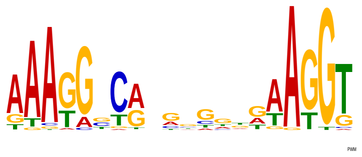	$AA\overrightarrow{AGGCCA}NNNNNNNGA\overrightarrow{AGGT}$	4560.86	DR:8	17
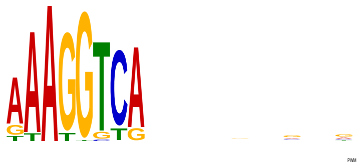	$AA\overrightarrow{AGGTCA}NNNNNNNNNGCCG$	987.50	M	18

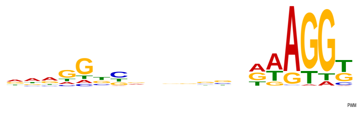	$AA\overrightarrow{AGGNNN}NNNNNNAA\overrightarrow{AGGT}$	973.71	DR:8	19
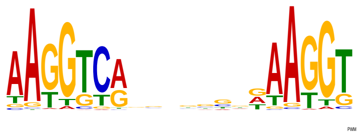	$A\overrightarrow{AGGTCN}NNNNNNNTA\overrightarrow{AGGT}$	1329.80	DR:9	20

406 NURR1-Bhimsaria2022-Rnd3 (S07-131)

PWM	Seed Sequence	Seed Seq Enrichment	Repeat	Rank
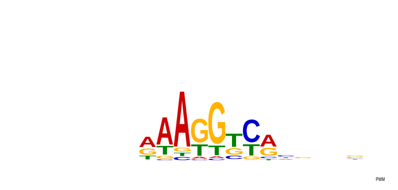	NNNNNNAAAGGTCANNNNNNN	29.38	M	1
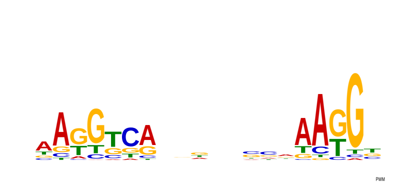	AAGGTCNNNNNNNNNAAGGT	129.81	DR:9	2
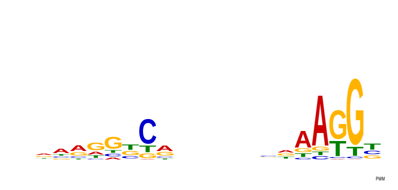	AAAGGNNNNNNNNNNAAGGT	60.37	DR:8	3
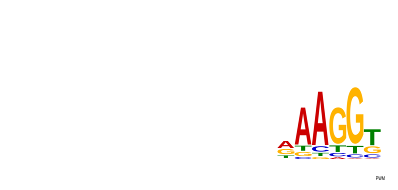	GGGGNNNNNNNNNNNAAGGT	32.60	M	4
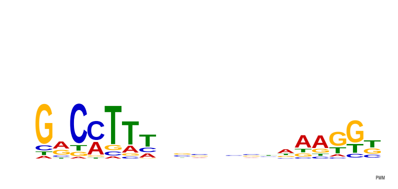	GACATTNNNNNNNNNNAGGT	37.47	ER:11	5
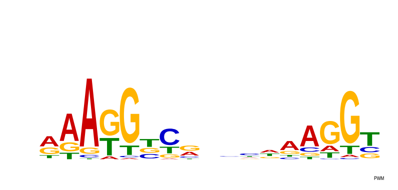	AAAGGTCNNNNNAAGGT	134.53	DR:5	6
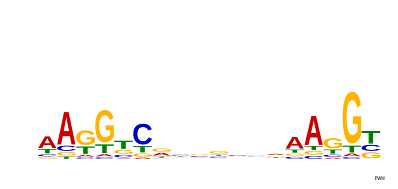	AAGGTCNNNNNNNAAGGT	79.91	DR:7	7
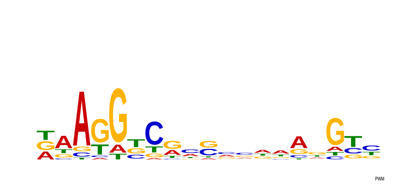	NAAGGTCNNNNNNAAGGTN	42.32	DR:6	8
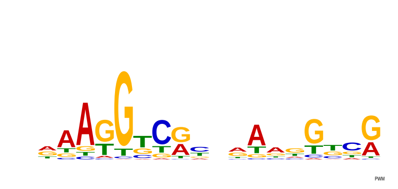	NAAGGTCNNNNNAGGTCN	47.00	DR:4	9

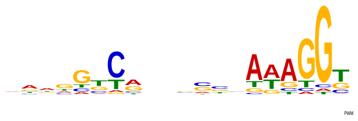	$AAAGNNNNNNNNNNNAT\overrightarrow{AGGT}$	27.58	M	10
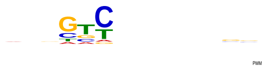	$NNNNAA\overrightarrow{AGNNNA}CAGNNNN$	3.27	M	11
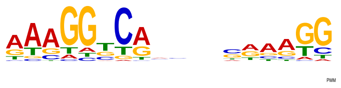	$GAA\overrightarrow{AGGTN}NNNNNNNAAGG$	51.25	M	12
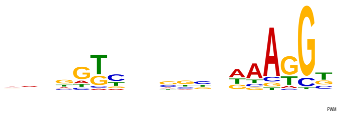	$CAAAGNNNNNNNNNNA\overrightarrow{AGGT}$	29.32	M	13
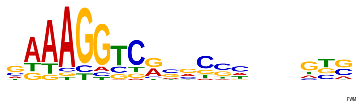	$NAA\overrightarrow{AGGTCN}NNNNNN\overrightarrow{AGGTN}$	35.85	DR:6	14
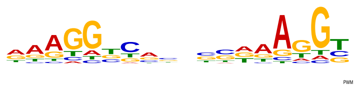	$AA\overrightarrow{AGGTN}NNNNNNA\overrightarrow{AGGT}$	50.92	DR:6	15

407 NURR1-Rep2-SMiLE-seq-Rnd1 (SRR3405072)

PWM	Seed Sequence	Seed Seq Enrichment	Repeat	Rank
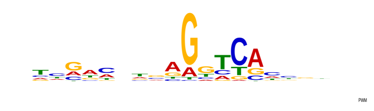	NNNNNNCTGAGGTCANNNNNNN	5.93	M	1
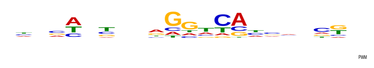	NNNNNNTGAGGTCAANNNNNNN	4.35	M	2
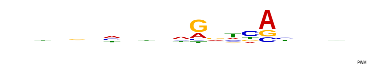	NNNNNNCTGAGGTCNNNNNN	2.59	M	3
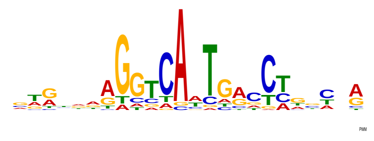	NNNNNNAGGTCANNGACCNNNNNN	25.90	IR:1	4
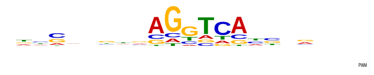	NNNNNNTGAGGTCATNNNNNN	6.21	M	5
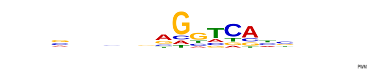	NNNNNNTGAGGTCANNNNNN	4.59	M	6
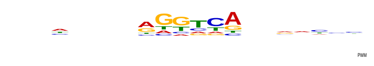	NNNNNNGAGGTCAANNNNNNN	4.02	M	7
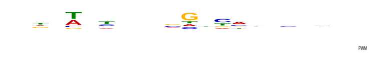	NNNNNNTGAGNTCAANNNNNNN	2.19	M	8
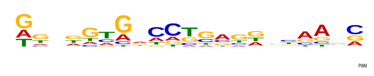	NNNNNTGACCNNAGGTNNNNN	4.39	ER:1	9

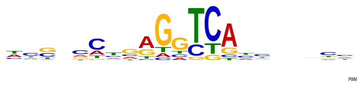	NNNNNNTGAGGTCACNNNNNN	5.98	M	10
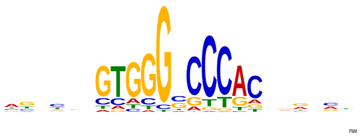	NNNNNNGTGGNNCCCACNNNNNN	9.23	M	11
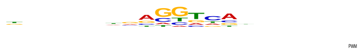	NNNNNNTGAGNTCATNNNNNN	3.10	M	12
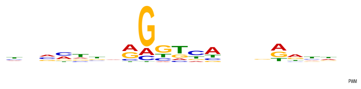	NNNNNNGAGGTCATTNNNNNN	3.99	M	13
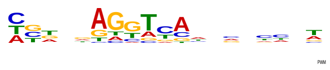	NNNNNNGAGGTCAAANNNNNN	4.43	M	14
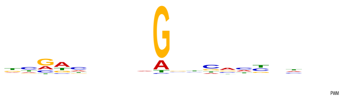	NNNNNNCTGANGTCANNNNNN	1.82	M	15
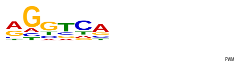	NNNNNNGAGGTCATNNNNNN	4.11	M	16
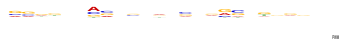	NNGGGGNNNNNNNNNNNNNNNGGGGNN	2.09	M	17
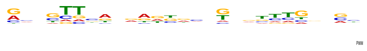	NNNNTTCANNNNNNNNNNNNTTTGNNNN	2.87	M	18

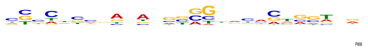	*NNNNNCCGANNNNNNNNNCACTNNNNN*	2.14	M	19
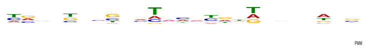	*NNNNNCCGANNNNNNNTTGGNNNNN*	1.56	M	20

408 NURR1-RXRA-Rep1-SMiLE-seq-Rnd1 (SRR3405118)

PWM	Seed Sequence	Seed Seq Enrichment	Repeat	Rank
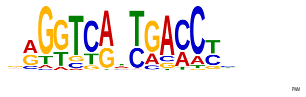	NNNNNNGGTCANTGACCNNNNNN	516.63	IR:1	1
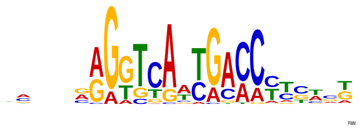	NNNNNNGGGTCAANGACCNNNNNN	670.28	IR:1	2
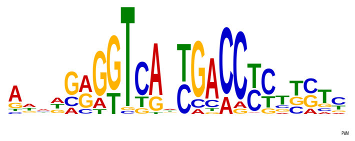	NNNNNNGGGTCANNGACCTNNNNNN	759.52	IR:1	3
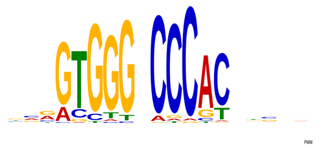	NNNNNNGTGGGNCCCANNNNNNN	40.08	M	4
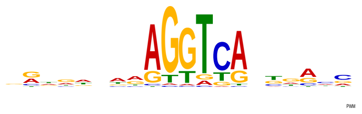	NNNNNNAAAGGTCANNNNNN	23.67	M	5
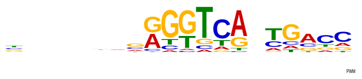	NNNNNNCGGGGTCANNNNNN	11.32	M	6
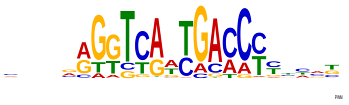	NNNNNNGGGTCANTGACCNNNNNN	875.53	IR:1	7
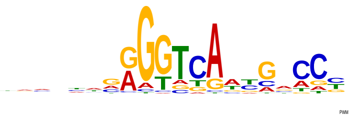	NNNNNNGGGGTCAANNNNNN	20.48	M	8

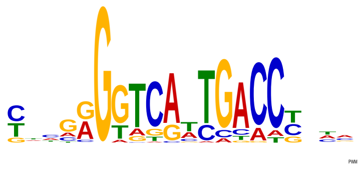	*NNNNAGTTCNNTGACCNNNN*	160.99	IR:1	9
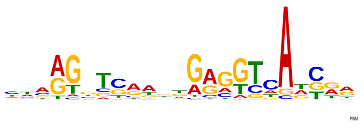	*NNNNNGTCANNNNNNGGTCNNNNN*	7.65	DR:4	10
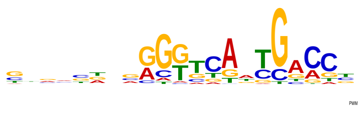	*NNNNNNCGGGNTCAANNNNNN*	7.73	M	11
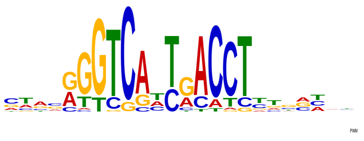	*NNNNNGGTCANNNNNCTTTNNNNN*	46.97	M	12
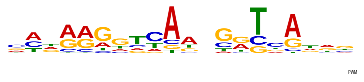	*NNNNNNGTCAAAGGNNNNNN*	7.11	DR:1	13
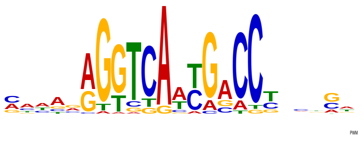	*NNNNNNGGTCAACGACCNNNNNN*	579.85	IR:1	14
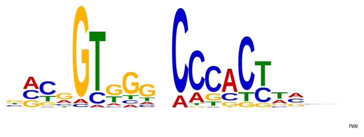	*NNNNNNGGGCNCCACNNNNNN*	3.89	M	15
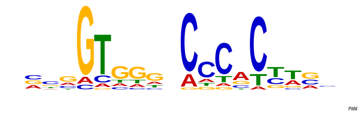	*NNNNNNGGGCCCCANNNNNN*	3.91	M	16
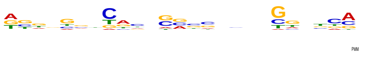	*NNGGGGNNNNNNNNNNNNNGGGTNN*	2.80	M	17

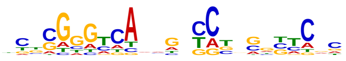 PWM	$NNNN\overrightarrow{NGGTC}NNNNNNNNNGGGGNNNNN$	3.72	M	18
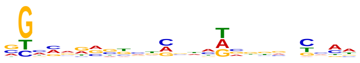 PWM	$NNNN\overrightarrow{AGGGN}NNNNNNNNNGGGGNNNN$	2.31	M	19
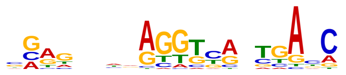 PWM	$NNNNNNAA\overrightarrow{AGNTC}AANNNNNN$	8.90	M	20

409 NURR1-Jolma2013-Rnd2 (ERR195038)

PWM	Seed Sequence	Seed Seq Enrichment	Repeat	Rank
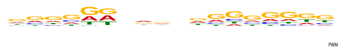	NNGGGGNNNNNNNGGGGNN	8.01	M	1
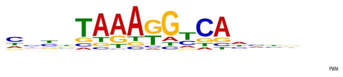	NNNNNNAAAGGTCANNNNNN	4.14	M	2
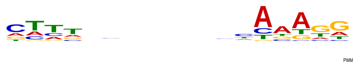	CTTTNNNNNNNNNNAAGG	3.69	M	3
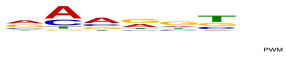	AAAAGGTC	2.31	M	4
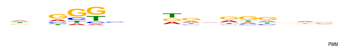	NNAGGGNNNNNNGGGANN	3.28	M	5
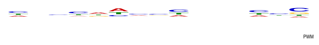	NNTCCCNNNNNCTCTNN	1.89	M	6
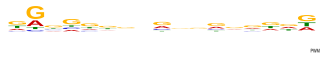	NNNNGGGGNNNGGGGNNNN	5.86	M	7
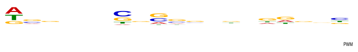	NGGTTNNNNNNNNNGGGGN	2.82	M	8
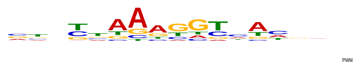	NNNNAAAAGGTCANNNN	3.50	M	9

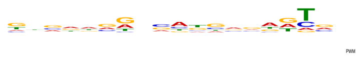	*NNNAAGGNNNNNAGAGNNN*	2.59	M	10
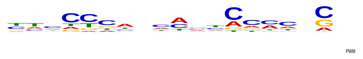	*NNCCCCNNNNNNNCACTNN*	3.41	M	11
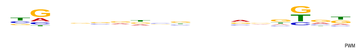	*NNNGGGTNNNCAGGNNN*	2.13	DR:2	12
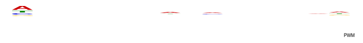	*NTTTGNNNNNNGAAAN*	1.68	M	13
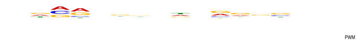	*NNNNGTGTNAAGGNNNN*	1.57	DR:0	14
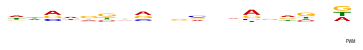	*NNAAGGNNNNNNNAAAGNN*	2.49	M	15
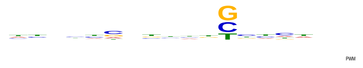	*NNTTTCNNNNNNCTCTNN*	1.68	M	16
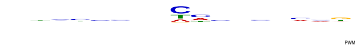	*NNCTCCNNNNNNTCACNN*	2.23	M	17
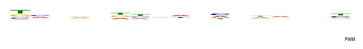	*NNAGGTNNNNNAAAGNN*	1.62	M	18

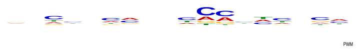	*NNCCACNNNNNNNCACTNN*	1.86	M	19
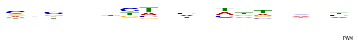	*NTCTCNNNNNNNNTTCTN*	1.68	M	20

410 NURR1-Jolma2013-Rnd3 (ERR195039)

PWM	Seed Sequence	Seed Seq Enrichment	Repeat	Rank
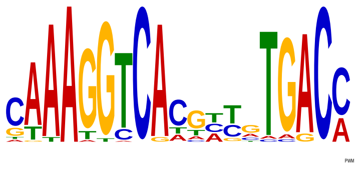	CAAAGGTCNNNNNGTGACC	5728.36	IR:5	1
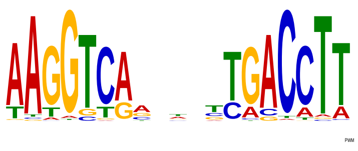	NAGGTCNNNNNNNGACCTN	198.62	IR:5	2
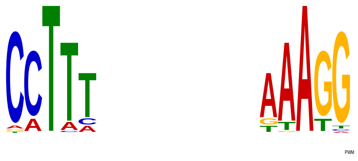	CCTTTNNNNNNNNNAAAGG	218.03	M	3
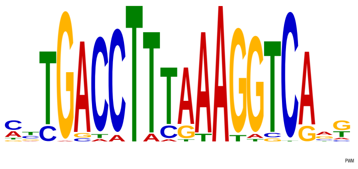	NNNGACCTTNNAAGGTCNNN	1912.43	ER:4	4
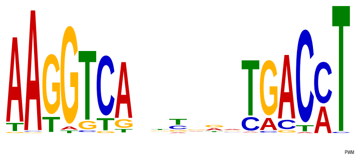	NAGGTCANNNNNNTGACCN	371.08	IR:6	5
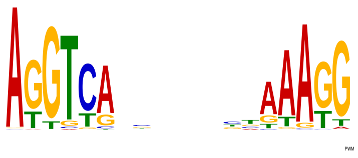	AGGTCNNNNNNNNNAAAGG	213.12	M	6
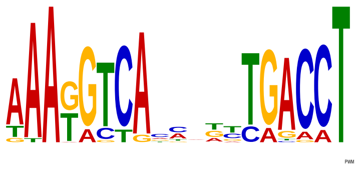	AAATGTCANNNNNNGACCT	2708.10	IR:5	7
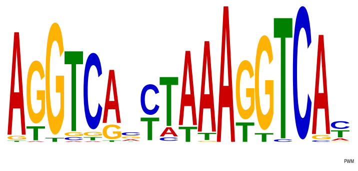	AGGTCNNNNNAAGGTCAT	1891.05	DR:5	8
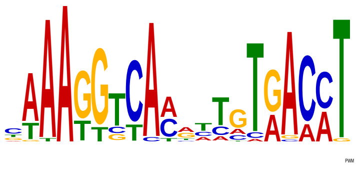	CAAAGGTCNNNNNNNGACCT	2851.19	IR:5	9

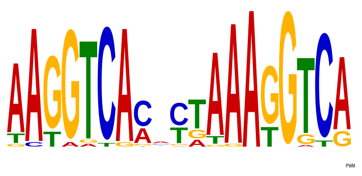	NAGGTCANNNNNNAAGGTCN	471.91	DR:6	10
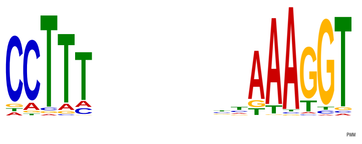	CCTTNNNNNNNNNNNNNAGGT	47.18	M	11
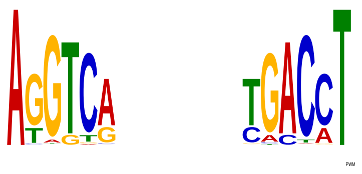	AGGTCANNNNNNNNTGACCT	586.09	IR:7	12
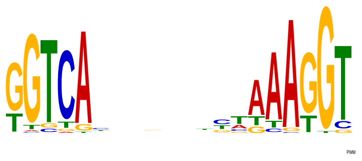	GGTCANNNNNNNNNNNAAGGT	190.78	DR:11	13
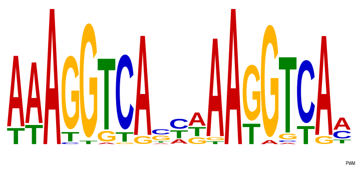	NNAGGTCANNNAAGGTCNN	483.91	DR:4	14
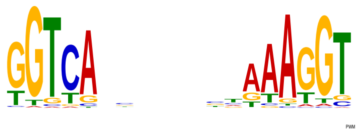	GGTCANNNNNNNNNNNNAGGT	91.20	DR:10	15
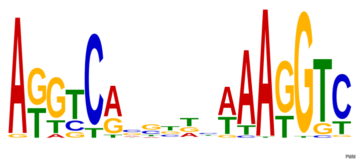	AGGTCANNNNNNNAAGGTC	327.45	DR:7	16
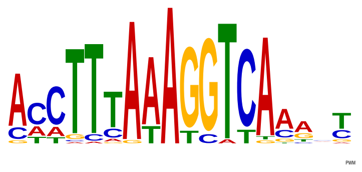	NNCTTTANAGGTCAAANN	1306.51	M	17
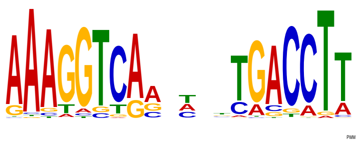	NNAGGTCNNNNNNNGACCNN	61.86	IR:5	18

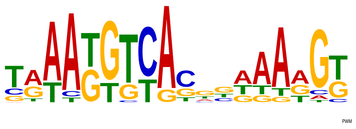	$NAA\overrightarrow{AGGTCN}NNNNAA\overrightarrow{AGGN}$	141.75	DR:5	19
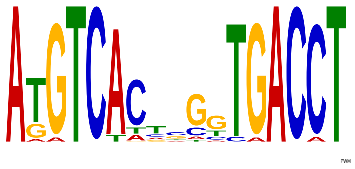	$\overrightarrow{ATGTCA}CTNNN\overleftarrow{NGACCT}$	1809.65	IR:5	20

411 NURR1-Jolma2013-Rnd4 (ERR195040)

PWM	Seed Sequence	Seed Seq Enrichment	Repeat	Rank
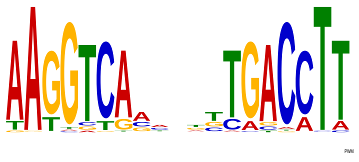	NNNGTCANNNNNTGACNNN	69.66	IR:5	1
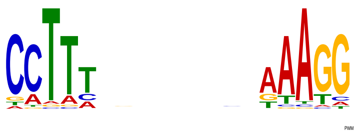	CCTTNNNNNNNNNNNAAGG	21.78	M	2
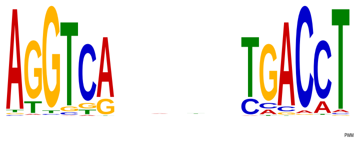	NGGTCNNNNNNNNNGACCN	27.13	IR:7	3
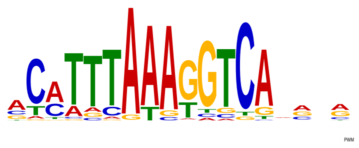	NNNTTTANNNNGTCACNNN	51.93	M	4
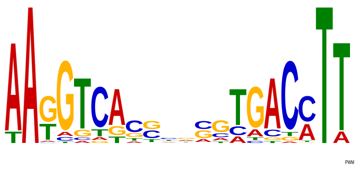	NNGGTCNNNNNNNNGACCNN	12.23	IR:6	5
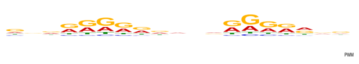	NNNGGGGNNNNNGGGGNNN	14.29	M	6
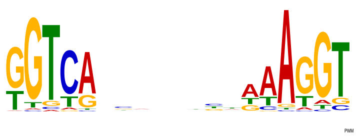	GGTCNNNNNNNNNNNAGGT	32.21	DR:10	7
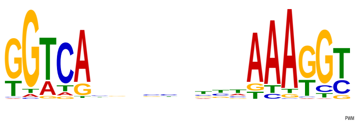	GGTCNNNNNNNNNNNNAGGT	18.55	DR:11	8
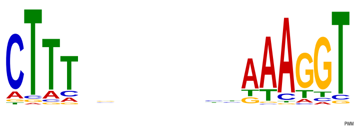	CTTTNNNNNNNNNNNAGGT	19.22	M	9

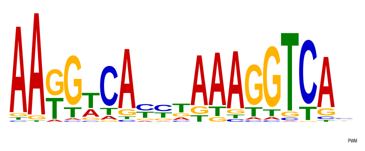	*NNNGTCANNNNNNAGGTNNN*	14.40	DR:5	10
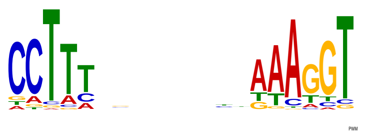	*CCTTNNNNNNNNNNNNAGGT*	22.21	M	11
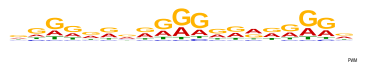	*GGGGNNNNNNNNNNNGGGG*	12.65	M	12
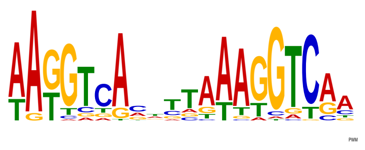	*NNNGTCANNNNNNAGGTNNN*	10.01	DR:6	13
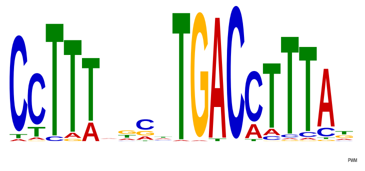	*CCTTNNNNNNNNNCTTTAT*	53.02	M	14
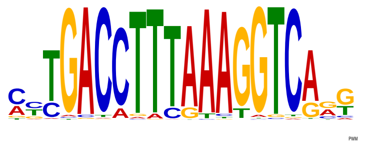	*NNNGACCNNNNNNGGTCNNN*	78.61	ER:4	15
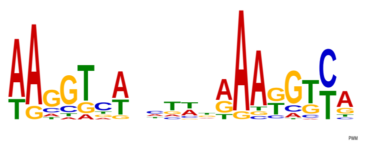	*NNGGTCNNNNNNNNAGGTNN*	4.56	DR:7	16
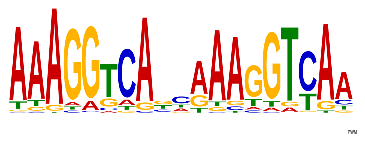	*NNNGGTCNNNNNAGGTNNN*	11.80	DR:4	17
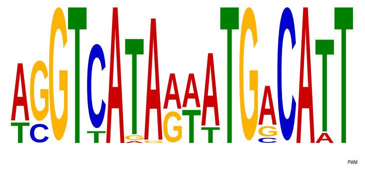	*NNGTCATANNATGACANN*	278.42	DR:4	18

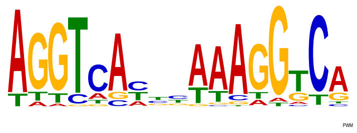	$\overrightarrow{NGGTCN}NNNNN\overrightarrow{AGGTTN}$	9.10	DR:5	19
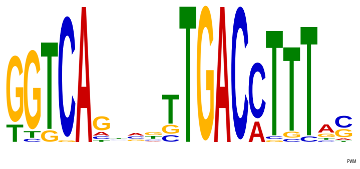	$\overrightarrow{GGTCN}NNNNNNNNNNTTAG$	27.91	M	20

412 NURR1-Yin2017-Rnd4 (ERR1005269)

PWM	Seed Sequence	Seed Seq Enrichment	Repeat	Rank
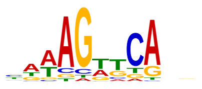	NNNNNNAAAGTTCANNNNNN	6.26	M	1
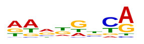	NNNNNNGTAANNGTCANNNNNN	5.14	DR:0	2
	NNNNNNGCGGNCCGANNNNNN	2.85	M	3
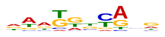	NNNNNNAAAGNNNACGTNNNNNN	3.64	M	4
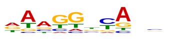	NNNNNNAAGGNNATCGNNNNNN	3.65	M	5
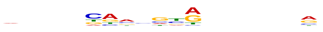	NNNNNNTCAANGTAANNNNNN	2.66	DR:0	6
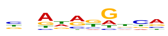	NNNNNNCAATNNGTCANNNNNN	3.17	M	7
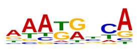	NNNNNNTAAANNCCANNNNNN	3.50	M	8
	NNNNNNGCTANNGTCANNNNNNN	3.47	M	9

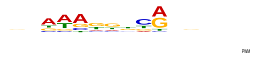	$NNNNNNCGCAN\overrightarrow{NGGTCN}NNNNN$	3.03	M	10
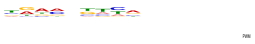	$NNNNNNTTGA\overrightarrow{NNNNTCA}ANNNNNN$	2.72	M	11
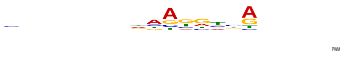	$NNNNNNCGATN\overrightarrow{NGGTCN}NNNNN$	2.13	M	12
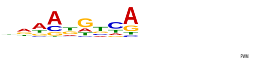	$NNNN\overrightarrow{NNGTAA}\overrightarrow{NNNNTCA}GNNNNNN$	4.07	DR:0	13
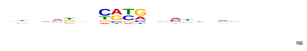	$NNNNNNTAAANNNNNNNNNNNTTTANNNNNN$	2.29	M	14
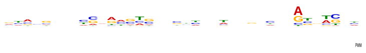	$NTACGNNNNNNNNNNNNNNNNNNNNNNNNNN\overrightarrow{NNGACA}N$	2.07	M	15
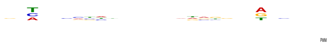	$NNNNN\overleftarrow{NGACC}NNNNNNNNNN\overrightarrow{NGGTCN}NNNNN$	2.34	ER:9	16
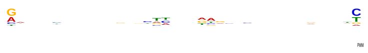	$NN\overrightarrow{AGGTNN}NNNNNNNNNNNNNNNNNNNNNNNNN\overleftarrow{NNACCT}NN$	1.52	IR:23	17
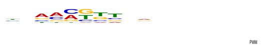	$NNNN\overrightarrow{NNGTGA}\overrightarrow{NNNNTCA}CNNNNNN$	2.57	DR:1	18

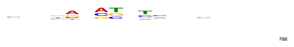	$NNNNNNTTGAN\overrightarrow{NNNTC}AANNNNNNN$	2.29	M	19
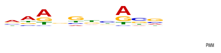	$NNNNNNGCGAN\overrightarrow{NGGGC}NNNNNN$	2.25	M	20

413 NURR77-Yin2017-Rnd2 (ERR1005253)

PWM	Seed Sequence	Seed Seq Enrichment	Repeat	Rank
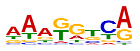	NNNNNNGTAANNGTCANNNNNN	7.68	DR:0	1
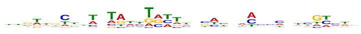	NNNTTGCNNNNNNNNNNNNNNNNNNNNNNNNTCGTNNN	2.66	M	2
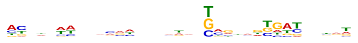	NNTTAANNNNNNNNNNNNNNNNNNNNNNNATTANN	2.46	M	3
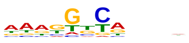	NNNNNNAAAGNNNAAACNNNNNN	3.94	M	4
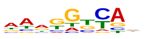	NNNNNNAAAGNNNAACGNNNNNN	4.44	M	5
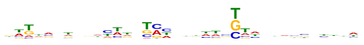	NTTTANNNNNNNNNNNNNNNNNNNNNNNNNNTTTTN	2.47	M	6
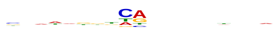	NNNNNNAAAGNNNNNGACANNNNNN	2.75	M	7
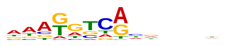	NNNNNNTAAANNNCGCGNNNNNN	3.70	M	8
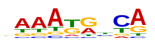	NNNNNNAAATNCCAANNNNNN	4.66	M	9

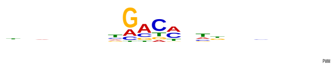	*NNNNNNTGCTNNNNTTTANNNNNN*	2.59	M	10
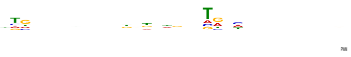	*NNNNNNCTTTNNNNNNNNNNNNNNNCTTTNNNNNN*	2.56	M	11
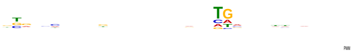	*NNNNNNCTTTNNNNNNNNNNNNNNNNNCTTTNNNNNN*	3.11	M	12
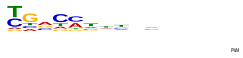	*NNNNNNTACGNNNTTTANNNNNN*	3.28	M	13
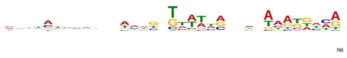	*NNNNTTATNNNNNNNNNNNNNNNNNNNNNAAATNNNN*	3.25	M	14
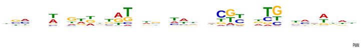	*NNATGTNNNNNNNNNNNNNNNNNNNNNNNNNCTTTNN*	2.46	M	15
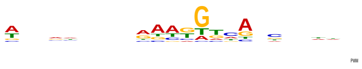	*NNNNNNCGTAANNNTCAGNNNNNN*	4.74	DR:0	16
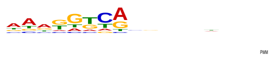	*NNNNNNAAAGNNNACGANNNNNN*	4.22	M	17
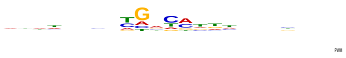	*NNNNNNTCGTNNNNTTTANNNNNN*	3.11	M	18

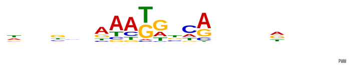	$NNNNNNTAA\overrightarrow{ANNNC}AATGNNNNNN$	5.72	M	19
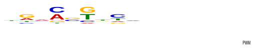	*NNNNNNTTAANNNTTAANNNNNNN*	3.05	M	20

414 NURR77-Yin2017-Rnd3 (ERR1005257)

PWM	Seed Sequence	Seed Seq Enrichment	Repeat	Rank
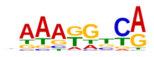	*NNNNNNGTAANNGTCANNNNNN*	8.78	DR:0	1
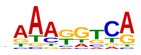	*NNNNNNGTAANNNNTCAANNNNNN*	8.20	DR:0	2
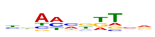	*NNNNNNATGANNNNTCATNNNNNN*	3.99	M	3
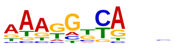	*NNNNNNAAAGNNNAAACNNNNNN*	5.45	M	4
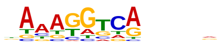	*NNNNNNCGAANNNNTCAANNNNNN*	5.98	M	5
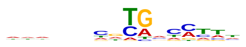	*NNNNNNGTCGNNNNCTTTNNNNNN*	3.85	M	6
	NNNNNNACCTNNNNNNNNAGGTNNNNNN	3.44	ER:8	7
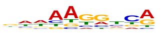	*NNNNNNGATANNNNTCATNNNNNN*	3.18	M	8
	NNNNNNACCTNNNNNNNNNAGGTNNNNNN	3.39	ER:9	9

	NNNNNN$\overleftarrow{TGACN}$NNNNNNNNNNNNNN$\overrightarrow{NGTCA}$NNNNNN	2.91	ER:11	10
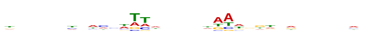	NNNNNN$\overleftarrow{TGACN}$NNNNNNNNNNNNN$\overrightarrow{NGTCA}$NNNNNN	3.36	ER:10	11
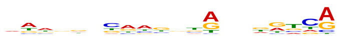	NNNNNNCA$\overrightarrow{AGNTAA}$ANNNNNN	3.81	M	12
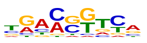	NNNNN$\overrightarrow{NGTAA}$NNN$\overleftarrow{TTACN}$NNNNN	4.04	IR:3	13
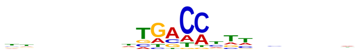	NNNNNNCGTTNNNNTTTTNNNNNN	3.64	M	14
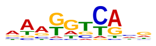	NNNNNNTAAANNNN$\overrightarrow{ACGTN}$NNNNN	4.93	M	15
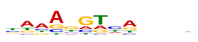	NNNNNNTTAANNNNCAGANNNNNN	3.19	M	16
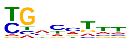	NNNNNNTACGNNNTTTANNNNNN	3.56	M	17
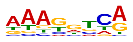	NNNNNNCGTANN$\overrightarrow{NGTCG}$NNNNNN	3.92	M	18

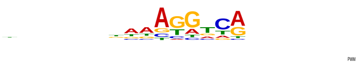	*NNNNNNCTAANNNTCGTNNNNNN*	3.65	M	19
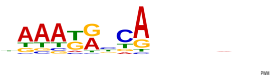	$NNNNNNAA\overrightarrow{ATNCCA}ANNNNNN$	5.20	M	20

415 NURR77-Yin2017-Rnd4 (ERR1005259)

PWM	Seed Sequence	Seed Seq Enrichment	Repeat	Rank
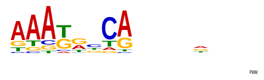	*NNNNNNTAAANNNCAAGNNNNNN*	7.55	M	1
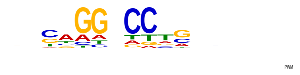	*NNNNNNCAGGNCCTGNNNNNN*	7.28	M	2
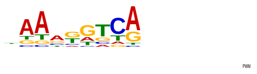	*NNNNNNGTAANNNTCAGNNNNNN*	6.07	DR:0	3
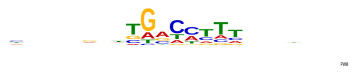	*NNNNNNGTTTNNNNTTTTNNNNNN*	4.44	M	4
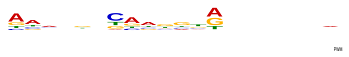	*NNNNNNTCAANGTAANNNNNN*	3.49	DR:0	5
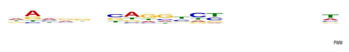	*NNNNNNTCAGNTCTGNNNNNN*	2.95	M	6
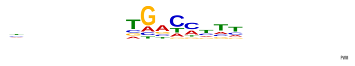	*NNNNNNTGTTNNNNTTTTNNNNNN*	3.51	M	7
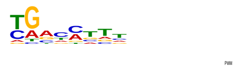	*NNNNNNTCGTNNNNTTTANNNNNN*	3.63	M	8
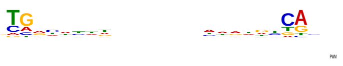	*NNNNNNATTTNNNNNNNAAATNNNNNN*	3.00	M	9

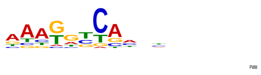 PWM	*NNNNNNTAAANNNNAAGTNNNNNN*	5.04	M	10
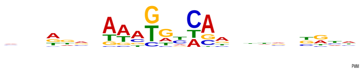 PWM	*NNNNNNAAAANNNNAAATTNNNNNN*	5.36	M	11
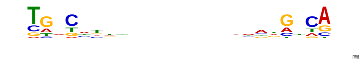 PWM	*NNNNNNATTTNNNNNNNNAAATNNNNNN*	2.83	M	12
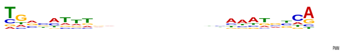 PWM	*NNNNNNATTTNNNNNNNNNNNNAAATNNNNNN*	3.50	M	13
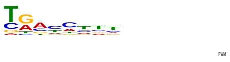 PWM	*NNNNNNGAATNNNNTTTTNNNNNN*	2.75	M	14
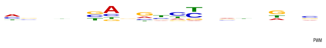 PWM	*NNNNNNTGAGNTCTANNNNNN*	2.72	M	15
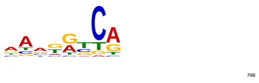 PWM	*NNNNNNTAAANNNNACAGNNNNNN*	3.30	M	16
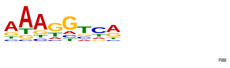 PWM	*NNNNNNCGTANNNNTCAGNNNNNN*	3.53	M	17
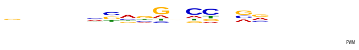 PWM	*NNNNNNGAGGNCCTANNNNNN*	2.68	M	18

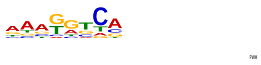	*NNNNNNTAAANNNNNAATCNNNNNN*	3.73	M	19
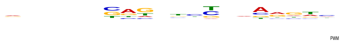	$NNNNNNAC\overrightarrow{AGNTCT}ANNNNNN$	2.49	M	20

416 NURR77-Yin2017-Rnd4 (ERR1005260)

PWM	Seed Sequence	Seed Seq Enrichment	Repeat	Rank
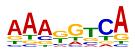	NNNNNNGTAANNNTCAANNNNNN	6.06	DR:0	1
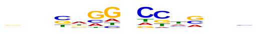	NNNNNNCAGGNCCTGNNNNNN	4.07	M	2
	NNNNNNGGGGNNNNNGGGGNNNNNN	2.85	M	3
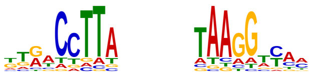	NNNNNNCCTTANNNNNNTAAGGNNNNNN	10.42	ER:10	4
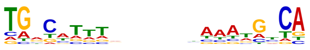	NNNNNNATTTNNNNNNNAAATNNNNNN	3.46	M	5
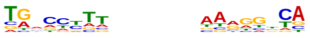	NNNNNNCTTTNNNNNNNAAAGNNNNNN	3.92	M	6
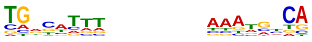	NNNNNNATTTNNNNNNNNAAATNNNNNN	3.56	M	7
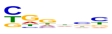	NNNNNNACGGTCCGNNNNNN	2.64	M	8
	NNNNNNGACCNNNNNNNNNNNGGTCNNNNNN	3.09	ER:10	9

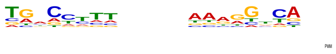	*NNNNNNCTTTNNNNNAAAGNNNNNN*	3.64	M	10
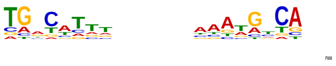	*NNNNNNATTTNNNNNNAAATNNNNNN*	3.46	M	11
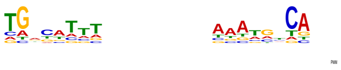	*NNNNNNATTTNNNNNNNNNAAATNNNNNN*	3.40	M	12
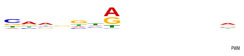	*NNNNNNACA$\overrightarrow{ANGTA}$ANNNNNN*	2.59	M	13
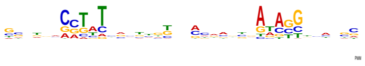	*NNC$\overleftarrow{TGANNN}$NNNNNNNNNNNNNNNNNNNNNN$\overrightarrow{NNNTCA}$GNN*	1.81	ER:20	14
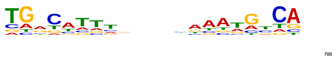	*NNNNNNATTTNNNNNAAATNNNNNN*	3.06	M	15
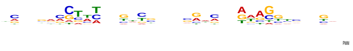	*NNNT$\overleftarrow{TGANNN}$NNNNNNNNNNNNNNNNNNNN$\overrightarrow{NNNTCA}$ANNN*	1.64	ER:19	16
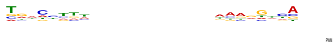	*NNN$\overleftarrow{NNNCCT}$TNNNNNNNNNNNNNNA$\overrightarrow{AGGNNN}$NNN*	2.93	ER:16	17
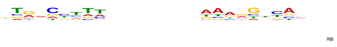	*NNN$\overleftarrow{NNNCCT}$TNNNNNNNNNNNA$\overrightarrow{AGGNNN}$NNN*	2.85	ER:12	18

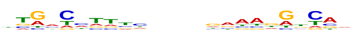 PWM	*NNNNNNTTTCNNNNGAAANNNNNN*	3.02	M	19
PWM	*NNNNNNACCTNNNNNNNNNNNNNNNNNAGGTNNNNNN*	1.97	ER:17	20

417 NURR77-Yin2017-Rnd4 (ERR1005261)

PWM	Seed Sequence	Seed Seq Enrichment	Repeat	Rank
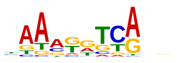 PWM	NNNNNNGTAANNNTCACNNNNNN	8.67	DR:0	1
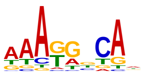 PWM	NNNNNNAAAANNNCAACNNNNNN	5.84	M	2
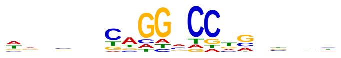 PWM	NNNNNNCAGGNCCTCNNNNNN	5.47	M	3
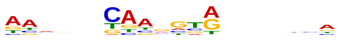 PWM	NNNNNNTCAANGTAANNNNNN	5.59	DR:0	4
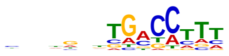 PWM	NNNNNNGTTTNNNNTTTTNNNNNN	4.98	M	5
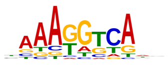 PWM	NNNNNNGTAANNNNCAACNNNNNN	5.95	M	6
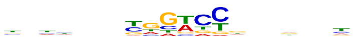 PWM	NNNNNNTTGGTCCGNNNNNN	3.88	M	7
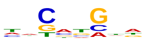 PWM	NNNNNNTACATGTANNNNNN	3.43	M	8
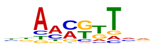 PWM	NNNNNNGTGANNNNTCACNNNNNN	3.98	DR:1	9

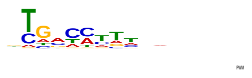	NNNNNNTCGTNNNNTTTTNNNNNN	3.93	M	10
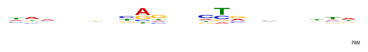	NNNNNNNTCAGNNCTGANNNNNNN	3.48	IR:4	11
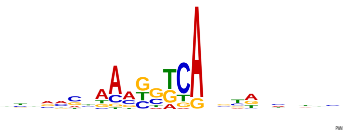	NNNNNNGTAANNNNCAGGTANNNNNN	10.24	DR:5	12
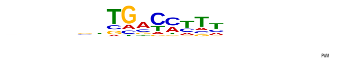	NNNNNNTGTTNNNNTTTTNNNNNN	4.21	M	13
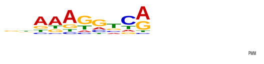	NNNNNNCGTANNNNTCGTNNNNNN	3.57	M	14
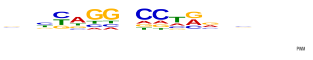	NNNNNNTAGGNCCTANNNNNN	4.17	M	15
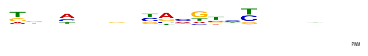	NNNNNNTTCANGTCTNNNNNN	2.89	M	16
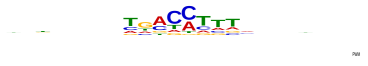	NNNNNNTCTGNNNNTTCGNNNNNN	2.85	M	17
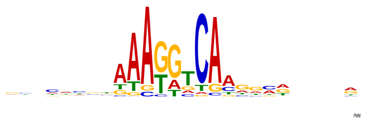	NNNNNNGTAANNNNCAAGGCNNNNNN	21.29	DR:6	18

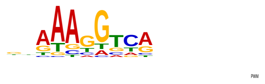	$NNNNNNCGTAN\overrightarrow{NNNTCA}GNNNNNNN$	5.00	M	19
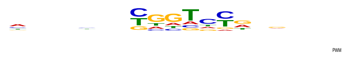	$NNNNNNTA\overrightarrow{CGGTCC}NNNNNN$	2.82	M	20

418 GCNF-Yin2017-Rnd2 (ERR1005288)

PWM	Seed Sequence	Seed Seq Enrichment	Repeat	Rank
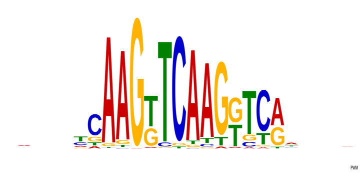	NNNNNNCAAGTTCNAGGTCNNNNNN	1775.29	DR:0	1
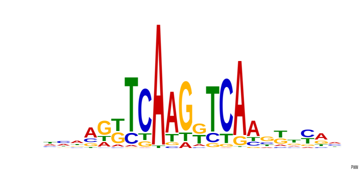	NNNNNNAGGTCAANNTCAAGNNNNNN	966.96	DR:0	2
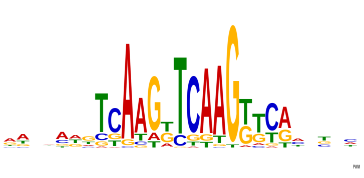	NNNNNNGTCAANNTCAAGTTCNNNNNN	2968.11	M	3
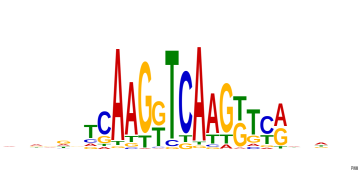	NNNNNNTCAANNTCAAGGTCNNNNNN	1641.57	M	4
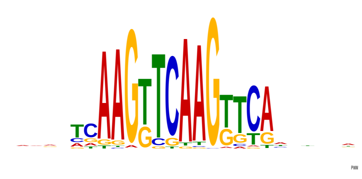	NNNNNNCAAGTNNAAGTTCANNNNNN	1674.79	DR:0	5
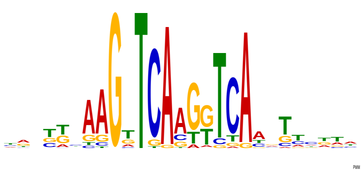	NNNNNNAAGTTCANNGTCAAGNNNNNN	1909.52	DR:0	6
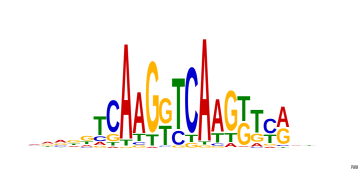	NNNNNNGTCAANNNCAAGGTCNNNNNN	857.28	M	7
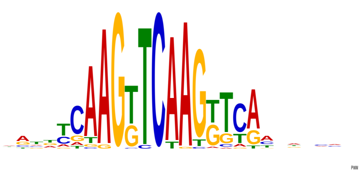	NNNNNNAAGTTCNAGGTCACNNNNNN	1866.26	DR:0	8
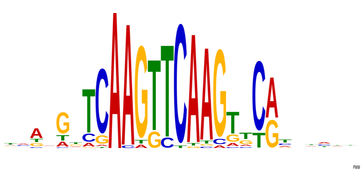	NNNNNNTCAAGNNCAAGTGCGNNNNNN	1355.85	M	9

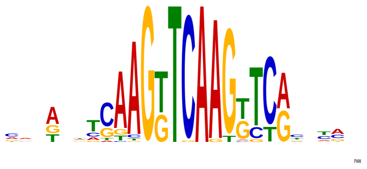	NNNNNNTGAAGTTCNAGGTCNNNNNN	1259.59	ER:3	10
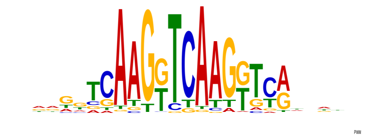	NNNNNNTCAANNNNAAGGTCNNNNNN	174.42	DR:6	11
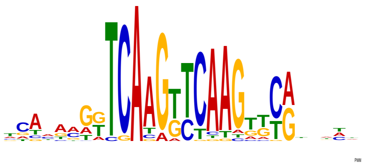	NNNNNNGGTCAANNNCAAGTGCNNNNNN	948.35	M	12
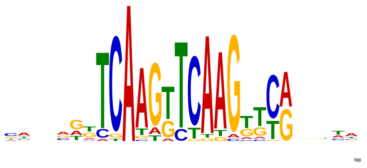	NNNNNNGTCAANNTCAAGTGCNNNNNN	1402.61	M	13
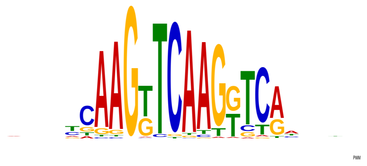	NNNNNNAAGTTCNAGGTCNNNNNN	737.77	DR:0	14
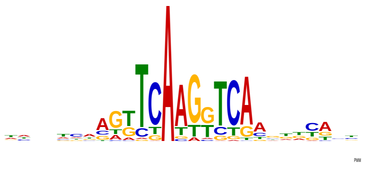	NNNNNNAAGGTCANNNTCAAGNNNNNN	645.91	DR:0	15
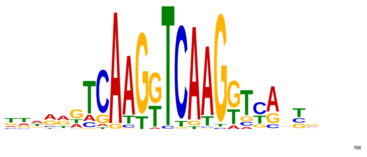	NNNNNNTCAANNNCAAGGTTANNNNNN	668.55	M	16
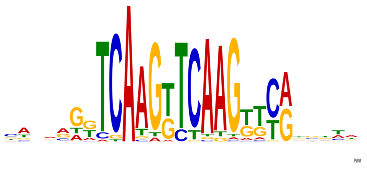	NNNNNNGTCAANNNCAAGTGCNNNNNN	452.32	M	17
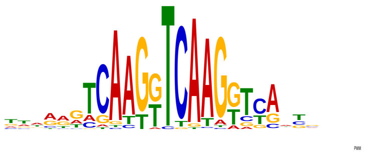	NNNNNNTCAANNNNAAGGTTANNNNNN	206.62	DR:6	18

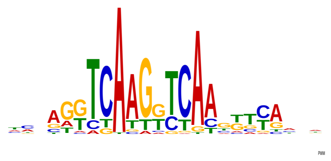	*NNNNNNAGGTCAANNNCAAGGNNNNNNN*	491.76	M	19
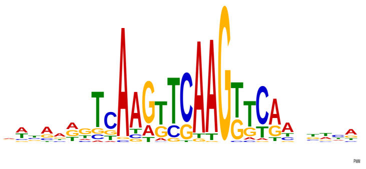	*NNNNNNGTCAANNNNNAAGTTCANNNNNNN*	847.79	DR:6	20

419 GCNF-Yin2017-Rnd2 (ERR1005289)

PWM	Seed Sequence	Seed Seq Enrichment	Repeat	Rank
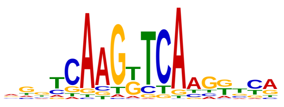	NNNNNNTCAAGNTCAAGNNNNNN	58.39	DR:0	1
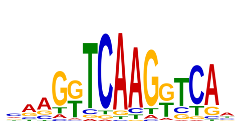	NNNNNNAGGTCNAGGTCNNNNNN	37.77	DR:0	2
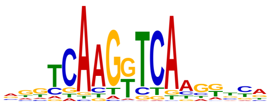	NNNNNNGTCAANNTCAAGNNNNNN	44.52	DR:0	3
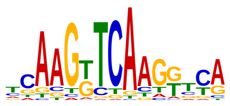	NNNNNNCAAGNNCAAGGTNNNNNN	36.40	DR:0	4
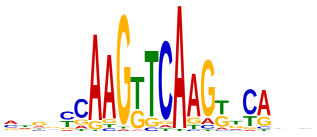	NNNNNNCAAGTNCAAGTGCNNNNNN	83.54	DR:0	5
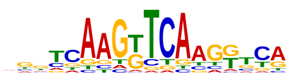	NNNNNNCAAGNNCAAGNNNNNN	11.79	M	6
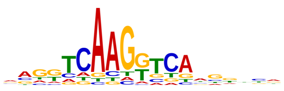	NNNNNNGGTCNNNNTCAANNNNNN	5.97	DR:0	7
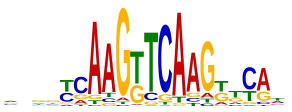	NNNNNNCAAGNNCAAGTGCNNNNNN	40.02	DR:0	8
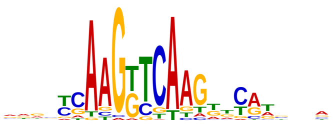	NNNNNNTCAAGNNCAAGTGCNNNNNN	75.25	M	9

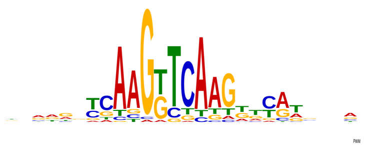	NNNNNNTCAANNNCAAGTGCNNNNNN	22.54	M	10
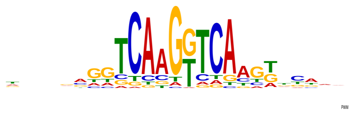	NNNNNNCGGTCANNNTCAAGNNNNNN	31.42	DR:0	11
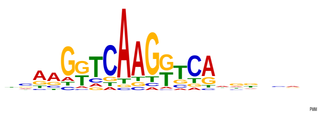	NNNNNNAGTTCNNNNTCAANNNNNN	9.05	DR:0	12
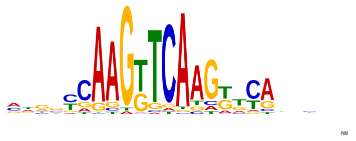	NNNNNNCAAGTNNAAGTGCNNNNNN	26.30	DR:0	13
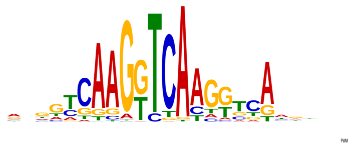	NNNNNNCAAGNNCAAGGCCNNNNNN	30.07	DR:0	14
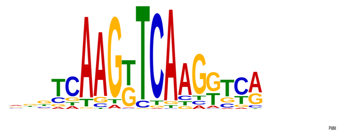	NNNNNNCAAGNNCAAGGTTNNNNNN	41.91	DR:0	15

420 GCNF-Yin2017-Rnd3 (ERR1005290)

PWM	Seed Sequence	Seed Seq Enrichment	Repeat	Rank
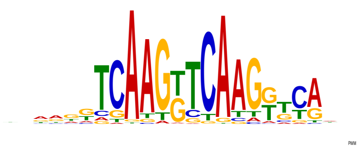	NNNNNNTCAANNNCAAGNNNNNN	248.79	DR:0	1
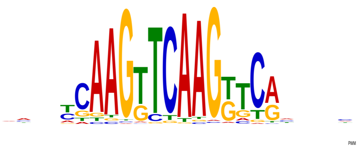	NNNNNNAAGTNNNAGTTCNNNNNN	291.78	DR:1	2
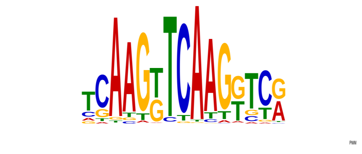	NNNNNNTCAANNNNNAGGTCNNNNNN	302.52	DR:6	3
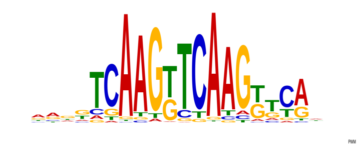	NNNNNNTCAANNNNAAGTNNNNNN	169.73	DR:5	4
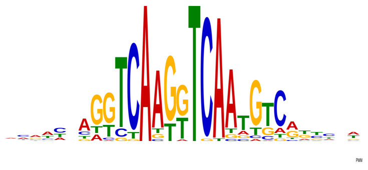	NNNNNNAGGTCNNNNTCAATGTCNNNNNN	2199.94	M	5
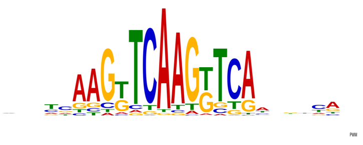	NNNNNNAGTTNNNNNTCAANNNNNN	95.08	DR:0	6
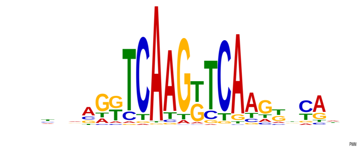	NNNNNNAGGTCNNNNNCAAGNNNNNN	168.95	M	7
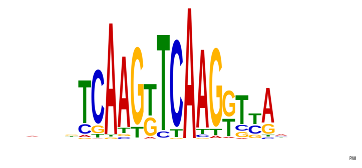	NNNNNNTCAANNNNAAGGTTANNNNNN	1069.74	DR:6	8
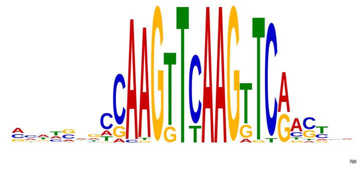	NNNNNNAGCCAAGTNNNAGTTCNNNNNN	1871.79	DR:6	9

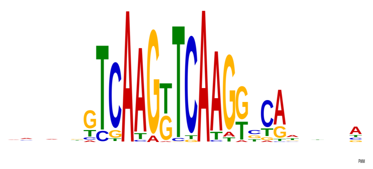	NNNNNNGTCAANNNNNAAGGCCANNNNNNN	1312.56	DR:6	10
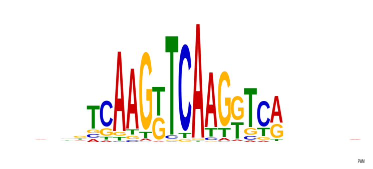	NNNNNNTCAANNNNNNNNGTCANNNNNN	76.18	DR:6	11
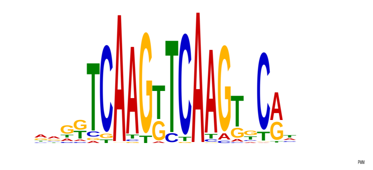	NNNNNNTCAANNNNNAAGTGCGNNNNNN	738.92	DR:6	12
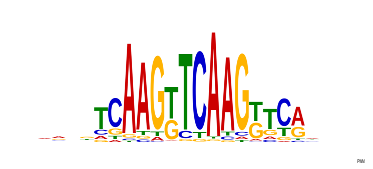	NNNNNNTCAANNNNNNAGTTNNNNNN	109.99	DR:6	13
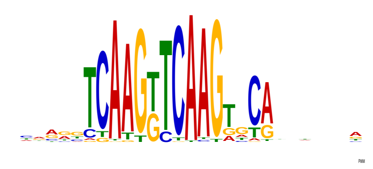	NNNNNNTCAANNNNNAAGTGCAGNNNNNN	1077.29	DR:6	14
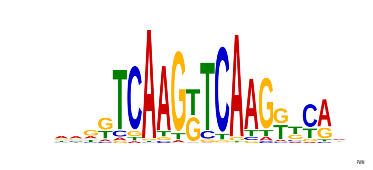	NNNNNNGTCANNNNNCAAGNNNNNN	169.42	M	15
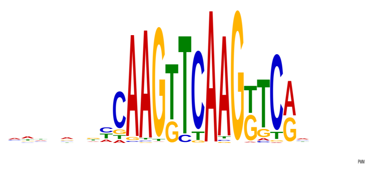	NNNNNNGCCAAGNNNNAGTTCNNNNNN	504.10	DR:6	16
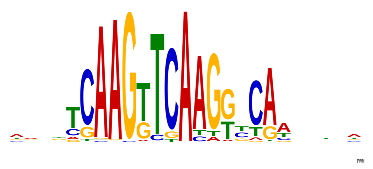	NNNNNNAAGTNNAAGGCCANNNNNN	627.20	DR:0	17
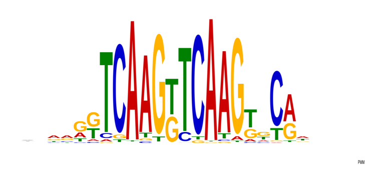	NNNNNNGTCANNNNNAAGTGCNNNNNN	290.92	DR:6	18

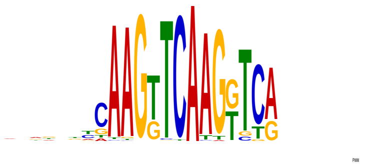	NNNNNNCCAAGTNNNNNGTCANNNNNNN	202.33	DR:1	19
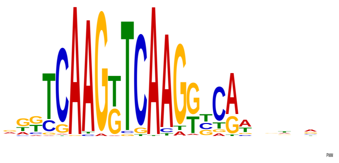	NNNNNNCAAGNNNAAGGCCATNNNNNN	965.62	DR:0	20

421 GCNF-Yin2017-Rnd3 (ERR1005291)

PWM	Seed Sequence	Seed Seq Enrichment	Repeat	Rank
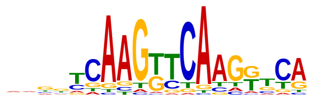	NNNNNNCAAGNNCAAGNNNNNN	28.82	M	1
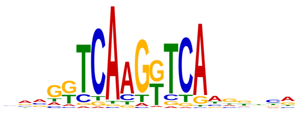	NNNNNNGGTCANNNTCAANNNNNN	48.11	DR:0	2
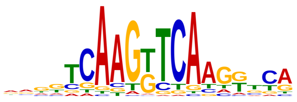	NNNNNNTCAANNNCAAGNNNNNN	28.57	DR:0	3
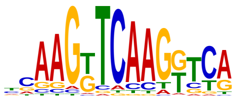	NNNNNNAGTTNNAGGTCNNNNNN	35.04	DR:0	4
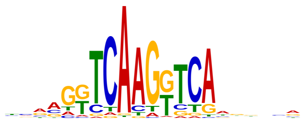	NNNNNNAGGTCNNNNTCAANNNNNN	33.03	DR:0	5
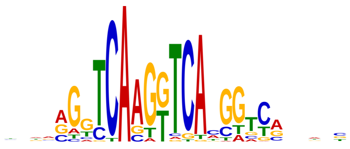	NNNNNNGGTCANNNTCACGGTCNNNNNN	269.99	M	6
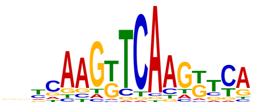	NNNNNNCAAGNNNNAGGTNNNNNN	12.65	M	7
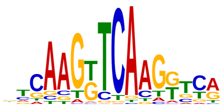	NNNNNNTCAANNNNNAGGTCNNNNNN	21.88	DR:6	8
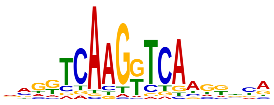	NNNNNNGGTCNNNNNCAAGNNNNNN	12.04	M	9

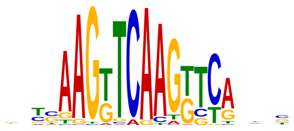	NNNNNNTGAAGTTNNAGGTCNNNNNN	158.17	ER:3	10
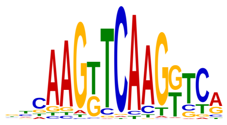	NNNNNNCCAAGNNNNAGGTCNNNNNN	37.65	M	11
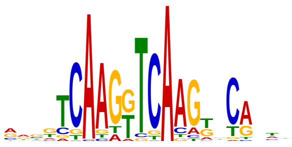	NNNNNNTCAANNNCAAGTGCGNNNNNN	106.11	M	12
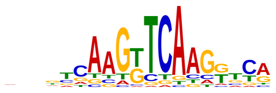	NNNNNNTCAANNNNNAGTTNNNNNN	8.90	DR:6	13
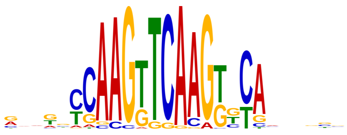	NNNNNNCAAGTNNAAGTGCACNNNNNN	229.12	DR:0	14
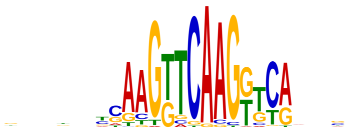	NNNNNNCTCAAGNNNNAGTTCNNNNNN	44.06	DR:6	15
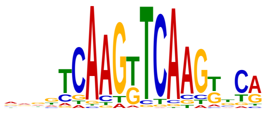	NNNNNNTCAANNNNAAGTGCNNNNNN	33.74	DR:6	16
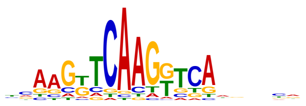	NNNNNNAGTTNNNNNTCAANNNNNN	9.93	DR:0	17
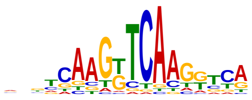	NNNNNNTCAANNNNNNGGTCNNNNNN	8.23	DR:6	18

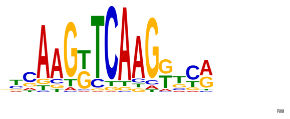	$NNN\overrightarrow{NNNTCA}ANNNNNN\overrightarrow{AGTTCN}NNNNNN$	18.99	DR:6	19
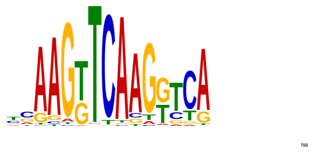	$NNNNNNA\overrightarrow{AGTTNN}\overrightarrow{NGGTCA}GNNNNNN$	67.75	DR:0	20

422 GCNF-Yin2017-Rnd4 (ERR1005292)

PWM	Seed Sequence	Seed Seq Enrichment	Repeat	Rank
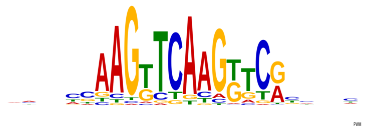	NNNNNNAAGTNNNNGTTCNNNNNN	51.21	DR:1	1
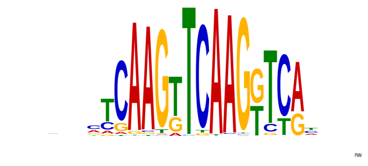	NNNNNNCTCAANNNNNAGGTNNNNNN	100.16	DR:6	2
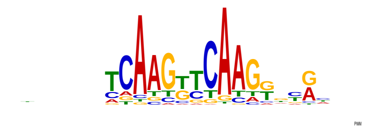	NNNNNNGTCANNNNNAAGTNNNNNN	41.32	DR:5	3
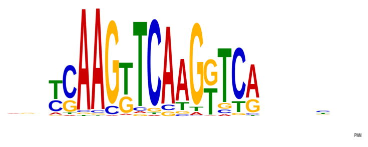	NNNNNNAGTTNNNNNTCAGNNNNNN	41.68	DR:0	4
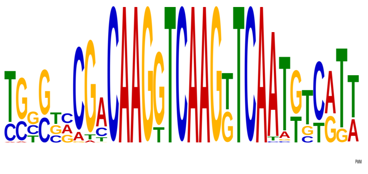	NNNNNNCGACAAGNNNNNNNTCAATNNNNNN	1098.53	DR:6	5
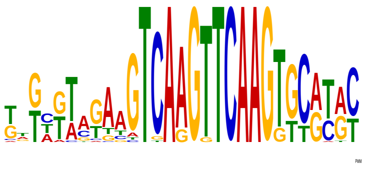	NNNNNNAGAAGTCANNNNNAAGTNNNNNN	597.07	DR:5	6
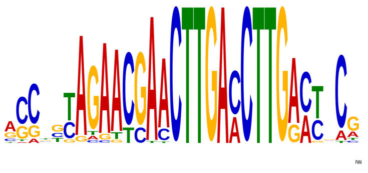	NNNNNNAGAACGAANNNNNNCTTGNNNNNN	615.05	M	7
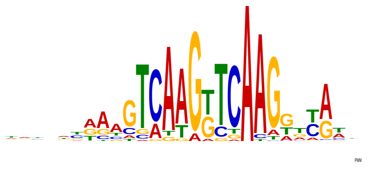	NNNNNNAAAGTCNNNNNCAAGNNNNNN	111.63	M	8
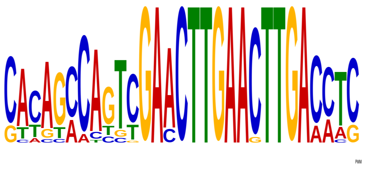	NNNNNNCAGTCGAANNNNNACTTNNNNNN	254.62	IR:5	9

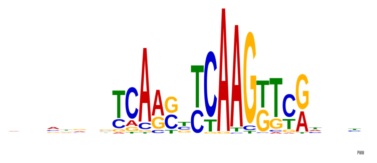	NNNNNNAGTCANNNNNNAGTTNNNNNN	48.62	DR:6	10
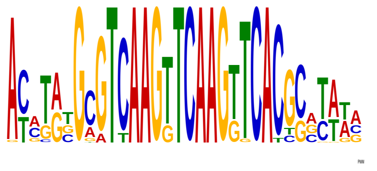	NNNNNNGCGTCAANNNNNNNNTTCACGNNNNNN	904.11	DR:6	11
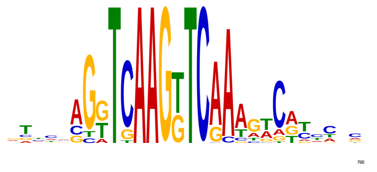	NNNNNNGGTCNNNNTCAAAGTCNNNNNN	361.12	M	12
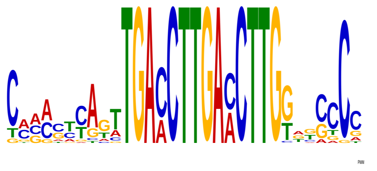	NNNNNNTAGTTGANNNNNNNCTTGGNNNNNN	310.36	M	13
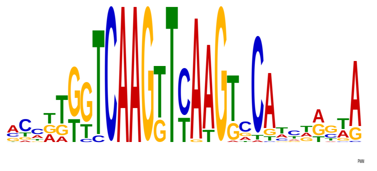	NNNNNNGTCANNNNNAAGTCCATNNNNNN	376.34	DR:6	14
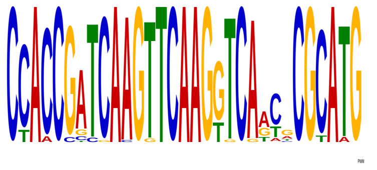	NNNNNNATCAANNNNNNNGTCAACGNNNNNN	466.04	DR:6	15
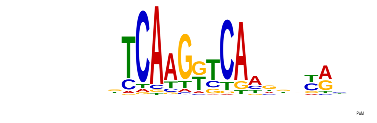	NNNNNNGGTCNNNNNCAAGNNNNNN	38.48	M	16
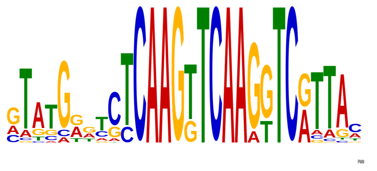	NNNNNATACTCAANNNNNNGGTCNNNNN	216.46	DR:6	17
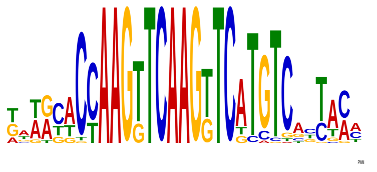	NNNNNNCCAAGNNNNNNTTCATGTCNNNNNN	1369.30	M	18

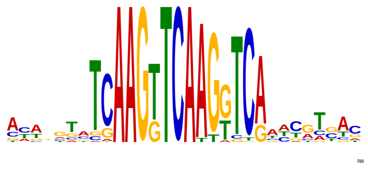	$NNNN\overrightarrow{NNATCA}ANNNN\overrightarrow{NNNTCA}AANNNNNNN$	113.95	DR:6	19
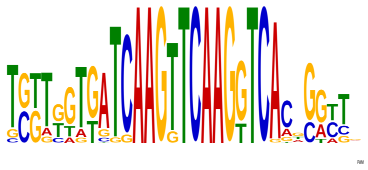	$NNNNNN\overrightarrow{TGATCA}ANNNNN\overrightarrow{NNGTCA}CNNNNNN$	296.04	DR:6	20

423 GCNF-Yin2017-Rnd4 (ERR1005293)

PWM	Seed Sequence	Seed Seq Enrichment	Repeat	Rank
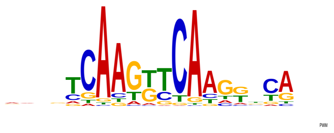	*NNNNNNTCAANNNCAAGNNNNNN*	50.88	DR:0	1
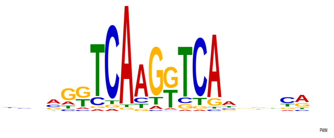	*NNNNNNGGTCNNNNTCAANNNNNN*	37.32	DR:0	2
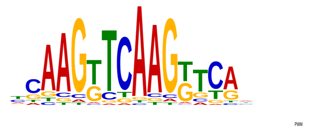	*NNNNNNAAGTNNNAGTTNNNNNN*	28.35	DR:1	3
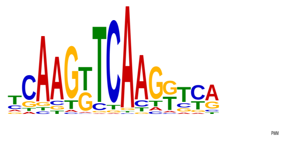	*NNNNNNTCAANNNNNNAGGTNNNNNN*	23.27	DR:6	4
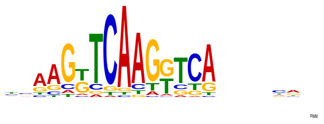	*NNNNNNAGTTNNNNNTCAANNNNNN*	22.33	DR:0	5
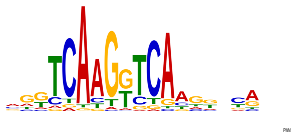	*NNNNNNGGTCNNNNNCAAGNNNNNN*	27.22	M	6
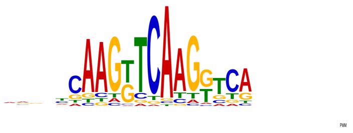	*NNNNNNCAAGNNNNNNNNTCAGNNNNNN*	13.18	M	7
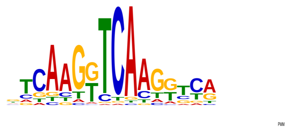	*NNNNNNGTCANNNNNNNGGTCNNNNNN*	12.95	DR:6	8
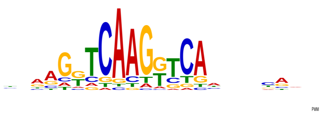	*NNNNNNAGTTNNNNNNCAAGNNNNNN*	13.06	M	9

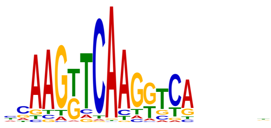	NNNNNNCAAGNNNNNNNNTCAAANNNNNN	20.34	M	10
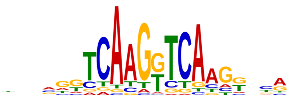	NNNNNNGGTCNNNNNNAAGTNNNNNN	14.47	DR:5	11
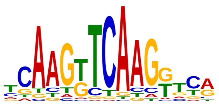	NNNNNNTCAANNNNNNAGTTNNNNNN	22.04	DR:6	12
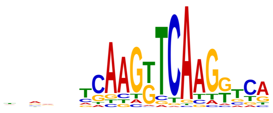	NNNNNNTCAANNNNNNNNNTCAGNNNNNN	9.94	DR:6	13
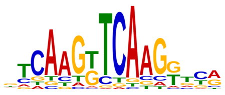	NNNNNNGTCANNNNNNAGTTNNNNNN	12.91	DR:6	14
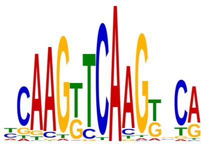	NNNNNNCAAGNNNNAGTGCAANNNNNN	80.56	M	15
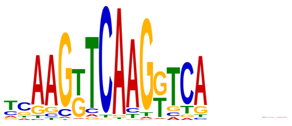	NNNNNNAGTTNNNNNTCATGNNNNNN	22.07	DR:0	16
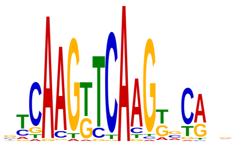	NNNNNNTCAANNNNAAGTGCAANNNNNN	185.60	DR:6	17
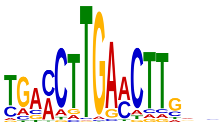	NNNNNNGTTGANNNNNNNCTTGNNNNNN	20.52	M	18

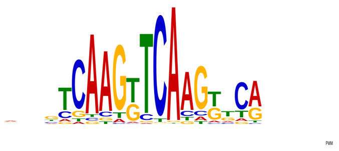	$NNN\overrightarrow{NNNTC}AANNNNNN\overrightarrow{AGTGC}NNNNNN$	23.69	DR:6	19
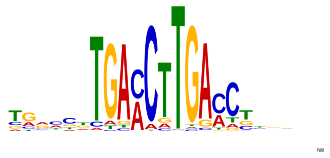	$NNNNNN\overrightarrow{CCGTGA}NNN\overleftarrow{NGACC}NNNNNN$	42.65	IR:3	20
